# Quantitative Proteomics Analysis Expands the Roles of Lysine *β*-Hydroxybutyrylation Pathway in Response to Environmental *β*-Hydroxybutyrate

**DOI:** 10.1155/2022/4592170

**Published:** 2022-02-24

**Authors:** Wanting Hou, Guobin Liu, Xuelian Ren, Xianming Liu, Lei He, He Huang

**Affiliations:** ^1^School of Pharmacy, Nanchang University, Nanchang 330006, China; ^2^Shanghai Institute of Materia Medica, Chinese Academy of Sciences, Shanghai 201203, China; ^3^School of Chinese Materia Medica, Nanjing University of Chinese Medicine, Nanjing 210023, China; ^4^Bruker (Beijing) Scientific Technology Co., Ltd., Beijing 100192, China; ^5^University of Chinese Academy of Sciences, Beijing 100049, China

## Abstract

Lysine *β*-hydroxybutyrylation (Kbhb) is a newly identified protein posttranslational modification (PTM) derived from *β*-hydroxybutyrate (BHB), a product of ketone body metabolism in liver. BHB could serve as an energy source and play a role in the suppression of oxidative stress. The plasma concentration of BHB could increase up to 20 mM during starvation and in pathological conditions. Despite the progress, how the cells derived from extrahepatic tissues respond to elevated environmental BHB remains largely unknown. Given that BHB can significantly drive Kbhb, we characterized the BHB-induced lysine *β*-hydroxybutyrylome and acetylome by quantitative proteomics. A total of 840 unique Kbhb sites on 429 proteins were identified, with 42 sites on 39 proteins increased by more than 50% in response to BHB. The results showed that the upregulated Kbhb induced by BHB was involved in aminoacyl-tRNA biosynthesis, 2-oxocarboxylic acid metabolism, citrate cycle, glycolysis/gluconeogenesis, and pyruvate metabolism pathways. Moreover, some BHB-induced Kbhb substrates were significantly involved in diseases such as cancer. Taken together, we investigate the dynamics of lysine *β*-hydroxybutyrylome and acetylome induced by environmental BHB, which reveals the roles of Kbhb in regulating various biological processes and expands the biological functions of BHB.

## 1. Introduction

Protein posttranslational modifications (PTMs) can change the physical and chemical properties of the protein, affecting the spatial conformation, activity, subcellular localization, protein folding, and so on [1]. They play vital roles in various cellular processes, such as gene expression regulation, cell division, protein degradation, signal transduction, and protein interactions [2]. In-depth study of protein PTMs is of great significance in elaborating the mechanism of life activities, discovering drug targets, and laying the foundation for clinical treatment [3]. With the development of high-resolution mass spectrometry (MS) and proteomics techniques, many new types of PTMs have been identified in recent years, such as lysine 2-hydroxyisobutyrylation, benzoylation, and lactylation [4–8].

A wide array of evidence has demonstrated that endogenous metabolites in mammalian cells are tightly coupled to PTMs [9, 10]. A noteworthy example is *β*-hydroxybutyrate (BHB), an intermediate product of liver fatty acid oxidation and decomposition [11]. BHB, acetoacetate, and acetone are collectively referred to as ketone bodies, in which BHB accounts for about 80% [12, 13]. BHB can be used as an endogenous biologically active small molecule and has critical protective effects on nerves, cardiovascular, and other tissues and organs [12, 14–18]. Recently, we discovered a new type of PTM, lysine *β*-hydroxybutyrylation (Kbhb), and revealed that BHB served as a precursor for Kbhb [19]. Our results showed that starvation, ketogenic diet, and pharmacologically induced diabetes could enhance BHB generation in the liver, in turn mediating cellular signaling functions [20–22]. Notably, when blood sugar levels are low, BHB can be generated in the liver and transported to other energy-consumption tissues as a compensatory energy source [23]. In such conditions, how the cells derived from extrahepatic tissues respond to elevated environmental BHB is still unclear.

To explore the roles of the Kbhb pathway induced by elevated BHB levels, we quantitatively analyzed the dynamics of Kbhb in response to environmental BHB in mouse embryonic fibroblast (MEF) cells. In total, we identified 840 unique Kbhb sites across 429 proteins, and 42 sites from 39 proteins were increased by more than 50% (log_2_ (treated/untreated) > 0.585) in response to BHB. Functional enrichment analysis indicated that significant upregulated Kbhb sites were involved in aminoacyl-tRNA biosynthesis, 2-oxocarboxylic acid metabolism, citrate cycle, pyruvate metabolism, fructose and mannose metabolism, as well as glycolysis/gluconeogenesis pathways.

We also identified 5587 lysine acetylation (Kac) sites across 2303 proteins. Unlike the Kbhb pathway, the dynamic Kac proteins induced by environmental BHB were mainly related to sphingolipid signaling, necroptosis, cellular senescence, and ubiquitin-mediated proteolysis pathways. The diversity of Kbhb and Kac pathways indicated their different roles in response to elevated BHB, therefore, mediating diverse downstream cellular processes.

In addition, we found that some dynamic Kbhb sites were located on important positions for protein substrate/cofactor binding, implying that elevated BHB may affect the functions of substrate proteins by inducing Kbhb on these positions.

Together, this study identified and quantified the dynamics of Kbhb and Kac sites in respond to environmental BHB, which expanded the roles of Kbhb and laid a foundation for further exploring the mechanisms of Kbhb pathway under various physiological and pathological conditions.

## 2. Experimental Section

### 2.1. Materials

Pan anti-Kbhb (PTM-1204) and anti-Kac antibody (PTM-104) were purchased from PTM Biolabs. Anti-histone H3 antibody (M1306-4) was purchased from Huabio. Anti-beta actin antibody (66009-1) was purchased from Proteintech Group. “Light” (^12^C_6_^14^N_2_-Lys, ^12^C_6_^14^N_4_-Arg) (ULM-8766-PK) and “heavy” (^13^C_6_^15^N_2_-Lys, ^13^C_6_^15^N_4_-Arg) (CNLM-291-H-PK) amino acids were purchased from Cambridge Isotope Laboratories. Trypsin used for modified sequencing was purchased from Promega (Madison, WI).

### 2.2. Stable Isotope Labeling and BHB Treatment of Cells

MEF cells were cultured in DMEM free of lysine/arginine, 10% dialyzed FBS, and either “light” (^12^C_6_^14^N_2_-Lys, ^12^C_6_^14^N_4_-Arg) or “heavy” (^13^C_6_^15^N_2_-Lys, ^13^C_6_^15^N_4_-Arg) amino acids (100 mg/L) with 1%penicillin and streptomycin in a 5% CO_2_ atmosphere at 37°C. The cells were cultured for at least 7 generations to ensure a labeling efficiency of more than 98%. MEF cells were treated with 5 mM BHB for 24 hours in order to perform proteomics.

### 2.3. Protein Extraction and Trypsin Digestion

According to the previously described methods [24], the collected “heavy” and “light” cells were washed three times with precooled phosphate-buffered saline. Then, we added protein lysis buffer (8 M urea, 2 mM EDTA, 3 *μ*M TSA, 50 mM NAM, 5 mM DTT, and 1% Protease inhibitor cocktail III) to resuspend the cells and sonicated them with a sonic dismembrator (JY96-IIN, Jingxin, Shanghai, China) on ice. After centrifugation at 18 000 g for 3 min at 4°C, transfer the proteins to a new tube and determine the concentration by BCA method. Proteins from “heavy” and “light” cells were equally mixed and reduced for 1 h with 10 mM dithiothreitol at 37°C and alkylated with 20 mM iodoacetamide for 45 min in a dark environment at 25°C. Then, block the excess iodoacetamide by 20 mM cysteine. Next, 100 mM NH_4_HCO_3_ was added to the protein samples to make sure the urea concentration was less than 2 M. Then, trypsin was added to the proteins at a 1 : 50 trypsin-protein mass ratio for digestion overnight to obtain peptide samples.

### 2.4. Immunoaffinity Enrichment

Kbhb and Kac peptides were immunoaffinity enriched as previously described [24]. Briefly, the peptides were dissolved in NETN buffer (100 mM NaCl, 1 mM EDTA, 50 mM Tris-HCl, 0.5% NP-40, pH 8.0) and incubated with 20 *μ*L of pan anti-Kbhb or pan anti-Kac antibodies conjugated agarose beads on a shaker with gentle shaking at 4°C overnight. In order to remove nonspecific enrichment, we washed the beads with NETN buffer four times and with ddH_2_O twice. Finally, using 0.1% trifluoroacetic acid, the eluted peptides were evaporated to dryness in a vacuum and desalted for mass spectrometry identification.

### 2.5. Mass Spectrometry

A nanoElute nanoflow UHPLC was coupled online to a hybrid trapped ion mobility spectrometry-quadrupole time of flight mass spectrometer (timsTOF Pro, Bruker Daltonics, Bremen, Germany). Peptides were separated in 60 min at a flow rate of 300 nL min^−1^ on a 25 cm × 75 *μ*m column with a pulled emitter tip, packed with 1.9 *μ*m ReproSil-Pur C18-AQ particles (Dr. Maisch, Germany). Mobile phases A and B were 0.1% formic acid in water and 0.1% formic acid in 80% acetonitrile, respectively. The LC method consisting of a gradient of 2-85% in HPLC mobile phase B (0.1% formic acid in acetonitrile, *v*/*v*) and in mobile phase A (0.1% formic acid and 2% acetonitrile in water). The eluted peptides were subjected to capillary source followed by the timsTOF Pro mass spectrometry. The electrospray voltage applied was 1.60 kV. Precursors and fragments were analyzed at the TOF detector, with a MS/MS scan range from 100 to 1700 *m*/*z*. The timsTOF Pro was operated in parallel accumulation serial fragmentation (PASEF) mode. Precursors with charge states 0 to 5 were selected for fragmentation, and 10 PASEF-MS/MS scans were acquired per cycle. The dynamic exclusion was set to 30 s.

### 2.6. Database Search and Data Filter Criteria

For the acquired raw files, searching was performed based on UniProt Mouse protein database (17027 entries, http://http://www.uniprot.org) using Peaks Studio (Version 10.5, Bioinformatics Solutions Inc). The enzyme was specified as trypsin, the peptide mass tolerance was set at 20 ppm, and the MS/MS mass tolerance was set at 0.05 Da. Oxidation of methionine residues (+15.99 Da), acetylation of proteins N-termini (+42.01 Da), *β*-hydroxybutyrylation on lysine, and acetylation on lysine were specified as variable modifications. Maximum missed cleavages per peptide were set as 3. Peptide sequences matching the Human UniProt database were obtained at a false discovery rate (FDR) ≤ 0.01 for both proteins and peptides. A decoy fusion method was employed by PEAKS for FDR calculation. Quantitative data were generated by the PEAKS Q module using “Precursor Ion Quantification” under the “Quantifications” options, and “SILAC-2plex (R10, K8)” method was selected.

### 2.7. Bioinformatics Analysis

Similar to our previously reported methods [24], use a hypergeometric test in clusterProfiler package in R for KEGG pathway enrichment analysis [25]. The protein-protein interaction network of dynamic Kbhb proteins was described by STRING database (v11,http://www.string-db.org/) [26] and visualized in Cytoscape (v.3.8.2) [27].

## 3. Results

### 3.1. Systematic Profiling of Kbhb in Response to BHB

**Figure 1:**
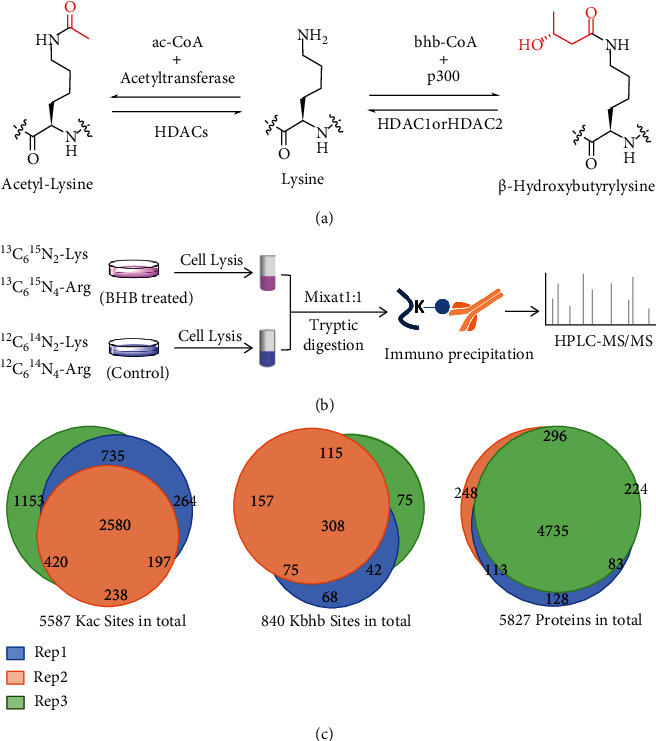
Systematic profiling of lysine *β*-hydroxybutylome and acetylome. (a) Illustration of enzymatic reaction for acetyl-lysine and *β*-hydroxybutyryl-lysine. (b) Schematic representation of experimental workflow for the identification and quantification of Kbhb and Kac in MEF cells. (c) Pie chart shows experimental reproducibility of three biological replicates.

Kbhb is a newly identified PTM driven by a product of lipid metabolism, BHB (Figure 1(a)). To explore the roles of Kbhb pathway in response to environmental BHB, we carried out quantitative Kbhb and Kac proteomics studies in MEF cells treated with BHB (5 mM for 24 hours). To globally identify Kbhb and Kac sites and characterize their diversification stimulated by BHB, we used stable isotope labeling by amino acids in cell culture (SILAC) combined with immunoaffinity enrichment and tandem mass analysis methods. Three biological replicate experiments were performed wherein MEF cells were metabolically labeled with “heavy” amino acids (^13^C_6_^15^N_2_-Lys, ^13^C_6_^15^N_4_-Arg) and “light” amino acids (^12^C_6_^14^N_2_-Lys, ^12^C_6_^14^N_4_-Arg) which were treated and untreated, respectively (Figure 1(b)). Proteins lysed from “heavy” and “light” cells were equally combined and digested by trypsin. Then, the Kbhb and Kac-containing peptides were enriched with immobilized anti-Kbhb and anti-Kac antibodies followed by HPLC-MS/MS analysis on a timsTOF Pro (Bruker) mass spectrometer.

Totally, we identified 840 unique Kbhb sites across 429 proteins and 5587 Kac sites across 2303 proteins. Moreover, 64% Kbhb sites and 70% Kac sites were identified in at least 2 biological replicates, demonstrating good reproducibility (Figure 1(c) and Table S1, S2, S3).

### 3.2. Characterization of Kbhb Proteome

**Figure 2:**
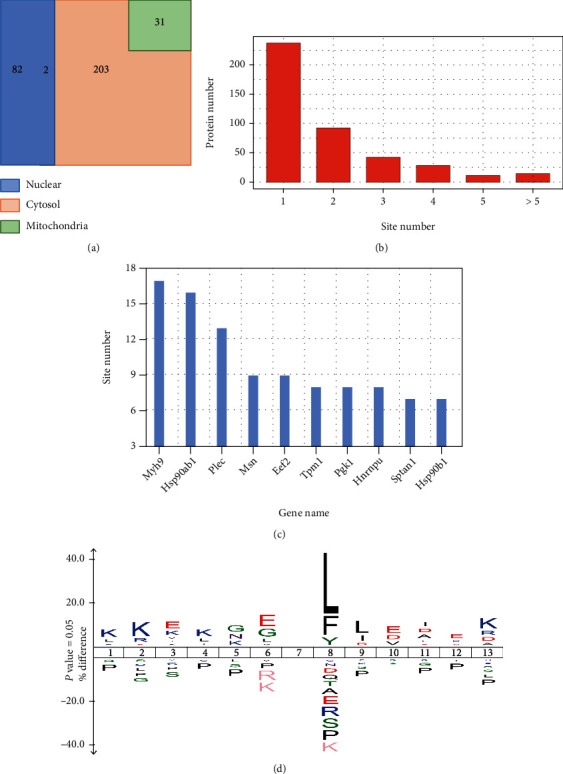
Characterization of Kbhb proteome. (a) Venn diagram shows cellular compartment distribution of Kbhb proteins. (b) Distribution of the Kbhb sites number per protein. (c) The bar graph shows the proteins containing more than 6 Kbhb sites. (d) Consensus sequence logo shows a representative sequence for BHB-induced Kbhb sites.

First, we explored the subcellular distribution of Kbhb substrates in MEF cells by cellular compartment analysis. The results showed that 26% of Kbhb proteins were localized in the nucleus, while 74% were in the cytosol (Figure 2(a)). However, only 10% of Kbhb proteins were annotated in mitochondria. These results differed from our previously reported Kbhb proteome in HEK293 cells, in which 78% of the Kbhb protein were localized in the nucleus [19]. In this study, 74% of Kbhb proteins were localized exclusively or partially in the cytosol, suggesting that Kbhb pathway may play different roles between cells and tissue sources.

The identified Kbhb proteins had various site numbers. For example, 240 (56%) proteins had a single Kbhb site, and 189 (44%) proteins had at least two Kbhb sites (Figure 2(b) and Table S3). Moreover, some proteins were heavily modified by Kbhb. Plectin (Plec), a protein that interlinks intermediate filaments with microtubules and microfilaments and anchors intermediate filaments to desmosomes or hemidesmosomes [28] contains 13 Kbhb sites. Heat shock protein HSP 90-beta (Hsp90ab1) is a molecular chaperone that promotes the maturation, structural maintenance, and proper regulation of specific target proteins involved in cell cycle control and signal transduction [29, 30] and had 16 Kbhb sites. In addition, myosin heavy chain 9 (Myh9) that participates in cytokinesis, cell shape, secretion, and capping [31, 32] had 17 Kbhb sites (Figure 2(c) and Table S3). Multiple Kbhb sites in these proteins may have significant effects on their cellular functions.

Next, we identified the enriched motifs of BHB-induced Kbhb sites using IceLogo. Flanking sequence analysis suggested that the hydrophobic amino acids Leu and Phe at +1 position were overrepresented, while acidic amino acid Asp or Glu were enriched at +3 and -1 positions (Figure 2(d)). These results were different from our previously reported Kbhb motif in HEK293 cells, in which positive-charged lysine was preferred at most positions (-6, -5, -4, -3, +3, +4, +5, and+6), while underrepresentation of negative-charged amino acids at the −2 position [19]. The difference suggests that environmental BHB may induce Kbhb in specific substrates in the presence of Kbhb transferases.

### 3.3. Quantitative Analysis of BHB-Induced Kbhb Proteome

**Figure 3:**
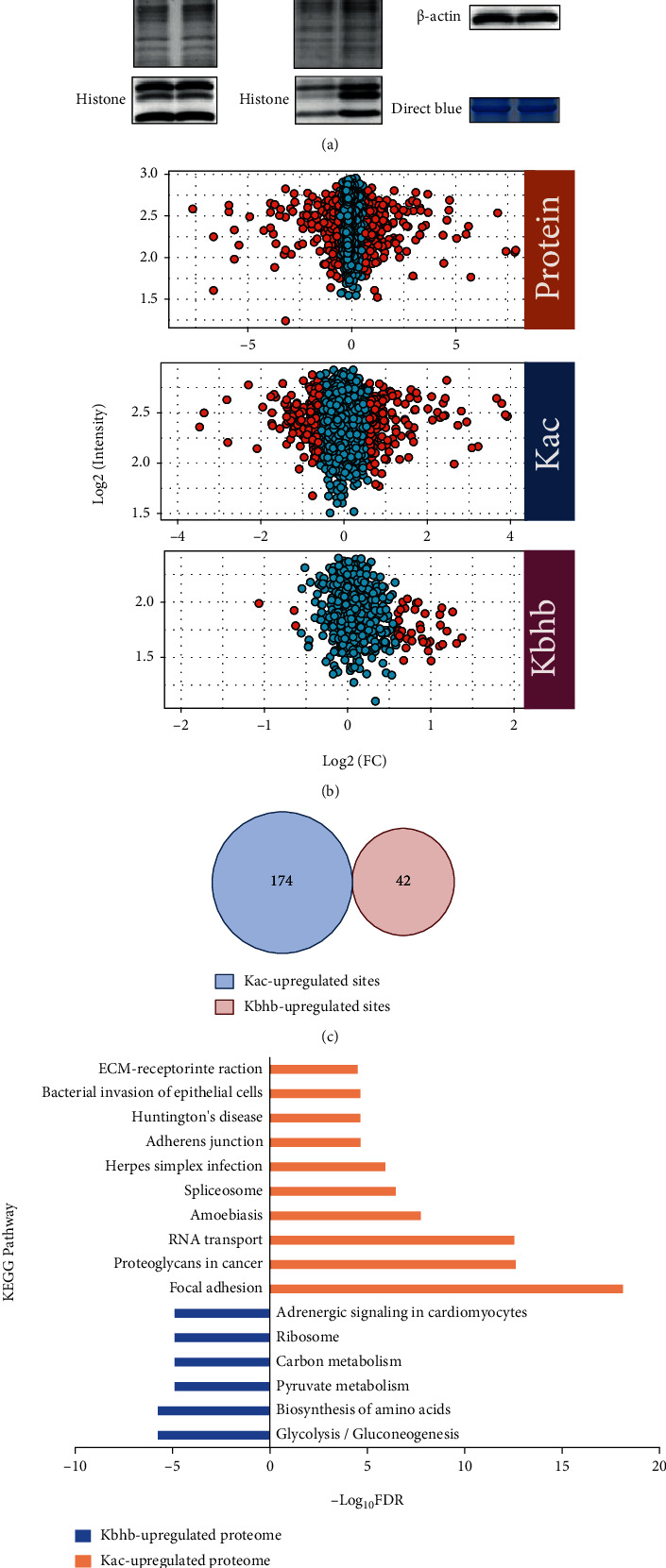
Quantitative analysis of BHB-induced Kbhb and Kac proteome. (a) BHB enhances Kbhb of proteins in MEF cells. Histone Kbhb and Kac levels were analyzed in control and BHB treated MEF cells by immuno-blotting with the indicated antibodies. (b) The scatter plot shows quantification of Kbhb and Kac sites. (c) Venn diagram shows no crossover between the upregulated Kbhb and Kac sites. (d) The KEGG pathway of Kbhb and Kac proteome induced by BHB.

Western blot analysis confirmed that BHB treatment increased global Kbhb levels obviously in MEF cells, while the global Kac levels were slightly affected (Figure 3(a)). To profile the dynamics of Kbhb and Kac, we quantitatively analyzed the BHB-induced Kbhb and Kac proteomes. Among the identified Kbhb sites, 42 of them from 39 proteins were increased by more than 50% (log_2_ (treated/untreated) > 0.585) in response to BHB treatment in MEF cells (Figure 3(b) and Table S3), while only 6 Kbhb sites from 6 proteins decreased by 33% (log_2_ (treated/untreated) < −0.578). Unlike Kbhb, a similar number of Kac sites were increased or decreased after BHB treatment (174 Kac sites increased by more than 50% and 189 Kac sites decreased by 33%, respectively) (Figure 3(c) and Table S2).

Next, we compared the Kbhb and Kac sites increased by more than 50% after BHB treatment. Strikingly, none of them is overlapped (Figure 3(c)), suggesting that environmental BHB influenced cellular pathways through Kbhb and Kac differentially. In support of this notion, Kyoto Encyclopedia of Genes and Genomes (KEGG) pathway analysis of the dynamic Kbhb and Kac substrates revealed that BHB-induced Kac primarily targeted proteins enriched in focal adhesion, proteoglycans in cancer, and RNA transport, whereas the proteins with BHB-induced Kbhb were involved in carbon metabolism, biosynthesis of amino acids, and glycolysis/gluconeogenesis (Figure 3(d)).

### 3.4. Biological Functions Potentially Affected by BHB

**Figure 4:**
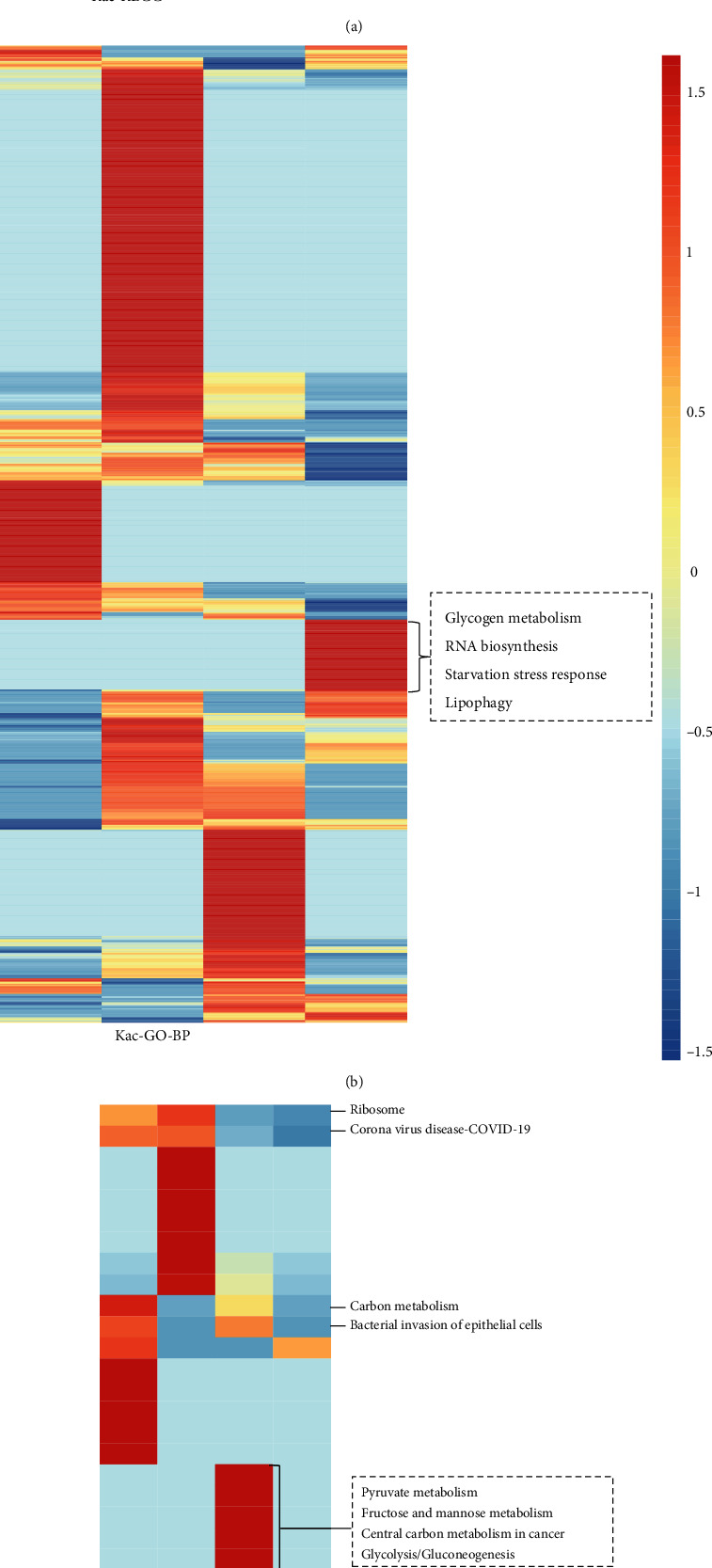
The KEGG and GO biological process analysis. (a) The KEGG pathway of Kac proteome. (b) The GO annotation of Kac proteome. (c) The KEGG pathway of Kbhb proteome. (d) The GO annotation of Kbhb proteome. All the Kbhb and Kac sites were divided into four groups on average according to their dynamics in response to BHB treatment (in ascending order of the treated/control ratio).

To better understand the roles of Kbhb induced by environmental BHB, we divided all the Kbhb and Kac sites into four groups on average according to their dynamics in response to BHB treatment (in ascending order of the treated/control ratio). Then, KEGG and Gene Ontology (GO) biological process analyses on each group were performed. The adjusted *p* values were calculated and normalized by *z*-score to compare the four groups (Figure 4). In the KEGG category, pathways related to aminoacyl-tRNA biosynthesis (adjust *p* value = 1.50*E*-04), 2-oxocarboxylic acid metabolism (adjust *p* value = 1.46*E*-05), and citrate cycle (TCA cycle) (adjust *p* value = 1.01*E*-04) were significantly enriched in Kbhb substrates with high treated/control ratios, while ribosome (adjust *p* value = 3.87*E*-05), bacterial invasion of epithelial cells (adjust *p* value = 2.12*E*-03), and carbon metabolism (adjust *p* value = 1.72*E*-03) pathways were enriched with low Kbhb level upon BHB treatment. Unlike Kbhb, necroptosis (adjust *p* value = 1.51*E*-04), mRNA surveillance pathway (adjust *p* value = 3.41*E*-03), and sphingolipid signaling (adjust *p* value = 3.40*E*-02) pathways were largely enriched with increased Kac levels, whereas the proteins with decreased Kac levels in response to BHB treatment were mainly involved in nucleocytoplasmic transport (adjust *p* value = 1.81*E*-06), spliceosome (adjust *p* value = 1.85*E*-11), and carbon metabolism (adjust *p* value = 4.08*E*-04) pathways.

Consistent with above observation, GO biological process analysis showed that BHB treatment produced a more profound impact on the Kbhb level among tricarboxylic acid cycle (adjust *p* value = 8.23*E*-04) and cell programmed cell death (adjust *p* value =1.18*E*-02), while the proteins involved in glycogen metabolic process (adjust *p* value = 4.07*E*-03), RNA biosynthetic process (adjust *p* value = 1.29*E*-06), and apoptotic process (adjust *p* value = 2.76*E*-06) were enriched with increased acetylation levels. These patterns further support that the environmental BHB can differentially mediate Kbhb and Kac levels in these processes, thereby inducing diverse downstream cellular effects.

### 3.5. Interaction Network of BHB-Induced Kbhb and Kac Proteomes

**Figure 5:**
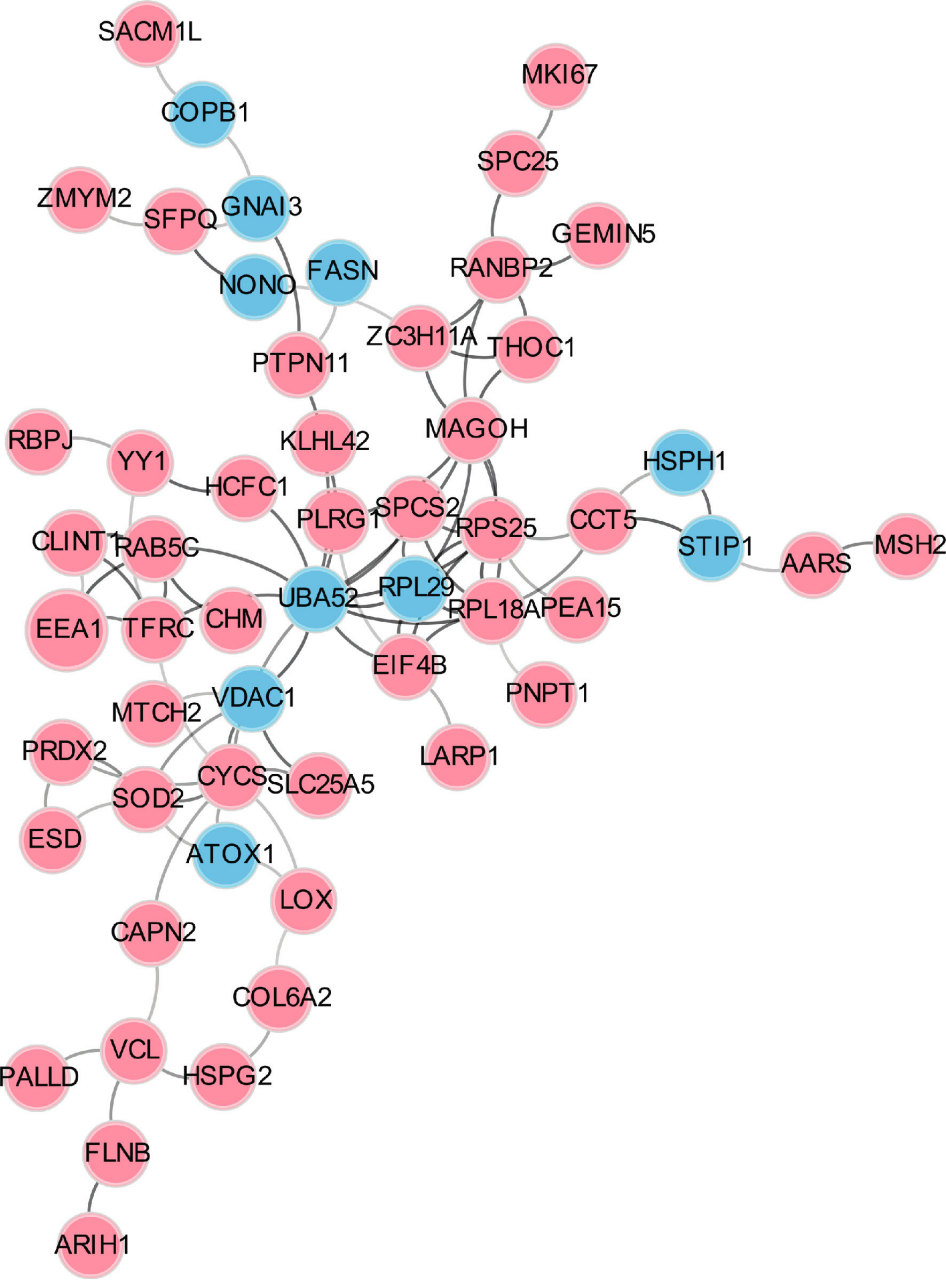
Interaction network of Kbhb and Kac proteome. The blue and red circle represented the proteins with upregulated Kbhb and Kac levels, respectively.

Protein interaction network refers to the interaction between multiple protein molecules to participate in signal transduction, energy metabolism, genetic regulation, and other physiological and biochemical processes [33, 34]. Systematic analysis of the interaction between many proteins in biological systems is crucial for understanding the functional roles of proteins in biological bodies, the regulatory mechanisms of signal transduction and energy metabolism under different physiological states, and the functional relationships among proteins [35–37]. Therefore, we next analyzed the interaction networks of the proteins with BHB-induced Kbhb and Kac sites based on the Search Tool for the Retrieval of Interacting Genes/Proteins (STRING) database (Figure 5). Interestingly, multiple members of the ribosomal protein family interact tightly, including RPL29, RPL18A, and RPS25. Among them, RPL29 contains upregulated Kac sites, and the other two have upregulated Kbhb sites. Ribosomal protein plays an essential role in protein biosynthesis in cells. The results suggested that though the upregulated Kbhb and Kac sites did not overlap, fluctuations of these Kbhb and Kac sites induced by BHB may synergistically affect the protein synthesis process.

### 3.6. Potential Impact of Kbhb on Protein Functions

**Figure 6:**
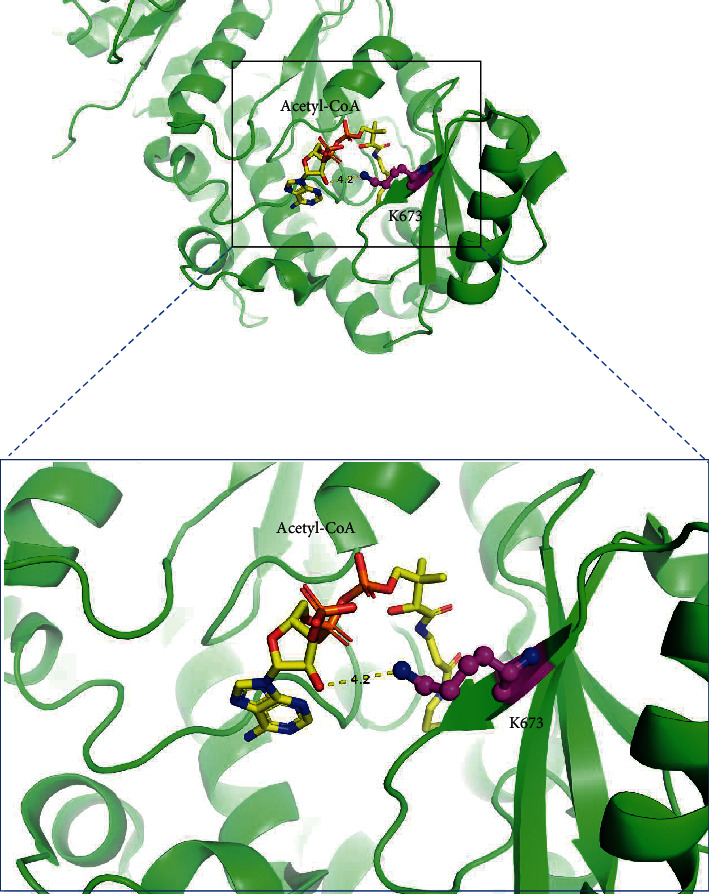
Three-dimensional structure of Fasn shows the Kbhb site and the substrate binding pocket.

A growing body of evidence has demonstrated that PTMs on certain residues can regulate protein functions and participate in a range of important biological processes [38–40]. In order to find out the potential influence of Kbhb on protein functions better, we mapped the BHB induced Kbhb sites with the UniProt database (Table 1). Interestingly, some Kbhb sites were located on or close to important residues for protein functions. For example, fatty acid synthase (Fasn) is a key enzyme in the process of fat synthesis. It has acyltransferase and malonyltransferase activities and can catalyze the synthesis of palmitate under certain conditions, such as the presence of NADPH [41, 42]. It is worth noting that Lys673 of Fasn exhibited significantly higher Kbhb levels upon BHB treatment. Interestingly, this position was located at the acyl and malonyl transferases region. Crystal structure of Fasn showed that K673 was exposed in the binding pocket of acetyl-CoA, with the nitrogen atom of K673 only 4.2 Å away from acetyl-CoA (Figure 6). Therefore, elevated Kbhb level at K673 may potentially influence the transferase activity of Fasn.

Another example is Translin (Tsn), which identifies the ends of single-stranded DNA that break down during chromosomal translocations [43]. This protein has single- and double-stranded endoribonuclease activity and is primarily involved in immune-related gene fragments. The cyclic heterooctamer consisted of six TSN and two Translin-associated protein X subunits is necessary for DNA/RNA binding [44]. Our data showed that *β*-hydroxybutyrylation at residue Lys187 of Tsn was increased by 75% in response to BHB-treatment (Table 1). Notably, Lys187 was located in the leucine zipper region that served as a dimerization module. Kbhb at this position may affect the heterooctamer assemble and regulate its functions.

Apart from the above, Enolase 1 (Eno1) catalyzes the conversion of 2-phosphoglycerate to phosphoenolpyruvate [45, 46]. In addition to glycolysis, it is also involved in the regulation of growth, hypoxia, and allergies [45, 47–49]. It is well known that glycolysis is a common pathway of glucose catabolism in all organisms. Through glycolysis, glucose is degraded to generate ATP, which provides partial energy for life activities and intermediate products or carbon skeleton for other metabolic pathways. The level of Kbhb on key residue Lys256 of Eno1 increased 100% after BHB treatment, which may influence its catalytic function in glycolysis and lead to metabolic stress (Table 1).

## 4. Discussion

BHB metabolism is one of the key ways to maintain body's physiological homeostasis. In mammals, BHB is produced primarily in the liver by fatty acids and transported to peripheral tissues in response to nutritional deficiencies. In addition to being an energy fuel for tissues other than the liver, BHB plays a pivotal role as a driver of protein PTM. We recently discovered an evolutionarily conserved PTM, Kbhb, on core histones. Levels of Kbhb are very dynamic and are influenced by physiological conditions and nutrition sources (e.g., starvation and absence of dietary carbohydrates). These findings support a model in which BHB functions link the outside environment to cellular functions through Kbhb pathway. In humans, serum concentration of BHB can increase up to 20 mM during starvation and in pathological conditions such as diabetes mellitus (DM) and alcoholic liver damage. Despite the progress, how the cells derived from extrahepatic tissues respond to elevated environmental BHB remains largely unknown.

Our study identified the Kbhb and Kac substrates in MEF cells in response to elevated environmental BHB through quantitative proteomics. We found that 840 unique Kbhb sites on 429 proteins, including 42 sites of 39 proteins increased by 1.5 folds (log_2_ (treated/untreated) > 0.585) in response to BHB treatment (Table S1, S2, S3). Notably, the results showed that the upregulated Kbhb and Kac sites did not overlap after BHB treatment, suggesting that BHB-induced Kbhb and Kac regulate different cellular functions. Pathway enrichment analysis also verified these results. For example, aminoacyl-tRNA biosynthesis, 2-oxocarboxylic acid metabolism, and citrate cycle pathways are significantly enriched in the upregulated Kbhb substrates, while amino acid metabolism, ubiquitin-mediated proteolysis, and sphingolipid signaling pathways are largely enriched with increased Kac levels. These results suggest that elevated BHB may regulate energy metabolism and protein degradation through Kbhb and Kac pathways, respectively, in response to environmental glucose deficiency. Furthermore, we revealed that BHB-induced Kbhb may occupy key residues of some proteins with important biological functions, implying that BHB may regulate specific protein functions through the Kbhb pathway.

BHB has been reported to be an inhibitor of histone deacetylase (HDACs) [50]. Although the level of Kbhb increased significantly after BHB treatment, our results showed that there was no obvious increase in histone Kac level in MEF cells treated with 5 mM of BHB (Figure 3(a)), which is consistent with our previously studies [19]. This is not surprising because it was reported that butyrate, but not *β*-hydroxybutyrate, promotes histone Kac in multiple cell lines [50].

In summary, our study revealed the dynamic proteomes of Kbhb and Kac in response to environmental BHB, which expanded the roles of Kbhb pathway and suggested a new understanding of the biological functions of BHB.

## Figures and Tables

**Table 1:** Kbhb sites on key residues involving substrate/cofactor binding, protein interaction, and cancer biomarkers.

Protein	Site	Feature
Fasn	673	Acyl and malonyl transferases
Uba52	6	Lung cancer biomarker
Tsn	187	Leucine-zipper region
Pdlim1	254	Binding with zinc at 258 and 261 sites
Pgam5	140	Phosphatase activity of serine/threonine residues
Gstm1	136	Breast cancer biomarker
Aco2	50	Catalyzes citrate to isocitrate
Eno1	256	Participates in glycolysis process

## Data Availability

The mass spectrometry proteomics data have been deposited to the ProteomeXchange Consortium via the PRIDE partner repository with the dataset identifier PXD029481.

## Supplementary Materials

Supplementary Materials Supplementary Materials Table S1/S2/S3 are the complete lists of identified proteins/Kac sites/Kbhb sites in MEF cells with or without BHB treated.
