# Supplementary material for: Quantitative Proteomics Analysis Expands the Roles of Lysine β-Hydroxybutyrylation Pathway in Response to Environmental β-Hydroxybutyrate
Source: Oxid Med Cell Longev. 2022 Feb 24;2022:4592170. doi: 10.1155/2022/4592170 (PMC8894020; doi:10.1155/2022/4592170)
Supplement: Supplementary Materials — Table S1/S2/S3 are the complete lists of identified proteins/Kac sites/Kbhb sites in MEF cells with or without BHB treated. [file 4592170.f1.zip › Table S1. The identification of whole cell proteins in MEF.pdf]

Table S1:The identification of whole cell proteins in MEF.

Gene name	UniprotID	Description	Ratio_average
Sgcb	P82349	Beta-sarcoglycan OS=Mus musculus OX=10090 GN=Sgcb PE=1 SV=1	0.683333333
Jund	P15066	Transcription factor jun-D OS=Mus musculus OX=10090 GN=Jund PE=1 SV=1	0.876666667
Tmem14c	Q9CQN6	Transmembrane protein 14C OS=Mus musculus OX=10090 GN=Tmem14c PE=1 SV=1	0.886666667
Snrpe	P62305	Small nuclear ribonucleoprotein E OS=Mus musculus OX=10090 GN=Snrpe PE=1 SV=1	0.913333333
G6pc3	Q6NSQ9	Glucose-6-phosphatase 3 OS=Mus musculus OX=10090 GN=G6pc3 PE=1 SV=1	0.85
Slc25a22	Q9D6M3	Mitochondrial glutamate carrier 1 OS=Mus musculus OX=10090 GN=Slc25a22 PE=1 SV=1	0.983333333
Prorsd1	Q9D820	Prolyl-tRNA synthetase associated domain-containing protein 1 OS=Mus musculus OX=10090 GN=P	0.85
Men1	O88559	Menin OS=Mus musculus OX=10090 GN=Men1 PE=1 SV=2	1.073333333
Fancd2	Q80V62	Fanconi anemia group D2 protein homolog OS=Mus musculus OX=10090 GN=Fancd2 PE=1 SV=2	
	Q8BR90	UPF0600 protein C5orf51 homolog OS=Mus musculus OX=10090 PE=1 SV=1	
Jam3	Q9D8B7	Junctional adhesion molecule C OS=Mus musculus OX=10090 GN=Jam3 PE=1 SV=2	0.826666667
Camsap1	A2AHC3	Calmodulin-regulated spectrin-associated protein 1 OS=Mus musculus OX=10090 GN=Camsap1 PE=	0.81
Eml3	Q8VC03	Echinoderm microtubule-associated protein-like 3 OS=Mus musculus OX=10090 GN=Eml3 PE=1 SV=	
Srek1	Q8BZX4	Splicing regulatory glutamine/lysine-rich protein 1 OS=Mus musculus OX=10090 GN=Srek1 PE=2 SV	1.273333333
Mfsd10	Q9D2V8	Major facilitator superfamily domain-containing protein 10 OS=Mus musculus OX=10090 GN=Mfsd	0.946666667
Htra1	Q9R118	Serine protease HTRA1 OS=Mus musculus OX=10090 GN=Htra1 PE=1 SV=2	1.636666667
Serpinb8	O08800	Serpin B8 OS=Mus musculus OX=10090 GN=Serpinb8 PE=1 SV=2	
Phc1	Q64028	Polyhomeotic-like protein 1 OS=Mus musculus OX=10090 GN=Phc1 PE=1 SV=2	0.08
Gxylt1	Q3UHH8	Glucoside xylosyltransferase 1 OS=Mus musculus OX=10090 GN=Gxylt1 PE=1 SV=2	
Col11a1	Q61245	Collagen alpha-1(XI) chain OS=Mus musculus OX=10090 GN=Col11a1 PE=1 SV=2	
Elovl1	Q9JLJ5	Elongation of very long chain fatty acids protein 1 OS=Mus musculus OX=10090 GN=Elovl1 PE=1 SV=	
Ythdc1	E9Q5K9	YTH domain-containing protein 1 OS=Mus musculus OX=10090 GN=Ythdc1 PE=1 SV=2	0.723333333
Sirt1	Q923E4	NAD-dependent protein deacetylase sirtuin-1 OS=Mus musculus OX=10090 GN=Sirt1 PE=1 SV=2	
Bloc1s3	Q5U5M8	Biogenesis of lysosome-related organelles complex 1 subunit 3 OS=Mus musculus OX=10090 GN=Bl	0.423333333
Lsm5	P62322	U6 snRNA-associated Sm-like protein LSm5 OS=Mus musculus OX=10090 GN=Lsm5 PE=3 SV=2	0.826666667
Ss18	Q62280	Protein SSXT OS=Mus musculus OX=10090 GN=Ss18 PE=1 SV=2	0.926666667
Fbxo11	Q7TPD1	F-box only protein 11 OS=Mus musculus OX=10090 GN=Fbxo11 PE=1 SV=3	1.59
Jun	P05627	Transcription factor AP-1 OS=Mus musculus OX=10090 GN=Jun PE=1 SV=3	0.2
Ltbp2	O08999	Latent-transforming growth factor beta-binding protein 2 OS=Mus musculus OX=10090 GN=Ltbp2 I	
Wdr13	Q91V09	WD repeat-containing protein 13 OS=Mus musculus OX=10090 GN=Wdr13 PE=1 SV=1	1.405
Pmm1	O35621	Phosphomannomutase 1 OS=Mus musculus OX=10090 GN=Pmm1 PE=1 SV=1	0.805

Lamtor2	Q9JHS3	Ragulator complex protein LAMTOR2 OS=Mus musculus OX=10090 GN=Lamtor2 PE=1 SV=1	0.773333333
Zfyve27	Q3TXX3	Protrudin OS=Mus musculus OX=10090 GN=Zfyve27 PE=1 SV=2	
Ppox	P51175	Protoporphyrinogen oxidase OS=Mus musculus OX=10090 GN=Ppox PE=1 SV=1	0.776666667
Brms1l	Q3U1T3	Breast cancer metastasis-suppressor 1-like protein OS=Mus musculus OX=10090 GN=Brms1l PE=2 SV	
Gmeb2	P58929	Glucocorticoid modulatory element-binding protein 2 OS=Mus musculus OX=10090 GN=Gmeb2 PE	1.33
Tcaf1	Q8BNE1	TRPM8 channel-associated factor 1 OS=Mus musculus OX=10090 GN=Tcaf1 PE=2 SV=1	
Stx2	Q00262	Syntaxin-2 OS=Mus musculus OX=10090 GN=Stx2 PE=1 SV=1	
Prpf18	Q8BM39	Pre-mRNA-splicing factor 18 OS=Mus musculus OX=10090 GN=Prpf18 PE=1 SV=1	2.775
Ptprs	B0V2N1	Receptor-type tyrosine-protein phosphatase S OS=Mus musculus OX=10090 GN=Ptprs PE=1 SV=1	
Tmem126b	Q9D1R1	Complex I assembly factor TMEM126B mitochondrial OS=Mus musculus OX=10090 GN=Tmem126b	1.19
Ccm2	Q8K2Y9	Cerebral cavernous malformations protein 2 homolog OS=Mus musculus OX=10090 GN=Ccm2 PE=1	0.94
Fbln5	Q9WVH9	Fibulin-5 OS=Mus musculus OX=10090 GN=Fbln5 PE=1 SV=1	0.843333333
Ccdc186	Q8C9S4	Coiled-coil domain-containing protein 186 OS=Mus musculus OX=10090 GN=Ccdc186 PE=1 SV=2	1.61
Kansl2	Q8BQR4	KAT8 regulatory NSL complex subunit 2 OS=Mus musculus OX=10090 GN=Kansl2 PE=1 SV=1	
Chrac1	Q9JKP8	Chromatin accessibility complex protein 1 OS=Mus musculus OX=10090 GN=Chrac1 PE=1 SV=1	0.82
Ints8	Q80V86	Integrator complex subunit 8 OS=Mus musculus OX=10090 GN=Ints8 PE=1 SV=1	0.866666667
Aifm2	Q8BUE4	Ferroptosis suppressor protein 1 OS=Mus musculus OX=10090 GN=Aifm2 PE=1 SV=1	1.035
	Q9D2X8	Uncharacterized protein C8orf76 homolog OS=Mus musculus OX=10090 PE=1 SV=1	
Plp2	Q9R1Q7	Proteolipid protein 2 OS=Mus musculus OX=10090 GN=Plp2 PE=1 SV=1	1.036666667
Tfb2m	Q3TL26	Dimethyladenosine transferase 2 mitochondrial OS=Mus musculus OX=10090 GN=Tfb2m PE=2 SV=1	
Herc2	Q4U2R1	E3 ubiquitin-protein ligase HERC2 OS=Mus musculus OX=10090 GN=Herc2 PE=1 SV=3	0.5
Hacd2	Q9D3B1	Very-long-chain (3R)-3-hydroxyacyl-CoA dehydratase 2 OS=Mus musculus OX=10090 GN=Hacd2 PE=	0.95
Cfap410	Q8C6G1	Cilia- and flagella-associated protein 410 OS=Mus musculus OX=10090 GN=Cfap410 PE=1 SV=1	
Wdr73	Q9CWR1	WD repeat-containing protein 73 OS=Mus musculus OX=10090 GN=Wdr73 PE=1 SV=1	
Srxn1	Q9D975	Sulfiredoxin-1 OS=Mus musculus OX=10090 GN=Srxn1 PE=2 SV=1	0.855
Znf740	Q6NZQ6	Zinc finger protein 740 OS=Mus musculus OX=10090 GN=Znf740 PE=1 SV=1	1
Kdelr3	Q8R1L4	ER lumen protein-retaining receptor 3 OS=Mus musculus OX=10090 GN=Kdelr3 PE=1 SV=1	
Ect2	Q07139	Protein ECT2 OS=Mus musculus OX=10090 GN=Ect2 PE=1 SV=2	
Styk1	Q6J9G1	Tyrosine-protein kinase STYK1 OS=Mus musculus OX=10090 GN=Styk1 PE=2 SV=2	
Pgghg	Q8BP56	Protein-glucosylgalactosylhydroxylysine glucosidase OS=Mus musculus OX=10090 GN=Pgghg PE=1 S	
Fbxl15	Q91W61	F-box/LRR-repeat protein 15 OS=Mus musculus OX=10090 GN=Fbxl15 PE=1 SV=2	0.99
Helb	Q6NVF4	DNA helicase B OS=Mus musculus OX=10090 GN=Helb PE=1 SV=2	1.12
Tpst2	O88856	Protein-tyrosine sulfotransferase 2 OS=Mus musculus OX=10090 GN=Tpst2 PE=1 SV=1	
	Q3U6N9	UPF0488 protein C8orf33 homolog OS=Mus musculus OX=10090 PE=2 SV=1	1.063333333

Dnajc5	P60904	DnaJ homolog subfamily C member 5 OS=Mus musculus OX=10090 GN=Dnajc5 PE=1 SV=1	0.863333333
Mthfd2l	D3YZG8	Probable bifunctional methylenetetrahydrofolate dehydrogenase/cyclohydrolase 2 OS=Mus muscu	
Ormdl1	Q921I0	ORM1-like protein 1 OS=Mus musculus OX=10090 GN=Ormdl1 PE=2 SV=1	0.883333333
Sgo2	Q7TSY8	Shugoshin 2 OS=Mus musculus OX=10090 GN=Sgo2 PE=1 SV=1	0.81
Polr3e	Q9CZT4	DNA-directed RNA polymerase III subunit RPC5 OS=Mus musculus OX=10090 GN=Polr3e PE=1 SV=2	
Polr2k	Q63871	DNA-directed RNA polymerases I II and III subunit RPABC4 OS=Mus musculus OX=10090 GN=Polr2k	0.746666667
Trappc9	Q3U0M1	Trafficking protein particle complex subunit 9 OS=Mus musculus OX=10090 GN=Trappc9 PE=1 SV=2	0.74
Olfml2b	Q3V1G4	Olfactomedin-like protein 2B OS=Mus musculus OX=10090 GN=Olfml2b PE=1 SV=2	
Fgfr1op	Q66JX5	Centrosomal protein 43 OS=Mus musculus OX=10090 GN=Cep43 PE=1 SV=1	1.835
Cplx2	P84086	Complexin-2 OS=Mus musculus OX=10090 GN=Cplx2 PE=1 SV=1	0.48
Sec61g	P60060	Protein transport protein Sec61 subunit gamma OS=Mus musculus OX=10090 GN=Sec61g PE=3 SV=	0.846666667
Baiap2l1	Q9DBJ3	Brain-specific angiogenesis inhibitor 1-associated protein 2-like protein 1 OS=Mus musculus OX=10	1.15
Mid1ip1	Q9CQ20	Mid1-interacting protein 1 OS=Mus musculus OX=10090 GN=Mid1ip1 PE=1 SV=1	0.725
Clk3	O35492	Dual specificity protein kinase CLK3 OS=Mus musculus OX=10090 GN=Clk3 PE=1 SV=2	
Pdss2	Q33DR3	Decaprenyl-diphosphate synthase subunit 2 OS=Mus musculus OX=10090 GN=Pdss2 PE=1 SV=2	1
Wdr41	Q3UDP0	WD repeat-containing protein 41 OS=Mus musculus OX=10090 GN=Wdr41 PE=1 SV=1	1.273333333
Pdrg1	P59048	p53 and DNA damage-regulated protein 1 OS=Mus musculus OX=10090 GN=Pdrg1 PE=2 SV=1	0.865
Trps1	Q925H1	Zinc finger transcription factor Trps1 OS=Mus musculus OX=10090 GN=Trps1 PE=1 SV=1	
Selenoi	Q80TA1	Ethanolaminephosphotransferase 1 OS=Mus musculus OX=10090 GN=Selenoi PE=2 SV=3	0.92
Supt7l	Q9CZV5	STAGA complex 65 subunit gamma OS=Mus musculus OX=10090 GN=Supt7l PE=1 SV=1	0.953333333
Rex1bd	Q9CYZ6	Required for excision 1-B domain-containing protein OS=Mus musculus OX=10090 GN=Rex1bd PE=	0.263333333
Sfswap	Q3USH5	Splicing factor suppressor of white-apricot homolog OS=Mus musculus OX=10090 GN=Sfswap PE=1	1.515
Smarcal1	Q8BJL0	SWI/SNF-related matrix-associated actin-dependent regulator of chromatin subfamily A-like protein	1.195
Dnajc17	Q91WT4	DnaJ homolog subfamily C member 17 OS=Mus musculus OX=10090 GN=Dnajc17 PE=1 SV=2	0.685
Mipep	A6H611	Mitochondrial intermediate peptidase OS=Mus musculus OX=10090 GN=Mipep PE=1 SV=1	
Uckl1	Q91YL3	Uridine-cytidine kinase-like 1 OS=Mus musculus OX=10090 GN=Uckl1 PE=1 SV=1	
Ears2	Q9CXJ1	Probable glutamate--tRNA ligase mitochondrial OS=Mus musculus OX=10090 GN=Ears2 PE=1 SV=1	
Usp25	P57080	Ubiquitin carboxyl-terminal hydrolase 25 OS=Mus musculus OX=10090 GN=Usp25 PE=1 SV=2	
Junb	P09450	Transcription factor jun-B OS=Mus musculus OX=10090 GN=Junb PE=1 SV=1	1.283333333
Tmem41b	Q8K1A5	Transmembrane protein 41B OS=Mus musculus OX=10090 GN=Tmem41b PE=1 SV=1	0.71
Mrpl52	Q9D0Y8	39S ribosomal protein L52 mitochondrial OS=Mus musculus OX=10090 GN=Mrpl52 PE=1 SV=1	0.933333333
Cox17	P56394	Cytochrome c oxidase copper chaperone OS=Mus musculus OX=10090 GN=Cox17 PE=1 SV=2	
Mgrn1	Q9D074	E3 ubiquitin-protein ligase MGRN1 OS=Mus musculus OX=10090 GN=Mgrn1 PE=1 SV=2	
Cnep1r1	Q3UJ81	Nuclear envelope phosphatase-regulatory subunit 1 OS=Mus musculus OX=10090 GN=Cnep1r1 PE=	1.6

Nrf1	Q9WU00	Nuclear respiratory factor 1 OS=Mus musculus OX=10090 GN=Nrf1 PE=1 SV=2	0.72
Tmem199	Q5SYH2	Transmembrane protein 199 OS=Mus musculus OX=10090 GN=Tmem199 PE=1 SV=1	0.793333333
Gc	P21614	Vitamin D-binding protein OS=Mus musculus OX=10090 GN=Gc PE=1 SV=2	0.033333333
Zcrb1	Q9CZ96	Zinc finger CCHC-type and RNA-binding motif-containing protein 1 OS=Mus musculus OX=10090 GN	1.065
Cfb	P04186	Complement factor B OS=Mus musculus OX=10090 GN=Cfb PE=1 SV=2	0.213333333
Tmem256	Q5F285	Transmembrane protein 256 OS=Mus musculus OX=10090 GN=Tmem256 PE=1 SV=1	0.735
Mpg	Q04841	DNA-3-methyladenine glycosylase OS=Mus musculus OX=10090 GN=Mpg PE=2 SV=3	1.69
Zdhhc13	Q9CWU2	Palmitoyltransferase ZDHHC13 OS=Mus musculus OX=10090 GN=Zdhhc13 PE=1 SV=2	1.155
Ntrk3	Q6VNS1	NT-3 growth factor receptor OS=Mus musculus OX=10090 GN=Ntrk3 PE=1 SV=1	
Osbpl5	Q9ER64	Oxysterol-binding protein-related protein 5 OS=Mus musculus OX=10090 GN=Osbpl5 PE=1 SV=3	
Tnfaip8	Q921Z5	Tumor necrosis factor alpha-induced protein 8 OS=Mus musculus OX=10090 GN=Tnfaip8 PE=1 SV=1	0.47
Gemin8	Q8BHE1	Gem-associated protein 8 OS=Mus musculus OX=10090 GN=Gemin8 PE=1 SV=1	
Gng5	Q80SZ7	Guanine nucleotide-binding protein G(I)/G(S)/G(O) subunit gamma-5 OS=Mus musculus OX=10090	0.75
Higd1a	Q9JLR9	HIG1 domain family member 1A mitochondrial OS=Mus musculus OX=10090 GN=Higd1a PE=1 SV=	0.883333333
Ammecr1l	Q8JZZ6	AMMECR1-like protein OS=Mus musculus OX=10090 GN=Ammecr1l PE=1 SV=1	1.695
Znf428	Q8C1M2	Zinc finger protein 428 OS=Mus musculus OX=10090 GN=Znf428 PE=1 SV=1	1.48
Fez1	Q8K0X8	Fasciculation and elongation protein zeta-1 OS=Mus musculus OX=10090 GN=Fez1 PE=1 SV=2	0.78
Nepro	Q8R2U2	Nucleolus and neural progenitor protein OS=Mus musculus OX=10090 GN=Nepro PE=2 SV=1	0.585
Rabgap1l	A6H6A9	Rab GTPase-activating protein 1-like OS=Mus musculus OX=10090 GN=Rabgap1l PE=1 SV=1	
Pdzrn3	Q69ZS0	E3 ubiquitin-protein ligase PDZRN3 OS=Mus musculus OX=10090 GN=Pdzrn3 PE=1 SV=3	
Slc35e1	Q8CD26	Solute carrier family 35 member E1 OS=Mus musculus OX=10090 GN=Slc35e1 PE=1 SV=2	1.42
Rab28	Q99KL7	Ras-related protein Rab-28 OS=Mus musculus OX=10090 GN=Rab28 PE=1 SV=1	
Mlh1	Q9JK91	DNA mismatch repair protein Mlh1 OS=Mus musculus OX=10090 GN=Mlh1 PE=1 SV=2	
Atpaf2	Q91YY4	ATP synthase mitochondrial F1 complex assembly factor 2 OS=Mus musculus OX=10090 GN=Atpaf2	
Ttc28	Q80XJ3	Tetratricopeptide repeat protein 28 OS=Mus musculus OX=10090 GN=Ttc28 PE=1 SV=3	
Tbc1d10b	Q8BHL3	TBC1 domain family member 10B OS=Mus musculus OX=10090 GN=Tbc1d10b PE=1 SV=2	
Emc10	Q3TAS6	ER membrane protein complex subunit 10 OS=Mus musculus OX=10090 GN=Emc10 PE=2 SV=2	
Myd88	P22366	Myeloid differentiation primary response protein MyD88 OS=Mus musculus OX=10090 GN=Myd88	0.485
Znf451	Q8C0P7	E3 SUMO-protein ligase ZNF451 OS=Mus musculus OX=10090 GN=Znf451 PE=1 SV=1	
Snx15	Q91WE1	Sorting nexin-15 OS=Mus musculus OX=10090 GN=Snx15 PE=1 SV=1	
Cdc20	Q9JJ66	Cell division cycle protein 20 homolog OS=Mus musculus OX=10090 GN=Cdc20 PE=1 SV=2	1.18
Ndufaf5	A2APY7	Arginine-hydroxylase NDUFAF5 mitochondrial OS=Mus musculus OX=10090 GN=Ndufaf5 PE=1 SV=	0.95
Abhd13	Q80UX8	Protein ABHD13 OS=Mus musculus OX=10090 GN=Abhd13 PE=2 SV=1	
Mtif2	Q91YJ5	Translation initiation factor IF-2 mitochondrial OS=Mus musculus OX=10090 GN=Mtif2 PE=1 SV=2	

Mob3a	Q8BSU7	MOB kinase activator 3A OS=Mus musculus OX=10090 GN=Mob3a PE=1 SV=1	0.843333333
Nek1	P51954	Serine/threonine-protein kinase Nek1 OS=Mus musculus OX=10090 GN=Nek1 PE=1 SV=2	
Tmem63a	Q91YT8	CSC1-like protein 1 OS=Mus musculus OX=10090 GN=Tmem63a PE=1 SV=1	
Zc3h8	Q9JJ48	Zinc finger CCCH domain-containing protein 8 OS=Mus musculus OX=10090 GN=Zc3h8 PE=1 SV=2	
Mrpl51	Q9CPY1	39S ribosomal protein L51 mitochondrial OS=Mus musculus OX=10090 GN=Mrpl51 PE=1 SV=1	
Zcchc24	B2RVL6	Zinc finger CCHC domain-containing protein 24 OS=Mus musculus OX=10090 GN=Zcchc24 PE=1 SV=	0.89
Ccdc91	Q9D8L5	Coiled-coil domain-containing protein 91 OS=Mus musculus OX=10090 GN=Ccdc91 PE=1 SV=2	0.615
Yaf2	Q99LW6	YY1-associated factor 2 OS=Mus musculus OX=10090 GN=Yaf2 PE=1 SV=1	0.933333333
Dpysl4	O35098	Dihydropyrimidinase-related protein 4 OS=Mus musculus OX=10090 GN=Dpysl4 PE=1 SV=1	
Lzts2	Q91YU6	Leucine zipper putative tumor suppressor 2 OS=Mus musculus OX=10090 GN=Lzts2 PE=1 SV=3	0.84
Ncoa2	Q61026	Nuclear receptor coactivator 2 OS=Mus musculus OX=10090 GN=Ncoa2 PE=1 SV=3	8.395
Pold3	Q9EQ28	DNA polymerase delta subunit 3 OS=Mus musculus OX=10090 GN=Pold3 PE=1 SV=2	0.266666667
Twist2	Q9D030	Twist-related protein 2 OS=Mus musculus OX=10090 GN=Twist2 PE=1 SV=1	
Hmgcr	Q01237	3-hydroxy-3-methylglutaryl-coenzyme A reductase OS=Mus musculus OX=10090 GN=Hmgcr PE=1 S\	
Ift74	Q8BKE9	Intraflagellar transport protein 74 homolog OS=Mus musculus OX=10090 GN=Ift74 PE=1 SV=2	
Rnf13	O54965	E3 ubiquitin-protein ligase RNF13 OS=Mus musculus OX=10090 GN=Rnf13 PE=1 SV=2	0.716666667
Rc3h1	Q4VGL6	Roquin-1 OS=Mus musculus OX=10090 GN=Rc3h1 PE=1 SV=1	
Mvb12a	Q78HU3	Multivesicular body subunit 12A OS=Mus musculus OX=10090 GN=Mvb12a PE=1 SV=1	
Ormdl3	Q9CPZ6	ORM1-like protein 3 OS=Mus musculus OX=10090 GN=Ormdl3 PE=2 SV=1	0.796666667
Eapp	Q5BU09	E2F-associated phosphoprotein OS=Mus musculus OX=10090 GN=Eapp PE=1 SV=2	1.033333333
Ctdspl2	Q8BG15	CTD small phosphatase-like protein 2 OS=Mus musculus OX=10090 GN=Ctdspl2 PE=1 SV=1	2.723333333
Ndufa12	Q7TMF3	NADH dehydrogenase [ubiquinone] 1 alpha subcomplex subunit 12 OS=Mus musculus OX=10090 GI	2.12
Grk5	Q8VEB1	G protein-coupled receptor kinase 5 OS=Mus musculus OX=10090 GN=Grk5 PE=1 SV=2	0.665
Tor3a	Q9ER38	Torsin-3A OS=Mus musculus OX=10090 GN=Tor3a PE=1 SV=2	0.535
Dhx33	Q80VY9	ATP-dependent RNA helicase DHX33 OS=Mus musculus OX=10090 GN=Dhx33 PE=1 SV=1	
Rabif	Q91X96	Guanine nucleotide exchange factor MSS4 OS=Mus musculus OX=10090 GN=Rabif PE=1 SV=1	1.03
Ccny	Q8BGU5	Cyclin-Y OS=Mus musculus OX=10090 GN=Ccny PE=1 SV=1	0.696666667
Fbxw11	Q5SRY7	F-box/WD repeat-containing protein 11 OS=Mus musculus OX=10090 GN=Fbxw11 PE=1 SV=1	
Nsun5	Q8K4F6	28S rRNA (cytosine-C(5))-methyltransferase OS=Mus musculus OX=10090 GN=Nsun5 PE=1 SV=2	
Optn	Q8K3K8	Optineurin OS=Mus musculus OX=10090 GN=Optn PE=1 SV=1	
Ciao3	Q7TMW6	Cytosolic iron-sulfur assembly component 3 OS=Mus musculus OX=10090 GN=Ciao3 PE=1 SV=2	0.825
Saraf	Q8R3Q0	Store-operated calcium entry-associated regulatory factor OS=Mus musculus OX=10090 GN=Saraf P	
Yju2	Q9D6J3	Splicing factor YJU2 OS=Mus musculus OX=10090 GN=Yju2 PE=1 SV=1	0.405
Chkb	O55229	Choline/ethanolamine kinase OS=Mus musculus OX=10090 GN=Chkb PE=1 SV=3	0.935

Mzt2	Q9CQ25	Mitotic-spindle organizing protein 2 OS=Mus musculus OX=10090 GN=Mzt2 PE=1 SV=1	0.816666667
Fam118a	Q91YN1	Protein FAM118A OS=Mus musculus OX=10090 GN=Fam118a PE=1 SV=1	
Cacna1a	P97445	Voltage-dependent P/Q-type calcium channel subunit alpha-1A OS=Mus musculus OX=10090 GN=Ca	
Rnf115	Q9D0C1	E3 ubiquitin-protein ligase RNF115 OS=Mus musculus OX=10090 GN=Rnf115 PE=1 SV=1	0.973333333
Isg20l2	Q3U1G5	Interferon-stimulated 20 kDa exonuclease-like 2 OS=Mus musculus OX=10090 GN=Isg20l2 PE=1 SV=	0.933333333
Rai1	Q61818	Retinoic acid-induced protein 1 OS=Mus musculus OX=10090 GN=Rai1 PE=1 SV=3	
Tcf3	P15806	Transcription factor E2-alpha OS=Mus musculus OX=10090 GN=Tcf3 PE=1 SV=2	
Slc39a6	Q8C145	Zinc transporter ZIP6 OS=Mus musculus OX=10090 GN=Slc39a6 PE=1 SV=1	1.203333333
Arl6ip4	Q9JM93	ADP-ribosylation factor-like protein 6-interacting protein 4 OS=Mus musculus OX=10090 GN=Arl6ip	0.885
Frmd6	Q8C0V9	FERM domain-containing protein 6 OS=Mus musculus OX=10090 GN=Frmd6 PE=1 SV=2	
Gabpb1	Q00420	GA-binding protein subunit beta-1 OS=Mus musculus OX=10090 GN=Gabpb1 PE=1 SV=2	0.63
Mt2	P02798	Metallothionein-2 OS=Mus musculus OX=10090 GN=Mt2 PE=1 SV=2	0.563333333
Zfpm2	Q8CCH7	Zinc finger protein ZFPM2 OS=Mus musculus OX=10090 GN=Zfpm2 PE=1 SV=2	
Rcc1l	Q9CYF5	RCC1-like G exchanging factor-like protein OS=Mus musculus OX=10090 GN=Rcc1l PE=1 SV=1	
Trmu	Q9DAT5	Mitochondrial tRNA-specific 2-thiouridylase 1 OS=Mus musculus OX=10090 GN=Trmu PE=1 SV=1	
Cr1l	Q64735	Complement component receptor 1-like protein OS=Mus musculus OX=10090 GN=Cr1l PE=1 SV=1	
Gmeb1	Q9JL60	Glucocorticoid modulatory element-binding protein 1 OS=Mus musculus OX=10090 GN=Gmeb1 PE	0.955
Bet1l	O35153	BET1-like protein OS=Mus musculus OX=10090 GN=Bet1l PE=3 SV=1	0.7
Crls1	Q80ZM8	Cardiolipin synthase (CMP-forming) OS=Mus musculus OX=10090 GN=Crls1 PE=1 SV=1	
Loxl1	P97873	Lysyl oxidase homolog 1 OS=Mus musculus OX=10090 GN=Loxl1 PE=2 SV=3	1.996666667
Trappc6b	Q9D289	Trafficking protein particle complex subunit 6B OS=Mus musculus OX=10090 GN=Trappc6b PE=1 SV	1.815
	Q9CRC3	UPF0235 protein C15orf40 homolog OS=Mus musculus OX=10090 PE=1 SV=1	1.193333333
Atp6ap2	Q9CYN9	Renin receptor OS=Mus musculus OX=10090 GN=Atp6ap2 PE=1 SV=2	0.79
Fars2	Q99M01	Phenylalanine--tRNA ligase mitochondrial OS=Mus musculus OX=10090 GN=Fars2 PE=1 SV=1	
Exd2	Q8VEG4	Exonuclease 3'-5' domain-containing protein 2 OS=Mus musculus OX=10090 GN=Exd2 PE=1 SV=2	0.965
Hsd17b8	P50171	Estradiol 17-beta-dehydrogenase 8 OS=Mus musculus OX=10090 GN=Hsd17b8 PE=1 SV=2	1.345
Strada	Q3UUJ4	STE20-related kinase adapter protein alpha OS=Mus musculus OX=10090 GN=Strada PE=1 SV=1	
Myo19	Q5SV80	Unconventional myosin-XIX OS=Mus musculus OX=10090 GN=Myo19 PE=1 SV=1	
mt-Co3	P00416	Cytochrome c oxidase subunit 3 OS=Mus musculus OX=10090 GN=mt-Co3 PE=1 SV=2	0.993333333
Cd99l2	Q8BIF0	CD99 antigen-like protein 2 OS=Mus musculus OX=10090 GN=Cd99l2 PE=1 SV=1	1.826666667
Cln8	Q9QUK3	Protein CLN8 OS=Mus musculus OX=10090 GN=Cln8 PE=1 SV=1	0.75
Vti1a	O89116	Vesicle transport through interaction with t-SNAREs homolog 1A OS=Mus musculus OX=10090 GN=	1.12
Aktip	Q64362	AKT-interacting protein OS=Mus musculus OX=10090 GN=Aktip PE=1 SV=1	0.32
Tbc1d12	Q6A039	TBC1 domain family member 12 OS=Mus musculus OX=10090 GN=Tbc1d12 PE=1 SV=2	0.905

Scoc	Q78YZ6	Short coiled-coil protein OS=Mus musculus OX=10090 GN=Scoc PE=1 SV=1	1.333333333
Paip2	Q9D6V8	Polyadenylate-binding protein-interacting protein 2 OS=Mus musculus OX=10090 GN=Paip2 PE=1 S	0.843333333
Txndc15	Q6P6J9	Thioredoxin domain-containing protein 15 OS=Mus musculus OX=10090 GN=Txndc15 PE=1 SV=1	1.01
Mtres1	Q9CQF4	Mitochondrial transcription rescue factor 1 OS=Mus musculus OX=10090 GN=Mtres1 PE=1 SV=1	0.89
Trappc2	Q9CQP2	Trafficking protein particle complex subunit 2 OS=Mus musculus OX=10090 GN=Trappc2 PE=1 SV=1	0.83
Ndufb7	Q9CR61	NADH dehydrogenase [ubiquinone] 1 beta subcomplex subunit 7 OS=Mus musculus OX=10090 GN=	0.685
Rhod	P97348	Rho-related GTP-binding protein RhoD OS=Mus musculus OX=10090 GN=Rhod PE=1 SV=1	0.783333333
Cox7a2l	Q61387	Cytochrome c oxidase subunit 7A-related protein mitochondrial OS=Mus musculus OX=10090 GN=	1.203333333
Socs6	Q9JLY0	Suppressor of cytokine signaling 6 OS=Mus musculus OX=10090 GN=Socs6 PE=1 SV=2	0.906666667
Ankrd39	Q9D2X0	Ankyrin repeat domain-containing protein 39 OS=Mus musculus OX=10090 GN=Ankrd39 PE=1 SV=1	1.555
St5	Q924W7	DENN domain-containing protein 2B OS=Mus musculus OX=10090 GN=Dennd2b PE=1 SV=1	0.65
Surf1	P09925	Surfeit locus protein 1 OS=Mus musculus OX=10090 GN=Surf1 PE=1 SV=3	1.186666667
Ostc	Q78XF5	Oligosaccharyltransferase complex subunit OSTC OS=Mus musculus OX=10090 GN=Ostc PE=1 SV=1	0.8
Slc66a3	Q8C6U2	Solute carrier family 66 member 3 OS=Mus musculus OX=10090 GN=Slc66a3 PE=1 SV=1	0.746666667
Cars2	Q8BYM8	Probable cysteine--tRNA ligase mitochondrial OS=Mus musculus OX=10090 GN=Cars2 PE=1 SV=2	0.9
Yipf4	Q8C407	Protein YIPF4 OS=Mus musculus OX=10090 GN=Yipf4 PE=1 SV=1	0.89
Gypc	Q78HU7	Glycophorin-C OS=Mus musculus OX=10090 GN=Gypc PE=1 SV=2	0.68
Bloc1s5	Q8R015	Biogenesis of lysosome-related organelles complex 1 subunit 5 OS=Mus musculus OX=10090 GN=Blo	
Rarg	P18911	Retinoic acid receptor gamma OS=Mus musculus OX=10090 GN=Rarg PE=1 SV=3	
Tbc1d22a	Q8R5A6	TBC1 domain family member 22A OS=Mus musculus OX=10090 GN=Tbc1d22a PE=1 SV=3	0.815
Elof1	P60003	Transcription elongation factor 1 homolog OS=Mus musculus OX=10090 GN=Elof1 PE=1 SV=1	1.15
Zcchc9	Q8R1J3	Zinc finger CCHC domain-containing protein 9 OS=Mus musculus OX=10090 GN=Zcchc9 PE=2 SV=2	0.85
Polr3k	Q9CQZ7	DNA-directed RNA polymerase III subunit RPC10 OS=Mus musculus OX=10090 GN=Polr3k PE=3 SV=1	
Ndufs4	Q9CXZ1	NADH dehydrogenase [ubiquinone] iron-sulfur protein 4 mitochondrial OS=Mus musculus OX=1009	
C2cd5	Q7TPS5	C2 domain-containing protein 5 OS=Mus musculus OX=10090 GN=C2cd5 PE=1 SV=2	
Senp1	P59110	Sentrin-specific protease 1 OS=Mus musculus OX=10090 GN=Senp1 PE=1 SV=1	0.345
Abhd10	Q6PE15	Mycophenolic acid acyl-glucuronide esterase mitochondrial OS=Mus musculus OX=10090 GN=Abh	2.206666667
Serpinf2	Q61247	Alpha-2-antiplasmin OS=Mus musculus OX=10090 GN=Serpinf2 PE=1 SV=1	0.023333333
Tmem237	Q3V0J1	Transmembrane protein 237 OS=Mus musculus OX=10090 GN=Tmem237 PE=1 SV=1	0.92
Peg3	Q3URU2	Paternally-expressed gene 3 protein OS=Mus musculus OX=10090 GN=Peg3 PE=1 SV=1	
Pik3r2	O08908	Phosphatidylinositol 3-kinase regulatory subunit beta OS=Mus musculus OX=10090 GN=Pik3r2 PE=	0.96
Orc2	Q60862	Origin recognition complex subunit 2 OS=Mus musculus OX=10090 GN=Orc2 PE=1 SV=1	1.49
Sepsecs	Q6P6M7	O-phosphoseryl-tRNA(Sec) selenium transferase OS=Mus musculus OX=10090 GN=Sepsecs PE=1 SV=2	
Ggnbp2	Q5SV77	Gametogenetin-binding protein 2 OS=Mus musculus OX=10090 GN=Ggnbp2 PE=1 SV=1	0.9

Magi3	Q9EQJ9	Membrane-associated guanylate kinase WW and PDZ domain-containing protein 3 OS=Mus muscul	
Man1b1	A2AJ15	Endoplasmic reticulum mannosyl-oligosaccharide 1 2-alpha-mannosidase OS=Mus musculus OX=10	
Sirt5	Q8K2C6	NAD-dependent protein deacylase sirtuin-5 mitochondrial OS=Mus musculus OX=10090 GN=Sirt5 I	0.883333333
Rabl2	E9Q9D5	Rab-like protein 2A OS=Mus musculus OX=10090 GN=Rabl2 PE=1 SV=1	2.32
Dhrs13	Q5SS80	Dehydrogenase/reductase SDR family member 13 OS=Mus musculus OX=10090 GN=Dhrs13 PE=1 SV	
Slc29a3	Q99P65	Equilibrative nucleoside transporter 3 OS=Mus musculus OX=10090 GN=Slc29a3 PE=1 SV=1	0.463333333
Gtf2a1	Q99PM3	Transcription initiation factor IIA subunit 1 OS=Mus musculus OX=10090 GN=Gtf2a1 PE=1 SV=2	
Cpeb4	Q7TN98	Cytoplasmic polyadenylation element-binding protein 4 OS=Mus musculus OX=10090 GN=Cpeb4 P	
Pold4	Q9CWP8	DNA polymerase delta subunit 4 OS=Mus musculus OX=10090 GN=Pold4 PE=1 SV=1	
Nfatc4	Q8K120	Nuclear factor of activated T-cells cytoplasmic 4 OS=Mus musculus OX=10090 GN=Nfatc4 PE=1 SV=2	1.85
Tssc4	Q9JHE7	Protein TSSC4 OS=Mus musculus OX=10090 GN=Tssc4 PE=1 SV=1	
Wars2	Q9CYK1	Tryptophan--tRNA ligase mitochondrial OS=Mus musculus OX=10090 GN=Wars2 PE=1 SV=2	
	Q8R3Y5	Uncharacterized protein C19orf47 homolog OS=Mus musculus OX=10090 PE=1 SV=2	0.94
Actr6	Q9D864	Actin-related protein 6 OS=Mus musculus OX=10090 GN=Actr6 PE=1 SV=2	0.45
Mex3c	Q05A36	RNA-binding E3 ubiquitin-protein ligase MEX3C OS=Mus musculus OX=10090 GN=Mex3c PE=2 SV=2	0.585
Gtf2h1	Q9DBA9	General transcription factor IIH subunit 1 OS=Mus musculus OX=10090 GN=Gtf2h1 PE=1 SV=2	
Scamp4	Q9JKV5	Secretory carrier-associated membrane protein 4 OS=Mus musculus OX=10090 GN=Scamp4 PE=1 S\	0.44
Tomm20	Q9DCC8	Mitochondrial import receptor subunit TOM20 homolog OS=Mus musculus OX=10090 GN=Tomm2	0.75
Pole4	Q9CQ36	DNA polymerase epsilon subunit 4 OS=Mus musculus OX=10090 GN=Pole4 PE=1 SV=1	0.583333333
Synpo	Q8CC35	Synaptopodin OS=Mus musculus OX=10090 GN=Synpo PE=1 SV=2	
Eef1akmt2	Q9D853	EEF1A lysine methyltransferase 2 OS=Mus musculus OX=10090 GN=Eef1akmt2 PE=1 SV=1	2.513333333
Thnsl2	Q80W22	Threonine synthase-like 2 OS=Mus musculus OX=10090 GN=Thnsl2 PE=1 SV=1	0.725
Krtcap2	Q5RL79	Keratinocyte-associated protein 2 OS=Mus musculus OX=10090 GN=Krtcap2 PE=1 SV=2	0.77
Ces2c	Q91WG0	Acylcarnitine hydrolase OS=Mus musculus OX=10090 GN=Ces2c PE=1 SV=1	
Akap8l	Q9R0L7	A-kinase anchor protein 8-like OS=Mus musculus OX=10090 GN=Akap8l PE=1 SV=1	
Ptdss1	Q99LH2	Phosphatidylserine synthase 1 OS=Mus musculus OX=10090 GN=Ptdss1 PE=1 SV=1	1.01
Wdtc1	Q80ZK9	WD and tetratricopeptide repeats protein 1 OS=Mus musculus OX=10090 GN=Wdtc1 PE=1 SV=1	1.115
Stat2	Q9WVL2	Signal transducer and activator of transcription 2 OS=Mus musculus OX=10090 GN=Stat2 PE=1 SV=1	
Mtmr12	Q80TA6	Myotubularin-related protein 12 OS=Mus musculus OX=10090 GN=Mtmr12 PE=1 SV=2	
Golga7	Q91W53	Golgin subfamily A member 7 OS=Mus musculus OX=10090 GN=Golga7 PE=1 SV=1	1.286666667
Ercc2	O08811	General transcription and DNA repair factor IIH helicase subunit XPD OS=Mus musculus OX=10090 (	0.813333333
Retreg3	Q9CQV4	Reticulophagy regulator 3 OS=Mus musculus OX=10090 GN=Retreg3 PE=1 SV=1	0.975
Coq4	Q8BGB8	Ubiquinone biosynthesis protein COQ4 homolog mitochondrial OS=Mus musculus OX=10090 GN=(	1.115
Erg28	Q9ERY9	Ergosterol biosynthetic protein 28 homolog OS=Mus musculus OX=10090 GN=Erg28 PE=2 SV=1	0.415

Chac2	Q9CQG1	Putative glutathione-specific gamma-glutamylcyclotransferase 2 OS=Mus musculus OX=10090 GN=(	0.803333333
Usp28	Q5I043	Ubiquitin carboxyl-terminal hydrolase 28 OS=Mus musculus OX=10090 GN=Usp28 PE=1 SV=1	1.4
Tipin	Q91WA1	TIMELESS-interacting protein OS=Mus musculus OX=10090 GN=Tipin PE=1 SV=2	0.746666667
Polrmt	Q8BKF1	DNA-directed RNA polymerase mitochondrial OS=Mus musculus OX=10090 GN=Polrmt PE=1 SV=1	
Synj1	Q8CHC4	Synaptojanin-1 OS=Mus musculus OX=10090 GN=Synj1 PE=1 SV=3	
Vkorc1	Q9CRC0	Vitamin K epoxide reductase complex subunit 1 OS=Mus musculus OX=10090 GN=Vkorc1 PE=1 SV=1	1.243333333
Mpv17	P19258	Protein Mpv17 OS=Mus musculus OX=10090 GN=Mpv17 PE=1 SV=1	
Rnf170	Q8CBG9	E3 ubiquitin-protein ligase RNF170 OS=Mus musculus OX=10090 GN=Rnf170 PE=1 SV=1	
Spag5	Q7TME2	Sperm-associated antigen 5 OS=Mus musculus OX=10090 GN=Spag5 PE=1 SV=1	1.025
Apba3	O88888	Amyloid-beta A4 precursor protein-binding family A member 3 OS=Mus musculus OX=10090 GN=Ap	
Gabpb2	P81069	GA-binding protein subunit beta-2 OS=Mus musculus OX=10090 GN=Gabpb2 PE=1 SV=2	0.92
Alg9	Q8VDI9	Alpha-1 2-mannosyltransferase ALG9 OS=Mus musculus OX=10090 GN=Alg9 PE=2 SV=1	
	Q9CWU4	UPF0690 protein C1orf52 homolog OS=Mus musculus OX=10090 PE=1 SV=1	1.45
Plgrkt	Q9D3P8	Plasminogen receptor (KT) OS=Mus musculus OX=10090 GN=Plgrkt PE=1 SV=1	1.353333333
Pdcl	Q9DBX2	Phosducin-like protein OS=Mus musculus OX=10090 GN=Pdcl PE=1 SV=1	0.53
Lmbrd1	Q8K0B2	Lysosomal cobalamin transport escort protein LMBD1 OS=Mus musculus OX=10090 GN=Lmbrd1 PE	0.866666667
Sesn2	P58043	Sestrin-2 OS=Mus musculus OX=10090 GN=Sesn2 PE=1 SV=1	
Sesn3	Q9CYP7	Sestrin-3 OS=Mus musculus OX=10090 GN=Sesn3 PE=1 SV=1	
Bend3	Q6PAL0	BEN domain-containing protein 3 OS=Mus musculus OX=10090 GN=Bend3 PE=1 SV=1	
Pomp	Q9CQT5	Proteasome maturation protein OS=Mus musculus OX=10090 GN=Pomp PE=1 SV=1	1.093333333
Tmsb10	Q6ZWY8	Thymosin beta-10 OS=Mus musculus OX=10090 GN=Tmsb10 PE=1 SV=3	0.613333333
Cyhr1	Q9QXA1	Cysteine and histidine-rich protein 1 OS=Mus musculus OX=10090 GN=Cyhr1 PE=1 SV=1	0.77
Rgl3	Q3UYI5	Ral guanine nucleotide dissociation stimulator-like 3 OS=Mus musculus OX=10090 GN=Rgl3 PE=1 SV	
Pex7	P97865	Peroxisomal targeting signal 2 receptor OS=Mus musculus OX=10090 GN=Pex7 PE=1 SV=1	1.99
Mtnd3	P03899	NADH-ubiquinone oxidoreductase chain 3 OS=Mus musculus OX=10090 GN=Mtnd3 PE=1 SV=3	0.69
Sppl2a	Q9JJF9	Signal peptide peptidase-like 2A OS=Mus musculus OX=10090 GN=Sppl2a PE=1 SV=2	0.83
Znhit2	Q9QY66	Zinc finger HIT domain-containing protein 2 OS=Mus musculus OX=10090 GN=Znhit2 PE=2 SV=2	
Znf787	Q8BIF9	Zinc finger protein 787 OS=Mus musculus OX=10090 GN=Znf787 PE=2 SV=3	
Mmaa	Q8C7H1	Methylmalonic aciduria type A homolog mitochondrial OS=Mus musculus OX=10090 GN=Mmaa PE	
Smg5	Q6ZPY2	Protein SMG5 OS=Mus musculus OX=10090 GN=Smg5 PE=1 SV=2	1.54
Inpp5f	Q8CDA1	Phosphatidylinositide phosphatase SAC2 OS=Mus musculus OX=10090 GN=Inpp5f PE=1 SV=1	
Gdpd1	Q9CRY7	Lysophospholipase D GDPD1 OS=Mus musculus OX=10090 GN=Gdpd1 PE=1 SV=1	1.57
Bcl2	P10417	Apoptosis regulator Bcl-2 OS=Mus musculus OX=10090 GN=Bcl2 PE=1 SV=3	0.986666667
Znrf2	Q71FD5	E3 ubiquitin-protein ligase ZNRF2 OS=Mus musculus OX=10090 GN=Znrf2 PE=1 SV=1	

Cmtm7	Q9ESD6	CKLF-like MARVEL transmembrane domain-containing protein 7 OS=Mus musculus OX=10090 GN=(	0.68
Ddx58	Q6Q899	Antiviral innate immune response receptor RIG-I OS=Mus musculus OX=10090 GN=Ddx58 PE=1 SV=:	0.99
Hus1	Q8BQY8	Checkpoint protein HUS1 OS=Mus musculus OX=10090 GN=Hus1 PE=2 SV=1	
Dpm3	Q9D1Q4	Dolichol-phosphate mannosyltransferase subunit 3 OS=Mus musculus OX=10090 GN=Dpm3 PE=1 S'	1.113333333
Gan	Q8CA72	Gigaxonin OS=Mus musculus OX=10090 GN=Gan PE=1 SV=2	1.22
	Q5XFZ0	UPF0711 protein C18orf21 homolog OS=Mus musculus OX=10090 PE=2 SV=2	1.08
Plac8	Q9JI48	Placenta-specific gene 8 protein OS=Mus musculus OX=10090 GN=Plac8 PE=1 SV=1	1.356666667
Slc11a2	P49282	Natural resistance-associated macrophage protein 2 OS=Mus musculus OX=10090 GN=Slc11a2 PE=1	
Ankib1	Q6ZPS6	Ankyrin repeat and IBR domain-containing protein 1 OS=Mus musculus OX=10090 GN=Ankib1 PE=1	
Mid1	O70583	E3 ubiquitin-protein ligase Midline-1 OS=Mus musculus OX=10090 GN=Mid1 PE=1 SV=2	0.815
Btg1	P62325	Protein BTG1 OS=Mus musculus OX=10090 GN=Btg1 PE=1 SV=1	1.385
Acp2	P24638	Lysosomal acid phosphatase OS=Mus musculus OX=10090 GN=Acp2 PE=1 SV=2	0.906666667
Calhm2	Q8VEC4	Calcium homeostasis modulator protein 2 OS=Mus musculus OX=10090 GN=Calhm2 PE=2 SV=1	0.85
Yeats2	Q3TUF7	YEATS domain-containing protein 2 OS=Mus musculus OX=10090 GN=Yeats2 PE=1 SV=2	
Slc25a51	Q5HZI9	Solute carrier family 25 member 51 OS=Mus musculus OX=10090 GN=Slc25a51 PE=1 SV=1	0.853333333
Tsc2	Q61037	Tuberin OS=Mus musculus OX=10090 GN=Tsc2 PE=1 SV=1	
Pimreg	Q8BFY7	Protein PIMREG OS=Mus musculus OX=10090 GN=Pimreg PE=1 SV=1	0.495
Gtf2h3	Q8VD76	General transcription factor IIH subunit 3 OS=Mus musculus OX=10090 GN=Gtf2h3 PE=1 SV=1	
Chka	O54804	Choline kinase alpha OS=Mus musculus OX=10090 GN=Chka PE=1 SV=3	
Leprot	O89013	Leptin receptor gene-related protein OS=Mus musculus OX=10090 GN=Leprot PE=1 SV=1	
Secisbp2l	Q6A098	Selenocysteine insertion sequence-binding protein 2-like OS=Mus musculus OX=10090 GN=Secisbp:	1.635
Ccdc137	Q8R0K4	Coiled-coil domain-containing protein 137 OS=Mus musculus OX=10090 GN=Ccdc137 PE=2 SV=1	
Mcee	Q9D1I5	Methylmalonyl-CoA epimerase mitochondrial OS=Mus musculus OX=10090 GN=Mcee PE=1 SV=1	0.75
Serpina3f	Q80X76	Serine protease inhibitor A3F OS=Mus musculus OX=10090 GN=Serpina3f PE=1 SV=3	0.53
Bin3	Q9JI08	Bridging integrator 3 OS=Mus musculus OX=10090 GN=Bin3 PE=1 SV=1	
Rgn	Q64374	Regucalcin OS=Mus musculus OX=10090 GN=Rgn PE=1 SV=1	0.856666667
Fkbp14	P59024	Peptidyl-prolyl cis-trans isomerase FKBP14 OS=Mus musculus OX=10090 GN=Fkbp14 PE=1 SV=1	1.275
Fyttd1	Q91Z49	UAP56-interacting factor OS=Mus musculus OX=10090 GN=Fyttd1 PE=1 SV=1	1.156666667
Slc33a1	Q99J27	Acetyl-coenzyme A transporter 1 OS=Mus musculus OX=10090 GN=Slc33a1 PE=1 SV=1	1.086666667
Tor2a	P0C7W3	Prosalusin OS=Mus musculus OX=10090 GN=Tor2a PE=3 SV=1	
Tor2a	Q8R1J9	Torsin-2A OS=Mus musculus OX=10090 GN=Tor2a PE=1 SV=1	
Fgfr1op2	Q9CRA9	FGFR1 oncogene partner 2 homolog OS=Mus musculus OX=10090 GN=Fgfr1op2 PE=1 SV=1	
Chmp1a	Q921W0	Charged multivesicular body protein 1a OS=Mus musculus OX=10090 GN=Chmp1a PE=1 SV=1	0.7
Pja1	O55176	E3 ubiquitin-protein ligase Praja-1 OS=Mus musculus OX=10090 GN=Pja1 PE=1 SV=3	

Arrb1	Q8BWG8	Beta-arrestin-1 OS=Mus musculus OX=10090 GN=Arrb1 PE=1 SV=1	2.515
Slc30a5	Q8R4H9	Zinc transporter 5 OS=Mus musculus OX=10090 GN=Slc30a5 PE=1 SV=1	
Fbxo5	Q7TSG3	F-box only protein 5 OS=Mus musculus OX=10090 GN=Fbxo5 PE=1 SV=1	0.993333333
Tm4sf1	Q64302	Transmembrane 4 L6 family member 1 OS=Mus musculus OX=10090 GN=Tm4sf1 PE=1 SV=1	0.926666667
Crcp	O35427	DNA-directed RNA polymerase III subunit RPC9 OS=Mus musculus OX=10090 GN=Crcp PE=2 SV=1	0.823333333
Znf346	Q9R0B7	Zinc finger protein 346 OS=Mus musculus OX=10090 GN=Znf346 PE=1 SV=1	0.9
Vps39	Q8R5L3	Vam6/Vps39-like protein OS=Mus musculus OX=10090 GN=Vps39 PE=1 SV=1	1.27
Elf2	Q9JHC9	ETS-related transcription factor Elf-2 OS=Mus musculus OX=10090 GN=Elf2 PE=1 SV=1	
Ssna1	Q9JJ94	Sjoegren syndrome nuclear autoantigen 1 homolog OS=Mus musculus OX=10090 GN=Ssna1 PE=1 S\	1.003333333
Mfsd5	Q921Y4	Molybdate-anion transporter OS=Mus musculus OX=10090 GN=Mfsd5 PE=2 SV=1	0.425
Klf4	Q60793	Krueppel-like factor 4 OS=Mus musculus OX=10090 GN=Klf4 PE=1 SV=3	0.735
Khdc4	Q3TCX3	KH homology domain-containing protein 4 OS=Mus musculus OX=10090 GN=Khdc4 PE=1 SV=1	
Tbp	P29037	TATA-box-binding protein OS=Mus musculus OX=10090 GN=Tbp PE=1 SV=1	0.5
Fbxl8	Q8CIG9	F-box/LRR-repeat protein 8 OS=Mus musculus OX=10090 GN=Fbxl8 PE=1 SV=1	0.99
Cmtr2	Q8BWQ4	Cap-specific mRNA (nucleoside-2'-O-)-methyltransferase 2 OS=Mus musculus OX=10090 GN=Cmtr2 I	
Epb41l5	Q8BGS1	Band 4.1-like protein 5 OS=Mus musculus OX=10090 GN=Epb41l5 PE=1 SV=1	
Ccdc90b	Q8C3X2	Coiled-coil domain-containing protein 90B mitochondrial OS=Mus musculus OX=10090 GN=Ccdc9	0.86
Crtc1	Q68ED7	CREB-regulated transcription coactivator 1 OS=Mus musculus OX=10090 GN=Crtc1 PE=1 SV=1	0.766666667
Kmt2a	P55200	Histone-lysine N-methyltransferase 2A OS=Mus musculus OX=10090 GN=Kmt2a PE=1 SV=3	
Hscb	Q8K3A0	Iron-sulfur cluster co-chaperone protein HscB OS=Mus musculus OX=10090 GN=Hscb PE=1 SV=2	
Mtbp	Q8BJS8	Mdm2-binding protein OS=Mus musculus OX=10090 GN=Mtbp PE=1 SV=1	
Sap130	Q8BIH0	Histone deacetylase complex subunit SAP130 OS=Mus musculus OX=10090 GN=Sap130 PE=1 SV=2	3.49
Sox9	Q04887	Transcription factor SOX-9 OS=Mus musculus OX=10090 GN=Sox9 PE=1 SV=2	
Ip6k1	Q6PD10	Inositol hexakisphosphate kinase 1 OS=Mus musculus OX=10090 GN=Ip6k1 PE=2 SV=1	
Ankrd54	Q91WK7	Ankyrin repeat domain-containing protein 54 OS=Mus musculus OX=10090 GN=Ankrd54 PE=1 SV=1	0.666666667
Gale	Q8R059	UDP-glucose 4-epimerase OS=Mus musculus OX=10090 GN=Gale PE=1 SV=1	0.965
Atad5	Q4QY64	ATPase family AAA domain-containing protein 5 OS=Mus musculus OX=10090 GN=Atad5 PE=1 SV=1	1.125
Rnf123	Q5XPI3	E3 ubiquitin-protein ligase RNF123 OS=Mus musculus OX=10090 GN=Rnf123 PE=1 SV=1	0.005
Osr1	Q9WVG7	Protein odd-skipped-related 1 OS=Mus musculus OX=10090 GN=Osr1 PE=1 SV=1	0.875
Mvb12b	Q6KAU4	Multivesicular body subunit 12B OS=Mus musculus OX=10090 GN=Mvb12b PE=1 SV=2	1.56
Arhgap11a	Q80Y19	Rho GTPase-activating protein 11A OS=Mus musculus OX=10090 GN=Arhgap11a PE=1 SV=2	1.08
Pex16	Q91XC9	Peroxisomal membrane protein PEX16 OS=Mus musculus OX=10090 GN=Pex16 PE=1 SV=2	
Tgoln1	Q62313	Trans-Golgi network integral membrane protein 1 OS=Mus musculus OX=10090 GN=Tgoln1 PE=1 SV	
Tgoln2	Q62314	Trans-Golgi network integral membrane protein 2 OS=Mus musculus OX=10090 GN=Tgoln2 PE=1 SV	

Fez2	Q6TYB5	Fasciculation and elongation protein zeta-2 OS=Mus musculus OX=10090 GN=Fez2 PE=2 SV=1	0.976666667
Synj2bp	Q9D6K5	Synaptojanin-2-binding protein OS=Mus musculus OX=10090 GN=Synj2bp PE=1 SV=1	1.626666667
Cldnd1	Q9CQX5	Claudin domain-containing protein 1 OS=Mus musculus OX=10090 GN=Cldnd1 PE=1 SV=1	0.946666667
Serpind1	P49182	Heparin cofactor 2 OS=Mus musculus OX=10090 GN=Serpind1 PE=1 SV=1	0.076666667
Atp7a	Q64430	Copper-transporting ATPase 1 OS=Mus musculus OX=10090 GN=Atp7a PE=1 SV=3	
Npc1	O35604	NPC intracellular cholesterol transporter 1 OS=Mus musculus OX=10090 GN=Npc1 PE=1 SV=2	1.743333333
Klf13	Q9JJZ6	Krueppel-like factor 13 OS=Mus musculus OX=10090 GN=Klf13 PE=1 SV=2	0.695
Atp6v1f	Q9D1K2	V-type proton ATPase subunit F OS=Mus musculus OX=10090 GN=Atp6v1f PE=1 SV=2	1.416666667
Zcchc10	Q9CX48	Zinc finger CCHC domain-containing protein 10 OS=Mus musculus OX=10090 GN=Zcchc10 PE=2 SV=	0.91
Nrm	Q8VC65	Nurim OS=Mus musculus OX=10090 GN=Nrm PE=1 SV=1	1.283333333
Ccnc	Q62447	Cyclin-C OS=Mus musculus OX=10090 GN=Ccnc PE=2 SV=4	
Fam168a	Q8BGZ2	Protein FAM168A OS=Mus musculus OX=10090 GN=Fam168a PE=1 SV=1	
Gse1	Q3U3C9	Genetic suppressor element 1 OS=Mus musculus OX=10090 GN=Gse1 PE=1 SV=2	
Fau	P62862	40S ribosomal protein S30 OS=Mus musculus OX=10090 GN=Fau PE=1 SV=1	1.243333333
Dnaaf2	Q8BPI1	Protein kintoun OS=Mus musculus OX=10090 GN=Dnaaf2 PE=1 SV=2	
Dap	Q91XC8	Death-associated protein 1 OS=Mus musculus OX=10090 GN=Dap PE=1 SV=3	0.506666667
Acad11	Q80XL6	Acyl-CoA dehydrogenase family member 11 OS=Mus musculus OX=10090 GN=Acad11 PE=1 SV=2	
Bbx	Q8VBW5	HMG box transcription factor BBX OS=Mus musculus OX=10090 GN=Bbx PE=1 SV=2	
Pttg1ip	Q8R143	Pituitary tumor-transforming gene 1 protein-interacting protein OS=Mus musculus OX=10090 GN=	0.866666667
Atpaf1	Q811I0	ATP synthase mitochondrial F1 complex assembly factor 1 OS=Mus musculus OX=10090 GN=Atpaf1	1.18
Eva1b	Q8K2Y3	Protein eva-1 homolog B OS=Mus musculus OX=10090 GN=Eva1b PE=1 SV=1	0.285
Alkbh8	Q80Y20	Alkylated DNA repair protein alkB homolog 8 OS=Mus musculus OX=10090 GN=Alkbh8 PE=1 SV=1	0.345
Mif4gd	Q3UBZ5	MIF4G domain-containing protein OS=Mus musculus OX=10090 GN=Mif4gd PE=1 SV=1	
Fam89b	Q9QUI1	Leucine repeat adapter protein 25 OS=Mus musculus OX=10090 GN=Fam89b PE=1 SV=1	
Psmb8	P28063	Proteasome subunit beta type-8 OS=Mus musculus OX=10090 GN=Psmb8 PE=1 SV=2	
Ubac2	Q8R1K1	Ubiquitin-associated domain-containing protein 2 OS=Mus musculus OX=10090 GN=Ubac2 PE=1 S\	
	Q922R1	UPF0183 protein C16orf70 homolog OS=Mus musculus OX=10090 PE=1 SV=2	
Slc25a20	Q9Z2Z6	Mitochondrial carnitine/acylcarnitine carrier protein OS=Mus musculus OX=10090 GN=Slc25a20 P	0.91
Ttpal	Q9D3D0	Alpha-tocopherol transfer protein-like OS=Mus musculus OX=10090 GN=Ttpal PE=1 SV=3	
Zfyve21	Q8VCM3	Zinc finger FYVE domain-containing protein 21 OS=Mus musculus OX=10090 GN=Zfyve21 PE=1 SV=2	
Mcur1	Q9CXD6	Mitochondrial calcium uniporter regulator 1 OS=Mus musculus OX=10090 GN=Mcur1 PE=1 SV=1	0.376666667
Nubpl	Q9CWD8	Iron-sulfur protein NUBPL OS=Mus musculus OX=10090 GN=Nubpl PE=1 SV=2	0.516666667
Adat1	Q9JHI2	tRNA-specific adenosine deaminase 1 OS=Mus musculus OX=10090 GN=Adat1 PE=1 SV=1	0.845
Cdkn1a	P39689	Cyclin-dependent kinase inhibitor 1 OS=Mus musculus OX=10090 GN=Cdkn1a PE=1 SV=4	0.8

Sdhd	Q9CXV1	Succinate dehydrogenase [ubiquinone] cytochrome b small subunit mitochondrial OS=Mus muscul	0.966666667
Pik3ca	P42337	Phosphatidylinositol 4 5-bisphosphate 3-kinase catalytic subunit alpha isoform OS=Mus musculus (	0.89
Tsen15	Q8R3W5	tRNA-splicing endonuclease subunit Sen15 OS=Mus musculus OX=10090 GN=Tsen15 PE=1 SV=1	0.946666667
Cep63	Q3UPP8	Centrosomal protein of 63 kDa OS=Mus musculus OX=10090 GN=Cep63 PE=1 SV=2	
Anapc13	Q8R034	Anaphase-promoting complex subunit 13 OS=Mus musculus OX=10090 GN=Anapc13 PE=3 SV=1	1.086666667
Gjb3	P28231	Gap junction beta-3 protein OS=Mus musculus OX=10090 GN=Gjb3 PE=1 SV=1	1.29
Ndufb1	P0DN34	NADH dehydrogenase [ubiquinone] 1 beta subcomplex subunit 1 OS=Mus musculus OX=10090 GN=	1.056666667
Pnisr	A2AJT4	Arginine/serine-rich protein PNISR OS=Mus musculus OX=10090 GN=Pnisr PE=1 SV=1	
Prss2	P07146	Anionic trypsin-2 OS=Mus musculus OX=10090 GN=Prss2 PE=1 SV=1	0.486666667
Slc16a13	Q8CE94	Monocarboxylate transporter 13 OS=Mus musculus OX=10090 GN=Slc16a13 PE=2 SV=1	0.55
Fam126a	Q6P9N1	Hyccin OS=Mus musculus OX=10090 GN=Fam126a PE=1 SV=3	1.025
Ggh	Q9Z0L8	Gamma-glutamyl hydrolase OS=Mus musculus OX=10090 GN=Ggh PE=1 SV=2	0.69
Rdh13	Q8CEE7	Retinol dehydrogenase 13 OS=Mus musculus OX=10090 GN=Rdh13 PE=1 SV=1	1.236666667
Clk4	O35493	Dual specificity protein kinase CLK4 OS=Mus musculus OX=10090 GN=Clk4 PE=1 SV=1	
Sac3d1	A6H687	SAC3 domain-containing protein 1 OS=Mus musculus OX=10090 GN=Sac3d1 PE=1 SV=1	
Cbx8	Q9QXV1	Chromobox protein homolog 8 OS=Mus musculus OX=10090 GN=Cbx8 PE=1 SV=1	0.69
Dtnbp1	Q91WZ8	Dysbindin OS=Mus musculus OX=10090 GN=Dtnbp1 PE=1 SV=1	
Haus4	Q8BFT2	HAUS augmin-like complex subunit 4 OS=Mus musculus OX=10090 GN=Haus4 PE=1 SV=1	0.986666667
Rapgef2	Q8CHG7	Rap guanine nucleotide exchange factor 2 OS=Mus musculus OX=10090 GN=Rapgef2 PE=1 SV=2	
Sdhaf4	Q8BTE0	Succinate dehydrogenase assembly factor 4 mitochondrial OS=Mus musculus OX=10090 GN=Sdhaf	0.92
Phka2	Q8BWJ3	Phosphorylase b kinase regulatory subunit alpha liver isoform OS=Mus musculus OX=10090 GN=Ph	
Cd276	Q8VE98	CD276 antigen OS=Mus musculus OX=10090 GN=Cd276 PE=1 SV=1	0.925
Slc25a15	Q9WVD5	Mitochondrial ornithine transporter 1 OS=Mus musculus OX=10090 GN=Slc25a15 PE=1 SV=1	1.24
Slc35b2	Q91ZN5	Adenosine 3'-phospho 5'-phosphosulfate transporter 1 OS=Mus musculus OX=10090 GN=Slc35b2 P	0.99
Rftn2	Q8CHX7	Raftlin-2 OS=Mus musculus OX=10090 GN=Rftn2 PE=1 SV=3	1.436666667
Dmxl1	Q6PNC0	DmX-like protein 1 OS=Mus musculus OX=10090 GN=Dmxl1 PE=1 SV=1	
Bnip3l	Q9Z2F7	BCL2/adenovirus E1B 19 kDa protein-interacting protein 3-like OS=Mus musculus OX=10090 GN=Br	
Magee1	Q6PCZ4	Melanoma-associated antigen E1 OS=Mus musculus OX=10090 GN=Magee1 PE=1 SV=1	12.56333333
Wdr76	A6PWY4	WD repeat-containing protein 76 OS=Mus musculus OX=10090 GN=Wdr76 PE=1 SV=1	
Gsdme	Q9Z2D3	Gasdermin-E OS=Mus musculus OX=10090 GN=Gsdme PE=1 SV=1	
Rabac1	Q9Z0S9	Prenylated Rab acceptor protein 1 OS=Mus musculus OX=10090 GN=Rabac1 PE=1 SV=1	0.95
Med13	Q5SWW4	Mediator of RNA polymerase II transcription subunit 13 OS=Mus musculus OX=10090 GN=Med13 PI	
Rbpms	Q9WVB0	RNA-binding protein with multiple splicing OS=Mus musculus OX=10090 GN=Rbpms PE=1 SV=2	1.155
Qsox1	Q8BND5	Sulfhydryl oxidase 1 OS=Mus musculus OX=10090 GN=Qsox1 PE=1 SV=1	0.133333333

Taf5	Q8C092	Transcription initiation factor TFIID subunit 5 OS=Mus musculus OX=10090 GN=Taf5 PE=1 SV=1	
Pcdh19	Q80TF3	Protocadherin-19 OS=Mus musculus OX=10090 GN=Pcdh19 PE=1 SV=3	1.44
Borcs6	Q9D6W8	BLOC-1-related complex subunit 6 OS=Mus musculus OX=10090 GN=Borcs6 PE=1 SV=1	
Aqp1	Q02013	Aquaporin-1 OS=Mus musculus OX=10090 GN=Aqp1 PE=1 SV=3	0.9
Eml2	Q7TNG5	Echinoderm microtubule-associated protein-like 2 OS=Mus musculus OX=10090 GN=Eml2 PE=1 SV=	
Prmt6	Q6NZB1	Protein arginine N-methyltransferase 6 OS=Mus musculus OX=10090 GN=Prmt6 PE=1 SV=2	
Apoa1	Q00623	Apolipoprotein A-I OS=Mus musculus OX=10090 GN=Apoa1 PE=1 SV=2	0.066666667
Hira	Q61666	Protein HIRA OS=Mus musculus OX=10090 GN=Hira PE=1 SV=3	0.11
Mrps18a	Q99N85	28S ribosomal protein S18a mitochondrial OS=Mus musculus OX=10090 GN=Mrps18a PE=2 SV=1	1.416666667
Bloc1s2	Q9CWG9	Biogenesis of lysosome-related organelles complex 1 subunit 2 OS=Mus musculus OX=10090 GN=Bl	
Taf2	Q8C176	Transcription initiation factor TFIID subunit 2 OS=Mus musculus OX=10090 GN=Taf2 PE=2 SV=2	0.915
Polr3d	Q91WD1	DNA-directed RNA polymerase III subunit RPC4 OS=Mus musculus OX=10090 GN=Polr3d PE=2 SV=2	
Tmsb4x	P20065	Thymosin beta-4 OS=Mus musculus OX=10090 GN=Tmsb4x PE=1 SV=1	2.91
Nsl1	Q8K305	Kinetochore-associated protein NSL1 homolog OS=Mus musculus OX=10090 GN=Nsl1 PE=2 SV=1	
Znf706	Q9D115	Zinc finger protein 706 OS=Mus musculus OX=10090 GN=Znf706 PE=1 SV=1	1.49
Hsdl2	Q2TPA8	Hydroxysteroid dehydrogenase-like protein 2 OS=Mus musculus OX=10090 GN=Hsdl2 PE=1 SV=1	0.51
FRRS1	Q8K385	Ferric-chelate reductase 1 OS=Mus musculus OX=10090 GN=FRRS1 PE=1 SV=1	1.11
Ca5b	Q9QZA0	Carbonic anhydrase 5B mitochondrial OS=Mus musculus OX=10090 GN=Ca5b PE=1 SV=2	0.69
Fam160a2	Q3U2I3	FTS and Hook-interacting protein OS=Mus musculus OX=10090 GN=Fam160a2 PE=1 SV=2	
St3gal5	O88829	Lactosylceramide alpha-2 3-sialyltransferase OS=Mus musculus OX=10090 GN=St3gal5 PE=2 SV=2	
Tsen54	Q8C2A2	tRNA-splicing endonuclease subunit Sen54 OS=Mus musculus OX=10090 GN=Tsen54 PE=2 SV=2	
Cir1	Q9DA19	Corepressor interacting with RBPJ 1 OS=Mus musculus OX=10090 GN=Cir1 PE=1 SV=2	1.386666667
Itpripl1	A2ASA8	Inositol 1 4 5-trisphosphate receptor-interacting protein-like 1 OS=Mus musculus OX=10090 GN=It	
Atxn3	Q9CVD2	Ataxin-3 OS=Mus musculus OX=10090 GN=Atxn3 PE=1 SV=2	
Znrd1	Q791N7	DNA-directed RNA polymerase I subunit RPA12 OS=Mus musculus OX=10090 GN=Polr1h PE=1 SV=1	1.743333333
Zbtb8os	Q505B7	Protein archease OS=Mus musculus OX=10090 GN=Zbtb8os PE=1 SV=2	1.08
Atp5mpl	P56379	ATP synthase subunit ATP5MPL mitochondrial OS=Mus musculus OX=10090 GN=Atp5mpl PE=1 SV=	0.88
Lgals8	Q9JL15	Galectin-8 OS=Mus musculus OX=10090 GN=Lgals8 PE=1 SV=1	
Cited2	O35740	Cbp/p300-interacting transactivator 2 OS=Mus musculus OX=10090 GN=Cited2 PE=1 SV=2	2.84
Col4a2	P08122	Collagen alpha-2(IV) chain OS=Mus musculus OX=10090 GN=Col4a2 PE=1 SV=4	
Rpl37	Q9D823	60S ribosomal protein L37 OS=Mus musculus OX=10090 GN=Rpl37 PE=3 SV=3	0.79
Vrk3	Q8K3G5	Inactive serine/threonine-protein kinase VRK3 OS=Mus musculus OX=10090 GN=Vrk3 PE=1 SV=2	
Slc39a11	Q8BWY7	Zinc transporter ZIP11 OS=Mus musculus OX=10090 GN=Slc39a11 PE=1 SV=1	
Dync2h1	Q45VK7	Cytoplasmic dynein 2 heavy chain 1 OS=Mus musculus OX=10090 GN=Dync2h1 PE=1 SV=1	0.9

Trafd1	Q3UDK1	TRAF-type zinc finger domain-containing protein 1 OS=Mus musculus OX=10090 GN=Trafd1 PE=1 SV	
Nckipsd	Q9ESJ4	NCK-interacting protein with SH3 domain OS=Mus musculus OX=10090 GN=Nckipsd PE=1 SV=2	
Paip2b	Q91W45	Polyadenylate-binding protein-interacting protein 2B OS=Mus musculus OX=10090 GN=Paip2b PE=	0.57
Pcgf5	Q3UK78	Polycomb group RING finger protein 5 OS=Mus musculus OX=10090 GN=Pcgf5 PE=1 SV=1	0.67
Ggta1	P23336	N-acetyllactosaminide alpha-1 3-galactosyltransferase OS=Mus musculus OX=10090 GN=Ggta1 PE=2	1.036666667
Midn	Q3TPJ7	Midnolin OS=Mus musculus OX=10090 GN=Midn PE=2 SV=1	
Cotl1	Q9CQI6	Coactosin-like protein OS=Mus musculus OX=10090 GN=Cotl1 PE=1 SV=3	0.856666667
Bap18	Q9DCT6	Chromatin complexes subunit BAP18 OS=Mus musculus OX=10090 GN=Bap18 PE=1 SV=1	0.97
Hpx	Q91X72	Hemopexin OS=Mus musculus OX=10090 GN=Hpx PE=1 SV=2	0.073333333
Rpusd2	Q149F1	RNA pseudouridylate synthase domain-containing protein 2 OS=Mus musculus OX=10090 GN=Rpus	
Pank4	Q80YV4	4'-phosphopantetheine phosphatase OS=Mus musculus OX=10090 GN=Pank4 PE=1 SV=2	
Flcn	Q8QZS3	Folliculin OS=Mus musculus OX=10090 GN=Flcn PE=1 SV=1	0.62
Ccdc80	Q8R2G6	Coiled-coil domain-containing protein 80 OS=Mus musculus OX=10090 GN=Ccdc80 PE=1 SV=2	0.566666667
Ptgr2	Q8VDQ1	Prostaglandin reductase 2 OS=Mus musculus OX=10090 GN=Ptgr2 PE=1 SV=2	
Tmbim1	Q8BJZ3	Protein lifeguard 3 OS=Mus musculus OX=10090 GN=Tmbim1 PE=1 SV=1	
Faap100	A2ACJ2	Fanconi anemia core complex-associated protein 100 OS=Mus musculus OX=10090 GN=Faap100 PE	
Maf1	Q9D0U6	Repressor of RNA polymerase III transcription MAF1 homolog OS=Mus musculus OX=10090 GN=Maf	
Ndufa3	Q9CQ91	NADH dehydrogenase [ubiquinone] 1 alpha subcomplex subunit 3 OS=Mus musculus OX=10090 GN	0.593333333
Ripk2	P58801	Receptor-interacting serine/threonine-protein kinase 2 OS=Mus musculus OX=10090 GN=Ripk2 PE=	
Procr	Q64695	Endothelial protein C receptor OS=Mus musculus OX=10090 GN=Procr PE=1 SV=3	
Hdac10	Q6P3E7	Polyamine deacetylase HDAC10 OS=Mus musculus OX=10090 GN=Hdac10 PE=2 SV=2	0.925
Oard1	Q8R5F3	ADP-ribose glycohydrolase OARD1 OS=Mus musculus OX=10090 GN=Oard1 PE=1 SV=2	1.603333333
Crtc3	Q91X84	CREB-regulated transcription coactivator 3 OS=Mus musculus OX=10090 GN=Crtc3 PE=1 SV=2	
Cmtm4	Q8CJ61	CKLF-like MARVEL transmembrane domain-containing protein 4 OS=Mus musculus OX=10090 GN=(	
Serinc3	Q9QZI9	Serine incorporator 3 OS=Mus musculus OX=10090 GN=Serinc3 PE=1 SV=2	0.895
Acap1	Q8K2H4	Arf-GAP with coiled-coil ANK repeat and PH domain-containing protein 1 OS=Mus musculus OX=10	170.0066667
Wwtr1	Q9EPK5	WW domain-containing transcription regulator protein 1 OS=Mus musculus OX=10090 GN=Wwtr1	3.015
Anapc10	Q8K2H6	Anaphase-promoting complex subunit 10 OS=Mus musculus OX=10090 GN=Anapc10 PE=1 SV=1	0.765
Mier1	Q5UAK0	Mesoderm induction early response protein 1 OS=Mus musculus OX=10090 GN=Mier1 PE=1 SV=2	
Trim44	Q9QXA7	Tripartite motif-containing protein 44 OS=Mus musculus OX=10090 GN=Trim44 PE=1 SV=2	2.135
Nudt7	Q99P30	Peroxisomal coenzyme A diphosphatase NUDT7 OS=Mus musculus OX=10090 GN=Nudt7 PE=1 SV=2	1.175
Trim59	Q922Y2	Tripartite motif-containing protein 59 OS=Mus musculus OX=10090 GN=Trim59 PE=1 SV=2	
Fam92a	Q8BP22	Protein FAM92A OS=Mus musculus OX=10090 GN=Fam92a PE=1 SV=1	
Tfb1m	Q8JZM0	Dimethyladenosine transferase 1 mitochondrial OS=Mus musculus OX=10090 GN=Tfb1m PE=1 SV=1	

Ccnd2	P30280	G1/S-specific cyclin-D2 OS=Mus musculus OX=10090 GN=Ccnd2 PE=1 SV=1	
Rtkn2	Q14B46	Rhotekin-2 OS=Mus musculus OX=10090 GN=Rtkn2 PE=1 SV=2	
Fhdc1	Q3ULZ2	FH2 domain-containing protein 1 OS=Mus musculus OX=10090 GN=Fhdc1 PE=1 SV=3	
Zbtb33	Q8BN78	Transcriptional regulator Kaiso OS=Mus musculus OX=10090 GN=Zbtb33 PE=1 SV=1	
Card19	Q9D1I2	Caspase recruitment domain-containing protein 19 OS=Mus musculus OX=10090 GN=Card19 PE=1	1.456666667
Mcoln1	Q99J21	Mucolipin-1 OS=Mus musculus OX=10090 GN=Mcoln1 PE=1 SV=1	
	Q91W34	RUS family member 1 OS=Mus musculus OX=10090 GN=Rusf1 PE=1 SV=1	1.24
Tbc1d13	Q8R3D1	TBC1 domain family member 13 OS=Mus musculus OX=10090 GN=Tbc1d13 PE=1 SV=1	1.99
Iqcb1	Q8BP00	IQ calmodulin-binding motif-containing protein 1 OS=Mus musculus OX=10090 GN=Iqcb1 PE=1 SV=	1.08
Yipf6	Q8BR70	Protein YIPF6 OS=Mus musculus OX=10090 GN=Yipf6 PE=1 SV=1	
Spred1	Q924S8	Sprouty-related EVH1 domain-containing protein 1 OS=Mus musculus OX=10090 GN=Spred1 PE=1	
Mrpl17	Q9D8P4	39S ribosomal protein L17 mitochondrial OS=Mus musculus OX=10090 GN=Mrpl17 PE=1 SV=1	1.966666667
Msantd2	Q6NZR2	Myb/SANT-like DNA-binding domain-containing protein 2 OS=Mus musculus OX=10090 GN=Msantc	
Abcd1	P48410	ATP-binding cassette sub-family D member 1 OS=Mus musculus OX=10090 GN=Abcd1 PE=1 SV=1	1.7
Prr11	Q8BHE0	Proline-rich protein 11 OS=Mus musculus OX=10090 GN=Prr11 PE=2 SV=1	
Taf6l	Q8R2K4	TAF6-like RNA polymerase II p300/CBP-associated factor-associated factor 65 kDa subunit 6L OS=Mu	2.035
Zfp39	Q02525	Zinc finger protein 39 OS=Mus musculus OX=10090 GN=Zfp39 PE=2 SV=2	
Ubtd2	Q6PGH0	Ubiquitin domain-containing protein 2 OS=Mus musculus OX=10090 GN=Ubtd2 PE=2 SV=1	5.56
	Q5EBG8	Uncharacterized protein C1orf50 homolog OS=Mus musculus OX=10090 PE=1 SV=1	1.033333333
Arl6ip1	Q9JKW0	ADP-ribosylation factor-like protein 6-interacting protein 1 OS=Mus musculus OX=10090 GN=Arl6ip	0.833333333
Bloc1s4	Q8VED2	Biogenesis of lysosome-related organelles complex 1 subunit 4 OS=Mus musculus OX=10090 GN=Blo	
Smdt1	Q9DB10	Essential MCU regulator mitochondrial OS=Mus musculus OX=10090 GN=Smdt1 PE=1 SV=1	0.77
Tspan9	Q8BJU2	Tetraspanin-9 OS=Mus musculus OX=10090 GN=Tspan9 PE=1 SV=1	4.743333333
Cnot6l	Q8VEG6	CCR4-NOT transcription complex subunit 6-like OS=Mus musculus OX=10090 GN=Cnot6l PE=1 SV=2	
Znhit3	Q9CQK1	Zinc finger HIT domain-containing protein 3 OS=Mus musculus OX=10090 GN=Znhit3 PE=1 SV=1	0.32
Mad2l1bp	Q9DCX1	MAD2L1-binding protein OS=Mus musculus OX=10090 GN=Mad2l1bp PE=1 SV=2	
Eif2ak4	Q9QZ05	eIF-2-alpha kinase GCN2 OS=Mus musculus OX=10090 GN=Eif2ak4 PE=1 SV=2	
Jtb	O88824	Protein JTB OS=Mus musculus OX=10090 GN=Jtb PE=1 SV=1	1.23
Arl2bp	Q9D385	ADP-ribosylation factor-like protein 2-binding protein OS=Mus musculus OX=10090 GN=Arl2bp PE=	0.953333333
Armc9	Q9D2I5	LisH domain-containing protein ARMC9 OS=Mus musculus OX=10090 GN=Armc9 PE=1 SV=1	1.11
Mtrf1l	Q8BJU9	Peptide chain release factor 1-like mitochondrial OS=Mus musculus OX=10090 GN=Mtrf1l PE=2 SV=	0.645
Chmp2a	Q9DB34	Charged multivesicular body protein 2a OS=Mus musculus OX=10090 GN=Chmp2a PE=1 SV=1	
Acot13	Q9CQR4	Acyl-coenzyme A thioesterase 13 OS=Mus musculus OX=10090 GN=Acot13 PE=1 SV=1	1.43
Pusl1	A2ADA5	tRNA pseudouridine synthase-like 1 OS=Mus musculus OX=10090 GN=Pusl1 PE=1 SV=1	0.99

Epg5	Q80TA9	Ectopic P granules protein 5 homolog OS=Mus musculus OX=10090 GN=Epg5 PE=1 SV=2	
Scrn3	Q3TMH2	Secernin-3 OS=Mus musculus OX=10090 GN=Scrn3 PE=1 SV=1	
Tmem161a	Q8VCA6	Transmembrane protein 161A OS=Mus musculus OX=10090 GN=Tmem161a PE=2 SV=1	0.576666667
Plekhg5	Q66T02	Pleckstrin homology domain-containing family G member 5 OS=Mus musculus OX=10090 GN=Plekl	0.89
Mpnd	Q3TV65	MPN domain-containing protein OS=Mus musculus OX=10090 GN=Mpnd PE=2 SV=2	
Plcd3	Q8K2J0	1-phosphatidylinositol 4 5-bisphosphate phosphodiesterase delta-3 OS=Mus musculus OX=10090 G	
Adnp2	Q8CHC8	Activity-dependent neuroprotector homeobox protein 2 OS=Mus musculus OX=10090 GN=Adnp2 P	
Mettl14	Q3UIK4	N6-adenosine-methyltransferase non-catalytic subunit OS=Mus musculus OX=10090 GN=Mettl14 P	0.505
Cdyl	Q9WTK2	Chromodomain Y-like protein OS=Mus musculus OX=10090 GN=Cdyl PE=1 SV=1	
Kiaa2013	Q91X21	Uncharacterized protein KIAA2013 OS=Mus musculus OX=10090 GN=Kiaa2013 PE=1 SV=1	1.175
Plaat3	Q8R3U1	Phospholipase A and acyltransferase 3 OS=Mus musculus OX=10090 GN=Plaat3 PE=1 SV=2	
Ube2w	Q8VDW4	Ubiquitin-conjugating enzyme E2 W OS=Mus musculus OX=10090 GN=Ube2w PE=1 SV=1	
Cers4	Q9D6J1	Ceramide synthase 4 OS=Mus musculus OX=10090 GN=Cers4 PE=1 SV=1	0.743333333
Uxt	Q9WTZ0	Protein UXT OS=Mus musculus OX=10090 GN=Uxt PE=1 SV=2	0.885
Elovl5	Q8BHI7	Elongation of very long chain fatty acids protein 5 OS=Mus musculus OX=10090 GN=Elovl5 PE=1 SV=	0.895
Sesn1	P58006	Sestrin-1 OS=Mus musculus OX=10090 GN=Sesn1 PE=1 SV=3	
Tonsl	Q6NZL6	Tonsoku-like protein OS=Mus musculus OX=10090 GN=Tonsl PE=1 SV=2	
Pnkd	Q69ZP3	Probable hydrolase PNKD OS=Mus musculus OX=10090 GN=Pnkd PE=1 SV=2	
Ccdc97	Q9DBT3	Coiled-coil domain-containing protein 97 OS=Mus musculus OX=10090 GN=Ccdc97 PE=1 SV=1	
Cry1	P97784	Cryptochrome-1 OS=Mus musculus OX=10090 GN=Cry1 PE=1 SV=1	
Svbp	Q99LQ4	Small vasohibin-binding protein OS=Mus musculus OX=10090 GN=Svbp PE=1 SV=1	
	Q9CQ90	Uncharacterized protein C9orf85 homolog OS=Mus musculus OX=10090 PE=2 SV=1	
Smim12	Q78RX3	Small integral membrane protein 12 OS=Mus musculus OX=10090 GN=Smim12 PE=3 SV=1	
Dlg5	E9Q9R9	Disks large homolog 5 OS=Mus musculus OX=10090 GN=Dlg5 PE=1 SV=1	1.17
Trub2	Q91WG3	Mitochondrial mRNA pseudouridine synthase Trub2 OS=Mus musculus OX=10090 GN=Trub2 PE=2 S	1.036666667
Ptcd1	Q8C2E4	Pentatricopeptide repeat-containing protein 1 mitochondrial OS=Mus musculus OX=10090 GN=Pt	
Trim35	Q8C006	Tripartite motif-containing protein 35 OS=Mus musculus OX=10090 GN=Trim35 PE=1 SV=2	0.865
Gtf2h4	O70422	General transcription factor IIH subunit 4 OS=Mus musculus OX=10090 GN=Gtf2h4 PE=1 SV=1	
Rassf8	Q8CJ96	Ras association domain-containing protein 8 OS=Mus musculus OX=10090 GN=Rassf8 PE=2 SV=1	0.476666667
Rgs10	Q9CQE5	Regulator of G-protein signaling 10 OS=Mus musculus OX=10090 GN=Rgs10 PE=1 SV=1	
Dym	Q8CHY3	Dymeclin OS=Mus musculus OX=10090 GN=Dym PE=1 SV=1	0.923333333
Atrn	Q9WU60	Attractin OS=Mus musculus OX=10090 GN=Atrn PE=1 SV=3	
Aph1a	Q8BVF7	Gamma-secretase subunit APH-1A OS=Mus musculus OX=10090 GN=Aph1a PE=1 SV=2	
Syngr2	O55101	Synaptogyrin-2 OS=Mus musculus OX=10090 GN=Syngr2 PE=2 SV=1	0.643333333

Ttc26	Q8BS45	Intraflagellar transport protein 56 OS=Mus musculus OX=10090 GN=Ttc26 PE=1 SV=1	1.14
Cyb561d2	Q9WUE3	Cytochrome b561 domain-containing protein 2 OS=Mus musculus OX=10090 GN=Cyb561d2 PE=1 S	
	Q8CIL4	Uncharacterized protein C1orf131 homolog OS=Mus musculus OX=10090 PE=1 SV=1	
Tmem258	P61166	Transmembrane protein 258 OS=Mus musculus OX=10090 GN=Tmem258 PE=1 SV=1	1.063333333
Haus2	Q9CQS9	HAUS augmin-like complex subunit 2 OS=Mus musculus OX=10090 GN=Haus2 PE=2 SV=1	0.59
Tceal9	Q9DD24	Transcription elongation factor A protein-like 9 OS=Mus musculus OX=10090 GN=Tceal9 PE=3 SV=1	
Ddx11	Q6AXC6	ATP-dependent DNA helicase DDX11 OS=Mus musculus OX=10090 GN=Ddx11 PE=1 SV=2	
Actr5	Q80US4	Actin-related protein 5 OS=Mus musculus OX=10090 GN=Actr5 PE=1 SV=3	
Phrf1	A6H619	PHD and RING finger domain-containing protein 1 OS=Mus musculus OX=10090 GN=Phrf1 PE=1 SV=	1.5
Rce1	P57791	CAAX prenyl protease 2 OS=Mus musculus OX=10090 GN=Rce1 PE=2 SV=1	
Mrpl48	Q8JZS9	39S ribosomal protein L48 mitochondrial OS=Mus musculus OX=10090 GN=Mrpl48 PE=1 SV=1	0.906666667
Tmem9	Q9CR23	Transmembrane protein 9 OS=Mus musculus OX=10090 GN=Tmem9 PE=1 SV=1	1.075
Parp4	E9PYK3	Protein mono-ADP-ribosyltransferase PARP4 OS=Mus musculus OX=10090 GN=Parp4 PE=1 SV=1	0.395
Tpst1	O70281	Protein-tyrosine sulfotransferase 1 OS=Mus musculus OX=10090 GN=Tpst1 PE=1 SV=1	
Tmem245	B1AZA5	Transmembrane protein 245 OS=Mus musculus OX=10090 GN=Tmem245 PE=1 SV=1	0.743333333
Yeats4	Q9CR11	YEATS domain-containing protein 4 OS=Mus musculus OX=10090 GN=Yeats4 PE=2 SV=1	1.4
Zfand1	Q8BFR6	AN1-type zinc finger protein 1 OS=Mus musculus OX=10090 GN=Zfand1 PE=1 SV=1	0.84
Ccdc32	Q8BS39	Coiled-coil domain-containing protein 32 OS=Mus musculus OX=10090 GN=Ccdc32 PE=2 SV=2	
Pphln1	Q8K2H1	Periphilin-1 OS=Mus musculus OX=10090 GN=Pphln1 PE=1 SV=1	
Emc6	Q9CQW0	ER membrane protein complex subunit 6 OS=Mus musculus OX=10090 GN=Emc6 PE=1 SV=1	0.963333333
Map2k7	Q8CE90	Dual specificity mitogen-activated protein kinase kinase 7 OS=Mus musculus OX=10090 GN=Map2k	
Pkp4	Q68FH0	Plakophilin-4 OS=Mus musculus OX=10090 GN=Pkp4 PE=1 SV=1	
Ctnnd2	O35927	Catenin delta-2 OS=Mus musculus OX=10090 GN=Ctnnd2 PE=1 SV=1	
Coq8b	Q566J8	Atypical kinase COQ8B mitochondrial OS=Mus musculus OX=10090 GN=Coq8b PE=1 SV=1	2.475
Tmem19	Q91W52	Transmembrane protein 19 OS=Mus musculus OX=10090 GN=Tmem19 PE=1 SV=1	0.88
Lmf1	Q3U3R4	Lipase maturation factor 1 OS=Mus musculus OX=10090 GN=Lmf1 PE=1 SV=1	
Trex1	Q91XB0	Three-prime repair exonuclease 1 OS=Mus musculus OX=10090 GN=Trex1 PE=1 SV=2	
Lin54	Q571G4	Protein lin-54 homolog OS=Mus musculus OX=10090 GN=Lin54 PE=2 SV=2	
Pdzd11	Q9CZG9	PDZ domain-containing protein 11 OS=Mus musculus OX=10090 GN=Pdzd11 PE=1 SV=1	
Alas1	Q8VC19	5-aminolevulinate synthase nonspecific mitochondrial OS=Mus musculus OX=10090 GN=Alas1 PE=	
Znf131	Q8K3J5	Zinc finger protein 131 OS=Mus musculus OX=10090 GN=Znf131 PE=1 SV=1	
Slc30a4	O35149	Zinc transporter 4 OS=Mus musculus OX=10090 GN=Slc30a4 PE=1 SV=2	
Ubac1	Q8VDI7	Ubiquitin-associated domain-containing protein 1 OS=Mus musculus OX=10090 GN=Ubac1 PE=1 S\	0.963333333
Ppm1l	Q8BHN0	Protein phosphatase 1L OS=Mus musculus OX=10090 GN=Ppm1l PE=1 SV=1	

Fry	E9Q8I9	Protein furry homolog OS=Mus musculus OX=10090 GN=Fry PE=1 SV=1	228.0233333
Rad17	Q6NXW6	Cell cycle checkpoint protein RAD17 OS=Mus musculus OX=10090 GN=Rad17 PE=1 SV=2	
Atg13	Q91YI1	Autophagy-related protein 13 OS=Mus musculus OX=10090 GN=Atg13 PE=1 SV=2	
Cox7b	P56393	Cytochrome c oxidase subunit 7B mitochondrial OS=Mus musculus OX=10090 GN=Cox7b PE=3 SV=	0.9
Retreg2	Q6NS82	Reticulophagy regulator 2 OS=Mus musculus OX=10090 GN=Retreg2 PE=1 SV=2	
Vma21	Q78T54	Vacuolar ATPase assembly integral membrane protein Vma21 OS=Mus musculus OX=10090 GN=Vma	0.393333333
Arid4a	F8VPQ2	AT-rich interactive domain-containing protein 4A OS=Mus musculus OX=10090 GN=Arid4a PE=1 SV	
Chmp1b1	Q99LU0	Charged multivesicular body protein 1b-1 OS=Mus musculus OX=10090 GN=Chmp1b1 PE=1 SV=1	1.6
Rhbdd2	Q8VEK2	Rhomboid domain-containing protein 2 OS=Mus musculus OX=10090 GN=Rhbdd2 PE=1 SV=1	
Hid1	Q8R1F6	Protein HID1 OS=Mus musculus OX=10090 GN=Hid1 PE=1 SV=1	
	Q3TTL0	Uncharacterized protein C3orf38 homolog OS=Mus musculus OX=10090 PE=2 SV=1	
Slc35a1	Q61420	CMP-sialic acid transporter OS=Mus musculus OX=10090 GN=Slc35a1 PE=1 SV=2	
Skp2	Q9Z0Z3	S-phase kinase-associated protein 2 OS=Mus musculus OX=10090 GN=Skp2 PE=1 SV=1	
Hcls1	P49710	Hematopoietic lineage cell-specific protein OS=Mus musculus OX=10090 GN=Hcls1 PE=1 SV=2	0.82
Slc14a1	Q8VHL0	Urea transporter 1 OS=Mus musculus OX=10090 GN=Slc14a1 PE=1 SV=2	0.99
Cep97	Q9CZ62	Centrosomal protein of 97 kDa OS=Mus musculus OX=10090 GN=Cep97 PE=1 SV=1	
Tmem192	Q9CXT7	Transmembrane protein 192 OS=Mus musculus OX=10090 GN=Tmem192 PE=1 SV=1	
Atg12	Q9CQY1	Ubiquitin-like protein ATG12 OS=Mus musculus OX=10090 GN=Atg12 PE=1 SV=1	0.913333333
Stxbp4	Q9WV89	Syntaxin-binding protein 4 OS=Mus musculus OX=10090 GN=Stxbp4 PE=1 SV=1	
Afg1l	Q3V384	AFG1-like ATPase OS=Mus musculus OX=10090 GN=Afg1l PE=1 SV=1	
Sp2	Q9D2H6	Transcription factor Sp2 OS=Mus musculus OX=10090 GN=Sp2 PE=2 SV=2	
Pnpla2	Q8BJ56	Patatin-like phospholipase domain-containing protein 2 OS=Mus musculus OX=10090 GN=Pnpla2 P	
Mt-Cyb	P00158	Cytochrome b OS=Mus musculus OX=10090 GN=Mt-Cyb PE=1 SV=1	0.966666667
Cept1	Q8BGS7	Choline/ethanolaminephosphotransferase 1 OS=Mus musculus OX=10090 GN=Cept1 PE=1 SV=1	0.693333333
Ar	P19091	Androgen receptor OS=Mus musculus OX=10090 GN=Ar PE=1 SV=1	
Baz2a	Q91YE5	Bromodomain adjacent to zinc finger domain protein 2A OS=Mus musculus OX=10090 GN=Baz2a PE	
Bcorl1	A2AQH4	BCL-6 corepressor-like protein 1 OS=Mus musculus OX=10090 GN=Bcorl1 PE=2 SV=1	25.955
Slc35a3	Q8R1T4	UDP-N-acetylglucosamine transporter OS=Mus musculus OX=10090 GN=Slc35a3 PE=2 SV=1	
Nagpa	Q8BJ48	N-acetylglucosamine-1-phosphodiester alpha-N-acetylglucosaminidase OS=Mus musculus OX=1009	
Cpn1	Q9JJN5	Carboxypeptidase N catalytic chain OS=Mus musculus OX=10090 GN=Cpn1 PE=1 SV=1	
Ahsg	P29699	Alpha-2-HS-glycoprotein OS=Mus musculus OX=10090 GN=Ahsg PE=1 SV=1	0.09
Ajuba	Q91XC0	LIM domain-containing protein ajuba OS=Mus musculus OX=10090 GN=Ajuba PE=1 SV=1	
Aptx	Q7TQC5	Aprataxin OS=Mus musculus OX=10090 GN=Aptx PE=1 SV=2	
Lin9	Q8C735	Protein lin-9 homolog OS=Mus musculus OX=10090 GN=Lin9 PE=1 SV=2	0.443333333

Nfkbia	Q9Z1E3	NF-kappa-B inhibitor alpha OS=Mus musculus OX=10090 GN=Nfkbia PE=1 SV=2	
Chmp1b2	Q9CQD4	Charged multivesicular body protein 1b-2 OS=Mus musculus OX=10090 GN=Chmp1b2 PE=2 SV=2	1.046666667
Fam185a	Q7TPD2	Protein FAM185A OS=Mus musculus OX=10090 GN=Fam185a PE=2 SV=1	
B4galt1	P15535	Beta-1 4-galactosyltransferase 1 OS=Mus musculus OX=10090 GN=B4galt1 PE=1 SV=1	
Scg2	Q03517	Secretogranin-2 OS=Mus musculus OX=10090 GN=Scg2 PE=1 SV=1	0.845
Rfx5	Q9JL61	DNA-binding protein Rfx5 OS=Mus musculus OX=10090 GN=Rfx5 PE=1 SV=2	
Ift46	Q9DB07	Intraflagellar transport protein 46 homolog OS=Mus musculus OX=10090 GN=Ift46 PE=1 SV=1	
Zkscan1	Q8BGS3	Zinc finger protein with KRAB and SCAN domains 1 OS=Mus musculus OX=10090 GN=Zkscan1 PE=2 S	
Abl2	Q4JIM5	Tyrosine-protein kinase ABL2 OS=Mus musculus OX=10090 GN=Abl2 PE=1 SV=1	
Pou2f1	P25425	POU domain class 2 transcription factor 1 OS=Mus musculus OX=10090 GN=Pou2f1 PE=1 SV=3	
Serpina7	P61939	Thyroxine-binding globulin OS=Mus musculus OX=10090 GN=Serpina7 PE=2 SV=1	0.01
Banp	Q8VBU8	Protein BANP OS=Mus musculus OX=10090 GN=Banp PE=1 SV=1	
Nlrp4f	Q66X05	NACHT LRR and PYD domains-containing protein 4F OS=Mus musculus OX=10090 GN=Nlrp4f PE=2 S	
Nsrp1	Q5NCR9	Nuclear speckle splicing regulatory protein 1 OS=Mus musculus OX=10090 GN=Nsrp1 PE=1 SV=1	0.955
Sh2d4b	A6X942	SH2 domain-containing protein 4B OS=Mus musculus OX=10090 GN=Sh2d4b PE=2 SV=1	
Alg8	Q6P8H8	Probable dolichyl pyrophosphate Glc1Man9GlcNAc2 alpha-1 3-glucosyltransferase OS=Mus muscul	0.54
Fancl	Q9CR14	E3 ubiquitin-protein ligase FANCL OS=Mus musculus OX=10090 GN=Fancl PE=1 SV=1	
Dgke	Q9R1C6	Diacylglycerol kinase epsilon OS=Mus musculus OX=10090 GN=Dgke PE=1 SV=1	1.205
Vps8	Q0P5W1	Vacuolar protein sorting-associated protein 8 homolog OS=Mus musculus OX=10090 GN=Vps8 PE=1	
Actrt2	Q9D9L5	Actin-related protein T2 OS=Mus musculus OX=10090 GN=Actrt2 PE=2 SV=1	
Snx24	Q9CRB0	Sorting nexin-24 OS=Mus musculus OX=10090 GN=Snx24 PE=1 SV=1	0.86
Thap12	Q9CUX1	52 kDa repressor of the inhibitor of the protein kinase OS=Mus musculus OX=10090 GN=Thap12 PE=	
Orc1	Q9Z1N2	Origin recognition complex subunit 1 OS=Mus musculus OX=10090 GN=Orc1 PE=1 SV=2	
Oasl2	Q9Z2F2	2'-5'-oligoadenylate synthase-like protein 2 OS=Mus musculus OX=10090 GN=Oasl2 PE=1 SV=2	0.016666667
Kif27	Q7M6Z4	Kinesin-like protein KIF27 OS=Mus musculus OX=10090 GN=Kif27 PE=1 SV=1	0.625
Atxn7	Q8R4I1	Ataxin-7 OS=Mus musculus OX=10090 GN=Atxn7 PE=1 SV=2	4.52
Inpp5b	Q8K337	Type II inositol 1 4 5-trisphosphate 5-phosphatase OS=Mus musculus OX=10090 GN=Inpp5b PE=1 SV	
Nlgn4l	B0F2B4	Neuroligin 4-like OS=Mus musculus OX=10090 GN=Nlgn4l PE=1 SV=1	
Ncoa6	Q9JL19	Nuclear receptor coactivator 6 OS=Mus musculus OX=10090 GN=Ncoa6 PE=1 SV=1	0.535
Cnr2	P47936	Cannabinoid receptor 2 OS=Mus musculus OX=10090 GN=Cnr2 PE=1 SV=1	0.236666667
Kdm1b	Q8CIG3	Lysine-specific histone demethylase 1B OS=Mus musculus OX=10090 GN=Kdm1b PE=1 SV=1	
Rad51ap1	Q8C551	RAD51-associated protein 1 OS=Mus musculus OX=10090 GN=Rad51ap1 PE=1 SV=2	
Cbfa2t2	O70374	Protein CBFA2T2 OS=Mus musculus OX=10090 GN=Cbfa2t2 PE=1 SV=3	
Smcr8	Q3UMB5	Guanine nucleotide exchange protein SMCR8 OS=Mus musculus OX=10090 GN=Smcr8 PE=1 SV=2	

Slc46a2	Q8CA03	Thymic stromal cotransporter protein OS=Mus musculus OX=10090 GN=Slc46a2 PE=1 SV=2	
Xpc	P51612	DNA repair protein complementing XP-C cells homolog OS=Mus musculus OX=10090 GN=Xpc PE=1	
Zdhhc6	Q9CPV7	Palmitoyltransferase ZDHHC6 OS=Mus musculus OX=10090 GN=Zdhhc6 PE=1 SV=1	
Csmd3	Q80T79	CUB and sushi domain-containing protein 3 OS=Mus musculus OX=10090 GN=Csmd3 PE=1 SV=3	
Tmem222	Q8BVA2	Transmembrane protein 222 OS=Mus musculus OX=10090 GN=Tmem222 PE=1 SV=1	1.77
Vat1l	Q80TB8	Synaptic vesicle membrane protein VAT-1 homolog-like OS=Mus musculus OX=10090 GN=Vat1l PE=	
Kif18a	Q91WD7	Kinesin-like protein KIF18A OS=Mus musculus OX=10090 GN=Kif18a PE=2 SV=1	0.656666667
Tex15	F8VPN2	Testis-expressed protein 15 OS=Mus musculus OX=10090 GN=Tex15 PE=2 SV=1	
Gcnt4	E9Q649	Beta-1 3-galactosyl-O-glycosyl-glycoprotein beta-1 6-N-acetylglucosaminyltransferase 4 OS=Mus mu	0.475
Gzf1	Q4VBD9	GDNF-inducible zinc finger protein 1 OS=Mus musculus OX=10090 GN=Gzf1 PE=1 SV=2	
Acp6	Q8BP40	Lysophosphatidic acid phosphatase type 6 OS=Mus musculus OX=10090 GN=Acp6 PE=1 SV=1	0.62
Abhd16a	Q9Z1Q2	Phosphatidylserine lipase ABHD16A OS=Mus musculus OX=10090 GN=Abhd16a PE=1 SV=3	
Adgra3	Q7TT36	Adhesion G protein-coupled receptor A3 OS=Mus musculus OX=10090 GN=Adgra3 PE=2 SV=3	0.896666667
Trak1	Q6PD31	Trafficking kinesin-binding protein 1 OS=Mus musculus OX=10090 GN=Trak1 PE=1 SV=1	
Myrip	Q8K3I4	Rab effector MyRIP OS=Mus musculus OX=10090 GN=Myrip PE=1 SV=1	0.105
Lrch1	P62046	Leucine-rich repeat and calponin homology domain-containing protein 1 OS=Mus musculus OX=10	
Dnah8	Q91XQ0	Dynein heavy chain 8 axonemal OS=Mus musculus OX=10090 GN=Dnah8 PE=1 SV=2	0.92
Plch1	Q4KWH5	1-phosphatidylinositol 4 5-bisphosphate phosphodiesterase eta-1 OS=Mus musculus OX=10090 GN	
Pp2d1	Q8BVT6	Protein phosphatase 2C-like domain-containing protein 1 OS=Mus musculus OX=10090 GN=Pp2d1	0.98
Mamdc4	A2AJA7	Apical endosomal glycoprotein OS=Mus musculus OX=10090 GN=Mamdc4 PE=3 SV=1	
Tada3	Q8R0L9	Transcriptional adapter 3 OS=Mus musculus OX=10090 GN=Tada3 PE=1 SV=1	
Fas	P25446	Tumor necrosis factor receptor superfamily member 6 OS=Mus musculus OX=10090 GN=Fas PE=1 S\	
Limk2	O54785	LIM domain kinase 2 OS=Mus musculus OX=10090 GN=Limk2 PE=1 SV=2	
Ubn2	Q80WC1	Ubinuclein-2 OS=Mus musculus OX=10090 GN=Ubn2 PE=1 SV=2	0.886666667
Pigh	Q5M9N4	Phosphatidylinositol N-acetylglucosaminyltransferase subunit H OS=Mus musculus OX=10090 GN=	
Mob2	Q8VI63	MOB kinase activator 2 OS=Mus musculus OX=10090 GN=Mob2 PE=1 SV=1	
Fem1c	Q8CEF1	Protein fem-1 homolog C OS=Mus musculus OX=10090 GN=Fem1c PE=2 SV=1	
Elavl3	Q60900	ELAV-like protein 3 OS=Mus musculus OX=10090 GN=Elavl3 PE=1 SV=1	1.775
Tada1	Q99LM9	Transcriptional adapter 1 OS=Mus musculus OX=10090 GN=Tada1 PE=2 SV=1	
Agbl3	Q8CDP0	Cytosolic carboxypeptidase 3 OS=Mus musculus OX=10090 GN=Agbl3 PE=1 SV=1	235.3933333
Skint3	A7TZF0	Selection and upkeep of intraepithelial T-cells protein 3 OS=Mus musculus OX=10090 GN=Skint3 PE	0.793333333
Cdc14a	Q6GQT0	Dual specificity protein phosphatase CDC14A OS=Mus musculus OX=10090 GN=Cdc14a PE=1 SV=2	
Tcf7	Q00417	Transcription factor 7 OS=Mus musculus OX=10090 GN=Tcf7 PE=1 SV=2	
Tcf7l1	Q9Z1J1	Transcription factor 7-like 1 OS=Mus musculus OX=10090 GN=Tcf7l1 PE=1 SV=2	

Tcf7l2	Q924A0	Transcription factor 7-like 2 OS=Mus musculus OX=10090 GN=Tcf7l2 PE=1 SV=2	
Lef1	P27782	Lymphoid enhancer-binding factor 1 OS=Mus musculus OX=10090 GN=Lef1 PE=1 SV=1	
Ryr1	E9PZQ0	Ryanodine receptor 1 OS=Mus musculus OX=10090 GN=Ryr1 PE=1 SV=1	0.89
Tex47	Q9D5W8	Testis-expressed protein 47 OS=Mus musculus OX=10090 GN=Tex47 PE=2 SV=1	0.785
Mrm3	Q5ND52	rRNA methyltransferase 3 mitochondrial OS=Mus musculus OX=10090 GN=Mrm3 PE=2 SV=1	0.23
Crtc2	Q3U182	CREB-regulated transcription coactivator 2 OS=Mus musculus OX=10090 GN=Crtc2 PE=1 SV=2	
Tex14	Q7M6U3	Inactive serine/threonine-protein kinase TEX14 OS=Mus musculus OX=10090 GN=Tex14 PE=1 SV=2	
Top3a	O70157	DNA topoisomerase 3-alpha OS=Mus musculus OX=10090 GN=Top3a PE=2 SV=1	
Tnk2	O54967	Activated CDC42 kinase 1 OS=Mus musculus OX=10090 GN=Tnk2 PE=1 SV=2	
Parp14	Q2EMV9	Protein mono-ADP-ribosyltransferase PARP14 OS=Mus musculus OX=10090 GN=Parp14 PE=1 SV=3	0.11
Lrp8	Q924X6	Low-density lipoprotein receptor-related protein 8 OS=Mus musculus OX=10090 GN=Lrp8 PE=1 SV=	
Snx19	Q6P4T1	Sorting nexin-19 OS=Mus musculus OX=10090 GN=Snx19 PE=1 SV=1	1.12
Isca2	Q9DCB8	Iron-sulfur cluster assembly 2 homolog mitochondrial OS=Mus musculus OX=10090 GN=Isca2 PE=1	
Lto1	Q8CH62	Protein LTO1 homolog OS=Mus musculus OX=10090 GN=Lto1 PE=1 SV=1	
Leng1	Q9DB98	Leukocyte receptor cluster member 1 homolog OS=Mus musculus OX=10090 GN=Leng1 PE=2 SV=1	
Odf2	A3KGV1	Outer dense fiber protein 2 OS=Mus musculus OX=10090 GN=Odf2 PE=1 SV=1	
Sspo	Q8CG65	SCO-spondin OS=Mus musculus OX=10090 GN=Sspo PE=2 SV=2	0.053333333
Atp5f1e	P56382	ATP synthase subunit epsilon mitochondrial OS=Mus musculus OX=10090 GN=Atp5f1e PE=1 SV=2	
Smyd3	Q9CWR2	Histone-lysine N-methyltransferase SMYD3 OS=Mus musculus OX=10090 GN=Smyd3 PE=2 SV=1	
Oprd1	P32300	Delta-type opioid receptor OS=Mus musculus OX=10090 GN=Oprd1 PE=1 SV=1	
Slc38a1	Q8K2P7	Sodium-coupled neutral amino acid transporter 1 OS=Mus musculus OX=10090 GN=Slc38a1 PE=1 S	
Ptx3	P48759	Pentraxin-related protein PTX3 OS=Mus musculus OX=10090 GN=Ptx3 PE=1 SV=2	
Mtus1	Q5HZI1	Microtubule-associated tumor suppressor 1 homolog OS=Mus musculus OX=10090 GN=Mtus1 PE=1	
Glce	Q9EPS3	D-glucuronyl C5-epimerase OS=Mus musculus OX=10090 GN=Glce PE=1 SV=2	0.84
Ttc5	Q99LG4	Tetratricopeptide repeat protein 5 OS=Mus musculus OX=10090 GN=Ttc5 PE=1 SV=2	
Arhgap23	Q69ZH9	Rho GTPase-activating protein 23 OS=Mus musculus OX=10090 GN=Arhgap23 PE=1 SV=2	
Lrrc23	O35125	Leucine-rich repeat-containing protein 23 OS=Mus musculus OX=10090 GN=Lrrc23 PE=1 SV=1	0.75
Casp2	P29594	Caspase-2 OS=Mus musculus OX=10090 GN=Casp2 PE=1 SV=5	
Meioc	A2AG06	Meiosis-specific coiled-coil domain-containing protein MEIOC OS=Mus musculus OX=10090 GN=Me	
Dnmt3a	O88508	DNA (cytosine-5)-methyltransferase 3A OS=Mus musculus OX=10090 GN=Dnmt3a PE=1 SV=2	
Sycp1	Q62209	Synaptonemal complex protein 1 OS=Mus musculus OX=10090 GN=Sycp1 PE=1 SV=2	
Il27ra	O70394	Interleukin-27 receptor subunit alpha OS=Mus musculus OX=10090 GN=Il27ra PE=2 SV=2	0.02
Rspry1	Q8BVR6	RING finger and SPRY domain-containing protein 1 OS=Mus musculus OX=10090 GN=Rspry1 PE=1 S	0.86
Wdr93	Q402B2	WD repeat-containing protein 93 OS=Mus musculus OX=10090 GN=Wdr93 PE=2 SV=1	

Rasl10a	Q8K5A4	Ras-like protein family member 10A OS=Mus musculus OX=10090 GN=Rasl10a PE=2 SV=1	
Hunk	O88866	Hormonally up-regulated neu tumor-associated kinase OS=Mus musculus OX=10090 GN=Hunk PE=2	0
Tmc5	Q32NZ6	Transmembrane channel-like protein 5 OS=Mus musculus OX=10090 GN=Tmc5 PE=2 SV=1	0
Eif2ak1	Q9Z2R9	Eukaryotic translation initiation factor 2-alpha kinase 1 OS=Mus musculus OX=10090 GN=Eif2ak1 PI	0.94
Cdc45	Q9Z1X9	Cell division control protein 45 homolog OS=Mus musculus OX=10090 GN=Cdc45 PE=1 SV=2	
Abhd6	Q8R2Y0	Monoacylglycerol lipase ABHD6 OS=Mus musculus OX=10090 GN=Abhd6 PE=1 SV=1	
Lrrc45	Q8CIM1	Leucine-rich repeat-containing protein 45 OS=Mus musculus OX=10090 GN=Lrrc45 PE=2 SV=2	0.98
Ghdc	Q99J23	GH3 domain-containing protein OS=Mus musculus OX=10090 GN=Ghdc PE=2 SV=2	
Slc22a8	O88909	Solute carrier family 22 member 8 OS=Mus musculus OX=10090 GN=Slc22a8 PE=1 SV=2	
Selenon	D3Z2R5	Selenoprotein N OS=Mus musculus OX=10090 GN=Selenon PE=3 SV=2	0.75
Sort1	Q6PHU5	Sortilin OS=Mus musculus OX=10090 GN=Sort1 PE=1 SV=1	0.675
Med12l	Q8BQM9	Mediator of RNA polymerase II transcription subunit 12-like protein OS=Mus musculus OX=10090 G	
Resf1	Q5DTW7	Retroelement silencing factor 1 OS=Mus musculus OX=10090 GN=Resf1 PE=1 SV=2	1.165
Rtn4rl1	Q8K0S5	Reticulon-4 receptor-like 1 OS=Mus musculus OX=10090 GN=Rtn4rl1 PE=1 SV=1	0.975
Reep3	Q99KK1	Receptor expression-enhancing protein 3 OS=Mus musculus OX=10090 GN=Reep3 PE=1 SV=1	
Zfp292	Q9Z2U2	Zinc finger protein 292 OS=Mus musculus OX=10090 GN=Zfp292 PE=1 SV=2	
Tnip1	Q9WUU8	TNFAIP3-interacting protein 1 OS=Mus musculus OX=10090 GN=Tnip1 PE=1 SV=1	
Irs4	Q9Z0Y7	Insulin receptor substrate 4 OS=Mus musculus OX=10090 GN=Irs4 PE=1 SV=2	0.17
Ift20	Q61025	Intraflagellar transport protein 20 homolog OS=Mus musculus OX=10090 GN=Ift20 PE=1 SV=1	
Lrrc20	Q8CI70	Leucine-rich repeat-containing protein 20 OS=Mus musculus OX=10090 GN=Lrrc20 PE=1 SV=1	
Wnt7b	P28047	Protein Wnt-7b OS=Mus musculus OX=10090 GN=Wnt7b PE=1 SV=1	
Selenbp2	Q63836	Selenium-binding protein 2 OS=Mus musculus OX=10090 GN=Selenbp2 PE=1 SV=2	
Ccdc110	Q3V125	Coiled-coil domain-containing protein 110 OS=Mus musculus OX=10090 GN=Ccdc110 PE=1 SV=1	
Pan3	Q640Q5	PAN2-PAN3 deadenylation complex subunit Pan3 OS=Mus musculus OX=10090 GN=Pan3 PE=1 SV=3	
Mbd2	Q9Z2E1	Methyl-CpG-binding domain protein 2 OS=Mus musculus OX=10090 GN=Mbd2 PE=2 SV=2	3.895
Ptprz1	B9EKR1	Receptor-type tyrosine-protein phosphatase zeta OS=Mus musculus OX=10090 GN=Ptprz1 PE=1 SV=	
Mov10l1	Q99MV5	RNA helicase Mov10l1 OS=Mus musculus OX=10090 GN=Mov10l1 PE=1 SV=1	
Scarf1	Q5ND28	Scavenger receptor class F member 1 OS=Mus musculus OX=10090 GN=Scarf1 PE=1 SV=1	52.81
Nxph1	Q61200	Neurexophilin-1 OS=Mus musculus OX=10090 GN=Nxph1 PE=2 SV=2	
Cep162	Q6ZQ06	Centrosomal protein of 162 kDa OS=Mus musculus OX=10090 GN=Cep162 PE=1 SV=2	0.02
Znf462	B1AWL2	Zinc finger protein 462 OS=Mus musculus OX=10090 GN=Znf462 PE=1 SV=1	
Uvrag	Q8K245	UV radiation resistance-associated protein OS=Mus musculus OX=10090 GN=Uvrag PE=1 SV=2	0.685
Atpsckmt	Q9D1Z3	ATP synthase subunit C lysine N-methyltransferase OS=Mus musculus OX=10090 GN=Atpsckmt PE=1	
Ndrg4	Q8BTG7	Protein NDRG4 OS=Mus musculus OX=10090 GN=Ndrg4 PE=1 SV=1	

Adcy7	P51829	Adenylate cyclase type 7 OS=Mus musculus OX=10090 GN=Adcy7 PE=1 SV=2	
Mrm1	Q99J25	rRNA methyltransferase 1 mitochondrial OS=Mus musculus OX=10090 GN=Mrm1 PE=2 SV=2	
Meikin	Q5F2C3	Meiosis-specific kinetochore protein OS=Mus musculus OX=10090 GN=Meikin PE=1 SV=1	0.85
Auts2	A0A087WPF7	Autism susceptibility gene 2 protein homolog OS=Mus musculus OX=10090 GN=Auts2 PE=1 SV=2	
Fam3a	Q9D8T0	Protein FAM3A OS=Mus musculus OX=10090 GN=Fam3a PE=2 SV=1	
Adgrg4	B7ZCC9	Adhesion G-protein coupled receptor G4 OS=Mus musculus OX=10090 GN=Adgrg4 PE=3 SV=1	0.9
Adam25	Q9R159	Disintegrin and metalloproteinase domain-containing protein 25 OS=Mus musculus OX=10090 GN=	0
Syn3	Q8JZP2	Synapsin-3 OS=Mus musculus OX=10090 GN=Syn3 PE=1 SV=2	0.686666667
Capn15	Q9JLG8	Calpain-15 OS=Mus musculus OX=10090 GN=Capn15 PE=1 SV=1	
Slc26a2	Q62273	Sulfate transporter OS=Mus musculus OX=10090 GN=Slc26a2 PE=1 SV=1	
Nodal	P43021	Nodal OS=Mus musculus OX=10090 GN=Nodal PE=2 SV=2	
Ntrk1	Q3UFB7	High affinity nerve growth factor receptor OS=Mus musculus OX=10090 GN=Ntrk1 PE=1 SV=2	
Nynrin	Q5DTZ0	Protein NYNRIN OS=Mus musculus OX=10090 GN=Nynrin PE=2 SV=2	
Ugcg	O88693	Ceramide glucosyltransferase OS=Mus musculus OX=10090 GN=Ugcg PE=1 SV=1	
Ky	Q8C8H8	Kyphoscoliosis peptidase OS=Mus musculus OX=10090 GN=Ky PE=1 SV=1	
Trim26	Q99PN3	Tripartite motif-containing protein 26 OS=Mus musculus OX=10090 GN=Trim26 PE=2 SV=3	
Gimap4	Q99JY3	GTPase IMAP family member 4 OS=Mus musculus OX=10090 GN=Gimap4 PE=1 SV=2	
Dzip1	Q8BMD2	Zinc finger protein DZIP1 OS=Mus musculus OX=10090 GN=Dzip1 PE=1 SV=3	
Ankrd49	Q8VE42	Ankyrin repeat domain-containing protein 49 OS=Mus musculus OX=10090 GN=Ankrd49 PE=1 SV=1	
Kndc1	Q0KK55	Kinase non-catalytic C-lobe domain-containing protein 1 OS=Mus musculus OX=10090 GN=Kndc1 P	0.15
Abca8a	Q8K442	ATP-binding cassette sub-family A member 8-A OS=Mus musculus OX=10090 GN=Abca8a PE=1 SV=2	
Abca9	Q8K449	ATP-binding cassette sub-family A member 9 OS=Mus musculus OX=10090 GN=Abca9 PE=1 SV=2	
Ranbp3l	Q6PDH4	Ran-binding protein 3-like OS=Mus musculus OX=10090 GN=Ranbp3l PE=1 SV=1	0.91
Setd5	Q5XJV7	Histone-lysine N-methyltransferase SETD5 OS=Mus musculus OX=10090 GN=Setd5 PE=1 SV=2	
Limch1	Q3UH68	LIM and calponin homology domains-containing protein 1 OS=Mus musculus OX=10090 GN=Limch	0.405
Dlgap1	Q9D415	Disks large-associated protein 1 OS=Mus musculus OX=10090 GN=Dlgap1 PE=1 SV=3	1.936666667
Cnnm3	Q32NY4	Metal transporter CNNM3 OS=Mus musculus OX=10090 GN=Cnnm3 PE=1 SV=2	0.99
Aff3	P51827	AF4/FMR2 family member 3 OS=Mus musculus OX=10090 GN=Aff3 PE=1 SV=2	
Ankar	A2RT91	Ankyrin and armadillo repeat-containing protein OS=Mus musculus OX=10090 GN=Ankar PE=2 SV=1	
Rnf10	Q3UIW5	RING finger protein 10 OS=Mus musculus OX=10090 GN=Rnf10 PE=2 SV=2	
Slc9c1	Q6UJY2	Sodium/hydrogen exchanger 10 OS=Mus musculus OX=10090 GN=Slc9c1 PE=1 SV=3	
Obi1	Q8K2Y0	ORC ubiquitin ligase 1 OS=Mus musculus OX=10090 GN=Obi1 PE=1 SV=2	
Igsf8	Q8R366	Immunoglobulin superfamily member 8 OS=Mus musculus OX=10090 GN=Igsf8 PE=1 SV=2	
Hdac9	Q99N13	Histone deacetylase 9 OS=Mus musculus OX=10090 GN=Hdac9 PE=1 SV=2	

Fbxo32	Q9CPU7	F-box only protein 32 OS=Mus musculus OX=10090 GN=Fbxo32 PE=2 SV=1	
Prdm8	Q8BZ97	PR domain zinc finger protein 8 OS=Mus musculus OX=10090 GN=Prdm8 PE=1 SV=1	
Stxbp2	Q64324	Syntaxin-binding protein 2 OS=Mus musculus OX=10090 GN=Stxbp2 PE=1 SV=1	
Map4k1	P70218	Mitogen-activated protein kinase kinase kinase kinase 1 OS=Mus musculus OX=10090 GN=Map4k1 P	
Ncoa3	O09000	Nuclear receptor coactivator 3 OS=Mus musculus OX=10090 GN=Ncoa3 PE=1 SV=2	
Patj	Q63ZW7	InaD-like protein OS=Mus musculus OX=10090 GN=Patj PE=1 SV=2	
Traf3ip1	Q149C2	TRAF3-interacting protein 1 OS=Mus musculus OX=10090 GN=Traf3ip1 PE=1 SV=2	
Nod2	Q8K3Z0	Nucleotide-binding oligomerization domain-containing protein 2 OS=Mus musculus OX=10090 GN	
Kmo	Q91WN4	Kynurenine 3-monooxygenase OS=Mus musculus OX=10090 GN=Kmo PE=1 SV=1	0
Lnx2	Q91XL2	Ligand of Numb protein X 2 OS=Mus musculus OX=10090 GN=Lnx2 PE=1 SV=2	
Lama3	Q61789	Laminin subunit alpha-3 OS=Mus musculus OX=10090 GN=Lama3 PE=1 SV=4	
Troap	B7ZNG4	Tastin OS=Mus musculus OX=10090 GN=Troap PE=2 SV=1	0.9
Sema3f	O88632	Semaphorin-3F OS=Mus musculus OX=10090 GN=Sema3f PE=2 SV=2	
Cep350	E9Q309	Centrosome-associated protein 350 OS=Mus musculus OX=10090 GN=Cep350 PE=1 SV=1	
Map3k1	P53349	Mitogen-activated protein kinase kinase kinase 1 OS=Mus musculus OX=10090 GN=Map3k1 PE=1 SV	
Cenpa	O35216	Histone H3-like centromeric protein A OS=Mus musculus OX=10090 GN=Cenpa PE=2 SV=1	
Atp9a	O70228	Probable phospholipid-transporting ATPase IIA OS=Mus musculus OX=10090 GN=Atp9a PE=1 SV=3	
Cplane1	Q8CE72	Ciliogenesis and planar polarity effector 1 OS=Mus musculus OX=10090 GN=Cplane1 PE=1 SV=4	
Ttc21a	Q8C0S4	Tetratricopeptide repeat protein 21A OS=Mus musculus OX=10090 GN=Ttc21a PE=1 SV=2	
Id3	P41133	DNA-binding protein inhibitor ID-3 OS=Mus musculus OX=10090 GN=Id3 PE=1 SV=1	
Mab21l4	Q8CEZ4	Protein mab-21-like 4 OS=Mus musculus OX=10090 GN=Mab21l4 PE=1 SV=2	
Slc37a2	Q9WU81	Glucose-6-phosphate exchanger SLC37A2 OS=Mus musculus OX=10090 GN=Slc37a2 PE=1 SV=1	
Slc37a1	Q8R070	Glucose-6-phosphate exchanger SLC37A1 OS=Mus musculus OX=10090 GN=Slc37a1 PE=2 SV=1	
Plekhg6	Q8R0J1	Pleckstrin homology domain-containing family G member 6 OS=Mus musculus OX=10090 GN=Plekl	
Phlpp2	Q8BXA7	PH domain leucine-rich repeat-containing protein phosphatase 2 OS=Mus musculus OX=10090 GN=	
Aox1	O54754	Aldehyde oxidase 1 OS=Mus musculus OX=10090 GN=Aox1 PE=1 SV=2	
Ap1s3	Q7TN05	AP-1 complex subunit sigma-3 OS=Mus musculus OX=10090 GN=Ap1s3 PE=1 SV=1	
Dhdh	Q9DBB8	Trans-1 2-dihydrobenzene-1 2-diol dehydrogenase OS=Mus musculus OX=10090 GN=Dhdh PE=1 SV=	
Lnx1	O70263	E3 ubiquitin-protein ligase LNX OS=Mus musculus OX=10090 GN=Lnx1 PE=1 SV=1	
Svep1	A2AVA0	Sushi von Willebrand factor type A EGF and pentraxin domain-containing protein 1 OS=Mus muscu	
Pecr	Q99MZ7	Peroxisomal trans-2-enoyl-CoA reductase OS=Mus musculus OX=10090 GN=Pecr PE=1 SV=1	
Adcy4	Q91WF3	Adenylate cyclase type 4 OS=Mus musculus OX=10090 GN=Adcy4 PE=1 SV=1	
Chil3	O35744	Chitinase-like protein 3 OS=Mus musculus OX=10090 GN=Chil3 PE=1 SV=2	
Egr1	P08046	Early growth response protein 1 OS=Mus musculus OX=10090 GN=Egr1 PE=1 SV=2	

Aff4	Q9ESC8	AF4/FMR2 family member 4 OS=Mus musculus OX=10090 GN=Aff4 PE=1 SV=1	
Tsga10	Q6NY15	Testis-specific gene 10 protein OS=Mus musculus OX=10090 GN=Tsga10 PE=1 SV=1	
Cldn1	O88551	Claudin-1 OS=Mus musculus OX=10090 GN=Cldn1 PE=1 SV=1	
Reln	Q60841	Reelin OS=Mus musculus OX=10090 GN=Reln PE=1 SV=3	
Cntrl	A2AL36	Centriolin OS=Mus musculus OX=10090 GN=Cntrl PE=1 SV=2	
Obscn	A2AAJ9	Obscurin OS=Mus musculus OX=10090 GN=Obscn PE=1 SV=3	
Stard5	Q9EPQ7	StAR-related lipid transfer protein 5 OS=Mus musculus OX=10090 GN=Stard5 PE=1 SV=2	
Rgs9	O54828	Regulator of G-protein signaling 9 OS=Mus musculus OX=10090 GN=Rgs9 PE=1 SV=3	
Ccdc89	Q9DA73	Coiled-coil domain-containing protein 89 OS=Mus musculus OX=10090 GN=Ccdc89 PE=1 SV=2	
Ccnl2	Q9JJA7	Cyclin-L2 OS=Mus musculus OX=10090 GN=Ccnl2 PE=1 SV=1	
Lancl3	Q8CD19	LanC-like protein 3 OS=Mus musculus OX=10090 GN=Lancl3 PE=2 SV=2	
Fbxo41	Q6NS60	F-box only protein 41 OS=Mus musculus OX=10090 GN=Fbxo41 PE=1 SV=3	
Brip1	Q5SXJ3	Fanconi anemia group J protein homolog OS=Mus musculus OX=10090 GN=Brip1 PE=2 SV=1	
Fsip2	A2ARZ3	Fibrous sheath-interacting protein 2 OS=Mus musculus OX=10090 GN=Fsip2 PE=1 SV=3	
Unc45b	Q8CGY6	Protein unc-45 homolog B OS=Mus musculus OX=10090 GN=Unc45b PE=1 SV=1	0.856666667
Cnksr3	Q8BMA3	Connector enhancer of kinase suppressor of ras 3 OS=Mus musculus OX=10090 GN=Cnksr3 PE=1 SV=	
Iqcd	Q9D3V1	Dynein regulatory complex protein 10 OS=Mus musculus OX=10090 GN=Iqcd PE=2 SV=1	
Kif26a	Q52KG5	Kinesin-like protein KIF26A OS=Mus musculus OX=10090 GN=Kif26a PE=1 SV=2	
Ets1	P27577	Protein C-ets-1 OS=Mus musculus OX=10090 GN=Ets1 PE=1 SV=2	
Zan	O88799	Zonadhesin OS=Mus musculus OX=10090 GN=Zan PE=1 SV=1	
Hif1a	Q61221	Hypoxia-inducible factor 1-alpha OS=Mus musculus OX=10090 GN=Hif1a PE=1 SV=3	0.01
Fancm	Q8BGE5	Fanconi anemia group M protein homolog OS=Mus musculus OX=10090 GN=Fancm PE=1 SV=3	1.125
Acsf3	Q3URE1	Malonate--CoA ligase ACSF3 mitochondrial OS=Mus musculus OX=10090 GN=Acsf3 PE=1 SV=2	
Pik3cd	O35904	Phosphatidylinositol 4 5-bisphosphate 3-kinase catalytic subunit delta isoform OS=Mus musculus C	
Cndp1	Q8BUG2	Beta-Ala-His dipeptidase OS=Mus musculus OX=10090 GN=Cndp1 PE=2 SV=1	0.315
Cflar	O35732	CASP8 and FADD-like apoptosis regulator OS=Mus musculus OX=10090 GN=Cflar PE=1 SV=3	
Sardh	Q99LB7	Sarcosine dehydrogenase mitochondrial OS=Mus musculus OX=10090 GN=Sardh PE=1 SV=1	
Ap4m1	Q9JKC7	AP-4 complex subunit mu-1 OS=Mus musculus OX=10090 GN=Ap4m1 PE=1 SV=1	
Fam83g	Q5SWY7	Protein FAM83G OS=Mus musculus OX=10090 GN=Fam83g PE=1 SV=1	
Ptk2b	Q9QVP9	Protein-tyrosine kinase 2-beta OS=Mus musculus OX=10090 GN=Ptk2b PE=1 SV=2	
Adcy3	Q8VHH7	Adenylate cyclase type 3 OS=Mus musculus OX=10090 GN=Adcy3 PE=1 SV=2	
Acrbp	Q3V140	Acrosin-binding protein OS=Mus musculus OX=10090 GN=Acrbp PE=1 SV=1	
Abca1	P41233	Phospholipid-transporting ATPase ABCA1 OS=Mus musculus OX=10090 GN=Abca1 PE=1 SV=4	
Fam216a	Q9DB54	Protein FAM216A OS=Mus musculus OX=10090 GN=Fam216a PE=2 SV=1	

Hmcn1	D3YXG0	Hemicentin-1 OS=Mus musculus OX=10090 GN=Hmcn1 PE=1 SV=1	
Znf496	Q5SXI5	Zinc finger protein 496 OS=Mus musculus OX=10090 GN=Znf496 PE=1 SV=1	
	Q8BGC1	UPF0489 protein C5orf22 homolog OS=Mus musculus OX=10090 PE=1 SV=1	
Tec	P24604	Tyrosine-protein kinase Tec OS=Mus musculus OX=10090 GN=Tec PE=1 SV=2	
Zp2	P20239	Zona pellucida sperm-binding protein 2 OS=Mus musculus OX=10090 GN=Zp2 PE=1 SV=1	
Mucl3	Q3TNW5	Mucin-like protein 3 OS=Mus musculus OX=10090 GN=Mucl3 PE=2 SV=1	
Gsdmdc1	Q9D8T2	Gasdermin-D OS=Mus musculus OX=10090 GN=Gsdmdc1 PE=1 SV=1	
Trim67	Q505D9	Tripartite motif-containing protein 67 OS=Mus musculus OX=10090 GN=Trim67 PE=2 SV=1	
Cmya5	Q70KF4	Cardiomyopathy-associated protein 5 OS=Mus musculus OX=10090 GN=Cmya5 PE=1 SV=2	
Frem2	Q6NVD0	FRAS1-related extracellular matrix protein 2 OS=Mus musculus OX=10090 GN=Frem2 PE=1 SV=2	
Dennd2c	Q6P9P8	DENN domain-containing protein 2C OS=Mus musculus OX=10090 GN=Dennd2c PE=1 SV=1	
Znf507	Q6ZPY5	Zinc finger protein 507 OS=Mus musculus OX=10090 GN=Znf507 PE=2 SV=2	
Ebf2	O08792	Transcription factor COE2 OS=Mus musculus OX=10090 GN=Ebf2 PE=1 SV=4	
Sipa1l1	Q8C0T5	Signal-induced proliferation-associated 1-like protein 1 OS=Mus musculus OX=10090 GN=Sipa1l1 P	9.735
Rxra	P28700	Retinoic acid receptor RXR-alpha OS=Mus musculus OX=10090 GN=Rxra PE=1 SV=1	1.51
Bmp2k	Q91Z96	BMP-2-inducible protein kinase OS=Mus musculus OX=10090 GN=Bmp2k PE=1 SV=1	0.73
Smap2	Q7TN29	Stromal membrane-associated protein 2 OS=Mus musculus OX=10090 GN=Smap2 PE=1 SV=1	4.736666667
Rab27b	Q99P58	Ras-related protein Rab-27B OS=Mus musculus OX=10090 GN=Rab27b PE=1 SV=3	0.92
Arfrp1	Q8BXL7	ADP-ribosylation factor-related protein 1 OS=Mus musculus OX=10090 GN=Arfrp1 PE=1 SV=2	0.86
Ifitm2	Q99J93	Interferon-induced transmembrane protein 2 OS=Mus musculus OX=10090 GN=Ifitm2 PE=1 SV=1	0.993333333
Prkd1	Q62101	Serine/threonine-protein kinase D1 OS=Mus musculus OX=10090 GN=Prkd1 PE=1 SV=2	1.295
Numbl	O08919	Numb-like protein OS=Mus musculus OX=10090 GN=Numbl PE=1 SV=3	0.633333333
Sumo2	P61957	Small ubiquitin-related modifier 2 OS=Mus musculus OX=10090 GN=Sumo2 PE=1 SV=1	0.896666667
Mastl	Q8C0P0	Serine/threonine-protein kinase greatwall OS=Mus musculus OX=10090 GN=Mastl PE=2 SV=2	
Meis2	P97367	Homeobox protein Meis2 OS=Mus musculus OX=10090 GN=Meis2 PE=1 SV=2	
Fam76b	Q80XP8	Protein FAM76B OS=Mus musculus OX=10090 GN=Fam76b PE=1 SV=1	
Sumo3	Q9Z172	Small ubiquitin-related modifier 3 OS=Mus musculus OX=10090 GN=Sumo3 PE=1 SV=1	0.86
Rxrb	P28704	Retinoic acid receptor RXR-beta OS=Mus musculus OX=10090 GN=Rxrb PE=1 SV=2	
Kdelr1	Q99JH8	ER lumen protein-retaining receptor 1 OS=Mus musculus OX=10090 GN=Kdelr1 PE=1 SV=1	1.21
Prrx2	Q06348	Paired mesoderm homeobox protein 2 OS=Mus musculus OX=10090 GN=Prrx2 PE=2 SV=2	
Tcf4	Q60722	Transcription factor 4 OS=Mus musculus OX=10090 GN=Tcf4 PE=1 SV=1	1.46
Atf1	P81269	Cyclic AMP-dependent transcription factor ATF-1 OS=Mus musculus OX=10090 GN=Atf1 PE=1 SV=1	0.673333333
Helz2	E9QAM5	Helicase with zinc finger domain 2 OS=Mus musculus OX=10090 GN=Helz2 PE=1 SV=1	
Hoxc6	P10629	Homeobox protein Hox-C6 OS=Mus musculus OX=10090 GN=Hoxc6 PE=2 SV=2	

Ppfia2	Q8BSS9	Liprin-alpha-2 OS=Mus musculus OX=10090 GN=Ppfia2 PE=1 SV=2	
Rab30	Q923S9	Ras-related protein Rab-30 OS=Mus musculus OX=10090 GN=Rab30 PE=1 SV=1	0.9
Bod1	Q5SQY2	Biorientation of chromosomes in cell division protein 1 OS=Mus musculus OX=10090 GN=Bod1 PE=	
Tnrc6a	Q3UHK8	Trinucleotide repeat-containing gene 6A protein OS=Mus musculus OX=10090 GN=Tnrc6a PE=1 SV=	
Ube2b	P63147	Ubiquitin-conjugating enzyme E2 B OS=Mus musculus OX=10090 GN=Ube2b PE=1 SV=1	0.716666667
Ssbp3	Q9D032	Single-stranded DNA-binding protein 3 OS=Mus musculus OX=10090 GN=Ssbp3 PE=1 SV=2	
Samd4b	Q80XS6	Protein Smaug homolog 2 OS=Mus musculus OX=10090 GN=Samd4b PE=1 SV=1	
Ppfia3	P60469	Liprin-alpha-3 OS=Mus musculus OX=10090 GN=Ppfia3 PE=1 SV=2	
Map3k2	Q61083	Mitogen-activated protein kinase kinase kinase 2 OS=Mus musculus OX=10090 GN=Map3k2 PE=1 SV	
Ltf	P08071	Lactotransferrin OS=Mus musculus OX=10090 GN=Ltf PE=1 SV=4	0.26
Ube2d1	P61080	Ubiquitin-conjugating enzyme E2 D1 OS=Mus musculus OX=10090 GN=Ube2d1 PE=1 SV=1	0.916666667
Helz	Q6DFV5	Probable helicase with zinc finger domain OS=Mus musculus OX=10090 GN=Helz PE=1 SV=2	
Rel	P15307	Proto-oncogene c-Rel OS=Mus musculus OX=10090 GN=Rel PE=1 SV=2	
Kifc3	O35231	Kinesin-like protein KIFC3 OS=Mus musculus OX=10090 GN=Kifc3 PE=1 SV=4	0.42
Rassf2	Q8BMS9	Ras association domain-containing protein 2 OS=Mus musculus OX=10090 GN=Rassf2 PE=1 SV=1	0.945
Daxx	O35613	Death domain-associated protein 6 OS=Mus musculus OX=10090 GN=Daxx PE=1 SV=1	
Map1lc3a	Q91VR7	Microtubule-associated proteins 1A/1B light chain 3A OS=Mus musculus OX=10090 GN=Map1lc3a I	0.975
Pzp	Q61838	Pregnancy zone protein OS=Mus musculus OX=10090 GN=Pzp PE=1 SV=3	0.11
Hbb-y	P02104	Hemoglobin subunit epsilon-Y2 OS=Mus musculus OX=10090 GN=Hbb-y PE=1 SV=2	0
Tbc1d4	Q8BYJ6	TBC1 domain family member 4 OS=Mus musculus OX=10090 GN=Tbc1d4 PE=1 SV=2	
Pard6b	Q9JK83	Partitioning defective 6 homolog beta OS=Mus musculus OX=10090 GN=Pard6b PE=1 SV=2	0.983333333
Runx3	Q64131	Runt-related transcription factor 3 OS=Mus musculus OX=10090 GN=Runx3 PE=1 SV=3	
Rrad	O88667	GTP-binding protein RAD OS=Mus musculus OX=10090 GN=Rrad PE=1 SV=1	1.22
Lrrc1	Q80VQ1	Leucine-rich repeat-containing protein 1 OS=Mus musculus OX=10090 GN=Lrrc1 PE=1 SV=2	
Fignl1	Q8BPY9	Fidgetin-like protein 1 OS=Mus musculus OX=10090 GN=Fignl1 PE=1 SV=1	
Gabarap	Q9DCD6	Gamma-aminobutyric acid receptor-associated protein OS=Mus musculus OX=10090 GN=Gabarap I	0.83
Raet1e	Q9CZQ6	Retinoic acid early-inducible protein 1-epsilon OS=Mus musculus OX=10090 GN=Raet1e PE=1 SV=1	
Rhot2	Q8JZN7	Mitochondrial Rho GTPase 2 OS=Mus musculus OX=10090 GN=Rhot2 PE=1 SV=1	
Fgfr1	P16092	Fibroblast growth factor receptor 1 OS=Mus musculus OX=10090 GN=Fgfr1 PE=1 SV=2	0.976666667
Fgfr3	Q61851	Fibroblast growth factor receptor 3 OS=Mus musculus OX=10090 GN=Fgfr3 PE=1 SV=1	0.976666667
Fgfr2	P21803	Fibroblast growth factor receptor 2 OS=Mus musculus OX=10090 GN=Fgfr2 PE=1 SV=4	0.976666667
Fgfr4	Q03142	Fibroblast growth factor receptor 4 OS=Mus musculus OX=10090 GN=Fgfr4 PE=1 SV=3	0.976666667
Zscan21	Q07231	Zinc finger and SCAN domain-containing protein 21 OS=Mus musculus OX=10090 GN=Zscan21 PE=2	
Taok3	Q8BYC6	Serine/threonine-protein kinase TAO3 OS=Mus musculus OX=10090 GN=Taok3 PE=1 SV=2	

Bnip2	O54940	BCL2/adenovirus E1B 19 kDa protein-interacting protein 2 OS=Mus musculus OX=10090 GN=Bnip2	1.423333333
Clic6	Q8BHB9	Chloride intracellular channel protein 6 OS=Mus musculus OX=10090 GN=Clic6 PE=1 SV=1	
Clic5	Q8BXK9	Chloride intracellular channel protein 5 OS=Mus musculus OX=10090 GN=Clic5 PE=1 SV=1	
Rps6kb1	Q8BSK8	Ribosomal protein S6 kinase beta-1 OS=Mus musculus OX=10090 GN=Rps6kb1 PE=1 SV=2	
Wdr19	Q3UGF1	WD repeat-containing protein 19 OS=Mus musculus OX=10090 GN=Wdr19 PE=1 SV=1	
Gmpr	Q9DCZ1	GMP reductase 1 OS=Mus musculus OX=10090 GN=Gmpr PE=1 SV=1	
Rps6kb2	Q9Z1M4	Ribosomal protein S6 kinase beta-2 OS=Mus musculus OX=10090 GN=Rps6kb2 PE=1 SV=1	
Magi1	Q6RHR9	Membrane-associated guanylate kinase WW and PDZ domain-containing protein 1 OS=Mus muscul	
Mapk12	O08911	Mitogen-activated protein kinase 12 OS=Mus musculus OX=10090 GN=Mapk12 PE=1 SV=1	
Tnrc6c	Q3UHC0	Trinucleotide repeat-containing gene 6C protein OS=Mus musculus OX=10090 GN=Tnrc6c PE=1 SV=	
Hoxc9	P09633	Homeobox protein Hox-C9 OS=Mus musculus OX=10090 GN=Hoxc9 PE=2 SV=2	
Pfkfb2	P70265	6-phosphofructo-2-kinase/fructose-2 6-bisphosphatase 2 OS=Mus musculus OX=10090 GN=Pfkfb2 P	
Zc3h6	Q8BYK8	Zinc finger CCCH domain-containing protein 6 OS=Mus musculus OX=10090 GN=Zc3h6 PE=2 SV=2	
Csnk1g1	Q8BTH8	Casein kinase I isoform gamma-1 OS=Mus musculus OX=10090 GN=Csnk1g1 PE=1 SV=2	
Vwa3a	Q3UVV9	von Willebrand factor A domain-containing protein 3A OS=Mus musculus OX=10090 GN=Vwa3a PE	
Tdrd5	Q5VCS6	Tudor domain-containing protein 5 OS=Mus musculus OX=10090 GN=Tdrd5 PE=1 SV=3	
Mast1	Q9R1L5	Microtubule-associated serine/threonine-protein kinase 1 OS=Mus musculus OX=10090 GN=Mast1	
Hint2	Q9D0S9	Histidine triad nucleotide-binding protein 2 mitochondrial OS=Mus musculus OX=10090 GN=Hint2	3.945
Erbb2	P70424	Receptor tyrosine-protein kinase erbB-2 OS=Mus musculus OX=10090 GN=Erbb2 PE=1 SV=3	
Bicd1	Q8BR07	Protein bicaudal D homolog 1 OS=Mus musculus OX=10090 GN=Bicd1 PE=1 SV=2	
Gpatch11	Q3UFS4	G patch domain-containing protein 11 OS=Mus musculus OX=10090 GN=Gpatch11 PE=1 SV=2	1.7
Wwc1	Q5SXA9	Protein KIBRA OS=Mus musculus OX=10090 GN=Wwc1 PE=1 SV=1	
Kcnq2	Q9Z351	Potassium voltage-gated channel subfamily KQT member 2 OS=Mus musculus OX=10090 GN=Kcnq2	
Setd1b	Q8CFT2	Histone-lysine N-methyltransferase SETD1B OS=Mus musculus OX=10090 GN=Setd1b PE=1 SV=2	
Kat6b	Q8BRB7	Histone acetyltransferase KAT6B OS=Mus musculus OX=10090 GN=Kat6b PE=1 SV=3	
Vps18	Q8R307	Vacuolar protein sorting-associated protein 18 homolog OS=Mus musculus OX=10090 GN=Vps18 P	12.73
Spp1	P10923	Osteopontin OS=Mus musculus OX=10090 GN=Spp1 PE=1 SV=1	0.78
Zfand5	O88878	AN1-type zinc finger protein 5 OS=Mus musculus OX=10090 GN=Zfand5 PE=1 SV=1	0.63
Polk	Q9QUG2	DNA polymerase kappa OS=Mus musculus OX=10090 GN=Polk PE=1 SV=1	1.07
Kdm6a	O70546	Lysine-specific demethylase 6A OS=Mus musculus OX=10090 GN=Kdm6a PE=1 SV=2	1.123333333
Dph6	Q9CQ28	Diphthine--ammonia ligase OS=Mus musculus OX=10090 GN=Dph6 PE=1 SV=1	0.515
Syde1	Q9DBZ9	Rho GTPase-activating protein SYDE1 OS=Mus musculus OX=10090 GN=Syde1 PE=1 SV=1	1
Cks2	P56390	Cyclin-dependent kinases regulatory subunit 2 OS=Mus musculus OX=10090 GN=Cks2 PE=1 SV=1	0.956666667
Sod3	O09164	Extracellular superoxide dismutase [Cu-Zn] OS=Mus musculus OX=10090 GN=Sod3 PE=1 SV=1	1.03

Ovca2	Q9D7E3	Esterase OVCA2 OS=Mus musculus OX=10090 GN=Ovca2 PE=1 SV=1	0.773333333
Atp2c1	Q80XR2	Calcium-transporting ATPase type 2C member 1 OS=Mus musculus OX=10090 GN=Atp2c1 PE=1 SV=:	2.255
Fn3krp	Q8K274	Ketosamine-3-kinase OS=Mus musculus OX=10090 GN=Fn3krp PE=1 SV=2	0.535
Cenph	Q9QYM8	Centromere protein H OS=Mus musculus OX=10090 GN=Cenph PE=1 SV=1	1.26
Eef2k	O08796	Eukaryotic elongation factor 2 kinase OS=Mus musculus OX=10090 GN=Eef2k PE=1 SV=1	1.81
Mt1	P02802	Metallothionein-1 OS=Mus musculus OX=10090 GN=Mt1 PE=1 SV=1	1.166666667
Cirbp	P60824	Cold-inducible RNA-binding protein OS=Mus musculus OX=10090 GN=Cirbp PE=1 SV=1	0.91
Leng8	Q8CBY3	Leukocyte receptor cluster member 8 homolog OS=Mus musculus OX=10090 GN=Leng8 PE=1 SV=1	20.905
Scyl3	Q9DBQ7	Protein-associating with the carboxyl-terminal domain of ezrin OS=Mus musculus OX=10090 GN=Sc	0.94
Snrpc	Q62241	U1 small nuclear ribonucleoprotein C OS=Mus musculus OX=10090 GN=Snrpc PE=1 SV=1	0.863333333
	Q80X32	UPF0461 protein C5orf24 homolog OS=Mus musculus OX=10090 PE=2 SV=1	0.95
Tti1	Q91V83	TELO2-interacting protein 1 homolog OS=Mus musculus OX=10090 GN=Tti1 PE=1 SV=2	0.79
Isoc2a	P85094	Isochorismatase domain-containing protein 2A OS=Mus musculus OX=10090 GN=Isoc2a PE=1 SV=1	0.94
Kiaa0754	Q69ZZ9	Uncharacterized protein KIAA0754 OS=Mus musculus OX=10090 GN=Kiaa0754 PE=2 SV=2	1.06
Noa1	Q9JJG9	Nitric oxide-associated protein 1 OS=Mus musculus OX=10090 GN=Noa1 PE=1 SV=1	0.976666667
Gpsm1	Q6IR34	G-protein-signaling modulator 1 OS=Mus musculus OX=10090 GN=Gpsm1 PE=1 SV=3	0.996666667
Lamb2	Q61292	Laminin subunit beta-2 OS=Mus musculus OX=10090 GN=Lamb2 PE=1 SV=2	1.475
Vps11	Q91W86	Vacuolar protein sorting-associated protein 11 homolog OS=Mus musculus OX=10090 GN=Vps11 PI	
Crbn	Q8C7D2	Protein cereblon OS=Mus musculus OX=10090 GN=Crbn PE=1 SV=1	0.636666667
Ttyh3	Q6P5F7	Protein tweety homolog 3 OS=Mus musculus OX=10090 GN=Ttyh3 PE=1 SV=1	1.023333333
Cdkn1b	P46414	Cyclin-dependent kinase inhibitor 1B OS=Mus musculus OX=10090 GN=Cdkn1b PE=1 SV=2	1.093333333
Chchd1	Q9CQA6	Coiled-coil-helix-coiled-coil-helix domain-containing protein 1 OS=Mus musculus OX=10090 GN=Cl	1.32
Slc1a4	O35874	Neutral amino acid transporter A OS=Mus musculus OX=10090 GN=Slc1a4 PE=1 SV=1	0.783333333
Marcksl1	P28667	MARCKS-related protein OS=Mus musculus OX=10090 GN=Marcksl1 PE=1 SV=2	0.683333333
Ttc33	Q9D6K7	Tetratricopeptide repeat protein 33 OS=Mus musculus OX=10090 GN=Ttc33 PE=1 SV=1	0.92
Usp3	Q91W36	Ubiquitin carboxyl-terminal hydrolase 3 OS=Mus musculus OX=10090 GN=Usp3 PE=2 SV=1	0.54
Arhgap12	Q8C0D4	Rho GTPase-activating protein 12 OS=Mus musculus OX=10090 GN=Arhgap12 PE=1 SV=2	1.406666667
Carhsp1	Q9CR86	Calcium-regulated heat stable protein 1 OS=Mus musculus OX=10090 GN=Carhsp1 PE=1 SV=1	0.696666667
Lemd2	Q6DVA0	LEM domain-containing protein 2 OS=Mus musculus OX=10090 GN=Lemd2 PE=1 SV=1	0.923333333
Mrpl50	Q8VDT9	39S ribosomal protein L50 mitochondrial OS=Mus musculus OX=10090 GN=Mrpl50 PE=1 SV=2	0.996666667
Med31	Q9CXU1	Mediator of RNA polymerase II transcription subunit 31 OS=Mus musculus OX=10090 GN=Med31 PI	0.91
Bcas3	Q8CCN5	Breast carcinoma-amplified sequence 3 homolog OS=Mus musculus OX=10090 GN=Bcas3 PE=1 SV=2	0.93
Nusap1	Q9ERH4	Nucleolar and spindle-associated protein 1 OS=Mus musculus OX=10090 GN=Nusap1 PE=1 SV=1	1.406666667
Kif21a	Q9QXL2	Kinesin-like protein KIF21A OS=Mus musculus OX=10090 GN=Kif21a PE=1 SV=2	0.813333333

Slc44a1	Q6X893	Choline transporter-like protein 1 OS=Mus musculus OX=10090 GN=Slc44a1 PE=1 SV=3	0.956666667
Kti12	Q9D1R2	Protein KTI12 homolog OS=Mus musculus OX=10090 GN=Kti12 PE=1 SV=1	0.793333333
	Q922M7	Ashwin OS=Mus musculus OX=10090 PE=1 SV=1	0.7
Foxo3	Q9WVH4	Forkhead box protein O3 OS=Mus musculus OX=10090 GN=Foxo3 PE=1 SV=1	1.27
Dck	P43346	Deoxycytidine kinase OS=Mus musculus OX=10090 GN=Dck PE=1 SV=1	0.883333333
Ggact	Q923B0	Gamma-glutamylaminecyclotransferase OS=Mus musculus OX=10090 GN=Ggact PE=1 SV=1	2.33
Limd2	Q8BGB5	LIM domain-containing protein 2 OS=Mus musculus OX=10090 GN=Limd2 PE=1 SV=1	0.833333333
Tor1aip2	Q9ER81	Torsin-1A-interacting protein 2 isoform IFRG15 OS=Mus musculus OX=10090 GN=Tor1aip2 PE=1 SV	0.786666667
Smg6	P61406	Telomerase-binding protein EST1A OS=Mus musculus OX=10090 GN=Smg6 PE=1 SV=1	1.27
Cacul1	Q8R0X2	CDK2-associated and cullin domain-containing protein 1 OS=Mus musculus OX=10090 GN=Cacul1 I	0.716666667
Med28	Q920D3	Mediator of RNA polymerase II transcription subunit 28 OS=Mus musculus OX=10090 GN=Med28 PI	0.733333333
Jmjd1c	Q69ZK6	Probable JmjC domain-containing histone demethylation protein 2C OS=Mus musculus OX=10090	
Ercc3	P49135	General transcription and DNA repair factor IIH helicase subunit XPB OS=Mus musculus OX=10090 (	1.033333333
Msra	Q9D6Y7	Mitochondrial peptide methionine sulfoxide reductase OS=Mus musculus OX=10090 GN=Msra PE=1	0.68
Tnc	Q80YX1	Tenascin OS=Mus musculus OX=10090 GN=Tnc PE=1 SV=1	
Chmp5	Q9D7S9	Charged multivesicular body protein 5 OS=Mus musculus OX=10090 GN=Chmp5 PE=1 SV=1	1.053333333
Qrsl1	Q9CZN8	Glutamyl-tRNA(Gln) amidotransferase subunit A mitochondrial OS=Mus musculus OX=10090 GN=C	0.76
Angptl2	Q9R045	Angiopoietin-related protein 2 OS=Mus musculus OX=10090 GN=Angptl2 PE=2 SV=2	0.743333333
Cdc42ep4	Q9JM96	Cdc42 effector protein 4 OS=Mus musculus OX=10090 GN=Cdc42ep4 PE=1 SV=1	1.01
Rhbdd1	Q8BHC7	Rhomboid-related protein 4 OS=Mus musculus OX=10090 GN=Rhbdd1 PE=1 SV=1	1.193333333
Phf23	Q8BSN5	PHD finger protein 23 OS=Mus musculus OX=10090 GN=Phf23 PE=2 SV=2	0.82
Hgsnat	Q3UDW8	Heparan-alpha-glucosaminide N-acetyltransferase OS=Mus musculus OX=10090 GN=Hgsnat PE=1 SV	0.89
Timm17b	Q9Z0V7	Mitochondrial import inner membrane translocase subunit Tim17-B OS=Mus musculus OX=10090 (	6.506666667
Derpc	P0CG14	Decreased expression in renal and prostate cancer protein OS=Mus musculus OX=10090 GN=Derpc I	1.043333333
Tnfrsf10b	Q9QZM4	Tumor necrosis factor receptor superfamily member 10B OS=Mus musculus OX=10090 GN=Tnfrsf10	0.835
Med21	Q9CQ39	Mediator of RNA polymerase II transcription subunit 21 OS=Mus musculus OX=10090 GN=Med21 PI	0.713333333
Cd151	O35566	CD151 antigen OS=Mus musculus OX=10090 GN=Cd151 PE=1 SV=2	0.75
Ap1s1	P61967	AP-1 complex subunit sigma-1A OS=Mus musculus OX=10090 GN=Ap1s1 PE=1 SV=1	0.866666667
Pcdhga4	Q91XY4	Protocadherin gamma-A4 OS=Mus musculus OX=10090 GN=Pcdhga4 PE=1 SV=1	0.796666667
Ppt1	O88531	Palmitoyl-protein thioesterase 1 OS=Mus musculus OX=10090 GN=Ppt1 PE=1 SV=2	0.79
Nedd8	P29595	NEDD8 OS=Mus musculus OX=10090 GN=Nedd8 PE=1 SV=2	0.856666667
Bola3	Q8CEI1	BolA-like protein 3 OS=Mus musculus OX=10090 GN=Bola3 PE=1 SV=1	1.01
Aup1	P70295	Ancient ubiquitous protein 1 OS=Mus musculus OX=10090 GN=Aup1 PE=1 SV=1	1.753333333
Wdr44	Q6NVE8	WD repeat-containing protein 44 OS=Mus musculus OX=10090 GN=Wdr44 PE=1 SV=1	0.896666667

Unk	Q8BL48	RING finger protein unkempt homolog OS=Mus musculus OX=10090 GN=Unk PE=1 SV=1	0.906666667
Rps28	P62858	40S ribosomal protein S28 OS=Mus musculus OX=10090 GN=Rps28 PE=1 SV=1	0.943333333
Anapc11	Q9CPX9	Anaphase-promoting complex subunit 11 OS=Mus musculus OX=10090 GN=Anapc11 PE=3 SV=1	0.626666667
Tent4b	Q68ED3	Terminal nucleotidyltransferase 4B OS=Mus musculus OX=10090 GN=Tent4b PE=1 SV=2	0.62
Tmem30a	Q8VEK0	Cell cycle control protein 50A OS=Mus musculus OX=10090 GN=Tmem30a PE=1 SV=1	0.963333333
Dyrk1a	Q61214	Dual specificity tyrosine-phosphorylation-regulated kinase 1A OS=Mus musculus OX=10090 GN=Dyr	0.9
Dusp12	Q9D0T2	Dual specificity protein phosphatase 12 OS=Mus musculus OX=10090 GN=Dusp12 PE=2 SV=1	1.033333333
Pten	O08586	Phosphatidylinositol 3 4 5-trisphosphate 3-phosphatase and dual-specificity protein phosphatase P	0.99
Man1a2	P39098	Mannosyl-oligosaccharide 1 2-alpha-mannosidase IB OS=Mus musculus OX=10090 GN=Man1a2 PE=	0.89
Chmp6	P0C0A3	Charged multivesicular body protein 6 OS=Mus musculus OX=10090 GN=Chmp6 PE=1 SV=2	1.033333333
Kin	Q8K339	DNA/RNA-binding protein KIN17 OS=Mus musculus OX=10090 GN=Kin PE=1 SV=1	0.465
Slc7a6	Q8BGK6	Y+L amino acid transporter 2 OS=Mus musculus OX=10090 GN=Slc7a6 PE=1 SV=1	0.93
Jagn1	Q5XKN4	Protein jagunal homolog 1 OS=Mus musculus OX=10090 GN=Jagn1 PE=1 SV=2	0.83
Brap	Q99MP8	BRCA1-associated protein OS=Mus musculus OX=10090 GN=Brap PE=1 SV=1	1.806666667
Kirrel1	Q80W68	Kin of IRRE-like protein 1 OS=Mus musculus OX=10090 GN=Kirrel1 PE=1 SV=1	0.79
Mboat7	Q8CHK3	Lysophospholipid acyltransferase 7 OS=Mus musculus OX=10090 GN=Mboat7 PE=1 SV=1	1.086666667
Atg16l1	Q8C0J2	Autophagy-related protein 16-1 OS=Mus musculus OX=10090 GN=Atg16l1 PE=1 SV=1	0.91
Timm29	Q8BGX2	Mitochondrial import inner membrane translocase subunit Tim29 OS=Mus musculus OX=10090 GN	0.64
Ier3ip1	Q9CR20	Immediate early response 3-interacting protein 1 OS=Mus musculus OX=10090 GN=Ier3ip1 PE=3 SV	0.773333333
Slc12a9	Q99MR3	Solute carrier family 12 member 9 OS=Mus musculus OX=10090 GN=Slc12a9 PE=1 SV=2	1.26
Atp5md	Q78IK2	ATP synthase membrane subunit DAPIT mitochondrial OS=Mus musculus OX=10090 GN=Atp5md P	0.993333333
Brat1	Q8C3R1	BRCA1-associated ATM activator 1 OS=Mus musculus OX=10090 GN=Brat1 PE=1 SV=1	1.653333333
Mettl3	Q8C3P7	N6-adenosine-methyltransferase subunit METTL3 OS=Mus musculus OX=10090 GN=Mettl3 PE=1 SV=	0.94
Ndufaf1	Q9CWX2	Complex I intermediate-associated protein 30 mitochondrial OS=Mus musculus OX=10090 GN=Ndu	0.67
Tacc1	Q6Y685	Transforming acidic coiled-coil-containing protein 1 OS=Mus musculus OX=10090 GN=Tacc1 PE=1 S	1.226666667
Loxl4	Q924C6	Lysyl oxidase homolog 4 OS=Mus musculus OX=10090 GN=Loxl4 PE=2 SV=2	1.233333333
Ampd3	O08739	AMP deaminase 3 OS=Mus musculus OX=10090 GN=Ampd3 PE=1 SV=2	0.876666667
Vamp4	O70480	Vesicle-associated membrane protein 4 OS=Mus musculus OX=10090 GN=Vamp4 PE=1 SV=1	0.986666667
Ten1	Q9D7K2	CST complex subunit TEN1 OS=Mus musculus OX=10090 GN=Ten1 PE=1 SV=1	0.463333333
Xiap	Q60989	E3 ubiquitin-protein ligase XIAP OS=Mus musculus OX=10090 GN=Xiap PE=1 SV=2	0.66
Ncbp2	Q9CQ49	Nuclear cap-binding protein subunit 2 OS=Mus musculus OX=10090 GN=Ncbp2 PE=1 SV=1	1.016666667
Ric1	Q69ZJ7	Guanine nucleotide exchange factor subunit RIC1 OS=Mus musculus OX=10090 GN=Ric1 PE=1 SV=2	0.745
Dph1	Q5NCQ5	2-(3-amino-3-carboxypropyl)histidine synthase subunit 1 OS=Mus musculus OX=10090 GN=Dph1 P	0.86
Mtfr1l	Q9CWE0	Mitochondrial fission regulator 1-like OS=Mus musculus OX=10090 GN=Mtfr1l PE=1 SV=1	0.766666667

Chchd4	Q8VEA4	Mitochondrial intermembrane space import and assembly protein 40 OS=Mus musculus OX=10090	0.896666667
Bsdc1	Q80Y55	BSD domain-containing protein 1 OS=Mus musculus OX=10090 GN=Bsdc1 PE=1 SV=1	
Nqo2	Q9JI75	Ribosyldihydronicotinamide dehydrogenase [quinone] OS=Mus musculus OX=10090 GN=Nqo2 PE=1	0.95
Cdk2ap1	O35207	Cyclin-dependent kinase 2-associated protein 1 OS=Mus musculus OX=10090 GN=Cdk2ap1 PE=1 SV:	0.933333333
Lage3	Q9CR70	EKC/KEOPS complex subunit Lage3 OS=Mus musculus OX=10090 GN=Lage3 PE=2 SV=1	0.933333333
Slc39a7	Q31125	Zinc transporter SLC39A7 OS=Mus musculus OX=10090 GN=Slc39a7 PE=1 SV=2	0.81
Fanci	Q8K368	Fanconi anemia group I protein homolog OS=Mus musculus OX=10090 GN=Fanci PE=1 SV=2	1.74
Eif2ak2	Q03963	Interferon-induced double-stranded RNA-activated protein kinase OS=Mus musculus OX=10090 GN	0.686666667
Sigmar1	O55242	Sigma non-opioid intracellular receptor 1 OS=Mus musculus OX=10090 GN=Sigmar1 PE=1 SV=1	1.483333333
Cdca2	Q14B71	Cell division cycle-associated protein 2 OS=Mus musculus OX=10090 GN=Cdca2 PE=1 SV=2	1.06
Dnmbp	Q6TXD4	Dynamin-binding protein OS=Mus musculus OX=10090 GN=Dnmbp PE=1 SV=2	1.825
Cacna2d1	O08532	Voltage-dependent calcium channel subunit alpha-2/delta-1 OS=Mus musculus OX=10090 GN=Cacr	1.115
Aplp2	Q06335	Amyloid-like protein 2 OS=Mus musculus OX=10090 GN=Aplp2 PE=1 SV=4	0.7
Urgcp	Q5NCI0	Up-regulator of cell proliferation OS=Mus musculus OX=10090 GN=Urgcp PE=2 SV=1	0.76
Cdkn2aipnl	Q9D211	CDKN2AIP N-terminal-like protein OS=Mus musculus OX=10090 GN=Cdkn2aipnl PE=3 SV=1	1.066666667
Bud13	Q8R149	BUD13 homolog OS=Mus musculus OX=10090 GN=Bud13 PE=1 SV=1	0.9
Paat	Q9D2Q3	ATPase PAAT OS=Mus musculus OX=10090 GN=Paat PE=2 SV=1	0.7
Mzt1	Q8BUR9	Mitotic-spindle organizing protein 1 OS=Mus musculus OX=10090 GN=Mzt1 PE=1 SV=1	0.816666667
B3glct	Q8BHT6	Beta-1 3-glucosyltransferase OS=Mus musculus OX=10090 GN=B3glct PE=1 SV=3	0.985
Hjurp	Q6PG16	Holliday junction recognition protein OS=Mus musculus OX=10090 GN=Hjurp PE=1 SV=1	0.956666667
Kifap3	P70188	Kinesin-associated protein 3 OS=Mus musculus OX=10090 GN=Kifap3 PE=1 SV=1	1.105
Cep170b	Q80U49	Centrosomal protein of 170 kDa protein B OS=Mus musculus OX=10090 GN=Cep170b PE=1 SV=2	1.293333333
Uqcc2	Q9CQY6	Ubiquinol-cytochrome-c reductase complex assembly factor 2 OS=Mus musculus OX=10090 GN=Uq	1.19
Ints11	Q9CWS4	Integrator complex subunit 11 OS=Mus musculus OX=10090 GN=Ints11 PE=1 SV=1	1.08
Tp53bp2	Q8CG79	Apoptosis-stimulating of p53 protein 2 OS=Mus musculus OX=10090 GN=Tp53bp2 PE=1 SV=3	
Rybp	Q8CCI5	RING1 and YY1-binding protein OS=Mus musculus OX=10090 GN=Rybp PE=1 SV=1	1.13
Gemin7	Q9CWY4	Gem-associated protein 7 OS=Mus musculus OX=10090 GN=Gemin7 PE=2 SV=1	0.826666667
Scaf1	Q5U4C3	Splicing factor arginine/serine-rich 19 OS=Mus musculus OX=10090 GN=Scaf1 PE=1 SV=1	0.9
Frmd4a	Q8BIE6	FERM domain-containing protein 4A OS=Mus musculus OX=10090 GN=Frmd4a PE=1 SV=2	1.156666667
Pla2g6	P97819	85/88 kDa calcium-independent phospholipase A2 OS=Mus musculus OX=10090 GN=Pla2g6 PE=1 S	1.13
Pigu	Q8K358	Phosphatidylinositol glycan anchor biosynthesis class U protein OS=Mus musculus OX=10090 GN=F	1.076666667
Znf771	Q8BJ90	Zinc finger protein 771 OS=Mus musculus OX=10090 GN=Znf771 PE=2 SV=1	0.823333333
Taf6	Q62311	Transcription initiation factor TFIID subunit 6 OS=Mus musculus OX=10090 GN=Taf6 PE=1 SV=1	1.43
Twsg1	Q9EP52	Twisted gastrulation protein homolog 1 OS=Mus musculus OX=10090 GN=Twsg1 PE=1 SV=1	1.276666667

Zswim8	Q3UHH1	Zinc finger SWIM domain-containing protein 8 OS=Mus musculus OX=10090 GN=Zswim8 PE=1 SV=1	1.07
Ssr3	Q9DCF9	Translocon-associated protein subunit gamma OS=Mus musculus OX=10090 GN=Ssr3 PE=1 SV=1	0.836666667
Atf2	P16951	Cyclic AMP-dependent transcription factor ATF-2 OS=Mus musculus OX=10090 GN=Atf2 PE=1 SV=2	0.84
Eny2	Q9JIX0	Transcription and mRNA export factor ENY2 OS=Mus musculus OX=10090 GN=Eny2 PE=1 SV=1	0.92
Slc6a6	O35316	Sodium- and chloride-dependent taurine transporter OS=Mus musculus OX=10090 GN=Slc6a6 PE=1	0.943333333
Mrpl20	Q9CQL4	39S ribosomal protein L20 mitochondrial OS=Mus musculus OX=10090 GN=Mrpl20 PE=1 SV=1	0.866666667
Trappc1	Q5NCF2	Trafficking protein particle complex subunit 1 OS=Mus musculus OX=10090 GN=Trappc1 PE=1 SV=1	1.66
Praf2	Q9JIG8	PRA1 family protein 2 OS=Mus musculus OX=10090 GN=Praf2 PE=1 SV=1	0.796666667
Rint1	Q8BZ36	RAD50-interacting protein 1 OS=Mus musculus OX=10090 GN=Rint1 PE=1 SV=2	
Rnf121	Q8R1Z9	RING finger protein 121 OS=Mus musculus OX=10090 GN=Rnf121 PE=1 SV=1	1.07
Dse	Q8BLI4	Dermatan-sulfate epimerase OS=Mus musculus OX=10090 GN=Dse PE=2 SV=1	1.205
	Q8VDP2	UPF0428 protein CXorf56 homolog OS=Mus musculus OX=10090 PE=1 SV=1	0.966666667
Zfand6	Q9DCH6	AN1-type zinc finger protein 6 OS=Mus musculus OX=10090 GN=Zfand6 PE=1 SV=1	0.83
Mrpl55	Q9CZ83	39S ribosomal protein L55 mitochondrial OS=Mus musculus OX=10090 GN=Mrpl55 PE=1 SV=1	1.236666667
Dnajc24	Q91ZF0	DnaJ homolog subfamily C member 24 OS=Mus musculus OX=10090 GN=Dnajc24 PE=1 SV=4	0.585
Taco1	Q8K0Z7	Translational activator of cytochrome c oxidase 1 OS=Mus musculus OX=10090 GN=Taco1 PE=1 SV=	0.963333333
Rnaset2b	C0HKG6	Ribonuclease T2-B OS=Mus musculus OX=10090 GN=Rnaset2b PE=1 SV=1	1.113333333
Rnaset2a	C0HKG5	Ribonuclease T2-A OS=Mus musculus OX=10090 GN=Rnaset2a PE=1 SV=1	1.113333333
Ctla2a	P12399	Protein CTLA-2-alpha OS=Mus musculus OX=10090 GN=Ctla2a PE=2 SV=2	1.905
Tspan31	Q9CQ88	Tetraspanin-31 OS=Mus musculus OX=10090 GN=Tspan31 PE=1 SV=1	0.896666667
Nxt1	Q9QZV9	NTF2-related export protein 1 OS=Mus musculus OX=10090 GN=Nxt1 PE=1 SV=2	1.213333333
Ncaph2	Q8BSP2	Condensin-2 complex subunit H2 OS=Mus musculus OX=10090 GN=Ncaph2 PE=1 SV=1	0.855
Mpdz	Q8VBX6	Multiple PDZ domain protein OS=Mus musculus OX=10090 GN=Mpdz PE=1 SV=2	0.516666667
Cdca3	Q99M54	Cell division cycle-associated protein 3 OS=Mus musculus OX=10090 GN=Cdca3 PE=1 SV=2	1.156666667
Asb6	Q91ZU1	Ankyrin repeat and SOCS box protein 6 OS=Mus musculus OX=10090 GN=Asb6 PE=1 SV=1	0.703333333
Paox	Q8C0L6	Peroxisomal N(1)-acetyl-spermine/spermidine oxidase OS=Mus musculus OX=10090 GN=Paox PE=1	1.143333333
Sowahc	Q8C0J6	Ankyrin repeat domain-containing protein SOWAHC OS=Mus musculus OX=10090 GN=Sowahc PE=1	2.203333333
Rsrc1	Q9DBU6	Serine/Arginine-related protein 53 OS=Mus musculus OX=10090 GN=Rsrc1 PE=1 SV=1	0.565
Dguok	Q9QX60	Deoxyguanosine kinase mitochondrial OS=Mus musculus OX=10090 GN=Dguok PE=1 SV=3	1.143333333
Plin2	P43883	Perilipin-2 OS=Mus musculus OX=10090 GN=Plin2 PE=1 SV=2	1.18
Gosr2	O35166	Golgi SNAP receptor complex member 2 OS=Mus musculus OX=10090 GN=Gosr2 PE=1 SV=2	1.083333333
Cbx6	Q9DBY5	Chromobox protein homolog 6 OS=Mus musculus OX=10090 GN=Cbx6 PE=1 SV=2	0.613333333
Selenot	P62342	Thioredoxin reductase-like selenoprotein T OS=Mus musculus OX=10090 GN=Selenot PE=1 SV=2	0.84
Tbc1d23	Q8K0F1	TBC1 domain family member 23 OS=Mus musculus OX=10090 GN=Tbc1d23 PE=1 SV=1	0.665

Gemin2	Q9CQQ4	Gem-associated protein 2 OS=Mus musculus OX=10090 GN=Gemin2 PE=2 SV=1	3.023333333
Gpd1l	Q3ULJ0	Glycerol-3-phosphate dehydrogenase 1-like protein OS=Mus musculus OX=10090 GN=Gpd1l PE=1 S	0.913333333
Chchd5	Q9CQP3	Coiled-coil-helix-coiled-coil-helix domain-containing protein 5 OS=Mus musculus OX=10090 GN=C	0.806666667
Lmbrd2	Q8C561	G-protein coupled receptor-associated protein LMBRD2 OS=Mus musculus OX=10090 GN=Lmbrd2	3.79
Tmem11	Q8BK08	Transmembrane protein 11 mitochondrial OS=Mus musculus OX=10090 GN=Tmem11 PE=1 SV=1	0.756666667
Gng2	P63213	Guanine nucleotide-binding protein G(I)/G(S)/G(O) subunit gamma-2 OS=Mus musculus OX=10090	0.76
Zmat3	O54836	Zinc finger matrin-type protein 3 OS=Mus musculus OX=10090 GN=Zmat3 PE=2 SV=1	0.95
Atp5mf	P56135	ATP synthase subunit f mitochondrial OS=Mus musculus OX=10090 GN=Atp5mf PE=1 SV=3	0.846666667
Sin3b	Q62141	Paired amphipathic helix protein Sin3b OS=Mus musculus OX=10090 GN=Sin3b PE=1 SV=2	1.175
Hif1an	Q8BLR9	Hypoxia-inducible factor 1-alpha inhibitor OS=Mus musculus OX=10090 GN=Hif1an PE=1 SV=2	0.94
E2f4	Q8R0K9	Transcription factor E2F4 OS=Mus musculus OX=10090 GN=E2f4 PE=1 SV=1	0.31
Ecsit	Q9QZH6	Evolutionarily conserved signaling intermediate in Toll pathway mitochondrial OS=Mus musculus	1.22
Ccdc115	Q8VE99	Coiled-coil domain-containing protein 115 OS=Mus musculus OX=10090 GN=Ccdc115 PE=1 SV=1	0.99
Phf21a	Q6ZPK0	PHD finger protein 21A OS=Mus musculus OX=10090 GN=Phf21a PE=1 SV=3	0.963333333
Eral1	Q9CZU4	GTPase Era mitochondrial OS=Mus musculus OX=10090 GN=Eral1 PE=2 SV=1	0.806666667
Yrdc	Q3U5F4	YrdC domain-containing protein mitochondrial OS=Mus musculus OX=10090 GN=Yrdc PE=1 SV=1	0.646666667
Scamp2	Q9ERN0	Secretory carrier-associated membrane protein 2 OS=Mus musculus OX=10090 GN=Scamp2 PE=1 S\	1.563333333
Med11	Q9D8C6	Mediator of RNA polymerase II transcription subunit 11 OS=Mus musculus OX=10090 GN=Med11 P	1.11
Tsr2	Q8C8T8	Pre-rRNA-processing protein TSR2 homolog OS=Mus musculus OX=10090 GN=Tsr2 PE=1 SV=1	0.886666667
Lrrc14	Q8VC16	Leucine-rich repeat-containing protein 14 OS=Mus musculus OX=10090 GN=Lrrc14 PE=1 SV=1	
Usp48	Q3V0C5	Ubiquitin carboxyl-terminal hydrolase 48 OS=Mus musculus OX=10090 GN=Usp48 PE=1 SV=2	0.84
Zbtb7a	O88939	Zinc finger and BTB domain-containing protein 7A OS=Mus musculus OX=10090 GN=Zbtb7a PE=1 S\	0.73
Rbpms2	Q8VC52	RNA-binding protein with multiple splicing 2 OS=Mus musculus OX=10090 GN=Rbpms2 PE=1 SV=1	0.923333333
Rrp36	Q3UFY0	Ribosomal RNA processing protein 36 homolog OS=Mus musculus OX=10090 GN=Rrp36 PE=1 SV=1	1.203333333
Tmem109	Q3UBX0	Transmembrane protein 109 OS=Mus musculus OX=10090 GN=Tmem109 PE=1 SV=2	0.866666667
Rnd3	P61588	Rho-related GTP-binding protein RhoE OS=Mus musculus OX=10090 GN=Rnd3 PE=1 SV=1	1.29
Znf516	Q7TSH3	Zinc finger protein 516 OS=Mus musculus OX=10090 GN=Znf516 PE=1 SV=1	1.125
Tmem59	Q9QY73	Transmembrane protein 59 OS=Mus musculus OX=10090 GN=Tmem59 PE=1 SV=2	0.68
Urm1	Q9D2P4	Ubiquitin-related modifier 1 OS=Mus musculus OX=10090 GN=Urm1 PE=1 SV=1	0.336666667
Wfs1	P56695	Wolframin OS=Mus musculus OX=10090 GN=Wfs1 PE=1 SV=1	1.2
Gfra1	P97785	GDNF family receptor alpha-1 OS=Mus musculus OX=10090 GN=Gfra1 PE=1 SV=2	0.916666667
Reep5	Q60870	Receptor expression-enhancing protein 5 OS=Mus musculus OX=10090 GN=Reep5 PE=1 SV=1	0.943333333
Mex3d	Q3UE17	RNA-binding protein MEX3D OS=Mus musculus OX=10090 GN=Mex3d PE=1 SV=2	0.96
Dtd2	Q8BHA3	D-aminoacyl-tRNA deacylase 2 OS=Mus musculus OX=10090 GN=Dtd2 PE=1 SV=1	1

Cd47	Q61735	Leukocyte surface antigen CD47 OS=Mus musculus OX=10090 GN=Cd47 PE=1 SV=2	0.95
Tollip	Q9QZ06	Toll-interacting protein OS=Mus musculus OX=10090 GN=Tollip PE=1 SV=1	1.013333333
Fibp	Q9JI19	Acidic fibroblast growth factor intracellular-binding protein OS=Mus musculus OX=10090 GN=Fibp	1.196666667
Trappc4	Q9ES56	Trafficking protein particle complex subunit 4 OS=Mus musculus OX=10090 GN=Trappc4 PE=1 SV=1	1.076666667
Alkbh5	Q3TSG4	RNA demethylase ALKBH5 OS=Mus musculus OX=10090 GN=Alkbh5 PE=1 SV=2	1.876666667
Clcn7	O70496	H(+)/Cl(-) exchange transporter 7 OS=Mus musculus OX=10090 GN=Clcn7 PE=1 SV=1	1.073333333
Crat	P47934	Carnitine O-acetyltransferase OS=Mus musculus OX=10090 GN=Crat PE=1 SV=3	1.233333333
Arhgap22	Q8BL80	Rho GTPase-activating protein 22 OS=Mus musculus OX=10090 GN=Arhgap22 PE=1 SV=2	0.615
Mettl26	Q9DCS2	Methyltransferase-like 26 OS=Mus musculus OX=10090 GN=Mettl26 PE=1 SV=1	1.295
Pfn2	Q9JJV2	Profilin-2 OS=Mus musculus OX=10090 GN=Pfn2 PE=1 SV=3	0.9
Ebag9	Q9D0V7	Receptor-binding cancer antigen expressed on SiSo cells OS=Mus musculus OX=10090 GN=Ebag9 PE	0.6
Gstt1	Q64471	Glutathione S-transferase theta-1 OS=Mus musculus OX=10090 GN=Gstt1 PE=1 SV=4	0.875
Gas7	Q60780	Growth arrest-specific protein 7 OS=Mus musculus OX=10090 GN=Gas7 PE=1 SV=1	0.81
Mrpl30	Q9D7N6	39S ribosomal protein L30 mitochondrial OS=Mus musculus OX=10090 GN=Mrpl30 PE=1 SV=1	0.763333333
Coprs	Q9CQ13	Coordinator of PRMT5 and differentiation stimulator OS=Mus musculus OX=10090 GN=Coprs PE=1	0.826666667
Dgat1	Q9Z2A7	Diacylglycerol O-acyltransferase 1 OS=Mus musculus OX=10090 GN=Dgat1 PE=1 SV=1	0.983333333
Med18	Q9CZ82	Mediator of RNA polymerase II transcription subunit 18 OS=Mus musculus OX=10090 GN=Med18 P	0.916666667
Tsr3	Q5HZH2	18S rRNA aminocarboxypropyltransferase OS=Mus musculus OX=10090 GN=Tsr3 PE=1 SV=1	1.726666667
Mrpl41	Q9CQN7	39S ribosomal protein L41 mitochondrial OS=Mus musculus OX=10090 GN=Mrpl41 PE=1 SV=1	0.726666667
Pacs1	Q8K212	Phosphofurin acidic cluster sorting protein 1 OS=Mus musculus OX=10090 GN=Pacs1 PE=1 SV=2	1.81
Camsap2	Q8C1B1	Calmodulin-regulated spectrin-associated protein 2 OS=Mus musculus OX=10090 GN=Camsap2 PE=	1.346666667
Mtatp6	P00848	ATP synthase subunit a OS=Mus musculus OX=10090 GN=Mtatp6 PE=1 SV=1	0.846666667
Col16a1	Q8BLX7	Collagen alpha-1(XVI) chain OS=Mus musculus OX=10090 GN=Col16a1 PE=1 SV=2	0.77
Aasdhppt	Q9CQF6	L-aminoadipate-semialdehyde dehydrogenase-phosphopantetheinyl transferase OS=Mus musculus	0.665
Nfya	P23708	Nuclear transcription factor Y subunit alpha OS=Mus musculus OX=10090 GN=Nfya PE=1 SV=2	0.73
Hmg20a	Q9DC33	High mobility group protein 20A OS=Mus musculus OX=10090 GN=Hmg20a PE=1 SV=1	0.74
Eya3	P97480	Eyes absent homolog 3 OS=Mus musculus OX=10090 GN=Eya3 PE=1 SV=2	0.606666667
Uqcr10	Q8R1I1	Cytochrome b-c1 complex subunit 9 OS=Mus musculus OX=10090 GN=Uqcr10 PE=1 SV=1	0.973333333
Tbc1d31	Q6NXY1	TBC1 domain family member 31 OS=Mus musculus OX=10090 GN=Tbc1d31 PE=1 SV=1	0.81
Rell1	Q8K2J7	RELT-like protein 1 OS=Mus musculus OX=10090 GN=Rell1 PE=2 SV=2	1.09
Mapk7	Q9WVS8	Mitogen-activated protein kinase 7 OS=Mus musculus OX=10090 GN=Mapk7 PE=1 SV=1	4.045
Zdhhc5	Q8VDZ4	Palmitoyltransferase ZDHHC5 OS=Mus musculus OX=10090 GN=Zdhhc5 PE=1 SV=1	0.893333333
Macrod1	Q922B1	ADP-ribose glycohydrolase MACROD1 OS=Mus musculus OX=10090 GN=Macrod1 PE=1 SV=2	0.86
Igf2	P09535	Insulin-like growth factor II OS=Mus musculus OX=10090 GN=Igf2 PE=1 SV=1	0.57

Wdr59	Q8C0M0	GATOR complex protein WDR59 OS=Mus musculus OX=10090 GN=Wdr59 PE=1 SV=2	0.605
Faim	Q9WUD8	Fas apoptotic inhibitory molecule 1 OS=Mus musculus OX=10090 GN=Faim PE=1 SV=1	0.95
Bet1	O35623	BET1 homolog OS=Mus musculus OX=10090 GN=Bet1 PE=1 SV=1	1.046666667
Spc25	Q3UA16	Kinetochore protein Spc25 OS=Mus musculus OX=10090 GN=Spc25 PE=2 SV=1	0.73
Fahd1	Q8R0F8	Acylpyruvase FAHD1 mitochondrial OS=Mus musculus OX=10090 GN=Fahd1 PE=1 SV=2	0.6
Ly6e	Q64253	Lymphocyte antigen 6E OS=Mus musculus OX=10090 GN=Ly6e PE=1 SV=2	1.033333333
Rpl36	P47964	60S ribosomal protein L36 OS=Mus musculus OX=10090 GN=Rpl36 PE=1 SV=2	0.923333333
Dab2ip	Q3UHC7	Disabled homolog 2-interacting protein OS=Mus musculus OX=10090 GN=Dab2ip PE=1 SV=1	0.646666667
Sike1	Q9CPR7	Suppressor of IKBKE 1 OS=Mus musculus OX=10090 GN=Sike1 PE=1 SV=1	0.94
Acyp2	P56375	Acylphosphatase-2 OS=Mus musculus OX=10090 GN=Acyp2 PE=1 SV=2	1.04
Slc1a3	P56564	Excitatory amino acid transporter 1 OS=Mus musculus OX=10090 GN=Slc1a3 PE=1 SV=2	1.015
Znf148	Q61624	Zinc finger protein 148 OS=Mus musculus OX=10090 GN=Znf148 PE=1 SV=2	2.406666667
Mtg1	Q8R2R6	Mitochondrial ribosome-associated GTPase 1 OS=Mus musculus OX=10090 GN=Mtg1 PE=1 SV=2	0.675
Mycbp	Q9EQS3	c-Myc-binding protein OS=Mus musculus OX=10090 GN=Mycbp PE=1 SV=5	0.77
Msln	Q61468	Mesothelin OS=Mus musculus OX=10090 GN=Msln PE=1 SV=1	1.663333333
Pggt1b	Q8BUY9	Geranylgeranyl transferase type-1 subunit beta OS=Mus musculus OX=10090 GN=Pggt1b PE=1 SV=1	0.795
Calcoco1	Q8CGU1	Calcium-binding and coiled-coil domain-containing protein 1 OS=Mus musculus OX=10090 GN=Cal	1.075
Fbxo7	Q3U7U3	F-box only protein 7 OS=Mus musculus OX=10090 GN=Fbxo7 PE=1 SV=2	1.336666667
Cx3cl1	O35188	Fractalkine OS=Mus musculus OX=10090 GN=Cx3cl1 PE=1 SV=3	0.83
Clcn5	Q9WVD4	H(+)/Cl(-) exchange transporter 5 OS=Mus musculus OX=10090 GN=Clcn5 PE=1 SV=2	0.993333333
Vps72	Q62481	Vacuolar protein sorting-associated protein 72 homolog OS=Mus musculus OX=10090 GN=Vps72 P	1.33
Klra4	Q60651	Killer cell lectin-like receptor 4 OS=Mus musculus OX=10090 GN=Klra4 PE=1 SV=2	1.053333333
Aagab	Q8R2R3	Alpha- and gamma-adaptin-binding protein p34 OS=Mus musculus OX=10090 GN=Aagab PE=1 SV=2	1.016666667
Ankrd40	Q5SUE8	Ankyrin repeat domain-containing protein 40 OS=Mus musculus OX=10090 GN=Ankrd40 PE=1 SV=1	0.963333333
Coq3	Q8BMS4	Ubiquinone biosynthesis O-methyltransferase mitochondrial OS=Mus musculus OX=10090 GN=Coc	0.45
Vcpkmt	Q8C436	Protein-lysine methyltransferase METTL21D OS=Mus musculus OX=10090 GN=Vcpkmt PE=2 SV=2	
Prkag2	Q91WG5	5'-AMP-activated protein kinase subunit gamma-2 OS=Mus musculus OX=10090 GN=Prkag2 PE=1 SV	0.703333333
Twnk	Q8CIW5	Twinkle protein mitochondrial OS=Mus musculus OX=10090 GN=Twnk PE=2 SV=1	1.35
Dcaf5	Q80T85	DDB1- and CUL4-associated factor 5 OS=Mus musculus OX=10090 GN=Dcaf5 PE=1 SV=2	
	Q3TQI7	Telomere length and silencing protein 1 homolog OS=Mus musculus OX=10090 PE=1 SV=2	0.59
Mpp5	Q9JLB2	MAGUK p55 subfamily member 5 OS=Mus musculus OX=10090 GN=Mpp5 PE=1 SV=1	0.86
Drosha	Q5HZJ0	Ribonuclease 3 OS=Mus musculus OX=10090 GN=Drosha PE=1 SV=1	1.13
Nat2	P50295	Arylamine N-acetyltransferase 2 OS=Mus musculus OX=10090 GN=Nat2 PE=1 SV=1	3
Psrc1	Q9D0P7	Proline/serine-rich coiled-coil protein 1 OS=Mus musculus OX=10090 GN=Psrc1 PE=1 SV=2	1.316666667

Mrpl33	Q9CQP0	39S ribosomal protein L33 mitochondrial OS=Mus musculus OX=10090 GN=Mrpl33 PE=1 SV=1	0.806666667
Arap1	Q4LDD4	Arf-GAP with Rho-GAP domain ANK repeat and PH domain-containing protein 1 OS=Mus musculus (	1.14
Tbc1d10a	P58802	TBC1 domain family member 10A OS=Mus musculus OX=10090 GN=Tbc1d10a PE=1 SV=1	0.845
Afap1	Q80YS6	Actin filament-associated protein 1 OS=Mus musculus OX=10090 GN=Afap1 PE=1 SV=1	0.81
Tmx2	Q9D710	Thioredoxin-related transmembrane protein 2 OS=Mus musculus OX=10090 GN=Tmx2 PE=1 SV=1	0.9
Cnih4	Q9CX13	Protein cornichon homolog 4 OS=Mus musculus OX=10090 GN=Cnih4 PE=2 SV=1	0.776666667
Nsmce3	Q9CPR8	Non-structural maintenance of chromosomes element 3 homolog OS=Mus musculus OX=10090 GN	1.456666667
Lats1	Q8BYR2	Serine/threonine-protein kinase LATS1 OS=Mus musculus OX=10090 GN=Lats1 PE=1 SV=3	1.095
Terf2	O35144	Telomeric repeat-binding factor 2 OS=Mus musculus OX=10090 GN=Terf2 PE=1 SV=3	0.706666667
Fam234b	Q8BYI8	Protein FAM234B OS=Mus musculus OX=10090 GN=Fam234b PE=1 SV=1	1.033333333
Cox7c	P17665	Cytochrome c oxidase subunit 7C mitochondrial OS=Mus musculus OX=10090 GN=Cox7c PE=1 SV=	0.816666667
Cbx4	O55187	E3 SUMO-protein ligase CBX4 OS=Mus musculus OX=10090 GN=Cbx4 PE=1 SV=2	1.175
Nuf2	Q99P69	Kinetochore protein Nuf2 OS=Mus musculus OX=10090 GN=Nuf2 PE=1 SV=2	1.456666667
Slc30a7	Q9JKN1	Zinc transporter 7 OS=Mus musculus OX=10090 GN=Slc30a7 PE=1 SV=1	0.72
Mitd1	Q8VDV8	MIT domain-containing protein 1 OS=Mus musculus OX=10090 GN=Mitd1 PE=1 SV=1	1.225
Brca1	P48754	Breast cancer type 1 susceptibility protein homolog OS=Mus musculus OX=10090 GN=Brca1 PE=1 S'	1.145
Ccdc9	Q8VC31	Coiled-coil domain-containing protein 9 OS=Mus musculus OX=10090 GN=Ccdc9 PE=1 SV=1	
Pitpnm1	O35954	Membrane-associated phosphatidylinositol transfer protein 1 OS=Mus musculus OX=10090 GN=Pit	1.43
Glt8d1	Q6NSU3	Glycosyltransferase 8 domain-containing protein 1 OS=Mus musculus OX=10090 GN=Glt8d1 PE=1 S	0.606666667
Cdc42ep3	Q9CQC5	Cdc42 effector protein 3 OS=Mus musculus OX=10090 GN=Cdc42ep3 PE=1 SV=1	1.283333333
Fadd	Q61160	FAS-associated death domain protein OS=Mus musculus OX=10090 GN=Fadd PE=1 SV=1	
Wdr81	Q5ND34	WD repeat-containing protein 81 OS=Mus musculus OX=10090 GN=Wdr81 PE=1 SV=2	0.825
Tmem70	Q921N7	Transmembrane protein 70 mitochondrial OS=Mus musculus OX=10090 GN=Tmem70 PE=2 SV=2	6.803333333
Lpgat1	Q91YX5	Acyl-CoA:lysophosphatidylglycerol acyltransferase 1 OS=Mus musculus OX=10090 GN=Lpgat1 PE=1	1.055
Slc25a32	Q8BMG8	Mitochondrial folate transporter/carrier OS=Mus musculus OX=10090 GN=Slc25a32 PE=1 SV=1	1.24
Tprkb	Q8QZZ7	EKC/KEOPS complex subunit Tprkb OS=Mus musculus OX=10090 GN=Tprkb PE=1 SV=1	0.806666667
Steap3	Q8CI59	Metalloreductase STEAP3 OS=Mus musculus OX=10090 GN=Steap3 PE=1 SV=1	2.915
Hook2	Q7TMK6	Protein Hook homolog 2 OS=Mus musculus OX=10090 GN=Hook2 PE=1 SV=3	0.89
Lsm7	Q9CQQ8	U6 snRNA-associated Sm-like protein LSm7 OS=Mus musculus OX=10090 GN=Lsm7 PE=1 SV=1	0.923333333
Cxxc1	Q9CWW7	CXXC-type zinc finger protein 1 OS=Mus musculus OX=10090 GN=Cxxc1 PE=1 SV=1	0.87
Abt1	Q9QYL7	Activator of basal transcription 1 OS=Mus musculus OX=10090 GN=Abt1 PE=2 SV=1	1.356666667
Abcg2	Q7TMS5	Broad substrate specificity ATP-binding cassette transporter ABCG2 OS=Mus musculus OX=10090 GI	0.75
Mrfap1	Q9CQL7	MORF4 family-associated protein 1 OS=Mus musculus OX=10090 GN=Mrfap1 PE=1 SV=1	0.883333333
Sipa1	P46062	Signal-induced proliferation-associated protein 1 OS=Mus musculus OX=10090 GN=Sipa1 PE=1 SV=:	1.016666667

Snip1	Q8BIZ6	Smad nuclear-interacting protein 1 OS=Mus musculus OX=10090 GN=Snip1 PE=1 SV=1	0.526666667
Asb3	Q9WV72	Ankyrin repeat and SOCS box protein 3 OS=Mus musculus OX=10090 GN=Asb3 PE=1 SV=2	0.895
Smg7	Q5RJH6	Protein SMG7 OS=Mus musculus OX=10090 GN=Smg7 PE=2 SV=1	1.725
Plau	P06869	Urokinase-type plasminogen activator OS=Mus musculus OX=10090 GN=Plau PE=1 SV=1	1.435
Elp6	Q8BK75	Elongator complex protein 6 OS=Mus musculus OX=10090 GN=Elp6 PE=2 SV=1	0.963333333
Sash1	P59808	SAM and SH3 domain-containing protein 1 OS=Mus musculus OX=10090 GN=Sash1 PE=1 SV=1	0.925
Hba	P01942	Hemoglobin subunit alpha OS=Mus musculus OX=10090 GN=Hba PE=1 SV=2	0.13
Rbsn	Q80Y56	Rabenosyn-5 OS=Mus musculus OX=10090 GN=Rbsn PE=1 SV=1	1.893333333
Cisd2	Q9CQB5	CDGSH iron-sulfur domain-containing protein 2 OS=Mus musculus OX=10090 GN=Cisd2 PE=1 SV=1	0.69
Polr3c	Q9D483	DNA-directed RNA polymerase III subunit RPC3 OS=Mus musculus OX=10090 GN=Polr3c PE=1 SV=1	1.346666667
Cmc2	Q8K199	COX assembly mitochondrial protein 2 homolog OS=Mus musculus OX=10090 GN=Cmc2 PE=3 SV=1	0.853333333
Rnaseh2b	Q80ZV0	Ribonuclease H2 subunit B OS=Mus musculus OX=10090 GN=Rnaseh2b PE=1 SV=2	0.696666667
Pxk	Q8BX57	PX domain-containing protein kinase-like protein OS=Mus musculus OX=10090 GN=Pxk PE=1 SV=2	0.803333333
Tram2	Q924Z5	Translocating chain-associated membrane protein 2 OS=Mus musculus OX=10090 GN=Tram2 PE=1 S	0.81
Ddx55	Q6ZPL9	ATP-dependent RNA helicase DDX55 OS=Mus musculus OX=10090 GN=Ddx55 PE=1 SV=2	0.536666667
Rp2	Q9EPK2	Protein XRP2 OS=Mus musculus OX=10090 GN=Rp2 PE=1 SV=3	1.025
Gorasp1	Q91X51	Golgi reassembly-stacking protein 1 OS=Mus musculus OX=10090 GN=Gorasp1 PE=1 SV=3	7.68
Oxr1	Q4KMM3	Oxidation resistance protein 1 OS=Mus musculus OX=10090 GN=Oxr1 PE=1 SV=3	2.885
Itm2c	Q91VK4	Integral membrane protein 2C OS=Mus musculus OX=10090 GN=Itm2c PE=1 SV=2	0.473333333
Gcc1	Q9D4H2	GRIP and coiled-coil domain-containing protein 1 OS=Mus musculus OX=10090 GN=Gcc1 PE=1 SV=:	
Asrgl1	Q8C0M9	Isoaspartyl peptidase/L-asparaginase OS=Mus musculus OX=10090 GN=Asrgl1 PE=1 SV=1	1.06
Rbmx2	Q8R0F5	RNA-binding motif protein X-linked 2 OS=Mus musculus OX=10090 GN=Rbmx2 PE=1 SV=1	1.393333333
Ctdsp1	P58466	Carboxy-terminal domain RNA polymerase II polypeptide A small phosphatase 1 OS=Mus musculus (	1.056666667
Polr2l	P62876	DNA-directed RNA polymerases I II and III subunit RPABC5 OS=Mus musculus OX=10090 GN=Polr2l	0.83
Ift122	Q6NWV3	Intraflagellar transport protein 122 homolog OS=Mus musculus OX=10090 GN=Ift122 PE=1 SV=1	0.565
Slc35f6	Q8VE96	Solute carrier family 35 member F6 OS=Mus musculus OX=10090 GN=Slc35f6 PE=1 SV=1	1.15
Max	P28574	Protein max OS=Mus musculus OX=10090 GN=Max PE=1 SV=2	1.326666667
D8Ertd738e	Q8R1F0	Leydig cell tumor 10 kDa protein homolog OS=Mus musculus OX=10090 GN=D8Ertd738e PE=3 SV=1	1.446666667
Arhgap21	Q6DFV3	Rho GTPase-activating protein 21 OS=Mus musculus OX=10090 GN=Arhgap21 PE=1 SV=1	
Znf281	Q99LI5	Zinc finger protein 281 OS=Mus musculus OX=10090 GN=Znf281 PE=1 SV=1	1.35
Oxnad1	Q8VE38	Oxidoreductase NAD-binding domain-containing protein 1 OS=Mus musculus OX=10090 GN=Oxnac	1.663333333
Bcl2l1	Q64373	Bcl-2-like protein 1 OS=Mus musculus OX=10090 GN=Bcl2l1 PE=1 SV=1	0.88
Nectin2	P32507	Nectin-2 OS=Mus musculus OX=10090 GN=Nectin2 PE=1 SV=2	0.86
Abhd17b	Q7M759	Alpha/beta hydrolase domain-containing protein 17B OS=Mus musculus OX=10090 GN=Abhd17b P	1.3

Slc35b1	P97858	Solute carrier family 35 member B1 OS=Mus musculus OX=10090 GN=Slc35b1 PE=1 SV=1	1.843333333
Arrb2	Q91YI4	Beta-arrestin-2 OS=Mus musculus OX=10090 GN=Arrb2 PE=1 SV=1	1.036666667
Ift81	O35594	Intraflagellar transport protein 81 homolog OS=Mus musculus OX=10090 GN=Ift81 PE=1 SV=4	
B4gat1	Q8BWP8	Beta-1 4-glucuronyltransferase 1 OS=Mus musculus OX=10090 GN=B4gat1 PE=1 SV=1	2.36
Tst	P52196	Thiosulfate sulfurtransferase OS=Mus musculus OX=10090 GN=Tst PE=1 SV=3	1.116666667
Rsl24d1	Q99L28	Probable ribosome biogenesis protein RLP24 OS=Mus musculus OX=10090 GN=Rsl24d1 PE=2 SV=1	0.846666667
Tgs1	Q923W1	Trimethylguanosine synthase OS=Mus musculus OX=10090 GN=Tgs1 PE=1 SV=2	0.93
Lrp6	O88572	Low-density lipoprotein receptor-related protein 6 OS=Mus musculus OX=10090 GN=Lrp6 PE=1 SV=	0.77
Gpkow	Q56A08	G-patch domain and KOW motifs-containing protein OS=Mus musculus OX=10090 GN=Gpkow PE=1	
Slc30a6	Q8BJM5	Zinc transporter 6 OS=Mus musculus OX=10090 GN=Slc30a6 PE=1 SV=1	
Mpdu1	Q9R0Q9	Mannose-P-dolichol utilization defect 1 protein OS=Mus musculus OX=10090 GN=Mpdu1 PE=1 SV=	0.73
Plekha2	Q9ERS5	Pleckstrin homology domain-containing family A member 2 OS=Mus musculus OX=10090 GN=Plekh	0.97
Arpp19	P56212	cAMP-regulated phosphoprotein 19 OS=Mus musculus OX=10090 GN=Arpp19 PE=1 SV=2	0.85
Pi4kb	Q8BKC8	Phosphatidylinositol 4-kinase beta OS=Mus musculus OX=10090 GN=Pi4kb PE=1 SV=2	0.67
Chst14	Q80V53	Carbohydrate sulfotransferase 14 OS=Mus musculus OX=10090 GN=Chst14 PE=2 SV=2	1.69
Sirt2	Q8VDQ8	NAD-dependent protein deacetylase sirtuin-2 OS=Mus musculus OX=10090 GN=Sirt2 PE=1 SV=2	
Ralgapb	Q8BQZ4	Ral GTPase-activating protein subunit beta OS=Mus musculus OX=10090 GN=Ralgapb PE=1 SV=2	0.86
Ubxn6	Q99PL6	UBX domain-containing protein 6 OS=Mus musculus OX=10090 GN=Ubxn6 PE=1 SV=1	1.47
Ctu1	Q99J10	Cytoplasmic tRNA 2-thiolation protein 1 OS=Mus musculus OX=10090 GN=Ctu1 PE=2 SV=1	1.65
Mcam	Q8R2Y2	Cell surface glycoprotein MUC18 OS=Mus musculus OX=10090 GN=Mcam PE=1 SV=1	0.695
Irgq	Q8VIM9	Immunity-related GTPase family Q protein OS=Mus musculus OX=10090 GN=Irgq PE=1 SV=1	48.74
Bphl	Q8R164	Valacyclovir hydrolase OS=Mus musculus OX=10090 GN=Bphl PE=1 SV=1	1.055
Mmab	Q9D273	Corrinoid adenosyltransferase OS=Mus musculus OX=10090 GN=Mmab PE=1 SV=1	1.25
Tab2	Q99K90	TGF-beta-activated kinase 1 and MAP3K7-binding protein 2 OS=Mus musculus OX=10090 GN=Tab2	0.733333333
Maz	P56671	Myc-associated zinc finger protein OS=Mus musculus OX=10090 GN=Maz PE=1 SV=1	0.98
Mrps21	P58059	28S ribosomal protein S21 mitochondrial OS=Mus musculus OX=10090 GN=Mrps21 PE=1 SV=1	1.423333333
Znf703	P0CL69	Zinc finger protein 703 OS=Mus musculus OX=10090 GN=Znf703 PE=1 SV=1	0.333333333
Med6	Q921D4	Mediator of RNA polymerase II transcription subunit 6 OS=Mus musculus OX=10090 GN=Med6 PE=1	1.05
Egln1	Q91YE3	Egl nine homolog 1 OS=Mus musculus OX=10090 GN=Egln1 PE=1 SV=2	1.426666667
Ccdc167	Q9D162	Coiled-coil domain-containing protein 167 OS=Mus musculus OX=10090 GN=Ccdc167 PE=1 SV=2	0.526666667
Tdrd7	Q8K1H1	Tudor domain-containing protein 7 OS=Mus musculus OX=10090 GN=Tdrd7 PE=1 SV=1	2.385
Itih4	A6X935	Inter alpha-trypsin inhibitor heavy chain 4 OS=Mus musculus OX=10090 GN=Itih4 PE=1 SV=2	0
Cdkal1	Q91WE6	Threonylcarbamoyladenosine tRNA methylthiotransferase OS=Mus musculus OX=10090 GN=Cdkal1	0.65
Ccdc12	Q8R344	Coiled-coil domain-containing protein 12 OS=Mus musculus OX=10090 GN=Ccdc12 PE=1 SV=2	0.69

Npr2	Q6VVW5	Atrial natriuretic peptide receptor 2 OS=Mus musculus OX=10090 GN=Npr2 PE=1 SV=2	1.633333333
Wipi1	Q8R3E3	WD repeat domain phosphoinositide-interacting protein 1 OS=Mus musculus OX=10090 GN=Wipi1	0.833333333
Haus1	Q8BHX1	HAUS augmin-like complex subunit 1 OS=Mus musculus OX=10090 GN=Haus1 PE=1 SV=2	
Mpc1	P63030	Mitochondrial pyruvate carrier 1 OS=Mus musculus OX=10090 GN=Mpc1 PE=1 SV=1	1.245
Kxd1	Q80XH1	KxDL motif-containing protein 1 OS=Mus musculus OX=10090 GN=Kxd1 PE=1 SV=1	2.033333333
Ndufb3	Q9CQZ6	NADH dehydrogenase [ubiquinone] 1 beta subcomplex subunit 3 OS=Mus musculus OX=10090 GN=	0.793333333
Mphosph6	Q9D1Q1	M-phase phosphoprotein 6 OS=Mus musculus OX=10090 GN=Mphosph6 PE=1 SV=1	0.953333333
Hs6st2	Q80UW0	Heparan-sulfate 6-O-sulfotransferase 2 OS=Mus musculus OX=10090 GN=Hs6st2 PE=2 SV=3	1.225
Pgpep1	Q9ESW8	Pyroglutamyl-peptidase 1 OS=Mus musculus OX=10090 GN=Pgpep1 PE=1 SV=1	1.073333333
Tmem259	Q8CIV2	Membralin OS=Mus musculus OX=10090 GN=Tmem259 PE=1 SV=2	0.633333333
Thbd	P15306	Thrombomodulin OS=Mus musculus OX=10090 GN=Thbd PE=1 SV=1	0.98
Uimc1	Q5U5Q9	BRCA1-A complex subunit RAP80 OS=Mus musculus OX=10090 GN=Uimc1 PE=1 SV=2	1.88
Usp30	Q3UN04	Ubiquitin carboxyl-terminal hydrolase 30 OS=Mus musculus OX=10090 GN=Usp30 PE=1 SV=1	0.645
Phf2	Q9WTU0	Lysine-specific demethylase PHF2 OS=Mus musculus OX=10090 GN=Phf2 PE=1 SV=2	1.02
Creg1	O88668	Protein CREG1 OS=Mus musculus OX=10090 GN=Creg1 PE=1 SV=1	0.663333333
Tmem167a	Q9CR64	Protein kish-A OS=Mus musculus OX=10090 GN=Tmem167a PE=1 SV=1	0.91
Stambpl1	Q76N33	AMSH-like protease OS=Mus musculus OX=10090 GN=Stambpl1 PE=1 SV=1	0.905
Csf1	P07141	Macrophage colony-stimulating factor 1 OS=Mus musculus OX=10090 GN=Csf1 PE=1 SV=2	2.093333333
Vhl	P40338	von Hippel-Lindau disease tumor suppressor OS=Mus musculus OX=10090 GN=Vhl PE=1 SV=1	2.346666667
Timp2	P25785	Metalloproteinase inhibitor 2 OS=Mus musculus OX=10090 GN=Timp2 PE=1 SV=2	0.606666667
Sars2	Q9JJL8	Serine--tRNA ligase mitochondrial OS=Mus musculus OX=10090 GN=Sars2 PE=1 SV=2	
L3mbtl3	Q8BLB7	Lethal(3)malignant brain tumor-like protein 3 OS=Mus musculus OX=10090 GN=L3mbtl3 PE=1 SV=1	0.525
Ppp1r12c	Q3UMT1	Protein phosphatase 1 regulatory subunit 12C OS=Mus musculus OX=10090 GN=Ppp1r12c PE=1 SV=	
Mef2c	Q8CFN5	Myocyte-specific enhancer factor 2C OS=Mus musculus OX=10090 GN=Mef2c PE=1 SV=2	9.64
Mef2d	Q63943	Myocyte-specific enhancer factor 2D OS=Mus musculus OX=10090 GN=Mef2d PE=1 SV=2	9.64
Mef2a	Q60929	Myocyte-specific enhancer factor 2A OS=Mus musculus OX=10090 GN=Mef2a PE=1 SV=2	9.64
Ifi30	Q9ESY9	Gamma-interferon-inducible lysosomal thiol reductase OS=Mus musculus OX=10090 GN=Ifi30 PE=1	0.96
Rhobtb3	Q9CTN4	Rho-related BTB domain-containing protein 3 OS=Mus musculus OX=10090 GN=Rhobtb3 PE=2 SV=3	1.263333333
Tmem126a	Q9D8Y1	Transmembrane protein 126A OS=Mus musculus OX=10090 GN=Tmem126a PE=1 SV=1	1.103333333
Sgpp1	Q9JI99	Sphingosine-1-phosphate phosphatase 1 OS=Mus musculus OX=10090 GN=Sgpp1 PE=1 SV=1	0.973333333
Rufy2	Q8R4C2	RUN and FYVE domain-containing protein 2 OS=Mus musculus OX=10090 GN=Rufy2 PE=1 SV=2	1.403333333
Ptgfrn	Q9WV91	Prostaglandin F2 receptor negative regulator OS=Mus musculus OX=10090 GN=Ptgfrn PE=1 SV=2	
Fam168b	Q80XQ8	Myelin-associated neurite-outgrowth inhibitor OS=Mus musculus OX=10090 GN=Fam168b PE=1 SV	0.55
Crim1	Q9JLL0	Cysteine-rich motor neuron 1 protein OS=Mus musculus OX=10090 GN=Crim1 PE=2 SV=2	0.9

Timmdc1	Q8BUY5	Complex I assembly factor TIMMDC1 mitochondrial OS=Mus musculus OX=10090 GN=Timmdc1 PE	1.096666667
Orc5	Q9WUV0	Origin recognition complex subunit 5 OS=Mus musculus OX=10090 GN=Orc5 PE=2 SV=1	1.495
Hikeshi	Q9DD02	Protein Hikeshi OS=Mus musculus OX=10090 GN=Hikeshi PE=1 SV=1	0.953333333
Coq6	Q8R1S0	Ubiquinone biosynthesis monooxygenase COQ6 mitochondrial OS=Mus musculus OX=10090 GN=C	0.615
Uqcrq	Q9CQ69	Cytochrome b-c1 complex subunit 8 OS=Mus musculus OX=10090 GN=Uqcrq PE=1 SV=3	0.466666667
Tpgs1	Q99MS8	Tubulin polyglutamylase complex subunit 1 OS=Mus musculus OX=10090 GN=Tpgs1 PE=1 SV=1	1.08
Rrp15	Q9CYX7	RRP15-like protein OS=Mus musculus OX=10090 GN=Rrp15 PE=1 SV=2	0.89
Rpp25l	Q99JH1	Ribonuclease P protein subunit p25-like protein OS=Mus musculus OX=10090 GN=Rpp25l PE=1 SV=	1.16
Chmp2b	Q8BJF9	Charged multivesicular body protein 2b OS=Mus musculus OX=10090 GN=Chmp2b PE=1 SV=1	1
Gosr1	O88630	Golgi SNAP receptor complex member 1 OS=Mus musculus OX=10090 GN=Gosr1 PE=1 SV=2	0.716666667
Pole2	O54956	DNA polymerase epsilon subunit 2 OS=Mus musculus OX=10090 GN=Pole2 PE=2 SV=2	0.485
	Q8VE95	UPF0598 protein C8orf82 homolog OS=Mus musculus OX=10090 PE=1 SV=1	
Pisd	Q8BSF4	Phosphatidylserine decarboxylase proenzyme mitochondrial OS=Mus musculus OX=10090 GN=Pis	4.005
Dctn5	Q9QZB9	Dynactin subunit 5 OS=Mus musculus OX=10090 GN=Dctn5 PE=1 SV=1	0.916666667
Kyat3	Q71RI9	Kynurenine--oxoglutarate transaminase 3 OS=Mus musculus OX=10090 GN=Kyat3 PE=1 SV=1	0.736666667
Bloc1s1	O55102	Biogenesis of lysosome-related organelles complex 1 subunit 1 OS=Mus musculus OX=10090 GN=Bl	0.813333333
Mettl21A	Q9CQL0	Protein N-lysine methyltransferase METTL21A OS=Mus musculus OX=10090 GN=Mettl21A PE=2 SV=	0.7
Zbed3	Q9D0L1	Zinc finger BED domain-containing protein 3 OS=Mus musculus OX=10090 GN=Zbed3 PE=1 SV=1	8.436666667
Usp45	Q8K387	Ubiquitin carboxyl-terminal hydrolase 45 OS=Mus musculus OX=10090 GN=Usp45 PE=1 SV=1	1.086666667
Grpel2	O88396	GrpE protein homolog 2 mitochondrial OS=Mus musculus OX=10090 GN=Grpel2 PE=2 SV=2	1.053333333
Tmbim6	Q9D2C7	Bax inhibitor 1 OS=Mus musculus OX=10090 GN=Tmbim6 PE=1 SV=1	25.42666667
Bcl10	Q9Z0H7	B-cell lymphoma/leukemia 10 OS=Mus musculus OX=10090 GN=Bcl10 PE=1 SV=1	0.956666667
Clp1	Q99LI9	Polyribonucleotide 5'-hydroxyl-kinase Clp1 OS=Mus musculus OX=10090 GN=Clp1 PE=1 SV=1	2.025
Sec11c	Q9D8V7	Signal peptidase complex catalytic subunit SEC11C OS=Mus musculus OX=10090 GN=Sec11c PE=1 S	0.826666667
Ndufs5	Q99LY9	NADH dehydrogenase [ubiquinone] iron-sulfur protein 5 OS=Mus musculus OX=10090 GN=Ndufs5 P	0.97
Mtnd4	P03911	NADH-ubiquinone oxidoreductase chain 4 OS=Mus musculus OX=10090 GN=Mtnd4 PE=1 SV=1	0.7
Sap30	O88574	Histone deacetylase complex subunit SAP30 OS=Mus musculus OX=10090 GN=Sap30 PE=1 SV=1	0.735
Mgat2	Q921V5	Alpha-1 6-mannosyl-glycoprotein 2-beta-N-acetylglucosaminyltransferase OS=Mus musculus OX=1C	9.603333333
Tti2	Q8BGV4	TELO2-interacting protein 2 OS=Mus musculus OX=10090 GN=Tti2 PE=2 SV=1	1.67
Slc6a8	Q8VBW1	Sodium- and chloride-dependent creatine transporter 1 OS=Mus musculus OX=10090 GN=Slc6a8 PI	2.07
Ptpn13	Q64512	Tyrosine-protein phosphatase non-receptor type 13 OS=Mus musculus OX=10090 GN=Ptpn13 PE=1	
Ell	O08856	RNA polymerase II elongation factor ELL OS=Mus musculus OX=10090 GN=Ell PE=2 SV=2	0.32
Fdx1	P46656	Adrenodoxin mitochondrial OS=Mus musculus OX=10090 GN=Fdx1 PE=1 SV=1	0.876666667
Lman2l	P59481	VIP36-like protein OS=Mus musculus OX=10090 GN=Lman2l PE=1 SV=1	0.293333333

Plpp3	Q99JY8	Phospholipid phosphatase 3 OS=Mus musculus OX=10090 GN=Plpp3 PE=1 SV=1	1.1
Nudt16l1	Q8VHN8	Tudor-interacting repair regulator protein OS=Mus musculus OX=10090 GN=Nudt16l1 PE=1 SV=2	0.58
Cyth2	P63034	Cytohesin-2 OS=Mus musculus OX=10090 GN=Cyth2 PE=1 SV=2	0.485
Mtr	A6H5Y3	Methionine synthase OS=Mus musculus OX=10090 GN=Mtr PE=1 SV=1	0.75
Desi1	Q9CQT7	Desumoylating isopeptidase 1 OS=Mus musculus OX=10090 GN=Desi1 PE=1 SV=1	1.02
Sde2	Q8K1J5	Replication stress response regulator SDE2 OS=Mus musculus OX=10090 GN=Sde2 PE=1 SV=1	
Med20	Q9R0X0	Mediator of RNA polymerase II transcription subunit 20 OS=Mus musculus OX=10090 GN=Med20 PE	1.456666667
Kdm3a	Q6PCM1	Lysine-specific demethylase 3A OS=Mus musculus OX=10090 GN=Kdm3a PE=1 SV=1	1.895
Anapc16	Q9CPV2	Anaphase-promoting complex subunit 16 OS=Mus musculus OX=10090 GN=Anapc16 PE=3 SV=1	1.183333333
Vps41	Q5KU39	Vacuolar protein sorting-associated protein 41 homolog OS=Mus musculus OX=10090 GN=Vps41 PE	0.925
Ankrd27	Q3UMR0	Ankyrin repeat domain-containing protein 27 OS=Mus musculus OX=10090 GN=Ankrd27 PE=1 SV=2	0.37
Alg11	Q3TZM9	GDP-Man:Man(3)GlcNAc(2)-PP-Dol alpha-1 2-mannosyltransferase OS=Mus musculus OX=10090 GN	0.8
Mkrn2	Q9ERV1	Probable E3 ubiquitin-protein ligase makorin-2 OS=Mus musculus OX=10090 GN=Mkrn2 PE=1 SV=2	7.795
Aif1l	Q9EQX4	Allograft inflammatory factor 1-like OS=Mus musculus OX=10090 GN=Aif1l PE=1 SV=1	1.703333333
	Q8WUR0	Protein C19orf12 homolog OS=Mus musculus OX=10090 PE=1 SV=1	0.625
Bcl9l	Q67FY2	B-cell CLL/lymphoma 9-like protein OS=Mus musculus OX=10090 GN=Bcl9l PE=1 SV=1	0.765
Hmces	Q8R1M0	Abasic site processing protein HMCES OS=Mus musculus OX=10090 GN=Hmces PE=1 SV=1	1.07
Retsat	Q64FW2	All-trans-retinol 13 14-reductase OS=Mus musculus OX=10090 GN=Retsat PE=1 SV=3	0.896666667
Rpl39	P62892	60S ribosomal protein L39 OS=Mus musculus OX=10090 GN=Rpl39 PE=1 SV=2	0.87
Ralbp1	Q62172	RalA-binding protein 1 OS=Mus musculus OX=10090 GN=Ralbp1 PE=1 SV=4	4.395
Mrgbp	Q9DAT2	MRG/MORF4L-binding protein OS=Mus musculus OX=10090 GN=Mrgbp PE=1 SV=2	1.236666667
Gps2	Q921N8	G protein pathway suppressor 2 OS=Mus musculus OX=10090 GN=Gps2 PE=1 SV=1	1.02
Rdh14	Q9ERI6	Retinol dehydrogenase 14 OS=Mus musculus OX=10090 GN=Rdh14 PE=1 SV=1	0.583333333
Tor1a	Q9ER39	Torsin-1A OS=Mus musculus OX=10090 GN=Tor1a PE=1 SV=1	1.046666667
Avl9	Q80U56	Late secretory pathway protein AVL9 homolog OS=Mus musculus OX=10090 GN=Avl9 PE=1 SV=2	1.43
	Q9D9H8	UPF0565 protein C2orf69 homolog OS=Mus musculus OX=10090 PE=2 SV=3	1.84
Stx16	Q8BVI5	Syntaxin-16 OS=Mus musculus OX=10090 GN=Stx16 PE=1 SV=3	
Serpinf1	P97298	Pigment epithelium-derived factor OS=Mus musculus OX=10090 GN=Serpinf1 PE=1 SV=2	0.76
Ppcs	Q8VDG5	Phosphopantothenate--cysteine ligase OS=Mus musculus OX=10090 GN=Ppcs PE=1 SV=1	0.45
Taf8	Q9EQH4	Transcription initiation factor TFIID subunit 8 OS=Mus musculus OX=10090 GN=Taf8 PE=2 SV=1	1.31
Med13l	Q6JPI3	Mediator of RNA polymerase II transcription subunit 13-like OS=Mus musculus OX=10090 GN=Med1	0.495
Sirpa	P97797	Tyrosine-protein phosphatase non-receptor type substrate 1 OS=Mus musculus OX=10090 GN=Sirpa	6.21
Ift140	E9PY46	Intraflagellar transport protein 140 homolog OS=Mus musculus OX=10090 GN=Ift140 PE=1 SV=1	0.56
Fastkd5	Q7TMV3	FAST kinase domain-containing protein 5 mitochondrial OS=Mus musculus OX=10090 GN=Fastkd5	0.975

Tbc1d2b	Q3U0J8	TBC1 domain family member 2B OS=Mus musculus OX=10090 GN=Tbc1d2b PE=1 SV=2	0.855
Ndufb6	Q3UIU2	NADH dehydrogenase [ubiquinone] 1 beta subcomplex subunit 6 OS=Mus musculus OX=10090 GN=	0.75
Znf800	Q0VEE6	Zinc finger protein 800 OS=Mus musculus OX=10090 GN=Znf800 PE=1 SV=1	0.73
Mcub	Q810S1	Calcium uniporter regulatory subunit MCUb mitochondrial OS=Mus musculus OX=10090 GN=Mcul	0.665
Agpat3	Q9D517	1-acyl-sn-glycerol-3-phosphate acyltransferase gamma OS=Mus musculus OX=10090 GN=Agpat3 PE=	1.05
Phlda3	Q9WV95	Pleckstrin homology-like domain family A member 3 OS=Mus musculus OX=10090 GN=Phlda3 PE=1	1.325
Med30	Q9CQI9	Mediator of RNA polymerase II transcription subunit 30 OS=Mus musculus OX=10090 GN=Med30 PI	1.156666667
Elp5	Q99L85	Elongator complex protein 5 OS=Mus musculus OX=10090 GN=Elp5 PE=2 SV=1	1.136666667
Xkr5	Q5GH66	XK-related protein 5 OS=Mus musculus OX=10090 GN=Xkr5 PE=2 SV=1	0.48
Usp46	P62069	Ubiquitin carboxyl-terminal hydrolase 46 OS=Mus musculus OX=10090 GN=Usp46 PE=1 SV=1	0.87
Mea1	Q64327	Male-enhanced antigen 1 OS=Mus musculus OX=10090 GN=Mea1 PE=1 SV=1	1.44
Aven	Q9D9K3	Cell death regulator Aven OS=Mus musculus OX=10090 GN=Aven PE=1 SV=2	1.805
Arglu1	Q3UL36	Arginine and glutamate-rich protein 1 OS=Mus musculus OX=10090 GN=Arglu1 PE=1 SV=2	0.885
Ei24	Q61070	Etoposide-induced protein 2.4 OS=Mus musculus OX=10090 GN=Ei24 PE=1 SV=3	1.225
Hps5	P59438	Hermansky-Pudlak syndrome 5 protein homolog OS=Mus musculus OX=10090 GN=Hps5 PE=1 SV=2	
Tmem132a	Q922P8	Transmembrane protein 132A OS=Mus musculus OX=10090 GN=Tmem132a PE=1 SV=2	1.576666667
Acy3	Q91XE4	N-acyl-aromatic-L-amino acid amidohydrolase (carboxylate-forming) OS=Mus musculus OX=10090 (	0.993333333
Pot1	Q91WC1	Protection of telomeres protein 1 OS=Mus musculus OX=10090 GN=Pot1 PE=1 SV=1	0.84
Syf2	Q9D198	Pre-mRNA-splicing factor SYF2 OS=Mus musculus OX=10090 GN=Syf2 PE=2 SV=1	1.996666667
Sccpdh	Q8R127	Saccharopine dehydrogenase-like oxidoreductase OS=Mus musculus OX=10090 GN=Sccpdh PE=1 SV	
Crlf1	Q9JM58	Cytokine receptor-like factor 1 OS=Mus musculus OX=10090 GN=Crlf1 PE=1 SV=1	2.333333333
Nfe2l2	Q60795	Nuclear factor erythroid 2-related factor 2 OS=Mus musculus OX=10090 GN=Nfe2l2 PE=1 SV=2	0.066666667
Scrn1	Q9CZC8	Secernin-1 OS=Mus musculus OX=10090 GN=Scrn1 PE=1 SV=1	0.766666667
Comtd1	Q8BIG7	Catechol O-methyltransferase domain-containing protein 1 OS=Mus musculus OX=10090 GN=Comt	1.45
Pdxp	P60487	Pyridoxal phosphate phosphatase OS=Mus musculus OX=10090 GN=Pdxp PE=1 SV=1	0.845
Kat8	Q9D1P2	Histone acetyltransferase KAT8 OS=Mus musculus OX=10090 GN=Kat8 PE=1 SV=1	
Lrrc8d	Q8BGR2	Volume-regulated anion channel subunit LRRC8D OS=Mus musculus OX=10090 GN=Lrrc8d PE=1 SV=	0.655
Fam133b	Q9CVI2	Protein FAM133B OS=Mus musculus OX=10090 GN=Fam133b PE=1 SV=3	0.46
Gorab	Q8BRM2	RAB6-interacting golgin OS=Mus musculus OX=10090 GN=Gorab PE=1 SV=1	1.135
Pax7	P47239	Paired box protein Pax-7 OS=Mus musculus OX=10090 GN=Pax7 PE=1 SV=2	4.106666667
Cep89	Q9CZX2	Centrosomal protein of 89 kDa OS=Mus musculus OX=10090 GN=Cep89 PE=1 SV=1	
Atxn1	P54254	Ataxin-1 OS=Mus musculus OX=10090 GN=Atxn1 PE=1 SV=2	1.286666667
Veph1	A1A535	Ventricular zone-expressed PH domain-containing protein 1 OS=Mus musculus OX=10090 GN=Veph	128.975
Eif1a	Q60872	Eukaryotic translation initiation factor 1A OS=Mus musculus OX=10090 GN=Eif1a PE=2 SV=3	0.923333333

Nfib	P97863	Nuclear factor 1 B-type OS=Mus musculus OX=10090 GN=Nfib PE=1 SV=2	
Ptp4a2	O70274	Protein tyrosine phosphatase type IVA 2 OS=Mus musculus OX=10090 GN=Ptp4a2 PE=1 SV=1	
Dock6	Q8VDR9	Dedicator of cytokinesis protein 6 OS=Mus musculus OX=10090 GN=Dock6 PE=1 SV=4	
Tfcp2	Q9ERA0	Alpha-globin transcription factor CP2 OS=Mus musculus OX=10090 GN=Tfcp2 PE=1 SV=1	
Sbno2	Q7TNB8	Protein strawberry notch homolog 2 OS=Mus musculus OX=10090 GN=Sbno2 PE=1 SV=1	
Zfx	P17012	Zinc finger X-chromosomal protein OS=Mus musculus OX=10090 GN=Zfx PE=1 SV=2	
Krt1	P04104	Keratin type II cytoskeletal 1 OS=Mus musculus OX=10090 GN=Krt1 PE=1 SV=4	1.373333333
Pank1	Q8K4K6	Pantothenate kinase 1 OS=Mus musculus OX=10090 GN=Pank1 PE=1 SV=1	0.94
Ptp4a1	Q63739	Protein tyrosine phosphatase type IVA 1 OS=Mus musculus OX=10090 GN=Ptp4a1 PE=1 SV=1	2.26
Prkcg	P63318	Protein kinase C gamma type OS=Mus musculus OX=10090 GN=Prkcg PE=1 SV=1	11.675
Mink1	Q9JM52	Misshapen-like kinase 1 OS=Mus musculus OX=10090 GN=Mink1 PE=1 SV=3	
Osbp2	Q5QNQ6	Oxysterol-binding protein 2 OS=Mus musculus OX=10090 GN=Osbp2 PE=1 SV=1	21.41
Wnk3	Q80XP9	Serine/threonine-protein kinase WNK3 OS=Mus musculus OX=10090 GN=Wnk3 PE=1 SV=3	0.195
Zfp36l1	P23950	mRNA decay activator protein ZFP36L1 OS=Mus musculus OX=10090 GN=Zfp36l1 PE=1 SV=1	0.63
Ank2	Q8C8R3	Ankyrin-2 OS=Mus musculus OX=10090 GN=Ank2 PE=1 SV=2	
Chd6	A3KFM7	Chromodomain-helicase-DNA-binding protein 6 OS=Mus musculus OX=10090 GN=Chd6 PE=1 SV=1	0.845
Utp14b	Q6EJB6	U3 small nucleolar RNA-associated protein 14 homolog B OS=Mus musculus OX=10090 GN=Utp14b	
Mapk8ip3	Q9ESN9	C-Jun-amino-terminal kinase-interacting protein 3 OS=Mus musculus OX=10090 GN=Mapk8ip3 PE=	
Zfp2	P08043	Zinc finger protein 2 OS=Mus musculus OX=10090 GN=Zfp2 PE=2 SV=2	0.916666667
Rab13	Q9DD03	Ras-related protein Rab-13 OS=Mus musculus OX=10090 GN=Rab13 PE=1 SV=1	6.28
Epha3	P29319	Ephrin type-A receptor 3 OS=Mus musculus OX=10090 GN=Epha3 PE=1 SV=1	
Papolg	Q6PCL9	Poly(A) polymerase gamma OS=Mus musculus OX=10090 GN=Papolg PE=1 SV=1	2.17
Chd7	A2AJK6	Chromodomain-helicase-DNA-binding protein 7 OS=Mus musculus OX=10090 GN=Chd7 PE=1 SV=1	
Clgn	P52194	Calmegin OS=Mus musculus OX=10090 GN=Clgn PE=1 SV=2	0.97
Cul9	Q80TT8	Cullin-9 OS=Mus musculus OX=10090 GN=Cul9 PE=1 SV=2	1.08
Capns2	Q9D7J7	Calpain small subunit 2 OS=Mus musculus OX=10090 GN=Capns2 PE=2 SV=1	
Spag17	Q5S003	Sperm-associated antigen 17 OS=Mus musculus OX=10090 GN=Spag17 PE=1 SV=2	0.003333333
Stk10	O55098	Serine/threonine-protein kinase 10 OS=Mus musculus OX=10090 GN=Stk10 PE=1 SV=2	
Pde4d	Q01063	cAMP-specific 3' 5'-cyclic phosphodiesterase 4D OS=Mus musculus OX=10090 GN=Pde4d PE=1 SV=2	0.57
Syne1	Q6ZWR6	Nesprin-1 OS=Mus musculus OX=10090 GN=Syne1 PE=1 SV=2	3.483333333
Txnrd2	Q9JLT4	Thioredoxin reductase 2 mitochondrial OS=Mus musculus OX=10090 GN=Txnrd2 PE=1 SV=4	0.463333333
Ube2d3	P61079	Ubiquitin-conjugating enzyme E2 D3 OS=Mus musculus OX=10090 GN=Ube2d3 PE=1 SV=1	1.25
Ube2d2	P62838	Ubiquitin-conjugating enzyme E2 D2 OS=Mus musculus OX=10090 GN=Ube2d2 PE=1 SV=1	1.25
Dtl	Q3TLR7	Denticleless protein homolog OS=Mus musculus OX=10090 GN=Dtl PE=1 SV=2	2.54

Serpinb1a	Q9D154	Leukocyte elastase inhibitor A OS=Mus musculus OX=10090 GN=Serpinb1a PE=1 SV=1	0.43
Epn1	Q80VP1	Epsin-1 OS=Mus musculus OX=10090 GN=Epn1 PE=1 SV=3	0.883333333
Atp6v0a2	P15920	V-type proton ATPase 116 kDa subunit a2 OS=Mus musculus OX=10090 GN=Atp6v0a2 PE=1 SV=2	0.746666667
Kdelr2	Q9CQM2	ER lumen protein-retaining receptor 2 OS=Mus musculus OX=10090 GN=Kdelr2 PE=1 SV=1	0.87
Sipa1l2	Q80TE4	Signal-induced proliferation-associated 1-like protein 2 OS=Mus musculus OX=10090 GN=Sipa1l2 P	0.925
Runx2	Q08775	Runt-related transcription factor 2 OS=Mus musculus OX=10090 GN=Runx2 PE=1 SV=2	0.973333333
Nab1	Q61122	NGFI-A-binding protein 1 OS=Mus musculus OX=10090 GN=Nab1 PE=1 SV=2	0.83
Phc2	Q9QWH1	Polyhomeotic-like protein 2 OS=Mus musculus OX=10090 GN=Phc2 PE=1 SV=1	0.95
Dhrs7	Q9CXR1	Dehydrogenase/reductase SDR family member 7 OS=Mus musculus OX=10090 GN=Dhrs7 PE=1 SV=2	0.846666667
Cc2d1b	Q8BRN9	Coiled-coil and C2 domain-containing protein 1B OS=Mus musculus OX=10090 GN=Cc2d1b PE=1 S\	
Rcbtb1	Q6NXM2	RCC1 and BTB domain-containing protein 1 OS=Mus musculus OX=10090 GN=Rcbtb1 PE=1 SV=1	5.57
Creb1	Q01147	Cyclic AMP-responsive element-binding protein 1 OS=Mus musculus OX=10090 GN=Creb1 PE=1 SV=	0.85
Numb	Q9QZS3	Protein numb homolog OS=Mus musculus OX=10090 GN=Numb PE=1 SV=1	0.736666667
Med25	Q8VCB2	Mediator of RNA polymerase II transcription subunit 25 OS=Mus musculus OX=10090 GN=Med25 P	1.163333333
Tsc22d1	P62500	TSC22 domain family protein 1 OS=Mus musculus OX=10090 GN=Tsc22d1 PE=1 SV=2	2.003333333
Cdc34	Q8CFI2	Ubiquitin-conjugating enzyme E2 R1 OS=Mus musculus OX=10090 GN=Cdc34 PE=1 SV=1	2.233333333
Foxp4	Q9DBY0	Forkhead box protein P4 OS=Mus musculus OX=10090 GN=Foxp4 PE=1 SV=1	2.305
Map1lc3b	Q9CQV6	Microtubule-associated proteins 1A/1B light chain 3B OS=Mus musculus OX=10090 GN=Map1lc3b	1.556666667
Mrtfb	P59759	Myocardin-related transcription factor B OS=Mus musculus OX=10090 GN=Mrtfb PE=1 SV=1	0.65
Mtmr1	Q9Z2C4	Myotubularin-related protein 1 OS=Mus musculus OX=10090 GN=Mtmr1 PE=1 SV=1	1.225
Hbb-b2	P02089	Hemoglobin subunit beta-2 OS=Mus musculus OX=10090 GN=Hbb-b2 PE=1 SV=2	0.076666667
Hbb-b1	P02088	Hemoglobin subunit beta-1 OS=Mus musculus OX=10090 GN=Hbb-b1 PE=1 SV=2	0.076666667
Ube2a	Q9Z255	Ubiquitin-conjugating enzyme E2 A OS=Mus musculus OX=10090 GN=Ube2a PE=1 SV=1	0.9
Kif14	L0N7N1	Kinesin-like protein KIF14 OS=Mus musculus OX=10090 GN=Kif14 PE=1 SV=1	0.975
Mug1	P28665	Murinoglobulin-1 OS=Mus musculus OX=10090 GN=Mug1 PE=1 SV=3	0.196666667
Wwp2	Q9DBH0	NEDD4-like E3 ubiquitin-protein ligase WWP2 OS=Mus musculus OX=10090 GN=Wwp2 PE=1 SV=1	1.345
Ssbp2	Q9CYZ8	Single-stranded DNA-binding protein 2 OS=Mus musculus OX=10090 GN=Ssbp2 PE=1 SV=1	0.82
Fam76a	Q922G2	Protein FAM76A OS=Mus musculus OX=10090 GN=Fam76a PE=2 SV=1	1.226666667
Col2a1	P28481	Collagen alpha-1(II) chain OS=Mus musculus OX=10090 GN=Col2a1 PE=1 SV=2	32.895
Krt2	Q3TTY5	Keratin type II cytoskeletal 2 epidermal OS=Mus musculus OX=10090 GN=Krt2 PE=1 SV=1	1.27
Exog	Q8C163	Nuclease EXOG mitochondrial OS=Mus musculus OX=10090 GN=Exog PE=1 SV=1	
Evi5	P97366	Ecotropic viral integration site 5 protein OS=Mus musculus OX=10090 GN=Evi5 PE=1 SV=2	0.95
Kmt2d	Q6PDK2	Histone-lysine N-methyltransferase 2D OS=Mus musculus OX=10090 GN=Kmt2d PE=1 SV=2	
Gga3	Q8BMI3	ADP-ribosylation factor-binding protein GGA3 OS=Mus musculus OX=10090 GN=Gga3 PE=1 SV=2	0.715

Dmd	P11531	Dystrophin OS=Mus musculus OX=10090 GN=Dmd PE=1 SV=3	0.993333333
Chchd2	Q9D1L0	Coiled-coil-helix-coiled-coil-helix domain-containing protein 2 OS=Mus musculus OX=10090 GN=Cl	0.886666667
Jpt1	P97825	Jupiter microtubule associated homolog 1 OS=Mus musculus OX=10090 GN=Jpt1 PE=1 SV=3	0.813333333
Cd81	P35762	CD81 antigen OS=Mus musculus OX=10090 GN=Cd81 PE=1 SV=2	0.82
Pip5k1a	P70182	Phosphatidylinositol 4-phosphate 5-kinase type-1 alpha OS=Mus musculus OX=10090 GN=Pip5k1a	1.01
Ecd	Q9CS74	Protein ecdysoneless homolog OS=Mus musculus OX=10090 GN=Ecd PE=1 SV=2	0.653333333
Gfm2	Q8R2Q4	Ribosome-releasing factor 2 mitochondrial OS=Mus musculus OX=10090 GN=Gfm2 PE=1 SV=2	0.81
Degs1	O09005	Sphingolipid delta(4)-desaturase DES1 OS=Mus musculus OX=10090 GN=Degs1 PE=1 SV=1	0.946666667
Uxs1	Q91XL3	UDP-glucuronic acid decarboxylase 1 OS=Mus musculus OX=10090 GN=Uxs1 PE=1 SV=1	0.88
Pbx1	P41778	Pre-B-cell leukemia transcription factor 1 OS=Mus musculus OX=10090 GN=Pbx1 PE=1 SV=2	1.006666667
Rpl22	P67984	60S ribosomal protein L22 OS=Mus musculus OX=10090 GN=Rpl22 PE=1 SV=2	0.896666667
Bub1	O08901	Mitotic checkpoint serine/threonine-protein kinase BUB1 OS=Mus musculus OX=10090 GN=Bub1 P	0.843333333
Cks1b	P61025	Cyclin-dependent kinases regulatory subunit 1 OS=Mus musculus OX=10090 GN=Cks1b PE=3 SV=1	0.9
Chtop	Q9CY57	Chromatin target of PRMT1 protein OS=Mus musculus OX=10090 GN=Chtop PE=1 SV=2	0.86
Atg2b	Q80XK6	Autophagy-related protein 2 homolog B OS=Mus musculus OX=10090 GN=Atg2b PE=1 SV=3	0.723333333
Ift22	Q9DAI2	Intraflagellar transport protein 22 homolog OS=Mus musculus OX=10090 GN=Ift22 PE=1 SV=1	0.843333333
Arid5b	Q8BM75	AT-rich interactive domain-containing protein 5B OS=Mus musculus OX=10090 GN=Arid5b PE=1 SV	0.663333333
Ppp1r11	Q8K1L5	E3 ubiquitin-protein ligase PPP1R11 OS=Mus musculus OX=10090 GN=Ppp1r11 PE=1 SV=1	0.9
Gpatch4	Q3TFK5	G patch domain-containing protein 4 OS=Mus musculus OX=10090 GN=Gpatch4 PE=1 SV=1	0.856666667
Hypk	Q9CR41	Huntingtin-interacting protein K OS=Mus musculus OX=10090 GN=Hypk PE=1 SV=2	0.833333333
Coq5	Q9CXI0	2-methoxy-6-polyprenyl-1 4-benzoquinol methylase mitochondrial OS=Mus musculus OX=10090 G	0.946666667
Ergic2	Q9CR89	Endoplasmic reticulum-Golgi intermediate compartment protein 2 OS=Mus musculus OX=10090 G	0.92
Fbxo38	Q8BMI0	F-box only protein 38 OS=Mus musculus OX=10090 GN=Fbxo38 PE=1 SV=1	0.783333333
Commd8	Q9CZG3	COMM domain-containing protein 8 OS=Mus musculus OX=10090 GN=Commd8 PE=1 SV=1	0.586666667
Dbi	P31786	Acyl-CoA-binding protein OS=Mus musculus OX=10090 GN=Dbi PE=1 SV=2	0.856666667
Smg8	Q8VE18	Protein SMG8 OS=Mus musculus OX=10090 GN=Smg8 PE=1 SV=1	1.843333333
Ddrgk1	Q80WW9	DDRGK domain-containing protein 1 OS=Mus musculus OX=10090 GN=Ddrgk1 PE=1 SV=2	0.72
Yipf5	Q9EQQ2	Protein YIPF5 OS=Mus musculus OX=10090 GN=Yipf5 PE=1 SV=1	0.94
Hsbp1	Q9CQZ1	Heat shock factor-binding protein 1 OS=Mus musculus OX=10090 GN=Hsbp1 PE=1 SV=1	0.826666667
Rbx1	P62878	E3 ubiquitin-protein ligase RBX1 OS=Mus musculus OX=10090 GN=Rbx1 PE=1 SV=1	0.96
Slc7a6os	Q7TPE5	Probable RNA polymerase II nuclear localization protein SLC7A6OS OS=Mus musculus OX=10090 GI	0.913333333
Arsa	P50428	Arylsulfatase A OS=Mus musculus OX=10090 GN=Arsa PE=1 SV=2	0.856666667
Gstt3	Q99L20	Glutathione S-transferase theta-3 OS=Mus musculus OX=10090 GN=Gstt3 PE=1 SV=1	1.286666667
Brd7	O88665	Bromodomain-containing protein 7 OS=Mus musculus OX=10090 GN=Brd7 PE=1 SV=1	0.785

Ankmy2	Q3TPE9	Ankyrin repeat and MYND domain-containing protein 2 OS=Mus musculus OX=10090 GN=Ankmy2 F	0.826666667
Txn2	P97493	Thioredoxin mitochondrial OS=Mus musculus OX=10090 GN=Txn2 PE=1 SV=1	0.873333333
Dhrs7b	Q99J47	Dehydrogenase/reductase SDR family member 7B OS=Mus musculus OX=10090 GN=Dhrs7b PE=1 SV	1.156666667
Il1rn	P25085	Interleukin-1 receptor antagonist protein OS=Mus musculus OX=10090 GN=Il1rn PE=2 SV=1	0.81
Zeb1	Q64318	Zinc finger E-box-binding homeobox 1 OS=Mus musculus OX=10090 GN=Zeb1 PE=1 SV=1	0.716666667
Sf3b5	Q923D4	Splicing factor 3B subunit 5 OS=Mus musculus OX=10090 GN=Sf3b5 PE=1 SV=1	0.72
Mcfd2	Q8K5B2	Multiple coagulation factor deficiency protein 2 homolog OS=Mus musculus OX=10090 GN=Mcfd2	1.016666667
Mmp2	P33434	72 kDa type IV collagenase OS=Mus musculus OX=10090 GN=Mmp2 PE=1 SV=1	1.206666667
Gcsh	Q91WK5	Glycine cleavage system H protein mitochondrial OS=Mus musculus OX=10090 GN=Gcsh PE=1 SV=2	0.966666667
Dpy30	Q99LT0	Protein dpy-30 homolog OS=Mus musculus OX=10090 GN=Dpy30 PE=1 SV=1	0.88
Sumf1	Q8R0F3	Formylglycine-generating enzyme OS=Mus musculus OX=10090 GN=Sumf1 PE=1 SV=2	0.64
Mrpl1	Q99N96	39S ribosomal protein L1 mitochondrial OS=Mus musculus OX=10090 GN=Mrpl1 PE=1 SV=2	0.85
Kcmf1	Q80UY2	E3 ubiquitin-protein ligase KCMF1 OS=Mus musculus OX=10090 GN=Kcmf1 PE=1 SV=1	0.89
Ghitm	Q91VC9	Growth hormone-inducible transmembrane protein OS=Mus musculus OX=10090 GN=Ghitm PE=1	0.77
Lgmn	O89017	Legumain OS=Mus musculus OX=10090 GN=Lgmn PE=1 SV=1	0.836666667
Naa40	Q8VE10	N-alpha-acetyltransferase 40 OS=Mus musculus OX=10090 GN=Naa40 PE=1 SV=1	0.77
Mocs3	A2BDX3	Adenylyltransferase and sulfurtransferase MOCS3 OS=Mus musculus OX=10090 GN=Mocs3 PE=1 SV=	0.873333333
Nradd	Q8CJ26	Death domain-containing membrane protein NRADD OS=Mus musculus OX=10090 GN=Nradd PE=1	0.933333333
Gnl3l	Q6PGG6	Guanine nucleotide-binding protein-like 3-like protein OS=Mus musculus OX=10090 GN=Gnl3l PE=:	0.926666667
Tpmt	O55060	Thiopurine S-methyltransferase OS=Mus musculus OX=10090 GN=Tpmt PE=1 SV=1	1.323333333
Tbc1d9b	Q5SVR0	TBC1 domain family member 9B OS=Mus musculus OX=10090 GN=Tbc1d9b PE=1 SV=1	1.296666667
Taf10	Q8K0H5	Transcription initiation factor TFIID subunit 10 OS=Mus musculus OX=10090 GN=Taf10 PE=1 SV=1	0.913333333
Bid	P70444	BH3-interacting domain death agonist OS=Mus musculus OX=10090 GN=Bid PE=1 SV=2	0.98
Vamp3	P63024	Vesicle-associated membrane protein 3 OS=Mus musculus OX=10090 GN=Vamp3 PE=1 SV=1	0.903333333
Rad18	Q9QXK2	E3 ubiquitin-protein ligase RAD18 OS=Mus musculus OX=10090 GN=Rad18 PE=1 SV=2	0.656666667
	Q9D7E4	UPF0449 protein C19orf25 homolog OS=Mus musculus OX=10090 PE=1 SV=1	0.816666667
Rtn3	Q9ES97	Reticulon-3 OS=Mus musculus OX=10090 GN=Rtn3 PE=1 SV=2	0.933333333
Osmr	O70458	Oncostatin-M-specific receptor subunit beta OS=Mus musculus OX=10090 GN=Osmr PE=1 SV=1	0.465
Mrps16	Q9CPX7	28S ribosomal protein S16 mitochondrial OS=Mus musculus OX=10090 GN=Mrps16 PE=1 SV=1	0.946666667
Enpp3	Q6DYE8	Ectonucleotide pyrophosphatase/phosphodiesterase family member 3 OS=Mus musculus OX=1009(	1.106666667
Ldah	Q8BVA5	Lipid droplet-associated hydrolase OS=Mus musculus OX=10090 GN=Ldah PE=1 SV=1	0.92
Ypel5	P62700	Protein yippee-like 5 OS=Mus musculus OX=10090 GN=Ypel5 PE=1 SV=1	1.436666667
Coa7	Q921H9	Cytochrome c oxidase assembly factor 7 OS=Mus musculus OX=10090 GN=Coa7 PE=1 SV=1	0.843333333
Bcs1l	Q9CZP5	Mitochondrial chaperone BCS1 OS=Mus musculus OX=10090 GN=Bcs1l PE=1 SV=1	0.96

Slc38a2	Q8CFE6	Sodium-coupled neutral amino acid transporter 2 OS=Mus musculus OX=10090 GN=Slc38a2 PE=1 S	0.976666667
Mmgt1	Q8K273	Membrane magnesium transporter 1 OS=Mus musculus OX=10090 GN=Mmgt1 PE=1 SV=1	0.93
Fbxo3	Q9DC63	F-box only protein 3 OS=Mus musculus OX=10090 GN=Fbxo3 PE=1 SV=1	0.903333333
Eif4ebp2	P70445	Eukaryotic translation initiation factor 4E-binding protein 2 OS=Mus musculus OX=10090 GN=Eif4e	0.633333333
Stx17	Q9D0I4	Syntaxin-17 OS=Mus musculus OX=10090 GN=Stx17 PE=1 SV=1	0.633333333
Sypl1	O09117	Synaptophysin-like protein 1 OS=Mus musculus OX=10090 GN=Sypl1 PE=1 SV=2	0.926666667
Mrpl27	Q99N92	39S ribosomal protein L27 mitochondrial OS=Mus musculus OX=10090 GN=Mrpl27 PE=1 SV=1	0.886666667
Micu1	Q8VCX5	Calcium uptake protein 1 mitochondrial OS=Mus musculus OX=10090 GN=Micu1 PE=1 SV=1	1.12
Mpp7	Q8BVD5	MAGUK p55 subfamily member 7 OS=Mus musculus OX=10090 GN=Mpp7 PE=1 SV=2	1.016666667
Rpl22l1	Q9D7S7	60S ribosomal protein L22-like 1 OS=Mus musculus OX=10090 GN=Rpl22l1 PE=1 SV=1	0.913333333
Wbp4	Q61048	WW domain-binding protein 4 OS=Mus musculus OX=10090 GN=Wbp4 PE=1 SV=4	0.776666667
Abcb7	Q61102	ATP-binding cassette sub-family B member 7 mitochondrial OS=Mus musculus OX=10090 GN=Abcb	0.89
Npc2	Q9Z0J0	NPC intracellular cholesterol transporter 2 OS=Mus musculus OX=10090 GN=Npc2 PE=1 SV=1	0.786666667
Maea	Q4VC33	E3 ubiquitin-protein transferase MAEA OS=Mus musculus OX=10090 GN=Maea PE=1 SV=1	1.36
Fblim1	Q71FD7	Filamin-binding LIM protein 1 OS=Mus musculus OX=10090 GN=Fblim1 PE=1 SV=2	0.976666667
Pyroxd1	Q3TMV7	Pyridine nucleotide-disulfide oxidoreductase domain-containing protein 1 OS=Mus musculus OX=1	0.913333333
Isy1	Q69ZQ2	Pre-mRNA-splicing factor ISY1 homolog OS=Mus musculus OX=10090 GN=Isy1 PE=1 SV=2	0.79
Gpn3	Q9D3W4	GPN-loop GTPase 3 OS=Mus musculus OX=10090 GN=Gpn3 PE=2 SV=1	0.963333333
Peg10	Q7TN75	Retrotransposon-derived protein PEG10 OS=Mus musculus OX=10090 GN=Peg10 PE=1 SV=2	0.856666667
Sap30bp	Q02614	SAP30-binding protein OS=Mus musculus OX=10090 GN=Sap30bp PE=1 SV=2	0.826666667
Atp6ap1	Q9R1Q9	V-type proton ATPase subunit S1 OS=Mus musculus OX=10090 GN=Atp6ap1 PE=1 SV=1	0.883333333
Mgst3	Q9CPU4	Microsomal glutathione S-transferase 3 OS=Mus musculus OX=10090 GN=Mgst3 PE=1 SV=1	1.09
Sco2	Q8VCL2	Protein SCO2 homolog mitochondrial OS=Mus musculus OX=10090 GN=Sco2 PE=1 SV=1	0.88
Gripap1	Q8VD04	GRIP1-associated protein 1 OS=Mus musculus OX=10090 GN=Gripap1 PE=1 SV=1	0.755
Bud23	Q9CY21	Probable 18S rRNA (guanine-N(7))-methyltransferase OS=Mus musculus OX=10090 GN=Bud23 PE=1	0.866666667
Rsrc2	A2RTL5	Arginine/serine-rich coiled-coil protein 2 OS=Mus musculus OX=10090 GN=Rsrc2 PE=2 SV=1	0.86
Ttc9c	Q810A3	Tetratricopeptide repeat protein 9C OS=Mus musculus OX=10090 GN=Ttc9c PE=1 SV=1	0.97
Dctn3	Q9Z0Y1	Dynactin subunit 3 OS=Mus musculus OX=10090 GN=Dctn3 PE=1 SV=2	0.776666667
Lrch2	Q3UMG5	Leucine-rich repeat and calponin homology domain-containing protein 2 OS=Mus musculus OX=10	0.745
Rmc1	Q8VC42	Regulator of MON1-CCZ1 complex OS=Mus musculus OX=10090 GN=Rmc1 PE=2 SV=1	0.97
Bri3bp	Q8BXV2	BRI3-binding protein OS=Mus musculus OX=10090 GN=Bri3bp PE=1 SV=1	0.863333333
Mrpl22	Q8BU88	39S ribosomal protein L22 mitochondrial OS=Mus musculus OX=10090 GN=Mrpl22 PE=1 SV=1	0.7
Gbe1	Q9D6Y9	1 4-alpha-glucan-branching enzyme OS=Mus musculus OX=10090 GN=Gbe1 PE=1 SV=1	0.823333333
Chid1	Q922Q9	Chitinase domain-containing protein 1 OS=Mus musculus OX=10090 GN=Chid1 PE=1 SV=1	1.393333333

Gemin6	Q9CX53	Gem-associated protein 6 OS=Mus musculus OX=10090 GN=Gemin6 PE=1 SV=2	0.86
Mmp14	P53690	Matrix metalloproteinase-14 OS=Mus musculus OX=10090 GN=Mmp14 PE=2 SV=3	0.856666667
Abracl	Q4KML4	Costars family protein ABRACL OS=Mus musculus OX=10090 GN=Abracl PE=1 SV=1	0.916666667
Copz2	Q9JHH9	Coatomer subunit zeta-2 OS=Mus musculus OX=10090 GN=Copz2 PE=1 SV=1	0.766666667
Ndufs7	Q9DC70	NADH dehydrogenase [ubiquinone] iron-sulfur protein 7 mitochondrial OS=Mus musculus OX=10(	0.806666667
Ptpmt1	Q66GT5	Phosphatidylglycerophosphatase and protein-tyrosine phosphatase 1 OS=Mus musculus OX=10090	1.15
Bckdhb	Q6P3A8	2-oxoisovalerate dehydrogenase subunit beta mitochondrial OS=Mus musculus OX=10090 GN=Bck	0.973333333
Pir	Q9D711	Pirin OS=Mus musculus OX=10090 GN=Pir PE=1 SV=1	0.973333333
Tox4	Q8BU11	TOX high mobility group box family member 4 OS=Mus musculus OX=10090 GN=Tox4 PE=1 SV=3	0.956666667
Slc39a14	Q75N73	Metal cation symporter ZIP14 OS=Mus musculus OX=10090 GN=Slc39a14 PE=1 SV=1	1.146666667
Mtmr9	Q9Z2D0	Myotubularin-related protein 9 OS=Mus musculus OX=10090 GN=Mtmr9 PE=1 SV=2	1.113333333
Ints12	Q9D168	Integrator complex subunit 12 OS=Mus musculus OX=10090 GN=Ints12 PE=1 SV=1	1.26
Sf3b6	P59708	Splicing factor 3B subunit 6 OS=Mus musculus OX=10090 GN=Sf3b6 PE=1 SV=1	0.88
Ndufa11	Q9D8B4	NADH dehydrogenase [ubiquinone] 1 alpha subcomplex subunit 11 OS=Mus musculus OX=10090 GI	1.19
Dusp3	Q9D7X3	Dual specificity protein phosphatase 3 OS=Mus musculus OX=10090 GN=Dusp3 PE=1 SV=1	0.91
Gng12	Q9DAS9	Guanine nucleotide-binding protein G(I)/G(S)/G(O) subunit gamma-12 OS=Mus musculus OX=1009(	0.856666667
Hdhd2	Q3UGR5	Haloacid dehalogenase-like hydrolase domain-containing protein 2 OS=Mus musculus OX=10090 GI	1.236666667
Hacd3	Q8K2C9	Very-long-chain (3R)-3-hydroxyacyl-CoA dehydratase 3 OS=Mus musculus OX=10090 GN=Hacd3 PE=	0.793333333
Necap2	Q9D1J1	Adaptin ear-binding coat-associated protein 2 OS=Mus musculus OX=10090 GN=Necap2 PE=1 SV=1	1.116666667
Iscu	Q9D7P6	Iron-sulfur cluster assembly enzyme ISCU mitochondrial OS=Mus musculus OX=10090 GN=Iscu PE=	0.786666667
Efr3a	Q8BG67	Protein EFR3 homolog A OS=Mus musculus OX=10090 GN=Efr3a PE=1 SV=1	0.756666667
Rnf25	Q9QZR0	E3 ubiquitin-protein ligase RNF25 OS=Mus musculus OX=10090 GN=Rnf25 PE=1 SV=2	1.07
Ube2h	P62257	Ubiquitin-conjugating enzyme E2 H OS=Mus musculus OX=10090 GN=Ube2h PE=1 SV=1	1.05
Yif1a	Q91XB7	Protein YIF1A OS=Mus musculus OX=10090 GN=Yif1a PE=1 SV=1	0.753333333
Grn	P28798	Progranulin OS=Mus musculus OX=10090 GN=Grn PE=1 SV=2	1.583333333
Rilpl1	Q9JJC6	RILP-like protein 1 OS=Mus musculus OX=10090 GN=Rilpl1 PE=1 SV=1	0.803333333
Srp19	Q9D7A6	Signal recognition particle 19 kDa protein OS=Mus musculus OX=10090 GN=Srp19 PE=1 SV=1	0.886666667
Cdh13	Q9WTR5	Cadherin-13 OS=Mus musculus OX=10090 GN=Cdh13 PE=1 SV=2	0.94
Timm9	Q9WV98	Mitochondrial import inner membrane translocase subunit Tim9 OS=Mus musculus OX=10090 GN=	0.846666667
Acbd6	Q9D061	Acyl-CoA-binding domain-containing protein 6 OS=Mus musculus OX=10090 GN=Acbd6 PE=1 SV=2	0.796666667
Bag4	Q8CI61	BAG family molecular chaperone regulator 4 OS=Mus musculus OX=10090 GN=Bag4 PE=1 SV=2	2.283333333
Inip	Q3TXT3	SOSS complex subunit C OS=Mus musculus OX=10090 GN=Inip PE=3 SV=1	0.983333333
Lactb	Q9EP89	Serine beta-lactamase-like protein LACTB mitochondrial OS=Mus musculus OX=10090 GN=Lactb PE	1.083333333
Ramac	Q9CQY2	RNA guanine-N7 methyltransferase activating subunit OS=Mus musculus OX=10090 GN=Ramac PE=:	1.046666667

Nol7	Q9D7Z3	Nucleolar protein 7 OS=Mus musculus OX=10090 GN=Nol7 PE=1 SV=1	1.073333333
Snapin	Q9Z266	SNARE-associated protein Snapin OS=Mus musculus OX=10090 GN=Snapin PE=1 SV=1	0.72
Cdc37l1	Q9CZP7	Hsp90 co-chaperone Cdc37-like 1 OS=Mus musculus OX=10090 GN=Cdc37l1 PE=1 SV=1	0.82
Rwdd1	Q9CQK7	RWD domain-containing protein 1 OS=Mus musculus OX=10090 GN=Rwdd1 PE=1 SV=1	0.84
Chek1	O35280	Serine/threonine-protein kinase Chk1 OS=Mus musculus OX=10090 GN=Chek1 PE=1 SV=2	0.77
Tmem119	Q8R138	Transmembrane protein 119 OS=Mus musculus OX=10090 GN=Tmem119 PE=1 SV=1	0.8
Hspb11	Q9D6H2	Intraflagellar transport protein 25 homolog OS=Mus musculus OX=10090 GN=Hspb11 PE=1 SV=2	0.7
Impa2	Q91UZ5	Inositol monophosphatase 2 OS=Mus musculus OX=10090 GN=Impa2 PE=1 SV=1	0.833333333
MINDY3	Q9CV28	Ubiquitin carboxyl-terminal hydrolase MINDY-3 OS=Mus musculus OX=10090 GN=MINDY3 PE=1 SV	0.76
Prnp	P04925	Major prion protein OS=Mus musculus OX=10090 GN=Prnp PE=1 SV=2	0.903333333
Cox20	Q9D7J4	Cytochrome c oxidase assembly protein COX20 mitochondrial OS=Mus musculus OX=10090 GN=Co	0.86
Timm10b	Q9WV96	Mitochondrial import inner membrane translocase subunit Tim10 B OS=Mus musculus OX=10090 G	0.89
Clybl	Q8R4N0	Citramalyl-CoA lyase mitochondrial OS=Mus musculus OX=10090 GN=Clybl PE=1 SV=2	0.756666667
Ccnk	O88874	Cyclin-K OS=Mus musculus OX=10090 GN=Ccnk PE=1 SV=3	0.973333333
Med22	Q62276	Mediator of RNA polymerase II transcription subunit 22 OS=Mus musculus OX=10090 GN=Med22 PI	0.78
Vps37b	Q8R0J7	Vacuolar protein sorting-associated protein 37B OS=Mus musculus OX=10090 GN=Vps37b PE=1 SV=	0.873333333
Cd9	P40240	CD9 antigen OS=Mus musculus OX=10090 GN=Cd9 PE=1 SV=2	0.913333333
Armh3	Q6PD19	Armadillo-like helical domain-containing protein 3 OS=Mus musculus OX=10090 GN=Armh3 PE=1 S	0.763333333
Dr1	Q91WV0	Protein Dr1 OS=Mus musculus OX=10090 GN=Dr1 PE=1 SV=1	0.92
Atn1	O35126	Atrophin-1 OS=Mus musculus OX=10090 GN=Atn1 PE=1 SV=1	0.966666667
Gins2	Q9D600	DNA replication complex GINS protein PSF2 OS=Mus musculus OX=10090 GN=Gins2 PE=1 SV=1	0.906666667
Parg	O88622	Poly(ADP-ribose) glycohydrolase OS=Mus musculus OX=10090 GN=Parg PE=1 SV=2	1.403333333
Tp53rk	Q99PW4	EKC/KEOPS complex subunit Tp53rk OS=Mus musculus OX=10090 GN=Tp53rk PE=2 SV=1	0.863333333
Cd63	P41731	CD63 antigen OS=Mus musculus OX=10090 GN=Cd63 PE=1 SV=2	0.736666667
Ndufa6	Q9CQZ5	NADH dehydrogenase [ubiquinone] 1 alpha subcomplex subunit 6 OS=Mus musculus OX=10090 GN	1
Sgcd	P82347	Delta-sarcoglycan OS=Mus musculus OX=10090 GN=Sgcd PE=1 SV=1	1.55
Rbl1	Q64701	Retinoblastoma-like protein 1 OS=Mus musculus OX=10090 GN=Rbl1 PE=1 SV=3	4.11
Gng10	Q9CXP8	Guanine nucleotide-binding protein G(I)/G(S)/G(O) subunit gamma-10 OS=Mus musculus OX=1009(	1.063333333
Akt1s1	Q9D1F4	Proline-rich AKT1 substrate 1 OS=Mus musculus OX=10090 GN=Akt1s1 PE=1 SV=1	0.733333333
Orc3	Q9JK30	Origin recognition complex subunit 3 OS=Mus musculus OX=10090 GN=Orc3 PE=1 SV=1	1.236666667
Prkrip1	Q9CWV6	PRKR-interacting protein 1 OS=Mus musculus OX=10090 GN=Prkrip1 PE=1 SV=2	0.99
Nid2	O88322	Nidogen-2 OS=Mus musculus OX=10090 GN=Nid2 PE=1 SV=2	
Ndufs6	P52503	NADH dehydrogenase [ubiquinone] iron-sulfur protein 6 mitochondrial OS=Mus musculus OX=1009	0.86
Vps33a	Q9D2N9	Vacuolar protein sorting-associated protein 33A OS=Mus musculus OX=10090 GN=Vps33a PE=1 SV=	1.366666667

Dpy19l1	A6X919	Probable C-mannosyltransferase DPY19L1 OS=Mus musculus OX=10090 GN=Dpy19l1 PE=1 SV=1	1.26
Alg10b	Q3UGP8	Putative Dol-P-Glc:Glc(2)Man(9)GlcNAc(2)-PP-Dol alpha-1 2-glucosyltransferase OS=Mus musculus (	0.7
Ccna2	P51943	Cyclin-A2 OS=Mus musculus OX=10090 GN=Ccna2 PE=1 SV=2	0.89
Commd7	Q8BG94	COMM domain-containing protein 7 OS=Mus musculus OX=10090 GN=Commd7 PE=1 SV=1	1.113333333
Rbms1	Q91W59	RNA-binding motif single-stranded-interacting protein 1 OS=Mus musculus OX=10090 GN=Rbms1 F	0.823333333
Gpat4	Q8K2C8	Glycerol-3-phosphate acyltransferase 4 OS=Mus musculus OX=10090 GN=Gpat4 PE=1 SV=1	0.823333333
Gramd4	Q8CB44	GRAM domain-containing protein 4 OS=Mus musculus OX=10090 GN=Gramd4 PE=1 SV=1	1.426666667
Senp7	Q8BUH8	Sentrin-specific protease 7 OS=Mus musculus OX=10090 GN=Senp7 PE=1 SV=1	1.23
Sh3bgrl3	Q91VW3	SH3 domain-binding glutamic acid-rich-like protein 3 OS=Mus musculus OX=10090 GN=Sh3bgrl3 PI	0.956666667
Hspa13	Q8BM72	Heat shock 70 kDa protein 13 OS=Mus musculus OX=10090 GN=Hspa13 PE=1 SV=1	1.07
Cebpb	P28033	CCAAT/enhancer-binding protein beta OS=Mus musculus OX=10090 GN=Cebpb PE=1 SV=1	0.956666667
Lrrk2	Q5S006	Leucine-rich repeat serine/threonine-protein kinase 2 OS=Mus musculus OX=10090 GN=Lrrk2 PE=1	1.173333333
Srp14	P16254	Signal recognition particle 14 kDa protein OS=Mus musculus OX=10090 GN=Srp14 PE=1 SV=1	0.95
Pinx1	Q9CZX5	PIN2/TERF1-interacting telomerase inhibitor 1 OS=Mus musculus OX=10090 GN=Pinx1 PE=1 SV=2	0.783333333
Tdp2	Q9JJX7	Tyrosyl-DNA phosphodiesterase 2 OS=Mus musculus OX=10090 GN=Tdp2 PE=1 SV=1	1.076666667
Gadd45gip1	Q9CR59	Growth arrest and DNA damage-inducible proteins-interacting protein 1 OS=Mus musculus OX=100	1.01
Llph	Q9D945	Protein LLP homolog OS=Mus musculus OX=10090 GN=Llph PE=1 SV=1	1.056666667
Timm17a	Q9Z0V8	Mitochondrial import inner membrane translocase subunit Tim17-A OS=Mus musculus OX=10090 (	0.803333333
Lrig1	P70193	Leucine-rich repeats and immunoglobulin-like domains protein 1 OS=Mus musculus OX=10090 GN=	1.745
Gtf2e2	Q9D902	General transcription factor IIE subunit 2 OS=Mus musculus OX=10090 GN=Gtf2e2 PE=1 SV=2	0.77
Cdc26	Q99JP4	Anaphase-promoting complex subunit CDC26 OS=Mus musculus OX=10090 GN=Cdc26 PE=1 SV=1	1.37
Uchl1	Q9R0P9	Ubiquitin carboxyl-terminal hydrolase isozyme L1 OS=Mus musculus OX=10090 GN=Uchl1 PE=1 SV=	0.683333333
Rbm7	Q9CQT2	RNA-binding protein 7 OS=Mus musculus OX=10090 GN=Rbm7 PE=1 SV=1	1.07
Ndufc2	Q9CQ54	NADH dehydrogenase [ubiquinone] 1 subunit C2 OS=Mus musculus OX=10090 GN=Ndufc2 PE=1 SV=	1.056666667
Micall1	Q8BGT6	MICAL-like protein 1 OS=Mus musculus OX=10090 GN=Micall1 PE=1 SV=3	1.12
Arhgef17	Q80U35	Rho guanine nucleotide exchange factor 17 OS=Mus musculus OX=10090 GN=Arhgef17 PE=1 SV=2	0.74
Ccdc51	Q3URS9	Mitochondrial potassium channel OS=Mus musculus OX=10090 GN=Ccdc51 PE=1 SV=1	3.196666667
Dda1	Q9D9Z5	DET1- and DDB1-associated protein 1 OS=Mus musculus OX=10090 GN=Dda1 PE=1 SV=1	1.613333333
Mrps14	Q9CR88	28S ribosomal protein S14 mitochondrial OS=Mus musculus OX=10090 GN=Mrps14 PE=1 SV=1	0.86
Rbm45	Q8BHN5	RNA-binding protein 45 OS=Mus musculus OX=10090 GN=Rbm45 PE=1 SV=1	1.355
Cetn2	Q9R1K9	Centrin-2 OS=Mus musculus OX=10090 GN=Cetn2 PE=1 SV=1	0.91
Cox7a2	P48771	Cytochrome c oxidase subunit 7A2 mitochondrial OS=Mus musculus OX=10090 GN=Cox7a2 PE=1 S	0.98
Romo1	P60603	Reactive oxygen species modulator 1 OS=Mus musculus OX=10090 GN=Romo1 PE=2 SV=1	1.346666667
Mrpl32	Q9DCI9	39S ribosomal protein L32 mitochondrial OS=Mus musculus OX=10090 GN=Mrpl32 PE=1 SV=1	0.853333333

Sec61b	Q9CQS8	Protein transport protein Sec61 subunit beta OS=Mus musculus OX=10090 GN=Sec61b PE=1 SV=3	0.9
Fam172a	Q3TNH5	Cotranscriptional regulator FAM172A OS=Mus musculus OX=10090 GN=Fam172a PE=1 SV=2	0.876666667
Gins1	Q9CZ15	DNA replication complex GINS protein PSF1 OS=Mus musculus OX=10090 GN=Gins1 PE=1 SV=1	1.123333333
Sipa1l3	G3X9J0	Signal-induced proliferation-associated 1-like protein 3 OS=Mus musculus OX=10090 GN=Sipa1l3 P	0.866666667
Shq1	Q7TMX5	Protein SHQ1 homolog OS=Mus musculus OX=10090 GN=Shq1 PE=1 SV=2	1.436666667
Golm1	Q91XA2	Golgi membrane protein 1 OS=Mus musculus OX=10090 GN=Golm1 PE=1 SV=2	3.29
Naaa	Q9D7V9	N-acylethanolamine-hydrolyzing acid amidase OS=Mus musculus OX=10090 GN=Naaa PE=1 SV=2	0.63
Znfx1	Q8R151	NFX1-type zinc finger-containing protein 1 OS=Mus musculus OX=10090 GN=Znfx1 PE=1 SV=3	0.983333333
Ca13	Q9D6N1	Carbonic anhydrase 13 OS=Mus musculus OX=10090 GN=Ca13 PE=1 SV=1	0.67
Sdf4	Q61112	45 kDa calcium-binding protein OS=Mus musculus OX=10090 GN=Sdf4 PE=1 SV=1	0.716666667
Atxn7l3b	Q3UD01	Ataxin-7-like protein 3B OS=Mus musculus OX=10090 GN=Atxn7l3b PE=1 SV=1	0.826666667
Hdac4	Q6NZM9	Histone deacetylase 4 OS=Mus musculus OX=10090 GN=Hdac4 PE=1 SV=1	1.205
Nsd3	Q6P2L6	Histone-lysine N-methyltransferase NSD3 OS=Mus musculus OX=10090 GN=Nsd3 PE=1 SV=2	1.073333333
Commd6	Q3V4B5	COMM domain-containing protein 6 OS=Mus musculus OX=10090 GN=Commd6 PE=1 SV=1	1.176666667
S100a13	P97352	Protein S100-A13 OS=Mus musculus OX=10090 GN=S100a13 PE=1 SV=1	0.873333333
Cnot7	Q60809	CCR4-NOT transcription complex subunit 7 OS=Mus musculus OX=10090 GN=Cnot7 PE=1 SV=1	0.953333333
Rpap2	Q8VC34	Putative RNA polymerase II subunit B1 CTD phosphatase Rpap2 OS=Mus musculus OX=10090 GN=R	0.346666667
Aldh6a1	Q9EQ20	Methylmalonate-semialdehyde dehydrogenase [acylating] mitochondrial OS=Mus musculus OX=10	0.92
Cpt1a	P97742	Carnitine O-palmitoyltransferase 1 liver isoform OS=Mus musculus OX=10090 GN=Cpt1a PE=1 SV=4	1.193333333
Rheb	Q921J2	GTP-binding protein Rheb OS=Mus musculus OX=10090 GN=Rheb PE=1 SV=1	0.793333333
Ahdc1	Q6PAL7	AT-hook DNA-binding motif-containing protein 1 OS=Mus musculus OX=10090 GN=Ahdc1 PE=1 SV=	0.953333333
Dhcr7	O88455	7-dehydrocholesterol reductase OS=Mus musculus OX=10090 GN=Dhcr7 PE=1 SV=1	0.76
Pam16	Q9CQV1	Mitochondrial import inner membrane translocase subunit TIM16 OS=Mus musculus OX=10090 GN	1
Sfxn2	Q925N2	Sideroflexin-2 OS=Mus musculus OX=10090 GN=Sfxn2 PE=1 SV=1	1.14
Lyrm4	Q8K215	LYR motif-containing protein 4 OS=Mus musculus OX=10090 GN=Lyrm4 PE=1 SV=1	1.033333333
Timm10	P62073	Mitochondrial import inner membrane translocase subunit Tim10 OS=Mus musculus OX=10090 GN	1.133333333
Ess2	O70279	Splicing factor ESS-2 homolog OS=Mus musculus OX=10090 GN=Ess2 PE=1 SV=2	1.72
Fam210a	Q8BGY7	Protein FAM210A OS=Mus musculus OX=10090 GN=Fam210a PE=1 SV=1	0.78
Otud6b	Q8K2H2	Deubiquitinase OTUD6B OS=Mus musculus OX=10090 GN=Otud6b PE=1 SV=1	0.763333333
Pmf1	Q9CPV5	Polyamine-modulated factor 1 OS=Mus musculus OX=10090 GN=Pmf1 PE=1 SV=1	0.653333333
Kptn	Q8VCX6	KICSTOR complex protein kaptin OS=Mus musculus OX=10090 GN=Kptn PE=2 SV=1	0.746666667
Eloa	Q8CB77	Elongin-A OS=Mus musculus OX=10090 GN=Eloa PE=1 SV=3	0.785
Larp4b	Q6A0A2	La-related protein 4B OS=Mus musculus OX=10090 GN=Larp4b PE=1 SV=2	0.816666667
Znf830	Q8R1N0	Zinc finger protein 830 OS=Mus musculus OX=10090 GN=Znf830 PE=1 SV=1	0.826666667

Ska3	Q8C263	Spindle and kinetochore-associated protein 3 OS=Mus musculus OX=10090 GN=Ska3 PE=2 SV=1	0.69
Rps19bp1	Q8C6B9	Active regulator of SIRT1 OS=Mus musculus OX=10090 GN=Rps19bp1 PE=1 SV=1	0.84
Ccdc134	Q8C7V8	Coiled-coil domain-containing protein 134 OS=Mus musculus OX=10090 GN=Ccdc134 PE=1 SV=1	1.26
Adat2	Q6P6J0	tRNA-specific adenosine deaminase 2 OS=Mus musculus OX=10090 GN=Adat2 PE=1 SV=1	1.356666667
Hspb6	Q5EBG6	Heat shock protein beta-6 OS=Mus musculus OX=10090 GN=Hspb6 PE=1 SV=1	0.886666667
Cst3	P21460	Cystatin-C OS=Mus musculus OX=10090 GN=Cst3 PE=1 SV=2	0.833333333
Spcs1	Q9D958	Signal peptidase complex subunit 1 OS=Mus musculus OX=10090 GN=Spcs1 PE=2 SV=3	0.856666667
Cetn3	O35648	Centrin-3 OS=Mus musculus OX=10090 GN=Cetn3 PE=1 SV=1	1.006666667
	Q8BGA7	Uncharacterized protein C7orf26 homolog OS=Mus musculus OX=10090 PE=2 SV=1	3.016666667
Med17	Q8VCD5	Mediator of RNA polymerase II transcription subunit 17 OS=Mus musculus OX=10090 GN=Med17 P	1.076666667
Abhd11	Q8K4F5	Protein ABHD11 OS=Mus musculus OX=10090 GN=Abhd11 PE=1 SV=1	0.816666667
Dtd1	Q9DD18	D-aminoacyl-tRNA deacylase 1 OS=Mus musculus OX=10090 GN=Dtd1 PE=1 SV=2	1.096666667
Wdr91	Q7TMQ7	WD repeat-containing protein 91 OS=Mus musculus OX=10090 GN=Wdr91 PE=1 SV=1	1.306666667
Atp6v0c	P63082	V-type proton ATPase 16 kDa proteolipid subunit OS=Mus musculus OX=10090 GN=Atp6v0c PE=1 S	0.84
Paxip1	Q6NZQ4	PAX-interacting protein 1 OS=Mus musculus OX=10090 GN=Paxip1 PE=1 SV=1	1.113333333
Carnmt1	Q80UY1	Carnosine N-methyltransferase OS=Mus musculus OX=10090 GN=Carnmt1 PE=1 SV=1	1.013333333
Rchy1	Q9CR50	RING finger and CHY zinc finger domain-containing protein 1 OS=Mus musculus OX=10090 GN=Rchy	
Sprr1a	Q62266	Cornifin-A OS=Mus musculus OX=10090 GN=Sprr1a PE=1 SV=1	1.213333333
Rad51	Q08297	DNA repair protein RAD51 homolog 1 OS=Mus musculus OX=10090 GN=Rad51 PE=1 SV=1	1.596666667
D10Wsu102e	Q9CX66	Uncharacterized protein C12orf45 homolog OS=Mus musculus OX=10090 GN=D10Wsu102e PE=1 S	0.87
B2m	P01887	Beta-2-microglobulin OS=Mus musculus OX=10090 GN=B2m PE=1 SV=2	0.966666667
Becn1	O88597	Beclin-1 OS=Mus musculus OX=10090 GN=Becn1 PE=1 SV=3	1.12
Dad1	P61804	Dolichyl-diphosphooligosaccharide--protein glycosyltransferase subunit DAD1 OS=Mus musculus O	0.9
Syvn1	Q9DBY1	E3 ubiquitin-protein ligase synoviolin OS=Mus musculus OX=10090 GN=Syvn1 PE=1 SV=3	0.69
Vtn	P29788	Vitronectin OS=Mus musculus OX=10090 GN=Vtn PE=1 SV=2	0.23
Riox2	Q8CD15	Ribosomal oxygenase 2 OS=Mus musculus OX=10090 GN=Riox2 PE=1 SV=2	1.126666667
Dph2	Q9CR25	2-(3-amino-3-carboxypropyl)histidine synthase subunit 2 OS=Mus musculus OX=10090 GN=Dph2 P	0.76
Cd248	Q91V98	Endosialin OS=Mus musculus OX=10090 GN=Cd248 PE=1 SV=1	1.526666667
Dmap1	Q9JI44	DNA methyltransferase 1-associated protein 1 OS=Mus musculus OX=10090 GN=Dmap1 PE=1 SV=1	0.516666667
Mrps11	Q9DCA2	28S ribosomal protein S11 mitochondrial OS=Mus musculus OX=10090 GN=Mrps11 PE=1 SV=2	0.993333333
Trabd	Q99JY4	TraB domain-containing protein OS=Mus musculus OX=10090 GN=Trabd PE=1 SV=1	0.933333333
Pla2g15	Q8VEB4	Phospholipase A2 group XV OS=Mus musculus OX=10090 GN=Pla2g15 PE=1 SV=1	0.896666667
Ptcd2	Q8R3K3	Pentatricopeptide repeat-containing protein 2 mitochondrial OS=Mus musculus OX=10090 GN=Pt	1.233333333
Med8	Q9D7W5	Mediator of RNA polymerase II transcription subunit 8 OS=Mus musculus OX=10090 GN=Med8 PE=1	0.666666667

Acbd5	Q5XG73	Acyl-CoA-binding domain-containing protein 5 OS=Mus musculus OX=10090 GN=Acbd5 PE=1 SV=1	1.146666667
Thg1l	Q9CY52	Probable tRNA(His) guanylyltransferase OS=Mus musculus OX=10090 GN=Thg1l PE=1 SV=1	2.433333333
Tmem168	Q91VX9	Transmembrane protein 168 OS=Mus musculus OX=10090 GN=Tmem168 PE=1 SV=1	1.686666667
Mphosph8	Q3TYA6	M-phase phosphoprotein 8 OS=Mus musculus OX=10090 GN=Mphosph8 PE=1 SV=1	0.833333333
Micos13	Q8R404	MICOS complex subunit MIC13 OS=Mus musculus OX=10090 GN=Micos13 PE=1 SV=1	0.683333333
Klhl42	Q8BFQ9	Kelch-like protein 42 OS=Mus musculus OX=10090 GN=Klhl42 PE=2 SV=1	0.923333333
Swi5	Q8K3D3	DNA repair protein SWI5 homolog OS=Mus musculus OX=10090 GN=Swi5 PE=1 SV=1	0.806666667
Smg9	Q9DB90	Protein SMG9 OS=Mus musculus OX=10090 GN=Smg9 PE=1 SV=1	0.833333333
Trir	Q9D735	Telomerase RNA component interacting RNase OS=Mus musculus OX=10090 GN=Trir PE=1 SV=1	0.763333333
Washc3	Q9CR27	WASH complex subunit 3 OS=Mus musculus OX=10090 GN=Washc3 PE=1 SV=1	0.415
Camlg	P49070	Calcium signal-modulating cyclophilin ligand OS=Mus musculus OX=10090 GN=Camlg PE=1 SV=2	0.863333333
Vps37a	Q8CHS8	Vacuolar protein sorting-associated protein 37A OS=Mus musculus OX=10090 GN=Vps37a PE=1 SV=	0.97
Commd1	Q8K4M5	COMM domain-containing protein 1 OS=Mus musculus OX=10090 GN=Commd1 PE=1 SV=2	0.89
Rps29	P62274	40S ribosomal protein S29 OS=Mus musculus OX=10090 GN=Rps29 PE=1 SV=2	0.886666667
Atp5pf	P97450	ATP synthase-coupling factor 6 mitochondrial OS=Mus musculus OX=10090 GN=Atp5pf PE=1 SV=1	0.766666667
Zc3h13	E9Q784	Zinc finger CCCH domain-containing protein 13 OS=Mus musculus OX=10090 GN=Zc3h13 PE=1 SV=	0.893333333
Med29	Q9DB91	Mediator of RNA polymerase II transcription subunit 29 OS=Mus musculus OX=10090 GN=Med29 P	0.833333333
Synrg	Q5SV85	Synergin gamma OS=Mus musculus OX=10090 GN=Synrg PE=1 SV=1	0.873333333
Lzic	Q8K3C3	Protein LZIC OS=Mus musculus OX=10090 GN=Lzic PE=1 SV=1	1
Cuta	Q9CQ89	Protein CutA OS=Mus musculus OX=10090 GN=Cuta PE=1 SV=3	0.453333333
Cnot8	Q9D8X5	CCR4-NOT transcription complex subunit 8 OS=Mus musculus OX=10090 GN=Cnot8 PE=2 SV=1	1.2
Cdc42ep2	Q8JZX9	Cdc42 effector protein 2 OS=Mus musculus OX=10090 GN=Cdc42ep2 PE=1 SV=1	2.43
Ccdc43	Q9CR29	Coiled-coil domain-containing protein 43 OS=Mus musculus OX=10090 GN=Ccdc43 PE=1 SV=1	0.923333333
Nfkbib	Q60778	NF-kappa-B inhibitor beta OS=Mus musculus OX=10090 GN=Nfkbib PE=1 SV=2	1.07
Gstz1	Q9WVL0	Maleylacetoacetate isomerase OS=Mus musculus OX=10090 GN=Gstz1 PE=1 SV=1	1.516666667
Morc2a	Q69ZX6	ATPase MORC2A OS=Mus musculus OX=10090 GN=Morc2a PE=1 SV=2	1.01
Trmt11	Q9CWH5	tRNA (guanine(10)-N2)-methyltransferase homolog OS=Mus musculus OX=10090 GN=Trmt11 PE=1 S	1.136666667
Lamp2	P17047	Lysosome-associated membrane glycoprotein 2 OS=Mus musculus OX=10090 GN=Lamp2 PE=1 SV=2	0.873333333
Isca1	Q9D924	Iron-sulfur cluster assembly 1 homolog mitochondrial OS=Mus musculus OX=10090 GN=Isca1 PE=2	7.55
Zmym3	Q9JLM4	Zinc finger MYM-type protein 3 OS=Mus musculus OX=10090 GN=Zmym3 PE=1 SV=1	1.175
Mib1	Q80SY4	E3 ubiquitin-protein ligase MIB1 OS=Mus musculus OX=10090 GN=Mib1 PE=1 SV=1	
Akap10	O88845	A-kinase anchor protein 10 mitochondrial OS=Mus musculus OX=10090 GN=Akap10 PE=1 SV=3	1.48
Snrnp27	Q8K194	U4/U6.U5 small nuclear ribonucleoprotein 27 kDa protein OS=Mus musculus OX=10090 GN=Snrnp	0.623333333
Fam32a	Q9CR80	Protein FAM32A OS=Mus musculus OX=10090 GN=Fam32a PE=2 SV=1	1.006666667

Mrpl18	Q9CQL5	39S ribosomal protein L18 mitochondrial OS=Mus musculus OX=10090 GN=Mrpl18 PE=1 SV=1	0.616666667
Lipa	Q9Z0M5	Lysosomal acid lipase/cholesteryl ester hydrolase OS=Mus musculus OX=10090 GN=Lipa PE=1 SV=2	0.69
	Q9CYI0	Protein Njmu-R1 OS=Mus musculus OX=10090 PE=1 SV=2	0.583333333
Znhit6	Q3UFB2	Box C/D snoRNA protein 1 OS=Mus musculus OX=10090 GN=Znhit6 PE=1 SV=2	0.95
Nfyc	P70353	Nuclear transcription factor Y subunit gamma OS=Mus musculus OX=10090 GN=Nfyc PE=1 SV=2	1.305
Pan2	Q8BGF7	PAN2-PAN3 deadenylation complex catalytic subunit Pan2 OS=Mus musculus OX=10090 GN=Pan2 I	2.13
Tmco1	Q921L3	Calcium load-activated calcium channel OS=Mus musculus OX=10090 GN=Tmco1 PE=1 SV=1	0.846666667
Arih2	Q9Z1K6	E3 ubiquitin-protein ligase ARIH2 OS=Mus musculus OX=10090 GN=Arih2 PE=1 SV=1	0.506666667
Gltp	Q9JL62	Glycolipid transfer protein OS=Mus musculus OX=10090 GN=Gltp PE=1 SV=3	44.96
Agpat1	O35083	1-acyl-sn-glycerol-3-phosphate acyltransferase alpha OS=Mus musculus OX=10090 GN=Agpat1 PE=1	0.996666667
Ebp	P70245	3-beta-hydroxysteroid-Delta(8) Delta(7)-isomerase OS=Mus musculus OX=10090 GN=Ebp PE=1 SV=3	0.583333333
Fosl2	P47930	Fos-related antigen 2 OS=Mus musculus OX=10090 GN=Fosl2 PE=1 SV=2	1.22
Mcrip1	Q3UGS4	Mapk-regulated corepressor-interacting protein 1 OS=Mus musculus OX=10090 GN=Mcrip1 PE=1 S\	0.9
Mrpl14	Q9D1I6	39S ribosomal protein L14 mitochondrial OS=Mus musculus OX=10090 GN=Mrpl14 PE=1 SV=1	0.83
Polr3b	P59470	DNA-directed RNA polymerase III subunit RPC2 OS=Mus musculus OX=10090 GN=Polr3b PE=1 SV=2	0.936666667
Cdca5	Q9CPY3	Sororin OS=Mus musculus OX=10090 GN=Cdca5 PE=1 SV=1	0.893333333
	Q99M08	Uncharacterized protein C4orf3 homolog OS=Mus musculus OX=10090 PE=1 SV=1	1.033333333
Polr2j	O08740	DNA-directed RNA polymerase II subunit RPB11 OS=Mus musculus OX=10090 GN=Polr2j PE=1 SV=1	6.123333333
Spast	Q9QYY8	Spastin OS=Mus musculus OX=10090 GN=Spast PE=1 SV=3	1.776666667
Smyd2	Q8R5A0	N-lysine methyltransferase SMYD2 OS=Mus musculus OX=10090 GN=Smyd2 PE=1 SV=1	1.5
Palm2	Q8BR92	Paralemmin-2 OS=Mus musculus OX=10090 GN=Palm2 PE=1 SV=1	2.46
Kdsr	Q6GV12	3-ketodihydrosphingosine reductase OS=Mus musculus OX=10090 GN=Kdsr PE=1 SV=1	1.16
Atg101	Q9D8Z6	Autophagy-related protein 101 OS=Mus musculus OX=10090 GN=Atg101 PE=1 SV=1	0.676666667
Derl2	Q8BNI4	Derlin-2 OS=Mus musculus OX=10090 GN=Derl2 PE=1 SV=2	0.843333333
Rnf7	Q9WTZ1	RING-box protein 2 OS=Mus musculus OX=10090 GN=Rnf7 PE=1 SV=1	1.06
Wipf1	Q8K1I7	WAS/WASL-interacting protein family member 1 OS=Mus musculus OX=10090 GN=Wipf1 PE=1 SV=1	1.91
Klhl11	Q8CE33	Kelch-like protein 11 OS=Mus musculus OX=10090 GN=Klhl11 PE=1 SV=1	1.46
Fdxr	Q61578	NADPH:adrenodoxin oxidoreductase mitochondrial OS=Mus musculus OX=10090 GN=Fdxr PE=1 S\	1.14
Tmf1	B9EKI3	TATA element modulatory factor OS=Mus musculus OX=10090 GN=Tmf1 PE=1 SV=2	1.093333333
Lyplal1	Q3UFF7	Lysophospholipase-like protein 1 OS=Mus musculus OX=10090 GN=Lyplal1 PE=1 SV=3	0.85
Vps33b	P59016	Vacuolar protein sorting-associated protein 33B OS=Mus musculus OX=10090 GN=Vps33b PE=1 SV=	1.09
Mrpl54	Q9CPW3	39S ribosomal protein L54 mitochondrial OS=Mus musculus OX=10090 GN=Mrpl54 PE=1 SV=1	0.613333333
Bckdha	P50136	2-oxoisovalerate dehydrogenase subunit alpha mitochondrial OS=Mus musculus OX=10090 GN=Bc	1.273333333
Rpl34	Q9D1R9	60S ribosomal protein L34 OS=Mus musculus OX=10090 GN=Rpl34 PE=1 SV=2	0.836666667

Ttf2	Q5NC05	Transcription termination factor 2 OS=Mus musculus OX=10090 GN=Ttf2 PE=1 SV=2	0.7
Nudt3	Q9JI46	Diphosphoinositol polyphosphate phosphohydrolase 1 OS=Mus musculus OX=10090 GN=Nudt3 PE	0.9
Emc4	Q9CZX9	ER membrane protein complex subunit 4 OS=Mus musculus OX=10090 GN=Emc4 PE=1 SV=1	0.976666667
Mgp	P19788	Matrix Gla protein OS=Mus musculus OX=10090 GN=Mgp PE=3 SV=1	1.126666667
Fbn1	Q61554	Fibrillin-1 OS=Mus musculus OX=10090 GN=Fbn1 PE=1 SV=2	1.346666667
Arl6	O88848	ADP-ribosylation factor-like protein 6 OS=Mus musculus OX=10090 GN=Arl6 PE=1 SV=1	0.87
Wdr45	Q91VM3	WD repeat domain phosphoinositide-interacting protein 4 OS=Mus musculus OX=10090 GN=Wdr4	1.003333333
Pias4	Q9JM05	E3 SUMO-protein ligase PIAS4 OS=Mus musculus OX=10090 GN=Pias4 PE=1 SV=2	0.755
Mrpl58	Q8R035	Peptidyl-tRNA hydrolase ICT1 mitochondrial OS=Mus musculus OX=10090 GN=Mrpl58 PE=1 SV=1	0.99
Pcyt2	Q922E4	Ethanolamine-phosphate cytidylyltransferase OS=Mus musculus OX=10090 GN=Pcyt2 PE=1 SV=1	0.763333333
Stard13	Q923Q2	StAR-related lipid transfer protein 13 OS=Mus musculus OX=10090 GN=Stard13 PE=1 SV=5	0.523333333
Spout1	Q3UHX9	Putative methyltransferase C9orf114 homolog OS=Mus musculus OX=10090 GN=Spout1 PE=1 SV=1	0.626666667
Snrpg	P62309	Small nuclear ribonucleoprotein G OS=Mus musculus OX=10090 GN=Snrpg PE=1 SV=1	0.986666667
Cd14	P10810	Monocyte differentiation antigen CD14 OS=Mus musculus OX=10090 GN=Cd14 PE=1 SV=1	0.456666667
Soga1	E1U8D0	Protein SOGA1 OS=Mus musculus OX=10090 GN=Soga1 PE=1 SV=3	1.1
Fcf1	Q9CTH6	rRNA-processing protein FCF1 homolog OS=Mus musculus OX=10090 GN=Fcf1 PE=2 SV=2	0.81
Tle5	P63002	TLE family member 5 OS=Mus musculus OX=10090 GN=Tle5 PE=1 SV=1	0.693333333
Cdkn2a	P51480	Cyclin-dependent kinase inhibitor 2A OS=Mus musculus OX=10090 GN=Cdkn2a PE=1 SV=2	1.566666667
Slit3	Q9WVB4	Slit homolog 3 protein OS=Mus musculus OX=10090 GN=Slit3 PE=2 SV=2	1.853333333
Suds3	Q8BR65	Sin3 histone deacetylase corepressor complex component SDS3 OS=Mus musculus OX=10090 GN=S	1.17
Map3k4	O08648	Mitogen-activated protein kinase kinase kinase 4 OS=Mus musculus OX=10090 GN=Map3k4 PE=1 SV	0.936666667
Parl	Q5XJY4	Presenilins-associated rhomboid-like protein mitochondrial OS=Mus musculus OX=10090 GN=Parl	0.823333333
Foxg1	Q60987	Forkhead box protein G1 OS=Mus musculus OX=10090 GN=Foxg1 PE=1 SV=1	0.813333333
Ccdc127	Q3TC33	Coiled-coil domain-containing protein 127 OS=Mus musculus OX=10090 GN=Ccdc127 PE=1 SV=2	1.96
Ggct	Q9D7X8	Gamma-glutamylcyclotransferase OS=Mus musculus OX=10090 GN=Ggct PE=1 SV=1	0.883333333
Keap1	Q9Z2X8	Kelch-like ECH-associated protein 1 OS=Mus musculus OX=10090 GN=Keap1 PE=1 SV=1	0.665
Dcun1d5	Q9CXV9	DCN1-like protein 5 OS=Mus musculus OX=10090 GN=Dcun1d5 PE=1 SV=1	1.16
Nceh1	Q8BLF1	Neutral cholesterol ester hydrolase 1 OS=Mus musculus OX=10090 GN=Nceh1 PE=1 SV=1	1.363333333
Myo10	F8VQB6	Unconventional myosin-X OS=Mus musculus OX=10090 GN=Myo10 PE=1 SV=1	1.595
Med27	Q9DB40	Mediator of RNA polymerase II transcription subunit 27 OS=Mus musculus OX=10090 GN=Med27 PI	1.52
Unc5b	Q8K1S3	Netrin receptor UNC5B OS=Mus musculus OX=10090 GN=Unc5b PE=1 SV=1	1.016666667
Kct2	Q8K201	Keratinocyte-associated transmembrane protein 2 OS=Mus musculus OX=10090 GN=Kct2 PE=1 SV=:	0.923333333
Cdr2	P97817	Cerebellar degeneration-related protein 2 OS=Mus musculus OX=10090 GN=Cdr2 PE=1 SV=2	0.77
Kiaa1671	Q8BRV5	Uncharacterized protein KIAA1671 OS=Mus musculus OX=10090 GN=Kiaa1671 PE=2 SV=1	1.29

Tmem115	Q9WUH1	Transmembrane protein 115 OS=Mus musculus OX=10090 GN=Tmem115 PE=1 SV=1	1.12
Chuk	Q60680	Inhibitor of nuclear factor kappa-B kinase subunit alpha OS=Mus musculus OX=10090 GN=Chuk PE=	1.115
Commd2	Q8BXC6	COMM domain-containing protein 2 OS=Mus musculus OX=10090 GN=Commd2 PE=1 SV=1	0.95
Xrcc4	Q924T3	DNA repair protein XRCC4 OS=Mus musculus OX=10090 GN=Xrcc4 PE=1 SV=1	0.766666667
Mnd1	Q8K396	Meiotic nuclear division protein 1 homolog OS=Mus musculus OX=10090 GN=Mnd1 PE=1 SV=1	0.46
Vkorc1l1	Q6TEK5	Vitamin K epoxide reductase complex subunit 1-like protein 1 OS=Mus musculus OX=10090 GN=Vk	0.853333333
Slc25a30	Q9CR58	Kidney mitochondrial carrier protein 1 OS=Mus musculus OX=10090 GN=Slc25a30 PE=1 SV=1	0.98
Rtf2	Q99K95	Replication termination factor 2 OS=Mus musculus OX=10090 GN=Rtf2 PE=1 SV=1	0.796666667
Uhrf1bp1l	A2RSJ4	UHRF1-binding protein 1-like OS=Mus musculus OX=10090 GN=Uhrf1bp1l PE=1 SV=2	
Sema3a	O08665	Semaphorin-3A OS=Mus musculus OX=10090 GN=Sema3a PE=1 SV=2	1.14
Plg	P20918	Plasminogen OS=Mus musculus OX=10090 GN=Plg PE=1 SV=3	0.263333333
Riox1	Q9JJF3	Ribosomal oxygenase 1 OS=Mus musculus OX=10090 GN=Riox1 PE=1 SV=2	0.586666667
Iqsec1	Q8R0S2	IQ motif and SEC7 domain-containing protein 1 OS=Mus musculus OX=10090 GN=Iqsec1 PE=1 SV=2	0.843333333
Arhgef12	Q8R4H2	Rho guanine nucleotide exchange factor 12 OS=Mus musculus OX=10090 GN=Arhgef12 PE=1 SV=2	1.57
Eri3	Q8C460	ERI1 exoribonuclease 3 OS=Mus musculus OX=10090 GN=Eri3 PE=1 SV=1	0.82
Taf5l	Q91WQ5	TAF5-like RNA polymerase II p300/CBP-associated factor-associated factor 65 kDa subunit 5L OS=Mu	1.21
Fnbp1l	Q8K012	Formin-binding protein 1-like OS=Mus musculus OX=10090 GN=Fnbp1l PE=1 SV=2	0.696666667
Leo1	Q5XJE5	RNA polymerase-associated protein LEO1 OS=Mus musculus OX=10090 GN=Leo1 PE=1 SV=2	0.636666667
Med9	Q8VCS6	Mediator of RNA polymerase II transcription subunit 9 OS=Mus musculus OX=10090 GN=Med9 PE=2	0.93
Coq7	P97478	5-demethoxyubiquinone hydroxylase mitochondrial OS=Mus musculus OX=10090 GN=Coq7 PE=1 S	0.703333333
Mrps12	O35680	28S ribosomal protein S12 mitochondrial OS=Mus musculus OX=10090 GN=Mrps12 PE=2 SV=1	1.265
Rab3il1	Q8VDV3	Guanine nucleotide exchange factor for Rab-3A OS=Mus musculus OX=10090 GN=Rab3il1 PE=1 SV=1	1.06
Yipf3	Q3UDR8	Protein YIPF3 OS=Mus musculus OX=10090 GN=Yipf3 PE=1 SV=1	0.725
Adat3	Q6PAT0	Probable inactive tRNA-specific adenosine deaminase-like protein 3 OS=Mus musculus OX=10090 G	1.396666667
Coa3	Q9D2R6	Cytochrome c oxidase assembly factor 3 homolog mitochondrial OS=Mus musculus OX=10090 GN=	0.896666667
Mrps18c	Q8R2L5	28S ribosomal protein S18c mitochondrial OS=Mus musculus OX=10090 GN=Mrps18c PE=1 SV=1	0.915
Cbr2	P08074	Carbonyl reductase [NADPH] 2 OS=Mus musculus OX=10090 GN=Cbr2 PE=1 SV=1	1.24
Nde1	Q9CZA6	Nuclear distribution protein nudE homolog 1 OS=Mus musculus OX=10090 GN=Nde1 PE=1 SV=1	1.916666667
Siae	P70665	Sialate O-acetylesterase OS=Mus musculus OX=10090 GN=Siae PE=1 SV=3	0.923333333
Cmas	Q99KK2	N-acylneuraminate cytidylyltransferase OS=Mus musculus OX=10090 GN=Cmas PE=1 SV=2	1.18
Nrp2	O35375	Neuropilin-2 OS=Mus musculus OX=10090 GN=Nrp2 PE=1 SV=2	0.535
Szrd1	Q6NXN1	SUZ domain-containing protein 1 OS=Mus musculus OX=10090 GN=Szrd1 PE=1 SV=1	1.283333333
Vmp1	Q99KU0	Vacuole membrane protein 1 OS=Mus musculus OX=10090 GN=Vmp1 PE=1 SV=2	0.95
L2hgdh	Q91YP0	L-2-hydroxyglutarate dehydrogenase mitochondrial OS=Mus musculus OX=10090 GN=L2hgdh PE=1	0.893333333

Etnk1	Q9D4V0	Ethanolamine kinase 1 OS=Mus musculus OX=10090 GN=Etnk1 PE=1 SV=2	0.703333333
Mrpl28	Q9D1B9	39S ribosomal protein L28 mitochondrial OS=Mus musculus OX=10090 GN=Mrpl28 PE=1 SV=3	1.126666667
Ndufa13	Q9ERS2	NADH dehydrogenase [ubiquinone] 1 alpha subcomplex subunit 13 OS=Mus musculus OX=10090 GI	0.943333333
Ppt2	O35448	Lysosomal thioesterase PPT2 OS=Mus musculus OX=10090 GN=Ppt2 PE=1 SV=1	1.186666667
Ints14	Q8R3P6	Integrator complex subunit 14 OS=Mus musculus OX=10090 GN=Ints14 PE=1 SV=1	0.666666667
Tmem97	Q8VD00	Sigma intracellular receptor 2 OS=Mus musculus OX=10090 GN=Tmem97 PE=1 SV=1	0.646666667
Polr3f	Q921X6	DNA-directed RNA polymerase III subunit RPC6 OS=Mus musculus OX=10090 GN=Polr3f PE=1 SV=1	1.293333333
Glmp	Q9JHJ3	Glycosylated lysosomal membrane protein OS=Mus musculus OX=10090 GN=Glmp PE=1 SV=1	0.973333333
Ndufa2	Q9CQ75	NADH dehydrogenase [ubiquinone] 1 alpha subcomplex subunit 2 OS=Mus musculus OX=10090 GN	0.926666667
Aars2	Q14CH7	Alanine--tRNA ligase mitochondrial OS=Mus musculus OX=10090 GN=Aars2 PE=1 SV=1	1.626666667
Arfip2	Q8K221	Arfaptin-2 OS=Mus musculus OX=10090 GN=Arfip2 PE=1 SV=2	1.696666667
Hspb8	Q9JK92	Heat shock protein beta-8 OS=Mus musculus OX=10090 GN=Hspb8 PE=1 SV=1	0.856666667
Fam107b	Q3TGF2	Protein FAM107B OS=Mus musculus OX=10090 GN=Fam107b PE=1 SV=2	1.3
Maip1	Q8BHE8	m-AAA protease-interacting protein 1 mitochondrial OS=Mus musculus OX=10090 GN=Maip1 PE=1	0.973333333
Lpar1	P61793	Lysophosphatidic acid receptor 1 OS=Mus musculus OX=10090 GN=Lpar1 PE=1 SV=1	1.05
Cd82	P40237	CD82 antigen OS=Mus musculus OX=10090 GN=Cd82 PE=1 SV=1	1.153333333
Wdsub1	Q9D0I6	WD repeat SAM and U-box domain-containing protein 1 OS=Mus musculus OX=10090 GN=Wdsub1	1.29
Commd4	Q9CQ02	COMM domain-containing protein 4 OS=Mus musculus OX=10090 GN=Commd4 PE=1 SV=1	1.976666667
Elovl6	Q920L5	Elongation of very long chain fatty acids protein 6 OS=Mus musculus OX=10090 GN=Elovl6 PE=1 SV=	1.446666667
Arnt	P53762	Aryl hydrocarbon receptor nuclear translocator OS=Mus musculus OX=10090 GN=Arnt PE=1 SV=3	
Gpalpp1	Q69ZC8	GPALPP motifs-containing protein 1 OS=Mus musculus OX=10090 GN=Gpalpp1 PE=1 SV=2	
Ankrd46	Q8BTZ5	Ankyrin repeat domain-containing protein 46 OS=Mus musculus OX=10090 GN=Ankrd46 PE=1 SV=1	1.246666667
Casp12	O08736	Caspase-12 OS=Mus musculus OX=10090 GN=Casp12 PE=1 SV=1	8.74
Haus8	Q99L00	HAUS augmin-like complex subunit 8 OS=Mus musculus OX=10090 GN=Haus8 PE=1 SV=2	0.406666667
Hspa12b	Q9CZJ2	Heat shock 70 kDa protein 12B OS=Mus musculus OX=10090 GN=Hspa12b PE=1 SV=1	1.11
Hltf	Q6PCN7	Helicase-like transcription factor OS=Mus musculus OX=10090 GN=Hltf PE=1 SV=1	4.925
Coro7	Q9D2V7	Coronin-7 OS=Mus musculus OX=10090 GN=Coro7 PE=1 SV=2	1.326666667
Bmt2	Q8BXK4	S-adenosylmethionine sensor upstream of mTORC1 OS=Mus musculus OX=10090 GN=Bmt2 PE=2 SV	1.08
Med19	Q8C1S0	Mediator of RNA polymerase II transcription subunit 19 OS=Mus musculus OX=10090 GN=Med19 PI	1.05
Cox6c	Q9CPQ1	Cytochrome c oxidase subunit 6C OS=Mus musculus OX=10090 GN=Cox6c PE=1 SV=3	0.845
Cnot4	Q8BT14	CCR4-NOT transcription complex subunit 4 OS=Mus musculus OX=10090 GN=Cnot4 PE=1 SV=2	
Eogt	Q8BYW9	EGF domain-specific O-linked N-acetylglucosamine transferase OS=Mus musculus OX=10090 GN=Eo	0.5
Med7	Q9CZB6	Mediator of RNA polymerase II transcription subunit 7 OS=Mus musculus OX=10090 GN=Med7 PE=1	2.08
Rnf14	Q9JI90	E3 ubiquitin-protein ligase RNF14 OS=Mus musculus OX=10090 GN=Rnf14 PE=1 SV=2	1.34

Gvin1	Q80SU7	Interferon-induced very large GTPase 1 OS=Mus musculus OX=10090 GN=Gvin1 PE=1 SV=1	0.42
Dynll1	P63168	Dynein light chain 1 cytoplasmic OS=Mus musculus OX=10090 GN=Dynll1 PE=1 SV=1	0.903333333
Dynll2	Q9D0M5	Dynein light chain 2 cytoplasmic OS=Mus musculus OX=10090 GN=Dynll2 PE=1 SV=1	1.01
Rab3b	Q9CZT8	Ras-related protein Rab-3B OS=Mus musculus OX=10090 GN=Rab3b PE=1 SV=1	0.495
Naa11	Q3UX61	N-alpha-acetyltransferase 11 OS=Mus musculus OX=10090 GN=Naa11 PE=1 SV=1	
Stk25	Q9Z2W1	Serine/threonine-protein kinase 25 OS=Mus musculus OX=10090 GN=Stk25 PE=1 SV=2	
Krt5	Q922U2	Keratin type II cytoskeletal 5 OS=Mus musculus OX=10090 GN=Krt5 PE=1 SV=1	0
Smad5	P97454	Mothers against decapentaplegic homolog 5 OS=Mus musculus OX=10090 GN=Smad5 PE=1 SV=2	0.786666667
Ube2e1	P52482	Ubiquitin-conjugating enzyme E2 E1 OS=Mus musculus OX=10090 GN=Ube2e1 PE=1 SV=1	3.15
Ugt1a7c	Q6ZQM8	UDP-glucuronosyltransferase 1A7 OS=Mus musculus OX=10090 GN=Ugt1a7 PE=1 SV=1	
Akr1b7	P21300	Aldo-keto reductase family 1 member B7 OS=Mus musculus OX=10090 GN=Akr1b7 PE=1 SV=4	0.76
Krt79	Q8VED5	Keratin type II cytoskeletal 79 OS=Mus musculus OX=10090 GN=Krt79 PE=1 SV=2	0.003333333
Chd2	E9PZM4	Chromodomain-helicase-DNA-binding protein 2 OS=Mus musculus OX=10090 GN=Chd2 PE=1 SV=1	1.065
Plxna2	P70207	Plexin-A2 OS=Mus musculus OX=10090 GN=Plxna2 PE=1 SV=2	
Abi2	P62484	Abl interactor 2 OS=Mus musculus OX=10090 GN=Abi2 PE=1 SV=1	1.01
Smarcd3	Q6P9Z1	SWI/SNF-related matrix-associated actin-dependent regulator of chromatin subfamily D member 3 (	1.47
Igf1r	Q60751	Insulin-like growth factor 1 receptor OS=Mus musculus OX=10090 GN=Igf1r PE=1 SV=3	0.996666667
Scd1	P13516	Acyl-CoA desaturase 1 OS=Mus musculus OX=10090 GN=Scd1 PE=1 SV=2	0.983333333
Eif1ax	Q8BMJ3	Eukaryotic translation initiation factor 1A X-chromosomal OS=Mus musculus OX=10090 GN=Eif1ax	0.856666667
Srpk2	O54781	SRSF protein kinase 2 OS=Mus musculus OX=10090 GN=Srpk2 PE=1 SV=2	1.01
Erlin1	Q91X78	Erlin-1 OS=Mus musculus OX=10090 GN=Erlin1 PE=1 SV=2	0.673333333
Pank3	Q8R2W9	Pantothenate kinase 3 OS=Mus musculus OX=10090 GN=Pank3 PE=2 SV=1	0.89
Rab4a	P56371	Ras-related protein Rab-4A OS=Mus musculus OX=10090 GN=Rab4a PE=1 SV=2	0.683333333
Ring1	O35730	E3 ubiquitin-protein ligase RING1 OS=Mus musculus OX=10090 GN=Ring1 PE=1 SV=2	0.483333333
Ppp1r12b	Q8BG95	Protein phosphatase 1 regulatory subunit 12B OS=Mus musculus OX=10090 GN=Ppp1r12b PE=1 SV	0.686666667
Slc12a7	Q9WVL3	Solute carrier family 12 member 7 OS=Mus musculus OX=10090 GN=Slc12a7 PE=1 SV=1	1.01
Ptpn14	Q62130	Tyrosine-protein phosphatase non-receptor type 14 OS=Mus musculus OX=10090 GN=Ptpn14 PE=1	0.605
Armcx3	Q8BHS6	Armadillo repeat-containing X-linked protein 3 OS=Mus musculus OX=10090 GN=Armcx3 PE=1 SV=1	1.293333333
Ifitm3	Q9CQW9	Interferon-induced transmembrane protein 3 OS=Mus musculus OX=10090 GN=Ifitm3 PE=1 SV=1	1.066666667
Prrx1	P63013	Paired mesoderm homeobox protein 1 OS=Mus musculus OX=10090 GN=Prrx1 PE=1 SV=1	0.913333333
Parvb	Q9ES46	Beta-parvin OS=Mus musculus OX=10090 GN=Parvb PE=1 SV=1	0.966666667
Aurka	P97477	Aurora kinase A OS=Mus musculus OX=10090 GN=Aurka PE=1 SV=1	1.95
Cenpe	Q6RT24	Centromere-associated protein E OS=Mus musculus OX=10090 GN=Cenpe PE=1 SV=1	1.236666667
Mapre2	Q8R001	Microtubule-associated protein RP/EB family member 2 OS=Mus musculus OX=10090 GN=Mapre2 F	0.783333333

Znf22	Q9ERU3	Zinc finger protein 22 OS=Mus musculus OX=10090 GN=Znf22 PE=1 SV=2	1.276666667
Lsm1	Q8VC85	U6 snRNA-associated Sm-like protein LSm1 OS=Mus musculus OX=10090 GN=Lsm1 PE=1 SV=1	1.29
Rab33b	O35963	Ras-related protein Rab-33B OS=Mus musculus OX=10090 GN=Rab33b PE=1 SV=1	1.456666667
Agap3	Q8VHH5	Arf-GAP with GTPase ANK repeat and PH domain-containing protein 3 OS=Mus musculus OX=1009C	1.483333333
Rcbtb2	Q99LJ7	RCC1 and BTB domain-containing protein 2 OS=Mus musculus OX=10090 GN=Rcbtb2 PE=1 SV=1	0.923333333
Akr1e2	Q9DCT1	1 5-anhydro-D-fructose reductase OS=Mus musculus OX=10090 GN=Akr1e2 PE=1 SV=1	1.066666667
Tarsl2	Q8BLY2	Threonine--tRNA ligase 2 cytoplasmic OS=Mus musculus OX=10090 GN=Tars3 PE=1 SV=1	1.615
Dhx57	Q6P5D3	Putative ATP-dependent RNA helicase DHX57 OS=Mus musculus OX=10090 GN=Dhx57 PE=1 SV=2	0.913333333
Gna13	P27601	Guanine nucleotide-binding protein subunit alpha-13 OS=Mus musculus OX=10090 GN=Gna13 PE=	0.776666667
Agap1	Q8BXK8	Arf-GAP with GTPase ANK repeat and PH domain-containing protein 1 OS=Mus musculus OX=1009C	1.706666667
Tcf12	Q61286	Transcription factor 12 OS=Mus musculus OX=10090 GN=Tcf12 PE=1 SV=2	0.876666667
Hcfc2	Q9D968	Host cell factor 2 OS=Mus musculus OX=10090 GN=Hcfc2 PE=1 SV=2	1.14
Parp2	O88554	Poly [ADP-ribose] polymerase 2 OS=Mus musculus OX=10090 GN=Parp2 PE=1 SV=3	0.826666667
Ipo13	Q8K0C1	Importin-13 OS=Mus musculus OX=10090 GN=Ipo13 PE=1 SV=1	1.233333333
Papss2	O88428	Bifunctional 3'-phosphoadenosine 5'-phosphosulfate synthase 2 OS=Mus musculus OX=10090 GN=F	2.323333333
Gabarapl2	P60521	Gamma-aminobutyric acid receptor-associated protein-like 2 OS=Mus musculus OX=10090 GN=Gak	1.11
Bcl7c	O08664	B-cell CLL/lymphoma 7 protein family member C OS=Mus musculus OX=10090 GN=Bcl7c PE=1 SV=1	1.213333333
Pip4p1	Q3TWL2	Type 1 phosphatidylinositol 4 5-bisphosphate 4-phosphatase OS=Mus musculus OX=10090 GN=Pip	0.646666667
Tsc22d4	Q9EQN3	TSC22 domain family protein 4 OS=Mus musculus OX=10090 GN=Tsc22d4 PE=1 SV=2	0.65
Hirip3	Q8BLH7	HIRA-interacting protein 3 OS=Mus musculus OX=10090 GN=Hirip3 PE=1 SV=1	0.903333333
Dynlt1	P51807	Dynein light chain Tctex-type 1 OS=Mus musculus OX=10090 GN=Dynlt1 PE=1 SV=1	1.033333333
Rps26	P62855	40S ribosomal protein S26 OS=Mus musculus OX=10090 GN=Rps26 PE=1 SV=3	0.86
Lamtor5	Q9D1L9	Ragulator complex protein LAMTOR5 OS=Mus musculus OX=10090 GN=Lamtor5 PE=1 SV=1	0.903333333
Erh	P84089	Enhancer of rudimentary homolog OS=Mus musculus OX=10090 GN=Erh PE=1 SV=1	0.856666667
Psmd10	Q9Z2X2	26S proteasome non-ATPase regulatory subunit 10 OS=Mus musculus OX=10090 GN=Psmd10 PE=1	0.87
Ptma	P26350	Prothymosin alpha OS=Mus musculus OX=10090 GN=Ptma PE=1 SV=2	0.873333333
Rpl7l1	Q9D8M4	60S ribosomal protein L7-like 1 OS=Mus musculus OX=10090 GN=Rpl7l1 PE=1 SV=1	0.88
Commd3	Q63829	COMM domain-containing protein 3 OS=Mus musculus OX=10090 GN=Commd3 PE=1 SV=1	0.95
Rps21	Q9CQR2	40S ribosomal protein S21 OS=Mus musculus OX=10090 GN=Rps21 PE=1 SV=1	0.946666667
Trmt112	Q9DCG9	Multifunctional methyltransferase subunit TRM112-like protein OS=Mus musculus OX=10090 GN=T	0.853333333
Cnpy3	Q9DAU1	Protein canopy homolog 3 OS=Mus musculus OX=10090 GN=Cnpy3 PE=1 SV=1	0.933333333
Rictor	Q6QI06	Rapamycin-insensitive companion of mTOR OS=Mus musculus OX=10090 GN=Rictor PE=1 SV=2	1.456666667
Tfdp1	Q08639	Transcription factor Dp-1 OS=Mus musculus OX=10090 GN=Tfdp1 PE=1 SV=1	0.83
Dynlrb1	P62627	Dynein light chain roadblock-type 1 OS=Mus musculus OX=10090 GN=Dynlrb1 PE=1 SV=3	0.843333333

Suz12	Q80U70	Polycomb protein Suz12 OS=Mus musculus OX=10090 GN=Suz12 PE=1 SV=2	0.886666667
Rcor1	Q8CFE3	REST corepressor 1 OS=Mus musculus OX=10090 GN=Rcor1 PE=1 SV=3	0.873333333
Surf2	P09926	Surfeit locus protein 2 OS=Mus musculus OX=10090 GN=Surf2 PE=1 SV=1	0.986666667
Grcc10	O35127	Protein C10 OS=Mus musculus OX=10090 GN=Grcc10 PE=1 SV=1	1.563333333
Mettl2	Q8BMK1	tRNA N(3)-methylcytidine methyltransferase METTL2 OS=Mus musculus OX=10090 GN=Mettl2 PE=1	1
Snrpd1	P62315	Small nuclear ribonucleoprotein Sm D1 OS=Mus musculus OX=10090 GN=Snrpd1 PE=1 SV=1	0.8
Nom1	Q3UFM5	Nucleolar MIF4G domain-containing protein 1 OS=Mus musculus OX=10090 GN=Nom1 PE=1 SV=2	1.07
Tmem165	P52875	Transmembrane protein 165 OS=Mus musculus OX=10090 GN=Tmem165 PE=1 SV=2	0.78
Piezo1	E2JF22	Piezo-type mechanosensitive ion channel component 1 OS=Mus musculus OX=10090 GN=Piezo1 PE	0.79
Gtse1	Q8R080	G2 and S phase-expressed protein 1 OS=Mus musculus OX=10090 GN=Gtse1 PE=1 SV=2	0.763333333
Mxra7	Q9CZH7	Matrix-remodeling-associated protein 7 OS=Mus musculus OX=10090 GN=Mxra7 PE=1 SV=2	0.926666667
Etv6	P97360	Transcription factor ETV6 OS=Mus musculus OX=10090 GN=Etv6 PE=1 SV=1	1.01
Cdk5rap3	Q99LM2	CDK5 regulatory subunit-associated protein 3 OS=Mus musculus OX=10090 GN=Cdk5rap3 PE=1 SV=	0.593333333
Scarb1	Q61009	Scavenger receptor class B member 1 OS=Mus musculus OX=10090 GN=Scarb1 PE=1 SV=1	0.856666667
Xdh	Q00519	Xanthine dehydrogenase/oxidase OS=Mus musculus OX=10090 GN=Xdh PE=1 SV=5	1.096666667
Ndufa10	Q99LC3	NADH dehydrogenase [ubiquinone] 1 alpha subcomplex subunit 10 mitochondrial OS=Mus muscul	0.833333333
Uprt	B1AVZ0	Uracil phosphoribosyltransferase homolog OS=Mus musculus OX=10090 GN=Uprt PE=1 SV=1	0.93
Asf1a	Q9CQE6	Histone chaperone ASF1A OS=Mus musculus OX=10090 GN=Asf1a PE=1 SV=1	0.71
Adamts1	P97857	A disintegrin and metalloproteinase with thrombospondin motifs 1 OS=Mus musculus OX=10090 G	0.913333333
Purb	O35295	Transcriptional activator protein Pur-beta OS=Mus musculus OX=10090 GN=Purb PE=1 SV=3	0.796666667
Ubr1	O70481	E3 ubiquitin-protein ligase UBR1 OS=Mus musculus OX=10090 GN=Ubr1 PE=1 SV=2	1.46
Nfat5	Q9WV30	Nuclear factor of activated T-cells 5 OS=Mus musculus OX=10090 GN=Nfat5 PE=1 SV=2	1.526666667
Rnaseh2c	Q9CQ18	Ribonuclease H2 subunit C OS=Mus musculus OX=10090 GN=Rnaseh2c PE=1 SV=1	0.99
Plaur	P35456	Urokinase plasminogen activator surface receptor OS=Mus musculus OX=10090 GN=Plaur PE=1 SV=	2.183333333
Rbbp9	O88851	Putative hydrolase RBBP9 OS=Mus musculus OX=10090 GN=Rbbp9 PE=1 SV=2	0.813333333
Abitram	Q80ZQ9	Protein Abitram OS=Mus musculus OX=10090 GN=Abitram PE=1 SV=2	0.77
Lamp1	P11438	Lysosome-associated membrane glycoprotein 1 OS=Mus musculus OX=10090 GN=Lamp1 PE=1 SV=2	0.893333333
Bckdk	O55028	[3-methyl-2-oxobutanoate dehydrogenase [lipoamide]] kinase mitochondrial OS=Mus musculus OX	1.416666667
Pom121	Q8K3Z9	Nuclear envelope pore membrane protein POM 121 OS=Mus musculus OX=10090 GN=Pom121 PE=	1.093333333
Ube2j1	Q9JJZ4	Ubiquitin-conjugating enzyme E2 J1 OS=Mus musculus OX=10090 GN=Ube2j1 PE=1 SV=2	0.913333333
Psmg3	Q9CZH3	Proteasome assembly chaperone 3 OS=Mus musculus OX=10090 GN=Psmg3 PE=1 SV=1	0.893333333
Ufm1	P61961	Ubiquitin-fold modifier 1 OS=Mus musculus OX=10090 GN=Ufm1 PE=1 SV=1	0.813333333
Slu7	Q8BHJ9	Pre-mRNA-splicing factor SLU7 OS=Mus musculus OX=10090 GN=Slu7 PE=1 SV=1	0.836666667
Morf4l1	P60762	Mortality factor 4-like protein 1 OS=Mus musculus OX=10090 GN=Morf4l1 PE=1 SV=2	1.043333333

Ubr3	Q5U430	E3 ubiquitin-protein ligase UBR3 OS=Mus musculus OX=10090 GN=Ubr3 PE=1 SV=3	1.41
Smap	Q9R0P4	Small acidic protein OS=Mus musculus OX=10090 GN=Smap PE=1 SV=1	0.826666667
Cops7b	Q8BV13	COP9 signalosome complex subunit 7b OS=Mus musculus OX=10090 GN=Cops7b PE=1 SV=1	0.733333333
Ctsh	P49935	Pro-cathepsin H OS=Mus musculus OX=10090 GN=Ctsh PE=1 SV=2	0.723333333
Psmg4	P0C7N9	Proteasome assembly chaperone 4 OS=Mus musculus OX=10090 GN=Psmg4 PE=1 SV=1	0.676666667
Slc7a5	Q9Z127	Large neutral amino acids transporter small subunit 1 OS=Mus musculus OX=10090 GN=Slc7a5 PE=[illegible]	0.856666667
Phf10	Q9D8M7	PHD finger protein 10 OS=Mus musculus OX=10090 GN=Phf10 PE=1 SV=4	0.716666667
Nop10	Q9CQS2	H/ACA ribonucleoprotein complex subunit 3 OS=Mus musculus OX=10090 GN=Nop10 PE=3 SV=1	0.883333333
Pqbp1	Q91VJ5	Polyglutamine-binding protein 1 OS=Mus musculus OX=10090 GN=Pqbp1 PE=1 SV=1	0.836666667
Rpl31	P62900	60S ribosomal protein L31 OS=Mus musculus OX=10090 GN=Rpl31 PE=1 SV=1	0.95
Lin7c	O88952	Protein lin-7 homolog C OS=Mus musculus OX=10090 GN=Lin7c PE=1 SV=2	1.036666667
Shoc2	O88520	Leucine-rich repeat protein SHOC-2 OS=Mus musculus OX=10090 GN=Shoc2 PE=1 SV=2	0.946666667
Med4	Q9CQA5	Mediator of RNA polymerase II transcription subunit 4 OS=Mus musculus OX=10090 GN=Med4 PE=[illegible]	1.046666667
Vasn	Q9CZT5	Vasorin OS=Mus musculus OX=10090 GN=Vasn PE=2 SV=2	1.036666667
Ltv1	Q6NSQ7	Protein LTV1 homolog OS=Mus musculus OX=10090 GN=Ltv1 PE=1 SV=2	1.013333333
Ube2q1	Q7TSS2	Ubiquitin-conjugating enzyme E2 Q1 OS=Mus musculus OX=10090 GN=Ube2q1 PE=1 SV=2	0.826666667
Adprhl2	Q8CG72	ADP-ribose glycohydrolase ARH3 OS=Mus musculus OX=10090 GN=Adprs PE=1 SV=1	1.456666667
Riok2	Q9CQS5	Serine/threonine-protein kinase RIO2 OS=Mus musculus OX=10090 GN=Riok2 PE=1 SV=1	0.716666667
Nosip	Q9D6T0	Nitric oxide synthase-interacting protein OS=Mus musculus OX=10090 GN=Nosip PE=1 SV=1	0.913333333
Cox5b	P19536	Cytochrome c oxidase subunit 5B mitochondrial OS=Mus musculus OX=10090 GN=Cox5b PE=1 SV=	0.826666667
Pdlim2	Q8R1G6	PDZ and LIM domain protein 2 OS=Mus musculus OX=10090 GN=Pdlim2 PE=1 SV=1	0.77
Atp5mg	Q9CPQ8	ATP synthase subunit g mitochondrial OS=Mus musculus OX=10090 GN=Atp5mg PE=1 SV=1	0.886666667
Naa30	Q8CES0	N-alpha-acetyltransferase 30 OS=Mus musculus OX=10090 GN=Naa30 PE=1 SV=2	1.156666667
Enoph1	Q8BGB7	Enolase-phosphatase E1 OS=Mus musculus OX=10090 GN=Enoph1 PE=1 SV=1	1.386666667
Fosl1	P48755	Fos-related antigen 1 OS=Mus musculus OX=10090 GN=Fosl1 PE=2 SV=2	0.81
Nagk	Q9QZ08	N-acetyl-D-glucosamine kinase OS=Mus musculus OX=10090 GN=Nagk PE=1 SV=3	0.963333333
Ndufab1	Q9CR21	Acyl carrier protein mitochondrial OS=Mus musculus OX=10090 GN=Ndufab1 PE=1 SV=1	0.88
Frs2	Q8C180	Fibroblast growth factor receptor substrate 2 OS=Mus musculus OX=10090 GN=Frs2 PE=1 SV=3	0.663333333
Ethe1	Q9DCM0	Persulfide dioxygenase ETHE1 mitochondrial OS=Mus musculus OX=10090 GN=Ethe1 PE=1 SV=2	0.94
Irf3	P70671	Interferon regulatory factor 3 OS=Mus musculus OX=10090 GN=Irf3 PE=1 SV=1	0.826666667
N6amt1	Q6SKR2	Methyltransferase N6AMT1 OS=Mus musculus OX=10090 GN=N6amt1 PE=1 SV=1	1.433333333
Zdhhc20	Q5Y5T1	Palmitoyltransferase ZDHHC20 OS=Mus musculus OX=10090 GN=Zdhhc20 PE=1 SV=1	0.79
Gprc5a	Q8BHL4	Retinoic acid-induced protein 3 OS=Mus musculus OX=10090 GN=Gprc5a PE=1 SV=1	0.856666667
Arhgap10	Q6Y5D8	Rho GTPase-activating protein 10 OS=Mus musculus OX=10090 GN=Arhgap10 PE=1 SV=2	1.17

Ppil1	Q9D0W5	Peptidyl-prolyl cis-trans isomerase-like 1 OS=Mus musculus OX=10090 GN=Ppil1 PE=1 SV=1	0.883333333
Ctbs	Q8R242	Di-N-acetylchitobiase OS=Mus musculus OX=10090 GN=Ctbs PE=1 SV=2	0.763333333
Timp1	P12032	Metalloproteinase inhibitor 1 OS=Mus musculus OX=10090 GN=Timp1 PE=1 SV=2	0.97
Vps37c	Q8R105	Vacuolar protein sorting-associated protein 37C OS=Mus musculus OX=10090 GN=Vps37c PE=1 SV=	0.896666667
Fbln1	Q08879	Fibulin-1 OS=Mus musculus OX=10090 GN=Fbln1 PE=1 SV=2	0.326666667
Tax1bp1	Q3UKC1	Tax1-binding protein 1 homolog OS=Mus musculus OX=10090 GN=Tax1bp1 PE=1 SV=2	1.046666667
Gpatch8	A2A6A1	G patch domain-containing protein 8 OS=Mus musculus OX=10090 GN=Gpatch8 PE=1 SV=1	0.823333333
Chchd6	Q91VN4	MICOS complex subunit Mic25 OS=Mus musculus OX=10090 GN=Chchd6 PE=1 SV=2	0.893333333
Arhgef5	E9Q7D5	Rho guanine nucleotide exchange factor 5 OS=Mus musculus OX=10090 GN=Arhgef5 PE=1 SV=1	1
Osbpl2	Q8BX94	Oxysterol-binding protein-related protein 2 OS=Mus musculus OX=10090 GN=Osbpl2 PE=1 SV=1	0.826666667
Mff	Q6PCP5	Mitochondrial fission factor OS=Mus musculus OX=10090 GN=Mff PE=1 SV=1	0.843333333
Lsm3	P62311	U6 snRNA-associated Sm-like protein LSm3 OS=Mus musculus OX=10090 GN=Lsm3 PE=1 SV=2	0.92
Kri1	Q8VDQ9	Protein KRI1 homolog OS=Mus musculus OX=10090 GN=Kri1 PE=1 SV=3	0.716666667
	Q9D937	Uncharacterized protein C11orf98 homolog OS=Mus musculus OX=10090 PE=1 SV=1	0.883333333
Rrp8	Q9DB85	Ribosomal RNA-processing protein 8 OS=Mus musculus OX=10090 GN=Rrp8 PE=1 SV=1	0.806666667
Coq9	Q8K1Z0	Ubiquinone biosynthesis protein COQ9 mitochondrial OS=Mus musculus OX=10090 GN=Coq9 PE=:	0.97
Ccnl1	Q52KE7	Cyclin-L1 OS=Mus musculus OX=10090 GN=Ccnl1 PE=1 SV=1	0.803333333
Dcxr	Q91X52	L-xylulose reductase OS=Mus musculus OX=10090 GN=Dcxr PE=1 SV=2	0.9
Ubl7	Q91W67	Ubiquitin-like protein 7 OS=Mus musculus OX=10090 GN=Ubl7 PE=1 SV=2	0.98
Rpf1	Q7TND5	Ribosome production factor 1 OS=Mus musculus OX=10090 GN=Rpf1 PE=2 SV=2	0.956666667
Mrps10	Q80ZK0	28S ribosomal protein S10 mitochondrial OS=Mus musculus OX=10090 GN=Mrps10 PE=1 SV=1	1.356666667
Galm	Q8K157	Galactose mutarotase OS=Mus musculus OX=10090 GN=Galm PE=1 SV=1	1.153333333
Ncbp3	Q8BZR9	Nuclear cap-binding protein subunit 3 OS=Mus musculus OX=10090 GN=Ncbp3 PE=1 SV=1	0.916666667
Scfd2	Q8BTY8	Sec1 family domain-containing protein 2 OS=Mus musculus OX=10090 GN=Scfd2 PE=1 SV=1	1.126666667
Dnajc25	A2ALW5	DnaJ homolog subfamily C member 25 OS=Mus musculus OX=10090 GN=Dnajc25 PE=1 SV=1	0.57
Rbm4	Q8C7Q4	RNA-binding protein 4 OS=Mus musculus OX=10090 GN=Rbm4 PE=1 SV=1	1.013333333
Agpat4	Q8K4X7	1-acyl-sn-glycerol-3-phosphate acyltransferase delta OS=Mus musculus OX=10090 GN=Agpat4 PE=1	0.843333333
Ube2g1	P62254	Ubiquitin-conjugating enzyme E2 G1 OS=Mus musculus OX=10090 GN=Ube2g1 PE=1 SV=3	0.856666667
Ccdc25	Q78PG9	Coiled-coil domain-containing protein 25 OS=Mus musculus OX=10090 GN=Ccdc25 PE=1 SV=1	0.846666667
Ints9	Q8K114	Integrator complex subunit 9 OS=Mus musculus OX=10090 GN=Ints9 PE=1 SV=1	0.793333333
Srfbp1	Q9CZ91	Serum response factor-binding protein 1 OS=Mus musculus OX=10090 GN=Srfbp1 PE=1 SV=1	0.996666667
Mepce	Q8K3A9	7SK snRNA methylphosphate capping enzyme OS=Mus musculus OX=10090 GN=Mepce PE=1 SV=2	1.436666667
Kif22	Q3V300	Kinesin-like protein KIF22 OS=Mus musculus OX=10090 GN=Kif22 PE=2 SV=2	1.01
Sdhc	Q9CZB0	Succinate dehydrogenase cytochrome b560 subunit mitochondrial OS=Mus musculus OX=10090 G	0.776666667

Sh3glb2	Q8R3V5	Endophilin-B2 OS=Mus musculus OX=10090 GN=Sh3glb2 PE=1 SV=2	1.053333333
Atp6v1d	P57746	V-type proton ATPase subunit D OS=Mus musculus OX=10090 GN=Atp6v1d PE=1 SV=1	1.053333333
Trmt10a	Q8C1Z8	tRNA methyltransferase 10 homolog A OS=Mus musculus OX=10090 GN=Trmt10a PE=2 SV=2	2.846666667
Haus7	Q8BKT8	HAUS augmin-like complex subunit 7 OS=Mus musculus OX=10090 GN=Haus7 PE=1 SV=2	0.906666667
Cdipt	Q8VDP6	CDP-diacylglycerol--inositol 3-phosphatidyltransferase OS=Mus musculus OX=10090 GN=Cdipt PE=:	0.746666667
Dock5	B2RY04	Dedicator of cytokinesis protein 5 OS=Mus musculus OX=10090 GN=Dock5 PE=1 SV=2	0.656666667
Nsmce1	Q9D720	Non-structural maintenance of chromosomes element 1 homolog OS=Mus musculus OX=10090 GN	0.946666667
Fxn	O35943	Frataxin mitochondrial OS=Mus musculus OX=10090 GN=Fxn PE=1 SV=1	0.906666667
Fads3	Q9JJE7	Fatty acid desaturase 3 OS=Mus musculus OX=10090 GN=Fads3 PE=1 SV=2	0.823333333
Ube2f	Q9CY34	NEDD8-conjugating enzyme UBE2F OS=Mus musculus OX=10090 GN=Ube2f PE=1 SV=1	0.58
Cuedc2	Q9CXX9	CUE domain-containing protein 2 OS=Mus musculus OX=10090 GN=Cuedc2 PE=1 SV=1	0.846666667
Drap1	Q9D6N5	Dr1-associated corepressor OS=Mus musculus OX=10090 GN=Drap1 PE=1 SV=3	0.96
Ddx49	Q4FZF3	Probable ATP-dependent RNA helicase DDX49 OS=Mus musculus OX=10090 GN=Ddx49 PE=2 SV=1	0.74
Tyw1	Q8BJM7	S-adenosyl-L-methionine-dependent tRNA 4-demethylwyosine synthase TYW1 OS=Mus musculus O›	1.215
Dlgap4	B1AZP2	Disks large-associated protein 4 OS=Mus musculus OX=10090 GN=Dlgap4 PE=1 SV=1	0.903333333
Srsf10	Q9R0U0	Serine/arginine-rich splicing factor 10 OS=Mus musculus OX=10090 GN=Srsf10 PE=1 SV=2	0.883333333
Pde6d	O55057	Retinal rod rhodopsin-sensitive cGMP 3' 5'-cyclic phosphodiesterase subunit delta OS=Mus musculu	0.963333333
Cd99	Q8VCN6	CD99 antigen OS=Mus musculus OX=10090 GN=Cd99 PE=1 SV=1	0.81
Mrps17	Q9CQE3	28S ribosomal protein S17 mitochondrial OS=Mus musculus OX=10090 GN=Mrps17 PE=1 SV=1	0.973333333
N4bp1	Q6A037	NEDD4-binding protein 1 OS=Mus musculus OX=10090 GN=N4bp1 PE=1 SV=2	1.71
Mrpl9	Q99N94	39S ribosomal protein L9 mitochondrial OS=Mus musculus OX=10090 GN=Mrpl9 PE=1 SV=2	1.063333333
Mxra8	Q9DBV4	Matrix remodeling-associated protein 8 OS=Mus musculus OX=10090 GN=Mxra8 PE=1 SV=1	1.036666667
Trappc3	O55013	Trafficking protein particle complex subunit 3 OS=Mus musculus OX=10090 GN=Trappc3 PE=1 SV=1	0.93
Custos	Q3UY34	Protein CUSTOS OS=Mus musculus OX=10090 GN=Custos PE=2 SV=1	0.703333333
Prmt9	Q3U3W5	Protein arginine N-methyltransferase 9 OS=Mus musculus OX=10090 GN=Prmt9 PE=2 SV=2	2.7
Bcap29	Q61334	B-cell receptor-associated protein 29 OS=Mus musculus OX=10090 GN=Bcap29 PE=1 SV=1	1.166666667
Iah1	Q9DB29	Isoamyl acetate-hydrolyzing esterase 1 homolog OS=Mus musculus OX=10090 GN=Iah1 PE=1 SV=1	0.96
Itih2	Q61703	Inter-alpha-trypsin inhibitor heavy chain H2 OS=Mus musculus OX=10090 GN=Itih2 PE=1 SV=1	0.146666667
Cnot11	Q9CWN7	CCR4-NOT transcription complex subunit 11 OS=Mus musculus OX=10090 GN=Cnot11 PE=1 SV=1	0.73
Mospd2	Q9CWP6	Motile sperm domain-containing protein 2 OS=Mus musculus OX=10090 GN=Mospd2 PE=1 SV=2	1.103333333
Golt1b	Q9CR60	Vesicle transport protein GOT1B OS=Mus musculus OX=10090 GN=Golt1b PE=1 SV=1	0.803333333
Lonp2	Q9DBN5	Lon protease homolog 2 peroxisomal OS=Mus musculus OX=10090 GN=Lonp2 PE=1 SV=1	0.673333333
Cog8	Q9JJA2	Conserved oligomeric Golgi complex subunit 8 OS=Mus musculus OX=10090 GN=Cog8 PE=1 SV=3	0.766666667
Nudt4	Q8R2U6	Diphosphoinositol polyphosphate phosphohydrolase 2 OS=Mus musculus OX=10090 GN=Nudt4 PE	0.813333333

Tut4	B2RX14	Terminal uridylyltransferase 4 OS=Mus musculus OX=10090 GN=Tut4 PE=1 SV=2	0.753333333
Telo2	Q9DC40	Telomere length regulation protein TEL2 homolog OS=Mus musculus OX=10090 GN=Telo2 PE=1 SV=	1.023333333
Cep41	Q99NF3	Centrosomal protein of 41 kDa OS=Mus musculus OX=10090 GN=Cep41 PE=1 SV=1	1.306666667
Ptprk	P35822	Receptor-type tyrosine-protein phosphatase kappa OS=Mus musculus OX=10090 GN=Ptprk PE=1 SV	0.786666667
Cds2	Q99L43	Phosphatidate cytidylyltransferase 2 OS=Mus musculus OX=10090 GN=Cds2 PE=1 SV=1	0.83
Nsmce2	Q91VT1	E3 SUMO-protein ligase NSE2 OS=Mus musculus OX=10090 GN=Nsmce2 PE=2 SV=1	0.84
Atf7ip	Q7TT18	Activating transcription factor 7-interacting protein 1 OS=Mus musculus OX=10090 GN=Atf7ip PE=:	0.623333333
Ppif	Q99KR7	Peptidyl-prolyl cis-trans isomerase F mitochondrial OS=Mus musculus OX=10090 GN=Ppif PE=1 SV=	0.86
Pole3	Q9JKP7	DNA polymerase epsilon subunit 3 OS=Mus musculus OX=10090 GN=Pole3 PE=1 SV=1	0.783333333
Mrps26	Q80ZS3	28S ribosomal protein S26 mitochondrial OS=Mus musculus OX=10090 GN=Mrps26 PE=1 SV=1	0.856666667
Mrrf	Q9D6S7	Ribosome-recycling factor mitochondrial OS=Mus musculus OX=10090 GN=Mrrf PE=1 SV=1	0.826666667
Tma16	Q9CR02	Translation machinery-associated protein 16 OS=Mus musculus OX=10090 GN=Tma16 PE=1 SV=1	0.87
Mtpap	Q9D0D3	Poly(A) RNA polymerase mitochondrial OS=Mus musculus OX=10090 GN=Mtpap PE=1 SV=1	1.993333333
Myo6	Q64331	Unconventional myosin-VI OS=Mus musculus OX=10090 GN=Myo6 PE=1 SV=1	1.043333333
Cth	Q8VCN5	Cystathionine gamma-lyase OS=Mus musculus OX=10090 GN=Cth PE=1 SV=1	0.856666667
Mars2	Q499X9	Methionine--tRNA ligase mitochondrial OS=Mus musculus OX=10090 GN=Mars2 PE=1 SV=2	0.746666667
Nol3	Q9D1X0	Nucleolar protein 3 OS=Mus musculus OX=10090 GN=Nol3 PE=1 SV=1	1.04
Nit1	Q8VDK1	Deaminated glutathione amidase OS=Mus musculus OX=10090 GN=Nit1 PE=1 SV=2	1.433333333
Uhrf2	Q7TMI3	E3 ubiquitin-protein ligase UHRF2 OS=Mus musculus OX=10090 GN=Uhrf2 PE=1 SV=1	0.85
Aida	Q8C4Q6	Axin interactor dorsalization-associated protein OS=Mus musculus OX=10090 GN=Aida PE=1 SV=1	0.98
Timm8b	P62077	Mitochondrial import inner membrane translocase subunit Tim8 B OS=Mus musculus OX=10090 GI	0.933333333
Tdrd3	Q91W18	Tudor domain-containing protein 3 OS=Mus musculus OX=10090 GN=Tdrd3 PE=1 SV=4	1.123333333
Mrpl40	Q9Z2Q5	39S ribosomal protein L40 mitochondrial OS=Mus musculus OX=10090 GN=Mrpl40 PE=1 SV=2	0.863333333
Fbxo6	Q9QZN4	F-box only protein 6 OS=Mus musculus OX=10090 GN=Fbxo6 PE=1 SV=1	1.15
Tspan6	O70401	Tetraspanin-6 OS=Mus musculus OX=10090 GN=Tspan6 PE=1 SV=1	0.983333333
Pdp1	Q3UV70	[Pyruvate dehydrogenase [acetyl-transferring]]-phosphatase 1 mitochondrial OS=Mus musculus OX:	0.83
Sp3	O70494	Transcription factor Sp3 OS=Mus musculus OX=10090 GN=Sp3 PE=1 SV=2	0.91
Rexo2	Q9D8S4	Oligoribonuclease mitochondrial OS=Mus musculus OX=10090 GN=Rexo2 PE=1 SV=2	1.14
Crlf3	Q9Z2L7	Cytokine receptor-like factor 3 OS=Mus musculus OX=10090 GN=Crlf3 PE=1 SV=1	0.9
Med10	Q9CXU0	Mediator of RNA polymerase II transcription subunit 10 OS=Mus musculus OX=10090 GN=Med10 PI	0.83
Suox	Q8R086	Sulfite oxidase mitochondrial OS=Mus musculus OX=10090 GN=Suox PE=1 SV=2	0.983333333
Rangrf	Q9JIB0	Ran guanine nucleotide release factor OS=Mus musculus OX=10090 GN=Rangrf PE=1 SV=1	0.913333333
	Q8C5K5	Uncharacterized protein CXorf38 homolog OS=Mus musculus OX=10090 PE=1 SV=1	1
Ntpcr	Q9CQA9	Cancer-related nucleoside-triphosphatase homolog OS=Mus musculus OX=10090 GN=Ntpcr PE=1 S\	0.916666667

Nubp2	Q9R061	Cytosolic Fe-S cluster assembly factor NUBP2 OS=Mus musculus OX=10090 GN=Nubp2 PE=1 SV=1	0.92
Fzd2	Q9JIP6	Frizzled-2 OS=Mus musculus OX=10090 GN=Fzd2 PE=2 SV=1	0.783333333
Naa20	P61600	N-alpha-acetyltransferase 20 OS=Mus musculus OX=10090 GN=Naa20 PE=1 SV=1	0.88
Vti1b	O88384	Vesicle transport through interaction with t-SNAREs homolog 1B OS=Mus musculus OX=10090 GN=	1.02
Ttc4	Q8R3H9	Tetratricopeptide repeat protein 4 OS=Mus musculus OX=10090 GN=Ttc4 PE=1 SV=1	1.183333333
Cxcl12	P40224	Stromal cell-derived factor 1 OS=Mus musculus OX=10090 GN=Cxcl12 PE=2 SV=2	0.88
Mrpl53	Q9D1H8	39S ribosomal protein L53 mitochondrial OS=Mus musculus OX=10090 GN=Mrpl53 PE=1 SV=1	1
Gpn1	Q8VCE2	GPN-loop GTPase 1 OS=Mus musculus OX=10090 GN=Gpn1 PE=1 SV=1	0.97
Ndufb11	O09111	NADH dehydrogenase [ubiquinone] 1 beta subcomplex subunit 11 mitochondrial OS=Mus musculu	0.913333333
Ndufb9	Q9CQJ8	NADH dehydrogenase [ubiquinone] 1 beta subcomplex subunit 9 OS=Mus musculus OX=10090 GN=	0.886666667
Rab24	P35290	Ras-related protein Rab-24 OS=Mus musculus OX=10090 GN=Rab24 PE=1 SV=2	0.813333333
Mgmt	P26187	Methylated-DNA--protein-cysteine methyltransferase OS=Mus musculus OX=10090 GN=Mgmt PE=1	0.476666667
Brk1	Q91VR8	Protein BRICK1 OS=Mus musculus OX=10090 GN=Brk1 PE=1 SV=1	0.9
Dlk1	Q09163	Protein delta homolog 1 OS=Mus musculus OX=10090 GN=Dlk1 PE=1 SV=1	0.733333333
Ly6c1	P0CW02	Lymphocyte antigen 6C1 OS=Mus musculus OX=10090 GN=Ly6c1 PE=2 SV=1	0.826666667
Ndufa5	Q9CPP6	NADH dehydrogenase [ubiquinone] 1 alpha subcomplex subunit 5 OS=Mus musculus OX=10090 GN	0.85
Tmem173	Q3TBT3	Stimulator of interferon genes protein OS=Mus musculus OX=10090 GN=Sting1 PE=1 SV=2	
Mtco1	P00397	Cytochrome c oxidase subunit 1 OS=Mus musculus OX=10090 GN=Mtco1 PE=1 SV=2	0.716666667
Rbm5	Q91YE7	RNA-binding protein 5 OS=Mus musculus OX=10090 GN=Rbm5 PE=1 SV=1	0.736666667
Smc6	Q924W5	Structural maintenance of chromosomes protein 6 OS=Mus musculus OX=10090 GN=Smc6 PE=1 SV	1.033333333
Sdcbp	O08992	Syntenin-1 OS=Mus musculus OX=10090 GN=Sdcbp PE=1 SV=1	4.91
Rfk	Q8CFV9	Riboflavin kinase OS=Mus musculus OX=10090 GN=Rfk PE=1 SV=2	0.663333333
Fastkd2	Q922E6	FAST kinase domain-containing protein 2 mitochondrial OS=Mus musculus OX=10090 GN=Fastkd2	0.98
Acox1	Q9R0H0	Peroxisomal acyl-coenzyme A oxidase 1 OS=Mus musculus OX=10090 GN=Acox1 PE=1 SV=5	0.813333333
Rars2	Q3U186	Probable arginine--tRNA ligase mitochondrial OS=Mus musculus OX=10090 GN=Rars2 PE=1 SV=1	0.963333333
Polr1e	Q8K202	DNA-directed RNA polymerase I subunit RPA49 OS=Mus musculus OX=10090 GN=Polr1e PE=1 SV=2	1.043333333
Spryd4	Q91WK1	SPRY domain-containing protein 4 OS=Mus musculus OX=10090 GN=Spryd4 PE=1 SV=1	1.113333333
Mpc2	Q9D023	Mitochondrial pyruvate carrier 2 OS=Mus musculus OX=10090 GN=Mpc2 PE=1 SV=1	0.813333333
Czib	Q8BHG2	CXXC motif containing zinc binding protein OS=Mus musculus OX=10090 GN=Czib PE=1 SV=1	0.973333333
Cybc1	Q3TYS2	Cytochrome b-245 chaperone 1 OS=Mus musculus OX=10090 GN=Cybc1 PE=1 SV=2	0.803333333
Pex14	Q9R0A0	Peroxisomal membrane protein PEX14 OS=Mus musculus OX=10090 GN=Pex14 PE=1 SV=1	0.93
Vars2	Q3U2A8	Valine--tRNA ligase mitochondrial OS=Mus musculus OX=10090 GN=Vars2 PE=1 SV=2	1.75
Psmg2	Q9EST4	Proteasome assembly chaperone 2 OS=Mus musculus OX=10090 GN=Psmg2 PE=1 SV=1	1.05
Col12a1	Q60847	Collagen alpha-1(XII) chain OS=Mus musculus OX=10090 GN=Col12a1 PE=2 SV=3	10.44666667

Ttc21b	Q0HA38	Tetratricopeptide repeat protein 21B OS=Mus musculus OX=10090 GN=Ttc21b PE=1 SV=1	1.643333333
Chmp3	Q9CQ10	Charged multivesicular body protein 3 OS=Mus musculus OX=10090 GN=Chmp3 PE=1 SV=3	0.966666667
Xpo6	Q924Z6	Exportin-6 OS=Mus musculus OX=10090 GN=Xpo6 PE=1 SV=2	0.853333333
Armc1	Q9D7A8	Armadillo repeat-containing protein 1 OS=Mus musculus OX=10090 GN=Armc1 PE=1 SV=1	2.026666667
Tspo	P50637	Translocator protein OS=Mus musculus OX=10090 GN=Tspo PE=1 SV=1	0.78
Focad	A2AKG8	Focadhesin OS=Mus musculus OX=10090 GN=Focad PE=1 SV=1	1.61
Tmed3	Q78IS1	Transmembrane emp24 domain-containing protein 3 OS=Mus musculus OX=10090 GN=Tmed3 PE=1	0.76
Cyb5r1	Q9DB73	NADH-cytochrome b5 reductase 1 OS=Mus musculus OX=10090 GN=Cyb5r1 PE=1 SV=1	0.89
Psmf1	Q8BHL8	Proteasome inhibitor PI31 subunit OS=Mus musculus OX=10090 GN=Psmf1 PE=1 SV=1	0.763333333
Slc38a10	Q5I012	Putative sodium-coupled neutral amino acid transporter 10 OS=Mus musculus OX=10090 GN=Slc38	0.97
Ndufb5	Q9CQH3	NADH dehydrogenase [ubiquinone] 1 beta subcomplex subunit 5 mitochondrial OS=Mus musculus	0.993333333
Itm2b	O89051	Integral membrane protein 2B OS=Mus musculus OX=10090 GN=Itm2b PE=1 SV=1	1.076666667
Mrpl3	Q99N95	39S ribosomal protein L3 mitochondrial OS=Mus musculus OX=10090 GN=Mrpl3 PE=1 SV=1	1.263333333
Tmed1	Q3V009	Transmembrane emp24 domain-containing protein 1 OS=Mus musculus OX=10090 GN=Tmed1 PE=1	0.91
Ubap1	Q8BH48	Ubiquitin-associated protein 1 OS=Mus musculus OX=10090 GN=Ubap1 PE=1 SV=1	1.246666667
Mrpl2	Q9D773	39S ribosomal protein L2 mitochondrial OS=Mus musculus OX=10090 GN=Mrpl2 PE=1 SV=1	0.836666667
Nkrf	Q8BY02	NF-kappa-B-repressing factor OS=Mus musculus OX=10090 GN=Nkrf PE=2 SV=3	1.11
Cav2	Q9WVC3	Caveolin-2 OS=Mus musculus OX=10090 GN=Cav2 PE=1 SV=1	1.026666667
Mrpl16	Q99N93	39S ribosomal protein L16 mitochondrial OS=Mus musculus OX=10090 GN=Mrpl16 PE=1 SV=1	0.886666667
Nsa2	Q9CR47	Ribosome biogenesis protein NSA2 homolog OS=Mus musculus OX=10090 GN=Nsa2 PE=2 SV=1	0.93
Dctpp1	Q9QY93	dCTP pyrophosphatase 1 OS=Mus musculus OX=10090 GN=Dctpp1 PE=1 SV=1	0.926666667
Ube2s	Q921J4	Ubiquitin-conjugating enzyme E2 S OS=Mus musculus OX=10090 GN=Ube2s PE=1 SV=1	1.01
Foxc1	Q61572	Forkhead box protein C1 OS=Mus musculus OX=10090 GN=Foxc1 PE=1 SV=3	0.966666667
Trappc5	Q9CQA1	Trafficking protein particle complex subunit 5 OS=Mus musculus OX=10090 GN=Trappc5 PE=1 SV=1	1.333333333
Rngtt	O55236	mRNA-capping enzyme OS=Mus musculus OX=10090 GN=Rngtt PE=1 SV=1	1.026666667
Eps8l2	Q99K30	Epidermal growth factor receptor kinase substrate 8-like protein 2 OS=Mus musculus OX=10090 GN	0.793333333
Mrpl10	Q3TBW2	39S ribosomal protein L10 mitochondrial OS=Mus musculus OX=10090 GN=Mrpl10 PE=1 SV=2	1.18
Pop4	Q9CR08	Ribonuclease P protein subunit p29 OS=Mus musculus OX=10090 GN=Pop4 PE=2 SV=1	0.92
Znf593	Q9DB42	Zinc finger protein 593 OS=Mus musculus OX=10090 GN=Znf593 PE=1 SV=2	1.5
Eif1ad	Q3THJ3	Probable RNA-binding protein EIF1AD OS=Mus musculus OX=10090 GN=Eif1ad PE=1 SV=2	0.84
R3hcc1	Q8BSI6	R3H and coiled-coil domain-containing protein 1 OS=Mus musculus OX=10090 GN=R3hcc1 PE=1 SV	1.136666667
Kdm5c	P41230	Lysine-specific demethylase 5C OS=Mus musculus OX=10090 GN=Kdm5c PE=1 SV=4	1.253333333
Setd1a	E9PYH6	Histone-lysine N-methyltransferase SETD1A OS=Mus musculus OX=10090 GN=Setd1a PE=1 SV=1	0.636666667
Slc27a4	Q91VE0	Long-chain fatty acid transport protein 4 OS=Mus musculus OX=10090 GN=Slc27a4 PE=1 SV=1	0.84

Homer3	Q99JP6	Homer protein homolog 3 OS=Mus musculus OX=10090 GN=Homer3 PE=1 SV=2	0.945
Nt5dc1	Q8C5P5	5'-nucleotidase domain-containing protein 1 OS=Mus musculus OX=10090 GN=Nt5dc1 PE=1 SV=2	1.01
Ndufs8	Q8K3J1	NADH dehydrogenase [ubiquinone] iron-sulfur protein 8 mitochondrial OS=Mus musculus OX=100	0.823333333
Nfx1	B1AY10	Transcriptional repressor NF-X1 OS=Mus musculus OX=10090 GN=Nfx1 PE=1 SV=1	1.18
Fam136a	Q9CR98	Protein FAM136A OS=Mus musculus OX=10090 GN=Fam136a PE=1 SV=1	0.966666667
Sdc1	P18828	Syndecan-1 OS=Mus musculus OX=10090 GN=Sdc1 PE=1 SV=1	0.693333333
Ccn2	P29268	CCN family member 2 OS=Mus musculus OX=10090 GN=Ccn2 PE=2 SV=3	0.956666667
Tbpl1	P62340	TATA box-binding protein-like 1 OS=Mus musculus OX=10090 GN=Tbpl1 PE=1 SV=1	1.226666667
Islr	Q6GU68	Immunoglobulin superfamily containing leucine-rich repeat protein OS=Mus musculus OX=10090 (	0.68
Tigar	Q8BZA9	Fructose-2 6-bisphosphatase TIGAR OS=Mus musculus OX=10090 GN=Tigar PE=1 SV=1	0.773333333
Smtn	Q921U8	Smoothelin OS=Mus musculus OX=10090 GN=Smtn PE=1 SV=2	1.103333333
Gle1	Q8R322	Nucleoporin GLE1 OS=Mus musculus OX=10090 GN=Gle1 PE=1 SV=2	0.88
Rab3ip	Q68EF0	Rab-3A-interacting protein OS=Mus musculus OX=10090 GN=Rab3ip PE=1 SV=1	0.79
Tfpi	O54819	Tissue factor pathway inhibitor OS=Mus musculus OX=10090 GN=Tfpi PE=2 SV=1	1.946666667
Stx18	Q8VDS8	Syntaxin-18 OS=Mus musculus OX=10090 GN=Stx18 PE=1 SV=2	0.783333333
Tacc2	Q9JJG0	Transforming acidic coiled-coil-containing protein 2 OS=Mus musculus OX=10090 GN=Tacc2 PE=1 S	0.543333333
Coil	Q5SU73	Coilin OS=Mus musculus OX=10090 GN=Coil PE=1 SV=1	1.066666667
Dnajc1	Q61712	DnaJ homolog subfamily C member 1 OS=Mus musculus OX=10090 GN=Dnajc1 PE=1 SV=1	1.696666667
Fundc2	Q9D6K8	FUN14 domain-containing protein 2 OS=Mus musculus OX=10090 GN=Fundc2 PE=1 SV=1	0.786666667
Itsn2	Q9Z0R6	Intersectin-2 OS=Mus musculus OX=10090 GN=Itsn2 PE=1 SV=2	1.066666667
Ak6	Q8VCP8	Adenylate kinase isoenzyme 6 OS=Mus musculus OX=10090 GN=Ak6 PE=1 SV=1	1.163333333
Kat7	Q5SVQ0	Histone acetyltransferase KAT7 OS=Mus musculus OX=10090 GN=Kat7 PE=1 SV=1	1.016666667
Cep131	Q62036	Centrosomal protein of 131 kDa OS=Mus musculus OX=10090 GN=Cep131 PE=1 SV=2	0.836666667
Atp11c	Q9QZW0	Phospholipid-transporting ATPase 11C OS=Mus musculus OX=10090 GN=Atp11c PE=1 SV=2	1.1
Dnajb6	O54946	DnaJ homolog subfamily B member 6 OS=Mus musculus OX=10090 GN=Dnajb6 PE=1 SV=4	0.97
Ctif	Q6PEE2	CBP80/20-dependent translation initiation factor OS=Mus musculus OX=10090 GN=Ctif PE=1 SV=2	1.25
Ccs	Q9WU84	Copper chaperone for superoxide dismutase OS=Mus musculus OX=10090 GN=Ccs PE=1 SV=1	1.126666667
Cmss1	Q9CZT6	Protein CMSS1 OS=Mus musculus OX=10090 GN=Cmss1 PE=2 SV=1	1.693333333
Paxbp1	P58501	PAX3- and PAX7-binding protein 1 OS=Mus musculus OX=10090 GN=Paxbp1 PE=1 SV=3	0.736666667
Ptrhd1	D3Z4S3	Putative peptidyl-tRNA hydrolase PTRHD1 OS=Mus musculus OX=10090 GN=Ptrhd1 PE=1 SV=1	0.62
Dnttip1	Q99LB0	Deoxynucleotidyltransferase terminal-interacting protein 1 OS=Mus musculus OX=10090 GN=Dntti	0.923333333
Supt4h1b	Q9Z199	Transcription elongation factor SPT4-B OS=Mus musculus OX=10090 GN=Supt4h1b PE=2 SV=1	0.89
Supt4h1a	P63271	Transcription elongation factor SPT4-A OS=Mus musculus OX=10090 GN=Supt4h1a PE=2 SV=1	0.89
Twistnb	Q78WZ7	DNA-directed RNA polymerase I subunit RPA43 OS=Mus musculus OX=10090 GN=Polr1f PE=1 SV=1	0.686666667

Wdfy3	Q6VNB8	WD repeat and FYVE domain-containing protein 3 OS=Mus musculus OX=10090 GN=Wdfy3 PE=1 SV	1.915
Katnb1	Q8BG40	Katanin p80 WD40 repeat-containing subunit B1 OS=Mus musculus OX=10090 GN=Katnb1 PE=1 SV	1.086666667
Gsta4	P24472	Glutathione S-transferase A4 OS=Mus musculus OX=10090 GN=Gsta4 PE=1 SV=3	0.633333333
Snap29	Q9ERB0	Synaptosomal-associated protein 29 OS=Mus musculus OX=10090 GN=Snap29 PE=1 SV=1	0.993333333
Pld3	O35405	5'-3' exonuclease PLD3 OS=Mus musculus OX=10090 GN=Pld3 PE=1 SV=1	0.77
Trpc4ap	Q9JLV2	Short transient receptor potential channel 4-associated protein OS=Mus musculus OX=10090 GN=T	0.81
Dus2	Q9D7B1	tRNA-dihydrouridine(20) synthase [NAD(P)+]-like OS=Mus musculus OX=10090 GN=Dus2 PE=1 SV=1	0.923333333
Stx4	P70452	Syntaxin-4 OS=Mus musculus OX=10090 GN=Stx4 PE=1 SV=1	0.936666667
Chmp7	Q8R1T1	Charged multivesicular body protein 7 OS=Mus musculus OX=10090 GN=Chmp7 PE=1 SV=1	0.726666667
Sestd1	Q80UK0	SEC14 domain and spectrin repeat-containing protein 1 OS=Mus musculus OX=10090 GN=Sestd1 PE	0.806666667
Ocrl	Q6NVF0	Inositol polyphosphate 5-phosphatase OCRL OS=Mus musculus OX=10090 GN=Ocrl PE=1 SV=1	0.813333333
Ak5	Q920P5	Adenylate kinase isoenzyme 5 OS=Mus musculus OX=10090 GN=Ak5 PE=1 SV=2	1.07
Smug1	Q6P5C5	Single-strand selective monofunctional uracil DNA glycosylase OS=Mus musculus OX=10090 GN=Sm	1.776666667
Erap1	Q9EQH2	Endoplasmic reticulum aminopeptidase 1 OS=Mus musculus OX=10090 GN=Erap1 PE=1 SV=2	0.976666667
Polb	Q8K409	DNA polymerase beta OS=Mus musculus OX=10090 GN=Polb PE=1 SV=3	0.643333333
Map6	Q7TSJ2	Microtubule-associated protein 6 OS=Mus musculus OX=10090 GN=Map6 PE=1 SV=2	1.29
Tank	P70347	TRAF family member-associated NF-kappa-B activator OS=Mus musculus OX=10090 GN=Tank PE=1 S	1.403333333
Apoo	Q9DCZ4	MICOS complex subunit Mic26 OS=Mus musculus OX=10090 GN=Apoo PE=1 SV=2	1.053333333
Oxa1l	Q8BGA9	Mitochondrial inner membrane protein OXA1L OS=Mus musculus OX=10090 GN=Oxa1l PE=1 SV=1	0.836666667
Ankrd52	Q8BTI7	Serine/threonine-protein phosphatase 6 regulatory ankyrin repeat subunit C OS=Mus musculus OX=	1.215
Serinc1	Q9QZI8	Serine incorporator 1 OS=Mus musculus OX=10090 GN=Serinc1 PE=1 SV=1	1.233333333
Stim1	P70302	Stromal interaction molecule 1 OS=Mus musculus OX=10090 GN=Stim1 PE=1 SV=2	1.593333333
Tpd52	Q62393	Tumor protein D52 OS=Mus musculus OX=10090 GN=Tpd52 PE=1 SV=2	2.096666667
Uqcc1	Q9CWU6	Ubiquinol-cytochrome-c reductase complex assembly factor 1 OS=Mus musculus OX=10090 GN=Uq	0.753333333
Abraxas2	Q3TCJ1	BRISC complex subunit Abraxas 2 OS=Mus musculus OX=10090 GN=Abraxas2 PE=1 SV=1	0.843333333
Lsm6	P62313	U6 snRNA-associated Sm-like protein LSm6 OS=Mus musculus OX=10090 GN=Lsm6 PE=1 SV=1	1.426666667
Fkbp5	Q64378	Peptidyl-prolyl cis-trans isomerase FKBP5 OS=Mus musculus OX=10090 GN=Fkbp5 PE=1 SV=1	0.93
Dnajc11	Q5U458	DnaJ homolog subfamily C member 11 OS=Mus musculus OX=10090 GN=Dnajc11 PE=1 SV=2	0.843333333
Ighmbp2	P40694	DNA-binding protein SMUBP-2 OS=Mus musculus OX=10090 GN=Ighmbp2 PE=1 SV=1	0.945
Wbp2	P97765	WW domain-binding protein 2 OS=Mus musculus OX=10090 GN=Wbp2 PE=1 SV=1	0.926666667
Gcfc2	Q8BKT3	GC-rich sequence DNA-binding factor 2 OS=Mus musculus OX=10090 GN=Gcfc2 PE=1 SV=2	0.68
Efhd2	Q9D8Y0	EF-hand domain-containing protein D2 OS=Mus musculus OX=10090 GN=Efhd2 PE=1 SV=1	0.733333333
Slc1a5	P51912	Neutral amino acid transporter B(0) OS=Mus musculus OX=10090 GN=Slc1a5 PE=1 SV=2	1.076666667
Mrpl47	Q8K2Y7	39S ribosomal protein L47 mitochondrial OS=Mus musculus OX=10090 GN=Mrpl47 PE=1 SV=2	1.003333333

Serhl	Q9EPB5	Serine hydrolase-like protein OS=Mus musculus OX=10090 GN=Serhl PE=1 SV=1	0.623333333
Rmdn3	Q3UJU9	Regulator of microtubule dynamics protein 3 OS=Mus musculus OX=10090 GN=Rmdn3 PE=1 SV=2	0.833333333
Usp40	Q8BWR4	Ubiquitin carboxyl-terminal hydrolase 40 OS=Mus musculus OX=10090 GN=Usp40 PE=1 SV=2	0.93
Pelo	Q80X73	Protein pelota homolog OS=Mus musculus OX=10090 GN=Pelo PE=1 SV=3	2.02
Wdr7	Q920I9	WD repeat-containing protein 7 OS=Mus musculus OX=10090 GN=Wdr7 PE=1 SV=3	0.6
Mrps28	Q9CY16	28S ribosomal protein S28 mitochondrial OS=Mus musculus OX=10090 GN=Mrps28 PE=1 SV=1	0.773333333
Rpl35	Q6ZWV7	60S ribosomal protein L35 OS=Mus musculus OX=10090 GN=Rpl35 PE=1 SV=1	0.94
Bak1	O08734	Bcl-2 homologous antagonist/killer OS=Mus musculus OX=10090 GN=Bak1 PE=1 SV=3	1.016666667
Use1	Q9CQ56	Vesicle transport protein USE1 OS=Mus musculus OX=10090 GN=Use1 PE=1 SV=1	1.473333333
Tefm	Q5SSK3	Transcription elongation factor mitochondrial OS=Mus musculus OX=10090 GN=Tefm PE=1 SV=1	0.85
Ankzf1	Q80UU1	Ankyrin repeat and zinc finger domain-containing protein 1 OS=Mus musculus OX=10090 GN=Ankzf	1.153333333
Neo1	P97798	Neogenin OS=Mus musculus OX=10090 GN=Neo1 PE=1 SV=1	1.57
Sgce	O70258	Epsilon-sarcoglycan OS=Mus musculus OX=10090 GN=Sgce PE=1 SV=2	1.095
Gnb4	P29387	Guanine nucleotide-binding protein subunit beta-4 OS=Mus musculus OX=10090 GN=Gnb4 PE=1 S\	0.64
Fmnl2	A2APV2	Formin-like protein 2 OS=Mus musculus OX=10090 GN=Fmnl2 PE=1 SV=2	3.295
Akt3	Q9WUA6	RAC-gamma serine/threonine-protein kinase OS=Mus musculus OX=10090 GN=Akt3 PE=1 SV=1	
Ube2e3	P52483	Ubiquitin-conjugating enzyme E2 E3 OS=Mus musculus OX=10090 GN=Ube2e3 PE=1 SV=2	1.015
Braf	P28028	Serine/threonine-protein kinase B-raf OS=Mus musculus OX=10090 GN=Braf PE=1 SV=3	1.806666667
Mcts2	Q9CQ21	Malignant T-cell-amplified sequence 2 OS=Mus musculus OX=10090 GN=Mcts2 PE=1 SV=1	0.98
Rhob	P62746	Rho-related GTP-binding protein RhoB OS=Mus musculus OX=10090 GN=Rhob PE=1 SV=1	1.563333333
Rap2b	P61226	Ras-related protein Rap-2b OS=Mus musculus OX=10090 GN=Rap2b PE=1 SV=1	0.876666667
Nek6	Q9ES70	Serine/threonine-protein kinase Nek6 OS=Mus musculus OX=10090 GN=Nek6 PE=1 SV=1	0.786666667
Sec61a2	Q9JLR1	Protein transport protein Sec61 subunit alpha isoform 2 OS=Mus musculus OX=10090 GN=Sec61a2	1.106666667
Scd2	P13011	Stearoyl-CoA desaturase 2 OS=Mus musculus OX=10090 GN=Scd2 PE=1 SV=2	0.683333333
Dcun1d1	Q9QZ73	DCN1-like protein 1 OS=Mus musculus OX=10090 GN=Dcun1d1 PE=2 SV=1	0.956666667
Dysf	Q9ESD7	Dysferlin OS=Mus musculus OX=10090 GN=Dysf PE=1 SV=3	2.465
Rab4b	Q91ZR1	Ras-related protein Rab-4B OS=Mus musculus OX=10090 GN=Rab4b PE=1 SV=2	1.436666667
Insr	P15208	Insulin receptor OS=Mus musculus OX=10090 GN=Insr PE=1 SV=2	1.816666667
Rab22a	P35285	Ras-related protein Rab-22A OS=Mus musculus OX=10090 GN=Rab22a PE=1 SV=2	0.876666667
Cdk17	Q8K0D0	Cyclin-dependent kinase 17 OS=Mus musculus OX=10090 GN=Cdk17 PE=1 SV=2	0.88
Scaf8	Q6DID3	SR-related and CTD-associated factor 8 OS=Mus musculus OX=10090 GN=Scaf8 PE=1 SV=1	0.926666667
Cdk12	Q14AX6	Cyclin-dependent kinase 12 OS=Mus musculus OX=10090 GN=Cdk12 PE=1 SV=2	1.483333333
Grem1	O70326	Gremlin-1 OS=Mus musculus OX=10090 GN=Grem1 PE=2 SV=1	0.986666667
Rpe	Q8VEE0	Ribulose-phosphate 3-epimerase OS=Mus musculus OX=10090 GN=Rpe PE=1 SV=1	0.993333333

Pcbp4	P57724	Poly(rC)-binding protein 4 OS=Mus musculus OX=10090 GN=Pcbp4 PE=1 SV=1	0.893333333
Ptbp2	Q91Z31	Polypyrimidine tract-binding protein 2 OS=Mus musculus OX=10090 GN=Ptbp2 PE=1 SV=2	1.12
Hdac3	O88895	Histone deacetylase 3 OS=Mus musculus OX=10090 GN=Hdac3 PE=1 SV=1	1.003333333
Tead1	P30051	Transcriptional enhancer factor TEF-1 OS=Mus musculus OX=10090 GN=Tead1 PE=1 SV=2	0.606666667
Armc10	Q9D0L7	Armadillo repeat-containing protein 10 OS=Mus musculus OX=10090 GN=Armc10 PE=1 SV=1	1.123333333
Samd4a	Q8CBY1	Protein Smaug homolog 1 OS=Mus musculus OX=10090 GN=Samd4a PE=1 SV=2	0.746666667
Ppp2r5a	Q6PD03	Serine/threonine-protein phosphatase 2A 56 kDa regulatory subunit alpha isoform OS=Mus muscul	0.686666667
Lrrfip2	Q91WK0	Leucine-rich repeat flightless-interacting protein 2 OS=Mus musculus OX=10090 GN=Lrrfip2 PE=1 S	0.696666667
Srsf3	P84104	Serine/arginine-rich splicing factor 3 OS=Mus musculus OX=10090 GN=Srsf3 PE=1 SV=1	0.943333333
Rab12	P35283	Ras-related protein Rab-12 OS=Mus musculus OX=10090 GN=Rab12 PE=1 SV=3	1.026666667
Ano10	Q8BH79	Anoctamin-10 OS=Mus musculus OX=10090 GN=Ano10 PE=1 SV=1	0.79
Smap1	Q91VZ6	Stromal membrane-associated protein 1 OS=Mus musculus OX=10090 GN=Smap1 PE=1 SV=1	1.003333333
Trim24	Q64127	Transcription intermediary factor 1-alpha OS=Mus musculus OX=10090 GN=Trim24 PE=1 SV=1	1.333333333
Tbce	Q8CIV8	Tubulin-specific chaperone E OS=Mus musculus OX=10090 GN=Tbce PE=1 SV=1	0.85
Prune2	Q52KR3	Protein prune homolog 2 OS=Mus musculus OX=10090 GN=Prune2 PE=1 SV=2	0.906666667
Ddhd2	Q80Y98	Phospholipase DDHD2 OS=Mus musculus OX=10090 GN=Ddhd2 PE=1 SV=3	1.016666667
Sh3pxd2a	O89032	SH3 and PX domain-containing protein 2A OS=Mus musculus OX=10090 GN=Sh3pxd2a PE=1 SV=2	1.32
Rbm33	Q9CXK9	RNA-binding protein 33 OS=Mus musculus OX=10090 GN=Rbm33 PE=1 SV=2	1.086666667
Plekhf1	Q3TB82	Pleckstrin homology domain-containing family F member 1 OS=Mus musculus OX=10090 GN=Plekh	0.926666667
Casp6	O08738	Caspase-6 OS=Mus musculus OX=10090 GN=Casp6 PE=1 SV=1	0.736666667
Cap2	Q9CYT6	Adenylyl cyclase-associated protein 2 OS=Mus musculus OX=10090 GN=Cap2 PE=1 SV=1	1.216666667
C4b	P01029	Complement C4-B OS=Mus musculus OX=10090 GN=C4b PE=1 SV=3	0.11
Ube2r2	Q6ZWZ2	Ubiquitin-conjugating enzyme E2 R2 OS=Mus musculus OX=10090 GN=Ube2r2 PE=1 SV=1	0.896666667
Krt10	P02535	Keratin type I cytoskeletal 10 OS=Mus musculus OX=10090 GN=Krt10 PE=1 SV=3	0.433333333
Nhp2	Q9CRB2	H/ACA ribonucleoprotein complex subunit 2 OS=Mus musculus OX=10090 GN=Nhp2 PE=1 SV=1	0.863333333
Itpa	Q9D892	Inosine triphosphate pyrophosphatase OS=Mus musculus OX=10090 GN=Itpa PE=1 SV=2	0.826666667
Nudt5	Q9JKX6	ADP-sugar pyrophosphatase OS=Mus musculus OX=10090 GN=Nudt5 PE=1 SV=1	0.913333333
Hmga2	P52927	High mobility group protein HMGI-C OS=Mus musculus OX=10090 GN=Hmga2 PE=1 SV=1	0.59
Znrd2	P56873	Protein ZNRD2 OS=Mus musculus OX=10090 GN=Znrd2 PE=1 SV=1	0.963333333
Usp16	Q99LG0	Ubiquitin carboxyl-terminal hydrolase 16 OS=Mus musculus OX=10090 GN=Usp16 PE=1 SV=2	0.923333333
Cttnbp2nl	Q99LJ0	CTTNBP2 N-terminal-like protein OS=Mus musculus OX=10090 GN=Cttnbp2nl PE=1 SV=1	0.94
Nsd2	Q8BVE8	Histone-lysine N-methyltransferase NSD2 OS=Mus musculus OX=10090 GN=Nsd2 PE=1 SV=2	0.783333333
Rbm42	Q91V81	RNA-binding protein 42 OS=Mus musculus OX=10090 GN=Rbm42 PE=1 SV=2	0.72
Cops8	Q8VBV7	COP9 signalosome complex subunit 8 OS=Mus musculus OX=10090 GN=Cops8 PE=1 SV=1	0.843333333

Polr2i	P60898	DNA-directed RNA polymerase II subunit RPB9 OS=Mus musculus OX=10090 GN=Polr2i PE=1 SV=1	1.056666667
Stx12	Q9ER00	Syntaxin-12 OS=Mus musculus OX=10090 GN=Stx12 PE=1 SV=1	0.923333333
Scamp1	Q8K021	Secretory carrier-associated membrane protein 1 OS=Mus musculus OX=10090 GN=Scamp1 PE=1 S\	0.823333333
Ifrd1	P19182	Interferon-related developmental regulator 1 OS=Mus musculus OX=10090 GN=Ifrd1 PE=1 SV=2	1.053333333
Acp1	Q9D358	Low molecular weight phosphotyrosine protein phosphatase OS=Mus musculus OX=10090 GN=Acp	0.873333333
Tradd	Q3U0V2	Tumor necrosis factor receptor type 1-associated DEATH domain protein OS=Mus musculus OX=100	0.996666667
Uba5	Q8VE47	Ubiquitin-like modifier-activating enzyme 5 OS=Mus musculus OX=10090 GN=Uba5 PE=1 SV=2	0.88
Srsf2	Q62093	Serine/arginine-rich splicing factor 2 OS=Mus musculus OX=10090 GN=Srsf2 PE=1 SV=4	0.95
Nudt2	P56380	Bis(5'-nucleosyl)-tetraphosphatase [asymmetrical] OS=Mus musculus OX=10090 GN=Nudt2 PE=1 SV	0.896666667
Tax1bp3	Q9DBG9	Tax1-binding protein 3 OS=Mus musculus OX=10090 GN=Tax1bp3 PE=1 SV=1	0.88
Rps24	P62849	40S ribosomal protein S24 OS=Mus musculus OX=10090 GN=Rps24 PE=1 SV=1	0.91
Ubfd1	Q78JW9	Ubiquitin domain-containing protein UBFD1 OS=Mus musculus OX=10090 GN=Ubfd1 PE=1 SV=2	0.756666667
Pno1	Q9CPS7	RNA-binding protein PNO1 OS=Mus musculus OX=10090 GN=Pno1 PE=1 SV=1	0.81
Cers2	Q924Z4	Ceramide synthase 2 OS=Mus musculus OX=10090 GN=Cers2 PE=1 SV=1	0.926666667
Micu2	Q8CD10	Calcium uptake protein 2 mitochondrial OS=Mus musculus OX=10090 GN=Micu2 PE=1 SV=2	0.816666667
P3h4	Q8K2B0	Endoplasmic reticulum protein SC65 OS=Mus musculus OX=10090 GN=P3h4 PE=1 SV=1	0.986666667
Gap43	P06837	Neuromodulin OS=Mus musculus OX=10090 GN=Gap43 PE=1 SV=1	0.7
Serpine1	P22777	Plasminogen activator inhibitor 1 OS=Mus musculus OX=10090 GN=Serpine1 PE=1 SV=1	1.285
Sf3b4	Q8QZY9	Splicing factor 3B subunit 4 OS=Mus musculus OX=10090 GN=Sf3b4 PE=1 SV=1	0.866666667
F2	P19221	Prothrombin OS=Mus musculus OX=10090 GN=F2 PE=1 SV=1	0.27
Utp3	Q9JI13	Something about silencing protein 10 OS=Mus musculus OX=10090 GN=Utp3 PE=1 SV=1	0.873333333
Itprip	Q3TNL8	Inositol 1 4 5-trisphosphate receptor-interacting protein OS=Mus musculus OX=10090 GN=Itprip P	1.346666667
Sub1	P11031	Activated RNA polymerase II transcriptional coactivator p15 OS=Mus musculus OX=10090 GN=Sub1	0.92
Snf8	Q9CZ28	Vacuolar-sorting protein SNF8 OS=Mus musculus OX=10090 GN=Snf8 PE=1 SV=1	0.923333333
Stn1	Q8K2X3	CST complex subunit STN1 OS=Mus musculus OX=10090 GN=Stn1 PE=1 SV=2	0.85
Sp1	O89090	Transcription factor Sp1 OS=Mus musculus OX=10090 GN=Sp1 PE=1 SV=2	0.863333333
Exosc5	Q9CRA8	Exosome complex component RRP46 OS=Mus musculus OX=10090 GN=Exosc5 PE=1 SV=1	0.89
Tom1l2	Q5SRX1	TOM1-like protein 2 OS=Mus musculus OX=10090 GN=Tom1l2 PE=1 SV=1	1.263333333
Cyb5a	P56395	Cytochrome b5 OS=Mus musculus OX=10090 GN=Cyb5a PE=1 SV=2	0.933333333
Golph3	Q9CRA5	Golgi phosphoprotein 3 OS=Mus musculus OX=10090 GN=Golph3 PE=1 SV=1	0.98
Phf14	Q9D4H9	PHD finger protein 14 OS=Mus musculus OX=10090 GN=Phf14 PE=1 SV=1	1.016666667
Gid8	Q9D7M1	Glucose-induced degradation protein 8 homolog OS=Mus musculus OX=10090 GN=Gid8 PE=1 SV=1	0.946666667
Mtor	Q9JLN9	Serine/threonine-protein kinase mTOR OS=Mus musculus OX=10090 GN=Mtor PE=1 SV=2	1.113333333
Copz1	P61924	Coatomer subunit zeta-1 OS=Mus musculus OX=10090 GN=Copz1 PE=1 SV=1	0.826666667

Mrps36	Q9CQX8	28S ribosomal protein S36 mitochondrial OS=Mus musculus OX=10090 GN=Mrps36 PE=1 SV=1	0.86
Txndc12	Q9CQU0	Thioredoxin domain-containing protein 12 OS=Mus musculus OX=10090 GN=Txndc12 PE=1 SV=1	0.886666667
Rnps1	Q99M28	RNA-binding protein with serine-rich domain 1 OS=Mus musculus OX=10090 GN=Rnps1 PE=1 SV=1	0.813333333
Snrpf	P62307	Small nuclear ribonucleoprotein F OS=Mus musculus OX=10090 GN=Snrpf PE=1 SV=1	0.883333333
Cog4	Q8R1U1	Conserved oligomeric Golgi complex subunit 4 OS=Mus musculus OX=10090 GN=Cog4 PE=1 SV=1	0.786666667
Mlf2	Q99KX1	Myeloid leukemia factor 2 OS=Mus musculus OX=10090 GN=Mlf2 PE=1 SV=1	0.993333333
Gatd1	Q8BFQ8	Glutamine amidotransferase-like class 1 domain-containing protein 1 OS=Mus musculus OX=10090	0.833333333
Ube2g2	P60605	Ubiquitin-conjugating enzyme E2 G2 OS=Mus musculus OX=10090 GN=Ube2g2 PE=1 SV=1	0.993333333
Fkbp7	O54998	Peptidyl-prolyl cis-trans isomerase FKBP7 OS=Mus musculus OX=10090 GN=Fkbp7 PE=1 SV=1	1.16
Amdhd2	Q8JZV7	N-acetylglucosamine-6-phosphate deacetylase OS=Mus musculus OX=10090 GN=Amdhd2 PE=1 SV=	1.073333333
Cdk7	Q03147	Cyclin-dependent kinase 7 OS=Mus musculus OX=10090 GN=Cdk7 PE=1 SV=2	0.876666667
Nup58	Q8R332	Nucleoporin p58/p45 OS=Mus musculus OX=10090 GN=Nup58 PE=1 SV=1	0.86
Gne	Q91WG8	Bifunctional UDP-N-acetylglucosamine 2-epimerase/N-acetylmannosamine kinase OS=Mus musculu	0.553333333
Pip4k2c	Q91XU3	Phosphatidylinositol 5-phosphate 4-kinase type-2 gamma OS=Mus musculus OX=10090 GN=Pip4k2	0.903333333
Dis3l2	Q8CI75	DIS3-like exonuclease 2 OS=Mus musculus OX=10090 GN=Dis3l2 PE=1 SV=1	0.59
Vps13c	Q8BX70	Vacuolar protein sorting-associated protein 13C OS=Mus musculus OX=10090 GN=Vps13c PE=1 SV=	0.823333333
Pcnp	Q6P8I4	PEST proteolytic signal-containing nuclear protein OS=Mus musculus OX=10090 GN=Pcnp PE=1 SV=	0.8
Gcdh	Q60759	Glutaryl-CoA dehydrogenase mitochondrial OS=Mus musculus OX=10090 GN=Gcdh PE=1 SV=2	0.933333333
Pycr3	Q9DCC4	Pyrroline-5-carboxylate reductase 3 OS=Mus musculus OX=10090 GN=Pycr3 PE=1 SV=2	0.87
Lypla2	Q9WTL7	Acyl-protein thioesterase 2 OS=Mus musculus OX=10090 GN=Lypla2 PE=1 SV=1	0.893333333
Commd5	Q8R395	COMM domain-containing protein 5 OS=Mus musculus OX=10090 GN=Commd5 PE=1 SV=1	0.68
Timm8a1	Q9WVA2	Mitochondrial import inner membrane translocase subunit Tim8 A OS=Mus musculus OX=10090 GI	0.903333333
Ikbkg	O88522	NF-kappa-B essential modulator OS=Mus musculus OX=10090 GN=Ikbkg PE=1 SV=2	0.786666667
Pus10	Q9D3U0	tRNA pseudouridine synthase Pus10 OS=Mus musculus OX=10090 GN=Pus10 PE=1 SV=1	1.02
Nfkb1	P25799	Nuclear factor NF-kappa-B p105 subunit OS=Mus musculus OX=10090 GN=Nfkb1 PE=1 SV=2	0.92
Edf1	Q9JMG1	Endothelial differentiation-related factor 1 OS=Mus musculus OX=10090 GN=Edf1 PE=1 SV=1	0.856666667
Nudcd2	Q9CQ48	NudC domain-containing protein 2 OS=Mus musculus OX=10090 GN=Nudcd2 PE=1 SV=1	0.883333333
Ssr4	Q62186	Translocon-associated protein subunit delta OS=Mus musculus OX=10090 GN=Ssr4 PE=1 SV=1	0.863333333
Timm23	Q9WTQ8	Mitochondrial import inner membrane translocase subunit Tim23 OS=Mus musculus OX=10090 GN	1.14
Rnf114	Q9ET26	E3 ubiquitin-protein ligase RNF114 OS=Mus musculus OX=10090 GN=Rnf114 PE=1 SV=2	0.933333333
Edc3	Q8K2D3	Enhancer of mRNA-decapping protein 3 OS=Mus musculus OX=10090 GN=Edc3 PE=1 SV=1	0.82
Gar1	Q9CY66	H/ACA ribonucleoprotein complex subunit 1 OS=Mus musculus OX=10090 GN=Gar1 PE=1 SV=1	0.95
Clcc1	Q99LI2	Chloride channel CLIC-like protein 1 OS=Mus musculus OX=10090 GN=Clcc1 PE=1 SV=1	1
Lsm8	Q6ZWM4	U6 snRNA-associated Sm-like protein LSm8 OS=Mus musculus OX=10090 GN=Lsm8 PE=1 SV=3	0.91

Txndc17	Q9CQM5	Thioredoxin domain-containing protein 17 OS=Mus musculus OX=10090 GN=Txndc17 PE=1 SV=1	0.92
Polr1d	P97304	DNA-directed RNA polymerases I and III subunit RPAC2 OS=Mus musculus OX=10090 GN=Polr1d PE=	0.74
Glrx5	Q80Y14	Glutaredoxin-related protein 5 mitochondrial OS=Mus musculus OX=10090 GN=Glrx5 PE=1 SV=2	1.05
Sil1	Q9EPK6	Nucleotide exchange factor SIL1 OS=Mus musculus OX=10090 GN=Sil1 PE=1 SV=2	0.953333333
	Q9CXL3	Uncharacterized protein C7orf50 homolog OS=Mus musculus OX=10090 PE=1 SV=3	0.776666667
Akap13	E9Q394	A-kinase anchor protein 13 OS=Mus musculus OX=10090 GN=Akap13 PE=1 SV=1	1.356666667
Ntmt1	Q8R2U4	N-terminal Xaa-Pro-Lys N-methyltransferase 1 OS=Mus musculus OX=10090 GN=Ntmt1 PE=1 SV=3	0.886666667
Rrp7a	Q9D1C9	Ribosomal RNA-processing protein 7 homolog A OS=Mus musculus OX=10090 GN=Rrp7a PE=2 SV=1	0.876666667
Nfs1	Q9Z1J3	Cysteine desulfurase mitochondrial OS=Mus musculus OX=10090 GN=Nfs1 PE=1 SV=3	1.113333333
Cox6b1	P56391	Cytochrome c oxidase subunit 6B1 OS=Mus musculus OX=10090 GN=Cox6b1 PE=1 SV=2	0.88
Clptm1l	Q8BXA5	Cleft lip and palate transmembrane protein 1-like protein OS=Mus musculus OX=10090 GN=Clptm1	0.926666667
Mrps25	Q9D125	28S ribosomal protein S25 mitochondrial OS=Mus musculus OX=10090 GN=Mrps25 PE=1 SV=1	0.856666667
Dapk3	O54784	Death-associated protein kinase 3 OS=Mus musculus OX=10090 GN=Dapk3 PE=1 SV=1	0.943333333
Rps20	P60867	40S ribosomal protein S20 OS=Mus musculus OX=10090 GN=Rps20 PE=1 SV=1	0.93
Spc24	Q9D083	Kinetochore protein Spc24 OS=Mus musculus OX=10090 GN=Spc24 PE=1 SV=1	1.29
Fam111a	Q9D2L9	Protein FAM111A OS=Mus musculus OX=10090 GN=Fam111a PE=2 SV=1	0.963333333
Selenof	Q9ERR7	Selenoprotein F OS=Mus musculus OX=10090 GN=Selenof PE=1 SV=3	0.986666667
Dicer1	Q8R418	Endoribonuclease Dicer OS=Mus musculus OX=10090 GN=Dicer1 PE=1 SV=3	1.27
Ap2s1	P62743	AP-2 complex subunit sigma OS=Mus musculus OX=10090 GN=Ap2s1 PE=1 SV=1	0.936666667
Lgalsl	Q8VED9	Galectin-related protein OS=Mus musculus OX=10090 GN=Lgalsl PE=1 SV=1	0.816666667
Hdac6	Q9Z2V5	Histone deacetylase 6 OS=Mus musculus OX=10090 GN=Hdac6 PE=1 SV=3	0.953333333
H1-0	P10922	Histone H1.0 OS=Mus musculus OX=10090 GN=H1-0 PE=2 SV=4	0.873333333
Sap18	O55128	Histone deacetylase complex subunit SAP18 OS=Mus musculus OX=10090 GN=Sap18 PE=1 SV=1	0.87
Col4a1	P02463	Collagen alpha-1(IV) chain OS=Mus musculus OX=10090 GN=Col4a1 PE=1 SV=4	1.023333333
Rpl38	Q9JJI8	60S ribosomal protein L38 OS=Mus musculus OX=10090 GN=Rpl38 PE=1 SV=3	0.92
Trappc11	B2RXC1	Trafficking protein particle complex subunit 11 OS=Mus musculus OX=10090 GN=Trappc11 PE=1 SV	0.97
Rps25	P62852	40S ribosomal protein S25 OS=Mus musculus OX=10090 GN=Rps25 PE=1 SV=1	0.866666667
Glrx	Q9QUH0	Glutaredoxin-1 OS=Mus musculus OX=10090 GN=Glrx PE=1 SV=3	0.85
Utp11	Q9CZJ1	Probable U3 small nucleolar RNA-associated protein 11 OS=Mus musculus OX=10090 GN=Utp11 PE	0.833333333
Ttk	P35761	Dual specificity protein kinase TTK OS=Mus musculus OX=10090 GN=Ttk PE=1 SV=1	1.416666667
Xpnpep3	B7ZMP1	Xaa-Pro aminopeptidase 3 OS=Mus musculus OX=10090 GN=Xpnpep3 PE=1 SV=1	0.843333333
Mios	Q8VE19	GATOR complex protein MIOS OS=Mus musculus OX=10090 GN=Mios PE=1 SV=2	2.226666667
Cox15	Q8BJ03	Cytochrome c oxidase assembly protein COX15 homolog OS=Mus musculus OX=10090 GN=Cox15 P	0.823333333
Cnot3	Q8K0V4	CCR4-NOT transcription complex subunit 3 OS=Mus musculus OX=10090 GN=Cnot3 PE=1 SV=1	0.84

Ndc80	Q9D0F1	Kinetochore protein NDC80 homolog OS=Mus musculus OX=10090 GN=Ndc80 PE=1 SV=1	0.903333333
Dnajc21	E9Q8D0	DnaJ homolog subfamily C member 21 OS=Mus musculus OX=10090 GN=Dnajc21 PE=1 SV=1	0.82
Rpl27a	P14115	60S ribosomal protein L27a OS=Mus musculus OX=10090 GN=Rpl27a PE=1 SV=5	0.893333333
Zmym2	Q9CU65	Zinc finger MYM-type protein 2 OS=Mus musculus OX=10090 GN=Zmym2 PE=1 SV=3	0.583333333
Cryzl1	Q921W4	Quinone oxidoreductase-like protein 1 OS=Mus musculus OX=10090 GN=Cryzl1 PE=1 SV=1	0.92
Gpx7	Q99LJ6	Glutathione peroxidase 7 OS=Mus musculus OX=10090 GN=Gpx7 PE=1 SV=1	0.843333333
Surf4	Q64310	Surfeit locus protein 4 OS=Mus musculus OX=10090 GN=Surf4 PE=1 SV=1	0.93
Wdr92	Q8BGF3	WD repeat-containing protein 92 OS=Mus musculus OX=10090 GN=Wdr92 PE=1 SV=1	0.873333333
Baz1a	O88379	Bromodomain adjacent to zinc finger domain protein 1A OS=Mus musculus OX=10090 GN=Baz1a PE	1.176666667
Fam192a	Q91WE2	PSME3-interacting protein OS=Mus musculus OX=10090 GN=Psme3ip1 PE=1 SV=1	0.93
Ppp3r1	Q63810	Calcineurin subunit B type 1 OS=Mus musculus OX=10090 GN=Ppp3r1 PE=1 SV=3	1.023333333
Rttn	Q8R4Y8	Rotatin OS=Mus musculus OX=10090 GN=Rttn PE=1 SV=2	1.096666667
Polr2g	P62488	DNA-directed RNA polymerase II subunit RPB7 OS=Mus musculus OX=10090 GN=Polr2g PE=1 SV=1	0.7
Arpc5l	Q9D898	Actin-related protein 2/3 complex subunit 5-like protein OS=Mus musculus OX=10090 GN=Arpc5l P	0.946666667
Plscr3	Q9JIZ9	Phospholipid scramblase 3 OS=Mus musculus OX=10090 GN=Plscr3 PE=1 SV=1	1.03
Wdr48	Q8BH57	WD repeat-containing protein 48 OS=Mus musculus OX=10090 GN=Wdr48 PE=1 SV=1	0.563333333
Pik3c2a	Q61194	Phosphatidylinositol 4-phosphate 3-kinase C2 domain-containing subunit alpha OS=Mus musculus	1.056666667
Dcaf7	P61963	DDB1- and CUL4-associated factor 7 OS=Mus musculus OX=10090 GN=Dcaf7 PE=1 SV=1	1.003333333
Dhrs4	Q99LB2	Dehydrogenase/reductase SDR family member 4 OS=Mus musculus OX=10090 GN=Dhrs4 PE=1 SV=3	0.693333333
Zwint	Q9CQU5	ZW10 interactor OS=Mus musculus OX=10090 GN=Zwint PE=1 SV=1	0.903333333
Bola1	Q9D8S9	BolA-like protein 1 OS=Mus musculus OX=10090 GN=Bola1 PE=1 SV=1	0.943333333
Carmil1	Q6EDY6	F-actin-uncapping protein LRRC16A OS=Mus musculus OX=10090 GN=Carmil1 PE=1 SV=2	1.033333333
Cisd1	Q91WS0	CDGSH iron-sulfur domain-containing protein 1 OS=Mus musculus OX=10090 GN=Cisd1 PE=1 SV=1	0.986666667
Slc9a1	Q61165	Sodium/hydrogen exchanger 1 OS=Mus musculus OX=10090 GN=Slc9a1 PE=1 SV=1	1.216666667
Pfdn1	Q9CWM4	Prefoldin subunit 1 OS=Mus musculus OX=10090 GN=Pfdn1 PE=1 SV=1	0.836666667
Phpt1	Q9DAK9	14 kDa phosphohistidine phosphatase OS=Mus musculus OX=10090 GN=Phpt1 PE=1 SV=1	1.02
Fis1	Q9CQ92	Mitochondrial fission 1 protein OS=Mus musculus OX=10090 GN=Fis1 PE=1 SV=1	0.866666667
Ssu72	Q9CY97	RNA polymerase II subunit A C-terminal domain phosphatase SSU72 OS=Mus musculus OX=10090 G	0.873333333
Cog6	Q8R3I3	Conserved oligomeric Golgi complex subunit 6 OS=Mus musculus OX=10090 GN=Cog6 PE=1 SV=2	1.173333333
Mlst8	Q9DCJ1	Target of rapamycin complex subunit LST8 OS=Mus musculus OX=10090 GN=Mlst8 PE=1 SV=1	0.986666667
Sumo1	P63166	Small ubiquitin-related modifier 1 OS=Mus musculus OX=10090 GN=Sumo1 PE=1 SV=1	0.846666667
Dhps	Q3TXU5	Deoxyhypusine synthase OS=Mus musculus OX=10090 GN=Dhps PE=1 SV=2	0.94
Mrpl44	Q9CY73	39S ribosomal protein L44 mitochondrial OS=Mus musculus OX=10090 GN=Mrpl44 PE=1 SV=3	1.293333333
Sdhaf2	Q8C6I2	Succinate dehydrogenase assembly factor 2 mitochondrial OS=Mus musculus OX=10090 GN=Sdhaf	0.836666667

Ints10	Q8K2A7	Integrator complex subunit 10 OS=Mus musculus OX=10090 GN=Ints10 PE=1 SV=3	1.886666667
Tmx4	Q8C0L0	Thioredoxin-related transmembrane protein 4 OS=Mus musculus OX=10090 GN=Tmx4 PE=1 SV=2	0.83
Tarbp2	P97473	RISC-loading complex subunit TARBP2 OS=Mus musculus OX=10090 GN=Tarbp2 PE=1 SV=2	1.296666667
Guk1	Q64520	Guanylate kinase OS=Mus musculus OX=10090 GN=Guk1 PE=1 SV=2	0.81
Uck2	Q99PM9	Uridine-cytidine kinase 2 OS=Mus musculus OX=10090 GN=Uck2 PE=1 SV=1	0.906666667
Pin4	Q9CWW6	Peptidyl-prolyl cis-trans isomerase NIMA-interacting 4 OS=Mus musculus OX=10090 GN=Pin4 PE=1	0.793333333
Pdk1	Q8BFP9	[Pyruvate dehydrogenase (acetyl-transferring)] kinase isozyme 1 mitochondrial OS=Mus musculus O	0.866666667
Cnn1	Q08091	Calponin-1 OS=Mus musculus OX=10090 GN=Cnn1 PE=1 SV=1	0.916666667
Psen1	P49769	Presenilin-1 OS=Mus musculus OX=10090 GN=Psen1 PE=1 SV=1	1.23
Cactin	Q9CS00	Cactin OS=Mus musculus OX=10090 GN=Cactin PE=1 SV=2	1.236666667
Amacr	O09174	Alpha-methylacyl-CoA racemase OS=Mus musculus OX=10090 GN=Amacr PE=1 SV=4	0.846666667
Knop1	Q9Z2Q2	Lysine-rich nucleolar protein 1 OS=Mus musculus OX=10090 GN=Knop1 PE=1 SV=2	0.83
Slc39a10	Q6P5F6	Zinc transporter ZIP10 OS=Mus musculus OX=10090 GN=Slc39a10 PE=1 SV=1	0.956666667
Hebp1	Q9R257	Heme-binding protein 1 OS=Mus musculus OX=10090 GN=Hebp1 PE=1 SV=2	0.69
Hagh	Q99KB8	Hydroxyacylglutathione hydrolase mitochondrial OS=Mus musculus OX=10090 GN=Hagh PE=1 SV=	0.75
Fpgs	P48760	Folylpolyglutamate synthase mitochondrial OS=Mus musculus OX=10090 GN=Fpgs PE=1 SV=3	1.033333333
Exosc1	Q9DAA6	Exosome complex component CSL4 OS=Mus musculus OX=10090 GN=Exosc1 PE=1 SV=1	0.91
Spryd7	Q3TFQ1	SPRY domain-containing protein 7 OS=Mus musculus OX=10090 GN=Spryd7 PE=1 SV=2	0.966666667
Tpbg	Q9Z0L0	Trophoblast glycoprotein OS=Mus musculus OX=10090 GN=Tpbg PE=1 SV=3	1.373333333
Hsd17b7	O88736	3-keto-steroid reductase/17-beta-hydroxysteroid dehydrogenase 7 OS=Mus musculus OX=10090 GN	1.056666667
Sdf2l1	Q9ESP1	Stromal cell-derived factor 2-like protein 1 OS=Mus musculus OX=10090 GN=Sdf2l1 PE=1 SV=2	1.973333333
Uqcrb	Q9D855	Cytochrome b-c1 complex subunit 7 OS=Mus musculus OX=10090 GN=Uqcrb PE=1 SV=3	0.956666667
Ass1	P16460	Argininosuccinate synthase OS=Mus musculus OX=10090 GN=Ass1 PE=1 SV=1	1.156666667
Imp4	Q8VHZ7	U3 small nucleolar ribonucleoprotein protein IMP4 OS=Mus musculus OX=10090 GN=Imp4 PE=2 SV	0.92
F2r	P30558	Proteinase-activated receptor 1 OS=Mus musculus OX=10090 GN=F2r PE=1 SV=2	0.963333333
Aar2	Q9D2V5	Protein AAR2 homolog OS=Mus musculus OX=10090 GN=Aar2 PE=1 SV=3	0.58
Rida	P52760	2-iminobutanoate/2-iminopropanoate deaminase OS=Mus musculus OX=10090 GN=Rida PE=1 SV=3	1.06
Ttc3	O88196	E3 ubiquitin-protein ligase TTC3 OS=Mus musculus OX=10090 GN=Ttc3 PE=1 SV=2	1.473333333
Stx8	O88983	Syntaxin-8 OS=Mus musculus OX=10090 GN=Stx8 PE=1 SV=1	0.763333333
Gtf3c5	Q8R2T8	General transcription factor 3C polypeptide 5 OS=Mus musculus OX=10090 GN=Gtf3c5 PE=2 SV=2	0.95
Zyg11b	Q3UFS0	Protein zyg-11 homolog B OS=Mus musculus OX=10090 GN=Zyg11b PE=1 SV=2	0.68
Nt5c3a	Q9D020	Cytosolic 5'-nucleotidase 3A OS=Mus musculus OX=10090 GN=Nt5c3a PE=1 SV=4	1.076666667
Phf5a	P83870	PHD finger-like domain-containing protein 5A OS=Mus musculus OX=10090 GN=Phf5a PE=1 SV=1	0.916666667
Stard4	Q99JV5	StAR-related lipid transfer protein 4 OS=Mus musculus OX=10090 GN=Stard4 PE=1 SV=1	0.793333333

F3	P20352	Tissue factor OS=Mus musculus OX=10090 GN=F3 PE=1 SV=2	0.593333333
Mtmr10	Q7TPM9	Myotubularin-related protein 10 OS=Mus musculus OX=10090 GN=Mtmr10 PE=1 SV=2	1.13
Rpl36a	P83882	60S ribosomal protein L36a OS=Mus musculus OX=10090 GN=Rpl36a PE=3 SV=2	0.87
Lpcat3	Q91V01	Lysophospholipid acyltransferase 5 OS=Mus musculus OX=10090 GN=Lpcat3 PE=1 SV=1	0.853333333
Wdr45b	Q9CR39	WD repeat domain phosphoinositide-interacting protein 3 OS=Mus musculus OX=10090 GN=Wdr4	0.866666667
Slirp	Q9D8T7	SRA stem-loop-interacting RNA-binding protein mitochondrial OS=Mus musculus OX=10090 GN=Sl	0.943333333
Uevld	Q3U1V6	Ubiquitin-conjugating enzyme E2 variant 3 OS=Mus musculus OX=10090 GN=Uevld PE=1 SV=1	1.326666667
Mrpl46	Q9EQI8	39S ribosomal protein L46 mitochondrial OS=Mus musculus OX=10090 GN=Mrpl46 PE=1 SV=1	1.01
Frmd8	Q3UFK8	FERM domain-containing protein 8 OS=Mus musculus OX=10090 GN=Frmd8 PE=1 SV=2	0.96
Hmgcl	P38060	Hydroxymethylglutaryl-CoA lyase mitochondrial OS=Mus musculus OX=10090 GN=Hmgcl PE=1 SV=	0.833333333
Fkbp11	Q9D1M7	Peptidyl-prolyl cis-trans isomerase FKBP11 OS=Mus musculus OX=10090 GN=Fkbp11 PE=1 SV=1	0.84
Cfap20	Q8BTU1	Cilia- and flagella-associated protein 20 OS=Mus musculus OX=10090 GN=Cfap20 PE=1 SV=1	1.316666667
Ublcp1	Q8BGR9	Ubiquitin-like domain-containing CTD phosphatase 1 OS=Mus musculus OX=10090 GN=Ublcp1 PE=	0.803333333
Melk	Q61846	Maternal embryonic leucine zipper kinase OS=Mus musculus OX=10090 GN=Melk PE=1 SV=2	1.35
Amotl2	Q8K371	Angiomotin-like protein 2 OS=Mus musculus OX=10090 GN=Amotl2 PE=1 SV=1	0.82
Znf598	Q80YR4	E3 ubiquitin-protein ligase ZNF598 OS=Mus musculus OX=10090 GN=Znf598 PE=1 SV=1	0.77
Sdf2	Q9DCT5	Stromal cell-derived factor 2 OS=Mus musculus OX=10090 GN=Sdf2 PE=1 SV=1	0.866666667
Tmem87a	Q8BXN9	Transmembrane protein 87A OS=Mus musculus OX=10090 GN=Tmem87a PE=1 SV=1	0.776666667
Cdk5rap2	Q8K389	CDK5 regulatory subunit-associated protein 2 OS=Mus musculus OX=10090 GN=Cdk5rap2 PE=1 SV=	1.436666667
Rpp38	Q80UU2	Ribonuclease P protein subunit p38 OS=Mus musculus OX=10090 GN=Rpp38 PE=1 SV=1	1.153333333
Riok1	Q922Q2	Serine/threonine-protein kinase RIO1 OS=Mus musculus OX=10090 GN=Riok1 PE=1 SV=2	0.796666667
Adi1	Q99JT9	1 2-dihydroxy-3-keto-5-methylthiopentene dioxygenase OS=Mus musculus OX=10090 GN=Adi1 PE=	1.13
Taf9	Q8VI33	Transcription initiation factor TFIID subunit 9 OS=Mus musculus OX=10090 GN=Taf9 PE=1 SV=1	1.143333333
Prune1	Q8BIW1	Exopolyphosphatase PRUNE1 OS=Mus musculus OX=10090 GN=Prune1 PE=1 SV=1	0.916666667
Ddx28	Q9CWT6	Probable ATP-dependent RNA helicase DDX28 OS=Mus musculus OX=10090 GN=Ddx28 PE=2 SV=2	0.826666667
Uqcrh	P99028	Cytochrome b-c1 complex subunit 6 mitochondrial OS=Mus musculus OX=10090 GN=Uqcrh PE=1 S	0.883333333
Rpia	P47968	Ribose-5-phosphate isomerase OS=Mus musculus OX=10090 GN=Rpia PE=1 SV=2	1.11
Nipsnap3b	Q9CQE1	Protein NipSnap homolog 3B OS=Mus musculus OX=10090 GN=Nipsnap3b PE=1 SV=1	0.873333333
Sos1	Q62245	Son of sevenless homolog 1 OS=Mus musculus OX=10090 GN=Sos1 PE=1 SV=2	1.343333333
Slc44a2	Q8BY89	Choline transporter-like protein 2 OS=Mus musculus OX=10090 GN=Slc44a2 PE=1 SV=2	0.713333333
Dctn6	Q9WUB4	Dynactin subunit 6 OS=Mus musculus OX=10090 GN=Dctn6 PE=1 SV=1	0.73
Pym1	Q8CHP5	Partner of Y14 and mago OS=Mus musculus OX=10090 GN=Pym1 PE=1 SV=2	0.903333333
Itga3	Q62470	Integrin alpha-3 OS=Mus musculus OX=10090 GN=Itga3 PE=1 SV=1	1.34
Tjap1	Q9DCD5	Tight junction-associated protein 1 OS=Mus musculus OX=10090 GN=Tjap1 PE=1 SV=1	0.893333333

Setdb1	O88974	Histone-lysine N-methyltransferase SETDB1 OS=Mus musculus OX=10090 GN=Setdb1 PE=1 SV=1	1.316666667
Txndc9	Q9CQ79	Thioredoxin domain-containing protein 9 OS=Mus musculus OX=10090 GN=Txndc9 PE=1 SV=1	1.246666667
Zadh2	Q8BGC4	Prostaglandin reductase-3 OS=Mus musculus OX=10090 GN=Zadh2 PE=1 SV=1	0.923333333
Pef1	Q8BFY6	Peflin OS=Mus musculus OX=10090 GN=Pef1 PE=1 SV=1	0.973333333
Nqo1	Q64669	NAD(P)H dehydrogenase [quinone] 1 OS=Mus musculus OX=10090 GN=Nqo1 PE=1 SV=3	0.953333333
Rnf181	Q9CY62	E3 ubiquitin-protein ligase RNF181 OS=Mus musculus OX=10090 GN=Rnf181 PE=1 SV=1	1.23
Slain2	Q8CI08	SLAIN motif-containing protein 2 OS=Mus musculus OX=10090 GN=Slain2 PE=1 SV=2	1.323333333
Ndufb10	Q9DCS9	NADH dehydrogenase [ubiquinone] 1 beta subcomplex subunit 10 OS=Mus musculus OX=10090 GN	0.846666667
Itsn1	Q9Z0R4	Intersectin-1 OS=Mus musculus OX=10090 GN=Itsn1 PE=1 SV=2	0.576666667
Commd10	Q8JZY2	COMM domain-containing protein 10 OS=Mus musculus OX=10090 GN=Commd10 PE=1 SV=1	1.05
Ppih	Q9D868	Peptidyl-prolyl cis-trans isomerase H OS=Mus musculus OX=10090 GN=Ppih PE=1 SV=1	0.893333333
Chm	Q9QXG2	Rab proteins geranylgeranyltransferase component A 1 OS=Mus musculus OX=10090 GN=Chm PE=1	0.823333333
Gfer	P56213	FAD-linked sulfhydryl oxidase ALR OS=Mus musculus OX=10090 GN=Gfer PE=1 SV=2	0.766666667
Sec62	Q8BU14	Translocation protein SEC62 OS=Mus musculus OX=10090 GN=Sec62 PE=1 SV=1	0.833333333
Lsm4	Q9QXA5	U6 snRNA-associated Sm-like protein LSm4 OS=Mus musculus OX=10090 GN=Lsm4 PE=1 SV=1	0.953333333
Slmap	Q3URD3	Sarcolemmal membrane-associated protein OS=Mus musculus OX=10090 GN=Slmap PE=1 SV=2	0.75
Tango6	Q8C3S2	Transport and Golgi organization protein 6 homolog OS=Mus musculus OX=10090 GN=Tango6 PE=1	2.663333333
Impad1	Q80V26	Golgi-resident adenosine 3' 5'-bisphosphate 3'-phosphatase OS=Mus musculus OX=10090 GN=Bpnt2	1.08
Knstrn	Q9D9Z1	Small kinetochore-associated protein OS=Mus musculus OX=10090 GN=Knstrn PE=2 SV=2	0.823333333
Phlpp1	Q8CHE4	PH domain leucine-rich repeat-containing protein phosphatase 1 OS=Mus musculus OX=10090 GN=	1.21
Sufu	Q9Z0P7	Suppressor of fused homolog OS=Mus musculus OX=10090 GN=Sufu PE=1 SV=1	0.846666667
Nol12	Q8BG17	Nucleolar protein 12 OS=Mus musculus OX=10090 GN=Nol12 PE=1 SV=1	0.92
Haus3	Q8QZX2	HAUS augmin-like complex subunit 3 OS=Mus musculus OX=10090 GN=Haus3 PE=1 SV=1	1.373333333
Usp38	Q8BW70	Ubiquitin carboxyl-terminal hydrolase 38 OS=Mus musculus OX=10090 GN=Usp38 PE=1 SV=2	0.93
Foxk1	P42128	Forkhead box protein K1 OS=Mus musculus OX=10090 GN=Foxk1 PE=1 SV=2	0.465
Utp23	Q9CX11	rRNA-processing protein UTP23 homolog OS=Mus musculus OX=10090 GN=Utp23 PE=2 SV=1	0.973333333
Mrpl23	O35972	39S ribosomal protein L23 mitochondrial OS=Mus musculus OX=10090 GN=Mrpl23 PE=1 SV=1	0.953333333
Sorcs2	Q9EPR5	VPS10 domain-containing receptor SorCS2 OS=Mus musculus OX=10090 GN=Sorcs2 PE=1 SV=2	0.543333333
Atp5me	Q06185	ATP synthase subunit e mitochondrial OS=Mus musculus OX=10090 GN=Atp5me PE=1 SV=2	0.896666667
Fam177a1	Q8BR63	Protein FAM177A1 OS=Mus musculus OX=10090 GN=Fam177a1 PE=1 SV=1	0.93
Mdp1	Q9D967	Magnesium-dependent phosphatase 1 OS=Mus musculus OX=10090 GN=Mdp1 PE=1 SV=1	0.726666667
Rabl6	Q5U3K5	Rab-like protein 6 OS=Mus musculus OX=10090 GN=Rabl6 PE=1 SV=2	0.87
Lrrc8a	Q80WG5	Volume-regulated anion channel subunit LRRC8A OS=Mus musculus OX=10090 GN=Lrrc8a PE=1 SV=	2.9
Pam	P97467	Peptidyl-glycine alpha-amidating monooxygenase OS=Mus musculus OX=10090 GN=Pam PE=1 SV=2	2.783333333

Stx6	Q9JKK1	Syntaxin-6 OS=Mus musculus OX=10090 GN=Stx6 PE=1 SV=1	1.063333333
Lrch3	Q8BVU0	DISP complex protein LRCH3 OS=Mus musculus OX=10090 GN=Lrch3 PE=1 SV=3	1.245
Magt1	Q9CQY5	Magnesium transporter protein 1 OS=Mus musculus OX=10090 GN=Magt1 PE=1 SV=1	0.946666667
Acyp1	P56376	Acylphosphatase-1 OS=Mus musculus OX=10090 GN=Acyp1 PE=1 SV=2	0.733333333
Gpx8	Q9D7B7	Probable glutathione peroxidase 8 OS=Mus musculus OX=10090 GN=Gpx8 PE=1 SV=1	0.903333333
Tram1	Q91V04	Translocating chain-associated membrane protein 1 OS=Mus musculus OX=10090 GN=Tram1 PE=1 S	0.93
Tsen34	Q8BMZ5	tRNA-splicing endonuclease subunit Sen34 OS=Mus musculus OX=10090 GN=Tsen34 PE=2 SV=2	0.813333333
Gkap1	Q9JMB0	G kinase-anchoring protein 1 OS=Mus musculus OX=10090 GN=Gkap1 PE=1 SV=1	0.906666667
Steap1	Q9CWR7	Metalloreductase STEAP1 OS=Mus musculus OX=10090 GN=Steap1 PE=1 SV=2	1.653333333
Srp9	P49962	Signal recognition particle 9 kDa protein OS=Mus musculus OX=10090 GN=Srp9 PE=1 SV=2	0.88
Gpx1	P11352	Glutathione peroxidase 1 OS=Mus musculus OX=10090 GN=Gpx1 PE=1 SV=2	0.863333333
Ptpra	P18052	Receptor-type tyrosine-protein phosphatase alpha OS=Mus musculus OX=10090 GN=Ptpra PE=1 SV=	0.956666667
Mrps6	P58064	28S ribosomal protein S6 mitochondrial OS=Mus musculus OX=10090 GN=Mrps6 PE=1 SV=3	0.763333333
Ahsa2	Q8N9S3	Activator of 90 kDa heat shock protein ATPase homolog 2 OS=Mus musculus OX=10090 GN=Ahsa2 P	0.773333333
Rabggtb	P53612	Geranylgeranyl transferase type-2 subunit beta OS=Mus musculus OX=10090 GN=Rabggtb PE=1 SV=:	0.956666667
Ufc1	Q9CR09	Ubiquitin-fold modifier-conjugating enzyme 1 OS=Mus musculus OX=10090 GN=Ufc1 PE=1 SV=1	0.906666667
Gpaa1	Q9WTK3	Glycosylphosphatidylinositol anchor attachment 1 protein OS=Mus musculus OX=10090 GN=Gpaa:	1.33
Pdhx	Q8BKZ9	Pyruvate dehydrogenase protein X component mitochondrial OS=Mus musculus OX=10090 GN=Pd	0.823333333
Vwa8	Q8CC88	von Willebrand factor A domain-containing protein 8 OS=Mus musculus OX=10090 GN=Vwa8 PE=1	1
Ergic3	Q9CQE7	Endoplasmic reticulum-Golgi intermediate compartment protein 3 OS=Mus musculus OX=10090 G	0.896666667
Toe1	Q9D2E2	Target of EGR1 protein 1 OS=Mus musculus OX=10090 GN=Toe1 PE=1 SV=1	0.9
Arid2	E9Q7E2	AT-rich interactive domain-containing protein 2 OS=Mus musculus OX=10090 GN=Arid2 PE=1 SV=1	1.596666667
Mecp2	Q9Z2D6	Methyl-CpG-binding protein 2 OS=Mus musculus OX=10090 GN=Mecp2 PE=1 SV=1	0.526666667
Otulin	Q3UCV8	Ubiquitin thioesterase otulin OS=Mus musculus OX=10090 GN=Otulin PE=1 SV=1	0.753333333
Derl1	Q99J56	Derlin-1 OS=Mus musculus OX=10090 GN=Derl1 PE=1 SV=1	0.87
Morf4l2	Q9R0Q4	Mortality factor 4-like protein 2 OS=Mus musculus OX=10090 GN=Morf4l2 PE=1 SV=1	1.72
Rwdd4	Q9CPR1	RWD domain-containing protein 4 OS=Mus musculus OX=10090 GN=Rwdd4 PE=2 SV=1	1.023333333
Tm9sf1	Q9DBU0	Transmembrane 9 superfamily member 1 OS=Mus musculus OX=10090 GN=Tm9sf1 PE=2 SV=2	1.446666667
Casp7	P97864	Caspase-7 OS=Mus musculus OX=10090 GN=Casp7 PE=1 SV=2	1.09
Ascc1	Q9D8Z1	Activating signal cointegrator 1 complex subunit 1 OS=Mus musculus OX=10090 GN=Ascc1 PE=1 SV	1.15
Apmap	Q9D7N9	Adipocyte plasma membrane-associated protein OS=Mus musculus OX=10090 GN=Apmap PE=1 SV=	0.66
Surf6	P70279	Surfeit locus protein 6 OS=Mus musculus OX=10090 GN=Surf6 PE=1 SV=1	1.29
Eif1b	Q9CXU9	Eukaryotic translation initiation factor 1b OS=Mus musculus OX=10090 GN=Eif1b PE=1 SV=2	1.55
Mob1a	Q921Y0	MOB kinase activator 1A OS=Mus musculus OX=10090 GN=Mob1a PE=2 SV=3	

Mob1b	Q8BPB0	MOB kinase activator 1B OS=Mus musculus OX=10090 GN=Mob1b PE=1 SV=3	1.196666667
Mapk9	Q9WTU6	Mitogen-activated protein kinase 9 OS=Mus musculus OX=10090 GN=Mapk9 PE=1 SV=2	
Ube2v2	Q9D2M8	Ubiquitin-conjugating enzyme E2 variant 2 OS=Mus musculus OX=10090 GN=Ube2v2 PE=1 SV=4	0.963333333
Atp4a	Q64436	Potassium-transporting ATPase alpha chain 1 OS=Mus musculus OX=10090 GN=Atp4a PE=1 SV=3	
Ephb3	P54754	Ephrin type-B receptor 3 OS=Mus musculus OX=10090 GN=Ephb3 PE=1 SV=2	
Mapk8	Q91Y86	Mitogen-activated protein kinase 8 OS=Mus musculus OX=10090 GN=Mapk8 PE=1 SV=1	
Fyn	P39688	Tyrosine-protein kinase Fyn OS=Mus musculus OX=10090 GN=Fyn PE=1 SV=4	0.75
Cab39l	Q9DB16	Calcium-binding protein 39-like OS=Mus musculus OX=10090 GN=Cab39l PE=1 SV=3	
Csnk1e	Q9JMK2	Casein kinase I isoform epsilon OS=Mus musculus OX=10090 GN=Csnk1e PE=1 SV=2	
Plxna3	P70208	Plexin-A3 OS=Mus musculus OX=10090 GN=Plxna3 PE=1 SV=2	
Mbnl1	Q9JKP5	Muscleblind-like protein 1 OS=Mus musculus OX=10090 GN=Mbnl1 PE=1 SV=1	
Rps27	Q6ZWU9	40S ribosomal protein S27 OS=Mus musculus OX=10090 GN=Rps27 PE=1 SV=3	0.82
Rps27l	Q6ZWY3	40S ribosomal protein S27-like OS=Mus musculus OX=10090 GN=Rps27l PE=1 SV=3	0.79
Rprd1a	Q8VDS4	Regulation of nuclear pre-mRNA domain-containing protein 1A OS=Mus musculus OX=10090 GN=R	0.88
Smad1	P70340	Mothers against decapentaplegic homolog 1 OS=Mus musculus OX=10090 GN=Smad1 PE=1 SV=2	0.936666667
Rala	P63321	Ras-related protein Ral-A OS=Mus musculus OX=10090 GN=Rala PE=1 SV=1	0.723333333
Mapre3	Q6PER3	Microtubule-associated protein RP/EB family member 3 OS=Mus musculus OX=10090 GN=Mapre3 F	0.94
Cbx3	P23198	Chromobox protein homolog 3 OS=Mus musculus OX=10090 GN=Cbx3 PE=1 SV=2	0.9
Tor1aip2	Q8BYU6	Torsin-1A-interacting protein 2 OS=Mus musculus OX=10090 GN=Tor1aip2 PE=1 SV=1	0.93
Rab35	Q6PHN9	Ras-related protein Rab-35 OS=Mus musculus OX=10090 GN=Rab35 PE=1 SV=1	0.916666667
Kif1c	O35071	Kinesin-like protein KIF1C OS=Mus musculus OX=10090 GN=Kif1c PE=1 SV=2	1.465
Tmed4	Q8R1V4	Transmembrane emp24 domain-containing protein 4 OS=Mus musculus OX=10090 GN=Tmed4 PE=	0.853333333
Scaf4	Q7TSH6	SR-related and CTD-associated factor 4 OS=Mus musculus OX=10090 GN=Scaf4 PE=1 SV=1	1.246666667
Kif1b	Q60575	Kinesin-like protein KIF1B OS=Mus musculus OX=10090 GN=Kif1b PE=1 SV=2	1.996666667
Cbx1	P83917	Chromobox protein homolog 1 OS=Mus musculus OX=10090 GN=Cbx1 PE=1 SV=1	0.976666667
Ppp4r3b	Q922R5	Serine/threonine-protein phosphatase 4 regulatory subunit 3B OS=Mus musculus OX=10090 GN=P	1.01
Smarcd1	Q61466	SWI/SNF-related matrix-associated actin-dependent regulator of chromatin subfamily D member 1	1.436666667
Foxp1	P58462	Forkhead box protein P1 OS=Mus musculus OX=10090 GN=Foxp1 PE=1 SV=1	0.896666667
Gyg1	Q9R062	Glycogenin-1 OS=Mus musculus OX=10090 GN=Gyg1 PE=1 SV=3	0.843333333
H1-5	P43276	Histone H1.5 OS=Mus musculus OX=10090 GN=H1-5 PE=1 SV=2	0.81
Dip2a	Q8BWT5	Disco-interacting protein 2 homolog A OS=Mus musculus OX=10090 GN=Dip2a PE=1 SV=3	1.056666667
Atp6v0a1	Q9Z1G4	V-type proton ATPase 116 kDa subunit a1 OS=Mus musculus OX=10090 GN=Atp6v0a1 PE=1 SV=3	0.936666667
Otud7b	B2RUR8	OTU domain-containing protein 7B OS=Mus musculus OX=10090 GN=Otud7b PE=1 SV=1	0.943333333
Cdk13	Q69ZA1	Cyclin-dependent kinase 13 OS=Mus musculus OX=10090 GN=Cdk13 PE=1 SV=3	1.443333333

Tra2a	Q6PFR5	Transformer-2 protein homolog alpha OS=Mus musculus OX=10090 GN=Tra2a PE=1 SV=1	1
Unc119b	Q8C4B4	Protein unc-119 homolog B OS=Mus musculus OX=10090 GN=Unc119b PE=1 SV=1	1.163333333
Pip4p2	Q9CZX7	Type 2 phosphatidylinositol 4 5-bisphosphate 4-phosphatase OS=Mus musculus OX=10090 GN=Pip	0.726666667
Tulp3	O88413	Tubby-related protein 3 OS=Mus musculus OX=10090 GN=Tulp3 PE=1 SV=1	1.623333333
Cyld	Q80TQ2	Ubiquitin carboxyl-terminal hydrolase CYLD OS=Mus musculus OX=10090 GN=Cyld PE=1 SV=2	1.19
Ncs1	Q8BNY6	Neuronal calcium sensor 1 OS=Mus musculus OX=10090 GN=Ncs1 PE=1 SV=3	2.213333333
Mrtfa	Q8K4J6	Myocardin-related transcription factor A OS=Mus musculus OX=10090 GN=Mrtfa PE=1 SV=2	1.266666667
Abr	Q5SSL4	Active breakpoint cluster region-related protein OS=Mus musculus OX=10090 GN=Abr PE=1 SV=1	0.786666667
Ncor1	Q60974	Nuclear receptor corepressor 1 OS=Mus musculus OX=10090 GN=Ncor1 PE=1 SV=1	0.795
Dhx8	A2A4P0	ATP-dependent RNA helicase DHX8 OS=Mus musculus OX=10090 GN=Dhx8 PE=2 SV=1	0.923333333
Tbc1d1	Q60949	TBC1 domain family member 1 OS=Mus musculus OX=10090 GN=Tbc1d1 PE=1 SV=3	0.836666667
Gmpr2	Q99L27	GMP reductase 2 OS=Mus musculus OX=10090 GN=Gmpr2 PE=1 SV=2	0.55
Fcsk	Q7TMC8	L-fucose kinase OS=Mus musculus OX=10090 GN=Fcsk PE=1 SV=1	0.92
Cltb	Q6IRU5	Clathrin light chain B OS=Mus musculus OX=10090 GN=Cltb PE=1 SV=1	1.043333333
Hspbap1	Q8BK58	HSPB1-associated protein 1 OS=Mus musculus OX=10090 GN=Hspbap1 PE=1 SV=2	0.86
Rpl12	P35979	60S ribosomal protein L12 OS=Mus musculus OX=10090 GN=Rpl12 PE=1 SV=2	0.876666667
S100a10	P08207	Protein S100-A10 OS=Mus musculus OX=10090 GN=S100a10 PE=1 SV=2	0.953333333
ATP5IF1	O35143	ATPase inhibitor mitochondrial OS=Mus musculus OX=10090 GN=ATP5IF1 PE=1 SV=2	0.92
Basp1	Q91XV3	Brain acid soluble protein 1 OS=Mus musculus OX=10090 GN=Basp1 PE=1 SV=3	0.73
Npm3	Q9CPP0	Nucleoplasmin-3 OS=Mus musculus OX=10090 GN=Npm3 PE=1 SV=3	0.87
Fkbp1a	P26883	Peptidyl-prolyl cis-trans isomerase FKBP1A OS=Mus musculus OX=10090 GN=Fkbp1a PE=1 SV=2	0.89
Hadh	Q61425	Hydroxyacyl-coenzyme A dehydrogenase mitochondrial OS=Mus musculus OX=10090 GN=Hadh PE	1.02
Itgb5	O70309	Integrin beta-5 OS=Mus musculus OX=10090 GN=Itgb5 PE=1 SV=2	1.106666667
Heatr6	Q6P1G0	HEAT repeat-containing protein 6 OS=Mus musculus OX=10090 GN=Heatr6 PE=1 SV=1	0.803333333
Sra1	Q80VJ2	Steroid receptor RNA activator 1 OS=Mus musculus OX=10090 GN=Sra1 PE=1 SV=3	0.903333333
Tmem263	Q9DAM7	Transmembrane protein 263 OS=Mus musculus OX=10090 GN=Tmem263 PE=1 SV=1	0.853333333
Apool	Q78IK4	MICOS complex subunit Mic27 OS=Mus musculus OX=10090 GN=Apool PE=1 SV=1	0.786666667
Rpl21	O09167	60S ribosomal protein L21 OS=Mus musculus OX=10090 GN=Rpl21 PE=1 SV=3	0.923333333
Alb	P07724	Albumin OS=Mus musculus OX=10090 GN=Alb PE=1 SV=3	0.016666667
Cpox	P36552	Oxygen-dependent coproporphyrinogen-III oxidase mitochondrial OS=Mus musculus OX=10090 GI	0.94
Snrpd3	P62320	Small nuclear ribonucleoprotein Sm D3 OS=Mus musculus OX=10090 GN=Snrpd3 PE=1 SV=1	1.043333333
Osbpl9	A2A8Z1	Oxysterol-binding protein-related protein 9 OS=Mus musculus OX=10090 GN=Osbpl9 PE=1 SV=1	0.813333333
C1qbp	O35658	Complement component 1 Q subcomponent-binding protein mitochondrial OS=Mus musculus OX	0.903333333
Atp6v1g1	Q9CR51	V-type proton ATPase subunit G 1 OS=Mus musculus OX=10090 GN=Atp6v1g1 PE=1 SV=3	0.916666667

Rnf126	Q91YL2	E3 ubiquitin-protein ligase RNF126 OS=Mus musculus OX=10090 GN=Rnf126 PE=1 SV=1	1.07
Rpl37a	P61514	60S ribosomal protein L37a OS=Mus musculus OX=10090 GN=Rpl37a PE=1 SV=2	0.94
Znf330	Q922H9	Zinc finger protein 330 OS=Mus musculus OX=10090 GN=Znf330 PE=1 SV=1	0.75
Akap9	Q70FJ1	A-kinase anchor protein 9 OS=Mus musculus OX=10090 GN=Akap9 PE=1 SV=2	1.07
Banf1	O54962	Barrier-to-autointegration factor OS=Mus musculus OX=10090 GN=Banf1 PE=1 SV=1	0.85
Ppp1r14b	Q62084	Protein phosphatase 1 regulatory subunit 14B OS=Mus musculus OX=10090 GN=Ppp1r14b PE=1 SV	0.86
Clns1a	Q61189	Methylosome subunit pICln OS=Mus musculus OX=10090 GN=Clns1a PE=1 SV=1	0.926666667
Med12	A2AGH6	Mediator of RNA polymerase II transcription subunit 12 OS=Mus musculus OX=10090 GN=Med12 PI	1.5
Rbms2	Q8VC70	RNA-binding motif single-stranded-interacting protein 2 OS=Mus musculus OX=10090 GN=Rbms2 F	0.88
Arhgdib	Q61599	Rho GDP-dissociation inhibitor 2 OS=Mus musculus OX=10090 GN=Arhgdib PE=1 SV=3	0.926666667
Pxdn	Q3UQ28	Peroxidasin homolog OS=Mus musculus OX=10090 GN=Pxdn PE=1 SV=2	0.656666667
Cggbp1	Q8BHG9	CGG triplet repeat-binding protein 1 OS=Mus musculus OX=10090 GN=Cggbp1 PE=1 SV=1	0.946666667
Nop16	Q9CPT5	Nucleolar protein 16 OS=Mus musculus OX=10090 GN=Nop16 PE=1 SV=1	1.03
Tmed2	Q9R0Q3	Transmembrane emp24 domain-containing protein 2 OS=Mus musculus OX=10090 GN=Tmed2 PE=	0.853333333
Eif2b1	Q99LC8	Translation initiation factor eIF-2B subunit alpha OS=Mus musculus OX=10090 GN=Eif2b1 PE=1 SV=	0.956666667
Pdcd10	Q8VE70	Programmed cell death protein 10 OS=Mus musculus OX=10090 GN=Pdcd10 PE=1 SV=1	0.83
Msmo1	Q9CRA4	Methylsterol monooxygenase 1 OS=Mus musculus OX=10090 GN=Msmo1 PE=1 SV=1	0.876666667
Kdm2a	P59997	Lysine-specific demethylase 2A OS=Mus musculus OX=10090 GN=Kdm2a PE=1 SV=2	0.906666667
Poglut3	G5E897	Protein O-glucosyltransferase 3 OS=Mus musculus OX=10090 GN=Poglut3 PE=1 SV=1	1.19
Timm50	Q9D880	Mitochondrial import inner membrane translocase subunit TIM50 OS=Mus musculus OX=10090 GN	0.826666667
Wasl	Q91YD9	Neural Wiskott-Aldrich syndrome protein OS=Mus musculus OX=10090 GN=Wasl PE=1 SV=1	0.926666667
Rpl14	Q9CR57	60S ribosomal protein L14 OS=Mus musculus OX=10090 GN=Rpl14 PE=1 SV=3	0.913333333
Pura	P42669	Transcriptional activator protein Pur-alpha OS=Mus musculus OX=10090 GN=Pura PE=1 SV=1	0.873333333
Gss	P51855	Glutathione synthetase OS=Mus musculus OX=10090 GN=Gss PE=1 SV=1	1.016666667
Rps13	P62301	40S ribosomal protein S13 OS=Mus musculus OX=10090 GN=Rps13 PE=1 SV=2	0.89
Timm13	P62075	Mitochondrial import inner membrane translocase subunit Tim13 OS=Mus musculus OX=10090 GN	0.886666667
Slc2a1	P17809	Solute carrier family 2 facilitated glucose transporter member 1 OS=Mus musculus OX=10090 GN=S	0.936666667
Phactr4	Q501J7	Phosphatase and actin regulator 4 OS=Mus musculus OX=10090 GN=Phactr4 PE=1 SV=2	0.75
Gja1	P23242	Gap junction alpha-1 protein OS=Mus musculus OX=10090 GN=Gja1 PE=1 SV=2	1.12
Stambp	Q9CQ26	STAM-binding protein OS=Mus musculus OX=10090 GN=Stambp PE=1 SV=1	0.85
Cox6a1	P43024	Cytochrome c oxidase subunit 6A1 mitochondrial OS=Mus musculus OX=10090 GN=Cox6a1 PE=1 S	0.863333333
Bccip	Q9CWI3	BRCA2 and CDKN1A-interacting protein OS=Mus musculus OX=10090 GN=Bccip PE=1 SV=1	0.92
Cog3	Q8CI04	Conserved oligomeric Golgi complex subunit 3 OS=Mus musculus OX=10090 GN=Cog3 PE=1 SV=3	0.78
Sorbs3	Q9R1Z8	Vinexin OS=Mus musculus OX=10090 GN=Sorbs3 PE=1 SV=1	0.923333333

Gatd3a	Q9D172	Glutamine amidotransferase-like class 1 domain-containing protein 3A mitochondrial OS=Mus mus	0.856666667
Rfx1	P48377	MHC class II regulatory factor RFX1 OS=Mus musculus OX=10090 GN=Rfx1 PE=1 SV=2	1.22
Scamp3	O35609	Secretory carrier-associated membrane protein 3 OS=Mus musculus OX=10090 GN=Scamp3 PE=1 S\	0.9
Eipr1	Q8K0G5	EARP and GARP complex-interacting protein 1 OS=Mus musculus OX=10090 GN=Eipr1 PE=1 SV=2	0.896666667
Trmt61a	Q80XC2	tRNA (adenine(58)-N(1))-methyltransferase catalytic subunit TRMT61A OS=Mus musculus OX=1009(	0.863333333
Cgnl1	Q6AW69	Cingulin-like protein 1 OS=Mus musculus OX=10090 GN=Cgnl1 PE=1 SV=2	1.15
Wdr5	P61965	WD repeat-containing protein 5 OS=Mus musculus OX=10090 GN=Wdr5 PE=1 SV=1	0.76
Ndufv2	Q9D6J6	NADH dehydrogenase [ubiquinone] flavoprotein 2 mitochondrial OS=Mus musculus OX=10090 GN=	0.753333333
Stim2	P83093	Stromal interaction molecule 2 OS=Mus musculus OX=10090 GN=Stim2 PE=1 SV=2	0.843333333
Il1rl1	P14719	Interleukin-1 receptor-like 1 OS=Mus musculus OX=10090 GN=Il1rl1 PE=1 SV=2	0.893333333
Ppil2	Q9D787	RING-type E3 ubiquitin-protein ligase PPIL2 OS=Mus musculus OX=10090 GN=Ppil2 PE=1 SV=2	0.883333333
Grb14	Q9JLM9	Growth factor receptor-bound protein 14 OS=Mus musculus OX=10090 GN=Grb14 PE=1 SV=1	0.913333333
Dhcr24	Q8VCH6	Delta(24)-sterol reductase OS=Mus musculus OX=10090 GN=Dhcr24 PE=1 SV=1	0.983333333
Cbr4	Q91VT4	Carbonyl reductase family member 4 OS=Mus musculus OX=10090 GN=Cbr4 PE=1 SV=2	1.023333333
Kctd5	Q8VC57	BTB/POZ domain-containing protein KCTD5 OS=Mus musculus OX=10090 GN=Kctd5 PE=1 SV=1	0.926666667
Jmjd6	Q9ERI5	Bifunctional arginine demethylase and lysyl-hydroxylase JMJD6 OS=Mus musculus OX=10090 GN=J	0.916666667
Lsm2	O35900	U6 snRNA-associated Sm-like protein LSm2 OS=Mus musculus OX=10090 GN=Lsm2 PE=1 SV=1	0.843333333
Slc16a1	P53986	Monocarboxylate transporter 1 OS=Mus musculus OX=10090 GN=Slc16a1 PE=1 SV=1	0.876666667
Diexf	Q8BTT6	Digestive organ expansion factor homolog OS=Mus musculus OX=10090 GN=Diexf PE=2 SV=2	1.116666667
Fbxo22	Q78JE5	F-box only protein 22 OS=Mus musculus OX=10090 GN=Fbxo22 PE=1 SV=2	0.786666667
Naxe	Q8K4Z3	NAD(P)H-hydrate epimerase OS=Mus musculus OX=10090 GN=Naxe PE=1 SV=1	0.823333333
Cdc73	Q8JZM7	Parafibromin OS=Mus musculus OX=10090 GN=Cdc73 PE=1 SV=1	0.986666667
Dnph1	Q80VJ3	2'-deoxynucleoside 5'-phosphate N-hydrolase 1 OS=Mus musculus OX=10090 GN=Dnph1 PE=1 SV=2	1.01
Mtmr6	Q8VE11	Myotubularin-related protein 6 OS=Mus musculus OX=10090 GN=Mtmr6 PE=1 SV=1	1.373333333
Paf1	Q8K2T8	RNA polymerase II-associated factor 1 homolog OS=Mus musculus OX=10090 GN=Paf1 PE=1 SV=1	0.843333333
Cfap36	Q8C6E0	Cilia- and flagella-associated protein 36 OS=Mus musculus OX=10090 GN=Cfap36 PE=1 SV=1	1.043333333
Mrps2	Q924T2	28S ribosomal protein S2 mitochondrial OS=Mus musculus OX=10090 GN=Mrps2 PE=1 SV=1	0.863333333
Ripk3	Q9QZL0	Receptor-interacting serine/threonine-protein kinase 3 OS=Mus musculus OX=10090 GN=Ripk3 PE=	0.906666667
Polr2c	P97760	DNA-directed RNA polymerase II subunit RPB3 OS=Mus musculus OX=10090 GN=Polr2c PE=1 SV=2	0.833333333
Yif1b	Q9CX30	Protein YIF1B OS=Mus musculus OX=10090 GN=Yif1b PE=1 SV=2	0.796666667
Man2c1	Q91W89	Alpha-mannosidase 2C1 OS=Mus musculus OX=10090 GN=Man2c1 PE=1 SV=1	1.59
Poglut1	Q8BYB9	Protein O-glucosyltransferase 1 OS=Mus musculus OX=10090 GN=Poglut1 PE=1 SV=2	0.963333333
Itga6	Q61739	Integrin alpha-6 OS=Mus musculus OX=10090 GN=Itga6 PE=1 SV=3	0.746666667
Fam3c	Q91VU0	Protein FAM3C OS=Mus musculus OX=10090 GN=Fam3c PE=1 SV=1	0.98

Ppil3	Q9D6L8	Peptidyl-prolyl cis-trans isomerase-like 3 OS=Mus musculus OX=10090 GN=Ppil3 PE=1 SV=1	0.886666667
Babam2	Q8K3W0	BRISC and BRCA1-A complex member 2 OS=Mus musculus OX=10090 GN=Babam2 PE=1 SV=2	0.986666667
Mrpl12	Q9DB15	39S ribosomal protein L12 mitochondrial OS=Mus musculus OX=10090 GN=Mrpl12 PE=1 SV=2	0.856666667
Mtch1	Q791T5	Mitochondrial carrier homolog 1 OS=Mus musculus OX=10090 GN=Mtch1 PE=1 SV=1	0.843333333
Atg3	Q9CPX6	Ubiquitin-like-conjugating enzyme ATG3 OS=Mus musculus OX=10090 GN=Atg3 PE=1 SV=1	0.84
Bola2	Q8BGS2	BolA-like protein 2 OS=Mus musculus OX=10090 GN=Bola2 PE=1 SV=1	0.94
Exosc9	Q9JHI7	Exosome complex component RRP45 OS=Mus musculus OX=10090 GN=Exosc9 PE=1 SV=1	0.903333333
Rer1	Q9CQU3	Protein RER1 OS=Mus musculus OX=10090 GN=Rer1 PE=1 SV=1	0.836666667
Arhgef10	Q8C033	Rho guanine nucleotide exchange factor 10 OS=Mus musculus OX=10090 GN=Arhgef10 PE=1 SV=2	0.88
Ppp6c	Q9CQR6	Serine/threonine-protein phosphatase 6 catalytic subunit OS=Mus musculus OX=10090 GN=Ppp6c	0.99
Exosc2	Q8VBV3	Exosome complex component RRP4 OS=Mus musculus OX=10090 GN=Exosc2 PE=1 SV=1	0.876666667
Vps16	Q920Q4	Vacuolar protein sorting-associated protein 16 homolog OS=Mus musculus OX=10090 GN=Vps16 PI	1.166666667
Naf1	Q3UMQ8	H/ACA ribonucleoprotein complex non-core subunit NAF1 OS=Mus musculus OX=10090 GN=Naf1 P	0.76
Ccdc58	Q8R3Q6	Coiled-coil domain-containing protein 58 OS=Mus musculus OX=10090 GN=Ccdc58 PE=1 SV=1	0.913333333
Pdcd5	P56812	Programmed cell death protein 5 OS=Mus musculus OX=10090 GN=Pdcd5 PE=1 SV=3	0.93
Slc4a7	Q8BTY2	Sodium bicarbonate cotransporter 3 OS=Mus musculus OX=10090 GN=Slc4a7 PE=1 SV=2	0.953333333
Smn1	P97801	Survival motor neuron protein OS=Mus musculus OX=10090 GN=Smn1 PE=1 SV=1	0.876666667
Prkra	Q9WTX2	Interferon-inducible double-stranded RNA-dependent protein kinase activator A OS=Mus musculus	0.963333333
Znf207	Q9JMD0	BUB3-interacting and GLEBS motif-containing protein ZNF207 OS=Mus musculus OX=10090 GN=Zn	0.906666667
Ldlr	P35951	Low-density lipoprotein receptor OS=Mus musculus OX=10090 GN=Ldlr PE=1 SV=2	0.896666667
Arl6ip5	Q8R5J9	PRA1 family protein 3 OS=Mus musculus OX=10090 GN=Arl6ip5 PE=1 SV=2	0.896666667
Prpf39	Q8K2Z2	Pre-mRNA-processing factor 39 OS=Mus musculus OX=10090 GN=Prpf39 PE=1 SV=3	1.326666667
Exosc6	Q8BTW3	Exosome complex component MTR3 OS=Mus musculus OX=10090 GN=Exosc6 PE=1 SV=1	0.813333333
Ate1	Q9Z2A5	Arginyl-tRNA--protein transferase 1 OS=Mus musculus OX=10090 GN=Ate1 PE=1 SV=2	0.883333333
Pdpk1	Q9Z2A0	3-phosphoinositide-dependent protein kinase 1 OS=Mus musculus OX=10090 GN=Pdpk1 PE=1 SV=2	0.906666667
Zmym4	A2A791	Zinc finger MYM-type protein 4 OS=Mus musculus OX=10090 GN=Zmym4 PE=1 SV=1	1.4
Gnpat	P98192	Dihydroxyacetone phosphate acyltransferase OS=Mus musculus OX=10090 GN=Gnpat PE=1 SV=1	2.823333333
Polr2d	Q9D7M8	DNA-directed RNA polymerase II subunit RPB4 OS=Mus musculus OX=10090 GN=Polr2d PE=1 SV=2	1.086666667
Cd2bp2	Q9CWK3	CD2 antigen cytoplasmic tail-binding protein 2 OS=Mus musculus OX=10090 GN=Cd2bp2 PE=1 SV=:	1.183333333
Ppie	Q9QZH3	Peptidyl-prolyl cis-trans isomerase E OS=Mus musculus OX=10090 GN=Ppie PE=1 SV=2	0.883333333
Gmfb	Q9CQI3	Glia maturation factor beta OS=Mus musculus OX=10090 GN=Gmfb PE=1 SV=3	0.903333333
Lsm14a	Q8K2F8	Protein LSM14 homolog A OS=Mus musculus OX=10090 GN=Lsm14a PE=1 SV=1	0.933333333
Napg	Q9CWZ7	Gamma-soluble NSF attachment protein OS=Mus musculus OX=10090 GN=Napg PE=1 SV=1	1.236666667
Sqle	P52019	Squalene monooxygenase OS=Mus musculus OX=10090 GN=Sqle PE=2 SV=1	0.91

Pex3	Q9QXY9	Peroxisomal biogenesis factor 3 OS=Mus musculus OX=10090 GN=Pex3 PE=1 SV=1	1.083333333
Diablo	Q9JIQ3	Diablo homolog mitochondrial OS=Mus musculus OX=10090 GN=Diablo PE=1 SV=2	0.846666667
Ap3s1	Q9DCR2	AP-3 complex subunit sigma-1 OS=Mus musculus OX=10090 GN=Ap3s1 PE=1 SV=2	0.92
Ccnt1	Q9QWV9	Cyclin-T1 OS=Mus musculus OX=10090 GN=Ccnt1 PE=1 SV=3	1.196666667
Mettl1	Q9Z120	tRNA (guanine-N(7)-)-methyltransferase OS=Mus musculus OX=10090 GN=Mettl1 PE=1 SV=1	0.866666667
Magohb	Q9CQL1	Protein mago nashi homolog 2 OS=Mus musculus OX=10090 GN=Magohb PE=2 SV=1	0.87
Magoh	P61327	Protein mago nashi homolog OS=Mus musculus OX=10090 GN=Magoh PE=2 SV=1	0.87
Atp1b1	P14094	Sodium/potassium-transporting ATPase subunit beta-1 OS=Mus musculus OX=10090 GN=Atp1b1 P	0.96
Eif4enif1	Q9EST3	Eukaryotic translation initiation factor 4E transporter OS=Mus musculus OX=10090 GN=Eif4enif1 P	0.886666667
Snx18	Q91ZR2	Sorting nexin-18 OS=Mus musculus OX=10090 GN=Snx18 PE=1 SV=1	0.943333333
Prpf38a	Q4FK66	Pre-mRNA-splicing factor 38A OS=Mus musculus OX=10090 GN=Prpf38a PE=1 SV=1	1.153333333
Znf280c	Q6P3Y5	Zinc finger protein 280C OS=Mus musculus OX=10090 GN=Znf280c PE=1 SV=1	0.95
Loxl3	Q9Z175	Lysyl oxidase homolog 3 OS=Mus musculus OX=10090 GN=Loxl3 PE=1 SV=2	0.936666667
Pon2	Q62086	Serum paraoxonase/arylesterase 2 OS=Mus musculus OX=10090 GN=Pon2 PE=1 SV=2	0.72
Lpcat1	Q3TFD2	Lysophosphatidylcholine acyltransferase 1 OS=Mus musculus OX=10090 GN=Lpcat1 PE=1 SV=1	0.783333333
Atg5	Q99J83	Autophagy protein 5 OS=Mus musculus OX=10090 GN=Atg5 PE=1 SV=1	1.246666667
Brcc3	P46737	Lys-63-specific deubiquitinase BRCC36 OS=Mus musculus OX=10090 GN=Brcc3 PE=1 SV=1	0.72
Atm	Q62388	Serine-protein kinase ATM OS=Mus musculus OX=10090 GN=Atm PE=1 SV=2	0.696666667
Nol8	Q3UHX0	Nucleolar protein 8 OS=Mus musculus OX=10090 GN=Nol8 PE=1 SV=2	0.726666667
Ccdc86	Q9JJ89	Coiled-coil domain-containing protein 86 OS=Mus musculus OX=10090 GN=Ccdc86 PE=1 SV=2	0.823333333
Ssbp1	Q9CYR0	Single-stranded DNA-binding protein mitochondrial OS=Mus musculus OX=10090 GN=Ssbp1 PE=1 S	0.946666667
Ubr7	Q8BU04	Putative E3 ubiquitin-protein ligase UBR7 OS=Mus musculus OX=10090 GN=Ubr7 PE=1 SV=1	1.34
Stmn1	P54227	Stathmin OS=Mus musculus OX=10090 GN=Stmn1 PE=1 SV=2	0.863333333
Lamtor1	Q9CQ22	Ragulator complex protein LAMTOR1 OS=Mus musculus OX=10090 GN=Lamtor1 PE=1 SV=1	0.933333333
Man2b2	O54782	Epididymis-specific alpha-mannosidase OS=Mus musculus OX=10090 GN=Man2b2 PE=1 SV=2	0.713333333
Mydgf	Q9CPT4	Myeloid-derived growth factor OS=Mus musculus OX=10090 GN=Mydgf PE=1 SV=1	0.84
Mtmr3	Q8K296	Myotubularin-related protein 3 OS=Mus musculus OX=10090 GN=Mtmr3 PE=1 SV=2	1.16
Slc25a1	Q8JZU2	Tricarboxylate transport protein mitochondrial OS=Mus musculus OX=10090 GN=Slc25a1 PE=1 SV	0.873333333
Mrps18b	Q99N84	28S ribosomal protein S18b mitochondrial OS=Mus musculus OX=10090 GN=Mrps18b PE=1 SV=1	0.903333333
Pmm2	Q9Z2M7	Phosphomannomutase 2 OS=Mus musculus OX=10090 GN=Pmm2 PE=1 SV=1	0.92
Tut1	Q8R3F9	Speckle targeted PIP5K1A-regulated poly(A) polymerase OS=Mus musculus OX=10090 GN=Tut1 PE=	0.9
Hmmr	Q00547	Hyaluronan mediated motility receptor OS=Mus musculus OX=10090 GN=Hmmr PE=1 SV=4	0.876666667
Pfdn2	O70591	Prefoldin subunit 2 OS=Mus musculus OX=10090 GN=Pfdn2 PE=1 SV=2	0.903333333
Trip4	Q9QXN3	Activating signal cointegrator 1 OS=Mus musculus OX=10090 GN=Trip4 PE=1 SV=2	0.836666667

Gins4	Q99LZ3	DNA replication complex GINS protein SLD5 OS=Mus musculus OX=10090 GN=Gins4 PE=1 SV=1	1.163333333
Amfr	Q9R049	E3 ubiquitin-protein ligase AMFR OS=Mus musculus OX=10090 GN=Amfr PE=1 SV=2	0.656666667
Ints5	Q8CHT3	Integrator complex subunit 5 OS=Mus musculus OX=10090 GN=Ints5 PE=2 SV=1	1.253333333
Thyn1	Q91YJ3	Thymocyte nuclear protein 1 OS=Mus musculus OX=10090 GN=Thyn1 PE=1 SV=1	1.046666667
Sdc4	O35988	Syndecan-4 OS=Mus musculus OX=10090 GN=Sdc4 PE=1 SV=1	0.96
Gtf3c2	Q8BL74	General transcription factor 3C polypeptide 2 OS=Mus musculus OX=10090 GN=Gtf3c2 PE=2 SV=2	0.863333333
Ndufa8	Q9DCJ5	NADH dehydrogenase [ubiquinone] 1 alpha subcomplex subunit 8 OS=Mus musculus OX=10090 GN	0.796666667
Gins3	Q9CY94	DNA replication complex GINS protein PSF3 OS=Mus musculus OX=10090 GN=Gins3 PE=2 SV=1	0.913333333
Trim25	Q61510	E3 ubiquitin/ISG15 ligase TRIM25 OS=Mus musculus OX=10090 GN=Trim25 PE=1 SV=2	1.38
Polr2h	Q923G2	DNA-directed RNA polymerases I II and III subunit RPABC3 OS=Mus musculus OX=10090 GN=Polr2h	0.993333333
Cpe	Q00493	Carboxypeptidase E OS=Mus musculus OX=10090 GN=Cpe PE=1 SV=2	0.996666667
Ccdc50	Q810U5	Coiled-coil domain-containing protein 50 OS=Mus musculus OX=10090 GN=Ccdc50 PE=1 SV=1	0.773333333
Cwc15	Q9JHS9	Spliceosome-associated protein CWC15 homolog OS=Mus musculus OX=10090 GN=Cwc15 PE=1 SV	1.006666667
Bag1	Q60739	BAG family molecular chaperone regulator 1 OS=Mus musculus OX=10090 GN=Bag1 PE=1 SV=3	0.883333333
Cers5	Q9D6K9	Ceramide synthase 5 OS=Mus musculus OX=10090 GN=Cers5 PE=1 SV=1	1.21
Fam162a	Q9D6U8	Protein FAM162A OS=Mus musculus OX=10090 GN=Fam162a PE=1 SV=1	0.9
Aftph	Q80WT5	Aftiphilin OS=Mus musculus OX=10090 GN=Aftph PE=1 SV=2	0.973333333
Hs2st1	Q8R3H7	Heparan sulfate 2-O-sulfotransferase 1 OS=Mus musculus OX=10090 GN=Hs2st1 PE=1 SV=2	0.683333333
Gulp1	Q8K2A1	PTB domain-containing engulfment adapter protein 1 OS=Mus musculus OX=10090 GN=Gulp1 PE=1	0.856666667
Cda	P56389	Cytidine deaminase OS=Mus musculus OX=10090 GN=Cda PE=1 SV=2	0.853333333
Vamp7	P70280	Vesicle-associated membrane protein 7 OS=Mus musculus OX=10090 GN=Vamp7 PE=1 SV=1	0.846666667
Ehbp1	Q69ZW3	EH domain-binding protein 1 OS=Mus musculus OX=10090 GN=Ehbp1 PE=1 SV=3	1.296666667
Lsm12	Q9D0R8	Protein LSM12 homolog OS=Mus musculus OX=10090 GN=Lsm12 PE=1 SV=1	0.866666667
Stom	P54116	Erythrocyte band 7 integral membrane protein OS=Mus musculus OX=10090 GN=Stom PE=1 SV=3	0.946666667
Gtf2e1	Q9D0D5	General transcription factor IIE subunit 1 OS=Mus musculus OX=10090 GN=Gtf2e1 PE=1 SV=1	0.976666667
Ankrd13a	Q80UP5	Ankyrin repeat domain-containing protein 13A OS=Mus musculus OX=10090 GN=Ankrd13a PE=1 SV	0.893333333
Med15	Q924H2	Mediator of RNA polymerase II transcription subunit 15 OS=Mus musculus OX=10090 GN=Med15 PE	0.77
Tex30	Q3TUU5	Testis-expressed protein 30 OS=Mus musculus OX=10090 GN=Tex30 PE=1 SV=1	0.78
Pja2	Q80U04	E3 ubiquitin-protein ligase Praja-2 OS=Mus musculus OX=10090 GN=Pja2 PE=1 SV=2	0.703333333
Sord	Q64442	Sorbitol dehydrogenase OS=Mus musculus OX=10090 GN=Sord PE=1 SV=3	0.68
Gla	P51569	Alpha-galactosidase A OS=Mus musculus OX=10090 GN=Gla PE=1 SV=1	1.006666667
Arhgap17	Q3UIA2	Rho GTPase-activating protein 17 OS=Mus musculus OX=10090 GN=Arhgap17 PE=1 SV=1	1.01
Myo1d	Q5SYD0	Unconventional myosin-Id OS=Mus musculus OX=10090 GN=Myo1d PE=1 SV=1	0.726666667
Plpp1	Q61469	Phospholipid phosphatase 1 OS=Mus musculus OX=10090 GN=Plpp1 PE=1 SV=1	1.046666667

Mrps23	Q8VE22	28S ribosomal protein S23 mitochondrial OS=Mus musculus OX=10090 GN=Mrps23 PE=1 SV=1	0.906666667
Tab1	Q8CF89	TGF-beta-activated kinase 1 and MAP3K7-binding protein 1 OS=Mus musculus OX=10090 GN=Tab1	1.19
Ddx41	Q91VN6	Probable ATP-dependent RNA helicase DDX41 OS=Mus musculus OX=10090 GN=Ddx41 PE=1 SV=2	1.006666667
Aldh1a7	O35945	Aldehyde dehydrogenase cytosolic 1 OS=Mus musculus OX=10090 GN=Aldh1a7 PE=1 SV=1	0.923333333
Epdr1	Q99M71	Mammalian ependymin-related protein 1 OS=Mus musculus OX=10090 GN=Epdr1 PE=1 SV=1	0.95
Ep400	Q8CHI8	E1A-binding protein p400 OS=Mus musculus OX=10090 GN=Ep400 PE=1 SV=3	0.543333333
Armc6	Q8BNU0	Armadillo repeat-containing protein 6 OS=Mus musculus OX=10090 GN=Armc6 PE=1 SV=1	0.983333333
Setd7	Q8VHL1	Histone-lysine N-methyltransferase SETD7 OS=Mus musculus OX=10090 GN=Setd7 PE=1 SV=2	1.033333333
Tmem120a	Q8C1E7	Ion channel TACAN OS=Mus musculus OX=10090 GN=Tmem120a PE=1 SV=1	1.046666667
Ppp1r2	Q9DCL8	Protein phosphatase inhibitor 2 OS=Mus musculus OX=10090 GN=Ppp1r2 PE=1 SV=3	0.78
Efnb2	P52800	Ephrin-B2 OS=Mus musculus OX=10090 GN=Efnb2 PE=1 SV=1	0.973333333
Dcaf1	Q80TR8	DDB1- and CUL4-associated factor 1 OS=Mus musculus OX=10090 GN=Dcaf1 PE=1 SV=4	0.753333333
Smndc1	Q8BGT7	Survival of motor neuron-related-splicing factor 30 OS=Mus musculus OX=10090 GN=Smndc1 PE=1	0.836666667
Pald1	P70261	Paladin OS=Mus musculus OX=10090 GN=Pald1 PE=1 SV=1	1.193333333
Rpl35a	O55142	60S ribosomal protein L35a OS=Mus musculus OX=10090 GN=Rpl35a PE=1 SV=2	1.05
Bnip1	Q6QD59	Vesicle transport protein SEC20 OS=Mus musculus OX=10090 GN=Bnip1 PE=1 SV=1	0.796666667
Ddx31	Q6NZQ2	Probable ATP-dependent RNA helicase DDX31 OS=Mus musculus OX=10090 GN=Ddx31 PE=2 SV=2	0.75
Alg5	Q9DB25	Dolichyl-phosphate beta-glucosyltransferase OS=Mus musculus OX=10090 GN=Alg5 PE=1 SV=1	0.886666667
Scarb2	O35114	Lysosome membrane protein 2 OS=Mus musculus OX=10090 GN=Scarb2 PE=1 SV=3	0.766666667
Ppip5k2	Q6ZQB6	Inositol hexakisphosphate and diphosphoinositol-pentakisphosphate kinase 2 OS=Mus musculus O	0.94
Trappc12	Q8K2L8	Trafficking protein particle complex subunit 12 OS=Mus musculus OX=10090 GN=Trappc12 PE=1 S	1.043333333
Sh3bp4	Q921I6	SH3 domain-binding protein 4 OS=Mus musculus OX=10090 GN=Sh3bp4 PE=1 SV=1	1.583333333
Lias	Q99M04	Lipoyl synthase mitochondrial OS=Mus musculus OX=10090 GN=Lias PE=1 SV=1	1.093333333
Haus5	Q9D786	HAUS augmin-like complex subunit 5 OS=Mus musculus OX=10090 GN=Haus5 PE=1 SV=1	0.776666667
Kctd10	Q922M3	BTB/POZ domain-containing adapter for CUL3-mediated RhoA degradation protein 3 OS=Mus musc	0.896666667
Rabgef1	Q9JM13	Rab5 GDP/GTP exchange factor OS=Mus musculus OX=10090 GN=Rabgef1 PE=1 SV=1	1.076666667
Pgs1	Q8BHF7	CDP-diacylglycerol--glycerol-3-phosphate 3-phosphatidyltransferase mitochondrial OS=Mus musc	1.103333333
Ccz1	Q8C1Y8	Vacuolar fusion protein CCZ1 homolog OS=Mus musculus OX=10090 GN=Ccz1 PE=1 SV=1	1.186666667
Srsf9	Q9D0B0	Serine/arginine-rich splicing factor 9 OS=Mus musculus OX=10090 GN=Srsf9 PE=1 SV=1	0.87
Vamp8	O70404	Vesicle-associated membrane protein 8 OS=Mus musculus OX=10090 GN=Vamp8 PE=1 SV=1	0.963333333
Pon3	Q62087	Serum paraoxonase/lactonase 3 OS=Mus musculus OX=10090 GN=Pon3 PE=1 SV=2	0.833333333
Fnbp1	Q80TY0	Formin-binding protein 1 OS=Mus musculus OX=10090 GN=Fnbp1 PE=1 SV=2	4.103333333
Rufy1	Q8BIJ7	RUN and FYVE domain-containing protein 1 OS=Mus musculus OX=10090 GN=Rufy1 PE=1 SV=1	0.823333333
Traf2	P39429	TNF receptor-associated factor 2 OS=Mus musculus OX=10090 GN=Traf2 PE=1 SV=1	0.816666667

Pmvk	Q9D1G2	Phosphomevalonate kinase OS=Mus musculus OX=10090 GN=Pmvk PE=1 SV=3	0.87
Vps26c	O35075	Vacuolar protein sorting-associated protein 26C OS=Mus musculus OX=10090 GN=Vps26c PE=1 SV=	0.76
Virma	A2AIV2	Protein virilizer homolog OS=Mus musculus OX=10090 GN=Virma PE=1 SV=1	1.19
Itih3	Q61704	Inter-alpha-trypsin inhibitor heavy chain H3 OS=Mus musculus OX=10090 GN=Itih3 PE=1 SV=3	0.796666667
Trim16	Q99PP9	Tripartite motif-containing protein 16 OS=Mus musculus OX=10090 GN=Trim16 PE=1 SV=2	0.903333333
Rpl29	P47915	60S ribosomal protein L29 OS=Mus musculus OX=10090 GN=Rpl29 PE=1 SV=2	0.8
Agfg2	Q80WC7	Arf-GAP domain and FG repeat-containing protein 2 OS=Mus musculus OX=10090 GN=Agfg2 PE=1 S\	0.84
Mrpl21	Q9D1N9	39S ribosomal protein L21 mitochondrial OS=Mus musculus OX=10090 GN=Mrpl21 PE=1 SV=1	0.85
Mrpl11	Q9CQF0	39S ribosomal protein L11 mitochondrial OS=Mus musculus OX=10090 GN=Mrpl11 PE=1 SV=1	0.89
Golga1	Q9CW79	Golgin subfamily A member 1 OS=Mus musculus OX=10090 GN=Golga1 PE=1 SV=2	1.23
Lztfl1	Q9JHQ5	Leucine zipper transcription factor-like protein 1 OS=Mus musculus OX=10090 GN=Lztfl1 PE=1 SV=1	1.096666667
Mrps24	Q9CQV5	28S ribosomal protein S24 mitochondrial OS=Mus musculus OX=10090 GN=Mrps24 PE=1 SV=1	0.673333333
Tmed5	Q9CXE7	Transmembrane emp24 domain-containing protein 5 OS=Mus musculus OX=10090 GN=Tmed5 PE=	0.81
Sptlc1	O35704	Serine palmitoyltransferase 1 OS=Mus musculus OX=10090 GN=Sptlc1 PE=1 SV=2	0.856666667
Nenf	Q9CQ45	Neudesin OS=Mus musculus OX=10090 GN=Nenf PE=1 SV=1	0.93
Spcs3	Q6ZWQ7	Signal peptidase complex subunit 3 OS=Mus musculus OX=10090 GN=Spcs3 PE=1 SV=1	0.906666667
Nbn	Q9R207	Nibrin OS=Mus musculus OX=10090 GN=Nbn PE=1 SV=1	0.78
Mcm3ap	Q9WUU9	Germinal-center associated nuclear protein OS=Mus musculus OX=10090 GN=Mcm3ap PE=1 SV=2	2.036666667
Patl1	Q3TC46	Protein PAT1 homolog 1 OS=Mus musculus OX=10090 GN=Patl1 PE=1 SV=2	1.066666667
Tamm41	Q3TUH1	Phosphatidate cytidylyltransferase mitochondrial OS=Mus musculus OX=10090 GN=Tamm41 PE=1	0.78
Cd3eap	Q76KJ5	DNA-directed RNA polymerase I subunit RPA34 OS=Mus musculus OX=10090 GN=Polr1g PE=1 SV=2	1.083333333
Xrcc1	Q60596	DNA repair protein XRCC1 OS=Mus musculus OX=10090 GN=Xrcc1 PE=1 SV=2	1.156666667
Apob	E9Q414	Apolipoprotein B-100 OS=Mus musculus OX=10090 GN=Apob PE=1 SV=1	0.183333333
Tfam	P40630	Transcription factor A mitochondrial OS=Mus musculus OX=10090 GN=Tfam PE=1 SV=2	0.94
Pik3r1	P26450	Phosphatidylinositol 3-kinase regulatory subunit alpha OS=Mus musculus OX=10090 GN=Pik3r1 PE	1.303333333
Tatdn1	Q6P8M1	Putative deoxyribonuclease TATDN1 OS=Mus musculus OX=10090 GN=Tatdn1 PE=1 SV=1	1.09
Steap2	Q8BWB6	Metalloreductase STEAP2 OS=Mus musculus OX=10090 GN=Steap2 PE=1 SV=1	0.993333333
Tmx1	Q8VBT0	Thioredoxin-related transmembrane protein 1 OS=Mus musculus OX=10090 GN=Tmx1 PE=1 SV=1	0.676666667
F5	O88783	Coagulation factor V OS=Mus musculus OX=10090 GN=F5 PE=1 SV=1	0.223333333
Sumf2	Q8BPG6	Inactive C-alpha-formylglycine-generating enzyme 2 OS=Mus musculus OX=10090 GN=Sumf2 PE=1 S	0.963333333
Mrpl24	Q9CQ06	39S ribosomal protein L24 mitochondrial OS=Mus musculus OX=10090 GN=Mrpl24 PE=1 SV=1	0.746666667
Alkbh3	Q8K1E6	Alpha-ketoglutarate-dependent dioxygenase alkB homolog 3 OS=Mus musculus OX=10090 GN=Alkb	0.96
Sec11a	Q9R0P6	Signal peptidase complex catalytic subunit SEC11A OS=Mus musculus OX=10090 GN=Sec11a PE=1 S	0.903333333
Rpp40	Q8R1F9	Ribonuclease P protein subunit p40 OS=Mus musculus OX=10090 GN=Rpp40 PE=2 SV=2	0.963333333

Mrpl49	Q9CQ40	39S ribosomal protein L49 mitochondrial OS=Mus musculus OX=10090 GN=Mrpl49 PE=1 SV=1	1.166666667
Mrpl15	Q9CPR5	39S ribosomal protein L15 mitochondrial OS=Mus musculus OX=10090 GN=Mrpl15 PE=1 SV=1	0.86
Ago1	Q8CJG1	Protein argonaute-1 OS=Mus musculus OX=10090 GN=Ago1 PE=1 SV=2	
Strbp	Q91WM1	Spermatid perinuclear RNA-binding protein OS=Mus musculus OX=10090 GN=Strbp PE=1 SV=1	
Smad3	Q8BUN5	Mothers against decapentaplegic homolog 3 OS=Mus musculus OX=10090 GN=Smad3 PE=1 SV=2	
Itpr1	P11881	Inositol 1 4 5-trisphosphate receptor type 1 OS=Mus musculus OX=10090 GN=Itpr1 PE=1 SV=2	
Itpr2	Q9Z329	Inositol 1 4 5-trisphosphate receptor type 2 OS=Mus musculus OX=10090 GN=Itpr2 PE=1 SV=4	
Ube2v1	Q9CZY3	Ubiquitin-conjugating enzyme E2 variant 1 OS=Mus musculus OX=10090 GN=Ube2v1 PE=1 SV=1	3.273333333
Ephb4	P54761	Ephrin type-B receptor 4 OS=Mus musculus OX=10090 GN=Ephb4 PE=1 SV=2	
Ppp3cb	P48453	Serine/threonine-protein phosphatase 2B catalytic subunit beta isoform OS=Mus musculus OX=100	0.883333333
Csnk1d	Q9DC28	Casein kinase I isoform delta OS=Mus musculus OX=10090 GN=Csnk1d PE=1 SV=2	1.05
Tbl1xr1	Q8BHJ5	F-box-like/WD repeat-containing protein TBL1XR1 OS=Mus musculus OX=10090 GN=Tbl1xr1 PE=1 S	1.466666667
Cstf2t	Q8C7E9	Cleavage stimulation factor subunit 2 tau variant OS=Mus musculus OX=10090 GN=Cstf2t PE=1 SV=2	1.126666667
Rab6b	P61294	Ras-related protein Rab-6B OS=Mus musculus OX=10090 GN=Rab6b PE=1 SV=1	1.31
Rap2c	Q8BU31	Ras-related protein Rap-2c OS=Mus musculus OX=10090 GN=Rap2c PE=1 SV=1	1.666666667
Tia1	P52912	Nucleolysin TIA-1 OS=Mus musculus OX=10090 GN=Tia1 PE=1 SV=1	0.86
Ugt1a6	Q64435	UDP-glucuronosyltransferase 1-6 OS=Mus musculus OX=10090 GN=Ugt1a6 PE=1 SV=1	0.94
Srsf4	Q8VE97	Serine/arginine-rich splicing factor 4 OS=Mus musculus OX=10090 GN=Srsf4 PE=2 SV=1	0.943333333
Epha4	Q03137	Ephrin type-A receptor 4 OS=Mus musculus OX=10090 GN=Epha4 PE=1 SV=2	1.223333333
Txnrd3	Q99MD6	Thioredoxin reductase 3 OS=Mus musculus OX=10090 GN=Txnrd3 PE=1 SV=3	1.48
Stam	P70297	Signal transducing adapter molecule 1 OS=Mus musculus OX=10090 GN=Stam PE=1 SV=3	1.06
Ranbp9	P69566	Ran-binding protein 9 OS=Mus musculus OX=10090 GN=Ranbp9 PE=1 SV=1	1.09
Bcr	Q6PAJ1	Breakpoint cluster region protein OS=Mus musculus OX=10090 GN=Bcr PE=1 SV=3	1.273333333
Ppp2r5e	Q61151	Serine/threonine-protein phosphatase 2A 56 kDa regulatory subunit epsilon isoform OS=Mus muscu	0.85
Uaca	Q8CGB3	Uveal autoantigen with coiled-coil domains and ankyrin repeats OS=Mus musculus OX=10090 GN=U	1.926666667
Rab31	Q921E2	Ras-related protein Rab-31 OS=Mus musculus OX=10090 GN=Rab31 PE=1 SV=1	0.733333333
Ptpn21	Q62136	Tyrosine-protein phosphatase non-receptor type 21 OS=Mus musculus OX=10090 GN=Ptpn21 PE=1	0.996666667
Arpc1a	Q9R0Q6	Actin-related protein 2/3 complex subunit 1A OS=Mus musculus OX=10090 GN=Arpc1a PE=1 SV=1	0.81
Cdk9	Q99J95	Cyclin-dependent kinase 9 OS=Mus musculus OX=10090 GN=Cdk9 PE=1 SV=1	0.766666667
Gnpda1	O88958	Glucosamine-6-phosphate isomerase 1 OS=Mus musculus OX=10090 GN=Gnpda1 PE=1 SV=3	0.853333333
Anapc2	Q8BZQ7	Anaphase-promoting complex subunit 2 OS=Mus musculus OX=10090 GN=Anapc2 PE=1 SV=2	0.843333333
Srsf7	Q8BL97	Serine/arginine-rich splicing factor 7 OS=Mus musculus OX=10090 GN=Srsf7 PE=1 SV=1	0.903333333
Atl2	Q6PA06	Atlastin-2 OS=Mus musculus OX=10090 GN=Atl2 PE=1 SV=1	1.046666667
Egfr	Q01279	Epidermal growth factor receptor OS=Mus musculus OX=10090 GN=Egfr PE=1 SV=1	0.85

Prkab1	Q9R078	5'-AMP-activated protein kinase subunit beta-1 OS=Mus musculus OX=10090 GN=Prkab1 PE=1 SV=2	0.876666667
Snrpb2	Q9CQI7	U2 small nuclear ribonucleoprotein B'' OS=Mus musculus OX=10090 GN=Snrpb2 PE=1 SV=1	0.873333333
Ddah2	Q99LD8	N(G) N(G)-dimethylarginine dimethylaminohydrolase 2 OS=Mus musculus OX=10090 GN=Ddah2 PE	0.83
Fkbp15	Q6P9Q6	FK506-binding protein 15 OS=Mus musculus OX=10090 GN=Fkbp15 PE=1 SV=2	0.91
Ccdc124	Q9D8X2	Coiled-coil domain-containing protein 124 OS=Mus musculus OX=10090 GN=Ccdc124 PE=1 SV=1	0.973333333
Tars2	Q3UQ84	Threonine--tRNA ligase mitochondrial OS=Mus musculus OX=10090 GN=Tars2 PE=1 SV=1	0.866666667
Nexn	Q7TPW1	Nexilin OS=Mus musculus OX=10090 GN=Nexn PE=1 SV=3	0.8
Prkcd	P28867	Protein kinase C delta type OS=Mus musculus OX=10090 GN=Prkcd PE=1 SV=3	0.69
Mast4	Q811L6	Microtubule-associated serine/threonine-protein kinase 4 OS=Mus musculus OX=10090 GN=Mast4	0.99
Kif3a	P28741	Kinesin-like protein KIF3A OS=Mus musculus OX=10090 GN=Kif3a PE=1 SV=2	1.086666667
Clta	O08585	Clathrin light chain A OS=Mus musculus OX=10090 GN=Clta PE=1 SV=2	0.943333333
Uri1	Q3TLD5	Unconventional prefoldin RPB5 interactor OS=Mus musculus OX=10090 GN=Uri1 PE=1 SV=2	0.966666667
Rbm17	Q8JZX4	Splicing factor 45 OS=Mus musculus OX=10090 GN=Rbm17 PE=1 SV=1	0.796666667
C3	P01027	Complement C3 OS=Mus musculus OX=10090 GN=C3 PE=1 SV=3	0.14
Rplp1	P47955	60S acidic ribosomal protein P1 OS=Mus musculus OX=10090 GN=Rplp1 PE=1 SV=1	0.853333333
Lypla1	P97823	Acyl-protein thioesterase 1 OS=Mus musculus OX=10090 GN=Lypla1 PE=1 SV=1	0.83
Nup35	Q8R4R6	Nucleoporin NUP35 OS=Mus musculus OX=10090 GN=Nup35 PE=1 SV=2	0.893333333
Cyb5b	Q9CQX2	Cytochrome b5 type B OS=Mus musculus OX=10090 GN=Cyb5b PE=1 SV=1	0.91
Ptrh2	Q8R2Y8	Peptidyl-tRNA hydrolase 2 mitochondrial OS=Mus musculus OX=10090 GN=Ptrh2 PE=1 SV=1	0.876666667
Naca	P70670	Nascent polypeptide-associated complex subunit alpha muscle-specific form OS=Mus musculus OX	0.846666667
Naca	Q60817	Nascent polypeptide-associated complex subunit alpha OS=Mus musculus OX=10090 GN=Naca PE=	0.846666667
Tpp1	O89023	Tripeptidyl-peptidase 1 OS=Mus musculus OX=10090 GN=Tpp1 PE=1 SV=2	0.853333333
Eloc	P83940	Elongin-C OS=Mus musculus OX=10090 GN=Eloc PE=1 SV=1	0.826666667
Wdr77	Q99J09	Methylosome protein 50 OS=Mus musculus OX=10090 GN=Wdr77 PE=1 SV=1	0.943333333
Fndc3b	Q6NWW9	Fibronectin type III domain-containing protein 3B OS=Mus musculus OX=10090 GN=Fndc3b PE=1 S	0.776666667
Atox1	O08997	Copper transport protein ATOX1 OS=Mus musculus OX=10090 GN=Atox1 PE=1 SV=1	0.906666667
Efemp2	Q9WVJ9	EGF-containing fibulin-like extracellular matrix protein 2 OS=Mus musculus OX=10090 GN=Efemp2	0.856666667
Cd44	P15379	CD44 antigen OS=Mus musculus OX=10090 GN=Cd44 PE=1 SV=3	0.89
Mbd3	Q9Z2D8	Methyl-CpG-binding domain protein 3 OS=Mus musculus OX=10090 GN=Mbd3 PE=1 SV=1	0.853333333
Grsf1	Q8C5Q4	G-rich sequence factor 1 OS=Mus musculus OX=10090 GN=Grsf1 PE=1 SV=2	0.893333333
Cdh2	P15116	Cadherin-2 OS=Mus musculus OX=10090 GN=Cdh2 PE=1 SV=2	0.72
Pin1	Q9QUR7	Peptidyl-prolyl cis-trans isomerase NIMA-interacting 1 OS=Mus musculus OX=10090 GN=Pin1 PE=1	0.88
Blvrb	Q923D2	Flavin reductase (NADPH) OS=Mus musculus OX=10090 GN=Blvrb PE=1 SV=3	0.833333333
Jpt2	Q6PGH2	Jupiter microtubule associated homolog 2 OS=Mus musculus OX=10090 GN=Jpt2 PE=1 SV=1	0.823333333

Ndrg1	Q62433	Protein NDRG1 OS=Mus musculus OX=10090 GN=Ndrg1 PE=1 SV=1	0.8
Rps7	P62082	40S ribosomal protein S7 OS=Mus musculus OX=10090 GN=Rps7 PE=2 SV=1	0.91
Nptn	P97300	Neuroplastin OS=Mus musculus OX=10090 GN=Nptn PE=1 SV=3	0.806666667
Bub1b	Q9Z1S0	Mitotic checkpoint serine/threonine-protein kinase BUB1 beta OS=Mus musculus OX=10090 GN=Bu	0.66
Sh3bgrl	Q9JJU8	SH3 domain-binding glutamic acid-rich-like protein OS=Mus musculus OX=10090 GN=Sh3bgrl PE=1	0.883333333
Arpc5	Q9CPW4	Actin-related protein 2/3 complex subunit 5 OS=Mus musculus OX=10090 GN=Arpc5 PE=1 SV=3	0.826666667
Rbm3	O89086	RNA-binding protein 3 OS=Mus musculus OX=10090 GN=Rbm3 PE=1 SV=1	0.963333333
Emc8	O70378	ER membrane protein complex subunit 8 OS=Mus musculus OX=10090 GN=Emc8 PE=1 SV=1	1.01
Rpa2	Q62193	Replication protein A 32 kDa subunit OS=Mus musculus OX=10090 GN=Rpa2 PE=1 SV=1	0.816666667
Mrpl37	Q921S7	39S ribosomal protein L37 mitochondrial OS=Mus musculus OX=10090 GN=Mrpl37 PE=1 SV=1	0.75
Vps13a	Q5H8C4	Vacuolar protein sorting-associated protein 13A OS=Mus musculus OX=10090 GN=Vps13a PE=1 SV=	1.62
Cycs	P62897	Cytochrome c somatic OS=Mus musculus OX=10090 GN=Cycs PE=1 SV=2	0.75
Gipc1	Q9Z0G0	PDZ domain-containing protein GIPC1 OS=Mus musculus OX=10090 GN=Gipc1 PE=1 SV=1	0.986666667
Zfpl1	Q9DB43	Zinc finger protein-like 1 OS=Mus musculus OX=10090 GN=Zfpl1 PE=1 SV=1	0.973333333
Btf3l4	Q9CQH7	Transcription factor BTF3 homolog 4 OS=Mus musculus OX=10090 GN=Btf3l4 PE=1 SV=1	0.81
Pea15	Q62048	Astrocytic phosphoprotein PEA-15 OS=Mus musculus OX=10090 GN=Pea15 PE=1 SV=1	0.846666667
Chchd3	Q9CRB9	MICOS complex subunit Mic19 OS=Mus musculus OX=10090 GN=Chchd3 PE=1 SV=1	0.983333333
Thumpd3	P97770	THUMP domain-containing protein 3 OS=Mus musculus OX=10090 GN=Thumpd3 PE=1 SV=1	0.8
Denr	Q9CQJ6	Density-regulated protein OS=Mus musculus OX=10090 GN=Denr PE=1 SV=1	0.906666667
Aspscr1	Q8VBT9	Tether containing UBX domain for GLUT4 OS=Mus musculus OX=10090 GN=Aspscr1 PE=1 SV=1	0.973333333
Espl1	P60330	Separin OS=Mus musculus OX=10090 GN=Espl1 PE=1 SV=1	1.416666667
Mcu	Q3UMR5	Calcium uniporter protein mitochondrial OS=Mus musculus OX=10090 GN=Mcu PE=1 SV=2	0.85
Pafah1b2	Q61206	Platelet-activating factor acetylhydrolase IB subunit beta OS=Mus musculus OX=10090 GN=Pafah1b	0.82
Med1	Q925J9	Mediator of RNA polymerase II transcription subunit 1 OS=Mus musculus OX=10090 GN=Med1 PE=1	0.76
Mapkapk2	P49138	MAP kinase-activated protein kinase 2 OS=Mus musculus OX=10090 GN=Mapkapk2 PE=1 SV=2	0.896666667
Imp3	Q921Y2	U3 small nucleolar ribonucleoprotein protein IMP3 OS=Mus musculus OX=10090 GN=Imp3 PE=2 S\	0.82
Zranb2	Q9R020	Zinc finger Ran-binding domain-containing protein 2 OS=Mus musculus OX=10090 GN=Zranb2 PE=1	0.833333333
Pex19	Q8VCI5	Peroxisomal biogenesis factor 19 OS=Mus musculus OX=10090 GN=Pex19 PE=1 SV=1	0.833333333
Mfap1b	C0HKD9	Microfibrillar-associated protein 1B OS=Mus musculus OX=10090 GN=Mfap1b PE=1 SV=1	0.89
Mfap1a	C0HKD8	Microfibrillar-associated protein 1A OS=Mus musculus OX=10090 GN=Mfap1a PE=1 SV=1	0.89
Abcb10	Q9JI39	ATP-binding cassette sub-family B member 10 mitochondrial OS=Mus musculus OX=10090 GN=Abc	0.91
Tomm22	Q9CPQ3	Mitochondrial import receptor subunit TOM22 homolog OS=Mus musculus OX=10090 GN=Tomm2	0.826666667
Ddx52	Q8K301	Probable ATP-dependent RNA helicase DDX52 OS=Mus musculus OX=10090 GN=Ddx52 PE=2 SV=2	0.816666667
Sarnp	Q9D1J3	SAP domain-containing ribonucleoprotein OS=Mus musculus OX=10090 GN=Sarnp PE=1 SV=3	0.926666667

Cbwd1	Q8VEH6	COBW domain-containing protein 1 OS=Mus musculus OX=10090 GN=Cbwd1 PE=1 SV=1	0.9
Naalad2	Q9CZR2	N-acetylated-alpha-linked acidic dipeptidase 2 OS=Mus musculus OX=10090 GN=Naalad2 PE=1 SV=2	0.906666667
Rfc4	Q99J62	Replication factor C subunit 4 OS=Mus musculus OX=10090 GN=Rfc4 PE=1 SV=1	0.836666667
Syk	P48025	Tyrosine-protein kinase SYK OS=Mus musculus OX=10090 GN=Syk PE=1 SV=2	1.126666667
Ciao1	Q99KN2	Probable cytosolic iron-sulfur protein assembly protein CIAO1 OS=Mus musculus OX=10090 GN=Cia	0.773333333
Rbbp6	P97868	E3 ubiquitin-protein ligase RBBP6 OS=Mus musculus OX=10090 GN=Rbbp6 PE=1 SV=5	1.103333333
Snrpb	P27048	Small nuclear ribonucleoprotein-associated protein B OS=Mus musculus OX=10090 GN=Snrpb PE=1	0.923333333
Snrpn	P63163	Small nuclear ribonucleoprotein-associated protein N OS=Mus musculus OX=10090 GN=Snrpn PE=1	0.923333333
Arhgap5	P97393	Rho GTPase-activating protein 5 OS=Mus musculus OX=10090 GN=Arhgap5 PE=1 SV=2	1.206666667
Emc3	Q99KI3	ER membrane protein complex subunit 3 OS=Mus musculus OX=10090 GN=Emc3 PE=1 SV=3	0.84
Zwilch	Q8R060	Protein zwilch homolog OS=Mus musculus OX=10090 GN=Zwilch PE=1 SV=1	0.846666667
Mob4	Q6PEB6	MOB-like protein phocein OS=Mus musculus OX=10090 GN=Mob4 PE=1 SV=1	0.913333333
Rbm15	Q0VBL3	RNA-binding protein 15 OS=Mus musculus OX=10090 GN=Rbm15 PE=1 SV=1	0.96
Rab9a	Q9R0M6	Ras-related protein Rab-9A OS=Mus musculus OX=10090 GN=Rab9a PE=1 SV=1	0.883333333
Ngdn	Q9DB96	Neuroguidin OS=Mus musculus OX=10090 GN=Ngdn PE=1 SV=1	0.87
Fstl1	Q62356	Follistatin-related protein 1 OS=Mus musculus OX=10090 GN=Fstl1 PE=1 SV=2	1
Ist1	Q9CX00	IST1 homolog OS=Mus musculus OX=10090 GN=Ist1 PE=1 SV=1	0.923333333
Tmem209	Q8BRG8	Transmembrane protein 209 OS=Mus musculus OX=10090 GN=Tmem209 PE=2 SV=1	0.786666667
Vbp1	P61759	Prefoldin subunit 3 OS=Mus musculus OX=10090 GN=Vbp1 PE=1 SV=2	0.913333333
Pik3c3	Q6PF93	Phosphatidylinositol 3-kinase catalytic subunit type 3 OS=Mus musculus OX=10090 GN=Pik3c3 PE=	0.853333333
Rabggta	Q9JHK4	Geranylgeranyl transferase type-2 subunit alpha OS=Mus musculus OX=10090 GN=Rabggta PE=1 SV=	0.836666667
Ubl4a	P21126	Ubiquitin-like protein 4A OS=Mus musculus OX=10090 GN=Ubl4a PE=1 SV=1	0.886666667
Irs2	P81122	Insulin receptor substrate 2 OS=Mus musculus OX=10090 GN=Irs2 PE=1 SV=2	1.04
Lsm14b	Q8CGC4	Protein LSM14 homolog B OS=Mus musculus OX=10090 GN=Lsm14b PE=1 SV=3	0.88
Mgat1	P27808	Alpha-1 3-mannosyl-glycoprotein 2-beta-N-acetylglucosaminyltransferase OS=Mus musculus OX=10	0.98
Gnpnat1	Q9JK38	Glucosamine 6-phosphate N-acetyltransferase OS=Mus musculus OX=10090 GN=Gnpnat1 PE=1 SV=1	0.823333333
Msi2	Q920Q6	RNA-binding protein Musashi homolog 2 OS=Mus musculus OX=10090 GN=Msi2 PE=1 SV=1	0.83
Tinagl1	Q99JR5	Tubulointerstitial nephritis antigen-like OS=Mus musculus OX=10090 GN=Tinagl1 PE=1 SV=1	0.793333333
Ptges2	Q8BWM0	Prostaglandin E synthase 2 OS=Mus musculus OX=10090 GN=Ptges2 PE=1 SV=3	0.89
Mettl16	Q9CQG2	RNA N6-adenosine-methyltransferase METTL16 OS=Mus musculus OX=10090 GN=Mettl16 PE=1 SV=1	0.683333333
Ogfod1	Q3U0K8	Prolyl 3-hydroxylase OGFOD1 OS=Mus musculus OX=10090 GN=Ogfod1 PE=1 SV=1	0.86
Mnat1	P51949	CDK-activating kinase assembly factor MAT1 OS=Mus musculus OX=10090 GN=Mnat1 PE=1 SV=2	0.843333333
Psmb6	Q60692	Proteasome subunit beta type-6 OS=Mus musculus OX=10090 GN=Psmb6 PE=1 SV=3	0.856666667
Psmd8	Q9CX56	26S proteasome non-ATPase regulatory subunit 8 OS=Mus musculus OX=10090 GN=Psmd8 PE=1 SV	0.863333333

Dvl2	Q60838	Segment polarity protein dishevelled homolog DVL-2 OS=Mus musculus OX=10090 GN=Dvl2 PE=1 S	0.743333333
Arsb	P50429	Arylsulfatase B OS=Mus musculus OX=10090 GN=Arsb PE=1 SV=3	1.046666667
Rptor	Q8K4Q0	Regulatory-associated protein of mTOR OS=Mus musculus OX=10090 GN=Rptor PE=1 SV=1	1.73
Sun1	Q9D666	SUN domain-containing protein 1 OS=Mus musculus OX=10090 GN=Sun1 PE=1 SV=2	0.79
Prepl	Q8C167	Prolyl endopeptidase-like OS=Mus musculus OX=10090 GN=Prepl PE=1 SV=1	0.916666667
Akr7a2	Q8CG76	Aflatoxin B1 aldehyde reductase member 2 OS=Mus musculus OX=10090 GN=Akr7a2 PE=1 SV=3	0.973333333
Exosc4	Q921I9	Exosome complex component RRP41 OS=Mus musculus OX=10090 GN=Exosc4 PE=1 SV=3	0.903333333
Recql	Q9Z129	ATP-dependent DNA helicase Q1 OS=Mus musculus OX=10090 GN=Recql PE=1 SV=2	1.043333333
Mau2	Q9D2X5	MAU2 chromatid cohesion factor homolog OS=Mus musculus OX=10090 GN=Mau2 PE=1 SV=3	1.03
Ykt6	Q9CQW1	Synaptobrevin homolog YKT6 OS=Mus musculus OX=10090 GN=Ykt6 PE=1 SV=1	0.876666667
Nelfa	Q8BG30	Negative elongation factor A OS=Mus musculus OX=10090 GN=Nelfa PE=1 SV=1	0.776666667
Dera	Q91YP3	Deoxyribose-phosphate aldolase OS=Mus musculus OX=10090 GN=Dera PE=1 SV=1	0.746666667
Spag7	Q7TNE3	Sperm-associated antigen 7 OS=Mus musculus OX=10090 GN=Spag7 PE=1 SV=1	0.906666667
Emc7	Q9EP72	ER membrane protein complex subunit 7 OS=Mus musculus OX=10090 GN=Emc7 PE=1 SV=1	0.873333333
S100a11	P50543	Protein S100-A11 OS=Mus musculus OX=10090 GN=S100a11 PE=1 SV=1	0.903333333
Riok3	Q9DBU3	Serine/threonine-protein kinase RIO3 OS=Mus musculus OX=10090 GN=Riok3 PE=1 SV=3	0.99
Mecr	Q9DCS3	Enoyl-[acyl-carrier-protein] reductase mitochondrial OS=Mus musculus OX=10090 GN=Mecr PE=1 S	1.02
Rps23	P62267	40S ribosomal protein S23 OS=Mus musculus OX=10090 GN=Rps23 PE=1 SV=3	0.88
Crot	Q9DC50	Peroxisomal carnitine O-octanoyltransferase OS=Mus musculus OX=10090 GN=Crot PE=1 SV=1	0.906666667
Wiz	O88286	Protein Wiz OS=Mus musculus OX=10090 GN=Wiz PE=1 SV=2	0.75
Trmt5	Q9D0C4	tRNA (guanine(37)-N1)-methyltransferase OS=Mus musculus OX=10090 GN=Trmt5 PE=1 SV=1	1.006666667
Tsnax	Q9QZE7	Translin-associated protein X OS=Mus musculus OX=10090 GN=Tsnax PE=1 SV=1	0.876666667
Tor1b	Q9ER41	Torsin-1B OS=Mus musculus OX=10090 GN=Tor1b PE=1 SV=2	1.183333333
Arhgef18	Q6P9R4	Rho guanine nucleotide exchange factor 18 OS=Mus musculus OX=10090 GN=Arhgef18 PE=1 SV=3	0.806666667
Nubp1	Q9R060	Cytosolic Fe-S cluster assembly factor NUBP1 OS=Mus musculus OX=10090 GN=Nubp1 PE=1 SV=1	0.893333333
Hgh1	Q8C3I8	Protein HGH1 homolog OS=Mus musculus OX=10090 GN=Hgh1 PE=1 SV=1	0.883333333
Gopc	Q8BH60	Golgi-associated PDZ and coiled-coil motif-containing protein OS=Mus musculus OX=10090 GN=Go	0.913333333
	Q9D1K7	UPF0687 protein C20orf27 homolog OS=Mus musculus OX=10090 PE=1 SV=3	0.74
Tbl2	Q9R099	Transducin beta-like protein 2 OS=Mus musculus OX=10090 GN=Tbl2 PE=1 SV=2	1.01
Clpp	O88696	ATP-dependent Clp protease proteolytic subunit mitochondrial OS=Mus musculus OX=10090 GN=(	0.943333333
Inpp1	P49442	Inositol polyphosphate 1-phosphatase OS=Mus musculus OX=10090 GN=Inpp1 PE=1 SV=2	0.876666667
Hmbs	P22907	Porphobilinogen deaminase OS=Mus musculus OX=10090 GN=Hmbs PE=1 SV=2	0.803333333
Ssr1	Q9CY50	Translocon-associated protein subunit alpha OS=Mus musculus OX=10090 GN=Ssr1 PE=1 SV=1	0.88
5-Mar	Q3KNM2	E3 ubiquitin-protein ligase MARCHF5 OS=Mus musculus OX=10090 GN=Marchf5 PE=1 SV=1	0.84

Exoc6b	A6H5Z3	Exocyst complex component 6B OS=Mus musculus OX=10090 GN=Exoc6b PE=1 SV=1	0.783333333
Luzp1	Q8R4U7	Leucine zipper protein 1 OS=Mus musculus OX=10090 GN=Luzp1 PE=1 SV=2	0.916666667
Srrm1	Q52KI8	Serine/arginine repetitive matrix protein 1 OS=Mus musculus OX=10090 GN=Srrm1 PE=1 SV=2	0.776666667
Ermp1	Q3UVK0	Endoplasmic reticulum metallopeptidase 1 OS=Mus musculus OX=10090 GN=Ermp1 PE=1 SV=2	0.906666667
Wac	Q924H7	WW domain-containing adapter protein with coiled-coil OS=Mus musculus OX=10090 GN=Wac PE=	0.713333333
Gpx4	O70325	Phospholipid hydroperoxide glutathione peroxidase OS=Mus musculus OX=10090 GN=Gpx4 PE=1 S	0.763333333
Pigt	Q8BXQ2	GPI transamidase component PIG-T OS=Mus musculus OX=10090 GN=Pigt PE=1 SV=2	1.04
Wasf2	Q8BH43	Wiskott-Aldrich syndrome protein family member 2 OS=Mus musculus OX=10090 GN=Wasf2 PE=1 S	0.963333333
Qtrt1	Q9JMA2	Queuine tRNA-ribosyltransferase catalytic subunit 1 OS=Mus musculus OX=10090 GN=Qtrt1 PE=1 S\	1.286666667
Slc29a1	Q9JIM1	Equilibrative nucleoside transporter 1 OS=Mus musculus OX=10090 GN=Slc29a1 PE=1 SV=3	0.823333333
	Q91V76	Ester hydrolase C11orf54 homolog OS=Mus musculus OX=10090 PE=1 SV=1	0.81
Cnpy4	Q8BQ47	Protein canopy homolog 4 OS=Mus musculus OX=10090 GN=Cnpy4 PE=1 SV=1	0.91
Tmem106b	Q80X71	Transmembrane protein 106B OS=Mus musculus OX=10090 GN=Tmem106b PE=1 SV=1	1.006666667
Ppan	Q91YU8	Suppressor of SWI4 1 homolog OS=Mus musculus OX=10090 GN=Ppan PE=1 SV=2	0.85
Mrps35	Q8BJZ4	28S ribosomal protein S35 mitochondrial OS=Mus musculus OX=10090 GN=Mrps35 PE=1 SV=2	1.056666667
Pard3	Q99NH2	Partitioning defective 3 homolog OS=Mus musculus OX=10090 GN=Pard3 PE=1 SV=2	1.25
Necap1	Q9CR95	Adaptin ear-binding coat-associated protein 1 OS=Mus musculus OX=10090 GN=Necap1 PE=1 SV=2	1.09
Nacc1	Q7TSZ8	Nucleus accumbens-associated protein 1 OS=Mus musculus OX=10090 GN=Nacc1 PE=1 SV=1	0.753333333
Prpf38b	Q80SY5	Pre-mRNA-splicing factor 38B OS=Mus musculus OX=10090 GN=Prpf38b PE=1 SV=1	0.883333333
Thoc3	Q8VE80	THO complex subunit 3 OS=Mus musculus OX=10090 GN=Thoc3 PE=2 SV=1	1.123333333
Rabepk	Q8VCH5	Rab9 effector protein with kelch motifs OS=Mus musculus OX=10090 GN=Rabepk PE=1 SV=2	1.05
Incenp	Q9WU62	Inner centromere protein OS=Mus musculus OX=10090 GN=Incenp PE=1 SV=2	0.933333333
Ddi2	A2ADY9	Protein DDI1 homolog 2 OS=Mus musculus OX=10090 GN=Ddi2 PE=1 SV=1	0.73
Adam10	O35598	Disintegrin and metalloproteinase domain-containing protein 10 OS=Mus musculus OX=10090 GN=	0.91
Sugp1	Q8CH02	SURP and G-patch domain-containing protein 1 OS=Mus musculus OX=10090 GN=Sugp1 PE=1 SV=1	1.006666667
Thoc7	Q7TMY4	THO complex subunit 7 homolog OS=Mus musculus OX=10090 GN=Thoc7 PE=1 SV=2	0.943333333
Gk	Q64516	Glycerol kinase OS=Mus musculus OX=10090 GN=Gk PE=1 SV=2	0.793333333
Znf512	Q69Z99	Zinc finger protein 512 OS=Mus musculus OX=10090 GN=Znf512 PE=2 SV=2	1.033333333
DSCC1	Q14AI0	Sister chromatid cohesion protein DCC1 OS=Mus musculus OX=10090 GN=DSCC1 PE=2 SV=1	0.85
Nme4	Q9WV84	Nucleoside diphosphate kinase mitochondrial OS=Mus musculus OX=10090 GN=Nme4 PE=1 SV=1	0.883333333
Arl2	Q9D0J4	ADP-ribosylation factor-like protein 2 OS=Mus musculus OX=10090 GN=Arl2 PE=1 SV=1	0.896666667
Pcyt1a	P49586	Choline-phosphate cytidylyltransferase A OS=Mus musculus OX=10090 GN=Pcyt1a PE=1 SV=1	0.956666667
Ndufs2	Q91WD5	NADH dehydrogenase [ubiquinone] iron-sulfur protein 2 mitochondrial OS=Mus musculus OX=100!	0.693333333
Pfdn6	Q03958	Prefoldin subunit 6 OS=Mus musculus OX=10090 GN=Pfdn6 PE=1 SV=1	0.896666667

Tbcb	Q9D1E6	Tubulin-folding cofactor B OS=Mus musculus OX=10090 GN=Tbcb PE=1 SV=2	0.953333333
Cryab	P23927	Alpha-crystallin B chain OS=Mus musculus OX=10090 GN=Cryab PE=1 SV=2	0.846666667
Mrpl45	Q9D0Q7	39S ribosomal protein L45 mitochondrial OS=Mus musculus OX=10090 GN=Mrpl45 PE=1 SV=1	0.99
Osbpl1a	Q91XL9	Oxysterol-binding protein-related protein 1 OS=Mus musculus OX=10090 GN=Osbpl1a PE=1 SV=2	0.836666667
Commd9	Q8K2Q0	COMM domain-containing protein 9 OS=Mus musculus OX=10090 GN=Commd9 PE=1 SV=3	1.133333333
Wls	Q6DID7	Protein wntless homolog OS=Mus musculus OX=10090 GN=Wls PE=1 SV=1	0.79
Pkn1	P70268	Serine/threonine-protein kinase N1 OS=Mus musculus OX=10090 GN=Pkn1 PE=1 SV=3	1.286666667
Cog5	Q8C0L8	Conserved oligomeric Golgi complex subunit 5 OS=Mus musculus OX=10090 GN=Cog5 PE=1 SV=3	1.09
Arf6	P62331	ADP-ribosylation factor 6 OS=Mus musculus OX=10090 GN=Arf6 PE=1 SV=2	0.833333333
Lactb2	Q99KR3	Endoribonuclease LACTB2 OS=Mus musculus OX=10090 GN=Lactb2 PE=1 SV=1	0.796666667
Cog1	Q9Z160	Conserved oligomeric Golgi complex subunit 1 OS=Mus musculus OX=10090 GN=Cog1 PE=1 SV=3	1.206666667
Rcl1	Q9JJT0	RNA 3'-terminal phosphate cyclase-like protein OS=Mus musculus OX=10090 GN=Rcl1 PE=2 SV=1	0.83
Clpb	Q60649	Caseinolytic peptidase B protein homolog OS=Mus musculus OX=10090 GN=Clpb PE=1 SV=1	0.733333333
Ttc39b	Q8BYY4	Tetratricopeptide repeat protein 39B OS=Mus musculus OX=10090 GN=Ttc39b PE=1 SV=1	0.776666667
Cpsf4	Q8BQZ5	Cleavage and polyadenylation specificity factor subunit 4 OS=Mus musculus OX=10090 GN=Cpsf4 PI	0.923333333
Wtap	Q9ER69	Pre-mRNA-splicing regulator WTAP OS=Mus musculus OX=10090 GN=Wtap PE=1 SV=3	0.996666667
Erlec1	Q8VEH8	Endoplasmic reticulum lectin 1 OS=Mus musculus OX=10090 GN=Erlec1 PE=1 SV=1	1.036666667
Ciao2b	Q9D187	Cytosolic iron-sulfur assembly component 2B OS=Mus musculus OX=10090 GN=Ciao2b PE=1 SV=1	0.96
Dctd	Q8K2D6	Deoxycytidylate deaminase OS=Mus musculus OX=10090 GN=Dctd PE=2 SV=1	0.96
Isoc1	Q91V64	Isochorismatase domain-containing protein 1 OS=Mus musculus OX=10090 GN=Isoc1 PE=1 SV=1	0.836666667
Rpa3	Q9CQ71	Replication protein A 14 kDa subunit OS=Mus musculus OX=10090 GN=Rpa3 PE=1 SV=1	0.89
Lrrc41	Q8K1C9	Leucine-rich repeat-containing protein 41 OS=Mus musculus OX=10090 GN=Lrrc41 PE=1 SV=3	1.84
Hdac7	Q8C2B3	Histone deacetylase 7 OS=Mus musculus OX=10090 GN=Hdac7 PE=1 SV=2	1.116666667
Mtx1	P47802	Metaxin-1 OS=Mus musculus OX=10090 GN=Mtx1 PE=1 SV=1	0.906666667
Nipsnap1	O55125	Protein NipSnap homolog 1 OS=Mus musculus OX=10090 GN=Nipsnap1 PE=1 SV=1	0.796666667
Eif4e2	Q8BMB3	Eukaryotic translation initiation factor 4E type 2 OS=Mus musculus OX=10090 GN=Eif4e2 PE=1 SV=1	0.766666667
Gmppb	Q8BTZ7	Mannose-1-phosphate guanyltransferase beta OS=Mus musculus OX=10090 GN=Gmppb PE=1 SV=1	0.83
Dph5	Q9CWQ0	Diphthine methyl ester synthase OS=Mus musculus OX=10090 GN=Dph5 PE=1 SV=2	1.126666667
Nt5c	Q9JM14	5'(3')-deoxyribonucleotidase cytosolic type OS=Mus musculus OX=10090 GN=Nt5c PE=1 SV=1	0.883333333
Rpl24	Q8BP67	60S ribosomal protein L24 OS=Mus musculus OX=10090 GN=Rpl24 PE=1 SV=2	0.916666667
Mrpl38	Q8K2M0	39S ribosomal protein L38 mitochondrial OS=Mus musculus OX=10090 GN=Mrpl38 PE=1 SV=2	0.846666667
Ppig	A2AR02	Peptidyl-prolyl cis-trans isomerase G OS=Mus musculus OX=10090 GN=Ppig PE=1 SV=1	0.943333333
Hddc2	Q3SXD3	5'-deoxynucleotidase HDDC2 OS=Mus musculus OX=10090 GN=Hddc2 PE=1 SV=1	0.8
Ldb1	P70662	LIM domain-binding protein 1 OS=Mus musculus OX=10090 GN=Ldb1 PE=1 SV=2	0.643333333

Osbpl11	Q8CI95	Oxysterol-binding protein-related protein 11 OS=Mus musculus OX=10090 GN=Osbpl11 PE=1 SV=2	1.173333333
Hexim1	Q8R409	Protein HEXIM1 OS=Mus musculus OX=10090 GN=Hexim1 PE=1 SV=1	0.966666667
Rnf214	Q8BFU3	RING finger protein 214 OS=Mus musculus OX=10090 GN=Rnf214 PE=1 SV=1	1.196666667
Naga	Q9QWR8	Alpha-N-acetylgalactosaminidase OS=Mus musculus OX=10090 GN=Naga PE=1 SV=2	1.153333333
Nipsnap2	O55126	Protein NipSnap homolog 2 OS=Mus musculus OX=10090 GN=Nipsnap2 PE=1 SV=1	0.973333333
2-Mar	Q922Q1	Mitochondrial amidoxime reducing component 2 OS=Mus musculus OX=10090 GN=Mtarc2 PE=1 S\	0.833333333
Ech1	O35459	Delta(3 5)-Delta(2 4)-dienoyl-CoA isomerase mitochondrial OS=Mus musculus OX=10090 GN=Ech1	4.313333333
Smc5	Q8CG46	Structural maintenance of chromosomes protein 5 OS=Mus musculus OX=10090 GN=Smc5 PE=1 SV	1.103333333
Rtf1	A2AQ19	RNA polymerase-associated protein RTF1 homolog OS=Mus musculus OX=10090 GN=Rtf1 PE=1 SV=1	0.926666667
Ythdc2	B2RR83	3'-5' RNA helicase YTHDC2 OS=Mus musculus OX=10090 GN=Ythdc2 PE=1 SV=1	1.173333333
Ndufa4	Q62425	Cytochrome c oxidase subunit NDUFA4 OS=Mus musculus OX=10090 GN=Ndufa4 PE=1 SV=2	0.813333333
Dnajb12	Q9QYI4	DnaJ homolog subfamily B member 12 OS=Mus musculus OX=10090 GN=Dnajb12 PE=1 SV=2	1.183333333
Echdc1	Q9D9V3	Ethylmalonyl-CoA decarboxylase OS=Mus musculus OX=10090 GN=Echdc1 PE=1 SV=2	0.963333333
Pter	Q60866	Phosphotriesterase-related protein OS=Mus musculus OX=10090 GN=Pter PE=1 SV=1	0.906666667
Vps53	Q8CCB4	Vacuolar protein sorting-associated protein 53 homolog OS=Mus musculus OX=10090 GN=Vps53 PI	0.803333333
Zfyve16	Q80U44	Zinc finger FYVE domain-containing protein 16 OS=Mus musculus OX=10090 GN=Zfyve16 PE=1 SV=2	1.106666667
Glb1	P23780	Beta-galactosidase OS=Mus musculus OX=10090 GN=Glb1 PE=1 SV=1	1.046666667
Map3k7	Q62073	Mitogen-activated protein kinase kinase kinase 7 OS=Mus musculus OX=10090 GN=Map3k7 PE=1 SV	1.226666667
Pdcd6	P12815	Programmed cell death protein 6 OS=Mus musculus OX=10090 GN=Pdcd6 PE=1 SV=2	0.87
Nelfcd	Q922L6	Negative elongation factor D OS=Mus musculus OX=10090 GN=Nelfcd PE=1 SV=2	1.13
Vps52	Q8C754	Vacuolar protein sorting-associated protein 52 homolog OS=Mus musculus OX=10090 GN=Vps52 PI	1.096666667
Wdr55	Q9CX97	WD repeat-containing protein 55 OS=Mus musculus OX=10090 GN=Wdr55 PE=1 SV=2	0.873333333
Dcakd	Q8BHC4	Dephospho-CoA kinase domain-containing protein OS=Mus musculus OX=10090 GN=Dcakd PE=1 S\	0.823333333
Npepl1	Q6NSR8	Probable aminopeptidase NPEPL1 OS=Mus musculus OX=10090 GN=Npepl1 PE=1 SV=1	0.856666667
Ppp1r10	Q80W00	Serine/threonine-protein phosphatase 1 regulatory subunit 10 OS=Mus musculus OX=10090 GN=Pp	0.696666667
Pdcd2	P46718	Programmed cell death protein 2 OS=Mus musculus OX=10090 GN=Pdcd2 PE=2 SV=2	0.95
Ndufaf2	Q59J78	NADH dehydrogenase [ubiquinone] 1 alpha subcomplex assembly factor 2 OS=Mus musculus OX=10	0.733333333
Pigk	Q9CXY9	GPI-anchor transamidase OS=Mus musculus OX=10090 GN=Pigk PE=1 SV=2	0.97
Cavin3	Q91VJ2	Caveolae-associated protein 3 OS=Mus musculus OX=10090 GN=Cavin3 PE=1 SV=1	0.893333333
Dcp1a	Q91YD3	mRNA-decapping enzyme 1A OS=Mus musculus OX=10090 GN=Dcp1a PE=1 SV=1	0.876666667
Lamtor3	O88653	Ragulator complex protein LAMTOR3 OS=Mus musculus OX=10090 GN=Lamtor3 PE=1 SV=1	0.793333333
Cdh3	P10287	Cadherin-3 OS=Mus musculus OX=10090 GN=Cdh3 PE=1 SV=2	1.446666667
Arpin	Q9D0A3	Arpin OS=Mus musculus OX=10090 GN=Arpin PE=1 SV=1	0.726666667
Cbl	P22682	E3 ubiquitin-protein ligase CBL OS=Mus musculus OX=10090 GN=Cbl PE=1 SV=3	1.06

Apbb1ip	Q8R5A3	Amyloid beta A4 precursor protein-binding family B member 1-interacting protein OS=Mus muscul	0.706666667
Ggcx	Q9QYC7	Vitamin K-dependent gamma-carboxylase OS=Mus musculus OX=10090 GN=Ggcx PE=1 SV=1	0.823333333
Eri1	Q7TMF2	3'-5' exoribonuclease 1 OS=Mus musculus OX=10090 GN=Eri1 PE=1 SV=2	0.93
Lnpk	Q7TQ95	Endoplasmic reticulum junction formation protein lunapark OS=Mus musculus OX=10090 GN=Lnp	0.923333333
Aldoc	P05063	Fructose-bisphosphate aldolase C OS=Mus musculus OX=10090 GN=Aldoc PE=1 SV=4	
Arl8a	Q8VEH3	ADP-ribosylation factor-like protein 8A OS=Mus musculus OX=10090 GN=Arl8a PE=1 SV=1	0.663333333
Atp2b2	Q9R0K7	Plasma membrane calcium-transporting ATPase 2 OS=Mus musculus OX=10090 GN=Atp2b2 PE=1 S	0.826666667
Gfpt2	Q9Z2Z9	Glutamine--fructose-6-phosphate aminotransferase [isomerizing] 2 OS=Mus musculus OX=10090 G	
Nr2f1	Q60632	COUP transcription factor 1 OS=Mus musculus OX=10090 GN=Nr2f1 PE=2 SV=2	0.53
Cux1	P70403	Protein CASP OS=Mus musculus OX=10090 GN=Cux1 PE=1 SV=2	
Stk4	Q9JI11	Serine/threonine-protein kinase 4 OS=Mus musculus OX=10090 GN=Stk4 PE=1 SV=1	0.713333333
Rab8b	P61028	Ras-related protein Rab-8B OS=Mus musculus OX=10090 GN=Rab8b PE=1 SV=1	0.86
Sptb	P15508	Spectrin beta chain erythrocytic OS=Mus musculus OX=10090 GN=Sptb PE=1 SV=4	0.395
Kras	P32883	GTPase KRas OS=Mus musculus OX=10090 GN=Kras PE=1 SV=1	0.99
Kpna1	Q60960	Importin subunit alpha-5 OS=Mus musculus OX=10090 GN=Kpna1 PE=1 SV=2	0.773333333
Mark3	Q03141	MAP/microtubule affinity-regulating kinase 3 OS=Mus musculus OX=10090 GN=Mark3 PE=1 SV=2	1.34
Ifi204	P0DOV2	Interferon-activable protein 204 OS=Mus musculus OX=10090 GN=Ifi204 PE=1 SV=1	1.306666667
Ppp3ca	P63328	Serine/threonine-protein phosphatase 2B catalytic subunit alpha isoform OS=Mus musculus OX=10	1.77
Mbnl2	Q8C181	Muscleblind-like protein 2 OS=Mus musculus OX=10090 GN=Mbnl2 PE=2 SV=2	1.116666667
Rab6a	P35279	Ras-related protein Rab-6A OS=Mus musculus OX=10090 GN=Rab6a PE=1 SV=4	0.826666667
Akt2	Q60823	RAC-beta serine/threonine-protein kinase OS=Mus musculus OX=10090 GN=Akt2 PE=1 SV=1	0.87
Stk38l	Q7TSE6	Serine/threonine-protein kinase 38-like OS=Mus musculus OX=10090 GN=Stk38l PE=1 SV=2	0.89
Itch	Q8C863	E3 ubiquitin-protein ligase Itchy OS=Mus musculus OX=10090 GN=Itch PE=1 SV=2	1.133333333
Arid1b	E9Q4N7	AT-rich interactive domain-containing protein 1B OS=Mus musculus OX=10090 GN=Arid1b PE=1 SV	1.093333333
Rab5b	P61021	Ras-related protein Rab-5B OS=Mus musculus OX=10090 GN=Rab5b PE=1 SV=1	0.773333333
Git2	Q9JLQ2	ARF GTPase-activating protein GIT2 OS=Mus musculus OX=10090 GN=Git2 PE=1 SV=2	1.153333333
Git1	Q68FF6	ARF GTPase-activating protein GIT1 OS=Mus musculus OX=10090 GN=Git1 PE=1 SV=1	0.976666667
Ralb	Q9JIW9	Ras-related protein Ral-B OS=Mus musculus OX=10090 GN=Ralb PE=1 SV=1	0.886666667
Srsf6	Q3TWW8	Serine/arginine-rich splicing factor 6 OS=Mus musculus OX=10090 GN=Srsf6 PE=1 SV=1	1.006666667
Raf1	Q99N57	RAF proto-oncogene serine/threonine-protein kinase OS=Mus musculus OX=10090 GN=Raf1 PE=1 S	0.77
Rras	P10833	Ras-related protein R-Ras OS=Mus musculus OX=10090 GN=Rras PE=1 SV=1	0.8
Ppm1b	P36993	Protein phosphatase 1B OS=Mus musculus OX=10090 GN=Ppm1b PE=1 SV=1	0.926666667
Chd8	Q09XV5	Chromodomain-helicase-DNA-binding protein 8 OS=Mus musculus OX=10090 GN=Chd8 PE=1 SV=1	1.13
Cdk14	O35495	Cyclin-dependent kinase 14 OS=Mus musculus OX=10090 GN=Cdk14 PE=1 SV=2	1.21

Ubp1	Q811S7	Upstream-binding protein 1 OS=Mus musculus OX=10090 GN=Ubp1 PE=1 SV=1	1.023333333
Afg3l1	Q920A7	AFG3-like protein 1 OS=Mus musculus OX=10090 GN=Afg3l1 PE=1 SV=2	0.893333333
Cbx5	Q61686	Chromobox protein homolog 5 OS=Mus musculus OX=10090 GN=Cbx5 PE=1 SV=1	0.88
Arl3	Q9WUL7	ADP-ribosylation factor-like protein 3 OS=Mus musculus OX=10090 GN=Arl3 PE=1 SV=1	0.883333333
Ppic	P30412	Peptidyl-prolyl cis-trans isomerase C OS=Mus musculus OX=10090 GN=Ppic PE=1 SV=1	0.916666667
Dnajb4	Q9D832	DnaJ homolog subfamily B member 4 OS=Mus musculus OX=10090 GN=Dnajb4 PE=1 SV=1	1.03
Pgrmc2	Q80UU9	Membrane-associated progesterone receptor component 2 OS=Mus musculus OX=10090 GN=Pgrm	0.8
Rela	Q04207	Transcription factor p65 OS=Mus musculus OX=10090 GN=Rela PE=1 SV=1	0.86
Ptbp3	Q8BHD7	Polypyrimidine tract-binding protein 3 OS=Mus musculus OX=10090 GN=Ptbp3 PE=1 SV=1	1.06
Ppp6r1	Q7TSI3	Serine/threonine-protein phosphatase 6 regulatory subunit 1 OS=Mus musculus OX=10090 GN=Ppp	1.126666667
Srsf5	O35326	Serine/arginine-rich splicing factor 5 OS=Mus musculus OX=10090 GN=Srsf5 PE=1 SV=2	0.836666667
Ppp4r3a	Q6P2K6	Serine/threonine-protein phosphatase 4 regulatory subunit 3A OS=Mus musculus OX=10090 GN=Pp	1.386666667
Celf2	Q9Z0H4	CUGBP Elav-like family member 2 OS=Mus musculus OX=10090 GN=Celf2 PE=1 SV=1	1.056666667
A2m	Q6GQT1	Alpha-2-macroglobulin-P OS=Mus musculus OX=10090 GN=A2m PE=2 SV=2	0.066666667
Trim33	Q99PP7	E3 ubiquitin-protein ligase TRIM33 OS=Mus musculus OX=10090 GN=Trim33 PE=1 SV=2	1.16
Gnpda2	Q9CRC9	Glucosamine-6-phosphate isomerase 2 OS=Mus musculus OX=10090 GN=Gnpda2 PE=1 SV=1	0.97
Ewsr1	Q61545	RNA-binding protein EWS OS=Mus musculus OX=10090 GN=Ewsr1 PE=1 SV=2	0.81
Sod1	P08228	Superoxide dismutase [Cu-Zn] OS=Mus musculus OX=10090 GN=Sod1 PE=1 SV=2	0.876666667
Psmb4	P99026	Proteasome subunit beta type-4 OS=Mus musculus OX=10090 GN=Psmb4 PE=1 SV=1	0.91
Armcx2	Q6A058	Armadillo repeat-containing X-linked protein 2 OS=Mus musculus OX=10090 GN=Armcx2 PE=2 SV=2	0.893333333
Alad	P10518	Delta-aminolevulinic acid dehydratase OS=Mus musculus OX=10090 GN=Alad PE=1 SV=1	0.946666667
Pdap1	Q3UHX2	28 kDa heat- and acid-stable phosphoprotein OS=Mus musculus OX=10090 GN=Pdap1 PE=1 SV=1	0.84
Cfdp1	O88271	Craniofacial development protein 1 OS=Mus musculus OX=10090 GN=Cfdp1 PE=1 SV=1	0.936666667
Prmt3	Q922H1	Protein arginine N-methyltransferase 3 OS=Mus musculus OX=10090 GN=Prmt3 PE=1 SV=2	0.91
Tmed10	Q9D1D4	Transmembrane emp24 domain-containing protein 10 OS=Mus musculus OX=10090 GN=Tmed10 P	0.923333333
Rpl23	P62830	60S ribosomal protein L23 OS=Mus musculus OX=10090 GN=Rpl23 PE=1 SV=1	0.883333333
Bax	Q07813	Apoptosis regulator BAX OS=Mus musculus OX=10090 GN=Bax PE=1 SV=1	0.866666667
Get4	Q9D1H7	Golgi to ER traffic protein 4 homolog OS=Mus musculus OX=10090 GN=Get4 PE=1 SV=2	0.866666667
Lxn	P70202	Latexin OS=Mus musculus OX=10090 GN=Lxn PE=1 SV=2	0.943333333
Stoml2	Q99JB2	Stomatin-like protein 2 mitochondrial OS=Mus musculus OX=10090 GN=Stoml2 PE=1 SV=1	0.88
Strip1	Q8C079	Striatin-interacting protein 1 OS=Mus musculus OX=10090 GN=Strip1 PE=1 SV=2	0.816666667
Psmb2	Q9R1P3	Proteasome subunit beta type-2 OS=Mus musculus OX=10090 GN=Psmb2 PE=1 SV=1	0.846666667
Ociad1	Q9CRD0	OCIA domain-containing protein 1 OS=Mus musculus OX=10090 GN=Ociad1 PE=1 SV=1	0.813333333
Hmgn5	Q9JL35	High mobility group nucleosome-binding domain-containing protein 5 OS=Mus musculus OX=1009	0.84

M6pr	P24668	Cation-dependent mannose-6-phosphate receptor OS=Mus musculus OX=10090 GN=M6pr PE=1 SV=	0.806666667
Poldip2	Q91VA6	Polymerase delta-interacting protein 2 OS=Mus musculus OX=10090 GN=Poldip2 PE=1 SV=1	0.856666667
Fkbp8	O35465	Peptidyl-prolyl cis-trans isomerase FKBP8 OS=Mus musculus OX=10090 GN=Fkbp8 PE=1 SV=2	0.853333333
Axl	Q00993	Tyrosine-protein kinase receptor UFO OS=Mus musculus OX=10090 GN=Axl PE=1 SV=2	0.826666667
Nt5c3b	Q3UFY7	7-methylguanosine phosphate-specific 5'-nucleotidase OS=Mus musculus OX=10090 GN=Nt5c3b PE	0.893333333
Fkbp2	P45878	Peptidyl-prolyl cis-trans isomerase FKBP2 OS=Mus musculus OX=10090 GN=Fkbp2 PE=1 SV=1	0.906666667
Grpel1	Q99LP6	GrpE protein homolog 1 mitochondrial OS=Mus musculus OX=10090 GN=Grpel1 PE=1 SV=1	0.863333333
Otud4	B2RRE7	OTU domain-containing protein 4 OS=Mus musculus OX=10090 GN=Otud4 PE=1 SV=1	0.96
Map2k3	O09110	Dual specificity mitogen-activated protein kinase kinase 3 OS=Mus musculus OX=10090 GN=Map2k	0.786666667
Rrp9	Q91WM3	U3 small nucleolar RNA-interacting protein 2 OS=Mus musculus OX=10090 GN=Rrp9 PE=1 SV=1	0.91
Comt	O88587	Catechol O-methyltransferase OS=Mus musculus OX=10090 GN=Comt PE=1 SV=2	0.906666667
Ciapin1	Q8WTY4	Anamorsin OS=Mus musculus OX=10090 GN=Ciapin1 PE=1 SV=1	0.9
Sfr1	Q8BP27	Swi5-dependent recombination DNA repair protein 1 homolog OS=Mus musculus OX=10090 GN=Sf	0.79
Mlec	Q6ZQI3	Malectin OS=Mus musculus OX=10090 GN=Mlec PE=1 SV=2	1.01
Ttc1	Q91Z38	Tetratricopeptide repeat protein 1 OS=Mus musculus OX=10090 GN=Ttc1 PE=1 SV=1	0.886666667
Tut7	Q5BLK4	Terminal uridylyltransferase 7 OS=Mus musculus OX=10090 GN=Tut7 PE=1 SV=3	0.96
Aldh16a1	Q571I9	Aldehyde dehydrogenase family 16 member A1 OS=Mus musculus OX=10090 GN=Aldh16a1 PE=1 SV	0.893333333
Rrp1	P56183	Ribosomal RNA processing protein 1 homolog A OS=Mus musculus OX=10090 GN=Rrp1 PE=1 SV=2	1.096666667
Fmn1	Q05860	Formin-1 OS=Mus musculus OX=10090 GN=Fmn1 PE=1 SV=2	0.746666667
Cbfb	Q08024	Core-binding factor subunit beta OS=Mus musculus OX=10090 GN=Cbfb PE=1 SV=1	0.946666667
Mad2l1	Q9Z1B5	Mitotic spindle assembly checkpoint protein MAD2A OS=Mus musculus OX=10090 GN=Mad2l1 PE=	0.93
Bcl2l13	P59017	Bcl-2-like protein 13 OS=Mus musculus OX=10090 GN=Bcl2l13 PE=1 SV=2	1.006666667
Armc8	Q9DBR3	Armadillo repeat-containing protein 8 OS=Mus musculus OX=10090 GN=Armc8 PE=1 SV=2	0.856666667
Atp5pb	Q9CQQ7	ATP synthase F(0) complex subunit B1 mitochondrial OS=Mus musculus OX=10090 GN=Atp5pb PE=	0.913333333
Sik3	Q6P4S6	Serine/threonine-protein kinase SIK3 OS=Mus musculus OX=10090 GN=Sik3 PE=1 SV=3	1.02
Mmut	P16332	Methylmalonyl-CoA mutase mitochondrial OS=Mus musculus OX=10090 GN=Mmut PE=1 SV=2	0.84
Nudt9	Q8BVU5	ADP-ribose pyrophosphatase mitochondrial OS=Mus musculus OX=10090 GN=Nudt9 PE=1 SV=1	0.98
Acadm	P45952	Medium-chain specific acyl-CoA dehydrogenase mitochondrial OS=Mus musculus OX=10090 GN=A	0.906666667
Fcho2	Q3UQN2	F-BAR domain only protein 2 OS=Mus musculus OX=10090 GN=Fcho2 PE=1 SV=1	1.023333333
Rpl30	P62889	60S ribosomal protein L30 OS=Mus musculus OX=10090 GN=Rpl30 PE=1 SV=2	0.973333333
Gamt	O35969	Guanidinoacetate N-methyltransferase OS=Mus musculus OX=10090 GN=Gamt PE=1 SV=1	0.846666667
Mvk	Q9R008	Mevalonate kinase OS=Mus musculus OX=10090 GN=Mvk PE=1 SV=1	0.92
Yy1	Q00899	Transcriptional repressor protein YY1 OS=Mus musculus OX=10090 GN=Yy1 PE=1 SV=1	0.86
Rab11fip5	Q8R361	Rab11 family-interacting protein 5 OS=Mus musculus OX=10090 GN=Rab11fip5 PE=1 SV=2	0.846666667

Alg2	Q9DBE8	Alpha-1 3/1 6-mannosyltransferase ALG2 OS=Mus musculus OX=10090 GN=Alg2 PE=1 SV=2	0.92
Zc3h18	Q0P678	Zinc finger CCCH domain-containing protein 18 OS=Mus musculus OX=10090 GN=Zc3h18 PE=1 SV=:	0.706666667
Cwc27	Q3TKY6	Spliceosome-associated protein CWC27 homolog OS=Mus musculus OX=10090 GN=Cwc27 PE=1 SV	0.95
Hexa	P29416	Beta-hexosaminidase subunit alpha OS=Mus musculus OX=10090 GN=Hexa PE=1 SV=2	0.94
Prpf4b	Q61136	Serine/threonine-protein kinase PRP4 homolog OS=Mus musculus OX=10090 GN=Prpf4b PE=1 SV=3	0.723333333
Cav1	P49817	Caveolin-1 OS=Mus musculus OX=10090 GN=Cav1 PE=1 SV=1	0.853333333
Cpped1	Q8BFS6	Serine/threonine-protein phosphatase CPPED1 OS=Mus musculus OX=10090 GN=Cpped1 PE=1 SV=:	0.82
Psme2	P97372	Proteasome activator complex subunit 2 OS=Mus musculus OX=10090 GN=Psme2 PE=1 SV=4	0.886666667
Mcat	Q8R3F5	Malonyl-CoA-acyl carrier protein transacylase mitochondrial OS=Mus musculus OX=10090 GN=Mca	1.036666667
Ivd	Q9JHI5	Isovaleryl-CoA dehydrogenase mitochondrial OS=Mus musculus OX=10090 GN=Ivd PE=1 SV=1	0.826666667
Ctc1	Q5SUQ9	CST complex subunit CTC1 OS=Mus musculus OX=10090 GN=Ctc1 PE=1 SV=2	0.906666667
Ak1	Q9R0Y5	Adenylate kinase isoenzyme 1 OS=Mus musculus OX=10090 GN=Ak1 PE=1 SV=1	0.91
Wdr33	Q8K4P0	pre-mRNA 3' end processing protein WDR33 OS=Mus musculus OX=10090 GN=Wdr33 PE=1 SV=1	1.113333333
Armt1	A6H630	Damage-control phosphatase ARMT1 OS=Mus musculus OX=10090 GN=Armt1 PE=1 SV=1	1.106666667
Htt	P42859	Huntingtin OS=Mus musculus OX=10090 GN=Htt PE=1 SV=2	1.18
Ccnd1	P25322	G1/S-specific cyclin-D1 OS=Mus musculus OX=10090 GN=Ccnd1 PE=1 SV=1	1.12
Arl1	P61211	ADP-ribosylation factor-like protein 1 OS=Mus musculus OX=10090 GN=Arl1 PE=1 SV=1	0.883333333
Spg21	Q9CQC8	Maspardin OS=Mus musculus OX=10090 GN=Spg21 PE=1 SV=1	0.82
Adpgk	Q8VDL4	ADP-dependent glucokinase OS=Mus musculus OX=10090 GN=Adpgk PE=1 SV=2	0.836666667
Nol9	Q3TZX8	Polynucleotide 5'-hydroxyl-kinase NOL9 OS=Mus musculus OX=10090 GN=Nol9 PE=1 SV=1	0.996666667
Echs1	Q8BH95	Enoyl-CoA hydratase mitochondrial OS=Mus musculus OX=10090 GN=Echs1 PE=1 SV=1	0.89
Pofut1	Q91ZW2	GDP-fucose protein O-fucosyltransferase 1 OS=Mus musculus OX=10090 GN=Pofut1 PE=1 SV=1	0.81
Rab32	Q9CZE3	Ras-related protein Rab-32 OS=Mus musculus OX=10090 GN=Rab32 PE=1 SV=3	0.886666667
Golga5	Q9QYE6	Golgin subfamily A member 5 OS=Mus musculus OX=10090 GN=Golga5 PE=1 SV=2	0.766666667
Tsg101	Q61187	Tumor susceptibility gene 101 protein OS=Mus musculus OX=10090 GN=Tsg101 PE=1 SV=2	0.81
Map3k20	Q9ESL4	Mitogen-activated protein kinase kinase kinase 20 OS=Mus musculus OX=10090 GN=Map3k20 PE=1	0.91
Washc1	Q8VDD8	WASH complex subunit 1 OS=Mus musculus OX=10090 GN=Washc1 PE=1 SV=1	1.06
Nmd3	Q99L48	60S ribosomal export protein NMD3 OS=Mus musculus OX=10090 GN=Nmd3 PE=1 SV=1	0.843333333
Pfdn5	Q9WU28	Prefoldin subunit 5 OS=Mus musculus OX=10090 GN=Pfdn5 PE=1 SV=1	0.91
Adrm1	Q9JKV1	Proteasomal ubiquitin receptor ADRM1 OS=Mus musculus OX=10090 GN=Adrm1 PE=1 SV=2	0.833333333
Slc7a1	Q09143	High affinity cationic amino acid transporter 1 OS=Mus musculus OX=10090 GN=Slc7a1 PE=1 SV=1	0.94
Rprd2	Q6NXI6	Regulation of nuclear pre-mRNA domain-containing protein 2 OS=Mus musculus OX=10090 GN=Rpı	1.053333333
Qki	Q9QYS9	Protein quaking OS=Mus musculus OX=10090 GN=Qki PE=1 SV=1	0.823333333
Mtx2	O88441	Metaxin-2 OS=Mus musculus OX=10090 GN=Mtx2 PE=1 SV=1	1.11

Smpd2	O70572	Sphingomyelin phosphodiesterase 2 OS=Mus musculus OX=10090 GN=Smpd2 PE=1 SV=1	0.943333333
Shcbp1	Q9Z179	SHC SH2 domain-binding protein 1 OS=Mus musculus OX=10090 GN=Shcbp1 PE=1 SV=1	0.716666667
Pcid2	Q8BFV2	PCI domain-containing protein 2 OS=Mus musculus OX=10090 GN=Pcid2 PE=1 SV=1	0.91
Fer	P70451	Tyrosine-protein kinase Fer OS=Mus musculus OX=10090 GN=Fer PE=1 SV=2	0.9
Babam1	Q3UI43	BRISC and BRCA1-A complex member 1 OS=Mus musculus OX=10090 GN=Babam1 PE=1 SV=1	0.8
Osgep	Q8BWU5	Probable tRNA N6-adenosine threonylcarbamoyltransferase OS=Mus musculus OX=10090 GN=Osge	0.81
Phldb2	Q8K1N2	Pleckstrin homology-like domain family B member 2 OS=Mus musculus OX=10090 GN=Phldb2 PE=1	0.82
Snap47	Q8R570	Synaptosomal-associated protein 47 OS=Mus musculus OX=10090 GN=Snap47 PE=1 SV=1	0.83
Lsg1	Q3UM18	Large subunit GTPase 1 homolog OS=Mus musculus OX=10090 GN=Lsg1 PE=1 SV=2	0.836666667
Unc45a	Q99KD5	Protein unc-45 homolog A OS=Mus musculus OX=10090 GN=Unc45a PE=1 SV=2	0.806666667
Dag1	Q62165	Dystroglycan OS=Mus musculus OX=10090 GN=Dag1 PE=1 SV=4	0.756666667
Ints2	Q80UK8	Integrator complex subunit 2 OS=Mus musculus OX=10090 GN=Ints2 PE=1 SV=2	0.973333333
Abhd14b	Q8VCR7	Protein ABHD14B OS=Mus musculus OX=10090 GN=Abhd14b PE=1 SV=1	0.79
Pdzd8	B9EJ80	PDZ domain-containing protein 8 OS=Mus musculus OX=10090 GN=Pdzd8 PE=1 SV=1	0.783333333
Spin1	Q61142	Spindlin-1 OS=Mus musculus OX=10090 GN=Spin1 PE=1 SV=2	0.78
Smarcb1	Q9Z0H3	SWI/SNF-related matrix-associated actin-dependent regulator of chromatin subfamily B member 1	0.89
Gmppa	Q922H4	Mannose-1-phosphate guanyltransferase alpha OS=Mus musculus OX=10090 GN=Gmppa PE=1 SV=1	0.916666667
Slc5a3	Q9JKZ2	Sodium/myo-inositol cotransporter OS=Mus musculus OX=10090 GN=Slc5a3 PE=1 SV=2	0.866666667
Nob1	Q8BW10	RNA-binding protein NOB1 OS=Mus musculus OX=10090 GN=Nob1 PE=1 SV=1	0.88
Smad4	P97471	Mothers against decapentaplegic homolog 4 OS=Mus musculus OX=10090 GN=Smad4 PE=1 SV=2	0.83
Exosc7	Q9D0M0	Exosome complex exonuclease RRP42 OS=Mus musculus OX=10090 GN=Exosc7 PE=1 SV=2	0.85
Nudcd3	Q8R1N4	NudC domain-containing protein 3 OS=Mus musculus OX=10090 GN=Nudcd3 PE=1 SV=3	0.853333333
Hm13	Q9D8V0	Minor histocompatibility antigen H13 OS=Mus musculus OX=10090 GN=Hm13 PE=1 SV=1	0.853333333
Pkd2	O35245	Polycystin-2 OS=Mus musculus OX=10090 GN=Pkd2 PE=1 SV=2	0.89
Mak16	Q8BGS0	Protein MAK16 homolog OS=Mus musculus OX=10090 GN=Mak16 PE=1 SV=1	0.85
Dbr1	Q923B1	Lariat debranching enzyme OS=Mus musculus OX=10090 GN=Dbr1 PE=1 SV=2	0.943333333
App	P12023	Amyloid-beta A4 protein OS=Mus musculus OX=10090 GN=App PE=1 SV=3	1.003333333
Nelfe	P19426	Negative elongation factor E OS=Mus musculus OX=10090 GN=Nelfe PE=1 SV=2	0.713333333
Qrich1	Q3UA37	Glutamine-rich protein 1 OS=Mus musculus OX=10090 GN=Qrich1 PE=1 SV=1	0.833333333
Ube2m	P61082	NEDD8-conjugating enzyme Ubc12 OS=Mus musculus OX=10090 GN=Ube2m PE=1 SV=1	0.916666667
IntS13	Q8QZV7	Integrator complex subunit 13 OS=Mus musculus OX=10090 GN=IntS13 PE=1 SV=2	0.716666667
Fbxo4	Q8CHQ0	F-box only protein 4 OS=Mus musculus OX=10090 GN=Fbxo4 PE=1 SV=2	0.926666667
Chaf1b	Q9D0N7	Chromatin assembly factor 1 subunit B OS=Mus musculus OX=10090 GN=Chaf1b PE=1 SV=1	0.876666667
Chtf18	Q8BIW9	Chromosome transmission fidelity protein 18 homolog OS=Mus musculus OX=10090 GN=Chtf18 PE	0.963333333

Vps28	Q9D1C8	Vacuolar protein sorting-associated protein 28 homolog OS=Mus musculus OX=10090 GN=Vps28 PI	0.963333333
Nmral1	Q8K2T1	NmrA-like family domain-containing protein 1 OS=Mus musculus OX=10090 GN=Nmral1 PE=1 SV=1	0.83
Cdh11	P55288	Cadherin-11 OS=Mus musculus OX=10090 GN=Cdh11 PE=1 SV=1	0.93
Rpl28	P41105	60S ribosomal protein L28 OS=Mus musculus OX=10090 GN=Rpl28 PE=1 SV=2	0.923333333
Stx7	O70439	Syntaxin-7 OS=Mus musculus OX=10090 GN=Stx7 PE=1 SV=3	0.776666667
Usp36	B1AQJ2	Ubiquitin carboxyl-terminal hydrolase 36 OS=Mus musculus OX=10090 GN=Usp36 PE=1 SV=1	1.093333333
Fntb	Q8K2I1	Protein farnesyltransferase subunit beta OS=Mus musculus OX=10090 GN=Fntb PE=1 SV=1	1.016666667
Ripor1	Q68FE6	Rho family-interacting cell polarization regulator 1 OS=Mus musculus OX=10090 GN=Ripor1 PE=1 S	1.176666667
Fdft1	P53798	Squalene synthase OS=Mus musculus OX=10090 GN=Fdft1 PE=1 SV=2	1.073333333
Hsd17b11	Q9EQ06	Estradiol 17-beta-dehydrogenase 11 OS=Mus musculus OX=10090 GN=Hsd17b11 PE=1 SV=1	0.96
Atad1	Q9D5T0	ATPase family AAA domain-containing protein 1 OS=Mus musculus OX=10090 GN=Atad1 PE=1 SV=1	0.986666667
Mavs	Q8VCF0	Mitochondrial antiviral-signaling protein OS=Mus musculus OX=10090 GN=Mavs PE=1 SV=1	0.893333333
Akap1	O08715	A-kinase anchor protein 1 mitochondrial OS=Mus musculus OX=10090 GN=Akap1 PE=1 SV=4	0.846666667
Ccnh	Q61458	Cyclin-H OS=Mus musculus OX=10090 GN=Ccnh PE=1 SV=2	0.813333333
Coasy	Q9DBL7	Bifunctional coenzyme A synthase OS=Mus musculus OX=10090 GN=Coasy PE=1 SV=2	0.88
Stt3b	Q3TDQ1	Dolichyl-diphosphooligosaccharide--protein glycosyltransferase subunit STT3B OS=Mus musculus C	1.3
Decr1	Q9CQ62	2 4-dienoyl-CoA reductase mitochondrial OS=Mus musculus OX=10090 GN=Decr1 PE=1 SV=1	0.846666667
Fnta	Q61239	Protein farnesyltransferase/geranylgeranyltransferase type-1 subunit alpha OS=Mus musculus OX=1(	0.883333333
Trmt2a	Q8BNV1	tRNA (uracil-5-)-methyltransferase homolog A OS=Mus musculus OX=10090 GN=Trmt2a PE=1 SV=1	0.83
Tbc1d5	Q80XQ2	TBC1 domain family member 5 OS=Mus musculus OX=10090 GN=Tbc1d5 PE=1 SV=2	0.813333333
Tacc3	Q9JJ11	Transforming acidic coiled-coil-containing protein 3 OS=Mus musculus OX=10090 GN=Tacc3 PE=1 S	0.783333333
Top3b	Q9Z321	DNA topoisomerase 3-beta-1 OS=Mus musculus OX=10090 GN=Top3b PE=1 SV=1	0.93
Dnaja3	Q99M87	DnaJ homolog subfamily A member 3 mitochondrial OS=Mus musculus OX=10090 GN=Dnaja3 PE=1	0.876666667
Rpl13a	P19253	60S ribosomal protein L13a OS=Mus musculus OX=10090 GN=Rpl13a PE=1 SV=4	0.926666667
Mrpl4	Q9DCU6	39S ribosomal protein L4 mitochondrial OS=Mus musculus OX=10090 GN=Mrpl4 PE=1 SV=1	0.923333333
Stx5	Q8K1E0	Syntaxin-5 OS=Mus musculus OX=10090 GN=Stx5 PE=1 SV=3	0.916666667
Ndufs3	Q9DCT2	NADH dehydrogenase [ubiquinone] iron-sulfur protein 3 mitochondrial OS=Mus musculus OX=100!	0.866666667
Gba	P17439	Lysosomal acid glucosylceramidase OS=Mus musculus OX=10090 GN=Gba PE=1 SV=1	0.693333333
LRWD1	Q8BUI3	Leucine-rich repeat and WD repeat-containing protein 1 OS=Mus musculus OX=10090 GN=LRWD1 I	0.983333333
Shc1	P98083	SHC-transforming protein 1 OS=Mus musculus OX=10090 GN=Shc1 PE=1 SV=3	0.82
Cdc42bpa	Q3UU96	Serine/threonine-protein kinase MRCK alpha OS=Mus musculus OX=10090 GN=Cdc42bpa PE=1 SV=2	1.08
Kdm3b	Q6ZPY7	Lysine-specific demethylase 3B OS=Mus musculus OX=10090 GN=Kdm3b PE=1 SV=2	1.066666667
Polr2e	Q80UW8	DNA-directed RNA polymerases I II and III subunit RPABC1 OS=Mus musculus OX=10090 GN=Polr2e	1.04
Exoc8	Q6PGF7	Exocyst complex component 8 OS=Mus musculus OX=10090 GN=Exoc8 PE=1 SV=1	0.796666667

Abhd12	Q99LR1	Lysophosphatidylserine lipase ABHD12 OS=Mus musculus OX=10090 GN=Abhd12 PE=1 SV=2	1.003333333
Mpp6	Q9JLB0	MAGUK p55 subfamily member 6 OS=Mus musculus OX=10090 GN=Mpp6 PE=1 SV=1	0.95
Vps45	P97390	Vacuolar protein sorting-associated protein 45 OS=Mus musculus OX=10090 GN=Vps45 PE=1 SV=1	0.83
Pcyox1l	Q8C7K6	Prenylcysteine oxidase-like OS=Mus musculus OX=10090 GN=Pcyox1l PE=1 SV=1	0.863333333
Snu13	Q9D0T1	NHP2-like protein 1 OS=Mus musculus OX=10090 GN=Snu13 PE=1 SV=4	0.906666667
Apip	Q9WVQ5	Methylthioribulose-1-phosphate dehydratase OS=Mus musculus OX=10090 GN=Apip PE=1 SV=1	0.81
Atp6v1e1	P50518	V-type proton ATPase subunit E 1 OS=Mus musculus OX=10090 GN=Atp6v1e1 PE=1 SV=2	0.816666667
Rnaseh2a	Q9CWY8	Ribonuclease H2 subunit A OS=Mus musculus OX=10090 GN=Rnaseh2a PE=1 SV=2	0.943333333
Tcf20	Q9EPQ8	Transcription factor 20 OS=Mus musculus OX=10090 GN=Tcf20 PE=1 SV=3	0.87
Mycbp2	Q7TPH6	E3 ubiquitin-protein ligase MYCBP2 OS=Mus musculus OX=10090 GN=Mycbp2 PE=1 SV=3	1.126666667
Pole	Q9WVF7	DNA polymerase epsilon catalytic subunit A OS=Mus musculus OX=10090 GN=Pole PE=1 SV=3	0.686666667
Pih1d1	Q9CQJ2	PIH1 domain-containing protein 1 OS=Mus musculus OX=10090 GN=Pih1d1 PE=1 SV=1	0.886666667
Cc2d1a	Q8K1A6	Coiled-coil and C2 domain-containing protein 1A OS=Mus musculus OX=10090 GN=Cc2d1a PE=1 SV	0.943333333
Dennd4c	A6H8H2	DENN domain-containing protein 4C OS=Mus musculus OX=10090 GN=Dennd4c PE=1 SV=1	0.876666667
Exoc5	Q3TPX4	Exocyst complex component 5 OS=Mus musculus OX=10090 GN=Exoc5 PE=1 SV=2	1.256666667
Krr1	Q8BGA5	KRR1 small subunit processome component homolog OS=Mus musculus OX=10090 GN=Krr1 PE=2 S	0.853333333
Slc9a3r1	P70441	Na(+)/H(+) exchange regulatory cofactor NHE-RF1 OS=Mus musculus OX=10090 GN=Slc9a3r1 PE=1 S	0.766666667
Rbbp5	Q8BX09	Retinoblastoma-binding protein 5 OS=Mus musculus OX=10090 GN=Rbbp5 PE=1 SV=2	0.92
Sacs	Q9JLC8	Sacsin OS=Mus musculus OX=10090 GN=Sacs PE=1 SV=2	1.43
Osbpl8	B9EJ86	Oxysterol-binding protein-related protein 8 OS=Mus musculus OX=10090 GN=Osbpl8 PE=1 SV=1	0.806666667
Prcp	Q7TMR0	Lysosomal Pro-X carboxypeptidase OS=Mus musculus OX=10090 GN=Prcp PE=1 SV=2	0.963333333
Tubgcp4	Q9D4F8	Gamma-tubulin complex component 4 OS=Mus musculus OX=10090 GN=Tubgcp4 PE=1 SV=2	0.796666667
Reps1	O54916	RalBP1-associated Eps domain-containing protein 1 OS=Mus musculus OX=10090 GN=Reps1 PE=1 S	0.94
Odr4	Q4PJX1	Protein odr-4 homolog OS=Mus musculus OX=10090 GN=Odr4 PE=1 SV=2	0.866666667
Rbm34	Q8C5L7	RNA-binding protein 34 OS=Mus musculus OX=10090 GN=Rbm34 PE=1 SV=2	0.896666667
Ppil4	Q9CXG3	Peptidyl-prolyl cis-trans isomerase-like 4 OS=Mus musculus OX=10090 GN=Ppil4 PE=1 SV=2	1.19
Fam50a	Q9WV03	Protein FAM50A OS=Mus musculus OX=10090 GN=Fam50a PE=1 SV=1	0.906666667
Agk	Q9ESW4	Acylglycerol kinase mitochondrial OS=Mus musculus OX=10090 GN=Agk PE=1 SV=1	0.9
Atp6v0d1	P51863	V-type proton ATPase subunit d 1 OS=Mus musculus OX=10090 GN=Atp6v0d1 PE=1 SV=2	0.923333333
Fnbp4	Q6ZQ03	Formin-binding protein 4 OS=Mus musculus OX=10090 GN=Fnbp4 PE=1 SV=2	0.746666667
Limd1	Q9QXD8	LIM domain-containing protein 1 OS=Mus musculus OX=10090 GN=Limd1 PE=1 SV=2	0.706666667
Morc3	F7BJB9	MORC family CW-type zinc finger protein 3 OS=Mus musculus OX=10090 GN=Morc3 PE=1 SV=1	0.706666667
Grk2	Q99MK8	Beta-adrenergic receptor kinase 1 OS=Mus musculus OX=10090 GN=Grk2 PE=1 SV=2	0.886666667
Erp29	P57759	Endoplasmic reticulum resident protein 29 OS=Mus musculus OX=10090 GN=Erp29 PE=1 SV=2	0.916666667

Gclc	P97494	Glutamate--cysteine ligase catalytic subunit OS=Mus musculus OX=10090 GN=Gclc PE=1 SV=4	0.776666667
Cwc22	Q8C5N3	Pre-mRNA-splicing factor CWC22 homolog OS=Mus musculus OX=10090 GN=Cwc22 PE=1 SV=1	0.893333333
Dlgap5	Q8K4R9	Disks large-associated protein 5 OS=Mus musculus OX=10090 GN=Dlgap5 PE=1 SV=2	1.036666667
Mrps31	Q61733	28S ribosomal protein S31 mitochondrial OS=Mus musculus OX=10090 GN=Mrps31 PE=1 SV=1	0.906666667
Irgm1	Q60766	Immunity-related GTPase family M protein 1 OS=Mus musculus OX=10090 GN=Irgm1 PE=1 SV=1	0.836666667
Exosc8	Q9D753	Exosome complex component RRP43 OS=Mus musculus OX=10090 GN=Exosc8 PE=1 SV=1	0.873333333
Dbt	P53395	Lipoamide acyltransferase component of branched-chain alpha-keto acid dehydrogenase complex	0.916666667
Tbc1d17	Q8BYH7	TBC1 domain family member 17 OS=Mus musculus OX=10090 GN=Tbc1d17 PE=1 SV=2	0.96
Dpm1	O70152	Dolichol-phosphate mannosyltransferase subunit 1 OS=Mus musculus OX=10090 GN=Dpm1 PE=1 S	0.83
Trmt6	Q8CE96	tRNA (adenine(58)-N(1))-methyltransferase non-catalytic subunit TRM6 OS=Mus musculus OX=1009	1.35
Vps25	Q9CQ80	Vacuolar protein-sorting-associated protein 25 OS=Mus musculus OX=10090 GN=Vps25 PE=1 SV=1	0.99
Dhrs1	Q99L04	Dehydrogenase/reductase SDR family member 1 OS=Mus musculus OX=10090 GN=Dhrs1 PE=1 SV=1	0.923333333
Txnl4a	P83877	Thioredoxin-like protein 4A OS=Mus musculus OX=10090 GN=Txnl4a PE=1 SV=1	0.813333333
Sdad1	Q80UZ2	Protein SDA1 homolog OS=Mus musculus OX=10090 GN=Sdad1 PE=1 SV=1	0.81
Fam207a	P58468	Protein FAM207A OS=Mus musculus OX=10090 GN=Fam207a PE=1 SV=1	0.83
Slc25a17	O70579	Peroxisomal membrane protein PMP34 OS=Mus musculus OX=10090 GN=Slc25a17 PE=1 SV=1	0.936666667
Ctu2	Q3U308	Cytoplasmic tRNA 2-thiolation protein 2 OS=Mus musculus OX=10090 GN=Ctu2 PE=1 SV=1	1.11
Lclat1	Q3UN02	Lysocardiolipin acyltransferase 1 OS=Mus musculus OX=10090 GN=Lclat1 PE=1 SV=2	0.786666667
Mrps15	Q9DC71	28S ribosomal protein S15 mitochondrial OS=Mus musculus OX=10090 GN=Mrps15 PE=1 SV=2	0.88
Vrk2	Q8BN21	Serine/threonine-protein kinase VRK2 OS=Mus musculus OX=10090 GN=Vrk2 PE=1 SV=2	0.783333333
Eno2	P17183	Gamma-enolase OS=Mus musculus OX=10090 GN=Eno2 PE=1 SV=2	
Eif5a2	Q8BGY2	Eukaryotic translation initiation factor 5A-2 OS=Mus musculus OX=10090 GN=Eif5a2 PE=1 SV=3	
Ctnna2	Q61301	Catenin alpha-2 OS=Mus musculus OX=10090 GN=Ctnna2 PE=1 SV=3	0.875
H1-3	P43277	Histone H1.3 OS=Mus musculus OX=10090 GN=H1-3 PE=1 SV=2	0.753333333
Prkcb	P68404	Protein kinase C beta type OS=Mus musculus OX=10090 GN=Prkcb PE=1 SV=4	0.836666667
Hras	Q61411	GTPase HRas OS=Mus musculus OX=10090 GN=Hras PE=1 SV=2	
Crebbp	P45481	Histone lysine acetyltransferase CREBBP OS=Mus musculus OX=10090 GN=Crebbp PE=1 SV=3	1.315
Arl8b	Q9CQW2	ADP-ribosylation factor-like protein 8B OS=Mus musculus OX=10090 GN=Arl8b PE=1 SV=1	1.043333333
Rab2b	P59279	Ras-related protein Rab-2B OS=Mus musculus OX=10090 GN=Rab2b PE=1 SV=1	1.246666667
Rab8a	P55258	Ras-related protein Rab-8A OS=Mus musculus OX=10090 GN=Rab8a PE=1 SV=2	0.846666667
Eif1	P48024	Eukaryotic translation initiation factor 1 OS=Mus musculus OX=10090 GN=Eif1 PE=1 SV=2	0.86
Pcbp3	P57722	Poly(rC)-binding protein 3 OS=Mus musculus OX=10090 GN=Pcbp3 PE=1 SV=3	0.566666667
Prkacb	P68181	cAMP-dependent protein kinase catalytic subunit beta OS=Mus musculus OX=10090 GN=Prkacb PE	0.706666667
H1-4	P43274	Histone H1.4 OS=Mus musculus OX=10090 GN=H1-4 PE=1 SV=2	0.793333333

Rad23a	P54726	UV excision repair protein RAD23 homolog A OS=Mus musculus OX=10090 GN=Rad23a PE=1 SV=2	0.866666667
Rab5a	Q9CQD1	Ras-related protein Rab-5A OS=Mus musculus OX=10090 GN=Rab5a PE=1 SV=1	0.856666667
H2az1	P0C0S6	Histone H2A.Z OS=Mus musculus OX=10090 GN=H2az1 PE=1 SV=2	0.86
H2afv	Q3THW5	Histone H2A.V OS=Mus musculus OX=10090 GN=H2az2 PE=1 SV=3	0.86
Tial1	P70318	Nucleolysin TIAR OS=Mus musculus OX=10090 GN=Tial1 PE=1 SV=1	0.88
Sec61a1	P61620	Protein transport protein Sec61 subunit alpha isoform 1 OS=Mus musculus OX=10090 GN=Sec61a1	0.83
Mcts1	Q9DB27	Malignant T-cell-amplified sequence 1 OS=Mus musculus OX=10090 GN=Mcts1 PE=1 SV=1	0.883333333
Kif2c	Q922S8	Kinesin-like protein KIF2C OS=Mus musculus OX=10090 GN=Kif2c PE=1 SV=1	0.83
Camk1d	Q8BW96	Calcium/calmodulin-dependent protein kinase type 1D OS=Mus musculus OX=10090 GN=Camk1d P	0.543333333
Pip4k2b	Q80XI4	Phosphatidylinositol 5-phosphate 4-kinase type-2 beta OS=Mus musculus OX=10090 GN=Pip4k2b P	0.883333333
Snx12	O70493	Sorting nexin-12 OS=Mus musculus OX=10090 GN=Snx12 PE=1 SV=1	0.873333333
Map4k4	P97820	Mitogen-activated protein kinase kinase kinase kinase 4 OS=Mus musculus OX=10090 GN=Map4k4 P	0.976666667
Eef1b	O70251	Elongation factor 1-beta OS=Mus musculus OX=10090 GN=Eef1b PE=1 SV=5	0.89
Capns1	O88456	Calpain small subunit 1 OS=Mus musculus OX=10090 GN=Capns1 PE=1 SV=1	0.883333333
Cdk2	P97377	Cyclin-dependent kinase 2 OS=Mus musculus OX=10090 GN=Cdk2 PE=1 SV=2	0.876666667
Rnf2	Q9CQJ4	E3 ubiquitin-protein ligase RING2 OS=Mus musculus OX=10090 GN=Rnf2 PE=1 SV=1	0.893333333
Ranbp10	Q6VN19	Ran-binding protein 10 OS=Mus musculus OX=10090 GN=Ranbp10 PE=1 SV=2	0.8
Rnf40	Q3U319	E3 ubiquitin-protein ligase BRE1B OS=Mus musculus OX=10090 GN=Rnf40 PE=1 SV=2	0.81
Vps4a	Q8VEJ9	Vacuolar protein sorting-associated protein 4A OS=Mus musculus OX=10090 GN=Vps4a PE=1 SV=1	0.956666667
Ptgs1	P22437	Prostaglandin G/H synthase 1 OS=Mus musculus OX=10090 GN=Ptgs1 PE=1 SV=1	1.223333333
Stk39	Q9Z1W9	STE20/SPS1-related proline-alanine-rich protein kinase OS=Mus musculus OX=10090 GN=Stk39 PE=	0.893333333
Elmo1	Q8BPU7	Engulfment and cell motility protein 1 OS=Mus musculus OX=10090 GN=Elmo1 PE=1 SV=2	0.743333333
Hmgb3	O54879	High mobility group protein B3 OS=Mus musculus OX=10090 GN=Hmgb3 PE=1 SV=3	0.85
Ube2l3	P68037	Ubiquitin-conjugating enzyme E2 L3 OS=Mus musculus OX=10090 GN=Ube2l3 PE=1 SV=1	0.836666667
Fen1	P39749	Flap endonuclease 1 OS=Mus musculus OX=10090 GN=Fen1 PE=1 SV=1	0.87
Ppm1f	Q8CGA0	Protein phosphatase 1F OS=Mus musculus OX=10090 GN=Ppm1f PE=1 SV=1	0.93
Tra2b	P62996	Transformer-2 protein homolog beta OS=Mus musculus OX=10090 GN=Tra2b PE=1 SV=1	0.836666667
Ppp2r5c	Q60996	Serine/threonine-protein phosphatase 2A 56 kDa regulatory subunit gamma isoform OS=Mus muscu	0.86
Cdk6	Q64261	Cyclin-dependent kinase 6 OS=Mus musculus OX=10090 GN=Cdk6 PE=1 SV=2	0.696666667
Minpp1	Q9Z2L6	Multiple inositol polyphosphate phosphatase 1 OS=Mus musculus OX=10090 GN=Minpp1 PE=1 SV=	0.956666667
Rhog	P84096	Rho-related GTP-binding protein RhoG OS=Mus musculus OX=10090 GN=Rhog PE=1 SV=1	0.883333333
Epm2aip1	Q8VEH5	EPM2A-interacting protein 1 OS=Mus musculus OX=10090 GN=Epm2aip1 PE=1 SV=1	0.886666667
Twf2	Q9Z0P5	Twinfilin-2 OS=Mus musculus OX=10090 GN=Twf2 PE=1 SV=1	0.93
Cmpk1	Q9DBP5	UMP-CMP kinase OS=Mus musculus OX=10090 GN=Cmpk1 PE=1 SV=1	0.973333333

Cdk5	P49615	Cyclin-dependent-like kinase 5 OS=Mus musculus OX=10090 GN=Cdk5 PE=1 SV=1	0.94
Fads1	Q920L1	Acyl-CoA (8-3)-desaturase OS=Mus musculus OX=10090 GN=Fads1 PE=1 SV=1	0.913333333
Gga2	Q6P5E6	ADP-ribosylation factor-binding protein GGA2 OS=Mus musculus OX=10090 GN=Gga2 PE=1 SV=1	1.043333333
Pgrmc1	O55022	Membrane-associated progesterone receptor component 1 OS=Mus musculus OX=10090 GN=Pgrm	0.87
Cpne2	P59108	Copine-2 OS=Mus musculus OX=10090 GN=Cpne2 PE=1 SV=1	0.866666667
Rab34	Q64008	Ras-related protein Rab-34 OS=Mus musculus OX=10090 GN=Rab34 PE=1 SV=2	0.91
Mtmr2	Q9Z2D1	Myotubularin-related protein 2 OS=Mus musculus OX=10090 GN=Mtmr2 PE=1 SV=3	0.76
Gga1	Q8R0H9	ADP-ribosylation factor-binding protein GGA1 OS=Mus musculus OX=10090 GN=Gga1 PE=1 SV=1	0.873333333
Gsk3a	Q2NL51	Glycogen synthase kinase-3 alpha OS=Mus musculus OX=10090 GN=Gsk3a PE=1 SV=2	0.863333333
Spata5	Q3UMC0	ATPase family protein 2 homolog OS=Mus musculus OX=10090 GN=Spata5 PE=1 SV=2	0.84
Htra2	Q9JIY5	Serine protease HTRA2 mitochondrial OS=Mus musculus OX=10090 GN=Htra2 PE=1 SV=2	0.91
Stxbp3	Q60770	Syntaxin-binding protein 3 OS=Mus musculus OX=10090 GN=Stxbp3 PE=1 SV=1	0.926666667
Aurkb	O70126	Aurora kinase B OS=Mus musculus OX=10090 GN=Aurkb PE=1 SV=2	0.826666667
Csrp1	P97315	Cysteine and glycine-rich protein 1 OS=Mus musculus OX=10090 GN=Csrp1 PE=1 SV=3	0.96
Nutf2	P61971	Nuclear transport factor 2 OS=Mus musculus OX=10090 GN=Nutf2 PE=1 SV=1	0.86
Mtpn	P62774	Myotrophin OS=Mus musculus OX=10090 GN=Mtpn PE=1 SV=2	0.893333333
Hmox1	P14901	Heme oxygenase 1 OS=Mus musculus OX=10090 GN=Hmox1 PE=1 SV=1	0.933333333
Alyref	O08583	THO complex subunit 4 OS=Mus musculus OX=10090 GN=Alyref PE=1 SV=3	0.89
Eif3k	Q9DBZ5	Eukaryotic translation initiation factor 3 subunit K OS=Mus musculus OX=10090 GN=Eif3k PE=1 SV=	0.86
Mtch2	Q791V5	Mitochondrial carrier homolog 2 OS=Mus musculus OX=10090 GN=Mtch2 PE=1 SV=1	0.963333333
Ctsl	P06797	Cathepsin L1 OS=Mus musculus OX=10090 GN=Ctsl PE=1 SV=2	0.986666667
Ehmt2	Q9Z148	Histone-lysine N-methyltransferase EHMT2 OS=Mus musculus OX=10090 GN=Ehmt2 PE=1 SV=2	0.766666667
Aimp2	Q8R010	Aminoacyl tRNA synthase complex-interacting multifunctional protein 2 OS=Mus musculus OX=10(	0.893333333
Rbm8a	Q9CWZ3	RNA-binding protein 8A OS=Mus musculus OX=10090 GN=Rbm8a PE=1 SV=4	0.91
Pafah1b3	Q61205	Platelet-activating factor acetylhydrolase IB subunit gamma OS=Mus musculus OX=10090 GN=Pafal	0.883333333
Eif4h	Q9WUK2	Eukaryotic translation initiation factor 4H OS=Mus musculus OX=10090 GN=Eif4h PE=1 SV=3	0.953333333
Cdc37	Q61081	Hsp90 co-chaperone Cdc37 OS=Mus musculus OX=10090 GN=Cdc37 PE=1 SV=1	0.89
Prrc1	Q3UPH1	Protein PRRC1 OS=Mus musculus OX=10090 GN=Prrc1 PE=1 SV=1	0.756666667
Capg	P24452	Macrophage-capping protein OS=Mus musculus OX=10090 GN=Capg PE=1 SV=2	0.853333333
Park7	Q99LX0	Parkinson disease protein 7 homolog OS=Mus musculus OX=10090 GN=Park7 PE=1 SV=1	0.873333333
Nup62	Q63850	Nuclear pore glycoprotein p62 OS=Mus musculus OX=10090 GN=Nup62 PE=1 SV=2	0.836666667
Mpi	Q924M7	Mannose-6-phosphate isomerase OS=Mus musculus OX=10090 GN=Mpi PE=1 SV=1	0.833333333
Efemp1	Q8BPB5	EGF-containing fibulin-like extracellular matrix protein 1 OS=Mus musculus OX=10090 GN=Efemp1	0.846666667
Notch2	O35516	Neurogenic locus notch homolog protein 2 OS=Mus musculus OX=10090 GN=Notch2 PE=1 SV=2	0.76

Ftl1	P29391	Ferritin light chain 1 OS=Mus musculus OX=10090 GN=Ftl1 PE=1 SV=2	0.903333333
Clptm1	Q8VBZ3	Cleft lip and palate transmembrane protein 1 homolog OS=Mus musculus OX=10090 GN=Clptm1 PE	0.786666667
Emg1	O35130	Ribosomal RNA small subunit methyltransferase NEP1 OS=Mus musculus OX=10090 GN=Emg1 PE=1	0.826666667
U2af1	Q9D883	Splicing factor U2AF 35 kDa subunit OS=Mus musculus OX=10090 GN=U2af1 PE=1 SV=4	0.9
Qdpr	Q8BVI4	Dihydropteridine reductase OS=Mus musculus OX=10090 GN=Qdpr PE=1 SV=2	0.88
Sod2	P09671	Superoxide dismutase [Mn] mitochondrial OS=Mus musculus OX=10090 GN=Sod2 PE=1 SV=3	0.903333333
Sec22b	O08547	Vesicle-trafficking protein SEC22b OS=Mus musculus OX=10090 GN=Sec22b PE=1 SV=3	0.88
Dnttip2	Q8R2M2	Deoxynucleotidyltransferase terminal-interacting protein 2 OS=Mus musculus OX=10090 GN=Dntti	0.876666667
Slc25a10	Q9QZD8	Mitochondrial dicarboxylate carrier OS=Mus musculus OX=10090 GN=Slc25a10 PE=1 SV=2	0.813333333
Cpsf6	Q6NVF9	Cleavage and polyadenylation specificity factor subunit 6 OS=Mus musculus OX=10090 GN=Cpsf6 PI	0.81
P3h1	Q3V1T4	Prolyl 3-hydroxylase 1 OS=Mus musculus OX=10090 GN=P3h1 PE=1 SV=2	0.826666667
Dnajb11	Q99KV1	DnaJ homolog subfamily B member 11 OS=Mus musculus OX=10090 GN=Dnajb11 PE=1 SV=1	0.903333333
Emc2	Q9CRD2	ER membrane protein complex subunit 2 OS=Mus musculus OX=10090 GN=Emc2 PE=1 SV=1	0.94
Anp32e	P97822	Acidic leucine-rich nuclear phosphoprotein 32 family member E OS=Mus musculus OX=10090 GN=/	0.943333333
Dcps	Q9DAR7	m7GpppX diphosphatase OS=Mus musculus OX=10090 GN=Dcps PE=1 SV=1	0.84
Cops7a	Q9CZ04	COP9 signalosome complex subunit 7a OS=Mus musculus OX=10090 GN=Cops7a PE=1 SV=2	0.88
Ctsz	Q9WUU7	Cathepsin Z OS=Mus musculus OX=10090 GN=Ctsz PE=1 SV=1	0.806666667
Sh3bp1	P55194	SH3 domain-binding protein 1 OS=Mus musculus OX=10090 GN=Sh3bp1 PE=1 SV=3	0.966666667
Csrp2	P97314	Cysteine and glycine-rich protein 2 OS=Mus musculus OX=10090 GN=Csrp2 PE=1 SV=3	0.91
Ubxn4	Q8VCH8	UBX domain-containing protein 4 OS=Mus musculus OX=10090 GN=Ubxn4 PE=1 SV=1	0.876666667
Tbcc	Q8VCN9	Tubulin-specific chaperone C OS=Mus musculus OX=10090 GN=Tbcc PE=1 SV=1	1.14
Pcca	Q91ZA3	Propionyl-CoA carboxylase alpha chain mitochondrial OS=Mus musculus OX=10090 GN=Pcca PE=1	0.836666667
Pdcl3	Q8BVF2	Phosducin-like protein 3 OS=Mus musculus OX=10090 GN=Pdcl3 PE=1 SV=1	0.84
Mrps30	Q9D0G0	28S ribosomal protein S30 mitochondrial OS=Mus musculus OX=10090 GN=Mrps30 PE=1 SV=1	0.65
Ndufaf7	Q9CWG8	Protein arginine methyltransferase NDUFAF7 mitochondrial OS=Mus musculus OX=10090 GN=Ndu	1.01
Cstb	Q62426	Cystatin-B OS=Mus musculus OX=10090 GN=Cstb PE=1 SV=1	0.86
Tbca	P48428	Tubulin-specific chaperone A OS=Mus musculus OX=10090 GN=Tbca PE=1 SV=3	0.926666667
Ripk1	Q60855	Receptor-interacting serine/threonine-protein kinase 1 OS=Mus musculus OX=10090 GN=Ripk1 PE=	1.096666667
Suclg1	Q9WUM5	Succinate--CoA ligase [ADP/GDP-forming] subunit alpha mitochondrial OS=Mus musculus OX=1009	0.866666667
Topbp1	Q6ZQF0	DNA topoisomerase 2-binding protein 1 OS=Mus musculus OX=10090 GN=Topbp1 PE=1 SV=2	1.046666667
Glipr2	Q9CYL5	Golgi-associated plant pathogenesis-related protein 1 OS=Mus musculus OX=10090 GN=Glipr2 PE=1	0.89
Septin5	Q9Z2Q6	Septin-5 OS=Mus musculus OX=10090 GN=Septin5 PE=1 SV=2	0.89
Plpbp	Q9Z2Y8	Pyridoxal phosphate homeostasis protein OS=Mus musculus OX=10090 GN=Plpbp PE=1 SV=1	0.9
Thumpd1	Q99J36	THUMP domain-containing protein 1 OS=Mus musculus OX=10090 GN=Thumpd1 PE=1 SV=1	0.74

Pabpn1	Q8CCS6	Polyadenylate-binding protein 2 OS=Mus musculus OX=10090 GN=Pabpn1 PE=1 SV=3	0.896666667
Hibch	Q8QZS1	3-hydroxyisobutyryl-CoA hydrolase mitochondrial OS=Mus musculus OX=10090 GN=Hibch PE=1 SV	0.91
Preb	Q9WUQ2	Prolactin regulatory element-binding protein OS=Mus musculus OX=10090 GN=Preb PE=1 SV=1	0.82
Thoc6	Q5U4D9	THO complex subunit 6 homolog OS=Mus musculus OX=10090 GN=Thoc6 PE=1 SV=1	0.753333333
Rragc	Q99K70	Ras-related GTP-binding protein C OS=Mus musculus OX=10090 GN=Rragc PE=1 SV=1	0.863333333
Ccdc102a	Q3TMW1	Coiled-coil domain-containing protein 102A OS=Mus musculus OX=10090 GN=Ccdc102a PE=1 SV=2	0.883333333
Phf6	Q9D4J7	PHD finger protein 6 OS=Mus musculus OX=10090 GN=Phf6 PE=1 SV=1	0.856666667
Elp4	Q9ER73	Elongator complex protein 4 OS=Mus musculus OX=10090 GN=Elp4 PE=1 SV=2	0.873333333
Dnajc9	Q91WN1	DnaJ homolog subfamily C member 9 OS=Mus musculus OX=10090 GN=Dnajc9 PE=1 SV=2	0.796666667
Txlna	Q6PAM1	Alpha-taxilin OS=Mus musculus OX=10090 GN=Txlna PE=1 SV=1	0.906666667
Nufip2	Q5F2E7	Nuclear fragile X mental retardation-interacting protein 2 OS=Mus musculus OX=10090 GN=Nufip2	0.8
Yars2	Q8BYL4	Tyrosine--tRNA ligase mitochondrial OS=Mus musculus OX=10090 GN=Yars2 PE=1 SV=2	0.903333333
Lgals3	P16110	Galectin-3 OS=Mus musculus OX=10090 GN=Lgals3 PE=1 SV=3	0.763333333
Cryz	P47199	Quinone oxidoreductase OS=Mus musculus OX=10090 GN=Cryz PE=1 SV=1	0.88
Mrps7	Q80X85	28S ribosomal protein S7 mitochondrial OS=Mus musculus OX=10090 GN=Mrps7 PE=1 SV=1	0.953333333
Cnot2	Q8C5L3	CCR4-NOT transcription complex subunit 2 OS=Mus musculus OX=10090 GN=Cnot2 PE=1 SV=2	0.833333333
Nit2	Q9JHW2	Omega-amidase NIT2 OS=Mus musculus OX=10090 GN=Nit2 PE=1 SV=1	0.81
Pdcd4	Q61823	Programmed cell death protein 4 OS=Mus musculus OX=10090 GN=Pdcd4 PE=1 SV=1	0.756666667
Os9	Q8K2C7	Protein OS-9 OS=Mus musculus OX=10090 GN=Os9 PE=1 SV=2	0.81
Ddx54	Q8K4L0	ATP-dependent RNA helicase DDX54 OS=Mus musculus OX=10090 GN=Ddx54 PE=1 SV=1	0.9
Prpf31	Q8CCF0	U4/U6 small nuclear ribonucleoprotein Prp31 OS=Mus musculus OX=10090 GN=Prpf31 PE=1 SV=3	0.866666667
Snx17	Q8BVL3	Sorting nexin-17 OS=Mus musculus OX=10090 GN=Snx17 PE=1 SV=2	1.023333333
Nop53	Q8BK35	Ribosome biogenesis protein NOP53 OS=Mus musculus OX=10090 GN=Nop53 PE=1 SV=1	0.82
Ostf1	Q62422	Osteoclast-stimulating factor 1 OS=Mus musculus OX=10090 GN=Ostf1 PE=1 SV=2	0.866666667
Snrpd2	P62317	Small nuclear ribonucleoprotein Sm D2 OS=Mus musculus OX=10090 GN=Snrpd2 PE=1 SV=1	0.853333333
Fyco1	Q8VDC1	FYVE and coiled-coil domain-containing protein 1 OS=Mus musculus OX=10090 GN=Fyco1 PE=1 SV=	1.086666667
Pnkp	Q9JLV6	Bifunctional polynucleotide phosphatase/kinase OS=Mus musculus OX=10090 GN=Pnkp PE=1 SV=2	0.966666667
Maged1	Q9QYH6	Melanoma-associated antigen D1 OS=Mus musculus OX=10090 GN=Maged1 PE=1 SV=1	0.85
Nfu1	Q9QZ23	NFU1 iron-sulfur cluster scaffold homolog mitochondrial OS=Mus musculus OX=10090 GN=Nfu1 PI	0.746666667
Hpcal1	P62748	Hippocalcin-like protein 1 OS=Mus musculus OX=10090 GN=Hpcal1 PE=1 SV=2	0.89
Tcf25	Q8R3L2	Transcription factor 25 OS=Mus musculus OX=10090 GN=Tcf25 PE=1 SV=2	0.82
Nop9	Q8BMC4	Nucleolar protein 9 OS=Mus musculus OX=10090 GN=Nop9 PE=1 SV=1	1.083333333
Mphosph10	Q810V0	U3 small nucleolar ribonucleoprotein protein MPP10 OS=Mus musculus OX=10090 GN=Mphosph1	0.826666667
Hibadh	Q99L13	3-hydroxyisobutyrate dehydrogenase mitochondrial OS=Mus musculus OX=10090 GN=Hibadh PE=	0.843333333

Elob	P62869	Elongin-B OS=Mus musculus OX=10090 GN=Elob PE=1 SV=1	0.886666667
Gclm	O09172	Glutamate--cysteine ligase regulatory subunit OS=Mus musculus OX=10090 GN=Gclm PE=1 SV=1	0.886666667
Acot7	Q91V12	Cytosolic acyl coenzyme A thioester hydrolase OS=Mus musculus OX=10090 GN=Acot7 PE=1 SV=2	0.886666667
Wdr82	Q8BFQ4	WD repeat-containing protein 82 OS=Mus musculus OX=10090 GN=Wdr82 PE=1 SV=1	0.89
Skp1	Q9WTX5	S-phase kinase-associated protein 1 OS=Mus musculus OX=10090 GN=Skp1 PE=1 SV=3	0.86
Rab23	P35288	Ras-related protein Rab-23 OS=Mus musculus OX=10090 GN=Rab23 PE=1 SV=2	0.936666667
Mesd	Q9ERE7	LRP chaperone MESD OS=Mus musculus OX=10090 GN=Mesd PE=1 SV=1	0.92
Cdc42ep1	Q91W92	Cdc42 effector protein 1 OS=Mus musculus OX=10090 GN=Cdc42ep1 PE=1 SV=1	0.743333333
Exosc3	Q7TQK4	Exosome complex component RRP40 OS=Mus musculus OX=10090 GN=Exosc3 PE=1 SV=3	1.073333333
Cog2	Q921L5	Conserved oligomeric Golgi complex subunit 2 OS=Mus musculus OX=10090 GN=Cog2 PE=1 SV=2	0.693333333
Bag2	Q91YN9	BAG family molecular chaperone regulator 2 OS=Mus musculus OX=10090 GN=Bag2 PE=1 SV=1	0.903333333
Bsg	P18572	Basigin OS=Mus musculus OX=10090 GN=Bsg PE=1 SV=2	0.826666667
Dimt1	Q9D0D4	Probable dimethyladenosine transferase OS=Mus musculus OX=10090 GN=Dimt1 PE=2 SV=1	0.813333333
Rpap1	Q80TE0	RNA polymerase II-associated protein 1 OS=Mus musculus OX=10090 GN=Rpap1 PE=1 SV=2	0.833333333
Ints6	Q6PCM2	Integrator complex subunit 6 OS=Mus musculus OX=10090 GN=Ints6 PE=1 SV=1	0.92
Hspb1	P14602	Heat shock protein beta-1 OS=Mus musculus OX=10090 GN=Hspb1 PE=1 SV=3	0.78
Brox	Q8K2Q7	BRO1 domain-containing protein BROX OS=Mus musculus OX=10090 GN=Brox PE=1 SV=1	0.996666667
Hp1bp3	Q3TEA8	Heterochromatin protein 1-binding protein 3 OS=Mus musculus OX=10090 GN=Hp1bp3 PE=1 SV=1	0.86
Jak1	P52332	Tyrosine-protein kinase JAK1 OS=Mus musculus OX=10090 GN=Jak1 PE=1 SV=1	0.8
Soat1	Q61263	Sterol O-acyltransferase 1 OS=Mus musculus OX=10090 GN=Soat1 PE=1 SV=2	0.986666667
Eif4e	P63073	Eukaryotic translation initiation factor 4E OS=Mus musculus OX=10090 GN=Eif4e PE=1 SV=1	0.873333333
Slc38a4	Q8R1S9	Sodium-coupled neutral amino acid transporter 4 OS=Mus musculus OX=10090 GN=Slc38a4 PE=1 S	0.916666667
Arpc4	P59999	Actin-related protein 2/3 complex subunit 4 OS=Mus musculus OX=10090 GN=Arpc4 PE=1 SV=3	0.913333333
Phax	Q9JJT9	Phosphorylated adapter RNA export protein OS=Mus musculus OX=10090 GN=Phax PE=1 SV=1	1.096666667
Mkln1	O89050	Muskelin OS=Mus musculus OX=10090 GN=Mkln1 PE=1 SV=1	0.88
Timp3	P39876	Metalloproteinase inhibitor 3 OS=Mus musculus OX=10090 GN=Timp3 PE=1 SV=1	0.94
Ndrg3	Q9QYF9	Protein NDRG3 OS=Mus musculus OX=10090 GN=Ndrg3 PE=1 SV=1	0.913333333
Etfdh	Q921G7	Electron transfer flavoprotein-ubiquinone oxidoreductase mitochondrial OS=Mus musculus OX=1C	0.826666667
Rps19	Q9CZX8	40S ribosomal protein S19 OS=Mus musculus OX=10090 GN=Rps19 PE=1 SV=3	0.956666667
Scp2	P32020	Non-specific lipid-transfer protein OS=Mus musculus OX=10090 GN=Scp2 PE=1 SV=3	1.046666667
Cdc123	Q8CII2	Cell division cycle protein 123 homolog OS=Mus musculus OX=10090 GN=Cdc123 PE=1 SV=2	0.893333333
Eef1e1	Q9D1M4	Eukaryotic translation elongation factor 1 epsilon-1 OS=Mus musculus OX=10090 GN=Eef1e1 PE=1 S	0.973333333
Zc3h4	Q6ZPZ3	Zinc finger CCCH domain-containing protein 4 OS=Mus musculus OX=10090 GN=Zc3h4 PE=1 SV=2	0.8
Rraga	Q80X95	Ras-related GTP-binding protein A OS=Mus musculus OX=10090 GN=Rraga PE=1 SV=1	0.856666667

	Q6PIU9	Uncharacterized protein FLJ45252 homolog OS=Mus musculus OX=10090 PE=1 SV=2	0.86
Gtf2f2	Q8R0A0	General transcription factor IIF subunit 2 OS=Mus musculus OX=10090 GN=Gtf2f2 PE=1 SV=1	0.866666667
Ncdn	Q9Z0E0	Neurochondrin OS=Mus musculus OX=10090 GN=Ncdn PE=1 SV=1	1.24
Add3	Q9QYB5	Gamma-adducin OS=Mus musculus OX=10090 GN=Add3 PE=1 SV=2	0.8
Cpsf7	Q8BTV2	Cleavage and polyadenylation specificity factor subunit 7 OS=Mus musculus OX=10090 GN=Cpsf7 P	1.02
Mrps34	Q9JIK9	28S ribosomal protein S34 mitochondrial OS=Mus musculus OX=10090 GN=Mrps34 PE=1 SV=1	0.886666667
Sri	Q6P069	Sorcin OS=Mus musculus OX=10090 GN=Sri PE=1 SV=1	0.896666667
Dnajc8	Q6NZB0	DnaJ homolog subfamily C member 8 OS=Mus musculus OX=10090 GN=Dnajc8 PE=1 SV=2	0.936666667
Ezh2	Q61188	Histone-lysine N-methyltransferase EZH2 OS=Mus musculus OX=10090 GN=Ezh2 PE=1 SV=2	0.996666667
Dennd10	Q9D8N2	DENN domain-containing protein 10 OS=Mus musculus OX=10090 GN=Dennd10 PE=1 SV=2	0.873333333
Abcd3	P55096	ATP-binding cassette sub-family D member 3 OS=Mus musculus OX=10090 GN=Abcd3 PE=1 SV=2	0.843333333
Stat1	P42225	Signal transducer and activator of transcription 1 OS=Mus musculus OX=10090 GN=Stat1 PE=1 SV=1	0.82
Dctn4	Q8CBY8	Dynactin subunit 4 OS=Mus musculus OX=10090 GN=Dctn4 PE=1 SV=1	0.806666667
Plekho2	Q8K124	Pleckstrin homology domain-containing family O member 2 OS=Mus musculus OX=10090 GN=Plek	0.906666667
Qtrt2	B8ZXI1	Queuine tRNA-ribosyltransferase accessory subunit 2 OS=Mus musculus OX=10090 GN=Qtrt2 PE=1 S	0.73
Rbm22	Q8BHS3	Pre-mRNA-splicing factor RBM22 OS=Mus musculus OX=10090 GN=Rbm22 PE=1 SV=1	0.983333333
Map7d1	A2AJI0	MAP7 domain-containing protein 1 OS=Mus musculus OX=10090 GN=Map7d1 PE=1 SV=1	0.846666667
Lancl1	O89112	Glutathione S-transferase LANCL1 OS=Mus musculus OX=10090 GN=Lancl1 PE=1 SV=1	0.96
Champ1	Q8K327	Chromosome alignment-maintaining phosphoprotein 1 OS=Mus musculus OX=10090 GN=Champ1	0.926666667
Wipi2	Q80W47	WD repeat domain phosphoinositide-interacting protein 2 OS=Mus musculus OX=10090 GN=Wipi2	0.843333333
Faf1	P54731	FAS-associated factor 1 OS=Mus musculus OX=10090 GN=Faf1 PE=1 SV=2	0.876666667
Smpd4	Q6ZPR5	Sphingomyelin phosphodiesterase 4 OS=Mus musculus OX=10090 GN=Smpd4 PE=1 SV=2	1.083333333
Znf622	Q91VY9	Zinc finger protein 622 OS=Mus musculus OX=10090 GN=Znf622 PE=1 SV=1	1.033333333
Ptpn2	Q06180	Tyrosine-protein phosphatase non-receptor type 2 OS=Mus musculus OX=10090 GN=Ptpn2 PE=1 SV	0.886666667
Ash2l	Q91X20	Set1/Ash2 histone methyltransferase complex subunit ASH2 OS=Mus musculus OX=10090 GN=Ash2	1.026666667
Dhodh	O35435	Dihydroorotate dehydrogenase (quinone) mitochondrial OS=Mus musculus OX=10090 GN=Dhodh	0.973333333
Pde12	Q3TIU4	2' 5'-phosphodiesterase 12 OS=Mus musculus OX=10090 GN=Pde12 PE=1 SV=2	0.753333333
Tmem33	Q9CR67	Transmembrane protein 33 OS=Mus musculus OX=10090 GN=Tmem33 PE=1 SV=1	0.903333333
Trmt10c	Q3UFY8	tRNA methyltransferase 10 homolog C OS=Mus musculus OX=10090 GN=Trmt10c PE=1 SV=2	0.95
Bles03	Q8VD62	UPF0696 protein C11orf68 homolog OS=Mus musculus OX=10090 GN=Bles03 PE=1 SV=2	0.903333333
Ncstn	P57716	Nicastrin OS=Mus musculus OX=10090 GN=Ncstn PE=1 SV=3	1.02
Bcar1	Q61140	Breast cancer anti-estrogen resistance protein 1 OS=Mus musculus OX=10090 GN=Bcar1 PE=1 SV=2	0.983333333
Myadm	O35682	Myeloid-associated differentiation marker OS=Mus musculus OX=10090 GN=Myadm PE=1 SV=2	0.946666667
Ptpn12	P35831	Tyrosine-protein phosphatase non-receptor type 12 OS=Mus musculus OX=10090 GN=Ptpn12 PE=1	0.966666667

Trim56	Q80VI1	E3 ubiquitin-protein ligase TRIM56 OS=Mus musculus OX=10090 GN=Trim56 PE=1 SV=1	0.713333333
Ado	Q6PDY2	2-aminoethanethiol dioxygenase OS=Mus musculus OX=10090 GN=Ado PE=1 SV=2	0.89
Arpc3	Q9JM76	Actin-related protein 2/3 complex subunit 3 OS=Mus musculus OX=10090 GN=Arpc3 PE=1 SV=3	0.896666667
Exoc3	Q6KAR6	Exocyst complex component 3 OS=Mus musculus OX=10090 GN=Exoc3 PE=1 SV=2	1.01
Arhgef7	Q9ES28	Rho guanine nucleotide exchange factor 7 OS=Mus musculus OX=10090 GN=Arhgef7 PE=1 SV=2	0.893333333
Nck1	Q99M51	Cytoplasmic protein NCK1 OS=Mus musculus OX=10090 GN=Nck1 PE=1 SV=1	0.823333333
Nelfb	Q8C4Y3	Negative elongation factor B OS=Mus musculus OX=10090 GN=Nelfb PE=1 SV=2	0.92
Pgam5	Q8BX10	Serine/threonine-protein phosphatase PGAM5 mitochondrial OS=Mus musculus OX=10090 GN=Pg	0.816666667
Ivns1abp	Q920Q8	Influenza virus NS1A-binding protein homolog OS=Mus musculus OX=10090 GN=Ivns1abp PE=1 SV=	1.126666667
Ufl1	Q8CCJ3	E3 UFM1-protein ligase 1 OS=Mus musculus OX=10090 GN=Ufl1 PE=1 SV=2	0.906666667
Stat5b	P42232	Signal transducer and activator of transcription 5B OS=Mus musculus OX=10090 GN=Stat5b PE=1 S\	1.1
Specc1	Q5SXY1	Cytospin-B OS=Mus musculus OX=10090 GN=Specc1 PE=1 SV=2	0.853333333
Ptpn9	O35239	Tyrosine-protein phosphatase non-receptor type 9 OS=Mus musculus OX=10090 GN=Ptpn9 PE=1 SV	0.876666667
Mrpl13	Q9D1P0	39S ribosomal protein L13 mitochondrial OS=Mus musculus OX=10090 GN=Mrpl13 PE=1 SV=1	1.096666667
Ikbkb	O88351	Inhibitor of nuclear factor kappa-B kinase subunit beta OS=Mus musculus OX=10090 GN=Ikbkb PE=1	1.106666667
Zc2hc1a	Q8BJH1	Zinc finger C2HC domain-containing protein 1A OS=Mus musculus OX=10090 GN=Zc2hc1a PE=1 SV=	0.873333333
Pi4k2a	Q2TBE6	Phosphatidylinositol 4-kinase type 2-alpha OS=Mus musculus OX=10090 GN=Pi4k2a PE=1 SV=1	1.016666667
Mrps5	Q99N87	28S ribosomal protein S5 mitochondrial OS=Mus musculus OX=10090 GN=Mrps5 PE=1 SV=1	0.776666667
Bud31	Q6PGH1	Protein BUD31 homolog OS=Mus musculus OX=10090 GN=Bud31 PE=1 SV=2	0.893333333
Orc4	O88708	Origin recognition complex subunit 4 OS=Mus musculus OX=10090 GN=Orc4 PE=1 SV=2	0.693333333
Frg1	P97376	Protein FRG1 OS=Mus musculus OX=10090 GN=Frg1 PE=1 SV=2	0.96
Med16	Q6PGF3	Mediator of RNA polymerase II transcription subunit 16 OS=Mus musculus OX=10090 GN=Med16 P	0.996666667
Arhgap18	Q8K0Q5	Rho GTPase-activating protein 18 OS=Mus musculus OX=10090 GN=Arhgap18 PE=1 SV=1	0.746666667
Serpinc1	P32261	Antithrombin-III OS=Mus musculus OX=10090 GN=Serpinc1 PE=1 SV=1	0.093333333
Uba52	P62984	Ubiquitin-60S ribosomal protein L40 OS=Mus musculus OX=10090 GN=Uba52 PE=1 SV=2	0.783333333
Atp2b4	Q6Q477	Plasma membrane calcium-transporting ATPase 4 OS=Mus musculus OX=10090 GN=Atp2b4 PE=1 S\	0.756666667
Nras	P08556	GTPase NRas OS=Mus musculus OX=10090 GN=Nras PE=1 SV=1	1.103333333
H1-2	P15864	Histone H1.2 OS=Mus musculus OX=10090 GN=H1-2 PE=1 SV=2	0.806666667
Naa16	Q9DBB4	N-alpha-acetyltransferase 16 NatA auxiliary subunit OS=Mus musculus OX=10090 GN=Naa16 PE=2 S	0.913333333
Anp32b	Q9EST5	Acidic leucine-rich nuclear phosphoprotein 32 family member B OS=Mus musculus OX=10090 GN=/	0.853333333
Smad2	Q62432	Mothers against decapentaplegic homolog 2 OS=Mus musculus OX=10090 GN=Smad2 PE=1 SV=2	0.62
Yes1	Q04736	Tyrosine-protein kinase Yes OS=Mus musculus OX=10090 GN=Yes1 PE=1 SV=3	0.933333333
Rab5c	P35278	Ras-related protein Rab-5C OS=Mus musculus OX=10090 GN=Rab5c PE=1 SV=2	0.883333333
Mta3	Q924K8	Metastasis-associated protein MTA3 OS=Mus musculus OX=10090 GN=Mta3 PE=1 SV=1	0.956666667

Stk38	Q91VJ4	Serine/threonine-protein kinase 38 OS=Mus musculus OX=10090 GN=Stk38 PE=1 SV=1	0.776666667
Prkar2a	P12367	cAMP-dependent protein kinase type II-alpha regulatory subunit OS=Mus musculus OX=10090 GN=I	0.833333333
Sar1a	P36536	GTP-binding protein SAR1a OS=Mus musculus OX=10090 GN=Sar1a PE=1 SV=1	0.993333333
Gna11	P21278	Guanine nucleotide-binding protein subunit alpha-11 OS=Mus musculus OX=10090 GN=Gna11 PE=	0.896666667
Nfix	P70257	Nuclear factor 1 X-type OS=Mus musculus OX=10090 GN=Nfix PE=1 SV=2	0.763333333
Sar1b	Q9CQC9	GTP-binding protein SAR1b OS=Mus musculus OX=10090 GN=Sar1b PE=1 SV=1	0.85
Galnt1	O08912	Polypeptide N-acetylgalactosaminyltransferase 1 OS=Mus musculus OX=10090 GN=Galnt1 PE=1 SV=	0.773333333
Abi1	Q8CBW3	Abl interactor 1 OS=Mus musculus OX=10090 GN=Abi1 PE=1 SV=3	0.863333333
Tor1aip1	Q921T2	Torsin-1A-interacting protein 1 OS=Mus musculus OX=10090 GN=Tor1aip1 PE=1 SV=3	0.81
Khdrbs1	Q60749	KH domain-containing RNA-binding signal transduction-associated protein 1 OS=Mus musculus OX	0.846666667
Dpf2	Q61103	Zinc finger protein ubi-d4 OS=Mus musculus OX=10090 GN=Dpf2 PE=1 SV=1	0.993333333
Prpsap2	Q8R574	Phosphoribosyl pyrophosphate synthase-associated protein 2 OS=Mus musculus OX=10090 GN=Prp	0.906666667
Zfp36l2	P23949	mRNA decay activator protein ZFP36L2 OS=Mus musculus OX=10090 GN=Zfp36l2 PE=1 SV=2	0.883333333
Nfic	P70255	Nuclear factor 1 C-type OS=Mus musculus OX=10090 GN=Nfic PE=1 SV=1	0.906666667
Tmed9	Q99KF1	Transmembrane emp24 domain-containing protein 9 OS=Mus musculus OX=10090 GN=Tmed9 PE=	0.813333333
Stam2	O88811	Signal transducing adapter molecule 2 OS=Mus musculus OX=10090 GN=Stam2 PE=1 SV=1	0.796666667
H1-1	P43275	Histone H1.1 OS=Mus musculus OX=10090 GN=H1-1 PE=1 SV=2	0.796666667
Vapa	Q9WV55	Vesicle-associated membrane protein-associated protein A OS=Mus musculus OX=10090 GN=Vapa P	0.76
Gorasp2	Q99JX3	Golgi reassembly-stacking protein 2 OS=Mus musculus OX=10090 GN=Gorasp2 PE=1 SV=3	0.91
Hsd17b12	O70503	Very-long-chain 3-oxoacyl-CoA reductase OS=Mus musculus OX=10090 GN=Hsd17b12 PE=1 SV=1	0.783333333
Aprt	P08030	Adenine phosphoribosyltransferase OS=Mus musculus OX=10090 GN=Aprt PE=1 SV=2	0.963333333
Epn2	Q8CHU3	Epsin-2 OS=Mus musculus OX=10090 GN=Epn2 PE=1 SV=1	0.74
Crkl	P47941	Crk-like protein OS=Mus musculus OX=10090 GN=Crkl PE=1 SV=2	0.816666667
Parva	Q9EPC1	Alpha-parvin OS=Mus musculus OX=10090 GN=Parva PE=1 SV=1	0.93
Tnrc6b	Q8BKI2	Trinucleotide repeat-containing gene 6B protein OS=Mus musculus OX=10090 GN=Tnrc6b PE=1 SV=	1.053333333
Gsk3b	Q9WV60	Glycogen synthase kinase-3 beta OS=Mus musculus OX=10090 GN=Gsk3b PE=1 SV=2	0.91
Snrpa	Q62189	U1 small nuclear ribonucleoprotein A OS=Mus musculus OX=10090 GN=Snrpa PE=1 SV=3	0.863333333
Vapb	Q9QY76	Vesicle-associated membrane protein-associated protein B OS=Mus musculus OX=10090 GN=Vapb F	0.866666667
Gstm5	P48774	Glutathione S-transferase Mu 5 OS=Mus musculus OX=10090 GN=Gstm5 PE=1 SV=1	0.896666667
Bag5	Q8CI32	BAG family molecular chaperone regulator 5 OS=Mus musculus OX=10090 GN=Bag5 PE=1 SV=1	0.843333333
Strn4	P58404	Striatin-4 OS=Mus musculus OX=10090 GN=Strn4 PE=1 SV=2	0.843333333
Irf2bp1	Q8R3Y8	Interferon regulatory factor 2-binding protein 1 OS=Mus musculus OX=10090 GN=Irf2bp1 PE=1 SV=	0.76
Hars2	Q99KK9	Histidine--tRNA ligase mitochondrial OS=Mus musculus OX=10090 GN=Hars2 PE=1 SV=1	0.883333333
Iws1	Q8C1D8	Protein IWS1 homolog OS=Mus musculus OX=10090 GN=Iws1 PE=1 SV=1	0.946666667

Rhot1	Q8BG51	Mitochondrial Rho GTPase 1 OS=Mus musculus OX=10090 GN=Rhot1 PE=1 SV=1	0.84
Fabp5	Q05816	Fatty acid-binding protein 5 OS=Mus musculus OX=10090 GN=Fabp5 PE=1 SV=3	0.93
Fus	P56959	RNA-binding protein FUS OS=Mus musculus OX=10090 GN=Fus PE=1 SV=1	0.793333333
Arfgap1	Q9EPJ9	ADP-ribosylation factor GTPase-activating protein 1 OS=Mus musculus OX=10090 GN=Arfgap1 PE=1	0.856666667
Rplp2	P99027	60S acidic ribosomal protein P2 OS=Mus musculus OX=10090 GN=Rplp2 PE=1 SV=3	0.903333333
Eif6	O55135	Eukaryotic translation initiation factor 6 OS=Mus musculus OX=10090 GN=Eif6 PE=1 SV=2	0.89
Tpt1	P63028	Translationally-controlled tumor protein OS=Mus musculus OX=10090 GN=Tpt1 PE=1 SV=1	0.906666667
Atp5pd	Q9DCX2	ATP synthase subunit d mitochondrial OS=Mus musculus OX=10090 GN=Atp5pd PE=1 SV=3	0.903333333
Wdr61	Q9ERF3	WD repeat-containing protein 61 OS=Mus musculus OX=10090 GN=Wdr61 PE=1 SV=1	0.843333333
Psma1	Q9R1P4	Proteasome subunit alpha type-1 OS=Mus musculus OX=10090 GN=Psma1 PE=1 SV=1	0.92
Dazap1	Q9JII5	DAZ-associated protein 1 OS=Mus musculus OX=10090 GN=Dazap1 PE=1 SV=2	0.896666667
Psma4	Q9R1P0	Proteasome subunit alpha type-4 OS=Mus musculus OX=10090 GN=Psma4 PE=1 SV=1	0.96
Csnk2b	P67871	Casein kinase II subunit beta OS=Mus musculus OX=10090 GN=Csnk2b PE=1 SV=1	0.86
Psmb7	P70195	Proteasome subunit beta type-7 OS=Mus musculus OX=10090 GN=Psmb7 PE=1 SV=1	0.893333333
Fkbp3	Q62446	Peptidyl-prolyl cis-trans isomerase FKBP3 OS=Mus musculus OX=10090 GN=Fkbp3 PE=1 SV=2	0.876666667
Adh5	P28474	Alcohol dehydrogenase class-3 OS=Mus musculus OX=10090 GN=Adh5 PE=1 SV=3	0.82
Rfc2	Q9WUK4	Replication factor C subunit 2 OS=Mus musculus OX=10090 GN=Rfc2 PE=1 SV=1	0.763333333
Prdx5	P99029	Peroxiredoxin-5 mitochondrial OS=Mus musculus OX=10090 GN=Prdx5 PE=1 SV=2	0.856666667
Rpl10	Q6ZWV3	60S ribosomal protein L10 OS=Mus musculus OX=10090 GN=Rpl10 PE=1 SV=3	0.89
Luc7l3	Q5SUF2	Luc7-like protein 3 OS=Mus musculus OX=10090 GN=Luc7l3 PE=1 SV=1	0.85
Agfg1	Q8K2K6	Arf-GAP domain and FG repeat-containing protein 1 OS=Mus musculus OX=10090 GN=Agfg1 PE=1 SV	0.89
Palm	Q9Z0P4	Paralemmin-1 OS=Mus musculus OX=10090 GN=Palm PE=1 SV=1	0.8
Wdr12	Q9JJA4	Ribosome biogenesis protein WDR12 OS=Mus musculus OX=10090 GN=Wdr12 PE=1 SV=1	0.896666667
Prkci	Q62074	Protein kinase C iota type OS=Mus musculus OX=10090 GN=Prkci PE=1 SV=3	0.8
Psmb3	Q9R1P1	Proteasome subunit beta type-3 OS=Mus musculus OX=10090 GN=Psmb3 PE=1 SV=1	0.88
Vps29	Q9QZ88	Vacuolar protein sorting-associated protein 29 OS=Mus musculus OX=10090 GN=Vps29 PE=1 SV=1	0.906666667
Golim4	Q8BXA1	Golgi integral membrane protein 4 OS=Mus musculus OX=10090 GN=Golim4 PE=1 SV=1	0.746666667
Cox5a	P12787	Cytochrome c oxidase subunit 5A mitochondrial OS=Mus musculus OX=10090 GN=Cox5a PE=1 SV=	0.813333333
Ltbp3	Q61810	Latent-transforming growth factor beta-binding protein 3 OS=Mus musculus OX=10090 GN=Ltbp3 I	1.083333333
Mtco2	P00405	Cytochrome c oxidase subunit 2 OS=Mus musculus OX=10090 GN=Mtco2 PE=1 SV=1	0.863333333
Atp5f1d	Q9D3D9	ATP synthase subunit delta mitochondrial OS=Mus musculus OX=10090 GN=Atp5f1d PE=1 SV=1	0.883333333
Naxd	Q9CZ42	ATP-dependent (S)-NAD(P)H-hydrate dehydratase OS=Mus musculus OX=10090 GN=Naxd PE=1 SV=1	0.836666667
Creld2	Q9CYA0	Protein disulfide isomerase Creld2 OS=Mus musculus OX=10090 GN=Creld2 PE=1 SV=1	0.916666667
Pacsin3	Q99JB8	Protein kinase C and casein kinase II substrate protein 3 OS=Mus musculus OX=10090 GN=Pacsin3 P	0.846666667

Cstf1	Q99LC2	Cleavage stimulation factor subunit 1 OS=Mus musculus OX=10090 GN=Cstf1 PE=1 SV=1	0.87
Urod	P70697	Uroporphyrinogen decarboxylase OS=Mus musculus OX=10090 GN=Urod PE=1 SV=2	0.84
Nhlrc2	Q8BZW8	NHL repeat-containing protein 2 OS=Mus musculus OX=10090 GN=Nhlrc2 PE=1 SV=1	0.77
Map2k4	P47809	Dual specificity mitogen-activated protein kinase kinase 4 OS=Mus musculus OX=10090 GN=Map2k	0.856666667
Spcs2	Q9CYN2	Signal peptidase complex subunit 2 OS=Mus musculus OX=10090 GN=Spcs2 PE=1 SV=1	0.82
Cnpy2	Q9QXT0	Protein canopy homolog 2 OS=Mus musculus OX=10090 GN=Cnpy2 PE=1 SV=1	0.936666667
Ube2i	P63280	SUMO-conjugating enzyme UBC9 OS=Mus musculus OX=10090 GN=Ube2i PE=1 SV=1	0.806666667
Rbfox2	Q8BP71	RNA binding protein fox-1 homolog 2 OS=Mus musculus OX=10090 GN=Rbfox2 PE=1 SV=2	0.843333333
Nifk	Q91VE6	MKI67 FHA domain-interacting nucleolar phosphoprotein OS=Mus musculus OX=10090 GN=Nifk PE	0.816666667
Bpnt1	Q9Z0S1	3'(2') 5'-bisphosphate nucleotidase 1 OS=Mus musculus OX=10090 GN=Bpnt1 PE=1 SV=2	0.95
Spart	Q8R1X6	Spartin OS=Mus musculus OX=10090 GN=Spart PE=1 SV=1	0.78
Ehmt1	Q5DW34	Histone-lysine N-methyltransferase EHMT1 OS=Mus musculus OX=10090 GN=Ehmt1 PE=1 SV=2	0.886666667
Acot2	Q9QYR9	Acyl-coenzyme A thioesterase 2 mitochondrial OS=Mus musculus OX=10090 GN=Acot2 PE=1 SV=2	0.893333333
Psme3	P61290	Proteasome activator complex subunit 3 OS=Mus musculus OX=10090 GN=Psme3 PE=1 SV=1	0.91
Vasp	P70460	Vasodilator-stimulated phosphoprotein OS=Mus musculus OX=10090 GN=Vasp PE=1 SV=4	0.92
Cmtr1	Q9DBC3	Cap-specific mRNA (nucleoside-2'-O-)-methyltransferase 1 OS=Mus musculus OX=10090 GN=Cmtr1 I	1.006666667
RtcA	Q9D7H3	RNA 3'-terminal phosphate cyclase OS=Mus musculus OX=10090 GN=RtcA PE=1 SV=2	0.89
Zcchc8	Q9CYA6	Zinc finger CCHC domain-containing protein 8 OS=Mus musculus OX=10090 GN=Zcchc8 PE=1 SV=3	1.156666667
Fip1l1	Q9D824	Pre-mRNA 3'-end-processing factor FIP1 OS=Mus musculus OX=10090 GN=Fip1l1 PE=1 SV=1	0.823333333
Rps12	P63323	40S ribosomal protein S12 OS=Mus musculus OX=10090 GN=Rps12 PE=1 SV=2	0.906666667
Ankrd28	Q505D1	Serine/threonine-protein phosphatase 6 regulatory ankyrin repeat subunit A OS=Mus musculus OX=	0.916666667
Cask	O70589	Peripheral plasma membrane protein CASK OS=Mus musculus OX=10090 GN=Cask PE=1 SV=2	1.073333333
Sf3a2	Q62203	Splicing factor 3A subunit 2 OS=Mus musculus OX=10090 GN=Sf3a2 PE=1 SV=2	0.946666667
Pgp	Q8CHP8	Glycerol-3-phosphate phosphatase OS=Mus musculus OX=10090 GN=Pgp PE=1 SV=1	0.863333333
Rab11b	P46638	Ras-related protein Rab-11B OS=Mus musculus OX=10090 GN=Rab11b PE=1 SV=3	0.903333333
Psmg1	Q9JK23	Proteasome assembly chaperone 1 OS=Mus musculus OX=10090 GN=Psmg1 PE=1 SV=1	0.84
Chmp4b	Q9D8B3	Charged multivesicular body protein 4b OS=Mus musculus OX=10090 GN=Chmp4b PE=1 SV=2	0.816666667
Pogz	Q8BZH4	Pogo transposable element with ZNF domain OS=Mus musculus OX=10090 GN=Pogz PE=1 SV=2	0.993333333
Sh3glb1	Q9JK48	Endophilin-B1 OS=Mus musculus OX=10090 GN=Sh3glb1 PE=1 SV=1	0.86
Bcat2	O35855	Branched-chain-amino-acid aminotransferase mitochondrial OS=Mus musculus OX=10090 GN=Bca	1.11
Ddt	O35215	D-dopachrome decarboxylase OS=Mus musculus OX=10090 GN=Ddt PE=1 SV=3	0.883333333
Saal1	Q9D2C2	Protein SAAL1 OS=Mus musculus OX=10090 GN=Saal1 PE=1 SV=1	1.003333333
Dohh	Q99LN9	Deoxyhypusine hydroxylase OS=Mus musculus OX=10090 GN=Dohh PE=1 SV=2	0.9
Ckb	Q04447	Creatine kinase B-type OS=Mus musculus OX=10090 GN=Ckb PE=1 SV=1	0.836666667

Ctr9	Q62018	RNA polymerase-associated protein CTR9 homolog OS=Mus musculus OX=10090 GN=Ctr9 PE=1 SV=:	0.81
Polr1c	P52432	DNA-directed RNA polymerases I and III subunit RPAC1 OS=Mus musculus OX=10090 GN=Polr1c PE=	0.926666667
Tm9sf4	Q8BH24	Transmembrane 9 superfamily member 4 OS=Mus musculus OX=10090 GN=Tm9sf4 PE=1 SV=1	0.786666667
Sms	P97355	Spermine synthase OS=Mus musculus OX=10090 GN=Sms PE=1 SV=1	1.023333333
Galk2	Q68FH4	N-acetylgalactosamine kinase OS=Mus musculus OX=10090 GN=Galk2 PE=1 SV=1	0.85
Clpx	Q9JHS4	ATP-dependent Clp protease ATP-binding subunit clpX-like mitochondrial OS=Mus musculus OX=1(	0.766666667
Csnk1a1	Q8BK63	Casein kinase I isoform alpha OS=Mus musculus OX=10090 GN=Csnk1a1 PE=1 SV=2	0.796666667
Stat6	P52633	Signal transducer and transcription activator 6 OS=Mus musculus OX=10090 GN=Stat6 PE=1 SV=2	0.953333333
Uqcrfs1	Q9CR68	Cytochrome b-c1 complex subunit Rieske mitochondrial OS=Mus musculus OX=10090 GN=Uqcrfs1	0.9
Naa50	Q6PGB6	N-alpha-acetyltransferase 50 OS=Mus musculus OX=10090 GN=Naa50 PE=1 SV=1	0.89
Man2b1	O09159	Lysosomal alpha-mannosidase OS=Mus musculus OX=10090 GN=Man2b1 PE=1 SV=4	1.223333333
Acy1	Q99JW2	Aminoacylase-1 OS=Mus musculus OX=10090 GN=Acy1 PE=1 SV=1	1.143333333
Dclk1	Q9JLM8	Serine/threonine-protein kinase DCLK1 OS=Mus musculus OX=10090 GN=Dclk1 PE=1 SV=1	0.886666667
Fbxo30	Q8BJL1	F-box only protein 30 OS=Mus musculus OX=10090 GN=Fbxo30 PE=1 SV=2	0.926666667
Ndufa9	Q9DC69	NADH dehydrogenase [ubiquinone] 1 alpha subcomplex subunit 9 mitochondrial OS=Mus musculu	0.753333333
Pdxk	Q8K183	Pyridoxal kinase OS=Mus musculus OX=10090 GN=Pdxk PE=1 SV=1	0.836666667
Sdhb	Q9CQA3	Succinate dehydrogenase [ubiquinone] iron-sulfur subunit mitochondrial OS=Mus musculus OX=1(	0.98
Eci1	P42125	Enoyl-CoA delta isomerase 1 mitochondrial OS=Mus musculus OX=10090 GN=Eci1 PE=1 SV=2	0.873333333
Mccc2	Q3ULD5	Methylcrotonoyl-CoA carboxylase beta chain mitochondrial OS=Mus musculus OX=10090 GN=Mcc	0.92
Grhpr	Q91Z53	Glyoxylate reductase/hydroxypyruvate reductase OS=Mus musculus OX=10090 GN=Grhpr PE=1 SV=	0.97
Tiprl	Q8BH58	TIP41-like protein OS=Mus musculus OX=10090 GN=Tiprl PE=1 SV=1	0.963333333
Exoc2	Q9D4H1	Exocyst complex component 2 OS=Mus musculus OX=10090 GN=Exoc2 PE=1 SV=1	0.85
Senp3	Q9EP97	Sentrin-specific protease 3 OS=Mus musculus OX=10090 GN=Senp3 PE=1 SV=1	1.093333333
Pwp1	Q99LL5	Periodic tryptophan protein 1 homolog OS=Mus musculus OX=10090 GN=Pwp1 PE=1 SV=1	0.86
Cpq	Q9WVJ3	Carboxypeptidase Q OS=Mus musculus OX=10090 GN=Cpq PE=1 SV=1	0.926666667
Tsn	Q62348	Translin OS=Mus musculus OX=10090 GN=Tsn PE=1 SV=1	0.853333333
Urb1	Q571H0	Nucleolar pre-ribosomal-associated protein 1 OS=Mus musculus OX=10090 GN=Urb1 PE=1 SV=2	0.876666667
Cd2ap	Q9JLQ0	CD2-associated protein OS=Mus musculus OX=10090 GN=Cd2ap PE=1 SV=3	0.88
Smarcad1	Q04692	SWI/SNF-related matrix-associated actin-dependent regulator of chromatin subfamily A containing	0.9
Rpp30	O88796	Ribonuclease P protein subunit p30 OS=Mus musculus OX=10090 GN=Rpp30 PE=1 SV=1	0.843333333
Pola2	P33611	DNA polymerase alpha subunit B OS=Mus musculus OX=10090 GN=Pola2 PE=1 SV=2	0.9
Cep55	Q8BT07	Centrosomal protein of 55 kDa OS=Mus musculus OX=10090 GN=Cep55 PE=1 SV=2	0.903333333
Tecr	Q9CY27	Very-long-chain enoyl-CoA reductase OS=Mus musculus OX=10090 GN=Tecr PE=1 SV=1	0.843333333
Dcaf13	Q6PAC3	DDB1- and CUL4-associated factor 13 OS=Mus musculus OX=10090 GN=Dcaf13 PE=2 SV=2	0.88

Ascc2	Q91WR3	Activating signal cointegrator 1 complex subunit 2 OS=Mus musculus OX=10090 GN=Ascc2 PE=1 SV	0.85
Atp1b3	P97370	Sodium/potassium-transporting ATPase subunit beta-3 OS=Mus musculus OX=10090 GN=Atp1b3 PI	0.836666667
Ncapd3	Q6ZQK0	Condensin-2 complex subunit D3 OS=Mus musculus OX=10090 GN=Ncapd3 PE=1 SV=3	0.813333333
Tanc1	Q0VGY8	Protein TANC1 OS=Mus musculus OX=10090 GN=Tanc1 PE=1 SV=2	0.93
Hpf1	Q8CFE2	Histone PARylation factor 1 OS=Mus musculus OX=10090 GN=Hpf1 PE=1 SV=1	0.883333333
Trmt1	Q3TX08	tRNA (guanine(26)-N(2))-dimethyltransferase OS=Mus musculus OX=10090 GN=Trmt1 PE=1 SV=2	0.803333333
Acadsb	Q9DBL1	Short/branched chain specific acyl-CoA dehydrogenase mitochondrial OS=Mus musculus OX=1009(	1.08
Med14	A2ABV5	Mediator of RNA polymerase II transcription subunit 14 OS=Mus musculus OX=10090 GN=Med14 PI	1.11
Rabep1	O35551	Rab GTPase-binding effector protein 1 OS=Mus musculus OX=10090 GN=Rabep1 PE=1 SV=2	0.833333333
Vps51	Q3UVL4	Vacuolar protein sorting-associated protein 51 homolog OS=Mus musculus OX=10090 GN=Vps51 PI	0.733333333
Impact	O55091	Protein IMPACT OS=Mus musculus OX=10090 GN=Impact PE=1 SV=2	0.776666667
Ankh	Q9JHZ2	Progressive ankylosis protein OS=Mus musculus OX=10090 GN=Ankh PE=2 SV=1	0.876666667
Sptlc2	P97363	Serine palmitoyltransferase 2 OS=Mus musculus OX=10090 GN=Sptlc2 PE=1 SV=2	0.916666667
Snx27	Q3UHD6	Sorting nexin-27 OS=Mus musculus OX=10090 GN=Snx27 PE=1 SV=2	0.796666667
Polr1b	P70700	DNA-directed RNA polymerase I subunit RPA2 OS=Mus musculus OX=10090 GN=Polr1b PE=1 SV=2	0.856666667
Cdc27	A2A6Q5	Cell division cycle protein 27 homolog OS=Mus musculus OX=10090 GN=Cdc27 PE=1 SV=1	0.896666667
Ufd1	P70362	Ubiquitin recognition factor in ER-associated degradation protein 1 OS=Mus musculus OX=10090 G	0.933333333
Pccb	Q99MN9	Propionyl-CoA carboxylase beta chain mitochondrial OS=Mus musculus OX=10090 GN=Pccb PE=1 S	0.906666667
Mrto4	Q9D0I8	mRNA turnover protein 4 homolog OS=Mus musculus OX=10090 GN=Mrto4 PE=1 SV=1	0.893333333
Pask	Q8CEE6	PAS domain-containing serine/threonine-protein kinase OS=Mus musculus OX=10090 GN=Pask PE=	1.36
Rpl32	P62911	60S ribosomal protein L32 OS=Mus musculus OX=10090 GN=Rpl32 PE=1 SV=2	0.87
Abcf3	Q8K268	ATP-binding cassette sub-family F member 3 OS=Mus musculus OX=10090 GN=Abcf3 PE=1 SV=1	0.85
Anxa8	O35640	Annexin A8 OS=Mus musculus OX=10090 GN=Anxa8 PE=1 SV=2	0.83
Mvd	Q99JF5	Diphosphomevalonate decarboxylase OS=Mus musculus OX=10090 GN=Mvd PE=1 SV=2	0.936666667
Actr10	Q9QZB7	Actin-related protein 10 OS=Mus musculus OX=10090 GN=Actr10 PE=1 SV=2	1.046666667
Gtf2b	P62915	Transcription initiation factor IIB OS=Mus musculus OX=10090 GN=Gtf2b PE=1 SV=1	0.826666667
Psmd9	Q9CR00	26S proteasome non-ATPase regulatory subunit 9 OS=Mus musculus OX=10090 GN=Psmd9 PE=1 SV	0.906666667
Enpp1	P06802	Ectonucleotide pyrophosphatase/phosphodiesterase family member 1 OS=Mus musculus OX=1009(	0.726666667
Nt5dc3	Q3UHB1	5'-nucleotidase domain-containing protein 3 OS=Mus musculus OX=10090 GN=Nt5dc3 PE=1 SV=1	0.883333333
Sbds	P70122	Ribosome maturation protein SBDS OS=Mus musculus OX=10090 GN=Sbds PE=1 SV=4	0.833333333
Diaph2	O70566	Protein diaphanous homolog 2 OS=Mus musculus OX=10090 GN=Diaph2 PE=1 SV=2	0.79
Mpst	Q99J99	3-mercaptopyruvate sulfurtransferase OS=Mus musculus OX=10090 GN=Mpst PE=1 SV=4	0.85
Aga	Q64191	N(4)-(beta-N-acetylglucosaminyl)-L-asparaginase OS=Mus musculus OX=10090 GN=Aga PE=1 SV=1	0.886666667
Med24	Q99K74	Mediator of RNA polymerase II transcription subunit 24 OS=Mus musculus OX=10090 GN=Med24 PI	0.923333333

Plk1	Q07832	Serine/threonine-protein kinase PLK1 OS=Mus musculus OX=10090 GN=Plk1 PE=1 SV=2	0.91
Ube2z	Q3UE37	Ubiquitin-conjugating enzyme E2 Z OS=Mus musculus OX=10090 GN=Ube2z PE=1 SV=2	0.813333333
Wrnip1	Q91XU0	ATPase WRNIP1 OS=Mus musculus OX=10090 GN=Wrnip1 PE=1 SV=2	0.976666667
Pi4ka	E9Q3L2	Phosphatidylinositol 4-kinase alpha OS=Mus musculus OX=10090 GN=Pi4ka PE=1 SV=2	0.693333333
Ints4	Q8CIM8	Integrator complex subunit 4 OS=Mus musculus OX=10090 GN=Ints4 PE=1 SV=1	1.106666667
Ccnb1	P24860	G2/mitotic-specific cyclin-B1 OS=Mus musculus OX=10090 GN=Ccnb1 PE=1 SV=3	0.83
Usp8	Q80U87	Ubiquitin carboxyl-terminal hydrolase 8 OS=Mus musculus OX=10090 GN=Usp8 PE=1 SV=2	0.866666667
Rexo4	Q6PAQ4	RNA exonuclease 4 OS=Mus musculus OX=10090 GN=Rexo4 PE=2 SV=2	0.936666667
Eed	Q921E6	Polycomb protein EED OS=Mus musculus OX=10090 GN=Eed PE=1 SV=1	0.803333333
Ufsp2	Q99K23	Ufm1-specific protease 2 OS=Mus musculus OX=10090 GN=Ufsp2 PE=1 SV=1	0.96
Apaf1	O88879	Apoptotic protease-activating factor 1 OS=Mus musculus OX=10090 GN=Apaf1 PE=1 SV=3	0.956666667
Acad8	Q9D7B6	Isobutyryl-CoA dehydrogenase mitochondrial OS=Mus musculus OX=10090 GN=Acad8 PE=1 SV=2	0.853333333
Spats2	Q8K1N4	Spermatogenesis-associated serine-rich protein 2 OS=Mus musculus OX=10090 GN=Spats2 PE=1 SV=	0.84
Ubxn7	Q6P5G6	UBX domain-containing protein 7 OS=Mus musculus OX=10090 GN=Ubxn7 PE=1 SV=2	0.82
Paip1	Q8VE62	Polyadenylate-binding protein-interacting protein 1 OS=Mus musculus OX=10090 GN=Paip1 PE=1 S	0.9
Nek9	Q8K1R7	Serine/threonine-protein kinase Nek9 OS=Mus musculus OX=10090 GN=Nek9 PE=1 SV=2	0.94
Rsu1	Q01730	Ras suppressor protein 1 OS=Mus musculus OX=10090 GN=Rsu1 PE=1 SV=3	0.9
Hs1bp3	Q3TC93	HCLS1-binding protein 3 OS=Mus musculus OX=10090 GN=Hs1bp3 PE=1 SV=2	0.953333333
Mia2	Q91ZV0	Melanoma inhibitory activity protein 2 OS=Mus musculus OX=10090 GN=Mia2 PE=1 SV=3	1.09
Rpl26	P61255	60S ribosomal protein L26 OS=Mus musculus OX=10090 GN=Rpl26 PE=1 SV=1	0.913333333
Mrpl39	Q9JKF7	39S ribosomal protein L39 mitochondrial OS=Mus musculus OX=10090 GN=Mrpl39 PE=1 SV=4	0.723333333
Hexb	P20060	Beta-hexosaminidase subunit beta OS=Mus musculus OX=10090 GN=Hexb PE=1 SV=2	0.863333333
Wdr70	Q3TWF6	WD repeat-containing protein 70 OS=Mus musculus OX=10090 GN=Wdr70 PE=1 SV=1	0.86
Sorbs1	Q62417	Sorbin and SH3 domain-containing protein 1 OS=Mus musculus OX=10090 GN=Sorbs1 PE=1 SV=2	0.963333333
Cpd	O89001	Carboxypeptidase D OS=Mus musculus OX=10090 GN=Cpd PE=1 SV=2	0.793333333
Rhoa	Q9QUI0	Transforming protein RhoA OS=Mus musculus OX=10090 GN=Rhoa PE=1 SV=1	0.9
Rhoc	Q62159	Rho-related GTP-binding protein RhoC OS=Mus musculus OX=10090 GN=Rhoc PE=1 SV=2	0.84
Pak3	Q61036	Serine/threonine-protein kinase PAK 3 OS=Mus musculus OX=10090 GN=Pak3 PE=1 SV=2	0.863333333
H3c11	P68433	Histone H3.1 OS=Mus musculus OX=10090 GN=H3c1 PE=1 SV=2	0.86
Arf4	P61750	ADP-ribosylation factor 4 OS=Mus musculus OX=10090 GN=Arf4 PE=1 SV=2	0.863333333
Ep300	B2RWS6	Histone acetyltransferase p300 OS=Mus musculus OX=10090 GN=Ep300 PE=1 SV=2	0.82
Rab1b	Q9D1G1	Ras-related protein Rab-1B OS=Mus musculus OX=10090 GN=Rab1b PE=1 SV=1	0.85
Ythdf1	P59326	YTH domain-containing family protein 1 OS=Mus musculus OX=10090 GN=Ythdf1 PE=1 SV=1	0.756666667
Prps2	Q9CS42	Ribose-phosphate pyrophosphokinase 2 OS=Mus musculus OX=10090 GN=Prps2 PE=1 SV=4	0.846666667

Klc2	O88448	Kinesin light chain 2 OS=Mus musculus OX=10090 GN=Klc2 PE=1 SV=1	0.76
Sfxn3	Q91V61	Sideroflexin-3 OS=Mus musculus OX=10090 GN=Sfxn3 PE=1 SV=1	0.833333333
Cfl2	P45591	Cofilin-2 OS=Mus musculus OX=10090 GN=Cfl2 PE=1 SV=1	0.943333333
Gnaq	P21279	Guanine nucleotide-binding protein G(q) subunit alpha OS=Mus musculus OX=10090 GN=Gnaq PE=	0.966666667
Rprd1b	Q9CSU0	Regulation of nuclear pre-mRNA domain-containing protein 1B OS=Mus musculus OX=10090 GN=R	0.946666667
Fam98a	Q3TJZ6	Protein FAM98A OS=Mus musculus OX=10090 GN=Fam98a PE=1 SV=1	0.84
Fam98b	Q80VD1	Protein FAM98B OS=Mus musculus OX=10090 GN=Fam98b PE=1 SV=1	0.876666667
Snx3	O70492	Sorting nexin-3 OS=Mus musculus OX=10090 GN=Snx3 PE=1 SV=3	0.936666667
Afg3l2	Q8JZQ2	AFG3-like protein 2 OS=Mus musculus OX=10090 GN=Afg3l2 PE=1 SV=1	1.043333333
Araf	P04627	Serine/threonine-protein kinase A-Raf OS=Mus musculus OX=10090 GN=Araf PE=1 SV=2	1.246666667
Capza1	P47753	F-actin-capping protein subunit alpha-1 OS=Mus musculus OX=10090 GN=Capza1 PE=1 SV=4	0.846666667
Prpsap1	Q9D0M1	Phosphoribosyl pyrophosphate synthase-associated protein 1 OS=Mus musculus OX=10090 GN=Pr	0.956666667
Ppp4c	P97470	Serine/threonine-protein phosphatase 4 catalytic subunit OS=Mus musculus OX=10090 GN=Ppp4c	0.856666667
Slc12a4	Q9JIS8	Solute carrier family 12 member 4 OS=Mus musculus OX=10090 GN=Slc12a4 PE=1 SV=2	0.88
Rnf20	Q5DTM8	E3 ubiquitin-protein ligase BRE1A OS=Mus musculus OX=10090 GN=Rnf20 PE=1 SV=2	0.82
Erlin2	Q8BFZ9	Erlin-2 OS=Mus musculus OX=10090 GN=Erlin2 PE=1 SV=1	0.933333333
Elmo2	Q8BHL5	Engulfment and cell motility protein 2 OS=Mus musculus OX=10090 GN=Elmo2 PE=1 SV=1	0.87
Hint1	P70349	Histidine triad nucleotide-binding protein 1 OS=Mus musculus OX=10090 GN=Hint1 PE=1 SV=3	0.886666667
Hnrnpa0	Q9CX86	Heterogeneous nuclear ribonucleoprotein A0 OS=Mus musculus OX=10090 GN=Hnrnpa0 PE=1 SV=1	0.873333333
Yap1	P46938	Transcriptional coactivator YAP1 OS=Mus musculus OX=10090 GN=Yap1 PE=1 SV=2	0.776666667
Prdx3	P20108	Thioredoxin-dependent peroxide reductase mitochondrial OS=Mus musculus OX=10090 GN=Prdx3	0.92
Rac1	P63001	Ras-related C3 botulinum toxin substrate 1 OS=Mus musculus OX=10090 GN=Rac1 PE=1 SV=1	0.873333333
Rab21	P35282	Ras-related protein Rab-21 OS=Mus musculus OX=10090 GN=Rab21 PE=1 SV=4	0.886666667
Coro1b	Q9WUM3	Coronin-1B OS=Mus musculus OX=10090 GN=Coro1b PE=1 SV=1	0.82
Arfgap2	Q99K28	ADP-ribosylation factor GTPase-activating protein 2 OS=Mus musculus OX=10090 GN=Arfgap2 PE=1	0.98
Vps26a	P40336	Vacuolar protein sorting-associated protein 26A OS=Mus musculus OX=10090 GN=Vps26a PE=1 SV=	0.843333333
Ak2	Q9WTP6	Adenylate kinase 2 mitochondrial OS=Mus musculus OX=10090 GN=Ak2 PE=1 SV=5	0.92
Fads2	Q9Z0R9	Acyl-CoA 6-desaturase OS=Mus musculus OX=10090 GN=Fads2 PE=1 SV=1	0.873333333
Gatad2b	Q8VHR5	Transcriptional repressor p66-beta OS=Mus musculus OX=10090 GN=Gatad2b PE=1 SV=1	0.82
Sbf1	Q6ZPE2	Myotubularin-related protein 5 OS=Mus musculus OX=10090 GN=Sbf1 PE=1 SV=2	0.99
Cdk11b	P24788	Cyclin-dependent kinase 11B OS=Mus musculus OX=10090 GN=Cdk11b PE=1 SV=2	0.686666667
Vps26b	Q8C0E2	Vacuolar protein sorting-associated protein 26B OS=Mus musculus OX=10090 GN=Vps26b PE=1 SV=	0.94
Ube2c	Q9D1C1	Ubiquitin-conjugating enzyme E2 C OS=Mus musculus OX=10090 GN=Ube2c PE=1 SV=1	0.906666667
Rbm26	Q6NZN0	RNA-binding protein 26 OS=Mus musculus OX=10090 GN=Rbm26 PE=1 SV=2	0.91

Rbm27	Q5SFM8	RNA-binding protein 27 OS=Mus musculus OX=10090 GN=Rbm27 PE=1 SV=3	0.926666667
Bicd2	Q921C5	Protein bicaudal D homolog 2 OS=Mus musculus OX=10090 GN=Bicd2 PE=1 SV=1	1.126666667
Calm1	P0DP26	Calmodulin-1 OS=Mus musculus OX=10090 GN=Calm1 PE=1 SV=1	0.89
Calm3	P0DP28	Calmodulin-3 OS=Mus musculus OX=10090 GN=Calm3 PE=1 SV=1	0.89
Calm2	P0DP27	Calmodulin-2 OS=Mus musculus OX=10090 GN=Calm2 PE=1 SV=1	0.89
Cnbp	P53996	Cellular nucleic acid-binding protein OS=Mus musculus OX=10090 GN=Cnbp PE=1 SV=2	0.916666667
Idh3g	P70404	Isocitrate dehydrogenase [NAD] subunit gamma 1 mitochondrial OS=Mus musculus OX=10090 GN=	0.883333333
Rps15	P62843	40S ribosomal protein S15 OS=Mus musculus OX=10090 GN=Rps15 PE=1 SV=2	0.876666667
Grwd1	Q810D6	Glutamate-rich WD repeat-containing protein 1 OS=Mus musculus OX=10090 GN=Grwd1 PE=1 SV=2	0.843333333
Rcn2	Q8BP92	Reticulocalbin-2 OS=Mus musculus OX=10090 GN=Rcn2 PE=1 SV=1	0.826666667
Rps17	P63276	40S ribosomal protein S17 OS=Mus musculus OX=10090 GN=Rps17 PE=1 SV=2	0.866666667
Gcc2	Q8CHG3	GRIP and coiled-coil domain-containing protein 2 OS=Mus musculus OX=10090 GN=Gcc2 PE=1 SV=	1.063333333
Gas1	Q01721	Growth arrest-specific protein 1 OS=Mus musculus OX=10090 GN=Gas1 PE=1 SV=2	0.73
Fth1	P09528	Ferritin heavy chain OS=Mus musculus OX=10090 GN=Fth1 PE=1 SV=2	0.913333333
Snap23	O09044	Synaptosomal-associated protein 23 OS=Mus musculus OX=10090 GN=Snap23 PE=1 SV=1	0.96
Uchl3	Q9JKB1	Ubiquitin carboxyl-terminal hydrolase isozyme L3 OS=Mus musculus OX=10090 GN=Uchl3 PE=1 SV=	0.88
Myef2	Q8C854	Myelin expression factor 2 OS=Mus musculus OX=10090 GN=Myef2 PE=1 SV=1	0.94
Mat2b	Q99LB6	Methionine adenosyltransferase 2 subunit beta OS=Mus musculus OX=10090 GN=Mat2b PE=1 SV=1	0.883333333
Sparc	P07214	SPARC OS=Mus musculus OX=10090 GN=Sparc PE=1 SV=1	0.85
Rps6	P62754	40S ribosomal protein S6 OS=Mus musculus OX=10090 GN=Rps6 PE=1 SV=1	0.91
Hsd17b10	O08756	3-hydroxyacyl-CoA dehydrogenase type-2 OS=Mus musculus OX=10090 GN=Hsd17b10 PE=1 SV=4	0.8
Tsfm	Q9CZR8	Elongation factor Ts mitochondrial OS=Mus musculus OX=10090 GN=Tsfm PE=1 SV=1	0.86
Atp5po	Q9DB20	ATP synthase subunit O mitochondrial OS=Mus musculus OX=10090 GN=Atp5po PE=1 SV=1	0.91
Aldh7a1	Q9DBF1	Alpha-aminoadipic semialdehyde dehydrogenase OS=Mus musculus OX=10090 GN=Aldh7a1 PE=1 S	0.86
Rpl27	P61358	60S ribosomal protein L27 OS=Mus musculus OX=10090 GN=Rpl27 PE=1 SV=2	0.916666667
Galk1	Q9R0N0	Galactokinase OS=Mus musculus OX=10090 GN=Galk1 PE=1 SV=2	0.88
Snrnp40	Q6PE01	U5 small nuclear ribonucleoprotein 40 kDa protein OS=Mus musculus OX=10090 GN=Snrnp40 PE=1	0.86
Eif3j1	Q3UGC7	Eukaryotic translation initiation factor 3 subunit J-A OS=Mus musculus OX=10090 GN=Eif3j1 PE=2 S	0.933333333
Eif3j2	Q66JS6	Eukaryotic translation initiation factor 3 subunit J-B OS=Mus musculus OX=10090 GN=Eif3j2 PE=1 S	0.933333333
Tpd52l2	Q9CYZ2	Tumor protein D54 OS=Mus musculus OX=10090 GN=Tpd52l2 PE=1 SV=1	0.87
Hmox2	O70252	Heme oxygenase 2 OS=Mus musculus OX=10090 GN=Hmox2 PE=1 SV=1	0.923333333
Wdr26	Q8C6G8	WD repeat-containing protein 26 OS=Mus musculus OX=10090 GN=Wdr26 PE=1 SV=3	0.863333333
Pcyox1	Q9CQF9	Prenylcysteine oxidase OS=Mus musculus OX=10090 GN=Pcyox1 PE=1 SV=1	0.85
Sel1l	Q9Z2G6	Protein sel-1 homolog 1 OS=Mus musculus OX=10090 GN=Sel1l PE=1 SV=2	0.833333333

Nup43	P59235	Nucleoporin Nup43 OS=Mus musculus OX=10090 GN=Nup43 PE=1 SV=2	0.876666667
Glyr1	Q922P9	Putative oxidoreductase GLYR1 OS=Mus musculus OX=10090 GN=Glyr1 PE=1 SV=1	0.873333333
Pus1	Q9WU56	tRNA pseudouridine synthase A OS=Mus musculus OX=10090 GN=Pus1 PE=1 SV=2	0.893333333
Myo7a	P97479	Unconventional myosin-VIIa OS=Mus musculus OX=10090 GN=Myo7a PE=1 SV=2	1.016666667
Pdk3	Q922H2	[Pyruvate dehydrogenase (acetyl-transferring)] kinase isozyme 3 mitochondrial OS=Mus musculus O	1.006666667
Acap2	Q6ZQK5	Arf-GAP with coiled-coil ANK repeat and PH domain-containing protein 2 OS=Mus musculus OX=10	0.796666667
Tmlhe	Q91ZE0	Trimethyllysine dioxygenase mitochondrial OS=Mus musculus OX=10090 GN=Tmlhe PE=1 SV=2	0.936666667
Pgls	Q9CQ60	6-phosphogluconolactonase OS=Mus musculus OX=10090 GN=Pgls PE=1 SV=1	0.86
Rdh11	Q9QYF1	Retinol dehydrogenase 11 OS=Mus musculus OX=10090 GN=Rdh11 PE=1 SV=2	0.896666667
Ddx24	Q9ESV0	ATP-dependent RNA helicase DDX24 OS=Mus musculus OX=10090 GN=Ddx24 PE=1 SV=2	0.923333333
Tm9sf3	Q9ET30	Transmembrane 9 superfamily member 3 OS=Mus musculus OX=10090 GN=Tm9sf3 PE=1 SV=1	0.86
Aldh9a1	Q9JLJ2	4-trimethylaminobutyraldehyde dehydrogenase OS=Mus musculus OX=10090 GN=Aldh9a1 PE=1 SV	0.836666667
Srsf1	Q6PDM2	Serine/arginine-rich splicing factor 1 OS=Mus musculus OX=10090 GN=Srsf1 PE=1 SV=3	0.886666667
Vps50	Q8CI71	Syndetin OS=Mus musculus OX=10090 GN=Vps50 PE=1 SV=2	0.866666667
Ppp1r9b	Q6R891	Neurabin-2 OS=Mus musculus OX=10090 GN=Ppp1r9b PE=1 SV=1	0.936666667
Yme1l1	O88967	ATP-dependent zinc metalloprotease YME1L1 OS=Mus musculus OX=10090 GN=Yme1l1 PE=1 SV=1	0.913333333
Nolc1	E9Q5C9	Nucleolar and coiled-body phosphoprotein 1 OS=Mus musculus OX=10090 GN=Nolc1 PE=1 SV=1	0.873333333
Rpl19	P84099	60S ribosomal protein L19 OS=Mus musculus OX=10090 GN=Rpl19 PE=1 SV=1	0.9
Atg4b	Q8BGE6	Cysteine protease ATG4B OS=Mus musculus OX=10090 GN=Atg4b PE=1 SV=2	0.873333333
Sgta	Q8BJU0	Small glutamine-rich tetratricopeptide repeat-containing protein alpha OS=Mus musculus OX=1009	0.913333333
Col5a2	Q3U962	Collagen alpha-2(V) chain OS=Mus musculus OX=10090 GN=Col5a2 PE=1 SV=1	0.763333333
Taldo1	Q93092	Transaldolase OS=Mus musculus OX=10090 GN=Taldo1 PE=1 SV=2	0.833333333
Pum2	Q80U58	Pumilio homolog 2 OS=Mus musculus OX=10090 GN=Pum2 PE=1 SV=2	0.963333333
Erbin	Q80TH2	Erbin OS=Mus musculus OX=10090 GN=Erbin PE=1 SV=3	0.803333333
Wbp11	Q923D5	WW domain-binding protein 11 OS=Mus musculus OX=10090 GN=Wbp11 PE=1 SV=2	1.006666667
Akap8	Q9DBR0	A-kinase anchor protein 8 OS=Mus musculus OX=10090 GN=Akap8 PE=1 SV=1	0.983333333
Tkfc	Q8VC30	Triokinase/FMN cyclase OS=Mus musculus OX=10090 GN=Tkfc PE=1 SV=1	0.84
Cnot9	Q9JKY0	CCR4-NOT transcription complex subunit 9 OS=Mus musculus OX=10090 GN=Cnot9 PE=1 SV=1	1.013333333
Psma3	O70435	Proteasome subunit alpha type-3 OS=Mus musculus OX=10090 GN=Psma3 PE=1 SV=3	0.916666667
Nt5c2	Q3V1L4	Cytosolic purine 5'-nucleotidase OS=Mus musculus OX=10090 GN=Nt5c2 PE=1 SV=2	0.906666667
Timm44	O35857	Mitochondrial import inner membrane translocase subunit TIM44 OS=Mus musculus OX=10090 GN	0.913333333
Vps36	Q91XD6	Vacuolar protein-sorting-associated protein 36 OS=Mus musculus OX=10090 GN=Vps36 PE=1 SV=1	0.91
Manf	Q9CXI5	Mesencephalic astrocyte-derived neurotrophic factor OS=Mus musculus OX=10090 GN=Manf PE=1 S	0.86
Eml4	Q3UMY5	Echinoderm microtubule-associated protein-like 4 OS=Mus musculus OX=10090 GN=Eml4 PE=1 SV=	0.853333333

Gpc1	Q9QZF2	Glypican-1 OS=Mus musculus OX=10090 GN=Gpc1 PE=1 SV=1	0.823333333
Naa35	Q6PHQ8	N-alpha-acetyltransferase 35 NatC auxiliary subunit OS=Mus musculus OX=10090 GN=Naa35 PE=1 S	0.926666667
Ndc1	Q8VCB1	Nucleoporin NDC1 OS=Mus musculus OX=10090 GN=Ndc1 PE=1 SV=1	0.913333333
S100a4	P07091	Protein S100-A4 OS=Mus musculus OX=10090 GN=S100a4 PE=1 SV=1	0.86
Scyl2	Q8CFE4	SCY1-like protein 2 OS=Mus musculus OX=10090 GN=Scyl2 PE=1 SV=1	0.91
Cyc1	Q9D0M3	Cytochrome c1 heme protein mitochondrial OS=Mus musculus OX=10090 GN=Cyc1 PE=1 SV=1	0.803333333
Tpx2	A2APB8	Targeting protein for Xklp2 OS=Mus musculus OX=10090 GN=Tpx2 PE=1 SV=1	0.826666667
Eif2b2	Q99LD9	Translation initiation factor eIF-2B subunit beta OS=Mus musculus OX=10090 GN=Eif2b2 PE=1 SV=1	0.886666667
Msrb3	Q8BU85	Methionine-R-sulfoxide reductase B3 mitochondrial OS=Mus musculus OX=10090 GN=Msrb3 PE=1	0.923333333
Cox4i1	P19783	Cytochrome c oxidase subunit 4 isoform 1 mitochondrial OS=Mus musculus OX=10090 GN=Cox4i1	0.953333333
Lmf2	Q8C3X8	Lipase maturation factor 2 OS=Mus musculus OX=10090 GN=Lmf2 PE=1 SV=1	0.856666667
Idi1	P58044	Isopentenyl-diphosphate Delta-isomerase 1 OS=Mus musculus OX=10090 GN=Idi1 PE=1 SV=1	0.866666667
Bcas2	Q9D287	Pre-mRNA-splicing factor SPF27 OS=Mus musculus OX=10090 GN=Bcas2 PE=1 SV=1	0.913333333
Fam114a2	Q8VE88	Protein FAM114A2 OS=Mus musculus OX=10090 GN=Fam114a2 PE=1 SV=2	0.93
Bgn	P28653	Biglycan OS=Mus musculus OX=10090 GN=Bgn PE=1 SV=1	0.973333333
Map4k5	Q8BPM2	Mitogen-activated protein kinase kinase kinase kinase 5 OS=Mus musculus OX=10090 GN=Map4k5 P	0.793333333
Dap3	Q9ER88	28S ribosomal protein S29 mitochondrial OS=Mus musculus OX=10090 GN=Dap3 PE=1 SV=1	0.873333333
Vta1	Q9CR26	Vacuolar protein sorting-associated protein VTA1 homolog OS=Mus musculus OX=10090 GN=Vta1 P	0.89
Rpl11	Q9CXW4	60S ribosomal protein L11 OS=Mus musculus OX=10090 GN=Rpl11 PE=1 SV=4	0.923333333
Ccn1	P18406	CCN family member 1 OS=Mus musculus OX=10090 GN=Ccn1 PE=1 SV=1	0.936666667
Prkag1	O54950	5'-AMP-activated protein kinase subunit gamma-1 OS=Mus musculus OX=10090 GN=Prkag1 PE=1 SV	0.876666667
Nedd1	P33215	Protein NEDD1 OS=Mus musculus OX=10090 GN=Nedd1 PE=1 SV=2	0.876666667
Sqor	Q9R112	Sulfide:quinone oxidoreductase mitochondrial OS=Mus musculus OX=10090 GN=Sqor PE=1 SV=3	0.996666667
Fech	P22315	Ferrochelatase mitochondrial OS=Mus musculus OX=10090 GN=Fech PE=1 SV=2	0.933333333
Nucb2	P81117	Nucleobindin-2 OS=Mus musculus OX=10090 GN=Nucb2 PE=1 SV=2	1.013333333
Rpl18	P35980	60S ribosomal protein L18 OS=Mus musculus OX=10090 GN=Rpl18 PE=1 SV=3	0.91
Snrpa1	P57784	U2 small nuclear ribonucleoprotein A' OS=Mus musculus OX=10090 GN=Snrpa1 PE=1 SV=2	0.896666667
Exoc1	Q8R3S6	Exocyst complex component 1 OS=Mus musculus OX=10090 GN=Exoc1 PE=1 SV=4	0.963333333
Ppp1r21	Q3TDD9	Protein phosphatase 1 regulatory subunit 21 OS=Mus musculus OX=10090 GN=Ppp1r21 PE=1 SV=2	0.903333333
Trim32	Q8CH72	E3 ubiquitin-protein ligase TRIM32 OS=Mus musculus OX=10090 GN=Trim32 PE=1 SV=2	1.11
Zc3hc1	Q80YV2	Nuclear-interacting partner of ALK OS=Mus musculus OX=10090 GN=Zc3hc1 PE=1 SV=1	0.92
Slc25a11	Q9CR62	Mitochondrial 2-oxoglutarate/malate carrier protein OS=Mus musculus OX=10090 GN=Slc25a11 PE	0.813333333
Rpl18a	P62717	60S ribosomal protein L18a OS=Mus musculus OX=10090 GN=Rpl18a PE=1 SV=1	0.906666667
Ppme1	Q8BVQ5	Protein phosphatase methylesterase 1 OS=Mus musculus OX=10090 GN=Ppme1 PE=1 SV=5	0.886666667

EEF1AKNMT	Q91YR5	eEF1A lysine and N-terminal methyltransferase OS=Mus musculus OX=10090 GN=EEF1AKNMT PE=1 S	1.006666667
Plod2	Q9R0B9	Procollagen-lysine 2-oxoglutarate 5-dioxygenase 2 OS=Mus musculus OX=10090 GN=Plod2 PE=1 SV	0.96
Atp5f1c	Q91VR2	ATP synthase subunit gamma mitochondrial OS=Mus musculus OX=10090 GN=Atp5f1c PE=1 SV=1	0.99
Il6st	Q00560	Interleukin-6 receptor subunit beta OS=Mus musculus OX=10090 GN=Il6st PE=1 SV=2	0.746666667
Impa1	O55023	Inositol monophosphatase 1 OS=Mus musculus OX=10090 GN=Impa1 PE=1 SV=1	0.903333333
Hccs	P53702	Cytochrome c-type heme lyase OS=Mus musculus OX=10090 GN=Hccs PE=1 SV=2	0.8
Peak1	Q69Z38	Inactive tyrosine-protein kinase PEAK1 OS=Mus musculus OX=10090 GN=Peak1 PE=1 SV=4	0.983333333
Hgs	Q99LI8	Hepatocyte growth factor-regulated tyrosine kinase substrate OS=Mus musculus OX=10090 GN=Hgs	0.976666667
Ptpa	P58389	Serine/threonine-protein phosphatase 2A activator OS=Mus musculus OX=10090 GN=Ptpa PE=1 SV=	0.806666667
Pxn	Q8VI36	Paxillin OS=Mus musculus OX=10090 GN=Pxn PE=1 SV=1	0.926666667
Trip13	Q3UA06	Pachytene checkpoint protein 2 homolog OS=Mus musculus OX=10090 GN=Trip13 PE=1 SV=1	0.85
Psph	Q99LS3	Phosphoserine phosphatase OS=Mus musculus OX=10090 GN=Psph PE=1 SV=1	0.896666667
Ric8a	Q3TIR3	Synembryn-A OS=Mus musculus OX=10090 GN=Ric8a PE=1 SV=2	1.116666667
Chaf1a	Q9QWF0	Chromatin assembly factor 1 subunit A OS=Mus musculus OX=10090 GN=Chaf1a PE=1 SV=1	0.966666667
Crtap	Q9CYD3	Cartilage-associated protein OS=Mus musculus OX=10090 GN=Crtap PE=1 SV=3	0.933333333
Srbd1	Q497V5	S1 RNA-binding domain-containing protein 1 OS=Mus musculus OX=10090 GN=Srbd1 PE=2 SV=3	1.156666667
Dnajc3	Q91YW3	DnaJ homolog subfamily C member 3 OS=Mus musculus OX=10090 GN=Dnajc3 PE=1 SV=1	0.876666667
Npr3	P70180	Atrial natriuretic peptide receptor 3 OS=Mus musculus OX=10090 GN=Npr3 PE=1 SV=3	0.9
Nle1	Q8VEJ4	Notchless protein homolog 1 OS=Mus musculus OX=10090 GN=Nle1 PE=1 SV=4	0.8
Rpl13	P47963	60S ribosomal protein L13 OS=Mus musculus OX=10090 GN=Rpl13 PE=1 SV=3	0.873333333
Cpt2	P52825	Carnitine O-palmitoyltransferase 2 mitochondrial OS=Mus musculus OX=10090 GN=Cpt2 PE=1 SV=:	0.99
Grb2	Q60631	Growth factor receptor-bound protein 2 OS=Mus musculus OX=10090 GN=Grb2 PE=1 SV=1	0.94
Msto1	Q2YDW2	Protein misato homolog 1 OS=Mus musculus OX=10090 GN=Msto1 PE=1 SV=1	1.07
Wdr4	Q9EP82	tRNA (guanine-N(7)-)-methyltransferase non-catalytic subunit WDR4 OS=Mus musculus OX=10090 (	1.08
Exoc7	O35250	Exocyst complex component 7 OS=Mus musculus OX=10090 GN=Exoc7 PE=1 SV=2	0.853333333
Ddx56	Q9D0R4	Probable ATP-dependent RNA helicase DDX56 OS=Mus musculus OX=10090 GN=Ddx56 PE=2 SV=1	0.833333333
Xrn1	P97789	5'-3' exoribonuclease 1 OS=Mus musculus OX=10090 GN=Xrn1 PE=1 SV=1	0.866666667
Nif3l1	Q9EQ80	NIF3-like protein 1 OS=Mus musculus OX=10090 GN=Nif3l1 PE=1 SV=4	0.86
Uchl5	Q9WUP7	Ubiquitin carboxyl-terminal hydrolase isozyme L5 OS=Mus musculus OX=10090 GN=Uchl5 PE=1 SV=	0.903333333
Tfip11	Q9ERA6	Tuftelin-interacting protein 11 OS=Mus musculus OX=10090 GN=Tfip11 PE=1 SV=1	0.813333333
Chp1	P61022	Calcineurin B homologous protein 1 OS=Mus musculus OX=10090 GN=Chp1 PE=1 SV=2	0.9
Dok1	P97465	Docking protein 1 OS=Mus musculus OX=10090 GN=Dok1 PE=1 SV=2	0.903333333
Tom1	O88746	Target of Myb protein 1 OS=Mus musculus OX=10090 GN=Tom1 PE=1 SV=1	0.783333333
Scpep1	Q920A5	Retinoid-inducible serine carboxypeptidase OS=Mus musculus OX=10090 GN=Scpep1 PE=1 SV=2	0.873333333

Rps18	P62270	40S ribosomal protein S18 OS=Mus musculus OX=10090 GN=Rps18 PE=1 SV=3	0.913333333
Tk1	P04184	Thymidine kinase cytosolic OS=Mus musculus OX=10090 GN=Tk1 PE=1 SV=3	0.87
Ilkap	Q8R0F6	Integrin-linked kinase-associated serine/threonine phosphatase 2C OS=Mus musculus OX=10090 GN	0.873333333
Hbs1l	Q69ZS7	HBS1-like protein OS=Mus musculus OX=10090 GN=Hbs1l PE=1 SV=2	1.08
Hspe1	Q64433	10 kDa heat shock protein mitochondrial OS=Mus musculus OX=10090 GN=Hspe1 PE=1 SV=2	0.926666667
Eci2	Q9WUR2	Enoyl-CoA delta isomerase 2 OS=Mus musculus OX=10090 GN=Eci2 PE=1 SV=2	0.816666667
Pbk	Q9JJ78	Lymphokine-activated killer T-cell-originated protein kinase OS=Mus musculus OX=10090 GN=Pbk P	0.856666667
Cog7	Q3UM29	Conserved oligomeric Golgi complex subunit 7 OS=Mus musculus OX=10090 GN=Cog7 PE=1 SV=1	1.11
Ncapg2	Q6DFV1	Condensin-2 complex subunit G2 OS=Mus musculus OX=10090 GN=Ncapg2 PE=1 SV=2	0.736666667
Llgl1	Q80Y17	Lethal(2) giant larvae protein homolog 1 OS=Mus musculus OX=10090 GN=Llgl1 PE=1 SV=1	0.763333333
H6pd	Q8CFX1	GDH/6PGL endoplasmic bifunctional protein OS=Mus musculus OX=10090 GN=H6pd PE=1 SV=2	0.913333333
Parn	Q8VDG3	Poly(A)-specific ribonuclease PARN OS=Mus musculus OX=10090 GN=Parn PE=1 SV=1	1.013333333
Trip10	Q8CJ53	Cdc42-interacting protein 4 OS=Mus musculus OX=10090 GN=Trip10 PE=1 SV=2	0.99
Ddr2	Q62371	Discoidin domain-containing receptor 2 OS=Mus musculus OX=10090 GN=Ddr2 PE=1 SV=2	0.83
Nr2f2	P43135	COUP transcription factor 2 OS=Mus musculus OX=10090 GN=Nr2f2 PE=1 SV=2	0.853333333
Ythdf2	Q91YT7	YTH domain-containing family protein 2 OS=Mus musculus OX=10090 GN=Ythdf2 PE=1 SV=1	0.856666667
Prps1	Q9D7G0	Ribose-phosphate pyrophosphokinase 1 OS=Mus musculus OX=10090 GN=Prps1 PE=1 SV=4	0.853333333
Kpna6	O35345	Importin subunit alpha-7 OS=Mus musculus OX=10090 GN=Kpna6 PE=1 SV=2	0.973333333
Prkaca	P05132	cAMP-dependent protein kinase catalytic subunit alpha OS=Mus musculus OX=10090 GN=Prkaca PE	0.876666667
Mark2	Q05512	Serine/threonine-protein kinase MARK2 OS=Mus musculus OX=10090 GN=Mark2 PE=1 SV=3	0.946666667
Gstm2	P15626	Glutathione S-transferase Mu 2 OS=Mus musculus OX=10090 GN=Gstm2 PE=1 SV=2	0.916666667
Ehd4	Q9EQP2	EH domain-containing protein 4 OS=Mus musculus OX=10090 GN=Ehd4 PE=1 SV=1	0.85
Cstf2	Q8BIQ5	Cleavage stimulation factor subunit 2 OS=Mus musculus OX=10090 GN=Cstf2 PE=1 SV=2	0.816666667
Pycr1	Q922W5	Pyrroline-5-carboxylate reductase 1 mitochondrial OS=Mus musculus OX=10090 GN=Pycr1 PE=1 SV	0.81
Fmnl3	Q6ZPF4	Formin-like protein 3 OS=Mus musculus OX=10090 GN=Fmnl3 PE=1 SV=2	0.766666667
Bzw2	Q91VK1	Basic leucine zipper and W2 domain-containing protein 2 OS=Mus musculus OX=10090 GN=Bzw2 P	0.846666667
Camk1	Q91YS8	Calcium/calmodulin-dependent protein kinase type 1 OS=Mus musculus OX=10090 GN=Camk1 PE=	0.856666667
Gnao1	P18872	Guanine nucleotide-binding protein G(o) subunit alpha OS=Mus musculus OX=10090 GN=Gnao1 PE	0.963333333
Map1a	Q9QYR6	Microtubule-associated protein 1A OS=Mus musculus OX=10090 GN=Map1a PE=1 SV=2	1.373333333
Dlg1	Q811D0	Disks large homolog 1 OS=Mus musculus OX=10090 GN=Dlg1 PE=1 SV=1	1.02
Naa10	Q9QY36	N-alpha-acetyltransferase 10 OS=Mus musculus OX=10090 GN=Naa10 PE=1 SV=1	0.826666667
Rab10	P61027	Ras-related protein Rab-10 OS=Mus musculus OX=10090 GN=Rab10 PE=1 SV=1	0.86
Hdgfl2	Q3UMU9	Hepatoma-derived growth factor-related protein 2 OS=Mus musculus OX=10090 GN=Hdgfl2 PE=1 SV	0.846666667
Cpne3	Q8BT60	Copine-3 OS=Mus musculus OX=10090 GN=Cpne3 PE=1 SV=2	0.9

Oxsr1	Q6P9R2	Serine/threonine-protein kinase OSR1 OS=Mus musculus OX=10090 GN=Oxsr1 PE=1 SV=1	0.946666667
Smarcd2	Q99JR8	SWI/SNF-related matrix-associated actin-dependent regulator of chromatin subfamily D member 2 (	1.013333333
Rras2	P62071	Ras-related protein R-Ras2 OS=Mus musculus OX=10090 GN=Rras2 PE=1 SV=1	0.893333333
Vps4b	P46467	Vacuolar protein sorting-associated protein 4B OS=Mus musculus OX=10090 GN=Vps4b PE=1 SV=2	0.866666667
Hnrnpab	Q99020	Heterogeneous nuclear ribonucleoprotein A/B OS=Mus musculus OX=10090 GN=Hnrnpab PE=1 SV=	0.893333333
Cnn3	Q9DAW9	Calponin-3 OS=Mus musculus OX=10090 GN=Cnn3 PE=1 SV=1	0.9
Ppib	P24369	Peptidyl-prolyl cis-trans isomerase B OS=Mus musculus OX=10090 GN=Ppib PE=1 SV=2	0.89
Celf1	P28659	CUGBP Elav-like family member 1 OS=Mus musculus OX=10090 GN=Celf1 PE=1 SV=2	0.893333333
Ube2k	P61087	Ubiquitin-conjugating enzyme E2 K OS=Mus musculus OX=10090 GN=Ube2k PE=1 SV=3	0.863333333
Rrs1	Q9CYH6	Ribosome biogenesis regulatory protein homolog OS=Mus musculus OX=10090 GN=Rrs1 PE=1 SV=1	0.84
Enah	Q03173	Protein enabled homolog OS=Mus musculus OX=10090 GN=Enah PE=1 SV=2	0.913333333
Noc4l	Q8BHY2	Nucleolar complex protein 4 homolog OS=Mus musculus OX=10090 GN=Noc4l PE=2 SV=1	0.856666667
Uap1	Q91YN5	UDP-N-acetylhexosamine pyrophosphorylase OS=Mus musculus OX=10090 GN=Uap1 PE=1 SV=1	0.826666667
Apbb2	Q9DBR4	Amyloid-beta A4 precursor protein-binding family B member 2 OS=Mus musculus OX=10090 GN=Ap	0.803333333
Csk	P41241	Tyrosine-protein kinase CSK OS=Mus musculus OX=10090 GN=Csk PE=1 SV=2	0.863333333
Kif20b	Q80WE4	Kinesin-like protein KIF20B OS=Mus musculus OX=10090 GN=Kif20b PE=1 SV=3	1.143333333
Psma5	Q9Z2U1	Proteasome subunit alpha type-5 OS=Mus musculus OX=10090 GN=Psma5 PE=1 SV=1	0.883333333
Apex1	P28352	DNA-(apurinic or apyrimidinic site) endonuclease OS=Mus musculus OX=10090 GN=Apex1 PE=1 SV=	0.846666667
Set	Q9EQU5	Protein SET OS=Mus musculus OX=10090 GN=Set PE=1 SV=1	0.94
Rcc1	Q8VE37	Regulator of chromosome condensation OS=Mus musculus OX=10090 GN=Rcc1 PE=1 SV=1	0.933333333
Cope	O89079	Coatomer subunit epsilon OS=Mus musculus OX=10090 GN=Cope PE=1 SV=3	0.903333333
Bcat1	P24288	Branched-chain-amino-acid aminotransferase cytosolic OS=Mus musculus OX=10090 GN=Bcat1 PE	0.886666667
Rps14	P62264	40S ribosomal protein S14 OS=Mus musculus OX=10090 GN=Rps14 PE=1 SV=3	0.926666667
St13	Q99L47	Hsc70-interacting protein OS=Mus musculus OX=10090 GN=St13 PE=1 SV=1	0.886666667
Atxn2	O70305	Ataxin-2 OS=Mus musculus OX=10090 GN=Atxn2 PE=1 SV=1	0.95
Bcap31	Q61335	B-cell receptor-associated protein 31 OS=Mus musculus OX=10090 GN=Bcap31 PE=1 SV=4	0.89
Ptges3	Q9R0Q7	Prostaglandin E synthase 3 OS=Mus musculus OX=10090 GN=Ptges3 PE=1 SV=1	0.856666667
Ctsa	P16675	Lysosomal protective protein OS=Mus musculus OX=10090 GN=Ctsa PE=1 SV=1	0.883333333
Eif3f	Q9DCH4	Eukaryotic translation initiation factor 3 subunit F OS=Mus musculus OX=10090 GN=Eif3f PE=1 SV=:	0.873333333
Sephs1	Q8BH69	Selenide water dikinase 1 OS=Mus musculus OX=10090 GN=Sephs1 PE=1 SV=1	0.89
Opa1	P58281	Dynamin-like 120 kDa protein mitochondrial OS=Mus musculus OX=10090 GN=Opa1 PE=1 SV=1	1.063333333
Utp18	Q5SSI6	U3 small nucleolar RNA-associated protein 18 homolog OS=Mus musculus OX=10090 GN=Utp18 PE	0.866666667
Snrnp70	Q62376	U1 small nuclear ribonucleoprotein 70 kDa OS=Mus musculus OX=10090 GN=Snrnp70 PE=1 SV=2	0.906666667
Ppa2	Q91VM9	Inorganic pyrophosphatase 2 mitochondrial OS=Mus musculus OX=10090 GN=Ppa2 PE=1 SV=1	0.923333333

Rae1	Q8C570	mRNA export factor OS=Mus musculus OX=10090 GN=Rae1 PE=1 SV=1	0.87
Fhl3	Q9R059	Four and a half LIM domains protein 3 OS=Mus musculus OX=10090 GN=Fhl3 PE=1 SV=2	0.883333333
Acaa1a	Q921H8	3-ketoacyl-CoA thiolase A peroxisomal OS=Mus musculus OX=10090 GN=Acaa1a PE=1 SV=1	0.82
Faf2	Q3TDN2	FAS-associated factor 2 OS=Mus musculus OX=10090 GN=Faf2 PE=1 SV=2	0.86
Tbc1d8b	A3KGB4	TBC1 domain family member 8B OS=Mus musculus OX=10090 GN=Tbc1d8b PE=1 SV=1	0.8
Tm9sf2	P58021	Transmembrane 9 superfamily member 2 OS=Mus musculus OX=10090 GN=Tm9sf2 PE=1 SV=1	0.873333333
Cdv3	Q4VAA2	Protein CDV3 OS=Mus musculus OX=10090 GN=Cdv3 PE=1 SV=2	0.826666667
Psmb1	O09061	Proteasome subunit beta type-1 OS=Mus musculus OX=10090 GN=Psmb1 PE=1 SV=1	0.89
Wdr74	Q8VCG3	WD repeat-containing protein 74 OS=Mus musculus OX=10090 GN=Wdr74 PE=2 SV=1	0.81
Ergic1	Q9DC16	Endoplasmic reticulum-Golgi intermediate compartment protein 1 OS=Mus musculus OX=10090 G	0.876666667
Psmd4	O35226	26S proteasome non-ATPase regulatory subunit 4 OS=Mus musculus OX=10090 GN=Psmd4 PE=1 SV:	0.863333333
Brix1	Q9DCA5	Ribosome biogenesis protein BRX1 homolog OS=Mus musculus OX=10090 GN=Brix1 PE=1 SV=3	0.833333333
Nrbp1	Q99J45	Nuclear receptor-binding protein OS=Mus musculus OX=10090 GN=Nrbp1 PE=1 SV=1	0.836666667
Arhgap1	Q5FWK3	Rho GTPase-activating protein 1 OS=Mus musculus OX=10090 GN=Arhgap1 PE=1 SV=1	0.833333333
Pawr	Q925B0	PRKC apoptosis WT1 regulator protein OS=Mus musculus OX=10090 GN=Pawr PE=1 SV=2	0.89
Parp3	Q3ULW8	Protein mono-ADP-ribosyltransferase PARP3 OS=Mus musculus OX=10090 GN=Parp3 PE=2 SV=1	0.85
Hnrnpc	Q9Z204	Heterogeneous nuclear ribonucleoproteins C1/C2 OS=Mus musculus OX=10090 GN=Hnrnpc PE=1 S'	0.883333333
Nip7	Q9CXK8	60S ribosome subunit biogenesis protein NIP7 homolog OS=Mus musculus OX=10090 GN=Nip7 PE=	0.91
Dido1	Q8C9B9	Death-inducer obliterator 1 OS=Mus musculus OX=10090 GN=Dido1 PE=1 SV=4	0.686666667
Spr	Q64105	Sepiapterin reductase OS=Mus musculus OX=10090 GN=Spr PE=1 SV=1	0.843333333
Rps15a	P62245	40S ribosomal protein S15a OS=Mus musculus OX=10090 GN=Rps15a PE=1 SV=2	0.896666667
Gtf2f1	Q3THK3	General transcription factor IIF subunit 1 OS=Mus musculus OX=10090 GN=Gtf2f1 PE=1 SV=2	0.88
Slc12a2	P55012	Solute carrier family 12 member 2 OS=Mus musculus OX=10090 GN=Slc12a2 PE=1 SV=2	0.886666667
Trip6	Q9Z1Y4	Thyroid receptor-interacting protein 6 OS=Mus musculus OX=10090 GN=Trip6 PE=1 SV=1	0.94
Nub1	P54729	NEDD8 ultimate buster 1 OS=Mus musculus OX=10090 GN=Nub1 PE=1 SV=2	0.886666667
Rfc5	Q9D0F6	Replication factor C subunit 5 OS=Mus musculus OX=10090 GN=Rfc5 PE=1 SV=1	0.92
Rpl9	P51410	60S ribosomal protein L9 OS=Mus musculus OX=10090 GN=Rpl9 PE=2 SV=2	0.873333333
Pbdc1	Q9D0B6	Protein PBDC1 OS=Mus musculus OX=10090 GN=Pbdc1 PE=1 SV=1	0.896666667
Wdr46	Q9Z0H1	WD repeat-containing protein 46 OS=Mus musculus OX=10090 GN=Wdr46 PE=2 SV=1	0.85
Dnaaf5	B9EJR8	Dynein assembly factor 5 axonemal OS=Mus musculus OX=10090 GN=Dnaaf5 PE=1 SV=1	0.936666667
Lrpap1	P55302	Alpha-2-macroglobulin receptor-associated protein OS=Mus musculus OX=10090 GN=Lrpap1 PE=1	0.933333333
Pigs	Q6PD26	GPI transamidase component PIG-S OS=Mus musculus OX=10090 GN=Pigs PE=1 SV=3	0.953333333
Acsf2	Q8VCW8	Medium-chain acyl-CoA ligase ACSF2 mitochondrial OS=Mus musculus OX=10090 GN=Acsf2 PE=1 S'	0.876666667
Ppp1r8	Q8R3G1	Nuclear inhibitor of protein phosphatase 1 OS=Mus musculus OX=10090 GN=Ppp1r8 PE=1 SV=1	0.846666667

Gas2	P11862	Growth arrest-specific protein 2 OS=Mus musculus OX=10090 GN=Gas2 PE=1 SV=1	0.833333333
Mccc1	Q99MR8	Methylcrotonoyl-CoA carboxylase subunit alpha mitochondrial OS=Mus musculus OX=10090 GN=M	1.03
Ptgr1	Q91YR9	Prostaglandin reductase 1 OS=Mus musculus OX=10090 GN=Ptgr1 PE=1 SV=2	0.903333333
Lancl2	Q9JJK2	LanC-like protein 2 OS=Mus musculus OX=10090 GN=Lancl2 PE=1 SV=1	0.873333333
Nudt21	Q9CQF3	Cleavage and polyadenylation specificity factor subunit 5 OS=Mus musculus OX=10090 GN=Nudt21	0.933333333
Adprh	P54923	[Protein ADP-ribosylarginine] hydrolase OS=Mus musculus OX=10090 GN=Adprh PE=1 SV=1	0.813333333
Syap1	Q9D5V6	Synapse-associated protein 1 OS=Mus musculus OX=10090 GN=Syap1 PE=1 SV=1	0.943333333
Smyd5	Q3TYX3	SET and MYND domain-containing protein 5 OS=Mus musculus OX=10090 GN=Smyd5 PE=1 SV=2	0.863333333
Etfb	Q9DCW4	Electron transfer flavoprotein subunit beta OS=Mus musculus OX=10090 GN=Etfb PE=1 SV=3	0.87
Dhfr	P00375	Dihydrofolate reductase OS=Mus musculus OX=10090 GN=Dhfr PE=1 SV=3	0.856666667
Rpf2	Q9JJ80	Ribosome production factor 2 homolog OS=Mus musculus OX=10090 GN=Rpf2 PE=2 SV=2	0.896666667
Srprb	P47758	Signal recognition particle receptor subunit beta OS=Mus musculus OX=10090 GN=Srprb PE=1 SV=1	0.863333333
Sh3gl1	Q62419	Endophilin-A2 OS=Mus musculus OX=10090 GN=Sh3gl1 PE=1 SV=1	0.863333333
Wapl	Q65Z40	Wings apart-like protein homolog OS=Mus musculus OX=10090 GN=Wapl PE=1 SV=2	0.866666667
Vrk1	Q80X41	Serine/threonine-protein kinase VRK1 OS=Mus musculus OX=10090 GN=Vrk1 PE=1 SV=2	0.886666667
Kifbp	Q6ZPU9	KIF-binding protein OS=Mus musculus OX=10090 GN=Kifbp PE=1 SV=2	0.763333333
Zzef1	Q5SSH7	Zinc finger ZZ-type and EF-hand domain-containing protein 1 OS=Mus musculus OX=10090 GN=Zzef:	0.656666667
Rps10	P63325	40S ribosomal protein S10 OS=Mus musculus OX=10090 GN=Rps10 PE=1 SV=1	0.863333333
Thoc5	Q8BKT7	THO complex subunit 5 homolog OS=Mus musculus OX=10090 GN=Thoc5 PE=1 SV=2	0.983333333
Poldip3	Q8BG81	Polymerase delta-interacting protein 3 OS=Mus musculus OX=10090 GN=Poldip3 PE=1 SV=1	0.773333333
Hprt1	P00493	Hypoxanthine-guanine phosphoribosyltransferase OS=Mus musculus OX=10090 GN=Hprt1 PE=1 SV:	0.943333333
Pepd	Q11136	Xaa-Pro dipeptidase OS=Mus musculus OX=10090 GN=Pepd PE=1 SV=3	0.88
Wdr6	Q99ME2	WD repeat-containing protein 6 OS=Mus musculus OX=10090 GN=Wdr6 PE=1 SV=1	0.73
Txn	P10639	Thioredoxin OS=Mus musculus OX=10090 GN=Txn PE=1 SV=3	0.853333333
S100a6	P14069	Protein S100-A6 OS=Mus musculus OX=10090 GN=S100a6 PE=1 SV=3	0.95
Usp10	P52479	Ubiquitin carboxyl-terminal hydrolase 10 OS=Mus musculus OX=10090 GN=Usp10 PE=1 SV=3	0.863333333
Sltm	Q8CH25	SAFB-like transcription modulator OS=Mus musculus OX=10090 GN=Sltm PE=1 SV=1	0.85
Flot2	Q60634	Flotillin-2 OS=Mus musculus OX=10090 GN=Flot2 PE=1 SV=2	0.853333333
Igbp1	Q61249	Immunoglobulin-binding protein 1 OS=Mus musculus OX=10090 GN=Igbp1 PE=1 SV=1	0.826666667
Rpl23a	P62751	60S ribosomal protein L23a OS=Mus musculus OX=10090 GN=Rpl23a PE=1 SV=1	0.88
Pofut2	Q8VHI3	GDP-fucose protein O-fucosyltransferase 2 OS=Mus musculus OX=10090 GN=Pofut2 PE=1 SV=1	0.916666667
Acbd3	Q8BMP6	Golgi resident protein GCP60 OS=Mus musculus OX=10090 GN=Acbd3 PE=1 SV=3	0.926666667
Ak3	Q9WTP7	GTP:AMP phosphotransferase AK3 mitochondrial OS=Mus musculus OX=10090 GN=Ak3 PE=1 SV=3	0.826666667
Esf1	Q3V1V3	ESF1 homolog OS=Mus musculus OX=10090 GN=Esf1 PE=1 SV=1	0.883333333

Ubxn1	Q922Y1	UBX domain-containing protein 1 OS=Mus musculus OX=10090 GN=Ubxn1 PE=1 SV=1	0.796666667
Bysl	O54825	Bystin OS=Mus musculus OX=10090 GN=Bysl PE=1 SV=3	0.796666667
Atg7	Q9D906	Ubiquitin-like modifier-activating enzyme ATG7 OS=Mus musculus OX=10090 GN=Atg7 PE=1 SV=1	0.79
Gabpa	Q00422	GA-binding protein alpha chain OS=Mus musculus OX=10090 GN=Gabpa PE=1 SV=2	1.003333333
Prim1	P20664	DNA primase small subunit OS=Mus musculus OX=10090 GN=Prim1 PE=1 SV=1	0.913333333
Mrps22	Q9CXW2	28S ribosomal protein S22 mitochondrial OS=Mus musculus OX=10090 GN=Mrps22 PE=1 SV=1	0.923333333
Scrn2	Q8VCA8	Secernin-2 OS=Mus musculus OX=10090 GN=Scrn2 PE=1 SV=1	0.966666667
Fam160b1	Q8CDM8	Protein FAM160B1 OS=Mus musculus OX=10090 GN=Fam160b1 PE=1 SV=2	0.79
Ston1	Q8CDJ8	Stonin-1 OS=Mus musculus OX=10090 GN=Ston1 PE=1 SV=2	0.89
Ddx51	Q6P9R1	ATP-dependent RNA helicase DDX51 OS=Mus musculus OX=10090 GN=Ddx51 PE=1 SV=1	1.003333333
Mre11	Q61216	Double-strand break repair protein MRE11 OS=Mus musculus OX=10090 GN=Mre11 PE=1 SV=1	0.88
Adgrl2	Q8JZZ7	Adhesion G protein-coupled receptor L2 OS=Mus musculus OX=10090 GN=Adgrl2 PE=1 SV=3	0.98
Ints7	Q7TQK1	Integrator complex subunit 7 OS=Mus musculus OX=10090 GN=Ints7 PE=1 SV=1	1.033333333
Pik3r4	Q8VD65	Phosphoinositide 3-kinase regulatory subunit 4 OS=Mus musculus OX=10090 GN=Pik3r4 PE=1 SV=3	0.923333333
Ddx10	Q80Y44	Probable ATP-dependent RNA helicase DDX10 OS=Mus musculus OX=10090 GN=Ddx10 PE=1 SV=2	0.973333333
Mrpl19	Q9D338	39S ribosomal protein L19 mitochondrial OS=Mus musculus OX=10090 GN=Mrpl19 PE=1 SV=1	1.056666667
Rabep2	Q91WG2	Rab GTPase-binding effector protein 2 OS=Mus musculus OX=10090 GN=Rabep2 PE=1 SV=3	0.84
Ppp1r18	Q8BQ30	Phostensin OS=Mus musculus OX=10090 GN=Ppp1r18 PE=1 SV=1	0.903333333
Hist2h3c2	P84228	Histone H3.2 OS=Mus musculus OX=10090 GN=H3c2 PE=1 SV=2	
Gspt2	Q149F3	Eukaryotic peptide chain release factor GTP-binding subunit ERF3B OS=Mus musculus OX=10090 GN	
Arf2	Q8BSL7	ADP-ribosylation factor 2 OS=Mus musculus OX=10090 GN=Arf2 PE=1 SV=2	1.73
Rbmx	Q9WV02	RNA-binding motif protein X chromosome OS=Mus musculus OX=10090 GN=Rbmx PE=1 SV=1	
Rap1a	P62835	Ras-related protein Rap-1A OS=Mus musculus OX=10090 GN=Rap1a PE=1 SV=1	2.203333333
Arf5	P84084	ADP-ribosylation factor 5 OS=Mus musculus OX=10090 GN=Arf5 PE=1 SV=2	0.91
Rab2a	P53994	Ras-related protein Rab-2A OS=Mus musculus OX=10090 GN=Rab2a PE=1 SV=1	0.876666667
Cux1	P53564	Homeobox protein cut-like 1 OS=Mus musculus OX=10090 GN=Cux1 PE=1 SV=3	1.146666667
Anp32a	O35381	Acidic leucine-rich nuclear phosphoprotein 32 family member A OS=Mus musculus OX=10090 GN=/	0.886666667
Luc7l	Q9CYI4	Putative RNA-binding protein Luc7-like 1 OS=Mus musculus OX=10090 GN=Luc7l PE=1 SV=2	0.843333333
Ythdf3	Q8BYK6	YTH domain-containing family protein 3 OS=Mus musculus OX=10090 GN=Ythdf3 PE=1 SV=2	0.916666667
Ubqln1	Q8R317	Ubiquilin-1 OS=Mus musculus OX=10090 GN=Ubqln1 PE=1 SV=1	0.85
Luc7l2	Q7TNC4	Putative RNA-binding protein Luc7-like 2 OS=Mus musculus OX=10090 GN=Luc7l2 PE=1 SV=1	0.883333333
Camk2d	Q6PHZ2	Calcium/calmodulin-dependent protein kinase type II subunit delta OS=Mus musculus OX=10090 G	0.75
Cab39	Q06138	Calcium-binding protein 39 OS=Mus musculus OX=10090 GN=Cab39 PE=1 SV=2	0.826666667
Nmt2	O70311	Glycylpeptide N-tetradecanoyltransferase 2 OS=Mus musculus OX=10090 GN=Nmt2 PE=1 SV=1	0.856666667

Clasp2	Q8BRT1	CLIP-associating protein 2 OS=Mus musculus OX=10090 GN=Clasp2 PE=1 SV=1	0.863333333
Ubqln4	Q99NB8	Ubiquilin-4 OS=Mus musculus OX=10090 GN=Ubqln4 PE=1 SV=1	0.833333333
Chd1	P40201	Chromodomain-helicase-DNA-binding protein 1 OS=Mus musculus OX=10090 GN=Chd1 PE=1 SV=3	0.823333333
Pfkp	Q9WUA3	ATP-dependent 6-phosphofructokinase platelet type OS=Mus musculus OX=10090 GN=Pfkp PE=1 S\	0.833333333
Gnas	Q6R0H7	Guanine nucleotide-binding protein G(s) subunit alpha isoforms XLas OS=Mus musculus OX=10090	0.866666667
Gnas	P63094	Guanine nucleotide-binding protein G(s) subunit alpha isoforms short OS=Mus musculus OX=1009C	0.866666667
Fam49b	Q921M7	CYFIP-related Rac1 interactor B OS=Mus musculus OX=10090 GN=Cyrib PE=1 SV=1	0.936666667
Cdc42	P60766	Cell division control protein 42 homolog OS=Mus musculus OX=10090 GN=Cdc42 PE=1 SV=2	0.876666667
Abcb1b	P06795	ATP-dependent translocase ABCB1 OS=Mus musculus OX=10090 GN=Abcb1b PE=1 SV=1	0.706666667
Anxa11	P97384	Annexin A11 OS=Mus musculus OX=10090 GN=Anxa11 PE=1 SV=2	0.976666667
Dync1li2	Q6PDL0	Cytoplasmic dynein 1 light intermediate chain 2 OS=Mus musculus OX=10090 GN=Dync1li2 PE=1 S\	0.77
Arhgef25	Q9CWR0	Rho guanine nucleotide exchange factor 25 OS=Mus musculus OX=10090 GN=Arhgef25 PE=1 SV=1	1.02
Ube2n	P61089	Ubiquitin-conjugating enzyme E2 N OS=Mus musculus OX=10090 GN=Ube2n PE=1 SV=1	0.84
Casp3	P70677	Caspase-3 OS=Mus musculus OX=10090 GN=Casp3 PE=1 SV=1	0.896666667
Dnajb1	Q9QYJ3	DnaJ homolog subfamily B member 1 OS=Mus musculus OX=10090 GN=Dnajb1 PE=1 SV=3	0.743333333
Usp19	Q3UJD6	Ubiquitin carboxyl-terminal hydrolase 19 OS=Mus musculus OX=10090 GN=Usp19 PE=1 SV=1	0.813333333
Acsl1	P41216	Long-chain-fatty-acid--CoA ligase 1 OS=Mus musculus OX=10090 GN=Acsl1 PE=1 SV=2	0.886666667
Utp15	Q8C7V3	U3 small nucleolar RNA-associated protein 15 homolog OS=Mus musculus OX=10090 GN=Utp15 PE	0.996666667
Prc1	Q99K43	Protein regulator of cytokinesis 1 OS=Mus musculus OX=10090 GN=Prc1 PE=1 SV=2	0.84
Chordc1	Q9D1P4	Cysteine and histidine-rich domain-containing protein 1 OS=Mus musculus OX=10090 GN=Chordc1	0.85
Arhgdia	Q99PT1	Rho GDP-dissociation inhibitor 1 OS=Mus musculus OX=10090 GN=Arhgdia PE=1 SV=3	0.863333333
Psmd7	P26516	26S proteasome non-ATPase regulatory subunit 7 OS=Mus musculus OX=10090 GN=Psmd7 PE=1 SV	0.88
Cavin2	Q63918	Caveolae-associated protein 2 OS=Mus musculus OX=10090 GN=Cavin2 PE=1 SV=3	0.866666667
	P10404	MLV-related proviral Env polyprotein OS=Mus musculus OX=10090 PE=1 SV=3	0.856666667
Gstp1	P19157	Glutathione S-transferase P 1 OS=Mus musculus OX=10090 GN=Gstp1 PE=1 SV=2	0.843333333
Dld	O08749	Dihydrolipoyl dehydrogenase mitochondrial OS=Mus musculus OX=10090 GN=Dld PE=1 SV=2	0.916666667
Erp44	Q9D1Q6	Endoplasmic reticulum resident protein 44 OS=Mus musculus OX=10090 GN=Erp44 PE=1 SV=1	0.91
Lrrc59	Q922Q8	Leucine-rich repeat-containing protein 59 OS=Mus musculus OX=10090 GN=Lrrc59 PE=1 SV=1	0.903333333
Tomm40	Q9QYA2	Mitochondrial import receptor subunit TOM40 homolog OS=Mus musculus OX=10090 GN=Tomm4	0.856666667
Rpl17	Q9CPR4	60S ribosomal protein L17 OS=Mus musculus OX=10090 GN=Rpl17 PE=1 SV=3	0.916666667
Tyms	P07607	Thymidylate synthase OS=Mus musculus OX=10090 GN=Tyms PE=1 SV=1	0.933333333
Usp39	Q3TIX9	U4/U6.U5 tri-snRNP-associated protein 2 OS=Mus musculus OX=10090 GN=Usp39 PE=1 SV=2	1.023333333
Vat1	Q62465	Synaptic vesicle membrane protein VAT-1 homolog OS=Mus musculus OX=10090 GN=Vat1 PE=1 SV=	0.8
Golga2	Q921M4	Golgin subfamily A member 2 OS=Mus musculus OX=10090 GN=Golga2 PE=1 SV=3	1.096666667

Nup37	Q9CWU9	Nucleoporin Nup37 OS=Mus musculus OX=10090 GN=Nup37 PE=1 SV=2	0.853333333
Nol11	Q8BJW5	Nucleolar protein 11 OS=Mus musculus OX=10090 GN=Nol11 PE=2 SV=1	0.956666667
Psmb5	O55234	Proteasome subunit beta type-5 OS=Mus musculus OX=10090 GN=Psmb5 PE=1 SV=3	0.91
Drg1	P32233	Developmentally-regulated GTP-binding protein 1 OS=Mus musculus OX=10090 GN=Drg1 PE=1 SV=	0.913333333
Tes	P47226	Testin OS=Mus musculus OX=10090 GN=Tes PE=1 SV=1	0.843333333
Sucla2	Q9Z2I9	Succinate--CoA ligase [ADP-forming] subunit beta mitochondrial OS=Mus musculus OX=10090 GN=	0.893333333
Tomm34	Q9CYG7	Mitochondrial import receptor subunit TOM34 OS=Mus musculus OX=10090 GN=Tomm34 PE=1 SV	0.843333333
Ptpn1	P35821	Tyrosine-protein phosphatase non-receptor type 1 OS=Mus musculus OX=10090 GN=Ptpn1 PE=1 SV	1.01
Cnot10	Q8BH15	CCR4-NOT transcription complex subunit 10 OS=Mus musculus OX=10090 GN=Cnot10 PE=1 SV=1	1.033333333
Trrap	Q80YV3	Transformation/transcription domain-associated protein OS=Mus musculus OX=10090 GN=Trrap P	1.066666667
Dlst	Q9D2G2	Dihydrolipoyllysine-residue succinyltransferase component of 2-oxoglutarate dehydrogenase comp	0.886666667
Dus3l	Q91XI1	tRNA-dihydrouridine(47) synthase [NAD(P)(+)]-like OS=Mus musculus OX=10090 GN=Dus3l PE=1 SV=	0.876666667
Svil	Q8K4L3	Supervillin OS=Mus musculus OX=10090 GN=Svil PE=1 SV=1	0.856666667
Add1	Q9QYC0	Alpha-adducin OS=Mus musculus OX=10090 GN=Add1 PE=1 SV=2	0.923333333
Cherp	Q8CGZ0	Calcium homeostasis endoplasmic reticulum protein OS=Mus musculus OX=10090 GN=Cherp PE=1	0.83
Tgfb1i1	Q62219	Transforming growth factor beta-1-induced transcript 1 protein OS=Mus musculus OX=10090 GN=T	0.966666667
Mrps9	Q9D7N3	28S ribosomal protein S9 mitochondrial OS=Mus musculus OX=10090 GN=Mrps9 PE=1 SV=3	0.91
Ankle2	Q6P1H6	Ankyrin repeat and LEM domain-containing protein 2 OS=Mus musculus OX=10090 GN=Ankle2 PE=	0.786666667
Ccdc22	Q9JIG7	Coiled-coil domain-containing protein 22 OS=Mus musculus OX=10090 GN=Ccdc22 PE=1 SV=1	0.92
Rps5	P97461	40S ribosomal protein S5 OS=Mus musculus OX=10090 GN=Rps5 PE=1 SV=3	0.886666667
Aldh3a2	P47740	Aldehyde dehydrogenase family 3 member A2 OS=Mus musculus OX=10090 GN=Aldh3a2 PE=1 SV=2	0.92
Dpp9	Q8BVG4	Dipeptidyl peptidase 9 OS=Mus musculus OX=10090 GN=Dpp9 PE=1 SV=2	0.883333333
Atp6v1h	Q8BVE3	V-type proton ATPase subunit H OS=Mus musculus OX=10090 GN=Atp6v1h PE=1 SV=1	0.986666667
Cdc16	Q8R349	Cell division cycle protein 16 homolog OS=Mus musculus OX=10090 GN=Cdc16 PE=1 SV=1	0.926666667
Nadk2	Q8C5H8	NAD kinase 2 mitochondrial OS=Mus musculus OX=10090 GN=Nadk2 PE=1 SV=2	0.996666667
Aatf	Q9JKX4	Protein AATF OS=Mus musculus OX=10090 GN=Aatf PE=1 SV=1	0.953333333
Stub1	Q9WUD1	STIP1 homology and U box-containing protein 1 OS=Mus musculus OX=10090 GN=Stub1 PE=1 SV=1	0.95
Prpf3	Q922U1	U4/U6 small nuclear ribonucleoprotein Prp3 OS=Mus musculus OX=10090 GN=Prpf3 PE=1 SV=1	0.836666667
Aarsd1	Q3THG9	Alanyl-tRNA editing protein Aarsd1 OS=Mus musculus OX=10090 GN=Aarsd1 PE=1 SV=2	0.82
Zmpste24	Q80W54	CAAX prenyl protease 1 homolog OS=Mus musculus OX=10090 GN=Zmpste24 PE=1 SV=2	0.96
Snx7	Q9CY18	Sorting nexin-7 OS=Mus musculus OX=10090 GN=Snx7 PE=1 SV=1	0.976666667
Mad1l1	Q9WTX8	Mitotic spindle assembly checkpoint protein MAD1 OS=Mus musculus OX=10090 GN=Mad1l1 PE=1	0.91
Raly	Q64012	RNA-binding protein Raly OS=Mus musculus OX=10090 GN=Raly PE=1 SV=3	0.826666667
Gusb	P12265	Beta-glucuronidase OS=Mus musculus OX=10090 GN=Gusb PE=1 SV=2	0.836666667

Diaph3	Q9Z207	Protein diaphanous homolog 3 OS=Mus musculus OX=10090 GN=Diaph3 PE=1 SV=1	0.89
Heatr5b	Q8C547	HEAT repeat-containing protein 5B OS=Mus musculus OX=10090 GN=Heatr5b PE=1 SV=3	0.79
Rbm19	Q8R3C6	Probable RNA-binding protein 19 OS=Mus musculus OX=10090 GN=Rbm19 PE=1 SV=1	0.916666667
Noc2l	Q9WV70	Nucleolar complex protein 2 homolog OS=Mus musculus OX=10090 GN=Noc2l PE=1 SV=2	0.866666667
Ap3m1	Q9JKC8	AP-3 complex subunit mu-1 OS=Mus musculus OX=10090 GN=Ap3m1 PE=1 SV=1	0.856666667
Plbd2	Q3TCN2	Putative phospholipase B-like 2 OS=Mus musculus OX=10090 GN=Plbd2 PE=1 SV=2	0.853333333
Gtf3c1	Q8K284	General transcription factor 3C polypeptide 1 OS=Mus musculus OX=10090 GN=Gtf3c1 PE=1 SV=2	0.786666667
Sntb2	Q61235	Beta-2-syntrophin OS=Mus musculus OX=10090 GN=Sntb2 PE=1 SV=2	0.76
Syne3	Q4FZC9	Nesprin-3 OS=Mus musculus OX=10090 GN=Syne3 PE=1 SV=1	0.916666667
Glo1	Q9CPU0	Lactoylglutathione lyase OS=Mus musculus OX=10090 GN=Glo1 PE=1 SV=3	0.876666667
Smg1	Q8BKX6	Serine/threonine-protein kinase SMG1 OS=Mus musculus OX=10090 GN=Smg1 PE=1 SV=3	0.923333333
Stau1	Q9Z108	Double-stranded RNA-binding protein Staufen homolog 1 OS=Mus musculus OX=10090 GN=Stau1 P	0.923333333
Dek	Q7TNV0	Protein DEK OS=Mus musculus OX=10090 GN=Dek PE=1 SV=1	0.873333333
Cwf19l1	Q8CI33	CWF19-like protein 1 OS=Mus musculus OX=10090 GN=Cwf19l1 PE=1 SV=2	1.04
Trmt1l	A2RSY6	TRMT1-like protein OS=Mus musculus OX=10090 GN=Trmt1l PE=1 SV=1	1.046666667
Dhx30	Q99PU8	ATP-dependent RNA helicase DHX30 OS=Mus musculus OX=10090 GN=Dhx30 PE=1 SV=1	1.073333333
Srgap2	Q91Z67	SLIT-ROBO Rho GTPase-activating protein 2 OS=Mus musculus OX=10090 GN=Srgap2 PE=1 SV=2	0.986666667
Prmt7	Q922X9	Protein arginine N-methyltransferase 7 OS=Mus musculus OX=10090 GN=Prmt7 PE=1 SV=1	1.056666667
Elp3	Q9CZX0	Elongator complex protein 3 OS=Mus musculus OX=10090 GN=Elp3 PE=1 SV=1	0.926666667
Letm1	Q9Z2I0	Mitochondrial proton/calcium exchanger protein OS=Mus musculus OX=10090 GN=Letm1 PE=1 SV	0.876666667
Crnkl1	P63154	Crooked neck-like protein 1 OS=Mus musculus OX=10090 GN=Crnkl1 PE=1 SV=1	0.9
Gphn	Q8BUV3	Gephyrin OS=Mus musculus OX=10090 GN=Gphn PE=1 SV=2	0.92
Ppwd1	Q8CEC6	Peptidylprolyl isomerase domain and WD repeat-containing protein 1 OS=Mus musculus OX=1009(	1.103333333
Upf2	A2AT37	Regulator of nonsense transcripts 2 OS=Mus musculus OX=10090 GN=Upf2 PE=1 SV=1	0.93
Tbrg4	Q91YM4	FAST kinase domain-containing protein 4 OS=Mus musculus OX=10090 GN=Tbrg4 PE=1 SV=1	0.873333333
Dtymk	P97930	Thymidylate kinase OS=Mus musculus OX=10090 GN=Dtymk PE=1 SV=2	0.876666667
Rps16	P14131	40S ribosomal protein S16 OS=Mus musculus OX=10090 GN=Rps16 PE=1 SV=4	0.923333333
Col3a1	P08121	Collagen alpha-1(III) chain OS=Mus musculus OX=10090 GN=Col3a1 PE=1 SV=4	0.853333333
Ada	P03958	Adenosine deaminase OS=Mus musculus OX=10090 GN=Ada PE=1 SV=3	0.893333333
Galnt7	Q80VA0	N-acetylgalactosaminyltransferase 7 OS=Mus musculus OX=10090 GN=Galnt7 PE=1 SV=2	0.846666667
Mlkl	Q9D2Y4	Mixed lineage kinase domain-like protein OS=Mus musculus OX=10090 GN=Mlkl PE=1 SV=1	0.79
Arf1	P84078	ADP-ribosylation factor 1 OS=Mus musculus OX=10090 GN=Arf1 PE=1 SV=2	0.873333333
Smarca1	Q6PGB8	Probable global transcription activator SNF2L1 OS=Mus musculus OX=10090 GN=Smarca1 PE=1 SV=	0
Myl9	Q9CQ19	Myosin regulatory light polypeptide 9 OS=Mus musculus OX=10090 GN=Myl9 PE=1 SV=3	0.903333333

Rps27a	P62983	Ubiquitin-40S ribosomal protein S27a OS=Mus musculus OX=10090 GN=Rps27a PE=1 SV=2	0.873333333
Mnda	P0DOV1	Interferon-activable protein 205-B OS=Mus musculus OX=10090 GN=Mnda PE=1 SV=1	0.87
Gnb1	P62874	Guanine nucleotide-binding protein G(I)/G(S)/G(T) subunit beta-1 OS=Mus musculus OX=10090 GN=	0.95
Gnai3	Q9DC51	Guanine nucleotide-binding protein G(i) subunit alpha OS=Mus musculus OX=10090 GN=Gnai3 PE=	0.886666667
Rab1A	P62821	Ras-related protein Rab-1A OS=Mus musculus OX=10090 GN=Rab1A PE=1 SV=3	0.89
Camk2g	Q923T9	Calcium/calmodulin-dependent protein kinase type II subunit gamma OS=Mus musculus OX=1009C	0.856666667
Eif4g3	Q80XI3	Eukaryotic translation initiation factor 4 gamma 3 OS=Mus musculus OX=10090 GN=Eif4g3 PE=1 SV	1.29
Ywhag	P61982	14-3-3 protein gamma OS=Mus musculus OX=10090 GN=Ywhag PE=1 SV=2	0.93
Src	P05480	Neuronal proto-oncogene tyrosine-protein kinase Src OS=Mus musculus OX=10090 GN=Src PE=1 SV	0.866666667
Tbl1x	Q9QXE7	F-box-like/WD repeat-containing protein TBL1X OS=Mus musculus OX=10090 GN=Tbl1x PE=1 SV=2	0.836666667
Sfxn1	Q99JR1	Sideroflexin-1 OS=Mus musculus OX=10090 GN=Sfxn1 PE=1 SV=3	0.87
Kpna3	O35344	Importin subunit alpha-4 OS=Mus musculus OX=10090 GN=Kpna3 PE=1 SV=1	0.873333333
Rad23b	P54728	UV excision repair protein RAD23 homolog B OS=Mus musculus OX=10090 GN=Rad23b PE=1 SV=2	0.843333333
Septin10	Q8C650	Septin-10 OS=Mus musculus OX=10090 GN=Septin10 PE=1 SV=1	1.083333333
Rock1	P70335	Rho-associated protein kinase 1 OS=Mus musculus OX=10090 GN=Rock1 PE=1 SV=1	0.883333333
Fxr2	Q9WVR4	Fragile X mental retardation syndrome-related protein 2 OS=Mus musculus OX=10090 GN=Fxr2 PE=	0.923333333
Nek7	Q9ES74	Serine/threonine-protein kinase Nek7 OS=Mus musculus OX=10090 GN=Nek7 PE=1 SV=1	0.85
Stag1	Q9D3E6	Cohesin subunit SA-1 OS=Mus musculus OX=10090 GN=Stag1 PE=1 SV=3	1.576666667
Stk24	Q99KH8	Serine/threonine-protein kinase 24 OS=Mus musculus OX=10090 GN=Stk24 PE=1 SV=1	0.98
Hnrnpd	Q60668	Heterogeneous nuclear ribonucleoprotein D0 OS=Mus musculus OX=10090 GN=Hnrnpd PE=1 SV=2	0.86
Hnrnpdl	Q9Z130	Heterogeneous nuclear ribonucleoprotein D-like OS=Mus musculus OX=10090 GN=Hnrnpdl PE=1 S\	0.9
Srpk1	O70551	SRSF protein kinase 1 OS=Mus musculus OX=10090 GN=Srpk1 PE=1 SV=2	0.906666667
Ppm1a	P49443	Protein phosphatase 1A OS=Mus musculus OX=10090 GN=Ppm1a PE=1 SV=1	0.793333333
Nap1l4	Q78ZA7	Nucleosome assembly protein 1-like 4 OS=Mus musculus OX=10090 GN=Nap1l4 PE=1 SV=1	0.796666667
Rab14	Q91V41	Ras-related protein Rab-14 OS=Mus musculus OX=10090 GN=Rab14 PE=1 SV=3	0.873333333
Cpne1	Q8C166	Copine-1 OS=Mus musculus OX=10090 GN=Cpne1 PE=1 SV=1	0.913333333
Nab2	Q61127	NGFI-A-binding protein 2 OS=Mus musculus OX=10090 GN=Nab2 PE=1 SV=2	0.693333333
Akr1a1	Q9JII6	Aldo-keto reductase family 1 member A1 OS=Mus musculus OX=10090 GN=Akr1a1 PE=1 SV=3	0.91
Rab18	P35293	Ras-related protein Rab-18 OS=Mus musculus OX=10090 GN=Rab18 PE=1 SV=2	0.89
Coro1c	Q9WUM4	Coronin-1C OS=Mus musculus OX=10090 GN=Coro1c PE=1 SV=2	0.893333333
Snx6	Q6P8X1	Sorting nexin-6 OS=Mus musculus OX=10090 GN=Snx6 PE=1 SV=2	0.796666667
Arfgap3	Q9D8S3	ADP-ribosylation factor GTPase-activating protein 3 OS=Mus musculus OX=10090 GN=Arfgap3 PE=1	0.923333333
Taok1	Q5F2E8	Serine/threonine-protein kinase TAO1 OS=Mus musculus OX=10090 GN=Taok1 PE=1 SV=1	0.993333333
Prrc2b	Q7TPM1	Protein PRRC2B OS=Mus musculus OX=10090 GN=Prrc2b PE=1 SV=1	0.8

Mapk14	P47811	Mitogen-activated protein kinase 14 OS=Mus musculus OX=10090 GN=Mapk14 PE=1 SV=3	0.903333333
Ncor2	Q9WU42	Nuclear receptor corepressor 2 OS=Mus musculus OX=10090 GN=Ncor2 PE=1 SV=3	0.863333333
Dync1i2	O88487	Cytoplasmic dynein 1 intermediate chain 2 OS=Mus musculus OX=10090 GN=Dync1i2 PE=1 SV=1	0.826666667
Tardbp	Q921F2	TAR DNA-binding protein 43 OS=Mus musculus OX=10090 GN=Tardbp PE=1 SV=1	0.853333333
Dnpep	Q9Z2W0	Aspartyl aminopeptidase OS=Mus musculus OX=10090 GN=Dnpep PE=1 SV=2	0.823333333
Caprin1	Q60865	Caprin-1 OS=Mus musculus OX=10090 GN=Caprin1 PE=1 SV=2	0.84
Acat1	Q8QZT1	Acetyl-CoA acetyltransferase mitochondrial OS=Mus musculus OX=10090 GN=Acat1 PE=1 SV=1	0.856666667
Etfa	Q99LC5	Electron transfer flavoprotein subunit alpha mitochondrial OS=Mus musculus OX=10090 GN=Etfa P	0.866666667
Tmem43	Q9DBS1	Transmembrane protein 43 OS=Mus musculus OX=10090 GN=Tmem43 PE=1 SV=1	0.836666667
Psma7	Q9Z2U0	Proteasome subunit alpha type-7 OS=Mus musculus OX=10090 GN=Psma7 PE=1 SV=1	0.86
Fam91a1	Q3UVG3	Protein FAM91A1 OS=Mus musculus OX=10090 GN=Fam91a1 PE=1 SV=1	0.94
Aimp1	P31230	Aminoacyl tRNA synthase complex-interacting multifunctional protein 1 OS=Mus musculus OX=10(	0.856666667
Ranbp1	P34022	Ran-specific GTPase-activating protein OS=Mus musculus OX=10090 GN=Ranbp1 PE=1 SV=2	0.843333333
Otub1	Q7TQI3	Ubiquitin thioesterase OTUB1 OS=Mus musculus OX=10090 GN=Otub1 PE=1 SV=2	0.893333333
Myl6	Q60605	Myosin light polypeptide 6 OS=Mus musculus OX=10090 GN=Myl6 PE=1 SV=3	0.893333333
Adss2	P46664	Adenylosuccinate synthetase isozyme 2 OS=Mus musculus OX=10090 GN=Adss2 PE=1 SV=2	0.903333333
Pacsin2	Q9WVE8	Protein kinase C and casein kinase substrate in neurons protein 2 OS=Mus musculus OX=10090 GN=I	0.853333333
Ilf2	Q9CXY6	Interleukin enhancer-binding factor 2 OS=Mus musculus OX=10090 GN=Ilf2 PE=1 SV=1	0.87
Btf3	Q64152	Transcription factor BTF3 OS=Mus musculus OX=10090 GN=Btf3 PE=1 SV=3	0.843333333
Mdh1	P14152	Malate dehydrogenase cytoplasmic OS=Mus musculus OX=10090 GN=Mdh1 PE=1 SV=3	0.88
Emd	O08579	Emerin OS=Mus musculus OX=10090 GN=Emd PE=1 SV=1	0.936666667
Cops5	O35864	COP9 signalosome complex subunit 5 OS=Mus musculus OX=10090 GN=Cops5 PE=1 SV=3	0.873333333
Clint1	Q99KN9	Clathrin interactor 1 OS=Mus musculus OX=10090 GN=Clint1 PE=1 SV=2	0.856666667
Timeless	Q9R1X4	Protein timeless homolog OS=Mus musculus OX=10090 GN=Timeless PE=1 SV=3	0.93
Pcmt1	P23506	Protein-L-isoaspartate(D-aspartate) O-methyltransferase OS=Mus musculus OX=10090 GN=Pcmt1 PI	0.85
Mri1	Q9CQT1	Methylthioribose-1-phosphate isomerase OS=Mus musculus OX=10090 GN=Mri1 PE=1 SV=1	0.933333333
Nisch	Q80TM9	Nischarin OS=Mus musculus OX=10090 GN=Nisch PE=1 SV=2	1.093333333
Lims1	Q99JW4	LIM and senescent cell antigen-like-containing domain protein 1 OS=Mus musculus OX=10090 GN=I	0.856666667
Gmds	Q8K0C9	GDP-mannose 4 6 dehydratase OS=Mus musculus OX=10090 GN=Gmds PE=1 SV=1	0.916666667
Aip	O08915	AH receptor-interacting protein OS=Mus musculus OX=10090 GN=Aip PE=1 SV=1	0.88
Lemd3	Q9WU40	Inner nuclear membrane protein Man1 OS=Mus musculus OX=10090 GN=Lemd3 PE=1 SV=2	0.803333333
Mat2a	Q3THS6	S-adenosylmethionine synthase isoform type-2 OS=Mus musculus OX=10090 GN=Mat2a PE=1 SV=2	0.92
Nploc4	P60670	Nuclear protein localization protein 4 homolog OS=Mus musculus OX=10090 GN=Nploc4 PE=1 SV=3	0.826666667
Adk	P55264	Adenosine kinase OS=Mus musculus OX=10090 GN=Adk PE=1 SV=2	0.903333333

Rfc1	P35601	Replication factor C subunit 1 OS=Mus musculus OX=10090 GN=Rfc1 PE=1 SV=2	0.93
Prim2	P33610	DNA primase large subunit OS=Mus musculus OX=10090 GN=Prim2 PE=1 SV=1	0.92
Cops6	O88545	COP9 signalosome complex subunit 6 OS=Mus musculus OX=10090 GN=Cops6 PE=1 SV=1	0.94
Ctcf	Q61164	Transcriptional repressor CTCF OS=Mus musculus OX=10090 GN=Ctcf PE=1 SV=2	0.91
Pcolce	Q61398	Procollagen C-endopeptidase enhancer 1 OS=Mus musculus OX=10090 GN=Pcolce PE=1 SV=2	0.84
Tubgcp3	P58854	Gamma-tubulin complex component 3 OS=Mus musculus OX=10090 GN=Tubgcp3 PE=1 SV=2	0.876666667
Aebp1	Q640N1	Adipocyte enhancer-binding protein 1 OS=Mus musculus OX=10090 GN=Aebp1 PE=1 SV=1	0.76
Cpsf3	Q9QXK7	Cleavage and polyadenylation specificity factor subunit 3 OS=Mus musculus OX=10090 GN=Cpsf3 PI	0.983333333
Ctnnbl1	Q9CWL8	Beta-catenin-like protein 1 OS=Mus musculus OX=10090 GN=Ctnnbl1 PE=1 SV=1	0.956666667
Sf1	Q64213	Splicing factor 1 OS=Mus musculus OX=10090 GN=Sf1 PE=1 SV=6	0.75
Lbr	Q3U9G9	Delta(14)-sterol reductase LBR OS=Mus musculus OX=10090 GN=Lbr PE=1 SV=2	0.87
Actl6a	Q9Z2N8	Actin-like protein 6A OS=Mus musculus OX=10090 GN=Actl6a PE=1 SV=2	0.886666667
Fndc3a	Q8BX90	Fibronectin type-III domain-containing protein 3A OS=Mus musculus OX=10090 GN=Fndc3a PE=1 S	0.713333333
Eif2d	Q61211	Eukaryotic translation initiation factor 2D OS=Mus musculus OX=10090 GN=Eif2d PE=1 SV=3	0.81
Arhgef1	Q61210	Rho guanine nucleotide exchange factor 1 OS=Mus musculus OX=10090 GN=Arhgef1 PE=1 SV=2	0.826666667
Osbpl3	Q9DBS9	Oxysterol-binding protein-related protein 3 OS=Mus musculus OX=10090 GN=Osbpl3 PE=1 SV=2	0.88
Pspc1	Q8R326	Paraspeckle component 1 OS=Mus musculus OX=10090 GN=Pspc1 PE=1 SV=1	0.876666667
Eps15	P42567	Epidermal growth factor receptor substrate 15 OS=Mus musculus OX=10090 GN=Eps15 PE=1 SV=1	0.803333333
Pmpcb	Q9CXT8	Mitochondrial-processing peptidase subunit beta OS=Mus musculus OX=10090 GN=Pmpcb PE=1 SV	0.833333333
Col6a2	Q02788	Collagen alpha-2(VI) chain OS=Mus musculus OX=10090 GN=Col6a2 PE=1 SV=3	0.93
Gys1	Q9Z1E4	Glycogen [starch] synthase muscle OS=Mus musculus OX=10090 GN=Gys1 PE=1 SV=2	0.893333333
Atad2	Q8CDM1	ATPase family AAA domain-containing protein 2 OS=Mus musculus OX=10090 GN=Atad2 PE=1 SV=1	0.97
P3h3	Q8CG70	Prolyl 3-hydroxylase 3 OS=Mus musculus OX=10090 GN=P3h3 PE=1 SV=1	0.826666667
Carm1	Q9WVG6	Histone-arginine methyltransferase CARM1 OS=Mus musculus OX=10090 GN=Carm1 PE=1 SV=2	0.896666667
Lman2	Q9DBH5	Vesicular integral-membrane protein VIP36 OS=Mus musculus OX=10090 GN=Lman2 PE=1 SV=2	0.9
Asna1	O54984	ATPase GET3 OS=Mus musculus OX=10090 GN=Get3 PE=1 SV=2	0.876666667
Mfge8	P21956	Lactadherin OS=Mus musculus OX=10090 GN=Mfge8 PE=1 SV=3	0.896666667
Ptcd3	Q14C51	Pentatricopeptide repeat domain-containing protein 3 mitochondrial OS=Mus musculus OX=1009	0.836666667
Ngef	Q8CHT1	Ephexin-1 OS=Mus musculus OX=10090 GN=Ngef PE=1 SV=1	0.793333333
Noc3l	Q8VI84	Nucleolar complex protein 3 homolog OS=Mus musculus OX=10090 GN=Noc3l PE=2 SV=2	0.96
Ppp5c	Q60676	Serine/threonine-protein phosphatase 5 OS=Mus musculus OX=10090 GN=Ppp5c PE=1 SV=3	1.013333333
Xrcc5	P27641	X-ray repair cross-complementing protein 5 OS=Mus musculus OX=10090 GN=Xrcc5 PE=1 SV=4	0.84
Nol10	Q5RJG1	Nucleolar protein 10 OS=Mus musculus OX=10090 GN=Nol10 PE=2 SV=1	0.983333333
Gtpbp1	O08582	GTP-binding protein 1 OS=Mus musculus OX=10090 GN=Gtpbp1 PE=1 SV=2	0.973333333

Phldb1	Q6PDH0	Pleckstrin homology-like domain family B member 1 OS=Mus musculus OX=10090 GN=Phldb1 PE=1	0.82
Sec16a	E9QAT4	Protein transport protein Sec16A OS=Mus musculus OX=10090 GN=Sec16a PE=1 SV=1	0.876666667
Vcpip1	Q8CDG3	Deubiquitinating protein VCIP135 OS=Mus musculus OX=10090 GN=Vcpip1 PE=1 SV=1	0.87
Ints1	Q6P4S8	Integrator complex subunit 1 OS=Mus musculus OX=10090 GN=Ints1 PE=1 SV=2	1.096666667
Rps11	P62281	40S ribosomal protein S11 OS=Mus musculus OX=10090 GN=Rps11 PE=1 SV=3	0.926666667
Gnl1	P36916	Guanine nucleotide-binding protein-like 1 OS=Mus musculus OX=10090 GN=Gnl1 PE=1 SV=4	0.846666667
Trim47	Q8C0E3	E3 ubiquitin-protein ligase TRIM47 OS=Mus musculus OX=10090 GN=Trim47 PE=1 SV=2	1.066666667
Asl	Q91YI0	Argininosuccinate lyase OS=Mus musculus OX=10090 GN=Asl PE=1 SV=1	0.816666667
Xrcc6	P23475	X-ray repair cross-complementing protein 6 OS=Mus musculus OX=10090 GN=Xrcc6 PE=1 SV=5	0.733333333
Ddx47	Q9CWX9	Probable ATP-dependent RNA helicase DDX47 OS=Mus musculus OX=10090 GN=Ddx47 PE=2 SV=2	0.96
Ccdc93	Q7TQK5	Coiled-coil domain-containing protein 93 OS=Mus musculus OX=10090 GN=Ccdc93 PE=1 SV=1	0.883333333
Acads	Q07417	Short-chain specific acyl-CoA dehydrogenase mitochondrial OS=Mus musculus OX=10090 GN=Acac	0.91
Pold2	O35654	DNA polymerase delta subunit 2 OS=Mus musculus OX=10090 GN=Pold2 PE=1 SV=2	0.816666667
Dcaf8	Q8N7N5	DDB1- and CUL4-associated factor 8 OS=Mus musculus OX=10090 GN=Dcaf8 PE=1 SV=1	0.933333333
Zc3h11a	Q6NZF1	Zinc finger CCCH domain-containing protein 11A OS=Mus musculus OX=10090 GN=Zc3h11a PE=1 S'	0.89
Ebna1bp2	Q9D903	Probable rRNA-processing protein EBP2 OS=Mus musculus OX=10090 GN=Ebna1bp2 PE=2 SV=1	0.873333333
Pgm3	Q9CYR6	Phosphoacetylglucosamine mutase OS=Mus musculus OX=10090 GN=Pgm3 PE=1 SV=1	0.933333333
Smarce1	O54941	SWI/SNF-related matrix-associated actin-dependent regulator of chromatin subfamily E member 1 (	0.783333333
Lig3	P97386	DNA ligase 3 OS=Mus musculus OX=10090 GN=Lig3 PE=1 SV=2	0.986666667
Appl1	Q8K3H0	DCC-interacting protein 13-alpha OS=Mus musculus OX=10090 GN=Appl1 PE=1 SV=1	0.883333333
Eif2b4	Q61749	Translation initiation factor eIF-2B subunit delta OS=Mus musculus OX=10090 GN=Eif2b4 PE=1 SV=:	0.84
Eml1	Q05BC3	Echinoderm microtubule-associated protein-like 1 OS=Mus musculus OX=10090 GN=Eml1 PE=1 SV=	0.836666667
Supv3l1	Q80YD1	ATP-dependent RNA helicase SUPV3L1 mitochondrial OS=Mus musculus OX=10090 GN=Supv3l1 PE	0.816666667
Ttc27	Q8CD92	Tetratricopeptide repeat protein 27 OS=Mus musculus OX=10090 GN=Ttc27 PE=1 SV=2	0.85
Eif2b5	Q8CHW4	Translation initiation factor eIF-2B subunit epsilon OS=Mus musculus OX=10090 GN=Eif2b5 PE=1 S\	0.833333333
Sec14l1	A8Y5H7	SEC14-like protein 1 OS=Mus musculus OX=10090 GN=Sec14l1 PE=1 SV=1	1.06
Ppp2ca	P63330	Serine/threonine-protein phosphatase 2A catalytic subunit alpha isoform OS=Mus musculus OX=10	0.846666667
Ppp2cb	P62715	Serine/threonine-protein phosphatase 2A catalytic subunit beta isoform OS=Mus musculus OX=100	1.08
Acacb	E9Q4Z2	Acetyl-CoA carboxylase 2 OS=Mus musculus OX=10090 GN=Acacb PE=1 SV=1	0.593333333
Atp1a2	Q6PIE5	Sodium/potassium-transporting ATPase subunit alpha-2 OS=Mus musculus OX=10090 GN=Atp1a2 F	
Ppp1cc	P63087	Serine/threonine-protein phosphatase PP1-gamma catalytic subunit OS=Mus musculus OX=10090 (	0.943333333
Myl12b	Q3THE2	Myosin regulatory light chain 12B OS=Mus musculus OX=10090 GN=Myl12b PE=1 SV=2	0.916666667
Septin6	Q9R1T4	Septin-6 OS=Mus musculus OX=10090 GN=Septin6 PE=1 SV=4	1.103333333
Rap1b	Q99JI6	Ras-related protein Rap-1b OS=Mus musculus OX=10090 GN=Rap1b PE=1 SV=2	0.866666667

Ppp2r2d	Q925E7	Serine/threonine-protein phosphatase 2A 55 kDa regulatory subunit B delta isoform OS=Mus muscu	9.945
Hdac2	P70288	Histone deacetylase 2 OS=Mus musculus OX=10090 GN=Hdac2 PE=1 SV=1	0.853333333
Ctbp1	O88712	C-terminal-binding protein 1 OS=Mus musculus OX=10090 GN=Ctbp1 PE=1 SV=2	0.88
Brd4	Q9ESU6	Bromodomain-containing protein 4 OS=Mus musculus OX=10090 GN=Brd4 PE=1 SV=2	1.21
Ctbp2	P56546	C-terminal-binding protein 2 OS=Mus musculus OX=10090 GN=Ctbp2 PE=1 SV=2	0.896666667
Pak1	O88643	Serine/threonine-protein kinase PAK 1 OS=Mus musculus OX=10090 GN=Pak1 PE=1 SV=1	0.963333333
Ywhah	P68510	14-3-3 protein eta OS=Mus musculus OX=10090 GN=Ywhah PE=1 SV=2	0.883333333
Hmgb2	P30681	High mobility group protein B2 OS=Mus musculus OX=10090 GN=Hmgb2 PE=1 SV=3	0.81
Slc25a13	Q9QXX4	Calcium-binding mitochondrial carrier protein Aralar2 OS=Mus musculus OX=10090 GN=Slc25a13	0.946666667
Prkar2b	P31324	cAMP-dependent protein kinase type II-beta regulatory subunit OS=Mus musculus OX=10090 GN=P	0.86
Capza2	P47754	F-actin-capping protein subunit alpha-2 OS=Mus musculus OX=10090 GN=Capza2 PE=1 SV=3	0.94
Mapre1	Q61166	Microtubule-associated protein RP/EB family member 1 OS=Mus musculus OX=10090 GN=Mapre1 F	0.843333333
Efl1	Q8C0D5	Elongation factor-like GTPase 1 OS=Mus musculus OX=10090 GN=Efl1 PE=1 SV=1	0.786666667
Cdk4	P30285	Cyclin-dependent kinase 4 OS=Mus musculus OX=10090 GN=Cdk4 PE=1 SV=1	0.86
Impdh1	P50096	Inosine-5'-monophosphate dehydrogenase 1 OS=Mus musculus OX=10090 GN=Impdh1 PE=1 SV=2	0.873333333
Galnt2	Q6PB93	Polypeptide N-acetylgalactosaminyltransferase 2 OS=Mus musculus OX=10090 GN=Galnt2 PE=1 SV=	0.896666667
Sbno1	Q689Z5	Protein strawberry notch homolog 1 OS=Mus musculus OX=10090 GN=Sbno1 PE=1 SV=2	0.866666667
Csnk2a2	O54833	Casein kinase II subunit alpha' OS=Mus musculus OX=10090 GN=Csnk2a2 PE=1 SV=1	0.893333333
Trnt1	Q8K1J6	CCA tRNA nucleotidyltransferase 1 mitochondrial OS=Mus musculus OX=10090 GN=Trnt1 PE=1 SV=	0.903333333
Gatad2a	Q8CHY6	Transcriptional repressor p66 alpha OS=Mus musculus OX=10090 GN=Gatad2a PE=1 SV=2	0.883333333
Twf1	Q91YR1	Twinfilin-1 OS=Mus musculus OX=10090 GN=Twf1 PE=1 SV=2	0.883333333
Crk	Q64010	Adapter molecule crk OS=Mus musculus OX=10090 GN=Crk PE=1 SV=1	0.856666667
Papss1	Q60967	Bifunctional 3'-phosphoadenosine 5'-phosphosulfate synthase 1 OS=Mus musculus OX=10090 GN=F	0.89
Dip2b	Q3UH60	Disco-interacting protein 2 homolog B OS=Mus musculus OX=10090 GN=Dip2b PE=1 SV=1	0.916666667
Ipo8	Q7TMY7	Importin-8 OS=Mus musculus OX=10090 GN=Ipo8 PE=1 SV=3	0.936666667
Scrib	Q80U72	Protein scribble homolog OS=Mus musculus OX=10090 GN=Scrib PE=1 SV=2	0.72
Rcn3	Q8BH97	Reticulocalbin-3 OS=Mus musculus OX=10090 GN=Rcn3 PE=1 SV=1	0.92
Fhl1	P97447	Four and a half LIM domains protein 1 OS=Mus musculus OX=10090 GN=Fhl1 PE=1 SV=3	0.896666667
Plin3	Q9DBG5	Perilipin-3 OS=Mus musculus OX=10090 GN=Plin3 PE=1 SV=1	0.836666667
Fh	P97807	Fumarate hydratase mitochondrial OS=Mus musculus OX=10090 GN=Fh PE=1 SV=3	0.836666667
Dctn2	Q99KJ8	Dynactin subunit 2 OS=Mus musculus OX=10090 GN=Dctn2 PE=1 SV=3	0.86
Psma6	Q9QUM9	Proteasome subunit alpha type-6 OS=Mus musculus OX=10090 GN=Psma6 PE=1 SV=1	0.883333333
Bub3	Q9WVA3	Mitotic checkpoint protein BUB3 OS=Mus musculus OX=10090 GN=Bub3 PE=1 SV=2	0.883333333
Dbnl	Q62418	Drebrin-like protein OS=Mus musculus OX=10090 GN=Dbnl PE=1 SV=2	0.856666667

Tcea1	P10711	Transcription elongation factor A protein 1 OS=Mus musculus OX=10090 GN=Tcea1 PE=1 SV=2	0.863333333
Rps2	P25444	40S ribosomal protein S2 OS=Mus musculus OX=10090 GN=Rps2 PE=1 SV=3	0.9
Zpr1	Q62384	Zinc finger protein ZPR1 OS=Mus musculus OX=10090 GN=Zpr1 PE=1 SV=1	0.75
RO60	O08848	60 kDa SS-A/Ro ribonucleoprotein OS=Mus musculus OX=10090 GN=RO60 PE=1 SV=1	0.85
Ppm1g	Q61074	Protein phosphatase 1G OS=Mus musculus OX=10090 GN=Ppm1g PE=1 SV=3	0.99
Ncaph	Q8C156	Condensin complex subunit 2 OS=Mus musculus OX=10090 GN=Ncaph PE=1 SV=1	0.883333333
Lasp1	Q61792	LIM and SH3 domain protein 1 OS=Mus musculus OX=10090 GN=Lasp1 PE=1 SV=1	0.866666667
Nans	Q99J77	Sialic acid synthase OS=Mus musculus OX=10090 GN=Nans PE=1 SV=1	0.886666667
Flot1	O08917	Flotillin-1 OS=Mus musculus OX=10090 GN=Flot1 PE=1 SV=1	0.89
Ppp4r2	Q0VGB7	Serine/threonine-protein phosphatase 4 regulatory subunit 2 OS=Mus musculus OX=10090 GN=Ppp	0.87
Capzb	P47757	F-actin-capping protein subunit beta OS=Mus musculus OX=10090 GN=Capzb PE=1 SV=3	0.856666667
Metap1	Q8BP48	Methionine aminopeptidase 1 OS=Mus musculus OX=10090 GN=Metap1 PE=1 SV=1	0.906666667
Ilvbl	Q8BU33	2-hydroxyacyl-CoA lyase 2 OS=Mus musculus OX=10090 GN=Ilvbl PE=1 SV=1	0.886666667
Elavl1	P70372	ELAV-like protein 1 OS=Mus musculus OX=10090 GN=Elavl1 PE=1 SV=2	0.903333333
Tubg1	P83887	Tubulin gamma-1 chain OS=Mus musculus OX=10090 GN=Tubg1 PE=1 SV=1	0.903333333
Hspa14	Q99M31	Heat shock 70 kDa protein 14 OS=Mus musculus OX=10090 GN=Hspa14 PE=1 SV=2	0.9
Nxn	P97346	Nucleoredoxin OS=Mus musculus OX=10090 GN=Nxn PE=1 SV=1	0.923333333
Seh1l	Q8R2U0	Nucleoporin SEH1 OS=Mus musculus OX=10090 GN=Seh1l PE=2 SV=1	0.9
Nrp1	P97333	Neuropilin-1 OS=Mus musculus OX=10090 GN=Nrp1 PE=1 SV=2	0.876666667
Ahsa1	Q8BK64	Activator of 90 kDa heat shock protein ATPase homolog 1 OS=Mus musculus OX=10090 GN=Ahsa1 P	0.856666667
Niban1	Q3UW53	Protein Niban 1 OS=Mus musculus OX=10090 GN=Niban1 PE=1 SV=2	0.846666667
Map1s	Q8C052	Microtubule-associated protein 1S OS=Mus musculus OX=10090 GN=Map1s PE=1 SV=2	0.856666667
Ik	Q9Z1M8	Protein Red OS=Mus musculus OX=10090 GN=Ik PE=1 SV=2	0.816666667
Nup54	Q8BTS4	Nuclear pore complex protein Nup54 OS=Mus musculus OX=10090 GN=Nup54 PE=1 SV=1	0.893333333
Prkaa1	Q5EG47	5'-AMP-activated protein kinase catalytic subunit alpha-1 OS=Mus musculus OX=10090 GN=Prkaa1	0.926666667
Ylpm1	Q9R0I7	YLP motif-containing protein 1 OS=Mus musculus OX=10090 GN=Ylpm1 PE=2 SV=2	0.873333333
Phb2	O35129	Prohibitin-2 OS=Mus musculus OX=10090 GN=Phb2 PE=1 SV=1	0.916666667
Gsr	P47791	Glutathione reductase mitochondrial OS=Mus musculus OX=10090 GN=Gsr PE=1 SV=3	0.936666667
Baiap2	Q8BKX1	Brain-specific angiogenesis inhibitor 1-associated protein 2 OS=Mus musculus OX=10090 GN=Baiap	0.816666667
Mical1	Q8VDP3	[F-actin]-monooxygenase MICAL1 OS=Mus musculus OX=10090 GN=Mical1 PE=1 SV=1	0.863333333
Sgpl1	Q8R0X7	Sphingosine-1-phosphate lyase 1 OS=Mus musculus OX=10090 GN=Sgpl1 PE=1 SV=1	0.876666667
Psme1	P97371	Proteasome activator complex subunit 1 OS=Mus musculus OX=10090 GN=Psme1 PE=1 SV=2	0.846666667
Zc3h14	Q8BJ05	Zinc finger CCCH domain-containing protein 14 OS=Mus musculus OX=10090 GN=Zc3h14 PE=1 SV=	0.986666667
Glrx3	Q9CQM9	Glutaredoxin-3 OS=Mus musculus OX=10090 GN=Glrx3 PE=1 SV=1	0.896666667

Tgm2	P21981	Protein-glutamine gamma-glutamyltransferase 2 OS=Mus musculus OX=10090 GN=Tgm2 PE=1 SV=4	0.826666667
Aaas	P58742	Aladin OS=Mus musculus OX=10090 GN=Aaas PE=1 SV=1	0.866666667
Ano6	Q6P9J9	Anoctamin-6 OS=Mus musculus OX=10090 GN=Ano6 PE=1 SV=1	0.816666667
Pnpt1	Q8K1R3	Polyribonucleotide nucleotidyltransferase 1 mitochondrial OS=Mus musculus OX=10090 GN=Pnpt	0.993333333
Nr3c1	P06537	Glucocorticoid receptor OS=Mus musculus OX=10090 GN=Nr3c1 PE=1 SV=1	0.893333333
Rpl6	P47911	60S ribosomal protein L6 OS=Mus musculus OX=10090 GN=Rpl6 PE=1 SV=3	0.906666667
RTRAF	Q9CQE8	RNA transcription translation and transport factor protein OS=Mus musculus OX=10090 GN=RTRAF	0.943333333
Rfc3	Q8R323	Replication factor C subunit 3 OS=Mus musculus OX=10090 GN=Rfc3 PE=1 SV=1	0.913333333
Anapc5	Q8BTZ4	Anaphase-promoting complex subunit 5 OS=Mus musculus OX=10090 GN=Anapc5 PE=1 SV=1	0.89
Wdr18	Q4VBE8	WD repeat-containing protein 18 OS=Mus musculus OX=10090 GN=Wdr18 PE=1 SV=1	0.963333333
Ikbip	Q9DBZ1	Inhibitor of nuclear factor kappa-B kinase-interacting protein OS=Mus musculus OX=10090 GN=Ikbi	0.903333333
Arhgap35	Q91YM2	Rho GTPase-activating protein 35 OS=Mus musculus OX=10090 GN=Arhgap35 PE=1 SV=3	1.046666667
Pdia5	Q921X9	Protein disulfide-isomerase A5 OS=Mus musculus OX=10090 GN=Pdia5 PE=1 SV=1	0.783333333
Lrrc47	Q505F5	Leucine-rich repeat-containing protein 47 OS=Mus musculus OX=10090 GN=Lrrc47 PE=1 SV=1	0.886666667
Utp6	Q8VCY6	U3 small nucleolar RNA-associated protein 6 homolog OS=Mus musculus OX=10090 GN=Utp6 PE=2	0.89
Nup88	Q8CEC0	Nuclear pore complex protein Nup88 OS=Mus musculus OX=10090 GN=Nup88 PE=1 SV=1	0.88
Samm50	Q8BGH2	Sorting and assembly machinery component 50 homolog OS=Mus musculus OX=10090 GN=Samm5	0.836666667
Racgap1	Q9WVM1	Rac GTPase-activating protein 1 OS=Mus musculus OX=10090 GN=Racgap1 PE=1 SV=1	0.836666667
Cpsf2	O35218	Cleavage and polyadenylation specificity factor subunit 2 OS=Mus musculus OX=10090 GN=Cpsf2 P	0.98
Dab2	P98078	Disabled homolog 2 OS=Mus musculus OX=10090 GN=Dab2 PE=1 SV=2	0.843333333
Lama5	Q61001	Laminin subunit alpha-5 OS=Mus musculus OX=10090 GN=Lama5 PE=1 SV=4	1.02
Setd3	Q91WC0	Actin-histidine N-methyltransferase OS=Mus musculus OX=10090 GN=Setd3 PE=1 SV=1	0.81
Rbm10	Q99KG3	RNA-binding protein 10 OS=Mus musculus OX=10090 GN=Rbm10 PE=1 SV=1	0.81
Wwc2	Q6NXJ0	Protein WWC2 OS=Mus musculus OX=10090 GN=Wwc2 PE=1 SV=1	0.883333333
Larp4	Q8BWW4	La-related protein 4 OS=Mus musculus OX=10090 GN=Larp4 PE=1 SV=2	0.886666667
Plcd1	Q8R3B1	1-phosphatidylinositol 4 5-bisphosphate phosphodiesterase delta-1 OS=Mus musculus OX=10090 G	1.096666667
Ccdc6	D3YZP9	Coiled-coil domain-containing protein 6 OS=Mus musculus OX=10090 GN=Ccdc6 PE=1 SV=1	1.016666667
Srpra	Q9DBG7	Signal recognition particle receptor subunit alpha OS=Mus musculus OX=10090 GN=Srpra PE=1 SV=	0.816666667
Tmem214	Q8BM55	Transmembrane protein 214 OS=Mus musculus OX=10090 GN=Tmem214 PE=1 SV=1	0.843333333
Vac14	Q80WQ2	Protein VAC14 homolog OS=Mus musculus OX=10090 GN=Vac14 PE=1 SV=1	1.17
Gtf3c4	Q8BMQ2	General transcription factor 3C polypeptide 4 OS=Mus musculus OX=10090 GN=Gtf3c4 PE=1 SV=2	0.803333333
Fto	Q8BGW1	Alpha-ketoglutarate-dependent dioxygenase FTO OS=Mus musculus OX=10090 GN=Fto PE=1 SV=1	0.95
Rps9	Q6ZWN5	40S ribosomal protein S9 OS=Mus musculus OX=10090 GN=Rps9 PE=1 SV=3	0.903333333
Rbmxl1	Q91VM5	RNA binding motif protein X-linked-like-1 OS=Mus musculus OX=10090 GN=Rbmxl1 PE=2 SV=1	0.856666667

Ppp1ca	P62137	Serine/threonine-protein phosphatase PP1-alpha catalytic subunit OS=Mus musculus OX=10090 GI	0.87
Ppp1cb	P62141	Serine/threonine-protein phosphatase PP1-beta catalytic subunit OS=Mus musculus OX=10090 GN	0.723333333
Ahcyl2	Q68FL4	Putative adenosylhomocysteinase 3 OS=Mus musculus OX=10090 GN=Ahcyl2 PE=1 SV=1	1.056666667
Eif5a	P63242	Eukaryotic translation initiation factor 5A-1 OS=Mus musculus OX=10090 GN=Eif5a PE=1 SV=2	0.963333333
Ppp2r1b	Q7TNP2	Serine/threonine-protein phosphatase 2A 65 kDa regulatory subunit A beta isoform OS=Mus muscul	0.813333333
Gnb2	P62880	Guanine nucleotide-binding protein G(I)/G(S)/G(T) subunit beta-2 OS=Mus musculus OX=10090 GN	0.846666667
Safb2	Q80YR5	Scaffold attachment factor B2 OS=Mus musculus OX=10090 GN=Safb2 PE=1 SV=2	1.043333333
Ubqln2	Q9QZM0	Ubiquilin-2 OS=Mus musculus OX=10090 GN=Ubqln2 PE=1 SV=2	0.856666667
Irf2bp2	E9Q1P8	Interferon regulatory factor 2-binding protein 2 OS=Mus musculus OX=10090 GN=Irf2bp2 PE=1 SV=	0.74
Prdx4	O08807	Peroxiredoxin-4 OS=Mus musculus OX=10090 GN=Prdx4 PE=1 SV=1	0.896666667
Pitpnb	P53811	Phosphatidylinositol transfer protein beta isoform OS=Mus musculus OX=10090 GN=Pitpnb PE=1 S	0.876666667
Pycr2	Q922Q4	Pyrroline-5-carboxylate reductase 2 OS=Mus musculus OX=10090 GN=Pycr2 PE=1 SV=1	0.916666667
Vdac3	Q60931	Voltage-dependent anion-selective channel protein 3 OS=Mus musculus OX=10090 GN=Vdac3 PE=1	0.926666667
Akr1b8	P45377	Aldose reductase-related protein 2 OS=Mus musculus OX=10090 GN=Akr1b8 PE=1 SV=2	0.933333333
Rrm2	P11157	Ribonucleoside-diphosphate reductase subunit M2 OS=Mus musculus OX=10090 GN=Rrm2 PE=1 SV	0.89
Brd2	Q7JJ13	Bromodomain-containing protein 2 OS=Mus musculus OX=10090 GN=Brd2 PE=1 SV=1	1.083333333
Anxa4	P97429	Annexin A4 OS=Mus musculus OX=10090 GN=Anxa4 PE=1 SV=4	0.906666667
Myo1b	P46735	Unconventional myosin-Ib OS=Mus musculus OX=10090 GN=Myo1b PE=1 SV=3	0.87
Papola	Q61183	Poly(A) polymerase alpha OS=Mus musculus OX=10090 GN=Papola PE=1 SV=4	0.896666667
Csnk2a1	Q60737	Casein kinase II subunit alpha OS=Mus musculus OX=10090 GN=Csnk2a1 PE=1 SV=2	0.853333333
Ddah1	Q9CWS0	N(G) N(G)-dimethylarginine dimethylaminohydrolase 1 OS=Mus musculus OX=10090 GN=Ddah1 PE	0.926666667
Ddx20	Q9JJY4	Probable ATP-dependent RNA helicase DDX20 OS=Mus musculus OX=10090 GN=Ddx20 PE=1 SV=2	0.893333333
Kif23	E9Q5G3	Kinesin-like protein KIF23 OS=Mus musculus OX=10090 GN=Kif23 PE=1 SV=1	0.986666667
Ank3	G5E8K5	Ankyrin-3 OS=Mus musculus OX=10090 GN=Ank3 PE=1 SV=1	0.833333333
Gnl3	Q8CI11	Guanine nucleotide-binding protein-like 3 OS=Mus musculus OX=10090 GN=Gnl3 PE=1 SV=2	0.876666667
Anxa7	Q07076	Annexin A7 OS=Mus musculus OX=10090 GN=Anxa7 PE=1 SV=2	0.853333333
Kifc1	Q9QWT9	Kinesin-like protein KIFC1 OS=Mus musculus OX=10090 GN=Kifc1 PE=1 SV=2	0.803333333
Tmod3	Q9JHJ0	Tropomodulin-3 OS=Mus musculus OX=10090 GN=Tmod3 PE=1 SV=1	0.843333333
Tns2	Q8CGB6	Tensin-2 OS=Mus musculus OX=10090 GN=Tns2 PE=1 SV=1	0.803333333
Acsl5	Q8JZR0	Long-chain-fatty-acid--CoA ligase 5 OS=Mus musculus OX=10090 GN=Acsl5 PE=1 SV=1	0.82
Pcnt	P48725	Pericentrin OS=Mus musculus OX=10090 GN=Pcnt PE=1 SV=2	1.06
Ranbp3	Q9CT10	Ran-binding protein 3 OS=Mus musculus OX=10090 GN=Ranbp3 PE=1 SV=2	0.913333333
Acat2	Q8CAY6	Acetyl-CoA acetyltransferase cytosolic OS=Mus musculus OX=10090 GN=Acat2 PE=1 SV=2	0.866666667
Uqcrc2	Q9DB77	Cytochrome b-c1 complex subunit 2 mitochondrial OS=Mus musculus OX=10090 GN=Uqcrc2 PE=1	0.923333333

U2af2	P26369	Splicing factor U2AF 65 kDa subunit OS=Mus musculus OX=10090 GN=U2af2 PE=1 SV=3	0.883333333
Pfn1	P62962	Profilin-1 OS=Mus musculus OX=10090 GN=Pfn1 PE=1 SV=2	0.89
Memo1	Q91VH6	Protein MEMO1 OS=Mus musculus OX=10090 GN=Memo1 PE=1 SV=1	0.9
Eif3h	Q91WK2	Eukaryotic translation initiation factor 3 subunit H OS=Mus musculus OX=10090 GN=Eif3h PE=1 SV=	0.92
Lyar	Q08288	Cell growth-regulating nucleolar protein OS=Mus musculus OX=10090 GN=Lyar PE=1 SV=2	0.966666667
Myg1	Q9JK81	MYG1 exonuclease OS=Mus musculus OX=10090 GN=Myg1 PE=1 SV=1	0.84
Ctsd	P18242	Cathepsin D OS=Mus musculus OX=10090 GN=Ctsd PE=1 SV=1	0.846666667
Klhdc4	Q921I2	Kelch domain-containing protein 4 OS=Mus musculus OX=10090 GN=Klhdc4 PE=2 SV=2	0.866666667
Cat	P24270	Catalase OS=Mus musculus OX=10090 GN=Cat PE=1 SV=4	0.833333333
Raver1	Q9CW46	Ribonucleoprotein PTB-binding 1 OS=Mus musculus OX=10090 GN=Raver1 PE=1 SV=2	0.836666667
Znf326	O88291	DBIRD complex subunit ZNF326 OS=Mus musculus OX=10090 GN=Znf326 PE=1 SV=1	0.846666667
Mthfd2	P18155	Bifunctional methylenetetrahydrofolate dehydrogenase/cyclohydrolase mitochondrial OS=Mus mu	0.866666667
Tagln2	Q9WVA4	Transgelin-2 OS=Mus musculus OX=10090 GN=Tagln2 PE=1 SV=4	0.91
Gps1	Q99LD4	COP9 signalosome complex subunit 1 OS=Mus musculus OX=10090 GN=Gps1 PE=1 SV=1	0.853333333
Gcat	O88986	2-amino-3-ketobutyrate coenzyme A ligase mitochondrial OS=Mus musculus OX=10090 GN=Gcat P	0.84
Rpl7a	P12970	60S ribosomal protein L7a OS=Mus musculus OX=10090 GN=Rpl7a PE=1 SV=2	0.893333333
Dlat	Q8BMF4	Dihydrolipoyllysine-residue acetyltransferase component of pyruvate dehydrogenase complex mit	0.85
Kif20a	P97329	Kinesin-like protein KIF20A OS=Mus musculus OX=10090 GN=Kif20a PE=1 SV=1	1.003333333
Rps8	P62242	40S ribosomal protein S8 OS=Mus musculus OX=10090 GN=Rps8 PE=1 SV=2	0.893333333
Blmh	Q8R016	Bleomycin hydrolase OS=Mus musculus OX=10090 GN=Blmh PE=1 SV=1	0.863333333
Rab7a	P51150	Ras-related protein Rab-7a OS=Mus musculus OX=10090 GN=Rab7a PE=1 SV=2	0.913333333
Tp53bp1	P70399	TP53-binding protein 1 OS=Mus musculus OX=10090 GN=Tp53bp1 PE=1 SV=3	0.846666667
Vcan	Q62059	Versican core protein OS=Mus musculus OX=10090 GN=Vcan PE=1 SV=2	0.95
Phip	Q8VDD9	PH-interacting protein OS=Mus musculus OX=10090 GN=Phip PE=1 SV=2	1.07
Rnmt	Q9D0L8	mRNA cap guanine-N7 methyltransferase OS=Mus musculus OX=10090 GN=Rnmt PE=1 SV=1	0.883333333
Pak1ip1	Q9DCE5	p21-activated protein kinase-interacting protein 1 OS=Mus musculus OX=10090 GN=Pak1ip1 PE=1 S	0.816666667
Aqr	Q8CFQ3	RNA helicase aquarius OS=Mus musculus OX=10090 GN=Aqr PE=1 SV=2	0.886666667
Rasa3	Q60790	Ras GTPase-activating protein 3 OS=Mus musculus OX=10090 GN=Rasa3 PE=1 SV=2	0.763333333
Rpl10a	P53026	60S ribosomal protein L10a OS=Mus musculus OX=10090 GN=Rpl10a PE=1 SV=3	0.896666667
Col5a1	O88207	Collagen alpha-1(V) chain OS=Mus musculus OX=10090 GN=Col5a1 PE=1 SV=2	0.716666667
Dnajc7	Q9QYI3	DnaJ homolog subfamily C member 7 OS=Mus musculus OX=10090 GN=Dnajc7 PE=1 SV=2	0.91
Eif3g	Q9Z1D1	Eukaryotic translation initiation factor 3 subunit G OS=Mus musculus OX=10090 GN=Eif3g PE=1 SV=	0.866666667
Zc3h15	Q3TIV5	Zinc finger CCCH domain-containing protein 15 OS=Mus musculus OX=10090 GN=Zc3h15 PE=1 SV=	0.833333333
Tsta3	P23591	GDP-L-fucose synthase OS=Mus musculus OX=10090 GN=Tsta3 PE=1 SV=3	0.84

Hadhb	Q99JY0	Trifunctional enzyme subunit beta mitochondrial OS=Mus musculus OX=10090 GN=Hadhb PE=1 SV	0.963333333
Adam9	Q61072	Disintegrin and metalloproteinase domain-containing protein 9 OS=Mus musculus OX=10090 GN=/	0.783333333
Me2	Q99KE1	NAD-dependent malic enzyme mitochondrial OS=Mus musculus OX=10090 GN=Me2 PE=1 SV=1	0.91
Anapc4	Q91W96	Anaphase-promoting complex subunit 4 OS=Mus musculus OX=10090 GN=Anapc4 PE=1 SV=1	0.746666667
Mpp1	P70290	55 kDa erythrocyte membrane protein OS=Mus musculus OX=10090 GN=Mpp1 PE=1 SV=1	0.846666667
Hspbp1	Q99P31	Hsp70-binding protein 1 OS=Mus musculus OX=10090 GN=Hspbp1 PE=1 SV=1	0.956666667
Cert	Q9EQG9	Ceramide transfer protein OS=Mus musculus OX=10090 GN=Cert1 PE=1 SV=1	1.046666667
Tbk1	Q9WUN2	Serine/threonine-protein kinase TBK1 OS=Mus musculus OX=10090 GN=Tbk1 PE=1 SV=1	0.883333333
Pwp2	Q8BU03	Periodic tryptophan protein 2 homolog OS=Mus musculus OX=10090 GN=Pwp2 PE=1 SV=1	0.82
Nup85	Q8R480	Nuclear pore complex protein Nup85 OS=Mus musculus OX=10090 GN=Nup85 PE=1 SV=1	0.863333333
Nf1	Q04690	Neurofibromin OS=Mus musculus OX=10090 GN=Nf1 PE=1 SV=1	1.02
Smu1	Q3UKJ7	WD40 repeat-containing protein SMU1 OS=Mus musculus OX=10090 GN=Smu1 PE=2 SV=2	0.84
Ccdc47	Q9D024	Coiled-coil domain-containing protein 47 OS=Mus musculus OX=10090 GN=Ccdc47 PE=1 SV=2	0.94
Filip1l	Q6P6L0	Filamin A-interacting protein 1-like OS=Mus musculus OX=10090 GN=Filip1l PE=1 SV=2	0.886666667
Tagln	P37804	Transgelin OS=Mus musculus OX=10090 GN=Tagln PE=1 SV=3	0.943333333
Acadl	P51174	Long-chain specific acyl-CoA dehydrogenase mitochondrial OS=Mus musculus OX=10090 GN=Acadl	0.88
Ddost	O54734	Dolichyl-diphosphooligosaccharide--protein glycosyltransferase 48 kDa subunit OS=Mus musculus (	0.866666667
Snx4	Q91YJ2	Sorting nexin-4 OS=Mus musculus OX=10090 GN=Snx4 PE=1 SV=1	0.923333333
Oga	Q9EQQ9	Protein O-GlcNAcase OS=Mus musculus OX=10090 GN=Oga PE=1 SV=2	0.833333333
Apeh	Q8R146	Acylamino-acid-releasing enzyme OS=Mus musculus OX=10090 GN=Apeh PE=1 SV=3	0.88
Xpo4	Q9ESJ0	Exportin-4 OS=Mus musculus OX=10090 GN=Xpo4 PE=1 SV=2	0.916666667
Arpc2	Q9CVB6	Actin-related protein 2/3 complex subunit 2 OS=Mus musculus OX=10090 GN=Arpc2 PE=1 SV=3	0.876666667
Rbpj	P31266	Recombining binding protein suppressor of hairless OS=Mus musculus OX=10090 GN=Rbpj PE=1 SV=	0.886666667
	Q8C3W1	Uncharacterized protein C1orf198 homolog OS=Mus musculus OX=10090 PE=1 SV=1	0.746666667
Idh3a	Q9D6R2	Isocitrate dehydrogenase [NAD] subunit alpha mitochondrial OS=Mus musculus OX=10090 GN=Idh	0.863333333
Htatsf1	Q8BGC0	HIV Tat-specific factor 1 homolog OS=Mus musculus OX=10090 GN=Htatsf1 PE=1 SV=1	0.906666667
Slfn9	B1ARD6	Schlafen family member 9 OS=Mus musculus OX=10090 GN=Slfn9 PE=2 SV=1	0.806666667
Snw1	Q9CSN1	SNW domain-containing protein 1 OS=Mus musculus OX=10090 GN=Snw1 PE=1 SV=3	0.873333333
Pum1	Q80U78	Pumilio homolog 1 OS=Mus musculus OX=10090 GN=Pum1 PE=1 SV=2	0.933333333
Pes1	Q9EQ61	Pescadillo homolog OS=Mus musculus OX=10090 GN=Pes1 PE=1 SV=1	0.893333333
Arih1	Q9Z1K5	E3 ubiquitin-protein ligase ARIH1 OS=Mus musculus OX=10090 GN=Arih1 PE=1 SV=3	0.813333333
Colec12	Q8K4Q8	Collectin-12 OS=Mus musculus OX=10090 GN=Colec12 PE=1 SV=1	0.893333333
Mrps27	Q8BK72	28S ribosomal protein S27 mitochondrial OS=Mus musculus OX=10090 GN=Mrps27 PE=1 SV=2	0.813333333
Dglucy	Q8BH86	D-glutamate cyclase mitochondrial OS=Mus musculus OX=10090 GN=Dglucy PE=1 SV=1	0.833333333

Relch	Q148V7	RAB11-binding protein RELCH OS=Mus musculus OX=10090 GN=Relch PE=1 SV=1	0.976666667
Atad3	Q925I1	ATPase family AAA domain-containing protein 3 OS=Mus musculus OX=10090 GN=Atad3 PE=1 SV=1	0.896666667
Fbxw8	Q8BIA4	F-box/WD repeat-containing protein 8 OS=Mus musculus OX=10090 GN=Fbxw8 PE=1 SV=2	0.84
Pdha1	P35486	Pyruvate dehydrogenase E1 component subunit alpha somatic form mitochondrial OS=Mus muscu	0.873333333
Vcam1	P29533	Vascular cell adhesion protein 1 OS=Mus musculus OX=10090 GN=Vcam1 PE=1 SV=1	0.87
Xab2	Q9DCD2	Pre-mRNA-splicing factor SYF1 OS=Mus musculus OX=10090 GN=Xab2 PE=1 SV=1	0.953333333
Acad9	Q8JZN5	Complex I assembly factor ACAD9 mitochondrial OS=Mus musculus OX=10090 GN=Acad9 PE=1 SV=	0.926666667
Sin3a	Q60520	Paired amphipathic helix protein Sin3a OS=Mus musculus OX=10090 GN=Sin3a PE=1 SV=3	0.89
Nemf	Q8CCP0	Nuclear export mediator factor Nemf OS=Mus musculus OX=10090 GN=Nemf PE=1 SV=2	0.923333333
Ampd2	Q9DBT5	AMP deaminase 2 OS=Mus musculus OX=10090 GN=Ampd2 PE=1 SV=1	1.01
Glmn	Q8BZM1	Glomulin OS=Mus musculus OX=10090 GN=Glmn PE=1 SV=1	0.84
Hist1h2ah	Q8CGP6	Histone H2A type 1-H OS=Mus musculus OX=10090 GN=H2ac12 PE=1 SV=3	0.89
Hist1h2af	Q8CGP5	Histone H2A type 1-F OS=Mus musculus OX=10090 GN=Hist1h2af PE=1 SV=3	0.89
Hist1h2an	C0HKE7	Histone H2A type 1-N OS=Mus musculus OX=10090 GN=Hist1h2an PE=1 SV=1	0.89
H2ac13	C0HKE6	Histone H2A type 1-I OS=Mus musculus OX=10090 GN=H2ac13 PE=1 SV=1	0.89
H2ac11	C0HKE5	Histone H2A type 1-G OS=Mus musculus OX=10090 GN=H2ac11 PE=1 SV=1	0.89
H2ac15	Q8CGP7	Histone H2A type 1-K OS=Mus musculus OX=10090 GN=H2ac15 PE=1 SV=3	0.89
Hist3h2a	Q8BFU2	Histone H2A type 3 OS=Mus musculus OX=10090 GN=H2aw PE=1 SV=3	0.89
H2ac7	C0HKE3	Histone H2A type 1-D OS=Mus musculus OX=10090 GN=H2ac7 PE=1 SV=1	0.89
Hist1h2ap	C0HKE9	Histone H2A type 1-P OS=Mus musculus OX=10090 GN=Hist1h2ap PE=1 SV=1	0.89
H2ac4	C0HKE1	Histone H2A type 1-B OS=Mus musculus OX=10090 GN=H2ac4 PE=1 SV=1	0.89
Hist1h2ac	C0HKE2	Histone H2A type 1-C OS=Mus musculus OX=10090 GN=H2ac6 PE=1 SV=1	0.89
H2ac8	C0HKE4	Histone H2A type 1-E OS=Mus musculus OX=10090 GN=H2ac8 PE=1 SV=1	0.89
Hist1h2ao	C0HKE8	Histone H2A type 1-O OS=Mus musculus OX=10090 GN=Hist1h2ao PE=1 SV=1	0.89
Hnrnph2	P70333	Heterogeneous nuclear ribonucleoprotein H2 OS=Mus musculus OX=10090 GN=Hnrnph2 PE=1 SV=:	0.88
Tpm3	P21107	Tropomyosin alpha-3 chain OS=Mus musculus OX=10090 GN=Tpm3 PE=1 SV=3	0.896666667
Gnai2	P08752	Guanine nucleotide-binding protein G(i) subunit alpha-2 OS=Mus musculus OX=10090 GN=Gnai2 PI	0.876666667
Arfgef1	G3X9K3	Brefeldin A-inhibited guanine nucleotide-exchange protein 1 OS=Mus musculus OX=10090 GN=Arfg	0.7
Hdac1	O09106	Histone deacetylase 1 OS=Mus musculus OX=10090 GN=Hdac1 PE=1 SV=1	0.886666667
Septin8	Q8CHH9	Septin-8 OS=Mus musculus OX=10090 GN=Septin8 PE=1 SV=4	0.81
Map2k1	P31938	Dual specificity mitogen-activated protein kinase kinase 1 OS=Mus musculus OX=10090 GN=Map2k:	0.963333333
Brd3	Q8K2F0	Bromodomain-containing protein 3 OS=Mus musculus OX=10090 GN=Brd3 PE=1 SV=2	0.96
Irf2bpl	Q8K3X4	Probable E3 ubiquitin-protein ligase IRF2BPL OS=Mus musculus OX=10090 GN=Irf2bpl PE=1 SV=1	1.036666667
Ywhaq	P68254	14-3-3 protein theta OS=Mus musculus OX=10090 GN=Ywhaq PE=1 SV=1	0.913333333

Kpna4	O35343	Importin subunit alpha-3 OS=Mus musculus OX=10090 GN=Kpna4 PE=1 SV=1	0.91
Pitpna	P53810	Phosphatidylinositol transfer protein alpha isoform OS=Mus musculus OX=10090 GN=Pitpna PE=1 S	0.88
Mta1	Q8K4B0	Metastasis-associated protein MTA1 OS=Mus musculus OX=10090 GN=Mta1 PE=1 SV=1	0.903333333
Hdgf	P51859	Hepatoma-derived growth factor OS=Mus musculus OX=10090 GN=Hdgf PE=1 SV=2	0.82
H2afy2	Q8CCK0	Core histone macro-H2A.2 OS=Mus musculus OX=10090 GN=Macroh2a2 PE=1 SV=3	0.863333333
Ldhb	P16125	L-lactate dehydrogenase B chain OS=Mus musculus OX=10090 GN=Ldhb PE=1 SV=2	0.82
Arid1a	A2BH40	AT-rich interactive domain-containing protein 1A OS=Mus musculus OX=10090 GN=Arid1a PE=1 SV	0.946666667
Bzw1	Q9CQC6	Basic leucine zipper and W2 domain-containing protein 1 OS=Mus musculus OX=10090 GN=Bzw1 P	0.863333333
Snx2	Q9CWK8	Sorting nexin-2 OS=Mus musculus OX=10090 GN=Snx2 PE=1 SV=2	0.83
Tmx3	Q8BXZ1	Protein disulfide-isomerase TMX3 OS=Mus musculus OX=10090 GN=Tmx3 PE=1 SV=2	0.83
Kif2a	P28740	Kinesin-like protein KIF2A OS=Mus musculus OX=10090 GN=Kif2a PE=1 SV=2	0.986666667
Strn	O55106	Striatin OS=Mus musculus OX=10090 GN=Strn PE=1 SV=2	0.96
Snx1	Q9WV80	Sorting nexin-1 OS=Mus musculus OX=10090 GN=Snx1 PE=1 SV=1	0.916666667
Utp14a	Q640M1	U3 small nucleolar RNA-associated protein 14 homolog A OS=Mus musculus OX=10090 GN=Utp14a	0.773333333
Clic1	Q9Z1Q5	Chloride intracellular channel protein 1 OS=Mus musculus OX=10090 GN=Clic1 PE=1 SV=3	0.866666667
Bod1l	E9Q6J5	Biorientation of chromosomes in cell division protein 1-like 1 OS=Mus musculus OX=10090 GN=Bo	0.89
Shmt1	P50431	Serine hydroxymethyltransferase cytosolic OS=Mus musculus OX=10090 GN=Shmt1 PE=1 SV=3	0.85
Clic4	Q9QYB1	Chloride intracellular channel protein 4 OS=Mus musculus OX=10090 GN=Clic4 PE=1 SV=3	0.813333333
Vdac2	Q60930	Voltage-dependent anion-selective channel protein 2 OS=Mus musculus OX=10090 GN=Vdac2 PE=1	0.903333333
Acsl4	Q9QUJ7	Long-chain-fatty-acid--CoA ligase 4 OS=Mus musculus OX=10090 GN=Acsl4 PE=1 SV=2	0.88
Nol6	Q8R5K4	Nucleolar protein 6 OS=Mus musculus OX=10090 GN=Nol6 PE=2 SV=2	0.84
Stxbp1	O08599	Syntaxin-binding protein 1 OS=Mus musculus OX=10090 GN=Stxbp1 PE=1 SV=2	0.89
Gns	Q8BFR4	N-acetylglucosamine-6-sulfatase OS=Mus musculus OX=10090 GN=Gns PE=1 SV=1	0.88
Gsto1	O09131	Glutathione S-transferase omega-1 OS=Mus musculus OX=10090 GN=Gsto1 PE=1 SV=2	0.886666667
Dstn	Q9R0P5	Destrin OS=Mus musculus OX=10090 GN=Dstn PE=1 SV=3	0.93
Sh3pxd2b	A2AAY5	SH3 and PX domain-containing protein 2B OS=Mus musculus OX=10090 GN=Sh3pxd2b PE=1 SV=1	0.816666667
Snx5	Q9D8U8	Sorting nexin-5 OS=Mus musculus OX=10090 GN=Snx5 PE=1 SV=1	0.886666667
Mif	P34884	Macrophage migration inhibitory factor OS=Mus musculus OX=10090 GN=Mif PE=1 SV=2	0.88
Sec13	Q9D1M0	Protein SEC13 homolog OS=Mus musculus OX=10090 GN=Sec13 PE=1 SV=3	0.91
Fbl	P35550	rRNA 2'-O-methyltransferase fibrillarin OS=Mus musculus OX=10090 GN=Fbl PE=1 SV=2	0.903333333
Ctsb	P10605	Cathepsin B OS=Mus musculus OX=10090 GN=Ctsb PE=1 SV=2	0.873333333
Plrg1	Q922V4	Pleiotropic regulator 1 OS=Mus musculus OX=10090 GN=Plrg1 PE=1 SV=1	0.86
Fhl2	O70433	Four and a half LIM domains protein 2 OS=Mus musculus OX=10090 GN=Fhl2 PE=1 SV=1	0.883333333
Eif3m	Q99JX4	Eukaryotic translation initiation factor 3 subunit M OS=Mus musculus OX=10090 GN=Eif3m PE=1 S\	0.866666667

Psma2	P49722	Proteasome subunit alpha type-2 OS=Mus musculus OX=10090 GN=Psma2 PE=1 SV=3	0.833333333
Sqstm1	Q64337	Sequestosome-1 OS=Mus musculus OX=10090 GN=Sqstm1 PE=1 SV=1	0.806666667
Abcf1	Q6P542	ATP-binding cassette sub-family F member 1 OS=Mus musculus OX=10090 GN=Abcf1 PE=1 SV=1	0.88
Fam114a1	Q9D281	Protein Noxp20 OS=Mus musculus OX=10090 GN=Fam114a1 PE=1 SV=1	0.85
Ppp1r7	Q3UM45	Protein phosphatase 1 regulatory subunit 7 OS=Mus musculus OX=10090 GN=Ppp1r7 PE=1 SV=2	0.863333333
Rbm39	Q8VH51	RNA-binding protein 39 OS=Mus musculus OX=10090 GN=Rbm39 PE=1 SV=2	0.88
Itga11	P61622	Integrin alpha-11 OS=Mus musculus OX=10090 GN=Itga11 PE=1 SV=1	0.946666667
Lox	P28301	Protein-lysine 6-oxidase OS=Mus musculus OX=10090 GN=Lox PE=1 SV=1	0.883333333
Nampt	Q99KQ4	Nicotinamide phosphoribosyltransferase OS=Mus musculus OX=10090 GN=Nampt PE=1 SV=1	0.85
P4ha2	Q60716	Prolyl 4-hydroxylase subunit alpha-2 OS=Mus musculus OX=10090 GN=P4ha2 PE=1 SV=1	0.946666667
Pdhb	Q9D051	Pyruvate dehydrogenase E1 component subunit beta mitochondrial OS=Mus musculus OX=10090 (	0.84
Pex5	O09012	Peroxisomal targeting signal 1 receptor OS=Mus musculus OX=10090 GN=Pex5 PE=1 SV=2	0.816666667
Fhod1	Q6P9Q4	FH1/FH2 domain-containing protein 1 OS=Mus musculus OX=10090 GN=Fhod1 PE=1 SV=3	0.86
Acaa2	Q8BWT1	3-ketoacyl-CoA thiolase mitochondrial OS=Mus musculus OX=10090 GN=Acaa2 PE=1 SV=3	0.873333333
Drg2	Q9QXB9	Developmentally-regulated GTP-binding protein 2 OS=Mus musculus OX=10090 GN=Drg2 PE=1 SV=	0.883333333
Nsfl1c	Q9CZ44	NSFL1 cofactor p47 OS=Mus musculus OX=10090 GN=Nsfl1c PE=1 SV=1	0.863333333
Mtdh	Q80WJ7	Protein LYRIC OS=Mus musculus OX=10090 GN=Mtdh PE=1 SV=1	0.833333333
Cops2	P61202	COP9 signalosome complex subunit 2 OS=Mus musculus OX=10090 GN=Cops2 PE=1 SV=1	0.903333333
Gaa	P70699	Lysosomal alpha-glucosidase OS=Mus musculus OX=10090 GN=Gaa PE=1 SV=2	0.89
Slc25a24	Q8BMD8	Calcium-binding mitochondrial carrier protein SCaMC-1 OS=Mus musculus OX=10090 GN=Slc25a2	0.853333333
Lss	Q8BLN5	Lanosterol synthase OS=Mus musculus OX=10090 GN=Lss PE=1 SV=2	0.92
Psap	Q61207	Prosaposin OS=Mus musculus OX=10090 GN=Psap PE=1 SV=2	0.823333333
Api5	O35841	Apoptosis inhibitor 5 OS=Mus musculus OX=10090 GN=Api5 PE=1 SV=2	0.886666667
Gpt2	Q8BGT5	Alanine aminotransferase 2 OS=Mus musculus OX=10090 GN=Gpt2 PE=1 SV=1	0.893333333
Cyp20a1	Q8BKE6	Cytochrome P450 20A1 OS=Mus musculus OX=10090 GN=Cyp20a1 PE=1 SV=1	0.823333333
Rpl15	Q9CZM2	60S ribosomal protein L15 OS=Mus musculus OX=10090 GN=Rpl15 PE=1 SV=4	0.876666667
Cdc23	Q8BGZ4	Cell division cycle protein 23 homolog OS=Mus musculus OX=10090 GN=Cdc23 PE=1 SV=2	0.93
Dbn1	Q9QXS6	Drebrin OS=Mus musculus OX=10090 GN=Dbn1 PE=1 SV=4	0.913333333
Akap2	O54931	A-kinase anchor protein 2 OS=Mus musculus OX=10090 GN=Akap2 PE=1 SV=3	0.813333333
Ppp4r1	Q8K2V1	Serine/threonine-protein phosphatase 4 regulatory subunit 1 OS=Mus musculus OX=10090 GN=Ppp	0.866666667
Emc1	Q8C7X2	ER membrane protein complex subunit 1 OS=Mus musculus OX=10090 GN=Emc1 PE=1 SV=1	0.873333333
Ckap2	Q3V1H1	Cytoskeleton-associated protein 2 OS=Mus musculus OX=10090 GN=Ckap2 PE=1 SV=1	0.813333333
Pgm2	Q7TSV4	Phosphoglucomutase-2 OS=Mus musculus OX=10090 GN=Pgm2 PE=1 SV=1	0.88
Rab3gap1	Q80UJ7	Rab3 GTPase-activating protein catalytic subunit OS=Mus musculus OX=10090 GN=Rab3gap1 PE=1	1.12

Atp6v1c1	Q9Z1G3	V-type proton ATPase subunit C 1 OS=Mus musculus OX=10090 GN=Atp6v1c1 PE=1 SV=4	0.873333333
Larp7	Q05CL8	La-related protein 7 OS=Mus musculus OX=10090 GN=Larp7 PE=1 SV=2	0.806666667
Pmpca	Q9DC61	Mitochondrial-processing peptidase subunit alpha OS=Mus musculus OX=10090 GN=Pmpca PE=1 S	0.866666667
Esyt2	Q3TZZ7	Extended synaptotagmin-2 OS=Mus musculus OX=10090 GN=Esyt2 PE=1 SV=1	0.923333333
Nid1	P10493	Nidogen-1 OS=Mus musculus OX=10090 GN=Nid1 PE=1 SV=2	0.963333333
Ncln	Q8VCM8	Nicalin OS=Mus musculus OX=10090 GN=Ncln PE=1 SV=2	0.863333333
Inppl1	Q6P549	Phosphatidylinositol 3 4 5-trisphosphate 5-phosphatase 2 OS=Mus musculus OX=10090 GN=Inppl1	1.016666667
Ccdc88a	Q5SNZ0	Girdin OS=Mus musculus OX=10090 GN=Ccdc88a PE=1 SV=2	1.03
Mov10	P23249	Putative helicase MOV-10 OS=Mus musculus OX=10090 GN=Mov10 PE=1 SV=2	0.953333333
U2surp	Q6NV83	U2 snRNP-associated SURP motif-containing protein OS=Mus musculus OX=10090 GN=U2surp PE=1	0.993333333
Ap1m1	P35585	AP-1 complex subunit mu-1 OS=Mus musculus OX=10090 GN=Ap1m1 PE=1 SV=3	0.903333333
Anapc7	Q9WVM3	Anaphase-promoting complex subunit 7 OS=Mus musculus OX=10090 GN=Anapc7 PE=1 SV=3	1.143333333
Ncoa5	Q91W39	Nuclear receptor coactivator 5 OS=Mus musculus OX=10090 GN=Ncoa5 PE=1 SV=1	0.8
Hook3	Q8BUK6	Protein Hook homolog 3 OS=Mus musculus OX=10090 GN=Hook3 PE=1 SV=2	0.916666667
Dock11	A2AF47	Dedicator of cytokinesis protein 11 OS=Mus musculus OX=10090 GN=Dock11 PE=1 SV=1	0.973333333
Nup50	Q9JIH2	Nuclear pore complex protein Nup50 OS=Mus musculus OX=10090 GN=Nup50 PE=1 SV=3	0.84
Med23	Q80YQ2	Mediator of RNA polymerase II transcription subunit 23 OS=Mus musculus OX=10090 GN=Med23 P	0.83
Blvra	Q9CY64	Biliverdin reductase A OS=Mus musculus OX=10090 GN=Blvra PE=1 SV=1	0.856666667
Mical2	Q8BML1	[F-actin]-monooxygenase MICAL2 OS=Mus musculus OX=10090 GN=Mical2 PE=1 SV=1	0.903333333
H2bc15	P10853	Histone H2B type 1-F/J/L OS=Mus musculus OX=10090 GN=H2bc7 PE=1 SV=2	0.633333333
H2bc12	Q8CGP1	Histone H2B type 1-K OS=Mus musculus OX=10090 GN=H2bc12 PE=1 SV=3	0.633333333
Hist1h2bh	Q64478	Histone H2B type 1-H OS=Mus musculus OX=10090 GN=H2bc9 PE=1 SV=3	0.633333333
H2bc8	Q6ZWY9	Histone H2B type 1-C/E/G OS=Mus musculus OX=10090 GN=H2bc4 PE=1 SV=3	0.633333333
Actr1b	Q8R5C5	Beta-centractin OS=Mus musculus OX=10090 GN=Actr1b PE=1 SV=1	0.843333333
Ywhab	Q9CQV8	14-3-3 protein beta/alpha OS=Mus musculus OX=10090 GN=Ywhab PE=1 SV=3	0.903333333
Tpm2	P58774	Tropomyosin beta chain OS=Mus musculus OX=10090 GN=Tpm2 PE=1 SV=1	0.86
Prkca	P20444	Protein kinase C alpha type OS=Mus musculus OX=10090 GN=Prkca PE=1 SV=3	0.843333333
Mapk3	Q63844	Mitogen-activated protein kinase 3 OS=Mus musculus OX=10090 GN=Mapk3 PE=1 SV=5	0.733333333
Gstm1	P10649	Glutathione S-transferase Mu 1 OS=Mus musculus OX=10090 GN=Gstm1 PE=1 SV=2	0.843333333
Nedd4l	Q8CFI0	E3 ubiquitin-protein ligase NEDD4-like OS=Mus musculus OX=10090 GN=Nedd4l PE=1 SV=2	0.81
Safb	D3YXK2	Scaffold attachment factor B1 OS=Mus musculus OX=10090 GN=Safb PE=1 SV=2	0.833333333
Ybx3	Q9JKB3	Y-box-binding protein 3 OS=Mus musculus OX=10090 GN=Ybx3 PE=1 SV=2	0.836666667
Nmt1	O70310	Glycylpeptide N-tetradecanoyltransferase 1 OS=Mus musculus OX=10090 GN=Nmt1 PE=1 SV=1	0.843333333
Clip2	Q9Z0H8	CAP-Gly domain-containing linker protein 2 OS=Mus musculus OX=10090 GN=Clip2 PE=1 SV=2	0.973333333

Psip1	Q99JF8	PC4 and SFRS1-interacting protein OS=Mus musculus OX=10090 GN=Psip1 PE=1 SV=1	0.84
Strn3	Q9ERG2	Striatin-3 OS=Mus musculus OX=10090 GN=Strn3 PE=1 SV=1	0.83
Pdia6	Q922R8	Protein disulfide-isomerase A6 OS=Mus musculus OX=10090 GN=Pdia6 PE=1 SV=3	0.893333333
Prdx2	Q61171	Peroxiredoxin-2 OS=Mus musculus OX=10090 GN=Prdx2 PE=1 SV=3	0.883333333
Pcna	P17918	Proliferating cell nuclear antigen OS=Mus musculus OX=10090 GN=Pcna PE=1 SV=2	0.886666667
Epha2	Q03145	Ephrin type-A receptor 2 OS=Mus musculus OX=10090 GN=Epha2 PE=1 SV=3	0.953333333
Arpc1b	Q9WV32	Actin-related protein 2/3 complex subunit 1B OS=Mus musculus OX=10090 GN=Arpc1b PE=1 SV=4	0.823333333
Cnn2	Q08093	Calponin-2 OS=Mus musculus OX=10090 GN=Cnn2 PE=1 SV=1	0.86
Actr2	P61161	Actin-related protein 2 OS=Mus musculus OX=10090 GN=Actr2 PE=1 SV=1	0.886666667
Gpc4	P51655	Glypican-4 OS=Mus musculus OX=10090 GN=Gpc4 PE=1 SV=2	0.86
Hars	Q61035	Histidine--tRNA ligase cytoplasmic OS=Mus musculus OX=10090 GN=Hars1 PE=1 SV=2	0.92
Fkbp9	Q9Z247	Peptidyl-prolyl cis-trans isomerase FKBP9 OS=Mus musculus OX=10090 GN=Fkbp9 PE=1 SV=1	0.91
Ddx50	Q99MJ9	ATP-dependent RNA helicase DDX50 OS=Mus musculus OX=10090 GN=Ddx50 PE=2 SV=1	0.866666667
Cit	P49025	Citron Rho-interacting kinase OS=Mus musculus OX=10090 GN=Cit PE=1 SV=3	1.02
Rps6ka4	Q9Z2B9	Ribosomal protein S6 kinase alpha-4 OS=Mus musculus OX=10090 GN=Rps6ka4 PE=1 SV=2	0.87
Rplp0	P14869	60S acidic ribosomal protein P0 OS=Mus musculus OX=10090 GN=Rplp0 PE=1 SV=3	0.873333333
Pebp1	P70296	Phosphatidylethanolamine-binding protein 1 OS=Mus musculus OX=10090 GN=Pebp1 PE=1 SV=3	0.843333333
Prkcsh	O08795	Glucosidase 2 subunit beta OS=Mus musculus OX=10090 GN=Prkcsh PE=1 SV=1	0.886666667
Picalm	Q7M6Y3	Phosphatidylinositol-binding clathrin assembly protein OS=Mus musculus OX=10090 GN=Picalm PI	0.873333333
Mtap	Q9CQ65	S-methyl-5'-thioadenosine phosphorylase OS=Mus musculus OX=10090 GN=Mtap PE=1 SV=1	0.86
Me1	P06801	NADP-dependent malic enzyme OS=Mus musculus OX=10090 GN=Me1 PE=1 SV=2	0.916666667
Dnaja1	P63037	DnaJ homolog subfamily A member 1 OS=Mus musculus OX=10090 GN=Dnaja1 PE=1 SV=1	0.94
Strap	Q9Z1Z2	Serine-threonine kinase receptor-associated protein OS=Mus musculus OX=10090 GN=Strap PE=1 S\	0.92
Sugt1	Q9CX34	Protein SGT1 homolog OS=Mus musculus OX=10090 GN=Sugt1 PE=1 SV=3	0.893333333
Sae1	Q9R1T2	SUMO-activating enzyme subunit 1 OS=Mus musculus OX=10090 GN=Sae1 PE=1 SV=1	0.903333333
Psmc6	P62334	26S proteasome regulatory subunit 10B OS=Mus musculus OX=10090 GN=Psmc6 PE=1 SV=1	0.873333333
Uba3	Q8C878	NEDD8-activating enzyme E1 catalytic subunit OS=Mus musculus OX=10090 GN=Uba3 PE=1 SV=2	0.93
Uqcrc1	Q9CZ13	Cytochrome b-c1 complex subunit 1 mitochondrial OS=Mus musculus OX=10090 GN=Uqcrc1 PE=1	0.89
Dnajc2	P54103	DnaJ homolog subfamily C member 2 OS=Mus musculus OX=10090 GN=Dnajc2 PE=1 SV=2	0.92
Psmd14	O35593	26S proteasome non-ATPase regulatory subunit 14 OS=Mus musculus OX=10090 GN=Psmd14 PE=1	0.88
Prdx6	O08709	Peroxiredoxin-6 OS=Mus musculus OX=10090 GN=Prdx6 PE=1 SV=3	0.833333333
Metap2	O08663	Methionine aminopeptidase 2 OS=Mus musculus OX=10090 GN=Metap2 PE=1 SV=1	0.973333333
Hat1	Q8BY71	Histone acetyltransferase type B catalytic subunit OS=Mus musculus OX=10090 GN=Hat1 PE=1 SV=1	0.866666667
Nsdhl	Q9R1J0	Sterol-4-alpha-carboxylate 3-dehydrogenase decarboxylating OS=Mus musculus OX=10090 GN=Nsc	0.85

Srp54	P14576	Signal recognition particle 54 kDa protein OS=Mus musculus OX=10090 GN=Srp54 PE=1 SV=2	0.876666667
Mcmbp	Q8R3C0	Mini-chromosome maintenance complex-binding protein OS=Mus musculus OX=10090 GN=Mcmb	0.88
Swap70	Q6A028	Switch-associated protein 70 OS=Mus musculus OX=10090 GN=Swap70 PE=1 SV=2	0.856666667
Bin1	O08539	Myc box-dependent-interacting protein 1 OS=Mus musculus OX=10090 GN=Bin1 PE=1 SV=1	0.856666667
Wars	P32921	Tryptophan--tRNA ligase cytoplasmic OS=Mus musculus OX=10090 GN=Wars1 PE=1 SV=2	0.883333333
Rcn1	Q05186	Reticulocalbin-1 OS=Mus musculus OX=10090 GN=Rcn1 PE=1 SV=1	0.913333333
Ehbp1l1	Q99MS7	EH domain-binding protein 1-like protein 1 OS=Mus musculus OX=10090 GN=Ehbp1l1 PE=1 SV=1	0.833333333
Srm	Q64674	Spermidine synthase OS=Mus musculus OX=10090 GN=Srm PE=1 SV=1	0.92
Sun2	Q8BJS4	SUN domain-containing protein 2 OS=Mus musculus OX=10090 GN=Sun2 PE=1 SV=3	1.01
Glod4	Q9CPV4	Glyoxalase domain-containing protein 4 OS=Mus musculus OX=10090 GN=Glod4 PE=1 SV=1	0.886666667
Nfkb2	Q9WTK5	Nuclear factor NF-kappa-B p100 subunit OS=Mus musculus OX=10090 GN=Nfkb2 PE=1 SV=1	0.8
Utp4	Q8R2N2	U3 small nucleolar RNA-associated protein 4 homolog OS=Mus musculus OX=10090 GN=Utp4 PE=2	1.013333333
Suclg2	Q9Z2I8	Succinate--CoA ligase [GDP-forming] subunit beta mitochondrial OS=Mus musculus OX=10090 GN=	0.91
Ugp2	Q91ZJ5	UTP--glucose-1-phosphate uridylyltransferase OS=Mus musculus OX=10090 GN=Ugp2 PE=1 SV=3	0.846666667
Washc4	Q3UMB9	WASH complex subunit 4 OS=Mus musculus OX=10090 GN=Washc4 PE=1 SV=2	0.906666667
Gnl2	Q99LH1	Nucleolar GTP-binding protein 2 OS=Mus musculus OX=10090 GN=Gnl2 PE=1 SV=2	0.873333333
Pbrm1	Q8BSQ9	Protein polybromo-1 OS=Mus musculus OX=10090 GN=Pbrm1 PE=1 SV=4	0.84
Ercc6l	Q8BHK9	DNA excision repair protein ERCC-6-like OS=Mus musculus OX=10090 GN=Ercc6l PE=1 SV=1	0.783333333
Herc4	Q6PAV2	Probable E3 ubiquitin-protein ligase HERC4 OS=Mus musculus OX=10090 GN=Herc4 PE=1 SV=2	0.84
Wdhd1	P59328	WD repeat and HMG-box DNA-binding protein 1 OS=Mus musculus OX=10090 GN=Wdhd1 PE=1 SV=	0.793333333
Vwa5a	Q99KC8	von Willebrand factor A domain-containing protein 5A OS=Mus musculus OX=10090 GN=Vwa5a PE	0.89
Rpap3	Q9D706	RNA polymerase II-associated protein 3 OS=Mus musculus OX=10090 GN=Rpap3 PE=1 SV=1	0.826666667
Cip2a	Q8BWY9	Protein CIP2A OS=Mus musculus OX=10090 GN=Cip2a PE=1 SV=3	0.91
Wdr11	Q8K1X1	WD repeat-containing protein 11 OS=Mus musculus OX=10090 GN=Wdr11 PE=1 SV=1	0.806666667
Rab3gap2	Q8BMG7	Rab3 GTPase-activating protein non-catalytic subunit OS=Mus musculus OX=10090 GN=Rab3gap2 F	0.84
Far1	Q922J9	Fatty acyl-CoA reductase 1 OS=Mus musculus OX=10090 GN=Far1 PE=1 SV=1	0.83
Mms19	Q9D071	MMS19 nucleotide excision repair protein homolog OS=Mus musculus OX=10090 GN=Mms19 PE=1	0.806666667
Hip1	Q8VD75	Huntingtin-interacting protein 1 OS=Mus musculus OX=10090 GN=Hip1 PE=1 SV=2	0.85
Ptk2	P34152	Focal adhesion kinase 1 OS=Mus musculus OX=10090 GN=Ptk2 PE=1 SV=4	0.896666667
Sec24a	Q3U2P1	Protein transport protein Sec24A OS=Mus musculus OX=10090 GN=Sec24a PE=1 SV=1	0.843333333
Ltn1	Q6A009	E3 ubiquitin-protein ligase listerin OS=Mus musculus OX=10090 GN=Ltn1 PE=1 SV=3	0.79
Pnn	O35691	Pinin OS=Mus musculus OX=10090 GN=Pnn PE=1 SV=4	0.956666667
Cdkn2aip	Q8BI72	CDKN2A-interacting protein OS=Mus musculus OX=10090 GN=Cdkn2aip PE=1 SV=1	0.986666667
Pdlim7	Q3TJD7	PDZ and LIM domain protein 7 OS=Mus musculus OX=10090 GN=Pdlim7 PE=1 SV=1	0.916666667

Rbm28	Q8CGC6	RNA-binding protein 28 OS=Mus musculus OX=10090 GN=Rbm28 PE=1 SV=4	1.026666667
Tex10	Q3URQ0	Testis-expressed protein 10 OS=Mus musculus OX=10090 GN=Tex10 PE=1 SV=1	0.97
Casp8	O89110	Caspase-8 OS=Mus musculus OX=10090 GN=Casp8 PE=1 SV=1	0.853333333
Cul5	Q9D5V5	Cullin-5 OS=Mus musculus OX=10090 GN=Cul5 PE=1 SV=3	0.753333333
Ndufv1	Q91YT0	NADH dehydrogenase [ubiquinone] flavoprotein 1 mitochondrial OS=Mus musculus OX=10090 GN	0.913333333
Dnajc10	Q9DC23	DnaJ homolog subfamily C member 10 OS=Mus musculus OX=10090 GN=Dnajc10 PE=1 SV=2	0.873333333
Prkar1a	Q9DBC7	cAMP-dependent protein kinase type I-alpha regulatory subunit OS=Mus musculus OX=10090 GN=P	0.83
Asap1	Q9QWY8	Arf-GAP with SH3 domain ANK repeat and PH domain-containing protein 1 OS=Mus musculus OX=1	0.84
Stt3a	P46978	Dolichyl-diphosphooligosaccharide--protein glycosyltransferase subunit STT3A OS=Mus musculus C	0.853333333
Aco1	P28271	Cytoplasmic aconitate hydratase OS=Mus musculus OX=10090 GN=Aco1 PE=1 SV=3	0.86
Rbm12	Q8R4X3	RNA-binding protein 12 OS=Mus musculus OX=10090 GN=Rbm12 PE=1 SV=3	0.923333333
Hist2h2ab	Q64522	Histone H2A type 2-B OS=Mus musculus OX=10090 GN=H2ac21 PE=1 SV=3	
Stk3	Q9JI10	Serine/threonine-protein kinase 3 OS=Mus musculus OX=10090 GN=Stk3 PE=1 SV=1	0.85
Klc1	O88447	Kinesin light chain 1 OS=Mus musculus OX=10090 GN=Klc1 PE=1 SV=3	0.836666667
Akt1	P31750	RAC-alpha serine/threonine-protein kinase OS=Mus musculus OX=10090 GN=Akt1 PE=1 SV=2	0.883333333
Tpm4	Q6IRU2	Tropomyosin alpha-4 chain OS=Mus musculus OX=10090 GN=Tpm4 PE=1 SV=3	0.896666667
G3bp2	P97379	Ras GTPase-activating protein-binding protein 2 OS=Mus musculus OX=10090 GN=G3bp2 PE=1 SV=	0.91
Igf2bp1	O88477	Insulin-like growth factor 2 mRNA-binding protein 1 OS=Mus musculus OX=10090 GN=Igf2bp1 PE=	0.83
Ctps2	P70303	CTP synthase 2 OS=Mus musculus OX=10090 GN=Ctps2 PE=1 SV=1	0.81
Cdk1	P11440	Cyclin-dependent kinase 1 OS=Mus musculus OX=10090 GN=Cdk1 PE=1 SV=3	0.87
Osbp	Q3B7Z2	Oxysterol-binding protein 1 OS=Mus musculus OX=10090 GN=Osbp PE=1 SV=3	0.896666667
Plod1	Q9R0E2	Procollagen-lysine 2-oxoglutarate 5-dioxygenase 1 OS=Mus musculus OX=10090 GN=Plod1 PE=1 SV	0.946666667
Triobp	Q99KW3	TRIO and F-actin-binding protein OS=Mus musculus OX=10090 GN=Triobp PE=1 SV=3	0.853333333
Bclaf1	Q8K019	Bcl-2-associated transcription factor 1 OS=Mus musculus OX=10090 GN=Bclaf1 PE=1 SV=2	0.816666667
Dync1li1	Q8R1Q8	Cytoplasmic dynein 1 light intermediate chain 1 OS=Mus musculus OX=10090 GN=Dync1li1 PE=1 S	0.823333333
Las1l	A2BE28	Ribosomal biogenesis protein LAS1L OS=Mus musculus OX=10090 GN=Las1l PE=1 SV=1	0.65
Marcks	P26645	Myristoylated alanine-rich C-kinase substrate OS=Mus musculus OX=10090 GN=Marcks PE=1 SV=2	0.733333333
Cops4	O88544	COP9 signalosome complex subunit 4 OS=Mus musculus OX=10090 GN=Cops4 PE=1 SV=1	0.88
Oat	P29758	Ornithine aminotransferase mitochondrial OS=Mus musculus OX=10090 GN=Oat PE=1 SV=1	0.916666667
Lman1	Q9D0F3	Protein ERGIC-53 OS=Mus musculus OX=10090 GN=Lman1 PE=1 SV=1	0.913333333
Cavin1	O54724	Caveolae-associated protein 1 OS=Mus musculus OX=10090 GN=Cavin1 PE=1 SV=1	0.85
Pelp1	Q9DBD5	Proline- glutamic acid- and leucine-rich protein 1 OS=Mus musculus OX=10090 GN=Pelp1 PE=1 SV=	0.823333333
Cttn	Q60598	Src substrate cortactin OS=Mus musculus OX=10090 GN=Cttn PE=1 SV=2	0.89
Napa	Q9DB05	Alpha-soluble NSF attachment protein OS=Mus musculus OX=10090 GN=Napa PE=1 SV=1	0.88

Ints3	Q7TPD0	Integrator complex subunit 3 OS=Mus musculus OX=10090 GN=Ints3 PE=1 SV=2	0.91
Pus7	Q91VU7	Pseudouridylate synthase 7 homolog OS=Mus musculus OX=10090 GN=Pus7 PE=2 SV=2	0.886666667
Cacybp	Q9CXW3	Calcyclin-binding protein OS=Mus musculus OX=10090 GN=Cacybp PE=1 SV=1	0.883333333
Poglut2	Q9JHP7	Protein O-glucosyltransferase 2 OS=Mus musculus OX=10090 GN=Poglut2 PE=1 SV=1	0.86
Agps	Q8C0I1	Alkyldihydroxyacetonephosphate synthase peroxisomal OS=Mus musculus OX=10090 GN=Agps PE=	0.88
Heatr3	Q8BQM4	HEAT repeat-containing protein 3 OS=Mus musculus OX=10090 GN=Heatr3 PE=1 SV=1	0.88
Cndp2	Q9D1A2	Cytosolic non-specific dipeptidase OS=Mus musculus OX=10090 GN=Cndp2 PE=1 SV=1	0.853333333
Pml	Q60953	Protein PML OS=Mus musculus OX=10090 GN=Pml PE=1 SV=3	0.896666667
Nae1	Q8VBW6	NEDD8-activating enzyme E1 regulatory subunit OS=Mus musculus OX=10090 GN=Nae1 PE=1 SV=1	0.926666667
Kdm1a	Q6ZQ88	Lysine-specific histone demethylase 1A OS=Mus musculus OX=10090 GN=Kdm1a PE=1 SV=2	0.853333333
Rb1cc1	Q9ESK9	RB1-inducible coiled-coil protein 1 OS=Mus musculus OX=10090 GN=Rb1cc1 PE=1 SV=3	0.93
Wdr75	Q3U821	WD repeat-containing protein 75 OS=Mus musculus OX=10090 GN=Wdr75 PE=1 SV=1	0.82
Ccar1	Q8CH18	Cell division cycle and apoptosis regulator protein 1 OS=Mus musculus OX=10090 GN=Ccar1 PE=1 S	0.813333333
Maoa	Q64133	Amine oxidase [flavin-containing] A OS=Mus musculus OX=10090 GN=Maoa PE=1 SV=3	0.813333333
Nup188	Q6ZQH8	Nucleoporin NUP188 homolog OS=Mus musculus OX=10090 GN=Nup188 PE=1 SV=2	0.916666667
Cebpz	P53569	CCAAT/enhancer-binding protein zeta OS=Mus musculus OX=10090 GN=Cebpz PE=1 SV=2	0.863333333
Hnrnpul1	Q8VDM6	Heterogeneous nuclear ribonucleoprotein U-like protein 1 OS=Mus musculus OX=10090 GN=Hnrnp	0.896666667
Ube2o	Q6ZPJ3	(E3-independent) E2 ubiquitin-conjugating enzyme UBE2O OS=Mus musculus OX=10090 GN=Ube2c	0.823333333
Cdc40	Q9DC48	Pre-mRNA-processing factor 17 OS=Mus musculus OX=10090 GN=Cdc40 PE=2 SV=1	0.843333333
Specc1l	Q2KN98	Cytospin-A OS=Mus musculus OX=10090 GN=Specc1l PE=1 SV=1	0.726666667
Lrrc8c	Q8R502	Volume-regulated anion channel subunit LRRC8C OS=Mus musculus OX=10090 GN=Lrrc8c PE=1 SV=	0.86
Diaph1	O08808	Protein diaphanous homolog 1 OS=Mus musculus OX=10090 GN=Diaph1 PE=1 SV=1	1
Prpf4	Q9DAW6	U4/U6 small nuclear ribonucleoprotein Prp4 OS=Mus musculus OX=10090 GN=Prpf4 PE=1 SV=1	0.916666667
Eefsec	Q9JHW4	Selenocysteine-specific elongation factor OS=Mus musculus OX=10090 GN=Eefsec PE=1 SV=2	0.863333333
Gigyf2	Q6Y7W8	GRB10-interacting GYF protein 2 OS=Mus musculus OX=10090 GN=Gigyf2 PE=1 SV=2	0.83
Ogfr	Q99PG2	Opioid growth factor receptor OS=Mus musculus OX=10090 GN=Ogfr PE=1 SV=1	0.86
Thoc1	Q8R3N6	THO complex subunit 1 OS=Mus musculus OX=10090 GN=Thoc1 PE=1 SV=1	0.803333333
Vps35l	Q8BWQ6	VPS35 endosomal protein sorting factor-like OS=Mus musculus OX=10090 GN=Vps35l PE=1 SV=2	0.976666667
Rad21	Q61550	Double-strand-break repair protein rad21 homolog OS=Mus musculus OX=10090 GN=Rad21 PE=1 S	0.906666667
Ddx27	Q921N6	Probable ATP-dependent RNA helicase DDX27 OS=Mus musculus OX=10090 GN=Ddx27 PE=1 SV=3	0.866666667
Rrp1b	Q91YK2	Ribosomal RNA processing protein 1 homolog B OS=Mus musculus OX=10090 GN=Rrp1b PE=1 SV=2	0.863333333
Tjp2	Q9Z0U1	Tight junction protein ZO-2 OS=Mus musculus OX=10090 GN=Tjp2 PE=1 SV=2	0.923333333
Pc	Q05920	Pyruvate carboxylase mitochondrial OS=Mus musculus OX=10090 GN=Pc PE=1 SV=1	0.933333333
Uhrf1	Q8VDF2	E3 ubiquitin-protein ligase UHRF1 OS=Mus musculus OX=10090 GN=Uhrf1 PE=1 SV=2	0.856666667

Cstf3	Q99LI7	Cleavage stimulation factor subunit 3 OS=Mus musculus OX=10090 GN=Cstf3 PE=1 SV=1	0.82
Itga5	P11688	Integrin alpha-5 OS=Mus musculus OX=10090 GN=Itga5 PE=1 SV=3	0.943333333
Ahcyl1	Q80SW1	S-adenosylhomocysteine hydrolase-like protein 1 OS=Mus musculus OX=10090 GN=Ahcyl1 PE=1 SV	0.873333333
Map2k2	Q63932	Dual specificity mitogen-activated protein kinase kinase 2 OS=Mus musculus OX=10090 GN=Map2k	0.876666667
H2afy	Q9QZQ8	Core histone macro-H2A.1 OS=Mus musculus OX=10090 GN=Macroh2a1 PE=1 SV=3	0.926666667
Igf2bp3	Q9CPN8	Insulin-like growth factor 2 mRNA-binding protein 3 OS=Mus musculus OX=10090 GN=Igf2bp3 PE=	0.87
Prdx1	P35700	Peroxiredoxin-1 OS=Mus musculus OX=10090 GN=Prdx1 PE=1 SV=1	0.866666667
Plod3	Q9R0E1	Multifunctional procollagen lysine hydroxylase and glycosyltransferase LH3 OS=Mus musculus OX=	0.856666667
Igf2bp2	Q5SF07	Insulin-like growth factor 2 mRNA-binding protein 2 OS=Mus musculus OX=10090 GN=Igf2bp2 PE=	0.916666667
Eif4b	Q8BGD9	Eukaryotic translation initiation factor 4B OS=Mus musculus OX=10090 GN=Eif4b PE=1 SV=1	0.83
Sart1	Q9Z315	U4/U6.U5 tri-snRNP-associated protein 1 OS=Mus musculus OX=10090 GN=Sart1 PE=1 SV=1	0.803333333
Aak1	Q3UHJ0	AP2-associated protein kinase 1 OS=Mus musculus OX=10090 GN=Aak1 PE=1 SV=2	0.903333333
Pdgfrb	P05622	Platelet-derived growth factor receptor beta OS=Mus musculus OX=10090 GN=Pdgfrb PE=1 SV=2	0.8
Nln	Q91YP2	Neurolysin mitochondrial OS=Mus musculus OX=10090 GN=Nln PE=1 SV=1	0.933333333
Fkbp10	Q61576	Peptidyl-prolyl cis-trans isomerase FKBP10 OS=Mus musculus OX=10090 GN=Fkbp10 PE=1 SV=2	0.936666667
Tomm70	Q9CZW5	Mitochondrial import receptor subunit TOM70 OS=Mus musculus OX=10090 GN=Tomm70 PE=1 SV	0.893333333
Pnp	P23492	Purine nucleoside phosphorylase OS=Mus musculus OX=10090 GN=Pnp PE=1 SV=2	0.863333333
Rpl5	P47962	60S ribosomal protein L5 OS=Mus musculus OX=10090 GN=Rpl5 PE=1 SV=3	0.956666667
Emilin1	Q99K41	EMILIN-1 OS=Mus musculus OX=10090 GN=Emilin1 PE=1 SV=1	0.9
Cs	Q9CZU6	Citrate synthase mitochondrial OS=Mus musculus OX=10090 GN=Cs PE=1 SV=1	0.926666667
Eif3i	Q9QZD9	Eukaryotic translation initiation factor 3 subunit I OS=Mus musculus OX=10090 GN=Eif3i PE=1 SV=1	0.906666667
Ftsj3	Q9DBE9	pre-rRNA 2'-O-ribose RNA methyltransferase FTSJ3 OS=Mus musculus OX=10090 GN=Ftsj3 PE=1 SV=	0.876666667
Txnl1	Q8CDN6	Thioredoxin-like protein 1 OS=Mus musculus OX=10090 GN=Txnl1 PE=1 SV=3	0.92
Hmgcs1	Q8JZK9	Hydroxymethylglutaryl-CoA synthase cytoplasmic OS=Mus musculus OX=10090 GN=Hmgcs1 PE=1	0.88
Atp6v1b2	P62814	V-type proton ATPase subunit B brain isoform OS=Mus musculus OX=10090 GN=Atp6v1b2 PE=1 SV	0.886666667
Pafah1b1	P63005	Platelet-activating factor acetylhydrolase IB subunit alpha OS=Mus musculus OX=10090 GN=Pafah1	0.856666667
Etf1	Q8BWY3	Eukaryotic peptide chain release factor subunit 1 OS=Mus musculus OX=10090 GN=Etf1 PE=1 SV=4	0.893333333
Grb10	Q60760	Growth factor receptor-bound protein 10 OS=Mus musculus OX=10090 GN=Grb10 PE=1 SV=2	0.843333333
Abcc1	O35379	Multidrug resistance-associated protein 1 OS=Mus musculus OX=10090 GN=Abcc1 PE=1 SV=1	0.963333333
Atp6v1a	P50516	V-type proton ATPase catalytic subunit A OS=Mus musculus OX=10090 GN=Atp6v1a PE=1 SV=2	0.93
Exoc4	O35382	Exocyst complex component 4 OS=Mus musculus OX=10090 GN=Exoc4 PE=1 SV=2	0.973333333
Acadvl	P50544	Very long-chain specific acyl-CoA dehydrogenase mitochondrial OS=Mus musculus OX=10090 GN=	0.856666667
Ipo11	Q8K2V6	Importin-11 OS=Mus musculus OX=10090 GN=Ipo11 PE=1 SV=1	0.833333333
Cyp51a1	Q8K0C4	Lanosterol 14-alpha demethylase OS=Mus musculus OX=10090 GN=Cyp51a1 PE=1 SV=1	0.866666667

Asah1	Q9WV54	Acid ceramidase OS=Mus musculus OX=10090 GN=Asah1 PE=1 SV=1	0.86
Xpnpep1	Q6P1B1	Xaa-Pro aminopeptidase 1 OS=Mus musculus OX=10090 GN=Xpnpep1 PE=1 SV=1	0.863333333
Ubap2	Q91VX2	Ubiquitin-associated protein 2 OS=Mus musculus OX=10090 GN=Ubap2 PE=1 SV=1	0.756666667
Mogs	Q80UM7	Mannosyl-oligosaccharide glucosidase OS=Mus musculus OX=10090 GN=Mogs PE=1 SV=1	0.836666667
Ero1a	Q8R180	ERO1-like protein alpha OS=Mus musculus OX=10090 GN=Ero1a PE=1 SV=2	0.886666667
Elp2	Q91WG4	Elongator complex protein 2 OS=Mus musculus OX=10090 GN=Elp2 PE=1 SV=1	1.08
Ap1g1	P22892	AP-1 complex subunit gamma-1 OS=Mus musculus OX=10090 GN=Ap1g1 PE=1 SV=3	0.94
Mdc1	Q5PSV9	Mediator of DNA damage checkpoint protein 1 OS=Mus musculus OX=10090 GN=Mdc1 PE=1 SV=1	0.766666667
Scyl1	Q9EQC5	N-terminal kinase-like protein OS=Mus musculus OX=10090 GN=Scyl1 PE=1 SV=1	0.88
Asph	Q8BSY0	Aspartyl/asparaginyl beta-hydroxylase OS=Mus musculus OX=10090 GN=Asph PE=1 SV=1	0.91
Exosc10	P56960	Exosome component 10 OS=Mus musculus OX=10090 GN=Exosc10 PE=1 SV=2	0.846666667
Ap2m1	P84091	AP-2 complex subunit mu OS=Mus musculus OX=10090 GN=Ap2m1 PE=1 SV=1	0.853333333
Ssrp1	Q08943	FACT complex subunit SSRP1 OS=Mus musculus OX=10090 GN=Ssrp1 PE=1 SV=2	0.903333333
Ilk	O55222	Integrin-linked protein kinase OS=Mus musculus OX=10090 GN=Ilk PE=1 SV=2	0.85
Colgalt1	Q8K297	Procollagen galactosyltransferase 1 OS=Mus musculus OX=10090 GN=Colgalt1 PE=1 SV=2	0.886666667
Kntc1	Q8C3Y4	Kinetochore-associated protein 1 OS=Mus musculus OX=10090 GN=Kntc1 PE=1 SV=2	1.003333333
Usp34	Q6ZQ93	Ubiquitin carboxyl-terminal hydrolase 34 OS=Mus musculus OX=10090 GN=Usp34 PE=1 SV=3	0.82
Actr1a	P61164	Alpha-centractin OS=Mus musculus OX=10090 GN=Actr1a PE=1 SV=1	0.92
Eno3	P21550	Beta-enolase OS=Mus musculus OX=10090 GN=Eno3 PE=1 SV=3	0.773333333
Tnpo2	Q99LG2	Transportin-2 OS=Mus musculus OX=10090 GN=Tnpo2 PE=1 SV=1	0.893333333
Rbbp4	Q60972	Histone-binding protein RBBP4 OS=Mus musculus OX=10090 GN=Rbbp4 PE=1 SV=5	0.87
Arfgef2	A2A5R2	Brefeldin A-inhibited guanine nucleotide-exchange protein 2 OS=Mus musculus OX=10090 GN=Arfg	0.836666667
Eef1d	P57776	Elongation factor 1-delta OS=Mus musculus OX=10090 GN=Eef1d PE=1 SV=3	0.89
Vdac1	Q60932	Voltage-dependent anion-selective channel protein 1 OS=Mus musculus OX=10090 GN=Vdac1 PE=1	0.9
Sec23ip	Q6NZC7	SEC23-interacting protein OS=Mus musculus OX=10090 GN=Sec23ip PE=1 SV=2	0.9
Nap1l1	P28656	Nucleosome assembly protein 1-like 1 OS=Mus musculus OX=10090 GN=Nap1l1 PE=1 SV=2	0.836666667
Kank2	Q8BX02	KN motif and ankyrin repeat domain-containing protein 2 OS=Mus musculus OX=10090 GN=Kank2	0.93
Tubgcp2	Q921G8	Gamma-tubulin complex component 2 OS=Mus musculus OX=10090 GN=Tubgcp2 PE=1 SV=2	0.963333333
Tns3	Q5SSZ5	Tensin-3 OS=Mus musculus OX=10090 GN=Tns3 PE=1 SV=1	0.806666667
Uap1l1	Q3TW96	UDP-N-acetylhexosamine pyrophosphorylase-like protein 1 OS=Mus musculus OX=10090 GN=Uap1	0.87
Ubap2l	Q80X50	Ubiquitin-associated protein 2-like OS=Mus musculus OX=10090 GN=Ubap2l PE=1 SV=1	0.866666667
Pgd	Q9DCD0	6-phosphogluconate dehydrogenase decarboxylating OS=Mus musculus OX=10090 GN=Pgd PE=1 S	0.866666667
NARS1	Q8BP47	Asparagine--tRNA ligase cytoplasmic OS=Mus musculus OX=10090 GN=NARS1 PE=1 SV=2	0.89
Rcc2	Q8BK67	Protein RCC2 OS=Mus musculus OX=10090 GN=Rcc2 PE=1 SV=1	0.866666667

Oxct1	Q9D0K2	Succinyl-CoA:3-ketoacid coenzyme A transferase 1 mitochondrial OS=Mus musculus OX=10090 GN	0.893333333
Rtcb	Q99LF4	RNA-splicing ligase RtcB homolog OS=Mus musculus OX=10090 GN=Rtcb PE=1 SV=1	0.866666667
Umps	P13439	Uridine 5'-monophosphate synthase OS=Mus musculus OX=10090 GN=Umps PE=1 SV=3	0.873333333
Rpl8	P62918	60S ribosomal protein L8 OS=Mus musculus OX=10090 GN=Rpl8 PE=1 SV=2	0.946666667
Eif2s1	Q6ZWX6	Eukaryotic translation initiation factor 2 subunit 1 OS=Mus musculus OX=10090 GN=Eif2s1 PE=1 SV	0.893333333
Scfd1	Q8BRF7	Sec1 family domain-containing protein 1 OS=Mus musculus OX=10090 GN=Scfd1 PE=1 SV=1	0.853333333
Acot9	Q9R0X4	Acyl-coenzyme A thioesterase 9 mitochondrial OS=Mus musculus OX=10090 GN=Acot9 PE=1 SV=1	0.866666667
Bag3	Q9JLV1	BAG family molecular chaperone regulator 3 OS=Mus musculus OX=10090 GN=Bag3 PE=1 SV=2	0.803333333
Nudc	O35685	Nuclear migration protein nudC OS=Mus musculus OX=10090 GN=Nudc PE=1 SV=1	0.846666667
Psmd5	Q8BJY1	26S proteasome non-ATPase regulatory subunit 5 OS=Mus musculus OX=10090 GN=Psmd5 PE=1 SV	0.913333333
Eif5	P59325	Eukaryotic translation initiation factor 5 OS=Mus musculus OX=10090 GN=Eif5 PE=1 SV=1	0.866666667
Zw10	O54692	Centromere/kinetochore protein zw10 homolog OS=Mus musculus OX=10090 GN=Zw10 PE=1 SV=3	0.88
Zc3hav1	Q3UPF5	Zinc finger CCCH-type antiviral protein 1 OS=Mus musculus OX=10090 GN=Zc3hav1 PE=1 SV=1	0.93
Aifm1	Q9Z0X1	Apoptosis-inducing factor 1 mitochondrial OS=Mus musculus OX=10090 GN=Aifm1 PE=1 SV=1	0.906666667
Hnrnpul2	Q00PI9	Heterogeneous nuclear ribonucleoprotein U-like protein 2 OS=Mus musculus OX=10090 GN=Hnrnp	0.836666667
Myo18a	Q9JMH9	Unconventional myosin-XVIIIa OS=Mus musculus OX=10090 GN=Myo18a PE=1 SV=2	0.883333333
Nucb1	Q02819	Nucleobindin-1 OS=Mus musculus OX=10090 GN=Nucb1 PE=1 SV=2	0.853333333
Obsl1	D3YYU8	Obscurin-like protein 1 OS=Mus musculus OX=10090 GN=Obsl1 PE=1 SV=1	0.783333333
Sh3kbp1	Q8R550	SH3 domain-containing kinase-binding protein 1 OS=Mus musculus OX=10090 GN=Sh3kbp1 PE=1 S	0.833333333
Wdr43	Q6ZQL4	WD repeat-containing protein 43 OS=Mus musculus OX=10090 GN=Wdr43 PE=1 SV=2	0.953333333
Rin1	Q921Q7	Ras and Rab interactor 1 OS=Mus musculus OX=10090 GN=Rin1 PE=1 SV=1	0.783333333
Rpl7	P14148	60S ribosomal protein L7 OS=Mus musculus OX=10090 GN=Rpl7 PE=1 SV=2	0.906666667
Plcg1	Q62077	1-phosphatidylinositol 4 5-bisphosphate phosphodiesterase gamma-1 OS=Mus musculus OX=1009	0.923333333
Sacm1l	Q9EP69	Phosphatidylinositol-3-phosphatase SAC1 OS=Mus musculus OX=10090 GN=Sacm1l PE=1 SV=1	0.86
Tsr1	Q5SWD9	Pre-rRNA-processing protein TSR1 homolog OS=Mus musculus OX=10090 GN=Tsr1 PE=1 SV=1	1.186666667
Pold1	P52431	DNA polymerase delta catalytic subunit OS=Mus musculus OX=10090 GN=Pold1 PE=1 SV=2	0.816666667
Arhgef40	Q3UPH7	Rho guanine nucleotide exchange factor 40 OS=Mus musculus OX=10090 GN=Arhgef40 PE=1 SV=1	0.816666667
Tpm1	P58771	Tropomyosin alpha-1 chain OS=Mus musculus OX=10090 GN=Tpm1 PE=1 SV=1	0.853333333
Gspt1	Q8R050	Eukaryotic peptide chain release factor GTP-binding subunit ERF3A OS=Mus musculus OX=10090 GI	0.84
Ppp2r2a	Q6P1F6	Serine/threonine-protein phosphatase 2A 55 kDa regulatory subunit B alpha isoform OS=Mus muscu	0.923333333
Pak2	Q8CIN4	Serine/threonine-protein kinase PAK 2 OS=Mus musculus OX=10090 GN=Pak2 PE=1 SV=1	0.85
Gdi1	P50396	Rab GDP dissociation inhibitor alpha OS=Mus musculus OX=10090 GN=Gdi1 PE=1 SV=3	0.826666667
Ybx1	P62960	Y-box-binding protein 1 OS=Mus musculus OX=10090 GN=Ybx1 PE=1 SV=3	0.87
Txndc5	Q91W90	Thioredoxin domain-containing protein 5 OS=Mus musculus OX=10090 GN=Txndc5 PE=1 SV=2	0.876666667

Copg2	Q9QXK3	Coatomer subunit gamma-2 OS=Mus musculus OX=10090 GN=Copg2 PE=1 SV=1	0.793333333
Ywhae	P62259	14-3-3 protein epsilon OS=Mus musculus OX=10090 GN=Ywhae PE=1 SV=1	0.903333333
Septin2	P42208	Septin-2 OS=Mus musculus OX=10090 GN=Septin2 PE=1 SV=2	0.873333333
Psmc1	P62192	26S proteasome regulatory subunit 4 OS=Mus musculus OX=10090 GN=Psmc1 PE=1 SV=1	0.876666667
Ruvbl1	P60122	RuvB-like 1 OS=Mus musculus OX=10090 GN=Ruvbl1 PE=1 SV=1	0.883333333
Eps15l1	Q60902	Epidermal growth factor receptor substrate 15-like 1 OS=Mus musculus OX=10090 GN=Eps15l1 PE=	0.913333333
Prpf40a	Q9R1C7	Pre-mRNA-processing factor 40 homolog A OS=Mus musculus OX=10090 GN=Prpf40a PE=1 SV=1	0.896666667
Tmtc3	Q8BRH0	Protein O-mannosyl-transferase TMTC3 OS=Mus musculus OX=10090 GN=Tmtc3 PE=1 SV=2	0.776666667
Acsl3	Q9CZW4	Long-chain-fatty-acid--CoA ligase 3 OS=Mus musculus OX=10090 GN=Acsl3 PE=1 SV=2	0.873333333
Mdh2	P08249	Malate dehydrogenase mitochondrial OS=Mus musculus OX=10090 GN=Mdh2 PE=1 SV=3	0.886666667
Rpsa	P14206	40S ribosomal protein SA OS=Mus musculus OX=10090 GN=Rpsa PE=1 SV=4	0.876666667
Tpi1	P17751	Triosephosphate isomerase OS=Mus musculus OX=10090 GN=Tpi1 PE=1 SV=4	0.846666667
Ahcy	P50247	Adenosylhomocysteinase OS=Mus musculus OX=10090 GN=Ahcy PE=1 SV=3	0.876666667
Cops3	O88543	COP9 signalosome complex subunit 3 OS=Mus musculus OX=10090 GN=Cops3 PE=1 SV=3	0.906666667
Rangap1	P46061	Ran GTPase-activating protein 1 OS=Mus musculus OX=10090 GN=Rangap1 PE=1 SV=2	0.906666667
Cast	P51125	Calpastatin OS=Mus musculus OX=10090 GN=Cast PE=1 SV=2	0.826666667
Rps3a	P97351	40S ribosomal protein S3a OS=Mus musculus OX=10090 GN=Rps3a PE=1 SV=3	0.936666667
Eif2s3x	Q9Z0N1	Eukaryotic translation initiation factor 2 subunit 3 X-linked OS=Mus musculus OX=10090 GN=Eif2s	0.876666667
Dnaja2	Q9QYJ0	DnaJ homolog subfamily A member 2 OS=Mus musculus OX=10090 GN=Dnaja2 PE=1 SV=1	0.903333333
Eif3d	O70194	Eukaryotic translation initiation factor 3 subunit D OS=Mus musculus OX=10090 GN=Eif3d PE=1 SV=	0.916666667
Fdps	Q920E5	Farnesyl pyrophosphate synthase OS=Mus musculus OX=10090 GN=Fdps PE=1 SV=1	0.876666667
Anxa5	P48036	Annexin A5 OS=Mus musculus OX=10090 GN=Anxa5 PE=1 SV=1	0.87
Pgm1	Q9D0F9	Phosphoglucomutase-1 OS=Mus musculus OX=10090 GN=Pgm1 PE=1 SV=4	0.88
Tcerg1	Q8CGF7	Transcription elongation regulator 1 OS=Mus musculus OX=10090 GN=Tcerg1 PE=1 SV=2	0.883333333
Rbm25	B2RY56	RNA-binding protein 25 OS=Mus musculus OX=10090 GN=Rbm25 PE=1 SV=2	0.863333333
Abcf2	Q99LE6	ATP-binding cassette sub-family F member 2 OS=Mus musculus OX=10090 GN=Abcf2 PE=1 SV=1	0.886666667
Dhx36	Q8VHK9	ATP-dependent DNA/RNA helicase DHX36 OS=Mus musculus OX=10090 GN=Dhx36 PE=1 SV=2	0.856666667
Eif3e	P60229	Eukaryotic translation initiation factor 3 subunit E OS=Mus musculus OX=10090 GN=Eif3e PE=1 SV=	0.883333333
Afdn	Q9QZQ1	Afadin OS=Mus musculus OX=10090 GN=Afdn PE=1 SV=3	0.89
Dock1	Q8BUR4	Dedicator of cytokinesis protein 1 OS=Mus musculus OX=10090 GN=Dock1 PE=1 SV=3	0.94
Ubtf	P25976	Nucleolar transcription factor 1 OS=Mus musculus OX=10090 GN=Ubtf PE=1 SV=1	0.853333333
Psmd6	Q99JI4	26S proteasome non-ATPase regulatory subunit 6 OS=Mus musculus OX=10090 GN=Psmd6 PE=1 SV	0.87
Nop14	Q8R3N1	Nucleolar protein 14 OS=Mus musculus OX=10090 GN=Nop14 PE=1 SV=2	0.94
Nvl	Q9DBY8	Nuclear valosin-containing protein-like OS=Mus musculus OX=10090 GN=Nvl PE=1 SV=1	0.85

Polr1a	O35134	DNA-directed RNA polymerase I subunit RPA1 OS=Mus musculus OX=10090 GN=Polr1a PE=1 SV=2	0.916666667
Lrrc40	Q9CRC8	Leucine-rich repeat-containing protein 40 OS=Mus musculus OX=10090 GN=Lrrc40 PE=1 SV=2	0.843333333
Nipbl	Q6KCD5	Nipped-B-like protein OS=Mus musculus OX=10090 GN=Nipbl PE=1 SV=1	0.97
Psme4	Q5SSW2	Proteasome activator complex subunit 4 OS=Mus musculus OX=10090 GN=Psme4 PE=1 SV=1	0.78
Nme1	P15532	Nucleoside diphosphate kinase A OS=Mus musculus OX=10090 GN=Nme1 PE=1 SV=1	0.903333333
Mapk1	P63085	Mitogen-activated protein kinase 1 OS=Mus musculus OX=10090 GN=Mapk1 PE=1 SV=3	0.743333333
Ywhaz	P63101	14-3-3 protein zeta/delta OS=Mus musculus OX=10090 GN=Ywhaz PE=1 SV=1	0.873333333
Rps6ka3	P18654	Ribosomal protein S6 kinase alpha-3 OS=Mus musculus OX=10090 GN=Rps6ka3 PE=1 SV=2	0.95
Hmgb1	P63158	High mobility group protein B1 OS=Mus musculus OX=10090 GN=Hmgb1 PE=1 SV=2	0.853333333
Idh1	O88844	Isocitrate dehydrogenase [NADP] cytoplasmic OS=Mus musculus OX=10090 GN=Idh1 PE=1 SV=2	0.88
Rock2	P70336	Rho-associated protein kinase 2 OS=Mus musculus OX=10090 GN=Rock2 PE=1 SV=1	0.916666667
Fmr1	P35922	Synaptic functional regulator FMR1 OS=Mus musculus OX=10090 GN=Fmr1 PE=1 SV=1	0.843333333
Wnk1	P83741	Serine/threonine-protein kinase WNK1 OS=Mus musculus OX=10090 GN=Wnk1 PE=1 SV=2	0.79
Cap1	P40124	Adenylyl cyclase-associated protein 1 OS=Mus musculus OX=10090 GN=Cap1 PE=1 SV=4	0.863333333
Septin7	O55131	Septin-7 OS=Mus musculus OX=10090 GN=Septin7 PE=1 SV=1	0.91
Atl3	Q91YH5	Atlastin-3 OS=Mus musculus OX=10090 GN=Atl3 PE=1 SV=1	0.92
Ddx18	Q8K363	ATP-dependent RNA helicase DDX18 OS=Mus musculus OX=10090 GN=Ddx18 PE=1 SV=1	0.863333333
Dhx29	Q6PGC1	ATP-dependent RNA helicase DHX29 OS=Mus musculus OX=10090 GN=Dhx29 PE=1 SV=1	1.02
Kpna2	P52293	Importin subunit alpha-1 OS=Mus musculus OX=10090 GN=Kpna2 PE=1 SV=2	0.846666667
Rps3	P62908	40S ribosomal protein S3 OS=Mus musculus OX=10090 GN=Rps3 PE=1 SV=1	0.893333333
Phb	P67778	Prohibitin OS=Mus musculus OX=10090 GN=Phb PE=1 SV=1	0.946666667
Sars	P26638	Serine--tRNA ligase cytoplasmic OS=Mus musculus OX=10090 GN=Sars1 PE=1 SV=3	0.85
Ncbp1	Q3UYV9	Nuclear cap-binding protein subunit 1 OS=Mus musculus OX=10090 GN=Ncbp1 PE=1 SV=2	0.913333333
Nxf1	Q99JX7	Nuclear RNA export factor 1 OS=Mus musculus OX=10090 GN=Nxf1 PE=1 SV=3	0.896666667
Psmd13	Q9WVJ2	26S proteasome non-ATPase regulatory subunit 13 OS=Mus musculus OX=10090 GN=Psmd13 PE=1	0.92
Psmc4	P54775	26S proteasome regulatory subunit 6B OS=Mus musculus OX=10090 GN=Psmc4 PE=1 SV=2	0.873333333
Rps4x	P62702	40S ribosomal protein S4 X isoform OS=Mus musculus OX=10090 GN=Rps4x PE=1 SV=2	0.906666667
Farsa	Q8C0C7	Phenylalanine--tRNA ligase alpha subunit OS=Mus musculus OX=10090 GN=Farsa PE=1 SV=1	0.883333333
Eif2a	Q8BJW6	Eukaryotic translation initiation factor 2A OS=Mus musculus OX=10090 GN=Eif2a PE=1 SV=2	0.843333333
Zfr	O88532	Zinc finger RNA-binding protein OS=Mus musculus OX=10090 GN=Zfr PE=1 SV=2	0.82
Rabgap1	A2AWA9	Rab GTPase-activating protein 1 OS=Mus musculus OX=10090 GN=Rabgap1 PE=1 SV=1	0.916666667
Ppid	Q9CR16	Peptidyl-prolyl cis-trans isomerase D OS=Mus musculus OX=10090 GN=Ppid PE=1 SV=3	0.873333333
Psmd11	Q8BG32	26S proteasome non-ATPase regulatory subunit 11 OS=Mus musculus OX=10090 GN=Psmd11 PE=1	0.883333333
Rbm14	Q8C2Q3	RNA-binding protein 14 OS=Mus musculus OX=10090 GN=Rbm14 PE=1 SV=1	0.92

Rpa1	Q8VEE4	Replication protein A 70 kDa DNA-binding subunit OS=Mus musculus OX=10090 GN=Rpa1 PE=1 SV=	0.9
Samhd1	Q60710	Deoxynucleoside triphosphate triphosphohydrolase SAMHD1 OS=Mus musculus OX=10090 GN=Sar	0.876666667
Erc1	Q99MI1	ELKS/Rab6-interacting/CAST family member 1 OS=Mus musculus OX=10090 GN=Erc1 PE=1 SV=1	1.063333333
Pdlim5	Q8CI51	PDZ and LIM domain protein 5 OS=Mus musculus OX=10090 GN=Pdlim5 PE=1 SV=4	0.87
Slc3a2	P10852	4F2 cell-surface antigen heavy chain OS=Mus musculus OX=10090 GN=Slc3a2 PE=1 SV=1	0.886666667
Thoc2	B1AZI6	THO complex subunit 2 OS=Mus musculus OX=10090 GN=Thoc2 PE=1 SV=1	0.886666667
Cpsf1	Q9EPU4	Cleavage and polyadenylation specificity factor subunit 1 OS=Mus musculus OX=10090 GN=Cpsf1 P	0.903333333
Gapvd1	Q6PAR5	GTPase-activating protein and VPS9 domain-containing protein 1 OS=Mus musculus OX=10090 GN=	0.846666667
Mvp	Q9EQK5	Major vault protein OS=Mus musculus OX=10090 GN=Mvp PE=1 SV=4	0.863333333
Iars2	Q8BIJ6	Isoleucine--tRNA ligase mitochondrial OS=Mus musculus OX=10090 GN=Iars2 PE=1 SV=1	0.8
Pum3	Q8BKS9	Pumilio homolog 3 OS=Mus musculus OX=10090 GN=Pum3 PE=1 SV=2	0.9
Sf3a3	Q9D554	Splicing factor 3A subunit 3 OS=Mus musculus OX=10090 GN=Sf3a3 PE=1 SV=2	0.876666667
Elac2	Q80Y81	Zinc phosphodiesterase ELAC protein 2 OS=Mus musculus OX=10090 GN=Elac2 PE=1 SV=1	0.853333333
Micall2	Q3TN34	MICAL-like protein 2 OS=Mus musculus OX=10090 GN=Micall2 PE=1 SV=1	0.816666667
Znf638	Q61464	Zinc finger protein 638 OS=Mus musculus OX=10090 GN=Znf638 PE=1 SV=2	0.836666667
Ncam1	P13595	Neural cell adhesion molecule 1 OS=Mus musculus OX=10090 GN=Ncam1 PE=1 SV=3	0.826666667
Gfm1	Q8K0D5	Elongation factor G mitochondrial OS=Mus musculus OX=10090 GN=Gfm1 PE=1 SV=1	0.986666667
Plcb3	P51432	1-phosphatidylinositol 4 5-bisphosphate phosphodiesterase beta-3 OS=Mus musculus OX=10090 G	0.82
Chd5	A2A8L1	Chromodomain-helicase-DNA-binding protein 5 OS=Mus musculus OX=10090 GN=Chd5 PE=1 SV=1	0.95
Hnrnph1	O35737	Heterogeneous nuclear ribonucleoprotein H OS=Mus musculus OX=10090 GN=Hnrnph1 PE=1 SV=3	0.87
Tmpo	Q61029	Lamina-associated polypeptide 2 isoforms beta/delta/epsilon/gamma OS=Mus musculus OX=1009(	0.88
Tln2	Q71LX4	Talin-2 OS=Mus musculus OX=10090 GN=Tln2 PE=1 SV=3	0.976666667
Pcbp2	Q61990	Poly(rC)-binding protein 2 OS=Mus musculus OX=10090 GN=Pcbp2 PE=1 SV=1	0.833333333
Pfkm	P47857	ATP-dependent 6-phosphofructokinase muscle type OS=Mus musculus OX=10090 GN=Pfkm PE=1 S\	0.9
Hnrnpf	Q9Z2X1	Heterogeneous nuclear ribonucleoprotein F OS=Mus musculus OX=10090 GN=Hnrnpf PE=1 SV=3	0.903333333
Stag2	O35638	Cohesin subunit SA-2 OS=Mus musculus OX=10090 GN=Stag2 PE=1 SV=3	0.943333333
Epb41l1	Q9Z2H5	Band 4.1-like protein 1 OS=Mus musculus OX=10090 GN=Epb41l1 PE=1 SV=2	0.846666667
Slc25a12	Q8BH59	Calcium-binding mitochondrial carrier protein Aralar1 OS=Mus musculus OX=10090 GN=Slc25a12	0.91
Lap3	Q9CPY7	Cytosol aminopeptidase OS=Mus musculus OX=10090 GN=Lap3 PE=1 SV=3	0.893333333
Anxa3	O35639	Annexin A3 OS=Mus musculus OX=10090 GN=Anxa3 PE=1 SV=4	0.916666667
Psmc5	P62196	26S proteasome regulatory subunit 8 OS=Mus musculus OX=10090 GN=Psmc5 PE=1 SV=1	0.883333333
Thrap3	Q569Z6	Thyroid hormone receptor-associated protein 3 OS=Mus musculus OX=10090 GN=Thrap3 PE=1 SV=:	0.856666667
Akr1b1	P45376	Aldo-keto reductase family 1 member B1 OS=Mus musculus OX=10090 GN=Akr1b1 PE=1 SV=3	0.94
Cyb5r3	Q9DCN2	NADH-cytochrome b5 reductase 3 OS=Mus musculus OX=10090 GN=Cyb5r3 PE=1 SV=3	0.873333333

Psat1	Q99K85	Phosphoserine aminotransferase OS=Mus musculus OX=10090 GN=Psat1 PE=1 SV=1	0.866666667
Dkc1	Q9ESX5	H/ACA ribonucleoprotein complex subunit DKC1 OS=Mus musculus OX=10090 GN=Dkc1 PE=1 SV=4	0.883333333
Lpp	Q8BFW7	Lipoma-preferred partner homolog OS=Mus musculus OX=10090 GN=Lpp PE=1 SV=1	0.823333333
Adsl	P54822	Adenylosuccinate lyase OS=Mus musculus OX=10090 GN=Adsl PE=1 SV=2	0.89
Actr3	Q99JY9	Actin-related protein 3 OS=Mus musculus OX=10090 GN=Actr3 PE=1 SV=3	0.9
Slc25a3	Q8VEM8	Phosphate carrier protein mitochondrial OS=Mus musculus OX=10090 GN=Slc25a3 PE=1 SV=1	0.896666667
Prpf19	Q99KP6	Pre-mRNA-processing factor 19 OS=Mus musculus OX=10090 GN=Prpf19 PE=1 SV=1	0.843333333
Washc2	Q6PGL7	WASH complex subunit 2 OS=Mus musculus OX=10090 GN=Washc2 PE=1 SV=1	0.773333333
Prmt1	Q9JIF0	Protein arginine N-methyltransferase 1 OS=Mus musculus OX=10090 GN=Prmt1 PE=1 SV=1	0.906666667
Ola1	Q9CZ30	Obg-like ATPase 1 OS=Mus musculus OX=10090 GN=Ola1 PE=1 SV=1	0.903333333
Sec63	Q8VHE0	Translocation protein SEC63 homolog OS=Mus musculus OX=10090 GN=Sec63 PE=1 SV=4	0.983333333
Rsl1d1	Q8BVY0	Ribosomal L1 domain-containing protein 1 OS=Mus musculus OX=10090 GN=Rsl1d1 PE=1 SV=1	0.866666667
Nup107	Q8BH74	Nuclear pore complex protein Nup107 OS=Mus musculus OX=10090 GN=Nup107 PE=1 SV=1	0.923333333
Ube3a	O08759	Ubiquitin-protein ligase E3A OS=Mus musculus OX=10090 GN=Ube3a PE=1 SV=2	0.923333333
Hnrnpll	Q921F4	Heterogeneous nuclear ribonucleoprotein L-like OS=Mus musculus OX=10090 GN=Hnrnpll PE=1 SV=	0.91
Ran	P62827	GTP-binding nuclear protein Ran OS=Mus musculus OX=10090 GN=Ran PE=1 SV=3	0.883333333
Got1	P05201	Aspartate aminotransferase cytoplasmic OS=Mus musculus OX=10090 GN=Got1 PE=1 SV=3	0.856666667
Lima1	Q9ERG0	LIM domain and actin-binding protein 1 OS=Mus musculus OX=10090 GN=Lima1 PE=1 SV=3	0.886666667
Ly75	Q60767	Lymphocyte antigen 75 OS=Mus musculus OX=10090 GN=Ly75 PE=1 SV=2	0.86
Farsb	Q9WUA2	Phenylalanine--tRNA ligase beta subunit OS=Mus musculus OX=10090 GN=Farsb PE=1 SV=2	0.913333333
Ccar2	Q8VDP4	Cell cycle and apoptosis regulator protein 2 OS=Mus musculus OX=10090 GN=Ccar2 PE=1 SV=2	0.846666667
Atp13a1	Q9EPE9	Manganese-transporting ATPase 13A1 OS=Mus musculus OX=10090 GN=Atp13a1 PE=1 SV=2	0.923333333
Myo1e	E9Q634	Unconventional myosin-Ie OS=Mus musculus OX=10090 GN=Myo1e PE=1 SV=1	0.96
Prmt5	Q8CIG8	Protein arginine N-methyltransferase 5 OS=Mus musculus OX=10090 GN=Prmt5 PE=1 SV=3	0.793333333
Eif4a2	P10630	Eukaryotic initiation factor 4A-II OS=Mus musculus OX=10090 GN=Eif4a2 PE=1 SV=2	0.866666667
Nme2	Q01768	Nucleoside diphosphate kinase B OS=Mus musculus OX=10090 GN=Nme2 PE=1 SV=1	0.886666667
Lmnb2	P21619	Lamin-B2 OS=Mus musculus OX=10090 GN=Lmnb2 PE=1 SV=2	0.956666667
Usp4	P35123	Ubiquitin carboxyl-terminal hydrolase 4 OS=Mus musculus OX=10090 GN=Usp4 PE=1 SV=3	0.866666667
Serpinb6	Q60854	Serpin B6 OS=Mus musculus OX=10090 GN=Serpinb6 PE=1 SV=1	0.88
Fxr1	Q61584	Fragile X mental retardation syndrome-related protein 1 OS=Mus musculus OX=10090 GN=Fxr1 PE=	0.883333333
Trio	Q0KL02	Triple functional domain protein OS=Mus musculus OX=10090 GN=Trio PE=1 SV=3	0.92
Ptgs2	Q05769	Prostaglandin G/H synthase 2 OS=Mus musculus OX=10090 GN=Ptgs2 PE=1 SV=1	0.99
Serbp1	Q9CY58	Plasminogen activator inhibitor 1 RNA-binding protein OS=Mus musculus OX=10090 GN=Serbp1 PE	0.866666667
Kif15	Q6P9L6	Kinesin-like protein KIF15 OS=Mus musculus OX=10090 GN=Kif15 PE=1 SV=1	0.896666667

Myo9b	Q9QY06	Unconventional myosin-IXb OS=Mus musculus OX=10090 GN=Myo9b PE=1 SV=2	0.906666667
Ube3c	Q80U95	Ubiquitin-protein ligase E3C OS=Mus musculus OX=10090 GN=Ube3c PE=1 SV=2	0.943333333
Uba2	Q9Z1F9	SUMO-activating enzyme subunit 2 OS=Mus musculus OX=10090 GN=Uba2 PE=1 SV=1	0.893333333
Nop58	Q6DFW4	Nucleolar protein 58 OS=Mus musculus OX=10090 GN=Nop58 PE=1 SV=1	0.893333333
Zyx	Q62523	Zyxin OS=Mus musculus OX=10090 GN=Zyx PE=1 SV=2	0.783333333
Psmc3	O88685	26S proteasome regulatory subunit 6A OS=Mus musculus OX=10090 GN=Psmc3 PE=1 SV=2	0.906666667
Got2	P05202	Aspartate aminotransferase mitochondrial OS=Mus musculus OX=10090 GN=Got2 PE=1 SV=1	0.9
Pdlim1	O70400	PDZ and LIM domain protein 1 OS=Mus musculus OX=10090 GN=Pdlim1 PE=1 SV=4	0.853333333
Psmc2	P46471	26S proteasome regulatory subunit 7 OS=Mus musculus OX=10090 GN=Psmc2 PE=1 SV=5	0.896666667
Ppa1	Q9D819	Inorganic pyrophosphatase OS=Mus musculus OX=10090 GN=Ppa1 PE=1 SV=1	0.9
Puf60	Q3UEB3	Poly(U)-binding-splicing factor PUF60 OS=Mus musculus OX=10090 GN=Puf60 PE=1 SV=2	0.883333333
Psmd12	Q9D8W5	26S proteasome non-ATPase regulatory subunit 12 OS=Mus musculus OX=10090 GN=Psmd12 PE=1	0.896666667
Gpi	P06745	Glucose-6-phosphate isomerase OS=Mus musculus OX=10090 GN=Gpi PE=1 SV=4	0.886666667
Snx9	Q91VH2	Sorting nexin-9 OS=Mus musculus OX=10090 GN=Snx9 PE=1 SV=1	0.913333333
Ssb	P32067	Lupus La protein homolog OS=Mus musculus OX=10090 GN=Ssb PE=1 SV=1	0.88
Aacs	Q9D2R0	Acetoacetyl-CoA synthetase OS=Mus musculus OX=10090 GN=Aacs PE=1 SV=1	0.86
Ctnnb1	Q02248	Catenin beta-1 OS=Mus musculus OX=10090 GN=Ctnnb1 PE=1 SV=1	0.903333333
Psmd3	P14685	26S proteasome non-ATPase regulatory subunit 3 OS=Mus musculus OX=10090 GN=Psmd3 PE=1 SV	0.89
Wdr3	Q8BHB4	WD repeat-containing protein 3 OS=Mus musculus OX=10090 GN=Wdr3 PE=1 SV=1	0.913333333
Bag6	Q9Z1R2	Large proline-rich protein BAG6 OS=Mus musculus OX=10090 GN=Bag6 PE=1 SV=1	0.83
Sympk	Q80X82	Symplekin OS=Mus musculus OX=10090 GN=Sympk PE=1 SV=2	0.873333333
Stat3	P42227	Signal transducer and activator of transcription 3 OS=Mus musculus OX=10090 GN=Stat3 PE=1 SV=2	0.94
Son	Q9QX47	Protein SON OS=Mus musculus OX=10090 GN=Son PE=1 SV=2	0.833333333
Ndufs1	Q91VD9	NADH-ubiquinone oxidoreductase 75 kDa subunit mitochondrial OS=Mus musculus OX=10090 GN	0.893333333
Tbc1d15	Q9CXF4	TBC1 domain family member 15 OS=Mus musculus OX=10090 GN=Tbc1d15 PE=1 SV=1	0.89
Rnpep	Q8VCT3	Aminopeptidase B OS=Mus musculus OX=10090 GN=Rnpep PE=1 SV=2	0.92
Bop1	P97452	Ribosome biogenesis protein BOP1 OS=Mus musculus OX=10090 GN=Bop1 PE=1 SV=1	0.866666667
Xpot	Q9CRT8	Exportin-T OS=Mus musculus OX=10090 GN=Xpot PE=1 SV=3	0.896666667
Washc5	Q8C2E7	WASH complex subunit 5 OS=Mus musculus OX=10090 GN=Washc5 PE=1 SV=2	0.846666667
Xpo7	Q9EPK7	Exportin-7 OS=Mus musculus OX=10090 GN=Xpo7 PE=1 SV=3	0.846666667
Mon2	Q80TL7	Protein MON2 homolog OS=Mus musculus OX=10090 GN=Mon2 PE=1 SV=2	0.963333333
Gak	Q99KY4	Cyclin-G-associated kinase OS=Mus musculus OX=10090 GN=Gak PE=1 SV=2	0.956666667
Usp24	B1AY13	Ubiquitin carboxyl-terminal hydrolase 24 OS=Mus musculus OX=10090 GN=Usp24 PE=1 SV=1	0.996666667
Esyt1	Q3U7R1	Extended synaptotagmin-1 OS=Mus musculus OX=10090 GN=Esyt1 PE=1 SV=2	0.853333333

Srrt	Q99MR6	Serrate RNA effector molecule homolog OS=Mus musculus OX=10090 GN=Srrt PE=1 SV=1	0.84
Septin11	Q8C1B7	Septin-11 OS=Mus musculus OX=10090 GN=Septin11 PE=1 SV=4	0.893333333
Atp2b1	G5E829	Plasma membrane calcium-transporting ATPase 1 OS=Mus musculus OX=10090 GN=Atp2b1 PE=1 S\	0.83
Mta2	Q9R190	Metastasis-associated protein MTA2 OS=Mus musculus OX=10090 GN=Mta2 PE=1 SV=1	0.936666667
Pygb	Q8CI94	Glycogen phosphorylase brain form OS=Mus musculus OX=10090 GN=Pygb PE=1 SV=3	0.863333333
Khsrp	Q3U0V1	Far upstream element-binding protein 2 OS=Mus musculus OX=10090 GN=Khsrp PE=1 SV=2	0.86
Nasp	Q99MD9	Nuclear autoantigenic sperm protein OS=Mus musculus OX=10090 GN=Nasp PE=1 SV=2	0.863333333
Fubp1	Q91WJ8	Far upstream element-binding protein 1 OS=Mus musculus OX=10090 GN=Fubp1 PE=1 SV=1	0.866666667
Ankrd17	Q99NH0	Ankyrin repeat domain-containing protein 17 OS=Mus musculus OX=10090 GN=Ankrd17 PE=1 SV=2	0.993333333
Ppia	P17742	Peptidyl-prolyl cis-trans isomerase A OS=Mus musculus OX=10090 GN=Ppia PE=1 SV=2	0.916666667
Itgb1	P09055	Integrin beta-1 OS=Mus musculus OX=10090 GN=Itgb1 PE=1 SV=1	0.863333333
Aldh2	P47738	Aldehyde dehydrogenase mitochondrial OS=Mus musculus OX=10090 GN=Aldh2 PE=1 SV=1	0.89
Col6a1	Q04857	Collagen alpha-1(VI) chain OS=Mus musculus OX=10090 GN=Col6a1 PE=1 SV=1	0.89
Hsd17b4	P51660	Peroxisomal multifunctional enzyme type 2 OS=Mus musculus OX=10090 GN=Hsd17b4 PE=1 SV=3	0.873333333
Ltbp1	Q8CG19	Latent-transforming growth factor beta-binding protein 1 OS=Mus musculus OX=10090 GN=Ltbp1 I	0.81
Ipo9	Q91YE6	Importin-9 OS=Mus musculus OX=10090 GN=Ipo9 PE=1 SV=3	0.916666667
Tufm	Q8BFR5	Elongation factor Tu mitochondrial OS=Mus musculus OX=10090 GN=Tufm PE=1 SV=1	0.876666667
Atxn10	P28658	Ataxin-10 OS=Mus musculus OX=10090 GN=Atxn10 PE=1 SV=2	0.88
Srp68	Q8BMA6	Signal recognition particle subunit SRP68 OS=Mus musculus OX=10090 GN=Srp68 PE=1 SV=2	0.896666667
Cdc5l	Q6A068	Cell division cycle 5-like protein OS=Mus musculus OX=10090 GN=Cdc5l PE=1 SV=2	0.853333333
Ptpn11	P35235	Tyrosine-protein phosphatase non-receptor type 11 OS=Mus musculus OX=10090 GN=Ptpn11 PE=1	0.93
Supt5h	O55201	Transcription elongation factor SPT5 OS=Mus musculus OX=10090 GN=Supt5h PE=1 SV=1	0.793333333
Nudcd1	Q6PIP5	NudC domain-containing protein 1 OS=Mus musculus OX=10090 GN=Nudcd1 PE=1 SV=2	0.943333333
Lamc1	P02468	Laminin subunit gamma-1 OS=Mus musculus OX=10090 GN=Lamc1 PE=1 SV=2	0.976666667
Ctdp1	Q7TSG2	RNA polymerase II subunit A C-terminal domain phosphatase OS=Mus musculus OX=10090 GN=Ctd	0.9
Gtpbp4	Q99ME9	Nucleolar GTP-binding protein 1 OS=Mus musculus OX=10090 GN=Gtpbp4 PE=1 SV=3	0.863333333
Naa25	Q8BWZ3	N-alpha-acetyltransferase 25 NatB auxiliary subunit OS=Mus musculus OX=10090 GN=Naa25 PE=1 S	0.9
Ankfy1	Q810B6	Rabankyrin-5 OS=Mus musculus OX=10090 GN=Ankfy1 PE=1 SV=2	0.88
Hells	Q60848	Lymphocyte-specific helicase OS=Mus musculus OX=10090 GN=Hells PE=1 SV=2	1.006666667
Nop2	Q922K7	Probable 28S rRNA (cytosine-C(5))-methyltransferase OS=Mus musculus OX=10090 GN=Nop2 PE=1 S	0.926666667
Ptk7	Q8BKG3	Inactive tyrosine-protein kinase 7 OS=Mus musculus OX=10090 GN=Ptk7 PE=1 SV=1	0.886666667
Dnm1	P39053	Dynamin-1 OS=Mus musculus OX=10090 GN=Dnm1 PE=1 SV=2	0.85
Ago2	Q8CJG0	Protein argonaute-2 OS=Mus musculus OX=10090 GN=Ago2 PE=1 SV=3	0.87
Hnrnpa3	Q8BG05	Heterogeneous nuclear ribonucleoprotein A3 OS=Mus musculus OX=10090 GN=Hnrnpa3 PE=1 SV=1	0.886666667

Txnrd1	Q9JMH6	Thioredoxin reductase 1 cytoplasmic OS=Mus musculus OX=10090 GN=Txnrd1 PE=1 SV=3	0.886666667
Hspa4l	P48722	Heat shock 70 kDa protein 4L OS=Mus musculus OX=10090 GN=Hspa4l PE=1 SV=2	0.89
Thbs1	P35441	Thrombospondin-1 OS=Mus musculus OX=10090 GN=Thbs1 PE=1 SV=1	0.753333333
Nono	Q99K48	Non-POU domain-containing octamer-binding protein OS=Mus musculus OX=10090 GN=Nono PE=1	0.85
Calu	O35887	Calumenin OS=Mus musculus OX=10090 GN=Calu PE=1 SV=1	0.846666667
Esd	Q9R0P3	S-formylglutathione hydrolase OS=Mus musculus OX=10090 GN=Esd PE=1 SV=1	0.903333333
Rpl3	P27659	60S ribosomal protein L3 OS=Mus musculus OX=10090 GN=Rpl3 PE=1 SV=3	0.91
Eif2s2	Q99L45	Eukaryotic translation initiation factor 2 subunit 2 OS=Mus musculus OX=10090 GN=Eif2s2 PE=1 SV	0.87
Dpp3	Q99KK7	Dipeptidyl peptidase 3 OS=Mus musculus OX=10090 GN=Dpp3 PE=1 SV=2	0.893333333
Hadha	Q8BMS1	Trifunctional enzyme subunit alpha mitochondrial OS=Mus musculus OX=10090 GN=Hadha PE=1 S	0.92
Arhgef2	Q60875	Rho guanine nucleotide exchange factor 2 OS=Mus musculus OX=10090 GN=Arhgef2 PE=1 SV=4	0.85
Kif11	Q6P9P6	Kinesin-like protein KIF11 OS=Mus musculus OX=10090 GN=Kif11 PE=1 SV=1	0.916666667
Ddx6	P54823	Probable ATP-dependent RNA helicase DDX6 OS=Mus musculus OX=10090 GN=Ddx6 PE=1 SV=1	0.86
Tcof1	O08784	Treacle protein OS=Mus musculus OX=10090 GN=Tcof1 PE=1 SV=1	0.733333333
Ptpn23	Q6PB44	Tyrosine-protein phosphatase non-receptor type 23 OS=Mus musculus OX=10090 GN=Ptpn23 PE=1	0.813333333
Heatr5a	Q5PRF0	HEAT repeat-containing protein 5A OS=Mus musculus OX=10090 GN=Heatr5a PE=1 SV=2	0.843333333
Lonp1	Q8CGK3	Lon protease homolog mitochondrial OS=Mus musculus OX=10090 GN=Lonp1 PE=1 SV=2	0.89
Nup160	Q9Z0W3	Nuclear pore complex protein Nup160 OS=Mus musculus OX=10090 GN=Nup160 PE=1 SV=2	0.846666667
Lnpep	Q8C129	Leucyl-cystinyl aminopeptidase OS=Mus musculus OX=10090 GN=Lnpep PE=1 SV=1	0.876666667
Rif1	Q6PR54	Telomere-associated protein RIF1 OS=Mus musculus OX=10090 GN=Rif1 PE=1 SV=2	0.846666667
H4f16	P62806	Histone H4 OS=Mus musculus OX=10090 GN=H4c1 PE=1 SV=2	0.93
Cep170	Q6A065	Centrosomal protein of 170 kDa OS=Mus musculus OX=10090 GN=Cep170 PE=1 SV=2	0.846666667
Sart3	Q9JLI8	Squamous cell carcinoma antigen recognized by T-cells 3 OS=Mus musculus OX=10090 GN=Sart3 PE	0.866666667
Msh2	P43247	DNA mismatch repair protein Msh2 OS=Mus musculus OX=10090 GN=Msh2 PE=1 SV=1	0.866666667
Rad50	P70388	DNA repair protein RAD50 OS=Mus musculus OX=10090 GN=Rad50 PE=1 SV=1	0.886666667
Adnp	Q9Z103	Activity-dependent neuroprotector homeobox protein OS=Mus musculus OX=10090 GN=Adnp PE=1	0.943333333
Thada	A8C756	Thyroid adenoma-associated protein homolog OS=Mus musculus OX=10090 GN=Thada PE=1 SV=1	0.87
Pkn2	Q8BWW9	Serine/threonine-protein kinase N2 OS=Mus musculus OX=10090 GN=Pkn2 PE=1 SV=3	0.926666667
Hspa2	P17156	Heat shock-related 70 kDa protein 2 OS=Mus musculus OX=10090 GN=Hspa2 PE=1 SV=2	0.61
Ddx39b	Q9Z1N5	Spliceosome RNA helicase Ddx39b OS=Mus musculus OX=10090 GN=Ddx39b PE=1 SV=1	0.853333333
Smarcc2	Q6PDG5	SWI/SNF complex subunit SMARCC2 OS=Mus musculus OX=10090 GN=Smarcc2 PE=1 SV=2	0.823333333
Pgam1	Q9DBJ1	Phosphoglycerate mutase 1 OS=Mus musculus OX=10090 GN=Pgam1 PE=1 SV=3	0.846666667
Eif4a3	Q91VC3	Eukaryotic initiation factor 4A-III OS=Mus musculus OX=10090 GN=Eif4a3 PE=1 SV=3	0.906666667
Dpysl2	O08553	Dihydropyrimidinase-related protein 2 OS=Mus musculus OX=10090 GN=Dpysl2 PE=1 SV=2	0.87

Dpysl3	Q62188	Dihydropyrimidinase-related protein 3 OS=Mus musculus OX=10090 GN=Dpysl3 PE=1 SV=1	0.85
Pcbp1	P60335	Poly(rC)-binding protein 1 OS=Mus musculus OX=10090 GN=Pcbp1 PE=1 SV=1	0.826666667
G3bp1	P97855	Ras GTPase-activating protein-binding protein 1 OS=Mus musculus OX=10090 GN=G3bp1 PE=1 SV=1	0.876666667
Sec23b	Q9D662	Protein transport protein Sec23B OS=Mus musculus OX=10090 GN=Sec23b PE=1 SV=1	0.89
Ctps1	P70698	CTP synthase 1 OS=Mus musculus OX=10090 GN=Ctps1 PE=1 SV=2	0.886666667
Shmt2	Q9CZN7	Serine hydroxymethyltransferase mitochondrial OS=Mus musculus OX=10090 GN=Shmt2 PE=1 SV=	0.903333333
Thop1	Q8C1A5	Thimet oligopeptidase OS=Mus musculus OX=10090 GN=Thop1 PE=1 SV=1	0.886666667
Nsf	P46460	Vesicle-fusing ATPase OS=Mus musculus OX=10090 GN=Nsf PE=1 SV=2	0.893333333
Npm1	Q61937	Nucleophosmin OS=Mus musculus OX=10090 GN=Npm1 PE=1 SV=1	0.903333333
Lta4h	P24527	Leukotriene A-4 hydrolase OS=Mus musculus OX=10090 GN=Lta4h PE=1 SV=4	0.853333333
Rnh1	Q91VI7	Ribonuclease inhibitor OS=Mus musculus OX=10090 GN=Rnh1 PE=1 SV=1	0.886666667
Ugdh	O70475	UDP-glucose 6-dehydrogenase OS=Mus musculus OX=10090 GN=Ugdh PE=1 SV=1	0.873333333
Syncrip	Q7TMK9	Heterogeneous nuclear ribonucleoprotein Q OS=Mus musculus OX=10090 GN=Syncrip PE=1 SV=2	0.88
Tfrc	Q62351	Transferrin receptor protein 1 OS=Mus musculus OX=10090 GN=Tfrc PE=1 SV=1	0.91
Sdha	Q8K2B3	Succinate dehydrogenase [ubiquinone] flavoprotein subunit mitochondrial OS=Mus musculus OX=	0.936666667
Niban2	Q8R1F1	Protein Niban 2 OS=Mus musculus OX=10090 GN=Niban2 PE=1 SV=2	0.88
Tbl3	Q8C4J7	Transducin beta-like protein 3 OS=Mus musculus OX=10090 GN=Tbl3 PE=2 SV=1	0.9
Tnpo3	Q6P2B1	Transportin-3 OS=Mus musculus OX=10090 GN=Tnpo3 PE=1 SV=1	0.893333333
Pla2g4a	P47713	Cytosolic phospholipase A2 OS=Mus musculus OX=10090 GN=Pla2g4a PE=1 SV=1	0.883333333
Atxn2l	Q7TQH0	Ataxin-2-like protein OS=Mus musculus OX=10090 GN=Atxn2l PE=1 SV=1	0.836666667
Pck2	Q8BH04	Phosphoenolpyruvate carboxykinase [GTP] mitochondrial OS=Mus musculus OX=10090 GN=Pck2 P	0.89
Larp1	Q6ZQ58	La-related protein 1 OS=Mus musculus OX=10090 GN=Larp1 PE=1 SV=3	0.85
Capn1	O35350	Calpain-1 catalytic subunit OS=Mus musculus OX=10090 GN=Capn1 PE=1 SV=1	0.853333333
Fermt2	Q8CIB5	Fermitin family homolog 2 OS=Mus musculus OX=10090 GN=Fermt2 PE=1 SV=1	0.856666667
Ddx19a	Q61655	ATP-dependent RNA helicase DDX19A OS=Mus musculus OX=10090 GN=Ddx19a PE=1 SV=2	0.876666667
Slc25a4	P48962	ADP/ATP translocase 1 OS=Mus musculus OX=10090 GN=Slc25a4 PE=1 SV=4	0.916666667
Ezr	P26040	Ezrin OS=Mus musculus OX=10090 GN=Ezr PE=1 SV=3	0.903333333
Dnm2	P39054	Dynamin-2 OS=Mus musculus OX=10090 GN=Dnm2 PE=1 SV=2	0.843333333
Rbbp7	Q60973	Histone-binding protein RBBP7 OS=Mus musculus OX=10090 GN=Rbbp7 PE=1 SV=1	0.87
Plxna1	P70206	Plexin-A1 OS=Mus musculus OX=10090 GN=Plxna1 PE=1 SV=1	0.89
Cfl1	P18760	Cofilin-1 OS=Mus musculus OX=10090 GN=Cfl1 PE=1 SV=3	0.87
Ehd2	Q8BH64	EH domain-containing protein 2 OS=Mus musculus OX=10090 GN=Ehd2 PE=1 SV=1	0.823333333
Cul7	Q8VE73	Cullin-7 OS=Mus musculus OX=10090 GN=Cul7 PE=1 SV=2	0.766666667
Inf2	Q0GNC1	Inverted formin-2 OS=Mus musculus OX=10090 GN=Inf2 PE=1 SV=1	0.876666667

Ruvbl2	Q9WTM5	RuvB-like 2 OS=Mus musculus OX=10090 GN=Ruvbl2 PE=1 SV=3	0.886666667
Kif4	P33174	Chromosome-associated kinesin KIF4 OS=Mus musculus OX=10090 GN=Kif4 PE=1 SV=3	0.883333333
Elp1	Q7TT37	Elongator complex protein 1 OS=Mus musculus OX=10090 GN=Elp1 PE=1 SV=2	0.84
Ppp6r3	Q922D4	Serine/threonine-protein phosphatase 6 regulatory subunit 3 OS=Mus musculus OX=10090 GN=Ppp	0.933333333
Hnrnpk	P61979	Heterogeneous nuclear ribonucleoprotein K OS=Mus musculus OX=10090 GN=Hnrnpk PE=1 SV=1	0.873333333
Asns	Q61024	Asparagine synthetase [glutamine-hydrolyzing] OS=Mus musculus OX=10090 GN=Asns PE=1 SV=3	0.863333333
Eif3l	Q8QZY1	Eukaryotic translation initiation factor 3 subunit L OS=Mus musculus OX=10090 GN=Eif3l PE=1 SV=1	0.923333333
Rpl4	Q9D8E6	60S ribosomal protein L4 OS=Mus musculus OX=10090 GN=Rpl4 PE=1 SV=3	0.88
Gls	D3Z7P3	Glutaminase kidney isoform mitochondrial OS=Mus musculus OX=10090 GN=Gls PE=1 SV=1	0.886666667
Nup133	Q8R0G9	Nuclear pore complex protein Nup133 OS=Mus musculus OX=10090 GN=Nup133 PE=1 SV=2	0.91
Por	P37040	NADPH--cytochrome P450 reductase OS=Mus musculus OX=10090 GN=Por PE=1 SV=2	0.88
Cul3	Q9JLV5	Cullin-3 OS=Mus musculus OX=10090 GN=Cul3 PE=1 SV=1	0.896666667
Abce1	P61222	ATP-binding cassette sub-family E member 1 OS=Mus musculus OX=10090 GN=Abce1 PE=1 SV=1	0.903333333
Ddx46	Q569Z5	Probable ATP-dependent RNA helicase DDX46 OS=Mus musculus OX=10090 GN=Ddx46 PE=1 SV=2	0.9
Nat10	Q8K224	RNA cytidine acetyltransferase OS=Mus musculus OX=10090 GN=Nat10 PE=1 SV=1	0.883333333
Arcn1	Q5XJY5	Coatomer subunit delta OS=Mus musculus OX=10090 GN=Arcn1 PE=1 SV=2	0.88
Lig1	P37913	DNA ligase 1 OS=Mus musculus OX=10090 GN=Lig1 PE=1 SV=2	0.853333333
Top1	Q04750	DNA topoisomerase 1 OS=Mus musculus OX=10090 GN=Top1 PE=1 SV=2	0.95
Ide	Q9JHR7	Insulin-degrading enzyme OS=Mus musculus OX=10090 GN=Ide PE=1 SV=1	0.876666667
Ubr5	Q80TP3	E3 ubiquitin-protein ligase UBR5 OS=Mus musculus OX=10090 GN=Ubr5 PE=1 SV=2	0.86
Ephx1	Q9D379	Epoxide hydrolase 1 OS=Mus musculus OX=10090 GN=Ephx1 PE=1 SV=2	0.85
Yars	Q91WQ3	Tyrosine--tRNA ligase cytoplasmic OS=Mus musculus OX=10090 GN=Yars1 PE=1 SV=3	0.833333333
Ap3b1	Q9Z1T1	AP-3 complex subunit beta-1 OS=Mus musculus OX=10090 GN=Ap3b1 PE=1 SV=2	0.89
Cdc42bpb	Q7TT50	Serine/threonine-protein kinase MRCK beta OS=Mus musculus OX=10090 GN=Cdc42bpb PE=1 SV=2	1.02
Gtf2i	Q9ESZ8	General transcription factor II-I OS=Mus musculus OX=10090 GN=Gtf2i PE=1 SV=3	0.9
Actbl2	Q8BFZ3	Beta-actin-like protein 2 OS=Mus musculus OX=10090 GN=Actbl2 PE=1 SV=1	2.4
Ddx39a	Q8VDW0	ATP-dependent RNA helicase DDX39A OS=Mus musculus OX=10090 GN=Ddx39a PE=1 SV=1	0.856666667
Slc25a5	P51881	ADP/ATP translocase 2 OS=Mus musculus OX=10090 GN=Slc25a5 PE=1 SV=3	0.84
Pfkl	P12382	ATP-dependent 6-phosphofructokinase liver type OS=Mus musculus OX=10090 GN=Pfkl PE=1 SV=4	0.89
Ilf3	Q9Z1X4	Interleukin enhancer-binding factor 3 OS=Mus musculus OX=10090 GN=Ilf3 PE=1 SV=2	0.84
Sec23a	Q01405	Protein transport protein Sec23A OS=Mus musculus OX=10090 GN=Sec23a PE=1 SV=2	0.876666667
Idh2	P54071	Isocitrate dehydrogenase [NADP] mitochondrial OS=Mus musculus OX=10090 GN=Idh2 PE=1 SV=3	0.873333333
Ptbp1	P17225	Polypyrimidine tract-binding protein 1 OS=Mus musculus OX=10090 GN=Ptbp1 PE=1 SV=2	0.88
Paics	Q9DCL9	Multifunctional protein ADE2 OS=Mus musculus OX=10090 GN=Paics PE=1 SV=4	0.873333333

Rack1	P68040	Receptor of activated protein C kinase 1 OS=Mus musculus OX=10090 GN=Rack1 PE=1 SV=3	0.876666667
Syne2	Q6ZWQ0	Nesprin-2 OS=Mus musculus OX=10090 GN=Syne2 PE=1 SV=2	0.956666667
Serpinh1	P19324	Serpin H1 OS=Mus musculus OX=10090 GN=Serpinh1 PE=1 SV=3	0.906666667
Cct6a	P80317	T-complex protein 1 subunit zeta OS=Mus musculus OX=10090 GN=Cct6a PE=1 SV=3	0.913333333
Eif3c	Q8R1B4	Eukaryotic translation initiation factor 3 subunit C OS=Mus musculus OX=10090 GN=Eif3c PE=1 SV=	0.92
Rpn2	Q9DBG6	Dolichyl-diphosphooligosaccharide--protein glycosyltransferase subunit 2 OS=Mus musculus OX=1(	0.873333333
P4ha1	Q60715	Prolyl 4-hydroxylase subunit alpha-1 OS=Mus musculus OX=10090 GN=P4ha1 PE=1 SV=2	0.866666667
Fscn1	Q61553	Fascin OS=Mus musculus OX=10090 GN=Fscn1 PE=1 SV=4	0.93
Capn2	O08529	Calpain-2 catalytic subunit OS=Mus musculus OX=10090 GN=Capn2 PE=1 SV=4	0.9
Nop56	Q9D6Z1	Nucleolar protein 56 OS=Mus musculus OX=10090 GN=Nop56 PE=1 SV=2	0.923333333
Plaa	P27612	Phospholipase A-2-activating protein OS=Mus musculus OX=10090 GN=Plaa PE=1 SV=4	0.906666667
Cd109	Q8R422	CD109 antigen OS=Mus musculus OX=10090 GN=Cd109 PE=1 SV=1	0.856666667
Prpf6	Q91YR7	Pre-mRNA-processing factor 6 OS=Mus musculus OX=10090 GN=Prpf6 PE=1 SV=1	0.813333333
Mtrex	Q9CZU3	Exosome RNA helicase MTR4 OS=Mus musculus OX=10090 GN=Mtrex PE=1 SV=1	0.846666667
Polr2b	Q8CFI7	DNA-directed RNA polymerase II subunit RPB2 OS=Mus musculus OX=10090 GN=Polr2b PE=1 SV=2	0.886666667
Acin1	Q9JIX8	Apoptotic chromatin condensation inducer in the nucleus OS=Mus musculus OX=10090 GN=Acin1	0.853333333
Usp7	Q6A4J8	Ubiquitin carboxyl-terminal hydrolase 7 OS=Mus musculus OX=10090 GN=Usp7 PE=1 SV=1	0.863333333
Golga3	P55937	Golgin subfamily A member 3 OS=Mus musculus OX=10090 GN=Golga3 PE=1 SV=3	1.02
Mia3	Q8BI84	Transport and Golgi organization protein 1 homolog OS=Mus musculus OX=10090 GN=Mia3 PE=1 S'	0.823333333
Cobll1	Q3UMF0	Cordon-bleu protein-like 1 OS=Mus musculus OX=10090 GN=Cobll1 PE=1 SV=2	0.796666667
Ap1b1	O35643	AP-1 complex subunit beta-1 OS=Mus musculus OX=10090 GN=Ap1b1 PE=1 SV=2	0.883333333
Epb41l3	Q9WV92	Band 4.1-like protein 3 OS=Mus musculus OX=10090 GN=Epb41l3 PE=1 SV=1	0.86
Spag9	Q58A65	C-Jun-amino-terminal kinase-interacting protein 4 OS=Mus musculus OX=10090 GN=Spag9 PE=1 SV	0.886666667
Pcm1	Q9R0L6	Pericentriolar material 1 protein OS=Mus musculus OX=10090 GN=Pcm1 PE=1 SV=2	0.946666667
Slk	O54988	STE20-like serine/threonine-protein kinase OS=Mus musculus OX=10090 GN=Slk PE=1 SV=2	0.903333333
Dhx15	O35286	Pre-mRNA-splicing factor ATP-dependent RNA helicase DHX15 OS=Mus musculus OX=10090 GN=Dh	0.913333333
Tbcd	Q8BYA0	Tubulin-specific chaperone D OS=Mus musculus OX=10090 GN=Tbcd PE=1 SV=1	0.98
Atp5f1b	P56480	ATP synthase subunit beta mitochondrial OS=Mus musculus OX=10090 GN=Atp5f1b PE=1 SV=2	0.9
Eps8	Q08509	Epidermal growth factor receptor kinase substrate 8 OS=Mus musculus OX=10090 GN=Eps8 PE=1 SV	0.803333333
Ttll12	Q3UDE2	Tubulin--tyrosine ligase-like protein 12 OS=Mus musculus OX=10090 GN=Ttll12 PE=1 SV=1	0.873333333
Glud1	P26443	Glutamate dehydrogenase 1 mitochondrial OS=Mus musculus OX=10090 GN=Glud1 PE=1 SV=1	0.9
Nup155	Q99P88	Nuclear pore complex protein Nup155 OS=Mus musculus OX=10090 GN=Nup155 PE=1 SV=1	0.866666667
Cul2	Q9D4H8	Cullin-2 OS=Mus musculus OX=10090 GN=Cul2 PE=1 SV=2	0.873333333
Pdxdc1	Q99K01	Pyridoxal-dependent decarboxylase domain-containing protein 1 OS=Mus musculus OX=10090 GN	0.93

Usp14	Q9JMA1	Ubiquitin carboxyl-terminal hydrolase 14 OS=Mus musculus OX=10090 GN=Usp14 PE=1 SV=3	0.843333333
Ddx42	Q810A7	ATP-dependent RNA helicase DDX42 OS=Mus musculus OX=10090 GN=Ddx42 PE=1 SV=3	0.9
Ube4b	Q9ES00	Ubiquitin conjugation factor E4 B OS=Mus musculus OX=10090 GN=Ube4b PE=1 SV=3	0.846666667
Sf3a1	Q8K4Z5	Splicing factor 3A subunit 1 OS=Mus musculus OX=10090 GN=Sf3a1 PE=1 SV=1	0.87
Cspg4	Q8VHY0	Chondroitin sulfate proteoglycan 4 OS=Mus musculus OX=10090 GN=Cspg4 PE=1 SV=3	0.9
Ahctf1	Q8CJF7	Protein ELYS OS=Mus musculus OX=10090 GN=Ahctf1 PE=1 SV=1	0.966666667
Supt6h	Q62383	Transcription elongation factor SPT6 OS=Mus musculus OX=10090 GN=Supt6h PE=1 SV=2	0.953333333
Smarca4	Q3TKT4	Transcription activator BRG1 OS=Mus musculus OX=10090 GN=Smarca4 PE=1 SV=1	0.95
Rdx	P26043	Radixin OS=Mus musculus OX=10090 GN=Rdx PE=1 SV=3	0.873333333
Ehd1	Q9WVK4	EH domain-containing protein 1 OS=Mus musculus OX=10090 GN=Ehd1 PE=1 SV=1	0.863333333
Smarcc1	P97496	SWI/SNF complex subunit SMARCC1 OS=Mus musculus OX=10090 GN=Smarcc1 PE=1 SV=2	0.816666667
Hk1	P17710	Hexokinase-1 OS=Mus musculus OX=10090 GN=Hk1 PE=1 SV=3	0.886666667
Trap1	Q9CQN1	Heat shock protein 75 kDa mitochondrial OS=Mus musculus OX=10090 GN=Trap1 PE=1 SV=1	0.9
Ppp1r12a	Q9DBR7	Protein phosphatase 1 regulatory subunit 12A OS=Mus musculus OX=10090 GN=Ppp1r12a PE=1 SV	0.87
Lrrfip1	Q3UZ39	Leucine-rich repeat flightless-interacting protein 1 OS=Mus musculus OX=10090 GN=Lrrfip1 PE=1 S	0.773333333
Septin9	Q80UG5	Septin-9 OS=Mus musculus OX=10090 GN=Septin9 PE=1 SV=1	0.886666667
Qars	Q8BML9	Glutamine--tRNA ligase OS=Mus musculus OX=10090 GN=Qars1 PE=1 SV=1	0.923333333
Cct2	P80314	T-complex protein 1 subunit beta OS=Mus musculus OX=10090 GN=Cct2 PE=1 SV=4	0.9
Hnrnpl	Q8R081	Heterogeneous nuclear ribonucleoprotein L OS=Mus musculus OX=10090 GN=Hnrnpl PE=1 SV=2	0.876666667
Pa2g4	P50580	Proliferation-associated protein 2G4 OS=Mus musculus OX=10090 GN=Pa2g4 PE=1 SV=3	0.9
Lgals1	P16045	Galectin-1 OS=Mus musculus OX=10090 GN=Lgals1 PE=1 SV=3	0.87
Cul1	Q9WTX6	Cullin-1 OS=Mus musculus OX=10090 GN=Cul1 PE=1 SV=1	0.946666667
Kars1	Q99MN1	Lysine--tRNA ligase OS=Mus musculus OX=10090 GN=Kars1 PE=1 SV=1	0.906666667
Palld	Q9ET54	Palladin OS=Mus musculus OX=10090 GN=Palld PE=1 SV=2	0.803333333
Nup214	Q80U93	Nuclear pore complex protein Nup214 OS=Mus musculus OX=10090 GN=Nup214 PE=1 SV=2	0.853333333
Tmpo	Q61033	Lamina-associated polypeptide 2 isoforms alpha/zeta OS=Mus musculus OX=10090 GN=Tmpo PE=1	0.816666667
Ddx17	Q501J6	Probable ATP-dependent RNA helicase DDX17 OS=Mus musculus OX=10090 GN=Ddx17 PE=1 SV=1	0.893333333
Ppp2r1a	Q76MZ3	Serine/threonine-protein phosphatase 2A 65 kDa regulatory subunit A alpha isoform OS=Mus muscu	0.823333333
Gfpt1	P47856	Glutamine--fructose-6-phosphate aminotransferase [isomerizing] 1 OS=Mus musculus OX=10090 GN	0.9
Clip1	Q922J3	CAP-Gly domain-containing linker protein 1 OS=Mus musculus OX=10090 GN=Clip1 PE=1 SV=1	0.88
Impdh2	P24547	Inosine-5'-monophosphate dehydrogenase 2 OS=Mus musculus OX=10090 GN=Impdh2 PE=1 SV=2	0.926666667
Cand2	Q6ZQ73	Cullin-associated NEDD8-dissociated protein 2 OS=Mus musculus OX=10090 GN=Cand2 PE=1 SV=2	0.826666667
Phgdh	Q61753	D-3-phosphoglycerate dehydrogenase OS=Mus musculus OX=10090 GN=Phgdh PE=1 SV=3	0.866666667
Mprip	P97434	Myosin phosphatase Rho-interacting protein OS=Mus musculus OX=10090 GN=Mprip PE=1 SV=2	0.846666667

Ubr2	Q6WKZ8	E3 ubiquitin-protein ligase UBR2 OS=Mus musculus OX=10090 GN=Ubr2 PE=1 SV=2	0.896666667
Atp5f1a	Q03265	ATP synthase subunit alpha mitochondrial OS=Mus musculus OX=10090 GN=Atp5f1a PE=1 SV=1	0.91
Ckap4	Q8BMK4	Cytoskeleton-associated protein 4 OS=Mus musculus OX=10090 GN=Ckap4 PE=1 SV=2	0.896666667
Calr	P14211	Calreticulin OS=Mus musculus OX=10090 GN=Calr PE=1 SV=1	0.88
Trim28	Q62318	Transcription intermediary factor 1-beta OS=Mus musculus OX=10090 GN=Trim28 PE=1 SV=3	0.876666667
Mcm7	Q61881	DNA replication licensing factor MCM7 OS=Mus musculus OX=10090 GN=Mcm7 PE=1 SV=1	0.896666667
Mars	Q68FL6	Methionine--tRNA ligase cytoplasmic OS=Mus musculus OX=10090 GN=Mars1 PE=1 SV=1	0.883333333
FAM120A	Q6A0A9	Constitutive coactivator of PPAR-gamma-like protein 1 OS=Mus musculus OX=10090 GN=FAM120A	0.846666667
Gmps	Q3THK7	GMP synthase [glutamine-hydrolyzing] OS=Mus musculus OX=10090 GN=Gmps PE=1 SV=2	0.896666667
Dars	Q922B2	Aspartate--tRNA ligase cytoplasmic OS=Mus musculus OX=10090 GN=Dars1 PE=1 SV=2	0.903333333
Cars	Q9ER72	Cysteine--tRNA ligase cytoplasmic OS=Mus musculus OX=10090 GN=Cars1 PE=1 SV=2	0.966666667
Dis3	Q9CSH3	Exosome complex exonuclease RRP44 OS=Mus musculus OX=10090 GN=Dis3 PE=1 SV=4	0.876666667
Gpd2	Q64521	Glycerol-3-phosphate dehydrogenase mitochondrial OS=Mus musculus OX=10090 GN=Gpd2 PE=1 S	0.923333333
Anapc1	P53995	Anaphase-promoting complex subunit 1 OS=Mus musculus OX=10090 GN=Anapc1 PE=1 SV=2	0.903333333
Cluh	Q5SW19	Clustered mitochondria protein homolog OS=Mus musculus OX=10090 GN=Cluh PE=1 SV=2	0.94
Itgav	P43406	Integrin alpha-V OS=Mus musculus OX=10090 GN=Itgav PE=1 SV=2	0.933333333
Hspa1b	P17879	Heat shock 70 kDa protein 1B OS=Mus musculus OX=10090 GN=Hspa1b PE=1 SV=3	0.853333333
Tnpo1	Q8BFY9	Transportin-1 OS=Mus musculus OX=10090 GN=Tnpo1 PE=1 SV=2	0.886666667
Hnrnpa1	P49312	Heterogeneous nuclear ribonucleoprotein A1 OS=Mus musculus OX=10090 GN=Hnrnpa1 PE=1 SV=2	0.876666667
Hk2	O08528	Hexokinase-2 OS=Mus musculus OX=10090 GN=Hk2 PE=1 SV=1	0.9
Naa15	Q80UM3	N-alpha-acetyltransferase 15 NatA auxiliary subunit OS=Mus musculus OX=10090 GN=Naa15 PE=1 S	0.863333333
Usp15	Q8R5H1	Ubiquitin carboxyl-terminal hydrolase 15 OS=Mus musculus OX=10090 GN=Usp15 PE=1 SV=1	0.896666667
Anxa1	P10107	Annexin A1 OS=Mus musculus OX=10090 GN=Anxa1 PE=1 SV=2	0.856666667
Sfpq	Q8VIJ6	Splicing factor proline- and glutamine-rich OS=Mus musculus OX=10090 GN=Sfpq PE=1 SV=1	0.846666667
Parp1	P11103	Poly [ADP-ribose] polymerase 1 OS=Mus musculus OX=10090 GN=Parp1 PE=1 SV=3	0.923333333
Wdr1	O88342	WD repeat-containing protein 1 OS=Mus musculus OX=10090 GN=Wdr1 PE=1 SV=3	0.903333333
Anxa2	P07356	Annexin A2 OS=Mus musculus OX=10090 GN=Anxa2 PE=1 SV=2	0.883333333
Aldh18a1	Q9Z110	Delta-1-pyrroline-5-carboxylate synthase OS=Mus musculus OX=10090 GN=Aldh18a1 PE=1 SV=2	0.876666667
Atic	Q9CWJ9	Bifunctional purine biosynthesis protein ATIC OS=Mus musculus OX=10090 GN=Atic PE=1 SV=2	0.866666667
Ddx1	Q91VR5	ATP-dependent RNA helicase DDX1 OS=Mus musculus OX=10090 GN=Ddx1 PE=1 SV=1	0.896666667
Uso1	Q9Z1Z0	General vesicular transport factor p115 OS=Mus musculus OX=10090 GN=Uso1 PE=1 SV=2	0.89
Vps35	Q9EQH3	Vacuolar protein sorting-associated protein 35 OS=Mus musculus OX=10090 GN=Vps35 PE=1 SV=1	0.876666667
Immt	Q8CAQ8	MICOS complex subunit Mic60 OS=Mus musculus OX=10090 GN=Immt PE=1 SV=1	0.903333333
Farp1	F8VPU2	FERM ARHGEF and pleckstrin domain-containing protein 1 OS=Mus musculus OX=10090 GN=Farp1	0.873333333

Nomo1	Q6GQT9	Nodal modulator 1 OS=Mus musculus OX=10090 GN=Nomo1 PE=1 SV=1	0.876666667
Cct4	P80315	T-complex protein 1 subunit delta OS=Mus musculus OX=10090 GN=Cct4 PE=1 SV=3	0.896666667
Rai14	Q9EP71	Ankycorbin OS=Mus musculus OX=10090 GN=Rai14 PE=1 SV=1	0.856666667
Prrc2a	Q7TSC1	Protein PRRC2A OS=Mus musculus OX=10090 GN=Prrc2a PE=1 SV=1	0.776666667
Hcfc1	Q61191	Host cell factor 1 OS=Mus musculus OX=10090 GN=Hcfc1 PE=1 SV=2	0.84
Eef1g	Q9D8N0	Elongation factor 1-gamma OS=Mus musculus OX=10090 GN=Eef1g PE=1 SV=3	0.903333333
Rpn1	Q91YQ5	Dolichyl-diphosphooligosaccharide--protein glycosyltransferase subunit 1 OS=Mus musculus OX=1(	0.916666667
Ctnnd1	P30999	Catenin delta-1 OS=Mus musculus OX=10090 GN=Ctnnd1 PE=1 SV=2	0.88
Gars1	Q9CZD3	Glycine--tRNA ligase OS=Mus musculus OX=10090 GN=Gars1 PE=1 SV=1	0.87
Mrc2	Q64449	C-type mannose receptor 2 OS=Mus musculus OX=10090 GN=Mrc2 PE=1 SV=3	0.933333333
G6pdx	Q00612	Glucose-6-phosphate 1-dehydrogenase X OS=Mus musculus OX=10090 GN=G6pdx PE=1 SV=3	0.883333333
Aldh1l2	Q8K009	Mitochondrial 10-formyltetrahydrofolate dehydrogenase OS=Mus musculus OX=10090 GN=Aldh1l2	0.836666667
Nckap1	P28660	Nck-associated protein 1 OS=Mus musculus OX=10090 GN=Nckap1 PE=1 SV=2	0.85
Csde1	Q91W50	Cold shock domain-containing protein E1 OS=Mus musculus OX=10090 GN=Csde1 PE=1 SV=1	0.906666667
Supt16h	Q920B9	FACT complex subunit SPT16 OS=Mus musculus OX=10090 GN=Supt16h PE=1 SV=2	0.91
Anln	Q8K298	Anillin OS=Mus musculus OX=10090 GN=Anln PE=1 SV=2	0.87
Myo5a	Q99104	Unconventional myosin-Va OS=Mus musculus OX=10090 GN=Myo5a PE=1 SV=2	0.88
Cul4a	Q3TCH7	Cullin-4A OS=Mus musculus OX=10090 GN=Cul4a PE=1 SV=1	0.89
Ctnna1	P26231	Catenin alpha-1 OS=Mus musculus OX=10090 GN=Ctnna1 PE=1 SV=1	0.896666667
Ldha	P06151	L-lactate dehydrogenase A chain OS=Mus musculus OX=10090 GN=Ldha PE=1 SV=3	0.876666667
Aco2	Q99KI0	Aconitate hydratase mitochondrial OS=Mus musculus OX=10090 GN=Aco2 PE=1 SV=1	0.893333333
Fkbp4	P30416	Peptidyl-prolyl cis-trans isomerase FKBP4 OS=Mus musculus OX=10090 GN=Fkbp4 PE=1 SV=5	0.893333333
Ncl	P09405	Nucleolin OS=Mus musculus OX=10090 GN=Ncl PE=1 SV=2	0.88
Edc4	Q3UJB9	Enhancer of mRNA-decapping protein 4 OS=Mus musculus OX=10090 GN=Edc4 PE=1 SV=2	0.876666667
Pls3	Q99K51	Plastin-3 OS=Mus musculus OX=10090 GN=Pls3 PE=1 SV=3	0.836666667
Usp5	P56399	Ubiquitin carboxyl-terminal hydrolase 5 OS=Mus musculus OX=10090 GN=Usp5 PE=1 SV=1	0.893333333
Flii	Q9JJ28	Protein flightless-1 homolog OS=Mus musculus OX=10090 GN=Flii PE=1 SV=1	0.916666667
Nup93	Q8BJ71	Nuclear pore complex protein Nup93 OS=Mus musculus OX=10090 GN=Nup93 PE=1 SV=1	0.886666667
Ogt	Q8CGY8	UDP-N-acetylglucosamine--peptide N-acetylglucosaminyltransferase 110 kDa subunit OS=Mus musc	0.92
Xrn2	Q9DBR1	5'-3' exoribonuclease 2 OS=Mus musculus OX=10090 GN=Xrn2 PE=1 SV=1	0.886666667
Ap2b1	Q9DBG3	AP-2 complex subunit beta OS=Mus musculus OX=10090 GN=Ap2b1 PE=1 SV=1	0.876666667
Ap2a2	P17427	AP-2 complex subunit alpha-2 OS=Mus musculus OX=10090 GN=Ap2a2 PE=1 SV=2	0.923333333
Ddx5	Q61656	Probable ATP-dependent RNA helicase DDX5 OS=Mus musculus OX=10090 GN=Ddx5 PE=1 SV=2	0.903333333
Hnrnpa2b1	O88569	Heterogeneous nuclear ribonucleoproteins A2/B1 OS=Mus musculus OX=10090 GN=Hnrnpa2b1 PE	0.846666667

Copg1	Q9QZE5	Coatomer subunit gamma-1 OS=Mus musculus OX=10090 GN=Copg1 PE=1 SV=1	0.903333333
Pdia4	P08003	Protein disulfide-isomerase A4 OS=Mus musculus OX=10090 GN=Pdia4 PE=1 SV=3	0.903333333
Clasp1	Q80TV8	CLIP-associating protein 1 OS=Mus musculus OX=10090 GN=Clasp1 PE=1 SV=2	0.903333333
Anxa6	P14824	Annexin A6 OS=Mus musculus OX=10090 GN=Anxa6 PE=1 SV=3	0.88
Tkt	P40142	Transketolase OS=Mus musculus OX=10090 GN=Tkt PE=1 SV=1	0.88
Eif5b	Q05D44	Eukaryotic translation initiation factor 5B OS=Mus musculus OX=10090 GN=Eif5b PE=1 SV=2	0.86
Rtn4	Q99P72	Reticulon-4 OS=Mus musculus OX=10090 GN=Rtn4 PE=1 SV=2	0.846666667
Gart	Q64737	Trifunctional purine biosynthetic protein adenosine-3 OS=Mus musculus OX=10090 GN=Gart PE=1 S	0.866666667
Atp2a2	O55143	Sarcoplasmic/endoplasmic reticulum calcium ATPase 2 OS=Mus musculus OX=10090 GN=Atp2a2 PE	0.903333333
Ap3d1	O54774	AP-3 complex subunit delta-1 OS=Mus musculus OX=10090 GN=Ap3d1 PE=1 SV=1	0.833333333
Xpo5	Q924C1	Exportin-5 OS=Mus musculus OX=10090 GN=Xpo5 PE=1 SV=1	0.893333333
Dnm1l	Q8K1M6	Dynamin-1-like protein OS=Mus musculus OX=10090 GN=Dnm1l PE=1 SV=2	0.88
Rrp12	Q6P5B0	RRP12-like protein OS=Mus musculus OX=10090 GN=Rrp12 PE=1 SV=1	0.92
Rrm1	P07742	Ribonucleoside-diphosphate reductase large subunit OS=Mus musculus OX=10090 GN=Rrm1 PE=1 S	0.863333333
Plxnb2	B2RXS4	Plexin-B2 OS=Mus musculus OX=10090 GN=Plxnb2 PE=1 SV=1	0.893333333
Nrdc	Q8BHG1	Nardilysin OS=Mus musculus OX=10090 GN=Nrdc PE=1 SV=1	0.873333333
Polr2a	P08775	DNA-directed RNA polymerase II subunit RPB1 OS=Mus musculus OX=10090 GN=Polr2a PE=1 SV=3	0.876666667
Ipo4	Q8VI75	Importin-4 OS=Mus musculus OX=10090 GN=Ipo4 PE=1 SV=1	0.91
Tuba8	Q9JJZ2	Tubulin alpha-8 chain OS=Mus musculus OX=10090 GN=Tuba8 PE=1 SV=1	
Tubb3	Q9ERD7	Tubulin beta-3 chain OS=Mus musculus OX=10090 GN=Tubb3 PE=1 SV=1	0.946666667
Lmnb1	P14733	Lamin-B1 OS=Mus musculus OX=10090 GN=Lmnb1 PE=1 SV=3	0.883333333
Ddx3x	Q62167	ATP-dependent RNA helicase DDX3X OS=Mus musculus OX=10090 GN=Ddx3x PE=1 SV=3	0.91
Tcp1	P11983	T-complex protein 1 subunit alpha OS=Mus musculus OX=10090 GN=Tcp1 PE=1 SV=3	0.89
Canx	P35564	Calnexin OS=Mus musculus OX=10090 GN=Canx PE=1 SV=1	0.876666667
Gsn	P13020	Gelsolin OS=Mus musculus OX=10090 GN=Gsn PE=1 SV=3	0.853333333
Cct7	P80313	T-complex protein 1 subunit eta OS=Mus musculus OX=10090 GN=Cct7 PE=1 SV=1	0.88
Matr3	Q8K310	Matrin-3 OS=Mus musculus OX=10090 GN=Matr3 PE=1 SV=1	0.87
Hyou1	Q9JKR6	Hypoxia up-regulated protein 1 OS=Mus musculus OX=10090 GN=Hyou1 PE=1 SV=1	0.866666667
Pfas	Q5SUR0	Phosphoribosylformylglycinamidine synthase OS=Mus musculus OX=10090 GN=Pfas PE=1 SV=1	0.846666667
Gemin5	Q8BX17	Gem-associated protein 5 OS=Mus musculus OX=10090 GN=Gemin5 PE=1 SV=2	0.886666667
Pola1	P33609	DNA polymerase alpha catalytic subunit OS=Mus musculus OX=10090 GN=Pola1 PE=1 SV=2	0.833333333
Ascc3	E9PZJ8	Activating signal cointegrator 1 complex subunit 3 OS=Mus musculus OX=10090 GN=Ascc3 PE=1 SV	0.91
Eno1	P17182	Alpha-enolase OS=Mus musculus OX=10090 GN=Eno1 PE=1 SV=3	0.893333333
Cul4b	A2A432	Cullin-4B OS=Mus musculus OX=10090 GN=Cul4b PE=1 SV=1	0.86

Ap2a1	P17426	AP-2 complex subunit alpha-1 OS=Mus musculus OX=10090 GN=Ap2a1 PE=1 SV=1	0.91
Kif5b	Q61768	Kinesin-1 heavy chain OS=Mus musculus OX=10090 GN=Kif5b PE=1 SV=3	0.906666667
Pabpc1	P29341	Polyadenylate-binding protein 1 OS=Mus musculus OX=10090 GN=Pabpc1 PE=1 SV=2	0.896666667
Cct5	P80316	T-complex protein 1 subunit epsilon OS=Mus musculus OX=10090 GN=Cct5 PE=1 SV=1	0.903333333
Npepps	Q11011	Puromycin-sensitive aminopeptidase OS=Mus musculus OX=10090 GN=Npepps PE=1 SV=2	0.85
Eif3b	Q8JZQ9	Eukaryotic translation initiation factor 3 subunit B OS=Mus musculus OX=10090 GN=Eif3b PE=1 SV=	0.89
Pdcd6ip	Q9WU78	Programmed cell death 6-interacting protein OS=Mus musculus OX=10090 GN=Pdcd6ip PE=1 SV=3	0.853333333
Srrm2	Q8BTI8	Serine/arginine repetitive matrix protein 2 OS=Mus musculus OX=10090 GN=Srrm2 PE=1 SV=3	0.826666667
Eif4g2	Q62448	Eukaryotic translation initiation factor 4 gamma 2 OS=Mus musculus OX=10090 GN=Eif4g2 PE=1 SV	0.853333333
Pitrm1	Q8K411	Presequence protease mitochondrial OS=Mus musculus OX=10090 GN=Pitrm1 PE=1 SV=1	0.876666667
Smc2	Q8CG48	Structural maintenance of chromosomes protein 2 OS=Mus musculus OX=10090 GN=Smc2 PE=1 SV	0.896666667
Msh6	P54276	DNA mismatch repair protein Msh6 OS=Mus musculus OX=10090 GN=Msh6 PE=1 SV=3	0.883333333
Tubb6	Q922F4	Tubulin beta-6 chain OS=Mus musculus OX=10090 GN=Tubb6 PE=1 SV=1	0.886666667
Eif4a1	P60843	Eukaryotic initiation factor 4A-I OS=Mus musculus OX=10090 GN=Eif4a1 PE=1 SV=1	0.89
Gdi2	Q61598	Rab GDP dissociation inhibitor beta OS=Mus musculus OX=10090 GN=Gdi2 PE=1 SV=1	0.893333333
Hspa9	P38647	Stress-70 protein mitochondrial OS=Mus musculus OX=10090 GN=Hspa9 PE=1 SV=3	0.876666667
Cct3	P80318	T-complex protein 1 subunit gamma OS=Mus musculus OX=10090 GN=Cct3 PE=1 SV=1	0.896666667
Hnrnpu	Q8VEK3	Heterogeneous nuclear ribonucleoprotein U OS=Mus musculus OX=10090 GN=Hnrnpu PE=1 SV=1	0.86
Mcm4	P49717	DNA replication licensing factor MCM4 OS=Mus musculus OX=10090 GN=Mcm4 PE=1 SV=1	0.91
Mcm6	P97311	DNA replication licensing factor MCM6 OS=Mus musculus OX=10090 GN=Mcm6 PE=1 SV=1	0.9
Rars	Q9D0I9	Arginine--tRNA ligase cytoplasmic OS=Mus musculus OX=10090 GN=Rars1 PE=1 SV=2	0.896666667
Mcm2	P97310	DNA replication licensing factor MCM2 OS=Mus musculus OX=10090 GN=Mcm2 PE=1 SV=3	0.866666667
Smc4	Q8CG47	Structural maintenance of chromosomes protein 4 OS=Mus musculus OX=10090 GN=Smc4 PE=1 SV	0.9
Atp1a1	Q8VDN2	Sodium/potassium-transporting ATPase subunit alpha-1 OS=Mus musculus OX=10090 GN=Atp1a1 F	0.893333333
P4hb	P09103	Protein disulfide-isomerase OS=Mus musculus OX=10090 GN=P4hb PE=1 SV=2	0.9
Uba6	Q8C7R4	Ubiquitin-like modifier-activating enzyme 6 OS=Mus musculus OX=10090 GN=Uba6 PE=1 SV=1	0.913333333
Mcm5	P49718	DNA replication licensing factor MCM5 OS=Mus musculus OX=10090 GN=Mcm5 PE=1 SV=2	0.883333333
Prrc2c	Q3TLH4	Protein PRRC2C OS=Mus musculus OX=10090 GN=Prrc2c PE=1 SV=3	0.82
Xpo1	Q6P5F9	Exportin-1 OS=Mus musculus OX=10090 GN=Xpo1 PE=1 SV=1	0.886666667
Ppfibp1	Q8C8U0	Liprin-beta-1 OS=Mus musculus OX=10090 GN=Ppfibp1 PE=1 SV=3	0.85
Map4	P27546	Microtubule-associated protein 4 OS=Mus musculus OX=10090 GN=Map4 PE=1 SV=3	0.833333333
Nsun2	Q1HFZ0	RNA cytosine C(5)-methyltransferase NSUN2 OS=Mus musculus OX=10090 GN=Nsun2 PE=1 SV=2	0.88
Nup98	Q6PFD9	Nuclear pore complex protein Nup98-Nup96 OS=Mus musculus OX=10090 GN=Nup98 PE=1 SV=2	0.92
Smarca5	Q91ZW3	SWI/SNF-related matrix-associated actin-dependent regulator of chromatin subfamily A member 5 (	0.866666667

Pgk1	P09411	Phosphoglycerate kinase 1 OS=Mus musculus OX=10090 GN=Pgk1 PE=1 SV=4	0.86
Pds5a	Q6A026	Sister chromatid cohesion protein PDS5 homolog A OS=Mus musculus OX=10090 GN=Pds5a PE=1 S'	0.903333333
Mcm3	P25206	DNA replication licensing factor MCM3 OS=Mus musculus OX=10090 GN=Mcm3 PE=1 SV=2	0.906666667
Atrx	Q61687	Transcriptional regulator ATRX OS=Mus musculus OX=10090 GN=Atrx PE=1 SV=3	0.853333333
Cse1l	Q9ERK4	Exportin-2 OS=Mus musculus OX=10090 GN=Cse1l PE=1 SV=1	0.886666667
Cct8	P42932	T-complex protein 1 subunit theta OS=Mus musculus OX=10090 GN=Cct8 PE=1 SV=3	0.906666667
Ogdh	Q60597	2-oxoglutarate dehydrogenase mitochondrial OS=Mus musculus OX=10090 GN=Ogdh PE=1 SV=3	0.916666667
Igf2r	Q07113	Cation-independent mannose-6-phosphate receptor OS=Mus musculus OX=10090 GN=Igf2r PE=1 S\	0.88
Msn	P26041	Moesin OS=Mus musculus OX=10090 GN=Msn PE=1 SV=3	0.886666667
Aldoa	P05064	Fructose-bisphosphate aldolase A OS=Mus musculus OX=10090 GN=Aldoa PE=1 SV=2	0.856666667
Nedd4	P46935	E3 ubiquitin-protein ligase NEDD4 OS=Mus musculus OX=10090 GN=Nedd4 PE=1 SV=3	0.86
Tars1	Q9D0R2	Threonine--tRNA ligase 1 cytoplasmic OS=Mus musculus OX=10090 GN=Tars1 PE=1 SV=2	0.87
Mthfd1	Q922D8	C-1-tetrahydrofolate synthase cytoplasmic OS=Mus musculus OX=10090 GN=Mthfd1 PE=1 SV=4	0.86
Myo1c	Q9WTI7	Unconventional myosin-Ic OS=Mus musculus OX=10090 GN=Myo1c PE=1 SV=2	0.883333333
Hspd1	P63038	60 kDa heat shock protein mitochondrial OS=Mus musculus OX=10090 GN=Hspd1 PE=1 SV=1	0.896666667
Ipo7	Q9EPL8	Importin-7 OS=Mus musculus OX=10090 GN=Ipo7 PE=1 SV=2	0.896666667
Prep	Q9QUR6	Prolyl endopeptidase OS=Mus musculus OX=10090 GN=Prep PE=1 SV=1	0.886666667
Cyfip1	Q7TMB8	Cytoplasmic FMR1-interacting protein 1 OS=Mus musculus OX=10090 GN=Cyfip1 PE=1 SV=1	0.87
Lamb1	P02469	Laminin subunit beta-1 OS=Mus musculus OX=10090 GN=Lamb1 PE=1 SV=3	0.89
Trip12	G5E870	E3 ubiquitin-protein ligase TRIP12 OS=Mus musculus OX=10090 GN=Trip12 PE=1 SV=1	0.843333333
Mthfd1l	Q3V3R1	Monofunctional C1-tetrahydrofolate synthase mitochondrial OS=Mus musculus OX=10090 GN=Mt	0.86
Rrbp1	Q99PL5	Ribosome-binding protein 1 OS=Mus musculus OX=10090 GN=Rrbp1 PE=1 SV=2	0.896666667
Psmd2	Q8VDM4	26S proteasome non-ATPase regulatory subunit 2 OS=Mus musculus OX=10090 GN=Psmd2 PE=1 SV:	0.883333333
Psmd1	Q3TXS7	26S proteasome non-ATPase regulatory subunit 1 OS=Mus musculus OX=10090 GN=Psmd1 PE=1 SV:	0.873333333
Hnrnpm	Q9D0E1	Heterogeneous nuclear ribonucleoprotein M OS=Mus musculus OX=10090 GN=Hnrnpm PE=1 SV=3	0.886666667
Sf3b3	Q921M3	Splicing factor 3B subunit 3 OS=Mus musculus OX=10090 GN=Sf3b3 PE=1 SV=1	0.896666667
Usp47	Q8BY87	Ubiquitin carboxyl-terminal hydrolase 47 OS=Mus musculus OX=10090 GN=Usp47 PE=1 SV=2	0.84
Hectd1	Q69ZR2	E3 ubiquitin-protein ligase HECTD1 OS=Mus musculus OX=10090 GN=Hectd1 PE=1 SV=2	0.936666667
Pds5b	Q4VA53	Sister chromatid cohesion protein PDS5 homolog B OS=Mus musculus OX=10090 GN=Pds5b PE=1 S	0.946666667
Hsph1	Q61699	Heat shock protein 105 kDa OS=Mus musculus OX=10090 GN=Hsph1 PE=1 SV=2	0.863333333
Eftud2	O08810	116 kDa U5 small nuclear ribonucleoprotein component OS=Mus musculus OX=10090 GN=Eftud2 F	0.863333333
Stip1	Q60864	Stress-induced-phosphoprotein 1 OS=Mus musculus OX=10090 GN=Stip1 PE=1 SV=1	0.9
Glg1	Q61543	Golgi apparatus protein 1 OS=Mus musculus OX=10090 GN=Glg1 PE=1 SV=1	0.87
Copb2	O55029	Coatomer subunit beta' OS=Mus musculus OX=10090 GN=Copb2 PE=1 SV=2	0.86

Ncapd2	Q8K2Z4	Condensin complex subunit 1 OS=Mus musculus OX=10090 GN=Ncapd2 PE=1 SV=2	0.823333333
Smc1a	Q9CU62	Structural maintenance of chromosomes protein 1A OS=Mus musculus OX=10090 GN=Smc1a PE=1	0.9
Tjp1	P39447	Tight junction protein ZO-1 OS=Mus musculus OX=10090 GN=Tjp1 PE=1 SV=2	0.86
Baz1b	Q9Z277	Tyrosine-protein kinase BAZ1B OS=Mus musculus OX=10090 GN=Baz1b PE=1 SV=2	0.933333333
Rnf213	E9Q555	E3 ubiquitin-protein ligase RNF213 OS=Mus musculus OX=10090 GN=Rnf213 PE=1 SV=2	0.886666667
Pdia3	P27773	Protein disulfide-isomerase A3 OS=Mus musculus OX=10090 GN=Pdia3 PE=1 SV=2	0.926666667
Epb41l2	O70318	Band 4.1-like protein 2 OS=Mus musculus OX=10090 GN=Epb41l2 PE=1 SV=2	0.823333333
Sec31a	Q3UPL0	Protein transport protein Sec31A OS=Mus musculus OX=10090 GN=Sec31a PE=1 SV=2	0.89
Copb1	Q9JIF7	Coatomer subunit beta OS=Mus musculus OX=10090 GN=Copb1 PE=1 SV=1	0.863333333
Lars	Q8BMJ2	Leucine--tRNA ligase cytoplasmic OS=Mus musculus OX=10090 GN=Lars1 PE=1 SV=2	0.863333333
Smc3	Q9CW03	Structural maintenance of chromosomes protein 3 OS=Mus musculus OX=10090 GN=Smc3 PE=1 SV	0.893333333
Itpr3	P70227	Inositol 1 4 5-trisphosphate receptor type 3 OS=Mus musculus OX=10090 GN=Itpr3 PE=1 SV=3	0.83
Hspa4	Q61316	Heat shock 70 kDa protein 4 OS=Mus musculus OX=10090 GN=Hspa4 PE=1 SV=1	0.883333333
Hdlbp	Q8VDJ3	Vigilin OS=Mus musculus OX=10090 GN=Hdlbp PE=1 SV=1	0.9
Ganab	Q8BHN3	Neutral alpha-glucosidase AB OS=Mus musculus OX=10090 GN=Ganab PE=1 SV=1	0.866666667
Dhx9	O70133	ATP-dependent RNA helicase A OS=Mus musculus OX=10090 GN=Dhx9 PE=1 SV=2	0.936666667
Tnks1bp1	P58871	182 kDa tankyrase-1-binding protein OS=Mus musculus OX=10090 GN=Tnks1bp1 PE=1 SV=2	0.81
Aars	Q8BGQ7	Alanine--tRNA ligase cytoplasmic OS=Mus musculus OX=10090 GN=Aars1 PE=1 SV=1	0.883333333
Kpnb1	P70168	Importin subunit beta-1 OS=Mus musculus OX=10090 GN=Kpnb1 PE=1 SV=2	0.873333333
Ecpas	Q6PDI5	Proteasome adapter and scaffold protein ECM29 OS=Mus musculus OX=10090 GN=Ecpas PE=1 SV=3	0.87
Ktn1	Q61595	Kinectin OS=Mus musculus OX=10090 GN=Ktn1 PE=1 SV=1	0.896666667
Dctn1	O08788	Dynactin subunit 1 OS=Mus musculus OX=10090 GN=Dctn1 PE=1 SV=3	0.896666667
Top2b	Q64511	DNA topoisomerase 2-beta OS=Mus musculus OX=10090 GN=Top2b PE=1 SV=2	0.916666667
Upf1	Q9EPU0	Regulator of nonsense transcripts 1 OS=Mus musculus OX=10090 GN=Upf1 PE=1 SV=2	0.906666667
Gapdh	P16858	Glyceraldehyde-3-phosphate dehydrogenase OS=Mus musculus OX=10090 GN=Gapdh PE=1 SV=2	0.893333333
Eea1	Q8BL66	Early endosome antigen 1 OS=Mus musculus OX=10090 GN=Eea1 PE=1 SV=2	0.866666667
Hspa5	P20029	Endoplasmic reticulum chaperone BiP OS=Mus musculus OX=10090 GN=Hspa5 PE=1 SV=3	0.89
Lmna	P48678	Prelamin-A/C OS=Mus musculus OX=10090 GN=Lmna PE=1 SV=2	0.876666667
Dock7	Q8R1A4	Dedicator of cytokinesis protein 7 OS=Mus musculus OX=10090 GN=Dock7 PE=1 SV=3	0.866666667
Col1a2	Q01149	Collagen alpha-2(I) chain OS=Mus musculus OX=10090 GN=Col1a2 PE=1 SV=2	0.76
Sf3b1	Q99NB9	Splicing factor 3B subunit 1 OS=Mus musculus OX=10090 GN=Sf3b1 PE=1 SV=1	0.853333333
Iars	Q8BU30	Isoleucine--tRNA ligase cytoplasmic OS=Mus musculus OX=10090 GN=Iars1 PE=1 SV=2	0.893333333
Tubb4a	Q9D6F9	Tubulin beta-4A chain OS=Mus musculus OX=10090 GN=Tubb4a PE=1 SV=3	0.98
Uba1	Q02053	Ubiquitin-like modifier-activating enzyme 1 OS=Mus musculus OX=10090 GN=Uba1 PE=1 SV=1	0.873333333

Fbln2	P37889	Fibulin-2 OS=Mus musculus OX=10090 GN=Fbln2 PE=1 SV=2	0.853333333
Tuba4a	P68368	Tubulin alpha-4A chain OS=Mus musculus OX=10090 GN=Tuba4a PE=1 SV=1	0.91
Ddx21	Q9JIK5	Nucleolar RNA helicase 2 OS=Mus musculus OX=10090 GN=Ddx21 PE=1 SV=3	0.923333333
Cnot1	Q6ZQ08	CCR4-NOT transcription complex subunit 1 OS=Mus musculus OX=10090 GN=Cnot1 PE=1 SV=2	0.893333333
Tubb2b	Q9CWF2	Tubulin beta-2B chain OS=Mus musculus OX=10090 GN=Tubb2b PE=1 SV=1	0.933333333
Tubb2a	Q7TMM9	Tubulin beta-2A chain OS=Mus musculus OX=10090 GN=Tubb2a PE=1 SV=1	1.953333333
Uggt1	Q6P5E4	UDP-glucose:glycoprotein glucosyltransferase 1 OS=Mus musculus OX=10090 GN=Uggt1 PE=1 SV=4	0.893333333
Acly	Q91V92	ATP-citrate synthase OS=Mus musculus OX=10090 GN=Acly PE=1 SV=1	0.87
Tpp2	Q64514	Tripeptidyl-peptidase 2 OS=Mus musculus OX=10090 GN=Tpp2 PE=1 SV=3	0.866666667
Nes	Q6P5H2	Nestin OS=Mus musculus OX=10090 GN=Nes PE=1 SV=1	0.873333333
Cand1	Q6ZQ38	Cullin-associated NEDD8-dissociated protein 1 OS=Mus musculus OX=10090 GN=Cand1 PE=1 SV=2	0.896666667
Snd1	Q78PY7	Staphylococcal nuclease domain-containing protein 1 OS=Mus musculus OX=10090 GN=Snd1 PE=1	0.886666667
Tubb4b	P68372	Tubulin beta-4B chain OS=Mus musculus OX=10090 GN=Tubb4b PE=1 SV=1	0.896666667
Vcp	Q01853	Transitional endoplasmic reticulum ATPase OS=Mus musculus OX=10090 GN=Vcp PE=1 SV=4	0.893333333
Dnmt1	P13864	DNA (cytosine-5)-methyltransferase 1 OS=Mus musculus OX=10090 GN=Dnmt1 PE=1 SV=5	0.89
Akap12	Q9WTQ5	A-kinase anchor protein 12 OS=Mus musculus OX=10090 GN=Akap12 PE=1 SV=1	0.83
Ddb1	Q3U1J4	DNA damage-binding protein 1 OS=Mus musculus OX=10090 GN=Ddb1 PE=1 SV=2	0.91
Utp20	Q5XG71	Small subunit processome component 20 homolog OS=Mus musculus OX=10090 GN=Utp20 PE=1 S	0.92
Tuba1c	P68373	Tubulin alpha-1C chain OS=Mus musculus OX=10090 GN=Tuba1c PE=1 SV=1	0.95
Tuba1a	P68369	Tubulin alpha-1A chain OS=Mus musculus OX=10090 GN=Tuba1a PE=1 SV=1	0.933333333
Acta1	P68134	Actin alpha skeletal muscle OS=Mus musculus OX=10090 GN=Acta1 PE=1 SV=1	23.79666667
Tubb5	P99024	Tubulin beta-5 chain OS=Mus musculus OX=10090 GN=Tubb5 PE=1 SV=1	0.9
Chd4	Q6PDQ2	Chromodomain-helicase-DNA-binding protein 4 OS=Mus musculus OX=10090 GN=Chd4 PE=1 SV=1	0.876666667
Ipo5	Q8BKC5	Importin-5 OS=Mus musculus OX=10090 GN=Ipo5 PE=1 SV=3	0.893333333
Man2a1	P27046	Alpha-mannosidase 2 OS=Mus musculus OX=10090 GN=Man2a1 PE=1 SV=2	0.886666667
Copa	Q8CIE6	Coatomer subunit alpha OS=Mus musculus OX=10090 GN=Copa PE=1 SV=2	0.876666667
Pdcd11	Q6NS46	Protein RRP5 homolog OS=Mus musculus OX=10090 GN=Pdcd11 PE=1 SV=2	0.9
Hsp90b1	P08113	Endoplasmin OS=Mus musculus OX=10090 GN=Hsp90b1 PE=1 SV=2	0.906666667
Lrpprc	Q6PB66	Leucine-rich PPR motif-containing protein mitochondrial OS=Mus musculus OX=10090 GN=Lrpprc	0.89
Numa1	E9Q7G0	Nuclear mitotic apparatus protein 1 OS=Mus musculus OX=10090 GN=Numa1 PE=1 SV=1	0.866666667
Smchd1	Q6P5D8	Structural maintenance of chromosomes flexible hinge domain-containing protein 1 OS=Mus musc	0.873333333
Eif4g1	Q6NZJ6	Eukaryotic translation initiation factor 4 gamma 1 OS=Mus musculus OX=10090 GN=Eif4g1 PE=1 SV	0.9
Ckap5	A2AGT5	Cytoskeleton-associated protein 5 OS=Mus musculus OX=10090 GN=Ckap5 PE=1 SV=1	0.896666667
Col1a1	P11087	Collagen alpha-1(I) chain OS=Mus musculus OX=10090 GN=Col1a1 PE=1 SV=4	0.683333333

Acta2	P62737	Actin aortic smooth muscle OS=Mus musculus OX=10090 GN=Acta2 PE=1 SV=1	0.953333333
Vars	Q9Z1Q9	Valine--tRNA ligase OS=Mus musculus OX=10090 GN=Vars1 PE=1 SV=1	0.866666667
Hspa8	P63017	Heat shock cognate 71 kDa protein OS=Mus musculus OX=10090 GN=Hspa8 PE=1 SV=1	0.883333333
Top2a	Q01320	DNA topoisomerase 2-alpha OS=Mus musculus OX=10090 GN=Top2a PE=1 SV=2	0.873333333
Vcl	Q64727	Vinculin OS=Mus musculus OX=10090 GN=Vcl PE=1 SV=4	0.89
Actn4	P57780	Alpha-actinin-4 OS=Mus musculus OX=10090 GN=Actn4 PE=1 SV=1	0.883333333
Actn1	Q7TPR4	Alpha-actinin-1 OS=Mus musculus OX=10090 GN=Actn1 PE=1 SV=1	0.883333333
Tpr	F6ZDS4	Nucleoprotein TPR OS=Mus musculus OX=10090 GN=Tpr PE=1 SV=1	0.856666667
Eif3a	P23116	Eukaryotic translation initiation factor 3 subunit A OS=Mus musculus OX=10090 GN=Eif3a PE=1 SV=	0.87
Hsp90aa1	P07901	Heat shock protein HSP 90-alpha OS=Mus musculus OX=10090 GN=Hsp90aa1 PE=1 SV=4	0.873333333
Eef1a1	P10126	Elongation factor 1-alpha 1 OS=Mus musculus OX=10090 GN=Eef1a1 PE=1 SV=3	0.853333333
Mybbp1a	Q7TPV4	Myb-binding protein 1A OS=Mus musculus OX=10090 GN=Mybbp1a PE=1 SV=2	0.883333333
Vim	P20152	Vimentin OS=Mus musculus OX=10090 GN=Vim PE=1 SV=3	0.883333333
Eprs	Q8CGC7	Bifunctional glutamate/proline--tRNA ligase OS=Mus musculus OX=10090 GN=Eprs1 PE=1 SV=4	0.896666667
Usp9x	P70398	Probable ubiquitin carboxyl-terminal hydrolase FAF-X OS=Mus musculus OX=10090 GN=Usp9x PE=1	0.86
Map1b	P14873	Microtubule-associated protein 1B OS=Mus musculus OX=10090 GN=Map1b PE=1 SV=2	0.873333333
Birc6	O88738	Baculoviral IAP repeat-containing protein 6 OS=Mus musculus OX=10090 GN=Birc6 PE=1 SV=2	0.906666667
Dst	Q91ZU6	Dystonin OS=Mus musculus OX=10090 GN=Dst PE=1 SV=2	0.913333333
Pkm	P52480	Pyruvate kinase PKM OS=Mus musculus OX=10090 GN=Pkm PE=1 SV=4	0.883333333
Hspg2	Q05793	Basement membrane-specific heparan sulfate proteoglycan core protein OS=Mus musculus OX=100	0.78
Mki67	E9PVX6	Proliferation marker protein Ki-67 OS=Mus musculus OX=10090 GN=Mki67 PE=1 SV=1	0.856666667
Iqgap1	Q9JKF1	Ras GTPase-activating-like protein IQGAP1 OS=Mus musculus OX=10090 GN=Iqgap1 PE=1 SV=2	0.883333333
Ranbp2	Q9ERU9	E3 SUMO-protein ligase RanBP2 OS=Mus musculus OX=10090 GN=Ranbp2 PE=1 SV=2	0.863333333
Ubr4	A2AN08	E3 ubiquitin-protein ligase UBR4 OS=Mus musculus OX=10090 GN=Ubr4 PE=1 SV=1	0.863333333
Huwe1	Q7TMY8	E3 ubiquitin-protein ligase HUWE1 OS=Mus musculus OX=10090 GN=Huwe1 PE=1 SV=5	0.893333333
Gcn1	E9PVA8	eIF-2-alpha kinase activator GCN1 OS=Mus musculus OX=10090 GN=Gcn1 PE=1 SV=1	0.89
Acaca	Q5SWU9	Acetyl-CoA carboxylase 1 OS=Mus musculus OX=10090 GN=Acaca PE=1 SV=1	0.86
Eef2	P58252	Elongation factor 2 OS=Mus musculus OX=10090 GN=Eef2 PE=1 SV=2	0.896666667
Snrnp200	Q6P4T2	U5 small nuclear ribonucleoprotein 200 kDa helicase OS=Mus musculus OX=10090 GN=Snrnp200 P	0.903333333
Prpf8	Q99PV0	Pre-mRNA-processing-splicing factor 8 OS=Mus musculus OX=10090 GN=Prpf8 PE=1 SV=2	0.883333333
Cad	B2RQC6	CAD protein OS=Mus musculus OX=10090 GN=Cad PE=1 SV=1	0.893333333
Macf1	Q9QXZ0	Microtubule-actin cross-linking factor 1 OS=Mus musculus OX=10090 GN=Macf1 PE=1 SV=2	0.86
Actb	P60710	Actin cytoplasmic 1 OS=Mus musculus OX=10090 GN=Actb PE=1 SV=1	0.9
Actg1	P63260	Actin cytoplasmic 2 OS=Mus musculus OX=10090 GN=Actg1 PE=1 SV=1	0.903333333

Hsp90ab1	P11499	Heat shock protein HSP 90-beta OS=Mus musculus OX=10090 GN=Hsp90ab1 PE=1 SV=3	0.883333333
Myof	Q69ZN7	Myoferlin OS=Mus musculus OX=10090 GN=Myof PE=1 SV=2	0.873333333
Tln1	P26039	Talin-1 OS=Mus musculus OX=10090 GN=Tln1 PE=1 SV=2	0.88
Cltc	Q68FD5	Clathrin heavy chain 1 OS=Mus musculus OX=10090 GN=Cltc PE=1 SV=3	0.896666667
Sptbn1	Q62261	Spectrin beta chain non-erythrocytic 1 OS=Mus musculus OX=10090 GN=Sptbn1 PE=1 SV=2	0.88
Fasn	P19096	Fatty acid synthase OS=Mus musculus OX=10090 GN=Fasn PE=1 SV=2	0.883333333
Sptan1	P16546	Spectrin alpha chain non-erythrocytic 1 OS=Mus musculus OX=10090 GN=Sptan1 PE=1 SV=4	0.87
Myh10	Q61879	Myosin-10 OS=Mus musculus OX=10090 GN=Myh10 PE=1 SV=2	0.913333333
Lrp1	Q91ZX7	Prolow-density lipoprotein receptor-related protein 1 OS=Mus musculus OX=10090 GN=Lrp1 PE=1	0.916666667
Flnb	Q80X90	Filamin-B OS=Mus musculus OX=10090 GN=Flnb PE=1 SV=3	0.873333333
Flnc	Q8VHX6	Filamin-C OS=Mus musculus OX=10090 GN=Flnc PE=1 SV=3	0.833333333
Myh9	Q8VDD5	Myosin-9 OS=Mus musculus OX=10090 GN=Myh9 PE=1 SV=4	0.893333333
Flna	Q8BTM8	Filamin-A OS=Mus musculus OX=10090 GN=Flna PE=1 SV=5	0.863333333
Fn1	P11276	Fibronectin OS=Mus musculus OX=10090 GN=Fn1 PE=1 SV=4	0.833333333
Dync1h1	Q9JHU4	Cytoplasmic dynein 1 heavy chain 1 OS=Mus musculus OX=10090 GN=Dync1h1 PE=1 SV=2	0.886666667
Plec	Q9QXS1	Plectin OS=Mus musculus OX=10090 GN=Plec PE=1 SV=3	0.873333333
