# Supplementary material for: Quantitative Proteomics Analysis Expands the Roles of Lysine β-Hydroxybutyrylation Pathway in Response to Environmental β-Hydroxybutyrate
Source: Oxid Med Cell Longev. 2022 Feb 24;2022:4592170. doi: 10.1155/2022/4592170 (PMC8894020; doi:10.1155/2022/4592170)
Supplement: Supplementary Materials — Table S1/S2/S3 are the complete lists of identified proteins/Kac sites/Kbhb sites in MEF cells with or without BHB treated. [file 4592170.f1.zip › Table S2. The identification of Kac sites and substrates.pdf]

Table S2:The identification of Kac sites and substrates.

Gene name	UniprotID	Site in pı	Peptide	Positio	Quality Sc	Ascore	Ratio:Samp	Ratio:Sam	Ratio:Sam	Ratio_averag
Cul4b	A2A432	872	FIC(+57.02)NDDFK(*)HK(*)	K8	57.45	33.18	0.7872340	1.162162	1.166666	1.0386876
Cul4b	A2A432	582	DK(*)VDHIIDTC(+57.02)FLK(*)	K2	52.98	175.94	-	-	1.022222222	
Cul4b	A2A432	766	LQEIFK(*)TFYLGK(*)	K6	49.32	113.99	0.7021276	1.108108	0.9	0.9034119
Tanc2	A2A690	14	MLLTGGK(*)SSR(*)	K7	58.1	1000	-	-	-	
Gpatch8	A2A6A1	61	LGQGLGK(*)SLQGR(*)	K7	38.61	1000	1.2234042	0.460526	0.909090	0.8643405
Chd5	A2A8L1	945	NMPAK(*)TELIVR(*)	K5	23.49	1000	-	-	0.476190476	
Osbpl9	A2A8Z1	688	DIDAATEAK(*)HR(*)	K9	45.9	1000	1.5384615	1.253333	1.525641	1.4391453
Sh3pxd2b	A2AAY5	565	GSSPK(*)PPGMILPMIPAK(*)HAPLAI	K17	7.42	143.73	1.1022727	-	-	
Dock11	A2AF47	1342	VHDAWLSK(*)HFGIDR(*)	K8	36.58	1000	0.7052631	-	0.829545	0.7674043
Dock11	A2AF47	1147	NLLIK(*)HAFDTR(*)	K5	35.89	1000	1.0210526	0.715596	1.181818	0.9728224
Med12	A2AGH6	1800	SQPATK(*)NEDYGMGPGR(*)	K6	60.64	1000	0.4179104	0.662337	0.366279	0.4821757
Ckap5	A2AGT5	1761	TLK(*)TLLHTLC(+57.02)K(*)	K3	59.89	171.6	-	-	0.766666667	
Ckap5	A2AGT5	940	QHVK(*)NLGIPVITVLGDSK(*)	K4	41.68	189.02	1.4	-	1.111111	1.2555556
Ckap5	A2AGT5	1799	MMK(*)HSMDQTGSK(*)	K3	33.2	119.03	1.06	-	1.233333	1.1466667
Ckap5	A2AGT5	200	DAVK(*)HTLQNINSVQLK(*)	K4	32.11	89.67	-	-	0.788888889	
Ckap5	A2AGT5	761	AFISNVK(*)TALAATNPAVR(*)	K7	20.1	1000	1.45	-	1.177777	1.3138889
Ubr4	A2AN08	4817	GQVVTK(*)TALLK(*)	K6	54.23	107.29	-	0.807692	0.956043	0.8818681
Ubr4	A2AN08	1432	FFTK(*)LFQLTEK(*)	K4	36.44	95.63	1.3888888	-	0.802197	1.0955433
Ubr4	A2AN08	3570	LIGSHTISK(*)VTVK(*)	K9	34.13	44.28	-	-	0.813186813	
Ubr4	A2AN08	920	EVEENWSK(*)HFSSDAAPQPR(*)	K8	17.1	1000	-	-	1.197802198	
Tpx2	A2APB8	58	GIGEPFQGK(*)NSLR(*)	K9	51.59	1000	1.6785714	0.704545	1.125	1.1693723
Tpx2	A2APB8	492	SPVFALK(*)NR(*)	K7	29.17	1000	-	0.693181	0.567307	0.6302448
Tpx2	A2APB8	523	AQPVPHYGVPYK(*)PHIAEAR(*)	K12	26.43	1000	-	-	0.634615385	
Tpx2	A2APB8	608	AEMWK(*)HQLEEEQK(*)	K5	8.65	131.72	-	-	1.028846154	
Ppig	A2AR02	61	STQK(*)PLHYK(*)SC(+57.02)LFHR(*	K9	75.58	61.26	0.75	-	0.765217	0.7576087
Ppig	A2AR02	705	SSTLK(*)NQEDEK(*)	K5	8.04	64.21	1.16	-	-	
Ppig	A2AR02	383	GDK(*)SELNEIK(*)	K3	4.58	34.57	-	-	0.773913043	
Upf2	A2AT37	364	EYFTSLTK(*)HLK(*)	K8	29.25	24.24	-	0.946236	0.850574	0.8984056
Rabgap1	A2AWA9	803	LMELAC(+57.02)NTK(*)ISQK(*)	K9	12.72	38.03	-	1.914634	-	
Mga	A2AWL7	2038	AGSK(*)NIDIMALQSIR(*)	K4	12.51	1000	-	-	-	
Arid1a	A2BH40	2018	LILLHHK(*)HPER(*)	K7	65	1000	0.8484848	0.479338	1.46875	0.9321912

Kansl3	A2RSY1	544	TK(*)VTTVTSTQK(*)	K2	22.53	54.49	-	-	-	
Trmt1l	A2RSY6	220	YIAASTAK(*)SSNEILK(*)	K8	34.07	34.57	-	-	0.539130435	
Chd6	A3KFM7	1903	GK(*)NLSFHQDEAK(*)	K2	62.92	235.31	0.8172043	0.631578	-	0.7243916
Phrf1	A6H619	768	GK(*)GIGSSFESFR(*)	K2	41.37	1000	-	0.530487	1.573529	1.0520086
Armt1	A6H630	40	HK(*)SEFFEK(*)	K2	79.62	95.35	-	1.015384	-	
Wdr76	A6PWY4	105	ALSSK(*)ADSLLLK(*)	K5	51.78	148.49	-	-	1.468085106	
Thada	A8C756	1649	LASK(*)IIAYR(*)	K4	28.47	1000	-	-	1.166666667	
Slfn9	B1ARD6	643	DFIQAK(*)NIC(+57.02)QAVTR(*)	K6	17.33	1000	0.9662921	-	1.176470	1.0713814
Thoc2	B1AZI6	1106	LTK(*)ASVHC(+57.02)LETGEYTHIR(*	K3	76.2	1000	0.8	0.897435	1.022727	0.9067211
	B2KF05		ESC(+57.02)SK(*)HASGTSFHLPQPSF	K5	72.96	1000	-	-	-	
Cad	B2RQC6	2036	HPQPGAVELAAK(*)HC(+57.02)R(*)	K12	94.76	1000	0.7916666	0.772151	0.989247	0.851022
Cad	B2RQC6	1325	NK(*)SELLPTVR(*)	K2	93.03	1000	0.8541666	0.898734	1.032258	0.9283863
Cad	B2RQC6	1411	LSSFVTK(*)GYR(*)	K7	83.3	1000	0.8541666	-	0.913978	0.8840726
Cad	B2RQC6	754	VSTK(*)IGSC(+57.02)MK(*)	K4	82.88	102.87	0.7291666	0.734177	0.978494	0.8139462
Cad	B2RQC6	1313	IPEK(*)NILLTIGSYK(*)	K4	77.12	149.94	0.8333333	0.696202	0.881720	0.8037521
Cad	B2RQC6	1934	QFTK(*)DQMSHLFNVAHTLR(*)	K4	62.91	1000	0.8958333	-	1	0.9479167
Cad	B2RQC6	2082	TITMVGDLK(*)HGR(*)	K9	50.08	1000	0.5520833	-	-	
Cad	B2RQC6	1323	NILLTIGSYK(*)NK(*)	K10	41.58	36.05	-	-	0.612903226	
Cad	B2RQC6	1625	K(*)EEILLIK(*)TAK(*)	K8	34.88	24.24	0.8854166	0.746835	0.913978	0.8487435
Cad	B2RQC6	2082	EELGTVNGMTITMVGDLK(*)HGR(*)	K18	27.7	1000	-	-	2.666666667	
Brpf1	B2RRD7	147	ENTETPAATPK(*)SGK(*)	K11	32.01	32.97	-	-	-	
Gnai1	B2RSH2	197	DLHFK(*)MFDVGGQR(*)	K5	40.03	1000	-	-	-	
Ep300	B2RWS6	1559	TSK(*)NK(*)SSLSR(*)	K5	90.41	1000	-	1.029411	0.84375	0.9365809
Ep300	B2RWS6	1557	TSK(*)NK(*)SSLSR(*)	K3	90.41	1000	-	1.029411	0.84375	0.9365809
Ep300	B2RWS6	1179	K(*)LEFSPQTLC(+57.02)C(+57.02)Y	K14	77.46	163.91	-	0.779411	0.9375	0.8584559
Ep300	B2RWS6	1541	EENTSNESTDVTK(*)GDSK(*)NAK(*)	K13	63.92	53.63	-	-	-	
Ep300	B2RWS6	1636	DK(*)HLEFSSLR(*)	K2	52.33	1000	-	0.882352	0.989583	0.9359681
Ep300	B2RWS6	1545	EENTSNESTDVTK(*)GDSK(*)NAK(*)K	K17	29.73	77.13	-	1.161764	0.895833	1.028799
Ep300	B2RWS6	1541	EENTSNESTDVTK(*)GDSK(*)NAK(*)K	K13	29.73	107.73	-	1.161764	0.895833	1.028799
Ep300	B2RWS6	1541	EENTSNESTDVTK(*)GDSK(*)	K13	25.62	31.27	-	1.823529	1.4375	1.6305147
Ep300	B2RWS6	1557	NNK(*)K(*)TSK(*)NK(*)	K7	21.84	30.83	-	0.382352	-	
Ep300	B2RWS6	1554	NNK(*)K(*)TSK(*)NK(*)	K4	21.84	100.21	-	0.382352	-	
Ep300	B2RWS6	1553	NNK(*)K(*)TSK(*)NK(*)	K3	21.84	123.86	-	0.382352	-	
Ep300	B2RWS6	1559	NK(*)SSLSR(*)	K2	13.14	1000	-	-	1.375	

Trappc11	B2RXC1	674	FVAK(*)TEDVGK(*)	K4	16.38	41.24	-	0.88	0.699029:	0.7895146
Plxnb2	B2RXS4	1239	IK(*)SQLEGLEESVR(*)	K2	3.87	1000	-	-	0.935483871	
Dock5	B2RY04	497	AIHPGAGYEGVSEYK(*)SVVYYQVK(*)	K15	36.28	150.89	-	-	1.108108108	
Rbm25	B2RY56	63	SIM(+15.99)APAPTVLVPTVSMVGK('	K19	53.18	1000	1.41	-	0.772727.	1.0913636
Rbm25	B2RY56	160	LLVK(*)VDAK(*)	K4	42.66	42.89	-	-	1.011363636	
Rbm25	B2RY56	734	HIK(*)SLIEK(*)	K3	11.26	81.69	1.43	-	1.25	1.34
Qtrt2	B8ZXI1	399	EALK(*)NDTLAQLK(*)	K4	34.42	75.39	-	-	0.87012987	
Mfap1b	C0HKD9	376	TILPK(*)VMQVK(*)	K5	25.81	38.33	-	-	0.547368421	
Mfap1b	C0HKD9	381	VMQVK(*)NFGR(*)	K5	9.03	1000	0.9897959	1.635135	-	1.3124655
Hist1h2an	C0HKE7	10	GK(*)QGGK(*)AR(*)	K6	99.68	1000	-	2.071428	-	
Hist1h2an	C0HKE7	6	GK(*)QGGK(*)AR(*)	K2	99.68	1000	-	2.071428	-	
Hist1h2an	C0HKE7	96	NDEELNK(*)LLGR(*)	K7	87.82	1000	0.9484536	0.892857	1.023255	0.9548555
Rnaset2b	C0HKG6	121	HEWVK(*)HGTC(+57.02)AAQVDALN	K5	22.71	70.13	-	-	0.637362637	
Samd1	D3YXK1	88	VSYK(*)GSISYR(*)	K4	13.76	1000	-	-	-	
Safb	D3YXK2	445	ATDLK(*)NLFSR(*)	K5	60.43	1000	1.0804597	0.959459	1.033707	1.0245424
Safb	D3YXK2	600	GVPVISVK(*)TSGSK(*)	K8	49.41	47.14	1.4022988	0.783783	1.460674:	1.2155856
Safb	D3YXK2	54	NLDSSGNK(*)SVLMER(*)	K8	17.99	1000	1.2988505	2.148648	1.595505	1.6810016
Obsl1	D3YYU8	261	TFWVNEGK(*)HAK(*)	K8	70.88	20.7	-	-	1.139240506	
Obsl1	D3YYU8	687	LILQAVK(*)HR(*)	K7	32.41	1000	1.6024096	1.232876	1.265822	1.3670364
Ccdc6	D3YZP9	259	FLK(*)NEVER(*)	K3	61.85	1000	0.9203539	0.790697	0.849056	0.8533694
Gls	D3Z7P3	169	FITALK(*)STGLR(*)	K6	87.5	1000	0.7741935	0.835443	0.882978	0.8308718
Gls	D3Z7P3	284	HSIGDTK(*)VPFC(+57.02)LQSC(+57	K7	13.98	55.8	1.2043010	0.835443	-	1.0198721
Gcn1	E9PVA8	2183	SK(*)ADYSSHLR(*)	K2	65.79	1000	0.7575757	-	1.021978	0.8897769
Gcn1	E9PVA8	2418	FVIQGAGSK(*)VDAAIR(*)	K9	65.14	1000	0.7575757	0.636363	0.868131	0.7540238
Gcn1	E9PVA8	914	LK(*)TLGTLVSHVTLR(*)	K2	55.52	1000	0.6868686	-	0.989010	0.8379398
Gcn1	E9PVA8	436	VFSLK(*)TSTSAVR(*)	K5	54.93	1000	0.9393939	1.025974	0.857142	0.9408369
Gcn1	E9PVA8	308	GLANQLK(*)SNSPR(*)	K7	51.75	1000	1.0808080	0.779220	1.186813	1.015614
Gcn1	E9PVA8	929	LLK(*)PEC(+57.02)ALDK(*)	K3	45.75	41.33	0.6868686	0.597402	1.186813	0.8236948
Gcn1	E9PVA8	936	LLK(*)PEC(+57.02)ALDK(*)SWC(+57	K10	40.72	23.7	-	-	0.78021978	
Gcn1	E9PVA8	561	LTNSK(*)VQQYYR(*)	K5	36.8	1000	0.8484848	-	1.868131	1.3583084
Gcn1	E9PVA8	268	DLILPTIQK(*)SLLR(*)	K9	6.16	1000	-	-	1.384615385	
Mki67	E9PVX6	1136	TGLSK(*)VDVR(*)	K5	62	1000	0.8229166	0.675675	1.528735	1.0091093
Mki67	E9PVX6	156	SFVYAK(*)GLSADSPASDGSK(*)	K6	44.45	119.02	1.2916666	0.986486	0.816091	1.031415
Mki67	E9PVX6	275	AK(*)SSGSTPVTAASSPK(*)	K2	43.75	185.37	0.9166666	0.554054	-	0.7353604

Mki67	E9PVX6	1372	LSK(*)TDLSK(*)	K3	29.86	61.26	-	1.216216	3.252873	2.2345449
Mki67	E9PVX6	1721	LAK(*)TSVGNIAVR(*)	K3	27.6	1000	-	-	1.114942529	
Mki67	E9PVX6	2928	DHATLQSK(*)SNPLLSPK(*)	K8	10.21	21.55	-	0.716216	-	
Prr12	E9PYL2	615	GK(*)GDGSELLAGPGGSAAER(*)	K2	83.07	1000	-	-	-	
Ascc3	E9PZJ8	573	ELTGDMQLSK(*)SEILR(*)	K10	88.87	1000	0.8811881	0.826666	0.835051	0.8476354
Ascc3	E9PZJ8	1826	IASYYYLK(*)HK(*)	K8	57.12	22.85	0.7326732	0.653333	0.855670	0.7472256
Ascc3	E9PZJ8	302	FLNSSSDHK(*)FQVLQDSC(+57.02)K	K9	36.05	100.79	-	-	0.886597938	
Ascc3	E9PZJ8	1145	LEEK(*)NLTVDK(*)	K4	31.59	121.92	0.6336633	1.28	0.876288	0.929984
Chd2	E9PZM4	623	LK(*)NDDSLLYK(*)	K2	46.33	117.11	-	-	1.739130435	
Arid1b	E9Q4N7	603	SQPPLAPGK(*)SNHEDLNLIQQER(*)	K9	96.63	1000	0.7884615	0.551724	0.759259	0.699815
Rnf213	E9Q555	483	STGFFQQAK(*)NR(*)	K9	41.76	1000	-	-	0.814814815	
Rnf213	E9Q555	4210	FLK(*)HVEEFC(+57.02)TR(*)	K3	39.76	1000	1.1	0.371794	1	0.8239316
Rnf213	E9Q555	3130	LEK(*)HYLDMNTVLQPWQK(*)	K3	28.79	88.07	-	-	0.314814815	
Nolc1	E9Q5C9	123	ASVPQHAGK(*)AAAK(*)	K9	40.75	55.36	0.7934782	1.038461	0.956521	0.9294872
Setd2	E9Q5F9	360	SSAPSK(*)SEDLGK(*)	K6	44.16	23.13	-	-	-	
Kif23	E9Q5G3	398	LTHLFK(*)NYFDGEGK(*)	K6	30.07	131.34	0.2072072	-	0.561224	0.3842158
Ythdc1	E9Q5K9	90	GK(*)SVTEYK(*)	K2	79.83	123.86	1.0985915	0.826086	0.909090	0.9445898
Bod1l	E9Q6J5	534	SQGK(*)STVDLEDSSAK(*)	K4	69.16	176.98	1.71	0.743589	1.269662	1.2410842
Bod1l	E9Q6J5	1386	ISIPLADK(*)SMSVTGDNK(*)	K8	36.84	67.89	0.91	0.935897	1.235955	1.0272842
Numa1	E9Q7G0	907	EVAC(+57.02)LK(*)TLVLK(*)	K6	49.36	81.69	1.7204301	0.855263	0.846153	1.1406157
Numa1	E9Q7G0	977	AALIK(*)SQGQQQEER(*)	K5	7.69	1000	1.4408602	-	-	
Tpr	F6ZDS4	787	SQNTK(*)ISTQLDFASK(*)	K5	97.89	251.58	1.0439560	0.948051	0.977528	0.9898454
Tpr	F6ZDS4	326	TSNEHLQK(*)HVEDLLTK(*)	K8	88.61	73.05	1.0769230	0.935064	1.011235	1.0077413
Tpr	F6ZDS4	1750	IK(*)PTPVVSTPSK(*)VTAAAMAGNK(	K12	77.36	66.12	0.8681318	0.584415	0.820224	0.7575907
Tpr	F6ZDS4	121	FK(*)VESEQQYFEIEK(*)	K2	62.18	294.89	1.4065934	1.584415	1.089887	1.3602989
Tpr	F6ZDS4	1062	QVTEEVHK(*)NIEVR(*)	K8	61.83	1000	1.3076923	1.012987	1.269662	1.1967807
Tpr	F6ZDS4	373	SAADDSEAK(*)SNELTR(*)	K9	45.67	1000	-	-	1.314606742	
Tpr	F6ZDS4	1030	LK(*)TSTSNVEQYR(*)	K2	44.24	1000	1.3296703	0.870129	1.191011	1.1302705
Tpr	F6ZDS4	1673	MTALK(*)SQYEGR(*)	K5	40.58	1000	1.0329670	1.025974	0.910112	0.9896845
Tpr	F6ZDS4	364	LSNLYK(*)SAADDSEAK(*)	K6	38.12	107.04	1.0219780	1.090909	1.011235	1.0413744
Tpr	F6ZDS4	1609	QDLQDK(*)TTEEQLR(*)	K6	34.6	1000	-	-	0.865168539	
Tpr	F6ZDS4	1563	DTLSQSETK(*)TK(*)	K9	29.37	25.66	1.7142857	-	1.426966	1.570626
Tpr	F6ZDS4	551	ANK(*)HSSVLER(*)	K3	20.99	1000	-	1.038961	1.280898	1.15993
Tpr	F6ZDS4	1718	QITLK(*)TTPASGER(*)	K5	9.24	1000	1.3406593	0.857142	-	1.0989011

Farp1	F8VPU2	696	LMHYK(*)HVLER(*)	K5	82.16	1000	0.9222222	0.825	0.945652:	0.8976248
Farp1	F8VPU2	775	LSGK(*)GLQQR(*)	K4	60.72	1000	-	0.9625	-	
Farp1	F8VPU2	638	NIQGMK(*)HLAAHLWK(*)	K6	56.35	192.66	1.1888888	-	0.956521	1.0727053
Farp1	F8VPU2	1006	LHFK(*)SHVYYFR(*)	K4	51.66	1000	-	-	0.836956522	
Farp1	F8VPU2	646	HLAAHLWK(*)HSEALEALETSIK(*)	K8	46.09	137.95	1.0666666	-	1.119565:	1.0931159
Farp1	F8VPU2	265	INAFNWAK(*)VR(*)	K8	44.42	1000	0.7666666	-	0.706521	0.7365942
Farp1	F8VPU2	303	DFC(+57.02)K(*)SFWK(*)	K4	32.41	76.73	1.6	-	0.956521	1.2782609
Farp1	F8VPU2	632	IGDVMLK(*)NIQGMK(*)	K7	31.37	81.36	-	1.5625	1.130434	1.3464674
Farp1	F8VPU2	704	LC(+57.02)K(*)HHPPNHADFR(*)	K3	19.65	1000	-	0.925	1.097826(	1.011413
Atp2b1	G5E829	590	SMSTVLK(*)NSDGSFR(*)	K7	64.68	1000	-	0.666666	-	
Atp2b1	G5E829	601	IFSK(*)GASEIILK(*)	K4	43.6	92.28	-	-	0.773809524	
Atp2b1	G5E829	476	HLDAC(+57.02)ETMGNATAIC(+57.(	K18	24.67	1000	1.1222222	-	-	
Atp2b1	G5E829	64	LK(*)TSPNEGLSGNPADLER(*)	K2	18.64	1000	-	-	1.166666667	
Trip12	G5E870	181	LASK(*)SATSAK(*)	K4	21.02	55.13	-	0.846153	1.120481!	0.9833179
Tubgcp6	G5E8P0	1620	QLQLFK(*)HEMQHFVK(*)	K6	9.98	30.66	-	-	-	
	G5E8P1		GK(*)PALVR(*)	K2	98.25	1000	-	-	-	
	G5E8P1		HPSSPC(+57.02)SIK(*)HSPTR(*)	K9	27.02	1000	-	-	-	
	G5E8P1		IGEHMQTK(*)SEEK(*)	K8	23.21	51.8	-	-	-	
	G5E8P1		SELISC(+57.02)IENGNYAK(*)AAR(*)	K14	5.33	1000	-	-	-	
Capn2	O08529	414	GC(+57.02)TFLVGLIQK(*)HR(*)	K11	71.91	1000	0.9285714	0.8125	0.913043	0.884705
Capn2	O08529	260	LVK(*)GHAYSVTGAEEVESSGSLQK(*)	K3	45.04	210.04	0.8265306	0.7875	-	0.8070153
Capn2	O08529	568	R(*)QDIK(*)SDGFSIETC(+57.02)K(*)	K5	41.91	187.15	0.7142857	-	0.858695(	0.7864907
Capn2	O08529	360	LTK(*)MDGNWR(*)	K3	41.75	1000	0.8979591	-	0.902173!	0.9000665
Capn2	O08529	568	QDIK(*)SDGFSIETC(+57.02)K(*)	K4	35.98	111.13	2.3367346	0.9	-	1.6183673
Capn2	O08529	69	ELGPYSSK(*)TR(*)	K8	32.37	1000	4.4081632	-	1.576086!	2.9921251
Capn2	O08529	603	EFYILWTK(*)IQK(*)	K8	11.49	22.34	0.8775510	-	1.108695(	0.9931233
Bin1	O08539	7	A(+42.01)EMGSK(*)GVTAGK(*)	K6	41.89	69.64	0.8901098	0.628205	0.761363(	0.7598929
Dpysl2	O08553	258	VMSK(*)SAAEVIAQAR(*)	K4	56.25	1000	0.7835051	0.653333	1.797752!	1.0781971
Dpysl2	O08553	423	TISAK(*)THNSALEYNIFEGMEC(+57.(	K5	43.99	1000	-	1.813333	0.775280!	1.2943071
Dpysl2	O08553	368	MSVIWDK(*)AVVTGK(*)	K7	30.77	37.05	0.5773195	-	-	
Emd	O08579	116	TYGEPESVGMSK(*)SFR(*)	K12	48.53	1000	1.7326732	0.906976	1.446808!	1.3621528
Gtpbp1	O08582	537	TGDK(*)ATVHFR(*)	K4	19.55	1000	-	0.831460	1.058823!	0.9451421
Alyref	O08583	85	QLPDK(*)WQHDLFDSGFGGGAGVET	K5	80.52	164.82	-	-	0.978021978	
Alyref	O08583	233	NSK(*)QQLSAEELDAQLDAYNAR(*)	K3	19.17	1000	2.0104166	-	1.648351(	1.8293842

Stxbp1	O08599	294	HK(*)HIAEVSQEVTR(*)	K2	45.03	1000	-	0.746666	0.734042!	0.7403546
Myh11	O08638	817	QQQLTAMK(*)VIQR(*)	K8	45.51	1000	-	-	-	
Metap2	O08663	385	GVVHDDMEC(+57.02)SHYMK(*)NFI	K14	54.67	1000	0.9541284	1.022988	1.208333:	1.0618168
Metap2	O08663	400	TK(*)HLLNVINENFGTLAFC(+57.02)F	K2	44.18	1000	-	-	0.916666667	
Prdx6	O08709	106	LPFPIIDDK(*)GR(*)	K9	58.06	1000	1.0659340	1.014705	1.131868:	1.070836
Ampd3	O08739	333	FIK(*)HTYQTEPDR(*)	K3	26.9	1000	-	-	0.454545455	
Polr2j	O08740	62	DPQVLFAGYK(*)VPHPLEHK(*)	K10	46.77	29.7	-	0.063492	-	
Polr2j	O08740	47	EDHTLGNIIK(*)SQLLK(*)	K10	24.33	55.6	-	-	0.385714286	
Dld	O08749	66	SAQLGFK(*)TVC(+57.02)IEK(*)	K7	74.24	134.33	0.94	0.805194	1.102040!	0.9490785
Dld	O08749	127	MMEQK(*)HSAVK(*)	K5	70.84	89.05	0.82	0.818181	-	0.8190909
Dld	O08749	159	ITGK(*)NQVTATK(*)	K4	67.87	142.27	-	-	0.755102041	
Dld	O08749	430	AK(*)TNADTDGMVK(*)	K2	61.77	111.69	0.96	0.961038	0.877551(	0.9328633
Dld	O08749	104	ALLNNSHYYHMAHGK(*)DFASR(*)	K15	15.42	1000	1.6	1.545454	0.877551(	1.3410019
Dld	O08749	143	ALTGGIAHLFK(*)QNK(*)	K11	11.73	28.36	-	-	1.020408163	
Hsd17b10	O08756	107	NK(*)IHTLEDFQR(*)	K2	51.51	1000	1.1648351	1.111111	1.025974(	1.1006401
Ube3a	O08759	28	AAAK(*)HLIER(*)	K4	50.88	1000	0.8842105	-	1.257425:	1.0708181
Tcof1	O08784	327	GPILATPGK(*)TGPAATQAK(*)	K9	85.7	71.74	1.0263157	0.892857	1.170454!	1.0298758
Tcof1	O08784	144	TVNSVSHPGSGK(*)TVVHLLSGK(*)	K12	76.08	50.04	1.2105263	1.125	-	1.1677632
Tcof1	O08784	521	VR(*)PVATQVK(*)TDR(*)	K9	69.36	1000	0.7763157	-	1.136363(	0.9563397
Tcof1	O08784	468	GANLPC(+57.02)PGK(*)VGSAALR(*)	K9	66.54	1000	0.9605263	1.267857	1.215909(	1.1480975
Tcof1	O08784	132	TANK(*)TVNSVSHPGSGK(*)	K4	65.02	106.18	1.5131578	1.357142	0.954545	1.2749487
Tcof1	O08784	153	TVVHLLSGK(*)SPK(*)	K9	56.7	49.37	1.3552631	-	-	
Tcof1	O08784	318	GISGK(*)GPILATPGK(*)	K5	53.96	140.79	-	1.232142	-	
Tcof1	O08784	630	VQGK(*)SGGK(*)GLQGK(*)	K8	41.41	58	-	0.303571	-	
Tcof1	O08784	626	VQGK(*)SGGK(*)GLQGK(*)	K4	41.41	123.21	-	0.303571	-	
Tcof1	O08784	635	GLQGK(*)AALGQGVAPVHTQK(*)	K5	41.03	102.84	0.7631578	0.625	1.181818:	0.8566587
Tcof1	O08784	1092	SK(*)SQHAADTALPK(*)	K2	28.43	213.66	-	1.178571	-	
Tcof1	O08784	442	VK(*)PLGK(*)SSQVR(*)PVSTVTPGSS(	K6	25.94	42.89	1	0.964285	1.363636:	1.1093074
Tcof1	O08784	442	VK(*)PLGK(*)SSQVR(*)	K6	19.01	48.12	-	1.535714	-	
Tcof1	O08784	922	AASQPSK(*)SEQSSR(*)	K7	13.89	1000	1.1578947	-	-	
Tcof1	O08784	241	TAPGPTK(*)LGNVAPTPAK(*)	K7	11.62	59.2	-	2.982142	-	
Dctn1	O08788	262	IQLEQVQEWK(*)SK(*)	K10	72.76	33.18	0.6336633	0.75	0.706521:	0.6967284
Dctn1	O08788	38	VEVIGK(*)GHR(*)	K6	63.26	1000	-	-	0.989130435	
Dctn1	O08788	618	LIC(+57.02)K(*)AELIR(*)	K4	57.3	1000	-	-	0.815217391	

Prkcsh	O08795	149	ILIEEWK(*)TAR(*)	K7	64.06	1000	0.5104166	0.519480	0.774193	0.6013636
Prkcsh	O08795	431	PK(*)HGGSPTSLGTWGSWAGPDHD	K2	28.98	100.67	-	-	1.784946237	
Prkcsh	O08795	480	LLC(+57.02)GK(*)ETVVTSTTEPSR(*)	K5	20.75	1000	1.8541666	0.831168	0.548387	1.0779075
Prdx4	O08807	243	QYTDK(*)HGEVC(+57.02)PAGWK(*)	K5	5.27	68.81	-	-	1.585106383	
Eftud2	O08810	935	EFMIK(*)TR(*)	K5	60.93	1000	-	-	0.769230769	
Eftud2	O08810	243	LIK(*)HAVQER(*)	K3	50.55	1000	0.9890109	1.077922	1.087912	1.0516151
Eftud2	O08810	351	LWGDIYFNPK(*)TR(*)	K10	33.57	1000	-	0.896103	1.197802	1.046953
Eftud2	O08810	141	NVTLC(+57.02)GHLHHGK(*)TC(+57	K12	28.59	1000	-	-	1.56043956	
RO60	O08848	239	TK(*)DDLEVIHLIEEHQLVR(*)	K2	13.67	1000	-	-	0.968085106	
Aip	O08915	103	HVVLYPLVAK(*)SLR(*)	K10	36.77	1000	-	1.038461	0.912087	0.9752747
Aip	O08915	273	GK(*)AHAAVWNAQEAQADFAK(*)	K2	7.56	140.19	-	3.051282	-	
Aip	O08915	210	YYDAIAC(+57.02)LK(*)NLQMK(*)	K9	3.16	38.32	-	-	2.043956044	
Sdcbp	O08992	215	DSSGHVGFIFK(*)SGK(*)	K11	29.15	20.7	0.1367521	-	0.463035	0.2998936
Sdcbp	O08992	180	VLK(*)QAFGEK(*)	K3	14.32	85.52	0.0987654	0.484662	0.377431	0.3202866
Atox1	O08997	60	TGK(*)AVSYLGPK(*)	K3	95.37	172.06	0.8679245	0.948051	0.865168	0.893715
Atox1	O08997	3	PK(*)HEFSVDMTC(+57.02)EGC(+57.	K2	91.8	1000	0.8396226	0.948051	0.943820	0.9104983
Atox1	O08997	57	VC(+57.02)IDSEHSSDTLLATLNK(*)T	K18	59.24	53.53	-	0.870129	0.831460	0.8507953
Ncoa3	O09000	88	EQGK(*)TISSDDDVQK(*)	K4	64.62	185.36	0.6783625	-	-	
Ncoa3	O09000	861	AVSLDSPVSVGSGPPVK(*)NVSAFPG	K17	21.35	85.7	-	-	-	
Psmb1	O09061	76	LTDK(*)TVIGC(+57.02)SGFHGDC(+5	K4	53.31	103.39	1.0752688	0.610389	0.938144	0.8746009
Map2k3	O09110	183	ALEHLHSK(*)LSVIHR(*)	K8	75.8	1000	1.6103896	0.934210	1.048192	1.1975976
Sypl1	O09117	57	GK(*)TEIQVNC(+57.02)PK(*)	K2	43.58	225.98	1.0416666	-	0.922222	0.9819444
Gsto1	O09131	59	NK(*)PEWFFEK(*)	K2	91.73	136.94	0.8125	-	0.967032	0.8897665
Gsto1	O09131	57	HEVININLK(*)NK(*)PEWFFEK(*)	K9	77.41	22.85	0.6979166	-	1.285714	0.9918155
Gsto1	O09131	219	IDAK(*)TYR(*)	K4	75.19	1000	0.90625	0.886075	1.098901	0.9637423
Gsto1	O09131	122	SK(*)VPPLIASFVR(*)	K2	29.73	1000	0.8645833	-	-	
Rpl21	O09167	97	IEHIK(*)HSK(*)	K5	98.57	77.24	-	0.845238	0.915789	0.8805138
Rpl21	O09167	60	C(+57.02)YHGK(*)TGR(*)	K5	90.29	1000	-	0.904761	0.968421	0.9365915
Rpl21	O09167	43	GDIVDIK(*)GMGTVQK(*)	K7	80.43	184.45	0.9387755	0.714285	0.989473	0.880845
Rpl21	O09167	50	GMGTVQK(*)GMPHK(*)	K7	66.04	77.53	-	0.869047	0.8	0.8345238
Rpl21	O09167	43	K(*)GDIVDIK(*)GMGTVQK(*)	K8	55.27	72.34	1.2857142	1.238095	1.178947	1.2342523
Rpl21	O09167	122	EK(*)GTWVQLK(*)	K2	23.19	100.91	1.0306122	0.916666	1.073684	1.0069877
Gclm	O09172	255	YSVIVK(*)SR(*)	K6	62.76	1000	0.9893617	0.7375	1.054347	0.9270698
Gclm	O09172	263	SK(*)GYILQAK(*)	K2	28.17	127.53	1.1702127	0.6125	-	0.8913564

Grcc10	O35127	86	LVK(*)SYEAQDPEIASLSGK(*)	K3	48.94	199.78	-	-	0.431137725	
Phb2	O35129	262	AAQNISK(*)TIATSQNR(*)	K7	42.82	1000	1.1649484	0.761904	1.095744(	1.0075326
Phb2	O35129	244	MLGEALSK(*)NPGYIK(*)	K8	34.94	47.38	-	1.261904	0.712765!	0.9873354
Emg1	O35130	70	HK(*)SMLLK(*)	K2	63.83	98.37	0.7349397	-	1.088888	0.9119143
Emg1	O35130	59	VGK(*)TYELLNC(+57.02)DR(*)	K3	60.48	1000	0.8554216	0.986666	0.944444	0.9288443
Polr1a	O35134	1058	SK(*)HLHEVLSR(*)	K2	32.6	1000	1.0760869	0.943820	0.914893(	0.9782669
ATP5IF1	O35143	64	TK(*)EQLAALR(*)	K2	51.99	1000	-	0.673913	0.958762	0.816338
Slc30a4	O35149	395	AK(*)HLLLNTFGMYK(*)	K2	6.14	99.02	-	-	1.126506024	
Psmd4	O35226	40	LQAQQDAVNIVC(+57.02)HSK(*)TR(	K15	90.02	1000	0.9247311	0.810810	0.967391	0.9009778
Psmd4	O35226	74	ILSK(*)LHTVQPK(*)	K4	63.03	148.49	1.4516129	0.837837	0.945652	1.0783676
Psmd4	O35226	98	VAHLALK(*)HR(*)	K7	58.49	1000	0.8924731	-	1	0.9462366
Psmd4	O35226	83	GK(*)ITFC(+57.02)TGIR(*)	K2	4.81	1000	-	-	3	
Ptpn9	O35239	218	IQILK(*)TSEVTQHLPR(*)	K5	11.17	1000	-	-	1.636363636	
Dhx15	O35286	491	AYK(*)TEMQDNTYPEILR(*)	K3	71.89	1000	0.8020833	0.841463	1	0.8811822
Dhx15	O35286	744	VLTTK(*)NYIR(*)	K5	58.32	1000	0.96875	0.939024	0.927083	0.9449526
Purb	O35295	72	FLK(*)IAEVGAGGSK(*)	K3	22.05	85.84	-	-	1.082191781	
Srsf5	O35326	25	FFK(*)GYGR(*)	K3	51.66	1000	0.7472527	1.307692	1.012195	1.0223801
Capn1	O35350	426	ESGC(+57.02)SFLLALMQK(*)HR(*)	K13	38.5	1000	-	-	1.06741573	
Capn1	O35350	266	DLEAITFK(*)NLVR(*)	K8	18.75	1000	-	-	1.370786517	
Abcc1	O35379	397	IK(*)TAVVGAVYR(*)	K2	71.7	1000	0.6666666	0.607843	1.094117(	0.7895425
Anp32a	O35381	20	TPSDVK(*)ELVLDNC(+57.02)K(*)	K6	63.22	150.89	0.9905660	0.918918	1.104651	1.004712
Anp32a	O35381	91	C(+57.02)PNLK(*)HLNLSGNK(*)	K5	29.48	150.63	1.5	0.918918	1.848837	1.4225854
Anp32a	O35381	91	LK(*)HLNLSGNK(*)	K2	28.41	115.01	0.3490566	-	0.511627!	0.4303423
Hax1	O35387	210	SYFK(*)SISVTK(*)	K4	16.42	79.01	-	-	-	
Crcp	O35427	34	NK(*)HSAGQQNLNAITYETLK(*)	K2	55.65	137.15	0.9772727	0.972602	0.976744	0.9755399
Dhodh	O35435	99	NPVGIAAGFDK(*)HGEAVDGLYK(*)	K11	27.94	21.3	-	-	1.181818182	
Dhodh	O35435	226	SLQGK(*)TELR(*)	K5	24.56	1000	-	-	0.886363636	
Dhodh	O35435	185	NK(*)TSVDAAADYVEGVR(*)	K2	5.64	1000	-	-	1.238636364	
Psmd14	O35593	253	EMLELAK(*)NYNK(*)	K7	66.12	33.81	0.7765957	0.784810	-	0.7807029
Psmd14	O35593	154	GK(*)VVIDAFR(*)	K2	50.16	1000	0.7127659	-	0.912087!	0.8124269
Pmm1	O35621	177	TEFAGK(*)GLR(*)	K6	6.47	1000	-	-	1.238095238	
Bet1	O35623	58	SLSIEIGHEVK(*)NQNK(*)	K11	55.93	38.26	1.0784313	-	0.664	0.8712157
Stag2	O35638	607	LEK(*)HLDALLR(*)	K3	68.41	1000	0.9545454	0.876543	0.945652	0.9255803
Stag2	O35638	622	NIVEK(*)HTDTDVLEAC(+57.02)SK(*	K5	32.99	160.66	-	1.222222	1.271739	1.2469807

Anxa3	O35639	263	LHQALK(*)GAGTDEFTLNR(*)	K6	56.02	1000	1.1530612	0.927710	1.712765!	1.2645127
Ap1b1	O35643	318	R(*)PEILK(*)HEMK(*)	K6	55.69	82.24	0.8	0.876712	0.869565:	0.8487592
C1qbp	O35658	171	FVVEVTK(*)TDGK(*)	K7	43.77	28.7	0.9052631	-	0.680851(	0.7930571
Nudc	O35685	197	GK(*)DVVVDIQR(*)	K2	88.92	1000	1	1	1.011627!	1.003876
Nudc	O35685	251	INK(*)MEWWNR(*)	K3	77.28	1000	0.9111111	1.025641	0.883720!	0.9401577
Nudc	O35685	298	QK(*)SMGLPTSDEQK(*)	K2	45.62	170.43	0.8666666	0.794871	1.127906!	0.9298151
Nudc	O35685	276	INPENSK(*)LSDLDSETR(*)	K7	29.81	1000	-	-	1.139534884	
Pnn	O35691	20	ESLK(*)NVDENIR(*)	K4	23.72	1000	0.3693693	0.974683	1.876288(	1.0734472
Sptlc1	O35704	210	LFK(*)HNDVADLER(*)	K3	24	1000	1.8181818	0.978260	0.75	1.1821476
Hnrnph1	O35737	185	YIEIFK(*)SSR(*)	K6	95.17	1000	1.0309278	0.987012	1.057471:	1.0251374
Hnrnph1	O35737	98	SNNVEMDWVLK(*)HTGPNSPDTANI	K11	80.76	1000	0.5979381	0.909090	0.931034	0.8126878
Api5	O35841	398	LALQGK(*)TGEALK(*)	K6	82.47	132.53	0.7765957	0.7625	0.880434	0.8065102
Api5	O35841	202	ILSGLK(*)SLQTVSGR(*)	K6	68.35	1000	0.8297872	-	1.054347!	0.9420675
Api5	O35841	251	QAVPLFSK(*)NVHSTR(*)	K8	27.17	1000	1	0.975	0.858695(	0.9445652
Api5	O35841	160	LK(*)TLPDEVLTK(*)	K2	9.59	75.99	-	1.3	-	
Api5	O35841	404	TGEALK(*)TEENK(*)	K6	3.7	71.64	-	-	1.206521739	
Timm44	O35857	138	ESLDEVSK(*)SDLGR(*)	K8	14.43	1000	-	1.0375	-	
Cops5	O35864	180	TISAGK(*)VNLGAFR(*)	K6	42.24	1000	1.2234042	0.813333	0.935483!	0.9907405
Slc1a4	O35874	31	TPETAMGK(*)SQR(*)	K8	11.42	1000	1.1978021	-	-	
Calu	O35887	287	LTK(*)EEIVDK(*)	K3	48.6	129.72	0.8387096	0.961538	0.867469!	0.8892393
Calu	O35887	79	IVSK(*)IDDDK(*)DGFVTVDELK(*)	K4	30.95	109.69	0.6666666	1.051282	1.096385!	0.9381114
Calu	O35887	114	QWK(*)GHDLNEDGLVSWEEYK(*)	K3	25.97	144.89	0.8602150	-	1.084337:	0.9722762
Lsm2	O35900	58	MLSVK(*)NC(+57.02)FIR(*)	K5	78.4	1000	0.9397590	0.857142	1.032258(	0.9430533
Lsm2	O35900	58	YPHMLSVK(*)NC(+57.02)FIR(*)	K8	67.11	1000	0.8795180	0.623376	0.956989:	0.8199613
Cavin1	O54724	319	TAVYK(*)VPPFTFHVK(*)	K5	75.58	85.31	1.0681818	-	0.811111	0.9396465
Cavin1	O54724	154	NFK(*)VMIYQDEVK(*)	K3	57.79	145	1.1931818	0.961038	1.077777	1.0773329
Cavin1	O54724	124	VSVNVK(*)TVR(*)	K6	44.64	1000	1.1818181	0.870129	1.055555!	1.0358345
Cavin1	O54724	124	K(*)VSVNVK(*)TVR(*)	K7	18.42	78.19	0.5909090	-	0.944444	0.7676768
Ddost	O54734	174	APTIVGK(*)SSLNPILFR(*)	K7	72.54	1000	0.6344086	-	0.758620(	0.6965146
Ap3d1	O54774	580	ASC(+57.02)ILQLVK(*)HVQK(*)	K9	58.1	82.24	1.1034482	0.907894	1	1.003781
Srpk2	O54781	111	VVK(*)SAQHYTETALDEIK(*)	K3	32.15	130.55	1.2903225	-	0.953703	1.1220131
Bysl	O54825	15	EK(*)HAPLAEQILAGNAVR(*)	K2	37.33	1000	-	-	0.655913978	
Bysl	O54825	389	YK(*)ADLATEQK(*)	K2	32.77	99.03	-	1.171428	1.118279!	1.1448541
Csnk2a2	O54833	151	ALDYC(+57.02)HSK(*)GIMHR(*)	K8	52.91	1000	0.7821782	0.962025	1.011363(	0.9185224

Hmgb3	O54879	112	IK(*)STNPGISIGDVAK(*)	K2	65.85	208.61	1.0714285	1.481012	1.461538	1.3379932
Reps1	O54916	105	FVASK(*)NEQESR(*)	K5	13.59	1000	-	-	0.836538462	
Dnajb6	O54946	314	LGSK(*)SNWEDDEQDR(*)	K4	34.69	1000	-	-	0.794871795	
Prkag1	O54950	99	YYK(*)SALVQIYELEEHK(*)	K3	48.96	206.61	1	-	-	
Asna1	O54984	50	GGVGK(*)TTC(+57.02)SC(+57.02)SL	K5	61.73	244	-	0.546666	1.045977	0.7963218
Slk	O54988	1017	HLQEK(*)HQLLK(*)	K5	77.73	115.97	0.85	0.641975	1.022222	0.8380658
Slk	O54988	179	LADFGVSAK(*)NTR(*)	K9	56.63	1000	1.06	0.901234	1.133333	1.0315226
Slk	O54988	1059	LIEELK(*)NR(*)	K6	30.56	1000	-	1.098765	1.022222	1.0604938
Slk	O54988	249	IAK(*)SEPPTLAQPSK(*)	K3	20.89	56.41	-	1.382716	0.9	1.141358
Impa1	O55023	28	EALK(*)NEMDVMIK(*)	K4	12.92	100.36	-	-	1.52173913	
Bckdk	O55028	192	YFLDK(*)TLTSR(*)	K5	34.12	1000	0.3904109	0.439153	-	0.4147822
Copb2	O55029	318	IIWAK(*)HSEVQQANLK(*)	K5	70.65	213.35	1.1052631	0.858974	1	0.9880792
Copb2	O55029	212	LVK(*)IWDYQNK(*)	K3	60.65	148.49	0.9368421	0.910256	0.952941	0.9333466
Copb2	O55029	386	NK(*)SFGSAQEFAWAHDSSEYAIR(*)	K2	48.56	1000	1.0105263	-	1.023529	1.0170279
Impact	O55091	278	FK(*)HINNC(+57.02)AR(*)	K2	8.79	1000	-	-	1.325581395	
Nipsnap2	O55126	77	LQFHNVK(*)PEC(+57.02)LDAYNK(*)	K7	42.69	66.16	-	-	1.073394495	
Sap18	O55128	24	EK(*)TC(+57.02)PLLLR(*)	K2	24.55	1000	0.4271844	-	-	
Septin7	O55131	194	IAK(*)ADTLTPEEC(+57.02)QQFK(*)	K3	34.72	156.91	-	-	1.27173913	
Septin7	O55131	185	LHEK(*)VNIIPLIAK(*)	K4	28.09	161.68	-	-	0.891304348	
Septin7	O55131	96	TVQVEQSK(*)VLIK(*)	K8	27.9	38.26	-	1.204819	0.576086	0.8904531
Rpl35a	O55142	73	VIWGK(*)VTR(*)	K5	94.54	1000	0.7685185	0.691489	0.769911	0.7433065
Rpl35a	O55142	29	EHTALLK(*)IEGVYAR(*)	K7	93.54	1000	0.8611111	0.776595	0.601769	0.7464923
Rpl35a	O55142	45	DETEFYLGK(*)R(*)	K9	84.11	1000	0.9444444	0.531914	0.654867	0.7104089
Rpl35a	O55142	8	LWC(+57.02)K(*)AIFAGYK(*)	K4	80.16	122.76	0.7592592	0.702127	0.769911	0.7437661
Rpl35a	O55142	15	AIFAGYK(*)R(*)	K7	50.46	1000	0.7222222	0.851063	0.787610	0.7869656
Rpl35a	O55142	54	AK(*)NNTVTPGGK(*)	K2	33.95	123.21	-	-	0.814159292	
Atp2a2	O55143	169	LTSIK(*)STTLR(*)	K5	68.46	1000	0.82	0.683544	0.815217	0.7729206
Atp2a2	O55143	189	VDQSILTGESVSVIK(*)HTDPVPDPR(*	K15	55.96	1000	0.81	-	0.586956	0.6984783
Atp2a2	O55143	533	VGSTK(*)VPMTPGVK(*)	K5	55.46	64	0.96	0.759493	1.010869	0.9101211
Atp2a2	O55143	514	MFVK(*)GAPEGVIDR(*)	K4	23.79	1000	-	1.012658	-	
Ilk	O55222	426	LMK(*)IC(+57.02)MNEDPAK(*)	K3	89.22	161.68	0.8709677	0.972602	0.988764	0.9441115
Ilk	O55222	184	NGTLNK(*)HSGIDFK(*)	K6	37.69	104.64	2.9139784	-	0.674157	1.7940679
Ilk	O55222	209	LNENHSGELWK(*)GR(*)	K11	16.32	1000	1.8494623	-	-	
Aurkb	O70126	115	ILFK(*)SQIEK(*)	K4	25.46	51.34	0.8333333	-	1.177777	1.0055556

Aurkb	O70126	314	LLK(*)HNPWQR(*)	K3	23.31	1000	-	-	1.2	
Dhx9	O70133	1026	NALIHK(*)SSVNC(+57.02)PFSSQDM	K6	91.74	251.71	0.77	0.785714	0.927835(	0.8278498
Dhx9	O70133	469	GEEPGK(*)SC(+57.02)GYSVR(*)	K6	40.3	1000	1.15	-	-	
Dhx9	O70133	757	LFTAHNNMTNYATVWASK(*)TNLEQF	K18	28.46	1000	0.36	-	0.721649	0.5408247
Dhx9	O70133	757	ATVWASK(*)TNLEQR(*)	K7	16.67	1000	1.57	0.797619	1.412371	1.2599967
Eif3d	O70194	426	GAVIATELK(*)NNSYK(*)	K9	61.09	98.37	0.78	0.888888	0.712765	0.7938849
Eif3d	O70194	41	LGK(*)VADWTGATYQDK(*)	K3	49.82	264.82	0.8	0.444444	0.680851(	0.6417652
Eif3d	O70194	90	TQK(*)TAYQR(*)	K3	2.98	1000	-	-	1.734042553	
Znf143	O70230	316	TSGDLQK(*)HIR(*)	K7	30.7	1000	-	-	-	
Eef1b	O70251	60	WYNHIK(*)SYEK(*)	K6	84.26	97.69	0.9607843	1.025974	1.147727	1.0448285
Eef1b	O70251	139	K(*)PAVVAK(*)SSILLDVK(*)PWDDET	K7	65.55	41.57	-	-	0.409090909	
Epb41l2	O70318	626	AQEAILK(*)HQASISELK(*)	K7	73.83	99.51	1.0449438	-	0.919540	0.982242
Epb41l2	O70318	479	LWK(*)VC(+57.02)VEHHTFYR(*)	K3	22.82	1000	-	-	1.264367816	
Epb41l2	O70318	626	AQEAILK(*)HQASISELK(*)R(*)	K7	14.91	54.49	-	-	1.574712644	
Epb41l2	O70318	151	PALK(*)SSVETQPAEEVR(*)	K4	7.05	1000	0.8539325	-	-	
Pdlim1	O70400	307	GHFFVEDQIYC(+57.02)EK(*)HAR(*)	K13	97.23	1000	1.0652173	1.052631	1.034090	1.0506466
Pdlim1	O70400	294	QK(*)GHFFVEDQIYC(+57.02)EK(*)	K2	74.98	187.28	0.9130434	0.973684	1.034090	0.9736062
Pdlim1	O70400	244	APVTK(*)VAASVGNAQK(*)	K5	65.42	219.4	0.8695652	0.736842	1.409090	1.0051661
Pdlim1	O70400	71	IK(*)GC(+57.02)ADNMTLTVSR(*)	K2	53.24	1000	0.6739130	0.710526	0.659090	0.6811768
Vamp8	O70404	47	NK(*)TEDLEATSEHFK(*)	K2	64.46	240.79	1.0101010	0.75	0.845454	0.8685185
Fhl2	O70433	183	EQPWHK(*)EC(+57.02)FVC(+57.02)	K6	71.74	159.26	0.7373737	0.670886	1.057471	0.8219104
Fhl2	O70433	118	MEYK(*)GSSWHETC(+57.02)FTC(+5	K4	45.56	1000	-	0.683544	-	
Fhl2	O70433	139	C(+57.02)QQPIGTK(*)SFIPK(*)	K8	19.14	51	-	2.177215	-	
Psma3	O70435	110	SNFGYNIPLK(*)HLADR(*)	K10	88.18	1000	1.12	0.987654	1.031914	1.0465231
Psma3	O70435	110	NIPLK(*)HLADR(*)	K5	75.74	1000	1.16	1.049382	1.127659	1.1123474
Psma3	O70435	57	LVLSK(*)LYEEGSNK(*)	K5	57.04	88.19	0.9	-	0.872340	0.8861702
Ugdh	O70475	100	TYGMGK(*)GR(*)	K6	83.55	1000	0.9684210	1	0.988888	0.98577
Ugdh	O70475	67	GK(*)NLFFSTNIDDAIR(*)	K2	69.61	1000	-	-	0.877777778	
Ugdh	O70475	434	MLK(*)PAFIFDGR(*)	K3	69.28	1000	0.8210526	-	0.722222	0.7716374
Ugdh	O70475	370	YLMDEGAHLHIYDPK(*)VPR(*)	K15	26.64	1000	0.6842105	-	-	
Ugdh	O70475	129	IVTEK(*)STVPVR(*)	K5	16.13	1000	-	-	0.422222222	
Ubr1	O70481	1380	TTC(+57.02)PQVLIHK(*)HLAR(*)	K10	54.38	1000	0.2652173	-	0.639455	0.4523366
Snx3	O70492	128	K(*)QGLEQFINK(*)VAGHPLAQNER(*	K10	70.55	23.02	-	-	0.828571429	
Snx3	O70492	54	VK(*)TNLPIFK(*)	K2	54.2	170.44	0.8526315	0.864197	0.723809	0.8135462

Snx3	O70492	128	QGLEQFINK(*)VAGHPLAQNER(*)	K9	42.38	1000	1.2	-	1.066666(	1.1333333
Sp3	O70494	636	VYGK(*)TSHLR(*)	K4	76.29	1000	-	0.505154	0.770642:	0.6378984
Sp3	O70494	700	SDHLAK(*)HIK(*)	K6	14.58	68.47	1.8208955	-	0.871559(	1.3462276
Hsd17b12	O70503	261	SAIK(*)TVGLQTR(*)	K4	26.65	1000	-	-	0.797468354	
Hsd17b12	O70503	167	LININVLSVC(+57.02)K(*)VTR(*)	K11	5.73	1000	-	-	1.64556962	
Kdm6a	O70546	799	ANNNVGPGTC(+57.02)DK(*)VNNIH	K12	71.93	49.96	1.4838709	0.792207	0.489898	0.9219926
Srpk1	O70551	112	VVK(*)SAEHYTETALDEIR(*)	K3	63.04	1000	0.7884615	1.561643	0.894736	1.0816141
Srpk1	O70551	499	IADLGNAC(+57.02)WVHK(*)HFTEDI	K12	45.05	1000	0.9230769	0.876712	1.147368	0.9823859
Cfdp1	O88271	163	ITK(*)VFDFAGEEVR(*)	K3	63.12	1000	1.3673469	0.810526	1.306818	1.1615638
Wdr1	O88342	321	GHSK(*)SIQC(+57.02)LTVHR(*)	K4	81.54	1000	0.9489795	0.848101	0.914893	0.9039915
Wdr1	O88342	147	NK(*)VINSVDIK(*)	K2	79.74	171.6	0.9897959	0.860759	0.914893	0.9218163
Wdr1	O88342	569	VK(*)IQDAHR(*)	K2	53.96	1000	0.9387755	-	-	
Wdr1	O88342	182	FK(*)FTIGDHSR(*)	K2	39.14	1000	1.0408163	-	1.276595	1.158706
Wdr1	O88342	38	GDHFLYTNGK(*)C(+57.02)VILR(*)	K10	36.12	1000	-	-	0.872340426	
Wdr1	O88342	106	IK(*)DIAWTEDSK(*)	K2	34.63	76.18	-	0.797468	-	
Wdr1	O88342	95	EHLLK(*)YEYQPFAGK(*)	K5	34.43	100.79	-	-	0.468085106	
Grpel2	O88396	159	SVFTK(*)HGLEK(*)	K5	29.52	29.85	-	-	0.880597015	
Klc1	O88447	68	K(*)DDESNLVEEK(*)SSMIR(*)	K11	63.67	92.69	-	0.763157	1	0.8815789
Klc1	O88447	383	TK(*)NNLASC(+57.02)YLK(*)	K2	61.03	135.34	-	0.539473	1.044943	0.7922088
Klc1	O88447	68	DDESNLVEEK(*)SSMIR(*)	K10	51.8	1000	-	-	0.93258427	
Capns1	O88456	136	HPDLK(*)TDGFGIDTC(+57.02)R(*)	K5	58.8	1000	1.0408163	1.025974	0.8	0.9555968
Capns1	O88456	180	WQAIYK(*)R(*)	K6	13.48	1000	-	0.948051	-	
Igf2bp1	O88477	228	QTQSK(*)IDVHR(*)	K5	28.19	1000	-	-	21.03529412	
Zfr	O88532	516	LQSTGNK(*)TEDLK(*)	K7	85.49	89.05	1.2619047	0.736842	1.011627	1.0034583
Zfr	O88532	509	INFVGGNK(*)LQSTGNK(*)	K8	67.77	118.11	1.4404761	0.763157	1.046511	1.0833819
Zfr	O88532	910	HAK(*)WFQAR(*)	K3	52.6	1000	0.8928571	-	0.918604	0.9057309
Zfr	O88532	730	YVMTK(*)HATIYPTEEELQAVQK(*)	K5	44.82	212.46	-	-	0.941860465	
Cops3	O88543	281	NLVSK(*)HSETFTR(*)	K5	68.27	1000	0.9795918	0.938271	0.935483	0.9511158
Cops3	O88543	125	GIGILK(*)QAIDK(*)	K6	11.51	105.53	3.3469387	-	-	
Cops4	O88544	227	LEALK(*)HALHC(+57.02)TILASAGQC	K5	73.6	1000	0.9583333	0.870129	1.010989	0.9464841
Cops4	O88544	150	LETYLK(*)IAR(*)	K6	37.47	1000	-	-	1.021978022	
Cops4	O88544	214	YNELSYK(*)TIVHESER(*)	K7	29.57	1000	-	1.519480	0.758241	1.1388611
Cops6	O88545	105	EYYYTK(*)EEQFK(*)	K6	40.01	61.3	-	-	0.950980392	
Hnrnpa2b1	O88569	104	EESGK(*)PGAHVTVK(*)	K5	34.31	113.97	-	-	0.862068966	

Pak1	O88643	114	LLQTSNITK(*)SEQK(*)	K9	38.46	22.37	-	1.256097	0.715789	0.9859435
Pak1	O88643	391	DIK(*)SDNILLGMDGSVK(*)	K3	4.7	84.54	-	-	4.726315789	
Psmc3	O88685	248	AC(+57.02)AAQTK(*)ATFLK(*)	K7	78.77	107.29	0.7551020	0.829268	0.586956	0.7237756
Psmc3	O88685	236	GVLMYGPPGTGK(*)TLLAR(*)	K12	77.53	1000	0.6734693	0.682926	1.076086	0.8108277
Psmc3	O88685	59	IMK(*)SEVLR(*)	K3	66.35	1000	0.8265306	1.256097	1.152173	1.0782674
Psmc3	O88685	149	LK(*)PGDLVGVNK(*)	K2	55.44	85.31	-	-	0.77173913	
Clpp	O88696	210	HTK(*)QSLQVIESAMER(*)	K3	15.57	1000	-	1.350649	-	
Orc4	O88708	7	TK(*)SNAHAEC(+57.02)LSQVQR(*)	K2	26.81	1000	3.3958333	-	-	
Ctbp1	O88712	348	IPDSLK(*)NC(+57.02)VNK(*)	K6	72.9	81.69	0.9032258	0.717948	0.913978	0.845051
Birc6	O88738	4044	VITDPSLSK(*)TDSFK(*)	K9	16.4	26.93	-	0.876543	-	
Tom1	O88746	106	TILPK(*)NNPPTIVHDK(*)	K5	3.96	25.89	-	-	1.138297872	
Sc5d	O88822	70	HPQFLK(*)NQVSR(*)	K6	20.82	1000	-	-	-	
Idh1	O88844	115	EAIIC(+57.02)K(*)NIPR(*)	K6	87.99	1000	0.7978723	0.675	0.966666	0.8131797
Idh1	O88844	203	SK(*)GWPLYLSTK(*)	K2	74.32	140.79	0.6382978	0.4375	0.744444	0.6067474
Idh1	O88844	212	GWPLYLSTK(*)NTILK(*)	K9	54.11	43.82	0.9042553	1.6375	1.088888	1.2102147
Idh1	O88844	224	FK(*)DIFQEIYDK(*)	K2	45.45	189.7	1.0638297	1.075	-	1.0694149
Arl6	O88848	119	EELDTLLNHPDIK(*)HR(*)	K13	49.64	1000	1.6567164	0.602040	0.96875	1.0758357
Zfand5	O88878	183	YSDK(*)HNC(+57.02)PYDYK(*)	K4	20.67	81.34	-	-	1.644736842	
Apaf1	O88879	637	IASC(+57.02)GADK(*)TLQVFK(*)	K8	26.44	25.65	-	-	0.675	
Yme1l1	O88967	327	GILLVGPPGTGK(*)TLLAR(*)	K12	40.53	1000	-	-	0.686868687	
Cpd	O89001	530	LYSLGK(*)SVESR(*)	K6	28.29	1000	0.4545454	2.909090	-	1.6818182
Lgmn	O89017	289	VMQFQGMK(*)HR(*)	K8	25.67	1000	1.1022727	-	0.581395	0.841834
Mkln1	O89050	182	LC(+57.02)LK(*)HFR(*)	K4	9.18	1000	-	0.929411	-	
Casp8	O89110	309	NK(*)DC(+57.02)FIC(+57.02)C(+57.	K2	47.85	121.36	1.0769230	-	0.853932	0.9654278
Vti1a	O89116	176	DADANLGK(*)SSR(*)	K8	15.08	1000	0.9236641	-	1.164948	1.0443063
Dhfr	P00375	92	GAHFLAK(*)SLDDALR(*)	K7	84.73	1000	1.3222222	1.197368	1.043956	1.1878489
Dhfr	P00375	92	FLAK(*)SLDDALR(*)	K4	42.05	1000	-	-	0.901098901	
Hprt1	P00493	103	LK(*)SYC(+57.02)NDQSTGDIK(*)	K2	56.1	240.84	1.1619047	1.142857	1.340425	1.2150625
Mtatp6	P00848	64	QMMLIHTPK(*)GR(*)	K9	52.74	1000	1.1666666	0.769230	1.141304	1.0257339
B2m	P01887	103	VK(*)HASMAEPK(*)	K2	19.43	171.6	0.8207547	-	0.946808	0.8837816
Hba	P01942	17	AAWGK(*)IGGHGAEYGAEALER(*)	K5	21.01	1000	1	0.416666	0.375	0.5972222
Lamc1	P02468	306	LMC(+57.02)NC(+57.02)K(*)HNTYG	K6	38.68	58.64	0.9137931	-	1.054945	0.9843691
Lamb1	P02469	1736	SK(*)LQLLEDLER(*)	K2	49.81	1000	-	-	0.77173913	
Lamb1	P02469	1708	VESLIAQK(*)TEESADAR(*)	K8	36.75	1000	1.1368421	-	0.771739	0.9542906

Lamb1	P02469	158	SSDFGK(*)AWGVYR(*)	K6	9.65	1000	-	-	1.130434783	
Mtatp8	P03930	48	VK(*)TPWELK(*)	K2	31.49	95.35	-	-	-	
Mtatp8	P03930	54	TPWELK(*)WTK(*)	K6	23.75	26.52	-	-	-	
Aldoa	P05064	147	DGADFAK(*)WR(*)	K7	87.8	1000	-	0.786666	0.988636:	0.8876515
Aldoa	P05064	108	GGVVGIK(*)VDK(*)	K7	76.97	53.53	0.9468085	0.72	1.0113636	0.892724
Aldoa	P05064	101	SK(*)GGVVGIK(*)	K2	71.53	122.76	0.9468085	0.92	0.954545	0.9404513
Aldoa	P05064	230	ALSDHHVYLEGTLLK(*)PNM(+15.99)	K15	54.06	49.75	0.3617021	-	0.965909(	0.6638056
Aldoa	P05064	294	LK(*)PWALTFSYGR(*)	K2	45.7	1000	-	-	0.977272727	
Aldoa	P05064	153	C(+57.02)VLK(*)IGEHTPSALAIMENA	K4	21.33	1000	-	-	0.920454545	
Prkaca	P05132	48	IK(*)TLGTGSFGR(*)	K2	79.33	1000	0.8404255	-	0.913043	0.8767345
Prkaca	P05132	62	VMLVK(*)HK(*)	K5	63.93	24.32	1.3085106	0.636363	0.902173	0.9490161
Prkaca	P05132	24	AK(*)EDFLK(*)	K2	53.06	142.99	-	0.519480	0.902173	0.7108272
Prkaca	P05132	286	FGNLK(*)NGVNDIK(*)	K5	17.36	110.43	-	-	1.434782609	
Got1	P05201	259	SK(*)NFGLYNER(*)	K2	74.48	1000	-	0.794871	-	
Got1	P05201	396	INMC(+57.02)GLTTK(*)NLDYVATSIH	K9	69.59	60.3	-	-	1.068181818	
Got1	P05201	378	QVEYLVNEK(*)HIYLLPSGR(*)	K9	49.09	1000	-	1.410256	0.897727	1.1539918
Got1	P05201	325	GNVK(*)TMADR(*)	K4	16.1	1000	-	-	1.409090909	
Got2	P05202	122	ASAELALGENNEVLK(*)SGR(*)	K15	86.68	1000	0.8163265	0.621951	0.933333	0.790537
Got2	P05202	90	AEAQIAAK(*)NLDK(*)	K8	70.11	38.26	-	0.487804	0.711111	0.599458
Got2	P05202	159	DVFLPK(*)PSWGNHTPIFR(*)	K6	45.93	1000	0.8469387	0.902439	0.9	0.8831259
Got2	P05202	279	AK(*)NMGLYGER(*)	K2	45.13	1000	0.8367346	0.792682	1.455555	1.0283244
Got2	P05202	345	QWLQEVK(*)GMADR(*)	K7	44.83	1000	0.9387755	0.463414	0.666666(	0.6896189
Got2	P05202	90	K(*)AEAQIAAK(*)NLDK(*)	K9	40.16	29.57	0.5510204	0.817073	0.433333	0.6004756
Got2	P05202	345	K(*)QWLQEVK(*)GMADR(*)	K8	38.26	104.64	0.9693877	-	0.844444	0.9069161
Got2	P05202	309	VESQLK(*)ILIR(*)	K6	36.76	1000	0.5510204	-	0.666666(	0.6088435
Got2	P05202	387	C(+57.02)FTGLK(*)PEQVER(*)	K6	7.04	1000	-	-	1.477777778	
Src	P05480	208	GLNVK(*)HYK(*)	K5	41.04	68.47	0.8421052	0.987179	0.885057	0.9047807
Pdgfrb	P05622	190	TYIC(+57.02)K(*)TTIGDR(*)	K5	37.7	1000	-	-	1.304878049	
Pdgfrb	P05622	696	NK(*)HTFLQR(*)	K2	27.94	1000	1.0125	-	-	
Pdgfrb	P05622	840	LVK(*)IC(+57.02)DFGLAR(*)	K3	26.64	1000	-	-	0.914634146	
Jun	P05627	56	AK(*)NSDLLTSPDVGLLK(*)	K2	38.23	227.68	-	-	2.555555556	
Ldha	P06151	155	ISGFPK(*)NR(*)	K6	99.12	1000	0.8829787	0.822784	0.944444	0.8834027
Ldha	P06151	59	LK(*)GEMMDLQHGSLFLK(*)	K2	84.42	243.73	0.9893617	0.898734	0.988888	0.9589949
Ldha	P06151	90	SK(*)LVIITAGAR(*)	K2	84.2	1000	0.8510638	0.860759	0.911111	0.8743115

Ldha	P06151	232	EVHK(*)QVVDSAYEVIK(*)	K4	82.12	215.31	1.0851063	0.759493	0.966666	0.9370889
Ldha	P06151	73	GEMMDLQHGSLFLK(*)TPK(*)	K14	77.22	32.97	0.9787234	0.721518	0.977777	0.8926734
Ldha	P06151	149	VAWK(*)ISGFPK(*)	K4	41.98	90.32	0.8723404	0.924050	0.888888	0.8950933
Gpi	P06745	142	SGDWK(*)GYTGK(*)	K5	85.97	95.62	1	0.987654	0.901098	0.9629177
Gpi	P06745	57	ILVDYSK(*)NLVNK(*)	K7	78.15	100.21	1.0425531	-	0.857142	0.949848
Gpi	P06745	130	MK(*)SFC(+57.02)QR(*)	K2	68.77	1000	0.8936170	0.654320	0.945054	0.8309977
Gpi	P06745	89	DNMFSGSK(*)INYTENR(*)	K8	68.15	1000	0.8829787	0.876543	1.340659	1.0333938
Gpi	P06745	241	DPSAVAK(*)HFVALSTNTAK(*)	K7	61.16	139.71	0.8829787	0.691358	0.901098	0.8251452
Gpi	P06745	73	EVMQMLVELAK(*)SR(*)	K11	53.83	1000	0.8723404	-	1.043956	0.9581482
Gpi	P06745	241	SAVAK(*)HFVALSTNTAK(*)	K5	44.89	176.98	0.6702127	0.592592	1.087912	0.7835725
Gpi	P06745	423	GLHHK(*)ILLANFLAQTEALMK(*)	K5	37.68	278.79	-	-	0.879120879	
Ctsl	P06797	54	AIWEK(*)NMR(*)	K5	57.1	1000	-	-	1.451612903	
Ctsl	P06797	116	IPK(*)SVDWR(*)	K3	53.22	1000	0.6944444	-	-	
Ctsl	P06797	33	FDQTFSAEWHQWK(*)STHR(*)	K13	49.51	1000	0.8796296	-	1.075268	0.9774492
Ctsl	P06797	154	SASGC(+57.02)LEGQMFLK(*)TGK(*)	K13	48.66	32.97	-	-	0.989247312	
Me1	P06801	60	IIK(*)NFER(*)	K3	56.97	1000	-	1.085365	0.869565	0.9774655
Me1	P06801	362	EVFAHEHEEMK(*)NLEAIVQK(*)	K11	51.57	139.69	0.9306930	-	0.978260	0.954477
Me1	P06801	337	IWLVDSK(*)GLIVK(*)	K7	46.24	94.22	0.9900990	-	0.847826	0.9189625
Me1	P06801	422	AEC(+57.02)SAEQC(+57.02)YK(*)VT	K10	39.09	44.44	-	1.207317	0.858695	1.0330064
S100a4	P07091	48	ELPSFLGK(*)R(*)	K8	73.46	1000	-	0.759493	0.919540	0.839517
S100a4	P07091	35	LNK(*)TELK(*)ELLTR(*)	K7	34.99	1000	1.1195652	0.683544	1.080459	0.9611898
S100a4	P07091	31	LNK(*)TELK(*)ELLTR(*)	K3	34.99	1000	1.1195652	0.683544	1.080459	0.9611898
Sparc	P07214	128	TFDSSC(+57.02)HFFATK(*)C(+57.02	K12	43.69	50.34	1.0752688	-	0.831460	0.9533647
Ttr	P07309	96	VELDTK(*)SYWK(*)	K6	11.82	77.13	-	-	-	
Ttr	P07309	35	C(+57.02)PLMVK(*)VLDAVR(*)	K6	2.24	1000	-	-	-	
Anxa2	P07356	233	YK(*)SYSPYDMLESIK(*)	K2	93.65	307.89	0.8969072	0.822784	0.820224	0.8466389
Anxa2	P07356	313	YGK(*)SLYYYIQQDTK(*)	K3	90.08	267.5	0.9278350	0.974683	1.011235	0.9712515
Anxa2	P07356	47	DALNIETAVK(*)TK(*)	K10	76.03	28.86	0.8247422	0.822784	0.910112	0.8525465
Anxa2	P07356	152	EMYK(*)TDLEK(*)	K4	74.24	87.95	0.9278350	0.632911	0.876404	0.8123836
Anxa2	P07356	176	LMVALAK(*)GR(*)	K7	71.72	1000	0.7938144	0.898734	0.955056	0.8825349
Anxa2	P07356	227	SVC(+57.02)HLQK(*)VFER(*)	K7	71.3	1000	0.9072164	0.810126	0.977528	0.8982904
Anxa2	P07356	302	SEVDMLK(*)IR(*)	K7	56.3	1000	0.9278350	0.822784	-	0.8753099
Anxa2	P07356	49	TK(*)GVDEVTIVNILTNR(*)	K2	28.94	1000	0.8762886	-	0.808988	0.8426387
Anxa2	P07356	324	SLYYYIQQDTK(*)GDYQK(*)	K11	26.16	47.64	1.5051546	-	0.808988	1.1570717

Anxa2	P07356	266	NK(*)PLYFADR(*)	K2	24.05	1000	-	-	1.247191011	
Anxa2	P07356	152	EMYK(*)TDLEK(*)DIISDTSGDFR(*)	K4	20.51	30.64	-	-	1.033707865	
Anxa2	P07356	28	LSLEGDHSTPPSAYGSVK(*)PYTNFDA	K18	9.95	1000	-	-	1.314606742	
Rrm1	P07742	17	VMFDK(*)ITSR(*)	K5	66.76	1000	0.7765957	0.658227	0.941860	0.792228
Rrm1	P07742	733	QGLK(*)TGMYYLR(*)	K4	43.72	1000	1.2978723	1.848101	1.313953	1.4866424
Rrm1	P07742	149	DFSYNYFGFK(*)TLER(*)	K10	40.22	1000	-	-	1.255813953	
Rrm1	P07742	243	QC(+57.02)ALISK(*)SAGGIGVAVSC(	K7	13.86	1000	-	-	1.395348837	
Hsp90aa1	P07901	41	NK(*)EIFLR(*)	K2	98.48	1000	0.9578947	0.910256	0.955056	0.9410691
Hsp90aa1	P07901	540	EFEGK(*)TLVSVTK(*)	K5	96.14	147.63	0.9263157	0.846153	0.943820	0.90543
Hsp90aa1	P07901	293	YIDQEELNK(*)TK(*)	K9	92.63	36.05	0.8105263	0.820512	0.955056	0.8620318
Hsp90aa1	P07901	112	ADLINNLGTIAK(*)SGTK(*)	K12	89.97	63.97	0.8736842	0.820512	0.977528	0.890575
Hsp90aa1	P07901	444	FYEQFSK(*)NIK(*)	K7	88.99	37.54	0.9263157	0.910256	0.887640	0.9080709
Hsp90aa1	P07901	490	ENQK(*)HIYFITGETK(*)	K4	88	116.87	0.9052631	0.807692	1	0.9043185
Hsp90aa1	P07901	408	EMLQQSK(*)ILK(*)	K7	87.19	50.22	0.9894736	0.820512	0.921348	0.9104449
Hsp90aa1	P07901	185	GTK(*)VILHLK(*)	K3	83.89	156.85	0.8947368	0.833333	1.988764	1.2389447
Hsp90aa1	P07901	568	TK(*)FENLC(+57.02)K(*)	K2	81.99	156.85	1.2210526	1.076923	1.033707	1.1105612
Hsp90aa1	P07901	100	TK(*)ADLINNLGTIAK(*)	K2	81.74	285.65	0.8315789	0.974358	0.932584	0.9128407
Hsp90aa1	P07901	295	TK(*)PIWTR(*)	K2	81.7	1000	0.8315789	0.833333	0.932584	0.8658322
Hsp90aa1	P07901	84	ELHINLIPSK(*)QDR(*)	K10	78.8	1000	0.8947368	0.858974	0.898876	0.8841959
Hsp90aa1	P07901	500	HIYFITGETK(*)DQVANSAFVER(*)	K10	69.48	1000	1.2947368	-	1.022471	1.1586044
Hsp90aa1	P07901	284	EK(*)YIDQEELNK(*)	K2	66.78	148.12	-	1.128205	1.011235	1.0697205
Hsp90aa1	P07901	153	VTVITK(*)HNDDEQYAWESSAGGSFT	K6	66.22	1000	0.6210526	-	1.179775	0.900414
Hsp90aa1	P07901	490	MK(*)ENQK(*)HIYFITGETK(*)	K6	66.21	52.86	1.4842105	0.782051	1.415730	1.2273307
Hsp90aa1	P07901	69	YESLTDPSK(*)LDSGK(*)	K9	57.89	32.32	1.0631578	0.679487	1.056179	0.9329416
Hsp90aa1	P07901	191	VILHLK(*)EDQTEYLEER(*)	K6	46.23	1000	-	2.038461	0.977528	1.5079948
Hsp90aa1	P07901	293	YIDQEELNK(*)TK(*)PIWTR(*)	K9	45.89	22.85	0.7473684	0.794871	0.685393	0.7425445
Hsp90aa1	P07901	191	LK(*)EDQTEYLEER(*)	K2	41.83	1000	1.0526315	1.012820	1.651685	1.2390458
Pdia4	P08003	526	VVVGK(*)TFDAIVMDPK(*)	K5	92.77	228.69	0.8247422	0.740740	0.956989	0.8408241
Pdia4	P08003	430	NK(*)VLEVAK(*)	K2	73.56	129.19	0.9381443	0.962962	0.989247	0.9634515
Pdia4	P08003	249	FDVSGYPTLK(*)IFR(*)	K10	70.8	1000	0.9072164	0.740740	0.903225	0.8503943
Pdia4	P08003	384	DYVVK(*)HALPLVGHR(*)	K5	60.23	1000	0.9381443	0.777777	0.956989	0.8909705
Pdia4	P08003	342	FLK(*)VSLGK(*)	K3	59.56	76.42	0.8762886	-	1.118279	0.9972841
Pdia4	P08003	227	AK(*)VDATEQTDLAK(*)	K2	43.52	219.15	1.1855670	-	1.107526	1.1465469
Pdia4	P08003	156	TQEEIVAK(*)VR(*)	K8	27.17	1000	1.0824742	0.728395	0.892473	0.9011141

Pdia4	P08003	249	R(*)FDVSGYPTLK(*)IFR(*)	K11	13.19	1000	0.8350515	-	0.881720	0.858386
Pdia4	P08003	508	LK(*)PVIK(*)SQPVPK(*)	K6	10.36	28.13	-	-	1.096774194	
Aprt	P08030	51	LLASHLK(*)STHSGK(*)	K7	83.18	123.86	1.0096153	0.815217	0.956989	0.927274
Aprt	P08030	57	STHSGK(*)IDYIAGLDSR(*)	K6	71.44	1000	0.9903846	0.836956	1.086021	0.9711209
Hsp90b1	P08113	95	LIINSLYK(*)NK(*)	K8	98.21	28.86	0.9183673	0.9125	0.904255	0.9117076
Hsp90b1	P08113	168	NLGTIAK(*)SGTSEFLNK(*)	K7	97.81	202.16	0.8673469	0.8125	0.882978	0.8542752
Hsp90b1	P08113	161	EELVK(*)NLGTIAK(*)	K5	97.23	136.94	0.8571428	0.8125	0.936170	0.8686044
Hsp90b1	P08113	142	EK(*)NLLHVTDTGVGMTR(*)	K2	93.83	1000	0.7857142	0.95	0.957446	0.8977204
Hsp90b1	P08113	356	EVEEDEYK(*)AFYK(*)	K8	88.45	77.13	1	0.875	0.946808	0.9406028
Hsp90b1	P08113	455	ETLQQHK(*)LLK(*)	K7	88.28	62.26	0.9285714	0.875	0.957446	0.9203394
Hsp90b1	P08113	214	VIVTSK(*)HNNDTQHIWESDSNEFSVI	K6	64.13	1000	1.0918367	-	1.042553	1.067195
Hsp90b1	P08113	671	AQAYQTGK(*)DISTNYYASQK(*)	K8	35.17	134.4	-	-	0.787234043	
Hsp90b1	P08113	733	SGYLLPDTK(*)AYGDR(*)	K9	32.07	1000	0.9489795	1.05	0.765957	0.9216457
Hsp90b1	P08113	360	AFYK(*)SFSK(*)	K4	13.42	49.79	-	-	1.14893617	
Col3a1	P08121	1424	HTGEWSK(*)TVFEYQTR(*)	K7	35.86	1000	-	-	0.837606838	
S100a10	P08207	28	FAGDK(*)DHLTK(*)EDLR(*)	K10	71.13	115.97	0.8834951	0.956521	1.043956	0.9613243
Mdh2	P08249	301	GLEK(*)NLGIGK(*)	K4	94.11	151.71	0.9021739	0.839506	0.849462	0.8637142
Mdh2	P08249	335	K(*)GEDFVK(*)NMK(*)	K7	84.74	56.34	0.9130434	0.728395	0.870967	0.8374688
Mdh2	P08249	45	SLLLK(*)NSPLVSR(*)	K5	79.71	1000	0.8695652	0.839506	0.913978	0.87435
Mdh2	P08249	185	ANTFVAELK(*)GLDPAR(*)	K9	72.88	1000	1.0434782	-	-	
Mdh2	P08249	241	AK(*)AGAGSATLSMAYAGAR(*)	K2	56.21	1000	0.5760869	0.716049	0.763440	0.6851924
Mdh2	P08249	301	K(*)GLEK(*)NLGIGK(*)	K5	53.16	49.79	0.9565217	0.703703	0.838709	0.8329784
Mdh2	P08249	78	ANVK(*)GYLGPEQLPDC(+57.02)LK(	K4	43.31	145.42	1.0652173	1.580246	0.806451	1.1506386
Mdh2	P08249	203	VNVPVIGGHAGK(*)TIIPLISQC(+57.0	K12	39.2	71.85	0.6847826	-	1.333333	1.009058
Mdh2	P08249	335	GEDFVK(*)NMK(*)	K6	7.31	24.24	-	-	2.559139785	
Polr2a	P08775	710	AK(*)QDVIEVIEK(*)	K2	66.16	161.68	0.9777777	0.75	0.913978	0.8805854
Polr2a	P08775	1115	TFHYAGVSAK(*)NVTLGVPR(*)	K10	43.65	1000	-	-	0.666666667	
Polr2a	P08775	767	SLSEYNNFK(*)SMVVSGAK(*)	K9	32.12	58.11	1.1444444	-	0.860215	1.0023297
Polr2a	P08775	445	PSDLHLQTGYK(*)VER(*)	K11	23.92	1000	-	-	1.129032258	
Polr2a	P08775	619	THSTHPDDEDSGPYK(*)HISPGDTK(*	K15	20.24	78.75	-	-	0.913978495	
Polr2a	P08775	151	GK(*)NIC(+57.02)EGGEEMDNK(*)	K2	3.89	49.63	-	-	1.935483871	
Itgb1	P09055	349	ELK(*)NLIPK(*)	K3	49.37	81.69	0.6702127	0.881578	0.887640	0.8131441
P4hb	P09103	330	YK(*)PESDELTAEK(*)	K2	90.08	112.07	0.7777777	0.886075	0.923913	0.8625889
P4hb	P09103	265	THILLFLPK(*)SVSDYDGK(*)	K9	86.77	97.31	0.8989898	0.696202	0.858695	0.8179627

P4hb	P09103	108	FFK(*)NGDTASPK(*)	K3	76.24	183.32	0.9898989	0.810126	0.847826(	0.8826172
P4hb	P09103	354	IK(*)PHLMSQEVPEDWDK(*)	K2	71.41	107.94	0.7171717	-	1	0.8585859
P4hb	P09103	273	SVSDYDGK(*)LSSFK(*)	K8	40.68	86.16	1.4141414	-	0.869565.	1.1418533
P4hb	P09103	354	IK(*)PHLMSQEVPEDWDK(*)QPVK(*	K2	31.8	75.66	0.1919191	1.240506	0.978260	0.8035621
P4hb	P09103	265	ILLFLPK(*)SVSDYDGK(*)	K7	26.05	71.07	1.1818181	-	0.793478.	0.9876482
P4hb	P09103	197	SGVFSK(*)YQLDK(*)	K6	18.24	65.44	1	-	0.826086!	0.9130435
Ncl	P09405	478	TGK(*)TSTWSGESK(*)	K3	97.5	208.83	0.8152173	0.814814	0.978021!	0.8693514
Ncl	P09405	431	SK(*)GIAYIEFK(*)	K2	93.29	183.32	0.9565217	0.975308	0.923076!	0.9516358
Ncl	P09405	569	SQPSK(*)TLFVK(*)	K5	93.24	96.85	0.8695652	0.876543	0.923076!	0.8897285
Ncl	P09405	514	ATFIK(*)VPQNPHGK(*)	K5	87.75	175.25	0.8260869	0.814814	0.868131	0.8363445
Ncl	P09405	429	LVSQDGK(*)SK(*)	K7	81.91	30.83	1.1413043	1.185185	1.076923(	1.1344709
Ncl	P09405	9	AGK(*)THGEAK(*)	K3	81.8	112.31	0.9130434	0.765432	0.945054!	0.8745102
Ncl	P09405	546	EALNSC(+57.02)NK(*)MEIEGR(*)	K8	81.08	1000	0.8695652	0.913580	1.164835	0.9826602
Ncl	P09405	469	SVSLYYTGEK(*)GQR(*)	K10	66.21	1000	0.9239130	1.185185	1.604395(	1.2378313
Ncl	P09405	524	PK(*)GYAFIEFASFEDAK(*)	K2	61.91	179.71	-	-	0.802197802	
Ncl	P09405	514	ATFIK(*)VPQNPHGK(*)PK(*)	K5	60.55	51.56	0.7826086	0.765432	0.791208	0.7797499
Ncl	P09405	439	GIAYIEFK(*)SEADAEK(*)	K8	56.29	110.43	0.9673913	0.864197	0.769230	0.8669399
Ncl	P09405	372	ALELTGLK(*)VFGNEIK(*)	K8	39.3	104.64	0.9130434	-	0.538461!	0.7257525
Ncl	P09405	574	VK(*)GLSEDTTEETLK(*)	K2	31.78	151.17	0.3260869	-	0.923076!	0.6245819
Ncl	P09405	320	IGNLNPNK(*)SVNELK(*)	K8	25.98	41.24	1.3586956	-	0.857142	1.1079193
Ncl	P09405	636	EAMEDGEIDGNK(*)VTLDWAK(*)PK(	K12	17.5	39.4	1.8913043	-	1.615384(	1.7533445
Pgk1	P09411	353	GTK(*)SLMDEVVK(*)	K3	98.86	192.66	0.8804347	0.85	0.976744	0.902393
Pgk1	P09411	30	VDFNVPMK(*)NNQITNNQR(*)	K8	88.83	1000	0.9239130	0.8125	0.930232!	0.8888819
Pgk1	P09411	156	ASLSK(*)LGDVYVNDAFGTAHR(*)	K5	88.45	1000	1.1630434	-	1.127906!	1.1454752
Pgk1	P09411	91	SLLGK(*)DVLFLK(*)	K5	83.59	156.85	0.8913043	0.775	0.988372(	0.8848921
Pgk1	P09411	146	AEPAK(*)IDAFR(*)	K5	77.18	1000	0.8804347	0.8375	0.930232!	0.8827224
Pgk1	P09411	220	VADK(*)IQLINNMLDK(*)	K4	69.17	276.46	0.9347826	0.825	0.918604(	0.8927958
Pgk1	P09411	388	WNTEDK(*)VSHVSTGGGASLELLEGK	K6	64.91	373.31	1.0326086	0.9125	1.244186(	1.0630982
Pgk1	P09411	272	DLMSK(*)AEK(*)	K5	59.93	45.7	0.9021739	0.8375	1.058139!	0.9326045
Pgk1	P09411	56	FC(+57.02)LDNGAK(*)SVVLMSHLGI	K8	47.79	1000	1.1521739	0.8875	1	1.0132246
Pgk1	P09411	56	FC(+57.02)LDNGAK(*)SVVLMSHLGI	K8	26.42	130.54	1.0326086	-	0.988372(	1.0104904
Pgk1	P09411	6	S(+42.01)LSNK(*)LTLDK(*)	K5	24.61	105.53	-	-	0.5	
Fth1	P09528	125	LATDK(*)NDPHLC(+57.02)DFIETYYL	K5	50.22	90.47	1.1212121	-	0.831578!	0.9763955
Fth1	P09528	54	NFAK(*)YFLHQSHEER(*)	K4	40.76	1000	-	-	0.842105263	

Hoxc9	P09633	119	HYALK(*)PDAYPGR(*)	K5	33.56	1000	-	-	-	
Sod2	P09671	53	SK(*)HHAAYVNNLNATEEK(*)	K2	38.26	247.16	1.0693069	1.592105	1.244680	1.302031
Sod2	P09671	194	YLQYK(*)NVR(*)PDYLK(*)	K5	11.68	78.75	-	3.026315	2.159574	2.5929451
H2az1	P0C0S6	116	IHK(*)SLIGK(*)	K3	93.5	107.29	0.9479166	0.797297	-	0.872607
H2az1	P0C0S6	8	G(+42.01)K(*)AGK(*)DSGK(*)AK(*)	K5	92.14	48.45	1.03125	1.027027	-	1.0291385
H2az1	P0C0S6	14	AGK(*)DSGK(*)AK(*)TK(*)	K9	71.6	39.76	1.2395833	1.486486	1.284090	1.3367202
H2az1	P0C0S6	12	AGK(*)DSGK(*)AK(*)TK(*)	K7	71.6	97.69	1.2395833	1.486486	1.284090	1.3367202
H2az1	P0C0S6	8	AGK(*)DSGK(*)AK(*)TK(*)	K3	71.6	216.51	1.2395833	1.486486	1.284090	1.3367202
H2az1	P0C0S6	8	AGGK(*)AGK(*)DSGK(*)	K7	64.95	76.73	0.96875	1.148648	1.136363	1.0845874
H2az1	P0C0S6	5	AGGK(*)AGK(*)DSGK(*)	K4	64.95	148.49	0.96875	1.148648	1.136363	1.0845874
H2az1	P0C0S6	12	AGGK(*)AGK(*)DSGK(*)AK(*)	K11	45.16	39.76	0.96875	0.027027	1.761363	0.9190469
H2az1	P0C0S6	8	AGGK(*)AGK(*)DSGK(*)AK(*)	K7	45.16	132.53	0.96875	0.027027	1.761363	0.9190469
H2az1	P0C0S6	5	AGGK(*)AGK(*)DSGK(*)AK(*)	K4	45.16	208.83	0.96875	0.027027	1.761363	0.9190469
H2az1	P0C0S6	12	GGK(*)AGK(*)DSGK(*)AK(*)	K10	27.96	30.83	1.3333333	-	1.272727	1.3030303
H2az1	P0C0S6	8	GGK(*)AGK(*)DSGK(*)AK(*)	K6	27.96	115.89	1.3333333	-	1.272727	1.3030303
H2az1	P0C0S6	5	GGK(*)AGK(*)DSGK(*)AK(*)	K3	27.96	195.94	1.3333333	-	1.272727	1.3030303
H2az1	P0C0S6	12	GK(*)AGK(*)DSGK(*)AK(*)	K9	27.09	30.83	-	1.040540	1.090909	1.0657248
H2az1	P0C0S6	5	GK(*)AGK(*)DSGK(*)AK(*)	K2	27.09	222.06	-	1.040540	1.090909	1.0657248
H2az1	P0C0S6	8	GGK(*)AGK(*)DSGK(*)	K6	9.02	77.13	-	-	1.272727273	
H2az1	P0C0S6	5	GGK(*)AGK(*)DSGK(*)	K3	9.02	163.4	-	-	1.272727273	
Ndufb1	P0DN34	40	NK(*)SMLFQR(*)	K2	8.02	1000	2.8915662	-	-	
Calm1	P0DP26	78	MK(*)DTDSEEEIR(*)	K2	42.65	1000	1.1287128	1.435897	1	1.1882034
Anxa1	P10107	250	YSQHDMNK(*)ALDLELK(*)	K8	88.73	140.21	1.0210526	0.866666	0.977011	0.9549103
Anxa1	P10107	287	LYEAMK(*)GAGTR(*)	K6	88.68	1000	0.8842105	0.906666	0.885057	0.8919782
Anxa1	P10107	185	ALLALAK(*)GDR(*)	K7	86.97	1000	0.8105263	0.84	0.977011	0.8758459
Anxa1	P10107	312	SEIDMNEIK(*)VFYQK(*)	K9	82.73	121.37	0.9368421	0.933333	0.908045	0.9260738
Anxa1	P10107	26	FLENQEQEYVQAVK(*)SYK(*)	K14	73.68	29.32	1.0315789	1	0.988505	1.0066949
Anxa1	P10107	242	VFQNYGK(*)YSQHDMNK(*)	K7	67.05	36.2	-	0.64	1.011494	0.8257471
Anxa1	P10107	29	SYK(*)GGPGSAVSPYPSFNVSSDVAAL	K3	53.39	93.61	0.7789473	1	1.264367	1.0144384
Anxa1	P10107	113	LK(*)TPAQFDADELR(*)	K2	26.28	1000	0.3789473	-	-	
Eef1a1	P10126	395	FLK(*)SGDAAIVDMVPGK(*)	K3	97.85	286.67	0.9042553	0.881578	0.988372	0.9247355
Eef1a1	P10126	395	FLK(*)SGDAAIVDMVPGK(*)PMC(+5	K3	96.22	187.76	-	-	0.686046512	
Eef1a1	P10126	212	MPWFK(*)GWK(*)	K5	88.38	68.47	0.9042553	-	1.209302	1.0567788
Eef1a1	P10126	20	VDSGK(*)STTTGHLIYK(*)	K5	85.48	184.95	0.9787234	1	1.058139	1.0122876

Eef1a1	P10126	5	GK(*)EK(*)THINIVVIGHVDSGK(*)	K4	83.65	238.57	0.5531914	1.105263	0.965116	0.8745236
Eef1a1	P10126	3	GK(*)EK(*)THINIVVIGHVDSGK(*)	K2	83.65	223.59	0.5531914	1.105263	0.965116	0.8745236
Eef1a1	P10126	453	VTK(*)SAQK(*)	K3	83.15	55.21	-	-	0.965116279	
Eef1a1	P10126	5	EK(*)THINIVVIGHVDSGK(*)	K2	74.15	340.36	0.9787234	0.894736	1	0.9578201
Eef1a1	P10126	20	THINIVVIGHVDSGK(*)STTTGHLIYK(*	K15	72.88	198.09	1.2127659	0.973684	1.046511	1.0776539
Eef1a1	P10126	36	STTTGHLIYK(*)C(+57.02)GGIDK(*)R	K16	70.8	1000	0.8936170	0.710526	1.965116	1.1897532
Eef1a1	P10126	30	STTTGHLIYK(*)C(+57.02)GGIDK(*)R	K10	70.8	1000	0.8936170	0.710526	1.965116	1.1897532
Eef1a1	P10126	20	IVVIGHVDSGK(*)STTTGHLIYK(*)	K11	67.84	57.1	0.9787234	0.644736	1.081395	0.9016185
Eef1a1	P10126	255	LPLQDVYK(*)IGGIGTVPVGR(*)	K8	46.57	1000	0.6595744	1.289473	1.186046	1.0450316
Eef1a1	P10126	318	PGDNVGFNVK(*)NVSVK(*)DVR(*)	K15	26.29	1000	-	-	1.174418605	
Eef1a1	P10126	313	PGDNVGFNVK(*)NVSVK(*)DVR(*)	K10	26.29	1000	-	-	1.174418605	
Eef1a1	P10126	408	SGDAAIVDMVPGK(*)PMC(+57.02)V	K13	18.79	1000	-	-	4.604651163	
Alad	P10518	184	AALLK(*)HGLGNR(*)	K5	67.1	1000	0.8703703	0.952941	0.890109	0.9044738
Ctsb	P10605	268	SGVYK(*)HEAGDMMGGHAIR(*)	K5	86.44	1000	1.1521739	0.822784	0.978021	0.9843269
Ctsb	P10605	223	EDK(*)HFGYTSYSVSNSVK(*)	K3	57.1	121.03	-	0.784810	0.879120	0.8319655
Ctsb	P10605	263	SDFLTYK(*)SGVYK(*)	K7	49.39	95.62	0.7065217	1.075949	0.813186	0.8652193
Ctsb	P10605	263	TVFSDFLTYK(*)SGVYK(*)	K10	39.82	71.86	2.0869565	-	1.131868	1.6094123
Txn	P10639	8	LIESK(*)EAFQEALAAAGDK(*)	K5	30.82	183.32	-	1.067567	-	
Gstm1	P10649	124	QK(*)PEFLK(*)	K2	10.69	81.69	-	-	0.905882353	
Tcea1	P10711	252	EHQMAK(*)TGGTQTDLFTC(+57.02)(	K6	90.21	295.03	1.0652173	0.959459	1.010752	1.0118098
Tcea1	P10711	32	ELK(*)NIPMTLELLQSTR(*)	K3	60.65	1000	0.8586956	-	0.903225	0.8809607
Slc3a2	P10852	159	GLVLGPIHK(*)NQK(*)	K9	81.94	57.45	0.7708333	0.769230	0.956521	0.8321953
Slc3a2	P10852	114	WWHK(*)GALYR(*)	K4	49.04	1000	0.8541666	0.897435	1.076086	0.9425632
Slc3a2	P10852	148	SHLEYLSTLK(*)VK(*)	K10	43.87	45.01	1.1770833	-	1.467391	1.3222373
H2bc14	P10854	6	PEPTK(*)SAPAPK(*)	K5	99.85	121.92	-	-	-	
H2bc14	P10854	21	K(*)AVTK(*)AQK(*)	K5	98.3	68.47	-	-	-	
H2bc14	P10854	21	AVTK(*)AQK(*)K(*)	K4	96.71	87.62	-	-	-	
H2bc14	P10854	12	PEPTK(*)SAPAPK(*)K(*)GSK(*)	K11	52.84	38.03	-	-	-	
H2bc14	P10854	6	PEPTK(*)SAPAPK(*)K(*)GSK(*)	K5	52.84	82.84	-	-	-	
H2bc14	P10854	21	GSK(*)K(*)AVTK(*)AQK(*)	K8	29	68.47	-	-	-	
H2bc14	P10854	17	GSK(*)K(*)AVTK(*)AQK(*)	K4	29	186.64	-	-	-	
H2bc14	P10854	16	GSK(*)K(*)AVTK(*)AQK(*)	K3	29	216.51	-	-	-	
H2bc14	P10854	21	SK(*)K(*)AVTK(*)AQK(*)	K7	21.09	77.24	-	-	-	
H2bc14	P10854	17	SK(*)K(*)AVTK(*)AQK(*)	K3	21.09	184.45	-	-	-	

H2bc14	P10854	16	SK(*)K(*)AVTK(*)AQK(*)	K2	21.09	217.23	-	-	-	
H2bc14	P10854	6	PTK(*)SAPAPK(*)	K3	16.71	72.73	-	-	-	
H2bc14	P10854	6	PEPTK(*)SAPAPK(*)K(*)	K5	16.2	93.6	-	-	-	
H2bc14	P10854	21	AVTK(*)AQK(*)	K4	4.85	22.45	-	-	-	
H1-0	P10922	55	YIK(*)SHYK(*)	K3	77.57	109.97	0.9215686	0.963855	1.025974	0.970466
H1-0	P10922	59	SHYK(*)VGENADSQIK(*)	K4	39.17	167.76	1.8333333	0.915662	1.233766	1.3275874
Col1a1	P11087	1419	TVIEYK(*)TTK(*)	K6	95.08	45.7	1.2352941	1.207547	1.083333	1.1753915
Col1a1	P11087	1413	FTYSTLVDGC(+57.02)TSHTGTWGK(	K19	45.84	29.67	-	-	1.071428571	
Col1a1	P11087	1360	C(+57.02)K(*)NSVAYMDQQTGNLK(	K2	39.46	184.14	1.8382352	1.943396	1.202380	1.6613375
Col1a1	P11087	1259	MC(+57.02)HSDWK(*)SGEYWIDPN	K7	39.42	109.2	0.75	-	1.416666	1.0833333
Parp1	P11103	620	LYEEK(*)TGNAWHSK(*)	K5	39.47	150.63	0.9019607	1.341772	0.760416	1.0013832
Rrm2	P11157	279	DEGLHC(+57.02)DFAC(+57.02)LMF	K14	48.2	41.96	0.79	-	1.223684	1.0068421
Fn1	P11276	648	WR(*)PK(*)TSTGR(*)	K4	81.37	1000	0.8444444	-	-	
Fn1	P11276	1970	SYTITGLQPGTDYK(*)IHLYTLNDNAR(	K14	74.91	1000	0.9555555	-	0.883720	0.9196382
Fn1	P11276	1039	GGQPK(*)QYNVGPLASK(*)	K5	73.24	185.69	0.6888888	0.702702	1.244186	0.8785925
Fn1	P11276	554	WK(*)C(+57.02)DPIDQC(+57.02)QD	K2	69.55	1000	1.0222222	0.797297	1.011627	0.9437158
Fn1	P11276	101	GFNC(+57.02)ESK(*)PEPEETC(+57.0	K17	57.49	79.54	-	0.648648	0.569767	0.609208
Fn1	P11276	1669	VTTTPK(*)NGLGPSK(*)	K6	56.51	51.59	-	0.783783	0.744186	0.7639849
Fn1	P11276	324	SVGMQWLK(*)SQGNK(*)	K8	50.51	56.94	0.8222222	3.013513	-	1.9178679
Fn1	P11276	2067	VIALK(*)NNQK(*)	K5	48.57	55.36	1.2666666	-	1.139534	1.2031008
Fn1	P11276	1927	TK(*)TETITGFQVDAIPANGQTPVQR(	K2	47.42	1000	1.4888888	-	1.209302	1.3490956
Fn1	P11276	117	PK(*)DSMIWDC(+57.02)TC(+57.02)	K2	46.07	1000	0.6333333	1.162162	1.011627	0.9357078
Fn1	P11276	2373	MSC(+57.02)TC(+57.02)LGNGK(*)G	K10	36.41	20.17	-	-	0.802325581	
Fn1	P11276	2026	ITGYIIK(*)YEK(*)	K7	31.42	30.72	0.9111111	0.459459	0.593023	0.6545313
Fn1	P11276	58	PGC(+57.02)FDNGK(*)HYQINQQWE	K8	27.32	1000	-	-	0.872093023	
Fn1	P11276	1726	PK(*)GLAFTDVDVDSIK(*)	K2	20.55	148.77	-	-	1.058139535	
Fn1	P11276	2349	WC(+57.02)HDNGVNYK(*)IGEK(*)	K10	15	34.3	1.7555555	-	-	
Fn1	P11276	2353	IGEK(*)WDR(*)	K4	7.88	1000	-	-	0.953488372	
Fn1	P11276	1069	NLQPGSEYTVTLVAVK(*)GNQQSPK(*	K16	6.8	21.15	-	-	1.372093023	
Lamp1	P11438	352	SVQVQAFK(*)VDSDR(*)	K8	51.54	1000	0.8421052	-	-	
Cdk1	P11440	283	MALK(*)HPYFDDLDNQIK(*)	K4	75.69	163.21	1.0111111	0.8125	0.879120	0.9009107
Cdk1	P11440	20	IGEGTYGVVYK(*)GR(*)	K11	68.87	1000	0.7888888	0.975	1.065934	0.9432743
Cdk1	P11440	245	WK(*)PGSLASHVK(*)	K2	66.66	106.14	0.7	0.7125	0.758241	0.7235806
Cdk1	P11440	254	VK(*)NLDENGLDLLSK(*)	K2	49.53	220.79	0.9666666	0.825	-	0.8958333

Hsp90ab1	P11499	531	EFDGK(*)SLVSVTK(*)	K5	95.76	154.98	1.0105263	0.950617	1.067415	1.0095198
Hsp90ab1	P11499	72	ELK(*)IDIIPNPQER(*)	K3	94.27	1000	0.9368421	0.851851	0.921348	0.9033474
Hsp90ab1	P11499	481	ETQK(*)SIYYITGESK(*)	K4	91.78	187.15	0.8421052	0.740740	1.078651	0.8871659
Hsp90ab1	P11499	435	FYEAFSK(*)NLK(*)	K7	82.11	34.94	0.9684210	0.839506	0.730337	0.8460881
Hsp90ab1	P11499	559	AK(*)FENLC(+57.02)K(*)	K2	71.23	151.86	1.4210526	1.111111	1.269662	1.2672756
Hsp90ab1	P11499	148	VVVITK(*)HNDDEQYAWESSAGGSFT	K6	58.23	1000	-	-	1.235955056	
Krt8	P11679	102	EQIK(*)SLNNK(*)FASFIDK(*)	K4	3.45	76.42	-	-	-	
Itga5	P11688	947	VWAK(*)TFLQR(*)	K4	72.14	1000	1.1363636	0.862068	0.944444	0.980959
Gas2	P11862	247	MLHNK(*)HVMVR(*)	K5	30.8	1000	1.2197802	1.041095	1.139534	1.1334703
Tcp1	P11983	317	DLK(*)HVAK(*)	K3	97.11	97.69	0.8	0.772151	0.741935	0.7713625
Tcp1	P11983	153	DC(+57.02)LINAAK(*)TSMSSK(*)	K8	93.69	104.48	0.8105263	0.721518	0.924731	0.8189255
Tcp1	P11983	365	IC(+57.02)DDELILIK(*)NTK(*)	K10	86.69	50.22	0.7684210	0.746835	0.860215	0.7918239
Tcp1	P11983	111	QK(*)IHPTSVISGYR(*)	K2	72.19	1000	0.8947368	0.759493	0.860215	0.8381485
Tcp1	P11983	33	IVK(*)SSFGPVGLDK(*)	K3	71.32	192.46	0.7789473	0.556962	0.935483	0.7571311
Tcp1	P11983	243	IAC(+57.02)LDFSLQK(*)TK(*)	K10	38.03	36.05	0.6	1.202531	0.838709	0.8804138
Tcp1	P11983	494	WIGLDLVHGK(*)PR(*)	K10	21.61	1000	1.4315789	1.506329	0.838709	1.2588726
Tcp1	P11983	33	AAASIANIVK(*)SSFGPVGLDK(*)	K10	19.7	102.98	-	-	1.129032258	
Gusb	P12265	460	PAAYYFK(*)TLITHTK(*)	K7	25.86	72.07	0.5652173	1.185185	0.871794	0.8740658
Gusb	P12265	346	YFQGVNK(*)HEDSDIR(*)	K7	10.25	1000	2.1086956	-	-	
Prkar2a	P12367	240	IIVK(*)NNAK(*)	K4	79.52	34.3	-	-	0.943181818	
Prkar2a	P12367	158	IVK(*)TDEHVIDQGDDGDNFYVIER(*)	K3	24.38	1000	-	-	1.477272727	
Pfkl	P12382	386	IYK(*)LLAHQK(*)	K3	57.86	139.02	0.8829787	0.802469	0.934782	0.8734102
Pfkl	P12382	677	NYGTK(*)LGVK(*)	K5	55.58	46.31	1.0212765	0.839506	1.043478	0.968087
Pfkl	P12382	616	MK(*)TDIQR(*)	K2	52.3	1000	0.8617021	1.160493	0.913043	0.9784131
Cox5a	P12787	58	NK(*)PDIDAWELR(*)	K2	57.61	1000	0.5795454	0.972972	1.121951	0.8914899
Rpl7a	P12970	245	HWGGNVLGPK(*)SVAR(*)	K10	80.21	1000	0.75	0.753246	0.934065	0.8124376
Rpl7a	P12970	37	R(*)PK(*)NFGIGQDIQPK(*)	K3	72.5	215.95	0.61	0.701298	0.604395	0.6385648
Rpl7a	P12970	75	LK(*)VPPAINQFTQALDR(*)	K2	54.17	1000	-	-	0.615384615	
Scd2	P13011	36	FEK(*)SSHHWGADVR(*)PELK(*)	K3	48.14	121.4	1.1298701	1.116666	0.941176	1.0625711
Gsn	P13020	546	PMIIYK(*)GGTSR(*)	K6	94.35	1000	0.9021739	0.802631	0.920454	0.8750867
Gsn	P13020	358	AALK(*)TASDFISK(*)	K4	93.24	175.25	0.9565217	0.763157	0.886363	0.8686811
Gsn	P13020	392	QFFK(*)NWR(*)	K4	91.85	1000	0.9456521	0.921052	1.011363	0.9593561
Gsn	P13020	97	VILK(*)TVQLR(*)	K4	81.14	1000	0.9021739	0.671052	0.920454	0.831227
Gsn	P13020	646	SQHVQVEEGSEPDAFWEALGGK(*)TA	K22	73.22	1000	-	-	0.954545455	

Gsn	P13020	546	IIYK(*)GGTSR(*)	K4	64.78	1000	1.0326086	1.065789	1.113636	1.0706782
Gsn	P13020	175	GGVASGFK(*)HVVPNEVVVQR(*)	K8	57.35	1000	1.5760869	0.921052	0.75	1.0823799
Gsn	P13020	582	AVEVMPK(*)SGALNSNDAFVLK(*)	K7	50.13	150.63	0.8695652	1.460526	0.579545	0.969879
Gsn	P13020	191	LFQVK(*)GR(*)	K5	45.89	1000	1.0434782	1.105263	0.909090	1.0192774
Gsn	P13020	160	SGYFK(*)SGLK(*)	K5	20.4	34.3	-	1	1	1
Gsn	P13020	540	EPAHLMSLFGGK(*)PMIIYK(*)	K12	13.46	90.57	-	-	1.056818182	
Ncam1	P13595	684	SK(*)AAHFVFR(*)	K2	74.75	1000	1.0329670	-	0.873563	0.9532651
Ncam1	P13595	121	LMFK(*)NAPTPQEFK(*)	K4	29.14	130.84	0.8241758	1.514285	-	1.1692308
Ncam1	P13595	539	AEWK(*)SLGEESWHFK(*)	K4	25.53	74.64	-	-	0.954022989	
Dnmt1	P13864	1124	GK(*)HQVSEPK(*)EPEAAIK(*)	K2	65.91	109.53	0.9263157	0.831168	0.936842	0.8981089
Dnmt1	P13864	1124	GK(*)GK(*)HQVSEPK(*)	K4	22.12	178.36	0.9894736	-	-	
Dnmt1	P13864	1122	GK(*)GK(*)HQVSEPK(*)	K2	22.12	198.17	0.9894736	-	-	
Dnmt1	P13864	932	VYC(+57.02)SSITK(*)NGVVYR(*)	K8	22.11	1000	0.5052631	-	-	
Dnmt1	P13864	83	VK(*)SLLNK(*)	K2	6.98	81.69	-	-	1.105263158	
Dnmt1	P13864	1124	GK(*)HQVSEPK(*)	K2	1.6	62.93	-	0.064935	-	
S100a6	P14069	47	ELTIGSK(*)LQDAEIAR(*)	K7	97.37	1000	0.8679245	0.925	0.898989	0.8973048
S100a6	P14069	26	YSGK(*)EGDK(*)HTLSK(*)	K8	19.23	45.16	-	-	1.090909091	
S100a6	P14069	22	YSGK(*)EGDK(*)HTLSK(*)	K4	19.23	100.79	-	-	1.090909091	
S100a6	P14069	26	EGDK(*)HTLSK(*)	K4	15.43	86.16	-	-	1.04040404	
Rpl27a	P14115	94	NK(*)TGVAPIIDVVR(*)	K2	94.13	1000	0.7835051	0.924050	1	0.9025186
Rpl27a	P14115	55	YHPGYFGK(*)VGMR(*)	K8	76.13	1000	1.0515463	1.088607	0.902173	1.0141093
Rpl27a	P14115	47	INFDK(*)YHPGYFGK(*)	K5	73	109.41	1.0309278	0.949367	0.891304	0.9571998
Rps16	P14131	73	VK(*)GGGHVAQIYAIR(*)	K2	84.5	1000	0.8571428	0.976470	0.978723	0.9374456
Rps16	P14131	90	QSISK(*)ALVAYYQK(*)	K5	72.14	115.01	0.8061224	0.729411	1.031914	0.8558164
Rpl7	P14148	149	LNK(*)ASINMLR(*)	K3	92.31	1000	0.8137254	0.771084	0.942528	0.8424462
Rpl7	P14148	62	FALK(*)TLR(*)	K4	91.01	1000	0.9705882	0.975903	-	0.9732459
Rpl7	P14148	224	FK(*)EANNFLWPFK(*)	K2	83.35	248.17	0.4803921	-	0.919540	0.6999662
Rpl7	P14148	126	GINGVSPK(*)VR(*)	K8	71.39	1000	0.7941176	0.939759	1.091954	0.9419436
Rpl7	P14148	170	LK(*)SVNELIYK(*)	K2	52.27	154.74	0.7058823	0.421686	1.206896	0.7781552
Rpl7	P14148	178	SVNELIYK(*)R(*)	K8	29.35	1000	-	-	1	
Rpl7	P14148	51	NFAELK(*)VK(*)	K6	18.29	28.86	1.1372549	0.662650	0.758620	0.8528421
Rpl7	P14148	62	K(*)FALK(*)TLR(*)	K5	12.14	77.51	0.8529411	-	0.896551	0.8747465
Mdh1	P14152	110	IFK(*)SQGTALEK(*)	K3	96.78	144.38	0.9898989	0.973684	1.089887	1.0178236
Mdh1	P14152	164	AK(*)SQIALK(*)	K2	66.75	156.85	1.0202020	0.842105	1.101123	0.9878103

Mdh1	P14152	205	LQGK(*)EVGVYEALK(*)	K4	52.07	60.15	-	0.671052	-	
Mdh1	P14152	179	LGVTADDVK(*)NVIIWGNHSSTQYPD	K9	49.82	126.75	-	-	0.943820225	
Calr	P14211	153	GK(*)NVLINK(*)	K2	97.6	176.18	0.9574468	0.9125	0.988888	0.9529452
Calr	P14211	64	DK(*)GLQTSQDAR(*)	K2	90.38	1000	0.9255319	0.9375	0.888888	0.9173069
Calr	P14211	98	TVK(*)HEQNIDC(+57.02)GGGYVK(*)	K3	85.39	293.5	1.2446808	1.0625	1.033333	1.1135047
Calr	P14211	48	SDFGK(*)FVLSSGK(*)	K5	74.88	122.76	1.0319148	1.0125	0.866666	0.9703605
Calr	P14211	151	VHVIFNYK(*)GK(*)	K8	74.62	28.86	0.9042553	0.9	1.033333	0.9458629
Calr	P14211	98	GQTLVVQFTVK(*)HEQNIDC(+57.02)	K11	67.47	67.58	0.9893617	-	0.844444	0.9169031
Calr	P14211	224	AK(*)IDDPTDSK(*)PEDWDK(*)PEHII	K2	15.69	32.94	-	-	0.922222222	
Srp54	P14576	120	TTTC(+57.02)SK(*)LAYYYQR(*)	K6	49.2	1000	0.8125	0.52	0.619565	0.6506884
Srp54	P14576	249	DK(*)VDVASVIVTK(*)LDGHAK(*)	K12	48.13	62.39	-	-	1.086956522	
Srp54	P14576	448	GGDMSK(*)NVSQSQMAK(*)	K6	17.76	97.31	-	-	1.72826087	
Srp54	P14576	323	LK(*)HGQFTLR(*)	K2	15.3	1000	-	1.186666	1.021739	1.1042029
Srp54	P14576	131	GWK(*)TC(+57.02)LIC(+57.02)ADTI	K3	14.36	1000	1.0416666	1.506666	-	1.2741667
Hspb1	P14602	202	AQIGGPEAGK(*)SEQSGAK(*)	K10	51.62	64.58	1.1666666	-	1.135802	1.1512346
Psmd3	P14685	292	IK(*)AIQLEYSEAR(*)	K2	36.21	1000	0.6702127	-	-	
Psmd3	P14685	400	HNVIK(*)TGVR(*)	K5	4.94	1000	1.5851063	-	1.438202	1.5116543
Lmnb1	P14733	533	VILK(*)NSQGEEVAQR(*)	K4	90.33	1000	0.9578947	0.848101	0.912087	0.906028
Lmnb1	P14733	112	FK(*)AEHDQLLLNYAK(*)	K2	86.95	243.69	1.1052631	0.987341	0.890109	0.9942383
Anxa6	P14824	406	QTFK(*)SHFGR(*)	K4	85.62	1000	1.2061855	1.194805	1.2	1.2003303
Anxa6	P14824	600	NK(*)PLFFADK(*)	K2	82.53	122.76	0.8350515	-	0.822222	0.8286369
Anxa6	P14824	607	NK(*)PLFFADK(*)LYK(*)	K9	81.38	44.44	1.1752577	0.662337	0.855555	0.897717
Anxa6	P14824	63	QEIC(+57.02)QNYK(*)SLYGK(*)	K8	70.17	53.48	-	0.766233	0.711111	0.7386724
Anxa6	P14824	647	YDK(*)SLHQAIEGDTSGDFMK(*)	K3	68.01	171.54	1.1030927	0.818181	-	0.9606373
Anxa6	P14824	3	A(+42.01)K(*)IAQGAMYR(*)	K2	58.88	1000	1.0515463	1.129870	0.966666	1.0493611
Anxa6	P14824	81	YELTGK(*)FER(*)	K6	57.4	1000	0.8659793	0.948051	0.811111	0.8750475
Anxa6	P14824	483	EDYHK(*)SLEDALSSDTSGHFR(*)	K5	47.76	1000	-	0.714285	-	
Anxa6	P14824	483	AINEAYK(*)EDYHK(*)SLEDALSSDTSG	K12	33.27	45.16	1.0412371	-	1.166666	1.1039519
Anxa6	P14824	306	SLYSMIK(*)NDTSGEYK(*)	K7	27.99	150.63	2	1.506493	1.922222	1.8095719
Anxa6	P14824	299	YEK(*)SLYSMIK(*)	K3	25.35	110.43	0.7422680	-	-	
Anxa6	P14824	306	IK(*)NDTSGEYK(*)	K2	22.38	78.17	-	-	1.877777778	
Anxa6	P14824	220	LVFDEYLK(*)TTGK(*)	K8	13.79	31.27	-	-	1.311111111	
Anxa6	P14824	265	AMK(*)GLGTR(*)	K3	10.94	1000	1.0721649	1.532467	1.655555	1.4200627
Rplp0	P14869	10	ATWK(*)SNYFLK(*)	K4	98.22	121.92	0.8602150	0.810126	0.955555	0.8752991

Rplp0	P14869	57	AVVLMGK(*)NTMMR(*)	K7	95.32	1000	0.8279569	0.784810	0.9	0.837589
Rplp0	P14869	146	TSFFQALGITTK(*)ISR(*)	K12	90.24	1000	-	-	1.055555556	
Rplp0	P14869	106	DMLLANK(*)VPAAAR(*)	K7	59.27	1000	0.6774193	0.848101	2.5222222	1.3492476
Rplp0	P14869	16	LK(*)IIQLLDDYPK(*)	K2	52.34	149.94	-	-	0.944444444	
Rplp0	P14869	50	GK(*)AVVLMGK(*)	K2	28.64	140.21	1.1720430	-	1.1111111	1.1415771
Map1b	P14873	506	LK(*)HLDFLK(*)	K2	64.81	129.72	0.8125	0.740259	1.0112359	0.8546652
Map1b	P14873	2192	TVTYK(*)HMDPPPAPMQDR(*)	K5	49.99	1000	-	-	0.651685393	
Map1b	P14873	2297	ATK(*)TTTTPEVK(*)	K3	38.03	107.73	0.7916666	0.870129	1.0337078	0.8985015
Map1b	P14873	429	LEMYVLNPVK(*)SSK(*)	K10	32.08	30.72	0.8541666	0.805194	1.0561797	0.9051804
Map1b	P14873	555	ESLK(*)PATK(*)PVASK(*)SVR(*)	K13	29.03	61.26	0.9583333	0.623376	1.0898876	0.8905325
Map1b	P14873	259	LSK(*)PC(+57.02)C(+57.02)YIFPGG	K3	20.78	1000	-	1.116883	0.7977528	0.957318
Map1b	P14873	2329	SAK(*)TATAGPGTTK(*)	K3	16.19	52.32	-	0.805194	0.8089887	0.8070918
Hmox1	P14901	196	MNTLEMTPEVK(*)HR(*)	K11	74.41	1000	0.9387755	0.929411	0.9484536	0.9388803
Jund	P15066	298	TLK(*)SQNTELASTASLLR(*)	K3	34.45	1000	0.6413043	-	-	
Cd44	P15379	74	LALSK(*)GFETC(+57.02)R(*)	K5	64.75	1000	0.6792452	-	0.8275862	0.7534157
Cd44	P15379	741	K(*)PSELNGEASK(*)SQEMVHLVNK(*	K11	34.6	66.25	0.7547169	-	0.9655172	0.8601171
Nme1	P15532	31	FEQK(*)GFR(*)	K4	98.19	1000	0.8865979	1.308641	0.9677419	1.0543273
Nme1	P15532	85	VWEGLNVVK(*)TGR(*)	K9	95.92	1000	0.8762886	0.827160	0.9354838	0.8796443
Nme1	P15532	124	NIIHGSDSVK(*)SAEK(*)	K10	87.16	97.69	0.9175257	0.888888	0.9677419	0.9247189
Nme1	P15532	85	MVWEGLNVVK(*)TGR(*)	K10	83.48	1000	0.8969072	0.740740	1.0860215	0.9078898
Nme1	P15532	31	R(*)FEQK(*)GFR(*)	K5	61.88	1000	0.6701030	0.629629	0.6236559	0.6411295
Nme1	P15532	12	TFIAIK(*)PDGVQR(*)	K6	60.16	1000	0.9381443	0.925925	0.8494623	0.9045109
Nme1	P15532	85	YM(+15.99)HSGPVVAMVWEGLNVV	K19	59.19	1000	-	-	0.774193548	
Nme1	P15532	100	VMLGETNPADSK(*)PGTIR(*)	K12	52.68	1000	0.7525773	0.716049	0.9247311	0.797786
H1-2	P15864	85	LGLK(*)SLVSK(*)	K4	96.52	115.97	1	0.795180	1.0821917	0.9591242
H1-2	P15864	75	ALAAAGYDVEK(*)NNSR(*)	K11	91.21	1000	1.1395348	0.951807	1.3424657	1.1446026
H1-2	P15864	46	ASGPPVSELITK(*)AVAASK(*)	K12	70.65	116.54	0.7674418	-	0.9041095	0.8357757
H1-2	P15864	90	SLVSK(*)GILVQTK(*)	K5	43.32	128.65	0.9186046	-	0.7945205	0.8565626
H1-2	P15864	46	K(*)ASGPPVSELITK(*)AVAASK(*)	K13	20.2	90.32	-	1.132530	-	
H1-2	P15864	97	GILVQTK(*)GTGASGSFK(*)	K7	13.91	62.82	-	1.602409	0.9315068	1.2669582
Lgals1	P16045	64	FNAHGDANTIVC(+57.02)NTK(*)EDG	K15	96.38	1000	1	0.792207	1.0344827	0.9422302
Lgals1	P16045	19	A(+42.01)C(+57.02)GLVASNLNLK(*	K18	84.75	42.99	0.8659793	0.974025	0.9310344	0.9236799
Lgals1	P16045	19	VASNLNLK(*)PGEC(+57.02)LK(*)VR	K14	84.54	47.92	0.7319587	0.818181	0.8965517	0.8155641
Lgals1	P16045	29	GEVASDAK(*)SFVLNLGK(*)	K8	83.95	197.82	0.8762886	0.753246	1.1379310	0.9224888

Lgals1	P16045	19	PGEC(+57.02)LK(*)VR(*)	K6	79.22	1000	0.8762886	0.662337	0.816091!	0.7849061
Lgals1	P16045	64	TIVC(+57.02)NTK(*)EDGTWGTEHR('	K7	68.15	1000	0.8453608	0.636363	0.942528	0.8080844
Lgals1	P16045	64	TK(*)EDGTWGTEHR(*)	K2	38.19	1000	-	-	1.229885057	
Lgals1	P16045	108	LPDGHEFK(*)FPNR(*)	K8	36.01	1000	1.5257731	-	-	
Lgals1	P16045	64	GDANTIVC(+57.02)NTK(*)EDGTWG	K11	35.9	1000	-	1.194805	0.965517	1.0801612
Lgals1	P16045	29	VR(*)GEVASDAK(*)SFVLNLGK(*)	K10	33.11	131.34	2.3092783	-	1.540229	1.9247541
Lgals1	P16045	19	LK(*)PGEC(+57.02)LK(*)VR(*)	K8	32.09	90.57	0.8659793	0.766233	0.919540	0.8505845
Lgals1	P16045	37	LGK(*)DSNNLC(+57.02)LHFNPR(*)	K3	25.69	1000	1.3092783	1.259740	1.425287	1.3314353
Lgals1	P16045	29	ASDAK(*)SFVLNLGK(*)	K5	3.44	154.74	-	-	0.494252874	
Lgals3	P16110	190	VIVC(+57.02)NTK(*)QDNNWGK(*)	K7	76.8	144.88	1	0.797101	1.171052	0.9893847
Ldhb	P16125	156	LSGLPK(*)HR(*)	K6	43.11	1000	0.7282608	0.928571	0.988095	0.8816425
Sptan1	P16546	2047	DQLLAAK(*)HIQSK(*)	K7	80.53	105.53	1.0106382	1.105263	0.945054	1.0203188
Sptan1	P16546	613	VQK(*)HQAFEAELSANQSR(*)	K3	75.93	1000	1.0106382	1	0.956043	0.9888941
Sptan1	P16546	229	TK(*)QDEVNAAWQR(*)	K2	73.85	1000	0.9468085	1.236842	1.021978	1.0685429
Sptan1	P16546	2349	LNHQEFK(*)SC(+57.02)LR(*)	K7	72.97	1000	0.8617021	0.960526	1	0.9407428
Sptan1	P16546	2404	ETENVK(*)SSEEIESAFR(*)	K6	59.5	1000	0.9361702	0.868421	0.967032	0.9238747
Sptan1	P16546	2333	EFSMMFK(*)HFDK(*)DK(*)	K7	47.97	48.12	1.2872340	0.789473	0.967032	1.0145802
Sptan1	P16546	84	LQK(*)HQAFEAEVQANSGAIVK(*)	K3	33.49	153.54	1.4361702	1.289473	-	1.3628219
Sptan1	P16546	814	GK(*)DLIGVQNLLK(*)	K2	19.79	150.82	-	-	1.010989011	
Sptan1	P16546	2426	ALSSEGK(*)PYVTK(*)EELYQNLTR(*)	K12	16.87	30.64	-	-	1.736263736	
Ctsa	P16675	55	ASDSK(*)HFHYWFVESQNDPK(*)	K5	46.3	146.08	1.4893617	0.802631	0.936842	1.0762785
Gapdh	P16858	252	LEK(*)PAK(*)YDDIK(*)	K6	76.57	42.6	0.7938144	0.843373	0.806818	0.8146687
Gapdh	P16858	192	TVDGPSGK(*)LWR(*)	K8	74.71	1000	0.8453608	0.843373	1.079545	0.9227599
Gapdh	P16858	217	AVGK(*)VIPELNGK(*)	K4	66.58	92.28	-	0.759036	0.568181	0.663609
Gapdh	P16858	70	GK(*)PITIFQER(*)	K2	37.99	1000	-	1.144578	0.647727	0.8961528
Gapdh	P16858	252	LEK(*)PAK(*)YDDIK(*)K(*)	K6	28.16	29.32	-	0.891566	0.340909	0.6162377
Gapdh	P16858	3	VK(*)VGVNGFGR(*)	K2	14.9	1000	-	-	2.045454545	
Zfx	P17012	489	AIEC(+57.02)DEC(+57.02)GK(*)HFS	K9	62.88	44.94	-	-	-	
Lamp2	P17047	67	TITIAVPDK(*)ATHDGSSC(+57.02)GD	K9	41.55	1000	-	0.792207	1.1375	0.9648539
Hmga1	P17095	7	S(+42.01)ESGSK(*)SSQPLASK(*)	K6	52.95	139.85	-	-	-	
Eno1	P17182	228	EALELLK(*)TAIAK(*)	K7	99.26	127.18	0.9042553	0.771084	0.901098	0.8588129
Eno1	P17182	60	FMGK(*)GVSQAVEHINK(*)	K4	98.41	281.19	0.9468085	0.867469	0.923076	0.9124518
Eno1	P17182	335	AASEK(*)SC(+57.02)NC(+57.02)LLLI	K5	91.07	171.6	0.9680851	0.819277	1	0.9291207
Eno1	P17182	193	IGAEVYHNLK(*)NVIK(*)	K10	77.88	88.98	0.9255319	0.855421	0.945054	0.9086695

Eno1	P17182	28	GNPTVEVDLYTAK(*)GLFR(*)	K13	67.93	1000	0.9148936	0.493975	0.879120{	0.7626635
Eno1	P17182	92	IDK(*)LMIEMDGTENK(*)	K3	60.43	139.71	-	-	0.769230769	
Eno1	P17182	256	SGK(*)YDLDFK(*)	K3	59.76	134.33	0.9042553	-	0.758241	0.8312485
Eno1	P17182	71	GVSQAVEHINK(*)TIAPALVSK(*)	K11	59.45	97.64	1.0744680	0.831325	1.021978(	0.9759238
Eno1	P17182	103	LMIEMDGTENK(*)SK(*)	K11	56.96	30.83	1.0957446	-	0.978021!	1.0368833
Eno1	P17182	343	SC(+57.02)NC(+57.02)LLLK(*)VNQI	K8	55.11	168.02	-	-	0.802197802	
Eno1	P17182	193	GAEVYHNLK(*)NVIK(*)	K9	43.84	63.97	0.9148936	1.084337	0.956043!	0.9850916
Eno1	P17182	262	YDLDFK(*)SPDDPSR(*)	K6	38.82	1000	-	-	1.131868132	
Ptbp1	P17225	217	TK(*)NNQFQALLQYADPVSAQHAK(*)	K2	81.56	413.48	0.9684210	0.907894	0.935483{	0.9372666
Ptbp1	P17225	366	VK(*)ILFNK(*)	K2	77.46	142.99	0.8947368	0.868421	0.956989	0.9067157
Ptbp1	P17225	258	IDFSK(*)LTSLNVK(*)	K5	72.73	142.27	0.7894736	0.75	0.903225{	0.8142332
Ptbp1	P17225	217	IITFTK(*)NNQFQALLQYADPVSAQHA	K6	63.23	150.82	0.7789473	-	0.892473	0.8357102
Ptbp1	P17225	91	VTNLLMLK(*)GK(*)	K8	56.65	33.18	0.7157894	0.907894	0.903225{	0.8423033
Ptbp1	P17225	211	FGTVLK(*)IITFTK(*)	K6	44.95	85.52	-	-	0.946236559	
Ap2a1	P17426	378	THIDTVINALK(*)TER(*)	K11	56.48	1000	0.5959595	-	0.876288(	0.7361241
Ap2a1	P17426	177	LYK(*)ASPDLVPMGEWTAR(*)	K3	29.75	1000	-	1.051948	-	
Ap2a1	P17426	894	IFK(*)ANHPMDAEVTK(*)	K3	9.73	122.88	-	-	1.278350515	
Ap2a2	P17427	117	LINNAIK(*)NDLASR(*)	K7	79.74	1000	1.28	0.876543	0.96875	1.0417644
Ap2a2	P17427	377	THIETVINALK(*)TER(*)	K11	60.2	1000	0.81	0.604938	0.958333	0.7910905
Ap2a2	P17427	569	SDSQLK(*)NADVELQQR(*)	K6	51.58	1000	1.29	0.617283	0.989583	0.9656224
Ppia	P17742	31	VPK(*)TAENFR(*)	K3	99.65	1000	0.82	0.795180	0.891304	0.835495
Ppia	P17742	131	HVVFGK(*)VK(*)	K6	97.88	36.05	0.78	0.795180	0.847826(	0.8076689
Ppia	P17742	49	GFGYK(*)GSSFHR(*)	K5	96.96	1000	0.82	0.867469	0.934782(	0.8740842
Ppia	P17742	44	ALSTGEK(*)GFGYK(*)	K7	96.62	94.22	0.9	0.915662	0.956521	0.9240615
Ppia	P17742	118	FIC(+57.02)TAK(*)TEWLDGK(*)	K6	82.32	110.43	0.79	0.867469	0.945652	0.8677074
Ppia	P17742	125	WLDGK(*)HVVFGK(*)	K5	78.1	123.86	0.95	0.867469	0.934782(	0.9174175
Ppia	P17742	125	TEWLDGK(*)HVVFGK(*)	K7	76.3	156.85	0.92	0.879518	0.967391	0.9223031
Ppia	P17742	118	IC(+57.02)TAK(*)TEWLDGK(*)	K5	60.04	125.04	1.12	0.903614	0.793478	0.9390309
Ppia	P17742	118	TAK(*)TEWLDGK(*)	K3	49.55	148.49	-	1.939759	0.978260{	1.45901
Ppia	P17742	31	VSFELFADK(*)VPK(*)TAENFR(*)	K12	48.74	68.47	1.02	1.409638	0.826086!	1.0852418
Ppia	P17742	125	EWLDGK(*)HVVFGK(*)	K6	40.73	143.14	0.9	0.963855	0.826086!	0.8966475
Ppia	P17742	28	VSFELFADK(*)VPK(*)	K9	27.55	41.59	27.02	-	-	
Ppia	P17742	118	AK(*)TEWLDGK(*)	K2	24.19	138.74	0.8	-	2.478260{	1.6391304
Ppia	P17742	49	GYK(*)GSSFHR(*)	K3	22.17	1000	-	-	1.043478261	

Ppia	P17742	131	TEWLDGK(*)HVVFGK(*)VK(*)	K13	22.1	20.41	-	0.987951	0.782608(	0.8852803
Ppia	P17742	125	TEWLDGK(*)HVVFGK(*)VK(*)	K7	22.1	135.81	-	0.987951	0.782608(	0.8852803
Tpi1	P17751	244	GWLK(*)SNVNDGVAQSTR(*)	K4	98.55	1000	0.8901098	0.891891	1.247191(	1.0097309
Tpi1	P17751	199	VVFEQTK(*)VIADNVK(*)	K7	96.38	128.65	0.9670329	0.945945	0.943820:	0.9522664
Tpi1	P17751	225	VVLAYEPVWAIGTGK(*)TATPQQAQE'	K15	72.07	59.65	1.0219780	1.094594	1.235955(	1.1175092
Tpi1	P17751	119	IAVAAQNC(+57.02)YK(*)VTNGAFTG	K10	68.24	170.4	1.1208791	-	1.662921:	1.3919002
Mthfd2	P18155	88	R(*)PHLSVILVGDNPASHSYVLNK(*)T	K22	49.43	1000	0.9247311	0.789473	0.956043!	0.8900829
Mthfd2	P18155	194	TGIPTLGK(*)NVVVAGR(*)	K8	46.93	1000	0.6666666	1.065789	0.912087!	0.8815147
Ctsd	P18242	355	YILK(*)VSQGGK(*)	K4	67.24	95.35	1	0.786666	0.988235:	0.9249673
Ctsd	P18242	343	LGGK(*)NYELHPDK(*)	K4	22.35	99.23	-	1.066666	1	1.0333333
Rps6ka3	P18654	525	TITK(*)TVEYLHAQGVVHR(*)	K4	49.04	1000	0.6346153	-	0.77	0.7023077
Cfl1	P18760	114	SK(*)MIYASSK(*)	K2	93.45	160.69	0.8775510	0.792207	1.081395:	0.9170514
Cfl1	P18760	19	VFNDMK(*)VR(*)	K6	89.26	1000	0.8877551	0.818181	1.023255!	0.9097309
Cfl1	P18760	132	LTGIK(*)HELQANC(+57.02)YEEVK(*)	K5	79.3	281.43	0.9285714	0.753246	1	0.8939394
Cfl1	P18760	132	K(*)LTGIK(*)HELQANC(+57.02)YEEV	K6	70.44	86.16	0.6530612	0.714285	0.755813!	0.7077203
Cfl1	P18760	92	YALYDATYETK(*)ESK(*)	K11	67.24	26.52	1.2346938	0.792207	1.058139!	1.0283471
Cfl1	P18760	132	LTGIK(*)HELQANC(+57.02)YEEVK(*)	K5	63.69	336.59	1.1326530	0.987012	0.802325!	0.9739972
Cfl1	P18760	132	K(*)LTGIK(*)HELQANC(+57.02)YEEV	K6	58.16	52.68	1.1020408	0.974025	1.069767·	1.0486114
Gnao1	P18872	193	TTGIVETHFTFK(*)NLHFR(*)	K12	54.69	1000	0.8782608	-	0.948453(	0.9133572
Fasn	P19096	70	FDASFFGVHPK(*)QAHTMDPQLR(*)	K11	92.5	1000	1.0421052	0.871794	0.989130·	0.9676769
Fasn	P19096	776	GVK(*)SSC(+57.02)TIIPLMK(*)	K3	88.64	149.94	0.6736842	0.820512	0.891304:	0.7951671
Fasn	P19096	2187	K(*)LQEMSSK(*)TDSATDTTAPK(*)	K8	84.12	81.81	0.6947368	0.769230	0.869565:	0.7778443
Fasn	P19096	1143	ELQLC(+57.02)K(*)GLAR(*)	K6	77.82	1000	0.8210526	0.756410	0.967391:	0.8482847
Fasn	P19096	2399	VAASVDLITK(*)SHHSLDR(*)	K10	76.07	1000	0.9578947	0.948717	1	0.9688709
Fasn	P19096	1840	YM(+15.99)AQGK(*)HIGK(*)	K6	69.53	63.97	-	0.782051	-	
Fasn	P19096	2198	TDSATDTTAPK(*)SR(*)	K11	69.12	1000	0.5052631	0.756410	-	0.6308367
Fasn	P19096	1920	TGYQAK(*)HIR(*)	K6	66.52	1000	1.0421052	0.717948	1.097826(	0.9526267
Fasn	P19096	1584	LSPDAIPGK(*)WASR(*)	K9	61.72	1000	-	-	1.163043478	
Fasn	P19096	2187	LQEMSSK(*)TDSATDTTAPK(*)	K7	59.56	180.02	0.9263157	-	0.826086!	0.8762014
Fasn	P19096	1844	HIGK(*)VLVQVR(*)	K4	58.81	1000	0.6947368	0.717948	1.032608(	0.8150981
Fasn	P19096	1491	LDPGSPELQQVLK(*)HDLVMNVYR(*)	K13	54.78	1000	1.0315789	-	1.423913(	1.227746
Fasn	P19096	2442	AK(*)TGGTYGEDLGADYNLSQVC(+57	K2	49.76	250.91	-	-	1.304347826	
Fasn	P19096	2431	AK(*)YHGNVTLLR(*)	K2	27.25	1000	0.6315789	-	1.717391:	1.1744851
Fasn	P19096	2384	VLEALLPLK(*)SLEDR(*)	K9	24.65	1000	0.7473684	-	0.434782(	0.5910755

Fasn	P19096	1826	C(+57.02)TVFPK(*)AQVEDAFR(*)	K6	17.57	1000	1.3578947	-	-	
Fasn	P19096	2187	SSK(*)TDSATDTTAPK(*)	K3	16.64	176.81	0.8631578	0.910256	1.391304	1.0549062
Gstp1	P19157	82	SLGLYGK(*)NQR(*)	K7	71.47	1000	0.9662921	1	0.988764	0.9850187
Gstp1	P19157	103	GK(*)YVTLIYTNYENGK(*)	K2	47.12	205.45	-	-	0.91011236	
Rpl13a	P19253	53	LK(*)YLAFLR(*)	K2	38.2	1000	-	1.123456	0.968421	1.0459389
Rpl13a	P19253	103	LK(*)VLDGIPPPYDK(*)	K2	29.12	176.98	-	0.716049	-	
Serpinh1	P19324	216	FHHK(*)MVDNR(*)	K4	94.51	1000	0.9897959	0.898734	1.042105	0.9768785
Serpinh1	P19324	286	LLTK(*)EQLK(*)	K4	91.58	97.69	0.8469387	0.810126	0.936842	0.8646358
Serpinh1	P19324	159	QHYNC(+57.02)EHSK(*)INFR(*)	K9	84.69	1000	1.0714285	0.962025	1.094736	1.0427302
Serpinh1	P19324	318	GVVEVTHDLQK(*)HLAGLGLTEAIDK(	K11	80.65	366.7	1.2040816	0.974683	0.926315	1.035027
Serpinh1	P19324	206	FK(*)PHWDEK(*)	K2	76.29	72.77	-	1	1.042105	1.0210526
Serpinh1	P19324	206	AMFFK(*)PHWDEK(*)	K5	74.89	121.2	1.1632653	1.354430	1.084210	1.2006354
Serpinh1	P19324	206	FFK(*)PHWDEK(*)	K3	67.49	143.14	0.7755102	0.620253	0.8	0.7319211
Serpinh1	P19324	295	AWMGK(*)MQK(*)	K5	49.99	28.96	0.9591836	0.822784	1.115789	0.9659193
Serpinh1	P19324	38	LSSK(*)ATTLAER(*)	K4	49.69	1000	0.8061224	-	0.894736	0.8504296
Serpinh1	P19324	150	SSK(*)QHYNC(+57.02)EHSK(*)	K3	36.84	105.42	1.2244897	1.151898	-	1.1881943
Serpinh1	P19324	212	PHWDEK(*)FHHK(*)	K6	28.05	58.99	-	-	1.094736842	
Cox5b	P19536	73	AASGTK(*)EDPNLVPSISNK(*)	K6	31.04	86.82	0.6477272	1.791666	0.681818	1.040404
Cox4i1	P19783	29	AHGSVVK(*)SEDYAFPTYADR(*)	K7	83.17	1000	0.9223300	0.779069	0.886597	0.8626659
Cox4i1	P19783	67	EK(*)ADWSSLSR(*)	K2	41.71	1000	0.9708737	1.488372	1.051546	1.1702641
Hspa5	P20029	524	NK(*)ITITNDQNR(*)	K2	83.05	1000	1.2446808	0.878048	0.890109	1.0042798
Hspa5	P20029	155	MK(*)ETAEAYLGK(*)	K2	80.82	203.33	1.0106382	1	0.901098	0.9705791
Hspa5	P20029	82	LIGDAAK(*)NQLTSNPENTVFDAK(*)	K7	46.62	142.24	0.7872340	-	1.054945	0.9210895
Hspa5	P20029	126	TK(*)PYIQVDIGGGQTK(*)	K2	39.35	100.88	1.0425531	0.817073	0.527472	0.7956996
Hspa5	P20029	574	SLK(*)NQIGDK(*)	K3	33.85	78.19	-	1.085365	-	
Hspa5	P20029	327	AK(*)FEELNMDLFR(*)	K2	16.34	1000	-	-	1.626373626	
Hspa5	P20029	383	EFFNGK(*)EPSR(*)	K6	5.24	1000	0.3191489	-	-	
Hexb	P20060	196	HFLPVK(*)TILK(*)	K6	33.74	35.82	0.7731958	0.821428	0.961538	0.8520543
Hexb	P20060	444	NYYK(*)VEPLNFEGSEK(*)	K4	6.31	41.98	-	-	0.91025641	
Prdx3	P20108	197	GLFIIDPNGVVK(*)HLSVNDLPVGR(*	K12	80.85	1000	-	-	1.225806452	
Vim	P20152	445	TLLIK(*)TVETR(*)	K5	98.35	1000	0.9375	0.936708	0.977777	0.9506622
Vim	P20152	292	NLQEAEEWYK(*)SK(*)	K10	96.74	30.83	0.8020833	0.873417	0.966666	0.8807226
Vim	P20152	120	FANYIDK(*)VR(*)	K7	94.23	1000	0.875	0.860759	0.9	0.8785865
Vim	P20152	139	ILLAELEQLK(*)GQGK(*)	K10	84.39	76.73	0.8854166	1.265822	0.955555	1.0355983

Vim	P20152	282	QQYESVAAK(*)NLQEAEEWYK(*)	K9	52.81	258.5	-	-	1.211111111	
Vim	P20152	439	ETNLESLPLVDTHSK(*)R(*)	K15	36.81	1000	-	-	0.944444444	
Vim	P20152	104	TNEK(*)VELQELNDR(*)	K4	23.41	1000	2.3020833	-	-	
Vim	P20152	129	FLEQQNK(*)ILLAELEQLK(*)	K7	20.37	168.06	-	-	1.077777778	
Vim	P20152	168	QVDQLTNDK(*)AR(*)	K9	16.9	1000	1.1875	1.101265	1.077777	1.1221812
Vim	P20152	294	SK(*)FADLSEAANR(*)	K2	9.88	1000	-	1.911392	-	
Vim	P20152	129	NK(*)ILLAELEQLK(*)	K2	9.58	187.15	-	-	1.188888889	
Prim1	P20664	153	EDFGFK(*)HR(*)	K6	53.18	1000	0.89	0.696629	1.094117	0.8935823
Tpm3	P21107	153	EAK(*)HIAEEADR(*)	K3	85.11	1000	0.8349514	0.807228	1.048192	0.896791
Gnaq	P21279	52	LLLLGTGESGK(*)STFIK(*)	K11	72.94	60.87	0.7767857	0.662921	1	0.8132357
Eno3	P21550	28	GNPTVEVDLHTAK(*)GR(*)	K13	38.07	1000	-	1.422535	0.905882	1.1642088
Eno3	P21550	71	AVEHINK(*)TLGPALLEK(*)	K7	31.03	100.79	-	1.253521	0.764705	1.0091135
Lmnb2	P21619	554	AAK(*)HSSVQGR(*)	K3	3.15	1000	-	-	0.106382979	
Mfge8	P21956	234	AEYLK(*)TFK(*)	K5	51.29	32.97	0.9801980	2.160493	1.287356	1.4760161
Tgm2	P21981	458	EVFTK(*)ANHLNK(*)	K5	18.09	102.87	1.4891304	1.731343	1.134831	1.4517684
Ap1g1	P22892	334	YVALTSLLK(*)TVQTDHNAVQR(*)	K9	74.63	1000	0.9578947	-	0.882978	0.9204367
Ap1g1	P22892	224	ILK(*)NLIMSGYSPEHDVSGISDPFLQ\	K3	19.21	1000	-	-	0.670212766	
Hmbs	P22907	74	IGEK(*)SLFTK(*)	K4	63.72	38.19	-	-	0.893617021	
Hmbs	P22907	70	ILDTALSK(*)IGEK(*)	K8	40.06	46.31	0.6629213	-	0.680851	0.6718862
Hmbs	P22907	132	FIGK(*)TLETLPEK(*)	K4	24.28	98.53	-	1.120689	-	
Eif3a	P23116	409	VTK(*)VLNWVR(*)	K3	81.02	1000	0.7826086	0.769230	1.131868	0.8945692
Eif3a	P23116	747	ALEHK(*)NR(*)	K5	74.45	1000	-	0.923076	0.945054	0.9340659
Eif3a	P23116	559	EEQHQLAVNAYLK(*)NSR(*)	K13	66.02	1000	0.9673913	0.833333	1.043956	0.9482269
Eif3a	P23116	285	VSTVFWK(*)SGNALFHASTLHR(*)	K7	64.51	1000	0.9673913	0.884615	1.065934	0.9726469
Eif3a	P23116	84	IK(*)SLEDVVR(*)	K2	60.98	1000	0.8260869	0.923076	1.032967	0.927377
Eif3a	P23116	1331	TK(*)NETDEDGWTTVR(*)	K2	59.39	1000	0.7391304	0.833333	0.725274	0.7659128
Eif3a	P23116	278	K(*)PPK(*)PQLMANYYNK(*)VSTVFW	K14	47.15	61.82	-	-	0.714285714	
Eif3a	P23116	278	YYNK(*)VSTVFWK(*)	K4	38.73	61.68	-	-	0.912087912	
Eif3a	P23116	84	NIC(+57.02)QQVNIK(*)SLEDVVR(*)	K9	20.29	1000	1.1630434	-	-	
Eif3a	P23116	285	WK(*)SGNALFHASTLHR(*)	K2	16.77	1000	1.0978260	-	0.857142	0.9774845
Eif3a	P23116	711	LEEIPLIK(*)SAYEEQR(*)	K8	16.19	1000	0.9891304	-	0.846153	0.9176421
Eif3a	P23116	711	R(*)LEEIPLIK(*)SAYEEQR(*)	K9	11.69	1000	-	-	1.098901099	
Cbx3	P23198	5	A(+42.01)SNK(*)TTLQK(*)	K4	89.89	98.37	0.5688073	0.805194	1.011904	0.7953023
Cbx3	P23198	44	VVNGK(*)VEYFLK(*)	K5	77.89	90.32	0.7798165	0.974025	0.976190	0.910011

Cbx3	P23198	5	SNK(*)TTLQK(*)	K3	71.09	115.97	-	0.948051	0.952380!	0.9502165
Gja1	P23242	13	LLDK(*)VQAYSTAGGK(*)	K4	13.2	135.36	1.4636363	-	-	
Mov10	P23249	509	SLESNPEQLQAMK(*)HIVR(*)	K13	39.64	1000	0.6237623	-	1.126436	0.8750996
Mov10	P23249	71	IANLAFVTK(*)TR(*)	K9	28.19	1000	1.0594059	-	1.367816(	1.213611
Mov10	P23249	121	AEYLHGK(*)HGVDVEVQGPHEAR(*)	K7	27.25	1000	0.9405940	-	0.712643(	0.8266189
Mov10	P23249	847	YC(+57.02)ITK(*)LDR(*)	K5	9.77	1000	-	-	0.931034483	
Mov10	P23249	696	SPLALK(*)HGLGYSLLER(*)	K6	8.77	1000	-	-	1.724137931	
Pnp	P23492	95	FHMYEGYSLSK(*)VTFPVR(*)	K11	56.49	1000	-	-	0.579545455	
Pnp	P23492	244	SLITNK(*)VVMDYENLEK(*)	K6	45.01	183.74	0.8736842	-	1.079545	0.9766148
Pcmt1	P23506	4	A(+42.01)WK(*)SGGASHSELIHNLR(	K3	89.18	1000	0.9892473	0.925	1.097560!	1.0039361
Pcmt1	P23506	206	MK(*)PLMGVIYVPLTDK(*)EK(*)	K2	35.04	90.57	-	-	1.341463415	
Pcmt1	P23506	27	TDK(*)VFEVMLATDR(*)	K3	5.32	1000	-	-	1.292682927	
Tsta3	P23591	199	VHLAK(*)SSDSALTVWGTGK(*)PR(*)	K5	55.3	152.87	1.0361445	0.675324	0.858695(	0.8567216
Tsta3	P23591	199	VHLAK(*)SSDSALTVWGTGK(*)	K5	45.24	166.43	0.7108433	-	0.967391	0.8391173
Glb1	P23780	288	TK(*)TLATSLYNLLAR(*)	K2	7.23	1000	-	-	1.078125	
Pcgf2	P23798	73	SDK(*)TLQDIVYK(*)	K3	31.01	183.32	-	-	-	
Cryab	P23927	103	VLGDVIEVHGK(*)HEER(*)	K11	65.12	1000	1.2105263	1.2	1.154761!	1.1884294
Zfp36l2	P23949	157	YK(*)TELC(+57.02)R(*)	K2	86.14	1000	0.8	0.670454	1.085365	0.8519401
Zfp36l2	P23949	157	YK(*)TELC(+57.02)R(*)PFEESGTC(+5	K2	76.78	47.75	0.5473684	-	-	
Cat	P24270	480	AVK(*)NFTDVHPDYGAR(*)	K3	61	1000	0.9784946	1.175675	1.036144!	1.0634383
Cat	P24270	449	TFYTK(*)VLNEEER(*)	K5	59.06	1000	1.2688172	-	1.301204	1.285011
Cat	P24270	237	FHYK(*)TDQGIK(*)	K4	46.18	109.28	1.5376344	1.391891	1.542168	1.490565
Cat	P24270	243	TDQGIK(*)NLPVGEAGR(*)	K6	42.46	1000	-	-	1.313253012	
Bcat1	P24288	215	AWK(*)GGTGDC(+57.02)K(*)	K3	40.15	110.43	-	-	0.956521739	
Bcat1	P24288	28	AK(*)DLIITPATVLK(*)	K2	34.59	156.9	0.5869565	1.353658	0.967391	0.9693355
Ppib	P24369	131	LK(*)HYGPGWVSMANAGK(*)	K2	88.68	300.97	0.9587628	0.962025	0.956043!	0.9589441
Ppib	P24369	71	TVPK(*)TVDNFVALATGEK(*)	K4	84.74	137.95	0.7938144	0.873417	0.846153	0.8377953
Ppib	P24369	171	HVVFGK(*)VLEGMDVVR(*)	K6	78.54	1000	0.9793814	1.101265	1.021978(	1.0342084
Ppib	P24369	186	K(*)VESTK(*)TDSR(*)	K6	75.28	98.37	0.7938144	1.531645	0.846153	1.0572046
Ppib	P24369	165	TSWLDGK(*)HVVFGK(*)	K7	74.26	135.5	1	0.987341	1	0.9957806
Ppib	P24369	116	GDGTGGK(*)SIYGER(*)	K7	72.91	1000	1.2268041	1.493670	1.087912(	1.2694624
Ppib	P24369	67	VVFGLFGK(*)TVPK(*)	K8	71.72	63.97	0.8659793	0.911392	0.945054!	0.9074756
Ppib	P24369	89	GFGYK(*)NSK(*)	K5	56.15	34.94	1.1752577	0.911392	0.813186	0.9666123
Capg	P24452	345	ESPIFK(*)QFFK(*)	K6	42.32	72.09	0.7553191	-	0.921348	0.8383337

Capg	P24452	349	QFFK(*)NWK(*)	K4	40.08	44.44	-	1.150684	1.011235	1.0809604
Lta4h	P24527	580	DLAAFDK(*)SHDQAVHTYQEHK(*)	K7	59.99	33.02	1.1505376	0.739726	1.088888	0.9930509
Lta4h	P24527	593	SHDQAVHTYQEHK(*)ASMHPVTAML	K13	42.35	1000	-	-	1.066666667	
Impdh2	P24547	349	PQATAVYK(*)VSEYAR(*)	K8	91.01	1000	0.8453608	0.726190	0.876288	0.8159467
Impdh2	P24547	438	IK(*)VAQGVSGAVQDK(*)	K2	67.04	261.96	0.8247422	0.75	0.876288	0.8170103
Impdh2	P24547	422	GMGSLDAMDK(*)HLSSQNR(*)	K10	59.23	1000	1.2989690	0.976190	1.082474	1.1192113
Impdh2	P24547	474	LIAGIQHSC(+57.02)QDIGAK(*)SLTQ	K15	44.91	1000	1.2268041	0.797619	0.969072	0.9978318
M6pr	P24668	31	IEEK(*)SC(+57.02)DLVGEK(*)	K4	7.02	99.23	-	-	2.393258427	
Cdk11b	P24788	708	INAEDGLK(*)HEYFR(*)	K8	22.4	1000	-	-	1.513157895	
Cdk11b	P24788	120	SHSAEGGK(*)HAR(*)	K8	8.02	1000	-	1.015873	-	
Ccnb1	P24860	405	YAASK(*)HAK(*)	K5	16.52	39.3	-	-	1.11627907	
Mcm3	P25206	293	SK(*)DVFEQLAR(*)	K2	62.41	1000	-	0.790123	0.824175	0.8071496
Mcm3	P25206	547	IYEK(*)HDSLLHGTK(*)	K4	47.12	143.22	1.04	0.864197	1.098901	1.0010329
Rps2	P25444	275	SPYQEFTDHLVK(*)THTR(*)	K12	95.99	1000	0.9489795	0.851851	0.978021	0.9262845
Rps2	P25444	263	ETVFTK(*)SPYQEFTDHLVK(*)	K6	88.6	99.65	0.8469387	0.753086	0.912087	0.837371
Rps2	P25444	65	AEDK(*)EWIPVTK(*)LGR(*)	K11	87.22	186.64	0.8061224	0.728395	0.901098	0.8118721
Rps2	P25444	65	EWIPVTK(*)LGR(*)	K7	67.69	1000	0.8061224	0.604938	0.912087	0.7743829
Rps2	P25444	173	GYWGNK(*)IGK(*)	K6	47.68	42.6	-	-	1.340659341	
Rps2	P25444	238	GC(+57.02)TATLGNFAK(*)ATFDAISK	K11	16.72	71.07	-	-	1.494505495	
Rps2	P25444	176	IGK(*)PHTVPC(+57.02)K(*)	K3	13.55	48.8	-	-	1.054945055	
Nfkb1	P25799	114	SLVGK(*)HC(+57.02)EDGVC(+57.02	K5	29.95	135.22	1.1754385	0.527027	1.147727	0.9500643
Ubtf	P25976	266	DYIQK(*)HPELNISEEGITK(*)	K5	58.01	90.21	0.8152173	0.740259	1.022988	0.8594885
Ubtf	P25976	47	FK(*)TTESHMDWEK(*)	K2	45.26	154.16	0.8260869	1.103896	1.114942	1.0149752
Tln1	P26039	1917	PAAVAAENEEIGAHIK(*)HR(*)	K16	92.72	1000	1.1578947	0.857142	0.902173	0.9724038
Tln1	P26039	284	IFQAHK(*)NC(+57.02)GQMSEIEAK(*	K6	87.71	187.15	0.9052631	0.753246	0.967391	0.8753004
Tln1	P26039	98	MLDGTVK(*)TIMVDDSK(*)	K7	83.45	127.58	0.9368421	0.870129	0.945652	0.9175414
Tln1	P26039	2115	VGDDPAVWQLK(*)NSAK(*)	K11	70.38	48.12	1.0736842	0.870129	0.565217	0.8363438
Tln1	P26039	1933	VQELGHGC(+57.02)SALVTK(*)AGAL	K14	46.38	179.71	-	1.974025	-	
Tln1	P26039	406	SK(*)DHFGLEGDEESTMLEDSVSPK(*	K2	41.29	89.97	1	-	-	
Tln1	P26039	306	SLK(*)TYGVSFFLVK(*)	K3	40.37	158.16	0.7894736	-	1.076086	0.9327803
Tln1	P26039	295	NC(+57.02)GQMSEIEAK(*)VR(*)	K11	38.94	1000	0.7789473	-	1.130434	0.9546911
Tln1	P26039	164	QK(*)LHTDDELNWLDHGR(*)	K2	35.83	1000	1.2	1.077922	1.119565	1.1324958
Tln1	P26039	2099	ALGDLISATK(*)AAAGK(*)	K10	34.74	48.54	1.6842105	1.857142	1.293478	1.6116105
Tln1	P26039	441	VGK(*)VEHGSVALPAIMR(*)	K3	33.43	1000	2.0631578	-	0.967391	1.5152746

Tln1	P26039	15	ISIGNVVK(*)TMQFEPSTMVYDAC(+5	K8	22.94	1000	-	-	0.989130435	
Tln1	P26039	1306	AQVVSNLK(*)GISMSSSK(*)	K8	20.81	68.3	0.7578947	1.181818	0.673913(	0.8712087
Ezr	P26040	143	EMHK(*)SGYLSSER(*)	K4	70.41	1000	0.8333333	0.918918	0.947368	0.8998736
Msn	P26041	296	R(*)K(*)PDTIEVQQMK(*)	K2	85.11	116.35	-	-	0.3	
Msn	P26041	3	PK(*)TISVR(*)	K2	84.29	1000	-	0.728395	-	
Msn	P26041	35	QLFDQVVK(*)TIGLR(*)	K8	84.03	1000	0.8315789	0.604938	1.033333	0.8232835
Msn	P26041	237	LTPK(*)IGFPWSEIR(*)	K4	72.02	1000	-	0.370370	0.9	0.6351852
Msn	P26041	143	EVHK(*)SGYLAGDK(*)	K4	58.87	118.18	0.9789473	0.950617	0.966666(	0.9654104
Msn	P26041	162	VLEQHK(*)LNK(*)	K6	32.47	77.24	1.1368421	1.234567	1.277777	1.2163959
Rdx	P26043	165	LTK(*)EQWEER(*)	K3	74.24	1000	0.8297872	0.987179	0.911111	0.9093593
Rdx	P26043	35	QLFDQVVK(*)TVGLR(*)	K8	15.54	1000	-	-	1.933333333	
Ctnna1	P26231	737	GPLK(*)NTSDVISAAK(*)	K4	59.44	140.01	0.6969696	0.392405	0.736263	0.6085462
U2af2	P26369	413	YGLVK(*)SIEIPR(*)PVDGVEVPGC(+5	K5	42.34	107.2	0.6428571	0.576923	0.977528(	0.7324361
U2af2	P26369	413	YGLVK(*)SIEIPR(*)	K5	20.24	1000	-	1.128205	1.044943	1.0865745
U2af2	P26369	462	VVVTK(*)YC(+57.02)DPDSYHR(*)	K5	16.45	1000	0.5714285	1.858974	1.561797	1.3307336
Glud1	P26443	480	FGK(*)HGGTIPVVPTAEFQDR(*)	K3	95.73	1000	0.9489795	0.827160	1.054945(	0.943695
Glud1	P26443	191	INPK(*)NYTDNELEK(*)	K4	79.31	85.33	-	0.827160	0.912087	0.8696242
Psmd7	P26516	204	GLNSK(*)LLDIR(*)	K5	87.56	1000	0.8736842	0.924050	0.977777	0.9251709
Psmd7	P26516	103	LHK(*)NDIAINELMK(*)	K3	76.77	135.36	0.8421052	0.784810	1.033333	0.8867496
Psmd7	P26516	199	ITNQVHGLK(*)GLNSK(*)	K9	67.01	86.16	1.2105263	0.886075	1.033333	1.0433119
Psmd7	P26516	279	SVVALHNLINNK(*)IANR(*)	K12	53.4	1000	1.2	-	0.977777	1.0888889
Sars	P26638	62	NLC(+57.02)SK(*)TIGEK(*)	K5	52.7	54.46	0.5555555	-	0.772727	0.6641414
Sars	P26638	323	VHQFEK(*)IEQFVYSSPHDNK(*)	K6	40.62	163.91	-	-	1.227272727	
Sars	P26638	419	MMDK(*)VEFVHMLNATMC(+57.02)	K4	7.36	1000	-	-	2.045454545	
Fkbp1a	P26883	53	FTLGK(*)QEVIR(*)	K5	92.36	1000	0.97	0.948717	0.842696(	0.9204715
Man2a1	P27046	190	DK(*)TQYIFNNMVLK(*)	K2	85.95	281.19	0.8210526	0.759493	0.902173	0.8275734
Man2a1	P27046	416	TK(*)VLLAPLGDDFR(*)	K2	85.33	1000	0.7789473	0.708860	0.869565	0.7857911
Man2a1	P27046	849	IYSDVTC(+57.02)FLEHVTHK(*)VR(*)	K15	54.61	1000	0.7684210	-	0.989130	0.8787757
Man2a1	P27046	975	GLGQGVHDNK(*)ITANLFR(*)	K10	51.36	1000	0.6842105	-	0.858695(	0.7714531
Man2a1	P27046	1064	MGK(*)GYSDEAALILHR(*)	K3	39.12	1000	1.8210526	-	1	1.4105263
Man2a1	P27046	886	ISSK(*)INNQNR(*)	K4	10.05	1000	-	1.227848	-	
Snrpb	P27048	36	AFDK(*)HMNLILC(+57.02)DC(+57.0	K4	80.88	1000	1.25	-	0.926315	1.0881579
Snrpb	P27048	32	IFIGTFK(*)AFDK(*)	K7	58.07	72.65	0.84	0.719512	0.915789	0.8251006
Snrpb	P27048	36	AFDK(*)HMNLILC(+57.02)DC(+57.0	K4	51.32	135.22	0.96	0.780487	1.336842	1.0257766

Map4	P27546	825	MTAK(*)SASADLSR(*)	K4	64.05	1000	0.8351648	-	0.976744:	0.9059545
Map4	P27546	988	DNIK(*)HVPGGGNVQIQNK(*)	K4	36.09	207.86	0.7802197	1.424657	1.290697(	1.1651917
Map4	P27546	911	SK(*)VGSTENIK(*)	K2	27.85	86.34	-	-	1.081395349	
Gna13	P27601	61	ILLLGAGESGK(*)STFLK(*)	K11	48.6	60.87	-	-	1.569620253	
Plaa	P27612	259	IWK(*)HGEC(+57.02)AQTIR(*)	K3	65.26	1000	0.9897959	0.924050	1.052631!	0.988826
Plaa	P27612	622	LSIK(*)HPNVNENFC(+57.02)NEK(*)	K4	50.38	91.71	1.0816326	0.658227	0.905263:	0.8817079
Plaa	P27612	244	SK(*)DFVTTAEDR(*)	K2	17.72	1000	-	-	0.410526316	
Plaa	P27612	690	ESLMSHAIELK(*)SGSNK(*)	K11	15.35	30.64	-	-	1.284210526	
Plaa	P27612	651	GK(*)PANQLLALR(*)	K2	10.11	1000	-	-	1.178947368	
Xrcc5	P27641	265	IVAYK(*)SIVQEK(*)	K5	58.02	76.27	1.0439560	-	0.939024:	0.9914902
Xrcc5	P27641	534	VK(*)TLFPLTEVIK(*)	K2	45.59	140.72	0.7252747	-	0.670731:	0.6980032
Rpl3	P27659	300	LIK(*)NNASTDYDLSDK(*)	K3	98.57	287.12	0.8877551	0.822784	0.947916(	0.8861522
Rpl3	P27659	120	FYK(*)NWHK(*)	K3	92.98	64.94	0.8163265	0.784810	0.84375	0.8149622
Rpl3	P27659	286	IYK(*)IGQGYLIK(*)	K3	90.33	171.49	-	0.772151	0.9375	0.8548259
Rpl3	P27659	103	TFK(*)TVFAEHISDEC(+57.02)K(*)	K3	86.91	310.88	0.9285714	0.772151	0.864583:	0.8551022
Rpl3	P27659	50	LGYK(*)AGMTHIVR(*)	K4	71.79	1000	0.9183673	0.784810	0.885416(	0.8628647
Rpl3	P27659	50	DDASK(*)PVHLTAFLGYK(*)AGMTHI\	K16	57.29	159.14	1.1734693	-	0.78125	0.9773597
Rpl3	P27659	272	AGQK(*)GYHHR(*)	K4	48.73	1000	0.9591836	-	-	
Rpl3	P27659	103	TFK(*)TVFAEHISDEC(+57.02)K(*)R(*	K3	45.59	186.68	0.7448979	-	0.895833:	0.8203656
Pdia3	P27773	366	YLK(*)SEPIPESNEGPVK(*)	K3	96.49	178.45	0.7920792	0.768292	0.831578!	0.7973169
Pdia3	P27773	218	FEDK(*)TVAYTEK(*)	K4	96.48	170.44	0.8811881	0.890243	0.947368	0.9062668
Pdia3	P27773	82	AK(*)VDC(+57.02)TANTNTC(+57.02	K2	82.27	323.97	0.8217821	0.695121	0.947368	0.8214242
Pdia3	P27773	104	YGVSGYPTLK(*)IFR(*)	K10	78.87	1000	0.7821782	1	1.305263:	1.0291471
Pdia3	P27773	274	NAK(*)GSNYWR(*)	K3	55.84	1000	0.9108910	1.146341	1.326315:	1.1278494
Pdia3	P27773	82	GIVPLAK(*)VDC(+57.02)TANTNTC(+	K7	44.09	101.74	0.9108910	1.853658	1.042105:	1.268885
Braf	P28028	607	QTAQGMDYLHAK(*)SIIHR(*)	K12	15.28	1000	-	-	0.6	
Aco1	P28271	621	IETVNK(*)SWNALAAPSEK(*)	K6	17.85	122.88	-	1.955882	1.428571	1.6922269
Aco1	P28271	266	PHPLVTSTDIVLTITK(*)HLR(*)	K16	13.24	1000	-	-	1.226190476	
Lox	P28301	314	VAEGHK(*)ASFC(+57.02)LEDTSC(+5	K6	40.81	1000	3.2574257	-	1.238636:	2.2480311
Lox	P28301	264	VK(*)NQGTSDFLPSR(*)	K2	40.66	1000	1.0990099	0.776315	0.761363(	0.8788964
Lox	P28301	264	VK(*)NQGTSDFLPSR(*)PR(*)	K2	29.18	1000	0.4950495	-	-	
Apex1	P28352	62	SATLK(*)IC(+57.02)SWNVDGLR(*)	K5	70.75	1000	-	-	0.786516854	
Adh5	P28474	107	FC(+57.02)LNPK(*)TNLC(+57.02)Q	K6	78.9	71.75	0.7840909	0.75	0.906976:	0.8136892
Adh5	P28474	323	GTAFGGWK(*)SVESVPK(*)	K8	67.24	136.94	1.1022727	-	1.197674	1.1499736

Nap1l1	P28656	82	VNALK(*)NLQVK(*)	K5	97.94	127.18	0.8764044	0.935064	0.929411	0.9136271
Nap1l1	P28656	82	R(*)VNALK(*)NLQVK(*)	K6	95.66	107.29	0.9887640	0.974025	1.011764	0.9915182
Nap1l1	P28656	116	YAVLYQPLFDK(*)R(*)	K11	44.61	1000	1.0561797	1.233766	0.788235	1.0260604
Nap1l1	P28656	116	K(*)YAVLYQPLFDK(*)R(*)	K12	43.85	127.06	0.9775280	-	0.870588	0.9240582
Nap1l1	P28656	266	GK(*)NVTLK(*)	K2	20.83	119.54	1.0561797	-	1.176470	1.1163252
Nap1l1	P28656	271	NVTLK(*)TIK(*)	K5	8.54	32.69	-	-	1.329411765	
Atxn10	P28658	384	LIGNLC(+57.02)YK(*)NK(*)	K8	85.73	24.32	-	0.784810	0.888888	0.8368495
Atxn10	P28658	371	AEGDIEHMTEGFK(*)SHLIR(*)	K13	71.26	1000	1.1052631	0.924050	1.377777	1.1356972
Nckap1	P28660	501	LQAYTSVSK(*)ASLSLADHR(*)	K9	29.48	1000	1.5384615	0.986842	1.204545	1.243283
Kif2a	P28740	101	TVAAVK(*)NDPPPR(*)	K6	67.01	1000	0.9038461	0.598039	0.744444	0.7487766
Kif2a	P28740	482	LEGAEINK(*)SLLALK(*)	K8	38.03	104.62	-	-	0.844444444	
Pabpc1	P29341	104	SGVGNIFIK(*)NLDK(*)	K9	93.52	72.09	0.8958333	0.925	0.989247	0.9366935
Pabpc1	P29341	231	SK(*)GFGFVSFER(*)	K2	85.75	1000	0.7916666	0.775	0.903225	0.8232975
Pabpc1	P29341	299	VK(*)NLDDGIDDER(*)	K2	83.32	1000	0.8541666	0.8375	0.903225	0.8649642
Pabpc1	P29341	606	SK(*)VDEAVAVLQAHQAK(*)	K2	70.46	174.34	0.7916666	0.825	0.870967	0.8292115
Pabpc1	P29341	333	SK(*)GFGFVC(+57.02)FSSPEEATK(*)	K2	67.26	238.57	-	-	0.849462366	
Pabpc1	P29341	108	NLDK(*)SIDNK(*)	K4	62.52	33.54	0.8958333	-	-	
Pabpc1	P29341	104	IFIK(*)NLDK(*)	K4	36.04	68.07	-	0.9	-	
Pabpc1	P29341	138	VVC(+57.02)DENGSK(*)GYGFVHFET	K9	32.9	1000	-	-	1.784946237	
Pabpc1	P29341	196	IK(*)NFGEDMDDER(*)	K2	32.4	1000	0.9583333	-	1.709677	1.3340054
Pabpc1	P29341	104	K(*)SGVGNIFIK(*)NLDK(*)	K10	11.86	49.79	1.4479166	-	-	
Vcam1	P29533	171	QEFSSEEMTK(*)SLETK(*)	K10	38.46	32.32	-	-	1.285714286	
Oat	P29758	66	GK(*)GIYMWDVEGR(*)	K2	79.87	1000	0.7961165	0.777777	1.021978	0.8652908
Oat	P29758	374	GK(*)GLLNAIVIR(*)	K2	65.85	1000	0.7766990	0.604938	-	0.6908187
Oat	P29758	392	DC(+57.02)DAWK(*)VC(+57.02)LR(	K6	41.34	1000	-	1.518518	-	
Vtn	P29788	222	TYLFK(*)GSQYWR(*)	K5	15.55	1000	-	0.259259	0.625	0.4421296
Tead1	P30051	370	LK(*)HLPEK(*)	K2	36.28	72.78	-	7.928571	-	
Cdk4	P30285	35	DPHSGHFVALK(*)SVR(*)	K11	73.79	1000	1.0543478	1.026315	0.922222	1.0009619
Ppic	P30412	83	GYGYK(*)GSIFHR(*)	K5	73.91	1000	1.0978260	0.854166	1.172413	1.0414688
Ppic	P30412	159	PTWLDGK(*)HVVFGK(*)	K7	60.04	134.33	1.1630434	0.6875	1.022988	0.957844
Ppic	P30412	159	TK(*)PTWLDGK(*)HVVFGK(*)	K9	56.28	55.13	0.8695652	0.614583	0.965517	0.8165553
Fkbp4	P30416	287	YK(*)QALLQYK(*)	K2	93.15	163.4	0.9270833	0.9	0.913043	0.9133756
Fkbp4	P30416	390	AAK(*)TQLAVC(+57.02)QQR(*)	K3	90.66	1000	0.8958333	0.8125	0.934782	0.8810386
Fkbp4	P30416	354	ALELDSNNEK(*)GLFR(*)	K10	85.19	1000	0.9895833	0.8625	0.891304	0.9144626

Fkbp4	P30416	222	GEHSIVYLK(*)PSYAFGSVGK(*)	K9	85.07	168.06	0.8854166	-	0.902173	0.8937953
Fkbp4	P30416	378	ADFQK(*)VLQLYPSNK(*)	K5	64.44	181.94	0.8333333	0.775	0.967391	0.8585749
Fkbp4	P30416	108	VGEVC(+57.02)HITC(+57.02)K(*)PE	K10	56.42	178.91	1.25	1.0125	1.032608	1.0983696
Hmgb2	P30681	50	WK(*)TMSAK(*)	K2	89.99	76.42	1.1931818	1.169014	1.035714	1.1326367
Hmgb2	P30681	114	IK(*)IEHPGLSIGDTAK(*)	K2	86.49	246.23	0.9659090	1.014084	0.904761	0.9615852
Hmgb2	P30681	12	GK(*)MSSYAFFVQTC(+57.02)R(*)	K2	83.49	1000	1.1931818	0.887323	1.023809	1.0347718
Hmgb2	P30681	12	GK(*)MSSYAFFVQTC(+57.02)R(*)EEI	K2	43.31	90.71	1.3181818	-	0.845238	1.08171
Hmgb2	P30681	3	GK(*)GDPNK(*)PR(*)	K2	24.99	115.97	0.8295454	-	-	
Ctnnd1	P30999	810	LVLINK(*)SGNR(*)	K6	79.25	1000	0.9387755	0.807692	1.011363	0.9192772
Ctnnd1	P30999	383	LDAVK(*)SNAAAYLQHLC(+57.02)YR	K5	60.57	1000	0.8061224	0.448717	0.977272	0.7440377
Ctnnd1	P30999	433	EVHLGAC(+57.02)GALK(*)NISFGR(*	K11	57.23	1000	0.9081632	0.820512	1.079545	0.9360738
Ctnnd1	P30999	433	K(*)EVHLGAC(+57.02)GALK(*)NISF(	K12	56.46	73.38	0.5612244	0.858974	0.795454	0.7385511
Ctnnd1	P30999	448	IAIK(*)NC(+57.02)DGVPALVR(*)	K4	56.41	1000	1.0816326	1.525641	-	1.3036368
Ctnnd1	P30999	749	ELIGK(*)HAIPNLVK(*)	K5	41.76	161.77	0.8265306	0.743589	0.943181	0.8377674
Ctnnd1	P30999	146	VVK(*)TMTTR(*)	K3	23.38	1000	-	1.076923	1.329545	1.2032343
Ctnnd1	P30999	146	K(*)VVK(*)TMTTR(*)	K4	10.36	68.31	1.0510204	-	-	
Hoxc10	P31257	195	TEHLESPQLGGK(*)VSFPETPK(*)	K12	65.02	131.34	-	-	-	
Rbpj	P31266	80	GDQTVLILHAK(*)VAQK(*)	K11	62.62	63.97	1.2386363	0.650602	0.863157	0.9174656
Rbpj	P31266	201	QSLK(*)NADLC(+57.02)IASGTK(*)	K4	30.09	112.13	4.1590909	6.891566	-	5.5253286
Rbpj	P31266	84	VAQK(*)SYGNEK(*)	K4	5.6	22.09	1.2045454	-	-	
Akt1	P31750	163	GTFGK(*)VILVK(*)	K5	35.42	87.95	0.6041666	1.513157	1.064516	1.0606136
Akt1	P31750	30	YFLLK(*)NDGTFIGYK(*)	K5	6.61	46.57	0.8854166	-	1.591397	1.2384073
Dbi	P31786	55	AK(*)WDSWNK(*)	K2	98.49	151.86	0.8723404	0.881578	1.229885	0.9946015
Dbi	P31786	17	LK(*)TQPTDEEMLFIYSHFK(*)	K2	69.07	276.92	0.8723404	1.052631	0.988505	0.9711593
Dbi	P31786	33	FK(*)QATVGDVNTDR(*)PGLLDLK(*)	K2	66.18	74.24	-	-	0.885057471	
Dbi	P31786	8	S(+42.01)QAEFDK(*)AAEEVK(*)R(*)	K7	58.38	108.94	-	0.789473	1.011494	0.900484
Dbi	P31786	51	PGLLDLK(*)GK(*)	K7	24.78	39.76	0.7446808	0.947368	1.022988	0.9050126
Ssb	P32067	404	DR(*)EEPASK(*)HK(*)	K8	87.75	45.01	0.9479166	0.848101	0.932584	0.9095341
Ssb	P32067	148	TLHK(*)TFK(*)	K4	73.7	44.44	0.2916666	-	0.606741	0.4492041
Ssb	P32067	340	FK(*)GSHVFTAAR(*)	K2	67.34	1000	0.9166666	0.759493	0.932584	0.8695815
Ssb	P32067	356	GK(*)GNR(*)PGYAGAPK(*)	K2	66.68	112.13	0.90625	0.797468	1.202247	0.9686552
Ssb	P32067	176	YK(*)DTNLLILFK(*)	K2	43.88	181.07	0.90625	-	0.764044	0.8351475
Ssb	P32067	228	ALEGK(*)MGC(+57.02)LLK(*)	K5	26.58	54.91	-	-	0.415730337	
Ssb	P32067	105	SPSR(*)PLPEVTDEYK(*)NDVK(*)	K14	22.11	25.11	0.7604166	1.025316	-	0.8928666

Ssb	P32067	303	VTWK(*)VLEGHAEK(*)	K4	8.14	107.73	-	0.493670	0.808988	0.6513298
Drg1	P32233	61	GGGGGGPGEGFDVAK(*)TGDAR(*)	K15	37.5	1000	0.5918367	0.987804	-	0.7898208
Serpinc1	P32261	124	AMTK(*)LGAC(+57.02)NDTLK(*)	K4	2.6	148.12	1.5	0	0.4	0.6333333
Kras	P32883	88	AINNTK(*)SFEDIHHYR(*)	K6	45.52	1000	-	-	1.0875	
Wars	P32921	260	IQK(*)HVTFNQVK(*)	K3	85.29	139.75	0.8541666	0.740259	0.945652	0.8466929
Wars	P32921	115	LIVQFGSSK(*)IDK(*)	K9	47.06	49.37	-	-	0.97826087	
Wars	P32921	55	MSYK(*)AAMGEEYK(*)	K4	19.75	135.81	-	-	1.760869565	
Kif4	P33174	884	ILVSK(*)LESSLNQSK(*)	K5	39.14	142.42	-	-	0.719101124	
Kif4	P33174	893	LESSLNQSK(*)ASC(+57.02)IDVQK(*)	K9	35.89	83.15	-	-	1.134831461	
Pola1	P33609	665	VPYWSK(*)IGR(*)	K6	38.17	1000	0.8333333	-	0.843373	0.8383534
Pola1	P33609	385	THVSC(+57.02)C(+57.02)VMVK(*)N	K10	33.74	1000	2.09375	-	1.746987	1.920369
Pola1	P33609	277	VDEK(*)PVTAK(*)TWDQETEPVER(*)	K9	22.08	38.19	1.5104166	1.267605	-	1.3890112
Pola2	P33611	500	VLK(*)HILTQR(*)	K3	76.39	1000	1.1927710	0.935897	0.844036	0.9909017
Ranbp1	P34022	134	AWVWNTHADFADEC(+57.02)PK(*)	K16	60.27	1000	-	-	0.795454545	
Ranbp1	P34022	111	IC(+57.02)ANHYITPMMELK(*)PNAG	K14	24.69	1000	-	-	1.897727273	
Ranbp1	P34022	97	TLK(*)IC(+57.02)ANHYITPMMELK(*	K3	12.22	100.98	-	-	1.715909091	
Ranbp1	P34022	154	TK(*)FEEC(+57.02)R(*)	K2	7	1000	-	3.311688	-	
Ptk2	P34152	78	VK(*)HVAC(+57.02)YGFR(*)	K2	31.71	1000	1.1274509	1.064102	1.011235	1.0675965
Mif	P34884	78	NYSK(*)LLC(+57.02)GLLSDR(*)	K4	96.62	1000	0.9285714	0.917808	0.946236	0.9308721
Mif	P34884	67	SGTNDPC(+57.02)ALC(+57.02)SLHS	K17	37.78	1000	1	1.931506	-	1.4657534
Usp4	P35123	232	QSLQSK(*)SSTAPSR(*)	K6	14.62	1000	-	-	0.924731183	
Ptpn11	P35235	280	YK(*)NILPFDHTR(*)	K2	64.43	1000	-	-	0.742268041	
Ptpn11	P35235	35	PSK(*)SNPGDFTLSVR(*)	K3	37.78	1000	-	0.891566	0.783505	0.8375357
Ptpn11	P35235	492	GVDC(+57.02)DIDVPK(*)TIQMVR(*	K10	36.67	1000	1.1313131	1.012048	0.814432	0.9859314
Ptpn11	P35235	131	GK(*)HGSFLVR(*)	K2	17.79	1000	2.7777777	2.795180	2.721649	2.7648693
Rab5c	P35278	184	K(*)LPK(*)NEPQNAAGAPGR(*)	K4	79.87	26.52	0.7021276	0.727272	0.829787	0.7530625
Rab5c	P35278	34	LVLLGESAVGK(*)SSLVLR(*)	K11	65.82	1000	0.6595744	-	0.829787	0.7446809
Rab5c	P35278	135	IVIALAGNK(*)ADLASK(*)	K9	51.16	102.87	0.5638297	0.961038	1	0.8416229
Rab5c	P35278	184	LPK(*)NEPQNAAGAPGR(*)	K3	19.45	1000	-	94.74025	1.095744	47.918002
Rab21	P35282	45	FNDK(*)HITTLQASFLTK(*)	K4	79.61	143.73	0.8105263	0.741176	0.860465	0.804056
Rab21	P35282	30	VVLLGEGC(+57.02)VGK(*)TSLVLR(*	K11	57.15	1000	0.8842105	0.529411	1.058139	0.8239206
Rab12	P35283	54	GVGK(*)TSLMER(*)	K4	6.33	1000	2.0865384	1.031578	1.522935	1.5470177
Rab24	P35290	20	EYVGK(*)TSLVER(*)	K5	34.37	1000	1.0921052	0.785046	1.508196	1.1284496
Rab18	P35293	48	VK(*)TISVDGNK(*)	K2	84.67	170.86	0.7352941	0.688311	0.931818	0.7851413

Rab18	P35293	21	ILIIGESGVGK(*)SSLLLR(*)	K11	46.46	1000	-	-	1.045454545	
Thbs1	P35441	1141	IMADSGPIYDK(*)TYAGGR(*)	K11	29.12	1000	0.3888888	-	0.397727	0.3933081
Thbs1	P35441	50	LVK(*)GQDLSSPAFR(*)	K3	23.34	1000	-	-	0.829545455	
Thbs1	P35441	1041	WK(*)QVTQSYWDTNPTR(*)	K2	5.46	1000	-	-	1	
Pdha1	P35486	313	SK(*)SDPIMLLK(*)	K2	70.5	131.72	0.8064516	0.772151	0.577777	0.7187938
Fbl	P35550	90	GNQSGK(*)NVMVEPHR(*)	K6	77.66	1000	0.8125	0.729411	0.766666	0.7695261
Fbl	P35550	108	GK(*)EDALVTK(*)	K2	65.64	184.45	0.7395833	0.682352	0.966666	0.796201
Fbl	P35550	149	SK(*)LAAAILGGVDQIHIK(*)PGAK(*)	K2	54.28	168.07	-	-	0.733333333	
Fbl	P35550	115	GK(*)EDALVTK(*)NLVPGESVYGEK(*)	K9	48.3	44.44	0.75	0.764705	0.977777	0.8308279
Fbl	P35550	298	MK(*)PQEQLTLEPYER(*)	K2	32.79	1000	-	1.094117	1.5	1.2970588
Fbl	P35550	90	R(*)GNQSGK(*)NVMVEPHR(*)	K7	22.26	1000	-	1.388235	1.444444	1.4163399
Canx	P35564	171	LLSK(*)TAELSLDQFHDK(*)	K4	97.04	240	0.875	0.909090	0.944444	0.9095118
Canx	P35564	218	TGVYEEK(*)HAK(*)	K7	89.92	49.37	0.8854166	-	0.877777	0.8815972
Canx	P35564	128	AK(*)HHAISAK(*)	K2	78.7	134.13	1.09375	0.779220	1.255555	1.0428421
Canx	P35564	228	R(*)PDADLK(*)TYFTDK(*)	K7	66.15	123.86	0.8333333	0.961038	0.855555	0.8833093
Canx	P35564	211	NPK(*)TGVYEEK(*)	K3	65.54	140.21	0.78125	0.753246	-	0.7672484
Canx	P35564	104	YDGK(*)WEVDEMK(*)	K4	64.13	161.52	-	0.610389	0.922222	0.7663059
Canx	P35564	48	SK(*)SDASTPPSPK(*)	K2	50.29	154.29	0.6041666	-	0.866666	0.7354167
Canx	P35564	211	HK(*)NPK(*)TGVYEEK(*)	K5	46.55	29.32	0.8541666	0.831168	0.833333	0.8395563
Canx	P35564	228	R(*)PDADLK(*)TYFTDK(*)K(*)	K7	28.15	95.35	0.9583333	1.181818	0.788888	0.9763468
Canx	P35564	114	ETK(*)LPGDK(*)	K3	8.57	41.35	0.71875	-	-	
Ap1m1	P35585	400	IIEK(*)SGYQALPWVR(*)	K4	56.52	1000	0.7345132	0.661764	0.944444	0.7802408
Ap1m1	P35585	237	SVELEDVK(*)FHQC(+57.02)VR(*)	K8	49.7	1000	0.5575221	-	0.933333	0.7454277
Ap1m1	P35585	229	SK(*)SVELEDVK(*)	K2	39.36	124.34	0.9469026	1.220588	-	1.0837454
Ap1m1	P35585	61	FMWIK(*)HNNLYLVATSK(*)	K5	13.32	71.87	-	-	1.244444444	
Ap1m1	P35585	326	FK(*)TTVGSVK(*)	K2	9.9	52.22	-	-	1.411111111	
Rfc1	P35601	726	GGIQELIGLIK(*)HTK(*)	K11	42.97	26.52	0.8557692	-	0.752688	0.8042287
Prdx1	P35700	136	GLFIIDDK(*)GILR(*)	K8	84.81	1000	0.9354838	0.716049	1.011627	0.8877204
Prdx1	P35700	178	HGEVC(+57.02)PAGWK(*)PGSDTIK	K10	51.16	20.85	1.7311827	1.111111	0.953488	1.2652608
Prdx1	P35700	192	SK(*)EYFSK(*)	K2	43.9	99.73	-	-	0.930232558	
Prdx1	P35700	168	LVQAFQFTDK(*)HGEVC(+57.02)PAG	K10	28.15	72.22	1.0860215	0.864197	1.825581	1.2586001
Prdx1	P35700	168	QFTDK(*)HGEVC(+57.02)PAGWK(*)	K5	24.56	168.62	1.1075268	-	1.034883	1.0712053
Cd81	P35762	148	AVVK(*)TFHETLNC(+57.02)C(+57.0	K4	44.39	1000	-	-	1.179775281	
Fmr1	P35922	83	EPC(+57.02)C(+57.02)WWLAK(*)VI	K9	50.76	1000	-	-	1.034090909	

Fmr1	P35922	310	LIQEIVDK(*)SGVVR(*)	K8	42.58	1000	-	-	0.795454545	
Fmr1	P35922	195	TK(*)LSLILR(*)	K2	39.65	1000	-	-	0.636363636	
Fmr1	P35922	371	ENTHFSQPNSTK(*)VQR(*)	K12	26.95	1000	-	-	1.159090909	
Rpl12	P35979	54	ATGDWK(*)GLR(*)	K6	95.3	1000	0.9230769	0.630952	0.863636:	0.8058886
Rpl12	P35979	99	NIK(*)HSGNITFDEIVNIAR(*)	K3	61.6	1000	1.2857142	-	1.056818:	1.1712662
Rpl18	P35980	99	LK(*)VC(+57.02)ALR(*)	K2	90.47	1000	0.7634408	0.690476	0.90625	0.7867224
Rpl18	P35980	19	EPK(*)SQDIYLR(*)	K3	87.43	1000	0.7849462	0.726190	0.916666(	0.8092678
Rpl18	P35980	78	ENK(*)TAVVVGTVTDDVR(*)	K3	72.95	1000	0.7096774	0.666666	0.875	0.750448
Rpl18	P35980	164	APGTPHSHTK(*)PYVR(*)	K10	70.12	1000	0.9677419	0.726190	0.802083:	0.8320052
Rpl18	P35980	132	ILTFDQLALESPK(*)GR(*)	K13	47.04	1000	-	0.619047	0.947916(	0.7834821
Rpl18	P35980	19	R(*)K(*)EPK(*)SQDIYLR(*)	K5	46.47	22.69	-	0.702380	0.9375	0.8199405
Rpl18	P35980	164	GTPHSHTK(*)PYVR(*)	K8	7.67	1000	-	-	1.5	
Ppm1b	P36993	314	DSELDK(*)HLESR(*)	K6	58.46	1000	0.6694915	-	0.807228!	0.7383602
Por	P37040	176	FAVFGLGNK(*)TYEHFNAMGK(*)	K9	61.17	25.74	-	-	0.83908046	
Por	P37040	481	VHPNSVHIC(+57.02)AVAVEYEAK(*)!	K18	35.15	1000	0.4949494	-	1.655172‹	1.075061
Tagln	P37804	17	EVQSK(*)IEK(*)	K5	87.11	47.36	0.9807692	1.012195	1.020618!	1.0045276
Tagln	P37804	172	DFTDSQLQEGK(*)HVIGLQMGSNR(*)	K11	69.84	1000	1.1923076	0.951219	0.917525:	1.020351
Tagln	P37804	57	LK(*)NGVILSK(*)	K2	29.55	118.11	-	-	1.164948454	
Tagln	P37804	75	LVNSLYPEGSK(*)PVK(*)	K11	26.88	24.24	0.7980769	0.731707	0.618556:	0.7161136
Fbln2	P37889	976	C(+57.02)SC(+57.02)AAGFLLAADGI	K14	84.08	1000	1.0326086	0.913043	0.968421(	0.9713577
Fbln2	P37889	976	LLAADGK(*)HC(+57.02)EDVNEC(+5	K7	55.71	1000	0.9456521	1.695652	-	1.3206522
Lig1	P37913	225	GAK(*)TLSSFFTPR(*)	K3	77.12	1000	0.8260869	0.586666	0.910112:	0.7742887
Lig1	P37913	280	NNYHPIEDAC(+57.02)WK(*)HGQK(	K12	40.02	34.3	0.9021739	-	1.235955(	1.0690645
Lig1	P37913	239	K(*)PAVK(*)TEVK(*)	K5	18.84	67.69	0.8369565	0.693333	0.786516!	0.7722689
Hsf1	P38532	162	LLAMK(*)HENEALWR(*)	K5	30.55	1000	-	-	-	
Hspa9	P38647	138	DTK(*)NVPFK(*)	K3	89.06	95.62	0.8842105	0.810126	0.943820:	0.8793858
Hspa9	P38647	563	DDIENMVK(*)NAEK(*)	K8	78.32	82.24	0.8421052	0.936708	0.910112:	0.8963088
Hspa9	P38647	175	MK(*)ETAENYLGHTAK(*)	K2	76.89	255.02	0.9473684	0.860759	1.011235!	0.939788
Hspa9	P38647	394	MPK(*)VQQTVQDLFGR(*)	K3	61.26	1000	0.9473684	0.569620	1.258426!	0.9251385
Hspa9	P38647	187	ETAENYLGHTAK(*)NAVITVPAYFNDS(	K12	60.69	1000	1.1052631	-	1.056179:	1.0807215
Hspa9	P38647	121	QAVTNPNNTFYATK(*)R(*)	K14	25.41	1000	-	-	0.651685393	
Hspa9	P38647	300	ETGVDLTK(*)DNMALQR(*)	K8	14.87	1000	-	-	1.719101124	
Dnm1	P39053	393	EISYAIK(*)NIHGIR(*)	K7	66.87	1000	0.9032258	0.96	1.149425:	1.004217
Dnm2	P39054	393	EISYAIK(*)NIHGVR(*)	K7	80.11	1000	0.9651162	0.922077	0.977777:	0.9549907

Dnm2	P39054	87	TEYAEFLHC(+57.02)K(*)SK(*)	K10	55.62	33.18	0.7906976	0.948051	-	0.8693748
Dnm2	P39054	77	SK(*)TEYAEFLHC(+57.02)K(*)	K2	14.01	207.31	-	1.285714	1.488888	1.3873016
Dnm2	P39054	677	DLMPK(*)TIMHLMINNTK(*)	K5	8	41.33	-	-	1.277777778	
Tjp1	P39447	9	AAAAK(*)STAMEETAIWEQHTVTLHR(	K5	38.82	1000	1.2022471	1.538461	0.901098	1.2139359
Tjp1	P39447	702	QIIDQDK(*)HALLDVTPNAVDR(*)	K7	33.18	1000	1.4494382	-	0.835164	1.1423015
Fen1	P39749	250	AVDLIQK(*)HK(*)	K7	90.98	30.36	0.9354838	0.831168	0.956043	0.9075656
Fen1	P39749	252	HK(*)SIEEIVR(*)	K2	59.84	1000	1.0967741	0.740259	0.989010	0.942015
Fen1	P39749	24	ENDIK(*)SYFGR(*)	K5	39.07	1000	1.1720430	1.857142	1	1.343062
Timp3	P39876	148	IK(*)SC(+57.02)YYLPC(+57.02)FVTS	K2	77.66	223.58	1.21	1.658227	0.980582	1.2829368
Timp3	P39876	188	QK(*)GGYC(+57.02)SWYR(*)	K2	35.5	1000	1.15	1.303797	0.883495	1.1124309
Cap1	P40124	411	VPTISINK(*)TDGC(+57.02)HAYLSK(*	K8	93.95	150.82	1.0760869	-	0.934065	1.0050764
Cap1	P40124	421	TDGC(+57.02)HAYLSK(*)NSLDC(+57	K10	84.61	267.5	1.0652173	0.815789	0.967032	0.9493466
Cap1	P40124	70	EIGGDVQK(*)HAEMVHTGLK(*)	K8	82.84	92.69	1.0543478	0.881578	0.956043	0.9639902
Cap1	P40124	271	SALFAQINQGESITHALK(*)HVSDDMK	K18	62.21	38.97	1.1956521	1.210526	0.978021	1.1280668
Cap1	P40124	208	EFHTTGLAWSK(*)TGPVAK(*)	K11	56.56	41.94	1.1956521	0.802631	0.901098	0.9664609
Cap1	P40124	197	IK(*)EFHTTGLAWSK(*)	K2	30.86	101.16	-	-	1.120879121	
Tkt	P40142	144	YFDK(*)ASYR(*)	K4	93.62	1000	1.0105263	1.103896	0.923913	1.0127785
Tkt	P40142	6	M(+42.01)EGYHK(*)PDQQK(*)	K6	92.19	95.62	0.9894736	0.896103	0.967391	0.9509896
Tkt	P40142	232	AFGQAK(*)HQPTAIIAK(*)	K6	78.54	218.32	0.9473684	0.870129	0.858695	0.8920646
Tkt	P40142	241	HQPTAIIAK(*)TFK(*)	K9	69.9	53.53	0.8631578	0.844155	0.913043	0.8734524
Tkt	P40142	465	AVELAANTK(*)GIC(+57.02)FIR(*)	K9	40.91	1000	0.8210526	-	0.815217	0.818135
Tkt	P40142	260	EAWHGK(*)PLPK(*)	K6	34.29	31.27	0.7263157	-	1.054347	0.8903318
Cxcl12	P40224	48	HLK(*)ILNTPNC(+57.02)ALQIVAR(*)	K3	16.76	1000	-	-	1.240963855	
Vps26a	P40336	288	YFK(*)QQEIILWR(*)	K3	51.25	1000	-	-	1.226190476	
Vps26a	P40336	38	VEK(*)HYLFYDGESVSGK(*)	K3	48.17	187.28	1.1978021	0.987179	1.190476	1.1251526
Rpl28	P41105	84	TTINK(*)NAR(*)	K5	79.42	1000	-	0.888888	0.903225	0.8960573
Cd63	P41731	138	TATILDK(*)LQK(*)	K7	20.32	37.54	1.2567567	-	1.223529	1.2401431
Septin2	P42208	174	AIHNK(*)VNIVPVIAK(*)	K5	83.36	161.68	0.9673913	0.846153	0.836956	0.8835006
Septin2	P42208	183	VNIVPVIAK(*)ADTLTLK(*)	K9	49.61	49.06	1.0217391	-	1.108695	1.0652174
Stat3	P42227	348	TGVQFTTK(*)VR(*)	K8	53.8	1000	0.9791666	0.8	0.900990	0.8933856
Stat3	P42227	631	DISGK(*)TQIQSVEPYTK(*)	K5	51.82	203.1	-	-	0.841584158	
Pura	P42669	272	FGHTFC(+57.02)K(*)YSEEMK(*)	K7	61.26	133.21	0.5625	0.743902	0.833333	0.7132453
Pura	P42669	143	ALK(*)SEFLVR(*)	K3	46.55	1000	1.0833333	0.792682	1.154761	1.0102594
Cct8	P42932	466	ANEVISK(*)LYSVHQEGNK(*)	K7	89.38	89.7	0.8979591	0.875	0.893617	0.8888587

Cct8	P42932	152	AHEILPELVC(+57.02)C(+57.02)SAK(	K14	86.35	1000	0.7857142	0.7	0.968085:	0.8179331
Cct8	P42932	318	LNSK(*)WDLR(*)	K4	76.23	1000	0.8469387	0.825	0.946808!	0.8729158
Cct8	P42932	20	DGAK(*)HFSGLEEAVYR(*)	K4	73.42	1000	1.0918367	0.9875	0.978723	1.0193534
Cct8	P42932	260	GTVLIK(*)TAEELMNFSK(*)	K6	68.31	228.69	-	-	0.765957447	
Cct8	P42932	459	ALAENSGVK(*)ANEVISK(*)	K9	63.51	142.27	0.8775510	0.8875	0.904255:	0.8897688
Cct8	P42932	171	TSIMSK(*)QYGSETFLAK(*)	K6	61.34	203.9	1.0204081	0.8625	0.946808!	0.9432389
Cct8	P42932	326	LC(+57.02)K(*)TVGATALPK(*)	K3	35.16	161.52	-	1.1875	0.808510(	0.9980053
Nr2f2	P43135	89	SSGK(*)HYGQFTC(+57.02)EGC(+57.	K4	38.23	112.13	1.3291139	0.259259	0.833333:	0.8072355
Nr2f2	P43135	100	HYGQFTC(+57.02)EGC(+57.02)K(*)S	K11	33.84	42.89	-	-	0.427083333	
Msh2	P43247	59	EVFK(*)TQGVIK(*)	K4	52.03	92.15	0.9550561	0.686746	0.829545	0.8237829
Msh2	P43247	918	AEVVAK(*)NNSFVNEIISR(*)	K6	50.75	1000	1.6067415	1.289156	2.068181	1.6546933
Msh2	P43247	73	YMGPAGSK(*)TLQSVVLSK(*)	K8	25.93	140.79	3.7640449	2.277108	2.204545	2.7485663
Msh2	P43247	104	VEVYK(*)NK(*)	K5	11.32	30.83	-	-	0.931818182	
H1-4	P43274	97	GTLVQTK(*)GTGASGSFK(*)	K7	71	184.08	-	2.043478	1.259259	1.6513688
H1-4	P43274	46	TSGPPVSELITK(*)AVAASK(*)	K12	56.11	95.51	-	-	0.950617284	
H1-4	P43274	192	AK(*)TVK(*)PK(*)	K2	15.66	77.24	1.1363636	-	-	
H1-1	P43275	121	AITTK(*)VSVK(*)	K5	98.67	82.24	1.1208791	1.073529	1.0875	1.0939695
H1-1	P43275	87	LGLK(*)SLVNK(*)	K4	86.45	96.85	1.1098901	1.029411	1.0625	1.0672673
H1-1	P43275	77	SLAAAGYDVEK(*)NNSR(*)	K11	78.84	1000	1.0659340	1.117647	1.05	1.0778604
H1-1	P43275	99	GTLVQTK(*)GTGAAGSFK(*)	K7	57.49	120.76	0.7142857	-	0.95	0.8321429
H1-1	P43275	92	SLVNK(*)GTLVQTK(*)	K5	25.69	110.43	0.8791208	1.235294	1.2	1.104805
H1-5	P43276	46	ATGPPVSELITK(*)AVSASK(*)	K12	76.67	33.66	0.9444444	0.855072	0.928571	0.9093628
H1-5	P43276	75	ALAAGGYDVEK(*)NNSR(*)	K11	55.25	1000	1.1888888	1.028985	1.142857	1.1202438
H1-5	P43276	46	K(*)ATGPPVSELITK(*)AVSASK(*)	K13	32.13	51.99	2.0777777	-	0.928571	1.5031746
Dck	P43346	211	LHYK(*)HESWLLHR(*)	K4	72.39	1000	0.9775280	-	0.891304:	0.9344162
Itgav	P43406	553	SPVHSK(*)TMTVFR(*)	K6	54.76	1000	0.6862745	0.746835	0.525252!	0.6527875
Sdc2	P43407	135	STDVYTEK(*)HSDNLFK(*)	K8	24.92	85.08	-	-	-	
Akr1b1	P45376	243	YNK(*)TTAQVLIR(*)	K3	96.18	1000	0.8823529	0.780487	0.928571	0.8638041
Akr1b1	P45376	86	LWC(+57.02)TFHDK(*)SMVK(*)	K8	88.29	97.69	1.1078431	0.878048	1.030612	1.0055014
Akr1b1	P45376	263	NLVVIPK(*)SVTPVR(*)	K7	82.62	1000	0.6960784	0.634146	0.816326!	0.7155171
Akr1b1	P45376	179	YK(*)PAVNQIEC(+57.02)HPYLTQEK(	K2	79.81	129.22	1.2549019	1.146341	1.010204(	1.1371492
Akr1b1	P45376	78	IVSK(*)LWC(+57.02)TFHDK(*)	K4	78.62	192.66	0.8725490	0.719512	0.938775!	0.8436122
Akr1b1	P45376	306	VC(+57.02)ALMSC(+57.02)AK(*)HK	K9	72.94	26.31	1.6568627	1.451219	1.581632(	1.5632383
Akr1b8	P45377	263	NVVVIPK(*)SVTPSR(*)	K7	68.02	1000	0.7777777	0.903614	0.785714	0.8223688

Akr1b8	P45377	22	MPIVGLGTWK(*)SPPNQVK(*)	K10	39.23	50.34	-	0.722891	0.806122	0.764507
Crebbp	P45481	1598	TNK(*)NK(*)SSISR(*)	K5	92.56	1000	0.6222222	0.554687	-	0.5884549
Crebbp	P45481	1596	TNK(*)NK(*)SSISR(*)	K3	92.56	1000	0.6222222	0.554687	-	0.5884549
Crebbp	P45481	1217	TLC(+57.02)C(+57.02)YGK(*)QLC(+5	K7	86.47	1000	0.6518518	0.507812	-	0.5798322
Crebbp	P45481	1712	FVYTC(+57.02)NEC(+57.02)K(*)HH\	K9	72.13	1000	0.6888888	0.601562	-	0.6452257
Crebbp	P45481	1712	YTC(+57.02)NEC(+57.02)K(*)HHVET	K7	68.83	1000	0.6296296	0.515625	-	0.5726273
Crebbp	P45481	1217	K(*)YEFSPQTLC(+57.02)C(+57.02)Y(	K14	63.25	163.91	0.3185185	0.640625	-	0.4795718
Crebbp	P45481	67	HK(*)QLSELLR(*)	K2	55.38	1000	0.5851851	0.53125	-	0.5582176
Crebbp	P45481	1628	LYATM(+15.99)EK(*)HK(*)	K7	49.72	27.16	0.8592592	0.671875	-	0.7655671
Crebbp	P45481	1763	MVK(*)WGLGLDDEGSSQGEPQSK(*)	K21	29.19	1000	0.7925925	-	-	
Crebbp	P45481	1745	MVK(*)WGLGLDDEGSSQGEPQSK(*)	K3	29.19	1000	0.7925925	-	-	
Crebbp	P45481	119	GK(*)SPLNQGDSSTPNLPK(*)	K2	25.59	170.25	0.5259259	1.101562	-	0.8137442
Crebbp	P45481	1217	GK(*)QLC(+57.02)TIPR(*)	K2	24.08	1000	1.0074074	1.21875	-	1.1130787
Crebbp	P45481	1587	GDSK(*)NAK(*)K(*)K(*)	K7	22.25	39.76	0.4074074	0.367187	-	0.3872975
Crebbp	P45481	1584	GDSK(*)NAK(*)K(*)K(*)	K4	22.25	127.18	0.4074074	0.367187	-	0.3872975
Crebbp	P45481	419	QIISHWK(*)NC(+57.02)TR(*)	K7	17.24	1000	-	0.6875	-	
Crebbp	P45481	1598	NK(*)SSISR(*)	K2	13.13	1000	-	-	-	
Crebbp	P45481	388	TMK(*)NVLNHMTHC(+57.02)QAGK(	K3	12.1	39.02	0.8518518	-	-	
Crebbp	P45481	1745	SHTHK(*)MVK(*)WGLGLDDEGSSQG	K8	8.07	40.42	-	0.90625	-	
Crebbp	P45481	1742	SHTHK(*)MVK(*)WGLGLDDEGSSQG	K5	8.07	34.05	-	0.90625	-	
Crebbp	P45481	1712	TC(+57.02)NEC(+57.02)K(*)HHVETI	K6	6.12	1000	-	-	-	
Cfl2	P45591	132	FTGIK(*)HEWQVNGLDDIK(*)	K5	19.09	85.14	-	-	1.542553191	
Rangap1	P46061	526	LLIHMGLLK(*)SEDK(*)	K9	91.45	97.69	0.8181818	0.911392	0.840425	0.8566666
Rangap1	P46061	477	LK(*)VASVFR(*)	K2	36.36	1000	-	1.063291	1.085106	1.0741988
Vps4b	P46467	217	LVK(*)NLFQLAR(*)	K3	57.94	1000	0.8333333	0.805194	1.118279	0.9189359
Vps4b	P46467	180	GILLFGPPGTGK(*)SYLAK(*)	K12	42.28	51	1.1888888	0.844155	0.935483	0.9895095
Psmc2	P46471	222	GVLLFGPPGTGK(*)TLC(+57.02)AR(*	K12	71.2	1000	-	0.777777	0.576086	0.6769324
Psmc2	P46471	356	THIFK(*)IHAR(*)	K5	47.85	1000	1	0.814814	0.891304	0.9020397
Rab11b	P46638	24	VVLIGDSGVGK(*)SNLLSR(*)	K11	93.51	1000	0.90625	0.839080	1	0.9151102
Rab11b	P46638	125	DHADSNIVIMLVGNK(*)SDLR(*)	K15	42.83	1000	0.8854166	-	0.965909	0.9256629
Rab11b	P46638	125	LVGNK(*)SDLR(*)	K5	6.17	1000	-	-	1.647727273	
Adss2	P46664	447	WIGVGK(*)SR(*)	K6	85.61	1000	0.8229166	0.841463	0.913978	0.8594529
Adss2	P46664	397	LDGETIPHFPANQEVLNK(*)VEVQYK(	K18	18.65	53.33	-	-	0.806451613	
Nedd4	P46935	453	GK(*)TDSNDLGPLPPGWEER(*)	K2	37.54	1000	0.9148936	3.026666	0.561797	1.5011193

Nedd4	P46935	429	SYYVDHNSK(*)TTTWSK(*)	K9	27.25	55.13	1.0319148	1.226666	0.797752	1.0187781
Nedd4	P46935	445	SK(*)IPAHLR(*)	K2	9.73	1000	1.1276595	-	0.842696	0.9851781
Yap1	P46938	306	LK(*)QQELFR(*)	K2	30.22	1000	-	-	0.466666667	
Yap1	P46938	239	NK(*)TTSWLDPR(*)	K2	8.54	1000	-	1.815384	-	
Stt3a	P46978	188	AVK(*)TGSIYWAAK(*)	K3	30.14	101.1	-	-	0.727272727	
Tes	P47226	188	YK(*)SEALGVGDVK(*)	K2	71.17	177.91	0.7126436	-	0.894117	0.8033807
Tes	P47226	85	LK(*)SDGIPMYK(*)	K2	53.7	150.89	1.6436781	-	1.435294	1.5394861
Aldh2	P47738	430	FK(*)TIEEVVGR(*)	K2	80.93	1000	0.9494949	0.759493	0.977528	0.8955056
Aldh2	P47738	377	ILGYIK(*)SGQQEGAK(*)	K6	79.83	105.52	0.7979797	0.759493	0.910112	0.8225286
Aldh2	P47738	157	YYAGWADK(*)YHGK(*)	K8	72.65	48.12	0.8484848	0.708860	1.011235	0.8561939
Aldh2	P47738	513	TVTVK(*)VPQK(*)	K5	64.42	42.89	0.6868686	0.620253	0.786516	0.6978796
Aldh2	P47738	161	YHGK(*)TIPIDGDFFSYTR(*)	K4	58.64	1000	-	-	0.528089888	
Capza2	P47754	268	TK(*)IDWNK(*)	K2	62.6	105.53	0.90625	0.779069	0.93	0.8717733
Capza2	P47754	273	IDWNK(*)ILSYK(*)	K5	44.19	98.37	0.9791666	1.069767	0.89	0.9796447
Capza2	P47754	278	ILSYK(*)IGK(*)	K5	7.56	34.94	-	1.383720	1.01	1.1968605
Capzb	P47757	145	IK(*)GC(+57.02)WDSIHVVEVQEK(*)	K2	84	113.99	0.8080808	-	1.022471	0.9152764
Capzb	P47757	159	GC(+57.02)WDSIHVVEVQEK(*)SSGI	K14	58.67	1000	1.1414141	0.913043	1.146067	1.0668417
Capzb	P47757	57	VVGK(*)DYLLC(+57.02)DYNR(*)	K4	57.41	1000	1.5252525	1.086956	-	1.3061045
Gsr	P47791	324	GLNLNK(*)VGIQTDEK(*)	K6	45.15	171.49	1.0425531	0.730337	0.632653	0.8018478
Gsr	P47791	479	MGATK(*)ADFDNTVAIHPTSSEELVTL	K5	10.92	1000	-	-	1.244897959	
Gsr	P47791	415	YGK(*)DNVK(*)	K3	5.55	67.69	-	-	1.153061224	
Map2k4	P47809	52	LNFANPPVK(*)STAR(*)	K9	8.18	1000	-	0.647887	-	
Mapk14	P47811	79	HMK(*)HENVIGLLDVFTPAR(*)	K3	19.07	1000	1.3541666	-	-	
Gfpt1	P47856	554	LATELYHQK(*)SVLIMGR(*)	K9	45.18	1000	0.5567010	-	0.967741	0.7622215
Gfpt1	P47856	190	ALVFK(*)SVHFPGQAVGTR(*)	K5	40.81	1000	0.7938144	0.7875	1.010752	0.8640224
Rpl6	P47911	102	VLATVTK(*)TVGGDK(*)	K7	89.37	57.71	1.125	0.666666	0.822222	0.8712963
Rpl6	P47911	218	IPK(*)HLTDAYFK(*)	K3	86.15	171.6	0.8461538	0.756410	0.888888	0.8304843
Rpl6	P47911	208	FVIATSTK(*)VDISDVK(*)	K8	48.04	128.65	1.6346153	-	1.4	1.5173077
Rpl29	P47915	101	LAFIAHPK(*)LGK(*)	K8	50.95	49.37	0.9259259	0.92	1.047619	0.964515
Fosl2	P47930	104	PGVIK(*)TIGTTVGR(*)	K5	56.16	1000	0.6571428	0.343065	0.524193	0.508134
Rpl5	P47962	158	TTTGNK(*)VFGALK(*)	K6	96.66	121.92	0.8446601	0.747126	0.917525	0.8364375
Rpl5	P47962	27	EGK(*)TDYYAR(*)	K3	85.57	1000	0.8640776	0.816091	0.876288	0.8521528
Rpl5	P47962	228	IK(*)NNVTPDMMEEMYK(*)	K2	83.97	226.1	0.8737864	0.827586	0.969072	0.8901483
Rpl13	P47963	123	SK(*)LILFPR(*)	K2	92.5	1000	0.8854166	0.92	0.879120	0.8948458

Rpl13	P47963	11	NGMILK(*)PHFHK(*)	K6	47.87	52.68	1.125	0.786666	0.989010	0.9668926
Rpl36	P47964	13	YPMAVGLNK(*)GHK(*)	K9	58.93	62.26	0.9387755	0.883720	0.903225	0.9085741
Rpl36	P47964	19	VTK(*)NVSK(*)PR(*)	K3	32.32	104.08	0.9693877	0.825581	0.666666	0.8205453
Anxa5	P48036	95	LYDAYELK(*)HALK(*)	K8	87.85	88.98	0.9263157	0.961538	1.011363	0.966406
Anxa5	P48036	56	QEIAQEFK(*)TLFGR(*)	K8	48.11	1000	0.9578947	1.115384	0.772727	0.9486689
Anxa5	P48036	74	SELTGK(*)FEK(*)	K6	40.63	37.54	1.2842105	-	0.954545	1.119378
Anxa5	P48036	68	DLVDDLK(*)SELTGK(*)	K7	36.14	113.99	-	-	0.863636364	
Tbca	P48428	41	MK(*)AEDGENYAIK(*)	K2	40.04	184.95	1.0679611	0.682352	0.944444	0.8982529
Ppp3cb	P48453	56	VDVLK(*)NHLVK(*)	K5	27.81	52.68	-	1.158730	0.757763	0.9582471
Lmna	P48678	457	NK(*)SNEDQSMGNWQIR(*)	K2	74.04	1000	0.8453608	0.858974	1.022727	0.9090208
Lmna	P48678	108	LQLELSK(*)VR(*)	K7	51.42	1000	1.0206185	1.448717	-	1.2346683
Lmna	P48678	208	EELDFQK(*)NIYSEELR(*)	K7	47.28	1000	0.7319587	2.089743	0.943181	1.2549614
Hspa4l	P48722	53	AIGNAAK(*)SQIVTNVR(*)	K7	49.67	1000	1.0449438	0.779069	0.902173	0.9087292
Hspa4l	P48722	430	SK(*)VITFHK(*)	K2	26.56	98.75	1.0561797	-	-	
Hspa4l	P48722	578	IK(*)SIDLPIQSSLYR(*)	K2	12.43	1000	-	-	1.130434783	
Pcnt	P48725	1931	SASGQAPLWAVAPSAGK(*)HHAER(*	K17	73.01	1000	1.0666666	0.662650	0.5125	0.7472724
Pcnt	P48725	271	IFQAK(*)HEAEVSLK(*)	K5	41.8	159.99	-	-	0.475	
Pcnt	P48725	2350	QLAQNNQLC(+57.02)VALK(*)HER(*	K13	41.55	1000	1.1066666	-	0.35	0.7283333
Pcnt	P48725	1249	QQLAALDK(*)HLR(*)	K8	23.37	1000	1.2533333	0.759036	0.74375	0.9187065
Cox7a2	P48771	46	LFQEDNGMPVHLK(*)GGASDALLYR(	K13	27.9	1000	-	-	0.46	
Slc25a4	P48962	166	IFK(*)SDGLK(*)	K3	95.2	55.6	0.6868686	0.7	0.84375	0.7435396
Slc25a4	P48962	96	YK(*)QIFLGGVDR(*)	K2	93.27	1000	1.3434343	0.875	0.802083	1.0068392
Slc25a4	P48962	147	LAADVGK(*)GSSQR(*)	K7	82.5	1000	0.8888888	0.7875	0.927083	0.8678241
Slc25a4	P48962	23	DFLAGGIAAAVSK(*)TAVAPIER(*)	K13	61.06	1000	0.8888888	-	0.791666	0.8402778
Slc25a4	P48962	163	EFNGLGDC(+57.02)LTK(*)IFK(*)	K11	49.93	55.63	0.9494949	-	1	0.9747475
Slc25a4	P48962	199	AAYFGVYDTAK(*)GMLPDPK(*)	K11	48.7	78.16	0.5151515	0.95	0.6875	0.7175505
Slc25a4	P48962	272	DEGANAFFK(*)GAWSNVLR(*)	K9	45.87	1000	1.1616161	-	0.802083	0.9818497
Slc25a4	P48962	23	VSK(*)TAVAPIER(*)	K3	11.94	1000	-	0.825	-	
Slc25a4	P48962	23	AVSK(*)TAVAPIER(*)	K4	9.67	1000	-	0.95	1.166666	1.0583333
Hnrnpa1	P49312	78	PHK(*)VDGR(*)	K3	93.34	1000	0.9157894	0.949367	1.033707	0.9662881
Hnrnpa1	P49312	3	S(+42.01)K(*)SESPK(*)EPEQLR(*)	K2	76.18	127.18	0.8947368	0.721518	0.876404	0.8308868
Hnrnpa1	P49312	183	ALSK(*)QEMASASSSQR(*)	K4	17.33	1000	-	-	1.539325843	
Hnrnpa1	P49312	166	IVIQK(*)YHTVNGHNC(+57.02)EVR(*	K5	8.71	1000	-	-	1.258426966	
Pcyt1a	P49586	341	WPFSGK(*)TSPSSSPASLSR(*)	K6	55.06	1000	-	0.567901	-	

Mcm4	P49717	219	SFSK(*)NLYR(*)	K4	83.81	1000	0.8947368	0.777777	0.824742	0.832419
Mcm4	P49717	412	SVYK(*)THIDVIHYR(*)	K4	77.21	1000	0.9368421	0.839506	0.917525	0.897958
Mcm4	P49717	857	ALADDDFLTVTGK(*)TVR(*)	K13	70.91	1000	-	0.901234	-	
Mcm4	P49717	751	FSNK(*)VEAIDVEEAK(*)	K4	65.61	176.42	0.8736842	1.395061	1.020618	1.0964548
Mcm4	P49717	751	FSNK(*)VEAIDVEEAK(*)R(*)	K4	37.54	143.6	-	-	1.020618557	
Mcm5	P49718	141	ILK(*)SDMMSHLVK(*)	K3	78.21	181.94	0.8817204	0.725	0.923913	0.8435445
Psma2	P49722	171	NYVNGK(*)TFLEK(*)	K6	69.51	77.53	1.2247191	1.173333	1	1.1326841
Psma2	P49722	70	VEPITK(*)HIGLVYSGMGPDYR(*)	K6	66.57	1000	-	0.36	0.860465	0.6102326
Cav1	P49817	57	DPK(*)HLNDDVVK(*)	K3	77.62	188.52	0.4301075	0.626666	0.772727	0.6098338
Cav1	P49817	57	EIDLVNR(*)DPK(*)HLNDDVVK(*)	K10	60.52	118.18	0.7956989	1.453333	0.897727	1.0489198
Cav1	P49817	47	QVYDAHTK(*)EIDLVNR(*)	K8	5.36	1000	-	-	2.318181818	
Impdh1	P50096	349	PQGTAVYK(*)VAEYAR(*)	K8	52.13	1000	-	-	0.801980198	
Ahcy	P50247	186	VPAINVNDSVTK(*)SK(*)	K12	81.27	20.41	0.7473684	0.636363	0.890109	0.7579473
Ahcy	P50247	188	SK(*)FDNLYGC(+57.02)R(*)	K2	57.12	1000	-	0.688311	-	
Ahcy	P50247	426	QAQYLGMPINGPFK(*)PDHYR(*)	K14	52.42	1000	0.6105263	-	1.120879	0.8657027
Ahcy	P50247	331	YWLK(*)NGR(*)	K4	27.94	1000	0.8736842	1.155844	0.835164	0.9548977
Gdi1	P50396	269	VVGVK(*)SEGEVAR(*)	K5	65.51	1000	0.7865168	0.948051	0.768292	0.8342872
Gdi1	P50396	309	IIC(+57.02)ILSHPIK(*)NTNDANSC(+	K10	10.76	1000	-	-	0.987804878	
Arsb	P50429	262	QSVHDPLQVPEEYMEPYGFIQDK(*)H	K23	11.94	1000	-	-	0.904761905	
Shmt1	P50431	380	VLEAC(+57.02)SIAC(+57.02)NK(*)N	K11	32.93	56.15	1.3764705	1.354430	-	1.3654505
Atp6v1a	P50516	139	WEFIPSK(*)NLR(*)	K7	62	1000	-	0.630952	0.84375	0.7373512
Atp6v1e1	P50518	52	LK(*)IMEYYEK(*)	K2	52.85	142.27	-	-	1.961538462	
Atp6v1e1	P50518	42	AEEEFNIEK(*)GR(*)	K9	18.43	1000	-	1.202531	-	
S100a11	P50543	47	MNTELAAFTK(*)NQK(*)	K10	65.82	24.24	0.8383838	0.7375	0.945652	0.840512
Acadvl	P50544	196	GILLYGTK(*)AQR(*)	K8	42.89	1000	0.6063829	-	0.842696	0.7245398
Pa2g4	P50580	93	NC(+57.02)VC(+57.02)HFSPLK(*)SD	K10	60.75	113.97	-	-	0.892473118	
Pa2g4	P50580	22	YK(*)MGGDIANR(*)	K2	10.36	1000	-	-	2	
Tspo	P50637	39	WYASLQK(*)PSWHPPR(*)	K7	60.57	1000	-	1.487179	0.855263	1.1712213
Cast	P51125	70	AASLGSSQPSR(*)PHVGEAATATK(*)V	K22	30.87	141.74	1.5681818	1.424657	0.701149	1.2313296
Rab7a	P51150	146	SK(*)NNIPYFETSAK(*)	K2	65.6	234.24	0.8247422	0.719512	0.884210	0.8094883
Rab7a	P51150	48	ATIGADFLTK(*)EVMVDDR(*)	K10	31	1000	0.7010309	-	2.263157	1.4820944
Acadl	P51174	322	AFGK(*)TVAHIQTVQHK(*)	K4	82.34	70.27	0.7676767	0.8	0.866666	0.8114478
Acadl	P51174	338	LAELK(*)THIC(+57.02)VTR(*)	K5	62.98	1000	0.8787878	0.626666	0.9	0.8018182
Rpl9	P51410	59	VDK(*)WWGNR(*)	K3	93.82	1000	0.8842105	0.775	1.011494	0.8902349

Plcb3	P51432	173	IPVK(*)NILK(*)	K4	24.25	52.86	0.8260869	-	1.328947:	1.0775172
Plcb3	P51432	219	FLNK(*)LC(+57.02)LR(*)PDIDK(*)	K4	8.53	50.04	0.8478260	-	-	
Hsd17b4	P51660	84	LVK(*)TALDTFGR(*)	K3	89.64	1000	1.1976744	0.939024	1.010638:	1.0491124
Hsd17b4	P51660	50	GIGK(*)GSSAADK(*)	K4	37.15	76.93	1.5116279	-	1.106382!	1.3090054
Gss	P51855	443	QGTTLVMNK(*)HVGHLLR(*)	K9	33.8	1000	-	0.89	1.024096:	0.9570482
Hdgf	P51859	39	IDEMPEAAVK(*)STANK(*)	K10	72.6	55.6	-	0.864864	0.963414(	0.9141397
Slc25a5	P51881	96	YK(*)QIFLGGVDK(*)	K2	97.85	208.83	0.8369565	0.942857	0.9	0.8932712
Slc25a5	P51881	268	DEGSK(*)AFFK(*)	K5	85.61	82.24	0.6086956	0.642857	0.666666(	0.6394065
Slc25a5	P51881	10	AK(*)DFLAGGVAAAISK(*)	K2	83.36	340.63	0.8695652	0.871428	0.944444	0.8951461
Slc25a5	P51881	272	AFFK(*)GAWSNVLR(*)	K4	76.74	1000	0.7173913	0.771428	0.866666(	0.7851622
Slc25a5	P51881	272	FK(*)GAWSNVLR(*)	K2	73.63	1000	0.5978260	0.985714	0.911111:	0.8315505
Slc25a5	P51881	96	YK(*)QIFLGGVDK(*)R(*)	K2	70.77	178.84	0.8804347	1	1	0.9601449
Slc25a5	P51881	147	LAADVGK(*)AGAER(*)	K7	61.94	1000	0.9347826	0.957142	1.288888	1.0602715
Slc25a5	P51881	23	DFLAGGVAAAISK(*)TAVAPIER(*)	K13	59.74	1000	0.9130434	1	1	0.9710145
Slc25a5	P51881	23	ISK(*)TAVAPIER(*)	K3	56.7	1000	1.0108695	0.785714	0.644444	0.8136761
Slc25a5	P51881	23	AISK(*)TAVAPIER(*)	K4	47.83	1000	-	1	-	
Slc25a5	P51881	163	GLGDC(+57.02)LVK(*)IYK(*)	K8	46.43	55.63	0.9673913	0.457142	0.822222:	0.7489188
Slc25a5	P51881	33	VK(*)LLLQVQHASK(*)	K2	29.24	58.09	-	-	1.688888889	
Slc25a5	P51881	155	EFK(*)GLGDC(+57.02)LVK(*)	K3	24.82	132.11	6.3478260	15.74285	5.855555!	9.3154129
Slc25a5	P51881	23	AAISK(*)TAVAPIER(*)	K5	22.48	1000	-	0.257142	-	
Slc25a5	P51881	199	AAYFGIYDTAK(*)GMLPDPK(*)	K11	22.45	78.16	0.8043478	-	1.377777	1.0910628
Slc25a5	P51881	23	GVAAAISK(*)TAVAPIER(*)	K8	9.14	1000	-	1.214285	-	
Kpna2	P52293	388	LVGVLSK(*)ADFK(*)	K7	89.18	88.98	0.8735632	0.802631	0.956043!	0.8774129
Usp10	P52479	506	LLTVIK(*)SSLSEK(*)	K6	63.33	76.27	0.8387096	0.742424	0.86	0.8137113
Usp10	P52479	696	LVK(*)NIDYPVDLEISR(*)	K3	50.26	1000	0.9462365	-	0.89	0.9181183
Usp10	P52479	687	FVYEK(*)TGGC(+57.02)QK(*)	K5	22.63	78.19	1.7849462	1.621212	0.63	1.3453861
Pkm	P52480	66	EMIK(*)SGMNVAR(*)	K4	98.28	1000	0.9255319	0.7375	0.956043!	0.8730253
Pkm	P52480	89	LNFSHGTHEYHAETIK(*)NVR(*)	K16	93.91	1000	0.9255319	0.825	1.131868:	0.9608
Pkm	P52480	270	IISK(*)IENHEGVR(*)	K4	87.95	1000	1.0638297	0.925	0.956043!	0.9816246
Pkm	P52480	498	VNLAMDVGK(*)AR(*)	K9	85.53	1000	0.8617021	0.8	0.967032!	0.876245
Pkm	P52480	166	NIC(+57.02)K(*)VVEVGSK(*)	K4	69.52	120.29	0.8510638	0.7125	0.912087!	0.8252172
Pkm	P52480	141	GATLK(*)ITLDNAYMEK(*)	K5	67.57	111.69	-	-	0.648351648	
Pkm	P52480	433	SGAIIVLTK(*)SGR(*)	K9	67.3	1000	1.0319148	0.75	0.890109	0.8906749
Pkm	P52480	433	C(+57.02)C(+57.02)SGAIIVLTK(*)SG	K11	66.45	1000	0.9893617	0.85	1.076923(	0.9720949

Pkm	P52480	3	PK(*)PHSEAGTAFIQTQQLHAAMADT	K2	56.83	1000	0.8510638	-	1.054945(	0.9530044
Pkm	P52480	115	PVAVALDTK(*)GPEIR(*)	K9	38.83	1000	1.0851063	1.05	1.164835:	1.0999805
Pkm	P52480	270	ISK(*)IENHEGVR(*)	K3	16.19	1000	1.2021276	1.025	1.065934(	1.0976872
Ndufs6	P52503	100	ETK(*)TGTC(+57.02)GYC(+57.02)GL	K3	18.88	100.3	1.4831460	1.493670	1.033333:	1.3367168
Stat6	P52633	613	DLAQLK(*)NLYPK(*)	K6	15.84	45.16	-	-	1.2	
Cpt2	P52825	495	QYGQTVATYESC(+57.02)STAAFK(*)H	K18	26.39	1000	0.9313725	1.56	-	1.2456863
Rpl10a	P53026	147	FPSLLTHNENMVAK(*)VDEVK(*)	K14	71.27	38.19	0.9247311	0.602409	0.849462:	0.7922011
Rpl10a	P53026	152	VDEVK(*)STIK(*)	K5	68.13	61.37	0.7956989	0.722891	0.827956!	0.7821825
Rpl10a	P53026	207	ALYIK(*)STMGK(*)PQR(*)	K5	60.42	86.16	0.7849462	0.746987	0.795698!	0.7758777
Rpl10a	P53026	207	ALYIK(*)STMGK(*)	K5	20.87	98.37	0.8172043	0.783132	0.978494(	0.8596105
Dbt	P53395	265	AMVK(*)TMSAALK(*)	K4	23.61	53.43	1.0329670	0.536585	-	0.7847762
Cebpz	P53569	390	ALLVQVINK(*)LGDPQNR(*)	K9	34.18	1000	1.7010309	-	0.842696(	1.2718638
Cebpz	P53569	401	IATK(*)ASHLLEVLLR(*)	K4	15.49	1000	1.0412371	-	-	
Hccs	P53702	264	MK(*)VAWWR(*)	K2	5.88	1000	-	-	1.428571429	
Pitpna	P53810	117	IETWHK(*)PDLGTQENVHK(*)	K6	77.87	159.14	0.82	0.792207	1.172413	0.9282072
Pitpna	P53810	202	FK(*)WWGLQNK(*)	K2	69.52	148.49	-	0.636363	0.839080	0.737722
Pitpna	P53810	68	IYHLQSK(*)VPTFVR(*)	K7	59.47	1000	0.94	0.831168	1.149425	0.9735314
Pitpnb	P53811	201	FK(*)WWGLQSK(*)	K2	68.97	157.88	0.8842105	-	0.902173!	0.8931922
Pitpnb	P53811	67	SK(*)VPAFVR(*)	K2	56.78	1000	1.0105263	0.710526	0.706521	0.8091915
Rab2a	P53994	120	LIGNK(*)SDLESR(*)	K5	26.41	1000	-	-	0.629213483	
Rab2a	P53994	120	QHSNSNMVIMLIGNK(*)SDLESR(*)	K15	9.73	1000	1.1122448	-	-	
Anapc1	P53995	143	IYK(*)SHELEK(*)	K3	37.01	56.47	-	-	0.903225806	
Anapc1	P53995	107	GSK(*)SQALAVYK(*)	K3	8.81	78.17	-	0.141025	-	
Cnbp	P53996	104	EQC(+57.02)C(+57.02)YNC(+57.02)	K9	83.71	1000	0.8834951	0.875	0.771739	0.8434114
Cnbp	P53996	153	C(+57.02)GETGHVAINC(+57.02)SK(	K13	80.44	1000	0.9902912	1.05	1.043478	1.0279232
Cnbp	P53996	153	VAINC(+57.02)SK(*)TSEVNC(+57.02	K7	31.28	1000	1.1844660	0.975	1.206521	1.1219959
Cnbp	P53996	104	ER(*)EQC(+57.02)C(+57.02)YNC(+5	K11	26.73	1000	1.0679611	1.2625	-	1.1652306
Idh2	P54071	272	DIFQEIFDK(*)HYK(*)	K9	82.89	49.37	0.9791666	0.727272	0.966292	0.8909105
Idh2	P54071	180	HAHGDQYK(*)ATDFVVDR(*)	K8	79.24	1000	0.9375	0.857142	0.943820	0.912821
Idh2	P54071	155	EPIIC(+57.02)K(*)NIPR(*)	K6	78.33	1000	1.3541666	0.727272	0.921348	1.0009292
Idh2	P54071	251	WPLYLSTK(*)NTILK(*)	K8	77.06	53.48	0.8020833	-	0.910112	0.8560978
Idh2	P54071	282	NK(*)IWYEHR(*)	K2	74.28	1000	0.8645833	1.012987	1.033707	0.9704261
Idh2	P54071	133	MWK(*)SPNGTIR(*)	K3	24.76	1000	-	-	0.966292135	
Dnajc2	P54103	85	DWK(*)NQDHYAVLGLGHVR(*)	K3	36.84	1000	0.9423076	-	-	

Dnajc2	P54103	117	AMVLK(*)HHPDK(*)	K5	29.45	45.16	0.7403846	0.551282	0.765957	0.6858747
Stmn1	P54227	128	DK(*)HVEEVR(*)	K2	56.89	1000	1.375	0.885057	1.023809	1.0946223
Stmn1	P54227	128	EK(*)DK(*)HVEEVR(*)	K4	11.81	22.85	-	0.701149	-	
Msh6	P54276	821	LLSK(*)IHNVGSPLK(*)	K4	54.95	202.16	1.0505050	0.935897	0.897727	0.9613766
Msh6	P54276	169	GGHFYSSK(*)SEILR(*)	K8	39.98	1000	1.1515151	1.076923	1.204545	1.1443279
Rad23b	P54728	6	MQVTLK(*)TLQQQTFK(*)	K6	81.52	216.51	0.9782608	0.945205	1.011363	0.9782767
Rad23b	P54728	36	GK(*)DAFPVAGQK(*)	K2	48.15	161.68	0.3260869	0.986301	1	0.7707961
Rad23b	P54728	67	IDEK(*)NFVVVMVTK(*)PK(*)	K4	45.33	143.22	-	-	0.852272727	
Faf1	P54731	145	GWK(*)TGDVEDSTVLK(*)	K3	35.42	134.4	0.7311827	-	-	
Psmc4	P54775	212	GVLMYGPPGC(+57.02)GK(*)TMLAK	K12	15.69	21.59	0.8478260	-	-	
Psmc4	P54775	404	AYK(*)TVIK(*)	K3	7.35	87.62	-	-	1.326086957	
Ddx6	P54823	321	QK(*)VHC(+57.02)LNTLFSR(*)	K2	34.93	1000	-	-	1.147727273	
Ddx6	P54823	146	NGTGK(*)SGAYLIPLLER(*)	K5	13.18	1000	-	1.342105	-	
Adprh	P54923	90	LYSLLAK(*)HYR(*)	K7	81.56	1000	1.2467532	1.013333	1.010869	1.0903187
Abcd3	P55096	61	AVVDK(*)VFLSR(*)	K5	40.3	1000	0.6701030	0.764705	0.988636	0.8078151
Kmt2a	P55200	3459	GNQLLAGK(*)TGTLTSQR(*)	K8	80.3	1000	-	0.354037	-	
Kmt2a	P55200	1200	C(+57.02)QNLQWMPSK(*)ASLQK(*)	K10	59.74	20.54	-	0.285714	-	
Kmt2a	P55200	1200	K(*)C(+57.02)QNLQWMPSK(*)ASLC	K11	36.73	29.88	-	0.422360	-	
Adk	P55264	60	YSLK(*)PNDQILAEDK(*)HK(*)	K14	68.55	39.76	0.9484536	0.851851	0.645161	0.8151556
Adk	P55264	73	FK(*)VEYHAGGSTQNSMK(*)	K2	39.77	105.23	-	-	0.88172043	
Adk	P55264	164	EK(*)HLDLER(*)	K2	39.17	1000	1.0721649	1.111111	0.709677	0.9643178
Adk	P55264	87	SMK(*)VAQWLIQEPHK(*)	K3	30.49	156.9	-	-	0.838709677	
Lrpap1	P55302	216	IK(*)SDTLISK(*)	K2	63.78	122.76	1.0384615	1.038461	0.908163	0.9950288
Lrpap1	P55302	309	LK(*)HVESIGDPEHISR(*)	K2	61.45	1000	1.2980769	1.025641	1.051020	1.1249128
Lrpap1	P55302	223	SDTLISK(*)HSELK(*)	K7	47.95	115.97	1.0769230	0.961538	0.969387	1.0026164
Lrpap1	P55302	293	IEK(*)HNHYQK(*)	K3	39.69	129.72	-	-	1.030612245	
Golga3	P55937	816	QDLAVK(*)SNQVEHLQQETATLR(*)	K6	39.11	1000	-	-	0.777777778	
Golga3	P55937	1353	VSELK(*)NNMK(*)	K5	22.39	55.36	-	-	0.424242424	
Cks2	P56390	34	QVPK(*)THLMSEEEWR(*)	K4	42.82	1000	-	0.781609	0.914893	0.8482514
Cox6b1	P56391	13	NYK(*)TAPFDSR(*)	K3	88.66	1000	0.6736842	0.860759	0.977777	0.8374072
Cox6b1	P56391	47	AMTAK(*)GGDVSVC(+57.02)EWYR(	K5	63.4	1000	1.0210526	0.734177	1.133333	0.9628544
Cox6b1	P56391	28	FPNQNQTK(*)NC(+57.02)WQNYLDI	K8	25.32	1000	-	-	1.888888889	
Cyb5a	P56395	19	YYTLEEIQK(*)HK(*)	K9	68.04	25.86	1.0612244	0.862068	0.894736	0.9393434
Cyb5a	P56395	24	DSK(*)STWVILHHK(*)	K3	66.35	126.05	1.0306122	0.873563	0.936842	0.9470059

Cyb5a	P56395	33	STWVILHHK(*)VYDLTK(*)	K9	61.51	30.85	0.8877551	-	0.968421(	0.9280881
Cyb5a	P56395	77	ELSK(*)TYIIGELHPDDR(*)	K4	17.03	1000	-	-	1.115789474	
Usp5	P56399	291	TDK(*)TMTELEIDMNQR(*)	K3	49.79	1000	0.8210526	1.455696	0.648936:	0.9752283
Usp5	P56399	178	QVSK(*)HAFNLK(*)	K4	29.19	95.35	-	0.645569	0.478723	0.5621465
Usp5	P56399	423	ALIGK(*)GHPEFSTNR(*)	K5	21.83	1000	1.2	-	-	
Atp5f1b	P56480	264	DATSK(*)VALVYGQMNEPPGAR(*)	K5	57.97	1000	0.9479166	0.4625	1.085106:	0.831841
Pdcd5	P56812	20	LAELQAK(*)HGDPGDAAQQEAK(*)	K7	79.62	166.43	0.6698113	1.075	0.806451(	0.850421
Fus	P56959	444	APK(*)PDGPGGGPGGSHMGGNYGD	K3	87.59	1000	0.9756097	0.888888	0.880952:	0.9151503
Fus	P56959	305	QIGIIK(*)TNK(*)	K6	57.86	20.7	1.0121951	-	0.809523	0.9108595
Fus	P56959	327	LK(*)GEATVSFDDPPSAK(*)	K2	10.79	39.94	1.4390243	-	-	
Exosc10	P56960	875	SMSFAVGK(*)SDR(*)	K8	25.83	1000	-	1.402597	-	
Exosc10	P56960	867	QSVGNK(*)SMSFAVGK(*)	K6	17.3	74.79	2.0740740	-	1.052083:	1.5630787
Erp29	P57759	38	LHTK(*)GALPLDTVTFYK(*)	K4	28.71	128.19	-	-	1.536842105	
Eef1d	P57776	10	A(+42.01)TNFLAHEK(*)IWFDK(*)	K9	75.32	127.18	-	-	1.280898876	
Eef1d	P57776	17	FK(*)YDDAER(*)	K2	71.16	1000	1.2210526	1.277108	1.258426	1.252196
Eef1d	P57776	117	LSSLEK(*)SSPTPR(*)	K6	56.41	1000	0.7789473	-	0.741573(	0.7602602
Eef1d	P57776	59	ENIQK(*)SLAGSSGPGASSGPGGDHS	K5	56.11	1000	0.7473684	-	1.539325	1.1433471
Actn4	P57780	284	IC(+57.02)K(*)VLAVNQENEHLMEDY	K3	64.09	1000	0.9791666	0.858974	1.076923(	0.971688
Actn4	P57780	626	WEK(*)VQQLVPK(*)	K3	61.81	148.49	0.7916666	0.846153	0.857142	0.8316545
Actn4	P57780	780	ASFNHFDK(*)DHGGALGPEEFK(*)	K8	54.5	157.14	1.5833333	0.833333	1	1.1388889
Actn4	P57780	115	INNVNK(*)ALDFIASK(*)	K6	48.07	99.23	0.9270833	1.141025	0.868131	0.9787469
Actn4	P57780	761	DAK(*)GISQEQMQEFR(*)	K3	46.65	1000	0.9583333	-	0.736263	0.8472985
Actn4	P57780	182	TAPYK(*)NVNVQNFHISWK(*)	K5	46.2	93.92	1.15625	0.5	1.054945(	0.9037317
Actn4	P57780	669	MVGPWIQTK(*)MEEIGR(*)	K9	40.22	1000	0.46875	-	0.835164	0.6519574
Actn4	P57780	433	ASIHEAWTDGK(*)EAMLK(*)	K11	35.03	98.37	0.9270833	0.871794	0.934065	0.9109814
Snrpa1	P57784	67	LK(*)TLLVNNNR(*)	K2	94.04	1000	0.8865979	0.925925	0.989010	0.933845
Snrpa1	P57784	179	SK(*)TFNPGAGLPTDK(*)	K2	76.04	151.17	1.5670103	0.851851	-	1.2094311
Snrpa1	P57784	221	LK(*)GLLQSGQIPGR(*)	K2	12.93	1000	-	-	1.967032967	
Tm9sf2	P58021	422	FYK(*)SFGGEK(*)	K3	75.5	66.56	0.7659574	0.5875	0.931818:	0.7617585
Tm9sf2	P58021	125	LVC(+57.02)TK(*)TYNTEK(*)	K5	73.4	129.72	0.8617021	0.675	0.863636:	0.8001128
Mrps6	P58064	95	IVK(*)HPLTQEVK(*)	K3	29.97	81.59	-	-	0.739130435	
Eef2	P58252	275	FSK(*)SANSPDGK(*)	K3	98.66	166.48	0.9368421	0.9	0.936170	0.9243374
Eef2	P58252	32	NM(+15.99)SVIAHVDHGK(*)STLTDS	K12	94.77	103.7	1.1052631	0.9625	0.989361	1.0190416
Eef2	P58252	598	SPNK(*)HNR(*)	K4	90.93	1000	0.9263157	0.8125	0.893617(	0.8774776

Eef2	P58252	426	VFSGVVSTGLK(*)VR(*)	K11	73.68	1000	0.8526315	0.825	0.787234(	0.8216219
Eef2	P58252	152	IK(*)PVLMMNK(*)	K2	66.91	106.82	0.6947368	0.7875	0.904255:	0.7954974
Eef2	P58252	481	LVK(*)TGTITTFEHAHNMR(*)	K3	60.48	1000	-	1.75	0.861702:	1.3058511
Eef2	P58252	32	VDHGK(*)STLTDSLVC(+57.02)K(*)	K5	59.03	129.4	1.1052631	0.75	0.734042!	0.8631019
Eef2	P58252	386	YR(*)C(+57.02)ELLYEGPPDDEAAMG	K20	55.68	29.88	-	-	0.957446809	
Eef2	P58252	32	MSVIAHVDHGK(*)STLTDSLVC(+57.(	K11	39.29	59.2	-	-	0.840425532	
Eef2	P58252	239	FAAK(*)GEGQLSAAER(*)	K4	18.72	1000	0.8	-	0.436170:	0.6180851
Opa1	P58281	847	HFLK(*)TALNHC(+57.02)NLC(+57.0	K4	24.59	1000	1.7373737	0.897435	0.543689:	1.0594997
Rnf216	P58283	22	GK(*)EWMAVR(*)	K2	46.7	1000	-	-	-	
Ptpa	P58389	296	MYK(*)AEC(+57.02)LEK(*)	K3	84.51	116.54	1.0930232	1	1.15	1.0810078
Ptpa	P58389	286	ISAVPSWSK(*)VNQGLIR(*)	K9	40.79	1000	0.8255813	0.986842	1.7375	1.1833078
Ptpa	P58389	286	TGPFAEHSNQLWNISAVPSWSK(*)VN	K22	20.85	1000	-	-	1.05	
Foxp1	P58462	538	NAATWK(*)NAVR(*)	K6	32.05	1000	-	1.101265	1.252427:	1.1768465
Paxbp1	P58501	252	ELGDFTPHDSEPGK(*)GR(*)	K14	21.36	1000	-	1.524590	0.902777	1.213684
Aaas	P58742	373	GHVGGAK(*)SATIVADLSETTIQTPDG	K7	26.16	1000	0.5434782	-	0.577777	0.560628
Aaas	P58742	189	VYNANSTIVPSLK(*)HR(*)	K13	21.33	1000	1.5217391	1.384615	-	1.4531773
Tpm1	P58771	152	EAK(*)HIAEDADR(*)	K3	96.49	1000	1.0106382	0.961038	1.011764	0.9944807
Tpm1	P58771	205	TVTNNLK(*)SLEAQAEK(*)	K7	70.04	131.34	0.8297872	1.051948	0.941176	0.9409706
Tpm1	P58771	248	SVTK(*)LEK(*)	K4	48.6	56.34	1.0212765	1.402597	1.070588:	1.1648207
Tpm1	P58771	189	AELSEGK(*)C(+57.02)AELEEELK(*)	K7	43.84	128	-	0.922077	1.847058	1.3845684
Tpm1	P58771	51	LK(*)GTEDELDK(*)YSEALK(*)	K2	21.99	81.34	1.6276595	1.636363	1.188235:	1.4840862
Tpm1	P58771	220	EDK(*)YEEEIK(*)	K3	4.34	41.94	-	-	1.682352941	
Tpm2	P58774	152	EAK(*)HIAEDSDR(*)	K3	85.19	1000	0.6526315	0.960526	1.080459	0.8978726
Ripk2	P58801	47	VAVK(*)HLHIHTPLLDSER(*)	K4	23.35	1000	-	-	-	
Tubgcp3	P58854	509	MIAVTK(*)SAESPR(*)	K6	29.03	1000	2.7111111	-	0.645161:	1.6781362
Tubgcp3	P58854	377	LK(*)TLAALVDHC(+57.02)QGR(*)	K2	24.83	1000	1.3	-	0.956989:	1.1284946
Tubgcp3	P58854	486	VLLIGK(*)SINFLHQVC(+57.02)HDQ	K6	16.77	32.61	1.1333333	-	0.903225	1.0182796
Gmeb2	P58929	136	EFVHLAGK(*)STLK(*)	K8	25.68	69.71	-	-	0.602484472	
Bcl2l13	P59017	57	LK(*)SEIEEELK(*)	K2	10.72	113.97	-	1.844155	1.467391:	1.6557736
Nup43	P59235	196	SIGQLK(*)IWDFR(*)	K6	8.6	1000	1.0842105	-	-	
Eif5	P59325	136	GMLDTHHK(*)LC(+57.02)TFILK(*)	K8	81.11	34.69	0.8854166	0.876712	1.087912(	0.9500137
Ythdf1	P59326	387	EFDWNLK(*)SGR(*)	K7	35.58	1000	-	-	0.986486486	
Wdhd1	P59328	664	GK(*)SDHYWVVGIHENPQQLR(*)	K2	61.82	1000	1.2682926	1.808219	1.253012(	1.4431746
Sirt6	P59941	93	PSK(*)THMALVQLER(*)	K3	61.91	1000	-	-	-	

Sirt6	P59941	267	LMK(*)HLGLEIPAWDGPC(+57.02)V	K3	40.23	157.13	-	-	-	
Ruvbl1	P60122	162	TISHVIIGLK(*)TAK(*)	K10	39.37	44.44	1.1649484	0.782051	1.166666(	1.0378888
Ruvbl1	P60122	22	IASHSHVK(*)GLGLDESGLAK(*)	K8	27.19	63.07	1.2371134	-	1.466666(	1.35189
Eif3e	P60229	409	TK(*)SLSFR(*)	K2	84.1	1000	1.0638297	0.9125	-	0.9881649
Eif3e	P60229	120	MLFDYLADK(*)HGFR(*)	K9	81.15	1000	1.0531914	0.9	1.021978(	0.9917232
Eif3e	P60229	387	IDSK(*)LGHVVMGNNAVSPYQQVIEK	K4	39.93	111.38	-	-	0.516483516	
Eif3e	P60229	359	MLADK(*)LNMTPEEAER(*)	K5	29.6	1000	0.9574468	-	0.802197!	0.8798223
Pcbp1	P60335	23	LLMHGK(*)EVGSIIGK(*)	K6	49.76	183.32	0.8777777	1.283783	1.035714!	1.0657586
Nploc4	P60670	52	NK(*)TGEITASSSK(*)	K2	37.89	187.15	0.9058823	1.123456	0.951219!	0.9935196
Actb	P60710	328	IK(*)IIAPPER(*)	K2	99.25	1000	0.8144329	0.819277	0.911111!	0.8482737
Actb	P60710	359	WISK(*)QEYDESGPSIVHR(*)	K4	98.76	1000	0.9072164	0.674698	1	0.8606384
Actb	P60710	113	VAPEEHPVLLTEAPLNPK(*)ANR(*)	K18	93.09	1000	0.7525773	0.734939	1.255555!	0.9143575
Actb	P60710	359	ISK(*)QEYDESGPSIVHR(*)	K3	92.41	1000	1.1340206	1.036144	0.888888!	1.0196847
Actb	P60710	359	QQMWISK(*)QEYDESGPSIVHR(*)	K7	77.43	1000	0.8556701	0.759036	0.988888!	0.867865
Actb	P60710	359	MWISK(*)QEYDESGPSIVHR(*)	K5	66.38	1000	0.9793814	0.915662	1.111111!	1.0020517
Actb	P60710	215	EK(*)LC(+57.02)YVALDFEQEM(+15.!	K2	49.51	242.12	-	-	1.133333333	
Actb	P60710	50	HQGVMVGMGQK(*)DSYVGDEAQSK	K11	22.31	118.56	-	-	1.155555556	
Actb	P60710	359	STFQQMWISK(*)QEYDESGPSIVHR(*	K10	14.96	1000	-	-	1.488888889	
Morf4l1	P60762	143	TSGLQQK(*)NVEVK(*)	K7	10.72	62.08	-	1.888888	-	
Cdc42	P60766	163	YVEC(+57.02)SALTQK(*)GLK(*)	K10	81.52	42.6	0.7446808	0.756410	0.890109!	0.797067
Eif4a1	P60843	54	GIYAYGFEK(*)PSAIQQR(*)	K9	50.46	1000	1.0531914	0.875	-	0.9640957
Eif4a1	P60843	174	YLSPK(*)YIK(*)	K5	47.24	22.45	0.9042553	0.85	0.752688!	0.8356478
Eif4a1	P60843	68	IK(*)GYDVIAQAQSGTGK(*)	K2	22.46	179.33	1.1914893	-	-	
Rps20	P60867	59	MPTK(*)TLR(*)	K4	95.16	1000	0.96	1.012658	0.82	0.9308861
Rps20	P60867	75	TPC(+57.02)GEGSK(*)TWDR(*)	K8	64.46	1000	0.82	0.772151	0.81	0.8007173
Rps20	P60867	75	K(*)TPC(+57.02)GEGSK(*)TWDR(*)	K9	45.71	64	0.8	0.987341	0.79	0.8591139
Chp1	P61022	114	SNK(*)LHFAFR(*)	K3	37.81	1000	-	0.839506	1.357142!	1.0983245
Cks1b	P61025	34	LVPK(*)THLMSESEWR(*)	K4	76.72	1000	0.9803921	0.948717	1.055555!	0.9948886
Rab10	P61027	49	IK(*)TVELQGK(*)	K2	79.13	157.88	0.7802197	0.765432	0.918604(	0.8214188
Rab10	P61027	136	GK(*)GEQIAR(*)	K2	9.04	1000	-	-	1.255813953	
Rab8b	P61028	3	A(+42.01)K(*)TYDYLFK(*)	K2	10.16	140.21	0.7340425	-	0.627906!	0.6809748
Ube2m	P61082	72	LVIC(+57.02)PDEGFYK(*)SGK(*)	K11	81.32	40.63	0.8804347	0.807692	0.752380!	0.8135027
Ube2k	P61087	24	SEETSK(*)NQIK(*)	K6	50.89	77.51	0.7659574	-	0.887640!	0.8267989
Ube2n	P61089	74	FMTK(*)IYHPNVDK(*)	K4	87.69	197.82	1	0.933333	0.954022!	0.9624521

Ube2n	P61089	94	DK(*)WSPALQIR(*)	K2	25.03	1000	-	-	1.459770115	
Ube2n	P61089	82	IYHPNVDK(*)LGR(*)	K8	24.87	1000	-	-	1.126436782	
Actr2	P61161	299	SEFYK(*)HIVLSGGSTMYPGLPSR(*)	K5	91.06	1000	0.9473684	-	0.9010989	0.9242337
Actr2	P61161	368	DK(*)DNFWMTR(*)	K2	76.17	1000	1	0.7625	0.9780219	0.9135073
Actr2	P61161	118	ILLTEPPMNPTK(*)NR(*)	K12	58.89	1000	0.6210526	-	0.8351648	0.7281087
Actr2	P61161	219	EK(*)LC(+57.02)YVGYNIEQEQK(*)	K2	50.83	221.44	0.6631578	-	0.9450549	0.8041064
Actr1a	P61164	285	AIQK(*)SDMDLR(*)	K4	81.52	1000	0.6542056	0.822784	0.9111111	0.7960338
Actr1a	P61164	96	SK(*)DQLQTFSEEHPVLLTEAPLNPR(*	K2	42.15	1000	-	-	0.866666667	
Cops2	P61202	331	ILK(*)TNHSNIMDDPFIR(*)	K3	74	1000	1.0707070	2.209876	0.9450549	1.4085462
Cops2	P61202	263	AHTDFFEAFK(*)NYDESGSPR(*)	K10	62.77	1000	0.6868686	-	0.9230769	0.8049728
Cops2	P61202	93	YK(*)QLLTYIR(*)	K2	55.86	1000	0.7777777	0.888888	1.1648351	0.9438339
Cops2	P61202	150	LWFK(*)TNTK(*)	K4	39.92	51.8	0.8585858	0.950617	0.9780219	0.929075
Cops2	P61202	225	IK(*)SAIPHPLIMGVIR(*)	K2	38.81	1000	0.9090909	0.913580	1	0.9408904
Cops2	P61202	243	EC(+57.02)GGK(*)MHLR(*)	K5	28.99	1000	1.0505050	1.197530	1.2197802	1.1559387
Cops2	P61202	225	ALYEQSLHIK(*)SAIPHPLIMGVIR(*)	K10	11.22	1000	-	-	1.274725275	
Cops2	P61202	77	QMIK(*)INFK(*)	K4	9.51	38.26	0.7878787	0.962962	-	0.8754209
Arl1	P61211	104	IGISK(*)SELVAMLEEEELR(*)K(*)	K5	21.36	132.96	-	-	0.916666667	
Abce1	P61222	419	ISPK(*)STGSVR(*)	K4	85.01	1000	0.6	0.5625	0.6703296	0.6109432
Abce1	P61222	397	LK(*)PDEGGEVPVLNVSYK(*)PQK(*)	K2	72.9	221.1	0.73	0.7	0.8791208	0.769707
Rpl26	P61255	69	GQQIGK(*)VVQVYR(*)	K6	97.6	1000	0.9223300	0.987654	0.9888888	0.9662911
Rpl26	P61255	63	GHYK(*)GQQIGK(*)	K4	71.98	132.53	0.8834951	0.888888	0.9333333	0.9019058
Psme3	P61290	14	LK(*)VDSFR(*)	K2	68.8	1000	0.9361702	0.851851	0.8367346	0.8749189
Psme3	P61290	6	A(+42.01)SLLK(*)VDQEVK(*)	K5	54.02	81.36	0.8829787	0.839506	1.3469387	1.0231412
Psme3	P61290	110	GMLK(*)SNQQLVDIIEK(*)	K4	39.41	232.86	-	-	1.173469388	
Magoh	P61327	16	GK(*)FGHEFLEFEFR(*)PDGK(*)	K2	61.53	135.32	-	-	1.078651685	
Magoh	P61327	41	YANNSNYK(*)NDVMIR(*)	K8	58.05	1000	-	5	1.1460674	3.0730337
Magoh	P61327	54	EAYVHK(*)SVMEELK(*)R(*)	K6	41.45	64.58	-	1.565789	1.3370786	1.4514341
Magoh	P61327	54	EAYVHK(*)SVMEELK(*)	K6	22.41	51.59	0.75	1.789473	-	1.2697368
Rpl27	P61358	93	YSVDIPLDK(*)TVVNK(*)	K9	92.81	95.62	0.8865979	0.875	0.9666666	0.9094215
Rpl27	P61358	27	AVIVK(*)NIDDGTSDR(*)	K5	90.36	1000	0.9175257	0.806818	1	0.9081147
Rpl27	P61358	128	NK(*)WFFQK(*)	K2	85.1	98.37	0.8659793	0.806818	0.9333333	0.8687103
Rpl27	P61358	27	AVIVK(*)NIDDGTSDR(*)PYSHALVAG	K5	62.8	1000	2.3402061	0.897727	1.1555555	1.4644963
Rpl27	P61358	73	SFVK(*)VYNYNHLMPTR(*)	K4	54.06	1000	0.9484536	1.079545	1.0444444	1.0241478
Rpl27	P61358	9	PGK(*)VVLVLAGR(*)	K3	29.8	1000	-	-	0.755555556	

Rpl27	P61358	27	K(*)AVIVK(*)NIDDGTSDR(*)PYSHAL'	K6	18.4	52.68	1.1443298	1.25	1.222222	1.2055174
Rpl27	P61358	27	VK(*)NIDDGTSDR(*)PYSHALVAGIDF	K2	7.14	1000	-	-	0.644444444	
Rpl37a	P61514	80	TTSAVTVK(*)SAIR(*)	K8	80.44	1000	0.9207920	0.783132	0.979591	0.8945055
Rpl37a	P61514	80	TVAGGAWTYNTTSAVTVK(*)SAIR(*)	K18	44.52	1000	1.0891089	-	1.306122	1.1976157
Rpl37a	P61514	80	NTTSAVTVK(*)SAIR(*)	K9	30.68	1000	0.9405940	0.975903	0.755102	0.8905332
Rpl37a	P61514	13	VGIVGK(*)YGTR(*)	K6	7.62	1000	-	-	1.602040816	
Copz1	P61924	46	AFEK(*)NIFNK(*)	K4	62.45	142.99	0.7472527	1.527777	1.2	1.1583435
Copz1	P61924	51	NIFNK(*)THR(*)	K5	54.97	1000	0.7912087	0.861111	1.047058	0.8997929
Sumo2	P61957	42	HTPLSK(*)LMK(*)	K6	74.11	57.45	0.7291666	-	0.739130	0.7341486
Sumo2	P61957	11	EGVK(*)TENNDHINLK(*)	K4	59.8	228.69	1.03125	0.913580	1	0.9816101
Ufm1	P61961	69	VPAATSAIITNDGIGINPAQTAGNVFLI	K28	28.56	1000	-	-	0.755813953	
Dcaf7	P61963	167	VNLVSGHVK(*)TQLIAHDK(*)	K9	42.54	71.07	0.9809523	0.347826	-	0.6643892
Wdr5	P61965	112	TLK(*)IWDVSSGK(*)	K3	53.83	86.34	1.1756756	0.848837	1.838235	1.2875827
Hnrnpk	P61979	198	VVLIGGK(*)PDR(*)	K7	96.89	1000	0.84375	0.842105	1.022222	0.9026925
Hnrnpk	P61979	405	DLAGSIIGK(*)GGQR(*)	K9	94.98	1000	0.9270833	0.986842	0.966666	0.9601974
Hnrnpk	P61979	52	ILLQSK(*)NAGAVIGK(*)	K6	94.36	193.63	0.9166666	0.934210	1.466666	1.105848
Hnrnpk	P61979	139	YK(*)GSDFDC(+57.02)ELR(*)	K2	53.02	1000	1.125	1.026315	1.033333	1.0615497
Hnrnpk	P61979	422	HESGASIK(*)IDEPLEGSEDR(*)	K8	47.09	1000	-	-	0.8	
Ywhag	P61982	120	NC(+57.02)SETQYESK(*)VFYLK(*)	K10	88.06	119.54	1.0495049	0.698795	1.031578	0.9266264
Ywhag	P61982	69	VISSIEQK(*)TSADGNEK(*)	K8	37.7	123.53	1.1980198	0.734939	1.147368	1.026776
Rps7	P62082	90	FSGK(*)HVVFIAQR(*)	K4	90	1000	1.0102040	0.962962	0.936170	0.9697791
Rps7	P62082	169	AQQNNVEHK(*)VETFSGVYK(*)	K9	89.4	140.79	1.0102040	1.049382	0.968085	1.009224
Rps7	P62082	160	VHLDK(*)AQQNNVEHK(*)	K5	76.4	225.26	1.2142857	1.160493	1.010638	1.1284726
Rps7	P62082	90	K(*)FSGK(*)HVVFIAQR(*)	K5	54.31	49.79	1.0102040	1.074074	0.968085	1.0174544
Rps7	P62082	155	LIK(*)VHLDK(*)	K3	48.77	115.97	-	0.975308	1.010638	0.9929735
Rps7	P62082	169	NVEHK(*)VETFSGVYK(*)	K5	38.48	59.43	-	0.913580	1.212765	1.0631731
Ppp1cb	P62141	25	PGK(*)IVQMTEAEVR(*)	K3	58.49	1000	0.8235294	0.766233	0.986111	0.8586248
Ppp1cb	P62141	259	AHQVVEDGYEFFAK(*)R(*)	K14	54.62	1000	0.9117647	-	1.236111	1.0739379
Ppp1cb	P62141	40	GLC(+57.02)IK(*)SR(*)	K5	40.49	1000	1.1911764	-	-	
Ppp1cb	P62141	25	GC(+57.02)R(*)PGK(*)IVQMTEAEVR	K6	37.56	1000	-	0.831168	2.25	1.5405844
Psmc1	P62192	423	SK(*)ENVLYK(*)	K2	68.82	132.53	1.0107526	1.089743	0.869565	0.9900205
Psmc1	P62192	69	IK(*)DYLLMEEEFIR(*)	K2	8.62	1000	-	-	1.195652174	
Psmc5	P62196	330	LDILK(*)IHSR(*)	K5	80.9	1000	1.2947368	0.936708	1.054945	1.0954636
Psmc5	P62196	170	EVIELPVK(*)HPELFEALGIAQPK(*)	K8	75.43	140.25	0.8631578	-	0.912087	0.8876229

Psmc5	P62196	397	DSEK(*)NMSIK(*)	K4	55.14	70.86	0.6842105	0.822784	1	0.8356651
Psmc5	P62196	196	GVLLYGPPGTGK(*)TLLAR(*)	K12	51.6	1000	0.8105263	0.708860	0.890109{	0.8031657
Psmc5	P62196	94	VHPEGK(*)FVVDVDK(*)	K6	25.92	85.08	1.2105263	-	0.989010{	1.0997687
Rps8	P62242	37	PAANTK(*)IGPR(*)	K6	72.17	1000	-	-	0.901098901	
Rps8	P62242	98	TLVK(*)NC(+57.02)IVLIDSTPYR(*)	K4	66.38	1000	0.9381443	0.8625	0.989010{	0.9298851
Rps15a	P62245	43	MK(*)HGYIGEFEIIDDHR(*)	K2	83.65	1000	0.8958333	0.865853	1.054945(	0.9388773
Rps15a	P62245	60	AGK(*)IVVNLTGR(*)	K3	52.39	1000	0.3854166	-	0.923076{	0.6542468
Rps15a	P62245	43	MMK(*)HGYIGEFEIIDDHR(*)	K3	42.57	1000	0.8020833	-	0.989010{	0.8955472
Ube2h	P62257	52	FYGPQGTPYEGGVWK(*)VR(*)	K15	48.46	1000	-	-	0.700854701	
Ube2h	P62257	22	LIESK(*)HEVTILGGLNEFVVK(*)	K5	15.83	110.25	-	-	1.666666667	
Ywhae	P62259	125	MK(*)GDYHR(*)	K2	95.54	1000	0.9795918	-	1.032608(	1.0061003
Ywhae	P62259	118	HLIPAANTGESK(*)VFYYK(*)	K12	95.11	105.53	0.8571428	0.827160	0.804347{	0.8295504
Ywhae	P62259	106	LIC(+57.02)C(+57.02)DILDVLDK(*)I	K12	72.75	69.26	1.0714285	0.728395	0.989130	0.9296514
Ywhae	P62259	50	NLLSVAYK(*)NVIGAR(*)	K8	68.92	1000	0.9183673	0.864197	0.989130	0.9238984
Ywhae	P62259	118	TGESK(*)VFYYK(*)	K5	24.29	127.18	-	1.604938	1.119565	1.3622517
Rps14	P62264	63	VK(*)ADR(*)DESSPYAAMLAAQDVAC	K2	45.25	1000	0.5	1.654761	0.597826(	0.9175293
Rps14	P62264	106	TK(*)TPGPGAQSALR(*)	K2	28.93	1000	-	-	0.880434783	
Rps14	P62264	86	C(+57.02)K(*)ELGITALHIK(*)	K2	12.34	123.42	-	-	1.086956522	
Rps23	P62267	48	ANPFGGASHAK(*)GIVLEK(*)	K11	81.07	113.99	0.8529411	0.944444	0.944444	0.9139434
Rps23	P62267	124	VVK(*)VANVSLLALYK(*)	K3	63.88	207.07	0.8921568	0.819444	1	0.9038671
Rps23	P62267	76	VQLIK(*)NGK(*)	K5	44.86	32.69	1.1274509	1.291666	1.266666(	1.2285948
Rps23	P62267	25	WHDK(*)QYK(*)	K4	25.53	30.72	-	0.986111	-	
Rps18	P62270	137	GQHTK(*)TTGR(*)	K5	74.35	1000	0.9696969	-	1.021739	0.9957181
Rps18	P62270	78	QYK(*)IPDWFLNR(*)	K3	66.75	1000	0.6969696	0.506024	0.880434	0.6944762
Rps11	P62281	45	NIGLGFK(*)TPK(*)	K7	97.27	41.2	0.8653846	0.913580	0.924731	0.901232
Rps11	P62281	107	HK(*)NMSVHLSPC(+57.02)FR(*)	K2	86.21	1000	0.8653846	0.814814	0.935483{	0.8718944
Rps11	P62281	38	YYK(*)NIGLGFK(*)	K3	78.87	184.45	0.8942307	1	0.860215(	0.9181486
Rps11	P62281	144	FNVLK(*)VTK(*)	K5	35.92	48.45	1.2211538	-	-	
Rps11	P62281	136	DVQIGDIVTVGEC(+57.02)R(*)PLSK(	K18	8.51	1000	1.4615384	-	-	
Rps11	P62281	38	YYK(*)NIGLGFK(*)TPK(*)	K3	5.19	72.22	-	0.987654	-	
Rps13	P62301	9	MHAPGK(*)GLSQSALPYR(*)	K6	95.73	1000	1.0638297	0.875	0.892473	0.9437676
Rps13	P62301	70	FVTGNK(*)ILR(*)	K6	53.94	1000	0.5212765	-	0.838709(	0.6799931
Rps13	P62301	78	SK(*)GLAPDLPEDLYHLIK(*)	K2	53.42	179.51	1.0744680	0.75	0.870967	0.8984786
Rps13	P62301	34	LTSDDVK(*)EQIYK(*)	K7	19.09	115.97	0.8297872	-	1.182795(	1.0062915

Snrpg	P62309	3	S(+42.01)K(*)AHPPELK(*)	K2	53.74	95.63	0.7663551	0.488372	-	0.6273636
Snrpd2	P62317	79	EMWTEVPK(*)SGK(*)	K8	63.53	22.34	1	0.736842	0.966292	0.9010447
Snrpd2	P62317	98	YISK(*)MFLR(*)	K4	5.68	1000	-	-	0.988764045	
Lsm5	P62322	31	IHIVMK(*)SDK(*)	K6	79.97	53.53	1.0113636	0.824324	0.930232	0.9219735
Arf6	P62331	7	VLSK(*)IFGNK(*)	K4	52.71	81.69	0.7222222	0.917808	1	0.8800101
Psmc6	P62334	180	GC(+57.02)LLYGPPGTGK(*)TLLAR(*	K12	77.9	1000	0.7978723	0.730769	0.988888	0.8391768
Psmc6	P62334	322	IHAGPITK(*)HGEIDYEAIVK(*)	K8	63.42	176.98	1	0.820512	0.977777	0.9327635
Psmc6	P62334	314	LDILK(*)IHAGPITK(*)	K5	43.24	175.25	0.9042553	0.730769	0.9	0.8450082
Rps4x	P62702	16	VAAPK(*)HWMLDK(*)	K5	96.14	143.14	0.8775510	0.777777	0.870967	0.8420988
Rps4x	P62702	134	IFVGTK(*)GIPHLVTHDAR(*)	K6	90.92	1000	0.9183673	0.901234	0.956989	0.9255304
Rps4x	P62702	106	LIYDTK(*)GR(*)	K6	85.4	1000	0.8163265	0.777777	-	0.7970522
Rps4x	P62702	94	MDVISIDK(*)TGENFR(*)	K8	53.07	1000	0.9693877	-	0.688172	0.8287799
Rps4x	P62702	230	LSNIFVIGK(*)GNK(*)PWISLPR(*)	K9	51.33	49.37	0.7040816	-	0.892473	0.7982774
Rps4x	P62702	22	HWMLDK(*)LTGVFAPR(*)	K6	47.07	1000	-	-	1.032258065	
Rps4x	P62702	71	FIK(*)IDGK(*)	K3	38.83	29.57	1.0102040	0.827160	1.043010	0.9601251
Rpl18a	P62717	76	VK(*)NFGIWLR(*)	K2	98.32	1000	0.7731958	0.768292	0.913978	0.818489
Rpl18a	P62717	41	IFAPNHVVAK(*)SR(*)	K10	74.28	1000	0.7525773	0.743902	0.827956	0.7748122
Rpl18a	P62717	11	EYK(*)VVGR(*)	K3	58.88	1000	0.8247422	0.646341	0.677419	0.7161677
Rpl18a	P62717	128	AHSIQIMK(*)VEEIAAGK(*)	K8	53.2	99.23	0.6907216	0.548780	0.774193	0.6712319
Rpl18a	P62717	41	APNHVVAK(*)SR(*)	K8	51.7	1000	0.8144329	0.560975	0.838709	0.7380394
Rpl18a	P62717	41	FAPNHVVAK(*)SR(*)	K9	43.13	1000	10.154639	-	6.634408	8.3945239
Acta2	P62737	361	WISK(*)QEYDEAGPSIVHR(*)	K4	78.3	1000	0.8971962	0.879518	0.760416	0.8457103
Acta2	P62737	115	VAPEEHPTLLTEAPLNPK(*)ANR(*)	K18	66.75	1000	0.6822429	0.662650	-	0.6724468
Acta2	P62737	361	ISK(*)QEYDEAGPSIVHR(*)	K3	59.11	1000	1.2990654	0.819277	0.833333	0.983892
Ap2s1	P62743	45	DAK(*)HTNFVEFR(*)	K3	73.93	1000	1.39	-	0.87	1.13
Ap2s1	P62743	18	LAK(*)WYMQFDDDEK(*)	K3	6.75	137.5	-	-	1.09	
Rpl23a	P62751	70	NK(*)LDHYAIIK(*)	K2	71.19	216.51	1.02	1.101265	1.047058	1.0561082
Rpl23a	P62751	106	ANK(*)HQIK(*)	K3	64.15	87.62	1.02	0.987341	1.047058	1.0181335
Rpl23a	P62751	70	R(*)NK(*)LDHYAIIK(*)	K3	50.02	150.63	1.05	1.012658	1.235294	1.0993174
Rps6	P62754	149	LFNLSK(*)EDDVR(*)	K6	81.27	1000	1	0.948717	1.063157	1.0039586
Rps6	P62754	203	NK(*)EEAAEYAK(*)	K2	77.95	142.27	0.9	1.025641	0.852631	0.9260909
Rps6	P62754	79	LLLSK(*)GHSC(+57.02)YR(*)	K5	68.36	1000	1	0.833333	0.905263	0.9128655
Rps6	P62754	79	LLLSK(*)GHSC(+57.02)YR(*)PR(*)	K5	61.86	1000	0.89	0.884615	0.936842	0.9038192
Rps6	P62754	2	MK(*)LNISFPATGC(+57.02)QK(*)	K2	30.92	126.79	-	1.012820	1.010526	1.0116734

Mtpn	P62774	66	GADINAPDK(*)HHITPLLSAVYEGHVS	K9	76.54	126.1	1.0294117	0.948051	0.808988	0.9288175
Mtpn	P62774	90	LLLSK(*)GADK(*)	K5	52.98	25.11	0.8725490	0.571428	1.089887	0.8446217
Mtpn	P62774	11	EFMWALK(*)NGDLDEVK(*)	K7	48.37	86.34	-	1.714285	1	1.3571429
Mtpn	P62774	24	DYVAK(*)GEDVNR(*)	K5	25.05	1000	1.0980392	1.350649	1	1.1495629
H4f16	P62806	17	G(+42.01)LGK(*)GGAK(*)R(*)	K8	99.79	32.48	0.79	0.917647	0.978723	0.8954568
H4f16	P62806	9	GK(*)GGK(*)GLGK(*)	K5	99.77	97.69	0.93	0.858823	0.829787	0.8728703
H4f16	P62806	6	GK(*)GGK(*)GLGK(*)	K2	99.77	186.64	0.93	0.858823	0.829787	0.8728703
H4f16	P62806	13	G(+42.01)K(*)GGK(*)GLGK(*)GGAK	K9	98.79	48.83	0.01	-	-	
H4f16	P62806	9	G(+42.01)K(*)GGK(*)GLGK(*)GGAK	K5	98.79	65.81	0.01	-	-	
H4f16	P62806	17	LGK(*)GGAK(*)R(*)	K7	97.64	1000	-	1.164705	0.925531	1.0451189
H4f16	P62806	13	LGK(*)GGAK(*)R(*)	K3	97.64	1000	-	1.164705	0.925531	1.0451189
H4f16	P62806	13	G(+42.01)GK(*)GLGK(*)GGAK(*)	K7	95.14	31.27	0.78	-	-	
H4f16	P62806	9	GGK(*)GLGK(*)	K3	84.23	97.69	1.24	1.141176	1.191489	1.1908886
H4f16	P62806	13	GK(*)GLGK(*)GGAK(*)	K6	79.3	55.21	0.88	0.929411	0.872340	0.8939174
H4f16	P62806	9	GK(*)GLGK(*)GGAK(*)	K2	79.3	150.89	0.88	0.929411	0.872340	0.8939174
H4f16	P62806	9	S(+42.01)GR(*)GK(*)GGK(*)GLGK(*	K8	74.18	67.69	-	0.929411	-	
H4f16	P62806	6	S(+42.01)GR(*)GK(*)GGK(*)GLGK(*	K5	74.18	83.89	-	0.929411	-	
H4f16	P62806	17	G(+42.01)GK(*)GLGK(*)GGAK(*)R(*	K11	70.54	139.85	0.75	43.88235	0.702127	15.111494
H4f16	P62806	13	G(+42.01)GK(*)GLGK(*)GGAK(*)R(*	K7	70.54	105.6	0.75	43.88235	0.702127	15.111494
H4f16	P62806	9	GGK(*)GLGK(*)GGAK(*)R(*)	K3	67.35	1000	0.87	0.847058	1.031914	0.9163246
H4f16	P62806	17	GK(*)GLGK(*)GGAK(*)R(*)	K10	62.54	1000	0.88	0.882352	0.861702	0.874685
H4f16	P62806	13	GK(*)GLGK(*)GGAK(*)R(*)	K6	62.54	1000	0.88	0.882352	0.861702	0.874685
H4f16	P62806	9	GK(*)GLGK(*)GGAK(*)R(*)	K2	62.54	1000	0.88	0.882352	0.861702	0.874685
H4f16	P62806	9	GGK(*)GLGK(*)GGAK(*)	K3	60.31	166.01	0.77	0.811764	0.829787	0.8038506
H4f16	P62806	13	GLGK(*)GGAK(*)R(*)	K4	60.14	1000	0.8	0.929411	1.010638	0.91335
H4f16	P62806	80	R(*)K(*)TVTAMDVVYALK(*)	K2	58.68	204.37	0.93	0.847058	1.106382	0.9611473
H4f16	P62806	32	DNIQGITK(*)PAIR(*)	K8	52.43	1000	0.75	0.788235	0.776595	0.7716103
H4f16	P62806	6	GK(*)GGK(*)GLGK(*)GGAK(*)	K2	49.07	254.8	0.26	0.694117	0.446808	0.4669754
H4f16	P62806	78	DAVTYTEHAK(*)R(*)	K10	47.08	1000	1.07	1.047058	1.276595	1.1312182
H4f16	P62806	92	TVTAMDVVYALK(*)R(*)	K12	40.47	1000	1.12	1.494117	0.861702	1.1586066
Atp6v1b2	P62814	460	FEK(*)NFITQGPYENR(*)	K3	73.29	1000	-	0.876712	0.943181	0.9099471
Atp6v1b2	P62814	403	LMK(*)SAIGEGMTR(*)	K3	73.19	1000	0.6571428	0.808219	0.727272	0.7308783
Rab1A	P62821	187	MGPGATAGGAEK(*)SNVK(*)	K12	54.18	48.83	0.84375	0.525641	0.903225	0.7575389
Rab1A	P62821	61	IK(*)LQIWDTAGQER(*)	K2	48.82	1000	1.0104166	0.807692	1.043010	0.9537066

Rab1A	P62821	24	LLLIGDSGVGK(*)SC(+57.02)LLLR(*)	K11	39	1000	-	-	0.935483871	
Ran	P62827	134	AK(*)SIVFHR(*)	K2	97.67	1000	0.9354838	1.074074	0.989010!	0.999523
Ran	P62827	99	VTYK(*)NVPNWHR(*)	K4	83.67	1000	0.8602150	0.864197	0.912087!	0.8788335
Ran	P62827	23	LVLVGDGGTGK(*)TTFVK(*)	K11	78.09	107.29	0.8924731	0.827160	0.791208	0.8369475
Ran	P62827	123	VC(+57.02)ENIPIVLC(+57.02)GNK(*	K13	72.78	67.69	1	0.814814	0.989010!	0.9346086
Rpl23	P62830	113	GEMK(*)GSAITGPVAK(*)	K4	9.84	58.64	-	0.881578	1.107526!	0.9945529
Rps15	P62843	77	EAPPM(+15.99)EK(*)PEVVK(*)THLR	K12	83.46	94.22	-	0.825	0.885057	0.8550287
Rps15	P62843	77	K(*)EAPPMEK(*)PEVVK(*)THLR(*)	K13	44.77	98.37	0.6770833	0.95	0.816091!	0.8143918
Rps24	P62849	68	THFGGGK(*)TTGFGMIYDSLDYAK(*)	K7	90.34	298.8	0.9708737	-	0.892473	0.9316735
Rps24	P62849	37	ATVPK(*)TEIR(*)	K5	69.86	1000	0.9029126	1.051948	1.064516	1.0064589
Rps24	P62849	32	QMVIDVLHPGK(*)ATVPK(*)	K11	62.94	22.36	1.2427184	0.935064	0.903225!	1.0270031
Rps24	P62849	37	VPK(*)TEIR(*)	K3	10.33	1000	-	0.870129	-	
Rps25	P62852	52	LNNLVLFDK(*)ATYDK(*)	K9	93.29	107.29	0.9892473	0.846153	1.011235!	0.948879
Rps25	P62852	94	AALQELLSK(*)GLIK(*)	K9	83.38	82.24	0.9462365	1.051282	0.898876	0.965465
Rps25	P62852	52	DK(*)LNNLVLFDK(*)ATYDK(*)	K11	65.29	32.16	0.8494623	0.589743	1.067415	0.8355406
Rps25	P62852	57	ATYDK(*)LC(+57.02)K(*)	K5	26.07	49.37	-	7.217948	6.078651	6.6483002
Rps26	P62855	82	LHYC(+57.02)VSC(+57.02)AIHSK(*)	K12	96.27	1000	1.1313131	1.228571	1.056179	1.1386881
Rps28	P62858	16	VTK(*)VLGR(*)	K3	92.23	1000	-	-	0.858585859	
Elob	P62869	11	HK(*)TTIFTDAK(*)	K2	98.04	211.64	0.8969072	0.907894	0.967741!	0.9241813
Elob	P62869	11	R(*)HK(*)TTIFTDAK(*)	K3	15.3	154.74	1.1546391	-	-	
Gnb1	P62874	57	GHLAK(*)IYAMHWGTDSR(*)	K5	88.12	1000	0.9270833	-	1	0.9635417
Gnb1	P62874	15	QEAEQLK(*)NQIR(*)	K7	76.62	1000	0.7083333	-	0.652631!	0.6804825
Gnb1	P62874	89	LIIWDSYTTNK(*)VHAIPLR(*)	K11	76.07	1000	1.0416666	0.755319	1.157894	0.9849602
Rpl30	P62889	32	YVLGYK(*)QTLK(*)	K6	92.96	58.99	0.8476190	0.8	0.865979	0.8378661
Rpl30	P62889	68	LAK(*)TGVHHYSGNNIELGTAC(+57.0	K3	78	132.17	0.9333333	0.8	0.886597!	0.8733104
Rpl30	P62889	87	TGVHHYSGNNIELGTAC(+57.02)GK(	K19	72.3	1000	1.0476190	1.022222	1.020618!	1.0301533
Rpl30	P62889	68	K(*)SEIEYYAMLAK(*)TGVHHYSGNNI	K12	16.29	41.98	1.5619047	-	-	
Rpl30	P62889	26	SGK(*)YVLGYK(*)	K3	14.95	102.87	-	-	0.463917526	
Rpl30	P62889	87	SGNNIELGTAC(+57.02)GK(*)YYR(*)	K13	11.85	1000	-	-	1.103092784	
Cycs	P62897	28	HK(*)TGPNLHGLFGR(*)	K2	67.22	1000	4.4090909	2.342857	0.547619	2.433189
Rpl31	P62900	70	LNK(*)AVWAK(*)	K3	89.3	111.23	0.86	0.850574	0.938775!	0.8831167
Rpl31	P62900	75	AVWAK(*)GIR(*)	K5	88.52	1000	0.9	0.839080	0.959183	0.8994214
Rps3	P62908	187	VK(*)IMLPWDPSGK(*)	K2	80.3	148.37	0.7578947	0.716049	0.869565	0.7811698
Rps3	P62908	132	FIMESGAK(*)GC(+57.02)EVVVSGK(*	K8	78.95	140.39	0.8	0.716049	1.054347	0.8567991

Rps3	P62908	108	YK(*)LLGGLAVR(*)	K2	36.93	1000	0.6526315	1.160493	1.0434782	0.9522012
Rps3	P62908	62	TQNVLGEK(*)GR(*)	K8	28.91	1000	1.4105263	0.654320	1.771739	1.2788621
Rpl32	P62911	67	TK(*)HMLPSGFR(*)	K2	79.57	1000	0.8260869	0.883116	1.0543478	0.9211839
Rpl32	P62911	106	SYC(+57.02)AEIAHNVSSK(*)NR(*)	K13	76.88	1000	0.9673913	0.974025	1.021739	0.9877188
Rpl32	P62911	93	ELEVLLMC(+57.02)NK(*)SYC(+57.0	K10	65.84	29.28	0.7717391	-	0.945652	0.8586957
Rpl8	P62918	60	GAPLAK(*)VVFR(*)	K6	46.23	1000	1.0925925	0.548780	0.882978	0.8414506
Ybx1	P62960	56	VIATK(*)VLGTVK(*)	K5	78.37	123.86	0.8817204	1.405063	1.146067	1.1442837
Ybx1	P62960	79	NDTK(*)EDVFVHQTAIK(*)	K4	74.72	176.98	0.9032258	1.063291	0.955056	0.9738577
Pfn1	P62962	116	TLVLLMGK(*)EGVHGGLINK(*)	K8	56.94	44.69	0.8989898	0.617283	1	0.838758
Pfn1	P62962	91	TK(*)STGGAPTFNVTVTMTAK(*)	K2	36.7	213.24	-	-	0.724137931	
Rps27a	P62983	6	MQIFVK(*)TLTGK(*)	K6	99.83	87.95	1.0120481	0.811764	0.882978	0.9022639
Rps27a	P62983	6	VK(*)TLTGK(*)	K2	97.19	105.53	1.2168674	0.929411	0.946808	1.0310292
Rps27a	P62983	6	QIFVK(*)TLTGK(*)	K5	79.68	82.34	-	-	0.808510638	
Rps27a	P62983	143	HYC(+57.02)GK(*)C(+57.02)C(+57.0	K5	78.7	161.68	1.1566265	1.035294	1.468085	1.2200019
Rps27a	P62983	6	IFVK(*)TLTGK(*)	K4	70.11	40.91	-	-	0.872340426	
Rps27a	P62983	11	TLTGK(*)TITLEVEPSDTIENVK(*)	K5	42.06	161.67	0.7469879	-	1.255319	1.0011536
Rps27a	P62983	48	LIFAGK(*)QLEDGR(*)	K6	31.14	1000	-	0.835294	-	
Rac1	P63001	166	GLK(*)TVFDEAIR(*)	K3	85.54	1000	0.8762886	0.822784	1.058139	0.919071
Rac1	P63001	116	HHC(+57.02)PNTPIILVGTK(*)LDLR(	K14	52.79	1000	0.9690721	-	1.011627	0.99035
Pafah1b1	P63005	228	MWEVQTGYC(+57.02)VK(*)TFTGHR	K11	58.53	1000	0.5824175	-	1.130434	0.8564262
Pafah1b1	P63005	360	FILSC(+57.02)ADDK(*)TLR(*)	K9	51.81	1000	0.8021978	-	-	
Hspa8	P63017	507	ITITNDK(*)GR(*)	K7	92.54	1000	1.0309278	1.025974	0.901098	0.9860003
Hspa8	P63017	102	PK(*)VQVEYK(*)	K2	91.85	156.85	0.8350515	0.844155	0.890109	0.8564391
Hspa8	P63017	539	VSSK(*)NSLESYAFNMK(*)	K4	90.97	281.19	0.8969072	0.883116	0.934065	0.9046967
Hspa8	P63017	328	LDK(*)SQIHDIVLVGGSTR(*)	K3	88.97	1000	0.9690721	0.948051	0.890109	0.9357447
Hspa8	P63017	128	MK(*)EIAEAYLGK(*)	K2	87.83	250.15	0.8041237	0.792207	0.923076	0.8398028
Hspa8	P63017	500	ENK(*)ITITNDK(*)	K3	76.8	128.65	0.8247422	0.766233	0.791208	0.7940616
Hspa8	P63017	112	GETK(*)SFYPEEVSSMVLTK(*)	K4	70.2	214.3	1.1030927	0.987012	0.967032	1.0190462
Hspa8	P63017	56	LIGDAAK(*)NQVAMNPTNTVFDAK(*)	K7	65.39	170.25	0.9381443	0.922077	1.241758	1.0339935
Hspa8	P63017	88	R(*)FDDAVVQSDMK(*)HWPFMVVN	K12	59.58	1000	1.9072164	-	1.010989	1.4591028
Hspa8	P63017	88	FDDAVVQSDMK(*)HWPFMVVNDA	K11	59.37	1000	-	-	0.967032967	
Hspa8	P63017	512	LSK(*)EDIER(*)	K3	51.79	1000	0.9381443	1.961038	1.153846	1.3510098
Hspa8	P63017	25	GK(*)VEIIANDQGNR(*)	K2	50.69	1000	0.8969072	0.987012	0.824175	0.9026987
Hspa8	P63017	573	ILDK(*)C(+57.02)NEIISWLDK(*)	K4	49.37	251.58	1.5051546	-	1.175824	1.3404894

Hspa8	P63017	88	R(*)FDDAVVQSDMK(*)HWPFM(+15	K12	40.27	37.72	-	-	1.010989011	
Hspa8	P63017	108	VQVEYK(*)GETK(*)	K6	38	58.99	0.8659793	1.324675	1.197802:	1.1294856
Hspa8	P63017	246	MVNHFIAEFK(*)R(*)	K10	36.89	1000	-	-	0.813186813	
Hspa8	P63017	451	AMTK(*)DNNLLGK(*)	K4	33.23	78.16	1.1340206	0.987012	1.252747:	1.1245936
Hspa8	P63017	357	LLQDFFNGK(*)ELNK(*)	K9	13.02	24.67	-	-	1.296703297	
Hspa8	P63017	25	QHGK(*)VEIIANDQGNR(*)	K4	4.92	1000	0.8144329	-	-	
Tpt1	P63028	112	VK(*)PFMTGAAEQIK(*)	K2	72.39	236.99	0.7623762	0.7625	0.945054!	0.8233104
Tpt1	P63028	19	DLISHDELFSDIYK(*)IR(*)	K14	56.22	1000	-	-	0.648351648	
Tpt1	P63028	97	DYMK(*)SLK(*)	K4	11.21	22.45	-	0.75	-	
Tpt1	P63028	85	HHLQETSFTK(*)EAYK(*)	K10	8.26	38.03	-	-	1.32967033	
Dnaja1	P63037	296	TIVITSHPGQIVK(*)HGDIK(*)	K13	97.63	65.44	0.8571428	0.764705	0.909090!	0.8436465
Dnaja1	P63037	130	LALQK(*)NVIC(+57.02)DK(*)	K5	87.09	143.14	0.7346938	0.705882	0.848484	0.7630204
Dnaja1	P63037	130	LALQK(*)NVIC(+57.02)DK(*)C(+57.(	K5	57.83	94.27	0.7448979	0.623529	1.424242	0.9308899
Dnaja1	P63037	46	FK(*)QISQAYEVLADSK(*)	K2	45.4	213.54	-	-	0.929292929	
Dnaja1	P63037	130	K(*)LALQK(*)NVIC(+57.02)DK(*)C(+	K6	13.37	39.44	1.4489795	0.988235	1.090909(	1.1760413
Hspd1	P63038	91	YK(*)NIGAK(*)	K2	92.4	115.97	1.0103092	0.975308	0.934065!	0.973228
Hspd1	P63038	523	GIIDPTK(*)VVR(*)	K7	67.32	1000	0.8453608	0.765432	0.879120	0.8299713
Hspd1	P63038	72	TVIIEQSWGSPK(*)VTK(*)	K12	61.01	57.45	-	0.543209	1.516483!	1.0298467
Hspd1	P63038	292	LK(*)VGLQVVAVK(*)	K2	53.28	195.94	-	-	1.010989011	
Hspd1	P63038	82	DGVTVAK(*)SIDLK(*)	K7	23.67	70.86	0.3608247	0.555555	0.615384(	0.5105883
Hspd1	P63038	481	IPAM(+15.99)TIAK(*)NAGVEGSLIVE	K8	9	167.97	-	-	1.230769231	
Eif4e	P63073	162	GDK(*)IAIWTTEC(+57.02)ENR(*)	K3	67.62	1000	2.0645161	0.779220	0.836956!	1.2268978
Eif4e	P63073	36	TESNQEVANPEHYIK(*)HPLQNR(*)	K15	52.71	1000	-	0.753246	1.206521	0.9798842
Eif4e	P63073	54	SK(*)TWQANLR(*)	K2	49.56	1000	0.8602150	0.831168	0.771739:	0.821041
Eif4e	P63073	206	IVIGYQSHADTATK(*)SGSTTK(*)	K14	39.85	22.09	-	1.623376	0.597826(	1.1106014
Mapk1	P63085	268	NYLLSLPHK(*)NK(*)	K9	67.56	36.05	0.9358974	0.628571	0.96	0.8414896
Mapk1	P63085	201	APEIMLNSK(*)GYTK(*)	K9	24.79	48.12	1.1282051	1	1.146666(	1.0916239
Gnas	P63094	96	VQDIK(*)NNLK(*)	K5	86.62	77.13	1	0.858974	0.944444	0.9344729
Gnas	P63094	216	FQVDK(*)VNFHMFDVGGQR(*)	K5	73.58	1000	0.8260869	-	0.911111:	0.868599
Gnas	P63094	91	ATK(*)VQDIK(*)	K3	25.12	127.18	0.4347826	0.846153	0.922222	0.7343862
Ywhaz	P63101	115	ASQPESK(*)VFYLK(*)	K7	86.88	115.97	0.8556701	0.820512	1.022988!	0.8997238
Ywhaz	P63101	68	VVSSIEQK(*)TEGAEK(*)	K8	65.31	33.48	-	0.538461	-	
Ywhaz	P63101	3	M(+42.01)DK(*)NELVQK(*)	K3	52.14	132.53	0.8350515	0.628205	1.011494:	0.824917
Nfyb	P63139	69	IMK(*)NAIPQTGK(*)	K3	45.53	131.72	-	-	-	

Hmgb1	P63158	114	IK(*)GEHPGLSIGDVAK(*)	K2	68.62	207.86	1.1505376	0.947368	1.321839(	1.139915
Dynll1	P63168	9	AVIK(*)NADMSEEMQQDSVEC(+57.0	K4	69.51	475.55	-	0.802197	1.153846:	0.978022
Rps17	P63276	49	NK(*)IAGYVTHLM(+15.99)K(*)	K2	76.27	250.23	0.9684210	1.08	1.055555!	1.0346589
Rps17	P63276	19	VIIEK(*)YYTR(*)	K5	59	1000	0.9263157	0.933333	1.044444	0.9680312
Rps12	P63323	93	LGEWVGLC(+57.02)K(*)IDR(*)	K9	91.16	1000	0.9897959	0.765432	1.053763	0.9363305
Rps12	P63323	116	DYGK(*)ESQAK(*)	K4	34.82	87.95	0.9081632	0.716049	0.849462:	0.8245583
Rps10	P63325	31	DVHMPK(*)HPELADK(*)	K6	77.2	61.6	1.0666666	0.973333	0.957446	0.9991489
Rps10	P63325	31	K(*)DVHMPK(*)HPELADK(*)	K7	56.54	110.43	1.0111111	0.96	0.861702:	0.9442711
Ppp2ca	P63330	29	QLSESQVK(*)SLC(+57.02)EK(*)	K8	57.9	87.95	1.4105263	1.732394	0.954545	1.365822
Csnk2b	P67871	147	C(+57.02)MDVYTPK(*)SSR(*)	K8	52.49	1000	1.0430107	0.986842	1	1.009951
Rpl22	P67984	69	SK(*)ITVTSEVPFSK(*)	K2	47.98	128.06	0.8709677	-	0.885416(	0.8781922
Ube2l3	P68037	138	FC(+57.02)K(*)NAEEFTK(*)	K3	85.57	163.4	0.8387096	0.8	1.060240!	0.8996502
Ube2l3	P68037	82	IYHPNIDEK(*)GQVC(+57.02)LPVISA	K9	52.03	156.83	1.0967741	-	0.963855	1.0303148
Ube2l3	P68037	82	IYHPNIDEK(*)GQVC(+57.02)LPVISA	K9	44.51	92.48	-	-	0.939759036	
Ube2l3	P68037	20	C(+57.02)GMK(*)NFR(*)	K4	43.82	1000	-	-	1.518072289	
Rack1	P68040	38	DK(*)TIIMWK(*)	K2	93	139.02	0.9130434	0.7375	0.923076!	0.8578735
Rack1	P68040	185	LK(*)TNHIGHTGYLNTVTVSPDGSLC(-	K2	82.73	322.5	1.0869565	0.9625	0.901098!	0.9835185
Rack1	P68040	175	LVK(*)VWNLANC(+57.02)K(*)	K3	78.91	161.77	0.8804347	0.8375	0.923076!	0.8803372
Rack1	P68040	172	SSNPIIVSC(+57.02)GWDK(*)LVK(*)	K13	75.25	44.84	0.9021739	0.825	0.934065!	0.8870799
Rack1	P68040	139	LWNTLGVC(+57.02)K(*)YTVQDESH!	K9	61.56	1000	-	-	1.164835165	
Rack1	P68040	106	FVGHTK(*)DVLSVAFSSDNR(*)	K6	46.49	1000	1.9347826	-	1.351648:	1.6432155
Rack1	P68040	271	IIVDELK(*)QEVISTSSK(*)	K7	18.92	22.83	1.0760869	-	-	
Rack1	P68040	106	TK(*)DVLSVAFSSDNR(*)	K2	3.47	1000	-	-	2.274725275	
Prkacb	P68181	24	AK(*)EDFLR(*)	K2	64.57	1000	1.1142857	-	1.301369	1.2078278
Ywhaq	P68254	49	NLLSVAYK(*)NVVGGR(*)	K8	83.83	1000	0.8333333	1.125	0.771739:	0.9100242
Ywhaq	P68254	122	MK(*)GDYFR(*)	K2	75.94	1000	0.8039215	0.7	0.826086!	0.7766695
Ywhaq	P68254	3	M(+42.01)EK(*)TELIQK(*)	K3	68.92	121.92	0.7843137	0.7625	0.771739:	0.772851
Ywhaq	P68254	68	VISSIEQK(*)TDTSDK(*)K(*)	K8	45.02	113.99	0.8431372	-	1	0.9215686
Ywhaq	P68254	68	VISSIEQK(*)TDTSDK(*)	K8	39.11	121.92	0.6078431	0.9375	0.826086!	0.7904767
Tuba4a	P68368	60	TIGGGDDSFTTFFC(+57.02)ETGAGK(	K20	25.12	1000	0.0109890	0.012345	0.019801!	0.0143789
Tuba1a	P68369	401	FDLMYAK(*)R(*)	K7	96.26	1000	0.82	0.841463	0.887755:	0.8497395
Tuba1a	P68369	394	LDHK(*)FDLMYAK(*)	K4	95.96	203.4	0.88	0.878048	0.897959:	0.885336
Tuba1a	P68369	311	HGK(*)YMAC(+57.02)C(+57.02)LLYI	K3	81.81	1000	0.87	0.939024	0.969387	0.9261374
Tuba1a	P68369	60	TIGGGDDSFNTFFSETGAGK(*)HVPR(	K20	75.05	1000	1.01	1.121951	0.959183(	1.0303783

Tuba1a	P68369	336	DVNAAIATIK(*)TK(*)	K10	68.62	33.18	0.81	0.780487	0.836734(	0.8090742
Tuba1a	P68369	60	FSETGAGK(*)HVPR(*)	K8	62.94	1000	0.93	0.792682	0.836734(	0.8531392
Tuba1a	P68369	96	QLFHPEQLITGK(*)EDAANNYAR(*)	K12	57.47	1000	0.87	-	0.938775!	0.9043878
Tuba1a	P68369	60	TFFSETGAGK(*)HVPR(*)	K10	34.49	1000	-	-	0.724489796	
Tubb4b	P68372	58	INVYYNEATGGK(*)YVPR(*)	K12	58.31	1000	1.1382978	0.372093	1.157303:	0.8892314
Tubb4b	P68372	297	MFDAK(*)NMMAAC(+57.02)DPR(*)	K5	44.13	1000	-	-	1.314606742	
Ywhah	P68510	110	FLIK(*)NC(+57.02)NDFQYESK(*)	K4	86.76	229.18	0.9895833	0.896103	0.967391:	0.9510262
Ywhah	P68510	127	MK(*)GDYYR(*)	K2	86.24	1000	0.9479166	0.870129	0.989130‹	0.9357257
Ywhah	P68510	50	NLLSVAYK(*)NVVGAR(*)	K8	78.75	1000	0.8854166	0.948051	0.978260{	0.9372432
Sbds	P70122	33	R(*)FEIAC(+57.02)YK(*)NK(*)	K8	69.39	45.01	0.8089887	0.5625	1.012345(	0.7946115
Sbds	P70122	35	NK(*)VVGWR(*)	K2	65.97	1000	0.7865168	-	-	
Kpnb1	P70168	62	VAAGLQIK(*)NSLTSK(*)	K8	89.68	121.92	0.6344086	0.734177	1.244444‹	0.8710101
Kpnb1	P70168	859	AK(*)TLATWATK(*)	K2	86.27	146.5	0.7956989	0.797468	0.933333:	0.8421669
Pip5k1a	P70182	125	FK(*)TYAPVAFR(*)	K2	35.05	1000	-	-	0.736842105	
Psmb7	P70195	127	MLK(*)QMLFR(*)	K3	44.76	1000	1.5473684	0.936708	1.361702:	1.2819265
Lxn	P70202	162	MLK(*)IQTVK(*)	K3	23.23	81.69	1.0782608	0.759493	1.235955(	1.0245699
Plxna1	P70206	1739	HTWK(*)SNC(+57.02)LPLR(*)	K4	9.27	1000	-	0.547619	0.978260{	0.76294
Itpr3	P70227	608	LLEK(*)HITK(*)	K4	60.73	109.97	0.8202247	0.820512	0.756097!	0.798945
Itpr3	P70227	307	FK(*)HLATGNYLAAEENPSYK(*)	K2	52.42	223.59	1.0898876	0.551282	0.841463‹	0.8275444
Itpr3	P70227	2596	NK(*)NLDWFPR(*)	K2	29.46	1000	1.2022471	-	-	
Itpr3	P70227	1111	VIK(*)SELDR(*)	K3	22.56	1000	-	0.756410	0.841463‹	0.7989368
Vamp7	P70280	121	HHSENK(*)SLDK(*)	K6	46.24	28.74	-	-	0.92	
Vamp7	P70280	172	TENLVDSSVTFK(*)TTSR(*)	K12	45.82	1000	-	-	1.08	
Hdac2	P70288	90	SIR(*)PDNMSEYSK(*)QMQR(*)	K12	42.1	1000	0.4945054	-	0.786516{	0.6405112
Hdac2	P70288	201	VMTVSFHK(*)YGEYFPGTGDLR(*)	K8	40.73	1000	-	-	1.314606742	
Hdac2	P70288	32	VC(+57.02)YYYDGDIGNYYYGQGHPI	K21	17.89	1000	-	-	1.898876404	
Pebp1	P70296	150	FK(*)VETFR(*)	K2	82.57	1000	0.9042553	-	1.130952:	1.0176039
Pebp1	P70296	93	MK(*)GNDISSGTVLSDYVGSGPPSGT(	K2	41.97	1000	0.8297872	-	-	
Pebp1	P70296	141	YVWLVYEQEQPLSC(+57.02)DEPILSI	K22	9.47	1000	-	-	2.583333333	
Hnrnph2	P70333	98	SNSVEMDWVLK(*)HTGPNSPDTAND	K11	51.01	1000	0.9795918	-	0.793478:	0.886535
Rock1	P70335	639	ITSLQEEVK(*)HLK(*)	K9	28.33	32.46	0.4040404	-	0.470588:	0.4373143
Rock2	P70336	310	IMDHK(*)NSLC(+57.02)FPEDTEISK(	K5	40.54	111.11	0.5686274	0.569767	1.057471:	0.7319554
Rock2	P70336	224	DVK(*)PDNMLLDK(*)HGHLK(*)	K11	32.51	46.87	0.8823529	0.848837	0.712643(	0.8146113
Rock2	P70336	511	ALLQHK(*)NAEYQR(*)	K6	19.76	1000	-	-	1.057471264	

Hint1	P70349	21	AQVAQPGGDTIFGK(*)IIR(*)	K14	66.53	1000	0.9793814	0.746987	1.046511	0.9242937
Elavl1	P70372	320	ILQVSFK(*)TNK(*)	K7	89.29	53.53	0.9157894	0.761904	0.913043	0.8635792
Elavl1	P70372	55	DK(*)VAGHSLGYGFVNYVTAK(*)	K2	73.31	265.69	0.9263157	0.761904	0.956521	0.8815808
Elavl1	P70372	191	FAANPNQNK(*)NMALLSQLYHSPAR(	K9	8.64	1000	-	-	1.695652174	
Rad50	P70388	446	TIELK(*)TEILTK(*)	K5	49.24	151.71	1.0681818	-	0.613861	0.8410216
Usp9x	P70398	447	HEAIVK(*)NVHDLLAK(*)	K6	66.12	107.73	1.0744680	1.052631	1.079545	1.0688817
Usp9x	P70398	884	GK(*)HLSFIVR(*)	K2	57.31	1000	0.7234042	0.842105	0.954545	0.8400183
Usp9x	P70398	303	NEAK(*)NDALSMIIK(*)	K4	56.99	85.06	0.6595744	-	0.613636	0.6366054
Usp9x	P70398	315	SLK(*)NLASR(*)	K3	56.73	1000	-	0.842105	1.113636	0.9778708
Usp9x	P70398	329	VPGQEETVK(*)NLEIFR(*)	K9	52.75	1000	1.0531914	-	1.068181	1.0606867
Usp9x	P70398	1370	AK(*)HSGDYFTLLR(*)	K2	48.46	1000	0.7553191	0.934210	1.147727	0.9457523
Usp9x	P70398	112	GLDVK(*)SEAC(+57.02)QR(*)	K5	34.78	1000	1.1276595	1.052631	1.034090	1.0714607
Usp9x	P70398	534	IK(*)ILDYSC(+57.02)SQDR(*)	K2	11.63	1000	1.2127659	-	-	
Cux1	P70403	144	EFAEVK(*)NQEVTIK(*)	K6	7.97	21.15	-	-	-	
Idh3g	P70404	149	TSLDLYANVIHC(+57.02)K(*)SLPGV\	K13	22.15	1000	-	-	0.693181818	
Bid	P70444	135	ALDEVK(*)TAFPR(*)	K6	31.64	1000	0.7058823	0.333333	0.351351	0.4635223
Vasp	P70460	343	VK(*)QELLEEVR(*)	K2	23.27	1000	1.2268041	1.271604	0.918367	1.1389255
Ldb1	P70662	271	DC(+57.02)LK(*)TC(+57.02)LFQK(*)	K4	33.46	94.27	1.1224489	0.811594	1.733333	1.2224588
Naca	P70670	2072	SK(*)NILFVITK(*)PDVYK(*)	K2	70.19	192.66	1.0219780	-	1.023529	1.0227537
Naca	P70670	2054	AMSK(*)LGLR(*)	K4	35.21	1000	1.0219780	0.910256	-	0.9661172
Casp3	P70677	11	TSVDSK(*)SINNFEVK(*)	K6	69.78	181.66	1.3296703	0.694117	0.913978	0.9792555
Casp3	P70677	88	NK(*)NDLTR(*)	K2	54.32	1000	1.1208791	-	1.096774	1.1088267
Casp3	P70677	19	SINNFEVK(*)TIHGSK(*)	K8	41.55	49.74	1.1318681	0.988235	1.225806	1.1153033
Casp3	P70677	156	PK(*)LFIIQAC(+57.02)R(*)	K2	39.58	1000	1.2307692	1.023529	-	1.1271493
Casp3	P70677	25	TIHGSK(*)SVDSGIYLDSSYK(*)	K6	6.65	75.67	1.6483516	-	-	
Urod	P70697	359	VGAFVDAVHK(*)HSR(*)	K10	49.27	1000	1.4516129	0.652173	1.611111	1.2382993
Ctps1	P70698	28	GVIASSVGTILK(*)SC(+57.02)GLHVT	K12	61.46	44.77	0.9479166	0.986842	1.010638	0.981799
Ctps1	P70698	557	LPHYLQK(*)GC(+57.02)R(*)	K7	34.77	1000	1.0729166	-	-	
Ctps1	P70698	460	TLFQTK(*)NSVMR(*)	K6	8.92	1000	-	1.592105	-	
Cct7	P80313	368	AK(*)TC(+57.02)TIILR(*)	K2	95.48	1000	0.9157894	0.797468	1.088888	0.9340489
Cct7	P80313	77	LLDVVHPAAK(*)TLVDIAK(*)	K10	72.73	61.68	1.1052631	0.911392	0.911111	0.9759222
Cct7	P80313	55	GK(*)ATISNDGATILK(*)	K2	54.26	153.88	-	-	0.811111111	
Cct7	P80313	401	AIK(*)NDSVVAGGGAIEMELSK(*)	K3	52.96	129.75	1.3052631	-	1	1.1526316
Cct7	P80313	148	K(*)QDK(*)VEQR(*)	K4	13.69	38.16	0.9789473	-	-	

Cct2	P80314	284	ILK(*)HGINC(+57.02)FINR(*)	K3	97.33	1000	0.9081632	0.825	1.010869!	0.9146776
Cct2	P80314	191	LK(*)GSGNLEAIHVIK(*)	K2	77.42	223.58	0.9693877	0.875	0.934782(	0.9263901
Cct2	P80314	72	DAALMVTNDGATILK(*)NIGVDNPAA	K15	76.68	59.2	-	-	1.206521739	
Cct2	P80314	176	LLTHHK(*)DHFTK(*)	K6	75.52	107.29	1.0204081	0.775	0.880434	0.8919476
Cct2	P80314	50	GMDK(*)ILLSSGR(*)	K4	75.46	1000	0.7448979	0.6625	0.869565	0.7589877
Cct2	P80314	181	DHFTK(*)LAVEAVLR(*)	K5	52.35	1000	0.8163265	0.8875	0.978260	0.8940291
Cct2	P80314	40	IGAIAIGDLVK(*)STLGPK(*)	K11	38.35	85.52	1.6224489	-	0.923913(	1.273181
Cct2	P80314	250	IK(*)IFGSR(*)	K2	31.75	1000	1.1428571	0.8125	-	0.9776786
Cct4	P80315	531	SILK(*)IDDVVNTR(*)	K4	90.05	1000	0.9479166	0.8125	0.935483	0.8986335
Cct4	P80315	126	LLQK(*)GIHPTIISESFQK(*)	K4	88.9	90.21	1	0.8125	0.956989	0.9231631
Cct4	P80315	302	TGC(+57.02)NVLLIQK(*)SILR(*)	K10	86.1	1000	0.875	0.7	0.967741	0.8475806
Cct4	P80315	384	ITGC(+57.02)TSPGK(*)TVTIVVR(*)	K9	80.86	1000	1.28125	0.7625	0.935483	0.993078
Cct4	P80315	395	GSNK(*)LVIEEAER(*)	K4	71.78	1000	0.96875	0.7625	0.752688	0.8279794
Cct4	P80315	79	GDVTITNDGATILK(*)QMQVLHPAAR	K14	50.43	1000	0.8229166	-	0.935483	0.8792003
Cct4	P80315	302	K(*)TGC(+57.02)NVLLIQK(*)SILR(*)	K11	43.69	116.35	0.71875	0.6125	0.967741	0.7663306
Cct4	P80315	321	MK(*)IMVVK(*)	K2	40.69	142.99	1.0520833	1.075	0.870967	0.9993504
Cct4	P80315	55	TSLGPK(*)GMDK(*)	K6	38.98	29.39	0.8333333	1.125	-	0.9791667
Cct4	P80315	375	LFK(*)ITGC(+57.02)TSPGK(*)	K3	34.14	91.09	-	0.725	-	
Cct5	P80316	176	TTLGSK(*)VINSC(+57.02)HR(*)	K6	74.52	1000	0.9489795	0.777777	0.902173	0.8763104
Cct5	P80316	35	LMGLEALK(*)SHIMAAK(*)	K8	70.7	110.43	0.9795918	0.691358	0.945652	0.8722007
Cct5	P80316	529	M(+15.99)ILK(*)IDDIR(*)	K4	66.49	1000	0.9387755	0.728395	0.815217	0.8274627
Cct5	P80316	265	HK(*)LDVMSVEDYK(*)	K2	60.99	168.62	1.0918367	0.950617	2.065217	1.3692238
Cct5	P80316	150	IAIQHLDK(*)ISDK(*)	K8	51.27	69.71	0.9897959	0.765432	0.967391	0.9075398
Cct5	P80316	214	VEGK(*)VGGR(*)	K4	39.04	1000	-	0.716049	0.891304	0.8036769
Cct5	P80316	170	VLVDINNPEPLIQTAK(*)TTLGSK(*)	K16	36.56	38	1.1836734	-	1.326086	1.2548802
Cct5	P80316	223	LEDTK(*)LIK(*)	K5	12.13	40.63	2.6428571	1.925925	2.739130	2.4359712
Cct6a	P80317	251	TEVNSGFFYK(*)SAEER(*)	K10	93.55	1000	0.8877551	0.743902	1.127659	0.9197724
Cct6a	P80317	5	A(+42.01)AVK(*)TLNPK(*)	K4	93.53	96.85	0.9183673	0.780487	0.957446	0.885434
Cct6a	P80317	199	HK(*)SETDTSLIR(*)	K2	91.84	1000	0.8571428	0.865853	0.893617(	0.8722045
Cct6a	P80317	381	GPNK(*)HTLTQIK(*)	K4	80.15	186.64	0.8775510	0.682926	0.691489	0.7506557
Cct6a	P80317	287	VC(+57.02)GDSDK(*)GFVVINQK(*)	K7	57.78	71.6	-	-	1.138297872	
Cct3	P80318	44	TC(+57.02)LGPK(*)SMMK(*)	K6	93.67	69.71	0.84375	0.784810	0.989361	0.8726406
Cct3	P80318	78	EIQVQHPAAK(*)SMIEISR(*)	K10	71	1000	1.1875	0.645569	0.914893(	0.9159877
Cct3	P80318	469	AK(*)HTQESC(+57.02)ETWGVNGET(	K2	70.33	219.61	1.3645833	1.202531	0.989361	1.1854922

Cct3	P80318	31	VQSGNINAAK(*)TIADIIR(*)	K10	49.34	1000	1.0729166	1.025316	0.755319:	0.9511841
Cct3	P80318	427	SK(*)AMTGVEQWPYR(*)	K2	25.77	1000	2.0416666	1.658227	1.957446{	1.8857804
Cct3	P80318	163	SSITTK(*)VISR(*)	K6	7.96	1000	-	-	0.893617021	
Wnk1	P83741	1949	FQVTTTANK(*)VGR(*)	K9	49.09	1000	0.9634146	0.628571	-	0.795993
Phf5a	P83870	3	A(+42.01)K(*)HHPDLIFC(+57.02)R('	K2	96.03	1000	1	0.892857	0.946808!	0.9465552
Phf5a	P83870	95	IVNLGSSK(*)TDLFYER(*)	K8	75.97	1000	0.9793814	0.833333	0.936170:	0.916295
Txnl4a	P83877	88	NK(*)HIMIDLGTGNNNK(*)	K2	49.25	189.17	1.0568181	1.676923	0.978021!	1.2372544
Rpl36a	P83882	89	C(+57.02)K(*)HFELGGDK(*)	K2	73.12	135.81	0.8080808	0.8	0.908045!	0.8387089
Rpl36a	P83882	22	HQPHK(*)VTQYK(*)	K5	71.19	107.29	1	0.853333	0.896551	0.9166284
Rpl36a	P83882	89	C(+57.02)K(*)HFELGGDK(*)K(*)	K2	13.99	154.74	1.2222222	1.333333	1.298850!	1.284802
Cbx1	P83917	139	WK(*)NSDEADLVPAK(*)	K2	67.58	158.48	0.5703703	0.875	0.720930:	0.7221002
Eloc	P83940	72	EIPSHVLSK(*)VC(+57.02)MYFTYK(*)	K9	52.82	131.34	-	-	1.217948718	
Eloc	P83940	80	VC(+57.02)MYFTYK(*)VR(*)	K8	17.92	1000	0.7979797	-	1.782051:	1.2900155
Hpca	P84075	174	GAK(*)SDPSIVR(*)	K3	61.32	1000	-	-	-	
Arf1	P84078	127	ANK(*)QDLPNAMNAAEITDK(*)	K3	4.06	83.43	-	-	0.705357143	
Ap2m1	P84091	308	VVIK(*)SNFK(*)	K4	75.09	69.71	-	-	0.896551724	
Ap2m1	P84091	308	VVIK(*)SNFK(*)PSLLAQK(*)	K4	55.91	77.13	0.8421052	1.067567	0.919540:	0.943071
Ap2m1	P84091	203	VVMK(*)SYLSGMPEC(+57.02)K(*)	K4	29.67	207.31	-	2.594594	-	
Ap2m1	P84091	345	YK(*)ASENAIVWK(*)	K2	12.33	137.2	-	-	1.942528736	
Rhog	P84096	96	HK(*)WHPEVC(+57.02)HHC(+57.02	K2	35.05	84.27	0.7474747	-	1.089887(	0.9186812
Rpl19	P84099	126	HMYHSLYLK(*)VK(*)	K9	89.25	26.31	0.9166666	0.869047	1.022222:	0.9359788
Rpl19	P84099	133	GNVFK(*)NK(*)	K5	85.86	26.31	0.8333333	0.726190	0.933333:	0.8309524
Rpl19	P84099	80	HMGIGK(*)R(*)	K6	29.94	1000	-	-	0.744444444	
Rpl19	P84099	186	K(*)EEIIK(*)TLSK(*)	K6	26.81	37.48	0.75	0.809523	-	0.7797619
Rpl19	P84099	92	MPEK(*)VTWMR(*)	K4	13.52	1000	-	-	1.3	
Rpl19	P84099	181	K(*)K(*)EEIIK(*)	K2	12.15	1000	0.9375	0.869047	-	0.9032738
Srsf3	P84104	23	VYVGNLGNNGNK(*)TELER(*)	K12	83.51	1000	0.77	0.777777	0.989247:	0.845675
Nptn	P97300	248	SENK(*)NEGQDAMMYC(+57.02)K(*)	K4	34.64	176.98	3.3866666	-	-	
Polr1d	P97304	15	ISGLK(*)TSMAEGER(*)	K5	25.08	1000	-	0.852459	-	
Mcm2	P97310	450	DNK(*)VAVGELTDEDVK(*)	K3	13.45	126.79	-	-	4.988888889	
Mcm6	P97311	407	SQFLK(*)HVDEFSPR(*)	K5	88.78	1000	0.8979591	0.753086	0.934065!	0.8617038
Mcm6	P97311	654	LLNK(*)SIIR(*)	K4	87.67	1000	0.8775510	0.864197	0.989010!	0.9102532
Mcm6	P97311	643	MHC(+57.02)C(+57.02)DEVQPK(*)H	K10	73.23	49.37	0.8571428	0.827160	0.967032!	0.8837788
Mcm6	P97311	197	FLLDTNK(*)SR(*)	K7	48.67	1000	1.1530612	-	0.989010!	1.0710361

Mcm6	P97311	326	ELR(*)DEEQTAESIK(*)NQMTVK(*)	K13	13.88	71.27	-	-	1.263736264	
Mcm6	P97311	611	DGSGVTK(*)SSWR(*)	K7	6.52	1000	-	-	1.582417582	
Csrp2	P97314	59	NLDSTTVAIHDEEIYC(+57.02)K(*)SC(	K17	65.42	72.78	0.8396226	-	1.108695	0.9741591
Csrp2	P97314	137	IIGAGK(*)PWHK(*)	K6	38.35	87.62	0.6509433	0.813333	0.782608	0.7489618
Csrp2	P97314	141	IIGAGK(*)PWHK(*)NC(+57.02)FR(*)	K10	36.38	58.99	0.8301886	0.946666	1.206521	0.994459
Csrp2	P97314	141	PWHK(*)NC(+57.02)FR(*)	K4	5.13	1000	-	-	1.326086957	
Csrp1	P97315	141	SWHK(*)SC(+57.02)FR(*)	K4	80.61	1000	0.8703703	0.837209	1.063829	0.9238032
Csrp1	P97315	173	GC(+57.02)YAK(*)NFGPK(*)	K5	71.54	87.95	0.8518518	0.802325	1.031914	0.8953641
Csrp1	P97315	137	VIGAGK(*)SWHK(*)	K6	66.4	59.3	0.3425925	0.906976	0.946808	0.7321259
Csrp1	P97315	59	NLDSTTVAVHGEEIYC(+57.02)K(*)SC	K17	65.81	77.53	0.8703703	0.662790	0.882978	0.8053799
Csrp1	P97315	69	YGPK(*)GYGYGQGAGTLSTDK(*)	K4	36.85	98.97	1.2129629	1.430232	-	1.3215978
Csrp1	P97315	168	GLESTTLADK(*)DGEIYC(+57.02)K(*)	K17	36.57	68.6	0.8703703	1.244186	0.319148	0.8112351
Kif20a	P97329	452	SK(*)QNLIPFR(*)	K2	35.51	1000	0.6083333	0.6875	0.455445	0.5837596
Nrp1	P97333	772	VLLHK(*)SLK(*)	K5	60.64	57.45	0.8421052	0.634408	1.266666	0.9143935
Nrp1	P97333	356	YYVK(*)TYR(*)	K4	10.95	1000	-	1.440860	-	
Rps3a	P97351	46	NIGK(*)TLVTR(*)	K4	96.55	1000	0.8627450	0.759036	0.875	0.8322604
Rps3a	P97351	94	LITEDVQGK(*)NC(+57.02)LTNFHGM	K9	87.72	1000	0.8725490	0.686746	0.979166	0.8461542
Rps3a	P97351	56	TQGTK(*)IASDGLK(*)	K5	80.2	148.49	0.7941176	0.734939	0.885416	0.8048247
Rps3a	P97351	85	FK(*)LITEDVQGK(*)	K2	33.51	142.42	-	0.337349	-	
Sptlc2	P97363	279	IFK(*)HNNMQSLEK(*)	K3	23.03	76.95	0.9204545	1.252873	1.3	1.157776
Sptlc2	P97363	213	QEIGNLDK(*)HEELEK(*)	K8	22.62	36.01	-	1.862068	1.25	1.5560345
Atp1b3	P97370	273	VSFK(*)VTAR(*)	K4	5.01	1000	-	-	1.125	
Psme1	P97371	166	VFELMTNLHTK(*)LEGFHTQISK(*)	K11	58.96	35.88	-	-	0.866666667	
Psme1	P97371	13	VHPEAQAK(*)VDVFR(*)	K8	37.94	1000	1.125	0.828947	0.877777	0.9439084
Psme2	P97372	156	TK(*)VEAFQTTISK(*)	K2	91.37	251.58	0.8787878	0.765432	0.906976	0.8503989
Frg1	P97376	8	A(+42.01)EYSYVK(*)STK(*)	K7	12.81	22.34	-	-	1.404761905	
G3bp2	P97379	76	VLSLNFSEC(+57.02)HTK(*)IR(*)	K12	61.19	1000	1.2621359	1.108108	0.927083	1.0991091
Anxa11	P97384	253	DLK(*)SELSGNFEK(*)	K3	50.86	174.15	0.6330275	0.681318	0.956989	0.7571118
Anxa11	P97384	477	MYGK(*)SLYHDITGDTSGDYR(*)	K4	40.37	1000	-	-	0.720430108	
Lig3	P97386	422	LIK(*)HDLK(*)	K3	10.12	87.62	1.2906976	-	-	
Arhgap5	P97393	544	ESLLLK(*)HIGFVYHPTK(*)	K6	37.17	59.58	0.2246376	-	0.54	0.3823188
Arhgap5	P97393	321	NTFSK(*)HIEQLK(*)	K5	35.9	90.32	0.5942028	-	0.92	0.7571014
Anxa4	P97429	293	LYGK(*)SLYSFIK(*)	K4	73.39	148.49	0.8910891	0.949367	0.902173	0.91421
Anxa4	P97429	246	SK(*)PSYFAER(*)	K2	72.1	1000	1.0396039	1.556962	1	1.1988553

Anxa4	P97429	57	SAYK(*)STIGR(*)	K4	20.86	1000	0.7920792	1.075949	1.065217	0.9777487
Anxa4	P97429	300	SLYSFIK(*)GDTSGDYR(*)	K7	12.84	1000	-	-	3.565217391	
Anxa4	P97429	213	NHLLHVFDEYK(*)R(*)	K11	8.86	1000	3.2376237	-	-	
Mprip	P97434	395	GWLTK(*)QYEDGQWK(*)	K5	38.77	118.18	-	-	1.107142857	
Fhl1	P97447	124	GTVWHK(*)DC(+57.02)FTC(+57.02)	K6	78.37	95.39	0.6428571	0.756410	1.215053	0.8714404
Fhl1	P97447	118	AIVAGDQNVEYK(*)GTVWHK(*)	K12	63.44	43.89	-	0.884615	-	
Fhl1	P97447	233	NPITGFGK(*)GSSVVAYEGQSWHDYC	K8	58.54	71.48	-	-	0.870967742	
Fhl1	P97447	160	FAK(*)HC(+57.02)VK(*)	K3	43.77	97.69	0.9693877	0.782051	1.043010	0.9314833
Atp5pf	P97450	79	LK(*)QMYGK(*)	K2	44.61	107.29	1.1898734	1	1.121951	1.1039415
Bop1	P97452	7	A(+42.01)GAC(+57.02)GK(*)PHMSI	K6	45.72	88.86	0.9895833	0.76	-	0.8747917
Smad4	P97471	506	MSFVK(*)GWGPDYPR(*)	K5	36.91	1000	-	-	1.5	
Smad4	P97471	106	WPDLHK(*)NELK(*)	K6	31.85	44.28	0.6860465	0.767123	0.688888	0.7140196
Smad4	P97471	110	NELK(*)HVK(*)	K4	28.62	57.45	0.7674418	0.479452	0.655555	0.6341498
Smarcc1	P97496	60	VWLGK(*)HYK(*)	K5	57.82	44.84	0.2857142	0.276315	0.705882	0.4226375
Smarcc1	P97496	102	HVTNPAFTK(*)LPAK(*)	K9	40.36	42.89	-	-	1.047058824	
Cpt1a	P97742	639	IAC(+57.02)EK(*)HQHLYR(*)	K5	14.27	1000	-	-	0.859649123	
Thumpd3	P97770	455	C(+57.02)FTK(*)ALSGMGHVWR(*)	K4	34.55	1000	-	0.764705	-	
Smn1	P97801	48	AVASFK(*)HALK(*)	K6	69.18	48.83	1.0769230	-	0.946808	1.0118658
Smn1	P97801	52	HALK(*)NGDIC(+57.02)ETPDK(*)PK	K4	16.9	102.09	2.4395604	1.551282	1.542553	1.8444652
Fh	P97807	411	MIK(*)NVLHSAR(*)	K3	59.64	1000	1	0.842105	1.129411	0.9905057
Fh	P97807	411	PMMIK(*)NVLHSAR(*)	K5	29.89	1000	-	-	1.035294118	
Anp32e	P97822	12	INMELK(*)NR(*)	K6	61.29	1000	1.1523809	1.0375	1.265306	1.151729
Anp32e	P97822	113	IK(*)DLSTVEALQNLK(*)NLK(*)	K14	56.51	28.96	-	-	0.795918367	
G3bp1	P97855	76	VMSQNFTNC(+57.02)HTK(*)IR(*)	K12	85.84	1000	1.0967741	0.95	1.055555	1.0341099
G3bp1	P97855	36	FYGK(*)NSSYAHGGLDSNGK(*)	K4	34.5	47.75	-	-	0.8	
Tubb5	P99024	154	SLGGGTGSGMGTLLISK(*)IR(*)	K17	76.25	1000	0.9285714	0.746835	0.870967	0.8487915
Tubb5	P99024	58	ISVYYNEATGGK(*)YVPR(*)	K12	68.2	1000	0.8571428	0.835443	0.881720	0.8581021
Tubb5	P99024	336	EVDEQMLNVQNK(*)NSSYFVEWIPNI	K12	56.78	79.53	-	-	1.462365591	
Tubb5	P99024	297	DAK(*)NMMAAC(+57.02)DPR(*)	K3	35.51	1000	-	0.594936	0.666666	0.6308017
Tubb5	P99024	154	QLTHSLGGGTGSGMGTLLISK(*)IR(*)	K21	21.85	1000	-	-	1.215053763	
Tubb5	P99024	297	VFDAK(*)NMMAAC(+57.02)DPR(*)	K5	12.29	1000	1.8367346	-	-	
Tubb5	P99024	297	ALTVPELTQQVFDAK(*)NM(+15.99)I	K15	6.74	1000	-	-	1.698924731	
Uqcrh	P99028	83	DHC(+57.02)VAHK(*)LFK(*)	K7	74.36	77.24	0.8303571	1.323076	0.863636	1.0056901
Gabpb1	Q00420	92	TPLHMAASEGHANIVEVLLK(*)HGAD	K20	21.55	47.82	-	-	1.3875	

Hmmr	Q00547	625	ITDLK(*)NQLR(*)	K5	45.18	1000	0.8235294	-	0.965517:	0.8945233
G6pdx	Q00612	471	IFTPLLHK(*)IDR(*)	K8	57.89	1000	0.90625	0.9125	0.921348:	0.9133661
Yy1	Q00899	401	FAQSTNLK(*)SHILTHAK(*)	K8	15.68	26.99	4.7666666	6.378378	3.712765!	4.9526037
Hnrnpul2	Q00PI9	404	VLC(+57.02)K(*)NC(+57.02)VVELNF	K4	55.21	257.85	1.6263736	0.837837	1.034883	1.1663651
Hnrnpul2	Q00PI9	504	GLEEPEMDPK(*)SR(*)	K10	35.7	1000	0.8021978	1.310810	0.848837	0.9872819
Hnrnpul2	Q00PI9	404	C(+57.02)K(*)NC(+57.02)VVELNFG(	K2	15.56	134.4	1.8791208	-	0.976744:	1.4279325
Hnrnpul2	Q00PI9	295	VC(+57.02)FEAK(*)VTQNLPMK(*)	K6	5.4	81.59	0.8351648	-	-	
Creb1	Q01147	323	VAVLENQNK(*)TLIEELK(*)	K9	19.26	78.16	-	-	1.384615385	
Col1a2	Q01149	1332	TNEWGK(*)TIIEYK(*)	K6	75.58	123.86	1.2857142	1.125	1.114942!	1.1752189
Col1a2	Q01149	1338	TIIEYK(*)TNK(*)PSR(*)	K6	70.4	68.47	0.9740259	1.015625	0.816091!	0.9352476
Col1a2	Q01149	1332	K(*)TNEWGK(*)TIIEYK(*)	K7	61	69.64	1.1168831	1.171875	0.908045!	1.0656014
Col1a2	Q01149	1230	AQANK(*)HVWLGETINGGSQFEYNVE	K5	36.48	89.28	-	-	0.850574713	
Col1a2	Q01149	980	GYPGSIGPTGAAGAPGPHGSVGPAGI	K26	16.96	1000	2.4545454	-	0.885057	1.6698015
Top2a	Q01320	1418	TATK(*)SQSSVSTAGTK(*)	K4	79.44	236.99	1.0315789	0.950617	1.139534!	1.040577
Top2a	Q01320	534	YGK(*)IMIMTDQDQDGSHIK(*)	K3	53.46	170.25	0.7578947	0.629629	1.023255!	0.8035934
Top2a	Q01320	190	MFK(*)QTWMDNMGR(*)	K3	26.37	1000	0.9263157	1.296296	1.034883	1.0858319
Top2a	Q01320	417	FK(*)AQIQLNK(*)	K2	7.73	93.6	-	-	1.151162791	
Sec23a	Q01405	185	MVQVHELGC(+57.02)EGISK(*)SYVF	K14	52.06	1000	0.5757575	1.026315	0.863636:	0.8219032
Sec23a	Q01405	392	VFTK(*)DIHGQFK(*)	K4	35.62	110.43	0.8383838	-	1.238636:	1.0385101
Rsu1	Q01730	89	LK(*)HLNLGMNR(*)	K2	76.84	1000	1.0108695	1.023255	1	1.0113751
Rsu1	Q01730	158	LTK(*)LQILSLR(*)	K3	67.73	1000	1	0.930232	0.847826(	0.9260195
Nme2	Q01768	49	ASEEHLK(*)QHYIDLK(*)	K7	76.41	186.64	1.6842105	0.888888	1	1.1910331
Nme2	Q01768	135	EIHLWFK(*)PEELIDYK(*)	K7	72.15	113.97	-	-	0.922222222	
Vcp	Q01853	251	GILLYGPPGTGK(*)TLIAR(*)	K12	77.9	1000	0.7083333	0.617283	0.835164!	0.7202607
Vcp	Q01853	386	LEILQIHTK(*)NMK(*)	K9	76.01	50.22	1.0729166	0.802469	0.967032!	0.9474729
Vcp	Q01853	524	GVLFYGPPGC(+57.02)GK(*)TLLAK(*	K12	66	56.94	0.9791666	1.530864	0.923076!	1.1443693
Uba1	Q02053	980	QFLDYFK(*)TEHK(*)	K7	82.16	51.96	1.0319148	0.923076	0.922222	0.9590713
Uba1	Q02053	89	GLGVEIAK(*)NIILGGVK(*)	K8	75.15	161.77	-	-	0.877777778	
Uba1	Q02053	528	LK(*)SDTAAAAVR(*)	K2	73.12	1000	1.1382978	1.243589	1.111111	1.1643329
Uba1	Q02053	296	GGIVSQVK(*)VPK(*)	K8	20.33	44.84	-	-	0.944444444	
Ctnnb1	Q02248	288	MVALLNK(*)TNVK(*)	K7	65.43	48.83	0.65625	0.623376	0.816326!	0.6986511
Col6a2	Q02788	254	VMK(*)HEAYGEC(+57.02)YK(*)	K3	50.98	99.51	0.7722772	0.493827	0.886597!	0.7175674
Col6a2	Q02788	135	ASFTK(*)SLQGIR(*)	K5	27.71	1000	3.6633663	10.41975	-	7.0415597
Nucb1	Q02819	301	EHVMK(*)NVDTNQDR(*)	K5	48.41	1000	1.4105263	0.974025	1.214285	1.1996127

Enah	Q03173	770	LK(*)QDILDEMR(*)	K2	52.4	1000	1.1590909	-	-	
Atp5f1a	Q03265	218	QTGK(*)TSIAIDTIINQK(*)	K4	80.7	242.08	0.9387755	0.795180	0.945652	0.8932028
Atp5f1a	Q03265	305	DNGK(*)HALIIYDDLSK(*)	K4	72.99	256.29	1.0204081	0.855421	1.282608	1.0528128
Atp5f1a	Q03265	45	QK(*)TGTAEMSSILEER(*)	K2	62.31	1000	0.6428571	0.698795	0.543478	0.6283769
Atp5f1a	Q03265	316	HALIIYDDLSK(*)QAVAYR(*)	K11	47.05	1000	-	-	0.836956522	
Atp5f1a	Q03265	503	LEPSK(*)ITK(*)	K5	6.47	22.45	4.6020408	-	-	
Atp5f1a	Q03265	506	ITK(*)FENAFLSHVISQHQSLLGNIR(*)	K3	5.21	1000	-	-	1.597826087	
Pfdn6	Q03958	25	DLSK(*)SMSGR(*)	K4	19.01	1000	1.1170212	-	0.884210	1.0006159
Rela	Q04207	310	TYETFK(*)SIMK(*)	K6	18.92	41.04	1.6375	0.926829	-	1.2821646
Nf1	Q04690	2173	VK(*)SAAVIAFR(*)	K2	41.84	1000	-	-	0.908045977	
Nf1	Q04690	2424	ILHTLLTLVNK(*)HR(*)	K11	34.18	1000	0.5555555	-	0.390804	0.4731801
Yes1	Q04736	236	LVK(*)HYTEHADGLC(+57.02)HK(*)	K3	12.15	126.66	-	-	1.295918367	
Top1	Q04750	577	LNTGILNK(*)HLQDLMEGLTAK(*)	K8	75.71	29.09	-	-	0.87628866	
Top1	Q04750	551	VFK(*)NLQLFMENK(*)	K3	73.92	203.33	-	-	0.783505155	
Top1	Q04750	722	QIALGTSK(*)LNYLDPR(*)	K8	47.59	1000	-	-	0.81443299	
Top1	Q04750	748	IYNK(*)TQR(*)	K4	29.71	1000	-	1.023255	0.814432	0.9188444
Top1	Q04750	293	EMTNDEK(*)NTITNLSK(*)	K7	23.84	75.74	1.1666666	0.825581	0.927835	0.973361
Top1	Q04750	652	SMMNLQSK(*)IDAK(*)	K8	23.18	48.12	-	1.034883	1.092783	1.0638336
Top1	Q04750	220	WK(*)FLEHK(*)	K2	9.97	78.33	-	0.534883	0.680412	0.607648
Top1	Q04750	23	LNDSHK(*)HK(*)	K6	5.8	39.76	-	-	1.298969072	
Col6a1	Q04857	987	VLVTGK(*)TAEYDVAFGER(*)	K6	47.7	1000	0.8947368	1.092105	-	0.9934211
Col6a1	Q04857	124	YFGK(*)GTYTDC(+57.02)AIK(*)	K4	47.44	113.07	0.8631578	0.855263	0.958333	0.8922515
Sox9	Q04887	249	TDVQAGK(*)VDLK(*)	K7	17.57	59.3	-	-	-	
Cdk18	Q04899	175	DLK(*)HANIVTLHDLIHTDR(*)	K3	17.42	1000	-	-	-	
Rcn1	Q05186	290	NK(*)DEMLTK(*)	K2	24.41	77.93	-	-	1.582417582	
Rcn1	Q05186	170	FK(*)ASDLDGDLTATR(*)	K2	16.29	1000	-	-	1.681318681	
Ptgs2	Q05769	197	FAQHFTHQFFK(*)TDHK(*)	K11	71.51	25.7	1.0618556	-	1.019607	1.0407318
Hspg2	Q05793	1001	GDK(*)VTSYGGELR(*)	K3	46.54	1000	0.4444444	14.42253	0.768292	5.2117574
Hspg2	Q05793	3673	FSSGITGC(+57.02)IK(*)NLVLHTAR(*	K10	35.85	1000	1.3209876	-	0.987804	1.1543963
Hspg2	Q05793	1795	SQSVR(*)PGADVTFIC(+57.02)TAK(*	K17	20.24	22.85	1.1111111	0.704225	1.109756	0.9750309
Hspg2	Q05793	606	QFLGNK(*)VDSYGGFLR(*)	K6	14.02	1000	-	-	1.207317073	
Fabp5	Q05816	10	DLEGK(*)WR(*)	K5	75.95	1000	-	-	0.896907216	
Fabp5	Q05816	55	AK(*)PDC(+57.02)IITC(+57.02)DGN	K17	67.06	69.64	-	0.597560	0.927835	0.762698
Fabp5	Q05816	40	AK(*)PDC(+57.02)IITC(+57.02)DGN	K2	41.78	118.22	-	1.878048	0.762886	1.3204677

Fabp5	Q05816	61	TESTVK(*)TTVFSC(+57.02)NLGEK(*)	K6	17.11	88.86	1.33	-	-	
Fabp5	Q05816	103	TETVC(+57.02)TFQDGALVQHQQWD	K21	9.12	1000	-	-	1.340206186	
Eml1	Q05BC3	274	HYAGHNDDVK(*)C(+57.02)LAVHPD	K10	36.61	1000	-	-	1.805555556	
Larp7	Q05CL8	377	VLSK(*)TEWMDLK(*)	K4	31.82	122.76	-	1.373333	0.902173	1.1377536
Eif5b	Q05D44	1091	MK(*)ILPQYIFNSR(*)	K2	39.36	1000	1.2473118	0.987179	0.908045	1.0475124
Eif5b	Q05D44	753	IVALNK(*)IDR(*)	K6	35.11	1000	1.1290322	0.897435	1.252873	1.0931139
Eif5b	Q05D44	1081	QEEFK(*)HIAVFPC(+57.02)K(*)	K5	29.13	101.72	0.5376344	1.192307	0.965517	0.8984864
Eif5b	Q05D44	1181	HFEATDILVSK(*)ISR(*)	K11	23.01	1000	0.6559139	-	1.770114	1.2130145
Cab39	Q06138	297	TQPILDILLK(*)NQTK(*)	K10	54.66	48.12	1.0674157	-	0.965909	1.0166624
Cab39	Q06138	196	HK(*)LLSAEFLEQHYDR(*)	K2	22.36	1000	1.3707865	1.929577	1.590909	1.6304244
Cab39	Q06138	45	ATEEVSK(*)NLVAMK(*)	K7	5.11	26.5	-	-	1.159090909	
Anxa7	Q07076	439	QM(+15.99)YQK(*)TLSTMIASDTSGD	K5	86.4	1000	1.5168539	-	0.903225	1.2100399
Anxa7	Q07076	405	LYYSMK(*)GAGTDDSTLVR(*)	K6	50.11	1000	-	0.797297	0.817204	0.8072508
Igf2r	Q07113	1994	HK(*)TYDLR(*)	K2	40.56	1000	1.1632653	-	1.233333	1.1982993
Igf2r	Q07113	178	LQK(*)HDLNPLIK(*)	K3	37.94	118.18	-	-	0.788888889	
Acads	Q07417	306	NAFGAPLTK(*)LQNIQFK(*)	K9	28.09	59.03	1.1979166	-	0.840425	1.0191711
Bax	Q07813	128	ALC(+57.02)TK(*)VPELIR(*)	K5	44.43	1000	0.7227722	-	0.759036	0.7409042
Cnn2	Q08093	19	GPSYGLSAEVK(*)NR(*)	K11	97.6	1000	0.8571428	0.789473	0.912087	0.8529015
Cnn2	Q08093	234	HIYDTK(*)LGTDK(*)	K6	80.53	115.97	0.8351648	0.697368	1	0.8441778
Cnn2	Q08093	74	LQPGSVPK(*)INR(*)	K8	79.29	1000	0.8681318	0.657894	0.934065	0.8200308
Cnn2	Q08093	134	TK(*)GLQSGVDIGVK(*)	K2	68.85	144.29	0.9010989	0.631578	0.868131	0.8002699
Cnn2	Q08093	25	LLSK(*)YDPQK(*)	K4	43.96	43.82	1.0329670	0.776315	0.923076	0.9107866
Cnn2	Q08093	194	HLYDPK(*)NHILPPMDHC(+57.02)TI	K6	37.12	53.37	-	-	0.802197802	
Lyar	Q08288	46	DFWGDDYK(*)SHVK(*)	K8	59.55	42.89	0.9174311	0.941176	0.927083	0.9285637
Lyar	Q08288	61	YGGK(*)GYEAK(*)	K4	54.32	115.97	-	-	1.364583333	
Lyar	Q08288	93	K(*)PNVSPK(*)VR(*)	K7	47.05	38.89	-	0.705882	1.197916	0.9518995
Lyar	Q08288	119	FQNWMK(*)NSLK(*)	K6	43.03	48.97	-	0.870588	1.15625	1.0134191
Zbtb14	Q08376	284	EQIAC(+57.02)QAC(+57.02)GK(*)TF	K10	17.47	1000	-	-	-	
Eps8	Q08509	336	FK(*)HGFNLLAK(*)	K2	61.47	159.25	1.0232558	0.942028	0.965116	0.9768004
Eps8	Q08509	160	EPTQSK(*)PDLHLFQC(+57.02)DEVK	K6	38.57	85.14	0.8837209	0.260869	1	0.7148635
Ssrp1	Q08943	90	LSDFFK(*)THYR(*)	K6	66.15	1000	-	-	0.791208791	
Ssrp1	Q08943	566	IK(*)SDHPGISITDLSK(*)	K2	65.18	137.95	0.8762886	0.891566	0.901098	0.8896513
Ssrp1	Q08943	304	LTK(*)NMSGSLYEMVSR(*)	K3	35.44	1000	-	-	1.340659341	
Inf2	Q0GNC1	715	LLPEK(*)HEIENLR(*)	K5	70.79	1000	1.0581395	0.923076	0.919191	0.9668028

Zc3h18	Q0P678	785	APAGGK(*)ASQQAAAPQPAVPGQPQ	K6	42.66	61.64	1.4285714	0.982758	0.857142	1.089491
Zc3h18	Q0P678	647	K(*)PAPPPAPPQATK(*)TTAPGPEPAK	K13	36.85	71.03	-	0.672413	1.357142	1.0147783
Rbis	Q0VG62	21	NVFHIASHK(*)TFK(*)	K9	46.81	57.45	-	-	-	
Ppp4r2	Q0VGB7	231	NK(*)HPDEDAVESEEHEVK(*)	K2	41.36	148.55	1	-	1.043010	1.0215054
Ppp4r2	Q0VGB7	231	NK(*)HPDEDAVESEEHEVK(*)R(*)	K2	16.97	109.25	-	-	0.989247312	
Rtel1	Q0VGM9	1162	TQSK(*)ISSFFR(*)	K4	39.59	1000	-	-	-	
Npepps	Q11011	822	ALSEEVR(*)PQDTVSVIGGVAGGSK(*)	K23	71.62	1000	-	0.802631	-	
Npepps	Q11011	337	ETALLIDPK(*)NSC(+57.02)SSSR(*)	K9	67.84	1000	0.7741935	0.578947	0.953488	0.7688764
Npepps	Q11011	262	SK(*)DGVC(+57.02)VR(*)	K2	65.74	1000	-	-	1.023255814	
Npepps	Q11011	737	EHVEGK(*)QILSADLR(*)	K6	43.49	1000	1.4516129	0.973684	0.895348	1.106882
Npepps	Q11011	737	FK(*)EHVEGK(*)QILSADLR(*)	K8	36.54	34.81	0.4086021	-	1.069767	0.7391848
Ptcd3	Q14C51	539	DK(*)HPPELQVAFADC(+57.02)AADI	K2	36.6	151.7	-	-	1.056179775	
Nsun2	Q1HFZ0	95	EILHC(+57.02)LK(*)NK(*)	K7	90.55	45.01	0.8817204	0.871794	0.978494	0.91067
Nsun2	Q1HFZ0	585	VINTGIK(*)VWC(+57.02)R(*)	K7	69.05	1000	1.5161290	1.025641	1.827956	1.4565757
Nsun2	Q1HFZ0	46	ENK(*)LFEHYYQELK(*)	K3	44.66	185.69	0.5483870	0.730769	1.139784	0.8063138
Specc1l	Q2KN98	262	EELNQLK(*)NENR(*)	K7	33.27	1000	0.9696969	1.260869	0.771084	1.0005503
Specc1l	Q2KN98	726	LQDQK(*)HDMER(*)	K5	5.84	1000	-	-	1.325301205	
Meaf6	Q2VPQ9	74	YLTNQK(*)NSNSK(*)NDR(*)	K11	98.29	1000	-	-	-	
Meaf6	Q2VPQ9	69	YLTNQK(*)NSNSK(*)NDR(*)	K6	98.29	1000	-	-	-	
Meaf6	Q2VPQ9	69	YLTNQK(*)NSNSK(*)	K6	96.81	96.85	-	-	-	
Meaf6	Q2VPQ9	6	A(+42.01)MHNK(*)TAPPQIPDTR(*)	K5	49.07	1000	-	-	-	
Dus4l	Q32M08	39	YSK(*)LAFR(*)	K3	10.42	1000	-	-	-	
Osbp	Q3B7Z2	228	VEDLSTC(+57.02)NDLIAK(*)HGTAL(	K13	71.54	1000	1.7340425	-	0.97	1.3520213
Emc10	Q3TAS6	220	NPQEQK(*)SFFAK(*)	K6	1.06	35.03	-	-	0.00862069	
Cul4a	Q3TCH7	561	TFYLGK(*)HSGR(*)	K6	88.39	1000	0.9479166	0.9	0.934065	0.9273275
Cul4a	Q3TCH7	465	LLVGK(*)SASVDAEK(*)	K5	87.14	161.77	0.75	0.85	0.879120	0.8263736
Cul4a	Q3TCH7	480	LK(*)HEC(+57.02)GAAFTSK(*)	K2	84.89	187.15	1.7708333	-	1.186813	1.4788233
Cul4a	Q3TCH7	668	IK(*)INQIQMK(*)	K2	68.51	149.73	0.78125	-	1.032967	0.9071085
Cul4a	Q3TCH7	555	LQEVFK(*)TFYLGK(*)	K6	57.67	121.92	0.7395833	0.5125	0.846153	0.6994124
Cul4a	Q3TCH7	330	VK(*)GGQHALLQHWSEYIK(*)	K2	38.77	120.4	0.8333333	-	1.131868	0.9826007
Cul4a	Q3TCH7	446	FIHGK(*)DVFEAFYK(*)	K5	30.79	159.25	1.4479166	-	1.131868	1.2898924
Cul4a	Q3TCH7	411	R(*)PNK(*)PAELIAK(*)HVDSK(*)	K11	26.7	35.03	1.1458333	-	0.923076	1.0344551
Cul4a	Q3TCH7	371	DK(*)VDHVVEVC(+57.02)FQR(*)	K2	26.2	1000	1.5520833	1.625	1.219780	1.4656212
Cul4a	Q3TCH7	490	LK(*)HEC(+57.02)GAAFTSK(*)LEGM	K12	25.55	72.73	-	-	0.89010989	

Plbd2	Q3TCN2	195	LTLLQLK(*)GLEDSYEGR(*)	K7	29.47	1000	-	-	1.021276596	
Khdc4	Q3TCX3	93	LQAPGK(*)SLTSNK(*)	K6	87.72	104.48	-	0.412587	-	
Stt3b	Q3TDQ1	751	FK(*)HLEEAFTSEHWLVR(*)	K2	47.8	1000	0.3714285	0.760869	0.988636	0.7069782
Hp1bp3	Q3TEA8	177	AC(+57.02)FQK(*)TGASVVAIR(*)	K5	34.11	1000	-	-	0.825581395	
Hp1bp3	Q3TEA8	302	VDIR(*)PQLLK(*)NALQR(*)	K9	5.62	1000	-	-	0.953488372	
Aarsd1	Q3THG9	235	NK(*)SNLIFLAGNR(*)	K2	53.03	1000	0.9195402	0.864864	1.117647	0.9673507
Aarsd1	Q3THG9	287	LLQK(*)NNLNLLR(*)	K4	26.4	1000	1	0.864864	0.647058	0.8373079
Eif1ad	Q3THJ3	90	AEISFVLC(+57.02)K(*)NHVR(*)	K9	39.09	1000	-	-	1.272727273	
Gtf2f1	Q3THK3	435	MDTGPQSLSGK(*)STPSSGDVQVTED	K11	38.16	1000	1.3092783	-	1.045977	1.1776277
Gtf2f1	Q3THK3	169	NK(*)VLNHFSIMQQR(*)	K2	29.99	1000	-	-	1.08045977	
Gtf2f1	Q3THK3	36	NAADK(*)VNFATWNQAR(*)	K5	23.54	1000	-	1.4	0.586206	0.9931034
Gmps	Q3THK7	389	AELIK(*)THHNDTELIR(*)	K5	55.23	1000	0.9361702	-	1.182795	1.059483
Gmps	Q3THK7	159	EEIVLLTHGDSVDK(*)VADGFK(*)	K14	40.19	33.66	1.0319148	-	1.032258	1.0320865
Mat2a	Q3THS6	303	WVAK(*)SLVK(*)	K4	77.23	44.28	-	0.915662	0.90625	0.9109563
Mat2a	Q3THS6	88	EAIK(*)HIGYDDSSK(*)	K4	68.11	77.76	-	-	0.770833333	
Mat2a	Q3THS6	289	DYTK(*)VDR(*)	K4	35.87	1000	-	0.891566	0.791666	0.8416165
Mat2a	Q3THS6	61	VAC(+57.02)ETVAK(*)TGMILLAGEIT	K8	12.11	1000	-	-	2.447916667	
H2afv	Q3THW5	14	AGK(*)DSGK(*)AK(*)AK(*)	K9	18.2	36.05	0.8645833	-	-	
H2afv	Q3THW5	12	AGK(*)DSGK(*)AK(*)AK(*)	K7	18.2	88.98	0.8645833	-	-	
H2afv	Q3THW5	8	AGK(*)DSGK(*)AK(*)AK(*)	K3	18.2	175.25	0.8645833	-	-	
Usp39	Q3TIX9	458	FTK(*)NNFFVEK(*)	K3	47.52	154.98	1.1034482	0.872340	0.958762	0.9781839
Pdlim7	Q3TJD7	238	YAPDK(*)TSTVLTR(*)	K5	40.56	1000	-	0.961038	-	
Smarca4	Q3TKT4	471	QK(*)HQEYLNSILQHAK(*)	K2	64.82	150.34	0.8333333	0.744186	0.978021	0.8518471
Smarca4	Q3TKT4	94	YNQMK(*)GMGMR(*)	K5	55.14	1000	0.8333333	0.720930	0.857142	0.8038021
Smarca4	Q3TKT4	1237	VIQAGMFDQK(*)SSSHER(*)	K10	31.91	1000	-	1.034883	0.736263	0.8855737
Smarca4	Q3TKT4	1033	GGTK(*)TLMNTIMQLR(*)	K4	19.46	1000	1.2777777	-	1.472527	1.3751526
Cwc27	Q3TKY6	126	ADELNNK(*)HTIFGK(*)	K7	49.21	85.52	1.0784313	-	0.987951	1.0331916
Cwc27	Q3TKY6	299	DASASVK(*)SAGDGEK(*)	K7	16.3	62.93	1.2745098	0.61	2.253012	1.379174
Cwc27	Q3TKY6	230	SK(*)SSHDLLK(*)	K2	13.22	100.04	-	1.66	2.180722	1.9203614
Prrc2c	Q3TLH4	49	HGLQSLGK(*)VGISR(*)	K8	64.66	1000	1.0434782	0.731343	0.896551	0.8904578
Micall2	Q3TN34	193	AAPGTAGSSVSSIC(+57.02)GVC(+57	K19	52.2	1000	1.5189873	0.692307	1.056818	1.0893711
	Q3TQI7	6	ITGK(*)TFR(*)	K4	99.42	1000	1.8076923	1.254545	1.171428	1.4112221
Tex30	Q3TUU5	86	AVLNYLK(*)TSGEYK(*)	K7	17.68	35.17	1.9480519	-	-	
Wdr70	Q3TWF6	517	LC(+57.02)VVK(*)TQR(*)	K5	27.1	1000	-	-	0.597222222	

Pip4p1	Q3TWL2	185	VIC(+57.02)GHC(+57.02)K(*)NTFLV	K7	40.55	1000	-	-	1.586206897	
Trmt1	Q3TX08	569	AR(*)PGGK(*)AASEDLAGR(*)	K6	40.1	1000	0.8241758	-	0.805194	0.8146853
Trmt1	Q3TX08	482	VSLSHAC(+57.02)K(*)NAVK(*)	K8	14.55	41.04	-	1.479452	1.324675	1.4020637
Psmd1	Q3TXS7	720	VINDK(*)HDDVMAK(*)	K5	68.37	186.64	0.9456521	0.779220	0.913978	0.8796171
Psmd1	Q3TXS7	354	NNNTDLMILK(*)NTK(*)	K10	60.01	22.34	1.4782608	1.025974	1	1.1680783
Psmd1	Q3TXS7	715	QLYSK(*)VINDK(*)	K5	55.97	68.6	0.9456521	0.870129	1.096774	0.9708521
Cybc1	Q3TYS2	81	VILK(*)TFSLYK(*)	K4	40.34	76.27	0.8409090	0.644736	0.974025	0.8198906
Smyd5	Q3TYX3	34	GK(*)GLFATQLIR(*)	K2	74.17	1000	0.6565656	0.916666	0.784090	0.7857744
Smyd5	Q3TYX3	182	DK(*)DHWVR(*)	K2	44.05	1000	0.8989898	-	1.102272	1.0006313
Nol9	Q3TZX8	255	FVDFLK(*)TYK(*)	K6	9.66	20.7	-	1.290697	-	
Esyt2	Q3TZZ7	335	GK(*)SDPYGIIR(*)	K2	11.45	1000	-	1.1	-	
Tbc1d2b	Q3U0J8	658	EMAC(+57.02)SPELK(*)NLIR(*)	K9	17.04	1000	-	0.655172	-	
Ogfod1	Q3U0K8	446	WK(*)TGHYTLVHDNTK(*)	K2	31.11	142.32	1.5056179	0.369565	1.207792	1.0276585
Khsrp	Q3U0V1	360	IQFK(*)QDDGTGPEK(*)	K4	62.33	140.39	0.6847826	1.066666	0.780219	0.8438897
Khsrp	Q3U0V1	170	GGEQINK(*)IQQDSGC(+57.02)K(*)	K7	43.67	150.63	-	0.346666	0.736263	0.5414652
Khsrp	Q3U0V1	252	AGLVIGK(*)GGETIK(*)	K7	39.72	41.24	0.7282608	0.813333	-	0.7707971
Ddb1	Q3U1J4	191	HVK(*)TYEVSLR(*)	K3	80.92	1000	0.9484536	0.911392	0.876288	0.9120449
Ddb1	Q3U1J4	141	LYDGLFK(*)VIPLDR(*)	K7	74.65	1000	-	-	0.917525773	
Ddb1	Q3U1J4	1067	SVGK(*)IEHSFWR(*)	K4	64.01	1000	0.9793814	0.936708	0.865979	0.9273566
Ddb1	Q3U1J4	915	TEC(+57.02)NHYNNIMALYLK(*)TK(*	K15	51.23	22.85	1.0103092	1.455696	0.835051	1.1003523
Ddb1	Q3U1J4	917	TK(*)GDFILVGDLMR(*)	K2	48.77	1000	0.9072164	-	0.969072	0.9381443
Sec24a	Q3U2P1	907	QK(*)SFQTGTSIR(*)	K2	30.54	1000	2.65625	2.211267	1.069767	1.979095
Rnf40	Q3U319	250	LQDLATQLQEK(*)HHR(*)	K11	58.15	1000	1.0340909	1.285714	0.949494	1.0897667
Rnf40	Q3U319	420	GLLLASK(*)NSHLR(*)	K7	47.94	1000	0.9886363	0.928571	0.828282	0.9151635
	Q3U4G0	50	FPSQSQATLLSIFSQEYQK(*)HIK(*)	K19	69.34	32.97	-	-	-	
Wdr75	Q3U821	621	PLYIQK(*)NVSR(*)	K6	89.93	1000	1.0365853	0.881578	0.886363	0.9348426
Wdr75	Q3U821	213	FTC(+57.02)VAC(+57.02)HPK(*)ED(	K9	37.33	40.04	1.0365853	-	1	1.0182927
Wdr75	Q3U821	135	LPK(*)STSQDVEAR(*)	K3	28.35	1000	-	-	0.920454545	
Col5a2	Q3U962	1415	NTVGYMDDQAK(*)NLK(*)	K11	22.72	24.24	2.2903225	-	-	
Col5a2	Q3U962	1457	NGNVGK(*)TIFEYR(*)	K6	8.32	1000	-	-	0.916666667	
Trip13	Q3UA06	185	VVLLHGPPGTGK(*)TSLC(+57.02)K(*	K12	66.26	70.86	0.9156626	0.714285	0.915789	0.8485793
Spc25	Q3UA16	67	MTEMILEYK(*)NQLC(+57.02)K(*)	K9	18.66	43.82	7.2571428	-	3.517647	5.387395
Qrich1	Q3UA37	638	YFLLK(*)TVDQHMK(*)	K5	11.52	100.04	-	2.181818	1.392857	1.7873377
Tmem109	Q3UBX0	215	SSGSHLEAK(*)VR(*)	K9	69.44	1000	-	1.4	0.855670	1.1278351

Otulin	Q3UCV8	180	FDGK(*)SEDLVEK(*)	K4	7.84	38.64	-	-	1.654320988	
Puf60	Q3UEB3	173	SIDMSWDSVTMK(*)HK(*)	K12	90.81	24.32	0.9468085	0.7875	0.978021	0.9041102
Puf60	Q3UEB3	258	IK(*)SC(+57.02)TLAR(*)	K2	78.64	1000	-	0.8875	1.065934	0.976717
Puf60	Q3UEB3	272	HK(*)GYGFIEYEK(*)	K2	51.34	178.84	1.1276595	-	-	
Puf60	Q3UEB3	85	YAMEQSIK(*)SVLVK(*)	K8	39.77	56.51	-	1.3	1.318681	1.3093407
Zyg11b	Q3UFS0	237	ELK(*)HLNHLDISDDK(*)	K3	32.11	56.41	2.3333333	-	1.421875	1.8776042
Gpatch11	Q3UFS4	107	TGK(*)SGIGHESSLK(*)	K3	18.12	137.5	-	-	0.826086957	
Nt5c3b	Q3UFY7	205	GQLIHTYNK(*)NSSVC(+57.02)ENSS	K9	29	163.39	1.3853211	1.256410	1.148148	1.2632932
Dip2b	Q3UH60	273	VSTK(*)IQQLLNTLK(*)	K4	35.02	178.84	-	-	1.011494253	
Snx27	Q3UHD6	46	IVK(*)SESGYGFNVR(*)	K3	50.75	1000	-	-	0.780821918	
Zswim8	Q3UHH1	593	VLGGTGSGGK(*)SSAGSGSK(*)	K10	15.43	75.74	-	-	1.085714286	
Nol8	Q3UHX0	156	VLPVLHLK(*)NQQK(*)	K8	42.44	48.12	-	-	1.166666667	
Pdap1	Q3UHX2	132	MHLAGK(*)TEQAK(*)	K6	75.13	119.54	1.2555555	1.027027	1.113636	1.132073
Pdap1	Q3UHX2	137	TEQAK(*)ADLAR(*)	K5	16.01	1000	-	0.783783	1.011363	0.8975737
Ndufb6	Q3UIU2	58	DGAVWK(*)NMVFK(*)	K6	36.8	80.41	-	-	2	
Edc4	Q3UJB9	1097	SK(*)NLTDAIAR(*)	K2	38.61	1000	1.5666666	-	-	
Edc4	Q3UJB9	1388	LFQFLQADPHNSLSK(*)AAR(*)	K15	6.83	1000	-	-	1.311827957	
Tax1bp1	Q3UKC1	47	SK(*)DWVGIFK(*)	K2	23.44	109.53	-	-	0.881355932	
Smu1	Q3UKJ7	214	FGQK(*)SHVEC(+57.02)AR(*)	K4	32.65	1000	1.2307692	1.08	1.081395	1.1307215
Ppp1r7	Q3UM45	102	VK(*)SLC(+57.02)LR(*)	K2	82.2	1000	0.9456521	0.855263	1.032967	0.9446275
Washc4	Q3UMB9	813	TSNNK(*)HLNTINIR(*)	K5	23.13	1000	1.21875	-	1.094736	1.1567434
Cobll1	Q3UMF0	309	EQTASAPATPLVSK(*)HR(*)	K14	11.78	1000	1.6746987	1.211267	1.176470	1.3541457
Cdk10	Q3UMM4	178	GC(+57.02)VK(*)TADFGLAR(*)	K4	48.32	1000	-	-	-	
Hdgfl2	Q3UMU9	550	VLGPK(*)VEALQK(*)	K5	22.64	37.35	2.0430107	-	1.534883	1.7889472
Hdgfl2	Q3UMU9	483	FALK(*)VDNPDVR(*)	K4	11.51	1000	1.9569892	4.84	2.906976	3.2346553
Lclat1	Q3UN02	366	FLHK(*)HPHLNSK(*)	K4	39.64	148.49	1.1686746	1.128571	0.903614	1.0669535
Lclat1	Q3UN02	112	IC(+57.02)LK(*)SSLK(*)	K4	37.84	52.86	0.6024096	1	0.855421	0.8192771
Zc3hav1	Q3UPF5	222	EHGLSSDVVQNIQDIC(+57.02)NNK(	K19	53.92	1000	0.7924528	-	1.126315	0.9593843
Zc3hav1	Q3UPF5	619	GC(+57.02)QQVNC(+57.02)NK(*)N	K9	11.19	1000	1.2075471	0.948717	-	1.0781326
Sec31a	Q3UPL0	646	EIVESC(+57.02)DLK(*)NWR(*)	K9	73.87	1000	1.0744680	0.779220	0.916666	0.9234518
Sec31a	Q3UPL0	509	IALALNK(*)VDGPDVALK(*)	K7	63.76	153.19	0.8723404	-	0.84375	0.8580452
Sec31a	Q3UPL0	660	AK(*)PDEFSALC(+57.02)DLLGTR(*)	K2	60	1000	-	-	0.989583333	
Sec31a	Q3UPL0	71	YHK(*)LIWGPHK(*)	K3	48.84	142.27	1.0319148	0.740259	0.927083	0.8997527
Sec31a	Q3UPL0	487	GK(*)YLELLGYR(*)	K2	43.31	1000	-	-	0.96875	

Sec31a	Q3UPL0	634	LITAVVMK(*)NWR(*)	K8	42.29	1000	0.7978723	1.051948	0.989583	0.9464679
Sec31a	Q3UPL0	619	YFAK(*)SQSK(*)	K4	29.52	52.34	1.0957446	-	0.71875	0.9072473
Sec31a	Q3UPL0	619	K(*)YFAK(*)SQSK(*)	K5	28.34	22.99	-	1.259740	0.791666	1.0257035
Sec31a	Q3UPL0	477	LK(*)VNFEEDSR(*)	K2	25.82	1000	0.9255319	1.428571	0.729166	1.0277567
Tex10	Q3URQ0	528	FFSK(*)IYQK(*)	K4	14.33	41.04	-	1.904109	1.443396	1.6737529
Ovos	Q3UU35	1071	QK(*)SNGC(+57.02)FR(*)	K2	5.18	1000	-	-	-	
Tbc1d24	Q3UUG6	319	ALR(*)QK(*)GITVK(*)QK(*)	K10	1.79	32.28	-	-	-	
Tbc1d24	Q3UUG6	314	ALR(*)QK(*)GITVK(*)QK(*)	K5	1.79	71.72	-	-	-	
Ncbp1	Q3UYV9	574	FHEVFK(*)TLAESDK(*)	K6	52.78	81.81	1.1789473	1.070588	0.819148	1.0228948
Lrrfip1	Q3UZ39	168	EK(*)HAHSILQFQFAEVK(*)	K2	70.71	179.71	-	0.955223	1.096385	1.0258047
Lrrfip1	Q3UZ39	394	GSFEPC(+57.02)PDYILGQTAEIDK(*)	K19	54.59	1000	-	-	0.879518072	
Lrrfip1	Q3UZ39	233	EELNALK(*)SAGEGTLGK(*)	K7	27.24	72.62	1.4268292	0.582089	1.397590	1.1355031
Esf1	Q3V1V3	832	NK(*)TEQFQAR(*)	K2	21.97	1000	-	1.441558	-	
Mthfd1l	Q3V3R1	890	DIELSPEAQSK(*)IDR(*)	K11	54.76	1000	0.6276595	0.821917	0.648351	0.6993097
Mthfd1l	Q3V3R1	546	LGIHK(*)TDPSTLTEEEVR(*)	K5	46.41	1000	0.7765957	-	0.725274	0.7509352
Srbd1	Q497V5	542	TLMGVDPGYK(*)HGC(+57.02)K(*)	K10	39.92	28.74	-	1.418604	-	
Pds5b	Q4VA53	528	AIFSK(*)VMVITR(*)	K5	75.87	1000	0.6274509	0.614457	0.888888	0.7102659
Pds5b	Q4VA53	964	QC(+57.02)LVK(*)NITVR(*)	K5	70.06	1000	0.7254901	0.698795	1.141414	0.8552332
Pds5b	Q4VA53	693	VAEAALQIFK(*)NTGSK(*)	K10	41.8	32.32	0.4313725	0.975903	0.929292	0.7788564
Pds5b	Q4VA53	840	WLLGMK(*)NNHSK(*)	K6	41.43	23.49	-	-	0.878787879	
Pds5b	Q4VA53	121	GLEDTK(*)SPQFNR(*)	K6	15.48	1000	1.0784313	1.253012	1.151515	1.1609862
Cdv3	Q4VAA2	226	K(*)TPQGPPEIYSDTQFPSLQSTAK(*)	K23	56.24	146.29	0.6739130	1.260273	1.192771	1.0423194
Ddx17	Q501J6	234	LIDFLESGK(*)TNLR(*)	K9	73.23	1000	0.8842105	0.975609	0.923076	0.9276324
Ddx17	Q501J6	341	ENK(*)TIIFVETK(*)	K3	72.01	71.6	0.6631578	0.560975	0.758241	0.6607918
Ddx17	Q501J6	53	FEK(*)NFYVEHPEVAR(*)	K3	64.28	1000	0.9368421	0.792682	0.945054	0.8915267
Ddx17	Q501J6	436	STNK(*)GTAYTFFTPGNLK(*)	K4	35.86	123.75	0.5894736	-	0.538461	0.5639676
Phactr4	Q501J7	686	SSEMEVHVDSK(*)HFTR(*)	K11	12.05	1000	-	-	2.241935484	
Ankrd28	Q505D1	95	AVASC(+57.02)SEEAVQILLK(*)HSAD	K15	29.76	1000	-	-	1.074766355	
Ccnl1	Q52KE7	205	ELGFC(+57.02)VHVK(*)HPHK(*)	K9	17.56	38.26	-	-	1.680851064	
Ddx46	Q569Z5	776	SSGFSGK(*)GFK(*)	K7	51.63	57.45	0.93	-	-	
Ddx46	Q569Z5	263	SGPTVTK(*)VVTVVTTK(*)	K7	50.76	118.18	0.88	0.701298	1.032258	0.8711856
Ddx46	Q569Z5	470	FSK(*)TLGLR(*)	K3	38.44	1000	0.72	-	-	
Thrap3	Q569Z6	555	GDFSSGK(*)SSFSITR(*)	K7	91.97	1000	1.0659340	1.041666	0.882978	0.9968598
Thrap3	Q569Z6	452	FMSK(*)VIAGASK(*)	K4	71.54	128.65	1.0769230	1.194444	1	1.0904558

Thrap3	Q569Z6	548	LGSK(*)GDFSSGK(*)	K4	26.76	86.63	-	1.027777	-	
Thrap3	Q569Z6	468	SGK(*)WESLHTGK(*)	K3	11.95	113.97	-	1.055555	-	
Urb1	Q571H0	1798	DK(*)HC(+57.02)YELC(+57.02)SR(*)	K2	51.49	1000	1.7457627	-	1.289156(	1.5174597
Spag9	Q58A65	431	NALNVVK(*)NDLIAK(*)	K7	21.71	95.35	1.1777777	1.1	1	1.0925926
Spag9	Q58A65	1059	C(+57.02)MTVVHDK(*)VWC(+57.02	K8	21.41	1000	-	-	1.59375	
Ndufaf2	Q59J78	101	IK(*)SQDFYEK(*)	K2	20.26	147.63	1.5270270	0.473684	1.528571	1.1764276
Tut7	Q5BLK4	157	R(*)LFQK(*)DLSSLEAM(+15.99)SEM	K5	4	46.26	0.0225563	-	-	
Kansl1l	Q5DTI6	863	AYSK(*)NVEGQDLVLK(*)	K4	96.86	251.58	-	-	-	
Kansl1l	Q5DTI6	852	WSLWEQSK(*)WHR(*)	K8	25.26	1000	-	-	-	
Usp22	Q5DU02	59	AK(*)SC(+57.02)VC(+57.02)HVC(+5	K2	57.93	1000	-	-	-	
Ehmt1	Q5DW34	430	GK(*)TDSPWIK(*)	K2	28.39	120.29	0.7530864	-	-	
Prkaa1	Q5EG47	280	EHEWFK(*)QDLPK(*)	K6	41.19	68.6	-	-	0.781609195	
Tmem256	Q5F285	46	ANK(*)HHFLHSLALLGVPSC(+57.02)	K3	41.75	1000	1.2	-	-	
Nufip2	Q5F2E7	664	AAIVYHTK(*)EMESIWNLQK(*)	K8	33.88	150.82	-	-	0.811111111	
Nufip2	Q5F2E7	293	GK(*)PAMGDVLR(*)	K2	15.11	1000	1.7571428	1.1	-	1.4285714
Arhgap1	Q5FWK3	218	YDDFLK(*)STQK(*)	K6	29.57	38.26	-	-	0.779069767	
Vps13a	Q5H8C4	39	GAVALK(*)NLVIK(*)	K6	46.15	127.18	0.5762711	0.331081	0.590062	0.4991381
Taf3	Q5HZG4	266	PLETK(*)SFTPK(*)	K5	55.73	65.71	-	-	-	
Ttf2	Q5NC05	790	LSILTK(*)SLLLR(*)	K6	37.71	1000	-	-	0.887640449	
Trappc1	Q5NCF2	70	YK(*)LHYYETPTGIK(*)	K2	42.31	122.88	-	-	1.127906977	
Mdc1	Q5PSV9	947	LSC(+57.02)QTTPAGK(*)ASR(*)	K10	17.88	1000	-	1.361111	-	
Mdc1	Q5PSV9	1418	TVPLGHK(*)SYSALSEPEPQSSASQSS(	K7	9.7	1000	-	0.930555	-	
Igf2bp2	Q5SF07	210	EGLTIK(*)NITK(*)	K6	34.39	28.74	0.8172043	0.666666	1.102040	0.8619706
Rbm27	Q5SFM8	734	MMGK(*)PQTSGAYVLNK(*)	K4	8.27	73.38	-	1.465346	-	
Tom1l2	Q5SRX1	106	IISPK(*)NNPPTIVQDK(*)	K5	31.51	133.99	1.0514705	0.691056	0.641666(	0.7947314
Psme4	Q5SSW2	156	ILYSK(*)TEHLR(*)	K5	51.21	1000	0.9195402	0.955882	1.202531(	1.0259847
Psme4	Q5SSW2	908	IIGDLLHFQGSHK(*)HEFDSR(*)	K13	34.28	1000	0.9195402	-	1.088607	1.0040739
Psme4	Q5SSW2	1499	LTQVYK(*)NVR(*)	K6	26.9	1000	0.9195402	1.205882	1.227848	1.1177569
Psme4	Q5SSW2	916	WK(*)SFNLVK(*)	K2	12.08	95.35	-	1.573529	-	
Ctc1	Q5SUQ9	913	MK(*)PGNAGAIK(*)	K2	22.04	46.68	-	1.132530	1.255813	1.194172
Ctc1	Q5SUQ9	921	MK(*)PGNAGAIK(*)TGVK(*)	K10	17.12	26.57	1.1262135	1.048192	-	1.0872032
Pfas	Q5SUR0	682	YLTNK(*)VDR(*)	K5	59.19	1000	0.7582417	1.519480	1.197674	1.1584656
Pfas	Q5SUR0	236	HWFFK(*)GQLHVDGK(*)	K5	49.73	135.81	0.9780219	1.168831	0.965116	1.0373231
Pfas	Q5SUR0	847	HPGGK(*)GHLLYVPLSPGQHR(*)	K5	21.36	1000	1.1868131	1.493506	1.686046	1.4554554

Kat7	Q5SVQ0	201	C(+57.02)PTPGC(+57.02)NSLGHLT(	K15	70.47	1000	0.75	0.504672	0.877777	0.7108169
Tbc1d9b	Q5SVR0	831	AK(*)HLASQYWGGNR(*)	K2	19.6	1000	5.2142857	0.231660	0.647058	2.0310016
Cluh	Q5SW19	824	SAK(*)HIFK(*)	K3	25.21	97.69	0.875	0.780487	0.802083	0.8191904
Cluh	Q5SW19	1161	VYESK(*)AEFR(*)	K5	12.87	1000	0.5384615	-	-	
Tsr1	Q5SWD9	130	MLLC(+57.02)PSLK(*)HR(*)	K8	47.16	1000	0.4720496	0.461538	0.875	0.6028627
Tsr1	Q5SWD9	759	LK(*)SQDTVLMNLYK(*)	K2	29	194.69	0.6583850	1.021978	0.413461	0.6979416
Acaca	Q5SWU9	740	ITIGNK(*)TC(+57.02)VFEK(*)	K6	80.12	112.31	-	0.721518	1.091954	0.9067365
Acaca	Q5SWU9	1315	EFTQQNK(*)ATLVEHGIR(*)	K7	74.24	1000	0.6413043	0.455696	0.908045	0.6683488
Acaca	Q5SWU9	2208	GVINDILDWK(*)TSR(*)	K10	34.78	1000	-	-	1.206896552	
Acaca	Q5SWU9	1434	HSDLVTK(*)EASFEYLQNEGER(*)	K7	21.3	1000	2.75	-	-	
Acaca	Q5SWU9	2134	DLVK(*)TMR(*)	K4	10.72	1000	-	-	0.91954023	
Tmprss13	Q5U405	516	NK(*)PGVYTK(*)	K2	20.96	70.1	-	-	-	
Dnajc11	Q5U458	45	LC(+57.02)MLYHPDK(*)HR(*)DPELk	K9	8.08	34.75	-	-	1.847826087	
Utp20	Q5XG71	1855	VC(+57.02)VLLK(*)NR(*)	K6	52.88	1000	0.5196078	0.807692	0.895833	0.7410445
Utp20	Q5XG71	2096	LLHLSLK(*)TSR(*)	K7	39.72	1000	1.1568627	1.025641	1.3125	1.1650013
Utp20	Q5XG71	278	TMAK(*)SSVVYIYK(*)	K4	27.09	150.89	1.2549019	1.256410	-	1.2556561
Utp20	Q5XG71	1150	FINPLK(*)NLR(*)	K6	25.89	1000	0.7647058	1.384615	-	1.0746606
Utp20	Q5XG71	1760	EQEC(+57.02)ISK(*)SVSFLPR(*)	K7	20.02	1000	1.6372549	1.820512	1.260416	1.5727281
Utp20	Q5XG71	2356	SLLSK(*)VDR(*)	K5	18.33	1000	-	0.923076	0.572916	0.7479968
Utp20	Q5XG71	2530	MK(*)SISLASC(+57.02)HQLHSK(*)	K2	16	144.36	-	-	0.895833333	
Utp20	Q5XG71	2351	MASMAIK(*)SLLSK(*)	K7	14.28	47.14	-	-	0.8125	
Arcn1	Q5XJY5	263	TSEATK(*)VHAPPINMESVHMK(*)	K6	51.2	56.26	1.1052631	1.025641	0.879120	1.0033417
Arcn1	Q5XJY5	44	LMNTGK(*)QHTFVETESVR(*)	K6	44.25	1000	0.8526315	0.628205	0.901098	0.7939785
Arcn1	Q5XJY5	243	LK(*)SEGETIMSSNMGK(*)	K2	36.18	187.28	1.1052631	0.679487	0.956043	0.9135981
Sin3a	Q60520	155	EFK(*)SQSIDTPGVISR(*)	K3	80.31	1000	0.7410714	1.157142	0.929411	0.942542
Xrcc1	Q60596	171	VTVTK(*)LGQFR(*)	K5	64.1	1000	1.0427350	0.6875	0.58	0.7700783
Ogdh	Q60597	74	SVHK(*)SWDIFFR(*)	K4	32.32	1000	1.1089108	-	0.548387	0.828649
Ogdh	Q60597	907	LLFC(+57.02)TGK(*)VYYDLTR(*)	K7	26.85	1000	1.2376237	-	1.010752	1.1241882
Ogdh	Q60597	860	K(*)PLIVFTPK(*)SLLR(*)	K9	19.1	135.81	1.0891089	1.123456	0.913978	1.0421814
Cttn	Q60598	42	WGAK(*)TVQGSGHQEHINIHK(*)	K4	73.67	106.28	0.9183673	0.909090	0.945652	0.9243701
Cttn	Q60598	110	LSK(*)HC(+57.02)SQVDSVR(*)	K3	67.15	1000	1.0714285	0.870129	0.836956	0.9261717
Cttn	Q60598	346	TVPIEAVTSK(*)TSNIR(*)	K10	64.5	1000	1.0306122	1.012987	1	1.0145331
Cttn	Q60598	208	VDK(*)SAVGFEYQGK(*)	K3	60.87	187.15	1.1122448	0.909090	0.847826	0.9563873
Cttn	Q60598	181	SAVGFDYQGK(*)TEK(*)	K10	58.44	32.97	-	-	0.902173913	

Cttn	Q60598	144	SAVGFEYQGK(*)TEK(*)	K10	53.26	28.96	1.3265306	1.909090	0.978260	1.4046275
Cttn	Q60598	295	LAK(*)HESQQDYAK(*)	K3	46.21	166.66	1.0816326	0.753246	0.869565	0.9014815
Cttn	Q60598	107	SAVGHEYQSK(*)LSK(*)	K10	40.39	24.24	1.4285714	0.974025	0.869565	1.0907209
Cttn	Q60598	144	VDQSAVGFEYQGK(*)TEK(*)	K13	38.89	26.52	0.6020408	0.883116	1.271739	0.9189656
Myl6	Q60605	56	VLGNPK(*)SDEMNVK(*)	K6	98.44	136.94	0.82	0.8	0.954545	0.8581818
Myl6	Q60605	81	NK(*)DQGTYEDYVEGLR(*)	K2	97.25	1000	1.06	1.1375	1.011363	1.0696212
Myl6	Q60605	26	TGDGK(*)ILYSQC(+57.02)GDVMR(*	K5	69	1000	1	0.575	1.125	0.9
Grb2	Q60631	50	DGFIPK(*)NYIEMK(*)	K6	49.37	60.84	0.9126213	0.9	0.757575	0.8567324
Clpb	Q60649	628	ITVEDSDK(*)HLLK(*)	K8	29.61	82.24	2.5192307	1.082352	0.783132	1.4615721
Klra4	Q60651	125	DQNILC(+57.02)DK(*)TR(*)	K8	43.66	1000	3.2985074	-	-	
Hnrnpd	Q60668	153	ESESVDK(*)VMDQK(*)	K7	38.55	100.21	-	-	1.860465116	
Hnrnpd	Q60668	72	IDASK(*)NEEDEGHSNSSPR(*)	K5	16.06	1000	-	-	2.034883721	
Hnrnpd	Q60668	251	YHNVGLSK(*)C(+57.02)EIK(*)	K8	6.9	52.86	1.6907216	1.226666	1.523255	1.4802147
Chuk	Q60680	466	MK(*)NTLISASQQLK(*)	K2	38.51	194.28	-	0.969387	0.496	0.7326939
Psmb6	Q60692	66	VTDK(*)LTPIHDHIFC(+57.02)C(+57.	K4	79.76	1000	0.8854166	0.858974	1.216867	0.9870862
Samhd1	Q60710	609	SLDAAGK(*)HFVQWC(+57.02)ALR(	K7	54.99	1000	1.0543478	0.846153	1.032258	0.9775866
Samhd1	Q60710	378	IC(+57.02)EVEYK(*)VK(*)	K7	50.69	33.18	0.9456521	0.961538	0.795698	0.9009632
Samhd1	Q60710	326	GR(*)PATK(*)SFLYEIVSNK(*)	K6	49.33	213.35	0.8804347	-	0.784946	0.8326905
Samhd1	Q60710	383	VK(*)EDK(*)TYIR(*)	K5	46.41	91.37	-	-	0.967741935	
Samhd1	Q60710	641	WNNK(*)TSSC(+57.02)LQEVSK(*)	K4	44.7	153.52	0.7173913	0.576923	1.043010	0.7791084
P4ha1	Q60715	382	ISK(*)SAWLSGYEDPVVSR(*)	K3	89.38	1000	0.9157894	0.776315	0.977528	0.8898778
P4ha1	Q60715	159	GNLPGVQHK(*)SFLTAEDC(+57.02)F	K9	62.68	229.43	0.7789473	-	0.966292	0.8726198
P4ha1	Q60715	288	QK(*)YEMLC(+57.02)R(*)	K2	48.99	1000	1.5368421	1.315789	1.303370	1.3853341
P4ha2	Q60716	385	VSK(*)SSWLEEDDDPVVAR(*)	K3	70.33	1000	-	0.698795	0.858585	0.7786905
P4ha2	Q60716	61	IK(*)SWASK(*)	K2	9.56	56.94	-	1.361445	-	
Bag1	Q60739	103	SK(*)NVTGTQVEEVTK(*)	K2	10.25	54.4	-	2.283950	-	
Khdrbs1	Q60749	152	K(*)DDEENYLDLFSHK(*)NMK(*)	K14	73.34	56.34	1.3085106	0.792207	1.409638	1.170119
Khdrbs1	Q60749	200	ISVLGK(*)GSMR(*)	K6	72.71	1000	0.8723404	0.766233	1.108433	0.9156693
Igf1r	Q60751	503	FTSTTTWK(*)NR(*)	K8	33.62	1000	0.8135593	1.092307	0.801724	0.9025304
Grb10	Q60760	613	LK(*)HHC(+57.02)IR(*)	K2	65.25	1000	1.0813953	-	1.068965	1.0751804
Irgm1	Q60766	275	IVGDK(*)VAVWK(*)	K5	54.79	112.56	0.9010989	0.88	0.988235	0.9231114
Irgm1	Q60766	289	IANESLK(*)NSLGVR(*)	K7	38.19	1000	0.8351648	1.16	0.929411	0.9748589
Ly75	Q60767	1526	LIFHVK(*)SSK(*)	K6	41.48	29.32	0.6588235	-	0.833333	0.7460784
Ly75	Q60767	612	WEVK(*)NC(+57.02)R(*)	K4	27.87	1000	-	-	1.155555556	

Ly75	Q60767	876	WWVK(*)TSENPIDR(*)	K4	25.67	1000	-	-	0.622222222	
Stxbp3	Q60770	19	IK(*)TAVFDDC(+57.02)R(*)	K2	27.49	1000	-	1.670731	2.172839	1.9217856
Dvl2	Q60838	58	YFFK(*)SMDQDFGVVK(*)	K4	21.21	58.64	-	1.246376	-	
Hells	Q60848	58	LQHLLEK(*)SNIYSK(*)	K7	49.44	90.32	0.9223300	0.820224	0.790909	0.844488
Hells	Q60848	97	LTEGK(*)SLVDGNGEK(*)PVMK(*)	K5	12.51	48.12	1.2330097	0.786516	0.990909	1.0034786
Serpinb6	Q60854	100	LFGDK(*)TC(+57.02)DLLASFK(*)	K5	80.46	178.24	0.9468085	0.753246	0.956989	0.8856815
Serpinb6	Q60854	193	VSK(*)NEEK(*)PVQMMFK(*)	K3	70.52	79.36	0.6914893	0.753246	0.860215	0.7683171
Serpinb6	Q60854	316	QGLFLSK(*)VVHK(*)	K7	69.37	88.98	1.0319148	0.675324	0.903225	0.8701551
Serpinb6	Q60854	109	TC(+57.02)DLLASFK(*)DSC(+57.02)	K9	53.29	51.63	-	-	0.666666667	
Serpinb6	Q60854	309	ADFSGMSSK(*)QGLFLSK(*)	K9	38.18	39.4	-	-	0.967741935	
Serpinb6	Q60854	172	AIYFK(*)GNWEK(*)	K5	11.09	73.54	0.9468085	0.805194	1	0.9173344
Stip1	Q60864	325	DAIHFYNK(*)SLAEHR(*)	K8	93.56	1000	0.9489795	0.8	1.010869	0.9199497
Stip1	Q60864	246	ALK(*)HYDR(*)	K3	87.68	1000	-	0.95	0.945652	0.9478261
Stip1	Q60864	446	DYTK(*)AMDVYQK(*)	K4	87.24	186.64	0.9591836	0.85	0.956521	0.9219018
Stip1	Q60864	373	GNEC(+57.02)FQK(*)GDYPQAMK(*)	K7	83.87	130.09	0.9591836	0.675	0.945652	0.8599453
Stip1	Q60864	68	TVDLK(*)PDWGK(*)	K5	83.67	69.39	0.8061224	0.7875	0.891304	0.8283089
Stip1	Q60864	317	YK(*)DAIHFYNK(*)	K2	83.53	242.89	1.0816326	0.9	0.934782	0.9721384
Stip1	Q60864	73	TVDLK(*)PDWGK(*)GYSR(*)	K10	79.4	64.28	0.8979591	0.8375	0.978260	0.9045734
Stip1	Q60864	325	YK(*)DAIHFYNK(*)SLAEHR(*)	K10	79.19	180.8	0.9693877	0.7375	0.989130	0.8986727
Stip1	Q60864	100	TYEEGLK(*)HEANNLQLK(*)	K7	69.15	97.64	1.7551020	0.45	0.978260	1.061121
Stip1	Q60864	169	ELIEQLQNK(*)PSDLGTK(*)LQDPR(*)	K16	59.95	81.81	0.7448979	0.475	0.956521	0.7254732
Stip1	Q60864	381	GDYPQAMK(*)HYTEAIK(*)	K8	51.39	100.91	1	0.85	0.815217	0.8884058
Stip1	Q60864	100	R(*)TYEEGLK(*)HEANNLQLK(*)	K8	25.27	74.73	-	-	0.923913043	
Caprin1	Q60865	145	LK(*)TVLELQYVLDK(*)	K2	14.7	212.2	-	-	1.511627907	
Pter	Q60866	90	AK(*)GGGALVENTTTGLSR(*)	K2	84.92	1000	1.1547619	0.702127	0.925531	0.9274738
Pter	Q60866	302	ILMAHDIHTK(*)HR(*)	K10	65.22	1000	1	-	1.010638	1.0053191
Reep5	Q60870	25	LEAK(*)TGVNR(*)	K4	84.2	1000	0.6134453	0.890410	0.868131	0.7906627
Reep5	Q60870	12	FLHEK(*)NC(+57.02)MTDLLAK(*)	K5	78	130.84	0.6806722	0.780821	0.989010	0.8168351
Arhgef2	Q60875	483	LIHEGC(+57.02)LLWK(*)TATGR(*)	K10	42.59	1000	0.5384615	0.486842	1.056818	0.6940406
Arhgef2	Q60875	601	LQQK(*)NQALVELLQK(*)	K4	42.53	207.31	0.9450549	0.776315	1.045454	0.9222751
Vdac2	Q60930	75	YK(*)WC(+57.02)EYGLTFTEK(*)	K2	84.04	215.31	0.9387755	0.888888	0.967391	0.9316852
Vdac2	Q60930	32	DIFNK(*)GFGFGLVK(*)	K5	70.17	216.51	0.9693877	-	0.858695	0.9140417
Vdac2	Q60930	40	GFGFGLVK(*)LDVK(*)	K8	14.63	55.36	-	-	1.293478261	
Vdac2	Q60930	278	LTLSALVDGK(*)SFNAGGHK(*)	K10	7.94	26.99	-	1.283950	-	

Vdac3	Q60931	63	YK(*)VC(+57.02)NYGLTFTQK(*)	K2	90.78	272.66	0.98	0.927710	0.905263	0.937658
Vdac3	Q60931	20	DVFNK(*)GYGFGMVK(*)	K5	86.15	189.04	0.8	0.734939	0.873684	0.8028747
Vdac3	Q60931	34	TK(*)SC(+57.02)SGVEFSTSGHAYTDT	K2	84.34	169.46	0.92	0.722891	1.010526	0.8844726
Vdac3	Q60931	28	GYGFGMVK(*)IDLK(*)	K8	78.84	88.98	0.96	0.771084	0.936842	0.8893088
Vdac3	Q60931	12	C(+99.03)NTPTYC(+57.02)DLGK(*)A	K11	69.39	20.7	-	0.734939	0.8	0.7674699
Vdac3	Q60931	163	SK(*)LSQNNFALGYK(*)	K2	57.07	267.5	0.47	0.530120	0.863157	0.6210928
Vdac3	Q60931	266	LTLSALIDGK(*)NFNAGGHK(*)	K10	31.91	35.1	1.15	-	0.947368	1.0486842
Vdac1	Q60932	33	DVFTK(*)GYGFGLIK(*)	K5	88.88	161.77	0.9895833	0.783132	0.945054	0.9059236
Vdac1	Q60932	25	A(+42.01)VPPTYADLGK(*)SAR(*)	K11	53.58	1000	1	1.566265	0.912087	1.159451
Vdac1	Q60932	279	LTLSALLDGK(*)NVNAGGHK(*)	K10	46.73	30.66	0.9791666	1.096385	0.725274	0.933609
Vdac1	Q60932	41	GYGFGLIK(*)LDLK(*)	K8	14.35	77.13	-	-	1.373626374	
Pml	Q60953	660	SVYSK(*)AVSLEAGLR(*)	K5	52.15	1000	-	0.873417	-	
Rbbp7	Q60973	119	IEC(+57.02)EIK(*)INHEGEVNR(*)	K6	87.89	1000	1.1098901	1	1.054945	1.0549451
Rbbp7	Q60973	4	A(+42.01)SK(*)EMFEDTVEER(*)	K3	49	1000	0.9120879	-	0.626373	0.7692308
Rbbp7	Q60973	295	ILATGSADK(*)TVALWDLR(*)	K9	17.29	1000	-	-	1.065934066	
Ncor1	Q60974	1347	GK(*)PYDGITTIK(*)	K2	76.74	77.76	-	0.970588	0.802197	0.886393
Ppp2r5c	Q60996	192	IYGK(*)FLGLR(*)	K4	68.82	1000	0.9560439	1.04	0.858695	0.9515799
Ppp2r5c	Q60996	183	DFLK(*)TTLHR(*)	K4	41.77	1000	1.3516483	-	0.923913	1.1377807
Asns	Q61024	168	GLVSLK(*)HSTTPFLK(*)	K6	84.59	197.82	0.9550561	0.769230	0.902173	0.875487
Asns	Q61024	478	EAFSDGITSVK(*)NSWFK(*)	K11	81.04	65.44	0.9438202	-	0.934782	0.9393014
Asns	Q61024	445	NGIEK(*)HLLR(*)	K5	77.63	1000	1.0112359	0.871794	0.967391	0.9501407
Asns	Q61024	556	TLTHYK(*)SAAK(*)	K6	76.92	77.13	0.8651685	-	-	
Asns	Q61024	232	LFPGFDLETVK(*)NNLR(*)	K11	67.52	1000	0.8539325	-	0.902173	0.8780532
Asns	Q61024	513	TK(*)EGYFYR(*)	K2	17.21	1000	1.0674157	0.846153	1.010869	0.974813
Ncoa2	Q61026	640	GQTK(*)LLQLLTTK(*)	K4	59.07	193.63	0.3445378	-	0.062027	0.2032825
Ncoa2	Q61026	780	TDPASNTK(*)LIAMK(*)	K8	58.44	95.62	0.2436974	-	0.040090	0.1418941
Ncoa2	Q61026	780	TDPASNTK(*)LIAMK(*)TVK(*)	K8	56.67	150.63	0.1484593	-	0.031770	0.0901147
Ncoa2	Q61026	785	TDPASNTK(*)LIAM(+15.99)K(*)TVK(	K13	26.92	20.7	0.1820728	-	0.012102	0.0970879
Ncoa2	Q61026	785	PASNTK(*)LIAMK(*)TVK(*)	K11	19.15	32.97	0.1512605	-	-	
Ncoa2	Q61026	780	PASNTK(*)LIAMK(*)TVK(*)	K6	19.15	132.61	0.1512605	-	-	
Tmpo	Q61029	302	LTGNFK(*)HASSILPITEFSDITR(*)	K6	57.11	1000	0.7291666	0.8875	0.840909	0.8191919
Tmpo	Q61029	302	FK(*)HASSILPITEFSDITR(*)	K2	19.4	1000	-	-	1.386363636	
Tmpo	Q61033	562	AATQASSTESC(+57.02)DK(*)HLDLAI	K13	57.22	1000	-	0.944444	0.941860	0.9431525
Tmpo	Q61033	460	SSFAK(*)SVVSHSLTTLGVEVSK(*)PPP	K5	53.89	80.24	0.7011494	-	0.860465	0.7808073

Tmpo	Q61033	17	LK(*)SELVANNVTLPAGEQR(*)	K2	34.38	1000	-	1.111111	1.127906	1.119509
Hars	Q61035	243	LDK(*)VSWEEVK(*)	K3	59.19	104.64	0.9339622	1	1.054347	0.9961034
Hars	Q61035	373	YDGLVGMFDPK(*)GR(*)	K11	38.61	1000	0.8773584	1.166666	0.467391	0.8371388
Hars	Q61035	250	VSWEEVK(*)NEMVGEK(*)	K7	33.57	122.76	0.9150943	-	0.532608	0.7238515
Ppm1g	Q61074	380	NAGGK(*)VTMDGR(*)	K5	98.73	1000	0.8584905	0.784946	0.887755	0.8437306
Ppm1g	Q61074	336	GK(*)QLIVANAGDSR(*)	K2	53.22	1000	0.8018867	0.516129	0.418367	0.5787944
Ppm1g	Q61074	172	VPPHTK(*)SGIGTGDEPGPQGLNGEA	K6	26.52	1000	0.8867924	-	-	
Cdc37	Q61081	243	IK(*)TADHQYMEGFK(*)	K2	84.7	215.31	1.0645161	0.925	1.021276	1.0035976
Cdc37	Q61081	161	QIK(*)HFGMLHR(*)	K3	79.42	1000	0.9354838	0.8375	0.989361	0.9207819
Cdc37	Q61081	111	EK(*)NMPWNVDTLSK(*)	K2	65.69	194.69	0.6451612	0.825	0.882978	0.78438
Cdc37	Q61081	127	DGFSK(*)SMVNTK(*)PEK(*)	K5	56.1	156.85	1.0537634	0.55	0.765957	0.789907
Cdc37	Q61081	241	QFFTK(*)IK(*)	K5	54.09	27.16	1.3333333	1.625	1.010638	1.3229905
Abcb7	Q61102	511	VAIVGGSGSGK(*)STIVR(*)	K11	31.48	1000	-	1.2	0.696969	0.9484848
Ppp2r5e	Q61151	200	DYLK(*)TVLHR(*)	K4	17.16	1000	-	1.125	1.465116	1.2950581
Ctcf	Q61164	429	MHILQK(*)HTENVAK(*)	K6	32.34	86.63	0.5106382	1.068965	1	0.8598679
Ctcf	Q61164	496	FK(*)C(+57.02)DQC(+57.02)DYAC(+	K2	26.7	1000	-	1.011494	-	
Prdx2	Q61171	135	GLFIIDAK(*)GVLR(*)	K8	61.04	1000	0.9473684	-	1.172043	1.0597057
Prdx2	Q61171	119	SLSQNYGVLK(*)NDEGIAYR(*)	K10	49.63	1000	0.5052631	0.922077	1.118279	0.8485402
Prdx2	Q61171	119	YGVLK(*)NDEGIAYR(*)	K5	19.52	1000	2.4105263	4.545454	1.354838	2.7702732
Papola	Q61183	635	PSGNTATK(*)VPNPIVGVK(*)	K8	85.95	66.16	0.9354838	0.654761	0.826086	0.8054442
Tsg101	Q61187	362	GVIDLDVFLK(*)HVR(*)	K10	45.39	1000	1.2637362	-	1.084337	1.1740368
Hcfc1	Q61191	217	SK(*)LVIYGGMSGC(+57.02)R(*)	K2	13.08	1000	-	3.358974	3.047058	3.2030166
Pafah1b2	Q61206	86	LK(*)NGELENIK(*)PK(*)	K2	58.29	242.89	0.9780219	0.910447	0.818181	0.9022172
Pafah1b2	Q61206	155	NAK(*)VNQLLK(*)	K3	9.43	85.52	1.0549450	1.089552	1.022727	1.0557415
Psap	Q61207	37	DVK(*)TAVDC(+57.02)GAVK(*)	K3	47.97	77.76	-	-	1.157303371	
Dyrk1a	Q61214	105	TYK(*)HINEVYYAK(*)	K3	43.32	138.5	0.7083333	-	-	
Dyrk1a	Q61214	212	LLELMNK(*)HDTEMK(*)	K7	38.65	62.39	-	-	1.411764706	
Sntb2	Q61235	307	EVK(*)HIAWLAEQAK(*)	K3	48.96	154.29	0.7857142	0.569444	0.791666	0.7156085
Sntb2	Q61235	9	ATK(*)AGLVELLLR(*)	K3	39.28	1000	-	-	1.486111111	
Igbp1	Q61249	177	LSALK(*)SAVESGQADDER(*)	K5	61.65	1000	1.2625	0.808219	0.789473	0.9533976
Igbp1	Q61249	42	TIQDK(*)VSK(*)	K5	41.74	47.36	-	-	1.042105263	
Tcf12	Q61286	151	QDLGLGSPAQLSSSGK(*)PGTPYYSFS	K16	14.97	1000	-	-	2.085365854	
Hspa4	Q61316	53	SIGAAAK(*)SQVISNAK(*)	K7	95.15	192.66	0.9473684	0.8125	0.966666	0.908845
Hspa4	Q61316	430	SK(*)VLTFYR(*)	K2	94.79	1000	0.9473684	0.8625	0.944444	0.9181043

Hspa4	Q61316	61	SQVISNAK(*)NTVQGFK(*)	K8	91.35	148.49	0.9473684	0.8	0.977777	0.9083821
Hspa4	Q61316	44	C(+57.02)TPAC(+57.02)VSFGPK(*)N	K11	73.88	1000	0.8	0.8625	0.877777	0.8467593
Hspa4	Q61316	221	LK(*)VLATAFDTTLGGR(*)	K2	68.44	1000	1.2	0.7125	1.055555	0.9893519
Hspa4	Q61316	720	NK(*)EDQYEHLDAADVTK(*)	K2	65.08	116.09	1.2210526	0.6625	0.9	0.9278509
Hspa4	Q61316	388	GC(+57.02)ALQC(+57.02)AILSPAFK	K14	60.81	1000	0.9157894	0.6125	1.022222	0.8501706
Hspa4	Q61316	185	GIYK(*)QDLPALEEK(*)PR(*)	K4	48.29	110.9	0.9052631	0.6625	0.977777	0.8485136
Hspa4	Q61316	737	VEK(*)STNEAMEWMNSK(*)	K3	44.41	264.82	0.6210526	0.7625	1.144444	0.8426657
Hspa4	Q61316	680	LAELK(*)SLGQPIK(*)	K5	38.58	93.6	-	-	0.766666667	
Hspa4	Q61316	619	NDAK(*)NAVEEYVYEMR(*)	K4	10.59	1000	-	-	1.022222222	
Hspa4	Q61316	718	VISSFK(*)NK(*)	K6	7.66	27.16	-	1.4	-	
Hadh	Q61425	127	LK(*)NELFQR(*)	K2	93.92	1000	0.8380952	0.673913	0.825688	0.7792321
Znf638	Q61464	1454	SK(*)PTQIGVNR(*)	K2	56.34	1000	1.1046511	0.816901	0.978723	0.9667587
Znf638	Q61464	876	ATGK(*)SAEESPSGTLEATEK(*)EPVNK	K4	54.71	124.59	0.8953488	-	0.819148	0.8572489
Znf638	Q61464	794	TMETVSSSSSAK(*)SGQIK(*)	K12	54.55	68.6	0.7558139	0.605633	0.776595	0.7126812
Znf638	Q61464	628	SLSSVK(*)SDSHLGAYSAHK(*)	K6	29.77	40.94	1.2790697	0.971830	0.957446	1.0694492
Znf638	Q61464	876	ATGK(*)SAEESPSGTLEATEK(*)	K4	16.46	148.55	-	-	1.914893617	
Smarcd1	Q61466	498	YFYSK(*)VQQR(*)	K5	14.96	1000	0.5131578	0.974358	0.776	0.7545056
Glg1	Q61543	512	ALNEAC(+57.02)ESVIQTAC(+57.02)	K15	78.53	1000	0.83	0.813333	0.918604	0.8539793
Glg1	Q61543	370	VSYSLAK(*)SC(+57.02)K(*)	K7	52.34	25.67	0.93	0.813333	0.918604	0.8873127
Glg1	Q61543	124	VC(+57.02)PK(*)HTWSNNLAVLEC(+	K4	51.27	1000	-	-	1.081395349	
Glg1	Q61543	1072	ADIFVDPVLHTAC(+57.02)ALDIK(*)H	K18	44.39	1000	0.86	1.253333	0.825581	0.9796382
Glg1	Q61543	1038	IK(*)TELC(+57.02)K(*)	K2	28.64	105.53	0.27	-	-	
Ewsr1	Q61545	438	DFQGSK(*)LK(*)	K6	39.74	30.83	-	1.140845	1.044943	1.0928944
Rad21	Q61550	577	ALAK(*)TGAESISLLELC(+57.02)R(*)	K4	30.83	1000	0.8686868	1.1875	0.838709	0.9649655
Fscn1	Q61553	471	YLK(*)GDHAGVLK(*)	K3	50.25	150.63	-	0.915662	0.969387	0.9425252
Fscn1	Q61553	220	SGK(*)VAFR(*)	K3	16.66	1000	1.1020408	0.867469	1.040816	1.0034423
Fdxr	Q61578	224	VK(*)TVWIVGR(*)	K2	34.31	1000	-	0.553030	1.621212	1.0871212
Fxr1	Q61584	207	NEEATK(*)HLEC(+57.02)TK(*)	K6	52.62	102.87	1.0574712	0.530864	0.907216	0.8318507
Fxr1	Q61584	310	VIQEIVDK(*)SGVVR(*)	K8	36.47	1000	-	0.950617	0.845360	0.8979891
Fxr1	Q61584	83	ANDQEPC(+57.02)GWWLAK(*)VR(*	K13	18.7	1000	0.9080459	1.296296	0.701030	0.9684577
Ktn1	Q61595	875	GK(*)EEQVNSMK(*)	K2	61.27	183.32	0.8571428	0.649350	0.872340	0.7929446
Ktn1	Q61595	1116	YK(*)SVLAETEGILQK(*)	K2	27.55	220.17	0.6632653	-	1.340425	1.0018454
Ktn1	Q61595	288	FK(*)DFLLSLK(*)	K2	13.75	136.94	1.6836734	-	-	
Ktn1	Q61595	203	EQDVLLSHQDTK(*)QEGGLGK(*)	K12	10.55	34.75	1.5510204	-	1.351063	1.4510421

Gdi2	Q61598	269	VVGVK(*)SEGEIAR(*)	K5	86.25	1000	0.7857142	0.75	0.922222	0.8193122
Gdi2	Q61598	309	VIC(+57.02)ILSHPIK(*)NTNDANSC(+	K10	60.24	1000	1	-	0.722222	0.8611111
Gdi2	Q61598	278	C(+57.02)K(*)QLIC(+57.02)DPSYVK	K2	40.11	150.82	-	-	1	
Gli3	Q61602	564	LENLK(*)THLR(*)	K5	5.9	1000	-	-	-	
Ddx19a	Q61655	445	GLAVNMVDSK(*)HSMNILNR(*)	K10	56.68	1000	1.0526315	0.789473	1.032608	0.958238
Ddx19a	Q61655	91	DPSSPLYSVK(*)SFEELR(*)	K10	18.87	1000	-	-	1.630434783	
Ddx5	Q61656	343	ENK(*)TIVFVETK(*)	K3	90.37	150.77	0.7872340	0.707317	0.894736	0.7964293
Ddx5	Q61656	56	FEK(*)NFYQEHPDLAR(*)	K3	73.98	1000	0.8617021	1.219512	0.905263	0.9954925
Ddx5	Q61656	197	LK(*)STC(+57.02)IYGGAPK(*)	K2	68.17	229.18	0.8510638	0.951219	0.957894	0.9200594
Ddx5	Q61656	388	DWVLNEFK(*)HGK(*)	K8	65.14	42.6	0.8510638	0.743902	0.957894	0.8509537
Ddx5	Q61656	236	LIDFLEC(+57.02)GK(*)TNLR(*)	K9	63.99	1000	1.2127659	0.939024	0.989473	1.047088
Ddx5	Q61656	375	DGWPAMGIHGDK(*)SQQER(*)	K12	55.53	1000	0.7553191	-	-	
Cbx5	Q61686	42	WK(*)GFSEEHNTWEPEK(*)	K2	59.97	86.39	0.7142857	-	1.011363	0.8628247
Cbx5	Q61686	102	SSFSNSADDIK(*)SK(*)	K11	38.93	22.87	-	1.769230	1.238636	1.5039336
Atrx	Q61687	197	VLIC(+57.02)K(*)NC(+57.02)FK(*)	K5	75.14	40.19	1.6086956	0.92	1.370786	1.2998274
Atrx	Q61687	778	GK(*)STETSIISK(*)	K2	49.06	128.64	1.2065217	0.906666	1.404494	1.1725609
Hspa1a	Q61696	328	MDK(*)AQIHDLVLVGGSTR(*)	K3	27.29	1000	-	-	-	
Hsph1	Q61699	430	SK(*)VLTFLR(*)	K2	83.62	1000	0.9684210	0.921052	0.931818	0.9404306
Hsph1	Q61699	249	LVEHFC(+57.02)AEFK(*)TK(*)	K10	72.23	39.76	0.9157894	0.763157	1.079545	0.9194976
Hsph1	Q61699	53	TIGVAAK(*)NQQITHANNTVSSFK(*)	K7	60.39	69.91	0.8947368	0.921052	1.5	1.1052632
Hsph1	Q61699	221	LK(*)VLGTAFDPFLGGK(*)	K2	29.24	216.84	0.6947368	-	1.045454	0.8700957
Itih3	Q61704	123	AVSQGK(*)TAGLVK(*)	K6	23.15	102.87	0	0.047619	0	0.015873
Mrps31	Q61733	358	QK(*)VEHIEWFR(*)	K2	13.84	1000	-	2.105769	0.733333	1.4195513
Mrps31	Q61733	163	QTTK(*)SELLR(*)	K4	13.23	1000	1.5641025	0.990384	1.222222	1.2589031
Phgdh	Q61753	58	SATK(*)VTADVINAAEK(*)	K4	97.27	257.85	0.9565217	0.873417	0.955056	0.9283319
Phgdh	Q61753	310	GK(*)SLTGVVNAQALTSAFSPHTK(*)	K2	20.4	230.38	-	-	2.325842697	
Kif5b	Q61768	690	EHLNK(*)VQTANEVK(*)	K5	40.43	161.77	0.8265306	1.594936	0.905263	1.1089102
Kif5b	Q61768	240	VSK(*)TGAEGAVLDEAK(*)	K3	38.92	170.17	0.8571428	1.075949	0.747368	0.8934869
Kif5b	Q61768	602	SEVK(*)TMVK(*)	K4	38.17	61.77	-	-	0.768421053	
Kif5b	Q61768	450	LK(*)TQMLDQEELLASTR(*)	K2	20.41	1000	-	-	0.673684211	
Kif5b	Q61768	644	IK(*)SLTEYLQNVEQK(*)	K2	18.1	150.01	1.5714285	-	1.315789	1.443609
Lasp1	Q61792	27	YWHK(*)AC(+57.02)FHC(+57.02)ET	K4	64.95	159.26	0.8854166	0.974025	1.057471	0.9723046
Lasp1	Q61792	23	VNC(+57.02)LDK(*)YWHK(*)	K6	56.63	76.73	1.2083333	1.220779	1.241379	1.2234973
Lasp1	Q61792	96	GK(*)GFSVVADTPELQR(*)	K2	43.15	1000	-	0.519480	1.103448	0.8114644

Rai1	Q61818	449	GVK(*)NLVSR(*)	K3	74.8	1000	-	0.114213	-	
Rai1	Q61818	1079	AAFK(*)SGK(*)R(*)	K7	43.65	1000	-	0.137055	-	
Rai1	Q61818	1076	AAFK(*)SGK(*)R(*)	K4	43.65	1000	-	0.137055	-	
Rai1	Q61818	1076	AAFK(*)SGK(*)	K4	20.58	34.94	-	0.197969	-	
Pdcd4	Q61823	392	SLWK(*)SSTITIDQMK(*)	K4	22.44	71.03	1.6091954	1.587301	1.532467	1.5763215
Pdcd4	Q61823	297	AALDK(*)ATVLLSMSK(*)	K5	17.98	137.04	0.8735632	-	0.870129	0.8718465
Foxc2	Q61850	213	VVVK(*)SEAASPALPVITK(*)	K4	4.01	87.85	-	-	-	
Myh10	Q61879	767	IGQSK(*)IFFR(*)	K5	89.84	1000	1.01	1.0375	0.978723	1.0087411
Myh10	Q61879	1188	ALEDETK(*)NHEAQIQDMR(*)	K7	88.32	1000	1.05	0.8125	0.936170	0.9328901
Myh10	Q61879	1925	EVSTLK(*)NR(*)	K6	76.93	1000	1.07	1.4625	1.010638	1.1810461
Myh10	Q61879	1809	LQELEGAVK(*)SK(*)	K9	74.81	36.05	0.86	0.75	0.787234	0.799078
Myh10	Q61879	1724	HAEQER(*)DELADEIANSASGK(*)SAL	K20	60.51	34.75	0.55	-	1.063829	0.8069149
Myh10	Q61879	1106	GDDETLHK(*)NNALK(*)	K8	60.28	105.53	1.11	1.0875	0.840425	1.0126418
Myh10	Q61879	663	ESLTK(*)LMATLR(*)	K5	54.55	1000	0.82	1.1375	0.936170	0.9645567
Myh10	Q61879	1520	ADMEDLMSSK(*)DDVGK(*)	K10	47.52	46.62	-	-	1.127659574	
Myh10	Q61879	870	QTK(*)VEGELEEMER(*)	K3	42.37	1000	-	-	0.64893617	
Myh10	Q61879	1241	ELAC(+57.02)EVK(*)VLQQVK(*)	K7	40.18	97.87	0.72	1.0125	0.648936	0.7938121
Myh10	Q61879	547	ATDK(*)TFVEK(*)	K4	35.98	83.98	0.85	-	0.968085	0.9090426
Myh10	Q61879	840	VFTK(*)VK(*)PLLQVTR(*)	K4	31.36	80	0.7	1.475	0.659574	0.9448582
Myh10	Q61879	414	AQTK(*)EQADFAVEALAK(*)	K4	30.37	191.08	-	1.8125	-	
Myh10	Q61879	1345	QLEEEK(*)NSLQEQQEEEEAR(*)K(*)	K6	27.78	24.79	-	-	0.829787234	
Myh10	Q61879	1031	NK(*)QEVMISDLEER(*)	K2	23.81	1000	1.12	-	0.882978	1.0014894
Myh10	Q61879	1525	DDVGK(*)NVHELEK(*)	K5	19.89	93.15	1.51	-	-	
Myh10	Q61879	203	VIQYLAHVASSHK(*)GR(*)	K13	14.32	1000	1.85	-	2.148936	1.9994681
Npm1	Q61937	150	SAPGGGNK(*)VPQK(*)	K8	98.59	76.73	0.8367346	0.875	0.838709	0.8501481
Npm1	Q61937	32	DYHFK(*)VDNDENEHQLSLR(*)	K5	81.33	1000	1.0714285	0.975	1.043010	1.0298131
Npm1	Q61937	32	ADK(*)DYHFK(*)VDNDENEHQLSLR(	K8	80.12	127.18	1.1326530	1.0375	0.709677	0.9599435
Npm1	Q61937	261	GGSLPK(*)VEAK(*)	K6	78.07	64.53	0.8367346	-	0.924731	0.8807329
Npm1	Q61937	271	FINYVK(*)NC(+57.02)FR(*)	K6	73.41	1000	0.8877551	1.05	1.043010	0.9935886
Npm1	Q61937	32	FK(*)VDNDENEHQLSLR(*)	K2	52.45	1000	0.9489795	0.95	0.892473	0.9304842
Npm1	Q61937	265	VEAK(*)FINYVK(*)	K4	48.24	95.35	1	0.7625	-	0.88125
Npm1	Q61937	271	INYVK(*)NC(+57.02)FR(*)	K5	28.69	1000	0.6428571	-	0.612903	0.6278802
Npm1	Q61937	150	SAPGGGNK(*)VPQK(*)K(*)	K8	12.67	87.95	-	-	0.838709677	
Pcbp2	Q61990	181	GVTIPYR(*)PK(*)PSSSPVIFAGGQDR(	K9	48.86	1000	0.8241758	1.013698	0.802325	0.8800667

Pcbp2	Q61990	181	PK(*)PSSSPVIFAGGQDR(*)	K2	11.68	1000	-	1.178082	-	
Pea15	Q62048	98	ISEEEELDTK(*)LTR(*)	K10	54.37	1000	4.7448979	-	1.083333	2.9141156
Pon3	Q62087	159	TLK(*)HELLK(*)	K3	67.74	142.99	0.7307692	1.444444	0.880434	1.0185495
Rhoc	Q62159	162	ISAFGYLEC(+57.02)SAK(*)TK(*)	K12	47.63	22.87	0.7764705	0.695121	0.717647	0.7297465
Dag1	Q62165	498	GGEPNQR(*)PELK(*)NHIDR(*)	K11	51.95	1000	-	-	0.9625	
Ddx3x	Q62167	66	DK(*)DAYSSFGSR(*)	K2	66.34	1000	0.8585858	0.609756	0.913043	0.7937951
Ddx3x	Q62167	81	GK(*)SSFFGDR(*)	K2	62.06	1000	-	-	0.934782609	
Ddx3x	Q62167	208	YTR(*)PTPVQK(*)HAIPIIK(*)	K9	55.1	184.45	1.0202020	0.792682	0.815217	0.8760341
Ddx3x	Q62167	208	PTPVQK(*)HAIPIIK(*)	K6	42.41	93.15	0.5959595	-	0.891304	0.743632
Ssr4	Q62186	94	YQVSWSLEHK(*)SAHAGTYEVR(*)	K10	77.4	1000	1.1758241	0.835443	1.044943	1.018737
Dpysl3	Q62188	258	VMSK(*)SAADLISQAR(*)	K4	88.29	1000	0.8617021	0.653333	0.906976	0.8073374
Dpysl3	Q62188	423	IVSAK(*)NHQSVAEYNIFEGMELR(*)	K5	44.75	1000	-	-	1	
Dpysl3	Q62188	480	FIPC(+57.02)SPFSDYVYK(*)R(*)	K13	6.79	1000	-	-	1.279069767	
Snrpa	Q62189	94	IQYAK(*)TDSDIIAK(*)	K5	78.22	183.32	0.8350515	0.947368	0.953488	0.9119694
Sf3a2	Q62203	10	PGGK(*)TGSGGVASSSESNR(*)	K4	95.17	1000	1	0.662790	0.81	0.8242636
Sf3a2	Q62203	10	M(+58.01)DFQHR(*)PGGK(*)TGSG	K10	75.98	1000	-	0.604651	0.77	0.6873256
Sptbn1	Q62261	1108	LLTQHENIK(*)NEIDNYEEDYQK(*)	K9	80.07	276.94	0.9680851	0.653846	1.021739	0.8812235
Sptbn1	Q62261	214	NALIHK(*)HR(*)PDLIDFDK(*)	K6	75.94	57.1	0.7234042	-	0.913043	0.8182239
Sptbn1	Q62261	1878	LQAAYAGDK(*)ADDIQK(*)	K9	68.93	112.31	0.9468085	0.782051	1.086956	0.9386054
Sptbn1	Q62261	1653	HQILEQAVEDYAETVHQLSK(*)TSR(*)	K20	61.54	1000	1.1170212	1.333333	1	1.1501182
Sptbn1	Q62261	1108	IK(*)NEIDNYEEDYQK(*)	K2	34.22	185.99	1.0425531	1.384615	0.967391	1.13152
Sptbn1	Q62261	558	VLLLSQDYGK(*)HLLGVEDLLQK(*)	K10	21.09	41.83	-	-	1.130434783	
Ss18	Q62280	13	GK(*)GEITPAAIQK(*)	K2	55.53	140.16	-	1.316455	-	
Trim28	Q62318	341	IQK(*)HQEHILR(*)	K3	84.26	1000	0.9468085	0.842105	0.913978	0.9009641
Trim28	Q62318	267	LGDK(*)HATLQK(*)	K4	82.82	151.86	1.1489361	0.973684	1.086021	1.0695473
Trim28	Q62318	214	TVYC(+57.02)NVHK(*)HEPLVLFC(+5	K8	76.44	1000	1.1702127	0.710526	1.193548	1.0247625
Trim28	Q62318	338	QHWTMTK(*)IQK(*)	K7	53.71	62.26	0.6702127	0.921052	0.913978	0.8350813
Trim28	Q62318	770	MFK(*)QFNK(*)	K3	25.22	42.89	-	-	1	
Tsn	Q62348	187	LLNLK(*)NDSLR(*)	K5	92.93	1000	0.8924731	0.905405	0.932584	0.9101543
Tsn	Q62348	69	EHFSTVK(*)THLTSLK(*)	K7	70.75	148.49	1.0967741	0.945945	0.966292	1.0030041
Tfrc	Q62351	632	DLNQFK(*)TDIR(*)	K6	90.65	1000	0.7741935	0.809523	0.979166	0.8542947
Tfrc	Q62351	380	LIVK(*)NVLK(*)	K4	89.86	83.43	0.8602150	0.892857	0.9375	0.8968574
Tfrc	Q62351	517	IMQDVK(*)HPVDGK(*)	K6	39.85	69.64	9.0430107	8.940476	0.78125	6.2549123
Tfrc	Q62351	683	IMK(*)VEYHFLSPYVSPR(*)	K3	23.6	1000	1.7311827	-	0.614583	1.1728831

Tfrc	Q62351	668	LTTDFHNAEK(*)TNR(*)	K10	17.31	1000	1.5161290	-	1.03125	1.2736895
Tfrc	Q62351	534	DSNWISK(*)VEK(*)	K7	14.68	25.67	-	-	1.083333333	
Snrnp70	Q62376	162	DMHSAYK(*)HADGK(*)	K7	69.41	95.62	0.8888888	0.975308	0.978260	0.9474861
Zrsr2	Q62377	328	GK(*)HC(+57.02)NFLHVFR(*)	K2	8.25	1000	-	-	-	
Zpr1	Q62384	119	EVVK(*)TDSATTR(*)	K4	6.31	1000	0.8095238	-	-	
Phlda1	Q62392	287	QAILAVK(*)STR(*)	K7	6.11	1000	-	-	-	
Tpd52	Q62393	101	EK(*)HLAELK(*)	K2	27.28	102.87	1.0674157	-	0.471698	0.7695569
Sorbs1	Q62417	269	SK(*)SEMNYIEGEK(*)	K2	12.6	172.15	-	-	4.346534653	
Dbnl	Q62418	176	VGK(*)DNFWAK(*)	K3	43.7	134.33	-	0.506329	1.022471	0.7644005
Ostf1	Q62422	3	S(+42.01)K(*)PPPK(*)PVK(*)PGQVK	K2	20.08	41.53	-	0.948717	-	
Cstb	Q62426	56	IK(*)VDVGGDK(*)	K2	89.57	140.72	-	0.675324	0.976744	0.8260344
Smad2	Q62432	420	MSFVK(*)GWGAEYR(*)	K5	87.16	1000	1.4181818	1.05	1.014084	1.1607554
Fkbp3	Q62446	48	LLGNIK(*)NVAK(*)	K6	74.84	51.96	1.1368421	0.987179	0.955555	1.0265257
Fkbp3	Q62446	99	DSK(*)SEETLDEGPPK(*)	K3	56.44	235.98	0.8736842	1.025641	0.788888	0.8960714
Fkbp3	Q62446	80	GTETISK(*)VSEQVK(*)	K7	50.84	123.86	1.2210526	1.282051	0.866666	1.1232569
Fkbp3	Q62446	170	VGVGK(*)VIR(*)	K5	15.47	1000	-	1.025641	-	
Eif4g2	Q62448	31	GAPQHYPK(*)TAGNSEFLGK(*)	K8	50.19	102.98	1.4725274	0.855263	0.943820	1.090537
Eif4g2	Q62448	574	APK(*)HFLPEMLSK(*)	K3	49.23	114.67	0.8901098	-	0.932584	0.9113471
Eif4g2	Q62448	271	AK(*)SLMDQYFAR(*)	K2	28.82	1000	1.0879120	0.921052	-	1.0044824
Eif4g2	Q62448	233	C(+57.02)IK(*)TLLEK(*)	K3	26.32	107.29	-	-	1.134831461	
Eif4g2	Q62448	170	LLISK(*)LQDEFENR(*)	K5	13.87	1000	-	-	1.292134831	
Eif4g2	Q62448	483	PAQSFLMNK(*)NQVPK(*)	K9	11.03	25.58	-	-	0.97752809	
Itga3	Q62470	146	YTK(*)VLWSGLEDQR(*)	K3	43.87	1000	-	1.333333	0.364	0.8486667
Zfp91	Q62511	176	SK(*)TGSLQLVC(+57.02)K(*)	K2	48.21	148.12	-	-	-	
Zyx	Q62523	525	NFHMK(*)C(+57.02)YK(*)	K5	31.83	57.45	1.3068181	1.238805	1.5125	1.3527081
Zyx	Q62523	228	AK(*)PHVQPQPVSSANTQPR(*)	K2	27.31	1000	-	1.791044	0.7	1.2455224
Commd3	Q63829	137	LEYQIK(*)TNQLHK(*)	K6	24.02	121.92	-	0.902439	0.778947	0.8406932
Map2k2	Q63932	88	ISELGAGNGGVVTK(*)AR(*)	K14	34.18	1000	-	1.727272	0.946808	1.3370406
Rab34	Q64008	115	FK(*)C(+57.02)IASTYYR(*)	K2	12	1000	-	1.892857	-	
Raly	Q64012	183	LK(*)SSELQTIK(*)	K2	80.77	151.55	1.0227272	0.918918	1.034883	0.9921766
Raly	Q64012	13	IQTSNVTNK(*)NDPK(*)	K9	74.26	77.51	0.9545454	0.891891	0.965116	0.9371845
Raly	Q64012	191	SSELQTIK(*)TELTQIK(*)	K8	55.48	148.49	0.8181818	1.527027	1.034883	1.1266975
Raly	Q64012	55	VAGC(+57.02)SVHK(*)GYAFVQYANE	K8	49.57	1000	0.7727272	1.391891	0.813953	0.9928576
Raly	Q64012	159	VK(*)TTIPVK(*)	K2	42.96	121.05	-	-	0.744186047	

Raly	Q64012	44	SDVETIFSK(*)YGR(*)	K9	9.26	1000	-	2.405405	-	
Utp14a	Q640M1	136	EVAFSK(*)TSQTLSK(*)	K6	58.11	127.53	-	0.810810	1.013888	0.9123499
Aebp1	Q640N1	583	TYSLGK(*)SSR(*)	K6	14.27	1000	1.7142857	-	0.561797	1.1380417
Spr	Q64105	227	LK(*)SDGALVDC(+57.02)GTSAQK(*)	K2	84.43	322.89	1.1555555	0.935064	0.918604	1.003075
Maoa	Q64133	168	IC(+57.02)WTK(*)TAR(*)	K5	64.61	1000	1.0919540	0.960526	1.148148	1.0668762
Maoa	Q64133	90	ELGIETYK(*)VNVNER(*)	K8	17.87	1000	0.4942528	0.684210	-	0.5892317
Btf3	Q64152	65	IGGK(*)GTAR(*)	K4	94.09	1000	1.1348314	1.259740	1.356321	1.2502979
Btf3	Q64152	44	MK(*)ETIMNQEK(*)	K2	61.64	112.87	1.0786516	0.831168	1.206896	1.0389057
Aga	Q64191	109	IK(*)NAIGVAR(*)	K2	74.61	1000	0.8673469	0.843373	1.023529	0.9114166
Actr3b	Q641P0	317	R(*)PLYK(*)NVVLSGGSTMFR(*)	K5	30.15	1000	-	-	-	
Sf1	Q64213	165	GNTLK(*)NIEK(*)	K5	72.77	68.07	0.8170731	0.968253	0.975	0.920109
Surf4	Q64310	139	SEGK(*)SMFAGVPTMR(*)	K4	70.94	1000	0.7282608	0.714285	-	0.7212733
Surf4	Q64310	22	VTK(*)QYLPHVAR(*)	K3	51.34	1000	1.0543478	1.178571	0.902912	1.0452773
Sqstm1	Q64337	165	EHSK(*)LIFPNPFGHLSDSFSHSR(*)	K4	56.85	1000	-	-	0.823529412	
Sqstm1	Q64337	422	LLQTK(*)NYDIGAALDTIQYSK(*)	K5	45.91	179.51	-	-	0.835294118	
Sqstm1	Q64337	157	C(+57.02)SVC(+57.02)PDYDLC(+57	K16	19.19	1000	1.2758620	-	-	
Rgn	Q64374	244	LPVDK(*)TTSC(+57.02)C(+57.02)FG	K5	17.97	71.74	0.1839080	0.214285	0.305555	0.2345831
Hspe1	Q64433	56	GK(*)SGEIEPVSVK(*)	K2	99.41	251.58	0.8415841	0.835294	0.869565	0.8488145
Hspe1	Q64433	40	SQGK(*)VLQATVVAVGSGGK(*)	K4	67.71	232.92	1.1287128	0.682352	0.858695	0.8899205
Hspe1	Q64433	80	VLLPEYGGTK(*)VVLDDK(*)	K10	53.22	68.95	0.4851485	0.564705	0.684782	0.5782123
Hspe1	Q64433	28	SAAETVTK(*)GGIMLPEK(*)	K8	41.74	98.53	1.0495049	0.552941	0.467391	0.6899458
Mrc2	Q64449	1236	TTSC(+57.02)DTK(*)LQGAVC(+57.0	K7	33.98	1000	-	-	1.010526316	
Mrc2	Q64449	1146	WK(*)DALLLC(+57.02)ESR(*)	K2	15.14	1000	-	2.455696	-	
Hist1h2bb	Q64475	6	PEPSK(*)SAPAPK(*)	K5	99.01	129.72	-	-	-	
Hist1h2bb	Q64475	6	PEPSK(*)SAPAPK(*)K(*)	K5	75.18	136.94	-	-	-	
Hist1h2bb	Q64475	12	PEPSK(*)SAPAPK(*)K(*)GSK(*)	K11	39.12	34.3	-	-	-	
Hist1h2bb	Q64475	6	PEPSK(*)SAPAPK(*)K(*)GSK(*)	K5	39.12	112.07	-	-	-	
Hist1h2bb	Q64475	21	GSK(*)K(*)AISK(*)AQK(*)	K8	28.58	62.26	-	-	-	
Hist1h2bb	Q64475	17	GSK(*)K(*)AISK(*)AQK(*)	K4	28.58	170.44	-	-	-	
Hist1h2bb	Q64475	16	GSK(*)K(*)AISK(*)AQK(*)	K3	28.58	197.82	-	-	-	
Hist1h2bb	Q64475	21	SK(*)K(*)AISK(*)AQK(*)	K7	6.49	40.63	-	-	-	
Hist1h2bb	Q64475	17	SK(*)K(*)AISK(*)AQK(*)	K3	6.49	128.79	-	-	-	
Hist1h2bb	Q64475	16	SK(*)K(*)AISK(*)AQK(*)	K2	6.49	154.54	-	-	-	
Hist1h2bh	Q64478	17	K(*)GSK(*)K(*)ALTK(*)	K5	94.27	47.31	1.1666666	1.083333	1.15625	1.1354167

Hist1h2bh	Q64478	16	K(*)GSK(*)K(*)ALTK(*)	K4	94.27	32.97	1.1666666	1.083333	1.15625	1.1354167
Hist1h2bh	Q64478	6	EPAK(*)SAPAPK(*)	K4	81.33	87.26	1.0909090	1	1.25	1.1136364
Hist1h2bh	Q64478	6	P(+42.01)EPAK(*)SAPAPK(*)	K5	79.68	47.92	1.3333333	1.05	1.21875	1.2006944
Hist1h2bh	Q64478	6	PEPAK(*)SAPAPK(*)K(*)	K5	78.64	115.21	1.0303030	0.883333	1.125	1.0128788
Hist1h2bh	Q64478	13	PEPAK(*)SAPAPK(*)K(*)GSK(*)	K12	37.88	37.81	0.9848484	1.016666	1.21875	1.0734217
Hist1h2bh	Q64478	12	PEPAK(*)SAPAPK(*)K(*)GSK(*)	K11	37.88	55.21	0.9848484	1.016666	1.21875	1.0734217
Hist1h2bh	Q64478	6	PEPAK(*)SAPAPK(*)K(*)GSK(*)	K5	37.88	196.17	0.9848484	1.016666	1.21875	1.0734217
Hist1h2bh	Q64478	21	GSK(*)K(*)ALTK(*)AQK(*)	K8	14.71	24.24	0.9242424	-	0.890625	0.9074337
Hist1h2bh	Q64478	17	GSK(*)K(*)ALTK(*)AQK(*)	K4	14.71	109.53	0.9242424	-	0.890625	0.9074337
Hist1h2bh	Q64478	16	GSK(*)K(*)ALTK(*)AQK(*)	K3	14.71	132.11	0.9242424	-	0.890625	0.9074337
Top2b	Q64511	459	LDDANDAGGK(*)HSLEC(+57.02)TLI	K10	60.23	122.77	1.0505050	1.523809	0.793478	1.1225976
Top2b	Q64511	623	YYK(*)GLGTSTAK(*)	K3	55.51	53.45	-	-	1.326086957	
Top2b	Q64511	1311	K(*)TPTSTGK(*)TNAK(*)	K8	43.09	41.04	-	0.773809	0.869565	0.8216874
Tpp2	Q64514	1200	VLIFAYK(*)HALVNK(*)	K7	79.53	129.72	0.7849462	0.805194	0.933333	0.8411581
Tpp2	Q64514	1130	QK(*)STLIDALC(+57.02)R(*)	K2	73	1000	1.2473118	0.987012	0.922222	1.0521823
Tpp2	Q64514	1123	TDPR(*)PDAATIK(*)NDMDK(*)	K11	60.78	56.99	0.8817204	1.064935	0.766666	0.9044407
Tpp2	Q64514	1229	ENWK(*)NC(+57.02)IQLMK(*)	K4	40.29	104.64	-	1.181818	0.722222	0.9520202
Tpp2	Q64514	1217	FATK(*)LVEEK(*)PTK(*)	K4	35.68	115.97	0.8494623	0.480519	0.744444	0.6914754
Tpp2	Q64514	655	VNESSHYDLAFTDVHFK(*)PGQIR(*)	K17	29.69	1000	1.6989247	-	1.433333	1.566129
Tpp2	Q64514	803	SWVQTLR(*)PVNAK(*)TR(*)	K12	14	1000	0.9354838	1.597402	-	1.2664432
Tpp2	Q64514	98	VLK(*)IPANWTNPLGK(*)	K3	8.95	90.32	-	-	1.011111111	
Gk	Q64516	346	DNLGIIK(*)SSEEIEK(*)	K7	16.25	96.04	32.5375	-	-	
Guk1	Q64520	17	PVVLSGPSGAGK(*)STLLK(*)	K12	82.65	84.49	1.2153846	0.551020	1.075	0.947135
Gpd2	Q64521	197	SSYVLSK(*)SR(*)	K7	57.05	1000	0.6730769	-	0.831460	0.7522688
Gpd2	Q64521	634	FHK(*)FDEDEK(*)	K3	48.5	97.87	-	-	1.011235955	
Gpd2	Q64521	94	GLK(*)TALVER(*)	K3	43.04	1000	1.4615384	-	-	
Gpd2	Q64521	706	LAILMK(*)TAEENLDR(*)	K6	23.33	1000	-	11.17857	4.224719	7.7016453
Hist2h2bb	Q64525	109	PGELAK(*)HAVSEGTK(*)	K6	95.08	216.51	-	-	-	
Hist2h2bb	Q64525	6	PAK(*)SAPAPK(*)	K3	94.59	132.53	-	-	-	
Hist2h2bb	Q64525	117	HAVSEGTK(*)AVTK(*)	K8	91.27	91.04	-	-	-	
Hist2h2bb	Q64525	13	SAPAPK(*)K(*)GSK(*)	K7	88.37	32.97	-	-	-	
Hist2h2bb	Q64525	12	SAPAPK(*)K(*)GSK(*)	K6	88.37	42.89	-	-	-	
Hist2h2bb	Q64525	109	LPGELAK(*)HAVSEGTK(*)	K7	88.13	217.23	-	-	-	
Hist2h2bb	Q64525	44	K(*)ESYSVYVYK(*)VLK(*)	K10	86.34	50.22	-	-	-	

Hist2h2bb	Q64525	6	PDPAK(*)SAPAPK(*)	K5	85.5	134.33	-	-	-	
Hist2h2bb	Q64525	44	ESYSVYVYK(*)VLK(*)	K9	77.58	37.81	-	-	-	
Hist2h2bb	Q64525	109	GELAK(*)HAVSEGTK(*)	K5	74.19	192.66	-	-	-	
Hist2h2bb	Q64525	13	SAPAPK(*)K(*)GSK(*)K(*)	K7	72.28	64.53	-	-	-	
Hist2h2bb	Q64525	12	SAPAPK(*)K(*)GSK(*)K(*)	K6	72.28	94.22	-	-	-	
Hist2h2bb	Q64525	109	LLLPGELAK(*)HAVSEGTK(*)	K9	70.34	118.18	-	-	-	
Hist2h2bb	Q64525	44	SVYVYK(*)VLK(*)	K6	69.06	53.53	-	-	-	
Hist2h2bb	Q64525	21	AVTK(*)VQK(*)	K4	62.25	55.63	-	-	-	
Hist2h2bb	Q64525	109	LLPGELAK(*)HAVSEGTK(*)	K8	60.7	107.15	-	-	-	
Hist2h2bb	Q64525	109	ELAK(*)HAVSEGTK(*)	K4	57.01	99.23	-	-	-	
Hist2h2bb	Q64525	109	LAK(*)HAVSEGTK(*)	K3	55.29	161.77	-	-	-	
Hist2h2bb	Q64525	109	AK(*)HAVSEGTK(*)	K2	52.06	197.82	-	-	-	
Hist2h2bb	Q64525	21	GSK(*)K(*)AVTK(*)VQK(*)	K8	50.74	68.47	-	-	-	
Hist2h2bb	Q64525	17	GSK(*)K(*)AVTK(*)VQK(*)	K4	50.74	186.64	-	-	-	
Hist2h2bb	Q64525	16	GSK(*)K(*)AVTK(*)VQK(*)	K3	50.74	216.51	-	-	-	
Hist2h2bb	Q64525	21	K(*)AVTK(*)VQK(*)	K5	50.54	77.24	-	-	-	
Hist2h2bb	Q64525	47	VLK(*)QVHPDTGISSK(*)	K3	46.46	215.31	-	-	-	
Hist2h2bb	Q64525	21	K(*)AVTK(*)VQK(*)K(*)	K5	39.13	72.65	-	-	-	
Hist2h2bb	Q64525	21	SK(*)K(*)AVTK(*)VQK(*)	K7	33.82	77.24	-	-	-	
Hist2h2bb	Q64525	17	SK(*)K(*)AVTK(*)VQK(*)	K3	33.82	184.45	-	-	-	
Hist2h2bb	Q64525	16	SK(*)K(*)AVTK(*)VQK(*)	K2	33.82	217.23	-	-	-	
Hist2h2bb	Q64525	13	PDPAK(*)SAPAPK(*)K(*)GSK(*)	K12	24.69	34.94	-	-	-	
Hist2h2bb	Q64525	12	PDPAK(*)SAPAPK(*)K(*)GSK(*)	K11	24.69	63.97	-	-	-	
Hist2h2bb	Q64525	6	PDPAK(*)SAPAPK(*)K(*)GSK(*)	K5	24.69	185.69	-	-	-	
Srm	Q64674	113	EVVK(*)HPSVESVVQC(+57.02)EIDED	K4	55.71	258.35	-	-	1.053191489	
Srm	Q64674	253	MLC(+57.02)SK(*)NPSTNFR(*)	K5	41.17	1000	0.7373737	0.506024	1.3191489	0.8541823
Srm	Q64674	194	ESYYQLMK(*)TALK(*)	K8	18.81	44.28	1.3030303	-	1.1702127	1.2366215
Vcl	Q64727	216	IFVTTK(*)NSK(*)	K6	86.33	30.72	1	0.923076	0.8586956	0.9272575
Vcl	Q64727	699	NPGNQAAYEHFETMK(*)NQWIDNVE	K15	85.52	77.76	-	-	0.945652174	
Vcl	Q64727	476	QVATALQNLQTK(*)TNR(*)	K12	82.15	1000	0.8865979	1.012820	0.8369565	0.912125
Vcl	Q64727	496	AAVHLEGK(*)IEQAQR(*)	K8	80.32	1000	0.8762886	0.794871	0.9239130	0.8650245
Vcl	Q64727	666	STVEGIQASVK(*)TAR(*)	K11	60.39	1000	0.5257731	0.820512	-	0.673143
Vcl	Q64727	276	ALASIDSK(*)LNQAK(*)	K8	51.18	51	-	-	1.02173913	
Vcl	Q64727	655	AAAVGTANK(*)STVEGIQASVK(*)	K9	50.98	141.98	-	-	0.858695652	

Vcl	Q64727	281	LNQAK(*)GWLR(*)	K5	49.16	1000	0.6597938	-	0.978260[illegible]	0.8190273
Vcl	Q64727	228	NQGIEEALK(*)NR(*)	K9	29.99	1000	0.6494845	-	-	
Vcl	Q64727	219	NSK(*)NQGIEEALK(*)	K3	25.84	202.16	3.7216494	5.987179	3.358695[illegible]	4.3558415
Gart	Q64737	251	IK(*)NTILQR(*)	K2	96.04	1000	0.9021739	0.833333	0.922222[illegible]	0.8859098
Gart	Q64737	162	GVIVAK(*)SQAEAC(+57.02)R(*)	K6	80.48	1000	0.5	0.525641	0.7	0.5752137
Gart	Q64737	832	DPK(*)SSSHIVLVISNK(*)	K3	59.61	191.91	-	0.730769	0.977777[illegible]	0.8542735
Gart	Q64737	844	SSSHIVLVISNK(*)AAVAGLDR(*)	K12	53.79	1000	-	-	0.988888889	
Ptpmt1	Q66GT5	53	IDHTVLLGALPLK(*)NMTR(*)	K13	27.82	1000	-	-	4.533333333	
Knl1	Q66JQ7	204	TGK(*)HISSSTELDK(*)ENAEIPVYSK(*	K3	24.04	77.37	-	-	-	
Bcl9l	Q67FY2	43	GHC(+57.02)PPAPAK(*)PMHPENK(	K16	87.82	23.15	-	0.543689	2.06	1.3018447
Bcl9l	Q67FY2	36	GHC(+57.02)PPAPAK(*)PMHPENK(	K9	87.82	49.25	-	0.543689	2.06	1.3018447
Bcl9l	Q67FY2	83	GVGAGSHGAK(*)ANQISPSNSSLK(*)	K10	77.45	106.18	-	0.582524	1.14	0.8612621
Bcl9l	Q67FY2	73	LGSK(*)GVGAGSHGAK(*)	K4	68.84	168.51	-	0.601941	1.48	1.0409709
Bcl9l	Q67FY2	1278	LMGK(*)GMTGR(*)	K4	59.2	1000	-	0.553398	1.04	0.796699
Bcl9l	Q67FY2	108	NPQAGVSPFSSLK(*)GK(*)VK(*)	K13	55.77	25.7	-	-	0.94	
Bcl9l	Q67FY2	73	VNQGPTC(+57.02)NLGSK(*)GVGAG	K12	39.73	30.19	-	0.650485	1.78	1.2152427
Bcl9l	Q67FY2	83	LGSK(*)GVGAGSHGAK(*)ANQISPSN	K14	13.38	101.58	-	0.766990	1.56	1.1634951
Bcl9l	Q67FY2	73	LGSK(*)GVGAGSHGAK(*)ANQISPSN	K4	13.38	139.63	-	0.766990	1.56	1.1634951
Bcl9l	Q67FY2	7	ILANK(*)TR(*)	K5	12.43	1000	-	-	-	
Sbno1	Q689Z5	440	AK(*)NLC(+57.02)PVGSSK(*)	K2	21.23	106.84	0.6	-	1.430232[illegible]	1.0151163
Cltc	Q68FD5	1501	LEK(*)HELIEFR(*)	K3	94.64	1000	0.9072164	0.8875	0.934782[illegible]	0.909833
Cltc	Q68FD5	487	ANVPNK(*)VIQC(+57.02)FAETGQVC	K6	81.94	249.09	0.8350515	0.775	0.913043[illegible]	0.8410317
Cltc	Q68FD5	1609	EYLTK(*)VDK(*)	K5	81.65	50.22	0.6391752	0.65	1.021739[illegible]	0.7703048
Cltc	Q68FD5	1516	IAAYLFK(*)GNNR(*)	K7	80.13	1000	-	0.7875	0.891304[illegible]	0.8394022
Cltc	Q68FD5	1347	VNIPK(*)VLR(*)	K5	77.42	1000	0.8556701	0.7125	0.826086[illegible]	0.7980857
Cltc	Q68FD5	1397	DIITK(*)VANVELYYK(*)	K5	71.24	199.69	-	-	0.815217391	
Cltc	Q68FD5	100	MK(*)AHTMTDDVTFWK(*)	K2	66.73	174.64	0.9072164	0.8375	1.010869[illegible]	0.9185287
Cltc	Q68FD5	737	YIQAAC(+57.02)K(*)TGQIK(*)	K7	64.99	81.69	0.8659793	0.9375	0.891304[illegible]	0.8982612
Cltc	Q68FD5	163	QK(*)WLLLTGISAQQNR(*)	K2	60.69	1000	0.8041237	0.9	0.891304[illegible]	0.8651427
Cltc	Q68FD5	1461	SVQNHNNK(*)SVNESLNNLFITEEDYC	K8	59.57	1000	-	-	0.956521739	
Cltc	Q68FD5	571	LLDALK(*)NNR(*)PSEGPLQTR(*)	K6	58.34	1000	0.7422680	-	0.891304[illegible]	0.8167862
Cltc	Q68FD5	86	AGK(*)TLQIFNIEMK(*)	K3	48.75	252.77	1.0103092	-	0.891304[illegible]	0.9508068
Cltc	Q68FD5	1264	EVC(+57.02)FAC(+57.02)VDGK(*)EF	K10	48.05	1000	-	0.85	-	
Cltc	Q68FD5	1254	TWK(*)EVC(+57.02)FAC(+57.02)VD	K3	43.78	154.29	0.8762886	-	0.586956[illegible]	0.7316226

Cltc	Q68FD5	269	QISEK(*)HDVVFLITK(*)	K5	38.09	165.61	0.6288659	1.2625	0.891304	0.9275568
Cltc	Q68FD5	1118	AQLQK(*)GMVK(*)	K5	25.84	26.36	-	-	1.695652174	
Cltc	Q68FD5	881	IHEGC(+57.02)EEPATHNALAK(*)IYI	K16	14.69	1000	0.9175257	-	2.195652	1.556589
Cltc	Q68FD5	456	EDK(*)LEC(+57.02)SEELGDLVK(*)	K3	14.2	126.66	-	-	1.760869565	
Cltc	Q68FD5	1612	VDK(*)LDASESLR(*)	K3	11.11	1000	1.2886597	1.35	0.858695	1.1657851
Galk2	Q68FH4	168	LSK(*)VELAEIC(+57.02)AK(*)	K3	46.64	159.26	-	-	0.378947368	
Galk2	Q68FH4	177	VELAEIC(+57.02)AK(*)SER(*)	K9	45.21	1000	0.7816091	0.835616	1.2	0.9390752
Galk2	Q68FH4	440	LAQEK(*)HSLFATK(*)	K5	37.86	105.32	1.2413793	0.643835	0.810526	0.8985804
Ahcyl2	Q68FL4	191	GSSDFC(+57.02)VK(*)NIK(*)	K8	27.07	29.32	0.6585365	0.309941	0.890625	0.619701
Mars	Q68FL6	337	EIC(+57.02)DK(*)YHAIHADIYR(*)	K5	57.83	1000	1.2150537	0.839506	1.054945	1.0365017
Msl2	Q69ZF8	406	TVLLSTK(*)SMK(*)	K7	69.7	49.37	-	-	-	
Jmjd1c	Q69ZK6	1150	SSSGANK(*)TDYLK(*)PEAGETGR(*)	K7	47.11	23.7	-	-	0.778761062	
Myof	Q69ZN7	1494	GK(*)SDENEDPSVVGEFK(*)	K2	78.14	196.81	0.8421052	0.846153	0.831460	0.8399066
Myof	Q69ZN7	1923	AK(*)TASLFEQR(*)	K2	73.61	1000	0.7578947	0.833333	0.752808	0.7813457
Myof	Q69ZN7	799	YC(+57.02)GK(*)TQTILLK(*)	K4	66.97	142.27	0.8421052	0.961538	0.876404	0.8933494
Myof	Q69ZN7	662	LQSNIEAVK(*)SGIQGK(*)	K9	56.35	104.48	-	0.743589	0.775280	0.7594353
Myof	Q69ZN7	450	LTK(*)NDVVGTTYLYLSK(*)	K3	43.41	208.61	-	1.448717	1	1.224359
Myof	Q69ZN7	19	FGK(*)PDPIVSVIFK(*)	K3	41.61	118.06	0.6842105	-	0.808988	0.7465996
Myof	Q69ZN7	625	TK(*)PVVTLTSYWEDISHR(*)	K2	37.21	1000	-	-	1.438202247	
Myof	Q69ZN7	1785	VIIWNTK(*)DVILDEK(*)	K7	31.21	125.04	0.9578947	-	0.977528	0.9677114
Myof	Q69ZN7	1701	DYHLDEFEANK(*)ILHQHLGAPEER(*)	K11	28.01	1000	-	-	0.719101124	
Myof	Q69ZN7	1817	QK(*)TDVHYR(*)	K2	3.86	1000	3.3578947	-	-	
Hectd1	Q69ZR2	229	GVDPAPLAK(*)HGLTEELLSR(*)	K9	46.3	1000	-	-	0.571428571	
Hectd1	Q69ZR2	2285	LMC(+57.02)MGDIK(*)SNMSK(*)	K8	39.76	38.77	-	0.988095	-	
Pds5a	Q6A026	1210	IISVTPVK(*)NIDTVK(*)	K8	81.98	74.28	1.0520833	0.682926	0.903225	0.879412
Pds5a	Q6A026	973	QC(+57.02)LLK(*)NISIR(*)	K5	52.96	1000	0.9270833	0.780487	0.967741	0.891771
Pds5a	Q6A026	1118	DFC(+57.02)NDK(*)SYISEETR(*)	K6	33.83	1000	-	-	4.795698925	
Pds5a	Q6A026	849	WLLGMK(*)NNQSK(*)	K6	30.03	36.47	0.9479166	0.902439	0.784946	0.878434
Swap70	Q6A028	537	DK(*)VAHHEGLIR(*)	K2	63.79	1000	1.1954022	0.948051	0.881720	1.0083916
Cdc5l	Q6A068	7	IMIK(*)GGVWR(*)	K4	46.13	1000	0.8260869	-	-	
Cdc5l	Q6A068	106	TAAQC(+57.02)LEHYEFLLDK(*)TAQ	K15	18.53	1000	-	-	1.177777778	
FAM120A	Q6A0A9	12	GVQGFQDYIEK(*)HC(+57.02)PSAV	K11	45.39	27.74	0.7127659	0.851351	1	0.8547058
FAM120A	Q6A0A9	930	TSK(*)SQGGVQPIPSQGGK(*)	K3	39.14	206.62	0.8723404	-	0.883720	0.8780307
FAM120A	Q6A0A9	576	IFHILTK(*)GEIK(*)	K7	36.43	59.3	0.6702127	1.094594	1.465116	1.0766412

FAM120A	Q6A0A9	424	NLTEQNSYSNIPHEGK(*)HTPLYER(*)	K16	36.08	1000	1.0957446	-	1.034883	1.0653142
Usp7	Q6A4J8	915	EEEITLYPDK(*)HGC(+57.02)VR(*)	K10	53.68	1000	1.1578947	0.896103	1.287356	1.113785
Usp7	Q6A4J8	935	AVELGDK(*)ASGR(*)	K7	16.15	1000	1.0105263	-	0.827586	0.9190563
Usp7	Q6A4J8	825	MNYFQVAK(*)TVAQR(*)	K8	12.91	1000	-	-	1.367816092	
Ncapg2	Q6DFV1	248	MIHETIK(*)NQLAGLQK(*)	K7	41.81	154.74	1.24	0.954545	1.175	1.1231818
Ncapg2	Q6DFV1	178	SLETK(*)SGADVC(+57.02)R(*)	K5	33.82	1000	-	-	0.725	
Nop58	Q6DFW4	365	MLAAK(*)TVLAIR(*)	K5	90.38	1000	0.7216494	-	0.967391	0.8445204
Nop58	Q6DFW4	310	LIAHAGSLLNLAK(*)HAASTVQILGAEK	K13	49.34	101.11	-	-	0.902173913	
Nop58	Q6DFW4	310	AGSLLNLAK(*)HAASTVQILGAEK(*)	K9	19.42	116.12	-	-	0.858695652	
Nop58	Q6DFW4	310	LAK(*)HAASTVQILGAEK(*)	K3	6.72	81.1	-	-	1.652173913	
Scaf8	Q6DID3	41	FYK(*)HVVQSVEK(*)	K3	59.46	192.66	-	1.191780	0.698529	0.9451551
A2m	Q6GQT1	1047	VLK(*)SFAQAR(*)	K3	10.55	1000	0.6666666	0.333333	0.181818	0.3939394
Nomo1	Q6GQT9	53	LYTK(*)HGTLK(*)	K4	74.16	105.53	0.8105263	0.842105	0.934782	0.8624714
Jade3	Q6IE82	601	YPLESK(*)SNC(+57.02)LQTSR(*)	K6	53.22	1000	-	-	-	
Jade3	Q6IE82	812	TK(*)THPHSHSSMQR(*)	K2	33.81	1000	-	-	-	
Jade3	Q6IE82	299	MEPVTK(*)ISHIPPSR(*)	K6	28.9	1000	-	-	-	
Jade3	Q6IE82	35	SK(*)NPK(*)EQK(*)	K5	12.76	49.37	-	-	-	
Jade3	Q6IE82	32	SK(*)NPK(*)EQK(*)	K2	12.76	113.99	-	-	-	
Gpsm1	Q6IR34	90	ALQFHK(*)HDLLLAR(*)	K6	49.19	1000	0.9655172	0.782608	0.933333	0.8938198
Tpm4	Q6IRU2	116	EAK(*)HITDEADR(*)	K3	75.61	1000	0.9690721	0.975308	0.890109	0.9448302
Tpm4	Q6IRU2	169	NVTNNLK(*)SLEAASEK(*)	K7	57.33	131.34	0.9896907	0.950617	1.010989	0.9837657
Tpm4	Q6IRU2	116	EAK(*)HITDEADR(*)K(*)	K3	40.09	135.47	1.2371134	1.197530	1.087912	1.1741855
Cltb	Q6IRU5	204	VAQLC(+57.02)DFNPK(*)SSK(*)	K10	59.24	57.45	0.8965517	0.822784	0.442176	0.7205045
Cltb	Q6IRU5	204	LC(+57.02)DFNPK(*)SSK(*)	K7	30.87	45.7	0.5977011	-	0.639455	0.6185785
Exoc3	Q6KAR6	38	VAGMLQR(*)PDQLDK(*)VEQYR(*)	K13	27.07	1000	1.2022471	0.552380	0.990825	0.9151513
Nipbl	Q6KCD5	610	SDPELSK(*)SEMK(*)	K7	33.82	44.28	-	0.485148	-	
Nipbl	Q6KCD5	1593	LLVHQFSNK(*)STEMALR(*)	K9	14.59	1000	-	-	1.449438202	
Pdcd11	Q6NS46	1049	HSLAIGDK(*)VTGTIK(*)	K8	32.38	66.36	1.0625	0.567901	-	0.8152006
Pdcd11	Q6NS46	378	FK(*)NAGAIFR(*)	K2	28.94	1000	-	-	0.849462366	
U2surp	Q6NV83	88	AFSIGK(*)MSTAK(*)	K6	32.69	56.94	-	1.056179	1.586538	1.3213591
Cpsf6	Q6NVF9	483	VLISSLQDC(+57.02)LHGIESK(*)SYGS	K16	75.14	1000	-	-	0.987654321	
Cpsf6	Q6NVF9	459	QSK(*)VSADDR(*)	K3	29.64	1000	1.1075268	-	1.197530	1.1525289
Dnajc8	Q6NZB0	40	DSVLTSK(*)NQIER(*)	K7	71.93	1000	0.8811881	0.825581	1.074468	0.9270792
Dnajc8	Q6NZB0	164	QAVYK(*)QTMK(*)	K5	11.67	28.7	2.4158415	-	-	

Zc3h11a	Q6NZF1	577	VAGK(*)PVLTAVSGVTR(*)	K4	39.67	1000	5.5121951	0.842696	-	3.1774459
Eif4g1	Q6NZJ6	929	LLK(*)NHDEESLEC(+57.02)LC(+57.0	K3	89.88	1000	0.9255319	0.768292	1.010638	0.9014876
Eif4g1	Q6NZJ6	1505	VDVQVLK(*)VR(*)	K7	74.46	1000	0.6489361	1.012195	0.893617	0.8515828
Eif4g1	Q6NZJ6	601	YEYK(*)SDQWK(*)PLNLEEK(*)	K4	25.11	23.7	0.8404255	-	0.765957	0.8031915
Znf740	Q6NZQ6	40	FDLSSK(*)GHR(*)	K6	39.74	1000	1.5256410	-	0.991803	1.2587222
Xpnpep1	Q6P1B1	322	AVK(*)NSAESDGMR(*)	K3	54.3	1000	0.6702127	1.064102	0.942528	0.8922814
Ppp2r2a	Q6P1F6	123	LWK(*)ISER(*)	K3	25.26	1000	-	-	0.652173913	
Heatr6	Q6P1G0	1140	VALK(*)HIHR(*)	K4	25.92	1000	0.9277108	-	0.963855	0.9457831
Nsd3	Q6P2L6	790	K(*)FPTAIFESK(*)GFR(*)	K10	69.92	142.42	0.5915492	0.820224	0.879120	0.7636316
Nsd3	Q6P2L6	471	IAWK(*)TAAAR(*)	K4	52.09	1000	1.1760563	1.101123	2.164835	1.4806717
Nsd3	Q6P2L6	532	GIGNK(*)TEISVR(*)	K5	45.61	1000	0.3943661	-	0.868131	0.631249
Nsd3	Q6P2L6	790	FPTAIFESK(*)GFR(*)	K9	18.84	1000	-	-	1	
Znf280c	Q6P3Y5	180	TGVDQTLGLK(*)HPSTSK(*)	K10	36.87	63.05	0.9833333	0.915492	1.063829	0.987552
Znf280c	Q6P3Y5	343	FMNHMK(*)HHLELEK(*)	K6	15.53	96.04	-	1.098591	1.393617	1.2461043
Sik3	Q6P4S6	41	IIDK(*)SQLDEENLK(*)	K4	46.86	186.74	1.0388349	0.589473	0.805555	0.8112881
Snrnp200	Q6P4T2	1552	PVYHAITK(*)HSPK(*)	K8	80.87	69.71	0.8686868	0.9375	0.793478	0.866555
Snrnp200	Q6P4T2	1890	FNDPHVK(*)TNLLLQAHLSR(*)	K7	64.11	1000	1.1212121	-	0.945652	1.0334321
Snrnp200	Q6P4T2	479	YAQAGFEGFK(*)TLNR(*)	K10	55.14	1000	1.5656565	1.1125	1.173913	1.2840232
Snrnp200	Q6P4T2	1025	VFSLSSEFK(*)NITVR(*)	K9	52.91	1000	-	-	0.945652174	
Snrnp200	Q6P4T2	1110	GWAQLTDK(*)TLNLC(+57.02)K(*)	K8	43.9	85.52	0.8888888	-	1.326086	1.1074879
Snrnp200	Q6P4T2	718	IMEHAGK(*)NQVLVFVHSR(*)	K7	42.83	1000	0.6969696	1	0.717391	0.804787
Snrnp200	Q6P4T2	770	TEAEQC(+57.02)K(*)NLELK(*)	K7	34.21	81.69	0.7878787	0.525	0.967391	0.76009
Snrnp200	Q6P4T2	523	EIGK(*)HINMDGTINVDDFK(*)	K4	28.47	145.95	2.4646464	-	1.260869	1.862758
Abcf1	Q6P542	339	TTLLK(*)HIANR(*)	K5	76.36	1000	-	-	0.920454545	
Rrp12	Q6P5B0	48	PSGK(*)SDLTVDAVK(*)	K4	76.05	186.74	0.97	0.682926	0.882978	0.8453019
Rrp12	Q6P5B0	672	TLITK(*)GC(+57.02)EAEADR(*)	K5	36.91	1000	0.68	-	0.872340	0.7761702
Rrp12	Q6P5B0	961	DVVK(*)SALGFIK(*)	K4	21.3	90.54	0.95	-	0.797872	0.8739362
Rrp12	Q6P5B0	265	AAQHGVC(+57.02)SVLK(*)GSDFMF	K11	4.46	43	-	-	0.829787234	
Smchd1	Q6P5D8	1350	GVYNK(*)STIEGPTIK(*)	K5	39.52	143.22	-	-	1.258426966	
Smchd1	Q6P5D8	900	GPVNSC(+57.02)QGK(*)NFNLK(*)	K9	36.31	70.86	3.9354838	-	-	
Smchd1	Q6P5D8	538	DK(*)NTLFTR(*)	K2	13	1000	-	-	1.348314607	
Smchd1	Q6P5D8	1558	GFNEEDTDTPLFIGK(*)VR(*)	K15	10.56	1000	1.1505376	-	-	
Smchd1	Q6P5D8	1093	IK(*)VNWTPEVNK(*)	K2	9.29	123.21	0.7311827	-	-	
Uggt1	Q6P5E4	362	AITK(*)TAVSAQLR(*)	K4	78.63	1000	0.84	0.898734	0.865168	0.8679676

Uggt1	Q6P5E4	972	IEYQFFEDK(*)HSAIK(*)	K9	68.77	27.86	0.94	0.911392	1.0224719	0.9579548
Uggt1	Q6P5E4	1280	IMMLSVLK(*)NTK(*)	K8	64.47	47.36	0.78	0.835443	0.8988764	0.8381065
Uggt1	Q6P5E4	783	QLLYDAIK(*)HQK(*)	K8	57.83	53.53	0.82	0.632911	0.7415730	0.7314948
Uggt1	Q6P5E4	356	DISQNFPTK(*)AR(*)	K9	50.27	1000	-	-	0.696629213	
Uggt1	Q6P5E4	1399	FWK(*)SGYWASHLAGR(*)	K3	36.24	1000	1.06	-	1.0112359	1.035618
Uggt1	Q6P5E4	731	GK(*)TAAIANSMNYLTK(*)	K2	32.54	212.95	1.11	-	1.1123595	1.1111798
Uggt1	Q6P5E4	930	IK(*)SHIQQLR(*)	K2	31.32	1000	-	0.987341	0.9101123	0.9487271
Uggt1	Q6P5E4	1027	VFMNC(+57.02)QSK(*)LSDMPLK(*)	K8	19.86	72.34	-	-	1.865168539	
Uggt1	Q6P5E4	1034	LSDMPLK(*)SFYR(*)	K7	19.3	1000	-	-	1.539325843	
Uggt1	Q6P5E4	222	SNEGK(*)INYVFR(*)	K5	16.89	1000	-	-	9.134831461	
Xpo1	Q6P5F9	693	QLGSILK(*)TNVR(*)	K7	89.77	1000	0.9673913	1.15	0.9255319	1.0143077
Xpo1	Q6P5F9	563	FLK(*)TVVNK(*)	K3	74.18	105.53	-	0.7875	0.8829787	0.8352394
Xpo1	Q6P5F9	455	DTDSINLYK(*)NMR(*)	K9	66.07	1000	-	-	0.595744681	
Xpo1	Q6P5F9	415	QLYLTVLSK(*)VR(*)	K9	49.88	1000	0.7608695	1.1	0.8191489	0.8933395
Ubxn7	Q6P5G6	418	LLALVK(*)HVQSK(*)	K6	54.25	107.29	1	-	1.0107526	1.0053763
Filip1l	Q6P6L0	667	DK(*)AQFLSQELEHAK(*)	K2	36.96	183.14	-	-	1.033707865	
Senp6	Q6P7W0	5	A(+42.01)AGK(*)SGGSAGALFLK(*)	K4	35.91	97.58	-	-	-	
Tatdn1	Q6P8M1	27	GVQK(*)HQDDLQDVIER(*)	K4	57.55	1000	0.9729729	0.422619	1.8125	1.069364
Snx6	Q6P8X1	386	NLVELAELELK(*)HAK(*)	K11	68.13	42.6	1.0618556	0.676056	1.1971830	0.978365
Snx6	Q6P8X1	366	QELIDFK(*)TR(*)	K7	67.87	1000	0.8247422	0.788732	1.2676056	0.9603601
Snx6	Q6P8X1	276	VSELFDK(*)TR(*)	K7	66.34	1000	0.9793814	1.098591	1.1549295	1.0776342
Snx6	Q6P8X1	386	K(*)NLVELAELELK(*)HAK(*)	K12	41.44	40.63	-	-	1.014084507	
Snx6	Q6P8X1	53	FTVHTK(*)SSLPNFK(*)	K6	32.53	93.6	1.1134020	1.366197	1.2253521	1.2349838
Snx6	Q6P8X1	193	NMVK(*)SADGVIVSGVK(*)	K4	21.38	73.38	-	1.281690	-	
Kif15	Q6P9L6	1170	ASK(*)TSLQHLVTK(*)	K3	36.93	124.12	-	-	0.670212766	
Kif15	Q6P9L6	1267	MK(*)TNLEEVQSALHSK(*)	K2	8.59	61.5	1.4943820	1.116279	0.8617021	1.1574544
Fhod1	Q6P9Q4	950	SLAK(*)HELSPALR(*)	K4	45.13	1000	-	0.625	1.0229885	0.8239943
Oxsr1	Q6P9R2	139	EVLEGLEYLHK(*)NGQIHR(*)	K11	40.26	1000	0.6504854	-	-	
Oxsr1	Q6P9R2	114	GEHK(*)SGVLDEPTIATILR(*)	K4	37.27	1000	-	-	0.75	
Dcaf13	Q6PAC3	49	ALNATK(*)LER(*)	K6	45.91	1000	-	0.550561	-	
Txlna	Q6PAM1	240	SK(*)LESLC(+57.02)R(*)	K2	21.3	1000	-	-	1.186046512	
Txlna	Q6PAM1	412	SSEVFTTFK(*)QEMEK(*)	K9	8.76	32.32	-	-	2.139534884	
Gapvd1	Q6PAR5	270	FIGYLK(*)QNTYC(+57.02)FPHSLR(*)	K6	60.25	1000	0.7865168	-	0.9651162	0.8758166
Gapvd1	Q6PAR5	1373	DK(*)VQC(+57.02)ILR(*)	K2	25.12	1000	-	-	1.395348837	

Herc4	Q6PAV2	606	VNEK(*)TGQLIQYDK(*)	K4	14.44	106.84	1.3333333	1.428571	-	1.3809524
Lrpprc	Q6PB66	452	TK(*)NVQGIIDILK(*)	K2	61.08	228.69	0.7628865	0.649350	0.763440	0.725226
Lrpprc	Q6PB66	1345	ALYEYLTAK(*)NLK(*)	K9	53.9	38.16	0.8453608	0.909090	0.741935	0.8321291
Lrpprc	Q6PB66	294	VEK(*)SDHYFMDR(*)	K3	53.86	1000	0.8041237	0.987012	0.881720	0.8909524
Lrpprc	Q6PB66	1048	SK(*)DAYNIFLK(*)	K2	48.76	197.82	0.7835051	0.948051	0.817204	0.8495871
Lrpprc	Q6PB66	101	ITK(*)GLLQR(*)	K3	9.49	1000	-	-	1.086021505	
Ints6	Q6PCM2	831	IFTLLK(*)HVQGSLQTR(*)	K6	34	1000	-	-	0.857142857	
Smarcc2	Q6PDG5	694	VASAAAK(*)SALEEFSK(*)	K7	70.09	117.11	1.1797752	0.871428	0.920454	0.9905528
Smarcc2	Q6PDG5	874	AK(*)HLAAVEER(*)	K2	61.57	1000	1.0337078	1.242857	0.829545	1.0353702
Smarcc2	Q6PDG5	704	MK(*)EEVPTALVEAHVR(*)	K2	54.83	1000	-	-	0.840909091	
Smarcc2	Q6PDG5	326	K(*)GPSTPYTK(*)SK(*)	K9	52.99	39.76	-	0.771428	0.738636	0.7550325
Smarcc2	Q6PDG5	275	ISAK(*)TLTDEVNSPDSDR(*)R(*)	K4	48.61	1000	-	0.714285	-	
Smarcc2	Q6PDG5	75	HVSNAPLTK(*)LPIK(*)	K9	34.53	49.79	-	1.5	1.011363	1.2556818
Smarcc2	Q6PDG5	101	FK(*)SDQGWR(*)	K2	31.08	1000	1.3033707	-	1.340909	1.3221399
Smarcc2	Q6PDG5	622	DDWNK(*)VSEHVGSR(*)	K5	25.41	1000	1.8876404	1.671428	1.272727	1.6105988
Smarcc2	Q6PDG5	275	ISAK(*)TLTDEVNSPDSDR(*)	K4	17.19	1000	-	1.685714	-	
Phldb1	Q6PDH0	1336	SPNPALTFC(+57.02)VK(*)THDR(*)	K11	19.89	1000	-	1.210526	-	
Ecpas	Q6PDI5	1470	YSPDVLK(*)NHAK(*)	K7	66.23	46.21	0.8367346	0.905405	0.797752	0.846631
Ecpas	Q6PDI5	1034	VK(*)HEVSGETVVFQGGGLGK(*)	K2	47.05	116.26	0.6326530	0.459459	1.101123	0.7310787
Ecpas	Q6PDI5	1413	NSVIQK(*)SC(+57.02)AFAMGHLVR(	K6	24.28	1000	1.1224489	-	0.640449	0.8814492
Ecpas	Q6PDI5	689	FVDK(*)TEWIK(*)	K4	10.91	73.54	-	-	0.640449438	
Dync1li2	Q6PDL0	73	TTLMTK(*)LQGAEHGK(*)	K6	52.91	118.18	1.1604938	0.695652	1.135802	0.9973162
Dync1li2	Q6PDL0	278	EEK(*)NLDLLYK(*)	K3	41.08	90.54	-	0.768115	-	
Dync1li2	Q6PDL0	55	LPSGK(*)NILVFGEDGSGK(*)	K5	29.06	71.31	0.8888888	1.579710	1.037037	1.1685454
Msl1	Q6PDM1	78	LK(*)EPGPPLASSQGGSPSPSPAGC(+	K27	81.38	113.22	-	-	-	
Msl1	Q6PDM1	78	EPGPPLASSQGGSPSPSPAGC(+57.0	K25	53.75	1000	-	-	-	
Msl1	Q6PDM1	78	GPPLASSQGGSPSPSPAGC(+57.02)	K23	6.81	1000	-	-	-	
Srsf1	Q6PDM2	48	DIDLK(*)NR(*)	K5	57.45	1000	0.6770833	1.148148	0.932584	0.9192719
Chd4	Q6PDQ2	1199	PGLGSK(*)TGSMSK(*)	K6	76.41	102.87	-	0.64	0.892473	0.7662366
Chd4	Q6PDQ2	1781	GNFLEIK(*)NK(*)	K7	75.6	36.05	0.7789473	0.573333	0.784946	0.712409
Chd4	Q6PDQ2	962	NMPSK(*)TELIVR(*)	K5	73.75	1000	0.8315789	0.8	0.870967	0.8341822
Chd4	Q6PDQ2	957	ADVFK(*)NMPSK(*)	K5	62.42	87.95	0.6947368	0.466666	0.913978	0.691794
Chd4	Q6PDQ2	952	LK(*)ADVFK(*)	K2	15.63	90.87	0.7684210	-	-	
Snrnp40	Q6PE01	287	IFQGNVHNFEK(*)NLLR(*)	K11	75.28	1000	1.0689655	0.833333	0.956989	0.953096

Mob4	Q6PEB6	70	ILEPPEGQDEGVWK(*)YEHLR(*)	K14	23.05	1000	-	-	1.151162791	
Mapre3	Q6PER3	76	LEHEYIHNFK(*)VLQAAFK(*)	K10	26.71	43.57	-	-	0.961038961	
Pik3c3	Q6PF93	753	HLDNLLLTK(*)TGK(*)	K9	18.7	24.24	-	1.105882	-	
Nup98	Q6PFD9	1721	IQQVDC(+57.02)SGYELEHLHTK(*)V	K17	54.76	1000	-	-	0.958762887	
Nup98	Q6PFD9	1039	LPISASHSSK(*)SR(*)	K10	11.93	1000	-	-	1.237113402	
Rad54b	Q6PFE3	13	SAAPSQVQGK(*)SFK(*)	K10	33.24	40.63	-	-	-	
Naa50	Q6PGB6	34	R(*)LNQVIFPVSYNDK(*)FYK(*)	K14	88.92	77.24	1.0425531	1.075949	-	1.0592513
Naa50	Q6PGB6	37	FYK(*)DVLEVGELAK(*)	K3	60.92	197.65	-	0.822784	0.872340	0.8475626
Naa50	Q6PGB6	34	LNQVIFPVSYNDK(*)FYK(*)	K13	60.24	30.72	0.9148936	1.075949	0.904255	0.9650328
Naa50	Q6PGB6	152	IEPADAHVLQK(*)NLK(*)	K11	58.26	32.97	-	-	0.872340426	
Dhx29	Q6PGC1	1231	IMC(+57.02)TK(*)SVDVTEK(*)	K5	28.61	67.13	1.2710280	-	-	
Bud31	Q6PGH1	125	SK(*)LEVGR(*)	K2	10.28	1000	-	0.8125	-	
Jpt2	Q6PGH2	11	M(+42.01)FQGADSQAGK(*)SGSR(*)	K11	91.53	1000	0.9673913	0.959459	1	0.9756169
Jpt2	Q6PGH2	124	DHVLLC(+57.02)EGEDSK(*)SDLK(*)	K12	89.25	49.79	7.9456521	1.067567	1.185185	3.3994683
Jpt2	Q6PGH2	67	R(*)TNPPGGK(*)GSGIFDESTPVQTR(	K8	76.83	1000	0.6521739	-	0.666666	0.6594203
Jpt2	Q6PGH2	67	TNPPGGK(*)GSGIFDESTPVQTR(*)	K7	73.58	1000	-	1.094594	-	
Jpt2	Q6PGH2	90	LNPPGGK(*)TSDIFGSPVTATAPLAHP	K7	70.98	161.47	0.8586956	0.729729	0.802469	0.7969648
Jpt2	Q6PGH2	55	MASNIFGPTEEPK(*)NIPK(*)	K13	24.67	33.81	1.3586956	1.594594	-	1.4766451
Washc2	Q6PGL7	72	QVDGLIQETK(*)ATHC(+57.02)R(*)	K10	14.23	1000	-	-	1.253012048	
Rab35	Q6PHN9	121	ILVGNK(*)NDDPER(*)	K6	36.61	1000	0.6868686	1.012048	1.096774	0.931897
Camk2d	Q6PHZ2	268	ITASEALK(*)HPWIC(+57.02)QR(*)	K8	78.77	1000	1.1234567	1.032258	1.109756	1.0884903
Camk2d	Q6PHZ2	69	LLK(*)HPNIVR(*)	K3	43.43	1000	1.1975308	-	0.975609	1.0865703
Camk2d	Q6PHZ2	318	NFSAAK(*)SLLK(*)	K6	26.89	24.67	-	-	0.890243902	
Nudcd1	Q6PIP5	220	K(*)YEITK(*)HHVLR(*)	K6	56.24	53.92	0.3636363	0.7	0.861702	0.6417795
Nudcd1	Q6PIP5	220	YEITK(*)HHVLR(*)	K5	22.26	1000	1.5151515	-	1.159574	1.337363
Rif1	Q6PR54	262	LLGK(*)TLHR(*)	K4	41.86	1000	-	-	2.341176471	
Rif1	Q6PR54	244	NK(*)NETYVLK(*)	K2	33.91	104.64	-	-	0.647058824	
Rictor	Q6QI06	124	LK(*)VDYLIAR(*)	K2	29.05	1000	0.3084577	-	0.696428	0.5024431
Ranbp10	Q6VN19	293	MTETPIQEEQASIK(*)NR(*)	K14	75.73	1000	-	0.420454	0.402173	0.4113142
Ubr2	Q6WKZ8	48	EVYQHLAHC(+57.02)VPK(*)IYC(+57	K12	49.78	1000	0.7010309	-	0.802197	0.7516144
Ubr2	Q6WKZ8	450	AFMDHLK(*)HR(*)	K7	43.08	1000	-	-	1.923076923	
Arhgap10	Q6Y5D8	147	NYSLIDK(*)HLTLSAR(*)	K7	33.38	1000	0.4808743	2.015873	1.219047	1.2385983
Jade1	Q6ZPI0	303	MEPITK(*)VSHIPSSR(*)	K6	66.18	1000	-	-	-	
Ube2o	Q6ZPJ3	212	VYDLK(*)NQIILK(*)	K5	54.39	116.54	1.1190476	0.944444	0.846153	0.969882

Smpd4	Q6ZPR5	460	TDLVSPK(*)NALMVFR(*)	K7	57.05	1000	0.5394736	0.392857	0.876404	0.6029118
Kifbp	Q6ZPU9	507	INSLNK(*)SALK(*)	K6	19.65	33.65	1.0340909	-	0.8875	0.9607955
Kdm3b	Q6ZPY7	1512	VAEDFVSPEHVK(*)HC(+57.02)FR(*)	K12	49.78	1000	0.7164179	-	0.881818	0.799118
Zc3h4	Q6ZPZ3	1160	TPNAGGK(*)TAEPASDTSAQPK(*)	K7	75.9	107.72	1.1046511	0.539473	0.846153	0.8300929
Fnbp4	Q6ZQ03	294	TTAASSSK(*)SGPVITK(*)	K8	19.92	128.65	-	0.958333	1.385542	1.1719378
Cnot1	Q6ZQ08	1947	LIALLVK(*)HSGEATNTVTK(*)	K7	62.51	40.85	-	0.902439	-	
Cnot1	Q6ZQ08	1958	HSGEATNTVTK(*)INLLNK(*)	K11	27.48	72.73	0.8152173	-	1.212765	1.0139917
Cnot1	Q6ZQ08	1889	VGQMHQQGILK(*)TDDLITR(*)	K11	26.06	1000	1.1086956	-	-	
Cand1	Q6ZQ38	971	LK(*)GYLISGSSYAR(*)	K2	89.3	1000	0.9791666	0.975	0.956989	0.9703853
Cand1	Q6ZQ38	620	LK(*)NEITR(*)	K2	82.56	1000	0.8645833	0.825	0.913978	0.8678539
Cand1	Q6ZQ38	1072	EVEMGPFK(*)HTVDDGLDIR(*)	K8	72.34	1000	1	0.9375	0.892473	0.9433244
Cand1	Q6ZQ38	826	EGPAVVGQFIQDVK(*)NSR(*)	K14	55.66	1000	0.6979166	1.6625	0.978494	1.1129704
Cand1	Q6ZQ38	577	LK(*)AADIDQEVK(*)	K2	19.98	136.44	0.9479166	0.7875	1.526881	1.0874328
Cand1	Q6ZQ38	1057	DLLDSVLPHLYNETK(*)VR(*)	K15	13.57	1000	-	-	1.860215054	
Larp1	Q6ZQ58	980	AK(*)NLDIDPK(*)	K2	8.66	161.52	4.2954545	5.540540	4.376344	4.7374464
Cand2	Q6ZQ73	1076	EVEMGPFK(*)HTVDDGLDVR(*)	K8	39.83	1000	-	-	0.785714286	
Usp34	Q6ZQ93	419	LSTQHIDC(+57.02)IWAAAQLK(*)HC	K16	28.06	1000	-	-	0.924050633	
Usp34	Q6ZQ93	3142	LIGGK(*)SNIR(*)PPR(*)PELNMC(+57	K5	19.72	48.48	1.3578947	-	1.291139	1.324517
Usp34	Q6ZQ93	1971	HK(*)TTLLELQK(*)	K2	6.27	89.48	0.8421052	-	0.873417	0.8577615
Topbp1	Q6ZQF0	472	SGSFSK(*)SALVPAER(*)	K6	5.25	1000	-	-	0.533333333	
Jade2	Q6ZQF7	38	LPSSTK(*)SGWPR(*)	K6	99.29	1000	-	-	-	
Jade2	Q6ZQF7	38	C(+57.02)SK(*)LPSSTK(*)SGWPR(*)	K9	75.53	1000	-	-	-	
Jade2	Q6ZQF7	32	C(+57.02)SK(*)LPSSTK(*)SGWPR(*)	K3	75.53	1000	-	-	-	
Jade2	Q6ZQF7	298	MEPITK(*)ISHIPASR(*)	K6	72.78	1000	-	-	-	
Jade2	Q6ZQF7	38	SSTK(*)SGWPR(*)	K4	46.67	1000	-	-	-	
Nup188	Q6ZQH8	908	LQSK(*)IEDMR(*)	K4	56.75	1000	0.8932038	1.133333	0.969072	0.9985365
Acap2	Q6ZQK5	282	ASNAFK(*)TWNR(*)	K6	35	1000	-	-	0.776315789	
Acap2	Q6ZQK5	319	LC(+57.02)TVK(*)HC(+57.02)EDIER	K5	29.01	1000	1.175	1.253012	0.789473	1.0724952
Acap2	Q6ZQK5	450	SLGVHFSK(*)VR(*)	K8	13.62	1000	1.35	-	-	
Wdr43	Q6ZQL4	483	VLQTK(*)NVNLIK(*)	K5	74.06	109.28	0.7448979	1.219512	0.811320	0.9252436
Rps9	Q6ZWN5	155	LDSQK(*)HIDFSLR(*)	K5	95.25	1000	0.9255319	0.783132	0.936170	0.8816116
Rps9	Q6ZWN5	121	LGLAK(*)SIHHAR(*)	K5	91.92	1000	0.8936170	0.879518	0.914893	0.8960096
Rps9	Q6ZWN5	93	MK(*)LDYILGLK(*)	K2	83.86	144.73	0.8617021	0.951807	1	0.9378365
Spcs3	Q6ZWQ7	49	IMLK(*)NVEDFTGPR(*)	K4	78.04	1000	0.84	0.8	1.091954	0.9106513

Rpl10	Q6ZWV3	78	YMVK(*)SC(+57.02)GK(*)	K4	94.53	72.09	0.9270833	0.848101	0.967391:	0.914192
Rpl10	Q6ZWV3	82	SC(+57.02)GK(*)DGFHIR(*)	K4	73.19	1000	0.8958333	0.784810	1.173913(	0.9515188
Rpl10	Q6ZWV3	13	YC(+57.02)K(*)NK(*)PYPK(*)	K3	57.29	52.04	0.7604166	-	0.804347!	0.7823822
Rpl35	Q6ZWV7	97	LTK(*)HEEK(*)	K3	87.96	88.98	0.9393939	-	-	
Rpl35	Q6ZWV7	14	K(*)K(*)EELLK(*)	K2	12.18	1000	0.9090909	0.752577	-	0.8308341
Rpl35	Q6ZWV7	79	YK(*)PLDLR(*)PK(*)	K2	10.66	79.54	0.7070707	-	-	
Ppp2r1a	Q76MZ3	542	FNVAK(*)SLQK(*)	K5	87.02	72.65	0.9555555	0.917808	0.964285	0.9458832
Ppp2r1a	Q76MZ3	475	FGK(*)EWAHATIIPK(*)	K3	66.97	235.31	1.1222222	0.958904	0.940476:	1.0072008
Ppp2r1a	Q76MZ3	280	AVGPEITK(*)TDLVPAFQNLM(+15.9!	K8	30.11	85.25	-	-	0.642857143	
Ppp2r1a	Q76MZ3	163	VSSAVK(*)AELR(*)	K6	26.98	1000	1.1222222	1.136986	0.964285	1.0744981
Ppp2r1a	Q76MZ3	188	AAASK(*)LGEFAK(*)	K5	15.73	66.56	-	-	1.25	
Stambpl1	Q76N33	122	TDELK(*)TDLLR(*)	K5	3.79	1000	-	-	0.416666667	
Snd1	Q78PY7	886	VITEYLNAQESAK(*)SAR(*)	K13	87.05	1000	0.8947368	1	0.967391:	0.9540427
Snd1	Q78PY7	758	VESPAK(*)VHVFYIDYGNR(*)	K6	68.15	1000	0.9684210	0.683544	0.956521	0.8694957
Snd1	Q78PY7	93	LIGK(*)EVC(+57.02)FTIENK(*)	K4	67.38	225.26	-	-	0.923913043	
Snd1	Q78PY7	366	LNSGDYK(*)TIHLSSIR(*)PPR(*)	K7	49.63	1000	0.6947368	-	0.891304:	0.7930206
Snd1	Q78PY7	515	AK(*)QFLPFLQR(*)	K2	31.1	1000	1.0736842	1.240506	0.858695(	1.0576287
Snd1	Q78PY7	586	TK(*)ELVLQR(*)	K2	23.4	1000	0.8736842	-	0.902173!	0.8879291
Znrd1	Q791N7	53	VVK(*)TSVVFNK(*)	K3	46.62	128.65	0.3313253	-	0.291666(	0.311496
Mtch2	Q791V5	293	VPC(+57.02)GK(*)TYC(+57.02)YDLF	K5	29.5	1000	-	-	0.95	
Mtch2	Q791V5	293	K(*)VPC(+57.02)GK(*)TYC(+57.02)Y	K6	16.9	45.16	-	2.487804	1.81	2.1489024
Picalm	Q7M6Y3	24	ITAAQHSVTGSAVSK(*)TVC(+57.02)K	K15	37.86	38.03	0.8775510	0.92	1.056179	0.9512436
Cyfip1	Q7TMB8	182	C(+57.02)SVK(*)NDHSAYK(*)	K4	76.74	163.4	-	0.95	1.045454!	0.9977273
Cyfip1	Q7TMB8	778	YK(*)SLELAIGR(*)	K2	64.26	1000	-	-	1.306818182	
Cyfip1	Q7TMB8	1188	VQK(*)HDGK(*)DEIIK(*)	K3	64.25	55.36	0.8494623	0.8	0.931818:	0.8604268
Cyfip1	Q7TMB8	260	MYLTPSEK(*)HMLLK(*)	K8	51.87	65.71	0.7741935	0.7625	0.965909(	0.8342009
Cyfip1	Q7TMB8	320	YIK(*)TSAHYEENK(*)	K3	36.52	178.84	1.0322580	-	1.147727:	1.0899927
Cyfip1	Q7TMB8	294	INLSK(*)IDK(*)	K5	20.44	32.69	0.7526881	-	0.943181!	0.847935
Cyfip1	Q7TMB8	1227	YLK(*)SGDGESTPVEHVR(*)	K3	10.29	1000	-	-	1.477272727	
Syncrip	Q7TMK9	100	SAFLC(+57.02)GVMK(*)TYR(*)	K9	92.44	1000	0.6979166	0.740259	0.956043!	0.7980735
Syncrip	Q7TMK9	256	TK(*)EQILEEFSK(*)	K2	80.62	222.06	0.75	0.844155	0.923076!	0.8390776
Syncrip	Q7TMK9	297	GFC(+57.02)FLEYEDHK(*)TAAQAR(*	K11	79.21	1000	0.7291666	0.701298	1.010989(	0.8138181
Syncrip	Q7TMK9	252	LFVGSIPK(*)SK(*)	K8	72.06	27.16	0.8645833	0.779220	0.901098!	0.848301
Syncrip	Q7TMK9	91	DSDLSHVQNK(*)SAFLC(+57.02)GVN	K10	63.36	159.26	0.84375	0.948051	1.010989(	0.9342637

Syncrip	Q7TMK9	338	VK(*)VLFVR(*)	K2	46.87	1000	2.1770833	0.662337	1.351648	1.3970231
Syncrip	Q7TMK9	371	LK(*)DYAFIHFDER(*)	K2	45.49	1000	0.7708333	-	0.505494	0.6381639
Syncrip	Q7TMK9	232	SGK(*)HIGVC(+57.02)ISVANNR(*)	K3	41.94	1000	1.0104166	0.974025	1.032967	1.0058032
Syncrip	Q7TMK9	363	SFSQFGK(*)LER(*)	K7	12.17	1000	3.5520833	-	-	
Prcp	Q7TMR0	477	LLEVK(*)HMK(*)	K5	64.57	68.47	1.2395833	0.898989	-	1.0692866
Prcp	Q7TMR0	75	YLVADK(*)HWQR(*)	K6	58.58	1000	0.9583333	0.767676	0.968085	0.8980317
Shq1	Q7TMX5	357	ILQLGK(*)SAVLK(*)	K6	24.68	64.28	0.5763888	0.390532	0.745762	0.5708947
Thoc7	Q7TMY4	32	INLLVK(*)SFIK(*)	K6	65.3	46.21	0.9292929	0.768292	0.843137	0.8469076
Huwe1	Q7TMY8	2267	IVNQPSSLFGSK(*)SASSK(*)	K12	89.46	46.62	0.9690721	1.075	1.054945	1.0330057
Huwe1	Q7TMY8	674	HQPTLK(*)TDATTAIIK(*)	K6	52.41	58.98	0.6494845	0.6625	0.857142	0.7230425
Huwe1	Q7TMY8	4142	HILGK(*)SVR(*)	K5	52.07	1000	0.7835051	-	-	
Huwe1	Q7TMY8	4137	SFYK(*)HILGK(*)	K4	51.01	61.3	0.9381443	1.825	1.153846	1.3056635
Huwe1	Q7TMY8	1254	AAFTC(+57.02)IK(*)NLWNR(*)	K7	50.43	1000	-	-	1.21978022	
Huwe1	Q7TMY8	1799	YAMMFAELK(*)STR(*)	K9	15.93	1000	34.505154	-	-	
Casd1	Q7TN73	117	EEGNK(*)HENIPFEDK(*)	K5	40.02	27.52	-	-	-	
Luc7l2	Q7TNC4	37	VC(+57.02)K(*)SHLLNC(+57.02)C(+	K3	76.87	1000	-	-	1.186813187	
Luc7l2	Q7TNC4	61	MDLGEC(+57.02)LK(*)VHDLALR(*)	K8	18.23	1000	-	-	1.648351648	
Spag7	Q7TNE3	187	DAAHMLQANK(*)TYGC(+57.02)VPV	K10	43.3	213.35	-	-	0.846153846	
Dek	Q7TNV0	372	K(*)DFIK(*)TTVK(*)	K5	26.82	55.36	1	1.269230	0.977011	1.0820808
Ints3	Q7TPD0	881	HWC(+57.02)MK(*)HDELLAEHIK(*)	K5	52.99	168.06	0.9680851	0.986486	0.876190	0.9435874
Ints3	Q7TPD0	896	ALLIK(*)NNSLPR(*)	K5	38.37	1000	-	-	0.619047619	
Fbxo11	Q7TPD1	119	TAC(+57.02)PTK(*)SSMEGASTSTTE	K6	19.1	1000	-	1.198581	-	
Prrc2b	Q7TPM1	50	HGLQSLGK(*)VATAR(*)	K8	37.01	1000	0.6725663	-	-	
Actn1	Q7TPR4	95	ISNVNK(*)ALDFIASK(*)	K6	75.89	161.77	0.8958333	0.833333	0.923076	0.8840812
Actn1	Q7TPR4	676	QYEK(*)SIVNYK(*)PK(*)	K4	63.62	90.16	0.8020833	0.923076	0.835164	0.8534417
Actn1	Q7TPR4	162	TAPYK(*)NVNIQNFHISWK(*)	K5	53.97	71.31	0.875	0.743589	1.142857	0.9204823
Actn1	Q7TPR4	741	DAK(*)GISQEQMNEFR(*)	K3	36.67	1000	0.90625	1.410256	0.901098	1.0725351
Actn1	Q7TPR4	413	ASIHEAWTDGK(*)EAMLR(*)	K11	34.42	1000	1.2083333	-	1.978021	1.5931777
Actn1	Q7TPR4	402	QK(*)ASIHEAWTDGK(*)	K2	25.14	223.6	0.9375	1.461538	1.120879	1.1733059
Actn1	Q7TPR4	684	PK(*)IDQLEC(+57.02)DHQLIQEALI	K2	11.65	38.52	-	-	1.098901099	
Mybbp1a	Q7TPV4	874	QEQDLLHK(*)TAR(*)	K8	89.94	1000	-	115.6625	2.089887	58.876194
Mybbp1a	Q7TPV4	496	ATPQIPETK(*)QHFSFPLDDR(*)	K9	84.51	1000	0.8125	0.7125	0.955056	0.8266854
Mybbp1a	Q7TPV4	1005	YPVIC(+57.02)K(*)NLLPVLAQHVAG	K6	78.53	1000	0.7395833	0.65	0.955056	0.7815465
Mybbp1a	Q7TPV4	866	GSTK(*)QEQDLLHK(*)	K4	69.79	193.63	-	0.975	1.337078	1.1560393

Mybbp1a	Q7TPV4	162	SVQLLK(*)ILSQHPNHLQGQPIK(*)	K6	62.3	145.13	0.78125	0.95	1.044943	0.9253979
Mybbp1a	Q7TPV4	484	FC(+57.02)LFHAFFK(*)TK(*)	K9	55.62	25.66	-	0.575	1.247191	0.9110955
Mybbp1a	Q7TPV4	9	SPTK(*)AEPATPAEAAQSDR(*)	K4	53.32	1000	1.0833333	0.4125	0.595505	0.697113
Mybbp1a	Q7TPV4	1186	LK(*)SEGTTPEK(*)	K2	52.7	101.72	0.8958333	0.85	1.033707	0.9265137
Mybbp1a	Q7TPV4	1005	YPVIC(+57.02)K(*)NLLPVLAQHVAG	K6	42.74	1000	0.6979166	-	1.033707	0.8658123
Mybbp1a	Q7TPV4	124	YSLQAMNK(*)AMMR(*)	K8	29.25	1000	1.0104166	-	0.977528	0.9939724
Mybbp1a	Q7TPV4	156	MK(*)SVQLLK(*)	K2	28.65	139.02	-	1.175	0.977528	1.076264
Mybbp1a	Q7TPV4	537	QTPDLAENGK(*)PWTYR(*)	K10	27.44	1000	1.3333333	-	0.707865	1.0205993
Mybbp1a	Q7TPV4	156	EALMK(*)SVQLLK(*)	K5	27.36	70.1	-	0.8	-	
Mybbp1a	Q7TPV4	1033	HQAQAC(+57.02)LMLQK(*)TLSAR(*	K11	21.66	1000	0.8541666	-	-	
Mybbp1a	Q7TPV4	484	HAFFK(*)TK(*)	K5	11.7	39.76	0.7291666	-	0.898876	0.8140215
Aptx	Q7TQC5	225	VVTSEHLELLK(*)HMHAVGEK(*)	K11	41.67	29.7	-	-	-	
Atxn2l	Q7TQH0	138	VK(*)NGTTYEGIFK(*)	K2	37.86	103.28	-	-	1.452380952	
Atxn2l	Q7TQH0	324	IAMENDDGR(*)TEEEK(*)HSAVQR(*)	K14	33.59	1000	0.8222222	0.935064	-	0.8786436
Ccdc93	Q7TQK5	167	AVK(*)TVVGLSDAYK(*)PR(*)	K3	23.07	147.32	-	1.357142	0.858823	1.1079832
Ccdc93	Q7TQK5	331	SK(*)HLEELQANHTSLK(*)	K2	11.38	25.98	0.8272727	-	-	
Prrc2a	Q7TSC1	29	GK(*)SLEIQK(*)PAVAPR(*)	K2	51.91	104.48	1.1978021	1.033333	1.560975	1.264037
Prrc2a	Q7TSC1	49	HGLQSLGK(*)VAIAR(*)	K8	17.32	1000	2.2087912	-	-	
Prrc2a	Q7TSC1	140	R(*)PPTAPENTPSVPSGVK(*)SWAQA	K17	15.13	1000	-	-	1.195121951	
Scaf4	Q7TSH6	41	LYK(*)HVVQIVEK(*)	K3	43.68	242.89	0.5268817	0.906666	1.132743	0.8554306
Atf7ip	Q7TT18	378	DSC(+57.02)DEGNK(*)VNSNVVEK(*	K8	34.4	118.18	-	-	2.461538462	
Atf7ip	Q7TT18	33	VK(*)GELLR(*)ADGK(*)	K2	1.59	100.79	0	-	0	0
Ahcyl1	Q80SW1	108	GSSNFC(+57.02)VK(*)NIK(*)	K8	81.78	68.47	0.6	0.614457	1.059523	0.7579939
Ahcyl1	Q80SW1	478	YK(*)QDVYLLPK(*)	K2	73.14	217.23	1.1052631	1.084337	1.035714	1.0751049
Ahcyl1	Q80SW1	524	NGPFK(*)PNYYR(*)	K5	46.6	1000	0.8421052	0.867469	1.190476	0.9666838
Ahcyl1	Q80SW1	389	MK(*)NSC(+57.02)IVC(+57.02)NM	K2	44.85	1000	0.8947368	-	1.071428	0.9830827
Ahcyl1	Q80SW1	286	QK(*)FDNLYC(+57.02)C(+57.02)R(*	K2	5.31	1000	-	-	2.702380952	
Gng5	Q80SZ7	27	VK(*)VSQAAADLK(*)	K2	31.04	91.09	-	0.892307	0.723684	0.807996
Selenoi	Q80TA1	247	SYK(*)SNTLK(*)	K3	67.24	65.44	0.9595959	1.079207	1.184210	1.0743381
Nisch	Q80TM9	48	VIQVTDGNHEWTIK(*)HR(*)	K14	6.87	1000	-	0.957264	-	
Ubr5	Q80TP3	657	EVVFVEDVK(*)NVPVGK(*)	K9	19.92	41.94	-	-	0.879518072	
Ubr5	Q80TP3	1242	TLIAGQK(*)SAR(*)	K7	7.86	1000	-	-	1.084337349	
Ubr5	Q80TP3	755	GVHAVLK(*)TGSWVR(*)	K7	4.68	1000	-	-	3.168674699	
Clasp1	Q80TV8	335	EILSDDK(*)HDWEQR(*)	K7	31.87	1000	0.9603960	1.688311	0.989247	1.2126517

Clasp1	Q80TV8	1032	IITWTTEPK(*)SSDVR(*)	K9	29.36	1000	0.8712871	-	-	
Clasp1	Q80TV8	1079	LLHNHLK(*)NSSNTGVGSPSNTIGR(*)	K7	12.38	1000	-	-	1.47311828	
Rere	Q80TZ9	569	EEDDGLSGK(*)HSMR(*)	K9	28.37	1000	-	-	-	
Pja2	Q80U04	46	HAYVSFK(*)PC(+57.02)MTR(*)	K7	78.17	1000	-	1.090909	1.053333	1.0721212
Pum2	Q80U58	1038	YTYGK(*)HILAK(*)	K5	47.03	95.62	0.7878787	1.347222	1.033898	1.0563331
Suz12	Q80U70	5	APQK(*)HGGGGGGGSGPSAGSGGG	K4	73.39	24.3	0.7821782	-	-	
Suz12	Q80U70	467	LYSLLK(*)HLK(*)	K6	36.79	49.37	0.8415841	1.063291	0.941860	0.9489119
Septin9	Q80UG5	69	FQDLGVK(*)NSEPAAR(*)	K7	59.78	1000	1.0105263	0.6125	0.879120	0.8340491
Septin9	Q80UG5	341	TIEIK(*)SITHDIEEK(*)	K5	36.93	119.03	1.2947368	-	0.461538	0.8781377
Septin9	Q80UG5	433	LSK(*)VVNIVPVIAK(*)	K3	32.75	160.24	0.8526315	0.9125	0.934065	0.8997325
Septin9	Q80UG5	336	IPK(*)TIEIK(*)	K3	32.15	105.53	0.7052631	0.7875	0.868131	0.786965
Sass6	Q80UK7	404	LK(*)NTVTIQQEK(*)	K2	16.7	140.79	-	-	-	
Naa15	Q80UM3	665	FLTPLK(*)NLVK(*)	K6	92.73	35.82	0.6938775	0.701298	1.011904	0.8023603
Naa15	Q80UM3	734	TVLK(*)QEMNR(*)	K4	60.39	1000	1.8877551	-	1.583333	1.7355442
Naa15	Q80UM3	78	NDLK(*)SHVC(+57.02)WHVYGLLQR	K4	60.29	1000	1.3571428	-	1.583333	1.4702381
Naa15	Q80UM3	747	LFGATNPK(*)NFNETFLK(*)	K8	53.8	161.77	0.8877551	0.753246	0.797619	0.8128736
Naa15	Q80UM3	320	MNFSK(*)GC(+57.02)PPVFNTLR(*)	K5	3.31	1000	-	-	1.30952381	
Taf1	Q80UV9	565	ILLGK(*)TGVIK(*)	K5	67.76	61.26	-	-	-	
Hs6st2	Q80UW0	422	VLLESAK(*)SNLK(*)	K7	23.85	20.17	-	-	0.413533835	
Fam98b	Q80VD1	299	TAC(+57.02)AINK(*)VLMGR(*)	K7	58.89	1000	0.1122448	2	1.010869	1.0410382
Fam98b	Q80VD1	263	YALSPK(*)TTVTLAHLLAAR(*)	K6	36.62	1000	-	-	0.782608696	
Trim56	Q80VI1	110	APGDVHSGK(*)PTC(+57.02)ALC(+5	K21	46.94	54.4	0.9014084	1.830508	-	1.3659585
Dnph1	Q80VJ3	40	YGK(*)VLTEHVADAELEPR(*)	K3	24.64	1000	-	-	0.78021978	
Dhx33	Q80VY9	387	VSK(*)TQAWQR(*)	K3	23.67	1000	-	-	-	
Wipi2	Q80W47	313	LPFC(+57.02)GHK(*)NIC(+57.02)SL	K7	68.22	55.39	0.8863636	0.666666	0.885057	0.8126959
Zmpste24	Q80W54	470	LQALK(*)NAK(*)	K5	69.27	34.94	0.5742574	-	-	
Zmpste24	Q80W54	251	SIDFPLTK(*)VYVVEGSK(*)	K8	63.77	101.72	-	-	0.885416667	
Zmpste24	Q80W54	429	AK(*)DLYSALIK(*)	K2	11.01	145.97	1.0594059	1.417582	0.791666	1.0895517
Kif20b	Q80WE4	753	TSSK(*)VDTSLTSNK(*)	K4	17.59	66.54	1.4705882	-	0.676691	1.07364
Kif20b	Q80WE4	1592	MAVK(*)HPGC(+57.02)PTPVTIK(*)	K4	5.81	26.12	-	-	1.210526316	
Mtdh	Q80WJ7	194	EVDEGAWETK(*)ISHR(*)	K10	48.2	1000	1.9891304	0.619718	0.632183	1.0803442
Mtdh	Q80WJ7	247	NK(*)GDSHLNVQVSNFK(*)	K2	26.56	61.5	0.9021739	1.126760	1.172413	1.0671161
Vac14	Q80WQ2	688	LQLLDVK(*)NNPYLIK(*)	K7	34.54	122.76	-	1.158878	0.655462	0.9071703
	Q80X32	119	AAGFK(*)TSPGR(*)PLGTTK(*)	K5	6.63	44.44	0.9922480	-	1.333333	1.1627907

Tmem106b	Q80X71	130	YIGVK(*)SAYVSYDAEK(*)	K5	88.94	189.31	0.7857142	0.679611	0.942528	0.8026182
Sympk	Q80X82	146	VALQWMVK(*)SR(*)	K8	36.32	1000	0.4411764	-	1.023529	0.7323529
Mrps7	Q80X85	150	IFHEALK(*)NC(+57.02)EPVIGLVPILK	K7	41.68	143.78	1.0851063	-	0.783505	0.9343058
Flnb	Q80X90	2530	AFVGQK(*)SSFLVDC(+57.02)SK(*)	K6	96.35	199.69	0.9157894	0.896103	0.922222	0.9113719
Flnb	Q80X90	1345	EAFTNK(*)SNVFTVVTR(*)	K6	91.36	1000	1.0315789	1.090909	0.944444	1.0223108
Flnb	Q80X90	2566	HVGK(*)QQYNVTYVVK(*)	K4	80.66	187.48	0.9789473	0.974025	0.955555	0.9695096
Flnb	Q80X90	1510	VK(*)VLPTYDASK(*)	K2	77.85	161.68	1.1157894	1.064935	0.833333	1.004686
Flnb	Q80X90	1897	YNDK(*)HIPGSPFTAK(*)	K4	77.07	101.25	1.2105263	0.909090	0.9	1.0065391
Flnb	Q80X90	313	NK(*)TYSVEYLPK(*)	K2	73.93	154.91	0.9052631	0.922077	0.877777	0.9017063
Flnb	Q80X90	1584	DGTYAVTYIPDK(*)TGR(*)	K12	65.03	1000	0.9578947	0.857142	1.011111	0.9420496
Flnb	Q80X90	349	SPFEVNVDK(*)AQGDASK(*)	K9	60.94	51.59	-	-	0.777777778	
Flnb	Q80X90	2507	SSTETC(+57.02)YSAIPK(*)SSSDASK(	K12	60.64	55.73	0.7789473	0.740259	-	0.7596036
Flnb	Q80X90	1449	TVDSSK(*)AGLAPLEVR(*)	K6	53.64	1000	0.9789473	1.571428	0.9	1.1501253
Flnb	Q80X90	715	AIK(*)HTIAVVWGGVNIPHSPYR(*)	K3	51.32	1000	0.9263157	-	1.011111	0.9687135
Flnb	Q80X90	1241	AFGPGIEGK(*)DVFR(*)	K9	50.74	1000	-	0.818181	-	
Flnb	Q80X90	476	ETADFK(*)VDTK(*)	K6	48.51	38.26	1.1473684	0.610389	-	0.878879
Flnb	Q80X90	2524	GAGLSK(*)AFVGQK(*)	K6	47.51	129.72	0.7052631	1.441558	0.955555	1.0341257
Flnb	Q80X90	65	MHHK(*)YHQR(*)	K4	40.7	1000	0.9368421	1.194805	1	1.0438824
Flnb	Q80X90	838	VK(*)VDPSHDASK(*)	K2	24.25	181.07	1.6947368	-	-	
Flnb	Q80X90	2195	GQHVTGSPFQFTVGPLGEGGAHK(*)\	K23	16.5	1000	3.2210526	-	1.466666	2.3438596
Flnb	Q80X90	2372	FNGSHVVGSPFK(*)VR(*)	K12	13.1	1000	0.2	0.402597	-	0.3012987
Flnb	Q80X90	746	VK(*)VFGPGVER(*)	K2	12.17	1000	-	-	1.344444444	
Flnb	Q80X90	2342	VHSPSGAVEEC(+57.02)HVSELEPDK	K20	11.21	1000	1.4842105	-	1.688888	1.5865497
Flnb	Q80X90	948	IK(*)INGLENR(*)	K2	8.37	1000	-	-	1.777777778	
Rraga	Q80X95	307	VDGPK(*)HSLLMR(*)	K5	34.5	1000	1.2298850	0.962025	1.230769	1.1408932
Trmt61a	Q80XC2	57	PFGSK(*)VIC(+57.02)SR(*)	K5	72.03	1000	0.9111111	1	0.77	0.8937037
Pip4k2b	Q80XI4	150	FVIK(*)TVSSEDVAEMHNILK(*)	K4	58.65	170.25	1.3098591	1.218390	0.775700	1.101317
Pip4k2b	Q80XI4	388	TVK(*)HGAGAEISTVNPEQYSK(*)	K3	41.26	102.18	0.9436619	-	0.635514	0.789588
Pip4k2b	Q80XI4	78	IK(*)VDNHLFNK(*)	K2	13.94	131.72	-	-	0.934579439	
Fam76b	Q80XP8	312	ETVEQLQAK(*)NR(*)	K9	38.06	1000	-	-	-	
Fam76b	Q80XP8	52	SEFQQESK(*)TNTIC(+57.02)K(*)	K8	18.42	129.15	-	-	-	
Elac2	Q80Y81	642	HC(+57.02)K(*)HAFGC(+57.02)ALV	K3	68.89	213.97	0.8118811	0.913043	1.081395	0.93544
Kdm5b	Q80Y84	832	SGGGK(*)SQNQLTVNELR(*)	K5	28.74	1000	-	-	-	
Znf598	Q80YR4	236	EK(*)HFLC(+57.02)EEGR(*)	K2	35.3	1000	-	0.953125	-	

Tnc	Q80YX1	2056	GAFWYK(*)NC(+57.02)HR(*)	K6	24.35	1000	-	-	-	
Mrps10	Q80ZK0	81	FTLLK(*)SVHIFK(*)	K5	42.19	121.92	0.3270142	0.712962	0.920454	0.6534772
Abitram	Q80ZQ9	43	GK(*)PC(+57.02)EDHC(+57.02)ILQ	K2	38.08	1000	-	-	0.91025641	
Rnaseh2b	Q80ZV0	301	SIDAFFGAK(*)NK(*)	K9	70.02	22.85	1.2769230	1.196969	1.179487	1.2177933
Ddx42	Q810A7	298	DMIGIAK(*)TGSGK(*)	K7	54.19	38.32	-	-	0.568421053	
Ddx42	Q810A7	50	LPQQSHSAFGAASSSSGFGK(*)SAPP	K20	45.52	28.74	-	-	0.631578947	
Ankfy1	Q810B6	11	LEK(*)HLMLLR(*)	K3	46.62	1000	-	1.181818	0.868131	1.024975
Ankfy1	Q810B6	217	SK(*)TEYPLHK(*)	K2	43.32	122.76	0.6979166	3.337662	0.802197	1.6125923
Ankfy1	Q810B6	937	VNELTK(*)HR(*)	K6	42.44	1000	1.8958333	1.727272	1.483516	1.7022075
Ankfy1	Q810B6	279	R(*)LESIATTLVSHK(*)ADVDMVDK(*)	K13	28.46	32.94	0.9375	-	-	
Grwd1	Q810D6	341	QFK(*)SGSPVATFK(*)	K3	56.5	85.33	0.8235294	0.808219	0.810526	0.8140916
Wdr82	Q8BFQ4	280	VAVLDGK(*)HTGPITC(+57.02)LQFN	K7	51.19	116.1	1.4	0.690476	1.522727	1.2044012
Wdr82	Q8BFQ4	84	YTHAANTVVYSSNK(*)IDDTIR(*)	K14	35.3	1000	-	-	1.034090909	
Klhl42	Q8BFQ9	333	GK(*)IYVIGGYTTR(*)	K2	35.89	1000	5.6521739	0.207865	-	2.9300195
Gns	Q8BFR4	258	NFNIHGTNK(*)HWLIR(*)	K9	78.2	1000	1.0957446	0.825	0.944444	0.955063
Tufm	Q8BFR5	70	DK(*)PHVNVGTIGHVDHGK(*)TTLTA	K17	52.27	40.16	1	-	1.136842	1.0684211
Tufm	Q8BFR5	234	DPELGVK(*)SVQK(*)	K7	32.19	38.26	-	0.743243	1.052631	0.8979374
Zfand1	Q8BFR6	39	SK(*)DSHGC(+57.02)SEVNVVK(*)	K2	7.6	73.35	-	-	5.847826087	
Haus4	Q8BFT2	257	LQTK(*)SELDR(*)	K4	79.58	1000	-	0.988636	-	
Pcid2	Q8BFV2	133	SK(*)VGDMLEK(*)	K2	96.87	161.52	1	1.068493	0.815533	0.9613424
Pcid2	Q8BFV2	32	DGASC(+57.02)AELVSFK(*)HPHVA	K12	34.5	1000	1.0927835	0.917808	-	1.0052959
Lpp	Q8BFW7	109	VQQGNPGGK(*)TLEER(*)	K9	54.9	1000	0.9204545	0.716216	0.894117	0.8435961
Lpp	Q8BFW7	10	S(+42.01)HPSWLPPK(*)STGEPLGH	K9	25.02	1000	-	-	0.682352941	
Lpp	Q8BFW7	494	ATGK(*)AYHPHC(+57.02)FTC(+57.0	K4	10.93	1000	-	-	1.517647059	
Pimreg	Q8BFY7	88	SAK(*)SALGAMSQR(*)	K3	56.85	1000	-	0.618421	3.086956	1.8526888
Tnpo1	Q8BFY9	869	ILHGFK(*)NQVGDENWR(*)	K6	91.96	1000	0.9361702	0.768292	0.955555	0.8866728
Tnpo1	Q8BFY9	81	SLSGLILK(*)NNVK(*)	K8	82.02	62.23	0.7872340	0.853658	0.888888	0.8432605
Tnpo1	Q8BFY9	66	LK(*)SEDEPTR(*)	K2	69.05	1000	0.8510638	0.768292	0.966666	0.8620077
Tnpo1	Q8BFY9	192	FLQFFK(*)HSSPK(*)	K6	53.53	100.21	0.9787234	1.024390	0.944444	0.9825194
Tnpo1	Q8BFY9	863	DMFC(+57.02)K(*)ILHGFK(*)	K5	41.66	143.14	-	-	0.877777778	
Hnrnpa3	Q8BG05	57	EHFEK(*)WGTLTDC(+57.02)VVMR(	K5	79.24	1000	0.9387755	0.8375	0.920454	0.89891
Hnrnpa3	Q8BG05	151	YGK(*)IETIEVMEDR(*)	K3	62.29	1000	0.7755102	0.825	0.920454	0.8403216
Hnrnpa3	Q8BG05	348	YGPMK(*)GGSFGGR(*)	K5	60.01	1000	0.8061224	-	-	
Psmd11	Q8BG32	358	LSK(*)ADVER(*)	K3	79.65	1000	1.0625	0.822784	0.877777	0.9210209

Rhot1	Q8BG51	166	NLK(*)NISELFYYAQK(*)	K3	37.74	235.98	1.4444444	-	1.142857:	1.2936508
Poldip3	Q8BG81	403	ALFK(*)SSGASVTTQPTEFK(*)	K4	64.2	232.92	0.3292682	0.378787	0.833333:	0.5137965
Poldip3	Q8BG81	147	LTK(*)TIQVPQQK(*)	K3	22.53	150.89	0.6951219	1.272727	-	0.9839246
Eif4b	Q8BGD9	529	DGNK(*)VDVVGATQGQAGSC(+57.0	K4	79.38	1000	5.1954022	3.68	0.942528:	3.2726437
Eif4b	Q8BGD9	194	DSDK(*)TDTDWR(*)	K4	55.63	1000	0.8045977	0.773333	1.068965!	0.8822989
Samm50	Q8BGH2	74	AK(*)NLIEVMR(*)	K2	70.2	1000	0.9222222	0.8	1.093023:	0.9384152
Aars	Q8BGQ7	558	VDDSSEDK(*)TEFTVK(*)	K8	88.29	104.62	-	17.62025	17.01098!	17.315621
Aars	Q8BGQ7	879	LFK(*)THSPQTSAMLFTVDNEAGK(*)	K3	71.39	280.15	0.9684210	0.962025	0.956043!	0.9621634
Aars	Q8BGQ7	625	SVLGEADQK(*)GSLVAPDR(*)	K9	66.35	1000	0.8105263	0.721518	1.098901(	0.8769821
Aars	Q8BGQ7	81	AGGK(*)HNDLDDVGK(*)	K4	59.1	225.98	1.0210526	0.936708	0.989010!	0.9822575
Aars	Q8BGQ7	451	LAQLK(*)SQGK(*)	K5	58.42	48.12	0.8105263	-	0.791208:	0.8008676
Aars	Q8BGQ7	564	VDDSSEDK(*)TEFTVK(*)NAQVR(*)	K14	47.84	112.31	0.8210526	1.493670	0.747252:	1.0206588
Ublcp1	Q8BGR9	33	QFLK(*)TLTGVLPER(*)	K4	35.51	1000	-	-	0.987804878	
Bola2	Q8BGS2	47	FEGK(*)PLLQR(*)	K4	12.41	1000	-	0.879518	-	
Micall1	Q8BGT6	739	LIHEK(*)HLLVR(*)	K5	13.91	1000	0.8897058	1.179245	1.414893(	1.1612816
Fto	Q8BGW1	74	EVPEAFLTLHK(*)HGC(+57.02)LFR(*)	K11	59.21	1000	0.8454545	0.948979	1.025974(	0.9401361
Eif5a2	Q8BGY2	47	IVEM(+15.99)STSK(*)TGK(*)	K8	96.32	47.36	-	-	0.955555556	
Eif5a2	Q8BGY2	55	AK(*)VHLVGIDIFTGK(*)	K2	68.34	323.97	-	-	0.811111111	
Cdc23	Q8BGZ4	147	GQVK(*)NEALR(*)	K4	60.76	1000	0.63	0.528089	-	0.5790449
Pck2	Q8BH04	90	YK(*)NC(+57.02)WLAR(*)	K2	83.86	1000	0.9191919	0.828947	1.054347!	0.9341624
Pck2	Q8BH04	108	VESK(*)TVIVTPSQR(*)	K4	62.73	1000	0.8484848	0.605263	0.967391:	0.8070464
Pck2	Q8BH04	491	GK(*)TIMHDPFAMR(*)	K2	55.88	1000	1.0404040	0.881578	0.913043	0.9450088
Pck2	Q8BH04	522	YLEHWLSMEGQK(*)GAR(*)	K12	16.66	1000	-	-	3.597826087	
Wdr48	Q8BH57	657	TVK(*)HFIWK(*)	K3	29.21	142.99	-	-	1.80952381	
Tiprl	Q8BH58	207	LYHEADK(*)TYMLR(*)	K7	58.06	1000	1.0322580	0.784946	0.912621:	0.9099419
Ehd2	Q8BH64	220	VVLNK(*)ADMVETQQLMR(*)	K5	67.29	1000	0.9166666	0.763157	1.022988!	0.9009377
Ehd2	Q8BH64	32	TK(*)LLPLEEHYR(*)	K2	34.38	1000	-	-	0.91954023	
Ehd2	Q8BH64	473	AK(*)TWMVGTK(*)	K2	25.84	61.82	0.9285714	-	-	
Sephs1	Q8BH69	16	ESFNPETYELDK(*)SFR(*)	K12	40.67	1000	-	-	0.816091954	
Sephs1	Q8BH69	32	GTGC(+57.02)K(*)VPQDVLQK(*)	K5	38.09	150.89	0.8476190	1.386666	1.034482:	1.0895895
Nup107	Q8BH74	409	IWK(*)ISC(+57.02)WR(*)	K3	29.26	1000	-	-	0.858695652	
Echs1	Q8BH95	185	AVGK(*)SLAMEMVLTGDR(*)	K4	58.79	1000	-	-	0.816091954	
Wdr3	Q8BHB4	5	GLTK(*)QYLR(*)	K4	50.42	1000	-	-	1	
Wdr3	Q8BHB4	365	IK(*)SFDLIHSPQGELK(*)	K2	42.91	86.39	0.6153846	1.397435	0.576086!	0.8629692

Wdr3	Q8BHB4	289	VVNLAVDK(*)TGR(*)	K8	34.73	1000	0.4903846	-	0.597826(	0.5441054
Dcakd	Q8BHC4	128	YMK(*)HTVVVYC(+57.02)DR(*)	K3	43.88	1000	1.3943661	0.934210	0.97	1.0995256
Ptbp3	Q8BHD7	402	ATLSK(*)HQAVQLPR(*)	K5	27.2	1000	1.7739130	3.424242	0.798076!	1.9987441
Ptbp3	Q8BHD7	108	ELK(*)TDNLPNQAR(*)	K3	25.01	1000	0.7652173	0.616161	-	0.6906895
Maip1	Q8BHE8	276	EFTQGVK(*)PDWTIAR(*)	K7	12.64	1000	1.1891891	-	-	
Nrdc	Q8BHG1	966	TK(*)QTLGYHVYPTC(+57.02)R(*)	K2	38.9	1000	-	-	1.191011236	
Nrdc	Q8BHG1	1076	SFSK(*)SDLVSWFK(*)	K4	34.82	131.72	1.3541666	-	1.134831	1.2444991
Nrdc	Q8BHG1	1084	SDLVSWFK(*)AHR(*)	K8	34.26	1000	0.875	1.220779	0.820224	0.9720013
Nrdc	Q8BHG1	891	YVVDK(*)LNFAPLER(*)	K5	8.49	1000	-	-	1.674157303	
Slu7	Q8BHJ9	288	NLDPNSAYYDPK(*)TR(*)	K12	5.75	1000	-	-	0.756097561	
Ercc6l	Q8BHK9	624	YFTK(*)QELK(*)	K4	27	34.3	-	-	1	
Ganab	Q8BHN3	491	NHGLYVK(*)TR(*)	K7	82.57	1000	0.9892473	0.870129	1.033333	0.9642368
Ganab	Q8BHN3	39	SNFK(*)TC(+57.02)DESSFC(+57.02)I	K4	73.83	189.7	0.9032258	0.987012	1	0.9634129
Ganab	Q8BHN3	908	PAAVVLQTK(*)GSPESR(*)	K9	68.76	1000	0.7956989	0.597402	0.977777	0.7902931
Ganab	Q8BHN3	269	LK(*)VTEGGEPYR(*)	K2	68.08	1000	-	0.727272	0.744444	0.7358586
Noc4l	Q8BHY2	120	ELALK(*)TLMK(*)	K5	20.94	63.97	0.8064516	0.927536	0.905263	0.8797503
Cdkn2aip	Q8BI72	142	AVEGK(*)NNSSVER(*)	K5	46.24	1000	1.0695652	0.741176	0.916666(	0.9091361
Cdkn2aip	Q8BI72	428	LYK(*)TVAWK(*)	K3	20.21	58	0.7391304	0.764705	0.958333	0.8207232
Cdkn2aip	Q8BI72	367	STAQVAASLLATK(*)SGASLGSVSQLA	K13	19.31	146.73	-	-	1.385416667	
Cdkn2aip	Q8BI72	30	C(+57.02)EGETDK(*)HWR(*)	K7	16.68	1000	-	-	1.40625	
Mia3	Q8BI84	1615	SFK(*)NQIAAHEK(*)	K3	12.07	131.34	-	-	0.736263736	
Comtd1	Q8BIG7	227	NK(*)TVEC(+57.02)VR(*)	K2	22.76	1000	-	-	3.119047619	
Iars2	Q8BIJ6	451	MLQATK(*)NVLK(*)	K6	17.46	76.73	0.4946236	-	0.791666(	0.6431452
Iars2	Q8BIJ6	540	TK(*)DEYLINSQTTEHIIK(*)	K2	10.99	93.17	-	-	1.708333333	
Cstf2	Q8BIQ5	189	IVDPEIALK(*)ILHR(*)	K9	33.92	1000	1.9036144	-	1	1.4518072
Zc3h14	Q8BJ05	352	QANK(*)NLILK(*)	K4	13.05	58	-	0.590476	-	
Nup93	Q8BJ71	59	TSQETADVK(*)ASVLLGSR(*)	K9	59.16	1000	0.9569892	-	1.086021!	1.0215054
Nup93	Q8BJ71	611	FTSDTK(*)PIINK(*)VASVAENK(*)	K11	44.73	39.44	0.6881720	-	0.827956!	0.7580645
Nup93	Q8BJ71	633	LYDLAK(*)NADK(*)	K6	30.54	82.24	1.1935483	1.2375	0.741935	1.0576613
Chmp2b	Q8BJF9	81	TFAVSSK(*)VTSMSTQTK(*)	K7	45.64	123.34	0.7339449	0.574712	0.682692	0.6637833
Chmp2b	Q8BJF9	107	MAGAMSTTAK(*)TMQAVNK(*)	K10	41.06	89.98	-	0.919540	0.865384(	0.8924624
Chmp2b	Q8BJF9	90	VTSMSTQTK(*)VMNSQMK(*)	K9	40.98	67.33	0.7706422	0.620689	-	0.6956659
Tyw1	Q8BJM7	385	GGC(+57.02)YK(*)HTFYGIESHR(*)	K5	32.43	1000	-	0.568627	0.609929(	0.5892783
Sun2	Q8BJS4	406	VQQLK(*)TEWK(*)	K5	11.06	82.24	-	-	3.126213592	

Sgta	Q8BJU0	161	AIGIDPGYSK(*)AYGR(*)	K10	79.84	1000	0.9484536	0.925925	0.791666(	0.8886821
Sgta	Q8BJU0	96	LK(*)TEGNEQMK(*)	K2	79.19	183.32	0.8865979	0.740740	0.875	0.8341129
Sgta	Q8BJU0	197	ALELDPDNDTYK(*)SNLK(*)	K12	52.81	28.7	0.9278350	0.827160	0.552083:	0.7690263
Sgta	Q8BJU0	175	MGLALSSLNK(*)HAEAVAYYK(*)	K10	38.57	20.18	1.0721649	1.111111	0.90625	1.029842
Sgta	Q8BJU0	175	SSLNK(*)HAEAVAYYK(*)	K5	24.26	90.94	-	-	0.5	
Nol11	Q8BJW5	185	LC(+57.02)K(*)SHSLSK(*)	K3	12.28	104.48	1.25	1.535714	-	1.3928571
Eif2a	Q8BJW6	223	ADK(*)VTMLWNK(*)	K3	68.66	184.45	0.9438202	0.797468	1.117647(	0.9529785
Eif2a	Q8BJW6	121	TGAC(+57.02)LK(*)SFIQK(*)	K6	46.42	115.97	0.5842696	-	1.164705!	0.8744878
Eif2a	Q8BJW6	192	VAVYVPGSK(*)GAPSFVR(*)	K9	22.29	1000	0.8988764	-	2.223529	1.5612029
Eif2a	Q8BJW6	216	FAGPQAALANK(*)SFFK(*)	K11	16.44	24.67	1.5056179	-	1.470588:	1.4881031
Psmd5	Q8BJY1	351	VGYQAK(*)NASTELK(*)	K6	64.81	128.65	0.9191919	0.740740	-	0.8299663
Psmd5	Q8BJY1	147	AAIK(*)SLSR(*)	K4	23.36	1000	-	-	0.244680851	
Psmd5	Q8BJY1	334	QVLQK(*)TGTR(*)	K5	17.39	1000	0.6565656	-	0.882978	0.7697722
Mrps35	Q8BJZ4	297	LK(*)NEGENEASLAQYK(*)	K2	47.05	244	0.5523809	0.263636	0.901960	0.5726594
Csnk1a1	Q8BK63	8	A(+42.01)SSSGSK(*)AEFIVGGK(*)	K7	85.41	148.75	1.0465116	1.026666	1.192307(	1.0884953
Csnk1a1	Q8BK63	130	IEYVHTK(*)NFIHR(*)	K7	62.89	1000	0.9883720	0.68	-	0.834186
Csnk1a1	Q8BK63	62	HPQLLYESK(*)LYK(*)	K9	56.54	53.53	0.9534883	0.84	1.115384(	0.9696243
Csnk1a1	Q8BK63	65	LYK(*)ILQGGVGIPHIR(*)	K3	34.12	1000	-	-	1.076923077	
Ahsa1	Q8BK64	94	LNWTGTSK(*)SGVQYK(*)	K8	78.69	102.87	0.7977528	0.810126	0.820224	0.809368
Ahsa1	Q8BK64	273	HIAMK(*)WR(*)	K5	24.84	1000	-	-	0.97752809	
Rcc2	Q8BK67	75	PATAGK(*)AAGAAAIITEPEHTK(*)	K6	49.77	179.51	0.5543478	0.828947	0.532608(	0.6386346
Ipo5	Q8BKC5	806	LEEHFK(*)NQELR(*)	K6	89.22	1000	1.2631578	1.075949	0.925531!	1.0882131
Ipo5	Q8BKC5	1052	IFSIIAEGEMHEAIK(*)HEDPC(+57.02	K15	59.93	56.54	0.4631578	0.670886	1.053191	0.7290785
Ipo5	Q8BKC5	806	AK(*)LEEHFK(*)NQELR(*)	K8	58.89	111.2	1.9157894	1.341772	1.106382!	1.4546482
Ipo5	Q8BKC5	556	GK(*)TIEC(+57.02)ISLIGLAVGK(*)	K2	57.45	314.84	0.9157894	-	1.010638:	0.9632139
Ipo5	Q8BKC5	963	TK(*)ENVNATENC(+57.02)ISAVGK(*	K2	49.41	241.23	1.1052631	1.886075	1.031914!	1.3410847
Ipo5	Q8BKC5	800	AK(*)LEEHFK(*)	K2	43.51	102.87	0.9684210	-	1.563829	1.2661254
Ipo5	Q8BKC5	360	IK(*)EHIMQMLQNPDWK(*)	K2	37.21	191.64	1.2210526	2.215189	0.946808!	1.461017
Ipo5	Q8BKC5	41	SK(*)ITFLLQAIR(*)	K2	33.77	1000	1.5473684	-	0.914893(	1.231131
Ipo5	Q8BKC5	1052	SIIAEGEMHEAIK(*)HEDPC(+57.02)A	K13	23.27	26.08	-	-	1.138297872	
Ipo5	Q8BKC5	539	LK(*)HIVENAVQK(*)	K2	15.11	140.79	-	-	1.095744681	
Cyp20a1	Q8BKE6	95	QHFNPNK(*)TSDPFETMLK(*)	K7	28.1	96.46	1.6704545	-	0.894117(	1.2822861
Ptk7	Q8BKG3	124	WIEAGPVVLK(*)HPASEAEIQPQTQVT	K10	50.48	1000	-	-	1	
Pum3	Q8BKS9	578	EGC(+57.02)FAK(*)TLVER(*)	K6	52.18	1000	-	0.573170	0.5625	0.5678354

Pum3	Q8BKS9	138	AK(*)HIWESLR(*)	K2	44.85	1000	0.9239130	0.536585	0.96875	0.8097495
Pum3	Q8BKS9	170	IK(*)TIAFAHDSTR(*)	K2	31.3	1000	0.5760869	0.951219	0.770833:	0.7660466
Pum3	Q8BKS9	323	EAVIK(*)HSLVHK(*)	K5	25.06	102.87	-	-	1	
Pum3	Q8BKS9	384	K(*)VIVK(*)TMK(*)	K5	8.73	50.22	-	0.524390	-	
Thoc5	Q8BKT7	424	K(*)TPNPANQYQFDK(*)VGILTLR(*)	K13	39.11	116.72	0.8316831	0.676767	0.842105:	0.7835187
Thoc5	Q8BKT7	424	TPNPANQYQFDK(*)VGILTLR(*)	K12	11.3	1000	-	-	1.010526316	
Smg1	Q8BKX6	1796	LLVK(*)HAGELR(*)	K4	12.65	1000	-	-	0.842696629	
Pdhx	Q8BKZ9	194	NILEK(*)HSLDASQGTATGPR(*)	K5	72.34	1000	1.3333333	0.757142	0.892473:	0.9943164
Eea1	Q8BL66	647	AK(*)TELLLSAEAAK(*)	K2	79	212.2	1.0638297	0.802631	0.833333:	0.8999316
Eea1	Q8BL66	324	DLDYTHLEEK(*)HNEESASR(*)	K10	74.66	1000	-	0.947368	0.966666(	0.9570175
Eea1	Q8BL66	746	ASK(*)EQALQSLQQQR(*)	K3	62.16	1000	1.0425531	0.855263	0.944444	0.9474203
Eea1	Q8BL66	898	ELK(*)HQLQVQAESALK(*)	K3	48.21	276.94	1.2872340	1.157894	1.311111:	1.25208
Eea1	Q8BL66	791	LDLQNK(*)SEILENIK(*)	K6	41.4	131.72	1.2659574	0.973684	0.855555!	1.0317324
Eea1	Q8BL66	717	EK(*)HLSLEQK(*)	K2	40.05	120.29	-	7.25	0.822222:	4.0361111
Eea1	Q8BL66	983	QIEALQGEVK(*)NAVSQK(*)	K10	34.32	69.64	-	-	0.333333333	
Eea1	Q8BL66	1340	ENQSLQIK(*)HTQALNR(*)	K8	29.08	1000	0.9148936	1.342105	1	1.0856663
Eea1	Q8BL66	303	GSINELTQK(*)NQNLTEK(*)	K9	25.35	93.6	2.4787234	-	1.577777	2.0282506
Eea1	Q8BL66	294	LK(*)GSINELTQK(*)	K2	12.49	93.23	-	-	0.744444444	
Eea1	Q8BL66	58	SLGSADELFK(*)HYQAVHDAGNDSGH	K10	7.12	1000	-	-	1.455555556	
Srsf7	Q8BL97	99	GLDGK(*)VIC(+57.02)GSR(*)	K5	88.71	1000	1.0329670	0.865168	1.109890:	1.0026752
Srsf7	Q8BL97	53	VYVGNLGTGAGK(*)GELER(*)	K12	53.66	1000	0.9890109	0.943820	0.923076!	0.9519694
Lss	Q8BLN5	15	GGPYK(*)TEPATDLTR(*)	K5	31.37	1000	-	3.011494	0.923076!	1.9672856
Lss	Q8BLN5	367	IK(*)DYLWLGLDGMK(*)	K2	29.61	106.36	-	-	0.857142857	
Prpf18	Q8BM39	300	IFSK(*)HVAHVLNDETQR(*)	K4	29.62	1000	-	-	-	
Prpf18	Q8BM39	327	LMTIC(+57.02)QK(*)HFPTDPSK(*)	K7	23.91	46.32	-	-	0.580769231	
Npat	Q8BMA5	559	VK(*)TNPQASESADSSETANR(*)	K2	26.89	1000	-	-	-	
Npat	Q8BMA5	528	NLVLSGK(*)NSQLLSQSTPLTTK(*)PSk	K7	21.17	28.73	-	-	-	
Npat	Q8BMA5	1125	ILSK(*)SETASSR(*)	K4	20.33	1000	-	-	-	
Srp68	Q8BMA6	456	EISLK(*)TLVFK(*)	K5	45.63	94.22	0.8383838	0.7625	1.033333:	0.8780724
Srp68	Q8BMA6	204	FEHQEWK(*)SAIEAFNK(*)	K7	43.97	84.61	1.3232323	1.4	1.322222:	1.3484848
Srp68	Q8BMA6	610	TK(*)SGLTGYIK(*)	K2	42.45	124.34	0.7373737	-	0.733333:	0.7353535
Srp68	Q8BMA6	163	AVK(*)HAEELER(*)	K3	23.71	1000	0.8686868	-	1.933333:	1.4010101
Slc25a24	Q8BMD8	318	LAVAK(*)TGQYSGIYGC(+57.02)AK(*	K5	90.19	243.69	0.8556701	0.828947	0.987951:	0.8908564
Slc25a24	Q8BMD8	96	AFK(*)SLDK(*)	K3	80.28	87.62	1.0515463	1.105263	0.987951:	1.0482538

Slc25a24	Q8BMD8	334	ILK(*)HEGFGAFYK(*)	K3	76.92	176.79	0.7835051	0.894736	0.963855	0.8806991
Slc25a24	Q8BMD8	96	LAFK(*)SLDK(*)	K4	27.62	63.97	0.7835051	0.802631	-	0.7930684
Slc25a24	Q8BMD8	311	IYPMEVLK(*)TR(*)	K8	23.32	1000	-	-	1.060240964	
Slc25a32	Q8BMG8	268	LQDQHVSYGGVTDVITK(*)TWR(*)	K17	37.03	1000	-	-	1.453488372	
Lars	Q8BMJ2	916	LK(*)NYMMPAK(*)	K2	80.12	142.27	0.75	0.740259	0.833333	0.774531
Lars	Q8BMJ2	721	MSK(*)STGNFLTLSQAVDK(*)	K3	72.69	293.07	-	0.870129	-	
Lars	Q8BMJ2	649	DAPFPK(*)TQIPK(*)	K6	60.32	52.68	0.6847826	0.597402	0.655555	0.6459136
Lars	Q8BMJ2	824	EALK(*)TGFFEFQAAK(*)	K4	56.16	166.08	0.9347826	-	0.866666	0.9007246
Lars	Q8BMJ2	1086	TEPGVPVSLVNPQPSSGHFSTK(*)IDIF	K22	54.92	1000	-	-	0.966666667	
Lars	Q8BMJ2	200	TIGLK(*)VDWR(*)	K5	47.97	1000	0.9456521	-	0.922222	0.9339372
Lars	Q8BMJ2	84	GK(*)SC(+57.02)LFPFGLHC(+57.02)	K2	46.7	149.49	0.7391304	1.064935	1.077777	0.9606144
Lars	Q8BMJ2	272	LK(*)VLEPYPSK(*)	K2	39.21	150.89	0.5760869	-	1.1	0.8380435
Lars	Q8BMJ2	9	K(*)GTAK(*)VDFLK(*)	K5	36.72	28.7	0.8260869	0.896103	-	0.8610954
Lars	Q8BMJ2	1058	EEC(+57.02)C(+57.02)PGK(*)PLNVF	K7	7.47	1000	-	-	1.688888889	
Lars	Q8BMJ2	71	SLSK(*)C(+57.02)EFAVGYQR(*)	K4	4.1	1000	-	-	4.844444444	
Eif1ax	Q8BMJ3	88	DYQDNK(*)ADVILK(*)	K6	90.38	134.33	1.3052631	1.041666	0.988888	1.1119396
Ckap4	Q8BMK4	567	LFLK(*)VEK(*)	K4	79.06	62.26	-	-	1.010989011	
Ckap4	Q8BMK4	312	ELVSLK(*)QEQQAFK(*)	K6	54.97	128.65	-	0.797468	-	
Ckap4	Q8BMK4	539	SVK(*)IETNENNLESAK(*)	K3	46.3	220.17	1.4343434	1.367088	1.494505	1.4319792
Ckap4	Q8BMK4	251	MK(*)VASLEESK(*)	K2	11.61	86.34	1.8484848	-	-	
Qars	Q8BML9	25	ETLK(*)NEALSTQLR(*)	K4	67.6	1000	0.84375	0.694117	0.75	0.7626225
Qars	Q8BML9	166	MIK(*)NEVDMQVLHLLGPK(*)	K3	35.7	174.34	0.8958333	-	0.885416	0.890625
Hadha	Q8BMS1	60	INSPNSK(*)VNTLNK(*)	K7	51.53	113.99	0.8543689	0.8125	0.623655	0.7635083
Ncs1	Q8BNY6	53	IYK(*)QFFPFGDPTK(*)	K3	10.45	82.84	-	-	1.607954545	
Sfr1	Q8BP27	255	IK(*)NDVTELENLIK(*)	K2	46.28	203.1	0.5	1.142857	0.876404	0.8397539
Sfr1	Q8BP27	202	NTC(+57.02)ESK(*)SSDTGSSNALPK(	K6	21.41	167.25	1.9102564	1.271428	1.449438	1.5437077
NARS1	Q8BP47	140	VK(*)VFGWVHR(*)	K2	81.81	1000	0.7849462	-	0.860215	0.8225806
NARS1	Q8BP47	115	IIIK(*)NDPSLPEPAC(+57.02)VK(*)	K4	77.33	160.66	1.1505376	0.740740	0.784946	0.8920749
NARS1	Q8BP47	153	QGK(*)NLMFLVLR(*)	K3	55.29	1000	0.7634408	0.814814	0.903225	0.8271605
NARS1	Q8BP47	456	FPVEIK(*)SFYMQR(*)	K6	54.89	1000	0.7634408	0.864197	0.849462	0.8257003
NARS1	Q8BP47	71	WDVISK(*)SQMK(*)	K6	17.8	44.28	0.9032258	-	0.978494	0.9408602
Rpl24	Q8BP67	27	TDGK(*)VFQFLNAK(*)	K4	92.02	152.83	0.9029126	0.974025	0.905263	0.9274006
Rpl24	Q8BP67	12	VELC(+57.02)SFSGYK(*)IYPGHGR(*)	K10	85.63	1000	0.5825242	0.753246	0.747368	0.6943798
Rpl24	Q8BP67	2	MK(*)VELC(+57.02)SFSGYK(*)	K2	64.97	188.26	0.9708737	0.935064	1.063157	0.9896989

Fra10ac1	Q8BP78	36	TVEDELLLTK(*)PFQK(*)	K10	35.26	20.17	-	-	-	
Rcn2	Q8BP92	27	SK(*)AEELHYPQGEHR(*)	K2	26.47	1000	1.2790697	0.337837	0.943181	0.8533631
Map4k5	Q8BPM2	797	SFK(*)SDEVTQEISDETR(*)	K3	16.35	1000	1.3023255	1.378048	-	1.3401872
Elmo1	Q8BPU7	408	EDK(*)HEC(+57.02)PFGR(*)	K3	28.33	1000	-	-	1.477272727	
Cpsf4	Q8BQZ5	69	TVVC(+57.02)K(*)HWLR(*)	K5	59.6	1000	0.9587628	1.055555	1.833333	1.2825506
Cpsf4	Q8BQZ5	51	AAC(+57.02)GK(*)GGMC(+57.02)PI	K5	39.49	1000	-	0.6	2.355555	1.4777778
Kat6b	Q8BRB7	860	QSSAK(*)VQSK(*)NK(*)	K9	77.22	30.36	-	-	-	
Kat6b	Q8BRB7	856	QSSAK(*)VQSK(*)NK(*)	K5	77.22	129.19	-	-	-	
Kat6b	Q8BRB7	413	LAVTDPTR(*)PGATTK(*)TTTSSTYISAS	K14	55.26	214.3	0.3654618	-	-	
Kat6b	Q8BRB7	394	VSTTPSSGHAASGK(*)HSSSR(*)	K14	45.18	1000	-	-	-	
Scfd1	Q8BRF7	428	IMSK(*)TTLDK(*)	K4	66.1	43.82	0.7826086	-	0.977528	0.8800684
Tmtc3	Q8BRH0	742	YYPDHTK(*)GLILK(*)	K7	37.48	65.44	-	-	0.895348837	
Tmtc3	Q8BRH0	634	ALELNPK(*)HK(*)	K7	34.71	39.76	1.2465753	-	-	
Kmt2c	Q8BRH4	2862	GK(*)TALLTTDQDMLEK(*)	K2	98.85	335.51	-	-	-	
Kmt2c	Q8BRH4	2796	DQGDK(*)TMVLEDK(*)	K5	91.9	158.8	-	-	-	
Kmt2c	Q8BRH4	2796	DQGDK(*)TMVLEDK(*)DLPQR(*)	K5	81.5	157.88	-	-	-	
Kmt2c	Q8BRH4	820	IGMGK(*)PAITK(*)	K5	59.62	32.32	-	-	-	
Pbrm1	Q8BSQ9	754	DALVLHK(*)VLLETR(*)	K7	36.97	1000	0.7710843	0.9	1.113924	0.9283361
Pbrm1	Q8BSQ9	128	AYYK(*)PDSPEYK(*)	K4	34.19	46.13	-	-	0.873417722	
Pbrm1	Q8BSQ9	1167	VGDC(+57.02)VFIK(*)SHGLVR(*)	K8	24.88	1000	-	-	1.050632911	
Asph	Q8BSY0	629	GAPK(*)TC(+57.02)ALLEK(*)	K4	48.18	46.67	-	0.818181	0.979381	0.8987816
Asph	Q8BSY0	571	ETGYTELVK(*)SLER(*)	K9	8.68	1000	-	-	0.865979381	
Cep55	Q8BT07	387	AK(*)SQITQLESLK(*)	K2	72.07	251.58	1	0.752941	0.806451	0.8531309
Cep55	Q8BT07	340	ILYDSLLK(*)HQEEQAR(*)	K8	65.74	1000	1.0322580	-	0.806451	0.9193548
Cep55	Q8BT07	129	ETDVLK(*)NQLSATTK(*)	K6	3.34	68.3	-	-	1.225806452	
Cpne3	Q8BT60	167	SDPYLEFHK(*)QTSDGHWLMVHR(*)	K9	61.12	1000	0.9278350	-	0.880434	0.9041349
Cpne3	Q8BT60	262	SYK(*)NSGVISVK(*)	K3	37.33	154.74	0.4639175	0.827160	0.456521	0.5825333
Cpne3	Q8BT60	158	DLFGK(*)SDPYLEFHK(*)	K5	19.52	96.27	1.8762886	1.888888	1.815217	1.8601316
Cpne3	Q8BT60	52	IK(*)NSLNPK(*)	K2	8.71	98.75	-	1.432098	-	
Flna	Q8BTM8	2607	TPC(+57.02)EEILVK(*)HMGSR(*)	K9	98.22	1000	0.9468085	0.870129	0.977272	0.9314037
Flna	Q8BTM8	781	VYGPGVAK(*)TGLK(*)	K8	95.61	63.97	0.8829787	0.779220	0.829545	0.8305817
Flna	Q8BTM8	2575	AYVGQK(*)SNFTVDC(+57.02)SK(*)	K6	84.31	181.94	0.9361702	0.909090	0.636363	0.8272083
Flna	Q8BTM8	508	VYTK(*)GAGSGELK(*)	K4	81.7	161.49	0.7872340	0.896103	0.920454	0.8679308
Flna	Q8BTM8	2569	GLGLSK(*)AYVGQK(*)	K6	81.63	102.87	0.9893617	0.935064	0.761363	0.8952634

Flna	Q8BTM8	2623	DK(*)GEYTLVVK(*)	K2	79.53	150.77	0.8936170	0.818181	0.965909(	0.8925693
Flna	Q8BTM8	376	SPFEVYVDK(*)SQGDASK(*)	K9	71.29	71.72	-	0.818181	0.818181!	0.8181818
Flna	Q8BTM8	1007	VASK(*)IVSPSGAAVPC(+57.02)K(*)	K4	57.8	126.66	0.7340425	0.584415	0.795454!	0.7046376
Flna	Q8BTM8	771	VNVGAGSHPNK(*)VK(*)	K11	56.68	24.32	0.9255319	-	0.829545	0.8775387
Flna	Q8BTM8	700	TGVAVNK(*)PAEFTVDAK(*)HAGK(*)	K16	49.32	42.89	0.9148936	0.883116	0.897727	0.8985793
Flna	Q8BTM8	700	PAEFTVDAK(*)HAGK(*)	K9	43.73	38.03	1.1808510	-	-	
Flna	Q8BTM8	684	GPGLEK(*)TGVAVNK(*)PAEFTVDAK(	K6	42.97	81.81	-	-	0.738636364	
Flna	Q8BTM8	906	GK(*)LDVQFSGLAK(*)	K2	39.59	192.46	0.6808510	1.285714	1.034090!	1.0002188
Flna	Q8BTM8	1294	ALTQTGGPHVK(*)AR(*)	K11	39.18	1000	1.4893617	1.129870	0.965909(	1.195047
Flna	Q8BTM8	975	IK(*)VSGLGDK(*)	K2	34.96	51.59	-	-	0.818181818	
Flna	Q8BTM8	2367	GAIDAK(*)VHSPSGALEEC(+57.02)Y\	K6	34.18	48.6	-	-	1.545454545	
Flna	Q8BTM8	383	SQGDASK(*)VTAQGPGLEPSGNIANK	K7	33.63	188.86	0.6808510	1.194805	-	0.9378281
Flna	Q8BTM8	1477	ANLPQSFQVDTSK(*)AGVAPLQVK(*)	K13	29.92	74.73	0.9574468	-	-	
Flna	Q8BTM8	2133	FADQHVPGSPFSVK(*)VTGEGR(*)	K14	26.57	1000	1.4468085	-	1.363636:	1.4052224
Flna	Q8BTM8	2217	YK(*)GQHVPGSPFQFTVGPLGEGGAH	K2	23.65	175.6	1.1702127	1.194805	1.011363(	1.1254605
Flna	Q8BTM8	569	SPFEVK(*)VGTEC(+57.02)GNQK(*)	K6	22.96	99.51	1.1063829	1.038961	1.022727	1.0560238
Flna	Q8BTM8	2584	SNFTVDC(+57.02)SK(*)AGNNMLLV(	K9	22.05	1000	-	1.870129	1.602272	1.7362013
Flna	Q8BTM8	2473	VK(*)MDC(+57.02)QEC(+57.02)PEG	K2	17.7	1000	-	1.324675	-	
Flna	Q8BTM8	2540	LVSNHSLHETSSVFVDSLTK(*)VATVP(	K20	16.81	61.11	-	-	1.295454545	
Flna	Q8BTM8	270	LK(*)PGAPLR(*)PK(*)	K2	14.83	107.29	-	-	1.022727273	
Flna	Q8BTM8	865	VK(*)VEPSHDASK(*)	K2	14.23	60.15	-	0.922077	-	
Flna	Q8BTM8	2387	VHSPSGALEEC(+57.02)YVTEIDQDK(	K20	4.73	1000	-	-	0.488636364	
Gmppb	Q8BTZ7	137	HHGQEGSILVTK(*)VEEPSK(*)	K12	30.86	78.19	0.7410714	1.459016	-	1.1000439
Pwp2	Q8BU03	43	VTVFDLK(*)NNR(*)	K7	17.68	1000	0.8795180	1.442857	1.354838	1.225738
Iars	Q8BU30	1115	LK(*)TVVTSVFGVK(*)	K2	84.93	131.64	0.8617021	-	0.936170	0.8989362
Iars	Q8BU30	1125	TVVTSVFGVK(*)NAK(*)	K10	79.1	24.24	0.8617021	0.975	1.095744(	0.9774823
Iars	Q8BU30	132	YSAEWK(*)STVTR(*)	K6	74.21	1000	-	0.7	-	
Iars	Q8BU30	24	ILEFWSK(*)HNC(+57.02)FQEC(+57.(	K7	61.47	45.63	0.9255319	0.9625	0.914893(	0.9343085
Iars	Q8BU30	290	YK(*)PLFDYFIK(*)	K2	48.92	89.48	1	-	0.978723	0.9893617
Iars	Q8BU30	796	DK(*)DTLSIHYLMLPR(*)	K2	42.9	1000	-	-	0.840425532	
Iars	Q8BU30	254	GK(*)LFILTEAR(*)	K2	29.17	1000	-	-	1	
Hook3	Q8BUK6	62	IK(*)TEVGDNWR(*)	K2	67.66	1000	0.8	0.852272	0.735632	0.7959683
Dock1	Q8BUR4	152	VTAK(*)IDYGNR(*)	K4	8.48	1000	-	-	1.542857143	
Gphn	Q8BUV3	138	GK(*)TLIINLPGSK(*)	K2	10.22	133.99	-	-	1.086538462	

Pggt1b	Q8BUY9	284	LLK(*)IFQYTNFEK(*)	K3	10.77	128.89	-	-	-	
Atp6v1h	Q8BVE3	371	LEWSPVHK(*)SEK(*)	K8	53.57	26.52	1.1153846	0.914893	0.979591	1.00329
Nsd2	Q8BVE8	588	SQAATK(*)NLSDAC(+57.02)K(*)PLK	K6	39.08	67.12	-	0.640449	1	0.8202247
Pdcl3	Q8BVF2	206	LSESGAIK(*)TALEENPK(*)	K8	31.52	60.24	0.9569892	1.922077	0.439024	1.1060305
Qdpr	Q8BVI4	99	SLFK(*)NC(+57.02)DMMWK(*)	K4	68.68	118.11	-	-	1.489130435	
Ppme1	Q8BVQ5	121	VK(*)NSEDLSAETMAK(*)	K2	48.08	223.58	0.5858585	0.666666	0.831578	0.6947014
Ppme1	Q8BVQ5	217	SLENAIEWSVK(*)SGQIR(*)	K11	39.61	1000	-	-	0.852631579	
Rsl1d1	Q8BVY0	253	LLFLK(*)TEK(*)	K5	82.35	37.81	0.7553191	0.696202	1.011494	0.8210053
Rsl1d1	Q8BVY0	187	K(*)VPVSVNLLAK(*)NLSK(*)	K11	58.77	38.26	0.9361702	0.506329	0.862068	0.7681894
Rsl1d1	Q8BVY0	187	VPVSVNLLAK(*)NLSK(*)	K10	54.63	58.99	0.6382978	0.810126	1.080459	0.8429614
Nob1	Q8BW10	282	VFC(+57.02)GHC(+57.02)GNK(*)TL	K9	47.36	40.63	0.7659574	0.471264	1.373493	0.8702386
Ptges2	Q8BWM0	114	TC(+57.02)PFC(+57.02)SK(*)VR(*)	K7	27.4	1000	-	-	1.258823529	
Vps35l	Q8BWQ6	309	FLSK(*)TGISEC(+57.02)LPR(*)	K4	37.33	1000	0.9896907	-	0.782178	0.8859345
Acaa2	Q8BWT1	340	ALDLDPSK(*)TNVSGGAIALGHPLGG	K8	11.35	1000	-	-	1.747126437	
Larp4	Q8BWW4	256	EEVK(*)TFQGK(*)	K4	49.77	45.16	-	-	0.735632184	
Larp4	Q8BWW4	261	TFQGK(*)PIMAR(*)	K5	24.32	1000	0.5294117	-	0.908045	0.7187289
Larp4	Q8BWW4	216	ALFK(*)NENC(+57.02)PK(*)	K4	17.06	95.35	-	1.077922	-	
Etf1	Q8BWY3	22	LIK(*)SLEAAR(*)	K3	57.21	1000	1	0.875	0.978947	0.9513158
Etf1	Q8BWY3	354	DK(*)SHFTDK(*)	K2	43.33	74.6	-	-	0.863157895	
Etf1	Q8BWY3	249	LQSK(*)VLK(*)	K4	30.35	53.53	1.1505376	-	-	
Cip2a	Q8BWY9	21	AVK(*)SEANATQLLR(*)	K3	32.39	1000	1.0101010	-	0.5	0.7550505
Naa25	Q8BWZ3	432	LLGLYHSMDK(*)SQK(*)	K10	34.22	44.44	-	1.469135	0.914893	1.1920147
Naa25	Q8BWZ3	170	DENLSK(*)TMFLPLAER(*)	K6	12.08	1000	1.4736842	-	0.840425	1.1570549
Rbbp5	Q8BX09	202	VTTGTSNTTAIK(*)SIEFAR(*)	K12	22.91	1000	-	1.192771	0.649350	0.9210609
Rbbp5	Q8BX09	179	ILVLK(*)TDSQDLVASFR(*)	K5	9.96	1000	-	-	1.038961039	
Pgam5	Q8BX10	161	AVETTDIISK(*)HLPGVSR(*)	K10	61.23	1000	0.9518072	0.891891	1.079545	0.9744149
Pgam5	Q8BX10	143	FNK(*)IVHSSMTR(*)	K3	15.3	1000	-	1.472972	-	
Gemin5	Q8BX17	428	SK(*)VTALC(+57.02)WHPNK(*)	K2	67.52	235.31	1	0.807228	0.806451	0.8712268
Gemin5	Q8BX17	426	NFWQGVK(*)SK(*)	K7	55.34	39.76	2.0888888	1.132530	2.086021	1.7691468
Gemin5	Q8BX17	998	VYEAVELLK(*)SNHLYR(*)	K9	43.93	1000	-	-	1.161290323	
Gemin5	Q8BX17	419	NNYDVK(*)NFWQGVK(*)	K6	39.45	81.81	-	-	0.870967742	
Gemin5	Q8BX17	344	IVFNLC(+57.02)SLK(*)TEDGK(*)	K9	12.33	34.83	10.433333	-	18.89247	14.662903
Vps13c	Q8BX70	192	LATQVIK(*)NVQVK(*)	K7	22.72	67.85	0.9047619	0.955882	0.6	0.8202148
Fndc3a	Q8BX90	384	TK(*)NSLTLQWK(*)	K2	91.66	193.63	1.1911764	1.059701	1.075949	1.1089424

Fndc3a	Q8BX90	528	YDGEDLAYTVK(*)NLR(*)	K11	7.21	1000	-	-	1.265822785	
Trerf1	Q8BXJ2	646	K(*)HPPIAAK(*)VEEPLK(*)	K8	77.21	114.34	-	-	-	
Tmx3	Q8BXZ1	270	LK(*)SIIQEVAR(*)	K2	71.53	1000	0.9325842	0.830985	0.865168!	0.8762462
Tmx3	Q8BXZ1	122	TK(*)DDIIEFAHR(*)	K2	51.14	1000	1.0224719	0.985915	1.056179	1.0215224
Hat1	Q8BY71	12	FLVEYK(*)SAVEK(*)	K6	82.37	103.33	1.0224719	0.904761	1.057471	0.9949017
Usp47	Q8BY87	1127	VK(*)VC(+57.02)QLLVNEQEPC(+57.	K2	41.99	209.69	-	-	1.313253012	
Usp47	Q8BY87	710	K(*)PDQIFQSYK(*)PGEVMVK(*)	K10	31.47	46.13	0.7362637	0.679487	0.469879!	0.6285435
Tbcd	Q8BYA0	336	LC(+57.02)APGK(*)SDQK(*)	K6	43.47	22.37	1.0267857	1.416666	1.173469	1.2056406
Poglut1	Q8BYB9	93	LGTHYQIIK(*)NR(*)	K9	6.01	1000	-	0.987951	1.458823!	1.2233877
Tbc1d17	Q8BYH7	355	LQWK(*)SVSAEQER(*)	K4	16.63	1000	2.5408163	1.048543	1.149425	1.5795951
Ythdf3	Q8BYK6	554	IIATFK(*)HTTSIFDDFAHYEK(*)	K6	50.95	132.96	-	-	0.93258427	
Parp12	Q8BZ20	443	FQAGK(*)HNYELHFK(*)	K5	58.89	40.94	-	-	-	
Kat6a	Q8BZ21	816	VK(*)VGK(*)SVSR(*)	K5	90.32	1000	-	-	-	
Kat6a	Q8BZ21	813	VK(*)VGK(*)SVSR(*)	K2	90.32	1000	-	-	-	
Kat6a	Q8BZ21	94	LDTK(*)QSVDWNK(*)	K4	67.38	142.27	-	-	-	
Kat6a	Q8BZ21	355	GPFSK(*)VR(*)	K5	14.09	1000	-	-	-	
Pogz	Q8BZH4	828	LAC(+57.02)TSC(+57.02)TFATSVGD	K18	38.74	1000	-	-	0.885057471	
Ncbp3	Q8BZR9	482	PAVSSTK(*)PDIR(*)	K7	34.67	1000	-	0.698795	1.014705	0.8567505
Ncbp3	Q8BZR9	446	ADSISTSNIK(*)NR(*)	K10	7.61	1000	-	-	1.926470588	
Srek1	Q8BZX4	186	ISAAIEPESGK(*)SNER(*)	K11	35.68	1000	0.49375	-	0.387096	0.4404234
Farsa	Q8C0C7	229	GVLPDSGHLHPLLK(*)VR(*)	K14	67.58	1000	0.8484848	0.894736	1.122222	0.955148
Farsa	Q8C0C7	397	EFFTK(*)LGITQLR(*)	K5	63.09	1000	0.4747474	0.460526	0.755555!	0.5636098
Farsa	Q8C0C7	481	ELVGHK(*)VNLQMVYDSPVC(+57.02	K6	38.15	1000	0.8282828	-	1.577777	1.2030303
Farsa	Q8C0C7	406	FK(*)PAYNPYTEPSMEVFSYHQGLK(*)	K2	31.56	89.11	1.0101010	-	1.022222	1.0161616
Ing4	Q8C0D7	148	SK(*)GK(*)NSDEEAPK(*)	K4	84.56	150.89	-	-	-	
Ing4	Q8C0D7	146	SK(*)GK(*)NSDEEAPK(*)	K2	84.56	213.35	-	-	-	
Ing4	Q8C0D7	236	GK(*)WFC(+57.02)PR(*)	K2	75.98	1000	-	-	-	
Ing4	Q8C0D7	156	SK(*)GK(*)NSDEEAPK(*)AAQK(*)	K12	25.25	47.31	-	-	-	
Ing4	Q8C0D7	148	SK(*)GK(*)NSDEEAPK(*)AAQK(*)	K4	25.25	261.96	-	-	-	
Ing4	Q8C0D7	146	SK(*)GK(*)NSDEEAPK(*)AAQK(*)	K2	25.25	320.47	-	-	-	
Vps26b	Q8C0E2	203	IK(*)HMEIDIIK(*)	K2	42.4	193.63	0.7314814	0.782051	1.072916	0.8621498
Trim47	Q8C0E3	365	ELSFTK(*)SSQVVK(*)	K6	45.96	90.89	-	-	0.848214286	
Trim47	Q8C0E3	261	TVALIK(*)SAAVAER(*)	K6	11.22	1000	0.6226415	-	-	
Epc2	Q8C0I4	23	GK(*)DMPDLNDC(+57.02)VSINR(*)	K2	53.46	1000	-	-	-	

Atg16l1	Q8C0J2	45	LLEK(*)SDLHSVLTQK(*)	K4	19.94	104.83	-	-	0.816	
Atg16l1	Q8C0J2	91	IK(*)HQEELTELHK(*)	K2	8.73	192.46	-	-	0.8	
Sowahc	Q8C0J6	51	FK(*)ELVNAVATVR(*)	K2	9.26	1000	-	-	0.303030303	
Asrgl1	Q8C0M9	287	GLGGLILVNK(*)TGDWVAK(*)	K10	37.68	51.17	-	-	-	
Ncaph	Q8C156	638	LK(*)QSMWSLLTK(*)	K2	67.66	182.71	-	-	0.935483871	
Ncaph	Q8C156	627	IEIHYAK(*)TAK(*)	K7	66.3	38.16	0.8144329	0.813333	0.849462:	0.8257429
Ncaph	Q8C156	620	VNK(*)IEIHYAK(*)	K3	45.84	154.98	1.1752577	0.666666	0.838709(	0.8935447
Ncaph	Q8C156	488	AATILTK(*)STLENQNWK(*)	K7	24.06	106.84	-	0.946666	-	
Cpne1	Q8C166	170	QGDGK(*)WQLAYR(*)	K5	88.62	1000	0.83	0.8625	0.968085:	0.8868617
Cpne1	Q8C166	181	TEVVK(*)NNLNPTWK(*)	K5	81.57	171.6	0.89	0.825	0.978723	0.8979078
Prepl	Q8C167	437	LFEETGHEDPITK(*)TSR(*)	K13	29.82	1000	-	-	0.844444444	
Cers6	Q8C172	92	VFTAITK(*)HPDEK(*)	K7	65.73	51	-	-	-	
Mbnl2	Q8C181	41	FAHPPK(*)SC(+57.02)QVENGR(*)	K6	37.8	1000	-	-	0.705882353	
Mbnl2	Q8C181	178	TDK(*)LEVC(+57.02)R(*)	K3	33.98	1000	-	0.464646	0.302521(	0.3835837
Mtrr	Q8C1A3	596	HFLK(*)TGVLTHLK(*)	K4	10.92	118.18	-	-	-	
Thop1	Q8C1A5	559	QIVLAK(*)VDQVLHTQTDADPAEEYAF	K6	73.05	1000	-	-	0.933333333	
Thop1	Q8C1A5	628	FK(*)QEGVLSPK(*)	K2	72.51	172.06	1	0.924050	0.911111:	0.9450539
Thop1	Q8C1A5	2	MK(*)PPAAC(+57.02)AGDVVDAASP	K2	58.86	1000	-	0.860759	1.122222	0.9914909
Thop1	Q8C1A5	667	DPK(*)QDAFLLSK(*)	K3	58.64	108.52	0.7422680	0.886075	0.811111:	0.8131517
Thop1	Q8C1A5	249	C(+57.02)K(*)EENC(+57.02)AILK(*)	K2	15.27	170.86	0.9896907	1.215189	1.288888	1.1645898
Septin11	Q8C1B7	184	VNIIPIIAK(*)ADTIAK(*)	K9	59.84	23.64	0.8585858	0.543209	1.079545	0.8271137
Septin11	Q8C1B7	175	LDSK(*)VNIIPIIAK(*)	K4	49.66	222.06	0.9191919	0.666666	1.102272	0.8960438
Septin11	Q8C1B7	175	K(*)LDSK(*)VNIIPIIAK(*)	K5	39.89	42.89	0.2222222	0.641975	1.113636:	0.659278
Septin11	Q8C1B7	162	SLK(*)SLDLVTMK(*)	K3	9.31	43.45	-	1.567901	-	
Iws1	Q8C1D8	600	ILQELPSVSQETLK(*)HSGIGR(*)	K14	49.43	1000	1.8461538	-	1.161290:	1.5037221
Trmt10a	Q8C1Z8	169	DIHIK(*)SEHYSELIK(*)	K5	44.28	45.63	-	-	0.741721854	
Rbm14	Q8C2Q3	164	GPALAIQSGDK(*)TK(*)	K11	55.46	20.41	0.8076923	0.695121	0.888888	0.7972344
Lmf2	Q8C3X8	567	YWFSK(*)PGDQSR(*)	K5	14.72	1000	-	-	1.375	
Kntc1	Q8C3Y4	2092	HQQIK(*)NFLNSC(+57.02)DAR(*)	K5	36.25	1000	-	0.526881	-	
Kntc1	Q8C3Y4	1092	DLLK(*)HYC(+57.02)NADTGR(*)	K4	34.6	1000	0.8648648	0.516129	0.731958	0.7043176
Kntc1	Q8C3Y4	1367	FIDK(*)AWQNYDK(*)	K4	28.21	93.15	-	-	1.587628866	
Kntc1	Q8C3Y4	1951	GLWK(*)NHSHEPMAVR(*)	K4	7.96	1000	0.2972972	-	0.309278:	0.3032878
Tbl3	Q8C4J7	593	LWTIK(*)SNEC(+57.02)VR(*)	K5	47.34	1000	0.8888888	1.329113	1.076086	1.0980299
Tbl3	Q8C4J7	407	MNK(*)AGQVAC(+57.02)VAQGSGH	K3	7.46	1000	2.2222222	-	-	

Heatr5b	Q8C547	841	GLAENK(*)STLGPEEVR(*)	K6	24.95	1000	-	-	0.904761905	
Heatr5b	Q8C547	42	VLVAANK(*)TDVK(*)	K7	10.37	42.89	-	-	1.154761905	
Rae1	Q8C570	212	IESPLK(*)HQHR(*)	K6	48.51	1000	0.8817204	0.906666	0.967741!	0.9187097
Rae1	Q8C570	212	R(*)IESPLK(*)HQHR(*)	K7	12.57	1000	1.4193548	1.44	1.129032:	1.3294624
Nadk2	Q8C5H8	64	VVVVAK(*)TTR(*)	K6	78.46	1000	0.7731092	0.744444	0.911111:	0.8095549
Nadk2	Q8C5H8	314	QK(*)SSGLNLC(+57.02)TGTGSK(*)	K2	65.19	171.83	0.6638655	0.666666	0.966666(	0.765733
Nadk2	Q8C5H8	407	C(+57.02)FSSK(*)VC(+57.02)VR(*)	K5	33.26	1000	1.1848739	0.766666	1.011111:	0.9875506
	Q8C5K5	16	LNC(+57.02)AEYK(*)NWVK(*)	K7	36.3	44.28	-	-	0.62745098	
Cnot2	Q8C5L3	307	SNLSTSGK(*)TTSSTDGPK(*)	K8	15.79	81.58	-	-	2.658227848	
Cwc22	Q8C5N3	2	M(+42.01)K(*)SSVAHMK(*)	K2	24.59	81.81	1.03125	1.5	0.590909(	1.0407197
Cwc22	Q8C5N3	147	MMQEQITDK(*)SSLAYQR(*)	K9	15.97	1000	-	-	1.761363636	
Grsf1	Q8C5Q4	308	TGEAYVQFEEPEMANQALLK(*)HR(*)	K20	38.3	1000	-	-	0.510638298	
Septin10	Q8C650	182	VNIIPLIAK(*)ADTISK(*)	K9	46.39	1000	-	-	0.988636364	
Septin10	Q8C650	188	ADTISK(*)SELQK(*)	K6	42.79	60.87	-	-	1.977272727	
Uba6	Q8C7R4	307	TFC(+57.02)FEPLESQIK(*)HPR(*)	K12	39.34	1000	-	-	1.358695652	
Uba6	Q8C7R4	544	IDAHLNK(*)VC(+57.02)PATESIYSDE	K7	37.29	172.71	0.79	-	-	
Uba6	Q8C7R4	537	INPQLK(*)IDAHLNK(*)	K6	35.75	81	-	-	0.793478261	
Uba6	Q8C7R4	839	ATK(*)SDLQMTVLSFEK(*)	K3	20.48	145.85	-	-	1.260869565	
Uba6	Q8C7R4	295	TPK(*)TFC(+57.02)FEPLESQIK(*)	K3	19.83	106.18	-	0.780487	1.423913(	1.1022004
Utp15	Q8C7V3	245	GGQLLVSLK(*)NHHK(*)	K9	64.61	38.26	0.8660714	0.738095	0.854368!	0.8195119
Utp15	Q8C7V3	230	YVK(*)VWDMLK(*)	K3	38.72	60.84	-	-	0.737864078	
Utp15	Q8C7V3	190	LNPDLFVTGSYDHTVK(*)IFDAR(*)	K16	29.87	1000	-	-	0.621359223	
Uba3	Q8C878	409	SPAITATLEGK(*)NR(*)	K11	91.02	1000	0.8282828	0.741176	0.789473(	0.786311
Uba3	Q8C878	147	VPNC(+57.02)NVVPHFNK(*)IQDFNI	K12	29.19	1000	-	-	0.894736842	
Ppfibp1	Q8C8U0	652	ELGIK(*)HSLHR(*)	K5	55.92	1000	0.8777777	0.736842	0.932584:	0.8490681
Dido1	Q8C9B9	1523	SSLGK(*)TELFSQEQQAPDPSQGAPN	K5	70.61	1000	0.9896907	-	1.333333:	1.161512
Ccdc186	Q8C9S4	905	LIK(*)HQHELEQR(*)	K3	17.13	1000	-	-	0.617834395	
Immt	Q8CAQ8	435	QHIELALEK(*)HK(*)	K9	64.56	39.76	1.0204081	0.784810	0.989361	0.9315267
Immt	Q8CAQ8	256	AHSNILK(*)TAMDNSEIAGEK(*)	K7	48.29	195.31	0.7551020	0.683544	-	0.7193232
Acat2	Q8CAY6	180	EAQDK(*)VAVLSQNR(*)	K5	76.2	1000	0.8571428	0.827160	0.988636:	0.8909799
Vwa8	Q8CC88	984	EVVNIVK(*)HLQK(*)	K7	15.99	48.83	-	0.813186	-	
Prpf31	Q8CCF0	438	QSVVYGGK(*)STIR(*)	K8	94.08	1000	1.0217391	0.6875	0.863636:	0.8576252
Ufl1	Q8CCJ3	289	YK(*)NTQLLFLK(*)	K2	10.64	112.87	-	-	1.115789474	
H2afy2	Q8CCK0	123	GK(*)SETILSPPPEK(*)	K2	38.01	215.31	-	0.802631	1.288888{	1.0457602

Pabpn1	Q8CCS6	209	FSGHPK(*)GFAYIEFSDK(*)	K6	50.74	116.35	0.62	-	0.826086	0.7230435
Ttc27	Q8CD92	777	AIGLAHVAMK(*)C(+57.02)AESK(*)	K10	40.91	87.95	1.0361445	1.4	1.082474	1.1728729
Vcpip1	Q8CDG3	631	LVTK(*)HFPGEFGSEILVQK(*)	K4	37.05	186.55	1.2093023	-	0.760869	0.9850859
Vcpip1	Q8CDG3	937	TMGMADGK(*)HC(+57.02)TFPHLP(	K8	8.97	21.51	1.4883720	-	-	
Ston1	Q8CDJ8	342	IHTVK(*)VEHVSYSEK(*)	K5	25.88	46.57	-	-	1.010989011	
Ston1	Q8CDJ8	695	SLGVESDAQPQK(*)HVC(+57.02)QR(	K12	23.34	1000	1.1711711	-	0.967032	1.0691021
Trmt6	Q8CE96	139	DK(*)TEFAQDK(*)	K2	6.1	98	0.3505747	-	0.813008	0.5817914
Nup88	Q8CEC0	532	ILAETPDSFEK(*)HIK(*)	K11	51.48	22.34	1	0.797619	1.258823	1.0188142
Nup88	Q8CEC0	424	YHC(+57.02)SHEAGVHSVGLTWIHK(	K19	48.31	22.45	1.2421052	-	-	
Ppwd1	Q8CEC6	127	IEEGIEFVK(*)HFR(*)	K9	38.42	1000	0.5035460	-	0.953703	0.7286249
Ppwd1	Q8CEC6	127	K(*)IEEGIEFVK(*)HFR(*)	K10	14.97	148.12	1.4680851	1.195121	1.361111	1.3414394
Naa30	Q8CES0	235	LITK(*)DLSEPYSIYTYR(*)	K4	83.16	1000	0.9468085	1.069306	0.526315	0.8474771
Naa30	Q8CES0	104	SK(*)VLSAAEAAAPDGASK(*)	K2	74.86	241.23	0.9361702	0.821782	0.651315	0.8030894
Hpf1	Q8CFE2	220	VVTK(*)TFHGAGLVVPVDK(*)	K4	34.22	86.52	1.1684210	0.675675	1.166666	1.0035878
Scyl2	Q8CFE4	493	IK(*)NAC(+57.02)LQTSSLAVR(*)	K2	27.88	1000	0.9684210	0.931818	1	0.9667464
Scyl2	Q8CFE4	609	TK(*)LEQLHVMQEQQR(*)	K2	16.69	1000	1.5263157	-	1.055555	1.2909357
Polr2b	Q8CFI7	409	GMFK(*)NLLK(*)	K4	30.47	64.16	0.8735632	-	-	
Polr2b	Q8CFI7	1143	NK(*)TQISLVR(*)	K2	10.39	1000	3.6206896	-	-	
Smc4	Q8CG47	585	VEEAK(*)SSLAMNR(*)	K5	76.28	1000	0.8686868	0.810126	0.913043	0.8639523
Smc4	Q8CG47	1265	LIGIYK(*)TYNSTK(*)	K6	56.52	67.8	0.9393939	0.594936	1.336956	0.9570957
Smc4	Q8CG47	352	EITEK(*)SNVLSNEMK(*)	K5	41.45	154.91	0.7272727	-	0.760869	0.7440711
Smc4	Q8CG47	91	LMITHIVNQNFK(*)SYAGEK(*)	K12	36.71	72.73	0.7676767	-	0.771739	0.7697079
Smc4	Q8CG47	957	EINDLK(*)TELK(*)	K6	27.92	38.03	1.0808080	-	1.260869	1.1708388
Smc4	Q8CG47	926	AQVAIK(*)TADR(*)	K6	21.21	1000	-	0.886075	0.423913	0.6549945
Smc4	Q8CG47	488	ELMGFNK(*)SVNEAR(*)	K7	9.8	1000	-	-	0.760869565	
Smc4	Q8CG47	903	AQQNK(*)LDTINK(*)	K5	7.53	35.17	-	-	1.02173913	
Smc2	Q8CG48	495	GK(*)HEALLAK(*)	K2	74.19	128.65	1.1938775	-	0.989473	1.0916756
Smc2	Q8CG48	919	ELDHSISK(*)HK(*)	K8	65.43	32.28	1.0816326	1.013157	0.831578	0.9754565
Smc2	Q8CG48	424	LK(*)HAQQELK(*)	K2	62.33	184.45	0.9081632	-	0.989473	0.9488185
Smc2	Q8CG48	4	MYVK(*)SIILEGFK(*)	K4	62.17	132.61	0.8061224	-	0.936842	0.8714823
Smc2	Q8CG48	312	VNTK(*)SQSAFDLK(*)	K4	60.76	152.83	0.8265306	0.855263	0.926315	0.8693699
Smc2	Q8CG48	706	ALEEELAGLK(*)NVAEK(*)	K10	39.88	77.53	-	-	0.810526316	
Smc2	Q8CG48	12	SIILEGFK(*)SYAQR(*)	K8	33.42	1000	0.8265306	0.565789	0.747368	0.7132295
Smc2	Q8CG48	343	ELQNSMAEDSK(*)ALAAK(*)	K11	23.24	51	3.0714285	-	-	

Smc2	Q8CG48	297	LK(*)SLEDAC(+57.02)AEAQR(*)	K2	21.9	1000	-	1.605263	-	
Smc2	Q8CG48	958	SAYDFK(*)TNNPK(*)	K6	19.66	77.53	1.8979591	-	1.4631578	1.6805585
Ppm1f	Q8CGA0	120	ALVTLLDAK(*)GLAR(*)	K9	42.62	1000	1.0526315	1.222222	0.7022900	0.9923813
Rbm28	Q8CGC6	539	GQSLGYAFAEFQK(*)HEHALR(*)	K13	76.53	1000	0.2857142	-	0.6568627	0.4712885
Rbm28	Q8CGC6	468	LK(*)NQNIFVSQTR(*)	K2	73.03	1000	0.75	0.5	0.9705882	0.7401961
Rbm28	Q8CGC6	463	FELLK(*)HQK(*)	K5	16.39	46.91	-	2.212765	1.5980392	1.9054026
Eprs	Q8CGC7	142	QNK(*)TLVHVK(*)	K3	73.97	112.31	0.9278350	0.884615	0.9787234	0.9303913
Eprs	Q8CGC7	341	AK(*)IDMSSNNGC(+57.02)MR(*)	K2	55.68	1000	0.6701030	1	0.8404255	0.8368429
Eprs	Q8CGC7	1361	FNHWELK(*)GVPVR(*)	K7	52.79	1000	0.7835051	0.871794	0.9893617	0.8815539
Eprs	Q8CGC7	1421	ASEDLK(*)THMVVSNTLEDFQK(*)	K6	49.06	135.57	1.2268041	1.269230	1.1382978	1.2114443
Eprs	Q8CGC7	578	INWGNINITK(*)IHK(*)	K10	45.45	40.63	0.9793814	0.782051	0.9042553	0.8885627
Eprs	Q8CGC7	318	NSVEK(*)NLQMWEEMK(*)	K5	38.39	120.76	0.7319587	-	-	
Eprs	Q8CGC7	139	GSAAWQEHLK(*)QNK(*)	K10	19.95	56.34	1.6288659	-	-	
Eprs	Q8CGC7	650	DSK(*)HEELMLGDPC(+57.02)LK(*)	K3	18.2	80.99	-	-	0.70212766	
Eprs	Q8CGC7	581	IHK(*)NADGK(*)	K3	17.84	41.96	0.8865979	-	-	
Eprs	Q8CGC7	991	DSSK(*)SQGSGLSSGGAGEGQGPK(*)	K4	16.95	120.79	-	-	1.478723404	
Eprs	Q8CGC7	943	AQYK(*)SLTGIEYK(*)PVSATGAEDK(*)	K4	15.85	63.64	-	-	0.914893617	
Tcerg1	Q8CGF7	1018	YITAK(*)ADFR(*)	K5	79.41	1000	0.7849462	0.810126	-	0.7975364
Tcerg1	Q8CGF7	755	ATFSEFAAK(*)HAK(*)	K9	71.29	44.44	1.1935483	0.860759	1.1505376	1.0682818
Tcerg1	Q8CGF7	798	IK(*)SDFFELLSNHHLDSQSR(*)	K2	16.39	1000	-	-	1.23655914	
Tcerg1	Q8CGF7	1018	DK(*)YITAK(*)ADFR(*)	K7	12.78	107.29	-	-	1.247311828	
Tcerg1	Q8CGF7	822	VK(*)DK(*)VESDPR(*)	K4	3.42	45.01	-	3.215189	-	
Lonp1	Q8CGK3	518	ILC(+57.02)FHGPPGVGK(*)TSIAR(*)	K12	46.82	1000	-	0.524390	0.9032258	0.713808
Lonp1	Q8CGK3	570	TK(*)TENPLVLIDEVDK(*)	K2	22.47	152.87	-	0.939024	0.6236559	0.7813402
Hist1h2bp	Q8CGP2	6	PEPVK(*)SVPAPK(*)	K5	76.02	101.67	-	-	-	
Hist1h2bp	Q8CGP2	13	PEPVK(*)SVPAPK(*)K(*)GSK(*)	K12	19.84	38.16	-	-	-	
Hist1h2bp	Q8CGP2	12	PEPVK(*)SVPAPK(*)K(*)GSK(*)	K11	19.84	49.79	-	-	-	
Hist1h2bp	Q8CGP2	6	PEPVK(*)SVPAPK(*)K(*)GSK(*)	K5	19.84	112.07	-	-	-	
Hist1h2bp	Q8CGP2	13	SVPAPK(*)K(*)GSK(*)K(*)	K7	7.58	28.7	-	-	-	
Hist1h2bp	Q8CGP2	12	SVPAPK(*)K(*)GSK(*)K(*)	K6	7.58	38.32	-	-	-	
Cherp	Q8CGZ0	910	NK(*)SYSFIAR(*)	K2	47.72	1000	1	1.027397	0.8089887	0.945462
Cherp	Q8CGZ0	888	DK(*)WDQYK(*)	K2	43.14	95.62	1.7931034	1.109589	1.3033707	1.4020211
Sugp2	Q8CH09	1020	EGHGLGSLGK(*)GIR(*)	K10	34.37	1000	-	-	-	
Ccar1	Q8CH18	785	IYK(*)SLLSLPEK(*)	K3	40.83	171.6	0.9529411	1.027027	0.9176470	0.9658718

Ccar1	Q8CH18	963	LLNK(*)VVLR(*)	K4	30.93	1000	0.9176470	1.189189	1.294117(	1.1336513
Ccar1	Q8CH18	785	IYK(*)SLLSLPEK(*)EDK(*)	K3	26.9	89.48	1.4117647	0.797297	0.952941:	1.0540011
Ccar1	Q8CH18	656	GLK(*)SQLIAR(*)	K3	6.42	1000	-	-	0.8	
Sltm	Q8CH25	401	AADLK(*)NLFGK(*)	K5	34.19	89.05	0.5957446	1.239436	0.822222:	0.8858012
Gcc2	Q8CHG3	1265	IQLAEMTSEK(*)HK(*)	K10	37.45	22.85	0.78125	-	-	
Gcc2	Q8CHG3	134	EIESC(+57.02)K(*)NELMAVHSEHSK(	K6	15.98	54.4	-	-	1.290322581	
Septin8	Q8CHH9	193	ADTISK(*)SELHK(*)	K6	67.11	65.44	1.1058823	0.784810	0.987341	0.9593448
Kat5	Q8CHK4	104	EVPASAQASGK(*)TLPIPVQITLR(*)	K11	24.67	1000	-	-	-	
Dym	Q8CHY3	216	LVK(*)TLLYNFIR(*)	K3	22.02	1000	-	-	0.540229885	
Gatad2a	Q8CHY6	200	GTQNIPAGK(*)TSLQTSSTR(*)	K9	45.51	1000	0.5543478	0.635135	0.818181	0.6692216
Gatad2a	Q8CHY6	573	VVGTGK(*)GTASNWK(*)	K6	36.49	59.03	0.7717391	0.797297	-	0.7845182
Gatad2a	Q8CHY6	601	VSPSLAVHK(*)TSSAVDR(*)	K9	16.17	1000	-	-	1.171717172	
Gnl3	Q8CI11	232	IC(+57.02)C(+57.02)GK(*)EALWK(*	K5	73.69	83.98	-	0.987179	0.915662(	0.9514211
Gnl3	Q8CI11	362	DSLHFFTK(*)LAQR(*)	K8	63.71	1000	1.5294117	1.294871	1.891566:	1.5719499
Gnl3	Q8CI11	177	LILVLNK(*)SDLVPK(*)	K7	45.28	90.16	0.8627450	0.923076	0.951807:	0.9125431
Gnl3	Q8CI11	209	ASTNLK(*)NR(*)	K6	13.14	1000	-	0.769230	1.096385!	0.9328082
Cwf19l1	Q8CI33	277	QAAGGK(*)HIPAPQEESAC(+57.02)C	K6	40.4	79.15	-	-	0.70212766	
Pdlim5	Q8CI51	432	GPFLVALGK(*)SWHPEEFNC(+57.02	K9	67.21	26.02	1.0898876	-	1	1.0449438
Pdlim5	Q8CI51	432	LVALGK(*)SWHPEEFNC(+57.02)AH(	K6	23.16	21.46	-	-	0.427083333	
Vps50	Q8CI71	396	VK(*)TYLLGTDLSIFK(*)	K2	34.92	129.85	-	-	0.679245283	
Pygb	Q8CI94	725	VEDVEALDQK(*)GYNAR(*)	K10	42.88	1000	-	0.807692	1.046511(	0.927102
Pygb	Q8CI94	290	VLYPNDNFFEGK(*)ELR(*)	K12	4.03	1000	-	-	3.337209302	
Fermt2	Q8CIB5	74	TWLLK(*)THWTLDK(*)	K5	69.96	127.53	0.9021739	-	0.955555!	0.9288647
Fermt2	Q8CIB5	408	DTSISC(+57.02)YK(*)SR(*)	K8	66.08	1000	1.0978260	1.24	1.4	1.245942
Fermt2	Q8CIB5	247	AK(*)TNQGWLDSSR(*)	K2	55.97	1000	0.8369565	0.88	1	0.9056522
Fermt2	Q8CIB5	112	VK(*)VNFSDR(*)	K2	49.79	1000	1.0543478	-	0.855555!	0.9549517
Fermt2	Q8CIB5	393	GYK(*)QYWC(+57.02)TFK(*)	K3	39.87	112.7	1.8913043	1.413333	1.266666(	1.5237681
Fermt2	Q8CIB5	605	MDASTGDAIK(*)TWR(*)	K10	37.11	1000	1.1739130	0.786666	1.5	1.1535266
Esco2	Q8CIB9	369	GGK(*)DQLVIDAGQK(*)	K3	40.46	132.72	-	-	-	
Copa	Q8CIE6	1119	NFK(*)TAATFAR(*)	K3	82.49	1000	0.9782608	0.910256	1.107526	0.9986814
Copa	Q8CIE6	438	MHSLLIK(*)NLK(*)	K7	81.91	44.84	1.0217391	0.923076	0.892473:	0.9457631
Copa	Q8CIE6	441	NLK(*)NEITK(*)	K3	81.32	100.21	0.9130434	1.153846	1	1.0222965
Copa	Q8CIE6	750	ILK(*)NC(+57.02)GQK(*)	K3	76.03	107.29	0.7173913	0.692307	0.784946:	0.7315484
Copa	Q8CIE6	13	VK(*)GLSFHPK(*)	K2	63.18	140.21	-	-	1.258064516	

Copa	Q8CIE6	198	GITGVDLFGTTDAVVK(*)HVLEGHDR(	K16	62.84	1000	-	-	0.76344086	
Copa	Q8CIE6	531	VK(*)SGAWDESGVFIYTTSNHIK(*)	K2	52.78	277.25	0.8586956	0.666666	0.935483	0.8202821
Copa	Q8CIE6	574	VK(*)GNNVYC(+57.02)LDR(*)	K2	49.04	1000	-	1.576923	1.720430	1.6486766
Copa	Q8CIE6	238	MNESK(*)AWEVDTC(+57.02)R(*)	K5	46.79	1000	1.2173913	1.346153	1.344086	1.3025437
Copa	Q8CIE6	993	DAGLK(*)NGVPAVGLK(*)	K5	45.96	148.12	0.7934782	0.589743	0.989247	0.7908231
Copa	Q8CIE6	76	IK(*)VWNYK(*)	K2	35.83	127.18	0.6304347	1.243589	0.827956	0.9006605
Copa	Q8CIE6	350	QLDFNSSK(*)DVAVMQLR(*)	K8	14.83	1000	-	-	1.193548387	
Prmt5	Q8CIG8	248	GFPVLSK(*)VQQR(*)	K7	26.65	1000	0.7469879	0.972222	-	0.8596051
Prmt5	Q8CIG8	516	LHNFHQLSAPK(*)PC(+57.02)FTFSH	K11	20.57	1000	-	-	1.108433735	
Cdc123	Q8CII2	24	SLTIK(*)SVILPLPQNVK(*)	K5	10.53	128.61	-	-	1.238095238	
Ints4	Q8CIM8	158	LVDVAC(+57.02)K(*)HLTDTSHGVR(	K7	49.86	1000	1.1047619	0.598039	0.808	0.8369337
Pak2	Q8CIN4	370	DIK(*)SDNVLLGMEGSVK(*)	K3	67.09	206.53	-	-	0.880434783	
Pak2	Q8CIN4	113	LLQTSNITK(*)LEQK(*)	K9	32.23	41.04	0.2934782	0.211267	0.380434	0.2950602
Trip10	Q8CJ53	166	LDQDINATK(*)ADVEK(*)	K9	17.67	29.96	-	-	1.434782609	
Ahctf1	Q8CJF7	2221	TAEIAGK(*)TLGR(*)	K7	73.77	1000	0.7692307	0.786666	0.705882	0.7539266
Ahctf1	Q8CJF7	2183	NTGK(*)GSSWSPPPVEIK(*)	K4	14.86	77.78	-	1.346666	-	
Ago2	Q8CJG0	727	VGK(*)SGNIPAGTTVDTK(*)	K3	34.6	107.9	0.6373626	0.740259	0.935483	0.7710354
Hmgcs1	Q8JZK9	409	ITASLC(+57.02)DLK(*)SR(*)	K9	77.6	1000	0.4347826	0.506329	-	0.4705559
Hmgcs1	Q8JZK9	273	LVQK(*)SLAR(*)	K4	58.29	1000	-	0.582278	0.741935	0.662107
Hmgcs1	Q8JZK9	206	GTHMQHAYDFYK(*)PDMLSEYPVVD(	K12	45.59	90.97	0.6086956	-	0.763440	0.6860683
Hmgcs1	Q8JZK9	291	DK(*)NSIYSGLEAFGDVK(*)	K2	41.67	208.29	-	0.835443	0.817204	0.8263237
Acad9	Q8JZN5	460	ELK(*)SGNVTTVMETIGR(*)	K3	43.7	1000	0.2916666	-	0.485981	0.388824
Acad9	Q8JZN5	232	AK(*)TEVVDSDGSK(*)	K2	10.99	156.93	1.5729166	-	0.990654	1.2817854
Afg3l2	Q8JZQ2	542	HLSDAINEK(*)HFEQAIER(*)	K9	16.68	1000	0.816	-	-	
Afg3l2	Q8JZQ2	617	EQYLYTK(*)EQLLDR(*)	K7	6.5	1000	-	-	1.566666667	
Eif3b	Q8JZQ9	193	LK(*)NVIHK(*)	K2	85	119.54	0.9368421	0.804878	0.9	0.8805734
Eif3b	Q8JZQ9	584	ISVSFYHVK(*)SNGK(*)	K9	56.06	55.36	1.0947368	0.987804	1.177777	1.0867732
Eif3b	Q8JZQ9	334	WSPK(*)GTYLATFHQR(*)	K4	51.47	1000	1.1684210	0.841463	0.922222	0.9773689
Eif3b	Q8JZQ9	518	NGDYLC(+57.02)VK(*)VDR(*)	K8	49.19	1000	0.6421052	0.792682	0.655555	0.6967812
Eif3b	Q8JZQ9	510	LHWQK(*)NGDYLC(+57.02)VK(*)	K5	45.93	101.72	1.4526315	-	1.344444	1.398538
Eif3b	Q8JZQ9	205	FGK(*)IINDYYPEEDGK(*)	K3	44.97	186.68	0.8	2.463414	0.9	1.3878049
Eif3b	Q8JZQ9	718	YSK(*)IFEQK(*)	K3	39.79	87.95	0.9052631	1.109756	1.066666	1.0272286
Eif3b	Q8JZQ9	237	GYIFLEYASPAHAVDAVK(*)NADGYK(	K18	35.61	23.15	-	-	0.977777778	
Eif3b	Q8JZQ9	217	IINDYYPEEDGK(*)TK(*)	K12	25.28	22.85	2.0842105	1.621951	1.055555	1.5872391

Acsl5	Q8JZR0	404	LVFSK(*)IQGSLGGK(*)	K5	54.39	118.18	0.7	0.75	-	0.725
Slc25a1	Q8JZU2	268	ILK(*)NEGPK(*)	K3	50.82	105.53	0.9473684	-	-	
Slc25a1	Q8JZU2	97	GLSSLLYGSIPK(*)AAVR(*)	K12	16.64	1000	1.0315789	2.356164	1.074468(	1.4874038
Rbm17	Q8JZX4	256	EGQGLGK(*)HEQGLSTALSVEK(*)	K7	74.74	44.86	1.4823529	0.648648	1.0125	1.0478339
Rbm17	Q8JZX4	276	GGK(*)IIVGDATEK(*)	K3	27.04	120.76	-	0.689189	-	
Aldh1l2	Q8K009	681	QIMK(*)SC(+57.02)AVSNLK(*)	K4	56.96	80.5	-	0.652777	-	
Aldh1l2	Q8K009	866	DINK(*)AMYVSDK(*)	K4	46.61	120.29	1.8709677	2.347222	1.511627!	1.9099393
Bclaf1	Q8K019	419	SVLADQGK(*)SFATSSHR(*)	K8	85.31	1000	1.2891566	0.92	0.954022!	1.0543932
Cyp51a1	Q8K0C4	261	EIK(*)NIFYK(*)	K3	87.48	84.49	0.7865168	1.012658	0.902173!	0.9004497
Cyp51a1	Q8K0C4	160	IIK(*)SGLNIAHFK(*)	K3	57.96	225.26	0.8202247	0.683544	0.956521	0.8200969
Gmds	Q8K0C9	294	SFMHIGK(*)TIVWEGK(*)	K7	61.5	104.8	-	-	0.595744681	
Gmds	Q8K0C9	287	EFVEK(*)SFMHIGK(*)	K5	51.88	56.54	0.71	0.641975	0.872340	0.7414386
Gmds	Q8K0C9	301	TIVWEGK(*)NENEVGR(*)	K7	39.7	1000	1.43	0.790123	0.680851(	0.9669915
Gfm1	Q8K0D5	710	SC(+57.02)TEGK(*)GEYTMEYC(+57.(	K6	44.96	1000	-	-	0.79245283	
Eipr1	Q8K0G5	86	GVLATC(+57.02)YNK(*)TTDSR(*)	K9	37.26	1000	0.5465116	-	-	
Arhgap18	Q8K0Q5	374	IK(*)NLC(+57.02)QELEAK(*)	K2	6.73	123.34	-	0.454545	-	
Plekho2	Q8K124	134	VDK(*)TC(+57.02)ALEHVTR(*)	K3	40.85	1000	-	-	0.35483871	
Pdxk	Q8K183	58	GQVLK(*)SQELHELYEGLK(*)	K5	51.38	93.08	-	0.526315	0.975903(	0.7511097
Pdxk	Q8K183	70	SQELHELYEGLK(*)VNDVNK(*)	K12	42.07	95.35	-	-	0.855421687	
Pdxk	Q8K183	247	LLAWTHK(*)HPDNLK(*)	K7	13.62	85.52	-	1.394736	1.518072	1.4564046
Cc2d1a	Q8K1A6	284	IAK(*)SFDPVLEALSR(*)	K3	15.5	1000	-	-	1.122222222	
Trnt1	Q8K1J6	328	NLGLFIVK(*)NR(*)	K8	74.32	1000	0.7878787	0.782051	0.872340	0.8140902
Trnt1	Q8K1J6	308	VQDDVTK(*)LDLR(*)	K7	66.34	1000	0.8585858	1.217948	-	1.0382673
Trnt1	Q8K1J6	320	EEK(*)NLGLFIVK(*)	K3	62.01	123.53	0.8282828	0.794871	0.968085	0.8637466
Trnt1	Q8K1J6	402	VGISSGK(*)EIGALLQQLR(*)	K7	53.62	1000	1.1515151	-	0.936170	1.0438427
Trnt1	Q8K1J6	283	VSK(*)NVEGFSPK(*)	K3	39.73	83.15	-	-	0.755319149	
Dnm1l	Q8K1M6	658	LIK(*)SYFLIVR(*)	K3	12.46	1000	-	-	1.325842697	
Phldb2	Q8K1N2	786	EK(*)NNLIMMLQR(*)	K2	9.27	1000	1.0104166	-	-	
Pnpt1	Q8K1R3	306	LYAVFTDYEHDK(*)VSR(*)	K12	12.44	1000	-	-	1.08490566	
Pnpt1	Q8K1R3	288	YTK(*)IIAMEK(*)	K3	6.85	94.27	3.0891089	-	5.509433!	4.2992714
Nat10	Q8K224	802	NVAK(*)SALPALGR(*)	K4	46.76	1000	1.0202020	-	0.954545	0.9873737
Abcf3	Q8K268	531	IC(+57.02)VVGENGAGK(*)STMLK(*)	K11	3.86	29.96	-	-	0.054945055	
Colgalt1	Q8K297	533	HPMSEYK(*)SHFSPR(*)	K7	62.85	1000	0.8089887	-	-	
Colgalt1	Q8K297	600	EAK(*)NSDVLQSPLDSTAR(*)	K3	31.24	1000	0.8651685	1.607594	-	1.2363817

Anln	Q8K298	914	LLTSITSK(*)SSLHSSVMASPGGLGAVI	K8	47.74	1000	-	-	0.680412371	
Anln	Q8K298	1081	DTLC(+57.02)VTK(*)NWLSADTK(*)	K7	39.37	105.21	-	-	0.93814433	
Anln	Q8K298	142	GLNSGSEASATSSVK(*)TR(*)	K15	6.02	1000	-	-	0.75257732	
Gulp1	Q8K2A1	184	IQDLETENMELK(*)NK(*)	K12	36.07	36.05	-	-	0.977011494	
P3h4	Q8K2B0	180	NPK(*)HELTAK(*)	K3	59.73	43.32	-	-	0.703389831	
Sdha	Q8K2B3	550	HLK(*)TFDR(*)	K3	79.34	1000	-	-	0.907216495	
Sdha	Q8K2B3	547	ISQLYGDLK(*)HLK(*)	K9	71.14	62.26	0.82	0.690476	0.855670	0.7887154
Sdha	Q8K2B3	250	AK(*)NTVIATGGYGR(*)	K2	58.9	1000	0.76	0.654761	0.835051	0.7499378
Sdha	Q8K2B3	517	LNMQK(*)SMQNHAAVFR(*)	K5	56.75	1000	0.85	0.857142	0.958762	0.8886352
Edc3	Q8K2D3	296	LLSVAEK(*)HGLTLER(*)	K7	54.04	1000	0.8631578	0.542168	1.514705	0.9733442
Lsm14a	Q8K2F8	17	ISLISK(*)AEIR(*)	K6	41.52	1000	-	-	0.844444444	
Otud6b	Q8K2H2	34	IQGMK(*)NAVPK(*)	K5	7.9	33.54	-	-	3.181818182	
Agfg1	Q8K2K6	318	VSTNK(*)AGLQTADK(*)	K5	14.35	101.72	0.5222222	0.5	-	0.5111111
Brox	Q8K2Q7	155	IAAGIFK(*)HLK(*)	K7	57.28	49.37	-	-	0.989361702	
Brox	Q8K2Q7	317	LGSLVK(*)NTLDK(*)	K6	27.73	29.85	-	-	1.893617021	
Nmral1	Q8K2T1	143	LAAGHFDGK(*)GEVEEYFR(*)	K9	35.73	1000	-	-	0.417721519	
Ppp4r1	Q8K2V1	724	IGVLK(*)HLHDFLK(*)	K5	48.36	41.87	-	-	0.820224719	
Ipo11	Q8K2V6	156	ALLTFYHVTK(*)TLASK(*)	K10	50.06	53.48	0.7555555	1.162162	1.046511	0.9880764
Ipo11	Q8K2V6	329	MIVK(*)NYAYK(*)PSK(*)	K4	26.23	115.97	1.6444444	-	1.127906	1.3861757
Prpf39	Q8K2Z2	484	NAK(*)SNNESSFYAIK(*)	K3	8.34	86.4	-	-	1.590909091	
Ncapd2	Q8K2Z4	35	EVLPVK(*)HLSSQLR(*)	K6	69.16	1000	1.0919540	1.028169	0.977528	1.0325504
Ncapd2	Q8K2Z4	416	LADK(*)SVLVC(+57.02)K(*)	K4	44.58	113.99	-	-	1.415730337	
Ncapd2	Q8K2Z4	775	GK(*)PEIVGSNLDALVR(*)	K2	42.28	1000	0.9655172	2.661971	1.382022	1.6698372
Matr3	Q8K310	3	S(+42.01)K(*)SFQQSSLGR(*)	K2	98.9	1000	1.0869565	0.670886	0.944444	0.9007623
Matr3	Q8K310	524	IK(*)NYILMR(*)	K2	88.54	1000	1.0326086	0.873417	0.955555	0.9538607
Matr3	Q8K310	532	MK(*)SQAFIEMETR(*)	K2	78.23	1000	0.9456521	0.620253	0.933333	0.8330796
Matr3	Q8K310	816	LC(+57.02)SLFYTNEEVAK(*)NTHC(+5	K13	42.89	29.08	-	-	1.188888889	
Champ1	Q8K327	721	GAVLHHLVNK(*)HNVHSPYK(*)	K10	23.06	25	-	-	0.662921348	
Champ1	Q8K327	702	YYC(+57.02)K(*)IC(+57.02)C(+57.0	K4	22.8	1000	-	1.689655	-	
Kin	Q8K339	3	GK(*)SDFLSPK(*)	K2	48.93	144.88	1.8510638	0.869565	-	1.3603145
Ddx18	Q8K363	567	NYFLHK(*)SAQEAYK(*)	K6	53.85	93.15	0.9777777	0.901234	1.011363	0.9634587
Ddx18	Q8K363	561	LIEK(*)NYFLHK(*)	K4	53.05	132.53	0.9222222	0.716049	1	0.8794239
Ddx18	Q8K363	646	IFK(*)HISK(*)	K3	42.67	77.13	1.4111111	0.740740	-	1.0759259
Ddx18	Q8K363	574	SAQEAYK(*)SYIR(*)	K7	41.49	1000	0.8444444	1.432098	0.772727	1.0164235

Ddx18	Q8K363	535	QSK(*)VPLNQFDFSWSK(*)	K3	7.84	38.88	0.9444444	-	-	
Hscb	Q8K3A0	118	HFSDK(*)HSTLVNDAYK(*)	K5	37.8	59.58	2.8695652	-	-	
Lzic	Q8K3C3	13	LK(*)QNLEEQLDR(*)	K2	35.38	1000	-	-	0.62244898	
Swi5	Q8K3D3	46	VIELEK(*)HISLLHEYNDIK(*)	K6	57.35	126.66	0.9375	-	1.183098!	1.0602993
Swi5	Q8K3D3	23	LK(*)EK(*)HDMLDK(*)	K4	9.07	52.04	-	-	1.549295775	
Appl1	Q8K3H0	289	NK(*)TGLVSSTWDR(*)	K2	10.21	1000	-	-	6.423076923	
Casc3	Q8K3W3	341	SAETLK(*)HEASYR(*)	K6	38.64	1000	-	-	-	
Pitrm1	Q8K411	1012	YLGIGK(*)STHGLAILGPENSK(*)	K6	43.95	121.81	0.8762886	-	0.670454!	0.7733716
Pitrm1	Q8K411	494	VEQYFK(*)NNQHK(*)	K6	25.95	38.33	-	1.089743	0.772727:	0.9312354
Pitrm1	Q8K411	488	FLQEK(*)VEQYFK(*)	K5	24.17	132.53	0.7835051	0.641025	-	0.7122654
Pitrm1	Q8K411	550	GLELQTQQSK(*)HQDASC(+57.02)LP	K10	22	20.33	0.4536082	0.653846	2.204545	1.104
Mta1	Q8K4B0	260	HAMDTLHK(*)NIYDISK(*)	K8	27.83	53.43	0.8989898	0.860215	1.025316	0.9281738
Svil	Q8K4L3	982	EVSTAK(*)SSLQENLDLK(*)	K6	19.97	81.97	1.0531914	-	1.542168	1.2976801
Colec12	Q8K4Q8	430	LVDSK(*)HGQLIK(*)	K5	35.8	72.73	-	-	0.528301887	
Agpat4	Q8K4X7	201	GLPSLK(*)HHLLPR(*)	K6	21.71	1000	1.5955056	2.511111	0.596638	1.5677518
Sf3a1	Q8K4Z5	55	NIVDK(*)TASFVAR(*)	K5	81.11	1000	-	0.974683	0.978021!	0.9763528
Sf3a1	Q8K4Z5	531	AK(*)GLVPEDDTK(*)	K2	29.74	62.82	-	2.468354	-	
Sf3a1	Q8K4Z5	715	VQVPNMQDK(*)TEWK(*)	K9	18.08	20.17	-	1.645569	-	
Ahsa2	Q8N9S3	156	ALK(*)TEFTTGMILPTK(*)	K3	33.53	186.68	-	0.873015	1.216867	1.0449417
Acat1	Q8QZT1	242	GK(*)PDVVVK(*)EDEEYK(*)R(*)	K2	62.99	71.75	-	0.734177	-	
Acat1	Q8QZT1	260	VDFSK(*)VPK(*)	K5	48.95	56.34	1.0219780	0.670886	0.816091!	0.8363187
Acat1	Q8QZT1	265	LK(*)TVFQK(*)	K2	48.61	91.35	1.2087912	0.810126	0.931034	0.9833174
Acat1	Q8QZT1	121	QATLGAGLPISTPC(+57.02)TTVNK(*	K19	26.13	71.72	2.0219780	-	0.586206	1.3040925
Acat1	Q8QZT1	242	GK(*)PDVVVK(*)	K2	10.03	69.64	-	0.632911	1.206896!	0.919904
IntS13	Q8QZV7	167	IIC(+57.02)ITNAK(*)SDSHVR(*)	K8	5.65	1000	5.6071428	-	-	
Eif3l	Q8QZY1	494	IQLLVFK(*)HK(*)	K7	86.49	39.76	0.8	0.738095	0.938775!	0.8256236
Eif3l	Q8QZY1	237	SVLNVLHSLVDK(*)SNINR(*)	K12	60.63	1000	0.9684210	-	0.785714:	0.8770677
Eif3l	Q8QZY1	61	HK(*)TVSDLIDQK(*)	K2	59.49	155.51	0.7473684	-	0.816326!	0.7818475
Eif3l	Q8QZY1	237	SLVDK(*)SNINR(*)	K5	53.3	1000	1.2947368	1.035714	0.806122	1.0455245
Eif3l	Q8QZY1	347	TK(*)SMFQR(*)	K2	52.33	1000	0.8526315	0.761904	1.071428!	0.8953216
Eif3l	Q8QZY1	143	HIYAK(*)VSGGPSLEQR(*)	K5	47.48	1000	0.7684210	1.095238	0.520408:	0.7946891
Eif3l	Q8QZY1	534	DMIHIADTK(*)VAR(*)	K9	38.63	1000	1.2	0.833333	0.989795!	1.0077098
Eif3l	Q8QZY1	356	TTYK(*)YEMINK(*)	K4	35.85	113.99	0.3473684	0.571428	-	0.4593985
Rab38	Q8QZZ8	105	WK(*)NDLDSK(*)	K2	10.68	104.48	-	-	-	

Aimp2	Q8R010	159	VLSTVHTHSSVK(*)NVPENLVK(*)	K12	85.95	85.91	1.1237113	0.769230	0.860215(	0.9177191
Aimp2	Q8R010	190	TLIWK(*)NVPK(*)	K5	47.48	67.69	0.7938144	0.833333	1.139784!	0.9223109
Aimp2	Q8R010	33	LPNVHSK(*)TTSPATDAGHVQETSEPS	K7	38.67	1000	-	-	0.387096774	
Blmh	Q8R016	391	DNQEGTFVK(*)WR(*)	K9	48.87	1000	-	1.194805	-	
Gspt1	Q8R050	350	TAGVK(*)HLIVLINK(*)	K5	70.35	216.51	0.9890109	0.986111	0.977528(	0.9842167
Gspt1	Q8R050	345	EHAM(+15.99)LAK(*)TAGVK(*)	K7	45.06	22.36	-	-	0.93258427	
Gspt1	Q8R050	621	DEGK(*)TIAIGK(*)	K4	24.34	56.47	-	1	-	
Gspt1	Q8R050	585	FVK(*)QDQVC(+57.02)IAR(*)	K3	17.6	1000	0.8021978	0.597222	0.730337(	0.709919
Hnrnpl	Q8R081	415	SK(*)PGAAMVEMADGYAVDR(*)	K2	88.66	1000	0.875	0.871794	0.966292:	0.9043623
Hnrnpl	Q8R081	490	FSTPEQAAK(*)NR(*)	K9	78.15	1000	0.8125	0.871794	0.977528(	0.8872743
Hnrnpl	Q8R081	62	LK(*)TENAGDQHGGGGGGGSGAAG(	K2	57.81	276.22	0.9791666	0.910256	0.831460(	0.9069613
Ilkap	Q8R0F6	250	YNEESQK(*)HAALSLSK(*)	K7	37.54	78.17	-	-	1.138297872	
Nup133	Q8R0G9	745	LNK(*)SSMYSQEEVLGK(*)	K3	77.35	279.2	0.7878787	1.1875	0.882978:	0.9527858
Nup133	Q8R0G9	134	IAVSPVTK(*)LSVC(+57.02)K(*)	K8	43.36	53.48	-	1.3125	0.553191	0.9328457
Tada3	Q8R0L9	109	LEGK(*)TGHGPGPGPGR(*)PK(*)	K4	78.43	155.24	-	0.429577	-	
Tada3	Q8R0L9	109	LEGK(*)TGHGPGPGPGR(*)	K4	42.33	1000	-	0.471830	-	
Sgpl1	Q8R0X7	431	FLK(*)SELENIK(*)	K3	60.77	124.87	0.4301075	-	0.988764(	0.7094358
Sgpl1	Q8R0X7	101	DLVK(*)NMPFLK(*)	K4	28.69	95.35	1.1827956	0.617283	0.764044!	0.8547082
Apeh	Q8R146	131	LK(*)SFNLSALEK(*)	K2	27.55	130.84	0.8804347	1.15	1.119565:	1.05
Bud13	Q8R149	47	PGGAGGK(*)GMR(*)	K7	61.61	1000	0.546875	0.692307	-	0.6195913
Bud13	Q8R149	446	TAHMYSGAK(*)TGLVTDVQR(*)	K9	36.97	1000	-	-	0.859375	
Bphl	Q8R164	271	LHLMPEGK(*)HNLHLR(*)	K8	26.46	1000	1.1818181	-	0.623762:	0.9027903
Dock7	Q8R1A4	1687	LTWLQNMAGK(*)HSER(*)	K10	26.4	1000	1.0537634	2.605263	1.208791:	1.6226059
Dock7	Q8R1A4	2070	NK(*)SLIGPDQK(*)	K2	11.78	102.9	-	-	0.813186813	
Dock7	Q8R1A4	1668	IAK(*)GYQTSPDLR(*)	K3	8.5	1000	-	-	2.21978022	
Dock7	Q8R1A4	233	HK(*)ELFALHPSPDEEEPIER(*)	K2	6.81	1000	-	-	1.615384615	
Eif3c	Q8R1B4	875	VFDHK(*)QGTYGGYFR(*)	K5	61.4	1000	0.9090909	1.2	0.969072:	1.0260544
Eif3c	Q8R1B4	748	MGDWK(*)TC(+57.02)HSFIINEK(*)	K5	58.6	79.67	0.8383838	0.6625	1.072164!	0.8576829
Eif3c	Q8R1B4	511	ILHTYYK(*)FDYK(*)	K7	57.13	38.26	0.8989898	1.25	1.020618!	1.0565362
Eif3c	Q8R1B4	551	LC(+57.02)K(*)YIYAK(*)	K3	44.18	82.34	0.8686868	0.425	1.051546:	0.7817444
Eif3c	Q8R1B4	318	MFAK(*)GTEITHAVVIK(*)	K4	41.76	63.27	0.3535353	-	0.804123:	0.5788295
Eif3c	Q8R1B4	625	QGLTK(*)DAHNALLDIQSSGR(*)	K5	36.64	1000	-	-	1.020618557	
Eif3c	Q8R1B4	710	MISK(*)QFHHQLR(*)	K4	34.97	1000	0.8080808	0.675	0.865979:	0.7830201
Niban2	Q8R1F1	513	LAPTC(+57.02)K(*)SELPR(*)	K6	73.44	1000	1.1290322	1.0625	1.142857:	1.1114631

Niban2	Q8R1F1	203	HC(+57.02)NNGIPENSK(*)VEGPAFT	K11	34.71	1000	-	-	1.846153846	
Niban2	Q8R1F1	330	AFILPK(*)AEVC(+57.02)VR(*)	K6	24.56	1000	-	22.925	4.714285	13.819643
Rpp40	Q8R1F9	19	HLLVC(+57.02)EK(*)SNFGHDK(*)	K7	44.73	100.91	0.6415094	-	1.161290	0.9013999
Pdlim2	Q8R1G6	333	GHFWVGNELYC(+57.02)EK(*)HAR(	K13	77.62	1000	1.1917808	1.027397	1.023529	1.0809025
Dync1li1	Q8R1Q8	80	NVLLLGEDGAGK(*)TSLIR(*)	K12	51.79	1000	0.9659090	0.895522	0.967391	0.9429409
Dync1li1	Q8R1Q8	68	LPTGK(*)NVLLLGEDGAGK(*)	K5	33.53	68.35	1.4204545	1.283582	0.75	1.1513455
Tmed4	Q8R1V4	218	HLK(*)SFFEAK(*)	K3	85.07	134.33	1.1466666	0.936708	0.882352	0.9885762
Ctbs	Q8R242	95	GDISLK(*)NIIDPTFR(*)	K6	10.07	1000	-	-	1.119402985	
Utp4	Q8R2N2	321	VEVK(*)NYDAALR(*)	K4	47.73	1000	0.5963302	1.058823	0.727272	0.7941422
Seh1l	Q8R2U0	12	SIAADHK(*)DLIHDVSFDFHGR(*)	K7	57	1000	0.9494949	-	0.934065	0.9417804
Seh1l	Q8R2U0	54	SESGDWHC(+57.02)TASWK(*)THSG	K13	40.66	1000	0.7373737	-	-	
Ubqln1	Q8R317	61	FK(*)SHIDQLVLIFAGK(*)	K2	46.55	229.43	-	-	1.180851064	
Cdc16	Q8R349	556	TLK(*)NIISPPWDFR(*)	K3	42.59	1000	0.5876288	-	0.795698	0.6916639
Commd5	Q8R395	62	LVVGALHGK(*)DC(+57.02)R(*)	K9	25.03	1000	-	1.394366	-	
Rbm19	Q8R3C6	727	IK(*)NLNFSTTEETLK(*)	K2	28.25	150.01	0.79	1.160493	0.542553	0.8310157
Rbm19	Q8R3C6	744	GVFSK(*)VGAIK(*)	K5	10.8	52.68	-	0.530864	-	
Rbm19	Q8R3C6	533	YNATK(*)SQVFDHETR(*)	K5	9.79	1000	0.84	-	-	
Ppp1r8	Q8R3G1	78	VHAALVYHK(*)HLK(*)	K9	49.33	56.34	1.2584269	1.680555	1.279569	1.4061841
Mterf3	Q8R3J4	181	LDFEK(*)HIK(*)	K5	50.85	29.32	-	-	-	
Thoc1	Q8R3N6	133	SNTFYSAGK(*)NYLLR(*)	K9	38.33	1000	-	-	0.556962025	
Thoc1	Q8R3N6	124	NVATWK(*)SNTFYSAGK(*)	K6	23.87	57.7	2.1204819	-	2.620253	2.3703675
Thoc1	Q8R3N6	258	ISWK(*)TFLK(*)	K4	21.48	52.86	1.2771084	-	0.810126	1.0436175
Ccdc58	Q8R3Q6	72	VIK(*)NC(+57.02)IAQTSAVVK(*)	K3	55.9	254.74	0.8080808	0.6625	0.915789	0.7954568
Exoc1	Q8R3S6	5	T(+42.01)AIK(*)HALQR(*)	K4	54.35	1000	1.0504201	0.805194	0.913978	0.9231978
Cd109	Q8R422	473	TYIQLK(*)TR(*)	K6	43.61	1000	1.3595505	-	0.943820	1.1516854
Nup85	Q8R480	92	IEEELSGK(*)SR(*)	K8	56.94	1000	-	0.894736	0.911111	0.902924
Nup85	Q8R480	631	LQDDDIETTK(*)VEMLR(*)	K10	46.75	1000	0.7526881	-	0.866666	0.8096774
Lrrc8c	Q8R502	550	ILSIK(*)SNVSK(*)	K5	31.9	29.88	-	0.578947	0.802469	0.6907083
Snap47	Q8R570	274	FIGK(*)PDVAYQLISAK(*)	K4	20.53	151.94	-	-	1.213333333	
Prpsap2	Q8R574	70	GK(*)DVFIIQTISK(*)	K2	46.86	137.5	1.5316455	-	1.152941	1.3422934
Actr1b	Q8R5C5	285	AIHK(*)SDMDLR(*)	K4	28.19	1000	0.6829268	-	1.914634	1.2987805
Usp15	Q8R5H1	125	VVEQGMFVK(*)HC(+57.02)K(*)	K9	55.13	57.45	0.8865979	0.646341	1	0.8443131
Usp15	Q8R5H1	21	SDIATLLK(*)TSLR(*)	K8	52.78	1000	0.9278350	1.341463	0.688888	0.9860625
Usp15	Q8R5H1	128	HC(+57.02)K(*)VEVYLTELK(*)	K3	36.66	140.59	-	-	1	

Usp15	Q8R5H1	125	K(*)VVEQGMFVK(*)HC(+57.02)K(*)	K10	25.48	62.26	1.0412371	0.841463	1.1666666	1.0164557
Usp15	Q8R5H1	363	AFK(*)TQVGR(*)	K3	13.31	1000	0.9278350	0.743902	1.2444444	0.9720606
Tmx1	Q8VBT0	82	VAK(*)VDVTEQTGLSGR(*)	K3	58.15	1000	1.1967213	0.951612	0.9625	1.0369447
Banp	Q8VBU8	283	EVQAVSNLSGQGK(*)HGK(*)	K13	63.54	27.07	-	1.383333	-	
Banp	Q8VBU8	339	GQSLAVK(*)SFSR(*)	K7	50.34	1000	-	0.633333	-	
Clptm1	Q8VBZ3	460	DK(*)STYIESSTK(*)	K2	22.29	142.42	0.8850574	1.142857	1.4177215	1.1485454
Clptm1	Q8VBZ3	534	LK(*)SVAHLPWR(*)	K2	12.7	1000	-	-	1.126582278	
Kctd5	Q8VC57	155	ISQMPVK(*)HVYR(*)	K7	9.32	1000	-	-	3.855421687	
Rbms2	Q8VC70	93	AILDK(*)TTNK(*)	K5	60.31	38.26	-	0.886075	0.9780219	0.932049
Rbms2	Q8VC70	18	PGISTFGYNK(*)NNK(*)	K10	34.08	49.37	-	-	0.725274725	
Rbms2	Q8VC70	83	LC(+57.02)QPYGK(*)IVSTK(*)	K7	32.91	56.94	0.9893617	1.025316	-	1.0073391
Ndc1	Q8VCB1	638	ASLK(*)TAIYR(*)	K4	27.5	1000	0.7009345	0.932432	-	0.8166835
Tubg2	Q8VCK3	301	LLQPK(*)NVMVSTGR(*)	K5	50.31	1000	-	-	-	
Med9	Q8VCS6	124	TK(*)NELLQK(*)	K2	57.58	156.85	0.7623762	0.720930	0.9565217	0.8132761
Acsf2	Q8VCW8	296	MPTK(*)TAEELR(*)	K4	33.36	1000	1.5113636	2.886075	2.6979166	2.3651188
Acsf2	Q8VCW8	182	QFK(*)TQQYYDILK(*)	K3	13.19	114.67	1.6477272	1.088607	-	1.3681674
Utp6	Q8VCY6	563	EELNHPFGK(*)PENC(+57.02)GQIYW	K9	43.64	1000	-	-	0.701030928	
Utp6	Q8VCY6	227	IIYK(*)NSISK(*)	K4	36.47	29.96	1.0795454	-	-	
Utp6	Q8VCY6	499	AVFK(*)SLQESR(*)	K4	10.49	1000	1.0227272	-	-	
Pik3r4	Q8VD65	993	GLLVAHLHEHK(*)SAVNR(*)	K11	20.08	1000	1.6465517	-	1.125	1.3857759
Hip1	Q8VD75	531	TQEQQDVLENLK(*)HELATSR(*)	K12	10.08	1000	-	-	1.545454545	
Myh9	Q8VDD5	1024	NK(*)HEAMITDLEER(*)	K2	99.29	1000	0.9489795	0.936708	0.9340659	0.9399181
Myh9	Q8VDD5	760	IGQSK(*)VFFR(*)	K5	98.37	1000	0.8571428	0.911392	0.9230769	0.8972041
Myh9	Q8VDD5	860	EK(*)HLAAENR(*)	K2	97.12	1000	0.8979591	0.848101	0.8571428	0.8677344
Myh9	Q8VDD5	1181	TLEDEAK(*)THEAQIQEMR(*)	K7	95.92	1000	1.0306122	0.924050	0.9450549	0.9665726
Myh9	Q8VDD5	1099	VEEEAAQK(*)NMALK(*)	K8	95.57	142.99	0.9489795	1.164556	0.7692307	0.9609224
Myh9	Q8VDD5	1775	SHAQK(*)NENAR(*)	K5	94.65	1000	0.9081632	0.886075	0.9780219	0.9240871
Myh9	Q8VDD5	580	VDYK(*)ADEWLMK(*)	K4	90.33	146.43	0.8571428	0.873417	0.9450549	0.8918718
Myh9	Q8VDD5	1338	QMEDEK(*)NSFR(*)	K6	89.76	1000	0.8265306	1.075949	0.6813186	0.8612662
Myh9	Q8VDD5	565	DK(*)ADFC(+57.02)IIHYAGK(*)	K2	87.59	281.19	0.9285714	0.886075	0.8131868	0.8759447
Myh9	Q8VDD5	1193	QK(*)HSQAVEELADQLEQTK(*)	K2	87.26	360.06	1.0102040	0.911392	0.8461538	0.9225834
Myh9	Q8VDD5	576	ADFC(+57.02)IIHYAGK(*)VDYK(*)	K11	83.63	109.97	0.9591836	1.063291	0.7802197	0.9342315
Myh9	Q8VDD5	1806	YK(*)ASIAALEAK(*)	K2	81.95	143.22	0.8571428	0.873417	0.9450549	0.8918718
Myh9	Q8VDD5	407	AQTK(*)EQADFAIEALAK(*)	K4	80.87	220.17	0.9489795	-	1	0.9744898

Myh9	Q8VDD5	1518	DDVGK(*)SVHELEK(*)	K5	78.11	136.94	0.8265306	0.898734	0.923076	0.8827806
Myh9	Q8VDD5	228	TVK(*)NDNSSR(*)	K3	77.68	1000	0.8775510	-	1.054945	0.966248
Myh9	Q8VDD5	737	GFMDGK(*)QAC(+57.02)VLMIK(*)	K6	74.93	216.51	0.9285714	0.797468	1.021978	0.9160059
Myh9	Q8VDD5	1410	VAAYDK(*)LEK(*)	K6	70.96	37.81	0.9183673	1.075949	-	0.9971584
Myh9	Q8VDD5	1513	TEMEDLMSSK(*)DDVGK(*)	K10	66.49	67.85	0.9591836	1.936708	1.164835	1.3535759
Myh9	Q8VDD5	1802	LQEM(+15.99)ESAVK(*)SK(*)	K9	65.05	22.85	1.1224489	1.430379	0.736263	1.0963642
Myh9	Q8VDD5	555	VVQEQGTHPK(*)FQK(*)	K10	61.82	55.63	0.8775510	0.797468	0.802197	0.8257391
Myh9	Q8VDD5	576	DK(*)ADFC(+57.02)IIHYAGK(*)VDY	K13	60.85	63.97	0.8571428	0.569620	0.835164	0.753976
Myh9	Q8VDD5	1022	LK(*)NK(*)HEAMITDLEER(*)	K2	60.45	20.41	1.0918367	1.151898	0.824175	1.0226371
Myh9	Q8VDD5	1274	TELADK(*)VTK(*)	K6	59.62	49.37	0.9387755	1.392405	0.780219	1.0371335
Myh9	Q8VDD5	435	INK(*)ALDK(*)	K3	59.31	87.62	0.5	-	0.945054	0.7225275
Myh9	Q8VDD5	1193	QK(*)HSQAVEELADQLEQTK(*)R(*)	K2	55.16	123.66	-	0.860759	0.923076	0.8919182
Myh9	Q8VDD5	545	SFVEK(*)VVQEQGTHPK(*)	K5	51.2	142.63	0.6530612	0.430379	0.835164	0.6395353
Myh9	Q8VDD5	835	VK(*)PLLNSIR(*)	K2	50.64	1000	0.4285714	0.493670	0.714285	0.5455093
Myh9	Q8VDD5	1631	DLEAHIDTANK(*)NR(*)	K11	47.66	1000	-	-	0.813186813	
Myh9	Q8VDD5	82	FSK(*)VEDM(+15.99)AELTC(+57.02)	K3	45.6	172.72	-	-	1.076923077	
Myh9	Q8VDD5	810	QQQLTAMK(*)VLQR(*)	K8	45.52	1000	1.1530612	0.721518	1.219780	1.0314535
Myh9	Q8VDD5	856	EAELTK(*)VR(*)	K6	38.72	1000	0.5816326	1.037974	0.725274	0.7816274
Myh9	Q8VDD5	682	AGK(*)LDPHLVLDQLR(*)	K3	36.21	1000	-	-	0.934065934	
Myh9	Q8VDD5	833	LFTK(*)VK(*)PLLNSIR(*)	K4	35.37	55.92	0.9591836	0.531645	0.725274	0.7387013
Myh9	Q8VDD5	289	LLSGAGEHLK(*)TDLLLEPYNK(*)	K10	31.66	141.74	1.6326530	-	1.329670	1.4811617
Myh9	Q8VDD5	199	LAHVASSHK(*)SK(*)	K9	31.55	30.83	0.6734693	-	1.021978	0.8477237
Myh9	Q8VDD5	38	LVWVPSSK(*)NGFEPASLK(*)	K8	20.7	35.44	1.0306122	1.240506	-	1.1355593
Myh9	Q8VDD5	1477	EK(*)ETK(*)ALSLAR(*)	K5	16.14	53.53	-	0.772151	0.912087	0.8421199
Myh9	Q8VDD5	637	IIGLDQVAGMSETALPGAFK(*)TR(*)	K20	15.92	1000	-	-	2.252747253	
Phip	Q8VDD9	773	ILTVSK(*)NHAHEHFLDLGDSK(*)	K6	43.01	37.92	-	0.850574	-	
Phip	Q8VDD9	1533	AFVSK(*)TNTSAMPGK(*)	K5	20.92	140.79	-	-	3.021052632	
Phip	Q8VDD9	116	SC(+57.02)K(*)HVVWK(*)	K3	19.16	100.21	-	0.942528	1.168421	1.0554749
Uhrf1	Q8VDF2	404	DWGK(*)GMAC(+57.02)VGR(*)	K4	61.57	1000	0.7582417	0.623376	0.808988	0.7302024
Uhrf1	Q8VDF2	564	YDGIYK(*)VVK(*)	K6	41.5	20.7	0.8131868	-	1.011235	0.9122114
Uhrf1	Q8VDF2	24	LTK(*)VQELR(*)	K3	4.67	1000	-	-	0.528089888	
Parn	Q8VDG3	319	LLDTK(*)LMASTQPFK(*)	K5	36.01	161.52	0.9787234	-	0.663551	0.8211374
Ppcs	Q8VDG5	46	VVLITSGGTK(*)VPLEAR(*)	K10	62.29	1000	2.5	-	1.895833	2.1979167
Hdlbp	Q8VDJ3	726	QTK(*)SFTVDIR(*)	K3	85.58	1000	-	-	7.395604396	

Hdlbp	Q8VDJ3	477	IPPDSEK(*)SNLIR(*)	K7	79.05	1000	0.9090909	0.7625	0.791208	0.8209332
Hdlbp	Q8VDJ3	445	MDYVEINIDHK(*)FHR(*)	K11	72.88	1000	1.2424242	1.05	1.219780	1.1707348
Hdlbp	Q8VDJ3	453	HLIGK(*)SGANINR(*)	K5	70.54	1000	0.8484848	0.8375	0.967032	0.8843393
Hdlbp	Q8VDJ3	1066	YHPK(*)IIGR(*)	K4	56.63	1000	-	-	0.813186813	
Hdlbp	Q8VDJ3	510	TK(*)DLIIEQR(*)	K2	53.89	1000	0.9494949	0.775	0.780219	0.8349049
Hdlbp	Q8VDJ3	594	QFHK(*)NIIGK(*)	K4	48.27	76.42	0.9292929	0.95	1.263736	1.0476764
Hdlbp	Q8VDJ3	599	NIIGK(*)GGANIK(*)	K5	45.19	86.11	0.7979797	1.025	0.791208	0.8713962
Hdlbp	Q8VDJ3	179	LQDLELK(*)TATK(*)	K7	30.38	20.17	0.9595959	-	-	
Hdlbp	Q8VDJ3	846	SGTQSDK(*)VTLK(*)	K7	26.08	55.36	-	1.375	0.901098	1.1380495
Hdlbp	Q8VDJ3	90	YK(*)DMNQFGEGEQAK(*)	K2	14.79	83.6	-	-	2.252747253	
Hdlbp	Q8VDJ3	466	IK(*)DQYK(*)VSVR(*)	K6	12.41	58.99	-	-	0.857142857	
Zgpat	Q8VDM1	352	GK(*)SLDQC(+57.02)AEILQK(*)	K2	54.65	164.78	-	-	-	
Psmd2	Q8VDM4	350	VPDDIYK(*)THLENNR(*)	K7	69.96	1000	0.9680851	0.8375	0.956043	0.920543
Psmd2	Q8VDM4	773	LAQGLTHLGK(*)GTLTLC(+57.02)PYI	K10	37.28	1000	-	-	0.978021978	
Psmd2	Q8VDM4	754	QLAQYHAK(*)DPNNLFMVR(*)	K8	33.09	1000	0.8191489	-	1.494505	1.1568272
Psmd2	Q8VDM4	860	PK(*)TITGFQTHTTPVLLAHGER(*)	K2	21.9	1000	0.8085106	-	1.197802	1.0031564
Hnrnpul1	Q8VDM6	441	TTWAIK(*)HAASNPSK(*)	K6	79.92	170.86	0.7979797	0.733333	0.884210	0.8051746
Hnrnpul1	Q8VDM6	376	VLVK(*)NC(+57.02)AVEFNFGQR(*)	K4	43.97	1000	-	0.946666	0.831578	0.8891228
Hnrnpul1	Q8VDM6	271	INEEISVK(*)HLPSTEPDPHVVR(*)	K8	22.92	1000	-	1.586666	-	
Hnrnpul1	Q8VDM6	419	SK(*)AEC(+57.02)EILMMVGLPAAGK	K2	7.81	90.71	-	-	1.915789474	
Atp1a1	Q8VDN2	476	YSK(*)IVEIPFNSTNK(*)	K3	61.93	160.69	0.84375	0.8	0.869565	0.8377717
Atp1a1	Q8VDN2	502	NPNASEPK(*)HLLVMK(*)	K8	55.27	134.33	0.9479166	0.8875	0.880434	0.9052838
Atp1a1	Q8VDN2	156	SSK(*)IMESFK(*)	K3	5	56.47	-	-	0.913043478	
Ccar2	Q8VDP4	557	IYK(*)TLLSLPEK(*)	K3	37.79	150.63	-	1	0.649484	0.8247423
Mitd1	Q8VDV8	64	TK(*)ISGYMDR(*)	K2	26.77	1000	-	-	0.741007194	
Ddx39a	Q8VDW0	190	NVK(*)HFVLDEC(+57.02)DK(*)	K3	93.31	242.72	0.9450549	0.921052	1.022222	0.9627766
Ddx39a	Q8VDW0	383	FGTK(*)GLAVTFVSDENDAK(*)	K4	5.15	132.96	-	-	1.144444444	
Naa40	Q8VE10	28	AAMDAVC(+57.02)AK(*)VDAANR(*)	K9	11.87	1000	1.1570247	-	-	
Pdcd10	Q8VE70	172	YSK(*)SFSDTLK(*)	K3	67.79	51.17	-	-	1.344086022	
Pdcd10	Q8VE70	179	SFSDTLK(*)TYFK(*)	K7	9.53	68.07	-	-	0.860215054	
Cul7	Q8VE73	1160	YYVLQK(*)SSSELFGPR(*)	K6	38.62	1000	0.475	-	-	
Rpe	Q8VEE0	159	FMEDMMPK(*)VHWLR(*)	K8	48.93	1000	-	-	1.075268817	
Rpa1	Q8VEE4	268	VYYFSK(*)GALK(*)	K6	52.36	35.82	1.1595744	0.271604	0.821052	0.750744
Rpa1	Q8VEE4	176	GK(*)PAGTGLLQPSGGTQSK(*)	K2	48.03	139.37	1.0851063	0.851851	1.031578	0.9895124

Rpa1	Q8VEE4	458	TLHEAK(*)SENLGQGDK(*)	K6	38.53	66.16	-	1.172839	0.873684:	1.0232619
Rpa1	Q8VEE4	322	AK(*)DALVDIIGIC(+57.02)K(*)	K2	29.3	267.5	0.9255319	1.123456	0.873684:	0.9742243
Nle1	Q8VEJ4	153	FWDLSTETPHFTC(+57.02)K(*)GHR(*	K14	39.57	1000	1.0853658	1.405405	0.964285:	1.1516857
Vps4a	Q8VEJ9	210	LVK(*)NLFELAR(*)	K3	38.43	1000	-	-	0.74	
Hnrnpu	Q8VEK3	328	HLYTK(*)DIDIHEVR(*)	K5	96.38	1000	0.9670329	0.857142	0.977777:	0.9339845
Hnrnpu	Q8VEK3	363	GIK(*)TC(+57.02)NC(+57.02)ETEDY(	K3	94.61	284.27	0.9120879	0.714285	1.044444:	0.8902727
Hnrnpu	Q8VEK3	541	APQC(+57.02)LGK(*)FIEIAAR(*)	K7	90.52	1000	1.0109890	0.610389	0.911111:	0.8441632
Hnrnpu	Q8VEK3	309	GK(*)VC(+57.02)FEMK(*)	K2	80.41	156.85	0.9230769	0.974025	1.122222:	1.0064417
Hnrnpu	Q8VEK3	181	EAAGK(*)SSGPTSLFAVTVAPPGAR(*)	K5	76.75	1000	1.0329670	0.935064	0.788888:	0.9189736
Hnrnpu	Q8VEK3	486	IGLPGAGK(*)TTWVTK(*)	K8	72.03	42.99	0.6813186	0.727272	1.044444:	0.8176786
Hnrnpu	Q8VEK3	673	SGK(*)NQFNR(*)	K3	68.79	1000	-	0.519480	0.877777:	0.6986291
Hnrnpu	Q8VEK3	492	TTWVTK(*)HAAENPGK(*)	K6	58.97	171.6	2.8461538	0.844155	1.433333:	1.707881
Hnrnpu	Q8VEK3	486	K(*)DC(+57.02)EVVMMIGLPGAGK(*	K16	58.59	55.13	0.4505494	1.077922	0.955555:	0.828009
Hnrnpu	Q8VEK3	486	MIGLPGAGK(*)TTWVTK(*)	K9	56.55	45.03	0.9230769	-	0.977777:	0.9504274
Hnrnpu	Q8VEK3	31	GLK(*)ADLMDR(*)	K3	49.36	1000	0.8241758	1.662337	0.944444:	1.1436526
Hnrnpu	Q8VEK3	500	HAAENPGK(*)YNILGTNTIMDK(*)	K8	33.94	35.28	-	3.220779	-	
Hnrnpu	Q8VEK3	541	GK(*)FIEIAAR(*)	K2	24.55	1000	1.0769230	0.805194	-	0.9410589
Hnrnpu	Q8VEK3	440	EK(*)PYFPIPEDC(+57.02)TFIQNVPLI	K2	23.64	1000	-	-	1.4	
Ing3	Q8VEK6	403	GK(*)WFC(+57.02)PQC(+57.02)TAA	K2	41.99	158.53	-	-	-	
Ing3	Q8VEK6	264	NNDFQLGK(*)EFSIPR(*)	K8	28.89	1000	-	-	-	
Ing3	Q8VEK6	167	FK(*)SEALLSTLTSDASK(*)	K2	15.68	148.34	-	-	-	
Slc25a3	Q8VEM8	308	GVWK(*)GLFAR(*)	K4	95.28	1000	0.6938775	0.692307	0.903225:	0.763137
Slc25a3	Q8VEM8	213	YK(*)GVAPLWMR(*)	K2	69.87	1000	0.7244897	0.769230	0.763440:	0.7523871
Rbm39	Q8VH51	291	SK(*)GYGFITFSDSEC(+57.02)AK(*)	K2	34.49	189.02	1.125	-	1.152173:	1.138587
Sec63	Q8VHE0	407	IK(*)TIQDLVSLK(*)	K2	35.52	124.12	0.7179487	0.835294	0.913978:	0.8224071
Sec63	Q8VHE0	633	SK(*)ITHPVYSLYFPEEK(*)	K2	13.07	64.77	-	1.247058	-	
Agap3	Q8VHH5	139	VGIVGNLSSGK(*)SALVHR(*)	K11	23.37	1000	-	-	0.786885246	
Pofut2	Q8VHI3	71	VASLLK(*)TLLK(*)	K6	49.84	49.79	0.7961165	-	0.954545:	0.875331
Pofut2	Q8VHI3	244	SMVFAK(*)HLR(*)	K6	38.61	1000	0.9514563	0.845238	-	0.8983472
Pofut2	Q8VHI3	171	VDAR(*)PC(+57.02)IDPLLYSQDK(*)I	K16	37.27	1000	1.3300970	-	-	
Pofut2	Q8VHI3	171	PC(+57.02)IDPLLYSQDK(*)HEYYR(*)	K12	8.15	1000	-	-	1.272727273	
Dhx36	Q8VHK9	855	VHTK(*)SDGLVSIHPK(*)	K4	36.79	124.32	0.7191011	1.013333	0.580645:	0.7710265
Slc14a1	Q8VHL0	361	MPLSK(*)VTYSEENR(*)	K5	45.06	1000	-	0.627272	1.113636:	0.8704545
Gatad2b	Q8VHR5	463	LK(*)NAFVK(*)	K2	27.26	81.69	-	-	1.3625	

Flnc	Q8VHX6	2686	TPC(+57.02)EEVYVK(*)HMGNR(*)	K9	93.36	1000	0.9555555	0.932432	0.988372(	0.9587867
Flnc	Q8VHX6	2464	VHTPSGAVEEC(+57.02)YVSELDSDK(	K20	93.07	1000	1.2444444	0.945945	1.046511(	1.0789673
Flnc	Q8VHX6	1936	FDDK(*)HIPGSPFTAK(*)	K4	89.97	94.89	1.0333333	0.878378	0.918604(	0.9434388
Flnc	Q8VHX6	2118	FADK(*)HVPGSPFTVK(*)	K4	86.97	152.36	0.9555555	0.797297	0.906976	0.8866099
Flnc	Q8VHX6	1368	AFGPGLEGGLVNK(*)ANR(*)	K13	84.01	1000	1.0333333	0.756756	-	0.895045
Flnc	Q8VHX6	1832	YAPTEK(*)GLHQMGIK(*)	K6	65.51	102.06	1.0777777	0.932432	0.918604(	0.9762716
Flnc	Q8VHX6	503	VFTK(*)GAGSGELK(*)	K4	62.97	117.11	-	1.067567	0.686046!	0.876807
Flnc	Q8VHX6	2617	LSGGHSLHETSTVLVETVTK(*)SSSSR(*	K20	59.52	1000	1.0888888	0.986486	0.965116	1.0134972
Flnc	Q8VHX6	2023	SGK(*)HVTNSPFK(*)	K3	51.46	131.34	0.9555555	1.027027	0.837209	0.9399306
Flnc	Q8VHX6	2617	SLHETSTVLVETVTK(*)SSSSR(*)	K15	50.62	1000	0.9333333	0.378378	0.872093(	0.7279349
Flnc	Q8VHX6	344	TYAVSYVPK(*)VAGLHK(*)	K9	47.41	30.1	0.8333333	1.783783	0.918604(	1.1785739
Flnc	Q8VHX6	2044	ILVGPSEIGDASK(*)VR(*)	K13	44.28	1000	0.8555555	-	1.034883	0.9452196
Flnc	Q8VHX6	1533	IK(*)VLPSHDASK(*)	K2	23.58	113.83	-	-	4.197674419	
Flnc	Q8VHX6	2548	SVTIDGPSK(*)VQLDC(+57.02)R(*)	K9	19.36	1000	-	1.459459	-	
Flnc	Q8VHX6	2654	VGQK(*)NSFTVDC(+57.02)SK(*)	K4	18.97	124.12	1.6222222	1.175675	1.441860	1.4132528
Cspg4	Q8VHY0	1716	NK(*)GLWVPEGQR(*)	K2	5.78	1000	-	-	1.366666667	
Sfpq	Q8VIJ6	324	GK(*)GFGFIK(*)	K2	83.41	82.63	1.1195652	0.75	1.011627	0.9603977
Sfpq	Q8VIJ6	405	STGK(*)GIVEFASK(*)	K4	80.34	188.52	1.0108695	0.657894	0.941860	0.8702083
Sfpq	Q8VIJ6	487	WK(*)SLDEMEK(*)	K2	72.05	163.4	0.8152173	0.736842	1.034883	0.8623144
Sfpq	Q8VIJ6	510	DK(*)LESEMEDAYHEHQANLLR(*)	K2	51.97	1000	-	-	1.139534884	
Sfpq	Q8VIJ6	224	PGGHPK(*)PPHR(*)	K6	51.33	1000	-	1.039473	0.848837	0.9441554
Sfpq	Q8VIJ6	224	PGGGPGMGAPGGHPK(*)PPHR(*)	K15	24.92	1000	-	4.289473	0.755813	2.5226438
Ciapin1	Q8WTY4	245	AC(+57.02)K(*)NC(+57.02)TC(+57.(	K3	74.16	1000	0.9038461	0.942857	0.864583	0.9037622
Acot7	Q91V12	268	HC(+57.02)K(*)TNIVTASVDAINFHDI	K3	61.65	178.3	-	0.818181	1.064516	0.941349
Acot7	Q91V12	158	LTNK(*)ATLWYVPLSLK(*)	K4	44.85	173.87	1.6354166	-	0.967741	1.3015793
Acot7	Q91V12	173	NVDK(*)VLEVPPIVYLR(*)	K4	4.82	1000	-	-	1.870967742	
Rab14	Q91V41	24	YIIIGDMGVGK(*)SC(+57.02)LLHQFT	K11	57.71	32.22	0.9473684	-	0.988764(	0.9680662
Sfxn3	Q91V61	169	SLTK(*)HLPPLVGR(*)	K4	30.49	1000	1.0618556	-	1.243589	1.1527227
Acly	Q91V92	968	VK(*)SINNPDMR(*)	K2	95.5	1000	0.6702127	0.948717	0.685393	0.768108
Acly	Q91V92	272	LTLLNPK(*)GR(*)	K7	79.82	1000	0.7765957	0.705128	0.988764(	0.823496
Acly	Q91V92	230	VDATADYIC(+57.02)K(*)VK(*)	K10	78.12	33.18	0.7659574	0.948717	0.887640	0.8674386
Acly	Q91V92	488	HTK(*)AIVWGMQTR(*)	K3	68	1000	0.7340425	0.910256	1.011235	0.8851783
Acly	Q91V92	765	SSEVQFGHAGAC(+57.02)ANQASETA	K23	58.62	56.99	0.9468085	-	0.797752	0.8722807
Acly	Q91V92	1070	LK(*)QGLYR(*)	K2	55.98	1000	1.1170212	0.910256	0.988764(	1.0053472

Acly	Q91V92	958	EGK(*)LIMGIGHR(*)	K3	50.43	1000	0.5	0.705128	0.977528	0.7275521
Acly	Q91V92	478	AK(*)PAMPQGK(*)SATLFSR(*)	K9	36.82	95.63	0.6702127	1.064102	0.752808	0.8290414
Acly	Q91V92	530	QK(*)FYWGHK(*)	K2	25.44	47.92	-	-	1.887640449	
Acly	Q91V92	544	EILIPVFK(*)NMADAMK(*)	K8	18.27	58.52	-	-	1.123595506	
Poldip2	Q91VA6	129	GK(*)THTYYQVLIDAR(*)	K2	49.68	1000	1.1686746	0.797468	0.905263	0.9571354
Eif4a3	Q91VC3	382	GVAINFVK(*)NDDIR(*)	K8	45.69	1000	0.6770833	0.571428	0.75	0.6661706
Eif4a3	Q91VC3	382	K(*)GVAINFVK(*)NDDIR(*)	K9	40.4	107.29	1.2083333	1.464285	0.967391	1.2133368
Ghitm	Q91VC9	77	IFK(*)IDQMGR(*)	K3	24.28	1000	1.7826086	1.352941	1.181818	1.4391227
Snx9	Q91VH2	288	YK(*)HFDWLYER(*)	K2	66.4	1000	1.0408163	0.925	0.989583	0.9851332
Snx9	Q91VH2	407	FTK(*)AMDDGVK(*)	K3	52.61	127.53	1.1530612	-	1.447916	1.3004889
Memo1	Q91VH6	115	IYGELWK(*)TGMFER(*)	K7	60.31	1000	-	-	0.927835052	
Cavin3	Q91VJ2	121	GK(*)LHVLLFK(*)	K2	48.05	184.45	0.8686868	1	1.103448	0.9907117
Bzw2	Q91VK1	391	GK(*)SVFLDQMK(*)	K2	75.43	183.32	1.0113636	1.068493	-	1.0399284
Bzw2	Q91VK1	320	K(*)EELVAEQALK(*)HLK(*)	K11	67.58	1000	0.9431818	1.013698	0.978494	0.9784584
Bzw2	Q91VK1	320	EELVAEQALK(*)HLK(*)	K10	63.69	1000	0.9431818	0.863013	0.870967	0.8923878
Bzw2	Q91VK1	192	LFK(*)AWMAEK(*)	K3	8.57	36.87	-	1.150684	1.516129	1.333407
Ppa2	Q91VM9	48	LFFK(*)HVAGHYISPFHDIPLK(*)	K4	37.66	174.61	1.7304347	-	1.023529	1.3769821
Chchd6	Q91VN4	203	IQEK(*)NAELYK(*)	K4	20.94	63.05	-	0.666666	-	
Atp5f1c	Q91VR2	115	QMK(*)NEVAALTAAGK(*)	K3	89.6	207.94	0.7431192	0.802325	0.941176	0.8288738
Atp5f1c	Q91VR2	39	SIK(*)NIQK(*)	K3	82.91	88.98	0.8256880	0.953488	0.921568	0.9002484
Atp5f1c	Q91VR2	138	IK(*)GILYR(*)	K2	82.7	1000	0.9266055	0.860465	0.901960	0.8963438
Atp5f1c	Q91VR2	262	MTAMDNASK(*)NASDMIDK(*)	K9	52.56	154.54	0.6513761	0.872093	0.901960	0.8084767
Ddx1	Q91VR5	132	GK(*)HYYEVSC(+57.02)HDQGLC(+5	K2	88.05	1000	0.9479166	0.768292	1.076923	0.9310441
Ddx1	Q91VR5	268	APDNYIVK(*)SQHTGNAQVSQTK(*)	K8	78.22	37.79	1.0729166	0.670731	0.901098	0.8815824
Ddx1	Q91VR5	82	TTIK(*)TGASVLNK(*)	K4	58.41	75.74	-	-	0.934065934	
Ddx1	Q91VR5	625	EK(*)VWYHVC(+57.02)SNR(*)	K2	49	1000	-	-	0.692307692	
Ddx1	Q91VR5	516	TK(*)IDC(+57.02)DNLEQYFMQQGG	K2	42.73	161.52	1.2916666	-	1	1.1458333
Ddx1	Q91VR5	505	EHK(*)MDQAIIFC(+57.02)R(*)	K3	36.95	1000	1.2395833	0.780487	0.956043	0.9920384
Ddx1	Q91VR5	516	TK(*)IDC(+57.02)DNLEQYFMQQGG	K2	32.46	161.52	1.625	-	1.010989	1.3179945
Ddx1	Q91VR5	234	NQALFPAC(+57.02)VLK(*)NAELK(*)	K11	21.36	38.19	0.65625	1.719512	-	1.1878811
Fam3c	Q91VU0	67	AC(+57.02)PEK(*)HFAFK(*)	K5	75.18	100.21	1.0943396	0.653846	1.047619	0.9319349
Fam3c	Q91VU0	201	SPFEQHIK(*)NNK(*)	K8	28.45	37.54	0.2264150	-	-	
Sh3bgrl3	Q91VW3	18	EIK(*)SQQSEVTR(*)	K3	34.1	1000	0.9636363	-	0.905263	0.9344498
Ubap2	Q91VX2	48	LAQVIFDK(*)NDSDFEAK(*)	K8	9.82	52.42	-	-	2.871794872	

Csde1	Q91W50	166	INFVIDNNK(*)HTGAVSAR(*)	K9	83.83	1000	1.0526315	0.875	0.927835(	0.9518222
Csde1	Q91W50	307	SK(*)VTLLEGDHVR(*)	K2	68.37	1000	1.0526315	0.8625	0.865979:	0.927037
Csde1	Q91W50	758	LK(*)NITLDDASAPR(*)	K2	43.92	1000	0.6526315	0.725	1.298969(	0.8922002
Csde1	Q91W50	288	IK(*)VDFVIPK(*)	K2	37.62	121.12	-	-	0.907216495	
Rbms1	Q91W59	97	AILDK(*)ATNK(*)	K5	61	88.98	-	0.574712	1.476190‹	1.0254516
Txndc5	Q91W90	230	IGK(*)VDC(+57.02)TQHYAVC(+57.0	K3	86.3	1000	0.9255319	0.810126	1.077777	0.9378121
Txndc5	Q91W90	104	VYVAK(*)VDC(+57.02)TADSDVC(+5	K5	40.54	1000	-	1.202531	1.122222	1.1623769
Anapc4	Q91W96	709	TMHFEK(*)HWR(*)	K6	62.85	1000	1.1153846	-	1.269230	1.1923077
Setd3	Q91WC0	222	YK(*)VIQTHPHANK(*)	K2	10.06	133.99	-	-	1.844444444	
Polr3d	Q91WD1	396	LVC(+57.02)SPDFESLLDHK(*)HR(*)	K14	25.37	1000	-	-	-	
Fam192a	Q91WE2	157	LLAGAVK(*)HK(*)	K7	44.1	36.05	0.9038461	0.722891	0.826086	0.8176082
Fam192a	Q91WE2	121	SNLNK(*)VGISAENK(*)	K5	13.62	99.23	1.5288461	-	1.271739	1.4002926
Fubp1	Q91WJ8	239	ITGDPYK(*)VQQAK(*)	K7	81.07	87.95	0.8241758	0.947368	0.838709	0.8700846
Fubp1	Q91WJ8	308	IQFK(*)PDDGTTPDR(*)	K4	42.66	1000	0.9010989	1.302631	1.010752	1.0714944
Eif3h	Q91WK2	227	SAVADK(*)HELLSLASSNHLGK(*)	K6	76.95	247.71	0.98	0.875	0.927083	0.9273611
Eif3h	Q91WK2	227	K(*)SAVADK(*)HELLSLASSNHLGK(*)	K7	9.71	41.57	-	-	1.489583333	
Yars	Q91WQ3	380	ILSVEK(*)HPDADSLYVEK(*)	K6	72.55	50.04	0.8555555	0.863013	-	0.8592846
Yars	Q91WQ3	513	QTNFMTK(*)LGFVSC(+57.02)K(*)	K7	63.6	71.65	-	-	1.103448276	
Yars	Q91WQ3	272	HVLFPLK(*)SEFVILR(*)	K7	51.3	1000	0.6888888	-	0.942528	0.8157088
Yars	Q91WQ3	265	IK(*)HVLFPLK(*)	K2	47.4	184.45	1.0222222	0.835616	1.091954(	0.9832642
Yars	Q91WQ3	310	DFAAEVVHPGDLK(*)NSVEVALNK(*)	K13	40.57	76.18	-	-	0.747126437	
Yars	Q91WQ3	356	GPAK(*)NSEPEEVIPSR(*)	K4	17.32	1000	-	1.753424	0.919540	1.3364824
Dap	Q91XC8	12	LETK(*)AGHPPAVK(*)	K4	8.07	21.81	2.8076923	-	-	
Vps36	Q91XD6	218	FK(*)SYLLSMGIANPVTR(*)	K2	23.31	1000	1.0796460	-	1.276315	1.1779809
Osbpl1a	Q91XL9	315	VPK(*)NSVQQSR(*)	K3	67.88	1000	1.0985915	-	0.989473	1.0440326
Wrnip1	Q91XU0	628	DLGYGK(*)GYK(*)	K6	40.88	28.96	0.8383838	-	0.75	0.7941919
Wrnip1	Q91XU0	296	FVTLSATNAK(*)TNDVR(*)	K10	21.71	1000	1.0101010	0.654545	0.452380	0.7056758
Pip4k2c	Q91XU3	101	FK(*)EYC(+57.02)PQVFR(*)	K2	44	1000	-	-	0.956043956	
Pip4k2c	Q91XU3	83	IK(*)VNNHFFHR(*)	K2	11.42	1000	-	-	1.703296703	
Mapk8	Q91Y86	166	SDC(+57.02)TLK(*)ILDFGLAR(*)	K6	44.88	1000	-	-	2.342857143	
Ipo9	Q91YE6	773	LVSTLISK(*)AGR(*)	K8	76.4	1000	0.8367346	0.662650	0.851063	0.783483
Ipo9	Q91YE6	277	AVTALVK(*)NFPK(*)	K7	75.44	77.13	1.0510204	1	0.946808	0.9992763
Ipo9	Q91YE6	884	VK(*)GEEIYSMDEGIR(*)	K2	48.1	1000	0.8979591	0.746987	-	0.8224736
Ipo9	Q91YE6	436	HLQEAEQTK(*)ASGTEHWWK(*)	K9	43.58	85.33	1.2551020	0.506024	-	0.8805631

Ipo9	Q91YE6	458	IHEAC(+57.02)MLALGSVK(*)SIITDS\	K13	20.38	115.01	-	-	1.021276596	
Ipo9	Q91YE6	466	SIITDSVK(*)NGR(*)	K8	14.9	1000	0.6836734	-	-	
Rbm5	Q91YE7	314	TIGVDFAK(*)SAR(*)	K8	55.98	1000	0.9042553	1.050847	1.323529	1.0928774
Rbm5	Q91YE7	787	GAGLGAK(*)GSAYGLSGADSYK(*)	K7	51.81	134.51	-	0.847457	-	
Rbm5	Q91YE7	169	LVIQGK(*)HIAMHYSNPR(*)	K6	9.19	1000	-	-	1.205882353	
Atl3	Q91YH5	315	EINGSK(*)VTC(+57.02)R(*)	K6	9.99	1000	-	1.108433	0.802197	0.9553158
Snx4	Q91YJ2	361	GMTTK(*)LFGQETPEQR(*)	K5	22.11	1000	-	0.902439	0.923076	0.912758
Uap1	Q91YN5	152	LQQLAEK(*)HHGNK(*)	K7	52.64	32.16	-	0.802631	-	
Uap1	Q91YN5	180	EFFTK(*)HK(*)	K5	9.25	25.66	1.1839080	0.973684	0.917647	1.0250798
Bag2	Q91YN9	111	NPQQEESLK(*)HATR(*)	K9	53.21	1000	1.3541666	-	1.097826	1.2259964
Bag2	Q91YN9	200	GAGSK(*)SLQNTDGK(*)	K5	31.03	115.01	0.9270833	-	1.217391	1.0722373
Nln	Q91YP2	583	QIVLSK(*)VDQSLHTNASLDAASEYAK	K6	51.03	48.25	-	-	1.01010101	
Dera	Q91YP3	218	MAGSDFIK(*)TSTGK(*)	K8	34.66	51	-	-	1.011494253	
Dera	Q91YP3	137	AAGC(+57.02)SIPVASVATGFPAGQT	K23	3.51	1000	-	-	1.83908046	
Rpn1	Q91YQ5	539	LK(*)TEGSDLC(+57.02)DR(*)	K2	98.04	1000	1.4081632	1	1.042553	1.1502388
Rpn1	Q91YQ5	188	NVESYTK(*)LGNPSR(*)	K7	62.32	1000	0.8265306	0.686746	0.946808	0.8200287
Rpn1	Q91YQ5	565	ELVLK(*)SAVEAER(*)	K5	57.83	1000	1.6224489	1.638554	1.861702	1.7075684
Rpn1	Q91YQ5	170	QFVVFEGNHYFYSPYPTK(*)TQTMR(*	K18	15.7	1000	-	-	2	
Twf1	Q91YR1	171	INEVQTDVSVDTK(*)HQTLQGVAFPIS	K13	57.24	1000	1.9375	-	1.021978	1.479739
Prpf6	Q91YR7	616	AEVLWLMGAK(*)SK(*)	K10	66.03	22.85	1.2988505	1.191780	1.607142	1.3659248
Prpf6	Q91YR7	189	LTPVPDSFFAK(*)HLQTGENHTSVDPF	K11	55.24	1000	0.9195402	0.698630	0.964285	0.8608187
Prpf6	Q91YR7	793	AGLK(*)NIANTLMAK(*)	K4	46.09	140.79	0.9655172	0.684931	1.214285	0.9549115
Prpf6	Q91YR7	585	AAYFEK(*)NHGTR(*)	K6	24	1000	1.3333333	1.424657	1.214285	1.3240922
Ptgr1	Q91YR9	5	VQAK(*)SWTLK(*)	K4	66.61	73.54	0.7755102	0.896103	1.09375	0.921788
Ptgr1	Q91YR9	197	TVK(*)SLEEALR(*)	K3	50.28	1000	0.8367346	1.038961	0.760416	0.8787041
Ndufv1	Q91YT0	375	LIEFYK(*)HESC(+57.02)GQC(+57.02	K6	43.81	1000	0.7920792	0.590361	1.188888	0.8571098
Ythdf2	Q91YT7	416	VFIIK(*)SYSEDDIHR(*)	K5	80.47	1000	1.0361445	0.876543	0.946236	0.9529748
Ythdf2	Q91YT7	408	DFDWNLK(*)HGR(*)	K7	72.39	1000	-	-	0.892473118	
Ythdf2	Q91YT7	548	IIASYK(*)HTTSIFDDFSHYEK(*)	K6	47.37	124.79	0.8433734	0.938271	1.032258	0.9379677
Dnajc3	Q91YW3	39	GVNADVEK(*)HLELGK(*)	K8	69.95	129.19	0.9887640	0.7625	0.904255	0.8851731
Dnajc3	Q91YW3	150	EAESQLVK(*)ADEMQR(*)	K8	7.44	1000	-	-	1.925531915	
Ptbp2	Q91Z31	92	VTNILMLK(*)GK(*)	K8	56.64	1000	1.1147540	0.873417	0.428571	0.8055811
Ptbp2	Q91Z31	137	ELK(*)TDNTLNQR(*)	K3	17.72	1000	-	1.772151	-	
Ptbp2	Q91Z31	411	VTLSK(*)HQTVQLPR(*)	K5	9.16	1000	2.7540983	-	-	

Grhpr	Q91Z53	235	MK(*)NTAIFINISR(*)	K2	10.71	1000	-	-	1.042553191	
Srgap2	Q91Z67	470	LQAK(*)HDLLQK(*)	K4	60.19	129.72	-	0.680412	-	
Ugp2	Q91ZJ5	127	GPK(*)SLIGVR(*)	K3	73.71	1000	0.8723404	0.786666	0.988235	0.8824141
Ugp2	Q91ZJ5	319	AHVDEFK(*)SVSK(*)	K7	57.5	28.7	-	-	1.588235294	
Ugp2	Q91ZJ5	438	LGSSFTK(*)VQDYLR(*)	K7	50.29	1000	-	-	1.564705882	
Ugp2	Q91ZJ5	438	TK(*)VQDYLR(*)	K2	27.76	1000	1.6702127	-	1.670588	1.6704005
Snx18	Q91ZR2	304	YK(*)HFDWLYAR(*)	K2	5.04	1000	-	-	1.517241379	
Dst	Q91ZU6	6136	QFK(*)SEAYQQQIEMER(*)	K3	42.52	1000	1.0404040	1.038461	0.752577	0.9438143
Dst	Q91ZU6	3903	SFSEDVISHK(*)GDLR(*)	K10	35.99	1000	-	-	0.804123711	
Dst	Q91ZU6	6479	EQIIELDK(*)TGTHLK(*)	K8	32.09	49.74	0.8787878	1.051282	1.020618	0.9835628
Mia2	Q91ZV0	685	LLDK(*)VSIVQK(*)	K4	4.96	41.24	-	-	1.190082645	
Smarca5	Q91ZW3	989	FDWFLK(*)SR(*)	K6	52.98	1000	0.8453608	-	1.011764	0.9285628
Smarca5	Q91ZW3	923	ALDTK(*)IGR(*)	K5	27.39	1000	0.9690721	-	1.305882	1.1374773
Smarca5	Q91ZW3	798	TIGYK(*)VPR(*)	K5	21.73	1000	-	-	0.8	
Smarca5	Q91ZW3	928	YK(*)APFHQLR(*)	K2	20.29	1000	1.3298969	0.923076	0.917647	1.0568736
Lrp1	Q91ZX7	2211	TILK(*)SIHLSDER(*)	K4	65.72	1000	0.8118811	0.772151	0.842105	0.8087128
Lrp1	Q91ZX7	1695	LDGSFK(*)NAVVQGLEQPHGLVVHPL	K6	37.96	1000	-	-	1.442105263	
Lrp1	Q91ZX7	1815	MGTC(+57.02)NK(*)ADGSGSVVLR(*	K6	21.4	1000	1.8910891	-	1.557894	1.7244919
Lrp1	Q91ZX7	348	VFFTDYGQIPK(*)VER(*)	K11	17.54	1000	0.8118811	-	1.105263	0.9585722
Scpep1	Q920A5	407	ALYTDPK(*)SSETAAFVK(*)	K7	63.2	114.67	1.3298969	0.8125	0.952941	1.0317794
Scpep1	Q920A5	255	EATQLWGK(*)AEMIIEK(*)	K8	42.19	63.15	-	-	0.882352941	
Supt16h	Q920B9	280	FK(*)SYC(+57.02)SNLVR(*)	K2	86.16	1000	1.03125	0.911392	0.989795	0.9774794
Supt16h	Q920B9	426	VK(*)NVGIFLK(*)	K2	67.73	170.44	0.8645833	0.962025	1.336734	1.0544478
Supt16h	Q920B9	781	TAFK(*)NFIEK(*)	K4	63.97	112.16	0.8125	1.164556	1.091836	1.0229646
Supt16h	Q920B9	904	YTEGVQSLNWTK(*)IMK(*)	K12	62.67	26.52	0.7708333	-	-	
Supt16h	Q920B9	366	EGSLVINSK(*)NQYK(*)	K9	60.72	55.36	0.8541666	1.012658	0.938775	0.9352001
Supt16h	Q920B9	696	GDK(*)VDILYNNIK(*)	K3	45.63	181.94	0.5208333	1.088607	0.755102	0.788181
Med28	Q920D3	131	DALVQK(*)HLTK(*)	K6	44.4	82.24	-	-	0.894117647	
Fdps	Q920E5	57	EVLEYNALGGK(*)YNR(*)	K11	47.18	1000	1.0212765	0.924050	0.533333	0.8262202
Msi2	Q920Q6	145	VEDAMLMFDK(*)TTNR(*)	K10	8.41	1000	1.7560975	-	-	
Ivns1abp	Q920Q8	480	GLK(*)NC(+57.02)DVFDPVTK(*)	K3	13.04	91.6	-	1.114035	-	
Bicd2	Q921C5	635	DQIK(*)HLQAAVDR(*)	K4	28.97	1000	0.8524590	1.052631	0.558823	0.8213047
Bicd2	Q921C5	351	LK(*)QQLVQMER(*)	K2	19.6	1000	0.9754098	0.614035	0.794117	0.7945209
Med6	Q921D4	241	NIQQTVSTK(*)GPPEK(*)R(*)	K14	70.43	1000	-	0.504424	0.659793	0.5821093

Med6	Q921D4	236	NIQQTVSTK(*)GPPEK(*)R(*)	K9	70.43	1000	-	0.504424	0.659793	0.5821093
Med6	Q921D4	227	ESTK(*)NIQQTVSTK(*)	K4	69.24	159.26	-	0.663716	0.907216	0.7854667
Tardbp	Q921F2	263	GISVHISNAEPK(*)HNSNR(*)	K12	76.68	1000	1.2	0.864864	1.011494	1.025453
Tardbp	Q921F2	160	FTEYETQVK(*)VMSQR(*)	K9	72.37	1000	-	-	1.724137931	
Tardbp	Q921F2	102	AVQK(*)TSDLIVLGLPWK(*)	K4	54.23	250.87	1.1684210	-	1.022988	1.0957048
Tardbp	Q921F2	145	SK(*)GFGFVR(*)	K2	52.19	1000	1.2842105	-	-	
Tardbp	Q921F2	145	TGHSK(*)GFGFVR(*)	K5	51.19	1000	1.0210526	1.121621	0.770114	0.9709297
Hnrnpll	Q921F4	544	ASAK(*)TLSGLLEWK(*)	K4	34.71	181.07	0.66	1.207792	-	0.9338961
Hnrnpll	Q921F4	555	C(+57.02)K(*)TDAVEALTALNHYQIR(	K2	20.09	1000	-	-	1.260416667	
Etfdh	Q921G7	343	WK(*)HHPSIQPTLEGGK(*)	K2	39.97	55	1.0219780	0.643835	-	0.8329068
Etfdh	Q921G7	152	FAILTEK(*)HR(*)	K7	34.29	1000	1.0879120	0.904109	0.690476	0.894166
Sh3bp4	Q921I6	481	VTVGLYGPK(*)HIHPSFK(*)	K9	27.64	29.06	0.5096525	0.646551	0.65	0.6020681
Cog2	Q921L5	57	LLK(*)TAMVELINK(*)	K3	56.07	199.32	1.2698412	-	1.154929	1.2123854
Sf3b3	Q921M3	294	GMIFVC(+57.02)SATHK(*)TK(*)	K11	65.97	24.24	0.8229166	0.734939	1.088888	0.8822484
Sf3b3	Q921M3	109	IHQETFGK(*)SGC(+57.02)R(*)	K8	65.52	1000	0.7916666	0.795180	0.966666	0.8511714
Sf3b3	Q921M3	26	AIHGNFSGTK(*)QQEIVVSR(*)	K10	59.87	1000	0.8854166	0.867469	1.066666	0.9398511
Sf3b3	Q921M3	984	C(+57.02)ENK(*)HIANYISGIQTIGHR(	K4	45.19	1000	0.9270833	0.590361	1.033333	0.8502594
Fam49b	Q921M7	308	YTTK(*)HLNDETTSK(*)	K4	24.05	178.24	1.2636363	1.256097	1.089887	1.2032072
Ddx27	Q921N6	511	TVINFTMPNTVK(*)HYVHR(*)	K12	18.91	1000	-	-	1.303370787	
Mrpl37	Q921S7	125	QALWLTK(*)TK(*)	K7	28.66	20.41	-	-	1.125	
Mrpl37	Q921S7	194	SQILK(*)HPSLAR(*)	K5	9.97	1000	-	-	1.3125	
Pdia5	Q921X9	458	DK(*)NQDLC(+57.02)QQEAVK(*)	K2	38.8	223.6	-	0.926470	2.439560	1.6830155
Mob1a	Q921Y0	149	NFMSVAK(*)TILK(*)	K7	63.09	48.12	-	-	0.454022989	
Dars	Q922B2	74	GK(*)QC(+57.02)FLVLR(*)	K2	82.68	1000	0.8571428	0.768292	1.043956	0.8897972
Dars	Q922B2	453	IK(*)AYIDSFR(*)	K2	54.65	1000	1.0306122	0.939024	1.186813	1.0521499
Dars	Q922B2	241	FK(*)NNAYLAQSPQLYK(*)	K2	36.57	189.17	0.7857142	-	1.175824	0.9807692
Ppp6r3	Q922D4	787	TETAVFK(*)SEEEK(*)	K7	38.33	48.54	0.8611111	0.922077	0.736842	0.8400104
Mthfd1	Q922D8	175	SK(*)IVGAPMHDLLLWNNATVTTC(+5	K2	52.65	199.48	0.9340659	-	0.977528	0.955797
Mthfd1	Q922D8	866	QGFGNLPIC(+57.02)MAK(*)THLSLS	K12	43.62	29.09	-	-	0.865168539	
Mthfd1	Q922D8	21	LK(*)NQVTR(*)	K2	36.32	1000	1.3296703	-	1.247191	1.2884307
Mthfd1	Q922D8	866	AK(*)THLSLSHNPEQK(*)	K2	15.9	109.74	-	-	1.292134831	
Pcyt2	Q922E4	158	MLLVTK(*)AHHSSQEMSSEYR(*)	K6	56.38	1000	-	-	0.557894737	
Pcyt2	Q922E4	327	VDLVC(+57.02)HGK(*)TEIVPDR(*)	K8	29.67	1000	0.4819277	1.411764	0.905263	0.9329852
Fastkd2	Q922E6	624	VAVLC(+57.02)VPK(*)SVYC(+57.02)	K8	4.49	1000	-	-	1.453781513	

Fam76a	Q922G2	281	EVTEQLQAK(*)NR(*)	K9	12.24	1000	-	0.932692	0.610738	0.7717153
Pdk3	Q922H2	374	LPVFNK(*)SAWR(*)	K6	40.69	1000	0.9626168	0.857142	0.666666	0.8288088
Pdk3	Q922H2	43	DNAC(+57.02)EK(*)TSYMFLR(*)	K6	28.55	1000	-	-	0.189189189	
Clip1	Q922J3	992	TK(*)HEEILQNLQK(*)	K2	39.24	228.69	-	1.083333	0.923076	1.0032051
Clip1	Q922J3	613	LK(*)TQIER(*)	K2	27.34	1000	1.1980198	-	0.912087	1.0550539
Nop2	Q922K7	408	LK(*)SVVGNLHR(*)	K2	54.09	1000	0.8653846	0.817073	0.956521	0.8796598
2-Mar	Q922Q1	66	VWIYPIK(*)SC(+57.02)K(*)	K7	40.39	40.63	0.8928571	0.811594	0.742268	0.8155731
Riok1	Q922Q2	210	IYK(*)TSILMFK(*)	K3	8.18	118.11	0.5263157	0.567164	0.753246	0.6155756
Lrrc59	Q922Q8	73	NK(*)LQQLPADFGR(*)	K2	77.97	1000	0.7156862	0.740740	0.988636	0.8150211
Lrrc59	Q922Q8	143	QC(+57.02)ANK(*)VLQHMK(*)	K5	60.74	87.26	0.7450980	0.839506	1	0.8615347
Ppp4r3b	Q922R5	777	SVVSQTTPASSNVASSK(*)TTSLATSVT	K17	2.9	64.8	0.0344827	0.253623	-	0.144053
Pdia6	Q922R8	85	VGAVNADK(*)HQSLGGQYGVQGFPT	K8	91.67	198.31	1.0510204	0.8125	0.822222	0.8952475
Pdia6	Q922R8	245	IFQK(*)GESPVDYDGGR(*)	K4	70.28	1000	0.9897959	0.8625	1.244444	1.0322468
Pdia6	Q922R8	368	MK(*)FALLK(*)	K2	64.65	111.23	-	-	0.9	
Pdia6	Q922R8	241	GFPTIK(*)IFQK(*)	K6	33.3	55.36	0.7551020	0.8625	0.788888	0.8021636
Pdia6	Q922R8	235	YGIK(*)GFPTIK(*)	K4	10.66	58.16	-	-	1.433333333	
Prpf3	Q922U1	495	VLGTEAVQDPTK(*)VEAHVR(*)	K12	50.23	1000	0.9896907	0.769230	1.382022	1.0469813
Plrg1	Q922V4	362	LWDLVAGK(*)TR(*)	K8	34.6	1000	-	9.155844	1.590361	5.3731028
Plrg1	Q922V4	7	M(+42.01)VEEVQK(*)HSVHTLVFR(*)	K7	29.64	1000	1.3571428	-	0.783132	1.0701377
Plrg1	Q922V4	319	TK(*)ASVHTLSGHTNAVATVR(*)	K2	27.8	1000	-	1.792207	1.289156	1.5406822
Prmt7	Q922X9	121	VINK(*)HSTEVTVGPDGDLPC(+57.02	K4	35.98	1000	0.9387755	0.137931	0.718446	0.5983844
Dbr1	Q923B1	128	IGGISGIFK(*)SHDYR(*)	K9	57.73	1000	0.8224299	0.930555	0.817307	0.8567644
Blvrb	Q923D2	178	VISK(*)HDLGHFMLR(*)	K4	69.36	1000	0.5833333	0.743589	1.578947	0.9686235
Wbp11	Q923D5	316	FADMPGK(*)SR(*)	K7	72.11	1000	0.9636363	0.63	0.902173	0.8319368
Wbp11	Q923D5	545	PK(*)ADDASAATIEK(*)	K2	48.33	122.98	-	-	1.304347826	
Wbp11	Q923D5	13	STSSTK(*)SGK(*)FMNPTDQAR(*)	K9	20.58	1000	-	0.74	-	
Wbp11	Q923D5	10	STSSTK(*)SGK(*)FMNPTDQAR(*)	K6	20.58	1000	-	0.74	-	
Wbp11	Q923D5	13	SGK(*)FMNPTDQAR(*)	K3	15.57	1000	-	0.47	0.945652	0.7078261
Rab30	Q923S9	22	IVLIGNAGVGK(*)TC(+57.02)LVR(*)	K11	37.16	1000	0.9166666	-	2.1875	1.5520833
Camk2g	Q923T9	268	ITADQALK(*)HPWVC(+57.02)QR(*)	K8	58.54	1000	0.8636363	0.721518	1.133333	0.9061629
Xpo5	Q924C1	413	MGFPSK(*)TDSPSC(+57.02)EYSR(*)	K6	35.1	1000	0.8421052	-	-	
Xpo5	Q924C1	57	LAEK(*)TQIAIVR(*)	K4	30.64	1000	0.6105263	0.891566	0.944444	0.8155123
Wac	Q924H7	302	LPTPTASLPAQK(*)TER(*)	K12	35.81	1000	-	0.591836	-	
Mta3	Q924K8	287	YGK(*)DFNDIR(*)	K3	42.59	1000	-	-	1.300970874	

Mta3	Q924K8	474	TTYFTK(*)IAR(*)	K6	9.85	1000	-	-	1.184466019	
Smc6	Q924W5	356	APEC(+57.02)MALK(*)TDVIAR(*)	K8	25.66	1000	-	0.430769	-	
Smc6	Q924W5	934	YK(*)TYQQFR(*)	K2	23.15	1000	-	-	1.765957447	
Pawr	Q925B0	220	YK(*)STTSAPEDEISNR(*)	K2	18.79	1000	1.8541666	-	-	
Atad3	Q925I1	182	HK(*)NEMLR(*)	K2	51.27	1000	1.2470588	-	0.835051!	1.0410552
Med1	Q925J9	1154	NSSQTGGK(*)PGSSPITK(*)	K8	50.28	99.23	0.9	1.050847	0.921348:	0.9573986
Med1	Q925J9	1076	GTVMVGK(*)PSSHSQYTSSGSVSSSGS	K7	45.89	119.08	1.225	1.254237	1.752808!	1.4106821
Taldo1	Q93092	219	SVTK(*)IYNYYK(*)	K4	90.19	121.92	1.0543478	1.013513	1.095238(	1.0543665
Taldo1	Q93092	230	FGYK(*)TIVMGASFR(*)	K4	47.26	1000	-	0.905405	0.976190	0.9407979
Taldo1	Q93092	81	LGGPQEEQIK(*)NAIDK(*)	K10	42.11	46.62	1.7608695	1.270270	1.035714:	1.355618
Taldo1	Q93092	215	SYEPQEDPGVK(*)SVTK(*)	K11	28.84	34.3	0.5108695	-	1.059523!	0.7851967
Hnrnpab	Q99020	271	GSGGGQGSTNYGK(*)SQR(*)	K13	90.17	1000	1.1836734	1.0125	1.066666(	1.0876134
Myo5a	Q99104	551	AFIIK(*)HFADK(*)	K5	34.02	61.26	-	0.884615	0.886597!	0.8856067
Myo5a	Q99104	405	DALAK(*)HIYAK(*)	K5	20.44	95.62	-	2.051282	-	
Myo5a	Q99104	1487	LVK(*)NLILELK(*)PR(*)	K3	17.04	125.04	0.9550561	-	-	
Myo5a	Q99104	556	HFADK(*)VEYQC(+57.02)EGFLEK(*)	K5	9.52	99.02	2.3707865	-	-	
Wdr77	Q99J09	121	FC(+57.02)K(*)YEHDDIVSTVTVLSSG	K3	43.77	196.93	-	-	0.99	
Thumpd1	Q99J36	19	GK(*)SQFLPAK(*)	K2	93.82	186.64	1.1298701	0.746268	0.923076!	0.9330719
Thumpd1	Q99J36	201	GTFQIVYK(*)SR(*)	K8	50.66	1000	1.7142857	-	1.371794!	1.5430403
Nrbp1	Q99J45	231	IGSVAPDTINNHVK(*)TC(+57.02)R(*	K14	26.17	1000	-	-	0.897727273	
Rfc4	Q99J62	124	VK(*)NFAQLTVSGSR(*)	K2	53.99	1000	0.9651162	0.739726	0.804347!	0.8363967
Nans	Q99J77	243	HITLDK(*)TWK(*)	K6	76.69	62.26	0.9782608	0.729411	0.921348:	0.8763403
Nans	Q99J77	55	FQK(*)SELEFK(*)	K3	66.06	112.31	-	0.764705	1.044943!	0.9048249
Nans	Q99J77	16	WVGGK(*)HPC(+57.02)FIIAEIGQNH	K5	47.58	67.42	-	-	0.91011236	
Cdk9	Q99J95	178	AFSLAK(*)NSQPNR(*)	K6	37.69	1000	1.3333333	-	1.405797:	1.3695652
Psip1	Q99JF8	405	FK(*)VSQVIMEK(*)	K2	63.45	161.78	0.8936170	0.905405	1.035714:	0.9449122
Psip1	Q99JF8	413	VSQVIMEK(*)STMLYNK(*)	K8	63.2	110.43	1.3936170	1.054054	-	1.2238355
Psip1	Q99JF8	16	MK(*)GYPHWPAR(*)	K2	63.02	1000	1.0319148	1.040540	1.035714:	1.0360566
Psip1	Q99JF8	358	IHAEIK(*)NSLK(*)	K6	61.47	88.98	1.3510638	1.378378	1.226190	1.3185442
Kdelr1	Q99JH8	83	FK(*)ATYDGNHDTFR(*)	K2	10.4	1000	-	-	0.842975207	
Psmd6	Q99JI4	130	TYDK(*)TVALGHR(*)	K4	83.74	1000	-	0.776315	0.978260!	0.8772883
Psmd6	Q99JI4	349	IDK(*)VNEIVETNR(*)PDSK(*)	K3	80.83	152.87	0.7741935	0.736842	0.902173!	0.8044032
Psmd6	Q99JI4	165	AK(*)SLIEEGGDWDR(*)	K2	54.42	1000	1.3010752	2.447368	1.260869!	1.6697711
Psmd6	Q99JI4	362	VNEIVETNR(*)PDSK(*)NWQYQETIK(	K13	23.07	46.57	1.9892473	-	0.967391:	1.4783193

Psmd6	Q99JI4	181	LK(*)VYQGLYC(+57.02)VAIR(*)	K2	18.51	1000	-	-	1.076086957	
Rap1b	Q99JI6	128	VVGK(*)EQGQNLAR(*)	K4	6	1000	-	-	1.741573034	
Cdc26	Q99JP4	68	IGYK(*)PQLK(*)	K4	12.74	41.04	-	1.132653	0.924050(	1.0283518
Lims1	Q99JW4	210	VVNAMGK(*)QWHVEHFVC(+57.02)	K7	64.94	71.53	0.7684210	0.917808	1.011235!	0.8991551
Eif3m	Q99JX4	324	MVYC(+57.02)K(*)IDQTQR(*)	K5	75.86	1000	0.7666666	-	1.010869!	0.8887681
Nxf1	Q99JX7	383	GSYFGTENLK(*)SLVLR(*)	K10	40.1	1000	0.6875	-	-	
Nxf1	Q99JX7	303	TLNLSGNELK(*)TER(*)	K10	24.9	1000	-	1.356321	0.906976	1.1316493
Nxf1	Q99JX7	6	A(+42.01)DEGK(*)SYNEHDDR(*)	K5	16.53	1000	-	0.609195	-	
Hadhb	Q99JY0	53	MK(*)NIVVVEGVR(*)	K2	44.27	1000	0.8454545	-	0.860215(	0.8528348
Plpp3	Q99JY8	282	TK(*)TSLSLPAPAIR(*)	K2	14.71	1000	-	2.1	0.794392!	1.4471963
Actr3	Q99JY9	317	R(*)PLYK(*)NIVLSGGSTMFR(*)	K5	67.58	1000	0.8019801	0.974683	0.944444	0.9070361
Arfgap2	Q99K28	7	A(+42.01)ASPSK(*)TEIQTIFK(*)	K6	25.3	82.59	1.0442477	-	0.766355	0.9053015
Nono	Q99K48	200	GIVEFSGK(*)PAAR(*)	K8	93.72	1000	0.9010989	0.92	0.898876	0.9066584
Nono	Q99K48	245	LVIK(*)NQQFHK(*)	K4	84.88	151.86	1.0989010	1.026666	0.955056	1.0268746
Nono	Q99K48	192	GR(*)PSGK(*)GIVEFSGK(*)	K6	83.48	131.72	1.0109890	0.426666	0.471910	0.6365219
Nono	Q99K48	111	DK(*)GFGFIR(*)	K2	79.82	1000	1.0989010	0.92	1	1.0063004
Nono	Q99K48	101	YGK(*)AGEVFIHK(*)	K3	69.38	242.89	1.0219780	1.053333	0.955056	1.0101225
Nono	Q99K48	128	TLAEIAK(*)VELDNMPLR(*)	K7	61.67	1000	-	-	1.101123596	
Nono	Q99K48	5	M(+42.01)QSNK(*)AFNLEK(*)	K5	53.03	87.26	1.0329670	0.68	0.696629	0.8031987
Nono	Q99K48	373	QQEGFK(*)GTFPDAR(*)	K6	52.29	1000	-	-	1.269662921	
Pls3	Q99K51	537	TLSEAGK(*)STSIQSFK(*)	K7	72.93	131.72	0.9468085	0.611111	1.2	0.9193065
Pls3	Q99K51	52	EANMPLPGYK(*)VR(*)	K10	69.37	1000	-	0.666666	0.894117(	0.7803922
Pls3	Q99K51	91	SSDIAK(*)TFR(*)	K6	39.66	1000	-	-	1.988235294	
Pls3	Q99K51	437	IK(*)VPVDWSK(*)	K2	38.16	79.54	0.7446808	-	-	
Pls3	Q99K51	85	VYIFQEVK(*)SSDIAK(*)	K8	11.18	30.1	-	-	0.729411765	
Pls3	Q99K51	300	DSK(*)AYFHLLNQIAPK(*)	K3	3.72	40.43	-	-	1.741176471	
Psat1	Q99K85	5	M(+42.01)EATK(*)QVVNFGPGPAK(*	K5	83.61	128.75	1.0212765	0.896103	1.033707	0.9836961
Psat1	Q99K85	333	AVELNMISLK(*)GHR(*)	K10	71.64	1000	1.2765957	1.194805	1.202247	1.2245494
Psat1	Q99K85	323	FLDK(*)AVELNMISLK(*)	K4	58.66	207.31	-	-	0.831460674	
Psat1	Q99K85	200	AGAQK(*)NVGSAGVTVVIVR(*)	K5	46.74	1000	1.2872340	-	1.089887(	1.1885608
Psat1	Q99K85	333	MISLK(*)GHR(*)	K5	35.66	1000	1.3297872	-	1.191011	1.2603992
Vwa5a	Q99KC8	551	LAAK(*)SLIQTK(*)	K4	13.77	45.03	-	0.931506	-	
Unc45a	Q99KD5	653	VK(*)TESPVLTNSC(+57.02)R(*)	K2	32.27	1000	1.9285714	1.203125	-	1.5658482
Rbm10	Q99KG3	920	ETLHK(*)TMVTR(*)	K5	49.53	1000	0.8505747	0.723076	0.758241	0.7772978

Rbm10	Q99KG3	670	ENFK(*)NSFQPISALR(*)	K4	28.89	1000	-	-	0.879120879	
Stk24	Q99KH8	26	ADPEELFTK(*)LEK(*)	K9	51.68	49.37	-	1.181818	0.96875	1.0752841
Aco2	Q99KI0	689	AIITK(*)SFAR(*)	K5	94.41	1000	0.7979797	0.792207	0.869565	0.8199176
Aco2	Q99KI0	50	YDLLEK(*)NINIVR(*)	K6	83.46	1000	0.6464646	0.636363	0.739130	0.6739862
Aco2	Q99KI0	411	C(+57.02)K(*)SQFTITPGSEQIR(*)	K2	70.62	1000	0.5454545	-	-	
Aco2	Q99KI0	31	AK(*)VAMSHFEPSEYIR(*)	K2	27.34	1000	1.1818181	0.766233	-	0.974026
Dctn2	Q99KJ8	290	LQSVLGK(*)VNEIAK(*)	K7	69.46	102.92	0.8461538	0.831168	0.933333	0.8702187
Dctn2	Q99KJ8	65	VGTK(*)GLDFSDR(*)	K4	66.5	1000	-	1.103896	1.155555	1.1297258
Dctn2	Q99KJ8	309	ASVEDADTQNK(*)VHQLYETIQR(*)	K11	56.56	1000	1.0549450	1.363636	1.044444	1.154342
Dctn2	Q99KJ8	185	LLLQLEATK(*)SSK(*)	K9	49.77	50.22	0.8791208	1.246753	0.9	1.0086247
Dctn2	Q99KJ8	146	QLAALK(*)QQLVASHLEK(*)	K6	42.41	83.29	0.7692307	1.259740	0.866666	0.9652126
Dctn2	Q99KJ8	140	LTPVVLAK(*)QLAALK(*)	K8	19.15	38	-	-	1.322222222	
Dpp3	Q99KK7	629	LQVLK(*)STGDVVAGR(*)	K5	81.11	1000	0.8686868	0.844155	1	0.9042809
Dpp3	Q99KK7	209	VVGQEGK(*)SHYEVR(*)	K7	59.65	1000	0.9797979	1.129870	1.010869	1.0401792
Dpp3	Q99KK7	131	VILGSK(*)AAQQR(*)PEEVR(*)	K6	6.62	1000	-	-	1.326086957	
Dpp3	Q99KK7	202	LFK(*)VVGQEGK(*)	K3	4.3	35.03	-	-	0.369565217	
Hars2	Q99KK9	443	AEMLYK(*)NNPK(*)	K6	27.08	77.13	-	-	21.11702128	
Ciao1	Q99KN2	53	IWGTEGDSWIC(+57.02)K(*)SVLSEG	K12	34.89	1000	-	-	1.376470588	
Clint1	Q99KN9	268	GEFK(*)DEEETVTTK(*)HIHITQATETTT	K13	64.8	45.63	0.8947368	-	1.147727	1.0212321
Clint1	Q99KN9	291	TANPSK(*)TIDLGAAAHYTGDK(*)	K6	44.31	55.05	0.6736842	0.810810	1.090909	0.858468
Clint1	Q99KN9	200	NK(*)SAFPFSDK(*)	K2	27.38	53.45	2.7368421	-	-	
Clint1	Q99KN9	473	SQNTDMVQK(*)SASK(*)	K9	24.43	77.51	1.2	0.945945	4.193181	2.1130426
Prpf19	Q99KP6	179	LQDK(*)ATVLTTER(*)	K4	76.46	1000	0.9042553	0.893333	0.988095	0.9285613
Prpf19	Q99KP6	428	NFK(*)TLQLDNNFEVK(*)	K3	67.9	170.43	0.9042553	0.84	0.988095	0.9107835
Nampt	Q99KQ4	469	VTK(*)SYSFDEVR(*)	K3	58.82	1000	0.9340659	0.641025	1.081395	0.8854956
Lactb2	Q99KR3	191	IK(*)ANIIYPGHGPVIHNAEAK(*)	K2	13.9	129.03	0.7816091	0.810810	-	0.79621
Lactb2	Q99KR3	255	DVPENLHK(*)MAEHNLLLHLR(*)	K8	12.18	1000	-	-	1.423076923	
Ppif	Q99KR7	166	TDWLDGK(*)HVVFGHVK(*)	K7	75.36	71.07	1.0731707	0.829545	0.897727	0.9334812
Ppif	Q99KR7	72	ADVVPK(*)TAENFR(*)	K6	65.75	1000	1.7804878	1.125	1.284090	1.3965262
Ppif	Q99KR7	90	GFGYK(*)GSTFHR(*)	K5	39.36	1000	-	-	0.75	
Gak	Q99KY4	443	MFLDAK(*)HPGHYAVYNLSPR(*)	K6	23.72	1000	-	-	0.094339623	
Pagr1a	Q99L02	202	LDK(*)VLSDMK(*)	K3	40.12	129.72	-	-	-	
St13	Q99L47	362	VMNLISK(*)LSAK(*)	K7	48.48	55.21	1.1808510	0.827160	1.032967	1.0136595
St13	Q99L47	355	YQSNPK(*)VMNLISK(*)	K6	38.61	110.43	1.3085106	0.888888	-	1.0986998

St13	Q99L47	50	AK(*)SEENTK(*)EEK(*)	K2	23.46	78.19	-	0.987654	-	
St13	Q99L47	152	LAILYAK(*)R(*)	K7	15.38	1000	1.1489361	0.839506	-	0.9942212
Nmd3	Q99L48	39	SK(*)VDISQGVPK(*)	K2	14.72	143.22	1.4404761	-	1.21875	1.3296131
Mcrs1	Q99L90	68	K(*)FDDELVESSLAK(*)SSTR(*)	K13	32.41	126.66	-	-	-	
Mcrs1	Q99L90	68	FDDELVESSLAK(*)SSTR(*)	K12	11.55	1000	-	-	-	
Mat2b	Q99LB6	209	VQFSNK(*)SANMDHWQQR(*)	K6	55.12	1000	1.0104166	0.723684	0.881720	0.8719404
Etfa	Q99LC5	206	LTK(*)SDR(*)PELTGAK(*)	K3	15.51	118.06	-	1.142857	1.287356	1.2151067
Eif2b1	Q99LC8	110	NK(*)IANLC(+57.02)HTFIK(*)	K2	28.13	82.84	-	-	1.270833333	
Gps1	Q99LD4	418	STTFEK(*)SLLMGK(*)	K6	45.47	95.51	-	1.054054	1.034090	1.0440725
Gps1	Q99LD4	447	NQIHVK(*)SPPR(*)	K6	29.09	1000	-	-	2.386363636	
Gps1	Q99LD4	402	IDSHSK(*)ILYAR(*)	K6	12.58	1000	-	-	1.727272727	
Abcf2	Q99LE6	320	YYTGNYDQYVK(*)TR(*)	K11	53.6	1000	0.9468085	0.666666	-	0.8067376
Abcf2	Q99LE6	344	MK(*)NYIAR(*)	K2	53.42	1000	1	-	0.978723	0.9893617
Abcf2	Q99LE6	613	SK(*)LVDEEPQLTK(*)	K2	18.63	119.76	0.5212765	-	0.223404	0.3723404
Rtcb	Q99LF4	17	INK(*)NC(+57.02)WR(*)	K3	35.83	1000	1.1134020	0.986666	-	1.0500344
Rtcb	Q99LF4	366	VEQHVVDGK(*)ER(*)	K9	34.84	1000	-	-	1.25	
Rtcb	Q99LF4	357	IAK(*)VEQHVVDGK(*)	K3	34.38	203.33	-	1.346666	0.909090	1.1278788
Rtcb	Q99LF4	251	MGIDHK(*)GQVC(+57.02)VMIHSGS	K6	24.76	1000	1	0.8	1.022727	0.9409091
Tnpo2	Q99LG2	182	FLQFFK(*)HC(+57.02)SPK(*)	K6	54.72	79.02	0.8172043	-	0.864583	0.8408938
Tnpo2	Q99LG2	852	DMFYK(*)ILHGFK(*)	K5	13.16	71.75	-	-	1.697916667	
Gnl2	Q99LH1	55	GK(*)VIK(*)PLQYQSTVASGTVAR(*)	K2	27.48	47.36	1.5833333	1.797101	-	1.6902174
Hgs	Q99LI8	422	MK(*)SNHMR(*)	K2	28.57	1000	-	-	1.445454545	
Clp1	Q99LI9	127	VMVVGPTDVGK(*)STVC(+57.02)R(*	K11	42.77	1000	0.1527377	-	-	
Grpel1	Q99LP6	157	VFTK(*)HGLLR(*)	K4	78.91	1000	1.1590909	0.891566	1.034090	1.0282494
Grpel1	Q99LP6	196	EPGTVALVSK(*)VGYK(*)	K10	26.31	63.97	1.2045454	0.987951	0.943181	1.0452264
Dpy30	Q99LT0	92	NK(*)AQFEDR(*)	K2	51.4	1000	0.6907216	-	1.043010	0.8668662
Chmp1b1	Q99LU0	59	QK(*)NQGVNFLR(*)	K2	53.03	1000	0.5906735	0.948717	0.893129	0.8108404
Gins4	Q99LZ3	142	EYMDHTETHFK(*)NVALK(*)	K11	57.36	38.32	1.0105263	-	-	
Lias	Q99M04	360	AGEFFLK(*)NLVAR(*)	K7	8.17	1000	-	-	1.363636364	
Rnps1	Q99M28	203	MHPHLSK(*)GYAYVEFENPDEAEK(*)	K7	36.38	145.13	-	-	1.851351351	
Rnps1	Q99M28	221	ALK(*)HMDGGQIDGQEITATAVLAPW	K3	5.57	1000	-	-	1.878378378	
Hspa14	Q99M31	248	LFK(*)HDVR(*)	K3	68.05	1000	1.0421052	0.764705	1.088888	0.9652333
Nck1	Q99M51	47	NSMNK(*)TGFVPSNYVER(*)	K5	7.22	1000	-	-	1.034883721	
Nasp	Q99MD9	241	GK(*)SISGAYVQNK(*)	K2	94.56	252.77	1.1931818	0.884615	0.870967	0.9829216

Nasp	Q99MD9	239	TEQESLC(+57.02)TEK(*)GK(*)	K10	33.57	22.85	1.1931818	1.307692	0.870967	1.1239473
Wdr6	Q99ME2	96	VVK(*)VSWGQSHLR(*)	K3	18.11	1000	-	-	1.315068493	
Gtpbp4	Q99ME9	36	TPTVIHK(*)HYQIHR(*)	K7	72.1	1000	0.9255319	0.768292	1.120481	0.9381022
Gtpbp4	Q99ME9	352	GNK(*)VNEVLNR(*)	K3	56.29	1000	0.4574468	-	0.939759	0.6986029
Gtpbp4	Q99ME9	203	TTK(*)SLFVGHMDYK(*)	K3	40.33	189.31	0.7127659	0.634146	1.156626	0.8345129
Gtpbp4	Q99ME9	36	K(*)TPTVIHK(*)HYQIHR(*)	K8	35.3	79.54	0.6808510	-	0.975903	0.8283773
Erc1	Q99MI1	439	NK(*)VEQLK(*)EELSSK(*)	K2	45.73	139.79	0.6228070	-	0.688679	0.6557431
Erc1	Q99MI1	319	LLEMLQSK(*)GLSAK(*)	K8	29.76	70.86	-	1.151515	0.735849	0.9436821
Kars1	Q99MN1	162	NYK(*)SEEEFVHINNK(*)	K3	67.2	195.68	0.9270833	0.867469	0.978494	0.9243493
Kars1	Q99MN1	139	ASGGK(*)LIFYDLR(*)	K5	58.67	1000	1.0104166	0.927710	1	0.9793758
Kars1	Q99MN1	361	MLSGMVK(*)SITGSYK(*)	K7	58.65	127.53	0.9479166	0.867469	1.096774	0.9707202
Kars1	Q99MN1	173	SEEEFVHINNK(*)LR(*)	K11	34.56	1000	-	-	0.559139785	
Kars1	Q99MN1	19	LSK(*)NELK(*)	K3	20.26	88.98	-	0.987951	0.967741	0.9778469
Srrt	Q99MR6	734	HIFNK(*)HAEK(*)	K5	52.2	76.73	1	0.932432	1.172413	1.0349487
Srrt	Q99MR6	418	PLHK(*)TC(+57.02)SLFMR(*)	K4	49.4	1000	0.5384615	0.608108	1.137931	0.7615002
Srrt	Q99MR6	610	VLDK(*)LLLYLR(*)	K4	9.81	1000	-	-	1.896551724	
Adar	Q99MU3	885	YNHHTAK(*)NSIFELAR(*)	K7	38.11	1000	-	-	-	
Raf1	Q99N57	462	QTAQGMDYLHAK(*)NIIHR(*)	K12	25.94	1000	-	-	1.191176471	
Mrps18b	Q99N84	97	NNK(*)VAGNPC(+57.02)PIC(+57.02	K3	26.06	1000	1.0471698	-	1.493975	1.2705729
Mrps18a	Q99N85	45	IQEGK(*)TTVIEGR(*)	K5	30.11	1000	-	0.464566	1.524390	0.9944786
Mrps18a	Q99N85	171	WNK(*)VGMAVGSPLLK(*)	K3	28.24	81.27	-	-	0.280487805	
Mrpl27	Q99N92	128	GAVLYK(*)TFVHVVPAK(*)PEGTFK(*)	K6	37.52	43	1.1445783	-	0.204301	0.6744397
Mrpl9	Q99N94	192	HFFK(*)NLGVVVAPHALR(*)	K4	39.25	1000	-	-	0.946428571	
Mrpl9	Q99N94	246	YK(*)HWLAQQAAK(*)	K2	31.36	159.26	1.6129032	-	0.839285	1.2260945
Mrpl9	Q99N94	58	WWK(*)VPLAGEGR(*)	K3	25.91	1000	0.4731182	-	0.830357	0.6517377
Mrpl3	Q99N95	116	DGQK(*)HAVTLLQVQDC(+57.02)HV	K4	20.13	88.91	-	1.179775	-	
Mrpl3	Q99N95	150	IAALTVGGK(*)TVSR(*)	K9	15.01	1000	-	0.662921	0.688073	0.6754974
Sf3b1	Q99NB9	1025	LTPILK(*)NR(*)	K6	80.61	1000	1.0869565	0.946666	1	1.0112077
Sf3b1	Q99NB9	6	IAK(*)THEDIEAQIR(*)	K3	74.5	1000	1.0869565	0.893333	0.887640	0.9559768
Sf3b1	Q99NB9	816	TEILPPFFK(*)HFWQHR(*)	K9	58.09	1000	1.0869565	-	0.977528	1.0322423
Sf3b1	Q99NB9	926	VK(*)PYLPQIC(+57.02)GTVLWR(*)	K2	42.02	1000	-	-	1.157303371	
Sf3b1	Q99NB9	741	GK(*)GLAAFLK(*)	K2	31.51	138.97	0.9782608	1.44	1.101123	1.1731282
Sf3b1	Q99NB9	252	GSETPGATPGSK(*)IWDPTPSHTPAG	K12	29.42	1000	1.3913043	0.8	-	1.0956522
Sf3b1	Q99NB9	554	HLLVK(*)VIDR(*)	K5	13.27	1000	-	0.853333	0.820224	0.836779

Sf3b1	Q99NB9	298	WDETPK(*)TER(*)	K6	10.45	1000	-	1.773333	0.898876	1.3361049
Hspbp1	Q99P31	92	EEVEQMK(*)NC(+57.02)LR(*)	K7	63.71	1000	0.8217821	0.7	1.052083	0.8579552
Hspbp1	Q99P31	247	VK(*)SAFLLQNLLVGHPEHK(*)	K2	50.9	273.37	0.7326732	0.633333	0.791666	0.7192244
Hspbp1	Q99P31	205	VK(*)ALFAISC(+57.02)LVR(*)	K2	17.75	1000	-	-	1.083333333	
Nuf2	Q99P69	402	IK(*)SAIQQLR(*)	K2	27.18	1000	-	-	1.130434783	
Rtn4	Q99P72	1036	GVIQAIQK(*)SDEGHPFR(*)	K8	80.63	1000	1.0109890	0.866666	1.056818	0.978158
Rtn4	Q99P72	760	ESLTEVSETVTQHK(*)HK(*)	K14	60.55	20.41	1	0.773333	-	0.8866667
Rtn4	Q99P72	1028	IYK(*)GVIQAIQK(*)	K3	49.53	150.63	-	-	0.886363636	
Rtn4	Q99P72	314	DK(*)EDLVC(+57.02)SAALHNPQESP	K2	46.61	93.92	-	-	0.738636364	
Rtn4	Q99P72	910	DEAHVSDEFSK(*)SR(*)	K11	20.97	1000	0.7472527	2.733333	1.329545	1.6033772
Nup155	Q99P88	1100	VLSK(*)LADMHSTEISLQQR(*)	K4	28.8	1000	0.6105263	1.513513	-	1.0620199
Rrbp1	Q99PL5	1368	VGAAEEELHK(*)SR(*)	K10	78.52	1000	0.0588235	-	-	
Rrbp1	Q99PL5	1135	SK(*)C(+57.02)EELSSLHGQLK(*)	K2	62.35	243.69	0.9411764	0.934210	1.043956	0.9731143
Rrbp1	Q99PL5	1406	EHTSHLEAELEK(*)HMAAASAEC(+57.	K12	49.39	121.4	1.4313725	-	0.890109	1.1607412
Rrbp1	Q99PL5	1570	LQELLK(*)TTQEQLTK(*)	K6	37.63	66.64	-	-	0.846153846	
Rrbp1	Q99PL5	1374	VTVK(*)HLEDIVEK(*)	K4	29.54	154.74	1.0098039	1.171052	0.989010	1.0566225
Rrbp1	Q99PL5	1486	LK(*)TQLER(*)	K2	27.35	1000	1.1862745	-	0.912087	1.0491812
Rrbp1	Q99PL5	890	TGVIQDTWHK(*)ATQK(*)	K10	10.4	25.11	1.1764705	-	2.164835	1.6706529
Ubxn6	Q99PL6	202	LQNK(*)VFQER(*)	K4	29.76	1000	0.4595744	0.229166	1.3	0.6629137
Gtf2a1	Q99PM3	49	TLWENK(*)LMQSR(*)	K6	27.94	1000	-	-	0.452631579	
Trim33	Q99PP7	778	TAEK(*)SAHSFK(*)	K4	92.22	151.86	0.6571428	0.925	0.71875	0.7669643
Trim33	Q99PP7	968	GK(*)TAQGLSPVDQR(*)	K2	79.26	1000	0.6142857	0.9125	0.671875	0.7328869
Trim33	Q99PP7	784	SAHSFK(*)SDQVK(*)	K6	37.18	95.62	0.4	0.5375	0.484375	0.4739583
Arhgdia	Q99PT1	52	YK(*)EALLGR(*)	K2	93.31	1000	1.0416666	1.051948	1.151162	1.0815925
Arhgdia	Q99PT1	141	IDK(*)TDYM(+15.99)VGSYGPR(*)	K3	83.3	1000	0.9270833	1.077922	0.965116	0.9900406
Dhx30	Q99PU8	785	TK(*)VSC(+57.02)LETVWVSR(*)	K2	15.97	1000	1.0254237	1	0.990654	1.0053593
Prpf8	Q99PV0	609	DLK(*)HLIYYR(*)	K3	82.79	1000	0.8958333	1.025641	1.153846	1.0251068
Prpf8	Q99PV0	746	AK(*)ADWWTNTAHYNR(*)	K2	81.47	1000	0.7916666	0.935897	0.912087	0.879884
Prpf8	Q99PV0	833	FSPIPFPPLSYK(*)HDTK(*)	K12	73.9	34.3	0.6770833	0.615384	0.802197	0.6982219
Prpf8	Q99PV0	1158	AVFWDIK(*)NR(*)	K7	69.2	1000	0.7291666	0.692307	1.032967	0.8181471
Prpf8	Q99PV0	1144	LMK(*)HDVNLGR(*)	K3	68.78	1000	1.0208333	1.153846	1.241758	1.1388126
Prpf8	Q99PV0	2049	TVNK(*)HGDEIITSTTSNYETQTFSSK(*	K4	60.11	189.27	1.1875	1.551282	0.934065	1.2242827
Prpf8	Q99PV0	1344	WSK(*)QTDVGITHFR(*)	K3	53.72	1000	0.8125	1.153846	1.043956	1.0034341
Prpf8	Q99PV0	1792	VTIHK(*)TFEGNLTTK(*)	K5	52.94	135.47	0.75	0.897435	0.813186	0.8202076

Prpf8	Q99PV0	428	ALDIPLVK(*)NWYR(*)	K8	49.65	1000	0.7395833	1.461538	0.967032	1.0560516
Prpf8	Q99PV0	1840	WK(*)TAEEVAALIR(*)	K2	49.38	1000	0.8645833	1.038461	0.879120	0.9273886
Prpf8	Q99PV0	555	LTK(*)LVVDSHVQYR(*)	K3	48.58	1000	0.7916666	0.615384	0.813186	0.7400794
Prpf8	Q99PV0	1434	HTLAYDK(*)GWR(*)	K7	42.71	1000	0.7291666	-	1.153846	0.9415064
Prpf8	Q99PV0	1449	QYQVLK(*)QNPFWWTHQR(*)	K6	39.98	1000	-	-	1	
Prpf8	Q99PV0	460	ALK(*)HR(*)PPK(*)	K3	14.5	79.02	0.7604166	-	-	
Prpf8	Q99PV0	853	EAYSVK(*)SR(*)	K6	7.57	1000	1.6666666	1.371794	1.307692	1.4487179
Ndufa5	Q9CPP6	36	TLDILK(*)HFPK(*)	K6	69.22	87.62	0.9090909	0.694444	0.857142	0.8202261
Atp5mg	Q9CPQ8	61	IIQSAK(*)TGSFK(*)	K6	91.88	100.21	0.9368421	1	1.042553	0.9931318
Atp5mg	Q9CPQ8	66	TGSFK(*)HLTVK(*)	K5	90.74	127.18	0.9894736	0.857142	0.925531	0.9240495
Rwdd4	Q9CPR1	177	GWNWVDVVK(*)HLSK(*)	K9	36.5	48.12	-	-	0.818181818	
Rpl17	Q9CPR4	96	MLK(*)NAESNAELK(*)	K3	95.84	166.66	0.9411764	0.864197	0.967391	0.9242551
Rpl17	Q9CPR4	13	YSLDPENPTK(*)SC(+57.02)K(*)	K10	92.15	29.32	0.7352941	0.740740	0.880434	0.7854899
Rpl17	Q9CPR4	27	VHFK(*)NTR(*)	K4	78.54	1000	0.9705882	0.925925	1	0.9655047
Rpl17	Q9CPR4	74	C(+57.02)AQAK(*)QWGWTQGR(*)	K5	66.71	1000	0.7745098	0.938271	0.956521	0.8897677
Rpl17	Q9CPR4	96	SAEFLLHMLK(*)NAESNAELK(*)	K10	65.25	130.84	1	-	0.967391	0.9836957
Rpl17	Q9CPR4	37	ETAQAIK(*)GMHIR(*)	K7	49.52	1000	1	0.679012	0.978260	0.8857577
Rpl17	Q9CPR4	159	EQIVPK(*)PEEEVAQK(*)	K6	48.25	43.13	-	1.148148	0.934782	1.0414654
Rpl17	Q9CPR4	96	K(*)SAEFLLHMLK(*)NAESNAELK(*)	K11	43.79	33.35	-	-	1.043478261	
Rpl17	Q9CPR4	96	LLHMLK(*)NAESNAELK(*)	K6	25.64	58.98	-	-	0.597826087	
Nanp	Q9CPT3	211	ATVWINK(*)SGR(*)	K7	94.9	1000	-	-	-	
Mydgf	Q9CPT4	138	SEEFEVTK(*)TAVSHR(*)PGAFK(*)	K8	90.61	36.4	1	0.72	-	0.86
Mydgf	Q9CPT4	130	ESDVPLK(*)SEEFEVTK(*)	K7	72.38	92.08	0.8461538	-	0.883720	0.8649374
Mydgf	Q9CPT4	138	ESDVPLK(*)SEEFEVTK(*)TAVSHR(*)	K15	53.41	49.35	0.7802197	-	0.941860	0.8610401
Mydgf	Q9CPT4	138	SEEFEVTK(*)TAVSHR(*)	K8	29.3	1000	-	1.266666	1.023255	1.1449612
Mydgf	Q9CPT4	93	PQGK(*)SYLYFTQFK(*)	K4	28.44	148.12	-	-	1.081395349	
Glo1	Q9CPU0	88	SEK(*)TAWTFSR(*)	K3	20.05	1000	-	1.039473	-	
Mrpl42	Q9CPV3	120	VFYTTK(*)HR(*)	K6	53.49	1000	-	-	-	
Pmf1	Q9CPV5	60	FTTC(+57.02)YK(*)HFHQLNPEVTQR	K6	60.58	1000	1.0144927	1.326086	0.925925	1.0888352
Arpc5	Q9CPW4	67	NPPINTK(*)SQAVK(*)	K7	79.83	51	0.8953488	0.986301	0.853932	0.9118609
Arpc5	Q9CPW4	112	YIYK(*)GFESPSDNSSAVLLQWHEK(*)	K4	63.64	193.56	1.4534883	-	1.370786	1.4121374
Arpc5	Q9CPW4	87	VLISFK(*)ANDIEK(*)	K6	20.61	64.21	-	-	1.235955056	
Ssr2	Q9CPW5	27	LLASK(*)SLLNR(*)	K5	93.25	1000	-	-	-	
Zmat2	Q9CPW7	8	A(+42.01)SGSGTK(*)NLDFR(*)	K7	87.21	1000	-	-	-	

Zmat2	Q9CPW7	55	DYK(*)VDLESK(*)	K3	49.64	112.31	-	-	-	
Atg3	Q9CPX6	185	AK(*)ADAGGEDAILQTR(*)	K2	38.33	1000	0.4021739	0.76	0.8	0.654058
Mrpl51	Q9CPY1	119	ISYLYK(*)HFNR(*)	K6	46.25	1000	-	1	-	
Lap3	Q9CPY7	356	NGK(*)TIQVDNTDAEGR(*)	K3	35.01	1000	-	-	0.806451613	
Chmp3	Q9CQ10	6	GLFGK(*)TQEK(*)PPK(*)	K5	64.65	111.84	0.7395833	0.458333	0.549180	0.5823657
Chmp3	Q9CQ10	92	AHMNSVLMGMK(*)NQLAVLR(*)	K11	47.38	1000	0.96875	-	-	
Chmp3	Q9CQ10	81	LYASK(*)AHMNSVLMGMK(*)	K5	45.27	35.27	0.8958333	-	0.770491	0.8331626
Chmp3	Q9CQ10	106	VAGSLQK(*)STEVMK(*)	K7	36.7	90.16	0.6041666	-	0.434426	0.5192964
Mcts2	Q9CQ21	18	ESVSNC(+57.02)IQLK(*)TSVIK(*)	K10	46.84	64.28	0.8367346	1.134831	0.654205	0.8752573
Stambp	Q9CQ26	107	AEELK(*)TELLR(*)	K5	14	1000	-	-	8.397959184	
Decr1	Q9CQ62	106	ATAEEISSK(*)TGNK(*)	K9	56.96	58.99	0.96875	-	0.962962	0.9658565
Mtap	Q9CQ65	51	IK(*)NVDC(+57.02)VLLAR(*)	K2	95.99	1000	1.0217391	0.822784	1.022988	0.9558375
Mtap	Q9CQ65	147	GVC(+57.02)HIPMAEPFC(+57.02)P	K14	88.91	1000	0.7826086	0.620253	0.954022	0.7856283
Mtap	Q9CQ65	271	NLK(*)NMAQFSVLPPR(*)	K3	66.48	1000	0.7934782	0.645569	0.954022	0.7976903
Vps25	Q9CQ80	161	ALQALQQEHK(*)AEIITVSDGR(*)	K10	9.6	1000	-	-	0.975409836	
Tspan31	Q9CQ88	153	TK(*)SSTC(+57.02)QMC(+57.02)GE	K2	47.33	1000	0.84	0.629629	0.976744	0.8154579
Trappc5	Q9CQA1	9	GK(*)SALLER(*)	K2	57.95	1000	0.5403726	0.653465	0.579710	0.5911827
Sdhb	Q9CQA3	82	IK(*)NEVDSTLTFR(*)	K2	81.38	1000	0.8584905	0.593406	0.855670	0.7691891
Bzw1	Q9CQC6	191	LFK(*)SWINEK(*)	K3	97.7	113.05	0.8191489	0.820512	0.988505	0.8760558
Bzw1	Q9CQC6	390	GK(*)SVFLEQMK(*)	K2	94.39	175.25	0.7659574	0.794871	0.896551	0.819127
Bzw1	Q9CQC6	319	EELVAEQAIK(*)HLK(*)	K10	63.68	47.36	0.8829787	0.807692	0.931034	0.8739018
Bzw1	Q9CQC6	368	YK(*)AEVLSEEPILK(*)	K2	50.72	195.68	-	-	1.057471264	
Bzw1	Q9CQC6	140	LLLFLK(*)GFSESER(*)	K6	36.26	1000	1.5	-	0.712643	1.1063218
Bzw1	Q9CQC6	116	AQVFNK(*)LIR(*)	K6	8.13	1000	-	0.782051	-	
Spg21	Q9CQC8	124	FAEYTHK(*)SPR(*)	K7	19.18	1000	-	1.296296	-	
Chmp1b2	Q9CQD4	59	QK(*)NQAINFLR(*)	K2	79.2	1000	0.7661290	1.069444	0.813559	0.8830443
Chmp1b2	Q9CQD4	97	SMAGVVK(*)SMDATLR(*)	K7	59.41	1000	0.5564516	0.736111	0.593220	0.6285944
Chmp1b2	Q9CQD4	6	S(+42.01)NMEK(*)HLFNLK(*)	K5	57.81	176.18	0.75	1.097222	0.940677	0.9293001
Chmp1b2	Q9CQD4	6	M(+42.01)EK(*)HLFNLK(*)	K3	42.47	143.14	0.7016129	0.902777	0.771186	0.791859
Nipsnap3b	Q9CQE1	48	TYFLK(*)PSK(*)TNEFLENFK(*)	K8	64.01	22.45	0.6226415	-	0.887640	0.755141
Mrps17	Q9CQE3	78	HVK(*)HELAEIIFK(*)	K3	62.73	222.06	0.8190476	0.676767	1.602272	1.032696
RTRAF	Q9CQE8	234	INEAIVAVQAIIADPK(*)TDHR(*)	K16	34.57	1000	-	-	1.103092784	
Mrpl11	Q9CQF0	115	EVAGLVSLK(*)HVYEIAC(+57.02)VK(	K9	26.32	31.37	1.4239130	-	1.242105	1.3330092
Nudt21	Q9CQF3	189	ALFAVPK(*)NYK(*)	K7	70.91	22.34	-	0.807228	0.979166	0.8931978

Mtres1	Q9CQF4	158	NK(*)VEDAFYK(*)	K2	44.14	79.39	-	1.243589	0.879518(	1.0615539
Snrpb2	Q9CQI7	111	AK(*)TMEQAAAAANK(*)	K2	85.63	306.51	0.9072164	0.878378	0.890109{	0.8919016
Snrpb2	Q9CQI7	85	IQYAK(*)TDSDIISK(*)	K5	59.49	118.18	0.7216494	0.554054	0.857142{	0.7109488
Pih1d1	Q9CQJ2	57	PGFC(+57.02)VK(*)TNSSEGK(*)	K6	72.52	32.62	-	-	0.819148936	
Rnf2	Q9CQJ4	133	INK(*)HNNQQALSHSIEEGLK(*)	K3	10.34	30.61	-	-	1.191011236	
Mrpl20	Q9CQL4	26	VQEVLK(*)HAQHFR(*)	K6	38.74	1000	1.2307692	1.657142	1.255813{	1.381242
Glrx3	Q9CQM9	153	LTHAAPC(+57.02)MLFMK(*)GTPQEI	K12	62.05	1000	0.8350515	0.839506	0.824175{	0.8329112
Glrx3	Q9CQM9	165	C(+57.02)GFSK(*)QMVEILHK(*)	K5	29.06	112.87	-	0.888888	0.901098{	0.8949939
Trap1	Q9CQN1	377	VLIQTK(*)AADILPK(*)	K6	71.94	122.76	-	-	1.04494382	
Trap1	Q9CQN1	216	MVADK(*)VEVYSR(*)	K5	67.26	1000	-	-	0.752808989	
Trap1	Q9CQN1	260	IIIHLK(*)SDC(+57.02)K(*)	K6	59.24	61.37	0.9797979	0.719512	-	0.8496551
Trap1	Q9CQN1	631	MQQLAK(*)TQEER(*)	K6	51.1	1000	1.0909090	0.719512	1.056179	0.9555337
Trap1	Q9CQN1	260	IIIHLK(*)SDC(+57.02)K(*)DFASESR(*	K6	42.09	42.68	0.8282828	0.475609	1.022471{	0.7754548
Trap1	Q9CQN1	89	GSVSK(*)HEFQAETK(*)	K5	40.81	193.63	1.5252525	-	1.056179	1.2907162
Trap1	Q9CQN1	600	VTNVK(*)VTFR(*)	K5	27.74	1000	-	-	1.179775281	
Trap1	Q9CQN1	334	FTLHYK(*)TDAPLNIR(*)	K6	23.78	1000	1.3939393	1.268292	-	1.331116
Atp5pb	Q9CQQ7	225	HVVK(*)SISVQQEK(*)	K4	89.46	172.06	1	0.929411	1.011111	0.9801743
Atp5pb	Q9CQQ7	210	R(*)K(*)EEEHMIDWVEK(*)	K2	85.91	235.98	1.0707070	0.729411	0.988888{	0.9296692
Atp5pb	Q9CQQ7	221	EEEHMIDWVEK(*)HVVK(*)	K11	72.7	49.79	1.6161616	-	1.522222	1.5691919
Atp5pb	Q9CQQ7	221	R(*)K(*)EEEHMIDWVEK(*)HVVK(*)	K13	63.74	88.98	1.1515151	0.952941	0.811111	0.9718558
Atp5pb	Q9CQQ7	221	K(*)EEEHMIDWVEK(*)HVVK(*)	K12	61.15	48.12	0.7878787	0.811764	0.966666(	0.8554367
Rps21	Q9CQR2	74	LAK(*)ADGIVSK(*)	K3	91.48	158.8	0.8333333	0.790697	-	0.8120155
Acot13	Q9CQR4	17	VMFK(*)VPGFDR(*)	K4	32.39	1000	-	0.796460	0.404624	0.6005422
Nop10	Q9CQS2	53	FK(*)VLMTQQPR(*)	K2	48.92	1000	0.9666666	1.025641	0.793814	0.9287074
Rab5if	Q9CQT9	27	VSVWSK(*)VLR(*)	K6	61.9	1000	-	-	-	
Txndc12	Q9CQU0	30	TGLGK(*)GFGDHIHWR(*)	K5	61.06	1000	-	0.523255	1.082352{	0.8028044
Ywhab	Q9CQV8	5	T(+42.01)MDK(*)SELVQK(*)	K4	62.87	95.35	0.9285714	0.8125	1.311827{	1.0176331
Ywhab	Q9CQV8	5	M(+42.01)TMDK(*)SELVQK(*)	K5	57.72	87.26	1.0306122	0.925	0.924731	0.9601145
Ywhab	Q9CQV8	70	VISSIEQK(*)TER(*)	K8	23.96	1000	-	-	1.634408602	
Ykt6	Q9CQW1	11	LYSLSVLYK(*)GDPK(*)	K9	34.1	52.86	-	-	0.527472527	
Ykt6	Q9CQW1	173	LDDLVSK(*)SEVLGTQSK(*)	K7	23.11	148.12	-	1.636363	0.648351(	1.1423576
Psmd9	Q9CR00	210	WAGK(*)GLLGC(+57.02)NIIPLQR(*)	K4	38.44	1000	0.6938775	-	1.117021	0.9054494
Psmd9	Q9CR00	30	R(*)K(*)EEIEAEIK(*)	K2	29.67	89.48	-	-	0.755319149	
Ufc1	Q9CR09	47	YVENNK(*)NSDNDWFR(*)	K6	25.76	1000	-	1.320512	-	

Ufc1	Q9CR09	17	VVSEIPVLK(*)TNAGPR(*)	K9	14.58	1000	0.9387755	-	0.84375	0.8912628
Ppid	Q9CR16	111	FEDENFHYK(*)HDR(*)	K9	80.22	1000	1.1052631	0.986842	1	1.0307018
Ppid	Q9CR16	349	AIQAELLK(*)VK(*)	K8	34.36	36.05	-	1.078947	0.824175	0.9515616
Ppid	Q9CR16	64	GTGSTTGK(*)PLHFK(*)	K8	33.96	61.26	-	-	0.395604396	
Ppid	Q9CR16	145	LDGK(*)HVVFGQVIK(*)	K4	26.01	178.84	0.9789473	1.065789	0.813186	0.9526412
Slc25a11	Q9CR62	162	GYK(*)NVFNALVR(*)	K3	65.86	1000	0.7701149	0.611111	0.858823	0.7466832
Slc25a11	Q9CR62	73	TSFHALTSILK(*)TEGLK(*)	K11	32.02	25.91	0.7931034	-	1.282352	1.0377282
Slc25a11	Q9CR62	62	EYK(*)TSFHALTSILK(*)	K3	17.44	176.98	1.1149425	1.583333	1.258823	1.3190331
Slc25a11	Q9CR62	256	MIDGK(*)PEYK(*)	K5	14.28	22.99	1.6666666	-	-	
Slc25a11	Q9CR62	57	MQLSGEGAK(*)TR(*)	K9	8.63	1000	-	-	0.364705882	
Tmem167a	Q9CR64	36	NK(*)TGLLGIFWK(*)	K2	66.2	250.15	1	-	0.754901	0.877451
Tmem33	Q9CR67	134	VLDAK(*)GSNSLPLLR(*)	K5	68.65	1000	0.7916666	0.626506	0.891304	0.7698257
Lage3	Q9CR70	52	NSSK(*)SMVPLTQR(*)	K4	65.72	1000	0.5543478	0.265486	-	0.4099173
Fam136a	Q9CR98	99	DSMDAGTK(*)ELQVK(*)	K8	11.59	100.21	-	-	1.361904762	
Golph3	Q9CRA5	224	VQEAVLDK(*)WVNDPHR(*)	K8	20.45	1000	-	1.111111	0.836956	0.9740338
Exosc5	Q9CRA8	73	EIFNK(*)ATLEVILR(*)PK(*)	K5	36.33	67.81	-	-	0.913043478	
Chchd3	Q9CRB9	157	SSEFYK(*)VTTEEYQK(*)	K6	17.08	85.91	-	-	1.660377358	
Gnpda2	Q9CRC9	160	LK(*)TLAMDTILANAK(*)	K2	43.29	204.37	-	-	0.934782609	
Gnpda2	Q9CRC9	256	YFK(*)GLMHVHNK(*)	K3	36.25	118.18	-	-	0.869565217	
Xpot	Q9CRT8	951	VFK(*)NYLK(*)	K3	19.31	72.09	0.7731958	-	0.945652	0.859424
Xpot	Q9CRT8	554	FVK(*)SLNK(*)	K3	9.01	68.07	1.1958762	-	-	
Ecd	Q9CS74	250	VFK(*)TFLPETR(*)	K3	24.83	1000	1.4375	0.616666	1.013888	1.0226852
Ecd	Q9CS74	463	AFISK(*)VSSHK(*)	K5	10.02	22.36	2.109375	-	-	
Dis3	Q9CSH3	118	DVTNNQEK(*)HFYTFTNEHHK(*)	K8	60.47	116.72	0.9684210	0.868421	0.880434	0.905759
Dis3	Q9CSH3	579	FTK(*)SVINSK(*)	K3	54.69	102.87	-	-	0.847826087	
Dis3	Q9CSH3	242	LQQGIK(*)SGSYLQGTFR(*)	K6	51.69	1000	0.8526315	0.578947	1.097826	0.843135
Dis3	Q9CSH3	281	EILIQGIK(*)HLNR(*)	K8	43.15	1000	-	-	0.880434783	
Dis3	Q9CSH3	709	SK(*)NLQIK(*)	K2	8.74	115.97	1.1157894	-	1.086956	1.101373
Snw1	Q9CSN1	441	GGK(*)DMAQSIYR(*)PSK(*)	K3	37.19	194.69	-	-	0.946236559	
Snw1	Q9CSN1	339	AGIK(*)THVEK(*)	K4	8.73	61.3	-	0.8	0.806451	0.8032258
Ranbp3	Q9CT10	337	LFVFDK(*)TSQSWVER(*)	K6	50.44	1000	0.75	0.695121	0.885416	0.7768462
Fcf1	Q9CTH6	137	LPC(+57.02)THK(*)GTYADDC(+57.0	K6	32.38	1000	0.8695652	-	1.267605	1.0685854
Smc1a	Q9CU62	540	VLGK(*)NMDAIIVDSEK(*)	K4	64.74	159.14	-	0.829268	0.881720	0.8554944
Smc1a	Q9CU62	536	YQIAVTK(*)VLGK(*)	K7	56.85	63.97	0.7052631	0.365853	0.741935	0.6043508

Smc1a	Q9CU62	110	INNK(*)VVQLHEYSEELEK(*)	K4	53.73	67.11	-	-	0.946236559	
Smc1a	Q9CU62	637	HK(*)TVALDGTLFQK(*)	K2	46.48	160.69	0.7789473	0.158536	0.731182	0.5562222
Smc1a	Q9CU62	13	LIEIENFK(*)SYK(*)	K8	33.64	26.52	1.3052631	1.158536	0.924731	1.1295103
Smc1a	Q9CU62	536	K(*)YQIAVTK(*)VLGK(*)	K8	28.91	52.86	0.8315789	0.890243	0.634408	0.7854105
Smc1a	Q9CU62	324	SLQNAQK(*)HYK(*)	K7	21.3	57.45	1.0315789	-	1.161290	1.0964346
Zmym2	Q9CU65	787	GEMK(*)HFC(+57.02)DQHC(+57.02	K4	16.64	1000	2.1875	-	1.94	2.06375
Arpc2	Q9CVB6	295	TITGK(*)TFSSR(*)	K5	82	1000	-	0.846153	0.903225	0.8746898
Arpc2	Q9CVB6	269	AK(*)TSDFLK(*)	K2	72.5	108.85	0.8804347	0.602564	1.096774	0.8599244
Arpc2	Q9CVB6	256	DYLHYHIK(*)C(+57.02)SK(*)	K8	13.67	34.94	-	1.807692	1.096774	1.4522333
Smc3	Q9CW03	629	HVFGK(*)TLIC(+57.02)R(*)	K5	85.2	1000	0.9484536	0.85	0.923076	0.9071768
Smc3	Q9CW03	624	AFK(*)HVFGK(*)	K3	72.71	119.54	0.8969072	0.9375	1.010989	0.9484654
Smc3	Q9CW03	1194	NK(*)VSHIDVITAEMAK(*)	K2	67.65	163.91	0.8556701	0.9	0.956043	0.9039047
Smc3	Q9CW03	106	VIGAK(*)K(*)DQYFLDK(*)	K6	61.21	163.4	2.1649484	1.875	1.670329	1.903426
Smc3	Q9CW03	105	VIGAK(*)K(*)DQYFLDK(*)	K5	61.21	188.52	2.1649484	1.875	1.670329	1.903426
Smc3	Q9CW03	1038	QVSK(*)NFSEVFQK(*)	K4	51.54	62.55	0.8762886	0.775	1.230769	0.960686
Smc3	Q9CW03	26	DQTIVDPFSSK(*)HNVIVGR(*)	K11	14.34	1000	-	0.8	1.142857	0.9714286
Golga1	Q9CW79	690	EINFEYLK(*)HVVLK(*)	K8	9.75	23.7	-	-	0.646258503	
Ndufaf7	Q9CWG8	47	IK(*)STGPITVAEYMK(*)	K2	21.4	186.68	-	0.605263	1	0.8026316
Ndufaf7	Q9CWG8	302	IASTGGAALIADYGHDGTK(*)TDTLR(*	K19	16.35	1000	-	-	1.89010989	
Bccip	Q9CWI3	233	TFMEAGK(*)SSSR(*)	K7	32.17	1000	0.9680851	-	0.958333	0.9632092
Atic	Q9CWJ9	406	SLFSNIVTK(*)NK(*)	K9	83.59	27.83	-	0.871794	0.921348	0.8965716
Atic	Q9CWJ9	66	VK(*)TLHPAVHAGILAR(*)	K2	80.92	1000	1.0752688	0.717948	1.112359	0.9685257
Atic	Q9CWJ9	461	LAGDK(*)ANSWWLR(*)	K5	73	1000	1.3440860	0.717948	0.955056	1.005697
Atic	Q9CWJ9	266	GAVDIPAAASFK(*)HVSPAGAAVGVPI	K12	55.38	1000	0.5806451	-	1	0.7903226
Atic	Q9CWJ9	199	QYSK(*)GISQMPLR(*)	K4	46.51	1000	-	0.589743	-	
Atic	Q9CWJ9	14	SVSDK(*)TGLVEFAR(*)	K5	43.38	1000	-	-	0.97752809	
Atic	Q9CWJ9	397	NNGVVDK(*)SLFSNIVTK(*)	K7	40.41	142.42	-	-	0.898876404	
Atic	Q9CWJ9	137	AAAK(*)NHAR(*)	K4	12.85	1000	-	-	0.966292135	
Snx2	Q9CWK8	167	VTTK(*)TSLSMFSK(*)	K4	63.28	72.62	-	-	1.769230769	
Snx2	Q9CWK8	248	TVK(*)HPTLLQDPDLR(*)	K3	50.23	1000	1.1585365	0.921052	0.868131	0.9825737
Snx2	Q9CWK8	456	EWEAK(*)VQQGER(*)	K5	46.59	1000	1.0609756	0.907894	0.780219	0.9163634
Snx2	Q9CWK8	116	IESK(*)SISAPVIFDR(*)	K4	36.48	1000	1.1097560	-	-	
Snx2	Q9CWK8	447	IQQAK(*)NEIR(*)	K5	9.81	1000	-	1.078947	-	
Ctnnbl1	Q9CWL8	95	SYK(*)NQELR(*)	K3	45.59	1000	-	-	1.104166667	

Cnot11	Q9CWN7	490	LLK(*)TLDTGETPSETK(*)	K3	40.52	126.79	-	-	1.513888889	
Mospd2	Q9CWP6	199	IVK(*)SWLGPEAVSLLK(*)	K3	42.77	167.97	0.6018518	-	0.901785	0.7518188
Nup37	Q9CWU9	219	MSAHWC(+57.02)LK(*)NTFK(*)	K8	17.59	28.7	0.9887640	-	1.712643	1.3507039
Mcm8	Q9CWV1	210	ATC(+57.02)YGK(*)YISIR(*)	K6	48.09	1000	-	-	-	
Sugt1	Q9CX34	212	VLSTK(*)IEIK(*)	K5	45.84	64.53	1.1515151	0.701298	0.869565	0.9074597
Sugt1	Q9CX34	60	AYC(+57.02)HILLGK(*)YR(*)	K9	27.48	1000	-	-	0.782608696	
Psmd8	Q9CX56	141	LTK(*)QQLILAR(*)	K3	90.34	1000	0.9890109	0.873417	0.977528	0.9466523
Psmd8	Q9CX56	230	IK(*)HPVSLEQYLMEGSYNK(*)	K2	41.5	230.7	-	-	0.629213483	
Psmd8	Q9CX56	230	VYIK(*)HPVSLEQYLMEGSYNK(*)	K4	38.64	186.26	-	-	1.325842697	
L3hypdh	Q9CXA2	299	AFK(*)SSATGSVFTGC(+57.02)AVR(*)	K3	29.64	1000	-	-	-	
Tmed5	Q9CXE7	164	LEDILESINSIK(*)SR(*)	K12	54.01	1000	-	-	0.663265306	
Tmed5	Q9CXE7	169	LSK(*)SGHIQTLLR(*)	K3	37.6	1000	-	-	1.193877551	
Tbc1d15	Q9CXF4	235	SK(*)VTNYIFDSLR(*)	K2	48.54	1000	-	-	1.26744186	
Tbc1d15	Q9CXF4	563	QQIMAK(*)HYGFNEILK(*)	K6	40.97	43	0.5980392	1.379746	1.023255	1.0003473
Tbc1d15	Q9CXF4	572	HYGFNEILK(*)HINELSMK(*)	K9	34.82	89.48	1.6078431	-	0.906976	1.2574099
Manf	Q9CXI5	154	GC(+57.02)AEK(*)SDYIR(*)	K5	74.86	1000	0.7096774	0.837837	-	0.7737576
Manf	Q9CXI5	149	ILDDWGEMC(+57.02)K(*)GC(+57.0	K10	35.65	55.6	-	-	1.813186813	
Nip7	Q9CXK8	96	YK(*)VWVK(*)PGAEQSFLYGNHVLK(	K2	39.48	105.6	-	-	0.936842105	
Nip7	Q9CXK8	115	VWVK(*)PGAEQSFLYGNHVLK(*)SGL	K19	23.8	124.59	0.9795918	-	-	
Rbm33	Q9CXK9	987	QLPHK(*)VLQVK(*)	K5	38.3	55.6	0.5384615	0.528735	-	0.5335986
Rbm33	Q9CXK9	945	LLVK(*)NQDITTASVQPK(*)	K4	28.37	164.29	1.4615384	1.114942	-	1.2882405
Rbm33	Q9CXK9	987	QLPHK(*)VLQVK(*)PMDMEETPHSP(	K5	7.01	23.7	-	0.873563	-	
Dhrs7	Q9CXR1	321	IQNFK(*)NNLDPDLPYK(*)	K5	36.71	118.06	0.6610169	-	2.049180	1.3550986
Med10	Q9CXU0	106	FK(*)SLLIQELSK(*)	K2	57.21	154.31	-	0.728260	0.916666	0.8224638
Mrps22	Q9CXW2	80	ILTK(*)ITGLDLQK(*)	K4	56.3	175.25	1.1888888	0.947368	0.792792	0.97635
Cacybp	Q9CXW3	147	VK(*)TDTVIILC(+57.02)R(*)	K2	91.29	1000	0.9183673	0.961038	1.011111	0.9635058
Cacybp	Q9CXW3	119	SFDLLVK(*)NLNGK(*)	K7	77.5	77.53	0.8673469	0.805194	0.855555	0.8426991
Cacybp	Q9CXW3	213	TINK(*)AWVESR(*)	K4	39.17	1000	1.3265306	0.831168	1.222222	1.1266406
Cacybp	Q9CXW3	147	K(*)VK(*)TDTVIILC(+57.02)R(*)	K3	31.03	32.28	-	0.935064	0.966666	0.9508658
Cacybp	Q9CXW3	43	IETELK(*)NK(*)	K6	17.1	30.83	1.7142857	-	1.2	1.4571429
Rpl11	Q9CXW4	169	WFQQK(*)YDGIILPGK(*)	K5	86.79	161.52	0.8080808	0.715909	0.944444	0.8228114
Rpl11	Q9CXW4	52	VLEQLTGQTPVFSK(*)AR(*)	K14	72.72	1000	0.8787878	0.772727	0.988888	0.8801347
Rpl11	Q9CXW4	78	GAK(*)AEEILEK(*)	K3	62.77	142.27	0.9090909	-	-	
Snx7	Q9CY18	322	LEDK(*)VEC(+57.02)ANNALK(*)	K4	11.82	95.39	-	-	1.361904762	

Tecr	Q9CY27	38	TLFTK(*)THPQWYPAR(*)	K5	73.53	1000	1.0227272	0.75	1.011235!	0.9279877
Tecr	Q9CY27	57	GK(*)SLK(*)DEDVLQK(*)	K2	48.39	62.75	1.0568181	0.789473	0.955056:	0.9337827
Tecr	Q9CY27	2	M(+15.99)K(*)HYEVEIR(*)	K2	43.49	1000	-	1.157894	-	
Tecr	Q9CY27	16	EK(*)LC(+57.02)FLDK(*)	K2	16.97	62.39	1.4772727	-	0.707865:	1.0925689
Chtop	Q9CY57	82	LGK(*)SNIQAR(*)	K3	9.63	1000	0.9361702	-	-	
Serbp1	Q9CY58	326	GFVLHK(*)SK(*)	K6	88.19	22.87	1.0744680	1.089743	1.022727:	1.062313
Serbp1	Q9CY58	52	EAGGGGVGGPGAK(*)SAAQAAAQTN	K13	77.25	210.26	-	1.102564	1.079545	1.0910548
Serbp1	Q9CY58	328	SK(*)SEEAHAEDSVMDHHFR(*)	K2	75.48	1000	1.1063829	1.025641	1.147727:	1.0932504
Serbp1	Q9CY58	211	SSFSHYSGLK(*)HEDK(*)R(*)	K10	72.51	69.71	1.0531914	1.038461	0.988636:	1.0267631
Serbp1	Q9CY58	302	AK(*)VEFNIR(*)	K2	68.37	1000	0.8723404	0.897435	1.068181	0.945986
Serbp1	Q9CY58	211	SSFSHYSGLK(*)HEDK(*)	K10	16.3	34.3	1.4255319	-	1.375	1.400266
Serbp1	Q9CY58	140	R(*)FEK(*)PLEEK(*)	K4	15.15	61.3	-	-	0.818181818	
Blvra	Q9CY64	233	FK(*)SGSLEEVPNVGVNK(*)	K2	47.14	189.02	-	-	0.62962963	
Blvra	Q9CY64	233	HISIHFK(*)SGSLEEVPNVGVNK(*)	K7	26.68	42.26	2.0714285	-	-	
Gar1	Q9CY66	123	EQVGK(*)VDEIFGQLR(*)	K5	54.08	1000	1	0.925	0.848214:	0.9244048
Mrpl44	Q9CY73	99	TAFINSC(+57.02)YIK(*)SEEAK(*)	K10	27.13	37.78	1.0948275	1.067164	0.710144!	0.9573789
Dsn1	Q9CYC5	174	ASSLSEELK(*)HFTDR(*)	K9	25.82	1000	-	-	-	
Crtap	Q9CYD3	173	AIAAAHTYLLK(*)HPDDEMMK(*)	K11	14.18	63.64	-	1.48	-	
Luc7l	Q9CYI4	37	VC(+57.02)K(*)SHLLDC(+57.02)C(+!	K3	49.1	1000	-	-	1.020833333	
Luc7l	Q9CYI4	61	MDLGEC(+57.02)TK(*)IHDLALR(*)	K8	45.97	1000	-	0.865671	1.010416(	0.9380442
Glipr2	Q9CYL5	53	ILK(*)HSPESSR(*)	K3	51.14	1000	1.0824742	1	0.897727:	0.9934005
Spcs2	Q9CYN2	50	IDK(*)WDGSAVK(*)	K3	55.85	84.68	1.1170212	1.293333	1.519480!	1.309945
Spcs2	Q9CYN2	57	WDGSAVK(*)NSLDDSAK(*)	K7	50.85	84.58	-	3.413333	1.012987(	2.2131602
Spcs2	Q9CYN2	137	EK(*)SIFLVAHR(*)	K2	20.24	1000	0.8617021	-	0.701298	0.7815004
Ssbp1	Q9CYR0	81	SGDSEVYQMGDVSQK(*)TTWHR(*)	K15	35.5	1000	-	-	1.052631579	
Ssbp1	Q9CYR0	122	VDYGEYMDK(*)NNVR(*)	K9	21.17	1000	-	-	2.189473684	
Ssbp1	Q9CYR0	113	IFVEGK(*)VDYGEYMDK(*)	K6	16.3	120.76	-	-	2.073684211	
Pgm3	Q9CYR6	350	VPVYC(+57.02)TK(*)TGVK(*)	K7	38.17	58.99	-	0.638554	0.782178:	0.7103662
Rrp15	Q9CYX7	179	GVVQLFNAVQK(*)HQR(*)	K11	73.48	1000	0.9607843	0.949367	1	0.9700505
Tpd52l2	Q9CYZ2	108	SWHDVQVSTAYVK(*)TSEK(*)	K13	23.95	42.89	-	0.935064	0.896551	0.9158083
Ssbp2	Q9CYZ8	6	GK(*)SNSSAVPSDSQAR(*)	K2	96.35	1000	-	3.25	0.493055!	1.8715278
Snf8	Q9CZ28	53	TNLEEFASK(*)HK(*)	K9	58.34	24.32	0.8558558	0.770270	0.880434	0.8355203
Ola1	Q9CZ30	190	VK(*)SWVIDQK(*)	K2	92.87	209.49	0.7979797	0.777777	0.978021!	0.8512599
Ola1	Q9CZ30	180	LK(*)PEYDIMC(+57.02)K(*)	K2	78.31	124.34	1.3434343	-	1.054945(	1.1991897

Ola1	Q9CZ30	216	EIEVLNK(*)HLFLTSK(*)	K7	57.91	128.65	0.8383838	0.691358	0.923076	0.8176063
Srfbp1	Q9CZ91	328	VPSK(*)TSTLETPWDVR(*)	K4	51.08	1000	-	0.611650	-	
Gars1	Q9CZD3	409	IYLYLTK(*)VGISPDK(*)	K7	78.34	122.76	0.6489361	0.631578	0.824175	0.7015636
Gars1	Q9CZD3	543	TFQLTK(*)DMVSVK(*)	K6	77.44	110.2	0.8085106	0.657894	0.879120	0.7818421
Gars1	Q9CZD3	537	GEFTIETEGK(*)TFQLTK(*)	K10	75.81	121.92	0.8510638	0.723684	0.901098	0.8252823
Gars1	Q9CZD3	553	FQK(*)TLHVEEVVPSVIEPSFGLGR(*)	K3	68.55	1000	-	-	0.747252747	
Gars1	Q9CZD3	467	ATK(*)VPLVAEK(*)PLK(*)	K3	51.63	195.71	0.7021276	0.684210	0.791208	0.725849
Gars1	Q9CZD3	636	NGVSHK(*)VDDSSGSIGR(*)	K6	38.68	1000	1.5957446	1.486842	1.054945	1.3791773
Gars1	Q9CZD3	467	ATK(*)VPLVAEK(*)PLK(*)EPK(*)	K3	26.84	104.64	0.7978723	0.815789	0.615384	0.7430155
Gars1	Q9CZD3	467	ATK(*)VPLVAEK(*)	K3	12.8	85.08	-	1.302631	1.340659	1.3216455
Psmg3	Q9CZH3	80	AK(*)NLVAFVSQEAGNR(*)	K2	41.81	1000	-	0.493506	1.019607	0.7565572
Utp11	Q9CZJ1	120	LK(*)SELHLLDFQGK(*)	K2	21.36	203.1	-	-	1.21978022	
Rpl15	Q9CZM2	176	GLGK(*)GHK(*)	K4	79.44	51.69	-	1.025641	1.125	1.0753205
Rpl15	Q9CZM2	56	AK(*)QGYVIYR(*)	K2	72.64	1000	0.8453608	1.064102	1.181818	1.0304272
Rpl15	Q9CZM2	83	GATYGK(*)PVHHGVNQLK(*)	K6	57.52	62.68	0.8041237	0.974358	0.943181	0.9072215
Rpl15	Q9CZM2	157	R(*)NPDTQWITK(*)PVHK(*)HR(*)	K14	20.42	26.57	1.6082474	-	-	
Shmt2	Q9CZN7	469	LQDFK(*)SFLLK(*)	K5	96.79	105.53	0.84375	0.759036	0.869565	0.8241171
Shmt2	Q9CZN7	280	YADVVTTTTHK(*)TLR(*)	K11	79.65	1000	0.8645833	0.759036	0.945652	0.8564239
Shmt2	Q9CZN7	305	TGK(*)EIPYTFEDR(*)	K3	55.5	1000	0.9270833	0.771084	1.108695	0.9356211
Shmt2	Q9CZN7	469	LQDFK(*)SFLLK(*)DPETSQR(*)	K5	44.35	38.19	1.25	1.277108	0.945652	1.1575869
Shmt2	Q9CZN7	409	VLELVSITANK(*)NTC(+57.02)PGDR(	K11	40.89	1000	-	0.578313	0.967391	0.7728523
Mtrex	Q9CZU3	794	VIQK(*)VEAFEHR(*)	K4	19.06	1000	-	1.067567	1.337209	1.2023884
Cs	Q9CZU6	450	PK(*)SMSTDGLMK(*)	K2	96.61	202.16	0.7623762	0.747126	0.877777	0.7957602
Cs	Q9CZU6	366	EFALK(*)HLPK(*)	K5	76.13	97.69	0.7524752	0.563218	0.711111	0.6756016
Cs	Q9CZU6	215	LPC(+57.02)VAAK(*)IYR(*)	K7	72.69	1000	-	-	0.7	
Cs	Q9CZU6	57	QQHGK(*)TVVGQITVDMMYGGMR(*	K5	48.24	1000	1.8415841	-	0.922222	1.3819032
Acsl3	Q9CZW4	397	NVMNK(*)VNEMSAFQR(*)	K5	38.54	1000	0.6170212	1.121951	-	0.8694862
Acsl3	Q9CZW4	706	ELK(*)THYQADIER(*)	K3	36.18	1000	-	0.780487	1.104651	0.9425695
Acsl3	Q9CZW4	521	NWEEGGYFNTDK(*)PHPR(*)	K12	28.34	1000	-	-	1.127906977	
Acsl3	Q9CZW4	509	LK(*)NWEEGGYFNTDK(*)PHPR(*)	K2	22.51	158.38	-	-	0.988372093	
Elp3	Q9CZX0	333	GTGLYELWK(*)SGR(*)	K9	35.33	1000	0.5757575	-	-	
Pinx1	Q9CZX5	43	GK(*)GLGAQEQGATEHIK(*)	K2	55.96	234.02	0.8673469	1.513513	0.81	1.0636202
Rps19	Q9CZX8	29	LK(*)VPEWVDTVK(*)	K2	95.31	158.68	0.8039215	0.790697	0.878787	0.824469
Rps19	Q9CZX8	77	GGAGVGSMTK(*)IYGGR(*)	K10	94.22	1000	0.9019607	0.883720	0.828282	0.8713215

Rps19	Q9CZX8	43	HK(*)ELAPYDENWFYTR(*)	K2	59.32	1000	1.0392156	2.767441	1.050505(	1.6190542
Ube2v1	Q9CZY3	68	IYSLK(*)IEC(+57.02)GPK(*)	K5	89.41	85.43	0.4423076	-	-	
Ube2v1	Q9CZY3	10	A(+42.01)ATTGSGVK(*)VPR(*)	K9	71.4	1000	1.0576923	0.109890	0.268072	0.4785516
Ube2v1	Q9CZY3	131	LMMSK(*)ENMK(*)	K5	45.3	42.89	0.7403846	0.098901	0.240963	0.3600832
Nt5c3a	Q9D020	247	GELIHVFNK(*)HDGALK(*)	K9	58.52	49.54	1.1075268	0.453125	0.833333	0.7979951
Mpc2	Q9D023	19	LMDK(*)VELLLPK(*)	K4	63.18	118.9	0.7528089	-	1	0.8764045
Ssbp3	Q9D032	6	GK(*)GSAVPSDGQAR(*)	K2	42.92	1000	-	0.532110	-	
Arpin	Q9D0A3	203	TGASWTDNIMAQK(*)SSER(*)	K13	28.48	1000	1.5789473	1.966666	1.109756(	1.55179
Pbdc1	Q9D0B6	66	LTK(*)VDDQIYSEFR(*)	K3	37.75	1000	1.5744680	-	0.978723	1.2765957
Trmt5	Q9D0C4	212	DHQLPFK(*)HLIGQVMVDK(*)	K7	5.24	21.51	-	-	1.444444444	
Gtf2e1	Q9D0D5	106	YK(*)LDHMR(*)	K2	8.16	1000	-	-	1.05952381	
Hnrnpm	Q9D0E1	133	AAEVLNK(*)HSLSGR(*)	K7	82.97	1000	1.125	0.9625	1.055555	1.0476852
Hnrnpm	Q9D0E1	241	ADILEDK(*)DGK(*)SR(*)	K10	40.34	53.53	0.8645833	0.7875	0.855555	0.8358796
Lman1	Q9D0F3	415	QVNEVK(*)NSMSETVR(*)	K6	71.3	1000	0.7777777	0.819277	1.065217	0.8874241
Rfc5	Q9D0F6	65	LPHLLLYGPPGTGK(*)TSTILAC(+57.0	K14	44.11	161.68	-	-	0.731958763	
Pgm1	Q9D0F9	8	IVTVK(*)TQAYPDQK(*)PGTSGLR(*)	K5	28.35	118.18	1.3222222	-	-	
Pgm1	Q9D0F9	349	VANATK(*)IALYETPTGWK(*)	K6	19.96	158.53	1.1	1.059523	1.144444	1.1013228
Mrto4	Q9D0I8	71	NK(*)VMMVALGR(*)	K2	53.6	1000	0.6421052	-	1.079545	0.8608254
Mrto4	Q9D0I8	109	TK(*)EEVNEWFTK(*)	K2	42.96	126.63	0.6631578	-	-	
Rars	Q9D0I9	143	EIAENITK(*)HLPNNK(*)	K8	86.31	90.32	0.8888888	0.860759	0.934065	0.8945714
Rars	Q9D0I9	629	VLK(*)VNMWR(*)	K3	75.82	1000	0.8484848	0.860759	1.021978(	0.9104075
Rars	Q9D0I9	563	ETK(*)IILDHEK(*)	K3	68.01	117.63	0.7979797	-	0.857142	0.8275613
Rars	Q9D0I9	30	LK(*)NC(+57.02)GC(+57.02)LEASPS	K2	36.45	1000	0.6767676	0.632911	0.439560	0.5830798
Arl2	Q9D0J4	29	LLMLGLDNAGK(*)TTILK(*)	K11	53.94	55.6	-	-	1.168539326	
Oxct1	Q9D0K2	436	TK(*)VVVTMEHSAK(*)	K2	61	187.15	-	0.6375	0.944444	0.7909722
Oxct1	Q9D0K2	421	MVK(*)GMGGAMDLVSSSK(*)	K3	50.52	205.45	-	0.5	0.266666	0.3833333
Exosc7	Q9D0M0	116	IFNNK(*)SSVDLR(*)	K5	53.73	1000	1.2659574	1.014925	1.031914	1.1042659
Cyc1	Q9D0M3	310	HK(*)WSVLK(*)	K2	74.06	98.37	-	0.746666	0.977777	0.8622222
Dynll2	Q9D0M5	9	AVIK(*)NADMSEDMQQDAVDC(+57.	K4	53.74	355.38	0.5963302	-	0.871559	0.733945
Chaf1b	Q9D0N7	123	ENWTVVK(*)TLR(*)	K7	75.12	1000	-	-	6.211111111	
Chaf1b	Q9D0N7	501	TLQTWGK(*)TAPR(*)	K7	61.35	1000	0.7157894	0.884615	-	0.8002024
Chaf1b	Q9D0N7	501	VTLNTLQTWGK(*)TAPR(*)	K11	31.81	1000	0.9789473	1.038461	1.844444	1.2872845
Tars1	Q9D0R2	278	IHK(*)NSSTYWEGK(*)	K3	75.66	159.26	0.9130434	0.948051	1.021739	0.9609449
Tars1	Q9D0R2	123	VNK(*)VVWDLDR(*)PLETDC(+57.02	K3	22.08	209.87	-	-	0.913043478	

Ddx56	Q9D0R4	337	GNK(*)ASDPESGVAR(*)	K3	9.01	1000	-	-	1.417582418	
Lsm12	Q9D0R8	168	IVEK(*)HFR(*)	K4	90.16	1000	0.9148936	0.858974	1.011363	0.9284105
Lsm12	Q9D0R8	168	K(*)IVEK(*)HFR(*)	K5	62.87	69.71	0.8297872	-	-	
Snu13	Q9D0T1	44	K(*)GANEATK(*)TLNR(*)	K8	45.37	51.48	0.8888888	-	0.913043	0.9009662
Snu13	Q9D0T1	86	SK(*)QALGR(*)	K2	8.78	1000	-	-	1.152173913	
Snu13	Q9D0T1	33	LLDLVQQSC(+57.02)NYK(*)QLR(*)	K12	6.76	1000	-	-	1.467391304	
Ppil1	Q9D0W5	37	TC(+57.02)K(*)NFAELAR(*)	K3	40.09	1000	0.7959183	0.597402	1.3	0.8977737
Serpinb1a	Q9D154	56	GSTAAQLSK(*)TFHFDSVEDIHSR(*)	K9	37.45	1000	-	-	3.03125	
Ints12	Q9D168	369	IGSGNSTSPSVPLK(*)PLPPLTLGK(*)T	K23	12.62	46.57	-	-	0.70754717	
Ints12	Q9D168	63	ISSTK(*)SLSIK(*)	K5	12.05	28.89	-	0.460869	1.122641	0.7917555
Gatd3a	Q9D172	217	ALGAK(*)HC(+57.02)VK(*)	K5	54.33	76.73	0.9690721	0.773333	1.011764	0.9180567
Cndp2	Q9D1A2	402	ALK(*)TVFGVEPDLTR(*)	K3	52.14	1000	1.2584269	1.013333	0.880434	1.0507317
Cndp2	Q9D1A2	203	NK(*)PC(+57.02)ITYGLR(*)	K2	46.48	1000	1.3146067	0.893333	1.130434	1.1127916
Ube2c	Q9D1C1	121	DK(*)WSALYDVR(*)	K2	56	1000	0.8571428	0.917647	1.208333	0.9943744
Tmed10	Q9D1D4	127	IPDQLVILDMK(*)HGVEAK(*)	K11	79.65	62.39	0.9714285	0.695121	1.111111	0.9258872
Tmed10	Q9D1D4	133	HGVEAK(*)NYEEIAK(*)	K6	37.52	128.65	1.0952380	1.012195	1.266666	1.1247
Tmed10	Q9D1D4	75	THLK(*)ITDSAGHILYAK(*)	K4	36.2	184.16	0.4857142	1.658536	0.933333	1.0258614
Agpat5	Q9D1E8	201	GLAVLK(*)HVLTPR(*)	K6	50.85	1000	-	-	-	
Rab1b	Q9D1G1	122	LLVGNK(*)SDLTTK(*)	K6	86.17	78.19	0.9130434	0.792207	1.058139	0.9211303
Get4	Q9D1H7	58	YMSQSK(*)HAEAR(*)	K6	30.02	1000	-	1.397435	-	
Get4	Q9D1H7	209	NK(*)NSALVVFTTYTQK(*)	K2	27.3	265.73	-	1.717948	-	
Sarnp	Q9D1J3	17	LAELK(*)QEC(+57.02)LAR(*)	K5	80.87	1000	0.9814814	1.189873	0.934065	1.0351403
	Q9D1K7	59	VGFLK(*)ILHR(*)	K5	37.29	1000	1.2261904	1.903225	1.105263	1.4115598
Sec13	Q9D1M0	285	ILAVSGGDNK(*)VTLWK(*)	K10	57.64	107.29	0.84375	0.642857	0.946236	0.8109479
Sec13	Q9D1M0	38	SVK(*)IFDVR(*)	K3	47.21	1000	0.8229166	0.785714	1.043010	0.8838806
Eef1e1	Q9D1M4	166	QHLSSIVFIK(*)NR(*)	K10	38.65	1000	1.0396039	1.024096	0.833333	0.9656779
Mrpl13	Q9D1P0	114	LAIYGMLPK(*)NLHR(*)	K9	17.3	1000	-	-	0.964028777	
Erp44	Q9D1Q6	140	QQK(*)SNPVHEIQSLDEVTNLDR(*)	K3	12.99	1000	-	-	1.64516129	
Kti12	Q9D1R2	143	AETGGK(*)SQAAGAVEEQR(*)	K6	52.17	1000	0.8173076	1.619047	0.793478	1.0766112
Rpl34	Q9D1R9	72	TQK(*)HVSR(*)	K3	82.63	1000	-	0.987179	-	
Rpl34	Q9D1R9	43	APK(*)SAC(+57.02)GVC(+57.02)PG	K3	76.28	1000	0.9647058	0.910256	0.829545	0.9015026
Fam114a1	Q9D281	333	LELLK(*)NDLISIK(*)	K5	38.75	102.62	-	-	1.011235955	
Fam114a1	Q9D281	254	TK(*)TLMER(*)	K2	6.06	1000	-	-	1.348314607	
Polr3h	Q9D2C6	69	LEDAYVFPGDGASHTK(*)VHFR(*)	K16	27.95	1000	-	-	-	

Znf687	Q9D2D7	1044	LILEK(*)HVQVR(*)	K5	34.95	1000	-	-	-	
Dlst	Q9D2G2	268	HK(*)DAFLK(*)	K2	38.71	105.53	1.3854166	1.064102	0.978260{	1.1425934
Ube2v2	Q9D2M8	66	IYSLK(*)VEC(+57.02)GSK(*)	K5	61.05	78.19	0.6422764	0.923076	0.943181{	0.8361784
Ube2v2	Q9D2M8	8	A(+42.01)VSTGVK(*)VPR(*)	K7	27.5	1000	0.7073170	0.948717	-	0.8280175
Aacs	Q9D2R0	530	INPK(*)TGGIIMLGR(*)	K4	91.42	1000	0.9670329	0.897435	0.966292:	0.943587
Mau2	Q9D2X5	86	THLQLGSVLYHHTK(*)NSEQAR(*)	K14	25.63	1000	-	-	0.741071429	
Acp1	Q9D358	113	AK(*)IELLGSYDPQK(*)	K2	43.88	183.46	-	0.730769	0.806451(	0.7686104
Ephx1	Q9D379	334	FSTWTK(*)SEYR(*)	K6	62.04	1000	0.7111111	1	-	0.8555556
Ephx1	Q9D379	288	EK(*)VFYNIMR(*)	K2	11.14	1000	0.8888888	-	-	
Rufy3	Q9D394	405	ALK(*)HELAFK(*)	K3	21.66	129.72	-	-	-	
Stag1	Q9D3E6	611	MEK(*)HLDALLK(*)	K3	38.79	93.6	0.9421487	-	-	
Stag1	Q9D3E6	626	FVVEK(*)HVESDVLEAC(+57.02)SK(*)	K5	26.71	98.81	0.9256198	-	0.449541:	0.6875806
Pus10	Q9D3U0	477	LHLK(*)TQAGTYIK(*)	K4	38.1	66.1	-	-	0.722772277	
Pus10	Q9D3U0	125	GFITK(*)VC(+57.02)QK(*)	K5	10.71	42.68	-	1.752212	-	
Cul2	Q9D4H8	317	MIEELQK(*)HIHDEGLR(*)	K7	66.81	1000	1.0212765	-	0.924731:	0.9730039
Cul2	Q9D4H8	472	QAC(+57.02)GYEFTSK(*)LHR(*)	K10	5.67	1000	-	-	1.397849462	
Phf6	Q9D4J7	299	AC(+57.02)VK(*)TYHYHC(+57.02)G\	K4	53.58	48.34	1.3877551	0.9	0.876404	1.0547199
Phf6	Q9D4J7	97	LMC(+57.02)SLC(+57.02)HC(+57.0:	K18	34.72	1000	0.8367346	-	0.887640	0.8621876
Sf3a3	Q9D554	264	LK(*)SALLALGLK(*)	K2	79.97	187.38	0.7575757	0.666666	0.848837:	0.7576932
Sf3a3	Q9D554	291	GK(*)SLESLDTSLFAK(*)	K2	63.34	287.12	0.8787878	0.820512	1.139534{	0.9462785
Atad1	Q9D5T0	274	LILK(*)NENVDR(*)	K4	73.56	1000	1.2142857	-	1.072727:	1.1435065
Sec23b	Q9D662	331	ATK(*)HYEMLANR(*)	K3	30.55	1000	-	-	0.79787234	
Cers5	Q9D6K9	113	GLSK(*)QLDWSVR(*)	K4	12.63	1000	-	0.775510	-	
Cers5	Q9D6K9	85	DSPVNK(*)VEPNDTLEK(*)	K6	10.39	58.98	-	-	0.934210526	
Ppil3	Q9D6L8	80	K(*)FEDEYSEYLK(*)HNVR(*)	K11	18.5	62.68	-	1.034482	1.04	1.0372414
Drap1	Q9D6N5	63	NAK(*)TMTTSHLK(*)	K3	43.41	89.48	-	-	0.693069307	
Idh3a	Q9D6R2	200	SNVTAVHK(*)ANIMR(*)	K8	30.1	1000	-	-	1.382022472	
Nosip	Q9D6T0	190	TVTC(+57.02)PMSGK(*)PLR(*)	K9	14.56	1000	1.6136363	1.179104	0.739495	1.1774122
Msra	Q9D6Y7	183	VLSK(*)HNFGPITTDIR(*)	K4	51.43	1000	1.6	1.013888	0.986111:	1.2
Nop56	Q9D6Z1	134	LHFHNLVK(*)GLTDLSAC(+57.02)K(*	K8	60.7	130.84	1.3636363	0.585365	1.03125	0.9934174
Trir	Q9D735	143	TEDEVLTSK(*)GDAWAK(*)	K9	80.37	98.75	1.025	0.729729	-	0.8773649
Ppil2	Q9D787	483	QGVGK(*)YIHPAATK(*)	K5	23.63	54.92	0.6489361	0.568181	1.036144!	0.7510875
Gpx8	Q9D7B7	84	FTDK(*)SYQTLR(*)	K4	37.99	1000	-	0.689655	0.927835(	0.8087451
RtcA	Q9D7H3	163	GYYPK(*)GGGEVIVR(*)	K5	62.85	1000	-	0.659793	0.806818:	0.733306

RtcA	Q9D7H3	313	IK(*)TGSVTLHTQTAIHFAEQLAK(*)	K2	25.52	47.89	-	-	1.102272727	
Rpl22l1	Q9D7S7	47	VNGK(*)TGNLGNVVHIER(*)	K4	39.41	1000	0.9797979	-	1.688172(	1.333985
Rpl22l1	Q9D7S7	63	NK(*)ITVVSEK(*)	K2	12.69	83.89	0.9898989	-	0.881720	0.9358097
Chmp5	Q9D7S9	103	DTK(*)TTVDAMK(*)	K3	34.44	70.39	0.4685314	1.164556	-	0.8165442
Med8	Q9D7W5	259	TNIK(*)SASMHPYQR(*)	K4	40.72	1000	1.1267605	0.810810	1.654545	1.1973723
Med8	Q9D7W5	255	MPSGIK(*)TNIK(*)	K6	19.68	63.97	1.5633802	1.027027	1.963636	1.5180146
Ggct	Q9D7X8	88	MNK(*)SNISSLDEQEGVK(*)	K3	56.13	144.36	1.0303030	0.535211	0.926315	0.83061
Ppa1	Q9D819	213	NK(*)DFAVDIIK(*)	K2	70.75	161.77	0.9484536	0.802469	-	0.8754614
Ppa1	Q9D819	128	HTGC(+57.02)C(+57.02)GDNDPIDV	K19	67.04	1000	1.0721649	0.469135	-	0.7706504
Ppa1	Q9D819	194	YK(*)VPDGK(*)PENEFAFNAEFK(*)	K2	66.41	96.93	-	-	1.108695652	
Ppa1	Q9D819	179	LK(*)PGYLEATVDWFR(*)	K2	25.29	1000	-	-	1.554347826	
Rpl37	Q9D823	68	HLK(*)IVYR(*)	K3	79.61	1000	-	-	1.272727273	
Rpl37	Q9D823	31	AYHLQK(*)STC(+57.02)GK(*)	K6	67.47	95.62	1.0543478	-	1.287878	1.1711133
Rpl37	Q9D823	14	NK(*)THTLC(+57.02)R(*)	K2	50.43	1000	0.6086956	-	1.181818	0.8952569
Fip1l1	Q9D824	270	ASSSVGK(*)WQDR(*)	K7	71.85	1000	0.9767441	0.736111	0.943820	0.8855585
Dnajb4	Q9D832	3	GK(*)DYYHILGIDK(*)	K2	41.11	168.06	-	0.612903	-	
Ppih	Q9D868	137	C(+57.02)DWLDGK(*)HVVFGK(*)	K7	19.42	57.71	-	1.306666	0.822222	1.0644444
U2af1	Q9D883	15	DK(*)VNC(+57.02)SFYFK(*)	K2	92.24	161.78	1.2020202	0.987341	0.956521	1.0486279
Chmp4b	Q9D8B3	14	LFGAGGGK(*)AGK(*)	K8	79.59	32.97	1.1829268	0.8625	1.144578	1.063335
Rpl4	Q9D8E6	106	MFAPTK(*)TWR(*)	K6	95.94	1000	0.9894736	0.913580	1.011363	0.9714725
Rpl4	Q9D8E6	274	AASLK(*)SNYNLPMHK(*)	K5	85.36	222.06	0.8736842	0.728395	0.852272	0.8181173
Rpl4	Q9D8E6	320	K(*)NPLK(*)NLR(*)	K5	81.99	82.63	0.8105263	-	-	
Rpl4	Q9D8E6	333	LNPYAK(*)TMR(*)	K6	81.59	1000	-	-	1.011363636	
Rpl4	Q9D8E6	364	LEAAATALATK(*)SEK(*)	K11	81.16	40.63	0.8736842	0.728395	0.977272	0.859784
Rpl4	Q9D8E6	14	PLISVYSEK(*)GESSGK(*)	K9	75.79	37.35	0.9052631	0.802469	0.909090	0.8722744
Rpl4	Q9D8E6	140	YAIC(+57.02)SALAASALPALVMSK(*)	K19	73.17	1000	1.0526315	-	1.011363	1.0319976
Rpl4	Q9D8E6	364	K(*)LEAAATALATK(*)SEK(*)	K12	71.19	34.87	0.9263157	0.876543	0.965909	0.9229227
Rpl4	Q9D8E6	20	GESSGK(*)NVTLPAVFK(*)	K6	62.79	181.07	0.7894736	0.802469	0.988636	0.8601931
Rpl4	Q9D8E6	175	LK(*)AWNDIK(*)	K2	10.07	95.35	1.0210526	-	0.977272	0.9991627
Rpl7l1	Q9D8M4	104	IEGVSLLVK(*)STIMK(*)	K9	41.39	87.95	0.8404255	-	0.804123	0.8222746
Phf10	Q9D8M7	156	EYPAK(*)HAEYSVILQEK(*)	K5	24.13	55.94	-	-	1.52	
Eef1g	Q9D8N0	434	AVNQGK(*)IFK(*)	K6	98.56	68.47	0.90625	0.902439	0.903225	0.9039716
Eef1g	Q9D8N0	147	ILGLLDTHLK(*)TR(*)	K10	97.96	1000	1.03125	0.926829	0.935483	0.964521
Eef1g	Q9D8N0	285	AK(*)DPFAHLPK(*)STFVLDEFK(*)	K10	77.81	109.88	0.9270833	0.402439	1.010752	0.7800917

Eef1g	Q9D8N0	277	AK(*)DPFAHLPK(*)	K2	72.84	166.48	0.8125	0.878048	0.838709(	0.8430862
Eef1g	Q9D8N0	285	DPFAHLPK(*)STFVLDEFK(*)	K8	66.67	178.84	1.0208333	-	0.526881	0.7738575
Eef1g	Q9D8N0	285	DPFAHLPK(*)STFVLDEFK(*)R(*)	K8	49.56	112.57	-	1.292682	0.935483	1.1140834
Eef1g	Q9D8N0	285	AHLPK(*)STFVLDEFK(*)	K5	38.58	123.34	-	1.146341	0.870967	1.0086546
Eef1g	Q9D8N0	147	GLLDTHLK(*)TR(*)	K8	36.74	1000	0.8333333	-	-	
Eef1g	Q9D8N0	285	AHLPK(*)STFVLDEFK(*)R(*)	K5	31.16	140.79	0.75	-	1.408602	1.0793011
Eef1g	Q9D8N0	285	AK(*)DPFAHLPK(*)STFVLDEFK(*)R(*	K10	26.64	101.72	1.3958333	1.170731	0.989247	1.1852708
Arfgap3	Q9D8S3	23	SVPTNK(*)VC(+57.02)FDC(+57.02)(	K6	56.11	66.64	0.89	0.506024	-	0.698012
Arfgap3	Q9D8S3	118	IK(*)TLATQATR(*)	K2	24.59	1000	-	1.614457	0.840425	1.2274417
Rexo2	Q9D8S4	160	HMPQFMK(*)HLHYR(*)	K7	66.64	1000	-	-	0.710280374	
Rexo2	Q9D8S4	153	FLDK(*)HMPQFMK(*)	K4	59.54	142.27	0.7377049	0.681415	0.925233	0.7814515
Snx5	Q9D8U8	385	NLIEMSELEIK(*)HAR(*)	K11	56.4	1000	0.8279569	0.731707	0.890109	0.8165914
Snx5	Q9D8U8	192	SVVK(*)SADEVLFSGVK(*)	K4	35.85	160.69	-	1.512195	0.890109	1.2011525
Snx5	Q9D8U8	238	SHK(*)NVADDYIHTAAC(+57.02)LHS	K3	27.13	74.33	-	-	0.505494505	
Hm13	Q9D8V0	61	GK(*)SSSDMPETITSR(*)	K2	86.74	1000	0.6373626	0.683544	0.930232	0.7503798
Psmd12	Q9D8W5	405	TIFAK(*)VDR(*)	K5	86.3	1000	-	0.864197	0.989010	0.9266043
Psmd12	Q9D8W5	147	LTK(*)TLATIK(*)	K3	47.04	95.35	0.7938144	-	0.945054	0.8694347
Ccdc124	Q9D8X2	116	AK(*)SHLELPLEENLNR(*)	K2	45.53	1000	1.1123595	0.861111	0.852631	0.9420341
Ccdc124	Q9D8X2	11	FQGENSK(*)SAAAR(*)	K7	44.02	1000	1.1235955	0.490740	0.842105	0.8188138
Ccdc124	Q9D8X2	44	ELEDAYWK(*)DEDK(*)HVMR(*)	K12	10.98	33.81	-	-	1.010526316	
Efhd2	Q9D8Y0	191	GAK(*)NFFEAK(*)	K3	8.82	51.01	-	-	2.014084507	
Ascc1	Q9D8Z1	95	TSINIPK(*)HGHEGEIVITGQHR(*)	K7	55.95	1000	1.0083333	-	0.735537	0.8719353
Ebna1bp2	Q9D903	157	LQTK(*)QAAMEK(*)	K4	21.89	71.79	1.21875	-	1.944444	1.5815972
	Q9D937	93	AAMEVEAPSK(*)STR(*)	K10	63.67	1000	1.2079207	0.693333	1.078651	0.9933019
Echdc1	Q9D9V3	123	GLIIHGAK(*)NTFC(+57.02)SGSDLN/	K8	12.47	67.58	1.2025316	0.703389	0.923913	0.9432782
Dda1	Q9D9Z5	13	GLPVYNK(*)SNFSR(*)	K7	43.46	1000	0.5795454	0.400862	-	0.4902038
Cir1	Q9DA19	3	GK(*)SFANFMC(+57.02)K(*)	K2	86.43	180.8	0.8372093	0.376923	0.515923	0.5766853
Exosc1	Q9DAA6	96	VHILYVGSTPLK(*)NAFR(*)	K12	19.45	1000	-	-	1.135416667	
Tmem263	Q9DAM7	68	SLEVTK(*)TAVTTVPSMGIGLVK(*)	K6	17.66	73.26	-	-	1.032608696	
Dcps	Q9DAR7	137	TTVVYPATEK(*)HLQK(*)	K10	70.4	38.26	0.9183673	1.014705	0.965116	0.9660632
Dcps	Q9DAR7	127	HLSDIK(*)TTVVYPATEK(*)	K6	16.45	88.74	-	1.470588	-	
Gng12	Q9DAS9	34	VSK(*)ASADLMSYC(+57.02)EEHAR(*	K3	71.14	1000	1.0843373	0.819444	0.911764	0.9385155
Gng12	Q9DAS9	4	S(+42.01)SK(*)TASTNSIAQAR(*)	K3	28.93	1000	-	1.430555	0.862745	1.1466503
Prpf4	Q9DAW6	215	TSQMQELHK(*)SLR(*)	K9	34.09	1000	-	-	0.848101266	

Cnn3	Q9DAW9	17	GPSYGLSAEVK(*)NK(*)	K11	86.27	36.05	0.9	0.740740	0.932584	0.857775	
Cnn3	Q9DAW9	256	VASQK(*)GMSVYGLGR(*)	K5	85.43	1000	0.02	0.666666	-	0.3433333	
Gon4l	Q9DB00	2091	HIC(+57.02)GK(*)AGSQSWLIESR(*)	K5	10.57	1000	-	-	-		
Napa	Q9DB05	22	VK(*)NSQSFFSGLFGGSSK(*)	K2	46.6	57.6	0.8350515	-	-		
Mrpl12	Q9DB15	153	EIK(*)NYVQGINLVQAK(*)	K3	46.82	204.01	1.29	2.152542	0.571428	1.3379903	
Atp5po	Q9DB20	90	VK(*)SLNDITK(*)	K2	84.22	157.88	0.8469387	0.841463	0.903225	0.863876	
Atp5po	Q9DB20	162	TVLK(*)SFLSPNQILK(*)	K4	83.55	206.72	0.8571428	0.731707	0.881720	0.8235235	
Atp5po	Q9DB20	176	LEIK(*)TDPSIMGGMIVR(*)	K4	50.45	1000	-	-	0.784946237		
Mcts1	Q9DB27	18	ENVSNC(+57.02)IQLK(*)TSVIK(*)	K10	68.03	55.6	0.7904761	0.794871	1.012195	0.8658477	
Chmp2a	Q9DB34	94	IQTLK(*)SNNSMAQAMK(*)	K5	38.8	168.62	-	-	1.552083333		
Cyb5r1	Q9DB73	81	HILGLPVGK(*)HVYLSAR(*)	K9	39.18	1000	-	-	0.684210526		
Uqcrc2	Q9DB77	92	LASSLTTK(*)GASSFK(*)	K8	56.55	33.98	0.6435643	-	-		
Uqcrc2	Q9DB77	183	IIENLHDVAYK(*)NALANPLYC(+57.0	K11	56.27	1000	-	-	0.85106383		
Ngdn	Q9DB96	74	LMDLSHLILDK(*)ASGASLQGHPAVL	K11	59.47	1000	-	-	0.968421053		
Ngdn	Q9DB96	115	LVK(*)TAVTGSLSENDPLR(*)	K3	42.77	1000	0.8875	-	1.168421	1.0279605	
Gtf2h1	Q9DBA9	239	LNTGSK(*)DLFAEC(+57.02)AK(*)	K6	24.14	135.81	-	-	1.676470588		
Prkar1a	Q9DBC7	134	AIEK(*)NVLFSHLDDNER(*)	K4	49.94	1000	0.8202247	0.712328	1.137931	0.8901615	
Prkar1a	Q9DBC7	70	QIQC(+57.02)LQK(*)TGIR(*)	K7	46.05	1000	0.8314606	0.712328	1.034482	0.8594241	
Prkar1a	Q9DBC7	24	EC(+57.02)ELYVQK(*)HNIQALLK(*)	K8	42.17	21.68	1.4269662	0.712328	1.310344	1.14988	
Prkar1a	Q9DBC7	261	VSILESLDK(*)WER(*)	K9	19.02	1000	-	-	1.24137931		
Aldh7a1	Q9DBF1	124	EK(*)IQLLGR(*)	K2	6.22	1000	-	-	1.173913043		
Plin3	Q9DBG5	199	VLVK(*)SEAWADNR(*)	K4	82.04	1000	0.8556701	0.972222	1.024390	0.9507609	
Plin3	Q9DBG5	170	TK(*)SAMTSGVQSVMGSR(*)	K2	72.75	1000	0.9278350	0.972222	1.073170	0.991076	
Rpn2	Q9DBG6	31	LTPTHYLTK(*)QDVER(*)	K9	73.41	1000	0.8191489	0.684210	0.858695	0.7873517	
Rpn2	Q9DBG6	442	LHNQK(*)TGQEVVFVAEPDNK(*)	K5	30.2	153.62	1.2765957	1.157894	0.695652	1.0433809	
Rpn2	Q9DBG6	392	ISTEVGITNVDLSTVDK(*)DQSIAPK(*)	K24	10.94	34.75	-	-	1.47826087		
Srpra	Q9DBG7	219	EEFIQK(*)HGK(*)	K6	51.62	57.45	0.5913978	0.597222	1.0625	0.7503734	
Srpra	Q9DBG7	329	GLVGSK(*)SLSR(*)	K6	35.86	1000	0.5806451	-	-		
Pgam1	Q9DBJ1	113	HGEAQVK(*)IWR(*)	K7	77.85	1000	0.8829787	-	1.172413	1.0276963	
Pgam1	Q9DBJ1	100	HYGGLTGLNK(*)AETAAK(*)	K10	64.86	139.02	1.0212765	0.986301	0.965517	0.9910317	
Akap8	Q9DBR0	527	SVLNNK(*)HIVK(*)	K6	79.87	55.36	0.7338709	0.655172	1.142857	0.8439668	
Akap8	Q9DBR0	404	SFEDEEIQK(*)HLQSK(*)	K9	65.96	38.19	-	0.551724	0.880952	0.7163383	
Akap8	Q9DBR0	521	TSLHVAK(*)SVLNNK(*)	K7	60.31	143.14	-	0.804597	0.988095	0.8963465	
Akap8	Q9DBR0	421	FISTK(*)LPDK(*)	K5	51.33	49.79	0.75	0.597701	0.928571	0.7587575	

Xrn2	Q9DBR1	368	LVNIYK(*)NVVHK(*)	K6	74.58	81.69	0.8494623	0.734177	0.872340	0.81866
Xrn2	Q9DBR1	189	LNNDPGWK(*)NLTVILSDASAPGEGE	K8	23.51	35.38	-	-	0.70212766	
Xrn2	Q9DBR1	373	NVVHK(*)TGGYLTESGYVNLQR(*)	K5	22.12	1000	1.1505376	0.949367	-	1.0499524
Xrn2	Q9DBR1	286	GK(*)HDELADSLPC(+57.02)AEGEFI	K2	20.73	1000	-	-	0.914893617	
Armc8	Q9DBR3	352	R(*)LDHDLK(*)HAHELR(*)	K7	14.36	1000	-	1.819672	-	
Apbb2	Q9DBR4	242	TGAK(*)TDC(+57.02)ALHR(*)	K4	15.76	1000	-	-	1.370786517	
Ppp1r12a	Q9DBR7	197	HAK(*)SGGTALHVAAAK(*)	K3	36.34	223.58	1.4193548	-	1	1.2096774
Osbpl3	Q9DBS9	128	VK(*)SEELFDEWVSK(*)	K2	13.36	223.6	1.5631067	1.5375	1.543209	1.5479389
Ampd2	Q9DBT5	28	SLPGNAPC(+57.02)LK(*)HFPLDLR(	K10	45.46	1000	-	-	0.702970297	
Ampd2	Q9DBT5	348	VDTHIHASSC(+57.02)MNQK(*)HLL	K14	28.85	1000	-	0.873684	1.019801	0.9467431
Ampd2	Q9DBT5	732	VK(*)SHWLGPNYTK(*)	K2	21.61	148.29	1.3551401	1.273684	1.128712	1.2525124
Riok3	Q9DBU3	293	VFK(*)TTLNEFK(*)	K3	54.73	83.89	0.6074766	0.571428	-	0.5894526
Riok3	Q9DBU3	165	DITTK(*)HDEVVC(+57.02)GR(*)	K5	39.38	1000	0.9813084	0.495238	1	0.8255155
Dnajc10	Q9DC23	392	TLLK(*)NEHIQVGR(*)	K4	60.43	1000	1.0425531	0.738095	1	0.9268828
Dnajc10	Q9DC23	325	EK(*)SSVLFLNSLDAK(*)	K2	33.31	184.16	-	1.452380	1	1.2261905
Cdc40	Q9DC48	551	AHDK(*)VC(+57.02)IGAVWHPHETS	K4	38.9	153.62	-	0.913580	1	0.9567901
Crot	Q9DC50	489	MLEAFAK(*)HNK(*)	K7	38.14	56.34	-	-	1	
Gnai3	Q9DC51	46	LLLLGAGESGK(*)STIVK(*)	K11	94.23	100.21	0.8155339	0.858974	0.964705	0.8797381
Gnai3	Q9DC51	180	VK(*)TTGIVETHFTFK(*)	K2	75.21	160.66	0.7864077	0.769230	0.941176	0.8322717
Gnai3	Q9DC51	92	LK(*)IDFGESAR(*)	K2	59.26	1000	0.7864077	0.935897	1.082352	0.934886
Pmpca	Q9DC61	242	EVLHSYLK(*)NYYTPDR(*)	K8	67.44	1000	-	0.573170	0.977777	0.7754743
Mrps11	Q9DCA2	149	VVVK(*)GMGPGR(*)	K4	47.36	1000	-	-	1.46	
Brix1	Q9DCA5	110	LFVINEVC(+57.02)EMK(*)NC(+57.0	K11	32.23	52.86	1.0879120	-	1.690476	1.3891941
Pycr3	Q9DCC4	225	MLQQEGK(*)HPAQLR(*)	K7	67.57	1000	1.2738095	0.802469	0.96875	1.0150096
Tomm20	Q9DCC8	35	SDPNFK(*)NR(*)	K6	38.74	1000	0.4031007	-	-	
Pgd	Q9DCD0	38	TVSK(*)VDDFLANEAK(*)	K4	69.36	234.4	0.9670329	0.864197	1.147727	0.9929859
Pgd	Q9DCD0	308	GPK(*)VVQLEGSK(*)	K3	47.76	114.79	0.8021978	0.938271	0.795454	0.845308
Pgd	Q9DCD0	119	AK(*)GILFVGSGVSGGEEGAR(*)	K2	35.03	1000	1.1098901	-	-	
Xab2	Q9DCD2	794	AQSK(*)IFFVR(*)	K4	28.7	1000	1.2871287	0.934210	0.660550	0.9606299
Pak1ip1	Q9DCE5	161	SAFIK(*)NIK(*)	K5	26.8	29.32	-	-	1.104651163	
Trmt112	Q9DCG9	2	MK(*)LLTHNLLSSHVR(*)	K2	67.24	1000	0.7311827	0.855263	1.091954	0.8928
Zfand6	Q9DCH6	201	YSDVHNC(+57.02)SYNYK(*)ADAAEK	K12	13.04	28.13	-	-	1.441176471	
Ppp1r2	Q9DCL8	18	NK(*)TSAASPPVVPSAEQPR(*)	K2	41.06	1000	0.9320388	-	2.290322	1.6111807
Ppp1r2	Q9DCL8	18	NK(*)TSAASPPVVPSAEQPR(*)PIVEE	K2	22.5	101.47	-	-	1.322580645	

Paics	Q9DCL9	261	VELLLK(*)SDSQC(+57.02)R(*)	K6	71.86	1000	0.8315789	0.714285	1.077777	0.8745475
Paics	Q9DCL9	53	K(*)NHLEGK(*)AAISNK(*)	K7	51.11	85.43	0.9578947	-	-	
Paics	Q9DCL9	53	NHLEGK(*)AAISNK(*)	K6	36.89	121.92	0.9789473	1.051948	0.866666	0.965854
Paics	Q9DCL9	125	FYPPK(*)VEMFFK(*)	K5	11.01	37.35	-	2.272727	-	
Cyb5r3	Q9DCN2	126	FPAGGK(*)MSQYLENMK(*)	K6	67.52	154.34	0.6464646	0.513157	0.977011	0.7122113
Mecr	Q9DCS3	267	LALNC(+57.02)VGGK(*)SSTELLR(*)	K9	78.51	1000	0.7757009	-	0.864583	0.8201421
Ndufb10	Q9DCS9	134	ELEQFTK(*)VTK(*)	K7	28.12	55.63	0.9887640	0.695121	1.048192	0.9106929
Sdf2	Q9DCT5	75	GK(*)TATVC(+57.02)ER(*)	K2	79.11	1000	1.0309278	-	0.938271	0.9845997
Sdf2	Q9DCT5	151	FK(*)HSSTDVLLSVTGEQYGR(*)	K2	44.13	1000	-	1.304878	0.691358	0.998118
Etfb	Q9DCW4	35	SGVVTDGVK(*)HSMNPFC(+57.02)E	K9	60.29	1000	-	-	1.123595506	
Etfb	Q9DCW4	116	EK(*)VDLLFLGK(*)	K2	29.54	175.25	0.8829787	0.987179	1.112359	0.9941726
Atp5pd	Q9DCX2	32	AIGNALK(*)SWNETFHAR(*)	K7	72.03	1000	0.9387755	0.829268	0.912087	0.8933772
Atp5pd	Q9DCX2	32	ALK(*)SWNETFHAR(*)	K3	36.3	1000	1.2551020	0.939024	0.945054	1.0463938
Rab13	Q9DD03	71	FK(*)TITTAYYR(*)	K2	2.32	1000	-	-	0.023564065	
Sacm1l	Q9EP69	483	YYK(*)NNFSDGFR(*)	K3	44.06	1000	-	-	0.776595745	
Sacm1l	Q9EP69	456	QYAGTGALK(*)TDFTR(*)	K9	12.48	1000	7.3373493	-	18.02127	12.679313
Sacm1l	Q9EP69	435	TYK(*)NAWADNANAC(+57.02)AK(*)	K3	10.94	143.73	1.3493975	2.395061	-	1.8722297
Rai14	Q9EP71	693	AK(*)SEEALSEMK(*)	K2	59.1	203.33	1.3516483	1.090909	0.853932	1.09883
Rai14	Q9EP71	54	HDSEGK(*)TAFHLAAAK(*)	K6	29.53	99.51	1.6483516	1.428571	1.303370	1.460098
Rai14	Q9EP71	109	LLQYK(*)SPAENIDNSGK(*)	K5	24.41	77.35	0.8241758	1.194805	1.460674	1.1598851
Emc7	Q9EP72	126	YVNYIK(*)TSEVVR(*)	K6	26.92	1000	-	-	1.390243902	
Wdr4	Q9EP82	244	QLQC(+57.02)C(+57.02)DLAGLQEP	K20	65.74	1000	1.0322580	0.684782	0.625899	0.78098
Senp3	Q9EP97	440	WTK(*)NVDIFNK(*)	K3	52.66	111.09	0.6086956	0.666666	0.603603	0.626322
Senp3	Q9EP97	41	LK(*)SGGGFGPDPGSGTTVPTR(*)	K2	22.02	1000	0.6869565	1.372549	-	1.0297528
Parva	Q9EPC1	274	TLITFVNK(*)HLNK(*)	K8	80.6	97.69	0.9714285	0.921052	0.857142	0.9165414
Parva	Q9EPC1	355	PR(*)PEDIVNC(+57.02)DLK(*)STLR(	K12	18.73	1000	0.9238095	-	-	
Arfgap1	Q9EPJ9	285	VQGVGSK(*)GWR(*)	K7	86.6	1000	0.7802197	0.902777	0.829787	0.8375949
Arfgap1	Q9EPJ9	114	DK(*)VATLAEGK(*)	K2	38.02	115.01	-	-	0.755319149	
Xpo7	Q9EPK7	338	LK(*)SNYQLGELVK(*)	K2	71.91	213.35	0.7111111	0.710526	0.920454	0.7806973
Ipo7	Q9EPL8	119	VQLTTC(+57.02)IHHIIK(*)HDYPSR(*	K12	95.52	1000	0.9139784	0.817073	0.925531	0.8855279
Ipo7	Q9EPL8	489	FK(*)SDQNLQTALELTR(*)	K2	68.13	1000	1.1612903	0.682926	0.595744	0.8133206
Upf1	Q9EPU0	486	FTAQGLPDLNHSQVYAVK(*)TVLQR(*	K18	57.25	1000	0.8695652	-	1.052631	0.9610984
Upf1	Q9EPU0	840	EK(*)DFIILSC(+57.02)VR(*)	K2	30.86	1000	1.6630434	-	-	
Upf1	Q9EPU0	434	MQSALK(*)TFAVDETSVSGYIYHK(*)	K6	14.42	34.72	-	-	1.052631579	

Cpsf1	Q9EPU4	807	LVFLVK(*)NFPVGQR(*)	K6	51.58	1000	0.7941176	-	0.966666(	0.8803922
Hsd17b11	Q9EQ06	282	FLQVLK(*)HR(*)	K6	73.9	1000	0.8611111	0.75	0.927083:	0.8460648
Pold3	Q9EQ28	386	SK(*)TFVDEEGC(+57.02)IVTEK(*)	K2	9.55	126.28	-	-	5.103448276	
Pes1	Q9EQ61	98	AYGK(*)SEWNAVER(*)	K4	82.64	1000	1.0561797	0.698795	0.822916(	0.8592972
Pes1	Q9EQ61	152	TGK(*)C(+57.02)HVQTIQLC(+57.02)	K3	79.05	1000	1	0.855421	0.916666(	0.9240295
Pes1	Q9EQ61	49	GIYPHEPK(*)HK(*)	K8	54.13	33.18	1	0.518072	0.770833:	0.7629685
Nif3l1	Q9EQ80	105	HITWK(*)TWK(*)	K5	63.44	34.94	-	-	1.207317073	
Nif3l1	Q9EQ80	100	PMK(*)HITWK(*)	K3	53.63	127.18	0.9897959	1.012820	1.146341	1.0496526
Nif3l1	Q9EQ80	180	SQDLDK(*)VMSTLR(*)	K6	42.43	1000	-	0.679487	-	
Nif3l1	Q9EQ80	233	QLYQK(*)TEILSLEK(*)	K5	12.74	97.31	-	1	-	
Scyl1	Q9EQC5	410	EQTVK(*)SMLLLAPK(*)	K5	41.07	123.53	0.9887640	0.519480	1.336734(	0.9483264
Cert	Q9EQG9	48	WVVLK(*)NNTLSYYK(*)	K5	9.84	36.2	-	1.674418	-	
Vps35	Q9EQH3	44	LMDALK(*)HASNMLGELR(*)	K6	67.3	1000	0.7894736	-	1.076923(	0.9331984
Vps35	Q9EQH3	419	LK(*)HFHPLFEYFDYESR(*)	K2	55.16	1000	1.1263157	-	0.967032!	1.0466744
Vps35	Q9EQH3	768	IR(*)EDLPNLESSEETEQINK(*)HFHNT	K19	45	1000	0.8315789	1.298701	1.087912(	1.0727308
Vps35	Q9EQH3	296	VK(*)NIIIALIDR(*)	K2	40.25	1000	-	-	0.89010989	
Vps35	Q9EQH3	639	MK(*)C(+57.02)FSEENHEPLR(*)	K2	20.61	1000	2.0315789	-	-	
Vps35	Q9EQH3	38	NK(*)LMDALK(*)	K2	12.56	102.87	0.9473684	-	-	
Vps35	Q9EQH3	768	EDLPNLESSEETEQINK(*)HFHNTLEHL	K17	11.04	1000	-	-	1.230769231	
Mvp	Q9EQK5	33	VEVGPK(*)TYIR(*)	K6	35.51	1000	1.3061224	0.828571	0.780219	0.9716379
Mvp	Q9EQK5	444	GTAK(*)VLQPSAAR(*)	K4	10.23	1000	-	-	0.824175824	
Set	Q9EQU5	131	VEVTEFEDIK(*)SGYR(*)	K10	71.04	1000	-	0.952941	0.797979	0.8754605
Set	Q9EQU5	166	EFHLNESGDPSSK(*)STEIK(*)	K13	68.57	119.54	0.9285714	0.776470	0.898989	0.8680106
Set	Q9EQU5	27	LEDK(*)SASPGLPK(*)	K4	58.12	131.72	0.7755102	0.682352	0.808080	0.7553147
Set	Q9EQU5	67	LNEQASEEILK(*)VEQK(*)	K11	32.16	77.13	1.2244897	1.258823	0.858585	1.1139664
Aif1l	Q9EQX4	83	LGVPK(*)THLEMK(*)	K5	44.4	77.93	0.5855855	1.030303	0.242514	0.6194679
Stx12	Q9ER00	44	IK(*)NLMSQLGTK(*)	K2	7.85	106.84	-	-	0.943820225	
Stx12	Q9ER00	53	NLMSQLGTK(*)QDSSK(*)	K9	6.73	47.14	-	-	0.595505618	
Cars	Q9ER72	495	SLK(*)NFITIK(*)	K3	74.58	102.92	0.7714285	0.643678	0.867346	0.7608179
Cars	Q9ER72	575	WEAEEVELNK(*)NFYGK(*)	K10	29.02	33.54	-	-	0.734693878	
Dap3	Q9ER88	182	FDQPLEASTWLK(*)NFK(*)	K12	46.57	24.24	-	-	0.813953488	
Dap3	Q9ER88	127	GTGK(*)TLSLC(+57.02)HAVHFC(+57	K4	27.75	1000	-	-	0.895348837	
Tfip11	Q9ERA6	171	GLGK(*)NAQGIINPIEAK(*)	K4	49.75	220.17	1.52	1.21875	-	1.369375
Tfip11	Q9ERA6	188	GK(*)GAVGAYGSER(*)	K2	19.32	1000	1.2	0.953125	1.0125	1.0552083

Lima1	Q9ERG0	466	DLWASK(*)SDNEETLGR(*)	K6	60.74	1000	-	-	1.157303371	
Lima1	Q9ERG0	210	GEHNQTK(*)SLWTQSR(*)	K7	59.05	1000	1.0192307	0.958904	1.101123!	1.0264195
Lima1	Q9ERG0	292	QSSPASYTNELK(*)TSESK(*)	K12	34.23	45.16	-	-	0.685393258	
Lima1	Q9ERG0	437	IYC(+57.02)K(*)PHFNQLFK(*)	K4	32.18	86.34	0.75	-	1.730337(	1.2401685
Lima1	Q9ERG0	393	ESC(+57.02)VEC(+57.02)QK(*)TVYP	K8	19.67	1000	1.4615384	-	1.213483:	1.3375108
Strn3	Q9ERG2	710	FFDNK(*)TGK(*)	K5	25.48	28.96	-	-	1.2	
Strn3	Q9ERG2	597	SGIK(*)NQLLSC(+57.02)SADGTIR(*)	K4	24.21	1000	-	-	0.788888889	
Cse1l	Q9ERK4	67	VC(+57.02)ASVTFK(*)NYIK(*)	K8	78.3	35.82	0.84375	0.8375	0.922222	0.8678241
Cse1l	Q9ERK4	427	HK(*)DAAIYLVTSLASK(*)	K2	55.07	216.84	0.9375	0.75	0.988888	0.8921296
Cse1l	Q9ERK4	165	HEFK(*)SNELWTEIK(*)	K4	49.95	165.61	0.8333333	0.7375	0.888888	0.8199074
Cse1l	Q9ERK4	425	NPSVNWK(*)HK(*)	K7	40.24	26.31	-	-	0.977777778	
Ranbp2	Q9ERU9	2018	DFK(*)TFLTNDQVK(*)	K3	50.06	95.39	0.8260869	-	0.989130	0.9076087
Ranbp2	Q9ERU9	106	IAELLC(+57.02)K(*)NDVTDGR(*)	K7	48.01	1000	0.9239130	-	-	
Ranbp2	Q9ERU9	1510	EWHC(+57.02)SLC(+57.02)SVK(*)N	K10	47.34	81.81	0.8586956	0.96	1.054347	0.9576812
Ranbp2	Q9ERU9	1133	SDDMFAFHGPGK(*)SVFTTAASELAN	K12	44.03	232.04	1.2065217	-	0.989130	1.0978261
Ranbp2	Q9ERU9	365	GK(*)QDFLK(*)	K2	39.59	107.29	-	-	1.130434783	
Ranbp2	Q9ERU9	776	SADSELK(*)HSTPSPTK(*)	K7	36.02	78.75	-	-	1.684782609	
Ranbp2	Q9ERU9	2938	GFGFK(*)NSIFHR(*)	K5	31.22	1000	1.7934782	-	1.336956!	1.5652174
Ranbp2	Q9ERU9	3014	K(*)AEHLDFK(*)HVVFGFVK(*)	K8	10.05	26.08	3.1304347	-	2.967391:	3.048913
Arhgef7	Q9ES28	514	LPTTGMTITK(*)LEDSENHR(*)	K10	36.37	1000	1.1724137	-	-	
Arhgef7	Q9ES28	704	VIEAYC(+57.02)TSAK(*)TR(*)	K10	13.71	1000	-	-	1.396226415	
C1qtnf3	Q9ES30	200	GK(*)SDTSSNHAVLK(*)	K2	0.7	215.31	-	-	-	
Parvb	Q9ES46	267	SLITFVNK(*)HLNK(*)	K8	45	77.51	1.5060240	1.802325	1.041322:	1.4498907
Nek7	Q9ES74	74	VQIFDLMDAK(*)AR(*)	K10	12.95	1000	0.7159090	-	0.694117(	0.7050134
Rtn3	Q9ES97	829	VYK(*)SVIQAVQK(*)	K3	91.89	216.51	0.7425742	0.841463	0.793814	0.7926174
Rtn3	Q9ES97	930	YK(*)TQIDHYVGIAR(*)	K2	83.08	1000	0.8514851	0.987804	0.907216	0.9155022
Rtn3	Q9ES97	837	SVIQAVQK(*)SEEGHPFK(*)	K8	39.11	58.11	1.2574257	1.243902	0.773195	1.091508
Xpo4	Q9ESJ0	647	WAK(*)TYLLVDEK(*)	K3	8.66	66.68	-	1.048780	-	
Psmg2	Q9EST4	188	IPGGGITK(*)TLYDESC(+57.02)SK(*)	K8	72.31	130.84	0.7087378	0.489583	0.689655	0.6293255
Psmg2	Q9EST4	149	YLLTPC(+57.02)LQK(*)SVQNK(*)	K9	19.99	46.62	0.8737864	1.104166	-	0.9889765
Brd4	Q9ESU6	1233	IK(*)NMGSWASLVQK(*)	K2	87.42	245.03	0.9655172	0.21	1	0.7251724
Brd4	Q9ESU6	1254	HPTTPSSTAK(*)SSSDSFEHFR(*)	K10	80.88	1000	1.1954022	0.29	1.013157	0.8328534
Brd4	Q9ESU6	76	TLWK(*)HQFAWPFQQPVDAVK(*)	K4	36.44	132.44	-	-	1.026315789	
Brd4	Q9ESU6	1053	HHK(*)SDPYSAGHLR(*)	K3	19.96	1000	-	-	1.263157895	

Brd4	Q9ESU6	72	VVLK(*)TLWK(*)	K4	18.67	97.69	1.2183908	0.36	1.210526	0.929639
Ddx24	Q9ESV0	143	VGGAK(*)SESLAQAAPR(*)	K5	48.5	1000	1.2072072	0.770270	-	0.9887387
Dkc1	Q9ESX5	144	LVK(*)SQQSAGK(*)	K3	65.31	120.29	-	-	0.97752809	
Gtf2i	Q9ESZ8	130	ALGK(*)STVVPVPYEK(*)	K4	50.82	147.65	0.7916666	1.012820	-	0.9022436
Tm9sf3	Q9ET30	97	FK(*)DDVMPGTYC(+57.02)EIDLDK(	K2	42.59	172.8	1.1630434	-	1.032608	1.0978261
Palld	Q9ET54	923	FK(*)EDLLNNGQPR(*)	K2	34.85	1000	5.8636363	6.957142	2.783132	5.2013039
Palld	Q9ET54	1069	QISPK(*)SDHYTIQR(*)	K5	31.05	1000	1.0909090	1.442857	1.349397	1.2943879
Palld	Q9ET54	1056	PK(*)IYWFK(*)	K2	25.49	142.99	0.875	-	0.963855	0.9194277
Elf2	Q9JHC9	341	GGK(*)NSSPLNC(+57.02)SR(*)	K3	16.07	1000	2.0555555	-	-	
Copz2	Q9JHH9	74	EQMVFEK(*)NVFNK(*)	K7	50.19	29.96	-	0.476190	-	
Ivd	Q9JHI5	76	AQEIDQTNDFK(*)NLR(*)	K11	47.41	1000	0.6777777	-	1	0.8388889
Exosc9	Q9JHI7	227	VMDGLLVIAMNK(*)HR(*)	K12	13.88	1000	1.7722772	-	-	
Tmod3	Q9JHJ0	230	TLEANTHVK(*)HFSLAATR(*)	K9	84.8	1000	1.1397849	1.082191	0.988505	1.0701608
Tmod3	Q9JHJ0	337	AANAITK(*)NNDLVR(*)	K7	71.7	1000	1.0107526	0.972602	0.931034	0.9714633
Tmod3	Q9JHJ0	221	DFAK(*)TLEANTHVK(*)	K4	42.92	143.22	-	-	1.011494253	
Tmod3	Q9JHJ0	58	NQTSK(*)SATGPFDR(*)	K5	38.57	1000	0.6881720	1.712328	0.908045	1.1028489
Poglut2	Q9JHP7	361	HISFFDFFK(*)HK(*)	K9	53.29	22.85	0.9677419	0.8625	1.070588	0.9669434
Poglut2	Q9JHP7	202	VYIK(*)THGEHVGFR(*)	K4	39.57	1000	-	1.175	1.152941	1.1639706
Poglut2	Q9JHP7	115	IEVK(*)HHGQHVAESPYVLR(*)	K4	20.54	1000	-	1.65	1.552941	1.6014706
Poglut2	Q9JHP7	412	HYIPVK(*)SNLSDLLEK(*)	K6	11.34	54.49	-	-	2.494117647	
Lztfl1	Q9JHQ5	244	SLEENLAAAK(*)HDLLR(*)	K10	52.68	1000	-	-	0.62601626	
Ide	Q9JHR7	483	VAIVSK(*)SFEGK(*)	K6	68.62	95.62	0.7956989	0.897435	1.760869	1.1513348
Ide	Q9JHR7	192	EVNAVDSEHEK(*)NVMNDAWR(*)	K11	66.49	1000	-	-	1.02173913	
Ide	Q9JHR7	436	GYTSK(*)IAGK(*)	K5	34.68	23.44	-	0.769230	-	
Ide	Q9JHR7	558	DTAMSK(*)LWFK(*)	K6	19.7	46.21	-	1.256410	-	
Cwc15	Q9JHS9	18	GK(*)GEGDLSQLSK(*)	K2	54.8	235.31	0.7529411	0.560747	0.509090	0.6075932
Cwc15	Q9JHS9	195	WDDDVVFK(*)NC(+57.02)AK(*)	K8	20.77	38.03	-	0.579439	0.845454	0.7124469
Dync1h1	Q9JHU4	954	IK(*)NVVHELR(*)	K2	86.61	1000	0.84375	0.911392	0.912087	0.8890768
Dync1h1	Q9JHU4	622	FK(*)VQYPQSQAC(+57.02)K(*)	K2	82.58	158.08	0.9479166	0.784810	0.846153	0.8596269
Dync1h1	Q9JHU4	574	DQLGTAK(*)NANEMFR(*)	K7	67.72	1000	0.8125	0.835443	0.934065	0.8606697
Dync1h1	Q9JHU4	1990	EHSNPNYDK(*)TSAPITC(+57.02)ELL	K9	62.83	186.68	0.8229166	0.721518	0.846153	0.7968632
Dync1h1	Q9JHU4	3230	SQELEVK(*)NAAANDK(*)	K7	62.36	136.94	-	-	0.879120879	
Dync1h1	Q9JHU4	3223	IK(*)SQELEVK(*)	K2	59.59	186.64	0.8229166	1.025316	0.945054	0.931096
Dync1h1	Q9JHU4	3469	ADLAAVEAK(*)VNR(*)	K9	57.74	1000	0.7916666	0.848101	0.934065	0.8579446

Dync1h1	Q9JHU4	1494	GWDDLFNK(*)VK(*)	K8	55.97	21.68	1.3020833	-	1	1.1510417
Dync1h1	Q9JHU4	2092	ALK(*)SVLVSAGNVK(*)	K3	53.68	145.28	1.2916666	-	0.879120	1.0853938
Dync1h1	Q9JHU4	3891	LK(*)GTVGEPTYDAEFQHFLR(*)	K2	48.49	1000	-	-	0.978021978	
Dync1h1	Q9JHU4	870	SLETC(+57.02)MYDHK(*)TFSEILNR(	K10	41.82	1000	-	-	0.967032967	
Dync1h1	Q9JHU4	3489	WEK(*)TSETFK(*)	K3	41.61	58.16	0.78125	0.455696	0.593406	0.6101176
Dync1h1	Q9JHU4	3755	SLLQALNEVK(*)GR(*)	K10	39.54	1000	-	-	0.43956044	
Dync1h1	Q9JHU4	1412	HWK(*)QLMK(*)	K3	37.41	109.97	-	-	1.252747253	
Dync1h1	Q9JHU4	1402	INMLVIELK(*)SEALK(*)	K9	36.27	45.16	-	-	1	
Dync1h1	Q9JHU4	1113	VQSK(*)VNLK(*)	K4	27.38	59.3	-	-	1.087912088	
Dync1h1	Q9JHU4	669	R(*)VEDVLGK(*)GWENHVEGQK(*)	K8	15.27	57.1	1.28125	1.037974	-	1.1596123
Dync1h1	Q9JHU4	1369	QNLDGLLNQLK(*)NFPAR(*)	K11	11.7	1000	-	-	1.197802198	
Dync1h1	Q9JHU4	1827	SK(*)IDNAK(*)	K2	10.83	95.62	-	-	1.164835165	
Dync1h1	Q9JHU4	2941	QPQGHLLLIGVSGAGK(*)TTLSR(*)	K16	9.85	1000	1.375	-	-	
Nit2	Q9JHW2	109	LYNTC(+57.02)SVFGPDGSLLVK(*)H	K17	40.49	1000	0.8522727	0.824324	0.432098	0.7028986
Eefsec	Q9JHW4	254	VK(*)SMQMFHTPVTSAMQGDR(*)	K2	60.31	1000	0.6631578	0.861111	0.989130	0.8377998
Ankh	Q9JHZ2	127	LHHVDESVGSK(*)TR(*)	K11	51.48	1000	-	-	0.922222222	
Utp3	Q9JI13	140	LYYDTDYGSK(*)SR(*)	K10	45.36	1000	-	-	1.150537634	
Abcb10	Q9JI39	661	LSTIK(*)NANFVAVLDHGK(*)	K5	48.14	115.9	-	-	1.344262295	
Prmt1	Q9JIF0	134	ANK(*)LDHVVTIIK(*)	K3	84.75	246.46	0.9793814	0.851851	0.819148	0.8834607
Copb1	Q9JIF7	932	AK(*)SQGMALSLGDK(*)	K2	73.91	175.94	0.4680851	-	0.382022	0.4250538
Copb1	Q9JIF7	366	EVIK(*)TNNVSEHEDTDK(*)	K4	7.74	69.38	-	-	1.595505618	
Ccdc22	Q9JIG7	423	LIHLASQWEK(*)HR(*)	K10	56.57	1000	1.4301075	0.848837	0.876288	1.0517445
Praf2	Q9JIG8	152	IENK(*)IESIGLK(*)	K4	65.54	157.88	0.8152173	0.845070	1.052631	0.9043065
Nup50	Q9JIH2	448	VK(*)TSEDADELHK(*)	K2	64.23	226.64	-	0.714285	0.891566	0.802926
Dazap1	Q9JII5	194	DSK(*)NQAPGQPGASQWGSR(*)	K3	19.22	1000	1.4444444	-	1.133333	1.2888889
Akr1a1	Q9JII6	127	GDNPFPK(*)NADGTVR(*)	K7	90.5	1000	0.7959183	1.119047	0.857142	0.9240363
Akr1a1	Q9JII6	240	HPDEPVLLEEPVVLALAEK(*)HGR(*)	K19	78.64	1000	1.0408163	0.726190	0.923076	0.8966946
Akr1a1	Q9JII6	263	VIC(+57.02)IPK(*)SINPSR(*)	K6	74.23	1000	0.8571428	0.880952	1	0.9126984
Akr1a1	Q9JII6	23	MPLIGLGTWK(*)SEPGQVK(*)	K10	65.59	83.89	0.9285714	0.75	0.857142	0.8452381
Akr1a1	Q9JII6	85	LWNTK(*)HHPEDVEPALR(*)	K5	54.11	1000	1.2346938	0.845238	1.197802	1.0925781
Akr1a1	Q9JII6	34	AAIK(*)HALSAGYR(*)	K4	51.23	1000	1.0816326	0.821428	0.989010	0.9640241
Akr1a1	Q9JII6	263	K(*)VIC(+57.02)IPK(*)SINPSR(*)	K7	26.21	76.27	0.7040816	0.809523	1.120879	0.8781615
Ddx21	Q9JIK5	39	SK(*)TEEATEGMEEAVSSK(*)	K2	95.57	337.17	0.9795918	0.901234	0.959183	0.94667
Ddx21	Q9JIK5	287	GVNFLFPIQAK(*)TFHHVYSGK(*)	K11	68.34	38.72	0.9081632	0.753086	0.887755	0.8496683

Ddx21	Q9JIK5	463	YMK(*)STYEQVDLIGK(*)	K3	66.4	232.86	-	0.913580	0.918367	0.9159738
Ddx21	Q9JIK5	296	TFHHVYSGK(*)DLIAQAR(*)	K9	53.05	1000	-	0.839506	-	
Ddx21	Q9JIK5	308	TGTGK(*)TFSFAIPLIEK(*)	K5	52.08	213.66	0.6836734	-	0.632653	0.6581633
Ddx21	Q9JIK5	742	VK(*)GMVFLK(*)	K2	34.97	129.19	0.6530612	0.962962	0.969387	0.861804
Ddx21	Q9JIK5	185	LK(*)NGLSQPSEEEVDIPK(*)PK(*)	K2	33.97	102.18	-	-	1.030612245	
Ddx21	Q9JIK5	148	LK(*)NGLSQPSEEEADIPK(*)PK(*)	K2	15.93	132.44	-	-	1.408163265	
Mrps34	Q9JIK9	144	LVPK(*)HEEEAFTAFTAK(*)PEDR(*)	K4	36.22	85.14	1.31	1.265822	-	1.2879114
Ercc6l2	Q9JIM3	905	IPK(*)NHIR(*)	K3	33.83	1000	-	-	-	
Diablo	Q9JIQ3	189	NHIQLVK(*)SQVQEVR(*)	K7	44.48	1000	0.9292929	1.051948	1.256410	1.0792171
Slc12a4	Q9JIS8	393	GEVVEK(*)HGLPSTDTLGLK(*)	K6	43.5	63.99	-	0.685393	-	
Ralb	Q9JIW9	115	VK(*)SEEDK(*)IPLLVVGNK(*)	K2	57.49	38.19	-	-	0.93814433	
Ralb	Q9JIW9	16	SQGSLVLHK(*)VIMVGSGGVGK(*)	K9	49.48	97.58	0.4943820	-	0.886597	0.69049
Ralb	Q9JIW9	167	ANVDK(*)VFFDLMR(*)	K5	29.89	1000	0.8988764	-	0.350515	0.6246959
Ralb	Q9JIW9	146	GK(*)AEEWGVQYVETSAK(*)	K2	21.53	172.92	-	-	1.391752577	
Eny2	Q9JIX0	74	GLEHVTVDDLVAEITPK(*)GR(*)	K17	39.27	1000	0.5887850	-	-	
Acin1	Q9JIX8	1048	IK(*)SHC(+57.02)FVTYSTVEEAVATR(	K2	41.22	1000	1.1313131	1.602739	1.333333	1.3557954
Htra2	Q9JIY5	395	EPSFPDVQHGVLIHK(*)VILGSPAHR(	K15	39.56	1000	0.8918918	-	0.953488	0.9226901
Flii	Q9JJ28	92	ANSLK(*)NSGVPDDIFK(*)	K5	42.64	150.82	0.7676767	-	1.115789	0.9417331
Pbk	Q9JJ78	89	ILK(*)NLNHPNIIGYR(*)	K3	21.02	1000	1.3953488	2.214285	1.103448	1.5710276
Rpf2	Q9JJ80	31	LTENIK(*)NAMLIK(*)	K6	32.38	129.15	0.8979591	-	0.608695	0.7533274
Ccdc86	Q9JJ89	38	ALVDFK(*)SNSEETGELK(*)	K6	54.16	153.52	1.3111111	-	0.964285	1.1376984
Wdr12	Q9JJA4	291	VWDVESGGLK(*)STLTGNK(*)	K10	62.45	118.11	0.8539325	0.650602	0.948453	0.8176629
Wdr12	Q9JJA4	44	LLETK(*)NELHK(*)	K5	38.07	41.96	0.8988764	0.759036	4.164948	1.9409537
Wdr12	Q9JJA4	214	MLK(*)IWSTVPTDEEDEMEEATNR(*)	K3	22.6	1000	-	-	0.865979381	
Wdr12	Q9JJA4	49	NELHK(*)HVEFDFLIK(*)	K5	9.03	22.83	-	-	3.340206186	
Rpl38	Q9JJI8	57	LK(*)QSLPPGLAVK(*)	K2	31	113.72	-	1.406976	0.709677	1.0583271
Lancl2	Q9JJK2	166	LK(*)SEC(+57.02)ESQEC(+57.02)ITK	K2	66.3	267.5	1.0215053	1.078947	1.096774	1.0657423
Lancl2	Q9JJK2	177	SEC(+57.02)ESQEC(+57.02)ITK(*)LL	K11	25.14	1000	-	-	1.688172043	
Phax	Q9JJT9	330	QAIK(*)SLNFQEDDDTSR(*)	K4	64.78	1000	0.3885350	0.443037	0.537634	0.4564025
Ddx20	Q9JJY4	519	LPVK(*)SHSEC(+57.02)GVLEK(*)	K4	8.4	30.19	-	0.959459	-	
Psmg1	Q9JK23	265	C(+57.02)LVK(*)NIPESTEILK(*)	K4	38.09	54.53	-	-	0.868131868	
Psmg1	Q9JK23	194	ALK(*)TQTFK(*)	K3	27.42	105.53	-	1.293333	0.934065	1.1136996
Sh3glb1	Q9JK48	188	AAETK(*)SSSEQELR(*)	K5	26.91	1000	0.6767676	0.888888	-	0.7828283
Sh3glb1	Q9JK48	48	TELDAHLENLLSK(*)AEC(+57.02)TK(	K13	23.57	33.54	1.0808080	-	0.816091	0.94845

Myg1	Q9JK81	254	FK(*)VDSSGEIVELAK(*)	K2	61.92	267.48	0.8064516	0.794520	1.034883	0.8786186
Myg1	Q9JK81	266	VDSSGEIVELAK(*)GGC(+57.02)PWK	K12	42.85	78.19	0.6559139	1.273972	0.790697	0.9068614
Hspb8	Q9JK92	137	QQEGGIVSK(*)NFTK(*)	K9	55.31	28.7	-	1.223880	0.894117	1.0589991
Uchl3	Q9JKB1	74	IK(*)SQGQDVTSSVYFMK(*)	K2	26	208.29	2.3695652	1.602409	0.707865	1.5599467
Ybx3	Q9JKB3	82	VLATK(*)VLGTVK(*)	K5	78.38	1000	0.8367346	1.608695	1.214285	1.2199054
Iqgap1	Q9JKF1	687	WVK(*)HWVK(*)	K3	86.35	82.24	0.8125	0.746835	0.977777	0.8457044
Iqgap1	Q9JKF1	1475	LTELGTVDPK(*)NR(*)	K10	60.48	1000	0.8333333	0.772151	0.977777	0.8610877
Iqgap1	Q9JKF1	1128	LPYDVTPEQALSHEEVK(*)TR(*)	K17	52.3	1000	1.8645833	-	1.733333	1.7989583
Iqgap1	Q9JKF1	1088	SLNIK(*)TDPVDIYK(*)	K5	48.95	118.44	1.3854166	-	0.777777	1.0815972
Iqgap1	Q9JKF1	924	NK(*)ITLQDVVSHSK(*)	K2	38.35	94.57	-	-	1.244444444	
Iqgap1	Q9JKF1	1532	SYIK(*)TC(+57.02)LDNLASK(*)	K4	36.91	158.68	0.6875	1.139240	1.666666	1.1644691
Iqgap1	Q9JKF1	88	LGNFFSPK(*)VVSLK(*)	K8	34.78	26.93	0.3333333	-	-	
Iqgap1	Q9JKF1	922	IGLLVK(*)NK(*)	K6	33.88	27.16	0.7708333	1.037974	1.311111	1.039973
Iqgap1	Q9JKF1	1037	SK(*)VDQIQEIVTGNPTVIK(*)	K2	31.09	157.79	0.78125	-	1.033333	0.9072917
Iqgap1	Q9JKF1	104	YK(*)ATGLHFR(*)	K2	11.73	1000	1.2604166	-	-	
Mrpl39	Q9JKF7	233	YK(*)VDFIEEK(*)	K2	37.29	142.27	1.8955223	1.440677	1.307692	1.5479642
Mrpl39	Q9JKF7	69	HVGK(*)TDPGTVFVMNK(*)	K4	19.73	112.22	-	2.237288	-	
Hyou1	Q9JKR6	106	YFQHLLGK(*)QADNPHVALYR(*)	K8	36.05	1000	0.3473684	1.578947	0.617977	0.8480978
Adrm1	Q9JKV1	113	LFFWMQEPK(*)TDQDEEHC(+57.02)	K9	48.7	1000	-	-	0.880434783	
Aatf	Q9JKX4	243	GGPEFASALK(*)NSHK(*)	K10	39.57	34.3	0.7156862	0.774647	1.265486	0.918607
Aatf	Q9JKX4	349	LGK(*)GFGAFER(*)	K3	23.22	1000	0.5686274	1.408450	-	0.9885391
Nudt5	Q9JKX6	13	ESTESSPGK(*)HLVTSEELISEGK(*)	K9	80.53	67.58	0.8125	0.795180	0.947368	0.851683
Nudt5	Q9JKX6	56	GK(*)SADAVSVIPVLQR(*)	K2	47.11	1000	1.8645833	0.807228	0.768421	1.1467444
Nudt5	Q9JKX6	32	FEK(*)TTYMDPTGK(*)	K3	38.08	142.42	1.1979166	0.831325	0.968421	0.999221
Hip1r	Q9JKY5	261	FHEQFHSLK(*)NFFR(*)	K9	28.4	1000	-	-	-	
Slc5a3	Q9JKZ2	290	VLAAK(*)NIAHAK(*)	K5	84.45	129.19	0.8876404	0.831168	0.936170	0.8849932
Slc5a3	Q9JKZ2	665	FIDWFC(+57.02)GFK(*)SK(*)	K9	25.76	24.32	1.2696629	-	1.829787	1.5497251
Aldh9a1	Q9JLJ2	368	LK(*)HGYYMTPC(+57.02)ILTNC(+57	K2	43.23	1000	1.4155844	1.32	0.949494	1.2283598
Dclk1	Q9JLM8	155	NVNPNWSVNVK(*)TTSASR(*)	K11	33.67	1000	1.9797979	-	0.758620	1.3692093
Cul3	Q9JLV5	542	FYLAK(*)HSGR(*)	K5	84.56	1000	0.8	1	0.836956	0.8789855
Cul3	Q9JLV5	68	NAYTMVLHK(*)HGEK(*)	K9	64.82	55.36	-	0.987012	0.945652	0.9663326
Cul3	Q9JLV5	271	ELISK(*)HMK(*)	K5	51.83	28.96	0.95	0.766233	0.858695	0.8583098
Cul3	Q9JLV5	459	LK(*)TEC(+57.02)GC(+57.02)QFTSK	K2	46.11	164.65	0.77	0.883116	0.663043	0.7720535
Cul3	Q9JLV5	700	VDDDR(*)K(*)HEIEAAIVR(*)	K6	44.48	1000	-	-	1.065217391	

Cul3	Q9JLV5	254	VMHC(+57.02)LDK(*)STEEPIVK(*)	K7	34.3	65.33	1.08	1.259740	1	1.1132468
Cul3	Q9JLV5	444	LLTNK(*)SVSDDSEK(*)	K5	5.29	36.2	-	-	2.304347826	
Pias4	Q9JM05	118	LPTK(*)TLK(*)PEVR(*)	K4	55.54	53.53	0.6017699	-	-	
Rabgef1	Q9JM13	213	IMDQIEK(*)HIMTR(*)	K7	35.57	1000	-	-	0.596638655	
Rabgef1	Q9JM13	151	TFHK(*)TGQEVYK(*)	K4	25.19	96.04	-	0.804597	0.4873949	0.6459963
Arpc3	Q9JM76	56	ANVFFK(*)NYEIK(*)	K6	96.14	111.23	0.8229166	0.756097	0.8901098	0.8230414
Arpc3	Q9JM76	61	NYEIK(*)NEADR(*)	K5	89.27	1000	1.125	1.024390	1.2747252	1.1413718
Arpc3	Q9JM76	123	PASK(*)QEDEMMR(*)	K4	49.76	1000	0.8125	0.707317	0.8131868	0.777668
Usp14	Q9JMA1	313	NALYIK(*)SSK(*)	K6	59.77	28.96	1.3736263	0.864864	1.6136363	1.2840425
Edf1	Q9JMG1	25	SK(*)QAILAAQR(*)	K2	35.29	1000	0.6744186	0.717647	0.9767441	0.7896033
Edf1	Q9JMG1	98	DLATK(*)INEK(*)PQVIADYESGR(*)	K5	32.1	49.79	-	1.235294	0.9186046	1.0769494
Txnrd1	Q9JMH6	209	VEDTVK(*)HDWEK(*)	K6	51.8	79.02	1.1276595	1.084337	1.0112359	1.074411
Txnrd1	Q9JMH6	203	NYGWK(*)VEDTVK(*)	K5	47.17	54.91	0.7765957	0.867469	0.9213483	0.855138
Txnrd1	Q9JMH6	369	QFVPTK(*)IEQIEAGTPGR(*)	K6	33.5	1000	1.9468085	-	1.0337078	1.4902582
Txnrd1	Q9JMH6	349	GFDQDMANK(*)IGEHMEEHGIK(*)	K9	15.9	20.33	2.3085106	-	0.8651685	1.5868396
Myo18a	Q9JMH9	1723	AK(*)TALEEQLSR(*)	K2	35.26	1000	0.8202247	1.839506	0.8315789	1.1637699
Csnk1e	Q9JMK2	122	IEYIHSK(*)NFIHR(*)	K7	56.38	1000	-	0.634408	-	
Csnk1e	Q9JMK2	45	TK(*)HPQLHIESK(*)	K2	15.05	181.94	-	0.784946	-	
Polk	Q9QUG2	460	LK(*)NVNFEVK(*)	K2	3.97	100.91	-	-	0.935897436	
Rhoa	Q9QUI0	104	WTPEVK(*)HFC(+57.02)PNVPIILVGI	K6	90.85	114.83	0.6938775	0.666666	0.9010989	0.753881
Rhoa	Q9QUI0	162	IGAFGYMEC(+57.02)SAK(*)TK(*)	K12	70.87	27.16	0.9489795	0.864197	0.9450549	0.9194107
Acsl4	Q9QUJ7	223	AALVDINC(+57.02)VK(*)HIIYVDNK(	K10	34.63	40.87	0.9333333	-	0.9130434	0.9231884
Psma6	Q9QUM9	45	GK(*)DC(+57.02)AVIVTQK(*)	K2	45.27	203.33	0.8282828	0.740259	0.9550561	0.8411996
Psma6	Q9QUM9	104	YK(*)YGYEIPVDMLC(+57.02)K(*)	K2	37.79	182.53	0.6969696	0.792207	0.8988764	0.796018
Prep	Q9QUR6	588	FHK(*)FTIGHAWTTDYGC(+57.02)SD	K3	55.17	50.24	0.7894736	-	-	
Prep	Q9QUR6	183	FTC(+57.02)MAWTHDGK(*)GMFYN	K11	36.5	90.21	1.2315789	-	-	
Prep	Q9QUR6	196	GMFYNSYPQQDGK(*)SDGTETSTNLH	K13	35.82	24.74	-	-	0.641304348	
Prep	Q9QUR6	157	FMK(*)VDGAK(*)	K3	26.21	87.95	0.8421052	0.936708	0.6630434	0.8139525
Prep	Q9QUR6	449	DGTK(*)IPMFIVHK(*)	K4	11.46	100.36	-	1.101265	-	
Prep	Q9QUR6	677	QSNPLLIHVDTK(*)AGHGAGK(*)PTAI	K12	8.85	26.08	-	-	3.054347826	
Pin1	Q9QUR7	65	C(+57.02)SHLLVK(*)HSQSR(*)	K7	49.35	1000	-	0.911392	1.1	1.0056962
Pin1	Q9QUR7	84	SK(*)EEALELINGYIQK(*)	K2	27.08	251.71	0.5578947	0.683544	0.6888888	0.6434426
Naga	Q9QWR8	321	SK(*)SHIEVFK(*)	K2	35.65	209.49	0.5806451	-	1.0879120	0.8342786
Kifc1	Q9QWT9	63	VMGAVTK(*)VDTSR(*)PR(*)	K7	88.03	1000	1.0744680	0.864406	0.9204545	0.9531098

Kifc1	Q9QWT9	63	VMGAVTK(*)VDTSR(*)	K7	69.9	1000	1.0425531	0.542372	0.977272	0.8540663
Kifc1	Q9QWT9	660	FASK(*)VNQC(+57.02)VIGTAQANK(	K4	36.33	205.45	0.7978723	1.050847	0.909090	0.9192702
Ccnt1	Q9QWV9	491	VHSAGDK(*)HNSIEDSVTK(*)	K7	74.49	66.12	0.5229885	0.475609	0.718446	0.5723483
Ccnt1	Q9QWV9	395	AK(*)HAEELAAQK(*)	K2	36.03	164.46	-	-	0.757281553	
Ccnt1	Q9QWV9	556	HSSQTSTLAHK(*)TYSLSSTLSSSSSTR(	K11	32.25	1000	0.4137931	0.548780	0.611650	0.5247414
Ccnt1	Q9QWV9	475	AVSSK(*)PEEIK(*)	K5	18.91	38.77	0.5804597	-	0.941747	0.7611037
Son	Q9QX47	2073	LTDLDK(*)AQLLEIAK(*)	K6	95.88	217.23	1.2	0.973333	0.936842	1.0367251
Son	Q9QX47	2073	R(*)LTDLDK(*)AQLLEIAK(*)	K7	77.52	216.51	1.1	1.106666	0.821052	1.0092398
Son	Q9QX47	2388	DLSGK(*)HPVSALMEIC(+57.02)NK(	K5	34.8	129.85	-	-	0.947368421	
Son	Q9QX47	141	LK(*)SHHDGNLESDSFLK(*)	K2	17.28	153.62	-	0.8	-	
Cyhr1	Q9QXA1	298	LLAAK(*)NINLR(*)	K5	36.44	1000	-	-	-	
Drg2	Q9QXB9	59	GEGFDVMK(*)SGDAR(*)	K8	20.89	1000	0.7021276	0.820512	-	0.7613202
Tbl1x	Q9QXE7	453	GVC(+57.02)IHTLTK(*)HQEPVYSVAF	K9	58.82	197.28	1.0813953	-	1.105882	1.0936389
Chm	Q9QXG2	93	DK(*)TIQHVEVFC(+57.02)YASQDLH	K2	14.21	151.7	-	-	1.329896907	
Chm	Q9QXG2	270	YAEFK(*)NITR(*)	K5	8.63	1000	-	3.492957	1.268041	2.3804995
Cpsf3	Q9QXK7	100	TFMTHATK(*)AIYR(*)	K8	26.85	1000	-	-	0.81443299	
Plec	Q9QXS1	1733	ASFAEK(*)TAQLER(*)	K6	86.61	1000	0.8936170	-	0.788888	0.841253
Plec	Q9QXS1	197	WVNK(*)HLIK(*)	K4	83.42	67.69	0.9148936	1.153846	0.944444	1.0043947
Plec	Q9QXS1	2263	LK(*)QSAEEQAQAQAQAQAAAEK(*)	K2	81.69	295.37	0.8085106	0.705128	0.9	0.8045463
Plec	Q9QXS1	1994	LK(*)TEAEIALK(*)	K2	77.69	175.25	0.9574468	1	0.788888	0.9154452
Plec	Q9QXS1	1251	STQGAEEVLK(*)THEEQLK(*)	K10	74.62	27.33	1.0106382	0.743589	0.933333	0.8958538
Plec	Q9QXS1	1234	LK(*)TISLVIR(*)	K2	74.16	1000	0.9361702	0.923076	0.888888	0.9160453
Plec	Q9QXS1	2107	SK(*)EQAELEAAR(*)	K2	73.67	1000	1.0638297	0.987179	1.222222	1.0910772
Plec	Q9QXS1	2033	LEEQAALHK(*)ADIEER(*)	K9	71.69	1000	1.3191489	0.679487	0.833333	0.9439898
Plec	Q9QXS1	2033	R(*)LEEQAALHK(*)ADIEER(*)	K10	68.92	1000	0.8829787	0.705128	1.588888	1.0589986
Plec	Q9QXS1	2910	EGVVGPELHHK(*)LLSAER(*)	K11	67.18	1000	1	0.756410	0.8	0.8521368
Plec	Q9QXS1	349	HK(*)PMLIDMNK(*)	K2	66.82	112.99	0.9680851	1.025641	1.066666	1.0201309
Plec	Q9QXS1	2314	QAADAEMEK(*)HK(*)	K9	64.77	36.05	0.9680851	1	0.866666	0.9449173
Plec	Q9QXS1	2055	QK(*)GLVEDTLR(*)	K2	57.53	1000	-	-	1	
Plec	Q9QXS1	2714	R(*)K(*)QEELQHLEQQR(*)	K2	54.13	1000	0.8617021	0.743589	1.1	0.901764
Plec	Q9QXS1	969	QVEVTVHK(*)GDQC(+57.02)QLVGP	K8	47.01	121.01	1.1063829	0.794871	0.922222	0.941159
Plec	Q9QXS1	4511	TK(*)MSAAQALK(*)	K2	44.39	141.48	-	0.384615	0.877777	0.6311966
Plec	Q9QXS1	3558	LYVHEAVK(*)AGVVGPELHEK(*)	K8	34.43	80.31	1.0319148	1.089743	0.933333	1.0183306
Plec	Q9QXS1	2086	AAAGK(*)AELELELGR(*)	K5	29.78	1000	1.1276595	-	-	

Plec	Q9QXS1	3391	VEVPVGSFK(*)GR(*)	K9	25.58	1000	0.8510638	0.974358	0.466666(	0.7640298
Plec	Q9QXS1	357	HK(*)PMLIDMNK(*)VYR(*)	K10	24.7	78.17	-	-	0.777777778	
Plec	Q9QXS1	3292	LLDAQLSTGGIVDPSK(*)SHR(*)	K16	22.09	1000	-	-	1.133333333	
Plec	Q9QXS1	592	DLDK(*)ADGMIR(*)	K4	14.15	1000	-	-	1.122222222	
Plec	Q9QXS1	2347	LQLEETDHQK(*)SILDEELQR(*)	K10	13.05	1000	1.1702127	-	-	
Plec	Q9QXS1	4355	QYDIDDAITK(*)NLIDR(*)	K10	13.02	1000	1.1702127	-	-	
Plec	Q9QXS1	247	FHK(*)LQNVQIALDYLR(*)	K3	12.28	1000	-	-	1.288888889	
Plec	Q9QXS1	578	LLASGK(*)VAQR(*)	K6	3.39	1000	-	-	0.788888889	
Macf1	Q9QXZ0	4202	LQQFMENK(*)SR(*)	K8	63.19	1000	1.375	-	-	
Macf1	Q9QXZ0	6226	HHVLK(*)NDVLAHQATVATVNK(*)	K5	25.7	157.56	-	-	1.010869565	
Macf1	Q9QXZ0	6367	GDSGSGSK(*)TEQSVALLEQK(*)	K8	5.35	112.13	-	-	1.108695652	
Vapb	Q9QY76	118	EAK(*)PEDLMDSK(*)LR(*)	K11	29.98	58.11	0.7634408	1.013157	0.681318(	0.8193058
Clic4	Q9QYB1	110	LSPK(*)HPESNTAGMDIFAK(*)	K4	48.73	115.47	-	-	0.547619048	
Clic4	Q9QYB1	130	IK(*)NSR(*)PEANEALER(*)	K2	26.06	1000	-	-	1.357142857	
Clic4	Q9QYB1	130	FSAYIK(*)NSR(*)PEANEALER(*)	K6	16.94	1000	0.8222222	1.342857	0.952380!	1.0391534
Add1	Q9QYC0	288	ILIQK(*)NLGPK(*)	K5	45.88	76.42	-	0.431578	0.449438:	0.4405086
Dnajc7	Q9QYI3	128	ALELDHK(*)NAQAQQEFK(*)	K7	11.64	77.76	-	1.151898	-	
Dnaja2	Q9QYJ0	152	NVLC(+57.02)SAC(+57.02)SGQGGK	K13	79.02	94.27	0.8958333	0.898734	0.927083:	0.9072169
Dnaja2	Q9QYJ0	226	GMK(*)HGQR(*)	K3	68.15	1000	-	0.962025	0.979166(	0.970596
Dnajb1	Q9QYJ3	195	LNPDGK(*)SIR(*)	K6	29.05	1000	1	-	-	
Cenph	Q9QYM8	45	AQTK(*)QQLLEYK(*)	K4	14.76	147.63	-	1.370370	0.562162:	0.9662663
Spast	Q9QYY8	517	LLLLK(*)NLLC(+57.02)K(*)	K5	28.5	119.54	1.3255813	-	0.399014:	0.8622981
Tollip	Q9QZ06	66	LAK(*)NYGMTR(*)	K3	5.93	1000	1.9571428	-	-	
Vps29	Q9QZ88	50	ESYDYLK(*)TLAGDVHIVR(*)	K7	54.56	1000	0.9081632	-	0.946236!	0.9271999
Vps29	Q9QZ88	43	IQHILC(+57.02)TGNLC(+57.02)TK(*	K13	22.71	79.39	1.4489795	2.506172	1.774193!	1.909782
Dctn5	Q9QZB9	21	SEYIETASGNK(*)VSR(*)	K11	27.49	1000	-	1.392857	1.149425:	1.2711412
Slc25a10	Q9QZD8	253	FK(*)GLFPAGIR(*)	K2	13.95	1000	-	0.921052	-	
Eif3i	Q9QZD9	91	QLALLK(*)TNSAVR(*)	K6	71.83	1000	1.1578947	0.795180	0.978723	0.9772663
Eif3i	Q9QZD9	224	LFDSTTLEHQK(*)TFR(*)	K11	55.33	1000	-	0.662650	0.925531!	0.7940913
Eif3i	Q9QZD9	2	MK(*)PILLQGHER(*)	K2	47.69	1000	0.7052631	0.554216	1.085106:	0.7815288
Eif3i	Q9QZD9	282	VK(*)GHFGPINSVAFHPDGK(*)	K2	42.68	209.24	0.9368421	0.975903	1.117021:	1.0099223
Copg1	Q9QZE5	317	VAMK(*)HPSAVTAC(+57.02)NLDLEI	K4	70.92	1000	-	-	0.904255319	
Copg1	Q9QZE5	465	ILHLLGQEGPK(*)TNNPSK(*)	K11	56.66	95.35	0.7628865	0.65	0.797872:	0.7369196
Copg1	Q9QZE5	823	SDK(*)VPENK(*)NTHTLLLAGVFR(*)	K8	42.05	47.14	1.1649484	-	1.095744(	1.1303466

Copg1	Q9QZE5	313	TLNK(*)VAMK(*)	K4	26.86	64.94	-	-	0.85106383	
Copg1	Q9QZE5	262	NK(*)HEMVVYEAASAIVNLPGC(+57.(	K2	7.69	109.58	-	-	0.5	
Tsnax	Q9QZE7	247	LYTLK(*)QSLAK(*)	K5	38.59	47.14	-	-	0.877358491	
Ppie	Q9QZH3	108	FSGK(*)TLEENK(*)EEEGPEPPK(*)	K4	11.95	41.57	-	1.111111	0.769230	0.9401709
Ripk3	Q9QZL0	307	DK(*)VDAAVSEVK(*)HYLSQHR(*)	K11	35.2	95.39	-	-	0.642105263	
Ripk3	Q9QZL0	230	EAELVDK(*)TSLIR(*)	K7	13.3	1000	-	-	1.178947368	
Ubqln2	Q9QZM0	66	FK(*)SQTDQLVLIFAGK(*)	K2	27.2	48.26	-	-	0.921348315	
Afdn	Q9QZQ1	1544	EIHELQNK(*)VDR(*)	K8	5.72	1000	-	-	1.296703297	
H2afy	Q9QZQ8	295	TVK(*)NC(+57.02)LALADDR(*)	K3	74.12	1000	0.6326530	0.717647	1.010526	0.7869421
H2afy	Q9QZQ8	307	LK(*)SIAFPSIGSGR(*)	K2	52.52	1000	0.8571428	-	-	
Zranb2	Q9R020	54	AGGTEIGK(*)TLAEK(*)	K8	21.39	115.97	-	0.666666	1.333333	1
Gyg1	Q9R062	218	TK(*)PWNYTYNPQTK(*)	K2	5.22	67.73	-	-	1.142857143	
Pex14	Q9R0A0	220	SEINSLK(*)GLLLNR(*)	K7	8.96	1000	-	-	0.78313253	
Plod3	Q9R0E1	503	DK(*)GIFLHLSNQHEFGR(*)	K2	61.27	1000	0.6989247	0.653846	1.220930	0.8579004
Plod3	Q9R0E1	218	LSLDHK(*)SR(*)	K6	55.99	1000	1.7849462	-	-	
Plod3	Q9R0E1	648	TYVGPMTEYLFPGYHTK(*)TR(*)	K17	42.73	1000	0.9784946	0.897435	-	0.9379653
Plod1	Q9R0E2	331	HHK(*)LQVEQFLAEHGSEYQSVK(*)	K3	25.69	125.43	-	-	1.099099099	
Ylpm1	Q9R0I7	688	DTPVK(*)SGGLLADPPK(*)	K5	32.26	168.62	-	0.878378	-	
Ylpm1	Q9R0I7	894	DMPTNK(*)VEQIPYGER(*)	K6	6.96	1000	0.4516129	-	-	
Pcm1	Q9R0L6	1073	LK(*)QMLTELMR(*)	K2	53.68	1000	-	-	2.525773196	
Rab9a	Q9R0M6	20	IILLGDGGVGK(*)SSLMNR(*)	K11	34.6	1000	1.2	-	1	1.1
Esd	Q9R0P3	200	WK(*)AYDATC(+57.02)LVK(*)	K2	16.1	154.34	-	3.792682	2.168421	2.980552
Smap	Q9R0P4	62	LVIGDHK(*)STSHFR(*)	K7	63.97	1000	1.0196078	1.134328	1.050632	1.0681897
Dstn	Q9R0P5	19	IFYDMK(*)VR(*)	K6	82.63	1000	0.9619047	0.9875	1.138297	1.0292342
Dstn	Q9R0P5	132	K(*)FPGIK(*)HEYQANGPEDLNR(*)	K6	75.64	45.16	0.9333333	0.675	0.691489	0.7666076
Dstn	Q9R0P5	132	FPGIK(*)HEYQANGPEDLNR(*)	K5	62.67	1000	0.8380952	0.8375	0.882978	0.852858
Dstn	Q9R0P5	92	YALYDASFETK(*)ESR(*)	K11	23.25	1000	-	1.8	-	
Sec11a	Q9R0P6	114	FLTK(*)GDNNAVDDR(*)	K4	67.49	1000	0.7040816	0.473684	0.597938	0.5919013
Sec11a	Q9R0P6	127	GLYK(*)QGQHWLEK(*)	K4	43.29	159.25	0.7448979	1.328947	0.649484	0.9077766
Ptges3	Q9R0Q7	65	HLNEIDLFHC(+57.02)IDPNDSK(*)H	K17	97.79	20.41	1.0989010	0.910256	1	1.0030525
Ptges3	Q9R0Q7	35	SK(*)LTFSC(+57.02)LGGSDNFK(*)	K2	93.52	310.73	1.1758241	0.961538	1.022727	1.0533633
Ptges3	Q9R0Q7	33	DVNVNFEK(*)SK(*)	K8	91.76	36.05	0.9010989	0.807692	0.886363	0.8650516
Acot9	Q9R0X4	155	IDMC(+57.02)K(*)HSLSPEQDIK(*)	K5	74.15	133.99	0.8210526	0.746835	1.034883	0.8675906
Acot9	Q9R0X4	423	HFK(*)SMSTPVTLK(*)	K3	72.8	140.79	-	1.113924	0.953488	1.0337062

Acot9	Q9R0X4	294	LK(*)SLDIC(+57.02)HPQER(*)	K2	54.72	1000	0.7263157	0.835443	0.941860	0.8345398
Acot9	Q9R0X4	183	MK(*)MFQLHNDEK(*)	K2	18.09	204.03	3.8421052	-	2.825581	3.3338433
Ak1	Q9R0Y5	21	IIFVVGGPGSGK(*)GTQC(+57.02)EK(	K12	16.46	26.5	0.7980769	1.2875	-	1.0427885
Sqor	Q9R112	365	TMC(+57.02)LIMK(*)NQR(*)PIK(*)	K7	32.43	77.93	0.744	1.013157	0.571428	0.7761955
Sqor	Q9R112	260	DVSVNYK(*)HNLIEVR(*)PDK(*)	K7	25.54	64.63	0.656	-	0.928571	0.7922857
Naa80	Q9R123	303	DLK(*)GC(+57.02)PIFWMEK(*)	K3	71.28	161.52	-	-	-	
Naa80	Q9R123	242	AFSK(*)PPC(+57.02)PQPPC(+57.02	K4	8.12	31.37	-	-	-	
Mta2	Q9R190	460	QTFLLQTTK(*)LTR(*)	K9	30.18	1000	-	0.639534	0.655913	0.6477244
Prpf40a	Q9R1C7	747	ESAFK(*)SMLK(*)	K5	45.46	20.17	0.8247422	0.827160	0.978021	0.8766416
Prpf40a	Q9R1C7	196	SDSGK(*)PYYYNSQTK(*)	K5	36.87	109.88	0.6288659	-	1.021978	0.825422
Nsdhl	Q9R1J0	115	VNFIGTK(*)TVIETC(+57.02)R(*)	K7	57.95	1000	0.7222222	0.72	0.811111	0.7511111
Psma4	Q9R1P0	199	VLNK(*)TMDVSK(*)	K4	72.96	134.33	-	0.736842	0.666666	0.7017544
Psma4	Q9R1P0	222	ESGK(*)TVIR(*)	K4	5.83	1000	-	-	0.962962963	
Psmb3	Q9R1P1	17	GK(*)NC(+57.02)VAIAADR(*)	K2	27.76	1000	-	1.604938	1.704545	1.6547419
Psmb2	Q9R1P3	29	VAASNIVQMK(*)DDHDK(*)	K10	69.24	30.64	1.1	0.961538	1.034883	1.0321407
Psma1	Q9R1P4	189	HMSEFMEC(+57.02)NLDELVK(*)HG	K15	75.88	1000	0.9423076	-	1.065217	1.0037625
Psma1	Q9R1P4	41	SK(*)THAVLVALK(*)	K2	61.99	276.35	0.8846153	0.8625	0.934782	0.893966
Psma1	Q9R1P4	115	LVSLIGSK(*)TQIPTQR(*)	K8	47.69	1000	-	-	0.847826087	
Sae1	Q9R1T2	57	GLGAEIAK(*)NLILAGVK(*)	K8	69.9	124.34	-	-	0.957446809	
Sae1	Q9R1T2	49	VLIVGMK(*)GLGAEIAK(*)	K7	69.38	77.91	1.06	-	0.691489	0.8757447
Sae1	Q9R1T2	145	DVIIK(*)VDQIC(+57.02)HR(*)	K5	61.43	1000	1.1	1.220779	0.808510	1.0430966
Sae1	Q9R1T2	187	VAK(*)VSQGVEDGPEAK(*)	K3	50.07	243.69	0.91	0.961038	1.170212	1.0137506
Myo1c	Q9WTI7	853	ELC(+57.02)MK(*)NMVWK(*)	K5	68.85	87.95	0.7234042	0.75	0.945054	0.8061531
Myo1c	Q9WTI7	704	HLGYK(*)PEEYK(*)	K5	41.71	95.62	0.7978723	-	-	
Myo1c	Q9WTI7	281	VSSINDK(*)SDWK(*)	K7	31.86	55.36	-	0.95	1.241758	1.0958791
Myo1c	Q9WTI7	740	QSLATK(*)IQAAWR(*)	K6	23.99	1000	1.1914893	1.2625	0.835164	1.0963847
Lypla2	Q9WTL7	203	VQFK(*)TYPGVMHSSC(+57.02)PQEI	K4	26.27	31.43	-	-	0.755319149	
Ruvbl2	Q9WTM5	9	A(+42.01)TVAATTK(*)VPEIR(*)	K8	63.81	1000	-	1.076923	0.846153	0.9615385
Ruvbl2	Q9WTM5	197	VQAGDVITIDK(*)ATGK(*)	K11	62.83	41.04	0.7216494	0.820512	0.967032	0.8363984
Ruvbl2	Q9WTM5	279	EQINAK(*)VAEWR(*)	K6	56.85	1000	-	0.897435	0.989010	0.9432234
Ruvbl2	Q9WTM5	186	DK(*)VQAGDVITIDK(*)	K2	43.2	194.28	1.2268041	0.717948	1.010989	0.9852473
Ak3	Q9WTP7	29	ITK(*)HFELK(*)	K3	88.96	95.62	0.8085106	0.897058	0.918604	0.8747247
Ak3	Q9WTP7	34	HFELK(*)HLSSGDLLR(*)	K5	77.26	1000	1.0744680	1.014705	1.046511	1.0452285
Akap12	Q9WTQ5	475	MLPK(*)HPEGIVSEVEMLSSQER(*)	K4	41.87	1000	-	-	0.829545455	

Akap12	Q9WTQ5	1505	AEPEILELESK(*)SNK(*)	K11	27.76	29.32	1.7752808	2.111111	0.965909(	1.6174337
Skp1	Q9WTX5	66	VIQWC(+57.02)THHK(*)DDPPPPED	K9	34.25	44.94	-	-	1.22826087	
Cul1	Q9WTX6	493	LK(*)QAC(+57.02)GFEYTSK(*)	K2	75	226.64	0.9405940	-	-	
Cul1	Q9WTX6	596	GELVTNC(+57.02)FK(*)NR(*)	K9	66.51	1000	0.8118811	0.806818	0.947368	0.8553559
Cul1	Q9WTX6	726	VLK(*)HQQLLGEVLTQLSSR(*)	K3	58.29	1000	0.8712871	-	0.968421(	0.9198541
Mad1l1	Q9WTX8	61	SK(*)SYLIQVER(*)	K2	12.54	1000	1.3333333	-	1.517241	1.4252874
Pfdn5	Q9WU28	124	IQPALQEK(*)HAMK(*)	K8	79.93	48.97	0.7924528	0.864864	0.956989	0.8714356
Pfdn5	Q9WU28	55	DC(+57.02)LNVLNK(*)SNEGK(*)	K8	68.08	79.02	0.6981132	0.932432	0.860215(	0.8302536
Pfdn5	Q9WU28	112	K(*)IDFLTK(*)QMEK(*)	K7	49.92	38.03	0.8396226	0.729729	0.752688	0.7740135
Ncor2	Q9WU42	1955	TPAK(*)NLAPHHASPDPPAPTSASDLI	K4	60.68	1000	0.8	0.735632	0.931034	0.8222222
Pus1	Q9WU56	236	LLAC(+57.02)YK(*)GTHNFHNFTSQK	K6	38.91	22.7	-	-	1.06	
Pdcd6ip	Q9WU78	60	PLDK(*)HEGALETLLR(*)	K4	83.69	1000	1.0561797	0.833333	0.988764(	0.9594257
Pdcd6ip	Q9WU78	501	AVLDK(*)AVQADGQVK(*)	K5	76.69	195.94	0.9775280	0.782051	1	0.9198598
Pdcd6ip	Q9WU78	285	LQHAAELIK(*)NVASR(*)	K9	67.96	1000	1.0898876	1.025641	1	1.0385096
Pdcd6ip	Q9WU78	627	K(*)QEGLLK(*)NIQVSHQEFSK(*)	K7	65.85	34.81	1.0337078	-	-	
Pdcd6ip	Q9WU78	60	SALGR(*)PLDK(*)HEGALETLLR(*)	K9	61.99	1000	1.0449438	0.705128	1.168539	0.9728705
Pdcd6ip	Q9WU78	101	DAFDK(*)GSLFGGSVK(*)	K5	53.03	109.98	0.8651685	0.794871	0.943820	0.8679535
Pdcd6ip	Q9WU78	209	MK(*)DAIIAK(*)	K2	45.78	129.72	0.8426966	-	1.191011	1.0168539
Pdcd6ip	Q9WU78	614	IYGGLTSK(*)VQESLK(*)	K8	45.5	102.87	1.0449438	0.692307	0.853932	0.863728
Pdcd6ip	Q9WU78	690	FQNK(*)C(+57.02)SDIVFAR(*)	K4	25.91	1000	-	1.653846	1.179775	1.4168107
Pdcd6ip	Q9WU78	640	MK(*)QSNNEANLR(*)	K2	10.13	1000	-	-	1.08988764	
Farsb	Q9WUA2	208	TDNHLK(*)HYLHIIESK(*)	K6	71.49	159.26	0.9326923	0.911392	0.967032	0.9370392
Farsb	Q9WUA2	443	AVHISNPK(*)TAEFQVAR(*)	K8	57.64	1000	0.9134615	0.822784	0.868131	0.8681261
Farsb	Q9WUA2	202	EYTAC(+57.02)ELMNIYK(*)TDNHLK(	K12	53.56	41.94	-	-	0.879120879	
Farsb	Q9WUA2	492	DVGAK(*)NYR(*)	K5	37.53	1000	-	-	0.747252747	
Farsb	Q9WUA2	190	TK(*)EYTAC(+57.02)ELMNIYK(*)	K2	22.77	218.12	1.8269230	1.556962	1.208791	1.5308921
Pfkp	Q9WUA3	293	ELVVK(*)NLGFDTR(*)	K5	56.15	1000	0.9647058	-	1.011494	0.9881001
Stub1	Q9WUD1	204	AQQAC(+57.02)IEAK(*)HDK(*)	K9	75.91	49.37	0.9537037	-	0.881720	0.9177121
Rfc2	Q9WUK4	158	TMEIYSK(*)TTR(*)	K7	36.39	1000	1.4626865	0.785714	0.706521	0.9849742
Arl3	Q9WUL7	30	ILLLGLDNAGK(*)TTLLK(*)	K11	52.55	87.95	1.0707070	1.405405	0.880434	1.1188491
Arl3	Q9WUL7	54	QLASEDISHITPTQGFNIK(*)SVQSQGF	K19	5.2	32.94	-	-	1.358695652	
Coro1b	Q9WUM3	339	FYK(*)LHER(*)	K3	36.37	1000	0.9010989	1.094594	-	0.9978467
Coro1b	Q9WUM3	21	HVFGQPVK(*)NDQC(+57.02)YEDIR(	K8	21.87	1000	-	-	1.283950617	
Coro1c	Q9WUM4	19	HVFGQAVK(*)NDQC(+57.02)YDDIR	K8	80.57	1000	0.8260869	0.6375	0.864583	0.7760568

Coro1c	Q9WUM4	391	NADPILISLK(*)HGYIPGK(*)	K10	42.84	21.15	0.7608695	1.2625	0.739583	0.9209843
Coro1c	Q9WUM4	337	FFK(*)LHER(*)	K3	14.12	1000	-	1.725	1.177083	1.4510417
Suclg1	Q9WUM5	57	NTK(*)IIC(+57.02)QGFTGK(*)	K3	85.49	143.22	0.7553191	0.756410	-	0.7558647
Suclg1	Q9WUM5	94	GGQK(*)HLGLPVFNTVK(*)	K4	71.11	215.31	0.7872340	0.692307	0.931818	0.8037866
Suclg1	Q9WUM5	66	IIC(+57.02)QGFTGK(*)QGTFHSQQA	K9	12.86	25.39	-	-	1.306818182	
Uchl5	Q9WUP7	282	YK(*)IENIR(*)	K2	27.04	1000	-	-	1.054945055	
Preb	Q9WUQ2	24	IDPK(*)TGLLIAAGGGGAAK(*)	K4	18.94	32.15	-	-	0.608247423	
Eci2	Q9WUR2	90	AK(*)WDAWNALGSLPK(*)	K2	52.9	126.69	1.2758620	-	-	
Rbmx	Q9WV02	72	DMNGK(*)SLDGK(*)	K5	39.82	43.82	-	-	-	
Rbmx	Q9WV02	80	AIK(*)VEQATK(*)PSFESGR(*)	K3	28.33	151.86	-	0.541353	-	
Fam50a	Q9WV03	316	NK(*)HIFPASR(*)	K2	77.03	1000	0.2659574	-	0.25	0.2579787
Fam50a	Q9WV03	275	GK(*)SGPLFNFDVHDDVR(*)	K2	28.33	1000	1.0851063	2.212765	0.619047	1.30564
Fam50a	Q9WV03	271	EDLIIPHHHSFYDFIVTK(*)AR(*)	K18	13.69	1000	-	-	1.25	
Arpc1b	Q9WV32	326	ASSEGGAATGAGLDSLHK(*)NSVSQIS	K18	81.79	49.25	1.3043478	1.098591	1.047619	1.1501861
Arpc1b	Q9WV32	44	WNK(*)VHELK(*)	K3	78.67	127.18	1.5543478	0.661971	1.369047	1.1951224
Arpc1b	Q9WV32	82	NAYVWTLK(*)GR(*)	K8	72.8	1000	1.1521739	0.830985	1.035714	1.0062914
Asah1	Q9WV54	309	ESLDVYELDPK(*)HGR(*)	K11	69.34	1000	1.0652173	0.987179	1.022727	1.0250414
Asah1	Q9WV54	324	WK(*)NTLFIDDR(*)	K2	47.13	1000	4.3586956	-	-	
Asah1	Q9WV54	309	K(*)ESLDVYELDPK(*)HGR(*)	K12	31.02	215.31	1.0434782	1.435897	1.125	1.2014586
Vapa	Q9WV55	125	EAK(*)PDELMDSK(*)LR(*)	K11	73.75	150.63	0.6629213	0.513888	0.985074	0.7206283
Vapa	Q9WV55	10	A(+42.01)SASGAMAK(*)HEQILVLDP	K9	61.07	121.81	0.8651685	-	1.149253	1.0072111
Vapa	Q9WV55	26	FK(*)GPFTDVVTTNLK(*)	K2	47.63	183.14	0.6179775	-	1.402985	1.0104813
Snx1	Q9WV80	490	SK(*)DFK(*)NHVMK(*)	K5	38.62	68.31	-	-	1.088888889	
Snx1	Q9WV80	290	MFNK(*)ATDAVSK(*)	K4	36.59	74.72	-	0.658536	0.655555	0.6570461
Snx1	Q9WV80	490	DFK(*)NHVMK(*)	K3	19.92	119.54	0.9611650	-	-	
Epb41l3	Q9WV92	146	GQVLFDK(*)VC(+57.02)EHLNLLEK(*	K7	57.15	43.7	-	-	0.89010989	
Epb41l3	Q9WV92	386	LWK(*)VC(+57.02)VEHHTFFR(*)	K3	47.91	1000	-	-	1.164835165	
Epb41l3	Q9WV92	280	VIELHK(*)SHR(*)	K6	23.76	1000	-	1.081081	1	1.0405405
Epb41l3	Q9WV92	785	TSEGLEQK(*)SHFESSTVR(*)	K8	9.17	1000	-	-	1.274725275	
Epb41l3	Q9WV92	569	TQDELMK(*)HQTNISELK(*)	K7	7.04	27.52	-	2.337837	-	
Phlda3	Q9WV95	104	FK(*)NQQAIQTVR(*)	K2	31.98	1000	-	0.175324	0.729729	0.4525272
Tagln2	Q9WVA4	171	NFSDNQLQEGK(*)NVIGLQMGTNR(*	K11	72.09	1000	0.9494949	-	0.956989	0.9532421
Tagln2	Q9WVA4	54	ENFQK(*)WLK(*)	K5	52.81	42.6	0.7878787	0.814814	0.860215	0.8209696
Pacsin2	Q9WVE8	122	EAFHK(*)QMMGGFK(*)	K5	48.71	115.21	1.0222222	0.8	1.058139	0.9601206

Pacsin2	Q9WVE8	264	HLDLSNVASYK(*)TIYR(*)	K11	45.23	1000	0.7666666	1.125	0.860465	0.9173773
Foxo3	Q9WVH4	241	SSWWIINPDGGK(*)SGK(*)	K12	77.39	24.24	0.3742690	0.544642	0.704081	0.5409978
Psmd13	Q9WVJ2	105	VK(*)SSDEAVILC(+57.02)K(*)	K2	78.12	228.69	0.9278350	0.890243	0.927835	0.9153047
Psmd13	Q9WVJ2	306	ITVNK(*)VELLVMK(*)	K5	68.73	136.94	0.8556701	0.817073	0.907216	0.8599866
Psmd13	Q9WVJ2	115	SSDEAVILC(+57.02)K(*)TAIGALK(*)	K10	68.15	110.43	-	0.792682	0.804123	0.7984033
Psmd13	Q9WVJ2	298	QLTFEEIAK(*)SAK(*)	K9	66.85	20.75	0.7216494	0.719512	0.938144	0.793102
Ehd1	Q9WVK4	220	VVLNK(*)ADQIETQQLMR(*)	K5	90.47	1000	0.8888888	0.8375	0.977528	0.9013057
Ehd1	Q9WVK4	208	ALK(*)NHEDK(*)	K3	75.09	115.97	1.0666666	-	0.943820	1.0052434
Racgap1	Q9WVM1	249	GK(*)SGPLQPVNSDSALNSR(*)	K2	70	1000	0.7956989	0.578947	0.853658	0.7427683
Fxr2	Q9WVR4	217	NEEATK(*)HLETSK(*)	K6	55.85	90.32	-	-	0.930693069	
Mapk7	Q9WVS8	35	AEPVHTAASVVAK(*)NLALLK(*)	K13	66.59	102.87	0.1636615	-	1.136363	0.6500126
Rad9a	Q9Z0F6	2	MK(*)C(+57.02)LITGGNVK(*)	K2	60.85	123.34	-	-	-	
Clip2	Q9Z0H8	232	VLVGGTK(*)TGVVR(*)	K7	19.56	1000	0.6603773	1.054945	1.021052	0.912125
Clip2	Q9Z0H8	365	EK(*)QQHIEQLLAER(*)	K2	18.66	1000	-	1.131868	0.526315	0.829092
Npc2	Q9Z0J0	116	LPVK(*)NEYPSIK(*)	K4	84.48	98	1.0238095	0.916666	1.0125	0.9843254
Npc2	Q9Z0J0	105	DK(*)VYSYLNK(*)	K2	65.95	136.94	-	-	0.85	
Eif2s3x	Q9Z0N1	183	LK(*)HILILQNK(*)	K2	92.26	242.89	1.0329670	0.8125	0.967391	0.9376194
Eif2s3x	Q9Z0N1	70	FK(*)NELER(*)	K2	87.9	1000	1.0549450	0.825	0.934782	0.9382426
Eif2s3x	Q9Z0N1	421	VSAVK(*)ADLGK(*)	K5	82.23	87.95	1.2967032	0.9125	1.097826	1.1023431
Eif2s3x	Q9Z0N1	449	R(*)VEK(*)HWR(*)	K4	67.02	1000	1.2087912	1.1	1.195652	1.1681478
Eif2s3x	Q9Z0N1	191	HILILQNK(*)IDLVK(*)	K8	62.81	107.29	0.8571428	0.7625	0.934782	0.8514752
Eif2s3x	Q9Z0N1	90	IYK(*)LDDPSC(+57.02)PR(*)PEC(+5	K3	8.79	1000	-	1.2125	-	
Twf2	Q9Z0P5	136	DDLSLAGYQK(*)HLSSC(+57.02)AAP	K10	48.26	1000	-	-	1	
Fads2	Q9Z0R9	409	SLC(+57.02)AK(*)HGIEYQEK(*)PLLR	K5	83.8	170.86	0.7872340	0.675324	0.879120	0.7805599
Fads2	Q9Z0R9	105	GK(*)SSQITEDFR(*)	K2	59.66	1000	-	0.610389	0.934065	0.7722278
Fads2	Q9Z0R9	404	IAPLVK(*)SLC(+57.02)AK(*)	K6	53.82	38.19	1.5319148	0.558441	1.142857	1.0777379
Fads2	Q9Z0R9	28	WEEIQK(*)HNLR(*)	K6	45.92	1000	0.7978723	-	0.857142	0.8275076
Fads2	Q9Z0R9	409	SLC(+57.02)AK(*)HGIEYQEK(*)	K5	24.15	66.1	-	-	1.142857143	
Fads2	Q9Z0R9	206	LK(*)GASANWWNHR(*)	K2	23.21	1000	0.5106382	1.025974	-	0.7683062
Bpnt1	Q9Z0S1	49	TSATDLQTK(*)ADR(*)	K9	64.96	1000	0.6597938	-	0.714285	0.6870398
Bpnt1	Q9Z0S1	277	EVK(*)HMNSAGVLAALR(*)	K3	49.17	1000	1.0206185	-	1.020408	1.0205134
Tjp2	Q9Z0U1	1090	IEIAQK(*)HPDIYAVPIK(*)	K6	39.42	47.27	1.2164948	-	0.567010	0.8917526
Tjp2	Q9Z0U1	769	QIIEQDK(*)HALLDVTPK(*)	K7	29.29	43	-	-	0.649484536	
Aifm1	Q9Z0X1	517	ATAQDNPK(*)SATEQSGTGIR(*)	K8	19.26	1000	1.5555555	1.453488	1.103448	1.3708307

Dctn3	Q9Z0Y1	156	ALLEGYNK(*)TTMLLSK(*)	K8	22.15	83.89	1.7444444	-	-	
Mettl1	Q9Z120	137	SNAMK(*)HLPNFFR(*)	K5	37.59	1000	1.2045454	-	-	
Hnrnpdl	Q9Z130	61	FGEVVDC(+57.02)TIK(*)TDPVTGR(*	K10	42.42	1000	0.6767676	-	0.4777777	0.5772727
Sumo3	Q9Z172	11	EGVK(*)TENDHINLK(*)	K4	60.3	143.22	1.2888888	1.051282	1.0222222	1.1207977
Mad2l1	Q9Z1B5	108	WQFDIEC(+57.02)DK(*)TAK(*)	K9	55.62	29.32	0.9072164	0.876404	1.0215053	0.9350421
Mad2l1	Q9Z1B5	192	SFTTTIHK(*)VNSMVAYK(*)	K8	28.77	53.45	-	-	1.247311828	
Uba2	Q9Z1F9	245	EWAK(*)STGYDPVK(*)	K4	58.9	107.73	0.8571428	-	0.6373626	0.7472527
Uba2	Q9Z1F9	164	GVTEC(+57.02)YEC(+57.02)HPK(*)F	K11	34.99	1000	1.5408163	1.316455	0.7252747	1.1941822
Uba2	Q9Z1F9	257	LFTK(*)LFK(*)	K4	30.02	68.47	1.0816326	0.734177	0.9560439	0.9239513
Uba2	Q9Z1F9	271	YLLTMDK(*)LWR(*)	K7	22.14	1000	-	-	1.142857143	
Atp6v1c1	Q9Z1G3	119	QSLK(*)NISEIIAK(*)	K4	26.2	124.34	-	1.6625	0.7865168	1.2245084
Arih1	Q9Z1K5	255	LK(*)YQHLITNSFVEC(+57.02)NR(*)	K2	18.33	1000	2.6506024	-	1.8255813	2.2380919
Rps6kb2	Q9Z1M4	80	GGYGK(*)VFQVR(*)	K5	15.03	1000	-	-	0.546296296	
Ddx39b	Q9Z1N5	188	SLNLK(*)HIK(*)	K5	96.62	68.47	0.9887640	0.842105	0.9230769	0.9179821
Ddx39b	Q9Z1N5	53	DFLLK(*)PELLR(*)	K5	84.32	1000	0.6966292	0.828947	0.9340659	0.8198808
Ddx39b	Q9Z1N5	191	HIK(*)HFILDEC(+57.02)DK(*)	K3	83.67	184.54	1.0561797	0.868421	0.9450549	0.9565519
Ddx39b	Q9Z1N5	36	K(*)DVK(*)GSYVSIHSSGFR(*)	K4	79.82	44.44	1.1797752	0.723684	0.9450549	0.9495048
Ddx39b	Q9Z1N5	36	DVK(*)GSYVSIHSSGFR(*)	K3	78.19	1000	1.1685393	1.078947	0.9670329	1.0715066
Ddx39b	Q9Z1N5	183	NK(*)SLNLK(*)	K2	70.11	100.21	0.8426966	1.078947	-	0.960822
Clic1	Q9Z1Q5	119	IK(*)NSNPALNDNLEK(*)	K2	81.78	264.82	0.9042553	0.833333	0.8522727	0.8632871
Vars	Q9Z1Q9	951	HFC(+57.02)NK(*)LWNATK(*)	K5	90.75	113.99	0.8681318	0.815789	0.9677419	0.8838878
Vars	Q9Z1Q9	551	IETMLGDVAVAVHPK(*)DPR(*)	K15	23.98	1000	-	1.118421	-	
Ap3b1	Q9Z1T1	338	SEAGVISK(*)SLVR(*)	K8	69.94	1000	0.7368421	1.302631	0.7708333	0.936769
Ap3b1	Q9Z1T1	90	NVASK(*)NIEIK(*)	K5	60.98	77.53	0.8421052	0.697368	0.8333333	0.7909357
Ap3b1	Q9Z1T1	524	MAK(*)SFTSEDDLVK(*)	K3	36.1	137.5	1.0947368	1.657894	0.9583333	1.2369883
Ap3b1	Q9Z1T1	85	NASELFPAVVK(*)NVASK(*)	K11	29.11	29.96	1	0.618421	-	0.8092105
Ilf3	Q9Z1X4	224	ANGLK(*)SC(+57.02)VIVIR(*)	K5	44.3	1000	1.0537634	1.408450	1.3295454	1.2639199
Ilf3	Q9Z1X4	17	HVMAK(*)HSSVYPTQEELEAVQNMVS	K5	32.03	1000	1.7741935	-	1.2840909	1.5291422
Uso1	Q9Z1Z0	586	LGFISK(*)HELYSR(*)	K6	46.1	1000	-	-	1.086956522	
Uso1	Q9Z1Z0	842	SVPVEGESEHVSAAK(*)TTDVEGR(*)	K15	41.06	1000	-	-	1.02173913	
Strap	Q9Z1Z2	104	HIVK(*)TVDFTQDSNYLLTGGQDK(*)	K4	60.26	257.92	-	-	0.381443299	
Strap	Q9Z1Z2	262	YDYNSGEELESYK(*)GHFGPIHC(+57.(	K13	53.34	1000	-	-	1.06185567	
Strap	Q9Z1Z2	298	LWQTVVGK(*)TYGLWK(*)	K8	48.61	104.48	1.0824742	-	1.2577319	1.1701031
Strap	Q9Z1Z2	164	QILSADDK(*)TVR(*)	K8	26.75	1000	-	-	0.896907216	

Strap	Q9Z1Z2	249	LYK(*)YDYNSGEELESYK(*)	K3	11.86	61.26	-	-	1.453608247	
Hnrnpc	Q9Z204	170	GK(*)SGFNSK(*)	K2	84.56	110.2	-	0.948717	0.9222222	0.9354701
Hnrnpc	Q9Z204	8	A(+42.01)SNVTNK(*)TDPR(*)	K7	79.12	1000	1.2061855	0.794871	-	1.0005287
Hnrnpc	Q9Z204	176	SGFNSK(*)SGQR(*)	K6	77.36	1000	1.0309278	-	1	1.0154639
Hnrnpc	Q9Z204	42	YGK(*)IVGC(+57.02)SVHK(*)	K3	75.04	183.32	1.2783505	1.371794	1.1777777	1.2759744
Hnrnpc	Q9Z204	207	QK(*)VDSLLESLEK(*)	K2	65.9	126.68	0.8350515	0.717948	0.9777777	0.8435927
Hnrnpc	Q9Z204	50	IVGC(+57.02)SVHK(*)GFAFVQYVNE	K8	65.76	1000	0.8969072	1	1.0111111	0.9693394
Hnrnpc	Q9Z204	39	K(*)SDVEAIFSK(*)YGK(*)	K10	50.98	24.24	1.3402061	1.730769	1.1777777	1.4162511
Hnrnpc	Q9Z204	39	SDVEAIFSK(*)YGK(*)	K9	46.04	37.54	-	-	1.022222222	
Baz1b	Q9Z277	502	SALSC(+57.02)VISK(*)TAR(*)	K9	83.69	1000	0.7272727	-	0.7978723	0.7625725
Baz1b	Q9Z277	964	ANLGK(*)NASVNAHHGPALEAVETTV	K5	48.43	69.52	0.7474747	-	0.8404255	0.7939501
Baz1b	Q9Z277	818	EENVLGK(*)VDR(*)	K7	47.03	1000	-	0.586206	-	
Baz1b	Q9Z277	1438	TEQC(+57.02)LLALLQK(*)HLPGHPY	K11	33.91	1000	-	-	0.79787234	
Baz1b	Q9Z277	410	GK(*)GILNGQK(*)	K2	10.81	85.08	-	0.091954	-	
Mbd3	Q9Z2D8	92	GK(*)PDLNTALPVR(*)	K2	16.98	1000	1.0747663	-	-	
Epb41l1	Q9Z2H5	115	VTLLDASEYEC(+57.02)EVEK(*)HGR(	K15	75.44	1000	1.1547619	0.784810	0.9450549	0.9615423
Epb41l1	Q9Z2H5	259	IMELHK(*)TYR(*)	K6	63.32	1000	1.7738095	1.101265	1.2417582	1.3722779
Epb41l1	Q9Z2H5	95	FK(*)SAIC(+57.02)R(*)	K2	42.77	1000	1.2857142	-	1.0879120	1.1868132
Suclg2	Q9Z2I8	94	GK(*)GVFNSGLK(*)	K2	34.62	75.74	-	-	1.021978022	
Suclg2	Q9Z2I8	219	MAENLGFLGSLK(*)NQAADQITK(*)	K12	20.01	100.79	-	-	1.637362637	
Suclg2	Q9Z2I8	387	ELELK(*)VPLVVR(*)	K5	16.78	1000	1.3009708	-	-	
Sucla2	Q9Z2I9	78	GFVAK(*)SSDEAYAIAK(*)	K5	77.16	168.62	0.8936170	0.722891	0.9120879	0.8428655
Septin5	Q9Z2Q6	90	INQTVEILK(*)HTVDIEEK(*)	K9	35.96	38.35	-	-	1.041666667	
Psma7	Q9Z2U0	38	GK(*)DIVVLGVEK(*)	K2	81.98	225.26	0.9368421	0.986486	0.9438202	0.9557163
Psma5	Q9Z2U1	196	EAIK(*)SSLIILK(*)	K4	86.47	128.65	0.9787234	0.790123	0.9888888	0.9192453
Zfp292	Q9Z2U2	1104	TYNSSQSIGK(*)HMK(*)	K10	61.88	62.26	-	57	-	
Hnrnpf	Q9Z2X1	185	YIEVFK(*)SSQEEVR(*)	K6	95.72	1000	0.8989898	0.846153	0.9042553	0.883133
Hnrnpf	Q9Z2X1	98	TEMDWVLK(*)HSGPNSADSANDGFV	K8	94.99	1000	1.1010101	0.807692	0.9148936	0.9411987
Hnrnpf	Q9Z2X1	87	YIEVFK(*)SHR(*)	K6	93.04	1000	1.0101010	0.871794	0.9680851	0.9499937
Hnrnpf	Q9Z2X1	224	YIGIVK(*)QAGLDR(*)	K6	81.26	1000	1	0.756410	0.8723404	0.8762502
Psmd10	Q9Z2X2	30	ILADK(*)SLATR(*)	K5	19.23	1000	-	0.736842	0.8433734	0.7901078
Plpbp	Q9Z2Y8	125	LADK(*)VNSSWQK(*)	K4	79.87	144.88	1.0638297	0.730337	0.8965517	0.8969062
Plpbp	Q9Z2Y8	99	WHFIGHLQK(*)QNVNK(*)	K9	12.42	39.44	-	1.022471	-	
Sart1	Q9Z315	672	APNK(*)SLPSAVYC(+57.02)IEDK(*)	K4	83.56	138.85	1.1860465	0.592105	0.9113924	0.8965147

Sart1	Q9Z315	797	IVLSGSGK(*)SMNANTITK(*)	K8	60.77	112.47	1.3488372	-	-

e
