# Supplementary material for: Quantitative Proteomics Analysis Expands the Roles of Lysine β-Hydroxybutyrylation Pathway in Response to Environmental β-Hydroxybutyrate
Source: Oxid Med Cell Longev. 2022 Feb 24;2022:4592170. doi: 10.1155/2022/4592170 (PMC8894020; doi:10.1155/2022/4592170)
Supplement: Supplementary Materials — Table S1/S2/S3 are the complete lists of identified proteins/Kac sites/Kbhb sites in MEF cells with or without BHB treated. [file 4592170.f1.zip › Table S3. The identification of Kbhb sites and substrates.pdf]

Table S3:The identification of Kbhb sites and substrates.

Gene name	UniprotID	Site in pro	Peptide	Positic	Quality Sc	Ascore	Ratio:Rep1	Ratio:Rep2	Ratio:Rep3	Ratio_average
Gls	D3Z7P3	163	IPVHK(*)FITALK(*)	K5	51.08	90.32	1.49462365	1.01265822	1.12765957	1.211647153
Prdx6	O08709	144	LK(*)LSILYPATTGR(*)	K2	72.66	1000	-	1.1617647	-	
Prdx6	O08709	56	AAK(*)LAPEFAK(*)	K3	55.48	81.81	-	0.92647058	0.80219780	0.864334195
Prkcsh	O08795	369	SK(*)FEEVER(*)	K2	65.35	1000	1.17708333	1.36363636	1.10752688	1.216082193
Prkcsh	O08795	158	QSK(*)LLELQAGK(*)	K3	65.1	101.72	0.97916666	-	-	
Atox1	O08997	60	TGK(*)AVSYLGPK(*)	K3	96.52	150.89	-	38.6363636	45.0112359	41.8237998
Phb2	O35129	250	NPGYIK(*)LR(*)	K6	54.8	1000	1.28865979	-	1.76595744	1.527308621
Phb2	O35129	147	SVVAK(*)FNASQLITQR(*)	K5	44.67	1000	1.03092783	-	-	
Psmd4	O35226	74	ILSK(*)LHTVQPK(*)	K4	14.51	104.64	-	-	0.913043478	
Anxa3	O35639	169	R(*)DESLK(*)VDEHLAK(*)	K6	23.99	83.89	-	1.37349397	-	
Nudc	O35685	276	INPENSK(*)LSDLDSETR(*)	K7	14.89	1000	1.65555555	-	-	
Api5	O35841	202	ILSGLK(*)SLQTVSGR(*)	K6	47.72	1000	1.28723404	0.8	0.90217391	0.996469319
Cops5	O35864	56	YC(+57.02)K(*)ISALALLK(*)	K3	27.63	150.63	-	1.36	-	
Cavin1	O54724	163	VMIYQDEVK(*)LPAK(*)	K9	39.03	58.99	0.55681818	1.33766233	0.94444444	0.946308321
Cavin1	O54724	167	LPAK(*)LSVSK(*)	K4	37.28	73.54	0.75	1	-	0.875
Sap18	O55128	24	EK(*)TC(+57.02)PLLLR(*)	K2	45.71	1000	-	1.6081081	-	
Rpl35a	O55142	8	LWC(+57.02)K(*)AIFAGYK(*)	K4	35	122.76	1.38888888	-	1.28318584	1.336037365
Pdlim1	O70400	254	VAASVGNAQK(*)LPIC(+57.02)	K10	34.7	95.35	1.88043478	1.39473684	-	1.637585813
Psma3	O70435	57	LVLSK(*)LYEEGSNK(*)	K5	33.19	150.77	-	1.1728395	1.5	1.336419753
Ugdh	O70475	107	AADLK(*)YIEAC(+57.02)AR(*)	K5	46.61	1000	-	1.05194805	-	
Hsd17b12	O70503	97	EK(*)FNVETR(*)	K2	66.61	1000	-	1.38571428	-	
Hsd17b12	O70503	86	SQDK(*)LNQVSNNIK(*)	K4	30.03	198.17	-	2.01428571	-	
Wdr1	O88342	182	FK(*)FTIGDHSR(*)	K2	56.5	1000	1.67346938	-	-	
Wdr1	O88342	405	DYSGQGVVK(*)LDVQPK(*)	K9	33.09	112.31	-	1.83544303	1.81914893	1.827295987
Wdr1	O88342	81	YIASGDISGK(*)LR(*)	K10	15.12	1000	-	1.73417721	-	
Cops6	O88545	233	VK(*)LILEYVK(*)	K2	17.05	142.27	-	0.81578947	-	
Hnrnpa2b1	O88569	173	IVLQK(*)YHTINGHNAEVR(*)	K5	74	1000	-	1.03947368	1.02298850	1.031231095
C4b	P01029	1497	ADLEK(*)LTSLSDR(*)	K5	23.42	1000	0.07692307	-	0.08333333	0.080128205
Lamc1	P02468	1301	LIDQK(*)LK(*)	K5	16.7	1000	-	0.7790697	-	
Lamb1	P02469	1736	SK(*)LQLLEDLER(*)	K2	57.81	1000	0.90526315	0.7375	-	0.821381579
Lamb1	P02469	1589	SATDVK(*)VTADMVK(*)	K6	46.75	107.64	-	0.825	-	

Lamb1	P02469	1208	ALK(*)ISGVIGPYR(*)	K3	23.98	1000	-	-	1.673913043	
Aldoa	P05064	312	ALQASALK(*)AWGGK(*)	K8	57.02	86.16	-	-	0.954545455	
Aldoa	P05064	28	IVAPGK(*)GILAADESTGSIAK(*)	K6	47.28	135.22	-	0.74666666	1.52272727	1.13469697
Aldoa	P05064	243	MVTPGHAC(+57.02)TQK(*)FSI	K11	29.13	1000	1.59574468	1.37333333	-	1.484539007
Tuba1b	P05213	394	LDHK(*)FDLMYAK(*)	K4	79.4	170.44	-	-	-	
Tuba1b	P05213	311	HGK(*)YMAC(+57.02)C(+57.0	K3	65.74	1000	-	-	-	
Tuba1b	P05213	60	TIGGGDDSFNTFFSETGAGK(*)H	K20	11.54	1000	-	-	-	
Ldha	P06151	90	SK(*)LVIITAGAR(*)	K2	89.21	1000	1.11702127	1.06329113	0.95555555	1.045289324
Ldha	P06151	126	IVK(*)YSPHC(+57.02)K(*)	K3	22.06	108.85	-	1.31645569	-	
Gpi	P06745	25	ANSANLK(*)LR(*)	K7	80.9	1000	-	1.06172839	1.05494505	1.058336725
Gpi	P06745	57	ILVDYSK(*)NLVNK(*)	K7	30.38	77.53	-	1.61728395	-	
S100a4	P07091	57	TDEAAFQK(*)VMSNLDSNR(*)	K8	74.06	1000	1.03260869	0.79746835	1.04597701	0.958684687
Anxa2	P07356	227	SVC(+57.02)HLQK(*)VFER(*)	K7	97.31	1000	-	-	36.69662921	
Anxa2	P07356	302	SEVDMLK(*)IR(*)	K7	43.44	1000	-	-	1.04494382	
Hsp90aa1	P07901	568	TK(*)FENLC(+57.02)K(*)	K2	94.79	151.86	1.08421052	0.93589743	1.01123595	1.010447972
Hsp90aa1	P07901	574	FENLC(+57.02)K(*)IMK(*)	K6	91.78	1000	1.01052631	0.87179487	1.15730337	1.013208186
Hsp90aa1	P07901	568	K(*)TK(*)FENLC(+57.02)K(*)	K3	86.25	36.05	-	0.65384615	1.17977528	0.916810718
Hsp90aa1	P07901	447	NIK(*)LGIHEDSQNR(*)	K3	65.39	1000	1.01052631	0.93589743	1.01123595	0.985886569
Hsp90aa1	P07901	540	EFEGK(*)TLVSVTK(*)	K5	21.58	78.16	-	1.82051282	-	
Hsp90aa1	P07901	586	K(*)VEK(*)VVVSNR(*)	K4	16.1	44.44	-	-	1.213483146	
Pdia4	P08003	347	VSLGK(*)LVLTHPEK(*)	K5	83.62	171.6	0.94845360	0.92592592	1	0.958126511
Pdia4	P08003	622	DLEHLSK(*)FIDEHATK(*)	K7	53.78	89.48	-	-	0.935483871	
Aprt	P08030	7	S(+42.01)EPELK(*)LVAR(*)	K6	14.85	1000	-	1.55434782	-	
Hsp90b1	P08113	75	SEK(*)FAFQAEVNR(*)	K3	90.4	1000	1	0.975	0.94680851	0.97393617
Hsp90b1	P08113	479	IADEK(*)YNDTFWK(*)	K5	78.39	170.44	1	1	0.98936170	0.996453901
Hsp90b1	P08113	455	ETLQQHK(*)LLK(*)	K7	67.77	62.26	-	0.8	1.25531914	1.027659575
Hsp90b1	P08113	493	IK(*)LGVIEDHSNR(*)	K2	66.7	1000	1.22448979	0.8875	-	1.055994898
Hsp90b1	P08113	87	MMK(*)LIINSLYK(*)	K3	63.8	150.63	1.16326530	0.8875	0.77659574	0.942453684
Hsp90b1	P08113	493	EFGTNIK(*)LGVIEDHSNR(*)	K7	63.28	1000	1.02040816	0.9625	0.95744680	0.980118324
Hsp90b1	P08113	597	EGVK(*)FDESEK(*)	K4	27.27	95.35	-	1.375	0.78723404	1.081117022
S100a10	P08207	57	IMK(*)DLDQC(+57.02)R(*)	K3	22.14	1000	-	1.22826087	1.09890109	1.163580985
Mdh2	P08249	185	ANTFVAELK(*)GLDPAR(*)	K9	51.62	1000	0.98913043	0.60493827	-	0.797034354
Mdh2	P08249	307	NLGIGK(*)ITPFEEK(*)	K6	28.93	81	-	1.22222222	-	
P4hb	P09103	273	SVSDYDGK(*)LSSFK(*)	K8	84.76	87.95	1.05050505	0.98734177	0.97826087	1.005369231

P4hb	P09103	226	EK(*)LLDFIK(*)	K2	79.02	101.67	0.97979798	1.05063291	0.989130435	1.006520442
P4hb	P09103	446	VHSFPTLK(*)FFPASADR(*)	K8	72.25	1000	0.949494949	0.78481012	1	0.911435025
P4hb	P09103	197	SGVFSK(*)YQLDK(*)	K6	27.46	127.18	1.353535354	1.49367088	-	1.42360312
Ncl	P09405	431	SK(*)GIAYIEFK(*)	K2	73.19	170.86	1.130434783	0.91358024	1.120879121	1.054964717
Ncl	P09405	379	VFGNEIK(*)LEK(*)PK(*)	K7	49.61	78.06	0.532608696	1.03703703	-	0.784822867
Ncl	P09405	372	ALELTGLK(*)VFGNEIK(*)	K8	37.87	137.02	1.77173913	1.41975308	1.373626374	1.521706197
Ncl	P09405	546	EALNSC(+57.02)NK(*)MEIEGR	K8	37.41	1000	2.663043478	-	1.010989011	1.837016245
Pgk1	P09411	48	AAVPSIK(*)FC(+57.02)LDNGA	K7	82.31	118.18	1.097826087	0.975	1	1.024275362
Pgk1	P09411	11	LTLDK(*)LDVK(*)	K5	78.09	72.65	1.010869565	1.025	1.058139535	1.031336367
Pgk1	P09411	146	AEPAK(*)IDAFR(*)	K5	76.68	1000	1.097826087	0.8375	0.941860465	0.959062184
Pgk1	P09411	156	ASLSK(*)LGDVYVNDAFGTAHR	K5	75.92	1000	1.076086957	0.9625	1.104651163	1.04774604
Pgk1	P09411	6	S(+42.01)LSNK(*)LTLDK(*)	K5	68.77	119.54	1.108695652	0.8875	-	0.998097826
Pgk1	P09411	91	SLLGK(*)DVLFLK(*)	K5	55.65	134.33	1.076086957	-	1.093023256	1.084555107
Pgk1	P09411	353	GTK(*)SLMDEVVK(*)	K3	54.37	121.9	0.923913043	1.0375	1.755813953	1.239075665
Pgk1	P09411	220	VADK(*)IQLINNMLDK(*)	K4	48.27	192.46	1.619565217	-	-	
Anxa1	P10107	242	VFQNYGK(*)YSQHDMNK(*)	K7	60.62	49.35	-	0.92	1	0.96
Anxa1	P10107	262	GDIEK(*)C(+57.02)LTTIVK(*)	K5	54.94	140.21	0.989473684	0.98666666	1.172413793	1.049518048
Anxa1	P10107	250	YSQHDMNK(*)ALDLELK(*)	K8	47.82	104.8	1.378947368	0.98666666	1.597701149	1.321105061
Eef1a1	P10126	146	TLGVK(*)QLIVGVNK(*)	K5	98.26	216.51	0.946808511	1.06578947	1.011627907	1.008075297
Eef1a1	P10126	392	K(*)LEDGPK(*)FLK(*)	K7	92.02	56.34	0.904255319	0.90789473	0.953488372	0.921879476
Eef1a1	P10126	392	LEDGPK(*)FLK(*)	K6	90.78	57.45	0.957446809	1	1.011627907	0.989691572
Eef1a1	P10126	41	TIEK(*)FEK(*)	K4	89.61	46.91	-	1.07894736	1.069767442	1.074357405
Txn	P10639	85	GQK(*)VGEFSGANK(*)	K3	28.68	135.76	-	-	1.228915663	
Gstm1	P10649	50	SQWLNEK(*)FK(*)	K7	28.71	22.85	1.146067416	0.77215189	1.317647059	1.078622125
Gstm1	P10649	136	MK(*)LYSEFLGK(*)	K2	20.72	113.97	1.426966292	1.79746835	-	1.612217323
Slc3a2	P10852	53	VAEDETEAGVK(*)FTGLSK(*)	K11	47.85	34.81	-	0.85897435	-	
H1-0	P10922	69	VGENADSQIK(*)LSIK(*)	K10	35.28	41.53	0.950980392	1.09638554	1.831168831	1.292844922
H1-0	P10922	85	QTK(*)GVGASGSFR(*)	K3	26.42	1000	-	1.38554216	1.779220779	1.582381474
Fn1	P11276	1115	IGFK(*)LGVR(*)	K4	50.53	1000	-	0.93243243	-	
Fn1	P11276	1049	QYNVGPLASK(*)YPLR(*)	K10	34.57	1000	1.166666667	-	0.976744186	1.071705427
Cdk1	P11440	143	GTIK(*)LADFGLAR(*)	K4	84.25	1000	1.011111111	0.9375	0.956043956	0.968218356
Hsp90ab1	P11499	559	AK(*)FENLC(+57.02)K(*)	K2	98.93	151.86	1.073684211	0.93827160	1.202247191	1.071401002
Hsp90ab1	P11499	565	FENLC(+57.02)K(*)LMK(*)	K6	91.86	34.94	1.010526316	0.83950617	1.157303371	1.002445287
Hsp90ab1	P11499	64	YESLTDPSK(*)LDSGK(*)	K9	91.8	52.68	1.042105263	0.90123456	0.887640449	0.943660093

Hsp90ab1	P11499	275	EK(*)YIDQEELNK(*)	K2	87.39	206.63	1.03157894	0.9382716	0.94382022	0.971223592
Hsp90ab1	P11499	72	ELK(*)IDIIPNPQER(*)	K3	87.08	1000	1.05263157	0.86419753	1.01123595	0.976021688
Hsp90ab1	P11499	354	NNIK(*)LYVR(*)	K4	86.71	1000	1.01052631	1.04938271	0.98876404	1.016224359
Hsp90ab1	P11499	438	NLK(*)LGIHEDSTNR(*)	K3	85.97	1000	0.98947368	0.88888888	1.04494382	0.974435464
Hsp90ab1	P11499	531	EFDGK(*)SLVSVTK(*)	K5	74.93	112.7	0.85263157	0.92592592	0.97752809	0.918695198
Hsp90ab1	P11499	180	GTK(*)VILHLK(*)	K3	73.6	143.14	1.01052631	0.91358024	1.01123595	0.978447506
Hsp90ab1	P11499	435	FYEAFSK(*)NLK(*)	K7	59.14	47.36	-	0.98765432	1.37078651	1.179220419
Hsp90ab1	P11499	354	K(*)NNIK(*)LYVR(*)	K5	56.51	61.69	1	0.72839506	1	0.909465021
Hsp90ab1	P11499	399	EMLQQSK(*)ILK(*)	K7	22.67	44.84	1.29473684	-	1.44943820	1.372087522
Hsp90ab1	P11499	577	K(*)VEK(*)VTISNR(*)	K4	22.27	47.09	-	0.71604938	-	
Hsp90ab1	P11499	69	LDSGK(*)ELK(*)	K5	18.88	37.81	-	1	1.03370786	1.016853933
Hsp90ab1	P11499	438	NLK(*)LGIHEDSTNR(*)R(*)	K3	13.75	1000	-	1.41975308	-	
Hsp90ab1	P11499	607	IMK(*)AQALR(*)	K3	12.09	1000	-	1.04938271	-	
Tcp1	P11983	484	NLK(*)WIGLDLVHGK(*)	K3	48.23	168.06	0.74736842	0.60759493	1.03225806	0.795740474
Tcp1	P11983	532	IDDLIK(*)LHPESK(*)	K6	35.42	68.66	1.45263157	1.30379746	-	1.378214524
Tcp1	P11983	233	IVNAK(*)IAC(+57.02)LDFSLQK	K5	32.46	183.74	-	-	1.989247312	
Pfkl	P12382	689	AMLWVSEK(*)LR(*)	K8	45.02	1000	-	1.14814814	-	
Rpl7a	P12970	217	GALAK(*)LVEAIR(*)	K5	75.88	1000	1.06	1.1038961	1.18681318	1.116903097
Rpl7a	P12970	97	QTATQLLK(*)LAHK(*)	K8	44.63	52.86	-	-	0.989010989	
Rpl7a	P12970	101	LAHK(*)YR(*)PETK(*)	K4	24.26	113.99	1.31	1.09090909	1.12087912	1.173929404
Scd2	P13011	208	AEK(*)LVMFQR(*)	K3	74.49	1000	1.10389610	1.51666666	1.51470588	1.378422884
Scd2	P13011	198	GGK(*)LDMSDLK(*)	K3	63.93	122.76	-	1.06666666	-	
Gsn	P13020	366	TASDFISK(*)MQYPR(*)	K8	57.37	1000	1.03260869	0.9868421	1.26136363	1.093604812
Gsn	P13020	243	ATQVSK(*)GIR(*)	K6	27.15	1000	-	0.96052631	-	
S100a6	P14069	40	ELIQK(*)ELTIGSK(*)	K5	86.45	110.43	0.80188679	0.8625	0.86868686	0.844357887
S100a6	P14069	47	ELTIGSK(*)LQDAEIAR(*)	K7	85.19	1000	0.83962264	0.9625	0.87878787	0.89363684
S100a6	P14069	35	ELK(*)ELIQK(*)	K3	73.7	115.97	0.94339622	0.9625	1.01010101	0.971999079
S100a6	P14069	26	EGDK(*)HTLSK(*)	K4	68.32	127.18	0.90566037	0.9875	0.88888888	0.927349755
Rpl27a	P14115	47	INFDK(*)YHPGYFGK(*)	K5	51.91	24.34	-	0.97468354	-	
Rps16	P14131	109	EIK(*)DILIQYDR(*)	K3	66.88	1000	1.13265306	-	1.01063829	1.07164568
Rps16	P14131	98	ALVAYYQK(*)YVDEASK(*)	K8	57.52	128.65	0.81632653	-	-	
Rps16	P14131	90	QSISK(*)ALVAYYQK(*)	K5	20.81	92.08	1.63265306	-	-	
Mdh1	P14152	170	SQIALK(*)LGVTADDVK(*)	K6	37.89	161.52	0.82828282	-	1.57303370	1.200658268
Rpsa	P14206	57	TWEK(*)LLLAAR(*)	K4	90.65	1000	0.88297872	0.88607594	1.01111111	0.926721928

Rpsa	P14206	89	AVLK(*)FAAATGATPIAGR(*)	K4	81.93	1000	0.95744680	0.96202531	0.98888888	0.969453671
Calr	P14211	64	DK(*)GLQTSQDAR(*)	K2	94.89	1000	1.04255319	1.0875	0.95555555	1.028536249
Calr	P14211	48	SDFGK(*)FVLSSGK(*)	K5	89.81	128.65	1.02127659	1.0125	1.05555555	1.029777384
Calr	P14211	55	FVLSSGK(*)FYGDLEK(*)	K7	82	98	1.02127659	0.9875	0.97777777	0.995518125
Calr	P14211	80	FYALSAK(*)FEPFSNK(*)	K7	72.4	100.04	1.51063829	0.7	0.92222222	1.04428684
Calr	P14211	111	HEQNIDC(+57.02)GGGYVK(*)I	K13	39.58	127.49	1.96808510	-	0.82222222	1.395153664
Lmnb1	P14733	135	ESDLSGAQIK(*)LR(*)	K10	84.93	1000	0.93684210	0.96202531	1	0.96628914
Lmnb1	P14733	103	AK(*)LQIELGK(*)	K2	76.73	138.97	1.12631578	1.01265822	0.93406593	1.02434665
Lmnb1	P14733	135	K(*)ESDLSGAQIK(*)LR(*)	K11	39.07	102.12	-	0.74683544	0.96703296	0.856934205
Lmnb1	P14733	110	LQIELGK(*)FK(*)	K7	38.64	33.18	2	0.65822784	-	1.329113924
Anxa6	P14824	81	YELTGK(*)FER(*)	K6	68.92	1000	1.25773195	1.07792207	-	1.167827019
Rplp0	P14869	77	GHLENNPALEK(*)LLPHIR(*)	K11	86.6	1000	0.97849462	0.86075949	0.93333333	0.924195817
H1-2	P15864	85	LGLK(*)SLVSK(*)	K4	98.48	127.18	1.15116279	0.89156626	1.13698630	1.059905119
H1-2	P15864	106	GTGASGSFK(*)LNK(*)	K9	97.81	37.54	-	0.86746988	1.17808219	1.022776036
H1-2	P15864	90	SLVSK(*)GILVQTK(*)	K5	54.84	170.44	1.20930232	-	1.13698630	1.173144314
Lgals1	P16045	108	LPDGHEFK(*)FPNR(*)	K8	92.68	1000	0.86597938	0.79220779	0.97701149	0.878399556
Lgals1	P16045	19	A(+42.01)C(+57.02)GLVASNLI	K18	74.72	23.64	0.86597938	1.63636363	0.81609195	1.10614499
Sptan1	P16546	144	GIK(*)LLQAQK(*)	K3	58.64	132.53	-	1.01315789	-	
Sptan1	P16546	441	EK(*)LSILSEER(*)	K2	51.85	1000	1.11702127	-	1.42857142	1.272796353
Sptan1	P16546	637	AGQK(*)LIDVNHYAK(*)	K4	44.82	130.84	-	0.52631578	-	
Sptan1	P16546	1419	EK(*)LDILDQER(*)	K2	43.42	1000	1.36170212	1.03947368	1.81318681	1.404787542
Sptan1	P16546	670	LLEATELK(*)GIK(*)	K8	42.17	26.52	0.55319148	-	-	
Sptan1	P16546	150	LLQAQK(*)LVQYLR(*)	K6	26.19	1000	-	1.48684210	-	
Sptan1	P16546	1334	AK(*)LGDSHDLQR(*)	K2	13.38	1000	-	-	1.087912088	
Gapdh	P16858	225	VIPELNGK(*)LTGMAFR(*)	K8	95.59	1000	1	0.80722891	1.02272727	0.94331873
Gapdh	P16858	192	TVDGPSGK(*)LWR(*)	K8	89.57	1000	0.85567010	0.85542168	0.96590909	0.892333627
Gapdh	P16858	252	LEK(*)PAK(*)YDDIK(*)	K6	71.75	56.34	-	0.66265060	0.73863636	0.700643483
Gapdh	P16858	64	AENGK(*)LVINGK(*)	K5	64.64	129.72	-	-	0.727272727	
Gapdh	P16858	137	FVMGVNHEK(*)YDNSLK(*)	K9	16.29	28.13	-	3.18072289	-	
Eno1	P17182	406	LAK(*)YNQILR(*)	K3	92.29	1000	1.06382978	0.95180722	0.96703296	0.994223328
Eno1	P17182	60	FMGK(*)GVSQAVEHINK(*)	K4	87.31	195.68	-	0.78313253	1.03296703	0.908049782
Eno1	P17182	92	IDK(*)LMIEMDGTENK(*)	K3	80.66	201.04	1	1.08433734	0.98901098	1.024449446
Eno1	P17182	256	SGK(*)YDLDFK(*)	K3	70.35	156.85	1.17021276	0.97590361	3.81318681	1.986434398
Eno1	P17182	335	AASEK(*)SC(+57.02)NC(+57.0	K5	60.65	139.75	-	0.92771084	0.92307692	0.925393883

Ptbp1	P17225	258	IDFSK(*)LTSLNVK(*)	K5	70.35	110.43	0.94736842	0.93421052	0.89247311	0.924684022
Ptbp1	P17225	83	GK(*)VTNLLMLK(*)	K2	40.6	132.11	0.78947368	-	-	
Ptbp1	P17225	265	LTSLNVK(*)YNNDK(*)	K7	30.86	47.64	-	2.26315789	-	
Ppia	P17742	131	HVVFGK(*)VK(*)	K6	95.4	36.05	-	1.04819277	0.88043478	0.964313777
Ppia	P17742	82	SIYGEK(*)FEDENFILK(*)	K6	87.33	137.2	0.85	0.85542168	0.90217391	0.869198533
Ppia	P17742	44	ALSTGEK(*)GFGYK(*)	K7	83.57	82.34	0.85	0.89156626	0.94565217	0.89573948
Ppia	P17742	125	TEWLDGK(*)HVVFGK(*)	K7	82.22	87.26	0.96	0.97590361	0.92391304	0.953272219
Ppia	P17742	49	GFGYK(*)GSSFHR(*)	K5	78.09	1000	-	0.95180722	1.01086956	0.981338397
Tpi1	P17751	238	TATPQQAQEVHEK(*)LR(*)	K13	87.72	1000	-	0.85135135	0.94382022	0.897585788
Tpi1	P17751	199	VVFEQTK(*)VIADNVK(*)	K7	72.97	161.52	1.21978022	1.06756756	0.96629213	1.084546641
Ctsd	P18242	339	VSSLPTVYLK(*)LGGK(*)	K10	42.97	31.27	1.27659574	1.02666666	-	1.151631206
Cfl1	P18760	132	LTGIK(*)HELQANC(+57.02)YEE	K5	91.55	186.68	1.12244898	0.94805194	1.01162790	1.027376278
Cfl1	P18760	114	SK(*)MIYASSK(*)	K2	87.01	128.79	-	1	1.17441860	1.087209303
Cfl1	P18760	19	VFNDMK(*)VR(*)	K6	66.68	1000	1.07142857	1.18181818	0.98837209	1.080539615
Cfl1	P18760	132	LTGIK(*)HELQANC(+57.02)YEE	K5	65.67	151.17	1.19387755	1.42857142	0.91860465	1.18035121
Fasn	P19096	673	QEGVFAK(*)EVR(*)	K7	39.3	1000	1.64210526	2.75641025	2.09782608	2.165447202
Fasn	P19096	1143	ELQLC(+57.02)K(*)GLAR(*)	K6	24.88	1000	-	1.46153846	-	
Rpl13a	P19253	191	IC(+57.02)K(*)FTEVLK(*)	K3	74.89	113.99	0.94117647	0.82716049	1.05263157	0.940322848
Rpl13a	P19253	191	K(*)IC(+57.02)K(*)FTEVLK(*)	K4	59.47	38.16	0.78431372	0.80246913	-	0.793391431
Rpl13a	P19253	148	LAHEVGWK(*)YQAVTATLEEK(*	K8	55.7	156.9	1.12745098	0.54320987	1.44210526	1.037588707
Serpinh1	P19324	100	AVLSAEK(*)LR(*)	K7	83.75	1000	0.92857142	0.77215189	0.97894736	0.893223565
Serpinh1	P19324	34	K(*)PLEAAAPGTAEK(*)LSSK(*)	K13	76.45	88.98	0.94897959	0.86075949	0.81052631	0.873421801
Serpinh1	P19324	318	GVVEVTHDLQK(*)HLAGLGLTE	K11	25.57	24.74	-	1.77215189	-	
Serpinh1	P19324	212	PHWDEK(*)FHHK(*)	K6	21.38	82.24	-	1.37974683	-	
Serpinh1	P19324	38	LSSK(*)ATTLAER(*)	K4	17.09	1000	-	-	1.178947368	
Hspa5	P20029	327	AK(*)FEELNMDLFR(*)	K2	70.58	1000	1.12765957	1.02439024	0.96703296	1.039694262
Hspa5	P20029	548	MVNDAEK(*)FAEEDK(*)K(*)	K7	46.96	84.46	0.82978723	0.75609756	-	0.792942398
Hspa5	P20029	345	PVQK(*)VLEDSDLK(*)	K4	16.43	101.72	-	1	-	
Vim	P20152	188	EK(*)LQEEMLQR(*)	K2	97.06	1000	0.95833333	1.01265822	0.94444444	0.971812002
Vim	P20152	294	SK(*)FADLSEAANR(*)	K2	91.03	1000	0.88541666	1.03797468	0.98888888	0.97076008
Vim	P20152	120	FANYIDK(*)VR(*)	K7	68.52	1000	1.05208333	0.97468354	1	1.008922292
Vim	P20152	104	TNEK(*)VELQELNDR(*)	K4	50.03	1000	1.19791666	0.84810126	-	1.023008967
Prkca	P20444	172	AEVTDEK(*)LHVTVR(*)	K7	15.95	1000	-	0.89873417	-	
Tpm3	P21107	232	ILTDK(*)LK(*)	K5	60.36	33.18	-	1.27710843	-	

Ubl4a	P21126	33	HLVSDK(*)LNVPVR(*)	K6	17.14	1000	1.21590909	-	0.94565217	1.080780633
Eno3	P21550	275	HISGEK(*)LGELYK(*)	K6	12.85	53.23	3.05263157	-	-	
Cbx3	P23198	50	VEYFLK(*)WK(*)	K6	23.58	39.76	1.71559633	-	-	
Bcat1	P24288	368	ILGK(*)LTDIQYGR(*)	K4	22.2	1000	1.45652173	-	-	
Ppib	P24369	165	TSWLDGK(*)HVVFGK(*)	K7	32.65	102.87	-	1.5949367C	-	
Rps2	P25444	65	AEDK(*)EWIPVTK(*)LGR(*)	K11	76.52	148.49	0.98979591	0.87654321	0.90109890	0.922479343
Rps2	P25444	65	EWIPVTK(*)LGR(*)	K7	66.68	1000	0.94897959	0.77777777	1.07692307	0.934560149
Tln1	P26039	1314	GISMSSSK(*)LLLAAK(*)	K8	61.32	113.99	-	0.96103896	-	
Tln1	P26039	1306	AQVVSNLK(*)GISMSSSK(*)	K8	61.24	135.04	0.95789473	0.96103896	0.88043478	0.933122827
Tln1	P26039	1544	TIK(*)ALDGDFTEENR(*)	K3	35.33	1000	-	1.87012987	-	
Ezr	P26040	79	K(*)ENPVQFK(*)FR(*)	K8	40.32	67.87	1.74509803	-	-	
Msn	P26041	79	ESPLLFK(*)FR(*)	K7	90.77	1000	1.18947368	0.71604938	0.95555555	0.953692874
Msn	P26041	388	AQSEAEK(*)LAK(*)	K7	83.6	40.63	1.07368421	0.88888888	0.97777777	0.980116959
Msn	P26041	79	K(*)ESPLLFK(*)FR(*)	K8	74.1	118.17	0.74736842	0.67901234	0.93333333	0.786571367
Msn	P26041	350	EK(*)EELMEK(*)LK(*)	K8	61.98	22.85	0.96842105	0.91358024	0.93333333	0.938444878
Msn	P26041	151	SGYLAGDK(*)LLPQR(*)	K8	61.6	1000	-	1.39506172	-	
Msn	P26041	60	AFSTWLK(*)LNK(*)	K7	60.57	30.72	0.85263157	-	0.92222222	0.887426901
Msn	P26041	523	HLK(*)ALTSELANAR(*)	K3	50.92	1000	0.86315789	0.74074074	1.03333333	0.879077323
Msn	P26041	27	TTGK(*)QLFDQVVK(*)	K4	50.81	115.01	2.86315789	0.67901234	-	1.771085121
Msn	P26041	162	VLEQHK(*)LNK(*)	K6	34.63	40.63	0	-	-	
Rdx	P26043	79	K(*)ENPLQFK(*)FR(*)	K8	48.82	51.59	1.09574468	0.73076923	-	0.913256956
Ctnna1	P26231	163	VVEDGILK(*)LR(*)	K8	29.95	1000	0.94949494	1.84810126	-	1.398798108
Glud1	P26443	84	GASIVEDK(*)LVEDLK(*)	K8	48.82	108.85	0.84693877	0.86419753	1.47252747	1.06122126
Glud1	P26443	527	TAMK(*)YNLGLDLR(*)	K4	27.16	1000	1.73469387	1.22222222	-	1.47845805
Psmd7	P26516	204	GLNSK(*)LLDIR(*)	K5	69.52	1000	1.08421052	-	1.06666666	1.075438597
Psmd7	P26516	199	ITNQVHGLK(*)GLNSK(*)	K9	38.48	61.26	1.47368421	1.31645569	1.1	1.296713302
Marcks	P26645	158	SFK(*)LSGFSFK(*)	K3	14.61	115.21	1.5	-	-	
Man2a1	P27046	1103	MSVLK(*)LFNK(*)	K5	31	87.62	-	1.26582278	-	
Snrpb	P27048	8	SSK(*)MLQHIDYR(*)	K3	56.99	1000	-	0.87804878	0.91578947	0.896919127
Rpl3	P27659	373	FIDTTSK(*)FGHGR(*)	K7	83.88	1000	1.02040816	1.01265822	0.91666666	0.983244353
Rpl3	P27659	366	IDLK(*)FIDTTSK(*)	K4	77.98	96.04	0.92857142	1.02531645	0.90625	0.953379295
Rpl3	P27659	286	IYK(*)IGQGYLIK(*)	K3	76.49	161.77	1.15306122	1.07594936	1.08333333	1.104114641
Rpl3	P27659	229	GYK(*)GVTSR(*)	K3	72.32	1000	1.10204081	1.05063291	0.98958333	1.04741902
Pdia3	P27773	335	GEK(*)FVMQEEFSR(*)	K3	87.32	1000	0.93069306	0.81707317	0.95789473	0.901886992

Pdia3	P27773	296	FLDAGHK(*)LNFAVASR(*)	K7	66.68	1000	0.93069306	0.90243902	1	0.944377364
Pdia3	P27773	214	PLHLANK(*)FEDK(*)	K7	53.13	81.18	-	0.69512195	0.83157894	0.763350449
Pdia3	P27773	218	FEDK(*)TVAYTEK(*)	K4	38.04	142.27	-	2.32926829	1.38947368	1.859370989
Adh5	P28474	120	VTQGK(*)GLMPDGTSR(*)	K5	37.61	1000	-	-	0.290697674	
Bgn	P28653	220	ISEAK(*)LTGIPK(*)	K5	32.33	102.61	-	-	1.62	
Nap1l1	P28656	82	VNALK(*)NLQVK(*)	K5	68.01	107.29	1.01123595	1.20779220	0.94117647	1.053401545
Nap1l1	P28656	82	R(*)VNALK(*)NLQVK(*)	K6	49.09	76.17	-	1.22077922	-	
Pabpc1	P29341	213	ELFGK(*)FGPALSVK(*)	K5	70.17	70.68	0.38541666	-	1.06451612	0.724966398
Pabpc1	P29341	512	TVPQYK(*)YAAGVR(*)	K6	30.73	1000	1.67708333	0.1625	1.22580645	1.021796595
Oat	P29758	374	GK(*)GLLNAIVIR(*)	K2	64.69	1000	0.94174757	0.82716049	0.90109890	0.890002323
Oat	P29758	107	SQVDK(*)LTLTSR(*)	K5	37.68	1000	1	-	0.73626373	0.868131868
Fkbp4	P30416	354	ALELDSNNEK(*)GLFR(*)	K10	54.84	1000	0.72916666	0.7375	-	0.733333334
Hmgb2	P30681	59	SK(*)FEDLAK(*)	K2	44.47	121.92	1.23863636	1.07042253	1.33333333	1.214130744
Dbi	P31786	55	AK(*)WDSWNK(*)	K2	68.62	143.14	-	-	0.931034483	
Dbi	P31786	61	WDSWNK(*)LK(*)	K6	54.16	26.31	1	0.60526315	0.80459770	0.803286953
Ssb	P32067	228	ALEGK(*)MGC(+57.02)LLK(*)	K5	53.52	85.52	-	0.82278481	1.19101123	1.006898023
Ssb	P32067	78	AK(*)LMEVSADK(*)	K2	51.39	98.53	-	-	0.595505618	
Ssb	P32067	303	VTWK(*)VLEGHAEK(*)	K4	12.12	99.23	-	-	1.426966292	
Serpinc1	P32261	261	SK(*)FSPENTR(*)	K2	21.04	1000	-	0	0	0
Ranbp1	P34022	182	VAEK(*)LEALSVR(*)	K4	66.13	1000	1.13636363	0.92207792	1.06818181	1.042207792
Mif	P34884	78	NYSK(*)LLC(+57.02)GLLSDR(*	K4	95.91	1000	1.15306122	1.09589041	0.95698924	1.068646961
Rab18	P35293	58	AK(*)LAIWDTAGQER(*)	K2	38.28	1000	1.56862745	-	-	
Fbl	P35550	149	SK(*)LAAAILGGVDQIHIK(*)PG	K2	49.01	170.87	0.6875	-	-	
Canx	P35564	119	LPGDK(*)GLVLMSR(*)	K5	93.58	1000	1	1.01298701	0.94444444	0.985810486
Canx	P35564	104	YDGK(*)WEVDEMK(*)	K4	52.97	72.34	1.28125	1.36363636	-	1.322443182
Canx	P35564	114	ETK(*)LPGDK(*)	K3	14.04	41.96	-	-	1.044444444	
Prdx1	P35700	136	GLFIIDDK(*)GILR(*)	K8	50.26	1000	1.30107526	1.07407407	1.24418604	1.20644513
Rpl12	P35979	54	ATGDWK(*)GLR(*)	K6	74.16	1000	0.98901098	0.94047619	1.06818181	0.999222999
Rpl12	P35979	61	ITVK(*)LTIQNR(*)	K4	33.99	1000	-	1.11904761	1.42045454	1.269751082
Rpl18	P35980	97	ILEVPK(*)LK(*)	K6	58.72	20.41	1.35483871	-	-	
Rpl18	P35980	30	LLVK(*)LYR(*)	K4	18.56	1000	-	1.33333333	-	
Sar1a	P36536	146	TDAISEEK(*)LR(*)	K8	69.64	1000	-	0.67469879	-	
Sar1a	P36536	27	SGK(*)LVFLGLDNAGK(*)	K3	67.89	223.6	0.69747899	0.90361445	0.875	0.825364483
Hspa9	P38647	345	HLNMK(*)LTR(*)	K5	76.37	1000	-	0.77215189	-	

Tkt	P40142	327	AYGLALAK(*)LGHASDR(*)	K8	56.19	1000	1.2	1.05194805	-	1.125974026
Tkt	P40142	465	AVELAANTK(*)GIC(+57.02)FIR	K9	20.95	1000	-	0.68831168	0.60869565	0.64850367
Rpl28	P41105	101	NK(*)YR(*)PDLR(*)	K2	70.18	1000	1.03883495	-	-	
Cd63	P41731	138	TATILDK(*)LQK(*)	K7	25.43	53.53	-	1.08064516	-	
Cct8	P42932	466	ANEVISK(*)LYSVHQEGNK(*)	K7	79.25	68.81	0.89795918	0.9875	0.81914893	0.90153604
Cct8	P42932	62	LEK(*)LFVTNDAATILR(*)	K3	62.93	1000	0.91836734	1.05	1.07446808	1.014278477
Cct8	P42932	318	LNSK(*)WDLR(*)	K4	20	1000	-	-	1.425531915	
H1-4	P43274	90	SLVSK(*)GTLVQTK(*)	K5	28.45	115.21	-	1.68115942	-	
H1-1	P43275	87	LGLK(*)SLVNK(*)	K4	93.94	98.37	1.13186813	1.11764705	1.1	1.116505064
H1-1	P43275	108	GTGAAGSFK(*)LNK(*)	K9	86.84	47.36	1.14285714	1.07352941	1.1625	1.126295518
Akr1b1	P45376	78	IVSK(*)LWC(+57.02)TFHDK(*)	K4	17.49	107.73	1.5	1.01219512	-	1.256097561
Rangap1	P46061	31	GLK(*)LNTAEDAK(*)	K3	55.9	154.74	0.90909090	0.93670886	1.20212766	1.01597581
Aldh2	P47738	443	ANDSK(*)YGLAAAVFTK(*)	K5	36.28	187.48	1.58585858	2.01265822	-	1.799258407
Capza1	P47753	97	NQISFK(*)FDHLR(*)	K6	32.69	1000	1.56989247	1.49295774	-	1.53142511
Capza2	P47754	97	IC(+57.02)FK(*)FDHLR(*)	K4	30.58	1000	-	0.74418604	1.45	1.097093024
Rpl6	P47911	247	EK(*)YEITEQR(*)	K2	89.19	1000	-	1.11538461	1.06666666	1.091025641
Rpl6	P47911	200	THQK(*)FVIATSTK(*)	K4	82.41	131.72	0.86538461	1.05128205	0.98888888	0.968518518
Rpl6	P47911	218	IPK(*)HLTDAYFK(*)	K3	67.77	171.6	-	1.05128205	0.98888888	1.02008547
Rpl29	P47915	101	LAFIAHPK(*)LGK(*)	K8	85.05	49.37	1.11111111	0.94666666	1.03571428	1.031164021
Rpl29	P47915	33	YESLK(*)GVDPK(*)	K5	34.61	81.69	-	3.54666666	1.25	2.398333334
Rpl5	P47962	48	YNTPK(*)YR(*)	K5	76.5	1000	1	0.88505747	-	0.942528736
Rpl5	P47962	43	NK(*)YNTPK(*)	K2	62.52	68.6	-	-	0.989690722	
Rpl5	P47962	158	TTTGNK(*)VFGALK(*)	K6	54.66	85.52	-	0.70114942	-	
Rpl5	P47962	270	WNR(*)PK(*)MSLAQK(*)	K5	13.05	69.64	-	-	1.360824742	
Rpl13	P47963	123	SK(*)LILFPR(*)	K2	48.18	1000	1.19791666	1.24	1.68131868	1.373078449
Anxa5	P48036	74	SELTGK(*)FEK(*)	K6	93.11	57.45	0.90526315	0.79487179	0.98863636	0.896257106
Anxa5	P48036	56	QEIAQEFK(*)TLFGR(*)	K8	39.71	1000	-	1.66666666	0.75	1.208333334
Lmna	P48678	270	TYSAK(*)LDNAR(*)	K5	95.68	1000	-	1.01282051	0.96590909	0.989364802
Lmna	P48678	171	GQVAK(*)LEAALGEAK(*)	K5	85.92	206.63	0.81443299	0.88461538	0.98863636	0.895894913
Lmna	P48678	135	LK(*)DLEALLNSK(*)	K2	73.57	203.33	1.01030927	1	0.98863636	0.999648547
Lmna	P48678	450	VAVEEVDEEGK(*)FVR(*)	K11	40.75	1000	1.02061855	0.75641025	1.70454545	1.160524756
Hnrnpa1	P49312	166	IVIQK(*)YHTVNGHNC(+57.02)	K5	20	1000	-	1.36708860	-	
Cav1	P49817	57	EIDLVNR(*)DPK(*)HLNDDVVK	K10	45.3	53.45	-	0.85333333	-	
Ahcy	P50247	188	SK(*)FDNLYGC(+57.02)R(*)	K2	61.53	1000	0.90526315	1.07792207	0.95604395	0.979743064

Ahcy	P50247	401	K(*)LDEAVAEAHLGK(*)LNVK(*	K13	46.59	25.7	-	0.76623376	0.59340659	0.67982018
S100a11	P50543	50	NQK(*)DPGVLDR(*)	K3	73.11	1000	-	1.125	1.05434782	1.089673913
Pa2g4	P50580	22	YK(*)MGGDIANR(*)	K2	45.65	1000	-	1.24691358	-	
Rpl9	P51410	121	NFLGEK(*)YIR(*)	K6	81.66	1000	1.06315789	1.025	0.90804597	0.998734624
Slc25a5	P51881	155	EFK(*)GLGDC(+57.02)LVK(*)	K3	78.33	124.34	1.17391304	1.24285714	0.97777777	1.131515988
Slc25a5	P51881	33	VK(*)LLLQVQHASK(*)	K2	77.85	250.23	1.09782608	1.12857142	1.1	1.108799172
Pkm	P52480	270	IISK(*)IENHEGVR(*)	K4	79.42	1000	-	0.875	1.02197802	0.948489011
Pkm	P52480	141	GATLK(*)ITLDNAYMEK(*)	K5	52.7	207.58	-	1.4625	-	
Pkm	P52480	498	VNLAMDVGK(*)AR(*)	K9	43.11	1000	-	-	1.043956044	
Pkm	P52480	166	NIC(+57.02)K(*)VVEVGSK(*)	K4	28.23	120.29	-	1.3375	1.72527472	1.531387363
Sult1a1	P52840	118	LLDQK(*)IK(*)	K5	16.77	22.85	-	-	-	
Rpl10a	P53026	156	STIK(*)FQMK(*)	K4	69.95	88.98	-	0.97590361	0.93548387	0.955693743
Rpl10a	P53026	133	AGK(*)FPSLLTHNENMVAK(*)	K3	37.35	88.07	2.27956989	1.46987951	0.81720430	1.522217904
Slc16a1	P53986	235	GEK(*)LSVFQTINK(*)	K3	58.57	181.94	-	0.80487804	-	
Idh2	P54071	384	GK(*)LDGNQDLIR(*)	K2	69.5	1000	-	0.71428571	0.80898876	0.761637239
Idh2	P54071	69	EK(*)LILPHVDVQLK(*)	K2	21.24	134.4	-	2.58441558	-	
Rad23b	P54728	45	DAFPVAGQK(*)LIYAGK(*)	K9	76.44	94.27	1.02173913	1.04109589	0.92045454	0.994429855
Atp5mf	P56135	48	YYNK(*)YINVR(*)	K4	30.24	1000	-	1.5	-	
Atp5f1b	P56480	201	GGK(*)IGLFGGAGVGK(*)	K3	72.53	178.02	1.09375	0.8875	1.02127659	1.000842199
Atp5f1b	P56480	124	GQK(*)VLDSGAPIK(*)	K3	46.89	140.39	-	0.7375	-	
Atp5f1b	P56480	480	MGK(*)LVPLK(*)	K3	12.94	95.62	2.07291666	-	-	
Eef1d	P57776	17	FK(*)YDDAER(*)	K2	83.1	1000	-	0.97590361	1	0.987951807
Eef1d	P57776	15	IWFDK(*)FK(*)	K5	64.43	28.86	1.24210526	1.02409638	-	1.133100825
Actn4	P57780	418	LDHLAEK(*)FR(*)	K7	81.44	1000	1.125	1.07692307	1.01098901	1.070970696
Actn4	P57780	332	TIQEMQQK(*)LEDFR(*)	K8	60.6	1000	1	0.94871794	0.82417582	0.924297924
Actn4	P57780	123	ALDFIASK(*)GVK(*)	K8	48.81	32.97	-	1.05128205	-	
Actn4	P57780	115	INNVNK(*)ALDFIASK(*)	K6	45.78	142.27	1	-	-	
Snrpa1	P57784	221	LK(*)GLLQSGQIPGR(*)	K2	28.3	1000	1.18556701	-	-	
Snrpa1	P57784	67	LK(*)TLLVNNNR(*)	K2	14.3	1000	-	1.23456790	-	
Idi1	P58044	192	AASGEIK(*)LTPWFK(*)	K7	22.1	90.32	1.27659574	-	-	
Eef2	P58252	498	VMK(*)FSVSPVVR(*)	K3	95.43	1000	0.96842105	0.9	0.95744680	0.941955954
Eef2	P58252	318	LIEK(*)LDIK(*)	K4	93.46	109.97	1.06315789	1.0125	0.92553191	1.000396603
Eef2	P58252	322	LDIK(*)LDSEDK(*)DK(*)	K4	83.21	113.99	0.93684210	0.95	0.91489361	0.933911907
Eef2	P58252	235	QFAEMYVAK(*)FAAK(*)	K9	80.39	59.3	0.91578947	0.95	0.98936170	0.951717059

Eef2	P58252	426	VFSGVVSTGLK(*)VR(*)	K11	71.55	1000	1.04210526	1.05	1.05319148	1.048432251
Eef2	P58252	400	ISK(*)MVPTSDK(*)	K3	69.99	94.43	1	1.2375	1	1.079166667
Eef2	P58252	314	K(*)EETAK(*)LIEK(*)	K6	51.46	61.3	-	-	1.159574468	
Eef2	P58252	226	TLK(*)QFAEMYVAK(*)	K3	24.44	141.67	-	-	1.79787234	
Eef2	P58252	400	YISK(*)MVPTSDK(*)	K4	12.98	51.59	-	-	1.606382979	
Tpm1	P58771	12	MQMLK(*)LDK(*)	K5	94.93	62.26	0.98936170	0.93506493	1.10588235	1.010102997
Tpm1	P58771	112	LATALQK(*)LEEAEK(*)	K7	88.64	121.92	0.82978723	0.93506493	0.95294117	0.905931115
Tpm1	P58771	231	VLSDK(*)LK(*)	K5	84.52	27.16	1.08510638	1	1.2	1.095035461
Tpm1	P58771	70	DAQEK(*)LELAEK(*)	K5	80.28	151.86	1.86170212	0.89610389	2.17647058	1.644758871
Tpm1	P58771	152	EAK(*)HIAEDADR(*)	K3	72.03	1000	1.06382978	0.98701298	1.11764705	1.056163278
Tpm1	P58771	205	TVTNNLK(*)SLEAQAEK(*)	K7	65	130.09	0.91489361	0.97402597	0.97647058	0.95513006
Tpm1	P58771	37	SK(*)QLEDELVSLQK(*)	K2	61.77	235.98	1.10638297	0.89610389	0.95294117	0.985142684
Tpm1	P58771	12	MQMLK(*)LDK(*)ENALDR(*)	K5	30.75	49.37	1.5	1.89610389	-	1.698051948
Tpm2	P58774	128	GMK(*)VIENR(*)	K3	63.39	1000	-	0.96052631	1.44827586	1.204401089
Tpm2	P58774	70	DAQEK(*)LEQAEK(*)	K5	50.69	151.86	-	0.96052631	1.03448275	0.997504538
Tpm2	P58774	12	MQMLK(*)LDK(*)ENAIDR(*)	K5	30.67	1000	1.48421052	1.92105263	-	1.702631579
Tpm2	P58774	205	IVTNNLK(*)SLEAQADK(*)	K7	13.68	135.81	-	2.51315789	-	
Arpc4	P59999	89	ILC(+57.02)HK(*)FMR(*)	K5	61.34	1000	-	0.93827160	0.81720430	0.877737953
Eif3e	P60229	359	MLADK(*)LNMTPEEAER(*)	K5	47.3	1000	-	1.325	-	
Cdc42	P60766	163	YVEC(+57.02)SALTQK(*)GLK(*	K10	77.98	62.26	-	1	1.15384615	1.076923077
Rps20	P60867	34	SLEK(*)VC(+57.02)ADLIR(*)	K4	88.27	1000	1.02	0.92405063	0.93	0.958016878
Ube2n	P61089	82	IYHPNVDK(*)LGR(*)	K8	54.49	1000	-	0.89333333	1.55172413	1.222528736
Ube2n	P61089	94	DK(*)WSPALQIR(*)	K2	48.36	1000	1.6	1.12	0.81609195	1.178697318
Actr2	P61161	388	VLEK(*)LGVTVR(*)	K4	56.97	1000	1.58947368	-	0.94505494	1.267264315
Actr2	P61161	322	ELK(*)QLYLER(*)	K3	47.09	1000	1.30526315	0.8	1.09890109	1.068054752
Actr2	P61161	46	STTK(*)VGNIEIK(*)	K4	23.92	129.86	-	1.2375	-	
Rpl26	P61255	2	MK(*)FNPFVTSDR(*)	K2	48.21	1000	-	-	1.155555556	
Rpl26	P61255	110	LK(*)LDK(*)DR(*)	K2	37.94	77.24	0.88349514	1.01234567	0.93333333	0.943058053
Rpl27	P61358	133	WFFQK(*)LR(*)	K5	82.36	1000	1.03092783	0.81818181	1.07777777	0.975629144
Rpl27	P61358	3	GK(*)FMK(*)PGK(*)	K2	63.07	78.06	0.72164948	-	1	0.860824743
Rpl27	P61358	128	NK(*)WFFQK(*)	K2	60.35	105.53	1.45360824	1.02272727	-	1.23816776
Rpl37a	P61514	13	VGIVGK(*)YGTR(*)	K6	65.3	1000	-	-	0.989795918	
Nutf2	P61971	55	AAIVEK(*)LSSLPFQK(*)	K6	58.32	135.81	1.42696629	0.88607594	-	1.156521121
Rps7	P62082	147	VK(*)LDGSR(*)	K2	77.51	1000	1.19387755	-	1.27659574	1.235236648

Rps7	P62082	74	SFQK(*)IQVR(*)	K4	15.72	1000	1.66326530	-	-	
Rps8	P62242	128	GAK(*)LTPEEEEILNK(*)	K3	73.21	142.64	-	0.6625	-	
Rps8	P62242	37	PAANTK(*)IGPR(*)	K6	55.98	1000	0.91752577	0.8625	0.91208791	0.897371228
Rps8	P62242	157	NAK(*)ISSLLEEQFQQGK(*)	K3	40.85	226.56	1.50515463	0.8125	1.75824175	1.358632132
Rps15a	P62245	71	LNK(*)C(+57.02)GVISPR(*)	K3	45.14	1000	1.04166666	1.23170731	1.43956044	1.237644808
Rps15a	P62245	88	DLEK(*)WQNNLLPSR(*)	K4	44.11	1000	1.16666666	-	-	
Rps15a	P62245	43	MK(*)HGYIGEFEIIDDHR(*)	K2	43	1000	1.60416666	-	-	
Ywhae	P62259	12	AK(*)LAEQAER(*)	K2	92.06	1000	1.09183673	0.90123456	1.01086956	1.001313623
Ywhae	P62259	123	VFYYK(*)MK(*)	K5	87.78	33.18	1.02040816	0.87654321	1.02173913	0.972896834
Ywhae	P62259	78	EENK(*)GGEDK(*)LK(*)	K9	87.12	36.05	0.92857142	0.75308642	0.97826087	0.886639573
Ywhae	P62259	50	NLLSVAYK(*)NVIGAR(*)	K8	32.92	1000	1.58163265	1.25925925	-	1.420445956
Rps14	P62264	96	ELGITALHIK(*)LR(*)	K10	89.6	1000	1.00980392	1.05952381	0.92391304	0.997746925
Rps14	P62264	106	TK(*)TPGPGAQSALR(*)	K2	79.91	1000	-	0.72619047	0.90217391	0.814182195
Rps23	P62267	54	GIVLEK(*)VGVEAK(*)	K6	25.57	113.99	1.25490196	1.80555555	-	1.530228759
Rps23	P62267	37	AHLGTALK(*)ANPFGGASHAK(	K8	21.36	70.27	1.14705882	-	-	
Rps18	P62270	106	YSQVLANGLDNK(*)LR(*)	K12	74.83	1000	1.04040404	1.06024096	0.92391304	1.008186016
Rps18	P62270	34	IAFAITAIK(*)GVGR(*)	K9	56.9	1000	1.07070707	0.90361445	1.09782608	1.024049205
Rps18	P62270	94	DGK(*)YSQVLANGLDNK(*)	K3	50.4	243.69	1.24242424	-	-	
Rps18	P62270	78	QYK(*)IPDWFLNR(*)	K3	31.33	1000	0.71717171	-	1.59782608	1.157498902
Rps18	P62270	34	AITAIK(*)GVGR(*)	K6	14.71	1000	-	0.83132530	-	
Rps11	P62281	38	YYK(*)NIGLGFK(*)	K3	66.67	62.93	1.06730769	-	-	
Rps13	P62301	112	DK(*)DAK(*)FR(*)	K5	83.53	91.37	1.22340425	0.975	1	1.066134752
Rps13	P62301	9	MHAPGK(*)GLSQSALPYR(*)	K6	58.71	1000	1.06382978	1.175	-	1.119414894
Rps13	P62301	70	FVTGNK(*)ILR(*)	K6	55.34	1000	1.32978723	-	0.76344086	1.046614047
Rps13	P62301	27	SVPTWLK(*)LTSDDVK(*)	K7	53.86	78.16	1	0.775	0.87096774	0.881989247
Rps13	P62301	78	SK(*)GLAPDLPEDLYHLIK(*)	K2	33.79	170.25	-	-	1.559139785	
Lsm6	P62313	23	PVVVK(*)LNSGVDYR(*)	K5	41.98	1000	0.35874439	-	-	
Psmc6	P62334	68	QLTEEK(*)FIVK(*)	K6	39.91	41.04	-	1.10256410	1.53333333	1.317948718
Psmc6	P62334	206	VVSSSIVDK(*)YIGESAR(*)	K9	33.06	1000	-	-	1.544444444	
Rps4x	P62702	53	LK(*)YALTGDEVK(*)	K2	82.01	181.94	0.96938775	0.93827160	1.11827957	1.00864631
Rps4x	P62702	134	IFVGTK(*)GIPHLVTHDAR(*)	K6	81.88	1000	0.91836734	0.95061728	1.03225806	0.967080899
Rps4x	P62702	16	VAAPK(*)HWMLDK(*)	K5	17.69	66.56	-	-	1.817204301	
Rpl23a	P62751	70	NK(*)LDHYAIIK(*)	K2	69.74	170.86	1.3	1.15189873	1.90588235	1.452593696
Rps6	P62754	2	MK(*)LNISFPATGC(+57.02)QK	K2	60.93	163.81	0.68	0.66666666	1.11578947	0.820818714

H4f16	P62806	9	G(+42.01)K(*)GGK(*)GLGK(*)	K5	77.07	69.71	0.94	0.91764705	1.06382978	0.973825615
H4f16	P62806	92	TVTAMDVVYALK(*)R(*)	K12	74.46	1000	1.03	0.92941176	1.11702127	1.025477681
H4f16	P62806	9	GGK(*)GLGK(*)	K3	51.69	82.24	1.41	-	1.17021276	1.290106383
Rab1A	P62821	119	YASENVNK(*)LLVGNK(*)	K8	80.06	84.46	1.21875	1	0.88172043	1.033490143
Rab1A	P62821	61	TIK(*)LQIWDTAGQER(*)	K3	63.72	1000	0.82291666	0.96153846	-	0.892227565
Rab1A	P62821	61	IK(*)LQIWDTAGQER(*)	K2	45.36	1000	-	-	1.086021505	
Ran	P62827	60	GPIK(*)FNVWDTAGQEK(*)	K4	75.63	195.68	0.78494623	0.85185185	1.16483516	0.933877751
Ran	P62827	71	FNVWDTAGQEK(*)FGGLR(*)	K11	55.36	1000	-	0.91358024	0.90109890	0.907339574
Rpl23	P62830	13	GGSSGAK(*)FR(*)	K7	67.77	1000	1.1875	1.18421052	1.05376344	1.141824656
Rps25	P62852	43	DK(*)LNNLVLFDK(*)	K2	91.7	246.46	0.93548387	0.83333333	0.95505618	0.907957795
Rps25	P62852	94	AALQELLSK(*)GLIK(*)	K9	88.5	82.24	0.95698924	1.08974359	1.03370786	1.026813567
Rps25	P62852	57	ATYDK(*)LC(+57.02)K(*)	K5	86.04	68.47	-	0.92307692	0.87640449	0.899740709
Rps25	P62852	66	YK(*)LITPAVVSER(*)	K2	18.01	1000	-	2.01282051	-	
Rps28	P62858	10	VQPIK(*)LAR(*)	K5	82.81	1000	1.09	0.94047619	0.82828282	0.952919673
Rpl30	P62889	44	AK(*)LVILANNC(+57.02)PALR(	K2	86.2	1000	0.92380952	0.84444444	1.02061855	0.929624175
Rpl30	P62889	26	SGK(*)YVLGYK(*)	K3	68.95	109.28	-	-	0.969072165	
Rpl31	P62900	75	AVWAK(*)GIR(*)	K5	15.5	1000	-	-	2.418367347	
Rps3	P62908	141	GC(+57.02)EVVVSGK(*)LR(*)	K9	84.82	1000	1.08421052	0.98765432	0.94565217	1.005839007
Rps3	P62908	108	YK(*)LLGGLAVR(*)	K2	81.48	1000	0.95789473	0.85185185	0.80434782	0.871364805
Rpl8	P62918	149	VK(*)LPSGSK(*)	K2	12.3	55.13	-	0.80487804	-	
Uba52	P62984	48	LIFAGK(*)QLEDGR(*)	K6	79.95	1000	1.80769230	0.96296296	0.89215686	1.220937378
Uba52	P62984	6	MQIFVK(*)TLTGK(*)	K6	21.62	38.32	3.38461538	1.48148148	1.59803921	2.154712027
Uba52	P62984	88	QLAQK(*)YNC(+57.02)DK(*)	K5	20.55	43.82	-	1.59259259	-	
Rac1	P63001	166	GLK(*)TVFDEAIR(*)	K3	50.6	1000	-	3.84810126	-	
Rac1	P63001	153	EIGAVK(*)YLEC(+57.02)SALTQ	K6	42.43	1000	-	1.08860759	1.38372093	1.236164263
Rac1	P63001	128	DDK(*)DTIEK(*)LK(*)	K8	22.46	21.68	-	-	1.395348837	
Pafah1b1	P63005	351	GVLFHSGGK(*)FILSC(+57.02)A	K9	17.25	127.49	-	1.81081081	-	
Hspa8	P63017	500	ENK(*)ITITNDK(*)	K3	72.21	72.07	-	0.92207792	0.93406593	0.928071928
Hspa8	P63017	348	IQK(*)LLQDFFNGK(*)	K3	62.41	184.54	0.98969072	1.01298701	1.28571428	1.096130674
Hspa8	P63017	128	MK(*)EIAEAYLGK(*)	K2	61.07	181.94	1.04123711	1.02597402	1.08791208	1.051707742
Hspa8	P63017	357	LLQDFFNGK(*)ELNK(*)	K9	51.14	35.13	0.86597938	-	1.19780219	1.03189079
Hspa8	P63017	550	NMK(*)ATVEDEK(*)	K3	20.6	84.68	-	-	1.076923077	
Tpt1	P63028	102	GK(*)LEEQK(*)PER(*)	K2	79.09	56.99	0.78217821	0.6625	-	0.722339109
Hspd1	P63038	473	ALK(*)IPAMTIAK(*)	K3	94.13	97.31	0.90721649	0.88888888	1.02197802	0.939361135

Hspd1	P63038	396	LAK(*)LSDGVAVLK(*)	K3	84.53	186.74	0.91752577	0.90123456	0.91208791	0.910282751
Hspd1	P63038	31	DVK(*)FGADAR(*)	K3	72.92	1000	0.91752577	1.02469135	1.01098901	0.984402047
Hmgb1	P63158	59	GK(*)FEDMAK(*)	K2	84.06	104.48	1.08602150	0.98684210	0.97701149	1.016625035
Hmgb1	P63158	157	YEK(*)DIAAYR(*)	K3	79.88	1000	-	-	0.862068966	
Eif5a	P63242	39	GR(*)PC(+57.02)K(*)IVEMSTSI	K5	61.4	55.71	-	-	1.37755102	
Actg1	P63260	284	SIMK(*)C(+57.02)DVDIR(*)	K4	86.81	1000	0.94174757	0.88461538	0.97777777	0.934713579
Actg1	P63260	328	IK(*)IIAPPER(*)	K2	86.11	1000	0.96116504	0.98717948	0.93333333	0.96055929
Actg1	P63260	50	HQGVMVGMGQK(*)DSYVGDE	K11	47.75	254.02	-	0.64102564	1.47777777	1.05940171
Actg1	P63260	315	MQK(*)EITALAPSTMK(*)	K3	34.85	141.98	-	1.44871794	-	
Actg1	P63260	284	NSIMK(*)C(+57.02)DVDIR(*)	K5	27.39	1000	1.41747572	0.94871794	0.74444444	1.036879374
Rps17	P63276	72	GISIK(*)LQEEER(*)	K5	83.97	1000	1.26315789	1.21333333	0.98888888	1.155126706
Rps17	P63276	49	NK(*)IAGYVTHLMK(*)	K2	80.91	189.31	1.02105263	1.02666666	0.94444444	0.997387914
Rps17	P63276	19	VIIEK(*)YYTR(*)	K5	59.42	1000	0.38947368	0.74666666	2.42222222	1.186120858
Ube2i	P63280	59	GTPWEGGLFK(*)LR(*)	K10	66.72	1000	1	0.95774647	0.94252873	0.966758405
Rpl22	P67984	84	YLK(*)YLTK(*)	K3	47.68	77.13	-	-	0.71875	
Rack1	P68040	183	VWNLANC(+57.02)K(*)LK(*)	K8	91.18	22.85	0.95652173	0.8875	0.96703296	0.937018235
Rack1	P68040	175	LVK(*)VWNLANC(+57.02)K(*)	K3	88.35	183.32	1.04347826	0.8875	1.03296703	0.987981765
Rack1	P68040	44	TIIMWK(*)LTR(*)	K6	75.87	1000	1.07608695	0.8375	1.17582417	1.029803711
Rack1	P68040	130	TIK(*)LWNTLGVC(+57.02)K(*)	K3	66.46	181.94	1.26086956	0.875	1.12087912	1.085582895
Ywhaq	P68254	120	VFYLK(*)MK(*)	K5	95.93	36.05	0.97058823	0.975	1.06521739	1.003601875
Ywhaq	P68254	3	M(+42.01)EK(*)TELIQK(*)	K3	73.23	134.33	1.04901960	-	-	
Ywhaq	P68254	85	EK(*)VESELR(*)	K2	17.66	1000	-	1.2375	-	
Tubb4b	P68372	58	INVYYNEATGGK(*)YVPR(*)	K12	41.81	1000	0.34042553	-	1.10112359	0.720774564
H3c11	P68433	57	YQK(*)STELLIR(*)	K3	98.49	1000	0.94565217	0.94871794	0.90909090	0.934487011
H3c11	P68433	24	K(+42.01)(*)QLATK(*)AAR(*)	K6	97.38	1000	278.260869	-	-	
H3c11	P68433	57	R(*)YQK(*)STELLIR(*)	K4	77.52	1000	1.43478260	1.07692307	1.03409090	1.181932198
H3c11	P68433	123	VTIMPK(*)DIQLAR(*)	K6	75.42	1000	1.43478260	0.91025641	-	1.17251951
H3c11	P68433	80	EIAQDFK(*)TDLR(*)	K7	60.24	1000	1.07608695	0.93589743	1.27272727	1.094903889
H3c11	P68433	123	R(*)VTIMPK(*)DIQLAR(*)	K7	46.2	1000	-	1.33333333	-	
Kpnb1	P70168	835	DVLK(*)LVEAR(*)	K4	61.62	1000	1.30107526	1.03797468	-	1.169524977
Kpnb1	P70168	23	LELEAAQK(*)FLER(*)	K8	43.68	1000	1.18279569	0.87341772	-	1.028106711
Rad50	P70388	874	SEK(*)LQIATNLQR(*)	K3	31.75	1000	-	1.32467532	-	
Naca	P70670	2054	AMSK(*)LGLR(*)	K4	13.68	1000	1.52747252	-	-	
Ctps1	P70698	309	YTK(*)FSDSYASVIK(*)	K3	32.58	133.99	-	2.01315789	-	

Cct7	P80313	47	GMDK(*)LIVDGR(*)	K4	57.32	1000	1.04210526:	0.9113924C	0.96666666	0.973388112
Cct7	P80313	67	ATISNDGATILK(*)LLDVVHPAA	K12	28.04	47.07	-	2	-	
Cct2	P80314	46	STLGPK(*)GMDK(*)	K6	54.18	61.37	-	-	0.597826087	
Cct2	P80314	181	DHFTK(*)LAVEAVLR(*)	K5	40.23	1000	-	1.1625	1.13043478:	1.146467392
Cct2	P80314	284	ILK(*)HGINC(+57.02)FINR(*)	K3	26.32	1000	-	2.075	-	
Cct2	P80314	82	NIGVDNPAAK(*)VLVDMSR(*)	K10	14.91	1000	-	1.0125	-	
Cct4	P80315	489	HAQGEK(*)TTGINVR(*)	K6	63.08	1000	1.17708333:	0.7625	0.77419354:	0.904592294
Cct4	P80315	395	GSNK(*)LVIEEAER(*)	K4	57.27	1000	-	0.75	-	
Cct4	P80315	143	ALEK(*)GLEILTDMSR(*)	K4	44.85	1000	0.76041666	1	1.88172043	1.214045699
Cct4	P80315	375	LFK(*)ITGC(+57.02)TSPGK(*)	K3	27.56	123.34	-	1.7375	-	
Cct5	P80316	265	HK(*)LDVMSVEDYK(*)	K2	68.6	168.06	0.97959183	0.91358024	-	0.946586042
Cct5	P80316	176	TTLGSK(*)VINSC(+57.02)HR(*	K6	61.13	1000	1.20408163:	1.1358024€	1.22826087	1.189381657
Cct5	P80316	223	LEDTK(*)LIK(*)	K5	56.47	62.26	1.06122449	-	1.05434782	1.057786158
Cct5	P80316	284	EK(*)FEEMIK(*)	K2	13.41	95.35	1.15306122	1.53086419	-	1.341962711
Cct6a	P80317	55	MLVSGAGDIK(*)LTK(*)	K10	80.31	32.97	0.87755102	0.85365853	0.93617021:	0.88912659
Cct6a	P80317	45	GTMK(*)MLVSGAGDIK(*)	K4	63.02	158.16	1.14285714:	0.82926829	0.91489361	0.962339684
Cct3	P80318	370	DPK(*)AC(+57.02)TILLR(*)	K3	72.04	1000	0.86458333:	0.49367088	1.05319148	0.803815236
Ap2m1	P84091	256	LSK(*)FDSER(*)	K3	14.8	1000	-	1.6081081C	1.37931034!	1.493709227
Rpl19	P84099	144	ILMEHIHK(*)LK(*)	K8	60.8	22.85	1.125	0.98809523	1.1	1.071031746
Rpl19	P84099	92	MPEK(*)VTWMR(*)	K4	42.82	1000	1.04166666	-	-	
Mcm6	P97311	205	FVDFQK(*)VR(*)	K6	42.26	1000	-	0.88888888	1.30769230	1.098290599
Rps3a	P97351	187	EVVNK(*)LIPDSIGK(*)	K5	90.33	92.28	0.95098039:	0.93975903	0.9375	0.942746476
Rps3a	P97351	85	FK(*)LITEDVQGK(*)	K2	84.16	146.24	0.95098039:	0.8313253C	0.88541666	0.889240787
Rps3a	P97351	56	TQGTK(*)IASDGLK(*)	K5	74.92	57.92	-	1.0602409€	0.89583333:	0.978037149
Rps3a	P97351	109	DK(*)MC(+57.02)SMVK(*)	K2	24.06	95.35	-	1	-	
Rps3a	P97351	227	FELGK(*)LMELHGEGGSSGK(*)	K5	23.71	49.25	-	2.0602409€	-	
Anxa4	P97429	259	SMK(*)GLGTDDNTLIR(*)	K3	29.21	1000	-	2.15189873	-	
Fhl1	P97447	4	S(+42.01)EK(*)FDC(+57.02)HY	K3	59.53	1000	0.91836734	0.97435897	-	0.946363161
Rps5	P97461	47	YAK(*)YLPHSAGR(*)	K3	33.8	1000	-	0.85185185	1.92134831!	1.386600084
Tubb5	P99024	58	ISVYYNEATGGK(*)YVPR(*)	K12	88.12	1000	1.01020408:	1.12658227	0.90322580	1.013337389
Top2a	Q01320	613	YYK(*)GLGTSTSK(*)	K3	19.41	63.7	-	-	1.26744186	
Rsu1	Q01730	158	LTK(*)LQILSLR(*)	K3	58	1000	1.19565217	0.86046511	1.22826087	1.09479272
Nme2	Q01768	39	LVAMK(*)FLR(*)	K5	97.35	1000	1.03157894	0.97530864	1.1	1.035629196
Nme2	Q01768	49	ASEEHLK(*)QHYIDLK(*)	K7	60.95	79.54	0.78947368	0.83950617	-	0.814489929

Vcp	Q01853	505	FLK(*)FGMTPSK(*)	K3	81.89	127.53	0.91666666	0.66666666	0.92307692	0.835470086
Uba1	Q02053	185	GIK(*)LVVADTR(*)	K3	60.48	1000	1.79787234	-	-	
Uba1	Q02053	838	ATLPSPDK(*)LPGFK(*)	K8	15.63	38.32	1.34042553	-	-	
Nucb1	Q02819	70	EK(*)LQAANAEDIK(*)	K2	42.98	165.51	0.87368421	1.63636363	1.22619047	1.245412774
Atp5f1a	Q03265	434	QVAGTMK(*)LELAQYR(*)	K7	64.19	1000	1.01020408	0.95180722	0.67391304	0.878641451
Atp5f1a	Q03265	539	ISEQSDAK(*)LK(*)	K8	61.16	26.31	1.47959183	0.95180722	-	1.215699533
Atp5f1a	Q03265	531	SDGK(*)ISEQSDAK(*)	K4	39.3	117.11	-	1.19277108	0.79347826	0.993124673
Top1	Q04750	722	QIALGTSK(*)LNYLDPR(*)	K8	39.59	1000	1.71568627	0.48837209	-	1.102029184
Fabp5	Q05816	10	DLEGK(*)WR(*)	K5	14.64	1000	-	1.58536585	-	
Cab39	Q06138	196	HK(*)LLSAEFLEQHYDR(*)	K2	60.27	1000	-	-	1.068181818	
Cnn2	Q08093	134	TK(*)GLQSGVDIGVK(*)	K2	73.48	256.29	0.94505494	0.88157894	0.94505494	0.923896279
Cnn2	Q08093	25	LLSK(*)YDPQK(*)	K4	69.05	98.37	-	0.89473684	0.97802197	0.93637941
Cnn2	Q08093	234	HIYDTK(*)LGTDK(*)	K6	67.82	70.86	-	1.09210526	0.87912087	0.985613071
Cnn2	Q08093	55	GLK(*)DGVILC(+57.02)TLMNK	K3	35.23	249.7	0.85714285	1.77631578	-	1.316729323
Npepps	Q11011	280	AEQGK(*)FALEVAAK(*)	K5	37.94	142.27	1.26881720	0.60526315	1.36046511	1.078181826
Ddb1	Q3U1J4	570	ILK(*)LPSFELLHK(*)	K3	32.78	143.22	1.48453608	1.21518987	-	1.349862978
Pdap1	Q3UHX2	126	YMK(*)MHLAGK(*)	K3	54.31	121.92	-	-	0.784090909	
Edc4	Q3UJB9	1097	SK(*)NLTDAIAR(*)	K2	17.02	1000	1.54444444	-	-	
Abracl	Q4KML4	25	NADGK(*)LSVK(*)	K5	15.78	48.97	-	1.26506024	1.05319148	1.159125865
Arcn1	Q5XJY5	309	ISDDK(*)FGR(*)	K5	15.3	1000	-	-	1.208791209	
Cttn	Q60598	235	GFGGK(*)FGVQTDR(*)	K5	37.52	1000	1.18367346	1.62337662	1.03260869	1.279886263
Cttn	Q60598	124	GFGGK(*)FGVQMDR(*)	K5	25.3	1000	-	-	1.206521739	
Cttn	Q60598	87	ASHGYGGK(*)FGVEQDR(*)	K8	20.54	1000	-	-	1.25	
Myl6	Q60605	56	VLGNPK(*)SDEMNVK(*)	K6	81.14	102.29	0.99	0.8625	0.82954545	0.894015152
Myl6	Q60605	26	TGDGK(*)ILYSQC(+57.02)GDV	K5	59.4	1000	-	0.925	-	
Ppp5c	Q60676	111	AASNMALGK(*)FR(*)	K9	53.45	1000	-	-	0.90625	
P4ha1	Q60715	320	NPK(*)FILAPAK(*)	K3	23.06	161.52	0.95789473	1.32894736	1.46067415	1.249172087
Serpinb6	Q60854	100	LFGDK(*)TC(+57.02)DLLASFK(	K5	37.54	195.94	-	1.76623376	-	
Stip1	Q60864	284	ELC(+57.02)EK(*)AIEVGR(*)	K5	33.54	1000	-	1.4625	-	
Stip1	Q60864	229	EK(*)ELGNDAYK(*)	K2	32.12	99.23	-	1.275	0.95652173	1.11576087
Stip1	Q60864	169	ELIEQLQNK(*)PSDLGTK(*)LQD	K16	26.81	83.89	-	3.15	2.03260869	2.591304348
Stip1	Q60864	136	NLPNLYQK(*)LENDPR(*)	K8	16.89	1000	1.74489795	2.2375	1.81521739	1.93253845
Vdac3	Q60931	163	SK(*)LSQNNFALGYK(*)	K2	43.41	213.66	1.3	0.96385542	1.44210526	1.235320228
Vdac1	Q60932	109	GLK(*)LTFDSSFSPNTGK(*)	K3	32.7	178.91	2.32291666	2.06024096	-	2.191578816

Hars	Q61035	257	NEMVGEK(*)GLAPEVADR(*)	K7	25.09	1000	-	1.74358974	-	
Mapre1	Q61166	204	VLK(*)LTVEDLEK(*)	K3	73.28	150.63	-	1.02531645	1.23456790	1.129942179
Mapre1	Q61166	220	DFYFGK(*)LR(*)	K6	21.59	1000	-	-	1.444444444	
Prdx2	Q61171	29	EIK(*)LSDYR(*)	K3	18.68	1000	1.10526315	-	-	
Hspa4	Q61316	610	MIMQDK(*)LEK(*)	K6	61.68	53.53	1.77894736	0.875	-	1.326973684
Hspa4	Q61316	675	QVYVDK(*)LAELK(*)	K6	50.72	76.42	1.32631578	0.775	1.08888888	1.063401559
Hspa4	Q61316	680	LAELK(*)SLGQPIK(*)	K5	49.36	142.27	-	1.1625	-	
Hspa4	Q61316	632	DK(*)LSGEYEK(*)	K2	42.18	148.49	-	1.0625	0.9	0.98125
Hspa4	Q61316	305	GK(*)FLEMC(+57.02)DDLLAR(	K2	32.57	1000	1.07368421	1.2375	1.72222222	1.344468811
Hspa4	Q61316	329	SVLEQSK(*)LK(*)	K7	30.66	20.41	1.47368421	-	1.77777777	1.625730995
Bcap31	Q61335	95	VNLQNNPGAMEHFHMK(*)LFF	K16	77.19	1000	-	1.03947368	1.17021276	1.104843225
Bcap31	Q61335	167	GAAEDGDK(*)LDIGNTEMK(*)	K8	44.5	159.26	1.26804123	-	1.28723404	1.27763764
Glg1	Q61543	626	AMDVK(*)LDPALQDK(*)	K5	29.07	109.84	-	1.25333333	-	
Gdi2	Q61598	210	IK(*)LYSESLAR(*)	K2	71.26	1000	1.08163265	1.525	-	1.303316327
Hsph1	Q61699	665	FIC(+57.02)EQEHEK(*)FLR(*)	K9	36.93	1000	-	1.28947368	3.01136363	2.15041866
Phgdh	Q61753	69	VTADVINAAEK(*)LQVVGR(*)	K11	57.02	1000	1.02173913	1.30379746	0.78651685	1.037351151
Phgdh	Q61753	394	QADVNLVNAK(*)LLVK(*)	K10	56.34	88.98	0.83695652	0.86075949	0.73033707	0.809351032
Myh10	Q61879	1800	AK(*)LQELEGAVK(*)	K2	83.3	161.68	1.14	0.9625	1.04255319	1.048351064
Myh10	Q61879	1331	QK(*)LNLSSR(*)	K2	68.41	1000	-	0.825	1.05319148	0.939095745
Myh10	Q61879	663	ESLTK(*)LMATLR(*)	K5	50.48	1000	0.54	-	1.11702127	0.828510639
Mcm7	Q61881	159	ADSVGK(*)LLTVR(*)	K6	49.01	1000	0.98958333	0.90123456	0.94565217	0.945490025
Npm1	Q61937	265	VEAK(*)FINYVK(*)	K4	96.52	143.14	1.01020408	1	0.94623655	0.985480214
Npm1	Q61937	248	AK(*)MQASIEK(*)	K2	87.15	128.65	-	0.9375	0.96774193	0.952620968
Srsf2	Q62093	36	VFEK(*)YGR(*)	K4	13.91	1000	-	-	1.408163265	
Sptbn1	Q62261	1354	EK(*)LTGLHK(*)	K2	66.27	101.67	1.12765957	1.35897435	1.18478260	1.223805514
Sptbn1	Q62261	1913	LVDTGDK(*)FR(*)	K7	49.68	1000	-	1.33333333	1.21739130	1.275362319
Tsn	Q62348	187	LLNLK(*)NDSLR(*)	K5	37.71	1000	2.35483871	1.14864864	-	1.75174368
Snrnp70	Q62376	118	VNYDTTESK(*)LR(*)	K9	48.29	1000	1.30303030	0.95061728	0.83695652	1.03020137
Sh3gl1	Q62419	67	AK(*)LTMLNTVSK(*)	K2	37.99	143.22	-	1.1125	1.22352941	1.168014706
Cstb	Q62426	34	ENQK(*)FDVFK(*)	K4	80.82	115.97	1.12631578	1.11688311	0.93023255	1.057810488
Eif4g2	Q62448	170	LLISK(*)LQDEFENR(*)	K5	27.89	1000	-	-	1.258426966	
Eif4g2	Q62448	760	DNISPK(*)LHVDK(*)	K6	20.28	46.62	-	1.5	-	
Zyx	Q62523	263	FAPVAPK(*)FTPVVSK(*)	K7	23.02	71.72	-	1.49253731	1.65	1.571268657
Raly	Q64012	165	TTIPVK(*)LFAR(*)	K6	36.15	1000	-	1.33783783	1.76744186	1.552639849

Btf3	Q64152	55	LAK(*)LQAQVR(*)	K3	83.01	1000	1.16853932	1.1818181	1.1379310	1.162762847
Btf3	Q64152	52	ETIMNQEK(*)LAK(*)	K8	43.24	32.69	1.33707865	1.74025974	1.1954022	1.424246897
Hist2h2bb	Q64525	117	HAVSEGTK(*)AVTK(*)	K8	79.76	72.09	-	-	-	
Hist2h2bb	Q64525	109	LLLPGELAK(*)HAVSEGTK(*)	K9	74.5	36.48	-	-	-	
Hist2h2bb	Q64525	47	VLK(*)QVHPDTGISSK(*)	K3	68.11	139.71	-	-	-	
Vcl	Q64727	778	EVENSEDPK(*)FR(*)	K9	76.24	1000	-	0.91025641	0.9347826	0.92251951
Vcl	Q64727	276	ALASIDSK(*)LNQAK(*)	K8	70.59	100.21	0.9690721	0.8846153	1.0326086	0.962098749
Cltc	Q68FD5	1612	VDK(*)LDASESLR(*)	K3	74.07	1000	0.98969072	0.9	0.5543478	0.814679516
Cltc	Q68FD5	163	QK(*)WLLLTGISAQQNR(*)	K2	28.87	1000	-	1.4375	-	
Myof	Q69ZN7	871	HK(*)FSDVTGK(*)	K2	18.08	104.64	1.3052631	-	1.7303370	1.517800119
Hist2h2aa2	Q6GSS7	96	NDEELNK(*)LLGK(*)	K7	88.57	87.62	-	-	-	
Tpm4	Q6IRU2	169	NVTNNLK(*)SLEAASEK(*)	K7	85.85	151.55	1.0103092	0.9012345	1.0439560	0.98516663
Tpm4	Q6IRU2	195	LLSDK(*)LK(*)	K5	85	33.18	0.9484536	1	1.0989010	1.015784902
Tpm4	Q6IRU2	116	EAK(*)HITDEADR(*)	K3	46.95	1000	-	0.5679012	-	
Tpm4	Q6IRU2	223	TIDDLEEK(*)LAQAK(*)	K8	21.06	80.41	-	-	1.505494505	
Dnajc8	Q6NZB0	108	AYK(*)LLLDQEQK(*)	K3	40.97	150.89	1.1980198	-	-	
Srsf1	Q6PDM2	38	DIEDVFYK(*)YGAIR(*)	K8	30.7	1000	1.59375	2.32098765	-	1.957368827
Srsf1	Q6PDM2	179	K(*)LDNTK(*)FR(*)	K6	14.52	86.16	1.0104166	-	-	
Rps9	Q6ZWN5	93	M(+15.99)K(*)LDYILGLK(*)	K2	90.75	154.74	1.0319148	0.8915662	1.0319148	0.985132018
Rps9	Q6ZWN5	116	LQTQVFK(*)LGLAK(*)	K7	86.71	87.95	0.9468085	0.91566265	0.9787234	0.947064855
Rps9	Q6ZWN5	47	VK(*)FTLAK(*)	K2	80.1	98.37	-	0.96385542	0.9574468	0.960651116
Rps9	Q6ZWN5	155	LDSQK(*)HIDFSLR(*)	K5	73.56	1000	1.0638297	0.8674698	1.0425531	0.991284286
Rps9	Q6ZWN5	30	LDQELK(*)LIGEYGLR(*)	K6	60.55	1000	1.0531914	-	1.0531914	1.053191489
Rps9	Q6ZWN5	91	IGVLDEGK(*)MK(*)	K8	58.16	30.83	1.2872340	0.96385542	1.0957446	1.115611382
Rpl10	Q6ZWV3	101	INK(*)MLSC(+57.02)AGADR(*	K3	72.68	1000	1.21875	0.8354430	-	1.027096519
Rpl10	Q6ZWV3	74	IC(+57.02)ANK(*)YMVK(*)	K5	68.54	82.24	1.2291666	0.89873417	1.2065217	1.111474194
Rpl10	Q6ZWV3	198	LIPDGC(+57.02)GVK(*)YIPNR(	K9	54.46	1000	0.7083333	0.65822784	-	0.683280591
Rpl10	Q6ZWV3	175	TK(*)FNADEFEDMVAEK(*)	K2	38.37	205.45	0.9895833	-	-	
Rpl35	Q6ZWV7	43	VTGGAASK(*)LSK(*)	K8	97.46	53.53	0.8686868	0.7216494	0.9883720	0.859569482
Rpl35	Q6ZWV7	25	QLDDLK(*)VELSQLR(*)	K6	29.29	1000	-	0.6701030	1.4883720	1.079237593
Ppp2r1a	Q76MZ3	188	AAASK(*)LGEFAK(*)	K5	89.23	132.53	1.0333333	0.93150684	0.9642857	0.976375299
Ppp2r1a	Q76MZ3	266	YMVADK(*)FTELQK(*)	K6	64.75	113.99	1.0777777	0.95890411	1.0476190	1.028100312
Syncrip	Q7TMK9	363	SFSQFGK(*)LER(*)	K7	51.57	1000	1.3958333	0.7662337	1.10989011	1.090652403
Dek	Q7TNV0	91	GQK(*)LC(+57.02)EIER(*)	K3	63.83	1000	-	0.9615384	1.0689655	1.01525199

Actn1	Q7TPR4	312	VPENTMHAMQQK(*)LEDFR(*)	K12	60.87	1000	1.10416666	0.83333333	0.82417582	0.920558608
Actn1	Q7TPR4	195	HR(*)PELIDYGK(*)LR(*)	K10	53.06	1000	1.63541666	0.92307692	1.15384615	1.237446581
Actn1	Q7TPR4	312	AMQQK(*)LEDFR(*)	K5	42.72	1000	1.17708333	-	-	
Mybbp1a	Q7TPV4	1097	TVNHEK(*)LSVDLTAPLGVLQSK	K6	61.68	298.8	0.98958333	-	-	
Mybbp1a	Q7TPV4	54	ATEK(*)LLEYLR(*)	K4	57.61	1000	1.10416666	-	-	
Septin9	Q80UG5	488	LVNEK(*)FR(*)	K5	71.3	1000	1.15789473	0.95	1.24175824	1.116550993
Flnb	Q80X90	495	GPK(*)GLEELVK(*)	K3	69.91	148.49	-	0.83116883	-	
Flnb	Q80X90	1005	EC(+57.02)STAK(*)FIPR(*)	K6	53.83	1000	-	1.11688311	-	
Flnb	Q80X90	1801	YAPTEVGLHEMHIK(*)YR(*)	K14	48.51	1000	1.16842105	0.70129870	0.9	0.923239918
Tufm	Q8BFR5	88	ILAEGGGAK(*)FK(*)	K9	33.06	22.85	1.40425531	1.08108108	-	1.2426682
Tnpo1	Q8BFY9	192	FLQFFK(*)HSSPK(*)	K6	32.23	115.97	-	2	-	
Hnrnpa3	Q8BG05	57	EHFEK(*)WGTLTDC(+57.02)VV	K5	23.38	1000	-	-	1.272727273	
Aars	Q8BGQ7	338	YSHEK(*)LNASR(*)	K5	28.81	1000	-	1.35443038	1.31868131	1.33655585
Aars	Q8BGQ7	476	AK(*)GLEATDDSPK(*)	K2	28.35	168.62	-	-	1.252747253	
Nup93	Q8BJ71	619	VASVAENK(*)GLFEEAAK(*)	K8	42.63	78.17	1.36559139	1.225	1.43010752	1.340232975
Nup93	Q8BJ71	718	LK(*)LVPLNQESVEER(*)	K2	32.37	1000	-	1	-	
Ipo5	Q8BKC5	800	AK(*)LEEHFK(*)	K2	74.22	129.15	0.95789473	1.15189873	1.22340425	1.111065909
Ipo5	Q8BKC5	712	LK(*)FYFHDGVR(*)	K2	39.95	1000	2.87368421	-	-	
Slc25a24	Q8BMD8	283	LLTEEGQK(*)LGTFER(*)	K8	37.58	1000	0.54639175	0.61842105	1.54216867	0.90232716
Eif1ax	Q8BMJ3	88	DYQDNK(*)ADVILK(*)	K6	30.6	69.64	-	-	1.733333333	
Ckap4	Q8BMK4	167	QDLTEK(*)AVK(*)	K6	78.4	53.53	-	1.16455696	0.95604395	1.060300459
Ckap4	Q8BMK4	361	R(*)LEEELQQLK(*)VGAHGSEEG	K10	48.92	82.05	1.08080808	1.16455696	-	1.122682522
Ckap4	Q8BMK4	335	LALQALTEK(*)LLR(*)	K9	45.68	1000	1.37373737	-	-	
Ckap4	Q8BMK4	524	SSVSQVESDLK(*)MLR(*)	K11	45.1	1000	0.95959596	0.98734177	-	0.973468866
Ckap4	Q8BMK4	251	MK(*)VASLEESK(*)	K2	21.73	78.75	-	-	1.406593407	
NARS1	Q8BP47	42	TGLK(*)ALMTVGK(*)	K4	14.5	78.16	-	1.71604938	-	
Rpl24	Q8BP67	27	TDGK(*)VFQFLNAK(*)	K4	47.81	154.74	1.02912621	-	0.89473684	0.961931528
Flna	Q8BTM8	120	ESIK(*)LVSIDSK(*)	K4	78.8	110.43	0.90425531	0.93506493	0.94318181	0.927500691
Flna	Q8BTM8	906	GK(*)LDVQFSGLAK(*)	K2	68.93	148.29	1.10638297	0.92207792	1.22727272	1.085244543
Flna	Q8BTM8	2563	VVAK(*)GLGLSK(*)	K4	68.54	151.86	1.11702127	-	0.88636363	1.001692457
Flna	Q8BTM8	127	LVSIDSK(*)AIVDGNLK(*)	K7	49.3	97.31	0.87234042	0.70129870	0.82954545	0.801061527
Iars	Q8BU30	254	GK(*)LFILTEAR(*)	K2	54.94	1000	-	1	1.04255319	1.021276596
Iars	Q8BU30	169	QLYDK(*)GLVYR(*)	K5	17.76	1000	2.25531914	-	-	
Etf1	Q8BWY3	171	EVLHK(*)FTVDLPK(*)	K5	22.66	68.46	1.72043010	-	-	

Pgam5	Q8BX10	140	LASLGLK(*)FNK(*)	K7	22.96	44.44	2.83132530	1.58108108	-	2.206203191
Gemin5	Q8BX17	738	LSQFK(*)PK(*)	K5	1.87	22.85	-	0	0	0
Septin11	Q8C1B7	366	ELHEK(*)FDLLK(*)	K5	81.95	115.97	1.03030303	0.85185185	0.98863636	0.956930415
Smc4	Q8CG47	387	EK(*)FTQLDLEDVQVR(*)	K2	19.61	1000	1.69696969	-	-	
Smc2	Q8CG48	1160	TK(*)FVDGVSTVAR(*)	K2	44.77	1000	-	-	1.042105263	
Smc2	Q8CG48	677	SQAASILTK(*)FQEVK(*)	K9	30.58	61.26	1.75510204	1.43421052	-	1.594656284
Eprs	Q8CGC7	1389	DTGEK(*)LTIAEK(*)	K5	53.85	108.85	0.89690721	1.16666666	0.93617021	0.999914699
Eprs	Q8CGC7	282	YAEK(*)LIQEGK(*)	K4	30.52	113.99	-	2.96153846	-	
H2bc12	Q8CGP1	6	PEPAK(*)SAPAPK(*)	K5	99.73	85.52	387.878787	-	400	393.939394
H2bc12	Q8CGP1	121	AVTK(*)YTSAK(*)	K4	48.53	51	1.28787878	1.26666666	0.953125	1.169223485
Hist1h2ah	Q8CGP6	96	NDEELNK(*)LLGR(*)	K7	99.3	1000	0.93814433	0.88095238	1.01162790	0.943574873
Eif3b	Q8JZQ9	243	NADGYK(*)LDK(*)	K6	24.38	32.97	-	-	1.433333333	
Eif3b	Q8JZQ9	353	GIALWGGDK(*)FK(*)	K9	13.4	21.68	-	1.89024390	-	
Bclaf1	Q8K019	591	SIFDHIK(*)LPQANK(*)	K7	11.27	36.87	-	1.24	-	
Dnm1l	Q8K1M6	244	VIPVK(*)LGIIGVVNR(*)	K5	53.93	1000	1.02083333	1.20253164	0.96629213	1.063219038
Sdha	Q8K2B3	179	AFGGQSLK(*)FGK(*)	K8	32.63	49.37	1.54	1.21428571	-	1.377142857
Matr3	Q8K310	473	VHLSQK(*)YK(*)	K6	26.63	45.01	-	-	2.044444444	
Eif3l	Q8QZY1	465	SFLK(*)LYTTMPVAK(*)	K4	22.89	140.79	-	1.69047619	-	
Gspt1	Q8R050	350	TAGVK(*)HLIVLINK(*)	K5	20.9	171.6	2.18681318	-	-	
Eif3c	Q8R1B4	551	LC(+57.02)K(*)YIYAK(*)	K3	16.84	98.37	-	0.6375	-	
Slc38a4	Q8R1S9	140	EGGSLIYEK(*)LGEK(*)	K9	37.12	48.12	-	1.32926829	-	
Nup58	Q8R332	300	LK(*)LETAQELK(*)	K2	79.95	139.75	-	0.84931506	0.70526315	0.777289113
Nup85	Q8R480	540	LTFLGK(*)YR(*)	K6	23.09	1000	-	1.31578947	-	
Arl6ip5	Q8R5J9	151	LENK(*)MEGIGLK(*)	K4	22.13	102.29	-	1.57692307	-	
Myh9	Q8VDD5	1793	AK(*)LQEMESAVK(*)	K2	97.68	195.41	0.97959183	0.91139240	0.97802197	0.956335407
Myh9	Q8VDD5	1324	QK(*)LSLSTK(*)	K2	93.3	109.62	0.94897959	0.97468354	1.04395604	0.989206393
Myh9	Q8VDD5	656	EQLAK(*)LMATLR(*)	K5	80.48	1000	0.87755102	0.88607594	1.12087912	0.96150203
Myh9	Q8VDD5	299	TDLLLEPYNK(*)YR(*)	K10	80.09	1000	1.16326530	1.02531645	0.94505494	1.044545569
Myh9	Q8VDD5	1024	NK(*)HEAMITDLEER(*)	K2	78.93	1000	0.94897959	0.98734177	1.02197802	0.986099795
Myh9	Q8VDD5	8	A(+42.01)QQAADK(*)YLYVDK(	K7	78.44	109.28	1.33673469	1.27848101	1.03296703	1.216060913
Myh9	Q8VDD5	989	K(*)LEEDQIIMEDQNC(+57.02)	K15	77.16	38.16	1.10204081	1.22784810	0.97802197	1.102636965
Myh9	Q8VDD5	860	EK(*)HLAAENR(*)	K2	74.49	1000	0.96938775	1.02531645	1.01098901	1.001897741
Myh9	Q8VDD5	1410	VAAYDK(*)LEK(*)	K6	73.56	37.54	-	0.70886075	0.90109890	0.80497983
Myh9	Q8VDD5	1477	ETK(*)ALSLAR(*)	K3	68.99	1000	-	0.94936708	1.30769230	1.128529699

Myh9	Q8VDD5	1459	TISAK(*)YAEER(*)	K5	56.3	1000	-	1.20253164	1.31868131	1.260606483
Myh9	Q8VDD5	580	VDYK(*)ADEWLMK(*)	K4	54.85	114.14	0.89795918	0.84810126	1.04395604	0.930005498
Myh9	Q8VDD5	682	AGK(*)LDPHLVLDQLR(*)	K3	46.89	1000	1.77551020	-	-	
Myh9	Q8VDD5	1620	K(*)LEMDLK(*)DLEAHIDTANK(	K7	40.23	22.09	0.79591836	-	-	
Myh9	Q8VDD5	1193	QK(*)HSQAVEELADQLEQTK(*)	K2	39.29	211.93	-	0.51898734	2.56043956	1.539713451
Myh9	Q8VDD5	1332	LK(*)QMEDEK(*)	K2	24.81	121.92	-	1.06329113	-	
Myh9	Q8VDD5	833	LFTK(*)VK(*)PLLNSIR(*)	K4	10.78	60.92	-	1.26582278	-	
Atp1a1	Q8VDN2	45	EVSMDDHK(*)LSLDELHR(*)	K8	51.14	1000	1.15625	-	-	
Ddx39a	Q8VDW0	190	NVK(*)HFVLDEC(+57.02)DK(*	K3	53.01	181.07	-	1.67105263	-	
Ddx39a	Q8VDW0	137	FSK(*)YMPSVK(*)	K3	23.65	49.54	-	-	1.144444444	
Hnrnpu	Q8VEK3	527	QMADTGK(*)LNTLLQR(*)	K7	78.22	1000	1.14285714	1.10389610	1.14444444	1.13039923
Hnrnpu	Q8VEK3	328	HLYTK(*)DIDIHEVR(*)	K5	74.77	1000	1.16483516	1.05194805	0.92222222	1.046335146
Hnrnpu	Q8VEK3	541	APQC(+57.02)LGK(*)FIEIAAR(	K7	71.23	1000	1.17582417	1.23376623	1.35555555	1.255048655
Hnrnpu	Q8VEK3	31	GLK(*)ADLMDR(*)	K3	67.27	1000	0.94505494	0.77922077	1.05555555	0.926610427
Hnrnpu	Q8VEK3	640	EEAQK(*)LLEQYK(*)	K5	39.76	143.14	0.67032967	-	-	
Hnrnpu	Q8VEK3	611	DLPEHAVLK(*)MK(*)	K9	28.72	30.36	-	2.07792207	1.73333333	1.905627706
Hnrnpu	Q8VEK3	500	HAAENPGK(*)YNILGTNTIMDK(	K8	26.25	106.18	-	1.29870129	-	
Hnrnpu	Q8VEK3	12	LK(*)VSELK(*)EELK(*)	K2	18.97	76.17	-	1.07792207	-	
Slc25a3	Q8VEM8	308	GVWK(*)GLFAR(*)	K4	72.7	1000	0.98979591	1	0.92473118	0.971509034
Slc25a3	Q8VEM8	242	TVEALYK(*)FVVPK(*)	K7	43.19	49.68	1.15306122	-	-	
Slc25a3	Q8VEM8	229	TMMK(*)FAC(+57.02)FER(*)	K4	16	1000	1.40816326	-	-	
Flnc	Q8VHX6	114	EHIK(*)LVSIDSK(*)	K4	68.7	163.4	0.88888888	0.85135135	1.04651162	0.928917289
Flnc	Q8VHX6	1832	YAPTEK(*)GLHQMGIK(*)	K6	39.04	115.01	0.88888888	1.20270270	1.02325581	1.038282469
Flnc	Q8VHX6	902	AK(*)LDVHFAGAAK(*)	K2	30.93	172.15	-	1.08108108	-	
Sfpq	Q8VIJ6	405	STGK(*)GIVEFASK(*)	K4	22.19	132.12	-	-	1.976744186	
Acly	Q91V92	265	SGASLK(*)LTLLNPK(*)	K6	64.03	161.52	1.19148936	0.83333333	0.83146067	0.952094456
Cavin3	Q91VJ2	121	GK(*)LHVLLFK(*)	K2	27.77	161.52	-	1.09756097	-	
Rbmxl1	Q91VM5	30	ALEAVFGK(*)YGR(*)	K8	59.08	1000	1.16666666	0.97402597	0.65476190	0.931818182
Ddx1	Q91VR5	358	LDDLVSTGK(*)LNLSQVR(*)	K9	28.58	1000	1.79166666	-	-	
Ddx1	Q91VR5	317	YIDNPK(*)LR(*)	K6	11.59	1000	-	-	1.285714286	
Rpn1	Q91YQ5	588	DTYLENEK(*)LSSGK(*)	K8	41.56	79.02	1.74489795	0.93975903	-	1.342328498
Rpn1	Q91YQ5	565	ELVLK(*)SAVEAER(*)	K5	21.72	1000	-	0.98795180	-	
Lrp1	Q91ZX7	2391	AEK(*)LYFSDATLDK(*)	K3	18.77	131.64	1.42574257	-	-	
Supt16h	Q920B9	216	HSK(*)LAESVEK(*)	K3	13.95	102.29	-	1.41772151	-	

Fdps	Q920E5	31	VLTEK(*)ELGHPEIGDAIAR(*)	K5	58.85	1000	-	0.88607594	-	
Fdps	Q920E5	57	EVLEYNALGGK(*)YNR(*)	K11	35.54	1000	-	1.55696202	0.97777777	1.267369902
Rheb	Q921J2	102	VIHGK(*)LLDMVGK(*)	K5	32.78	161.52	-	1.54794520	-	
Sf3b3	Q921M3	965	VLIGVGK(*)LLR(*)	K7	29.83	1000	-	1.04819277	-	
Clip1	Q922J3	812	EK(*)FASTSEEAVSAQTR(*)	K2	37.23	1000	-	0.91666666	1.73626373	1.326465202
Far1	Q922J9	29	VLLEK(*)LLR(*)	K5	52.12	1000	-	-	1.24691358	
Far1	Q922J9	63	VEEILSSK(*)LFDR(*)	K8	43.65	1000	1.06451612	1.25333333	1.45679012	1.258213195
Lrrc59	Q922Q8	73	NK(*)LQQLPADFGR(*)	K2	93.29	1000	0.98039215	0.96296296	1.03409090	0.99248201
Lrrc59	Q922Q8	111	NLK(*)WLDLK(*)	K3	53.77	115.97	-	1	1.52272727	1.261363637
Taldo1	Q93092	269	DNSK(*)LAPALSVK(*)	K4	36.1	118.18	-	-	0.988095238	
Rap1b	Q99JI6	5	EYK(*)LVVLGSGGVGK(*)	K3	48.12	159.14	-	1.36111111	-	
Eif3m	Q99JX4	155	WISDWK(*)LTTEK(*)	K6	32.92	76.42	-	1.41025641	-	
Actr3	Q99JY9	348	LK(*)LSEELSGGR(*)	K2	58.61	1000	-	0.87341772	1.02222222	0.947819972
Nono	Q99K48	297	EK(*)LEMEMEAAR(*)	K2	49.5	1000	1.01098901	-	3.56179775	2.286393382
Nono	Q99K48	98	K(*)LFEK(*)YGK(*)	K5	47.55	37.54	-	-	1.393258427	
Nono	Q99K48	192	GR(*)PSGK(*)GIVEFSGK(*)	K6	15.26	73.08	-	1.17333333	-	
Psat1	Q99K85	190	PVDVSK(*)FGVIFAGAQK(*)	K6	63.52	133.99	1.01063829	0.80519480	1.41573033	1.077187813
Psat1	Q99K85	269	NNGGAAAMEK(*)LSSIK(*)	K10	48.67	30.64	-	0.98701298	0.91011236	0.948562674
Tmed9	Q99KF1	160	DK(*)LSELQLR(*)	K2	47.07	1000	1.8	-	-	
Aco2	Q99KI0	50	YDLLEK(*)NINIVR(*)	K6	29.04	1000	2.05050505	1.70129870	-	1.875901876
Dctn2	Q99KJ8	65	VGTK(*)GLDFSDR(*)	K4	11.99	1000	-	-	1.244444444	
Prpf19	Q99KP6	428	NFK(*)TLQLDNNFEVK(*)	K3	39.12	183.46	1.29787234	-	-	
St13	Q99L47	141	AIDLFTDAIK(*)LNPR(*)	K10	61.65	1000	0.80851063	0.74074074	1.10989011	0.886380496
St13	Q99L47	362	VMNLISK(*)LSAK(*)	K7	16.36	42.89	-	1.75308642	-	
Gps1	Q99LD4	317	FYESK(*)YASC(+57.02)LK(*)	K5	19.59	63.05	-	2.62162162	-	
Kars1	Q99MN1	139	ASGGK(*)LIFYDLR(*)	K5	58.45	1000	0.97916666	0.87951807	-	0.92934237
Srrt	Q99MR6	525	LAAK(*)LIHTLDDR(*)	K4	16.63	1000	-	1.67567567	-	
Rrbp1	Q99PL5	1564	AAIK(*)LQELLK(*)	K4	55.99	134.33	0.98039215	-	0.86813186	0.924262013
Rrbp1	Q99PL5	1124	VAELHSK(*)LQSSEVEVK(*)	K7	30.17	74.73	-	0.97368421	-	
Atp5mg	Q9CPQ8	66	TGSFK(*)HLTVK(*)	K5	65.57	105.53	-	0.97402597	1.04255319	1.008289583
Mydgf	Q9CPT4	154	AELSK(*)LVIVAK(*)	K5	27.01	121.92	-	1.12	-	
Bzw1	Q9CQC6	60	FLDASGAK(*)LDYR(*)	K8	75.4	1000	1.07446808	0.96153846	0.97701149	1.004339347
Chmp1b2	Q9CQD4	12	HLFNLK(*)FAAK(*)	K6	28.11	48.83	-	1.38888888	-	
Ykt6	Q9CQW1	166	GEK(*)LDDLVSK(*)	K3	12.65	75.79	-	1.02597402	-	

Rpl14	Q9CR57	85	ADINTK(*)WAATR(*)	K6	74.65	1000	1.32352941	0.9	1.04347826	1.089002558
Rpl14	Q9CR57	103	AK(*)MTDFDR(*)	K2	70.64	1000	1.07843137	1.0375	0.94565217	1.020527849
Rpl14	Q9CR57	23	AGK(*)LVAIVDVIDQNR(*)	K3	40.04	1000	0.85294117	-	-	
Xpot	Q9CRT8	635	VLLEK(*)LMMAQDEER(*)	K5	13.97	1000	0.83505154	-	0.81521739	0.825134469
Pfdn1	Q9CWM4	28	VK(*)LADIQIEQLNR(*)	K2	52.32	1000	-	0.94520547	-	
Psmd8	Q9CX56	284	AYEK(*)ILFAEATR(*)	K4	10.23	1000	-	0.43037974	-	
Hnrnpa0	Q9CX86	159	AAVVK(*)FHPIQGHR(*)	K5	67.38	1000	-	0.79487179	0.93333333	0.864102564
Rpl11	Q9CXW4	85	AEEILEK(*)GLK(*)	K7	86.42	57.45	0.95959596	0.875	1	0.94486532
Rpl11	Q9CXW4	67	NEK(*)IAVHC(+57.02)TVR(*)	K3	33.43	1000	-	0.89772727	1.61111111	1.254419192
Rpl11	Q9CXW4	67	R(*)NEK(*)IAVHC(+57.02)TVR(	K4	13.45	1000	-	-	1.133333333	
Serbp1	Q9CY58	122	R(*)PDQQLQGDGK(*)LIDR(*)	K11	76.18	1000	0.98936170	0.85897435	-	0.924168031
Serbp1	Q9CY58	302	AK(*)VEFNIR(*)	K2	39.67	1000	0.54255319	-	-	
Gars1	Q9CZD3	187	TSGHVDK(*)FADFMVK(*)	K7	59.45	110.43	1.12765957	0.86842105	-	0.998040314
Rpl15	Q9CZM2	93	PVHHGVNQLK(*)FAR(*)	K10	64.24	1000	-	-	0.829545455	
Shmt2	Q9CZN7	464	TAK(*)LQDFK(*)	K3	59.21	96.93	0.86458333	0.91566265	-	0.890122992
Cs	Q9CZU6	327	DVSDEK(*)LR(*)	K6	58.16	1000	-	1.74712643	-	
Cs	Q9CZU6	459	SMSTDGLMK(*)FVDSK(*)	K9	51.85	87.95	0.99009901	0.86206896	1.26666666	1.039611548
Cs	Q9CZU6	327	EVGK(*)DVSDEK(*)LR(*)	K10	15.81	62.39	-	1.74712643	-	
Cs	Q9CZU6	366	EFALK(*)HLPK(*)	K5	14.24	69.71	1.95049505	-	-	
Rps19	Q9CZX8	38	VPEWVDTVK(*)LAK(*)	K9	62.95	40.63	0.84313725	0.89534883	0.92929292	0.889259674
Rps19	Q9CZX8	29	LK(*)VPEWVDTVK(*)	K2	58.41	105.42	-	0.74418604	1.09090909	0.917547569
Rps19	Q9CZX8	111	VLQALEGLK(*)MVEK(*)	K9	39.84	52.86	1.24509803	0.83720930	1.35353535	1.145280898
Rps19	Q9CZX8	77	GGAGVGSMTK(*)IYGGR(*)	K10	33.05	1000	-	1.61627907	1.27272727	1.444503172
Pbdc1	Q9D0B6	63	LISSVDPQFLK(*)LTK(*)	K11	35.05	28.96	-	0.79012345	-	
Hnrnpm	Q9D0E1	671	DK(*)FNEC(+57.02)GHVLYADI	K2	46.82	39.02	-	0.7	-	
Rars	Q9D0I9	484	DK(*)VLTEEELK(*)	K2	73.97	135.81	-	0.63291139	0.64835164	0.64063152
Tars1	Q9D0R2	242	ILNEK(*)VNTPTTTVYR(*)	K5	11.06	1000	-	1.20779220	-	
Tmed10	Q9D1D4	143	VEK(*)LK(*)PLEVELR(*)	K3	19.31	43.88	-	1.23170731	-	
Sarnp	Q9D1J3	10	A(+42.01)AETVELHK(*)LK(*)	K9	51.33	26.31	1.12037037	1.01265822	-	1.066514299
Eef1e1	Q9D1M4	138	EK(*)YLNVSR(*)	K2	16.98	1000	-	1.62650602	-	
Chordc1	Q9D1P4	134	LK(*)LSSGSEEDK(*)K(*)EEDSDI	K2	16.16	60.15	-	-	1.333333333	
Bcas2	Q9D287	177	NMQLTAGSK(*)LR(*)	K9	29.2	1000	1.14285714	1.30379746	1.32989690	1.258850506
Nop56	Q9D6Z1	375	YLANK(*)C(+57.02)SIASR(*)	K5	26.46	1000	-	2.09756097	-	
Ppa1	Q9D819	57	WSNAK(*)MEIATK(*)	K5	14.57	50.87	-	1.74074074	-	

Uqcrb	Q9D855	12	SAVSASSK(*)WLDGFR(*)	K8	14.8	1000	1.21311475	-	-	
Rpl4	Q9D8E6	239	LNILK(*)LAPGGHVGR(*)	K5	79.11	1000	0.76842105	0.85185185	1.125	0.915090968
Rpl4	Q9D8E6	327	IMLK(*)LNPYAK(*)	K4	61.53	113.99	-	1.07407407	-	
Rpl4	Q9D8E6	333	LNPYAK(*)TMR(*)	K6	48.25	1000	-	1.39506172	1.125	1.260030864
Eef1g	Q9D8N0	434	AVNQGK(*)IFK(*)	K6	97.78	50.22	-	-	36.83870968	
Eef1g	Q9D8N0	212	LC(+57.02)EK(*)MAQFDAK(*)	K4	94.27	148.49	1.05208333	0.96341463	0.97849462	0.99799753
Eef1g	Q9D8N0	147	ILGLLDTHLK(*)TR(*)	K10	57.34	1000	1.04166666	-	0.91397849	0.977822581
Eef1g	Q9D8N0	208	AILGEVK(*)LC(+57.02)EK(*)	K7	51.7	69.71	1.58333333	0.80487804	0.97849462	1.122235335
Cnn3	Q9DAW9	156	R(*)FDEGK(*)LK(*)	K6	14.84	30.83	-	-	0.775280899	
Atp5po	Q9DB20	192	IGEK(*)YVDMSAK(*)	K4	51.26	104.64	-	-	0.935483871	
Ap2b1	Q9DBG3	721	AK(*)GLEISGTFTHR(*)	K2	32.1	1000	-	1.3875	-	
Rpn2	Q9DBG6	460	NVYK(*)FELDTSER(*)	K4	23.3	1000	-	1.76315789	-	
Gnai3	Q9DC51	35	EVK(*)LLLLGAGESGK(*)	K3	35.74	176.98	-	3.03846153	1.91764705	2.478054299
Gnai3	Q9DC51	92	LK(*)IDFGESAR(*)	K2	17.46	1000	-	1.89743589	-	
Tomm20	Q9DCC8	56	AGLSK(*)LPDLK(*)	K5	15.04	81.69	-	-	-	
Eif3f	Q9DCH4	242	TVK(*)YAYYDTER(*)	K3	19.52	1000	-	1.14285714	-	
Paics	Q9DCL9	53	K(*)NHLEGK(*)AAISNK(*)	K7	16.96	46.17	-	-	1.444444444	
Etfb	Q9DCW4	176	LK(*)LPAVVTADLR(*)	K2	56.44	1000	-	0.79487179	-	
Pes1	Q9EQ61	81	FLLHEPIVNK(*)FR(*)	K10	25.25	1000	1.42696629	-	-	
Vps35	Q9EQH3	38	NK(*)LMDALK(*)	K2	67.06	123.86	-	0.93506493	1.17582417	1.055444556
Mvp	Q9EQK5	745	IEGEGSVLQAK(*)LK(*)	K11	23.68	22.85	2.31632653	-	-	
Rtn3	Q9ES97	954	IQAK(*)LPGIAK(*)	K4	44.04	62.39	-	-	2.206185567	
Anp32b	Q9EST5	116	R(*)LDC(+57.02)LK(*)SLDLFG	K6	24.14	1000	1.60439560	-	-	
Tmod3	Q9JHJ0	230	TLEANTHVK(*)HFSLAATR(*)	K9	23.85	1000	-	1.41095890	-	
Dync1h1	Q9JHU4	679	GWENHVEGQK(*)LK(*)	K10	19.52	32.28	-	1.37974683	1.59340659	1.486576714
Dync1h1	Q9JHU4	1117	VNLK(*)YDSWHK(*)	K4	16.91	76.27	-	2.29113924	-	
Prmt1	Q9JIF0	134	ANK(*)LDHVVTIIK(*)	K3	61.74	159.26	1.03092783	0.72839506	-	0.879661449
Copb1	Q9JIF7	298	ESDNNVK(*)LIVLDR(*)	K7	25.9	1000	1.68085106	-	2.32584269	2.003346881
Ddx21	Q9JIK5	750	GK(*)LGVC(+57.02)FDVR(*)	K2	53.59	1000	1.34693877	1.11111111	1.47959183	1.312547241
Eny2	Q9JIX0	19	AAINQK(*)LIETGER(*)	K6	59.47	1000	1.10280373	0.94871794	0.91208791	0.987869866
Iqgap1	Q9JKF1	1562	ISLK(*)YTAAR(*)	K4	24.95	1000	-	1.46835443	-	
Hyou1	Q9JKR6	391	VPK(*)VQEVLLK(*)	K3	40.92	102.29	-	0.72368421	1.19101123	0.957347724
Usp14	Q9JMA1	291	YLFTGLK(*)LR(*)	K7	65.27	1000	0.94505494	1.06756756	0.97727272	0.996631747
Prep	Q9QUR6	172	VK(*)FTC(+57.02)MAWTHDG	K2	24.97	183.46	-	-	2.793478261	

Plec	Q9QXS1	1983	VLTEK(*)LAAISEATR(*)	K5	93.41	1000	1.18085106	0.97435897	1.22222222	1.125810753
Plec	Q9QXS1	2460	EK(*)MQAVQEATR(*)	K2	67.36	1000	-	0.58974359	1.28888888	0.93931624
Plec	Q9QXS1	4482	FPVTEAVNK(*)GLVDK(*)	K9	64.79	81.69	0.86170212	0.93589743	1.12222222	0.973273929
Plec	Q9QXS1	311	EK(*)LLLWSQR(*)	K2	63.28	1000	0.86170212	0.94871794	2.13333333	1.31458447
Plec	Q9QXS1	3060	ATVSAPFGK(*)FQGR(*)	K9	47.09	1000	-	1.07692307	-	
Plec	Q9QXS1	748	LDLQYAK(*)LLNSSK(*)	K7	43.37	62.39	1.21276595	-	0.88888888	1.050827423
Plec	Q9QXS1	2660	AK(*)LEQLFQDEVAK(*)	K2	35.4	236.99	-	1.66666666	-	
Plec	Q9QXS1	2561	QAEEIGEK(*)LHR(*)	K8	33.67	1000	-	1.23076923	1.52222222	1.376495727
Plec	Q9QXS1	4511	TK(*)MSAAQALK(*)	K2	24.33	113.97	-	1.55128205	1.36666666	1.458974359
Plec	Q9QXS1	2033	R(*)LEEQAALHK(*)ADIEER(*)	K10	22.82	1000	-	1.38461538	-	
Plec	Q9QXS1	4147	DK(*)LLSAER(*)	K2	19.61	1000	-	-	1.255555556	
Plec	Q9QXS1	247	FHK(*)LQNVQIALDYLR(*)	K3	18.35	1000	-	2.62820512	-	
Plec	Q9QXS1	2055	QK(*)GLVEDTLR(*)	K2	15.33	1000	-	1.73076923	-	
Dnaja2	Q9QYJ0	134	TTK(*)LQLSK(*)	K3	35.62	95.62	-	0.88607594	0.90625	0.896162975
Esd	Q9R0P3	4	A(+42.01)LK(*)QISSNR(*)	K3	84.42	1000	0.95744680	0.95121951	0.94736842	0.952011581
Ptges3	Q9R0Q7	33	DVNVNFEK(*)SK(*)	K8	87.65	33.18	-	34.8717948	-	
Ptges3	Q9R0Q7	35	SK(*)LTFSC(+57.02)LGGSDNFI	K2	79.21	181.47	0.94505494	0.91025641	1.28409090	1.046467421
Acot9	Q9R0X4	102	DK(*)YVTVQNTVR(*)	K2	32.92	1000	1.27368421	-	1.45348837	1.363586292
Psma4	Q9R1P0	205	TMDVSK(*)LSAEK(*)	K6	57.3	52.68	-	1.63157894	0.66666666	1.149122807
Sae1	Q9R1T2	49	VLIVGMK(*)GLGAEIAK(*)	K7	71	161.77	0.95	0.90909090	0.76595744	0.875016119
Vapa	Q9WV55	188	LQGEMMK(*)LSEENR(*)	K7	19.14	1000	2.77528089	-	-	
Vapa	Q9WV55	125	EAK(*)PDELMDSK(*)LR(*)	K11	15.74	78.75	-	2	-	
Tagln2	Q9WVA4	54	ENFQK(*)WLK(*)	K5	38.77	32.69	-	1.23456790	0.90322580	1.068896854
Ehd1	Q9WVK4	32	QLYAQK(*)LLPLEEHYR(*)	K6	33.21	1000	1.57777777	0.8375	-	1.207638889
Eif2s3x	Q9Z0N1	183	LK(*)HILILQNK(*)	K2	58.6	217.23	1.36263736	0.7	-	1.031318682
Eif2s3x	Q9Z0N1	80	NITIK(*)LGYANAK(*)	K5	38.99	127.53	2.04395604	1.1875	0.69565217	1.309036073
Eif2s3x	Q9Z0N1	285	GGVAGGSILK(*)GVLK(*)	K10	35.31	63.97	0.83516483	1.6125	-	1.223832418
Aldh18a1	Q9Z110	649	IHAGPK(*)FASYLTFSPSEVK(*)	K6	27.22	187.28	-	2.22077922	-	
Vars	Q9Z1Q9	951	HFC(+57.02)NK(*)LWNATK(*)	K5	56.83	69.64	-	0.89473684	1.10752688	1.001131862
Vars	Q9Z1Q9	842	LTGK(*)LPFR(*)	K4	13.42	1000	1.21978022	2.19736842	-	1.708574321
Ilf3	Q9Z1X4	100	VGLVAK(*)GLLLK(*)	K6	52.32	95.62	1.31182795	-	1.14772727	1.229777615
Hnrnpc	Q9Z204	39	SDVEAIFSK(*)YGK(*)	K9	60.07	40.63	0.45360824	0.34615384	0.63333333	0.477698475
Hnrnpc	Q9Z204	39	K(*)SDVEAIFSK(*)YGK(*)	K10	54.11	32.97	-	0.66666666	-	
Hnrnpc	Q9Z204	207	QK(*)VDSLLESLEK(*)	K2	25.01	112.95	1.11340206	1.26923076	-	1.191316416

Psma7	Q9Z2U0	52	SVAK(*)LQDER(*)	K4	20.07	1000	-	1.04054054	-
Sart1	Q9Z315	183	LLNQK(*)LGK(*)	K5	20.34	57.45	-	0.9868421C	-
